# Machine Learning Interatomic Potential for Modeling the Mechanical and Thermal Properties of Naphthyl-Based Nanotubes

**DOI:** 10.1021/acs.jctc.4c01578

**Published:** 2025-01-28

**Authors:** Hugo X. Rodrigues, Hudson R. Armando, Daniel A. da Silva, João Paulo J. da Costa, Luiz A. Ribeiro, Marcelo L. Pereira

**Affiliations:** † Institute of Physics, University of Brasília, 70910-900 Brasília-DF, Brazil; ‡ Computational Materials Laboratory, University of Brasília, 70910-900 Brasília-DF, Brazil; § Physics Postgraduate Program, Institute of Physics, University of Brasília, 70910-900 Brasília-DF, Brazil; ∥ Department Lippstadt 2, Hamm-Lippstadt University of Applied Sciences, 59063 Hamm, Germany; ⊥ Professional Postgraduate Program in Electrical Engineering, Department of Electrical Engineering, College of Technology, University of Brasília, 70910-900 Brasília-DF, Brazil; # Department of Electrical Engineering, College of Technology, University of Brasília, 70910-900 Brasília-DF, Brazil

## Abstract

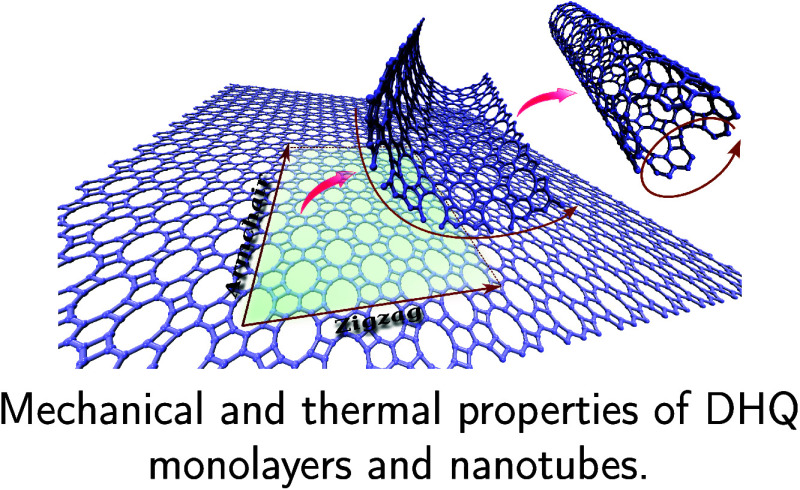

Two-dimensional (2D) nanomaterials are at the forefront of potential technological advancements. Carbon-based materials have been extensively studied since synthesizing graphene, which revealed properties of great interest for novel applications across diverse scientific and technological domains. New carbon allotropes continue to be explored theoretically, with several successful synthesis processes for carbon-based materials recently achieved. In this context, this study investigates the mechanical and thermal properties of DHQ-based monolayers and nanotubes, a carbon allotrope characterized by 4-, 6-, and 10-membered carbon rings, with a potential synthesis route using naphthalene as a molecular precursor. A machine-learned interatomic potential (MLIP) was developed to explore this nanomaterial’s mechanical and thermal behavior at larger scales than those accessible through the first-principles calculations. The MLIP was trained on data derived from the DFT/PBE (density functional theory/Perdew–Burke–Ernzerhof) level using ab initio molecular dynamics (AIMD). Classical molecular dynamics (CMD) simulations, employing the trained MLIP, revealed that Young’s modulus of DHQ-based nanotubes ranges from 127 to 243 N/m, depending on chirality and diameter, with fracture occurring at strains between 13.6 and 17.4% of the initial length. Regarding thermal response, a critical temperature of 2200 K was identified, marking the onset of a transition to an amorphous phase at higher temperatures.

## 1 Introduction

In recent years, nanomaterials have transformed the field of advanced technologies due to their unique properties and high surface-to-volume ratio,^1^ making them promising for diverse applications such as electronics,^2^ energy storage,^3^ sensors,^4,5^ and biomaterials.^6^ Atomic-level manipulation and control enable the development of devices with specific functionalities, paving new paths for miniaturization and energy efficiency.^7^ In this class of materials, dimensionality plays a crucial role in determining applications and properties.^8^ In contrast, three-dimensional (3D) materials exhibit more volumetric characteristics,^9^ and two-dimensional (2D) and one-dimensional (1D) materials, such as atomic sheets and nanotubes, offer greater control over electronic confinement and charge carrier mobility.^10^ Carbon, in particular, stands out due to the versatility of its covalent bonding and its capacity to form well-known structures,^11^ including fullerenes,^12^ nanotubes,^13^ graphite,^14^ and, notably, graphene^15^ — a single atomic layer of carbon atoms arranged in a hexagonal lattice with exceptional electrical,^16^ thermal,^17^ and mechanical properties.^18^

Since the advent of graphene, especially following its successful synthesis in 2004,^15^ there has been a significant surge of interest in 2D carbon-based materials.^19^ This development has encouraged extensive research into structures with distinct or analogous properties to those of graphene. Computational simulation, on the other hand, has proven essential in understanding the properties and application potential of new 2D materials.^20−23^ Notably, in recent years, at least four 2D systems have been successfully synthesized: the biphenylene network (BPN),^24^ fullerene network (2D-C_60_),^25^ holey graphyne (HGY),^26^ and γ-Graphyne (γ-GY),^27^ all of which were initially studied theoretically before synthesis.^28−32^ In this context, machine learning (ML) has also emerged as a powerful tool for modeling and predicting the behavior of these nanoscale systems.^33^

Among the wide range of materials proposed in recent years, two independent studies by Wang et al.^34^ and Álvares Paz et al.^35^ introduced a novel monolayer consisting of laterally bonded dehydrogenated naphthalene molecules. Wang and colleagues named this system DHQ-Graphene (DHQ) due to its composition of decagonal (D) pores and hexagonal (H) and quadrilateral (Q) carbon rings.^34^ In their study, Álvares Paz et al. also investigated two potential phases from naphthalene-based molecules 2D systems. One is a configuration combined vertically aligned and 30°-tilted molecules, forming monolayers with 4-, 6-, and 9-membered carbon rings, termed naphthylenes-β. The other configuration considered horizontally aligned naphthalene molecules, identical to Wang’s proposed configuration, resulting in a structure with 4-, 6-, and 10-membered rings, named naphthylenes-α.^35^ Both studies employed Density Functional Theory (DFT) to analyze the physical-chemical properties of these systems.

Álvares Paz et al. further explored quasi-1D configurations (nanoribbons of varying widths), focusing on the structural and electronic properties of these materials. They demonstrated that the DHQ monolayer is metallic, akin to graphene, with zigzag-edged nanoribbons retaining metallic behavior, while narrower armchair-edged configurations exhibited semiconductor characteristics. They also provided detailed insights into the energetic stability of DHQ.^35^ Meanwhile, Wang’s study confirmed DHQ’s stability, showcasing phonon dispersion without imaginary frequencies, ab initio molecular dynamics simulations for thermal stability, and elastic constants satisfying the Born-Huang mechanical stability criterion, with an in-plane stiffness of around 260 N/m. The authors also explored oxygen adsorption properties, obtaining adsorption energies up to −0.3 eV, and concluded by proposing a semiconductor variant of DHQ based on vertically stacked DHQ layers with interplanar distortions.^34^ Together, these works introduce a promising microporous system to the literature, featuring metallic properties and various topological adjustments that render it semiconducting.

Since the introduction of DHQ, further studies have broadened the understanding of this monolayer. Lima and collaborators characterized a similar topology based on boron nitride, assessing its structural, electronic, and optical properties.^36^ Dos Santos et al. examined spin-polarized electronic properties of DHQ-based nanostructures, showing via DFT simulations that DHQ monolayers and derived nanotubes can acquire spin-polarized semiconductor configurations, transitioning from a nonpolarized metallic state.^37^ Additionally, nanotubes may exhibit semiconductor-to-metal transitions under transverse electric fields, demonstrating the potential for nanoelectronic applications due to their tunable responses to external stimuli.^38^ Despite detailed studies on the 2D and quasi-1D topologies of DHQ, primarily regarding their electronic properties, there is no reported research on the mechanical properties of this material, particularly in nanotube topologies, nor large-scale studies addressing fracture patterns. Likewise, the thermal response of DHQ remains unexplored in the literature.

In this work, we conducted a series of ab initio molecular dynamics (AIMD) simulations based on first-principles calculations via DFT to construct a data set of atomic arrangements and energies present in DHQ monolayers under varying degrees of deformation and temperature. This data was employed to develop a machine learning interatomic potential (MLIP) using the Moment Tensor Potential (MTP) framework.^33,39^ The constructed force field underwent rigorous validation and was subsequently applied to investigate the mechanical properties of DHQ in larger-scale systems through classical molecular dynamics (CMD) simulations, including both 2D (monolayer) and quasi-1D (nanotube) topologies, which are beyond the feasible scales for DFT. Additionally, we examined the temperature response of the nanomaterial. Our results indicate that depending on chirality and tube diameter, Young’s modulus of DHQ nanotubes varies from 127 to 243 N/m, with fracture occurring between 13.6 and 17.4% strain relative to the initial length. Finally, we show that DHQ retains its structural integrity up to a thermal bath temperature of 2200 K, undergoing a phase transition beyond this threshold. The findings reported here contribute to a comprehensive understanding of a planar nanoporous carbon-based material and its underlying nanotubes and open new research avenues by providing a machine learning-based interatomic potential that enables new perspectives for CMD simulations, expanding the applications and insights into potential applications with DHQ.

## 2 Methodology

A rigorous and widely established methodological approach from the literature was employed to investigate the intrinsic properties of DHQ-based monolayer and nanotubes with varying chirality and diameters and their corresponding comparisons in the two-dimensional topology. This process encompasses the initial modeling of the systems, assessment of their structural stability, construction of a force field using machine learning based on data derived from first-principles calculations, and validation of the resulting interatomic potential. Additionally, this section provides a detailed description of the parameters and methods applied in each stage of the analysis of the mechanical and thermal properties of the nanomaterials under investigation.

### 2.1 System Modeling

**Figure 1:**
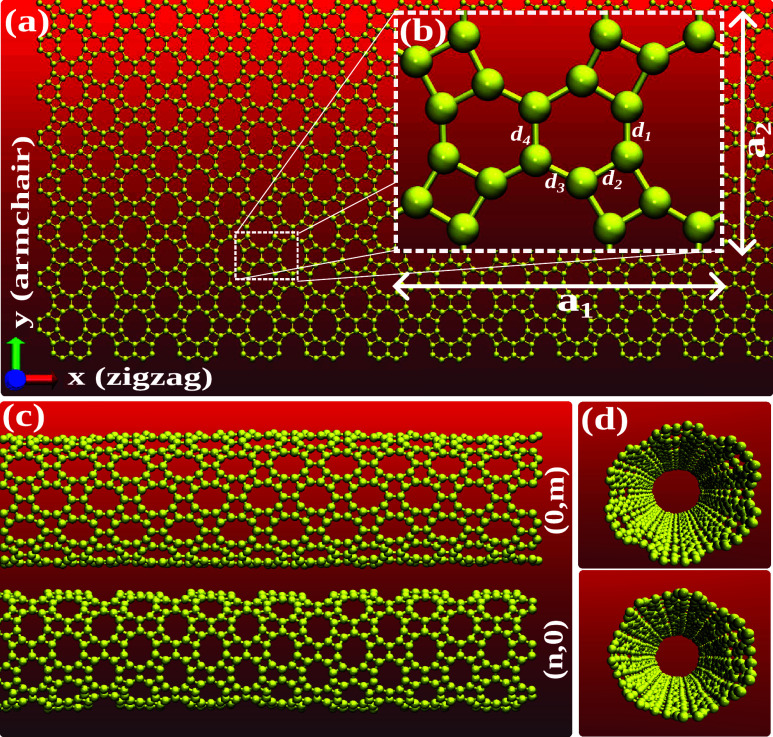
Schematic representation of the DHQ monolayer (a), highlighting the zigzag and armchair directions, and the unit cell (b) of the system with lattice vectors **a**_1_ and **a**_2_, showing the atomic bond lengths *d*_1_, *d*_2_, *d*_3_, and *d*_4_. Examples of DHQ nanotubes with armchair and zigzag chirality are shown in lateral (c) and front (d) views.

The DHQ is a planar system, entirely composed of carbon atoms, which can be understood as the lateral union, after dehydrogenation, of naphthalene molecules.^34,35^ The unit cell of the system is orthorhombic, with the CMMM symmetry group and Schoenflies^40^ name D2H-19, containing 20 carbon atoms, with sp^2^ hybridization, distributed in 4, 6, and 10-membered carbon rings. The investigated DHQ topologies and their unit cell are presented in Figure 1. In panel 1(a), the DHQ monolayer schematic representation is shown. In the horizontal direction, *x*, indicated by the red axis, is the zigzag termination of the naphthalene molecule, which coincides with that of graphene.^41^ The same applies to the vertical direction of the material plane, highlighted by the green arrow of the Cartesian axis, which will be referred to as an armchair here. In Figure 1b, the DHQ unit cell is shown, with the in-plane lattice vectors denoted by **a**_**1**_ and **a**_**2**_. Essentially, there are four different atomic types in the nanomaterial, which results in four different bond lengths in the system, considering the zero temperature in geometry optimization. From this, we have that *d*_1_ represents the bond lengths connecting the 4-membered rings, *d*_2_ those shared by 4- and 6-carbon atom rings, *d*_3_ the bond length between 6- and 10-membered rings, and finally, *d*_4_, representing the bond lengths shared between two adjacent benzene rings.

To model the DHQ nanotubes, we initially define a chiral vector **C**_*h*_ = *n***a**_1_ + *m***a**_2_, where **a**_1_ and **a**_2_ are orthogonal lattice vectors of the system, and *m* and *n* are integers. This vector corresponds to the rolling direction of the monolayer, also determining the nanotube circumference length (diameter), which, in addition to being a function of *n* and *m*, is given by the expression *d*_(*n*,*m*)_ = |**C**_*h*_|/π. It is also necessary to define a translational vector **T** = *p***a**_1_ + *q***a**_2_ (where *p* and *q* are integers), which corresponds to the shortest vector orthogonal to **C**_*h*_, generating the replications in the system, thus defining the longitudinal length of the generated nanotube. Note that a necessary condition to obtain *p* and *q* is **C**_*h*_ · **T** = 0, i.e., (*n***a**_1_ + *m***a**_2_) · (*p***a**_1_ + *q***a**_2_) = 0. Since **a**_1_ ⊥**a**_2_, the relationship between *n*, *m*, *p*, and *q* in the construction of nanotubes is given by *np*|**a**_1_|^2^ + *mq*|**a**_2_|^2^ = 0.^42^ The solutions to this model only make sense if *m* and *n* are not simultaneously zero. If *n* = 0, then **C**_*h*_ = m · **a**_2_, which corresponds to a nanotube with *d*_(0,*m*)_ = *m*^2^·|**a**_2_|^2^/π, with an armchair edge. When *n* = 0, it follows that *q* = 0, so *p* can take any integer value. With **T** defined as the shortest possible vector, so *p* = 1, which gives **T** = **a**_1_. Similarly, for *m* = 0, them a nanotube with a zigzag edge, with *d*_(*n*,0)_ = *n*^2^·|**a**_2_|^2^/π and **T** = **a**_2_.^43,44^ Chiral nanotubes can be obtained, even with reasonable approximations, for the ratio *p*/*q* ≈ – (*m*/*n*)(|**a**_2_|/|**a**_1_|), which is only possible for considerably high integers *p*, *q*, *m*, *n*, resulting in nanotubes with considerably large diameters.^45^ Even with techniques that allow calculating systems with thousands of atoms, such as classical molecular dynamics, nanotubes with huge diameters are essentially similar in their physical properties to their two-dimensional topology. Therefore, here we consider only nanotubes with armchair and zigzag chiralities. In our approach, we consider values of *n* (*m*) from 2 to 10, along with an extrapolated case of 20, corresponding to a diameter variation from approximately 4 to 60 Å, with the number of atoms ranging from 480 to 6400. Figure 1c,d presents the lateral and front views of nanotubes with zigzag (0, *m*) and armchair (*n*, 0) chiralities.

### 2.2 Classical Reactive Molecular Dynamics Simulations

Given the quasi-1D nature of the DHQ-based systems investigated here, with supercells reaching up to 6400 atoms, ab initio simulations become impractical. On the other hand, molecular dynamics (MD) simulations present a viable computational approach to investigate atomic dynamics on time and spatial scales that are not easily accessible via first-principles methods despite their relatively lower accuracy. In MD, atoms and molecules are treated as classical particles, governed by Newton’s equations of motion, where for each particle *i*, these equations are expressed as
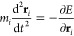
1where *m*_*i*_ represents the particle’s mass, **r**_*i*_ is its position, and *E* is the system’s total potential energy. The potential energy *E* is modeled by interatomic potentials that describe interactions, including bonded terms (e.g., bond stretching, angle bending, etc.) and nonbonded terms (e.g., van der Waals and Coulombic interactions). The choice of force field plays a crucial role in MD simulations, as it dictates the accuracy of the results in replicating the physical properties of materials.

Numerically, we employed the software Large-scale Atomic/Molecular Massively Parallel Simulator (LAMMPS)^46,47^ to perform fully atomistic MD simulations. The equations of motion were numerically integrated using the velocity-Verlet algorithm^48−50^ with a time step of 1 × 10^–4^ ps. Before any specific simulation process, all DHQ-based nanostructures were optimized at 0 K to reach a minimum energy configuration with zero pressure along the periodic directions. The systems were then equilibrated in an isothermal–isobaric ensemble (NPT) with constant particle number, pressure, and temperature, where the temperature was maintained at 300 K for 100 ps, and the pressure was kept at zero using a Nosé–Hoover thermostat and barostat.^51^ A second preparation stage was applied, again at 300 K, but with the canonical (NVT) ensemble, fixing the volume rather than the pressure, as was done initially.

For the mechanical analysis, uniaxial tensile deformation was applied along the nanotube’s longitudinal axis (and both principal axes of the plane in the case of two-dimensional systems), with an engineering strain rate of 1.0 × 10^–6^ fs^–1^. During the uniaxial deformation process, the direction perpendicular (just in 2D topology) to the strain was integrated with the NPT ensemble to measure the Poisson effect.^52^

Thermally, the DHQ monolayer was systematically evaluated at incremented temperatures from 100 K to higher values, using an NVT ensemble, over 200 ps (for each temperature). The system’s average total energy was evaluated in the last 100 ps of the simulation until a nonlinear variation in the energy response with temperature increase was observed, allowing the calculation of the specific heat of DHQ and the critical phase transition temperature of the nanomaterial.

For the DHQ-based system modeled in this study, reactive force fields such as (AI)REBO,^53,54^ Tersoff,^55^ and ReaxFF^56−59^ were applied. However, in all cases, the system yielded insufficient results in terms of structural characteristics and mechanical properties when compared with first-principles calculations, indicating that these interatomic potentials were not scalable for DHQ. Consequently, we trained a machine learning-based interatomic potential specific to DHQ, derived from first-principles calculations, which will be described in detail in the next section.

### 2.3 Machine Learning Force Field

To investigate the intrinsic physical properties of DHQ monolayers and nanotubes using classical MD, a machine learning-based approach was employed to derive a parametric force field specifically for DHQ, as existing empirical potentials were found to be nonscalable for this nanomaterial. The training was conducted using a database constructed from *ab initio* calculations. All details of the process are thoroughly discussed in this section.

#### 2.3.1 Database Construction via DFT Calculations

For the simulation of DHQ-based systems on scales larger than feasible with the DFT formalism, we built a data set through AIMD simulations. The calculations were performed using the Vienna Ab initio Simulation Package (VASP),^60^ employing the generalized gradient approximation (GGA) with the Perdew–Burke–Ernzerhof (PBE)^61^ functional for exchange-correlation and the Projected Augmented Wave (PAW) method for electron–ion interactions.^62^ A Monkhorst–Pack^63^ 2 × 2 × 1 grid was applied, with a cutoff energy of 500 eV, a convergence criterion of 10^–5^, and force components configured at 0.01 eV Å^–1^. For the DHQ structure, AIMD simulations were conducted on 2 × 2 × 1 supercells (80 atoms). The integration of Newton’s equations of motion used the Velocity-Verlet algorithm.^50^ Given the highly correlated data, temperature and structural deformation were varied using a time step of 1.0 fs with 500 simulation steps for each case. All simulations employed an NPT ensemble to ensure zero pressure in the undeformed directions, allowing system volume adjustments at different temperatures. Length constraints were applied only along the deformation directions. Temperature and pressure control were achieved using the Langevin thermostat and barostat.^64^ For structural deformation, strains varied from −15% (compression) to 15% (tension) in 5% increments, independently for the *x* and *y* directions, totaling 13 distinct simulation sets. AIMD simulations were conducted at varying temperatures from 300 to 1000 K. Additionally, a van der Waals interaction correction was included in all simulations. This process has been previously validated in the literature.^65^

Following this stage, the minimum interatomic distances were calculated throughout the dynamics using the MLIP method, and this value was changed in the MTP file. Various orders of Chebyshev polynomials^66^ were tested to determine the optimal response for the required task, and similarly, the MTP levels were chosen based on this.

The polynomial degrees were restricted according to the MTP level to minimize computational costs while maintaining model accuracy. For higher MTP levels, lower polynomial degrees were employed to assess the effectiveness of each trained force field, ensuring that the degrees did not fall below 6.

Once the AIMD training data set was generated using VASP, it was divided into 20% for validation and 80% for training. A 10% sample was initially used for training from the training data set, followed by retraining with the remaining data. The most suitable and nonredundant data points were selected based on the initial force field generated using the MLIP method. Potential parameters, such as le*v*_max_ and the number of basis functions *N*_Q_, were fine-tuned to achieve maximum precision while ensuring computational efficiency. This process resulted in selecting a potential that minimized the mean difference frequency when comparing the calculated and reference phonon spectra. This study selected the MTP with lev_max_ set to 26 and Chebyshev polynomials of degree 8. Additional intrinsic details regarding the machine learning training process are provided in the subsequent section.

#### 2.3.2 Force Field Training

An interatomic potential based on machine learning was developed using MTP.^33,67^ In this method, the potential energy of the system *E*_mtp_(cfg) for a specific atomic configuration cfg is obtained by summing the contributions of the local environments of individual atoms, as given by the expression:
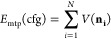
2where cfg represents a specific set containing each atom’s positions, atom types, and local environment in the system. The function *V*(**n_i_**) represents the energy associated with the environment of atom *i* and can be linearly expanded in terms of basis functions *B*_α_, as follows:
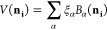
3where ξ_α_ are adjustable parameters determined during the training process.

The MTP formulation utilizes moment tensors *M*_μ_, ζ to capture atomic neighborhoods’ angular and radial characteristics. The radial part is described by a basis function *Q*^(β)^(|**r**_**ij**_|), defined as
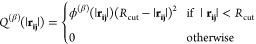
4where *R*_cut_ is the cutoff radius, ensuring a smooth decay of the function as the neighborhood limit is reached. The angular part is modeled by tensors of degree ζ using repeated outer products of position vectors **r_ij_** between atoms.

The training process, known as passive learning, involves fitting the parameters θ = {ξ_α_, *c*_μ_^(β)^} to the training data by minimizing the following objective function:
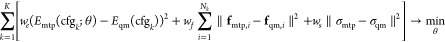
5where *w*_*e*_, *w*_*f*_, and *w*_*s*_ are weights that regulate the importance of energies, forces, and stress tensors, respectively.

This process involved the following steps: (1) initial generation of configurations for training, (2) partitioning of data into training and validation sets, (3) initial training of the force field with 10% of the training configurations using MLIP, (4) selection of nonredundant training configurations, (5) retraining of the force field by adding selected configurations, and (6) validation of the field by calculating the mean squared error in the predictions relative to the validation data.

#### 2.3.3 Force Field Validation

The first evaluation of the trained force field was based on calculating the mean squared errors against the training and validation data sets, which served as the initial step to assess the field’s capability in describing the material’s intrinsic properties. Fields with errors exceeding the expected range were discarded.

Subsequently, to verify the accuracy of the trained force field, we calculated the phonon dispersion of DHQ using the PHONOPY software,^68^ applying the DFT formalism. This dispersion calculation was also performed for DHQ using the trained force field for comparison. Since DHQ does not exhibit imaginary vibrational modes, any force fields that produced such modes were discarded. Only fields that reported exclusively real vibrational modes and closely matched the DFT results were retained.

## 3 Results

This section presents a detailed analysis of the results obtained from the construction of the force field, its validation, and its application in calculating the mechanical and thermal properties of DHQ.

### 3.1 Force Field Validation and DHQ Structural Properties

**Figure 2:**
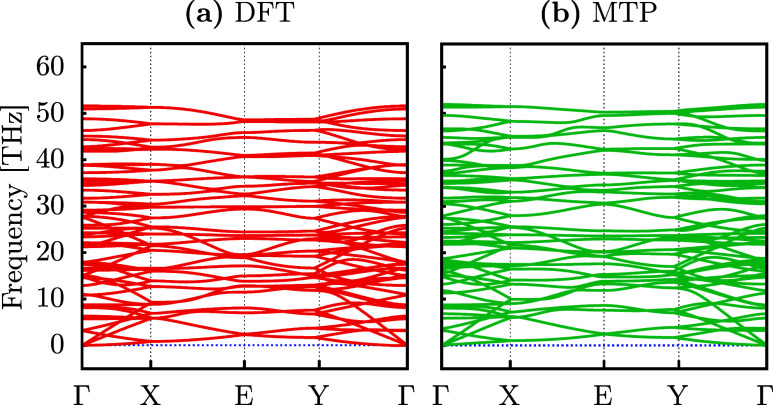
Phonon dispersion for the DHQ monolayer obtained from DFT calculations (a) and using the MTP-trained interatomic potential (b).

To assess the accuracy of the MLIP for DHQ, we compared the phonon dispersion obtained from first-principles calculations and that derived from the force field finalized through training on AIMD data. Figure 2 displays the results from both methodologies: in Figure 2a, the dispersion obtained using DFT is shown, while Figure 2b presents the same calculation based on the MTP-trained model. Notably, both trends are very similar, especially within the acoustic vibrational modes. A slight difference is observed in the higher-frequency optical modes, where DHQ exhibits maximum optical vibrational modes at 51.6 THz and 51.9 THz for DFT and MTP, respectively. The results align well with those reported by Wang and co-workers in their initial DHQ study, which indicated a maximum frequency of 52.8 THz for the optical modes. Notably, the absence of imaginary frequencies in the dispersion confirms the system’s dynamic stability and highlights the accuracy of the ML-derived force field.

While this validation underscores the reliability of the MLIP for dynamic properties, it is also worth considering its potential for exploring structural aspects, such as ring formation energies. Training the ML model specifically for the formation of each ring size would require a significant number of additional DFT calculations to enrich the training data set. However, ML models have been shown to provide a good compromise in predicting ring formation energies, as evidenced by studies comparing classical reactive and ML-based potentials. These findings further demonstrate the versatility of the developed MLIP in describing complex carbon-based structures efficiently.^69,70^

Once the system was confirmed to be stable, we analyzed its structural relations, disregarding temperature effects at this stage. For comparison purposes, we performed the optimization of the unit cell (see Figure 1b). From DFT calculations, we obtained the lattice vectors |**a**_1_| = 8.98 Å and |**a**_2_| = 6.69 Å, values consistent with those reported in the literature.^34^ Regarding the bond lengths, we obtained *d*_1_ = 1.38 Å, *d*_2_ = 1.46 Å, *d*_3_ = 1.40 Å, and *d*_4_ = 1.51 Å. These bond lengths also match those reported in the literature.^34^ For the classical optimization performed using the MLIP, we obtained |**a**_1_| = 8.98 Å and |**a**_2_| = 6.67 Å, with *d*_1_, *d*_2_, *d*_3_, and *d*_4_ equal to 1.37, 1.46, 1.40, and 1.50 Å, respectively. For comparison, optimization performed using the ReaxFF potential^56^ yielded |**a**_1_| = 10.38 Å and |**a**_2_| = 6.93 Å, with bond lengths *d*_1_ = 1.38 Å, *d*_2_ = 1.52 Å, *d*_3_ = 1.42 Å, and *d*_4_ = 1.45 Å, in addition to another bond in the 4-member ring, which appeared as a square with DFT and MLIP, but as a rectangle with ReaxFF (1.52 × 1.48 Å^2^), yielding a deviation of approximately 16% in the lattice vectors and nearly 5% in the bond lengths.

We varied the diameter for the nanotubes by replicating the unit cell along the direction of interest. The number of replications varied between 2 and 10 along both directions, with an extrapolated case involving 20 replications to study a nanotube with a considerably large diameter. Since the larger the nanotube diameter, the smaller the curvature effect, the system behaves similarly to the 2D system. Thus, we aimed to determine the limit at which curvature can no longer influence mechanical properties. For armchair (*n*, 0) nanotubes, the diameters range from 4.25 to21.24 Å for *n* values between 2 and 10, while for *n* = 20, the nanotube diameter is *d*_(20,0)_ = 42.47 Å. For all armchair cases, *p* = 12 replications of the translational vector were used to ensure that all nanotubes have at least 100 Å length. After optimization, we observed that curvature causes an elongation of the nanotube length in armchair configurations, where for *n* = 2, the length is 110 Å, and for *n* = 3, it is 109 Å. It decreases very slowly for *n* ≥ 4, with lengths of 108.4 and 107.8 Å for *n* values of 4 and 20, respectively. The number of atoms in the system can be expressed as *N*_(*n*,0)_ = 240 · *n*, varying from 480 to 4800 atoms. For zigzag (0, *m*) chirality, the diameters vary from 5.7 to28.6 Å for *m* values from 2 to 10, and for *m* = 20, nanotube has *d*_(0,20)_ = 57.2 Å. Unlike the armchair case, the zigzag chirality does not exhibit significant changes in length due to curvature, remaining around 106.6 Å in all cases. For the translational vector, *q* = 16 replications were used, with the number of atoms varying according to the expression *N*_(0,*m*)_ = 320 · *m*, ranging from 640 to 6400 atoms. All the structural details of the nanotubes are summarized in Table 1.

**Table 1:** Geometric Characteristics of DHQ Nanotubes after Optimization at 0 K Temperature

**(*n*,0)-DHQ-NT**				**(0,*m*)-DHQ-NT**			
	*N*	*D* [Å]	*L* [Å]		*N*	*D* [Å]	*L* [Å]
**(2,0)**	480	4.25	110.03	**(0,2)**	640	5.72	106.57
**(3,0)**	720	6.37	109.04	**(0,3)**	960	8.58	106.63
**(4,0)**	960	8.49	108.45	**(0,4)**	1280	11.44	106.69
**(5,0)**	1200	10.62	108.22	**(0,5)**	1600	14.30	106.72
**(6,0)**	1440	12.74	108.08	**(0,6)**	1920	17.16	106.73
**(7,0)**	1680	14.87	107.98	**(0,7)**	2240	20.02	106.74
**(8,0)**	1920	16.99	107.94	**(0,8)**	2560	22.88	106.74
**(9,0)**	2160	19.11	107.91	**(0,9)**	2880	25.74	106.74
**(10,0)**	2400	21.24	107.89	**(0,10)**	3200	28.59	106.74
**(20,0)**	4800	42.47	107.82	**(0,20)**	6400	57.19	106.75

### 3.2 Mechanical Properties

Before evaluating the mechanical properties of large-scale DHQ systems, including monolayers and nanotubes, we calculated the elastic constants of DHQ monolayers using DFT, based on the energy-deformation approach,^71^ to compare our results with those from CMD.

#### 3.2.1 DFT Calculations

For crystalline solids with orthorhombic symmetry, the total deformation energy is expressed as^72^
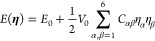
6where *E*_0_ is the energy of the equilibrium structure, *V*_0_ is the unit cell volume, *C*_αβ_ are the independent elastic constants, and η_α_ represents the components of the Lagrange strain tensor {**η**} in the Voigt notation.^73^

In the case of two-dimensional systems with rectangular symmetry, the total deformation energy is rewritten as^74^
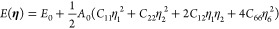
7where *A*_0_ is the area of the unit cell, and the coefficients *C*_11_, *C*_22_, *C*_12_, and *C*_66_ represent the independent elastic constants for the monolayer.

The components η_α_ refer to specific deformations, where η_1_ represents the deformation along the *x* direction (uniaxial horizontal elongation or compression), η_2_ represents the deformation along the *y* direction (uniaxial vertical elongation or compression), and η_6_ describes the shear deformation in the *xy* plane.

In this approach, the elastic constants are directly determined from the second derivative of the total energy concerning the deformation:^75,76^
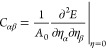
8

Different types of deformation were applied to calculate the elastic constants. For uniaxial deformation, with η_1_ = η and η_2_ = 0 along the *x* direction, using the expression:
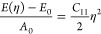
9and for the *y* direction, with η_1_ = 0 and η_2_ = η
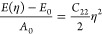
10For biaxial deformation, with η_1_ = η_2_ = η, the equation becomes
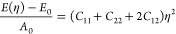
11Finally, for shear deformation, with η_6_ = 2η, we obtain
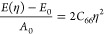
12

The calculations were performed by varying η in uniform intervals of Δ*η* = 0.005 within a deformation range from η = −0.03 to 0.03. The total energy was calculated for each value of η, and the results were fitted to a quadratic polynomial in η. This approach allowed for precisely determining the coefficients *C*_αβ_.

The obtained values for the elastic constants of the DHQ monolayer are *C*_11_ = 230.7 N/*m*, *C*_22_ = 299.4 N/*m*, *C*_12_ = 61.3 N/*m*, and *C*_66_ = 82.7 N/*m*. These values reflect the anisotropy of the DHQ monolayer and are consistent with the expected properties of two-dimensional materials with rectangular symmetry. Furthermore, the values of the elastic constants obtained here also satisfy the Born–Huang criterion, further indicating the stability of DHQ, as previously demonstrated by Wang et al.^34^

With these constants established, we can easily calculate Young’s modulus (*Y*(θ)) and Poisson’s ratio (ν(θ)) using the following relations:^77,78^
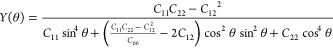
13and
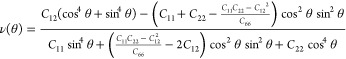
14where θ denotes the in-plane direction relative to the *x* axis.

**Figure 3:**
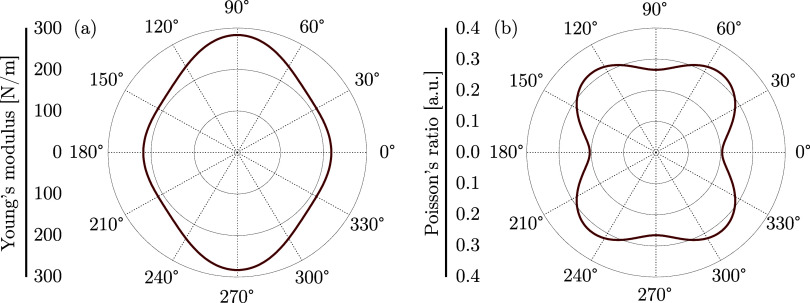
Young’s modulus (a) and Poisson’s ratio (b) for DHQ monolayers as a function of the in-plane deformation angle relative to the horizontal axis.

In Figure 3, Young’s modulus (a) and Poisson’s ratio (b) are shown as functions of the deformation angle θ in the plane, relative to the horizontal direction of DHQ (see Figure 1). The results demonstrate variations in both properties, confirming the mechanical anisotropy of the nanomaterial. Young’s modulus exhibits values of 212.1 to 283.2 N/m. The average value across all angles is ⟨*Y*⟩ = 233.8 N/m, with a standard deviation of σ_*Y*_ = 24.4 N/m, indicating moderate anisotropy. The minimum values of *Y* occur at θ = 30°, while the maximum values are found at θ = 90° (the *y*-direction). These extrema repeat periodically over the angular interval, with characteristic variations every 90°, consistent with the tetragonal symmetry associated with the *C*_4*v*_ point group observed in the atomic arrangement of DHQ. Another direction of interest is the horizontal direction, θ = 0°, where *Y* = 218.1 N/m.

The geometry of the DHQ structure strongly influences its hardness. As shown in Figure 1, despite being constructed from naphthalene molecules, the DHQ monolayer adopts a topology analogous to BPN nanosheets along two oblique directions relative to the horizontal axis. The square rings contribute significantly to the system’s rigidity in this configuration. In one case, their bonds are oriented at 30 and 60° concerning the *x*-axis, including their respective rotations. When the applied deformation aligns at 30°, the minimum Young’s modulus is observed, as this direction is directly perpendicular to the topology of the BPN-like molecular structure, where the square ring bonds are parallel. This topology explains the lower hardness in this direction, as no significant shearing occurs in these rings, resulting in an expansion of the bonds connecting the topological regions related to the BPN-like structure.

Furthermore, deformation applied at 60° also involves square ring bonds parallel to the deformation direction but with a longitudinal alignment to the BPN molecule arrangement. This configuration is notably stiffer than the deformation applied perpendicularly to these regions of DHQ. The Young’s modulus of BPN along the analogous 60° orientation is 259.7 N/m,^79^ compared to 238.7 N/m for DHQ. This reduction is attributed to the higher porosity of the DHQ structure.

As expected, ν exhibits anisotropic behavior, ranging from 0.21 to 0.32. The calculated average is ⟨ν⟩ = 0.28, with a standard deviation of σ_ν_ = 0.038, indicating moderate variation across angles. The minimum value of ν occurs at θ = 0° (the *x*-direction), corresponding to directions of higher elastic stiffness. In contrast, the maximum value is approximately θ = 50°, coinciding with directions of lower stiffness, where Young’s modulus is reduced. Similar to Young’s modulus, the variations in ν also exhibit a periodicity of 90°, corroborating the structural symmetry of DHQ. The Poisson’s ratio in the *y*-direction (vertical) is 0.27.

#### 3.2.2 MLIP-CMD Simulations

CMD simulations were performed to investigate the stress–strain behavior of the DHQ monolayer using the MLIP force field discussed and validated in the previous section. Furthermore, the complete protocol and parameters used in these simulations are described in Section 2. The atomic-level stress components were calculated based on the virial theorem and normalized by the in-plane area *A*_0_:
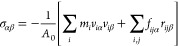
15where *m*_*i*_ is the mass of atom *i*, *v*_*i*α_ and *v*_*i*β_ are velocity components, *f*_*ij*α_ is the interatomic force between atoms *i* and *j*, and *r*_*ij*β_ is the displacement between atoms *i* and *j*. The first term represents the kinetic contribution, while the second represents interatomic interactions.

Uniaxial deformations were applied separately along the simulation box’s *x* and *y* directions to analyze the mechanical properties. The strain in each direction was defined as η_α_ = Δ*L*_α_/*L*_α0_, where *L*_α0_ is the initial length of the simulation box in the α-direction (α = *x*, *y*), and Δ*L*_α_ = *L*_α_ – *L*_α0_ is the corresponding change in length, with *L*_α_ being the deformed system length in the α-direction. The stress–strain relationship in the linear regime follows Hooke’s law σ_α_ = *Y*_α_ η_α_, where *Y*_α_ is the Young’s modulus in the α-direction.

**Figure 4:**
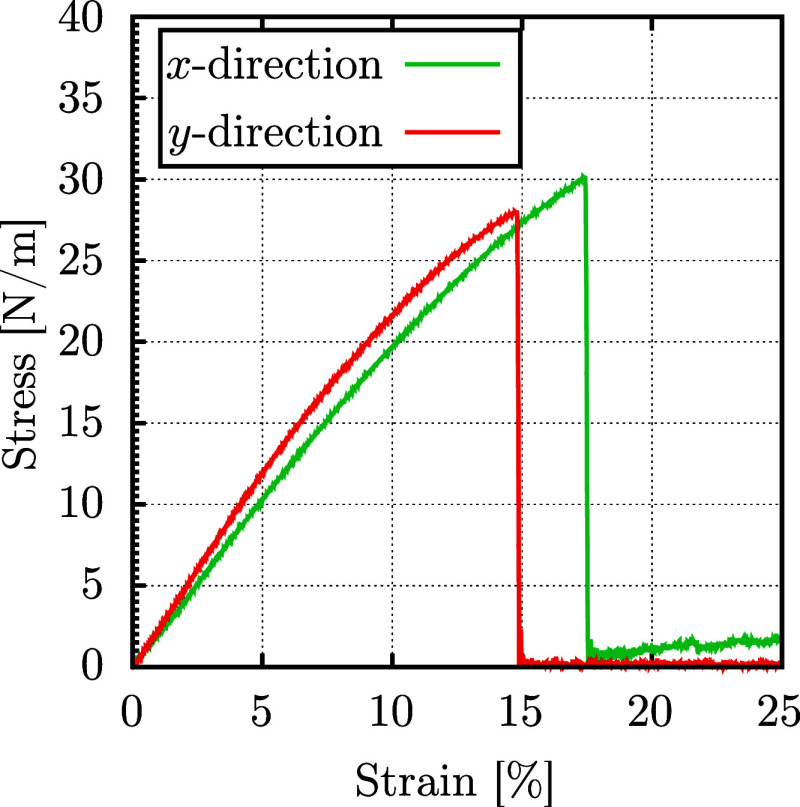
Stress–strain curve of the DHQ monolayer, with uniaxial deformation applied separately along the *x*-direction (green) and the *y*-direction (red).

Figure 4 illustrates the mechanical response of a 12 × 16 × 1 DHQ supercell containing 3840 atoms. The stress–strain response under uniaxial deformation applied separately along both directions exhibits the typical behavior of crystalline solids characterized by brittle fracture. This behavior aligns with the lower ν values observed in these directions, leading to an abrupt system failure. From the stress–strain curves, we obtained Young’s modulus of 202.2 N/m in the *x*-direction and 243.2 N/m in the *y*-direction. These values are consistent with DFT predictions of 218.1 and 283.2 N/m in the *x*- and *y*-directions, respectively.

It is worth noting that the elastic modulus values employed in MD simulations using MLIP account for temperature effects, which are known to weaken materials. Since these simulations were performed at room temperature, the observed differences in the mechanical characteristics of DHQ between energy-deformation and stress–strain models with temperature effects are relatively small. Additionally, CMD calculations reveal critical strain values of 17.4 and 14.8% for the *x* and *y* directions, respectively, with corresponding critical stresses of 30.2 and 28.1 N/m.

The system’s anisotropy has been previously discussed in the context of DFT under inclined deformations at 30 and 60° with respect to the *x*-axis. For the deformations investigated here in the *x* and *y* directions via CMD, this anisotropy can also be understood based on the initial geometry of the nanomaterial. This characteristic is more evident when analyzing snapshots of the system near its critical fracture point.

**Figure 5:**
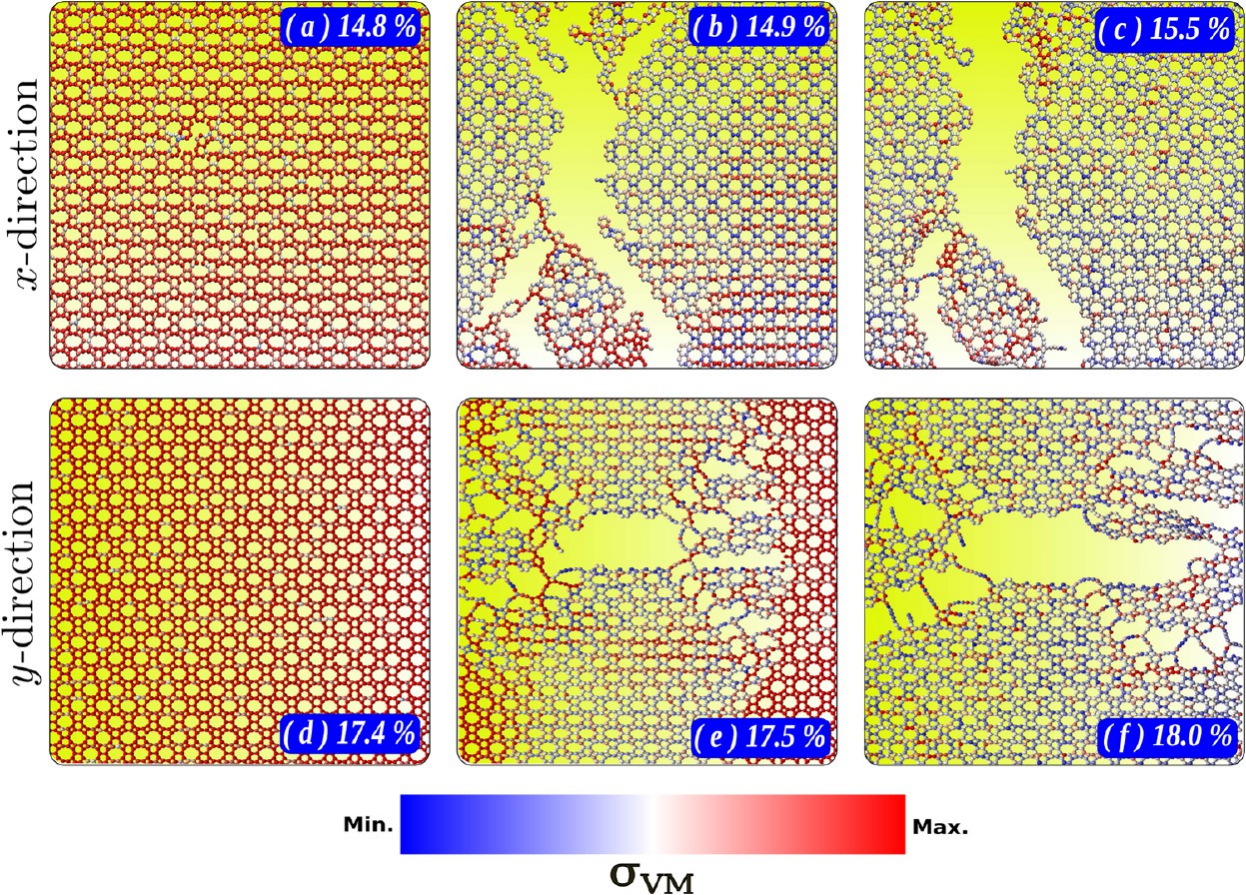
Snapshots of the DHQ monolayer deformation at 17.4% (a), 17.5% (b), and 18.0% (c) along the *x*-direction, and 14.8% (d), 14.9% (e), and 15.5% (f) along the *y*-direction. The color map represents the normalized von Mises stress distribution.

Figure 5 displays snapshots directly associated with the fracture process of DHQ monolayers. Panels 5a–c show snapshots under strain in the *x* direction at 17.4, 17.5, and 18.0%, respectively. Meanwhile, panels 5d–f present snapshots under strain in the *y* direction, with the systems stretched to 14.8, 14.9, and 15.5% of their initial size, respectively. The BWR color map represents the Von Mises stress σ_VM_^*k*^^80^ for each atom *k*, calculated using the expression:
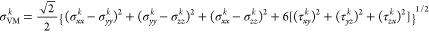
16where σ_*xx*_^*k*^, σ_*yy*_^*k*^, and σ_*zz*_^*k*^ are the normal stress components, and τ_*xy*_^*k*^, τ_*yz*_^*k*^, and τ_*zx*_^*k*^ are the shear stress components. The Von Mises stress allows for identifying fracture points or regions within the structure, providing insights into the material’s failure mechanisms.

Broadly, as previously discussed for cases involving 30 and 60° deformation, the DHQ structure can be understood as a lattice system of naphthalene units covalently bonded by four-membered rings. This geometry introduces nanoporosity through a 10-membered ring. The nanopore has an approximate width of 3.9 Å and height of 5.2 Å, which can be modeled as an ellipse with an eccentricity of 0.62 and its principal axis aligned along the *y* direction. Given the tetragonal symmetry and rigidity of DHQ, the nanoporosity of the 10-membered ring is directly related to the material’s hardness.

Under uniaxial strain in the *x* direction, the perpendicular expansion of the 10-membered ring occurs, a process that is structurally and energetically less costly than the opposite case, where strain is applied in the *y* direction, further compressing the nanopore. These effects are evident in panels 5a,d, where the ellipses are less eccentric under *x* direction strain. At the same time, no significant compression is observed compared to the initial state under *y* direction strain. These factors lead to higher stress accumulation under *y* strain, resulting in a larger Young’s modulus and relatively earlier fracture due to stress accumulation. This is further corroborated by the fact that ν is more significant in the *y* (0.27) than the *x* direction (0.21).

Furthermore, the fracture pattern in DHQ is quite specific. Under *x* direction strain, as shown in Figure 5b, a mere 0.1% additional strain is sufficient for the system to fracture almost entirely. This fracture initiates centrally at the hexagonal rings, propagating cracks diagonally and breaking primarily the more rigid four-membered rings, which accumulate higher stress (Figure 5c). In the case of *y* direction strain, the process is also brittle but exhibits greater preservation of hexagonal rings (Figure 5e). The rupture begins with dissociation in the four-membered rings due to stress accumulation, propagating nanocracks diagonally from the central fracture. The predominant fracture of four-carbon rings in the *y* direction corroborates the observation of lower critical strain (and stress accumulation) in this direction. In all cases, after the system fractures, small linear atomic chains (LACs) are formed due to the reconfiguration of atomic bonds (Figure 5c,f).

To evaluate the effect of curvature on the mechanical properties of DHQ, we investigated the rolling of the monolayer along two distinct directions. By replicating the unit cell along the *x* direction and performing self-rolling, a zigzag edge chirality (0, *m*) is obtained, which, when uniaxially deformed, corresponds to tension along the *y* direction of the monolayer. Conversely, rolling the unit cell along the *y* direction results in armchair chirality (*n*, 0), equivalent to longitudinal tension along the *x* direction of the monolayer.

**Figure 6:**
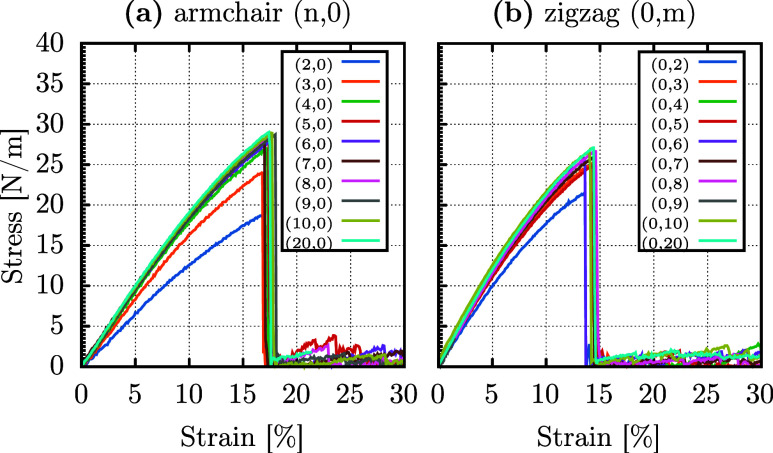
Stress–strain curves for DHQ nanotubes under longitudinal tension, with varying diameters, for (*n*, 0) armchair (a) and (0, *m*) zigzag (b) chiralities.

Figure 6 shows the stress–strain curves for DHQ nanotubes with varying diameters and chiralities (*n*, 0) armchair (Figure 6a) and (0, *m*) zigzag (Figure 6b). It is worth noting that all investigated chiralities and diameters were stable, showing no phase transitions at room temperature using simulations in NVT and NPT ensembles.

For the armchair case, the smallest diameter investigated was *d*_(2, 0)_ = 4.3 Å. As discussed earlier, the nanotube’s unit cell lengthened along the direction equivalent to the monolayer, and the system demonstrated significantly higher flexibility compared to the monolayer, with Young’s modulus of approximately 127 N/m, representing a reduction of nearly 38%. However, all nanotube systems examined exhibited no significant differences in their critical strain. For the (2, 0) DHQ nanotube, the critical strain was 17% (compared to 17.4% for the 2D system), occurring at approximately 19 N/m of critical stress, consistent with the same 38% reduction relative to the 2D system.

Increasing the replication to *d*_(3, 0)_ = 6.4 Å, Young’s modulus increased to 167.4 N/m, with a critical stress of 24.1 N/m, and a fracture occurring at the same critical strain of around 17%, as in the monolayer. The nanotubes revealed almost no significant changes in elastic constants for (*n*, 0) systems with *n* ≥ 4, equal in the structural optimization calculations. The Young’s modulus for these nanotubes ranged between 187 and 200 N/m. In all armchair cases, slight variations in ultimate stress were observed, with values around 29 N/m, compared to 30 N/m for the monolayer.

Thus, *n* = 3 is the critical number of replications for transitioning from quasi-1D behavior to 2D-like behavior in terms of Young’s modulus and critical stress, with no substantial differences in the strain percentage required for complete fracture of the nanotubes. Figure 6a also reveals minor stress fluctuations below 5 N/m after the systems’ critical strain. As observed for the monolayer, DHQ nanotubes exhibit brittle fracture, with the formation of small LACs during the process before complete separation.

A similar trend is observed for the zigzag case but is less pronounced. Smaller diameters, *d*_(0, 2)_ = 5.7 Å and *d*_(0, 3)_ = 8.6 Å, exhibit Young’s moduli of 206 and 227 N/m, respectively, representing reductions of 15 and 7% compared to the 243 N/m of the 2D system. This less pronounced reduction is associated with the smaller curvature of zigzag-edge nanotubes, which have higher diameters for *n* = *m*. For *m* ≥ 4, the values converge to those reported for the monolayer, with Young’s moduli ranging from 235 to 243 N/m and critical stress between 25.9 and 27.1 N/m. As in the armchair case, curvature does not significantly influence the critical strain. All the elastic properties of DHQ nanotubes are summarized in Table 2.

**Table 2:** Elastic Constants (Young’s modulus (*Y*), Critical Strain (η_*C*_), and Critical Stress (σ_*C*_)) for DHQ Nanotubes with Varying Diameters and Chiralities

(*n*, 0)	*Y* [N/m]	η_C_ [%]	σ_C_ [N/m]	(0, *m*)	*Y* [N/m]	η_C_ [%]	σ_C_ [N/m]
**(2,0)**	126.9	17.0	18.9	**(0,2)**	206.0	13.6	21.6
**(3,0)**	167.4	16.8	24.1	**(0,3)**	227.5	14.2	25.1
**(4,0)**	186.8	17.1	27.1	**(0,4)**	236.3	14.4	25.9
**(5,0)**	195.0	17.3	27.9	**(0,5)**	226.8	14.6	25.0
**(6,0)**	191.4	17.6	28.0	**(0,6)**	241.6	13.5	25.8
**(7,0)**	192.9	16.9	28.0	**(0,7)**	235.0	14.3	25.8
**(8,0)**	200.4	17.5	28.8	**(0,8)**	240.8	14.6	26.8
**(9,0)**	192.3	18.0	28.7	**(0,9)**	230.1	14.9	25.9
**(10,0)**	196.1	17.6	28.9	**(0,10)**	240.7	14.0	26.7
**(20,0)**	200.1	17.4	29.0	**(0,20)**	243.0	14.4	27.1

**Figure 7:**
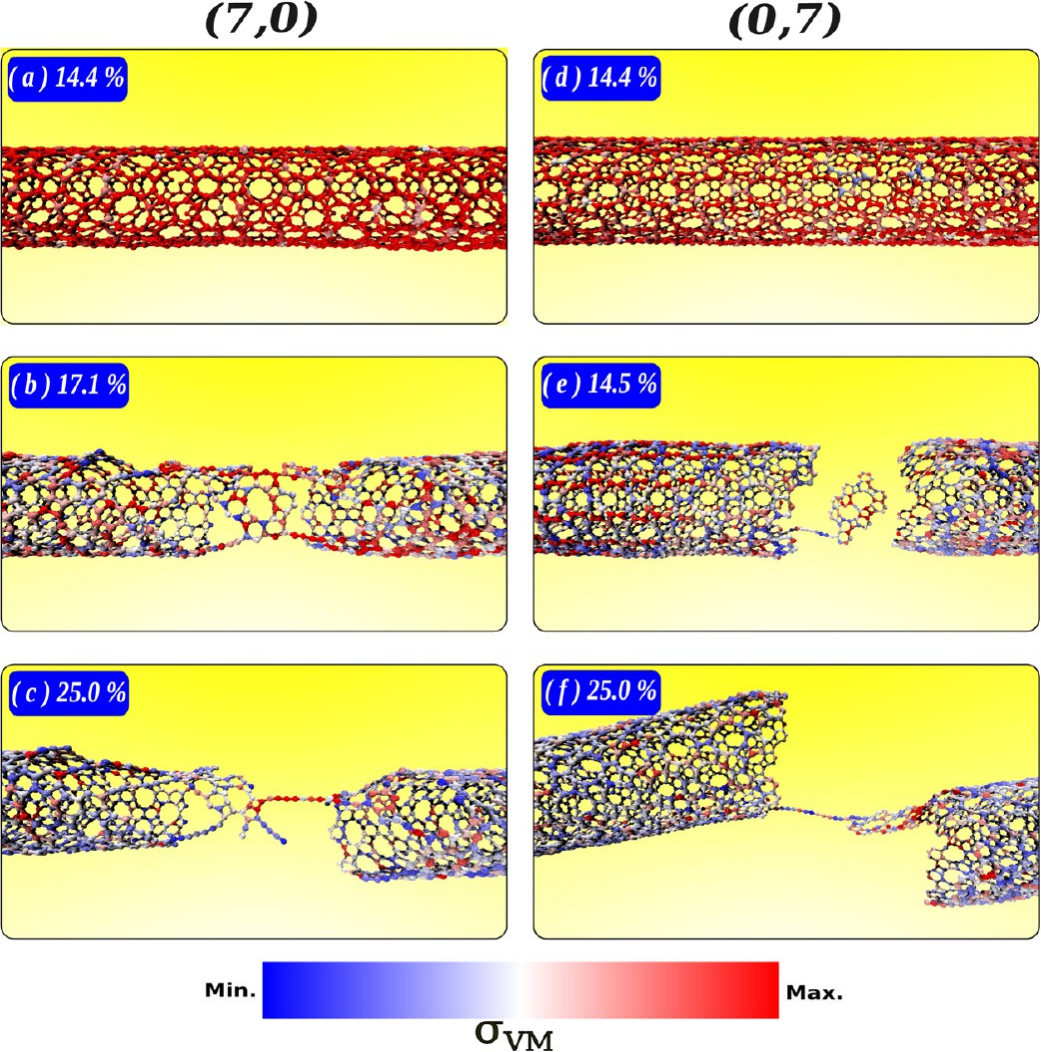
Snapshots of fracture patterns for representative cases: (7,0) armchair (a–c) and (0,7) zigzag (d–f) nanotubes.

To investigate the fracture patterns of DHQ nanotubes, we selected two representative cases. Figure 7 presents snapshots captured near and after the fracture of the armchair (panels 7a–c) and zigzag (panels 7d–f) DHQ nanotubes. For the armchair (7,0)-DHQ-NT, Figure 7a presents the snapshot at 17% longitudinal, showing the system fully stressed, as indicated by the von Mises stress scale. At 17.1% strain (panel 7b), the system is fractured, leaving a small region bonded by LACs. In Figure 7c, the nanotube was 25% strained, and a LAC remains, carrying the entire system’s stress before complete separation.

Similarly, for the zigzag (0,7)-DHQ-NT, the system is fully stressed at 14.4% strain (panel 7d), fractured at 15.5% strain (panel 7e), and at 25% strain (panel 7f), the LAC remains attached to one fragment of the DHQ.

This behavior highlights the increased stress accumulation caused by the finite topology of the nanotubes, contrasting with the infinite topology modeled in the monolayer. Nevertheless, we demonstrate that the fracture pattern and critical strain are unaffected by curvature. However, smaller-diameter nanotubes exhibit reduced rigidity, reflected in lower Young’s modulus values. In all cases, a slightly lower critical stress is also observed.

### 3.3 Thermal Analysis

It is widely recognized that the mechanical properties of nanostructures are temperature-dependent, with systems becoming more flexible at higher temperatures. Consequently, their Young’s modulus, critical strains, and stresses generally exhibit lower values as temperature increases.^81,82^ Based on this, we conducted CMD simulations to evaluate the temperature response of DHQ using the MLIP previously obtained and discussed in earlier sections.

CMD simulations were performed at various temperature regimes, ranging from 100 to 2400 K, each lasting for 200 ps, employing the parameters described in the methodology. We evaluated the energy response of these simulations to temperature increases, observing linear regimes within the same topology. Deviations from this linear trend indicate phase transitions or atomic rearrangements in the nanomaterial. The first observed phase transition was identified as the system’s critical temperature, specifically for DHQ in this context. It is also known from the literature that transitioning from a 2D topology to a quasi-1D topology does not strongly influence the temperature-induced phase change process.^43,83^

To quantify the specific heat (*c*), the first law of thermodynamics is applied as d*E* = δ*Q* – *p* d*V*, where *E*, *Q*, *p*, and *V* represent internal energy, heat, pressure, and volume, respectively. The internal energy as a function of temperature and volume is given by
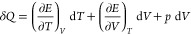
17The volume remains constant for the canonical ensemble used here, leading to the specific heat expression *C* = δ*Q*/d*T* = (∂*U*/∂*T*)_*V*_. Finally, the mass-specific heat capacity is *c* = *C*/*M*, where *M* is the mass of DHQ.

**Figure 8:**
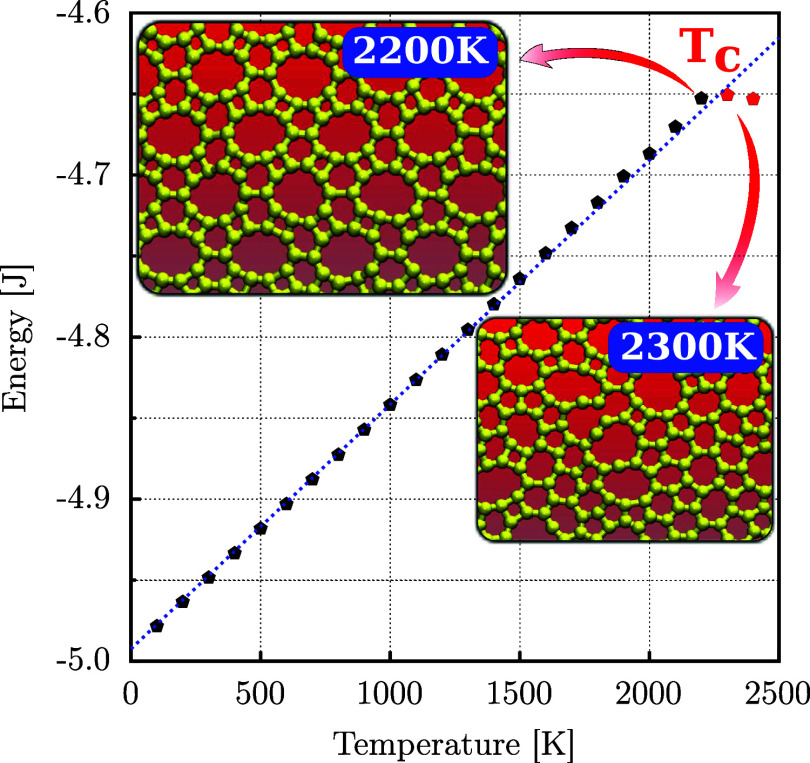
Energy as a function of temperature for DHQ monolayers obtained from CMD simulations using the MLIP force field. Insets show system snapshots at temperatures of 2200 and 2300 K. The dashed blue line represents the linear fit in the corresponding region.

Figure 8 shows the energy variation as a function of temperature for a DHQ monolayer containing 3840 carbon atoms. The primary insight from this data is the critical temperature *T*_C_ = 2200 K, determined with a temperature resolution of 100 K, representing the highest temperature at which DHQ retains its topology with 4-, 6-, and 10-membered carbon rings. The insets in the figure display snapshots after 200 ps of simulation for 2200 and 2300 K. At 2200 K, the DHQ topology is preserved. In contrast, at 2300 K, numerous reconstructions of covalent bonds result in an amorphous-like system.

At temperatures between 2200 and 2300 K, the system undergoes a phase transition characterized by the loss of its original periodic arrangement and the emergence of an amorphous structure. This high-temperature phase marks the formation of disordered ring networks with a wide range of ring sizes, including more extensive and irregular configurations. Occasional nonplanarity is also observed, consistent with the expected thermal destabilization of the structure under such extreme conditions. The MLIP demonstrated good agreement with DFT in capturing key features of this amorphous phase,^84,85^ including the onset of amorphization and thermal expansion trends. However, slight deviations in energy and force predictions were noted due to the training data set’s absence of explicit high-temperature configurations. These deviations could influence the fine details of the amorphization process and related thermodynamic properties. Future improvements could address this limitation by retraining the MLIP using an expanded data set incorporating high-temperature configurations derived from DFT calculations.

The critical temperature of DHQ reported here is considerably higher than in the literature for BPN, where the first phase transition occurs around 1100 K.^45,86^ The specific heat of DHQ, calculated from the linear energy-temperature response region, is *c* = 2.1 × 10^3^ J·kg^–1^·K^–1^, a value comparable to graphene.^87^ The connection between ring formation energetics and thermal stability advances the understanding of structural evolution under thermal conditions. Previous studies have shown that ML models can effectively predict structural transformations, including ring rearrangements, under thermal stress.^88,89^

## 4 Conclusions

This study investigates the mechanical and thermal properties of DHQ monolayers and nanotubes using a machine-learned interatomic potential developed from first-principles data. The MLIP accurately replicated critical physical characteristics, including phonon dispersion and structural parameters, demonstrating its robustness and applicability for large-scale molecular simulations.

Mechanical analyses revealed that Young’s modulus of DHQ nanotubes depends on their chirality and diameter, ranging from 127 to 243 N/m. The critical strain for fracture varied between 13.6 and 17.4%, with fracture patterns exhibiting directional anisotropy associated with the material’s topology. Thermal analyses identified a critical temperature of 2200 K, marking the onset of structural phase transitions. This critical temperature is significantly higher than reported for similar systems, such as BPN, and the specific heat is the same as graphene.

These results underscore the potential of DHQ-based nanotubes for applications in areas such as high-temperature nanodevices, thermal management systems, and lightweight mechanical reinforcements. The robustness of the developed machine-learned interatomic potential further supports its applicability in exploring other carbon-based nanostructures and their technological uses. Young’s modulus and fracture strain observed in DHQ nanotubes suggest their suitability as mechanical reinforcement materials in composite systems. Their directional anisotropy and robustness under strain make them particularly valuable for applications requiring tailored mechanical properties, such as aerospace and automotive industries, where lightweight yet strong materials are essential.

The findings reported here contribute to a comprehensive understanding of a planar nanoporous carbon-based material and its underlying nanotubes and open new research avenues by providing a machine learning-based interatomic potential that enables new perspectives for CMD simulations, expanding the applications and insights into potential applications with DHQ.

## Supporting Information Available

MLIP trained with AIMD simulation data and the files with the geometries of the monolayer and nanotubes in different chiralities and diameters (PDF)
