# Supplementary material for: Machine Learning Interatomic Potential for Modeling the Mechanical and Thermal Properties of Naphthyl-Based Nanotubes
Source: J Chem Theory Comput. 2025 Jan 28;21(5):2612–25. doi: 10.1021/acs.jctc.4c01578 (PMC11912207; doi:10.1021/acs.jctc.4c01578)
Supplement: Supplementary file 1 — ct4c01578_si_001.pdf [file ct4c01578_si_001.pdf]

Supplementary Information for Machine learning interatomic potential for modeling the mechanical and thermal properties of naphthyl-based nanotubes

Hugo X. Rodrigues,[†,‡] Hudson R. Armando,[¶,‡] Daniel A. da Silva,[§,∥] João Paulo J. da Costa,[§,∥] Luiz A. Ribeiro, Jr,[†,‡,¶] and Marcelo L. Pereira, Jr[*,⊥,¶]

†*University of Brasília, Institute of Physics, 70910900, Brasília, Federal District, Brazil.*

‡*Computational Materials Laboratory, University of Brasília, 70910900, Brasília, Federal District, Brazil.*

¶*University of Brasília, Institute of Physics, Physics Postgraduate Program, 70910900, Brasília, Federal District, Brazil.*

§*Hamm-Lippstadt University of Applied Sciences, Department Lippstadt 2, 59063, Hamm, Germany*

∥*University of Brasília, College of Technology, Department of Electrical Engineering, Professional Postgraduate Program in Electrical Engineering, 70910900, Brasília, Federal District, Brazil.*

⊥*University of Brasília, College of Technology, Department of Electrical Engineering, 70910900, Brasília, Federal District, Brazil.*

E-mail: marcelo.lopes@unb.br

```
MTP
version = 1.1.0
potential_name = MTP1m
scaling = 3.293853469160256e-02
species_count = 1
potential_tag =
radial_basis_type = RBChebyshev
	min_dist = 1.124490000000000e+00
	max_dist = 5.000000000000000e+00
	radial_basis_size = 8
	radial_funcs_count = 6
	radial_coeffs
		0-0
			{-7.556696308431224e-01, 3.171642201364693e-01,
-4.804441679692529e-01, -2.384058498040724e-01, 1.714167227265816e-01,
8.784286085015940e-02, 4.765903124100403e-02, 3.653592257875990e-02}
			{4.884593857879130e-02, 5.279875652189040e-01,
-1.359999908928957e-01, 5.523748492896770e-01, -5.372564444193891e-01,
2.504259782227227e-01, 1.905030741773152e-01, -8.706407926309173e-02}
			{2.129761868711816e-01, 2.736155150764200e-01,
-1.495485702334900e-01, 4.510457279710864e-01, 6.969398925394539e-01,
-3.003764884593257e-01, 2.145587422833360e-01, 1.788350268631979e-01}
			{-2.796359309679328e-01, 4.295593095734520e-01,
8.293779678720343e-01, -5.876881511193015e-02, 1.828767784194737e-01,
8.462604584748142e-02, 1.546734575137673e-02, -7.152786270967164e-02}
			{-3.930083792126278e-01, -5.900519643544681e-01,
1.355626893380648e-01, 4.672654697251094e-01, 1.212818668345674e-01,
3.392552479791967e-01, 3.605312269415714e-01, -2.970943264019493e-02}
			{-1.658592834015940e-01, -4.006112807079234e-02,
1.229045576957444e-02, 3.932608260583467e-01, 4.710975054677793e-02,
1.026766686060246e-01, -8.496145268392651e-01, 2.854365071660754e-01}
alpha_moments_count = 9396
alpha_index_basic_count = 706
alpha_index_basic = {{0, 0, 0, 0}, {0, 1, 0, 0}, {0, 0, 1, 0}, {0, 0, 0, 1}, {0, 2, 0, 0},
{0, 1, 1, 0}, {0, 1, 0, 1}, {0, 0, 2, 0}, {0, 0, 1, 1}, {0, 0, 0, 2}, {0, 3, 0, 0}, {0, 2,
1, 0}, {0, 2, 0, 1}, {0, 1, 2, 0}, {0, 1, 1, 1}, {0, 1, 0, 2}, {0, 0, 3, 0}, {0, 0, 2, 1},
{0, 0, 1, 2}, {0, 0, 0, 3}, {0, 4, 0, 0}, {0, 3, 1, 0}, {0, 3, 0, 1}, {0, 2, 2, 0}, {0, 2,
1, 1}, {0, 2, 0, 2}, {0, 1, 3, 0}, {0, 1, 2, 1}, {0, 1, 1, 2}, {0, 1, 0, 3}, {0, 0, 4, 0},
{0, 0, 3, 1}, {0, 0, 2, 2}, {0, 0, 1, 3}, {0, 0, 0, 4}, {0, 5, 0, 0}, {0, 4, 1, 0}, {0, 4,
0, 1}, {0, 3, 2, 0}, {0, 3, 1, 1}, {0, 3, 0, 2}, {0, 2, 3, 0}, {0, 2, 2, 1}, {0, 2, 1, 2},
{0, 2, 0, 3}, {0, 1, 4, 0}, {0, 1, 3, 1}, {0, 1, 2, 2}, {0, 1, 1, 3}, {0, 1, 0, 4}, {0, 0,
5, 0}, {0, 0, 4, 1}, {0, 0, 3, 2}, {0, 0, 2, 3}, {0, 0, 1, 4}, {0, 0, 0, 5}, {0, 6, 0, 0},
{0, 5, 1, 0}, {0, 5, 0, 1}, {0, 4, 2, 0}, {0, 4, 1, 1}, {0, 4, 0, 2}, {0, 3, 3, 0}, {0, 3,
2, 1}, {0, 3, 1, 2}, {0, 3, 0, 3}, {0, 2, 4, 0}, {0, 2, 3, 1}, {0, 2, 2, 2}, {0, 2, 1, 3},
{0, 2, 0, 4}, {0, 1, 5, 0}, {0, 1, 4, 1}, {0, 1, 3, 2}, {0, 1, 2, 3}, {0, 1, 1, 4}, {0, 1,
0, 5}, {0, 0, 6, 0}, {0, 0, 5, 1}, {0, 0, 4, 2}, {0, 0, 3, 3}, {0, 0, 2, 4}, {0, 0, 1, 5},
{0, 0, 0, 6}, {0, 7, 0, 0}, {0, 6, 1, 0}, {0, 6, 0, 1}, {0, 5, 2, 0}, {0, 5, 1, 1}, {0, 5,
0, 2}, {0, 4, 3, 0}, {0, 4, 2, 1}, {0, 4, 1, 2}, {0, 4, 0, 3}, {0, 3, 4, 0}, {0, 3, 3, 1},
{0, 3, 2, 2}, {0, 3, 1, 3}, {0, 3, 0, 4}, {0, 2, 5, 0}, {0, 2, 4, 1}, {0, 2, 3, 2}, {0, 2,
2, 3}, {0, 2, 1, 4}, {0, 2, 0, 5}, {0, 1, 6, 0}, {0, 1, 5, 1}, {0, 1, 4, 2}, {0, 1, 3, 3},
{0, 1, 2, 4}, {0, 1, 1, 5}, {0, 1, 0, 6}, {0, 0, 7, 0}, {0, 0, 6, 1}, {0, 0, 5, 2}, {0, 0,
4, 3}, {0, 0, 3, 4}, {0, 0, 2, 5}, {0, 0, 1, 6}, {0, 0, 0, 7}, {0, 8, 0, 0}, {0, 7, 1, 0},
{0, 7, 0, 1}, {0, 6, 2, 0}, {0, 6, 1, 1}, {0, 6, 0, 2}, {0, 5, 3, 0}, {0, 5, 2, 1}, {0, 5,
1, 2}, {0, 5, 0, 3}, {0, 4, 4, 0}, {0, 4, 3, 1}, {0, 4, 2, 2}, {0, 4, 1, 3}, {0, 4, 0, 4},
{0, 3, 5, 0}, {0, 3, 4, 1}, {0, 3, 3, 2}, {0, 3, 2, 3}, {0, 3, 1, 4}, {0, 3, 0, 5}, {0, 2,
6, 0}, {0, 2, 5, 1}, {0, 2, 4, 2}, {0, 2, 3, 3}, {0, 2, 2, 4}, {0, 2, 1, 5}, {0, 2, 0, 6},
{0, 1, 7, 0}, {0, 1, 6, 1}, {0, 1, 5, 2}, {0, 1, 4, 3}, {0, 1, 3, 4}, {0, 1, 2, 5}, {0, 1,
1, 6}, {0, 1, 0, 7}, {0, 0, 8, 0}, {0, 0, 7, 1}, {0, 0, 6, 2}, {0, 0, 5, 3}, {0, 0, 4, 4},
{0, 0, 3, 5}, {0, 0, 2, 6}, {0, 0, 1, 7}, {0, 0, 0, 8}, {0, 9, 0, 0}, {0, 8, 1, 0}, {0, 8,
0, 1}, {0, 7, 2, 0}, {0, 7, 1, 1}, {0, 7, 0, 2}, {0, 6, 3, 0}, {0, 6, 2, 1}, {0, 6, 1, 2},
{0, 6, 0, 3}, {0, 5, 4, 0}, {0, 5, 3, 1}, {0, 5, 2, 2}, {0, 5, 1, 3}, {0, 5, 0, 4}, {0, 4,
5, 0}, {0, 4, 4, 1}, {0, 4, 3, 2}, {0, 4, 2, 3}, {0, 4, 1, 4}, {0, 4, 0, 5}, {0, 3, 6, 0},
{0, 3, 5, 1}, {0, 3, 4, 2}, {0, 3, 3, 3}, {0, 3, 2, 4}, {0, 3, 1, 5}, {0, 3, 0, 6}, {0, 2,
7, 0}, {0, 2, 6, 1}, {0, 2, 5, 2}, {0, 2, 4, 3}, {0, 2, 3, 4}, {0, 2, 2, 5}, {0, 2, 1, 6},
{0, 2, 0, 7}, {0, 1, 8, 0}, {0, 1, 7, 1}, {0, 1, 6, 2}, {0, 1, 5, 3}, {0, 1, 4, 4}, {0, 1,
3, 5}, {0, 1, 2, 6}, {0, 1, 1, 7}, {0, 1, 0, 8}, {0, 0, 9, 0}, {0, 0, 8, 1}, {0, 0, 7, 2},
{0, 0, 6, 3}, {0, 0, 5, 4}, {0, 0, 4, 5}, {0, 0, 3, 6}, {0, 0, 2, 7}, {0, 0, 1, 8}, {0, 0,
0, 9}, {0, 10, 0, 0}, {0, 9, 1, 0}, {0, 9, 0, 1}, {0, 8, 2, 0}, {0, 8, 1, 1}, {0, 8, 0,
2}, {0, 7, 3, 0}, {0, 7, 2, 1}, {0, 7, 1, 2}, {0, 7, 0, 3}, {0, 6, 4, 0}, {0, 6, 3, 1},
```

{0, 6, 2, 2}, {0, 6, 1, 3}, {0, 6, 0, 4}, {0, 5, 5, 0}, {0, 5, 4, 1}, {0, 5, 3, 2}, {0, 5, 2, 3}, {0, 5, 1, 4}, {0, 5, 0, 5}, {0, 4, 6, 0}, {0, 4, 5, 1}, {0, 4, 4, 2}, {0, 4, 3, 3}, {0, 4, 2, 4}, {0, 4, 1, 5}, {0, 4, 0, 6}, {0, 3, 7, 0}, {0, 3, 6, 1}, {0, 3, 5, 2}, {0, 3, 4, 3}, {0, 3, 3, 4}, {0, 3, 2, 5}, {0, 3, 1, 6}, {0, 3, 0, 7}, {0, 2, 8, 0}, {0, 2, 7, 1}, {0, 2, 6, 2}, {0, 2, 5, 3}, {0, 2, 4, 4}, {0, 2, 3, 5}, {0, 2, 2, 6}, {0, 2, 1, 7}, {0, 2, 0, 8}, {0, 1, 9, 0}, {0, 1, 8, 1}, {0, 1, 7, 2}, {0, 1, 6, 3}, {0, 1, 5, 4}, {0, 1, 4, 5}, {0, 1, 3, 6}, {0, 1, 2, 7}, {0, 1, 1, 8}, {0, 1, 0, 9}, {0, 0, 10, 0}, {0, 0, 9, 1}, {0, 0, 8, 2}, {0, 0, 7, 3}, {0, 0, 6, 4}, {0, 0, 5, 5}, {0, 0, 4, 6}, {0, 0, 3, 7}, {0, 0, 2, 8}, {0, 0, 1, 9}, {0, 0, 0, 10}, {1, 0, 0, 0}, {1, 1, 0, 0}, {1, 0, 1, 0}, {1, 0, 0, 1}, {1, 2, 0, 0}, {1, 1, 1, 0}, {1, 1, 0, 1}, {1, 0, 2, 0}, {1, 0, 1, 1}, {1, 0, 0, 2}, {1, 3, 0, 0}, {1, 2, 1, 0}, {1, 2, 0, 1}, {1, 1, 2, 0}, {1, 1, 1, 1}, {1, 1, 0, 2}, {1, 0, 3, 0}, {1, 0, 2, 1}, {1, 0, 1, 2}, {1, 0, 0, 3}, {1, 4, 0, 0}, {1, 3, 1, 0}, {1, 3, 0, 1}, {1, 2, 2, 0}, {1, 2, 1, 1}, {1, 2, 0, 2}, {1, 1, 3, 0}, {1, 1, 2, 1}, {1, 1, 1, 2}, {1, 1, 0, 3}, {1, 0, 4, 0}, {1, 0, 3, 1}, {1, 0, 2, 2}, {1, 0, 1, 3}, {1, 0, 0, 4}, {1, 5, 0, 0}, {1, 4, 1, 0}, {1, 4, 0, 1}, {1, 3, 2, 0}, {1, 3, 1, 1}, {1, 3, 0, 2}, {1, 2, 3, 0}, {1, 2, 2, 1}, {1, 2, 1, 2}, {1, 2, 0, 3}, {1, 1, 4, 0}, {1, 1, 3, 1}, {1, 1, 2, 2}, {1, 1, 1, 3}, {1, 1, 0, 4}, {1, 0, 5, 0}, {1, 0, 4, 1}, {1, 0, 3, 2}, {1, 0, 2, 3}, {1, 0, 1, 4}, {1, 0, 0, 5}, {1, 6, 0, 0}, {1, 5, 1, 0}, {1, 5, 0, 1}, {1, 4, 2, 0}, {1, 4, 1, 1}, {1, 4, 0, 2}, {1, 3, 3, 0}, {1, 3, 2, 1}, {1, 3, 1, 2}, {1, 3, 0, 3}, {1, 2, 4, 0}, {1, 2, 3, 1}, {1, 2, 2, 2}, {1, 2, 1, 3}, {1, 2, 0, 4}, {1, 1, 5, 0}, {1, 1, 4, 1}, {1, 1, 3, 2}, {1, 1, 2, 3}, {1, 1, 1, 4}, {1, 1, 0, 5}, {1, 0, 6, 0}, {1, 0, 5, 1}, {1, 0, 4, 2}, {1, 0, 3, 3}, {1, 0, 2, 4}, {1, 0, 1, 5}, {1, 0, 0, 6}, {1, 7, 0, 0}, {1, 6, 1, 0}, {1, 6, 0, 1}, {1, 5, 2, 0}, {1, 5, 1, 1}, {1, 5, 0, 2}, {1, 4, 3, 0}, {1, 4, 2, 1}, {1, 4, 1, 2}, {1, 4, 0, 3}, {1, 3, 4, 0}, {1, 3, 3, 1}, {1, 3, 2, 2}, {1, 3, 1, 3}, {1, 3, 0, 4}, {1, 2, 5, 0}, {1, 2, 4, 1}, {1, 2, 3, 2}, {1, 2, 2, 3}, {1, 2, 1, 4}, {1, 2, 0, 5}, {1, 1, 6, 0}, {1, 1, 5, 1}, {1, 1, 4, 2}, {1, 1, 3, 3}, {1, 1, 2, 4}, {1, 1, 1, 5}, {1, 1, 0, 6}, {1, 0, 7, 0}, {1, 0, 6, 1}, {1, 0, 5, 2}, {1, 0, 4, 3}, {1, 0, 3, 4}, {1, 0, 2, 5}, {1, 0, 1, 6}, {1, 0, 0, 7}, {1, 8, 0, 0}, {1, 7, 1, 0}, {1, 7, 0, 1}, {1, 6, 2, 0}, {1, 6, 1, 1}, {1, 6, 0, 2}, {1, 5, 3, 0}, {1, 5, 2, 1}, {1, 5, 1, 2}, {1, 5, 0, 3}, {1, 4, 4, 0}, {1, 4, 3, 1}, {1, 4, 2, 2}, {1, 4, 1, 3}, {1, 4, 0, 4}, {1, 3, 5, 0}, {1, 3, 4, 1}, {1, 3, 3, 2}, {1, 3, 2, 3}, {1, 3, 1, 4}, {1, 3, 0, 5}, {1, 2, 6, 0}, {1, 2, 5, 1}, {1, 2, 4, 2}, {1, 2, 3, 3}, {1, 2, 2, 4}, {1, 2, 1, 5}, {1, 2, 0, 6}, {1, 1, 7, 0}, {1, 1, 6, 1}, {1, 1, 5, 2}, {1, 1, 4, 3}, {1, 1, 3, 4}, {1, 1, 2, 5}, {1, 1, 1, 6}, {1, 1, 0, 7}, {1, 0, 8, 0}, {1, 0, 7, 1}, {1, 0, 6, 2}, {1, 0, 5, 3}, {1, 0, 4, 4}, {1, 0, 3, 5}, {1, 0, 2, 6}, {1, 0, 1, 7}, {1, 0, 0, 8}, {1, 9, 0, 0}, {1, 8, 1, 0}, {1, 8, 0, 1}, {1, 7, 2, 0}, {1, 7, 1, 1}, {1, 7, 0, 2}, {1, 6, 3, 0}, {1, 6, 2, 1}, {1, 6, 1, 2}, {1, 6, 0, 3}, {1, 5, 4, 0}, {1, 5, 3, 1}, {1, 5, 2, 2}, {1, 5, 1, 3}, {1, 5, 0, 4}, {1, 4, 5, 0}, {1, 4, 4, 1}, {1, 4, 3, 2}, {1, 4, 2, 3}, {1, 4, 1, 4}, {1, 4, 0, 5}, {1, 3, 6, 0}, {1, 3, 5, 1}, {1, 3, 4, 2}, {1, 3, 3, 3}, {1, 3, 2, 4}, {1, 3, 1, 5}, {1, 3, 0, 6}, {1, 2, 7, 0}, {1, 2, 6, 1}, {1, 2, 5, 2}, {1, 2, 4, 3}, {1, 2, 3, 4}, {1, 2, 2, 5}, {1, 2, 1, 6}, {1, 2, 0, 7}, {1, 1, 8, 0}, {1, 1, 7, 1}, {1, 1, 6, 2}, {1, 1, 5, 3}, {1, 1, 4, 4}, {1, 1, 3, 5}, {1, 1, 2, 6}, {1, 1, 1, 7}, {1, 1, 0, 8}, {1, 0, 9, 0}, {1, 0, 8, 1}, {1, 0, 7, 2}, {1, 0, 6, 3}, {1, 0, 5, 4}, {1, 0, 4, 5}, {1, 0, 3, 6}, {1, 0, 2, 7}, {1, 0, 1, 8}, {1, 0, 0, 9}, {2, 0, 0, 0}, {2, 1, 0, 0}, {2, 0, 1, 0}, {2, 0, 0, 1}, {2, 2, 0, 0}, {2, 1, 1, 0}, {2, 1, 0, 1}, {2, 0, 2, 0}, {2, 0, 1, 1}, {2, 0, 0, 2}, {2, 3, 0, 0}, {2, 2, 1, 0}, {2, 2, 0, 1}, {2, 1, 2, 0}, {2, 1, 1, 1}, {2, 1, 0, 2}, {2, 0, 3, 0}, {2, 0, 2, 1}, {2, 0, 1, 2}, {2, 0, 0, 3}, {2, 4, 0, 0}, {2, 3, 1, 0}, {2, 3, 0, 1}, {2, 2, 2, 0}, {2, 2, 1, 1}, {2, 2, 0, 2}, {2, 1, 3, 0}, {2, 1, 2, 1}, {2, 1, 1, 2}, {2, 1, 0, 3}, {2, 0, 4, 0}, {2, 0, 3, 1}, {2, 0, 2, 2}, {2, 0, 1, 3}, {2, 0, 0, 4}, {2, 5, 0, 0}, {2, 4, 1, 0}, {2, 4, 0, 1}, {2, 3, 2, 0}, {2, 3, 1, 1}, {2, 3, 0, 2}, {2, 2, 3, 0}, {2, 2, 2, 1}, {2, 2, 1, 2}, {2, 2, 0, 3}, {2, 1, 4, 0}, {2, 1, 3, 1}, {2, 1, 2, 2}, {2, 1, 1, 3}, {2, 1, 0, 4}, {2, 0, 5, 0}, {2, 0, 4, 1}, {2, 0, 3, 2}, {2, 0, 2, 3}, {2, 0, 1, 4}, {2, 0, 0, 5}, {2, 6, 0, 0}, {2, 5, 1, 0}, {2, 5, 0, 1}, {2, 4, 2, 0}, {2, 4, 1, 1}, {2, 4, 0, 2}, {2, 3, 3, 0}, {2, 3, 2, 1}, {2, 3, 1, 2}, {2, 3, 0, 3}, {2, 2, 4, 0}, {2, 2, 3, 1}, {2, 2, 2, 2}, {2, 2, 1, 3}, {2, 2, 0, 4}, {2, 1, 5, 0}, {2, 1, 4, 1}, {2, 1, 3, 2}, {2, 1, 2, 3}, {2, 1, 1, 4}, {2, 1, 0, 5}, {2, 0, 6, 0}, {2, 0, 5, 1}, {2, 0, 4, 2}, {2, 0, 3, 3}, {2, 0, 2, 4}, {2, 0, 1, 5}, {2, 0, 0, 6}, {2, 7, 0, 0}, {2, 6, 1, 0}, {2, 6, 0, 1}, {2, 5, 2, 0}, {2, 5, 1, 1}, {2, 5, 0, 2}, {2, 4, 3, 0}, {2, 4, 2, 1}, {2, 4, 1, 2}, {2, 4, 0, 3}, {2, 3, 4, 0}, {2, 3, 3, 1}, {2, 3, 2, 2}, {2, 3, 1, 3}, {2, 3, 0, 4}, {2, 2, 5, 0}, {2, 2, 4, 1}, {2, 2, 3, 2}, {2, 2, 2, 3}, {2, 2, 1, 4}, {2, 2, 0, 5}, {2, 1, 6, 0}, {2, 1, 5, 1}, {2, 1, 4, 2}, {2, 1, 3, 3}, {2, 1, 2, 4}, {2, 1, 1, 5}, {2, 1, 0, 6}, {2, 0, 7, 0}, {2, 0, 6, 1}, {2, 0, 5, 2}, {2, 0, 4, 3}, {2, 0, 3, 4}, {2, 0, 2, 5}, {2, 0, 1, 6}, {2, 0, 0, 7}, {3, 0, 0, 0}, {3, 1, 0, 0}, {3, 0, 1, 0}, {3, 0, 0, 1}, {3, 2, 0, 0}, {3, 1, 1, 0}, {3, 1, 0, 1}, {3, 0, 2, 0}, {3, 0, 1, 1}, {3, 0, 0, 2}, {3, 3, 0, 0}, {3, 2, 1, 0}, {3, 2, 0, 1}, {3, 1, 2, 0}, {3, 1, 1, 1}, {3, 1, 0, 2}, {3, 0, 3, 0}, {3, 0, 2, 1}, {3, 0, 1, 2}, {3, 0, 0, 3}, {3, 4, 0, 0}, {3, 3, 1, 0}, {3, 3, 0, 1}, {3, 2, 2, 0}, {3, 2, 1, 1}, {3, 2, 0, 2}, {3, 1, 3, 0}, {3, 1, 2, 1}, {3, 1, 1, 2}, {3, 1, 0, 3}, {3, 0, 4, 0}, {3, 0, 3, 1}, {3, 0, 2, 2}, {3, 0, 1, 3}, {3, 0, 0, 4}, {3, 5, 0, 0}, {3, 4, 1, 0}, {3, 4, 0, 1}, {3, 3, 2, 0}, {3, 3, 1, 1}, {3, 3, 0, 2}, {3, 2, 3, 0}, {3, 2, 2, 1}, {3, 2, 1, 2}, {3, 2, 0, 3}, {3, 1, 4, 0}, {3, 1, 3, 1}, {3, 1, 2, 2}, {3, 1, 1, 3}, {3, 1, 0, 4}, {3, 0, 5, 0}, {3, 0, 4, 1}, {3, 0, 3, 2}, {3, 0, 2, 3},

{3, 0, 1, 4}, {3, 0, 0, 5}, {4, 0, 0, 0}, {4, 1, 0, 0}, {4, 0, 1, 0}, {4, 0, 0, 1}, {4, 2, 0, 0}, {4, 1, 1, 0}, {4, 1, 0, 1}, {4, 0, 2, 0}, {4, 0, 1, 1}, {4, 0, 0, 2}, {4, 3, 0, 0}, {4, 2, 1, 0}, {4, 2, 0, 1}, {4, 1, 2, 0}, {4, 1, 1, 1}, {4, 1, 0, 2}, {4, 0, 3, 0}, {4, 0, 2, 1}, {4, 0, 1, 2}, {4, 0, 0, 3}, {5, 0, 0, 0}, {5, 1, 0, 0}, {5, 0, 1, 0}, {5, 0, 0, 1}}
alpha_index_times_count = 82520
alpha_index_times = {{0, 0, 1, 706}, {1, 1, 1, 707}, {2, 2, 1, 707}, {3, 3, 1, 707}, {1, 4, 1, 708}, {2, 5, 1, 708}, {3, 6, 1, 708}, {1, 5, 1, 709}, {2, 7, 1, 709}, {3, 8, 1, 709}, {1, 6, 1, 710}, {2, 8, 1, 710}, {3, 9, 1, 710}, {1, 10, 1, 711}, {2, 11, 1, 711}, {3, 12, 1, 711}, {1, 11, 1, 712}, {2, 13, 1, 712}, {3, 14, 1, 712}, {1, 12, 1, 713}, {2, 14, 1, 713}, {3, 15, 1, 713}, {1, 13, 1, 714}, {2, 16, 1, 714}, {3, 17, 1, 714}, {1, 14, 1, 715}, {2, 17, 1, 715}, {3, 18, 1, 715}, {1, 15, 1, 716}, {2, 18, 1, 716}, {3, 19, 1, 716}, {4, 4, 1, 717}, {5, 5, 1, 717}, {6, 6, 1, 717}, {4, 5, 1, 718}, {5, 7, 1, 718}, {6, 8, 1, 718}, {4, 6, 1, 719}, {5, 8, 1, 719}, {6, 9, 1, 719}, {5, 5, 1, 720}, {7, 7, 1, 720}, {8, 8, 1, 720}, {5, 6, 1, 721}, {7, 8, 1, 721}, {8, 9, 1, 721}, {6, 6, 1, 722}, {8, 8, 1, 722}, {9, 9, 1, 722}, {4, 10, 1, 723}, {5, 11, 1, 723}, {6, 12, 1, 723}, {4, 11, 1, 724}, {5, 13, 1, 724}, {6, 14, 1, 724}, {4, 12, 1, 725}, {5, 14, 1, 725}, {6, 15, 1, 725}, {4, 13, 1, 726}, {5, 16, 1, 726}, {6, 17, 1, 726}, {4, 14, 1, 727}, {5, 17, 1, 727}, {6, 18, 1, 727}, {4, 15, 1, 728}, {5, 18, 1, 728}, {6, 19, 1, 728}, {5, 10, 1, 729}, {7, 11, 1, 729}, {8, 12, 1, 729}, {5, 11, 1, 730}, {7, 13, 1, 730}, {8, 14, 1, 730}, {5, 12, 1, 731}, {7, 14, 1, 731}, {8, 15, 1, 731}, {5, 13, 1, 732}, {7, 16, 1, 732}, {8, 17, 1, 732}, {5, 14, 1, 733}, {7, 17, 1, 733}, {8, 18, 1, 733}, {5, 15, 1, 734}, {7, 18, 1, 734}, {8, 19, 1, 734}, {6, 10, 1, 735}, {8, 11, 1, 735}, {9, 12, 1, 735}, {6, 11, 1, 736}, {8, 13, 1, 736}, {9, 14, 1, 736}, {6, 12, 1, 737}, {8, 14, 1, 737}, {9, 15, 1, 737}, {6, 13, 1, 738}, {8, 16, 1, 738}, {9, 17, 1, 738}, {6, 14, 1, 739}, {8, 17, 1, 739}, {9, 18, 1, 739}, {6, 15, 1, 740}, {8, 18, 1, 740}, {9, 19, 1, 740}, {4, 20, 1, 741}, {5, 21, 1, 741}, {6, 22, 1, 741}, {4, 21, 1, 742}, {5, 23, 1, 742}, {6, 24, 1, 742}, {4, 22, 1, 743}, {5, 24, 1, 743}, {6, 25, 1, 743}, {4, 23, 1, 744}, {5, 26, 1, 744}, {6, 27, 1, 744}, {4, 24, 1, 745}, {5, 27, 1, 745}, {6, 28, 1, 745}, {4, 25, 1, 746}, {5, 28, 1, 746}, {6, 29, 1, 746}, {4, 26, 1, 747}, {5, 30, 1, 747}, {6, 31, 1, 747}, {4, 27, 1, 748}, {5, 31, 1, 748}, {6, 32, 1, 748}, {4, 28, 1, 749}, {5, 32, 1, 749}, {6, 33, 1, 749}, {4, 29, 1, 750}, {5, 33, 1, 750}, {6, 34, 1, 750}, {5, 20, 1, 751}, {7, 21, 1, 751}, {8, 22, 1, 751}, {5, 21, 1, 752}, {7, 23, 1, 752}, {8, 24, 1, 752}, {5, 22, 1, 753}, {7, 24, 1, 753}, {8, 25, 1, 753}, {5, 23, 1, 754}, {7, 26, 1, 754}, {8, 27, 1, 754}, {5, 24, 1, 755}, {7, 27, 1, 755}, {8, 28, 1, 755}, {5, 25, 1, 756}, {7, 28, 1, 756}, {8, 29, 1, 756}, {5, 26, 1, 757}, {7, 30, 1, 757}, {8, 31, 1, 757}, {5, 27, 1, 758}, {7, 31, 1, 758}, {8, 32, 1, 758}, {5, 28, 1, 759}, {7, 32, 1, 759}, {8, 33, 1, 759}, {5, 29, 1, 760}, {7, 33, 1, 760}, {8, 34, 1, 760}, {6, 20, 1, 761}, {8, 21, 1, 761}, {9, 22, 1, 761}, {6, 21, 1, 762}, {8, 23, 1, 762}, {9, 24, 1, 762}, {6, 22, 1, 763}, {8, 24, 1, 763}, {9, 25, 1, 763}, {6, 23, 1, 764}, {8, 26, 1, 764}, {9, 27, 1, 764}, {6, 24, 1, 765}, {8, 27, 1, 765}, {9, 28, 1, 765}, {6, 25, 1, 766}, {8, 28, 1, 766}, {9, 29, 1, 766}, {6, 26, 1, 767}, {8, 30, 1, 767}, {9, 31, 1, 767}, {6, 27, 1, 768}, {8, 31, 1, 768}, {9, 32, 1, 768}, {6, 28, 1, 769}, {8, 32, 1, 769}, {9, 33, 1, 769}, {6, 29, 1, 770}, {8, 33, 1, 770}, {9, 34, 1, 770}, {4, 4, 1, 771}, {5, 5, 2, 771}, {6, 6, 2, 771}, {7, 7, 1, 771}, {8, 8, 2, 771}, {9, 9, 1, 771}, {4, 10, 1, 772}, {5, 11, 2, 772}, {6, 12, 2, 772}, {7, 13, 1, 772}, {8, 14, 2, 772}, {9, 15, 1, 772}, {4, 11, 1, 773}, {5, 13, 2, 773}, {6, 14, 2, 773}, {7, 16, 1, 773}, {8, 17, 2, 773}, {9, 18, 1, 773}, {4, 12, 1, 774}, {5, 14, 2, 774}, {6, 15, 2, 774}, {7, 17, 1, 774}, {8, 18, 2, 774}, {9, 19, 1, 774}, {4, 20, 1, 775}, {5, 21, 2, 775}, {6, 22, 2, 775}, {7, 23, 1, 775}, {8, 24, 2, 775}, {9, 25, 1, 775}, {4, 21, 1, 776}, {5, 23, 2, 776}, {6, 24, 2, 776}, {7, 26, 1, 776}, {8, 27, 2, 776}, {9, 28, 1, 776}, {4, 22, 1, 777}, {5, 24, 2, 777}, {6, 25, 2, 777}, {7, 27, 1, 777}, {8, 28, 2, 777}, {9, 29, 1, 777}, {4, 23, 1, 778}, {5, 26, 2, 778}, {6, 27, 2, 778}, {7, 30, 1, 778}, {8, 31, 2, 778}, {9, 32, 1, 778}, {4, 24, 1, 779}, {5, 27, 2, 779}, {6, 28, 2, 779}, {7, 31, 1, 779}, {8, 32, 2, 779}, {9, 33, 1, 779}, {4, 25, 1, 780}, {5, 28, 2, 780}, {6, 29, 2, 780}, {7, 32, 1, 780}, {8, 33, 2, 780}, {9, 34, 1, 780}, {4, 35, 1, 781}, {5, 36, 2, 781}, {6, 37, 2, 781}, {7, 38, 1, 781}, {8, 39, 2, 781}, {9, 40, 1, 781}, {4, 36, 1, 782}, {5, 38, 2, 782}, {6, 39, 2, 782}, {7, 41, 1, 782}, {8, 42, 2, 782}, {9, 43, 1, 782}, {4, 37, 1, 783}, {5, 39, 2, 783}, {6, 40, 2, 783}, {7, 42, 1, 783}, {8, 43, 2, 783}, {9, 44, 1, 783}, {4, 38, 1, 784}, {5, 41, 2, 784}, {6, 42, 2, 784}, {7, 45, 1, 784}, {8, 46, 2, 784}, {9, 47, 1, 784}, {4, 39, 1, 785}, {5, 42, 2, 785}, {6, 43, 2, 785}, {7, 46, 1, 785}, {8, 47, 2, 785}, {9, 48, 1, 785}, {4, 40, 1, 786}, {5, 43, 2, 786}, {6, 44, 2, 786}, {7, 47, 1, 786}, {8, 48, 2, 786}, {9, 49, 1, 786}, {4, 41, 1, 787}, {5, 45, 2, 787}, {6, 46, 2, 787}, {7, 50, 1, 787}, {8, 51, 2, 787}, {9, 52, 1, 787}, {4, 42, 1, 788}, {5, 46, 2, 788}, {6, 47, 2, 788}, {7, 51, 1, 788}, {8, 52, 2, 788}, {9, 53, 1, 788}, {4, 43, 1, 789}, {5, 47, 2, 789}, {6, 48, 2, 789}, {7, 52, 1, 789}, {8, 53, 2, 789}, {9, 54, 1, 789}, {4, 44, 1, 790}, {5, 48, 2, 790}, {6, 49, 2, 790}, {7, 53, 1, 790}, {8, 54, 2, 790}, {9, 55, 1, 790}, {4, 56, 1, 791}, {5, 57, 2, 791}, {6, 58, 2, 791}, {7, 59, 1, 791}, {8, 60, 2, 791}, {9, 61, 1, 791}, {4, 57, 1, 792}, {5, 59, 2, 792}, {6, 60, 2, 792}, {7, 62, 1, 792}, {8, 63, 2, 792}, {9, 64, 1, 792}, {4, 58, 1, 793}, {5, 60, 2, 793}, {6, 61, 2, 793}, {7, 63, 1, 793}, {8, 64, 2, 793}, {9, 65, 1, 793}, {4, 59, 1, 794}, {5, 62, 2, 794}, {6, 63, 2, 794}, {7, 66, 1, 794}, {8, 67, 2, 794}, {9, 68, 1,

794}, {4, 60, 1, 795}, {5, 63, 2, 795}, {6, 64, 2, 795}, {7, 67, 1, 795}, {8, 68, 2, 795}, {9, 69, 1, 795}, {4, 61, 1, 796}, {5, 64, 2, 796}, {6, 65, 2, 796}, {7, 68, 1, 796}, {8, 69, 2, 796}, {9, 70, 1, 796}, {4, 62, 1, 797}, {5, 66, 2, 797}, {6, 67, 2, 797}, {7, 71, 1, 797}, {8, 72, 2, 797}, {9, 73, 1, 797}, {4, 63, 1, 798}, {5, 67, 2, 798}, {6, 68, 2, 798}, {7, 72, 1, 798}, {8, 73, 2, 798}, {9, 74, 1, 798}, {4, 64, 1, 799}, {5, 68, 2, 799}, {6, 69, 2, 799}, {7, 73, 1, 799}, {8, 74, 2, 799}, {9, 75, 1, 799}, {4, 65, 1, 800}, {5, 69, 2, 800}, {6, 70, 2, 800}, {7, 74, 1, 800}, {8, 75, 2, 800}, {9, 76, 1, 800}, {4, 66, 1, 801}, {5, 71, 2, 801}, {6, 72, 2, 801}, {7, 77, 1, 801}, {8, 78, 2, 801}, {9, 79, 1, 801}, {4, 67, 1, 802}, {5, 72, 2, 802}, {6, 73, 2, 802}, {7, 78, 1, 802}, {8, 79, 2, 802}, {9, 80, 1, 802}, {4, 68, 1, 803}, {5, 73, 2, 803}, {6, 74, 2, 803}, {7, 79, 1, 803}, {8, 80, 2, 803}, {9, 81, 1, 803}, {4, 69, 1, 804}, {5, 74, 2, 804}, {6, 75, 2, 804}, {7, 80, 1, 804}, {8, 81, 2, 804}, {9, 82, 1, 804}, {4, 70, 1, 805}, {5, 75, 2, 805}, {6, 76, 2, 805}, {7, 81, 1, 805}, {8, 82, 2, 805}, {9, 83, 1, 805}, {10, 10, 1, 806}, {11, 11, 1, 806}, {12, 12, 1, 806}, {10, 11, 1, 807}, {11, 13, 1, 807}, {12, 14, 1, 807}, {10, 12, 1, 808}, {11, 14, 1, 808}, {12, 15, 1, 808}, {10, 13, 1, 809}, {11, 16, 1, 809}, {12, 17, 1, 809}, {10, 14, 1, 810}, {11, 17, 1, 810}, {12, 18, 1, 810}, {10, 15, 1, 811}, {11, 18, 1, 811}, {12, 19, 1, 811}, {11, 11, 1, 812}, {13, 13, 1, 812}, {14, 14, 1, 812}, {11, 12, 1, 813}, {13, 14, 1, 813}, {14, 15, 1, 813}, {11, 13, 1, 814}, {13, 16, 1, 814}, {14, 17, 1, 814}, {11, 14, 1, 815}, {13, 17, 1, 815}, {14, 18, 1, 815}, {11, 15, 1, 816}, {13, 18, 1, 816}, {14, 19, 1, 816}, {12, 12, 1, 817}, {14, 14, 1, 817}, {15, 15, 1, 817}, {12, 13, 1, 818}, {14, 16, 1, 818}, {15, 17, 1, 818}, {12, 14, 1, 819}, {14, 17, 1, 819}, {15, 18, 1, 819}, {12, 15, 1, 820}, {14, 18, 1, 820}, {15, 19, 1, 820}, {13, 13, 1, 821}, {16, 16, 1, 821}, {17, 17, 1, 821}, {13, 14, 1, 822}, {16, 17, 1, 822}, {17, 18, 1, 822}, {13, 15, 1, 823}, {16, 18, 1, 823}, {17, 19, 1, 823}, {14, 14, 1, 824}, {17, 17, 1, 824}, {18, 18, 1, 824}, {14, 15, 1, 825}, {17, 18, 1, 825}, {18, 19, 1, 825}, {15, 15, 1, 826}, {18, 18, 1, 826}, {19, 19, 1, 826}, {10, 10, 1, 827}, {11, 11, 2, 827}, {12, 12, 2, 827}, {13, 13, 1, 827}, {14, 14, 2, 827}, {15, 15, 1, 827}, {10, 11, 1, 828}, {11, 13, 2, 828}, {12, 14, 2, 828}, {13, 16, 1, 828}, {14, 17, 2, 828}, {15, 18, 1, 828}, {10, 12, 1, 829}, {11, 14, 2, 829}, {12, 15, 2, 829}, {13, 17, 1, 829}, {14, 18, 2, 829}, {15, 19, 1, 829}, {11, 11, 1, 830}, {13, 13, 2, 830}, {14, 14, 2, 830}, {16, 16, 1, 830}, {17, 17, 2, 830}, {18, 18, 1, 830}, {11, 12, 1, 831}, {13, 14, 2, 831}, {14, 15, 2, 831}, {16, 17, 1, 831}, {17, 18, 2, 831}, {18, 19, 1, 831}, {12, 12, 1, 832}, {14, 14, 2, 832}, {15, 15, 2, 832}, {17, 17, 1, 832}, {18, 18, 2, 832}, {19, 19, 1, 832}, {10, 20, 1, 833}, {11, 21, 2, 833}, {12, 22, 2, 833}, {13, 23, 1, 833}, {14, 24, 2, 833}, {15, 25, 1, 833}, {10, 21, 1, 834}, {11, 23, 2, 834}, {12, 24, 2, 834}, {13, 26, 1, 834}, {14, 27, 2, 834}, {15, 28, 1, 834}, {10, 22, 1, 835}, {11, 24, 2, 835}, {12, 25, 2, 835}, {13, 27, 1, 835}, {14, 28, 2, 835}, {15, 29, 1, 835}, {10, 23, 1, 836}, {11, 26, 2, 836}, {12, 27, 2, 836}, {13, 30, 1, 836}, {14, 31, 2, 836}, {15, 32, 1, 836}, {10, 24, 1, 837}, {11, 27, 2, 837}, {12, 28, 2, 837}, {13, 31, 1, 837}, {14, 32, 2, 837}, {15, 33, 1, 837}, {10, 25, 1, 838}, {11, 28, 2, 838}, {12, 29, 2, 838}, {13, 32, 1, 838}, {14, 33, 2, 838}, {15, 34, 1, 838}, {11, 20, 1, 839}, {13, 21, 2, 839}, {14, 22, 2, 839}, {16, 23, 1, 839}, {17, 24, 2, 839}, {18, 25, 1, 839}, {11, 21, 1, 840}, {13, 23, 2, 840}, {14, 24, 2, 840}, {16, 26, 1, 840}, {17, 27, 2, 840}, {18, 28, 1, 840}, {11, 22, 1, 841}, {13, 24, 2, 841}, {14, 25, 2, 841}, {16, 27, 1, 841}, {17, 28, 2, 841}, {18, 29, 1, 841}, {11, 23, 1, 842}, {13, 26, 2, 842}, {14, 27, 2, 842}, {16, 30, 1, 842}, {17, 31, 2, 842}, {18, 32, 1, 842}, {11, 24, 1, 843}, {13, 27, 2, 843}, {14, 28, 2, 843}, {16, 31, 1, 843}, {17, 32, 2, 843}, {18, 33, 1, 843}, {11, 25, 1, 844}, {13, 28, 2, 844}, {14, 29, 2, 844}, {16, 32, 1, 844}, {17, 33, 2, 844}, {18, 34, 1, 844}, {12, 20, 1, 845}, {14, 21, 2, 845}, {15, 22, 2, 845}, {17, 23, 1, 845}, {18, 24, 2, 845}, {19, 25, 1, 845}, {12, 21, 1, 846}, {14, 23, 2, 846}, {15, 24, 2, 846}, {17, 26, 1, 846}, {18, 27, 2, 846}, {19, 28, 1, 846}, {12, 22, 1, 847}, {14, 24, 2, 847}, {15, 25, 2, 847}, {17, 27, 1, 847}, {18, 28, 2, 847}, {19, 29, 1, 847}, {12, 23, 1, 848}, {14, 26, 2, 848}, {15, 27, 2, 848}, {17, 30, 1, 848}, {18, 31, 2, 848}, {19, 32, 1, 848}, {12, 24, 1, 849}, {14, 27, 2, 849}, {15, 28, 2, 849}, {17, 31, 1, 849}, {18, 32, 2, 849}, {19, 33, 1, 849}, {12, 25, 1, 850}, {14, 28, 2, 850}, {15, 29, 2, 850}, {17, 32, 1, 850}, {18, 33, 2, 850}, {19, 34, 1, 850}, {10, 35, 1, 851}, {11, 36, 2, 851}, {12, 37, 2, 851}, {13, 38, 1, 851}, {14, 39, 2, 851}, {15, 40, 1, 851}, {10, 36, 1, 852}, {11, 38, 2, 852}, {12, 39, 2, 852}, {13, 41, 1, 852}, {14, 42, 2, 852}, {15, 43, 1, 852}, {10, 37, 1, 853}, {11, 39, 2, 853}, {12, 40, 2, 853}, {13, 42, 1, 853}, {14, 43, 2, 853}, {15, 44, 1, 853}, {10, 38, 1, 854}, {11, 41, 2, 854}, {12, 42, 2, 854}, {13, 45, 1, 854}, {14, 46, 2, 854}, {15, 47, 1, 854}, {10, 39, 1, 855}, {11, 42, 2, 855}, {12, 43, 2, 855}, {13, 46, 1, 855}, {14, 47, 2, 855}, {15, 48, 1, 855}, {10, 40, 1, 856}, {11, 43, 2, 856}, {12, 44, 2, 856}, {13, 47, 1, 856}, {14, 48, 2, 856}, {15, 49, 1, 856}, {10, 41, 1, 857}, {11, 45, 2, 857}, {12, 46, 2, 857}, {13, 50, 1, 857}, {14, 51, 2, 857}, {15, 52, 1, 857}, {10, 42, 1, 858}, {11, 46, 2, 858}, {12, 47, 2, 858}, {13, 51, 1, 858}, {14, 52, 2, 858}, {15, 53, 1, 858}, {10, 43, 1, 859}, {11, 47, 2, 859}, {12, 48, 2, 859}, {13, 52, 1, 859}, {14, 53, 2, 859}, {15, 54, 1, 859}, {10, 44, 1, 860}, {11, 48, 2, 860}, {12, 49, 2, 860}, {13, 53, 1, 860}, {14, 54, 2, 860}, {15, 55, 1, 860}, {11, 35, 1, 861}, {13, 36, 2, 861}, {14, 37, 2, 861}, {16, 38, 1, 861}, {17, 39, 2, 861}, {18, 40, 1, 861}, {11, 36, 1, 862}, {13, 38, 2, 862}, {14, 39, 2, 862}, {16, 41, 1, 862}, {17, 42, 2, 862}, {18, 43, 1, 862}, {11, 37, 1, 863}, {13, 39, 2, 863}, {14, 40, 2,

863}, {16, 42, 1, 863}, {17, 43, 2, 863}, {18, 44, 1, 863}, {11, 38, 1, 864}, {13, 41, 2,
864}, {14, 42, 2, 864}, {16, 45, 1, 864}, {17, 46, 2, 864}, {18, 47, 1, 864}, {11, 39, 1,
865}, {13, 42, 2, 865}, {14, 43, 2, 865}, {16, 46, 1, 865}, {17, 47, 2, 865}, {18, 48, 1,
865}, {11, 40, 1, 866}, {13, 43, 2, 866}, {14, 44, 2, 866}, {16, 47, 1, 866}, {17, 48, 2,
866}, {18, 49, 1, 866}, {11, 41, 1, 867}, {13, 45, 2, 867}, {14, 46, 2, 867}, {16, 50, 1,
867}, {17, 51, 2, 867}, {18, 52, 1, 867}, {11, 42, 1, 868}, {13, 46, 2, 868}, {14, 47, 2,
868}, {16, 51, 1, 868}, {17, 52, 2, 868}, {18, 53, 1, 868}, {11, 43, 1, 869}, {13, 47, 2,
869}, {14, 48, 2, 869}, {16, 52, 1, 869}, {17, 53, 2, 869}, {18, 54, 1, 869}, {11, 44, 1,
870}, {13, 48, 2, 870}, {14, 49, 2, 870}, {16, 53, 1, 870}, {17, 54, 2, 870}, {18, 55, 1,
870}, {12, 35, 1, 871}, {14, 36, 2, 871}, {15, 37, 2, 871}, {17, 38, 1, 871}, {18, 39, 2,
871}, {19, 40, 1, 871}, {12, 36, 1, 872}, {14, 38, 2, 872}, {15, 39, 2, 872}, {17, 41, 1,
872}, {18, 42, 2, 872}, {19, 43, 1, 872}, {12, 37, 1, 873}, {14, 39, 2, 873}, {15, 40, 2,
873}, {17, 42, 1, 873}, {18, 43, 2, 873}, {19, 44, 1, 873}, {12, 38, 1, 874}, {14, 41, 2,
874}, {15, 42, 2, 874}, {17, 45, 1, 874}, {18, 46, 2, 874}, {19, 47, 1, 874}, {12, 39, 1,
875}, {14, 42, 2, 875}, {15, 43, 2, 875}, {17, 46, 1, 875}, {18, 47, 2, 875}, {19, 48, 1,
875}, {12, 40, 1, 876}, {14, 43, 2, 876}, {15, 44, 2, 876}, {17, 47, 1, 876}, {18, 48, 2,
876}, {19, 49, 1, 876}, {12, 41, 1, 877}, {14, 45, 2, 877}, {15, 46, 2, 877}, {17, 50, 1,
877}, {18, 51, 2, 877}, {19, 52, 1, 877}, {12, 42, 1, 878}, {14, 46, 2, 878}, {15, 47, 2,
878}, {17, 51, 1, 878}, {18, 52, 2, 878}, {19, 53, 1, 878}, {12, 43, 1, 879}, {14, 47, 2,
879}, {15, 48, 2, 879}, {17, 52, 1, 879}, {18, 53, 2, 879}, {19, 54, 1, 879}, {12, 44, 1,
880}, {14, 48, 2, 880}, {15, 49, 2, 880}, {17, 53, 1, 880}, {18, 54, 2, 880}, {19, 55, 1,
880}, {10, 56, 1, 881}, {11, 57, 2, 881}, {12, 58, 2, 881}, {13, 59, 1, 881}, {14, 60, 2,
881}, {15, 61, 1, 881}, {10, 57, 1, 882}, {11, 59, 2, 882}, {12, 60, 2, 882}, {13, 62, 1,
882}, {14, 63, 2, 882}, {15, 64, 1, 882}, {10, 58, 1, 883}, {11, 60, 2, 883}, {12, 61, 2,
883}, {13, 63, 1, 883}, {14, 64, 2, 883}, {15, 65, 1, 883}, {10, 59, 1, 884}, {11, 62, 2,
884}, {12, 63, 2, 884}, {13, 66, 1, 884}, {14, 67, 2, 884}, {15, 68, 1, 884}, {10, 60, 1,
885}, {11, 63, 2, 885}, {12, 64, 2, 885}, {13, 67, 1, 885}, {14, 68, 2, 885}, {15, 69, 1,
885}, {10, 61, 1, 886}, {11, 64, 2, 886}, {12, 65, 2, 886}, {13, 68, 1, 886}, {14, 69, 2,
886}, {15, 70, 1, 886}, {10, 62, 1, 887}, {11, 66, 2, 887}, {12, 67, 2, 887}, {13, 71, 1,
887}, {14, 72, 2, 887}, {15, 73, 1, 887}, {10, 63, 1, 888}, {11, 67, 2, 888}, {12, 68, 2,
888}, {13, 72, 1, 888}, {14, 73, 2, 888}, {15, 74, 1, 888}, {10, 64, 1, 889}, {11, 68, 2,
889}, {12, 69, 2, 889}, {13, 73, 1, 889}, {14, 74, 2, 889}, {15, 75, 1, 889}, {10, 65, 1,
890}, {11, 69, 2, 890}, {12, 70, 2, 890}, {13, 74, 1, 890}, {14, 75, 2, 890}, {15, 76, 1,
890}, {10, 66, 1, 891}, {11, 71, 2, 891}, {12, 72, 2, 891}, {13, 77, 1, 891}, {14, 78, 2,
891}, {15, 79, 1, 891}, {10, 67, 1, 892}, {11, 72, 2, 892}, {12, 73, 2, 892}, {13, 78, 1,
892}, {14, 79, 2, 892}, {15, 80, 1, 892}, {10, 68, 1, 893}, {11, 73, 2, 893}, {12, 74, 2,
893}, {13, 79, 1, 893}, {14, 80, 2, 893}, {15, 81, 1, 893}, {10, 69, 1, 894}, {11, 74, 2,
894}, {12, 75, 2, 894}, {13, 80, 1, 894}, {14, 81, 2, 894}, {15, 82, 1, 894}, {10, 70, 1,
895}, {11, 75, 2, 895}, {12, 76, 2, 895}, {13, 81, 1, 895}, {14, 82, 2, 895}, {15, 83, 1,
895}, {11, 56, 1, 896}, {13, 57, 2, 896}, {14, 58, 2, 896}, {16, 59, 1, 896}, {17, 60, 2,
896}, {18, 61, 1, 896}, {11, 57, 1, 897}, {13, 59, 2, 897}, {14, 60, 2, 897}, {16, 62, 1,
897}, {17, 63, 2, 897}, {18, 64, 1, 897}, {11, 58, 1, 898}, {13, 60, 2, 898}, {14, 61, 2,
898}, {16, 63, 1, 898}, {17, 64, 2, 898}, {18, 65, 1, 898}, {11, 59, 1, 899}, {13, 62, 2,
899}, {14, 63, 2, 899}, {16, 66, 1, 899}, {17, 67, 2, 899}, {18, 68, 1, 899}, {11, 60, 1,
900}, {13, 63, 2, 900}, {14, 64, 2, 900}, {16, 67, 1, 900}, {17, 68, 2, 900}, {18, 69, 1,
900}, {11, 61, 1, 901}, {13, 64, 2, 901}, {14, 65, 2, 901}, {16, 68, 1, 901}, {17, 69, 2,
901}, {18, 70, 1, 901}, {11, 62, 1, 902}, {13, 66, 2, 902}, {14, 67, 2, 902}, {16, 71, 1,
902}, {17, 72, 2, 902}, {18, 73, 1, 902}, {11, 63, 1, 903}, {13, 67, 2, 903}, {14, 68, 2,
903}, {16, 72, 1, 903}, {17, 73, 2, 903}, {18, 74, 1, 903}, {11, 64, 1, 904}, {13, 68, 2,
904}, {14, 69, 2, 904}, {16, 73, 1, 904}, {17, 74, 2, 904}, {18, 75, 1, 904}, {11, 65, 1,
905}, {13, 69, 2, 905}, {14, 70, 2, 905}, {16, 74, 1, 905}, {17, 75, 2, 905}, {18, 76, 1,
905}, {11, 66, 1, 906}, {13, 71, 2, 906}, {14, 72, 2, 906}, {16, 77, 1, 906}, {17, 78, 2,
906}, {18, 79, 1, 906}, {11, 67, 1, 907}, {13, 72, 2, 907}, {14, 73, 2, 907}, {16, 78, 1,
907}, {17, 79, 2, 907}, {18, 80, 1, 907}, {11, 68, 1, 908}, {13, 73, 2, 908}, {14, 74, 2,
908}, {16, 79, 1, 908}, {17, 80, 2, 908}, {18, 81, 1, 908}, {11, 69, 1, 909}, {13, 74, 2,
909}, {14, 75, 2, 909}, {16, 80, 1, 909}, {17, 81, 2, 909}, {18, 82, 1, 909}, {11, 70, 1,
910}, {13, 75, 2, 910}, {14, 76, 2, 910}, {16, 81, 1, 910}, {17, 82, 2, 910}, {18, 83, 1,
910}, {12, 56, 1, 911}, {14, 57, 2, 911}, {15, 58, 2, 911}, {17, 59, 1, 911}, {18, 60, 2,
911}, {19, 61, 1, 911}, {12, 57, 1, 912}, {14, 59, 2, 912}, {15, 60, 2, 912}, {17, 62, 1,
912}, {18, 63, 2, 912}, {19, 64, 1, 912}, {12, 58, 1, 913}, {14, 60, 2, 913}, {15, 61, 2,
913}, {17, 63, 1, 913}, {18, 64, 2, 913}, {19, 65, 1, 913}, {12, 59, 1, 914}, {14, 62, 2,
914}, {15, 63, 2, 914}, {17, 66, 1, 914}, {18, 67, 2, 914}, {19, 68, 1, 914}, {12, 60, 1,
915}, {14, 63, 2, 915}, {15, 64, 2, 915}, {17, 67, 1, 915}, {18, 68, 2, 915}, {19, 69, 1,
915}, {12, 61, 1, 916}, {14, 64, 2, 916}, {15, 65, 2, 916}, {17, 68, 1, 916}, {18, 69, 2,
916}, {19, 70, 1, 916}, {12, 62, 1, 917}, {14, 66, 2, 917}, {15, 67, 2, 917}, {17, 71, 1,
917}, {18, 72, 2, 917}, {19, 73, 1, 917}, {12, 63, 1, 918}, {14, 67, 2, 918}, {15, 68, 2,
918}, {17, 72, 1, 918}, {18, 73, 2, 918}, {19, 74, 1, 918}, {12, 64, 1, 919}, {14, 68, 2,
919}, {15, 69, 2, 919}, {17, 73, 1, 919}, {18, 74, 2, 919}, {19, 75, 1, 919}, {12, 65, 1,
920}, {14, 69, 2, 920}, {15, 70, 2, 920}, {17, 74, 1, 920}, {18, 75, 2, 920}, {19, 76, 1,

920}, {12, 66, 1, 921}, {14, 71, 2, 921}, {15, 72, 2, 921}, {17, 77, 1, 921}, {18, 78, 2, 921}, {19, 79, 1, 921}, {12, 67, 1, 922}, {14, 72, 2, 922}, {15, 73, 2, 922}, {17, 78, 1, 922}, {18, 79, 2, 922}, {19, 80, 1, 922}, {12, 68, 1, 923}, {14, 73, 2, 923}, {15, 74, 2, 923}, {17, 79, 1, 923}, {18, 80, 2, 923}, {19, 81, 1, 923}, {12, 69, 1, 924}, {14, 74, 2, 924}, {15, 75, 2, 924}, {17, 80, 1, 924}, {18, 81, 2, 924}, {19, 82, 1, 924}, {12, 70, 1, 925}, {14, 75, 2, 925}, {15, 76, 2, 925}, {17, 81, 1, 925}, {18, 82, 2, 925}, {19, 83, 1, 925}, {10, 84, 1, 926}, {11, 85, 2, 926}, {12, 86, 2, 926}, {13, 87, 1, 926}, {14, 88, 2, 926}, {15, 89, 1, 926}, {10, 85, 1, 927}, {11, 87, 2, 927}, {12, 88, 2, 927}, {13, 90, 1, 927}, {14, 91, 2, 927}, {15, 92, 1, 927}, {10, 86, 1, 928}, {11, 88, 2, 928}, {12, 89, 2, 928}, {13, 91, 1, 928}, {14, 92, 2, 928}, {15, 93, 1, 928}, {10, 87, 1, 929}, {11, 90, 2, 929}, {12, 91, 2, 929}, {13, 94, 1, 929}, {14, 95, 2, 929}, {15, 96, 1, 929}, {10, 88, 1, 930}, {11, 91, 2, 930}, {12, 92, 2, 930}, {13, 95, 1, 930}, {14, 96, 2, 930}, {15, 97, 1, 930}, {10, 89, 1, 931}, {11, 92, 2, 931}, {12, 93, 2, 931}, {13, 96, 1, 931}, {14, 97, 2, 931}, {15, 98, 1, 931}, {10, 90, 1, 932}, {11, 94, 2, 932}, {12, 95, 2, 932}, {13, 99, 1, 932}, {14, 100, 2, 932}, {15, 101, 1, 932}, {10, 91, 1, 933}, {11, 95, 2, 933}, {12, 96, 2, 933}, {13, 100, 1, 933}, {14, 101, 2, 933}, {15, 102, 1, 933}, {10, 92, 1, 934}, {11, 96, 2, 934}, {12, 97, 2, 934}, {13, 101, 1, 934}, {14, 102, 2, 934}, {15, 103, 1, 934}, {10, 93, 1, 935}, {11, 97, 2, 935}, {12, 98, 2, 935}, {13, 102, 1, 935}, {14, 103, 2, 935}, {15, 104, 1, 935}, {10, 94, 1, 936}, {11, 99, 2, 936}, {12, 100, 2, 936}, {13, 105, 1, 936}, {14, 106, 2, 936}, {15, 107, 1, 936}, {10, 95, 1, 937}, {11, 100, 2, 937}, {12, 101, 2, 937}, {13, 106, 1, 937}, {14, 107, 2, 937}, {15, 108, 1, 937}, {10, 96, 1, 938}, {11, 101, 2, 938}, {12, 102, 2, 938}, {13, 107, 1, 938}, {14, 108, 2, 938}, {15, 109, 1, 938}, {10, 97, 1, 939}, {11, 102, 2, 939}, {12, 103, 2, 939}, {13, 108, 1, 939}, {14, 109, 2, 939}, {15, 110, 1, 939}, {10, 98, 1, 940}, {11, 103, 2, 940}, {12, 104, 2, 940}, {13, 109, 1, 940}, {14, 110, 2, 940}, {15, 111, 1, 940}, {10, 99, 1, 941}, {11, 105, 2, 941}, {12, 106, 2, 941}, {13, 112, 1, 941}, {14, 113, 2, 941}, {15, 114, 1, 941}, {10, 100, 1, 942}, {11, 106, 2, 942}, {12, 107, 2, 942}, {13, 113, 1, 942}, {14, 114, 2, 942}, {15, 115, 1, 942}, {10, 101, 1, 943}, {11, 107, 2, 943}, {12, 108, 2, 943}, {13, 114, 1, 943}, {14, 115, 2, 943}, {15, 116, 1, 943}, {10, 102, 1, 944}, {11, 108, 2, 944}, {12, 109, 2, 944}, {13, 115, 1, 944}, {14, 116, 2, 944}, {15, 117, 1, 944}, {10, 103, 1, 945}, {11, 109, 2, 945}, {12, 110, 2, 945}, {13, 116, 1, 945}, {14, 117, 2, 945}, {15, 118, 1, 945}, {10, 104, 1, 946}, {11, 110, 2, 946}, {12, 111, 2, 946}, {13, 117, 1, 946}, {14, 118, 2, 946}, {15, 119, 1, 946}, {11, 84, 1, 947}, {13, 85, 2, 947}, {14, 86, 2, 947}, {16, 87, 1, 947}, {17, 88, 2, 947}, {18, 89, 1, 947}, {11, 85, 1, 948}, {13, 87, 2, 948}, {14, 88, 2, 948}, {16, 90, 1, 948}, {17, 91, 2, 948}, {18, 92, 1, 948}, {11, 86, 1, 949}, {13, 88, 2, 949}, {14, 89, 2, 949}, {16, 91, 1, 949}, {17, 92, 2, 949}, {18, 93, 1, 949}, {11, 87, 1, 950}, {13, 90, 2, 950}, {14, 91, 2, 950}, {16, 94, 1, 950}, {17, 95, 2, 950}, {18, 96, 1, 950}, {11, 88, 1, 951}, {13, 91, 2, 951}, {14, 92, 2, 951}, {16, 95, 1, 951}, {17, 96, 2, 951}, {18, 97, 1, 951}, {11, 89, 1, 952}, {13, 92, 2, 952}, {14, 93, 2, 952}, {16, 96, 1, 952}, {17, 97, 2, 952}, {18, 98, 1, 952}, {11, 90, 1, 953}, {13, 94, 2, 953}, {14, 95, 2, 953}, {16, 99, 1, 953}, {17, 100, 2, 953}, {18, 101, 1, 953}, {11, 91, 1, 954}, {13, 95, 2, 954}, {14, 96, 2, 954}, {16, 100, 1, 954}, {17, 101, 2, 954}, {18, 102, 1, 954}, {11, 92, 1, 955}, {13, 96, 2, 955}, {14, 97, 2, 955}, {16, 101, 1, 955}, {17, 102, 2, 955}, {18, 103, 1, 955}, {11, 93, 1, 956}, {13, 97, 2, 956}, {14, 98, 2, 956}, {16, 102, 1, 956}, {17, 103, 2, 956}, {18, 104, 1, 956}, {11, 94, 1, 957}, {13, 99, 2, 957}, {14, 100, 2, 957}, {16, 105, 1, 957}, {17, 106, 2, 957}, {18, 107, 1, 957}, {11, 95, 1, 958}, {13, 100, 2, 958}, {14, 101, 2, 958}, {16, 106, 1, 958}, {17, 107, 2, 958}, {18, 108, 1, 958}, {11, 96, 1, 959}, {13, 101, 2, 959}, {14, 102, 2, 959}, {16, 107, 1, 959}, {17, 108, 2, 959}, {18, 109, 1, 959}, {11, 97, 1, 960}, {13, 102, 2, 960}, {14, 103, 2, 960}, {16, 108, 1, 960}, {17, 109, 2, 960}, {18, 110, 1, 960}, {11, 98, 1, 961}, {13, 103, 2, 961}, {14, 104, 2, 961}, {16, 109, 1, 961}, {17, 110, 2, 961}, {18, 111, 1, 961}, {11, 99, 1, 962}, {13, 105, 2, 962}, {14, 106, 2, 962}, {16, 112, 1, 962}, {17, 113, 2, 962}, {18, 114, 1, 962}, {11, 100, 1, 963}, {13, 106, 2, 963}, {14, 107, 2, 963}, {16, 113, 1, 963}, {17, 114, 2, 963}, {18, 115, 1, 963}, {11, 101, 1, 964}, {13, 107, 2, 964}, {14, 108, 2, 964}, {16, 114, 1, 964}, {17, 115, 2, 964}, {18, 116, 1, 964}, {11, 102, 1, 965}, {13, 108, 2, 965}, {14, 109, 2, 965}, {16, 115, 1, 965}, {17, 116, 2, 965}, {18, 117, 1, 965}, {11, 103, 1, 966}, {13, 109, 2, 966}, {14, 110, 2, 966}, {16, 116, 1, 966}, {17, 117, 2, 966}, {18, 118, 1, 966}, {11, 104, 1, 967}, {13, 110, 2, 967}, {14, 111, 2, 967}, {16, 117, 1, 967}, {17, 118, 2, 967}, {18, 119, 1, 967}, {12, 84, 1, 968}, {14, 85, 2, 968}, {15, 86, 2, 968}, {17, 87, 1, 968}, {18, 88, 2, 968}, {19, 89, 1, 968}, {12, 85, 1, 969}, {14, 87, 2, 969}, {15, 88, 2, 969}, {17, 90, 1, 969}, {18, 91, 2, 969}, {19, 92, 1, 969}, {12, 86, 1, 970}, {14, 88, 2, 970}, {15, 89, 2, 970}, {17, 91, 1, 970}, {18, 92, 2, 970}, {19, 93, 1, 970}, {12, 87, 1, 971}, {14, 90, 2, 971}, {15, 91, 2, 971}, {17, 94, 1, 971}, {18, 95, 2, 971}, {19, 96, 1, 971}, {12, 88, 1, 972}, {14, 91, 2, 972}, {15, 92, 2, 972}, {17, 95, 1, 972}, {18, 96, 2, 972}, {19, 97, 1, 972}, {12, 89, 1, 973}, {14, 92, 2, 973}, {15, 93, 2, 973}, {17, 96, 1, 973}, {18, 97, 2, 973}, {19, 98, 1, 973}, {12, 90, 1, 974}, {14, 94, 2, 974}, {15, 95, 2, 974}, {17, 99, 1, 974}, {18, 100, 2, 974}, {19, 101, 1, 974}, {12, 91, 1, 975}, {14, 95, 2, 975}, {15, 96, 2, 975}, {17, 100, 1, 975}, {18, 101, 2, 975}, {19, 102, 1, 975}, {12, 92, 1, 976}, {14, 96, 2, 976}, {15, 97, 2, 976}, {17, 101, 1,

976}, {18, 102, 2, 976}, {19, 103, 1, 976}, {12, 93, 1, 977}, {14, 97, 2, 977}, {15, 98, 2, 977}, {17, 102, 1, 977}, {18, 103, 2, 977}, {19, 104, 1, 977}, {12, 94, 1, 978}, {14, 99, 2, 978}, {15, 100, 2, 978}, {17, 105, 1, 978}, {18, 106, 2, 978}, {19, 107, 1, 978}, {12, 95, 1, 979}, {14, 100, 2, 979}, {15, 101, 2, 979}, {17, 106, 1, 979}, {18, 107, 2, 979}, {19, 108, 1, 979}, {12, 96, 1, 980}, {14, 101, 2, 980}, {15, 102, 2, 980}, {17, 107, 1, 980}, {18, 108, 2, 980}, {19, 109, 1, 980}, {12, 97, 1, 981}, {14, 102, 2, 981}, {15, 103, 2, 981}, {17, 108, 1, 981}, {18, 109, 2, 981}, {19, 110, 1, 981}, {12, 98, 1, 982}, {14, 103, 2, 982}, {15, 104, 2, 982}, {17, 109, 1, 982}, {18, 110, 2, 982}, {19, 111, 1, 982}, {12, 99, 1, 983}, {14, 105, 2, 983}, {15, 106, 2, 983}, {17, 112, 1, 983}, {18, 113, 2, 983}, {19, 114, 1, 983}, {12, 100, 1, 984}, {14, 106, 2, 984}, {15, 107, 2, 984}, {17, 113, 1, 984}, {18, 114, 2, 984}, {19, 115, 1, 984}, {12, 101, 1, 985}, {14, 107, 2, 985}, {15, 108, 2, 985}, {17, 114, 1, 985}, {18, 115, 2, 985}, {19, 116, 1, 985}, {12, 102, 1, 986}, {14, 108, 2, 986}, {15, 109, 2, 986}, {17, 115, 1, 986}, {18, 116, 2, 986}, {19, 117, 1, 986}, {12, 103, 1, 987}, {14, 109, 2, 987}, {15, 110, 2, 987}, {17, 116, 1, 987}, {18, 117, 2, 987}, {19, 118, 1, 987}, {12, 104, 1, 988}, {14, 110, 2, 988}, {15, 111, 2, 988}, {17, 117, 1, 988}, {18, 118, 2, 988}, {19, 119, 1, 988}, {10, 10, 1, 989}, {11, 11, 3, 989}, {12, 12, 3, 989}, {13, 13, 3, 989}, {14, 14, 6, 989}, {15, 15, 3, 989}, {16, 16, 1, 989}, {17, 17, 3, 989}, {18, 18, 3, 989}, {19, 19, 1, 989}, {10, 20, 1, 990}, {11, 21, 3, 990}, {12, 22, 3, 990}, {13, 23, 3, 990}, {14, 24, 6, 990}, {15, 25, 3, 990}, {16, 26, 1, 990}, {17, 27, 3, 990}, {18, 28, 3, 990}, {19, 29, 1, 990}, {10, 21, 1, 991}, {11, 23, 3, 991}, {12, 24, 3, 991}, {13, 26, 3, 991}, {14, 27, 6, 991}, {15, 28, 3, 991}, {16, 30, 1, 991}, {17, 31, 3, 991}, {18, 32, 3, 991}, {19, 33, 1, 991}, {10, 22, 1, 992}, {11, 24, 3, 992}, {12, 25, 3, 992}, {13, 27, 3, 992}, {14, 28, 6, 992}, {15, 29, 3, 992}, {16, 31, 1, 992}, {17, 32, 3, 992}, {18, 33, 3, 992}, {19, 34, 1, 992}, {10, 35, 1, 993}, {11, 36, 3, 993}, {12, 37, 3, 993}, {13, 38, 3, 993}, {14, 39, 6, 993}, {15, 40, 3, 993}, {16, 41, 1, 993}, {17, 42, 3, 993}, {18, 43, 3, 993}, {19, 44, 1, 993}, {10, 36, 1, 994}, {11, 38, 3, 994}, {12, 39, 3, 994}, {13, 41, 3, 994}, {14, 42, 6, 994}, {15, 43, 3, 994}, {16, 45, 1, 994}, {17, 46, 3, 994}, {18, 47, 3, 994}, {19, 48, 1, 994}, {10, 37, 1, 995}, {11, 39, 3, 995}, {12, 40, 3, 995}, {13, 42, 3, 995}, {14, 43, 6, 995}, {15, 44, 3, 995}, {16, 46, 1, 995}, {17, 47, 3, 995}, {18, 48, 3, 995}, {19, 49, 1, 995}, {10, 38, 1, 996}, {11, 41, 3, 996}, {12, 42, 3, 996}, {13, 45, 3, 996}, {14, 46, 6, 996}, {15, 47, 3, 996}, {16, 50, 1, 996}, {17, 51, 3, 996}, {18, 52, 3, 996}, {19, 53, 1, 996}, {10, 39, 1, 997}, {11, 42, 3, 997}, {12, 43, 3, 997}, {13, 46, 3, 997}, {14, 47, 6, 997}, {15, 48, 3, 997}, {16, 51, 1, 997}, {17, 52, 3, 997}, {18, 53, 3, 997}, {19, 54, 1, 997}, {10, 40, 1, 998}, {11, 43, 3, 998}, {12, 44, 3, 998}, {13, 47, 3, 998}, {14, 48, 6, 998}, {15, 49, 3, 998}, {16, 52, 1, 998}, {17, 53, 3, 998}, {18, 54, 3, 998}, {19, 55, 1, 998}, {10, 56, 1, 999}, {11, 57, 3, 999}, {12, 58, 3, 999}, {13, 59, 3, 999}, {14, 60, 6, 999}, {15, 61, 3, 999}, {16, 62, 1, 999}, {17, 63, 3, 999}, {18, 64, 3, 999}, {19, 65, 1, 999}, {10, 57, 1, 1000}, {11, 59, 3, 1000}, {12, 60, 3, 1000}, {13, 62, 3, 1000}, {14, 63, 6, 1000}, {15, 64, 3, 1000}, {16, 66, 1, 1000}, {17, 67, 3, 1000}, {18, 68, 3, 1000}, {19, 69, 1, 1000}, {10, 58, 1, 1001}, {11, 60, 3, 1001}, {12, 61, 3, 1001}, {13, 63, 3, 1001}, {14, 64, 6, 1001}, {15, 65, 3, 1001}, {16, 67, 1, 1001}, {17, 68, 3, 1001}, {18, 69, 3, 1001}, {19, 70, 1, 1001}, {10, 59, 1, 1002}, {11, 62, 3, 1002}, {12, 63, 3, 1002}, {13, 66, 3, 1002}, {14, 67, 6, 1002}, {15, 68, 3, 1002}, {16, 71, 1, 1002}, {17, 72, 3, 1002}, {18, 73, 3, 1002}, {19, 74, 1, 1002}, {10, 60, 1, 1003}, {11, 63, 3, 1003}, {12, 64, 3, 1003}, {13, 67, 3, 1003}, {14, 68, 6, 1003}, {15, 69, 3, 1003}, {16, 72, 1, 1003}, {17, 73, 3, 1003}, {18, 74, 3, 1003}, {19, 75, 1, 1003}, {10, 61, 1, 1004}, {11, 64, 3, 1004}, {12, 65, 3, 1004}, {13, 68, 3, 1004}, {14, 69, 6, 1004}, {15, 70, 3, 1004}, {16, 73, 1, 1004}, {17, 74, 3, 1004}, {18, 75, 3, 1004}, {19, 76, 1, 1004}, {10, 62, 1, 1005}, {11, 66, 3, 1005}, {12, 67, 3, 1005}, {13, 71, 3, 1005}, {14, 72, 6, 1005}, {15, 73, 3, 1005}, {16, 77, 1, 1005}, {17, 78, 3, 1005}, {18, 79, 3, 1005}, {19, 80, 1, 1005}, {10, 63, 1, 1006}, {11, 67, 3, 1006}, {12, 68, 3, 1006}, {13, 72, 3, 1006}, {14, 73, 6, 1006}, {15, 74, 3, 1006}, {16, 78, 1, 1006}, {17, 79, 3, 1006}, {18, 80, 3, 1006}, {19, 81, 1, 1006}, {10, 64, 1, 1007}, {11, 68, 3, 1007}, {12, 69, 3, 1007}, {13, 73, 3, 1007}, {14, 74, 6, 1007}, {15, 75, 3, 1007}, {16, 79, 1, 1007}, {17, 80, 3, 1007}, {18, 81, 3, 1007}, {19, 82, 1, 1007}, {10, 65, 1, 1008}, {11, 69, 3, 1008}, {12, 70, 3, 1008}, {13, 74, 3, 1008}, {14, 75, 6, 1008}, {15, 76, 3, 1008}, {16, 80, 1, 1008}, {17, 81, 3, 1008}, {18, 82, 3, 1008}, {19, 83, 1, 1008}, {10, 84, 1, 1009}, {11, 85, 3, 1009}, {12, 86, 3, 1009}, {13, 87, 3, 1009}, {14, 88, 6, 1009}, {15, 89, 3, 1009}, {16, 90, 1, 1009}, {17, 91, 3, 1009}, {18, 92, 3, 1009}, {19, 93, 1, 1009}, {10, 85, 1, 1010}, {11, 87, 3, 1010}, {12, 88, 3, 1010}, {13, 90, 3, 1010}, {14, 91, 6, 1010}, {15, 92, 3, 1010}, {16, 94, 1, 1010}, {17, 95, 3, 1010}, {18, 96, 3, 1010}, {19, 97, 1, 1010}, {10, 86, 1, 1011}, {11, 88, 3, 1011}, {12, 89, 3, 1011}, {13, 91, 3, 1011}, {14, 92, 6, 1011}, {15, 93, 3, 1011}, {16, 95, 1, 1011}, {17, 96, 3, 1011}, {18, 97, 3, 1011}, {19, 98, 1, 1011}, {10, 87, 1, 1012}, {11, 90, 3, 1012}, {12, 91, 3, 1012}, {13, 94, 3, 1012}, {14, 95, 6, 1012}, {15, 96, 3, 1012}, {16, 99, 1, 1012}, {17, 100, 3, 1012}, {18, 101, 3, 1012}, {19, 102, 1, 1012}, {10, 88, 1, 1013}, {11, 91, 3, 1013}, {12, 92, 3, 1013}, {13, 95, 3, 1013}, {14, 96, 6, 1013}, {15, 97, 3, 1013}, {16, 100, 1, 1013}, {17, 101, 3, 1013}, {18, 102, 3, 1013}, {19, 103, 1, 1013}, {10, 89, 1, 1014}, {11, 92, 3, 1014}, {12, 93, 3, 1014}, {13, 96, 3, 1014}, {14, 97, 6, 1014}, {15, 98, 3, 1014}, {16,

101, 1, 1014}, {17, 102, 3, 1014}, {18, 103, 3, 1014}, {19, 104, 1, 1014}, {10, 90, 1,
1015}, {11, 94, 3, 1015}, {12, 95, 3, 1015}, {13, 99, 3, 1015}, {14, 100, 6, 1015}, {15,
101, 3, 1015}, {16, 105, 1, 1015}, {17, 106, 3, 1015}, {18, 107, 3, 1015}, {19, 108, 1,
1015}, {10, 91, 1, 1016}, {11, 95, 3, 1016}, {12, 96, 3, 1016}, {13, 100, 3, 1016}, {14,
101, 6, 1016}, {15, 102, 3, 1016}, {16, 106, 1, 1016}, {17, 107, 3, 1016}, {18, 108, 3,
1016}, {19, 109, 1, 1016}, {10, 92, 1, 1017}, {11, 96, 3, 1017}, {12, 97, 3, 1017}, {13,
101, 3, 1017}, {14, 102, 6, 1017}, {15, 103, 3, 1017}, {16, 107, 1, 1017}, {17, 108, 3,
1017}, {18, 109, 3, 1017}, {19, 110, 1, 1017}, {10, 93, 1, 1018}, {11, 97, 3, 1018}, {12,
98, 3, 1018}, {13, 102, 3, 1018}, {14, 103, 6, 1018}, {15, 104, 3, 1018}, {16, 108, 1,
1018}, {17, 109, 3, 1018}, {18, 110, 3, 1018}, {19, 111, 1, 1018}, {10, 94, 1, 1019}, {11,
99, 3, 1019}, {12, 100, 3, 1019}, {13, 105, 3, 1019}, {14, 106, 6, 1019}, {15, 107, 3,
1019}, {16, 112, 1, 1019}, {17, 113, 3, 1019}, {18, 114, 3, 1019}, {19, 115, 1, 1019},
{10, 95, 1, 1020}, {11, 100, 3, 1020}, {12, 101, 3, 1020}, {13, 106, 3, 1020}, {14, 107,
6, 1020}, {15, 108, 3, 1020}, {16, 113, 1, 1020}, {17, 114, 3, 1020}, {18, 115, 3, 1020},
{19, 116, 1, 1020}, {10, 96, 1, 1021}, {11, 101, 3, 1021}, {12, 102, 3, 1021}, {13, 107,
3, 1021}, {14, 108, 6, 1021}, {15, 109, 3, 1021}, {16, 114, 1, 1021}, {17, 115, 3, 1021},
{18, 116, 3, 1021}, {19, 117, 1, 1021}, {10, 97, 1, 1022}, {11, 102, 3, 1022}, {12, 103,
3, 1022}, {13, 108, 3, 1022}, {14, 109, 6, 1022}, {15, 110, 3, 1022}, {16, 115, 1, 1022},
{17, 116, 3, 1022}, {18, 117, 3, 1022}, {19, 118, 1, 1022}, {10, 98, 1, 1023}, {11, 103,
3, 1023}, {12, 104, 3, 1023}, {13, 109, 3, 1023}, {14, 110, 6, 1023}, {15, 111, 3, 1023},
{16, 116, 1, 1023}, {17, 117, 3, 1023}, {18, 118, 3, 1023}, {19, 119, 1, 1023}, {10, 120,
1, 1024}, {11, 121, 3, 1024}, {12, 122, 3, 1024}, {13, 123, 3, 1024}, {14, 124, 6, 1024},
{15, 125, 3, 1024}, {16, 126, 1, 1024}, {17, 127, 3, 1024}, {18, 128, 3, 1024}, {19, 129,
1, 1024}, {10, 121, 1, 1025}, {11, 123, 3, 1025}, {12, 124, 3, 1025}, {13, 126, 3, 1025},
{14, 127, 6, 1025}, {15, 128, 3, 1025}, {16, 130, 1, 1025}, {17, 131, 3, 1025}, {18, 132,
3, 1025}, {19, 133, 1, 1025}, {10, 122, 1, 1026}, {11, 124, 3, 1026}, {12, 125, 3, 1026},
{13, 127, 3, 1026}, {14, 128, 6, 1026}, {15, 129, 3, 1026}, {16, 131, 1, 1026}, {17, 132,
3, 1026}, {18, 133, 3, 1026}, {19, 134, 1, 1026}, {10, 123, 1, 1027}, {11, 126, 3, 1027},
{12, 127, 3, 1027}, {13, 130, 3, 1027}, {14, 131, 6, 1027}, {15, 132, 3, 1027}, {16, 135,
1, 1027}, {17, 136, 3, 1027}, {18, 137, 3, 1027}, {19, 138, 1, 1027}, {10, 124, 1, 1028},
{11, 127, 3, 1028}, {12, 128, 3, 1028}, {13, 131, 3, 1028}, {14, 132, 6, 1028}, {15, 133,
3, 1028}, {16, 136, 1, 1028}, {17, 137, 3, 1028}, {18, 138, 3, 1028}, {19, 139, 1, 1028},
{10, 125, 1, 1029}, {11, 128, 3, 1029}, {12, 129, 3, 1029}, {13, 132, 3, 1029}, {14, 133,
6, 1029}, {15, 134, 3, 1029}, {16, 137, 1, 1029}, {17, 138, 3, 1029}, {18, 139, 3, 1029},
{19, 140, 1, 1029}, {10, 126, 1, 1030}, {11, 130, 3, 1030}, {12, 131, 3, 1030}, {13, 135,
3, 1030}, {14, 136, 6, 1030}, {15, 137, 3, 1030}, {16, 141, 1, 1030}, {17, 142, 3, 1030},
{18, 143, 3, 1030}, {19, 144, 1, 1030}, {10, 127, 1, 1031}, {11, 131, 3, 1031}, {12, 132,
3, 1031}, {13, 136, 3, 1031}, {14, 137, 6, 1031}, {15, 138, 3, 1031}, {16, 142, 1, 1031},
{17, 143, 3, 1031}, {18, 144, 3, 1031}, {19, 145, 1, 1031}, {10, 128, 1, 1032}, {11, 132,
3, 1032}, {12, 133, 3, 1032}, {13, 137, 3, 1032}, {14, 138, 6, 1032}, {15, 139, 3, 1032},
{16, 143, 1, 1032}, {17, 144, 3, 1032}, {18, 145, 3, 1032}, {19, 146, 1, 1032}, {10, 129,
1, 1033}, {11, 133, 3, 1033}, {12, 134, 3, 1033}, {13, 138, 3, 1033}, {14, 139, 6, 1033},
{15, 140, 3, 1033}, {16, 144, 1, 1033}, {17, 145, 3, 1033}, {18, 146, 3, 1033}, {19, 147,
1, 1033}, {10, 130, 1, 1034}, {11, 135, 3, 1034}, {12, 136, 3, 1034}, {13, 141, 3, 1034},
{14, 142, 6, 1034}, {15, 143, 3, 1034}, {16, 148, 1, 1034}, {17, 149, 3, 1034}, {18, 150,
3, 1034}, {19, 151, 1, 1034}, {10, 131, 1, 1035}, {11, 136, 3, 1035}, {12, 137, 3, 1035},
{13, 142, 3, 1035}, {14, 143, 6, 1035}, {15, 144, 3, 1035}, {16, 149, 1, 1035}, {17, 150,
3, 1035}, {18, 151, 3, 1035}, {19, 152, 1, 1035}, {10, 132, 1, 1036}, {11, 137, 3, 1036},
{12, 138, 3, 1036}, {13, 143, 3, 1036}, {14, 144, 6, 1036}, {15, 145, 3, 1036}, {16, 150,
1, 1036}, {17, 151, 3, 1036}, {18, 152, 3, 1036}, {19, 153, 1, 1036}, {10, 133, 1, 1037},
{11, 138, 3, 1037}, {12, 139, 3, 1037}, {13, 144, 3, 1037}, {14, 145, 6, 1037}, {15, 146,
3, 1037}, {16, 151, 1, 1037}, {17, 152, 3, 1037}, {18, 153, 3, 1037}, {19, 154, 1, 1037},
{10, 134, 1, 1038}, {11, 139, 3, 1038}, {12, 140, 3, 1038}, {13, 145, 3, 1038}, {14, 146,
6, 1038}, {15, 147, 3, 1038}, {16, 152, 1, 1038}, {17, 153, 3, 1038}, {18, 154, 3, 1038},
{19, 155, 1, 1038}, {10, 135, 1, 1039}, {11, 141, 3, 1039}, {12, 142, 3, 1039}, {13, 148,
3, 1039}, {14, 149, 6, 1039}, {15, 150, 3, 1039}, {16, 156, 1, 1039}, {17, 157, 3, 1039},
{18, 158, 3, 1039}, {19, 159, 1, 1039}, {10, 136, 1, 1040}, {11, 142, 3, 1040}, {12, 143,
3, 1040}, {13, 149, 3, 1040}, {14, 150, 6, 1040}, {15, 151, 3, 1040}, {16, 157, 1, 1040},
{17, 158, 3, 1040}, {18, 159, 3, 1040}, {19, 160, 1, 1040}, {10, 137, 1, 1041}, {11, 143,
3, 1041}, {12, 144, 3, 1041}, {13, 150, 3, 1041}, {14, 151, 6, 1041}, {15, 152, 3, 1041},
{16, 158, 1, 1041}, {17, 159, 3, 1041}, {18, 160, 3, 1041}, {19, 161, 1, 1041}, {10, 138,
1, 1042}, {11, 144, 3, 1042}, {12, 145, 3, 1042}, {13, 151, 3, 1042}, {14, 152, 6, 1042},
{15, 153, 3, 1042}, {16, 159, 1, 1042}, {17, 160, 3, 1042}, {18, 161, 3, 1042}, {19, 162,
1, 1042}, {10, 139, 1, 1043}, {11, 145, 3, 1043}, {12, 146, 3, 1043}, {13, 152, 3, 1043},
{14, 153, 6, 1043}, {15, 154, 3, 1043}, {16, 160, 1, 1043}, {17, 161, 3, 1043}, {18, 162,
3, 1043}, {19, 163, 1, 1043}, {10, 140, 1, 1044}, {11, 146, 3, 1044}, {12, 147, 3, 1044},
{13, 153, 3, 1044}, {14, 154, 6, 1044}, {15, 155, 3, 1044}, {16, 161, 1, 1044}, {17, 162,
3, 1044}, {18, 163, 3, 1044}, {19, 164, 1, 1044}, {10, 165, 1, 1045}, {11, 166, 3, 1045},
{12, 167, 3, 1045}, {13, 168, 3, 1045}, {14, 169, 6, 1045}, {15, 170, 3, 1045}, {16, 171,

1, 1045}, {17, 172, 3, 1045}, {18, 173, 3, 1045}, {19, 174, 1, 1045}, {10, 166, 1, 1046}, {11, 168, 3, 1046}, {12, 169, 3, 1046}, {13, 171, 3, 1046}, {14, 172, 6, 1046}, {15, 173, 3, 1046}, {16, 175, 1, 1046}, {17, 176, 3, 1046}, {18, 177, 3, 1046}, {19, 178, 1, 1046}, {10, 167, 1, 1047}, {11, 169, 3, 1047}, {12, 170, 3, 1047}, {13, 172, 3, 1047}, {14, 173, 6, 1047}, {15, 174, 3, 1047}, {16, 176, 1, 1047}, {17, 177, 3, 1047}, {18, 178, 3, 1047}, {19, 179, 1, 1047}, {10, 168, 1, 1048}, {11, 171, 3, 1048}, {12, 172, 3, 1048}, {13, 175, 3, 1048}, {14, 176, 6, 1048}, {15, 177, 3, 1048}, {16, 180, 1, 1048}, {17, 181, 3, 1048}, {18, 182, 3, 1048}, {19, 183, 1, 1048}, {10, 169, 1, 1049}, {11, 172, 3, 1049}, {12, 173, 3, 1049}, {13, 176, 3, 1049}, {14, 177, 6, 1049}, {15, 178, 3, 1049}, {16, 181, 1, 1049}, {17, 182, 3, 1049}, {18, 183, 3, 1049}, {19, 184, 1, 1049}, {10, 170, 1, 1050}, {11, 173, 3, 1050}, {12, 174, 3, 1050}, {13, 177, 3, 1050}, {14, 178, 6, 1050}, {15, 179, 3, 1050}, {16, 182, 1, 1050}, {17, 183, 3, 1050}, {18, 184, 3, 1050}, {19, 185, 1, 1050}, {10, 171, 1, 1051}, {11, 175, 3, 1051}, {12, 176, 3, 1051}, {13, 180, 3, 1051}, {14, 181, 6, 1051}, {15, 182, 3, 1051}, {16, 186, 1, 1051}, {17, 187, 3, 1051}, {18, 188, 3, 1051}, {19, 189, 1, 1051}, {10, 172, 1, 1052}, {11, 176, 3, 1052}, {12, 177, 3, 1052}, {13, 181, 3, 1052}, {14, 182, 6, 1052}, {15, 183, 3, 1052}, {16, 187, 1, 1052}, {17, 188, 3, 1052}, {18, 189, 3, 1052}, {19, 190, 1, 1052}, {10, 173, 1, 1053}, {11, 177, 3, 1053}, {12, 178, 3, 1053}, {13, 182, 3, 1053}, {14, 183, 6, 1053}, {15, 184, 3, 1053}, {16, 188, 1, 1053}, {17, 189, 3, 1053}, {18, 190, 3, 1053}, {19, 191, 1, 1053}, {10, 174, 1, 1054}, {11, 178, 3, 1054}, {12, 179, 3, 1054}, {13, 183, 3, 1054}, {14, 184, 6, 1054}, {15, 185, 3, 1054}, {16, 189, 1, 1054}, {17, 190, 3, 1054}, {18, 191, 3, 1054}, {19, 192, 1, 1054}, {10, 175, 1, 1055}, {11, 180, 3, 1055}, {12, 181, 3, 1055}, {13, 186, 3, 1055}, {14, 187, 6, 1055}, {15, 188, 3, 1055}, {16, 193, 1, 1055}, {17, 194, 3, 1055}, {18, 195, 3, 1055}, {19, 196, 1, 1055}, {10, 176, 1, 1056}, {11, 181, 3, 1056}, {12, 182, 3, 1056}, {13, 187, 3, 1056}, {14, 188, 6, 1056}, {15, 189, 3, 1056}, {16, 194, 1, 1056}, {17, 195, 3, 1056}, {18, 196, 3, 1056}, {19, 197, 1, 1056}, {10, 177, 1, 1057}, {11, 182, 3, 1057}, {12, 183, 3, 1057}, {13, 188, 3, 1057}, {14, 189, 6, 1057}, {15, 190, 3, 1057}, {16, 195, 1, 1057}, {17, 196, 3, 1057}, {18, 197, 3, 1057}, {19, 198, 1, 1057}, {10, 178, 1, 1058}, {11, 183, 3, 1058}, {12, 184, 3, 1058}, {13, 189, 3, 1058}, {14, 190, 6, 1058}, {15, 191, 3, 1058}, {16, 196, 1, 1058}, {17, 197, 3, 1058}, {18, 198, 3, 1058}, {19, 199, 1, 1058}, {10, 179, 1, 1059}, {11, 184, 3, 1059}, {12, 185, 3, 1059}, {13, 190, 3, 1059}, {14, 191, 6, 1059}, {15, 192, 3, 1059}, {16, 197, 1, 1059}, {17, 198, 3, 1059}, {18, 199, 3, 1059}, {19, 200, 1, 1059}, {10, 180, 1, 1060}, {11, 186, 3, 1060}, {12, 187, 3, 1060}, {13, 193, 3, 1060}, {14, 194, 6, 1060}, {15, 195, 3, 1060}, {16, 201, 1, 1060}, {17, 202, 3, 1060}, {18, 203, 3, 1060}, {19, 204, 1, 1060}, {10, 181, 1, 1061}, {11, 187, 3, 1061}, {12, 188, 3, 1061}, {13, 194, 3, 1061}, {14, 195, 6, 1061}, {15, 196, 3, 1061}, {16, 202, 1, 1061}, {17, 203, 3, 1061}, {18, 204, 3, 1061}, {19, 205, 1, 1061}, {10, 182, 1, 1062}, {11, 188, 3, 1062}, {12, 189, 3, 1062}, {13, 195, 3, 1062}, {14, 196, 6, 1062}, {15, 197, 3, 1062}, {16, 203, 1, 1062}, {17, 204, 3, 1062}, {18, 205, 3, 1062}, {19, 206, 1, 1062}, {10, 183, 1, 1063}, {11, 189, 3, 1063}, {12, 190, 3, 1063}, {13, 196, 3, 1063}, {14, 197, 6, 1063}, {15, 198, 3, 1063}, {16, 204, 1, 1063}, {17, 205, 3, 1063}, {18, 206, 3, 1063}, {19, 207, 1, 1063}, {10, 184, 1, 1064}, {11, 190, 3, 1064}, {12, 191, 3, 1064}, {13, 197, 3, 1064}, {14, 198, 6, 1064}, {15, 199, 3, 1064}, {16, 205, 1, 1064}, {17, 206, 3, 1064}, {18, 207, 3, 1064}, {19, 208, 1, 1064}, {10, 185, 1, 1065}, {11, 191, 3, 1065}, {12, 192, 3, 1065}, {13, 198, 3, 1065}, {14, 199, 6, 1065}, {15, 200, 3, 1065}, {16, 206, 1, 1065}, {17, 207, 3, 1065}, {18, 208, 3, 1065}, {19, 209, 1, 1065}, {10, 186, 1, 1066}, {11, 193, 3, 1066}, {12, 194, 3, 1066}, {13, 201, 3, 1066}, {14, 202, 6, 1066}, {15, 203, 3, 1066}, {16, 210, 1, 1066}, {17, 211, 3, 1066}, {18, 212, 3, 1066}, {19, 213, 1, 1066}, {10, 187, 1, 1067}, {11, 194, 3, 1067}, {12, 195, 3, 1067}, {13, 202, 3, 1067}, {14, 203, 6, 1067}, {15, 204, 3, 1067}, {16, 211, 1, 1067}, {17, 212, 3, 1067}, {18, 213, 3, 1067}, {19, 214, 1, 1067}, {10, 188, 1, 1068}, {11, 195, 3, 1068}, {12, 196, 3, 1068}, {13, 203, 3, 1068}, {14, 204, 6, 1068}, {15, 205, 3, 1068}, {16, 212, 1, 1068}, {17, 213, 3, 1068}, {18, 214, 3, 1068}, {19, 215, 1, 1068}, {10, 189, 1, 1069}, {11, 196, 3, 1069}, {12, 197, 3, 1069}, {13, 204, 3, 1069}, {14, 205, 6, 1069}, {15, 206, 3, 1069}, {16, 213, 1, 1069}, {17, 214, 3, 1069}, {18, 215, 3, 1069}, {19, 216, 1, 1069}, {10, 190, 1, 1070}, {11, 197, 3, 1070}, {12, 198, 3, 1070}, {13, 205, 3, 1070}, {14, 206, 6, 1070}, {15, 207, 3, 1070}, {16, 214, 1, 1070}, {17, 215, 3, 1070}, {18, 216, 3, 1070}, {19, 217, 1, 1070}, {10, 191, 1, 1071}, {11, 198, 3, 1071}, {12, 199, 3, 1071}, {13, 206, 3, 1071}, {14, 207, 6, 1071}, {15, 208, 3, 1071}, {16, 215, 1, 1071}, {17, 216, 3, 1071}, {18, 217, 3, 1071}, {19, 218, 1, 1071}, {10, 192, 1, 1072}, {11, 199, 3, 1072}, {12, 200, 3, 1072}, {13, 207, 3, 1072}, {14, 208, 6, 1072}, {15, 209, 3, 1072}, {16, 216, 1, 1072}, {17, 217, 3, 1072}, {18, 218, 3, 1072}, {19, 219, 1, 1072}, {20, 20, 1, 1073}, {21, 21, 2, 1073}, {22, 22, 2, 1073}, {23, 23, 1, 1073}, {24, 24, 2, 1073}, {25, 25, 1, 1073}, {20, 21, 1, 1074}, {21, 23, 2, 1074}, {22, 24, 2, 1074}, {23, 26, 1, 1074}, {24, 27, 2, 1074}, {25, 28, 1, 1074}, {20, 22, 1, 1075}, {21, 24, 2, 1075}, {22, 25, 2, 1075}, {23, 27, 1, 1075}, {24, 28, 2, 1075}, {25, 29, 1, 1075}, {20, 23, 1, 1076}, {21, 26, 2, 1076}, {22, 27, 2, 1076}, {23, 30, 1, 1076}, {24, 31, 2, 1076}, {25, 32, 1, 1076}, {20, 24, 1, 1077}, {21, 27, 2, 1077}, {22, 28, 2, 1077}, {23, 31, 1, 1077}, {24, 32, 2, 1077}, {25, 33, 1, 1077}, {20, 25, 1, 1078}, {21, 28, 2, 1078}, {22, 29, 2, 1078}, {23, 32, 1, 1078}, {24, 33, 2, 1078}, {25, 34, 1, 1078}, {21, 21, 1, 1079}, {23, 23, 2, 1079}, {24,

24, 2, 1079}, {26, 26, 1, 1079}, {27, 27, 2, 1079}, {28, 28, 1, 1079}, {21, 22, 1, 1080},
{23, 24, 2, 1080}, {24, 25, 2, 1080}, {26, 27, 1, 1080}, {27, 28, 2, 1080}, {28, 29, 1,
1080}, {21, 23, 1, 1081}, {23, 26, 2, 1081}, {24, 27, 2, 1081}, {26, 30, 1, 1081}, {27,
31, 2, 1081}, {28, 32, 1, 1081}, {21, 24, 1, 1082}, {23, 27, 2, 1082}, {24, 28, 2, 1082},
{26, 31, 1, 1082}, {27, 32, 2, 1082}, {28, 33, 1, 1082}, {21, 25, 1, 1083}, {23, 28, 2,
1083}, {24, 29, 2, 1083}, {26, 32, 1, 1083}, {27, 33, 2, 1083}, {28, 34, 1, 1083}, {22,
22, 1, 1084}, {24, 24, 2, 1084}, {25, 25, 2, 1084}, {27, 27, 1, 1084}, {28, 28, 2, 1084},
{29, 29, 1, 1084}, {22, 23, 1, 1085}, {24, 26, 2, 1085}, {25, 27, 2, 1085}, {27, 30, 1,
1085}, {28, 31, 2, 1085}, {29, 32, 1, 1085}, {22, 24, 1, 1086}, {24, 27, 2, 1086}, {25,
28, 2, 1086}, {27, 31, 1, 1086}, {28, 32, 2, 1086}, {29, 33, 1, 1086}, {22, 25, 1, 1087},
{24, 28, 2, 1087}, {25, 29, 2, 1087}, {27, 32, 1, 1087}, {28, 33, 2, 1087}, {29, 34, 1,
1087}, {23, 23, 1, 1088}, {26, 26, 2, 1088}, {27, 27, 2, 1088}, {30, 30, 1, 1088}, {31,
31, 2, 1088}, {32, 32, 1, 1088}, {23, 24, 1, 1089}, {26, 27, 2, 1089}, {27, 28, 2, 1089},
{30, 31, 1, 1089}, {31, 32, 2, 1089}, {32, 33, 1, 1089}, {23, 25, 1, 1090}, {26, 28, 2,
1090}, {27, 29, 2, 1090}, {30, 32, 1, 1090}, {31, 33, 2, 1090}, {32, 34, 1, 1090}, {24,
24, 1, 1091}, {27, 27, 2, 1091}, {28, 28, 2, 1091}, {31, 31, 1, 1091}, {32, 32, 2, 1091},
{33, 33, 1, 1091}, {24, 25, 1, 1092}, {27, 28, 2, 1092}, {28, 29, 2, 1092}, {31, 32, 1,
1092}, {32, 33, 2, 1092}, {33, 34, 1, 1092}, {25, 25, 1, 1093}, {28, 28, 2, 1093}, {29,
29, 2, 1093}, {32, 32, 1, 1093}, {33, 33, 2, 1093}, {34, 34, 1, 1093}, {20, 35, 1, 1094},
{21, 36, 2, 1094}, {22, 37, 2, 1094}, {23, 38, 1, 1094}, {24, 39, 2, 1094}, {25, 40, 1,
1094}, {20, 36, 1, 1095}, {21, 38, 2, 1095}, {22, 39, 2, 1095}, {23, 41, 1, 1095}, {24,
42, 2, 1095}, {25, 43, 1, 1095}, {20, 37, 1, 1096}, {21, 39, 2, 1096}, {22, 40, 2, 1096},
{23, 42, 1, 1096}, {24, 43, 2, 1096}, {25, 44, 1, 1096}, {20, 38, 1, 1097}, {21, 41, 2,
1097}, {22, 42, 2, 1097}, {23, 45, 1, 1097}, {24, 46, 2, 1097}, {25, 47, 1, 1097}, {20,
39, 1, 1098}, {21, 42, 2, 1098}, {22, 43, 2, 1098}, {23, 46, 1, 1098}, {24, 47, 2, 1098},
{25, 48, 1, 1098}, {20, 40, 1, 1099}, {21, 43, 2, 1099}, {22, 44, 2, 1099}, {23, 47, 1,
1099}, {24, 48, 2, 1099}, {25, 49, 1, 1099}, {20, 41, 1, 1100}, {21, 45, 2, 1100}, {22,
46, 2, 1100}, {23, 50, 1, 1100}, {24, 51, 2, 1100}, {25, 52, 1, 1100}, {20, 42, 1, 1101},
{21, 46, 2, 1101}, {22, 47, 2, 1101}, {23, 51, 1, 1101}, {24, 52, 2, 1101}, {25, 53, 1,
1101}, {20, 43, 1, 1102}, {21, 47, 2, 1102}, {22, 48, 2, 1102}, {23, 52, 1, 1102}, {24,
53, 2, 1102}, {25, 54, 1, 1102}, {20, 44, 1, 1103}, {21, 48, 2, 1103}, {22, 49, 2, 1103},
{23, 53, 1, 1103}, {24, 54, 2, 1103}, {25, 55, 1, 1103}, {21, 35, 1, 1104}, {23, 36, 2,
1104}, {24, 37, 2, 1104}, {26, 38, 1, 1104}, {27, 39, 2, 1104}, {28, 40, 1, 1104}, {21,
36, 1, 1105}, {23, 38, 2, 1105}, {24, 39, 2, 1105}, {26, 41, 1, 1105}, {27, 42, 2, 1105},
{28, 43, 1, 1105}, {21, 37, 1, 1106}, {23, 39, 2, 1106}, {24, 40, 2, 1106}, {26, 42, 1,
1106}, {27, 43, 2, 1106}, {28, 44, 1, 1106}, {21, 38, 1, 1107}, {23, 41, 2, 1107}, {24,
42, 2, 1107}, {26, 45, 1, 1107}, {27, 46, 2, 1107}, {28, 47, 1, 1107}, {21, 39, 1, 1108},
{23, 42, 2, 1108}, {24, 43, 2, 1108}, {26, 46, 1, 1108}, {27, 47, 2, 1108}, {28, 48, 1,
1108}, {21, 40, 1, 1109}, {23, 43, 2, 1109}, {24, 44, 2, 1109}, {26, 47, 1, 1109}, {27,
48, 2, 1109}, {28, 49, 1, 1109}, {21, 41, 1, 1110}, {23, 45, 2, 1110}, {24, 46, 2, 1110},
{26, 50, 1, 1110}, {27, 51, 2, 1110}, {28, 52, 1, 1110}, {21, 42, 1, 1111}, {23, 46, 2,
1111}, {24, 47, 2, 1111}, {26, 51, 1, 1111}, {27, 52, 2, 1111}, {28, 53, 1, 1111}, {21,
43, 1, 1112}, {23, 47, 2, 1112}, {24, 48, 2, 1112}, {26, 52, 1, 1112}, {27, 53, 2, 1112},
{28, 54, 1, 1112}, {21, 44, 1, 1113}, {23, 48, 2, 1113}, {24, 49, 2, 1113}, {26, 53, 1,
1113}, {27, 54, 2, 1113}, {28, 55, 1, 1113}, {22, 35, 1, 1114}, {24, 36, 2, 1114}, {25,
37, 2, 1114}, {27, 38, 1, 1114}, {28, 39, 2, 1114}, {29, 40, 1, 1114}, {22, 36, 1, 1115},
{24, 38, 2, 1115}, {25, 39, 2, 1115}, {27, 41, 1, 1115}, {28, 42, 2, 1115}, {29, 43, 1,
1115}, {22, 37, 1, 1116}, {24, 39, 2, 1116}, {25, 40, 2, 1116}, {27, 42, 1, 1116}, {28,
43, 2, 1116}, {29, 44, 1, 1116}, {22, 38, 1, 1117}, {24, 41, 2, 1117}, {25, 42, 2, 1117},
{27, 45, 1, 1117}, {28, 46, 2, 1117}, {29, 47, 1, 1117}, {22, 39, 1, 1118}, {24, 42, 2,
1118}, {25, 43, 2, 1118}, {27, 46, 1, 1118}, {28, 47, 2, 1118}, {29, 48, 1, 1118}, {22,
40, 1, 1119}, {24, 43, 2, 1119}, {25, 44, 2, 1119}, {27, 47, 1, 1119}, {28, 48, 2, 1119},
{29, 49, 1, 1119}, {22, 41, 1, 1120}, {24, 45, 2, 1120}, {25, 46, 2, 1120}, {27, 50, 1,
1120}, {28, 51, 2, 1120}, {29, 52, 1, 1120}, {22, 42, 1, 1121}, {24, 46, 2, 1121}, {25,
47, 2, 1121}, {27, 51, 1, 1121}, {28, 52, 2, 1121}, {29, 53, 1, 1121}, {22, 43, 1, 1122},
{24, 47, 2, 1122}, {25, 48, 2, 1122}, {27, 52, 1, 1122}, {28, 53, 2, 1122}, {29, 54, 1,
1122}, {22, 44, 1, 1123}, {24, 48, 2, 1123}, {25, 49, 2, 1123}, {27, 53, 1, 1123}, {28,
54, 2, 1123}, {29, 55, 1, 1123}, {23, 35, 1, 1124}, {26, 36, 2, 1124}, {27, 37, 2, 1124},
{30, 38, 1, 1124}, {31, 39, 2, 1124}, {32, 40, 1, 1124}, {23, 36, 1, 1125}, {26, 38, 2,
1125}, {27, 39, 2, 1125}, {30, 41, 1, 1125}, {31, 42, 2, 1125}, {32, 43, 1, 1125}, {23,
37, 1, 1126}, {26, 39, 2, 1126}, {27, 40, 2, 1126}, {30, 42, 1, 1126}, {31, 43, 2, 1126},
{32, 44, 1, 1126}, {23, 38, 1, 1127}, {26, 41, 2, 1127}, {27, 42, 2, 1127}, {30, 45, 1,
1127}, {31, 46, 2, 1127}, {32, 47, 1, 1127}, {23, 39, 1, 1128}, {26, 42, 2, 1128}, {27,
43, 2, 1128}, {30, 46, 1, 1128}, {31, 47, 2, 1128}, {32, 48, 1, 1128}, {23, 40, 1, 1129},
{26, 43, 2, 1129}, {27, 44, 2, 1129}, {30, 47, 1, 1129}, {31, 48, 2, 1129}, {32, 49, 1,
1129}, {23, 41, 1, 1130}, {26, 45, 2, 1130}, {27, 46, 2, 1130}, {30, 50, 1, 1130}, {31,
51, 2, 1130}, {32, 52, 1, 1130}, {23, 42, 1, 1131}, {26, 46, 2, 1131}, {27, 47, 2, 1131},
{30, 51, 1, 1131}, {31, 52, 2, 1131}, {32, 53, 1, 1131}, {23, 43, 1, 1132}, {26, 47, 2,
1132}, {27, 48, 2, 1132}, {30, 52, 1, 1132}, {31, 53, 2, 1132}, {32, 54, 1, 1132}, {23,

44, 1, 1133}, {26, 48, 2, 1133}, {27, 49, 2, 1133}, {30, 53, 1, 1133}, {31, 54, 2, 1133},
{32, 55, 1, 1133}, {24, 35, 1, 1134}, {27, 36, 2, 1134}, {28, 37, 2, 1134}, {31, 38, 1,
1134}, {32, 39, 2, 1134}, {33, 40, 1, 1134}, {24, 36, 1, 1135}, {27, 38, 2, 1135}, {28,
39, 2, 1135}, {31, 41, 1, 1135}, {32, 42, 2, 1135}, {33, 43, 1, 1135}, {24, 37, 1, 1136},
{27, 39, 2, 1136}, {28, 40, 2, 1136}, {31, 42, 1, 1136}, {32, 43, 2, 1136}, {33, 44, 1,
1136}, {24, 38, 1, 1137}, {27, 41, 2, 1137}, {28, 42, 2, 1137}, {31, 45, 1, 1137}, {32,
46, 2, 1137}, {33, 47, 1, 1137}, {24, 39, 1, 1138}, {27, 42, 2, 1138}, {28, 43, 2, 1138},
{31, 46, 1, 1138}, {32, 47, 2, 1138}, {33, 48, 1, 1138}, {24, 40, 1, 1139}, {27, 43, 2,
1139}, {28, 44, 2, 1139}, {31, 47, 1, 1139}, {32, 48, 2, 1139}, {33, 49, 1, 1139}, {24,
41, 1, 1140}, {27, 45, 2, 1140}, {28, 46, 2, 1140}, {31, 50, 1, 1140}, {32, 51, 2, 1140},
{33, 52, 1, 1140}, {24, 42, 1, 1141}, {27, 46, 2, 1141}, {28, 47, 2, 1141}, {31, 51, 1,
1141}, {32, 52, 2, 1141}, {33, 53, 1, 1141}, {24, 43, 1, 1142}, {27, 47, 2, 1142}, {28,
48, 2, 1142}, {31, 52, 1, 1142}, {32, 53, 2, 1142}, {33, 54, 1, 1142}, {24, 44, 1, 1143},
{27, 48, 2, 1143}, {28, 49, 2, 1143}, {31, 53, 1, 1143}, {32, 54, 2, 1143}, {33, 55, 1,
1143}, {25, 35, 1, 1144}, {28, 36, 2, 1144}, {29, 37, 2, 1144}, {32, 38, 1, 1144}, {33,
39, 2, 1144}, {34, 40, 1, 1144}, {25, 36, 1, 1145}, {28, 38, 2, 1145}, {29, 39, 2, 1145},
{32, 41, 1, 1145}, {33, 42, 2, 1145}, {34, 43, 1, 1145}, {25, 37, 1, 1146}, {28, 39, 2,
1146}, {29, 40, 2, 1146}, {32, 42, 1, 1146}, {33, 43, 2, 1146}, {34, 44, 1, 1146}, {25,
38, 1, 1147}, {28, 41, 2, 1147}, {29, 42, 2, 1147}, {32, 45, 1, 1147}, {33, 46, 2, 1147},
{34, 47, 1, 1147}, {25, 39, 1, 1148}, {28, 42, 2, 1148}, {29, 43, 2, 1148}, {32, 46, 1,
1148}, {33, 47, 2, 1148}, {34, 48, 1, 1148}, {25, 40, 1, 1149}, {28, 43, 2, 1149}, {29,
44, 2, 1149}, {32, 47, 1, 1149}, {33, 48, 2, 1149}, {34, 49, 1, 1149}, {25, 41, 1, 1150},
{28, 45, 2, 1150}, {29, 46, 2, 1150}, {32, 50, 1, 1150}, {33, 51, 2, 1150}, {34, 52, 1,
1150}, {25, 42, 1, 1151}, {28, 46, 2, 1151}, {29, 47, 2, 1151}, {32, 51, 1, 1151}, {33,
52, 2, 1151}, {34, 53, 1, 1151}, {25, 43, 1, 1152}, {28, 47, 2, 1152}, {29, 48, 2, 1152},
{32, 52, 1, 1152}, {33, 53, 2, 1152}, {34, 54, 1, 1152}, {25, 44, 1, 1153}, {28, 48, 2,
1153}, {29, 49, 2, 1153}, {32, 53, 1, 1153}, {33, 54, 2, 1153}, {34, 55, 1, 1153}, {20,
56, 1, 1154}, {21, 57, 2, 1154}, {22, 58, 2, 1154}, {23, 59, 1, 1154}, {24, 60, 2, 1154},
{25, 61, 1, 1154}, {20, 57, 1, 1155}, {21, 59, 2, 1155}, {22, 60, 2, 1155}, {23, 62, 1,
1155}, {24, 63, 2, 1155}, {25, 64, 1, 1155}, {20, 58, 1, 1156}, {21, 60, 2, 1156}, {22,
61, 2, 1156}, {23, 63, 1, 1156}, {24, 64, 2, 1156}, {25, 65, 1, 1156}, {20, 59, 1, 1157},
{21, 62, 2, 1157}, {22, 63, 2, 1157}, {23, 66, 1, 1157}, {24, 67, 2, 1157}, {25, 68, 1,
1157}, {20, 60, 1, 1158}, {21, 63, 2, 1158}, {22, 64, 2, 1158}, {23, 67, 1, 1158}, {24,
68, 2, 1158}, {25, 69, 1, 1158}, {20, 61, 1, 1159}, {21, 64, 2, 1159}, {22, 65, 2, 1159},
{23, 68, 1, 1159}, {24, 69, 2, 1159}, {25, 70, 1, 1159}, {20, 62, 1, 1160}, {21, 66, 2,
1160}, {22, 67, 2, 1160}, {23, 71, 1, 1160}, {24, 72, 2, 1160}, {25, 73, 1, 1160}, {20,
63, 1, 1161}, {21, 67, 2, 1161}, {22, 68, 2, 1161}, {23, 72, 1, 1161}, {24, 73, 2, 1161},
{25, 74, 1, 1161}, {20, 64, 1, 1162}, {21, 68, 2, 1162}, {22, 69, 2, 1162}, {23, 73, 1,
1162}, {24, 74, 2, 1162}, {25, 75, 1, 1162}, {20, 65, 1, 1163}, {21, 69, 2, 1163}, {22,
70, 2, 1163}, {23, 74, 1, 1163}, {24, 75, 2, 1163}, {25, 76, 1, 1163}, {20, 66, 1, 1164},
{21, 71, 2, 1164}, {22, 72, 2, 1164}, {23, 77, 1, 1164}, {24, 78, 2, 1164}, {25, 79, 1,
1164}, {20, 67, 1, 1165}, {21, 72, 2, 1165}, {22, 73, 2, 1165}, {23, 78, 1, 1165}, {24,
79, 2, 1165}, {25, 80, 1, 1165}, {20, 68, 1, 1166}, {21, 73, 2, 1166}, {22, 74, 2, 1166},
{23, 79, 1, 1166}, {24, 80, 2, 1166}, {25, 81, 1, 1166}, {20, 69, 1, 1167}, {21, 74, 2,
1167}, {22, 75, 2, 1167}, {23, 80, 1, 1167}, {24, 81, 2, 1167}, {25, 82, 1, 1167}, {20,
70, 1, 1168}, {21, 75, 2, 1168}, {22, 76, 2, 1168}, {23, 81, 1, 1168}, {24, 82, 2, 1168},
{25, 83, 1, 1168}, {21, 56, 1, 1169}, {23, 57, 2, 1169}, {24, 58, 2, 1169}, {26, 59, 1,
1169}, {27, 60, 2, 1169}, {28, 61, 1, 1169}, {21, 57, 1, 1170}, {23, 59, 2, 1170}, {24,
60, 2, 1170}, {26, 62, 1, 1170}, {27, 63, 2, 1170}, {28, 64, 1, 1170}, {21, 58, 1, 1171},
{23, 60, 2, 1171}, {24, 61, 2, 1171}, {26, 63, 1, 1171}, {27, 64, 2, 1171}, {28, 65, 1,
1171}, {21, 59, 1, 1172}, {23, 62, 2, 1172}, {24, 63, 2, 1172}, {26, 66, 1, 1172}, {27,
67, 2, 1172}, {28, 68, 1, 1172}, {21, 60, 1, 1173}, {23, 63, 2, 1173}, {24, 64, 2, 1173},
{26, 67, 1, 1173}, {27, 68, 2, 1173}, {28, 69, 1, 1173}, {21, 61, 1, 1174}, {23, 64, 2,
1174}, {24, 65, 2, 1174}, {26, 68, 1, 1174}, {27, 69, 2, 1174}, {28, 70, 1, 1174}, {21,
62, 1, 1175}, {23, 66, 2, 1175}, {24, 67, 2, 1175}, {26, 71, 1, 1175}, {27, 72, 2, 1175},
{28, 73, 1, 1175}, {21, 63, 1, 1176}, {23, 67, 2, 1176}, {24, 68, 2, 1176}, {26, 72, 1,
1176}, {27, 73, 2, 1176}, {28, 74, 1, 1176}, {21, 64, 1, 1177}, {23, 68, 2, 1177}, {24,
69, 2, 1177}, {26, 73, 1, 1177}, {27, 74, 2, 1177}, {28, 75, 1, 1177}, {21, 65, 1, 1178},
{23, 69, 2, 1178}, {24, 70, 2, 1178}, {26, 74, 1, 1178}, {27, 75, 2, 1178}, {28, 76, 1,
1178}, {21, 66, 1, 1179}, {23, 71, 2, 1179}, {24, 72, 2, 1179}, {26, 77, 1, 1179}, {27,
78, 2, 1179}, {28, 79, 1, 1179}, {21, 67, 1, 1180}, {23, 72, 2, 1180}, {24, 73, 2, 1180},
{26, 78, 1, 1180}, {27, 79, 2, 1180}, {28, 80, 1, 1180}, {21, 68, 1, 1181}, {23, 73, 2,
1181}, {24, 74, 2, 1181}, {26, 79, 1, 1181}, {27, 80, 2, 1181}, {28, 81, 1, 1181}, {21,
69, 1, 1182}, {23, 74, 2, 1182}, {24, 75, 2, 1182}, {26, 80, 1, 1182}, {27, 81, 2, 1182},
{28, 82, 1, 1182}, {21, 70, 1, 1183}, {23, 75, 2, 1183}, {24, 76, 2, 1183}, {26, 81, 1,
1183}, {27, 82, 2, 1183}, {28, 83, 1, 1183}, {22, 56, 1, 1184}, {24, 57, 2, 1184}, {25,
58, 2, 1184}, {27, 59, 1, 1184}, {28, 60, 2, 1184}, {29, 61, 1, 1184}, {22, 57, 1, 1185},
{24, 59, 2, 1185}, {25, 60, 2, 1185}, {27, 62, 1, 1185}, {28, 63, 2, 1185}, {29, 64, 1,
1185}, {22, 58, 1, 1186}, {24, 60, 2, 1186}, {25, 61, 2, 1186}, {27, 63, 1, 1186}, {28,

64, 2, 1186}, {29, 65, 1, 1186}, {22, 59, 1, 1187}, {24, 62, 2, 1187}, {25, 63, 2, 1187}, {27, 66, 1, 1187}, {28, 67, 2, 1187}, {29, 68, 1, 1187}, {22, 60, 1, 1188}, {24, 63, 2, 1188}, {25, 64, 2, 1188}, {27, 67, 1, 1188}, {28, 68, 2, 1188}, {29, 69, 1, 1188}, {22, 61, 1, 1189}, {24, 64, 2, 1189}, {25, 65, 2, 1189}, {27, 68, 1, 1189}, {28, 69, 2, 1189}, {29, 70, 1, 1189}, {22, 62, 1, 1190}, {24, 66, 2, 1190}, {25, 67, 2, 1190}, {27, 71, 1, 1190}, {28, 72, 2, 1190}, {29, 73, 1, 1190}, {22, 63, 1, 1191}, {24, 67, 2, 1191}, {25, 68, 2, 1191}, {27, 72, 1, 1191}, {28, 73, 2, 1191}, {29, 74, 1, 1191}, {22, 64, 1, 1192}, {24, 68, 2, 1192}, {25, 69, 2, 1192}, {27, 73, 1, 1192}, {28, 74, 2, 1192}, {29, 75, 1, 1192}, {22, 65, 1, 1193}, {24, 69, 2, 1193}, {25, 70, 2, 1193}, {27, 74, 1, 1193}, {28, 75, 2, 1193}, {29, 76, 1, 1193}, {22, 66, 1, 1194}, {24, 71, 2, 1194}, {25, 72, 2, 1194}, {27, 77, 1, 1194}, {28, 78, 2, 1194}, {29, 79, 1, 1194}, {22, 67, 1, 1195}, {24, 72, 2, 1195}, {25, 73, 2, 1195}, {27, 78, 1, 1195}, {28, 79, 2, 1195}, {29, 80, 1, 1195}, {22, 68, 1, 1196}, {24, 73, 2, 1196}, {25, 74, 2, 1196}, {27, 79, 1, 1196}, {28, 80, 2, 1196}, {29, 81, 1, 1196}, {22, 69, 1, 1197}, {24, 74, 2, 1197}, {25, 75, 2, 1197}, {27, 80, 1, 1197}, {28, 81, 2, 1197}, {29, 82, 1, 1197}, {22, 70, 1, 1198}, {24, 75, 2, 1198}, {25, 76, 2, 1198}, {27, 81, 1, 1198}, {28, 82, 2, 1198}, {29, 83, 1, 1198}, {23, 56, 1, 1199}, {26, 57, 2, 1199}, {27, 58, 2, 1199}, {30, 59, 1, 1199}, {31, 60, 2, 1199}, {32, 61, 1, 1199}, {23, 57, 1, 1200}, {26, 59, 2, 1200}, {27, 60, 2, 1200}, {30, 62, 1, 1200}, {31, 63, 2, 1200}, {32, 64, 1, 1200}, {23, 58, 1, 1201}, {26, 60, 2, 1201}, {27, 61, 2, 1201}, {30, 63, 1, 1201}, {31, 64, 2, 1201}, {32, 65, 1, 1201}, {23, 59, 1, 1202}, {26, 62, 2, 1202}, {27, 63, 2, 1202}, {30, 66, 1, 1202}, {31, 67, 2, 1202}, {32, 68, 1, 1202}, {23, 60, 1, 1203}, {26, 63, 2, 1203}, {27, 64, 2, 1203}, {30, 67, 1, 1203}, {31, 68, 2, 1203}, {32, 69, 1, 1203}, {23, 61, 1, 1204}, {26, 64, 2, 1204}, {27, 65, 2, 1204}, {30, 68, 1, 1204}, {31, 69, 2, 1204}, {32, 70, 1, 1204}, {23, 62, 1, 1205}, {26, 66, 2, 1205}, {27, 67, 2, 1205}, {30, 71, 1, 1205}, {31, 72, 2, 1205}, {32, 73, 1, 1205}, {23, 63, 1, 1206}, {26, 67, 2, 1206}, {27, 68, 2, 1206}, {30, 72, 1, 1206}, {31, 73, 2, 1206}, {32, 74, 1, 1206}, {23, 64, 1, 1207}, {26, 68, 2, 1207}, {27, 69, 2, 1207}, {30, 73, 1, 1207}, {31, 74, 2, 1207}, {32, 75, 1, 1207}, {23, 65, 1, 1208}, {26, 69, 2, 1208}, {27, 70, 2, 1208}, {30, 74, 1, 1208}, {31, 75, 2, 1208}, {32, 76, 1, 1208}, {23, 66, 1, 1209}, {26, 71, 2, 1209}, {27, 72, 2, 1209}, {30, 77, 1, 1209}, {31, 78, 2, 1209}, {32, 79, 1, 1209}, {23, 67, 1, 1210}, {26, 72, 2, 1210}, {27, 73, 2, 1210}, {30, 78, 1, 1210}, {31, 79, 2, 1210}, {32, 80, 1, 1210}, {23, 68, 1, 1211}, {26, 73, 2, 1211}, {27, 74, 2, 1211}, {30, 79, 1, 1211}, {31, 80, 2, 1211}, {32, 81, 1, 1211}, {23, 69, 1, 1212}, {26, 74, 2, 1212}, {27, 75, 2, 1212}, {30, 80, 1, 1212}, {31, 81, 2, 1212}, {32, 82, 1, 1212}, {23, 70, 1, 1213}, {26, 75, 2, 1213}, {27, 76, 2, 1213}, {30, 81, 1, 1213}, {31, 82, 2, 1213}, {32, 83, 1, 1213}, {24, 56, 1, 1214}, {27, 57, 2, 1214}, {28, 58, 2, 1214}, {31, 59, 1, 1214}, {32, 60, 2, 1214}, {33, 61, 1, 1214}, {24, 57, 1, 1215}, {27, 59, 2, 1215}, {28, 60, 2, 1215}, {31, 62, 1, 1215}, {32, 63, 2, 1215}, {33, 64, 1, 1215}, {24, 58, 1, 1216}, {27, 60, 2, 1216}, {28, 61, 2, 1216}, {31, 63, 1, 1216}, {32, 64, 2, 1216}, {33, 65, 1, 1216}, {24, 59, 1, 1217}, {27, 62, 2, 1217}, {28, 63, 2, 1217}, {31, 66, 1, 1217}, {32, 67, 2, 1217}, {33, 68, 1, 1217}, {24, 60, 1, 1218}, {27, 63, 2, 1218}, {28, 64, 2, 1218}, {31, 67, 1, 1218}, {32, 68, 2, 1218}, {33, 69, 1, 1218}, {24, 61, 1, 1219}, {27, 64, 2, 1219}, {28, 65, 2, 1219}, {31, 68, 1, 1219}, {32, 69, 2, 1219}, {33, 70, 1, 1219}, {24, 62, 1, 1220}, {27, 66, 2, 1220}, {28, 67, 2, 1220}, {31, 71, 1, 1220}, {32, 72, 2, 1220}, {33, 73, 1, 1220}, {24, 63, 1, 1221}, {27, 67, 2, 1221}, {28, 68, 2, 1221}, {31, 72, 1, 1221}, {32, 73, 2, 1221}, {33, 74, 1, 1221}, {24, 64, 1, 1222}, {27, 68, 2, 1222}, {28, 69, 2, 1222}, {31, 73, 1, 1222}, {32, 74, 2, 1222}, {33, 75, 1, 1222}, {24, 65, 1, 1223}, {27, 69, 2, 1223}, {28, 70, 2, 1223}, {31, 74, 1, 1223}, {32, 75, 2, 1223}, {33, 76, 1, 1223}, {24, 66, 1, 1224}, {27, 71, 2, 1224}, {28, 72, 2, 1224}, {31, 77, 1, 1224}, {32, 78, 2, 1224}, {33, 79, 1, 1224}, {24, 67, 1, 1225}, {27, 72, 2, 1225}, {28, 73, 2, 1225}, {31, 78, 1, 1225}, {32, 79, 2, 1225}, {33, 80, 1, 1225}, {24, 68, 1, 1226}, {27, 73, 2, 1226}, {28, 74, 2, 1226}, {31, 79, 1, 1226}, {32, 80, 2, 1226}, {33, 81, 1, 1226}, {24, 69, 1, 1227}, {27, 74, 2, 1227}, {28, 75, 2, 1227}, {31, 80, 1, 1227}, {32, 81, 2, 1227}, {33, 82, 1, 1227}, {24, 70, 1, 1228}, {27, 75, 2, 1228}, {28, 76, 2, 1228}, {31, 81, 1, 1228}, {32, 82, 2, 1228}, {33, 83, 1, 1228}, {25, 56, 1, 1229}, {28, 57, 2, 1229}, {29, 58, 2, 1229}, {32, 59, 1, 1229}, {33, 60, 2, 1229}, {34, 61, 1, 1229}, {25, 57, 1, 1230}, {28, 59, 2, 1230}, {29, 60, 2, 1230}, {32, 62, 1, 1230}, {33, 63, 2, 1230}, {34, 64, 1, 1230}, {25, 58, 1, 1231}, {28, 60, 2, 1231}, {29, 61, 2, 1231}, {32, 63, 1, 1231}, {33, 64, 2, 1231}, {34, 65, 1, 1231}, {25, 59, 1, 1232}, {28, 62, 2, 1232}, {29, 63, 2, 1232}, {32, 66, 1, 1232}, {33, 67, 2, 1232}, {34, 68, 1, 1232}, {25, 60, 1, 1233}, {28, 63, 2, 1233}, {29, 64, 2, 1233}, {32, 67, 1, 1233}, {33, 68, 2, 1233}, {34, 69, 1, 1233}, {25, 61, 1, 1234}, {28, 64, 2, 1234}, {29, 65, 2, 1234}, {32, 68, 1, 1234}, {33, 69, 2, 1234}, {34, 70, 1, 1234}, {25, 62, 1, 1235}, {28, 66, 2, 1235}, {29, 67, 2, 1235}, {32, 71, 1, 1235}, {33, 72, 2, 1235}, {34, 73, 1, 1235}, {25, 63, 1, 1236}, {28, 67, 2, 1236}, {29, 68, 2, 1236}, {32, 72, 1, 1236}, {33, 73, 2, 1236}, {34, 74, 1, 1236}, {25, 64, 1, 1237}, {28, 68, 2, 1237}, {29, 69, 2, 1237}, {32, 73, 1, 1237}, {33, 74, 2, 1237}, {34, 75, 1, 1237}, {25, 65, 1, 1238}, {28, 69, 2, 1238}, {29, 70, 2, 1238}, {32, 74, 1, 1238}, {33, 75, 2, 1238}, {34, 76, 1, 1238}, {25, 66, 1, 1239}, {28, 71, 2, 1239}, {29, 72, 2, 1239}, {32, 77, 1, 1239}, {33, 78, 2, 1239}, {34, 79, 1, 1239}, {25, 67, 1, 1240}, {28, 72, 2, 1240}, {29,

73, 2, 1240}, {32, 78, 1, 1240}, {33, 79, 2, 1240}, {34, 80, 1, 1240}, {25, 68, 1, 1241}, {28, 73, 2, 1241}, {29, 74, 2, 1241}, {32, 79, 1, 1241}, {33, 80, 2, 1241}, {34, 81, 1, 1241}, {25, 69, 1, 1242}, {28, 74, 2, 1242}, {29, 75, 2, 1242}, {32, 80, 1, 1242}, {33, 81, 2, 1242}, {34, 82, 1, 1242}, {25, 70, 1, 1243}, {28, 75, 2, 1243}, {29, 76, 2, 1243}, {32, 81, 1, 1243}, {33, 82, 2, 1243}, {34, 83, 1, 1243}, {20, 84, 1, 1244}, {21, 85, 2, 1244}, {22, 86, 2, 1244}, {23, 87, 1, 1244}, {24, 88, 2, 1244}, {25, 89, 1, 1244}, {20, 85, 1, 1245}, {21, 87, 2, 1245}, {22, 88, 2, 1245}, {23, 90, 1, 1245}, {24, 91, 2, 1245}, {25, 92, 1, 1245}, {20, 86, 1, 1246}, {21, 88, 2, 1246}, {22, 89, 2, 1246}, {23, 91, 1, 1246}, {24, 92, 2, 1246}, {25, 93, 1, 1246}, {20, 87, 1, 1247}, {21, 90, 2, 1247}, {22, 91, 2, 1247}, {23, 94, 1, 1247}, {24, 95, 2, 1247}, {25, 96, 1, 1247}, {20, 88, 1, 1248}, {21, 91, 2, 1248}, {22, 92, 2, 1248}, {23, 95, 1, 1248}, {24, 96, 2, 1248}, {25, 97, 1, 1248}, {20, 89, 1, 1249}, {21, 92, 2, 1249}, {22, 93, 2, 1249}, {23, 96, 1, 1249}, {24, 97, 2, 1249}, {25, 98, 1, 1249}, {20, 90, 1, 1250}, {21, 94, 2, 1250}, {22, 95, 2, 1250}, {23, 99, 1, 1250}, {24, 100, 2, 1250}, {25, 101, 1, 1250}, {20, 91, 1, 1251}, {21, 95, 2, 1251}, {22, 96, 2, 1251}, {23, 100, 1, 1251}, {24, 101, 2, 1251}, {25, 102, 1, 1251}, {20, 92, 1, 1252}, {21, 96, 2, 1252}, {22, 97, 2, 1252}, {23, 101, 1, 1252}, {24, 102, 2, 1252}, {25, 103, 1, 1252}, {20, 93, 1, 1253}, {21, 97, 2, 1253}, {22, 98, 2, 1253}, {23, 102, 1, 1253}, {24, 103, 2, 1253}, {25, 104, 1, 1253}, {20, 94, 1, 1254}, {21, 99, 2, 1254}, {22, 100, 2, 1254}, {23, 105, 1, 1254}, {24, 106, 2, 1254}, {25, 107, 1, 1254}, {20, 95, 1, 1255}, {21, 100, 2, 1255}, {22, 101, 2, 1255}, {23, 106, 1, 1255}, {24, 107, 2, 1255}, {25, 108, 1, 1255}, {20, 96, 1, 1256}, {21, 101, 2, 1256}, {22, 102, 2, 1256}, {23, 107, 1, 1256}, {24, 108, 2, 1256}, {25, 109, 1, 1256}, {20, 97, 1, 1257}, {21, 102, 2, 1257}, {22, 103, 2, 1257}, {23, 108, 1, 1257}, {24, 109, 2, 1257}, {25, 110, 1, 1257}, {20, 98, 1, 1258}, {21, 103, 2, 1258}, {22, 104, 2, 1258}, {23, 109, 1, 1258}, {24, 110, 2, 1258}, {25, 111, 1, 1258}, {20, 99, 1, 1259}, {21, 105, 2, 1259}, {22, 106, 2, 1259}, {23, 112, 1, 1259}, {24, 113, 2, 1259}, {25, 114, 1, 1259}, {20, 100, 1, 1260}, {21, 106, 2, 1260}, {22, 107, 2, 1260}, {23, 113, 1, 1260}, {24, 114, 2, 1260}, {25, 115, 1, 1260}, {20, 101, 1, 1261}, {21, 107, 2, 1261}, {22, 108, 2, 1261}, {23, 114, 1, 1261}, {24, 115, 2, 1261}, {25, 116, 1, 1261}, {20, 102, 1, 1262}, {21, 108, 2, 1262}, {22, 109, 2, 1262}, {23, 115, 1, 1262}, {24, 116, 2, 1262}, {25, 117, 1, 1262}, {20, 103, 1, 1263}, {21, 109, 2, 1263}, {22, 110, 2, 1263}, {23, 116, 1, 1263}, {24, 117, 2, 1263}, {25, 118, 1, 1263}, {20, 104, 1, 1264}, {21, 110, 2, 1264}, {22, 111, 2, 1264}, {23, 117, 1, 1264}, {24, 118, 2, 1264}, {25, 119, 1, 1264}, {21, 84, 1, 1265}, {23, 85, 2, 1265}, {24, 86, 2, 1265}, {26, 87, 1, 1265}, {27, 88, 2, 1265}, {28, 89, 1, 1265}, {21, 85, 1, 1266}, {23, 87, 2, 1266}, {24, 88, 2, 1266}, {26, 90, 1, 1266}, {27, 91, 2, 1266}, {28, 92, 1, 1266}, {21, 86, 1, 1267}, {23, 88, 2, 1267}, {24, 89, 2, 1267}, {26, 91, 1, 1267}, {27, 92, 2, 1267}, {28, 93, 1, 1267}, {21, 87, 1, 1268}, {23, 90, 2, 1268}, {24, 91, 2, 1268}, {26, 94, 1, 1268}, {27, 95, 2, 1268}, {28, 96, 1, 1268}, {21, 88, 1, 1269}, {23, 91, 2, 1269}, {24, 92, 2, 1269}, {26, 95, 1, 1269}, {27, 96, 2, 1269}, {28, 97, 1, 1269}, {21, 89, 1, 1270}, {23, 92, 2, 1270}, {24, 93, 2, 1270}, {26, 96, 1, 1270}, {27, 97, 2, 1270}, {28, 98, 1, 1270}, {21, 90, 1, 1271}, {23, 94, 2, 1271}, {24, 95, 2, 1271}, {26, 99, 1, 1271}, {27, 100, 2, 1271}, {28, 101, 1, 1271}, {21, 91, 1, 1272}, {23, 95, 2, 1272}, {24, 96, 2, 1272}, {26, 100, 1, 1272}, {27, 101, 2, 1272}, {28, 102, 1, 1272}, {21, 92, 1, 1273}, {23, 96, 2, 1273}, {24, 97, 2, 1273}, {26, 101, 1, 1273}, {27, 102, 2, 1273}, {28, 103, 1, 1273}, {21, 93, 1, 1274}, {23, 97, 2, 1274}, {24, 98, 2, 1274}, {26, 102, 1, 1274}, {27, 103, 2, 1274}, {28, 104, 1, 1274}, {21, 94, 1, 1275}, {23, 99, 2, 1275}, {24, 100, 2, 1275}, {26, 105, 1, 1275}, {27, 106, 2, 1275}, {28, 107, 1, 1275}, {21, 95, 1, 1276}, {23, 100, 2, 1276}, {24, 101, 2, 1276}, {26, 106, 1, 1276}, {27, 107, 2, 1276}, {28, 108, 1, 1276}, {21, 96, 1, 1277}, {23, 101, 2, 1277}, {24, 102, 2, 1277}, {26, 107, 1, 1277}, {27, 108, 2, 1277}, {28, 109, 1, 1277}, {21, 97, 1, 1278}, {23, 102, 2, 1278}, {24, 103, 2, 1278}, {26, 108, 1, 1278}, {27, 109, 2, 1278}, {28, 110, 1, 1278}, {21, 98, 1, 1279}, {23, 103, 2, 1279}, {24, 104, 2, 1279}, {26, 109, 1, 1279}, {27, 110, 2, 1279}, {28, 111, 1, 1279}, {21, 99, 1, 1280}, {23, 105, 2, 1280}, {24, 106, 2, 1280}, {26, 112, 1, 1280}, {27, 113, 2, 1280}, {28, 114, 1, 1280}, {21, 100, 1, 1281}, {23, 106, 2, 1281}, {24, 107, 2, 1281}, {26, 113, 1, 1281}, {27, 114, 2, 1281}, {28, 115, 1, 1281}, {21, 101, 1, 1282}, {23, 107, 2, 1282}, {24, 108, 2, 1282}, {26, 114, 1, 1282}, {27, 115, 2, 1282}, {28, 116, 1, 1282}, {21, 102, 1, 1283}, {23, 108, 2, 1283}, {24, 109, 2, 1283}, {26, 115, 1, 1283}, {27, 116, 2, 1283}, {28, 117, 1, 1283}, {21, 103, 1, 1284}, {23, 109, 2, 1284}, {24, 110, 2, 1284}, {26, 116, 1, 1284}, {27, 117, 2, 1284}, {28, 118, 1, 1284}, {21, 104, 1, 1285}, {23, 110, 2, 1285}, {24, 111, 2, 1285}, {26, 117, 1, 1285}, {27, 118, 2, 1285}, {28, 119, 1, 1285}, {22, 84, 1, 1286}, {24, 85, 2, 1286}, {25, 86, 2, 1286}, {27, 87, 1, 1286}, {28, 88, 2, 1286}, {29, 89, 1, 1286}, {22, 85, 1, 1287}, {24, 87, 2, 1287}, {25, 88, 2, 1287}, {27, 90, 1, 1287}, {28, 91, 2, 1287}, {29, 92, 1, 1287}, {22, 86, 1, 1288}, {24, 88, 2, 1288}, {25, 89, 2, 1288}, {27, 91, 1, 1288}, {28, 92, 2, 1288}, {29, 93, 1, 1288}, {22, 87, 1, 1289}, {24, 90, 2, 1289}, {25, 91, 2, 1289}, {27, 94, 1, 1289}, {28, 95, 2, 1289}, {29, 96, 1, 1289}, {22, 88, 1, 1290}, {24, 91, 2, 1290}, {25, 92, 2, 1290}, {27, 95, 1, 1290}, {28, 96, 2, 1290}, {29, 97, 1, 1290}, {22, 89, 1, 1291}, {24, 92, 2, 1291}, {25, 93, 2, 1291}, {27, 96, 1, 1291}, {28, 97, 2, 1291}, {29, 98, 1, 1291}, {22, 90, 1, 1292}, {24, 94, 2, 1292}, {25, 95, 2, 1292}, {27, 99, 1, 1292}, {28, 100, 2, 1292}, {29, 101, 1,

1292}, {22, 91, 1, 1293}, {24, 95, 2, 1293}, {25, 96, 2, 1293}, {27, 100, 1, 1293}, {28, 101, 2, 1293}, {29, 102, 1, 1293}, {22, 92, 1, 1294}, {24, 96, 2, 1294}, {25, 97, 2, 1294}, {27, 101, 1, 1294}, {28, 102, 2, 1294}, {29, 103, 1, 1294}, {22, 93, 1, 1295}, {24, 97, 2, 1295}, {25, 98, 2, 1295}, {27, 102, 1, 1295}, {28, 103, 2, 1295}, {29, 104, 1, 1295}, {22, 94, 1, 1296}, {24, 99, 2, 1296}, {25, 100, 2, 1296}, {27, 105, 1, 1296}, {28, 106, 2, 1296}, {29, 107, 1, 1296}, {22, 95, 1, 1297}, {24, 100, 2, 1297}, {25, 101, 2, 1297}, {27, 106, 1, 1297}, {28, 107, 2, 1297}, {29, 108, 1, 1297}, {22, 96, 1, 1298}, {24, 101, 2, 1298}, {25, 102, 2, 1298}, {27, 107, 1, 1298}, {28, 108, 2, 1298}, {29, 109, 1, 1298}, {22, 97, 1, 1299}, {24, 102, 2, 1299}, {25, 103, 2, 1299}, {27, 108, 1, 1299}, {28, 109, 2, 1299}, {29, 110, 1, 1299}, {22, 98, 1, 1300}, {24, 103, 2, 1300}, {25, 104, 2, 1300}, {27, 109, 1, 1300}, {28, 110, 2, 1300}, {29, 111, 1, 1300}, {22, 99, 1, 1301}, {24, 105, 2, 1301}, {25, 106, 2, 1301}, {27, 112, 1, 1301}, {28, 113, 2, 1301}, {29, 114, 1, 1301}, {22, 100, 1, 1302}, {24, 106, 2, 1302}, {25, 107, 2, 1302}, {27, 113, 1, 1302}, {28, 114, 2, 1302}, {29, 115, 1, 1302}, {22, 101, 1, 1303}, {24, 107, 2, 1303}, {25, 108, 2, 1303}, {27, 114, 1, 1303}, {28, 115, 2, 1303}, {29, 116, 1, 1303}, {22, 102, 1, 1304}, {24, 108, 2, 1304}, {25, 109, 2, 1304}, {27, 115, 1, 1304}, {28, 116, 2, 1304}, {29, 117, 1, 1304}, {22, 103, 1, 1305}, {24, 109, 2, 1305}, {25, 110, 2, 1305}, {27, 116, 1, 1305}, {28, 117, 2, 1305}, {29, 118, 1, 1305}, {22, 104, 1, 1306}, {24, 110, 2, 1306}, {25, 111, 2, 1306}, {27, 117, 1, 1306}, {28, 118, 2, 1306}, {29, 119, 1, 1306}, {23, 84, 1, 1307}, {26, 85, 2, 1307}, {27, 86, 2, 1307}, {30, 87, 1, 1307}, {31, 88, 2, 1307}, {32, 89, 1, 1307}, {23, 85, 1, 1308}, {26, 87, 2, 1308}, {27, 88, 2, 1308}, {30, 90, 1, 1308}, {31, 91, 2, 1308}, {32, 92, 1, 1308}, {23, 86, 1, 1309}, {26, 88, 2, 1309}, {27, 89, 2, 1309}, {30, 91, 1, 1309}, {31, 92, 2, 1309}, {32, 93, 1, 1309}, {23, 87, 1, 1310}, {26, 90, 2, 1310}, {27, 91, 2, 1310}, {30, 94, 1, 1310}, {31, 95, 2, 1310}, {32, 96, 1, 1310}, {23, 88, 1, 1311}, {26, 91, 2, 1311}, {27, 92, 2, 1311}, {30, 95, 1, 1311}, {31, 96, 2, 1311}, {32, 97, 1, 1311}, {23, 89, 1, 1312}, {26, 92, 2, 1312}, {27, 93, 2, 1312}, {30, 96, 1, 1312}, {31, 97, 2, 1312}, {32, 98, 1, 1312}, {23, 90, 1, 1313}, {26, 94, 2, 1313}, {27, 95, 2, 1313}, {30, 99, 1, 1313}, {31, 100, 2, 1313}, {32, 101, 1, 1313}, {23, 91, 1, 1314}, {26, 95, 2, 1314}, {27, 96, 2, 1314}, {30, 100, 1, 1314}, {31, 101, 2, 1314}, {32, 102, 1, 1314}, {23, 92, 1, 1315}, {26, 96, 2, 1315}, {27, 97, 2, 1315}, {30, 101, 1, 1315}, {31, 102, 2, 1315}, {32, 103, 1, 1315}, {23, 93, 1, 1316}, {26, 97, 2, 1316}, {27, 98, 2, 1316}, {30, 102, 1, 1316}, {31, 103, 2, 1316}, {32, 104, 1, 1316}, {23, 94, 1, 1317}, {26, 99, 2, 1317}, {27, 100, 2, 1317}, {30, 105, 1, 1317}, {31, 106, 2, 1317}, {32, 107, 1, 1317}, {23, 95, 1, 1318}, {26, 100, 2, 1318}, {27, 101, 2, 1318}, {30, 106, 1, 1318}, {31, 107, 2, 1318}, {32, 108, 1, 1318}, {23, 96, 1, 1319}, {26, 101, 2, 1319}, {27, 102, 2, 1319}, {30, 107, 1, 1319}, {31, 108, 2, 1319}, {32, 109, 1, 1319}, {23, 97, 1, 1320}, {26, 102, 2, 1320}, {27, 103, 2, 1320}, {30, 108, 1, 1320}, {31, 109, 2, 1320}, {32, 110, 1, 1320}, {23, 98, 1, 1321}, {26, 103, 2, 1321}, {27, 104, 2, 1321}, {30, 109, 1, 1321}, {31, 110, 2, 1321}, {32, 111, 1, 1321}, {23, 99, 1, 1322}, {26, 105, 2, 1322}, {27, 106, 2, 1322}, {30, 112, 1, 1322}, {31, 113, 2, 1322}, {32, 114, 1, 1322}, {23, 100, 1, 1323}, {26, 106, 2, 1323}, {27, 107, 2, 1323}, {30, 113, 1, 1323}, {31, 114, 2, 1323}, {32, 115, 1, 1323}, {23, 101, 1, 1324}, {26, 107, 2, 1324}, {27, 108, 2, 1324}, {30, 114, 1, 1324}, {31, 115, 2, 1324}, {32, 116, 1, 1324}, {23, 102, 1, 1325}, {26, 108, 2, 1325}, {27, 109, 2, 1325}, {30, 115, 1, 1325}, {31, 116, 2, 1325}, {32, 117, 1, 1325}, {23, 103, 1, 1326}, {26, 109, 2, 1326}, {27, 110, 2, 1326}, {30, 116, 1, 1326}, {31, 117, 2, 1326}, {32, 118, 1, 1326}, {23, 104, 1, 1327}, {26, 110, 2, 1327}, {27, 111, 2, 1327}, {30, 117, 1, 1327}, {31, 118, 2, 1327}, {32, 119, 1, 1327}, {24, 84, 1, 1328}, {27, 85, 2, 1328}, {28, 86, 2, 1328}, {31, 87, 1, 1328}, {32, 88, 2, 1328}, {33, 89, 1, 1328}, {24, 85, 1, 1329}, {27, 87, 2, 1329}, {28, 88, 2, 1329}, {31, 90, 1, 1329}, {32, 91, 2, 1329}, {33, 92, 1, 1329}, {24, 86, 1, 1330}, {27, 88, 2, 1330}, {28, 89, 2, 1330}, {31, 91, 1, 1330}, {32, 92, 2, 1330}, {33, 93, 1, 1330}, {24, 87, 1, 1331}, {27, 90, 2, 1331}, {28, 91, 2, 1331}, {31, 94, 1, 1331}, {32, 95, 2, 1331}, {33, 96, 1, 1331}, {24, 88, 1, 1332}, {27, 91, 2, 1332}, {28, 92, 2, 1332}, {31, 95, 1, 1332}, {32, 96, 2, 1332}, {33, 97, 1, 1332}, {24, 89, 1, 1333}, {27, 92, 2, 1333}, {28, 93, 2, 1333}, {31, 96, 1, 1333}, {32, 97, 2, 1333}, {33, 98, 1, 1333}, {24, 90, 1, 1334}, {27, 94, 2, 1334}, {28, 95, 2, 1334}, {31, 99, 1, 1334}, {32, 100, 2, 1334}, {33, 101, 1, 1334}, {24, 91, 1, 1335}, {27, 95, 2, 1335}, {28, 96, 2, 1335}, {31, 100, 1, 1335}, {32, 101, 2, 1335}, {33, 102, 1, 1335}, {24, 92, 1, 1336}, {27, 96, 2, 1336}, {28, 97, 2, 1336}, {31, 101, 1, 1336}, {32, 102, 2, 1336}, {33, 103, 1, 1336}, {24, 93, 1, 1337}, {27, 97, 2, 1337}, {28, 98, 2, 1337}, {31, 102, 1, 1337}, {32, 103, 2, 1337}, {33, 104, 1, 1337}, {24, 94, 1, 1338}, {27, 99, 2, 1338}, {28, 100, 2, 1338}, {31, 105, 1, 1338}, {32, 106, 2, 1338}, {33, 107, 1, 1338}, {24, 95, 1, 1339}, {27, 100, 2, 1339}, {28, 101, 2, 1339}, {31, 106, 1, 1339}, {32, 107, 2, 1339}, {33, 108, 1, 1339}, {24, 96, 1, 1340}, {27, 101, 2, 1340}, {28, 102, 2, 1340}, {31, 107, 1, 1340}, {32, 108, 2, 1340}, {33, 109, 1, 1340}, {24, 97, 1, 1341}, {27, 102, 2, 1341}, {28, 103, 2, 1341}, {31, 108, 1, 1341}, {32, 109, 2, 1341}, {33, 110, 1, 1341}, {24, 98, 1, 1342}, {27, 103, 2, 1342}, {28, 104, 2, 1342}, {31, 109, 1, 1342}, {32, 110, 2, 1342}, {33, 111, 1, 1342}, {24, 99, 1, 1343}, {27, 105, 2, 1343}, {28, 106, 2, 1343}, {31, 112, 1, 1343}, {32, 113, 2, 1343}, {33, 114, 1, 1343}, {24, 100, 1, 1344}, {27, 106, 2, 1344}, {28, 107, 2, 1344}, {31, 113, 1, 1344}, {32, 114, 2, 1344}, {33, 115, 1, 1344},

{24, 101, 1, 1345}, {27, 107, 2, 1345}, {28, 108, 2, 1345}, {31, 114, 1, 1345}, {32, 115, 2, 1345}, {33, 116, 1, 1345}, {24, 102, 1, 1346}, {27, 108, 2, 1346}, {28, 109, 2, 1346}, {31, 115, 1, 1346}, {32, 116, 2, 1346}, {33, 117, 1, 1346}, {24, 103, 1, 1347}, {27, 109, 2, 1347}, {28, 110, 2, 1347}, {31, 116, 1, 1347}, {32, 117, 2, 1347}, {33, 118, 1, 1347}, {24, 104, 1, 1348}, {27, 110, 2, 1348}, {28, 111, 2, 1348}, {31, 117, 1, 1348}, {32, 118, 2, 1348}, {33, 119, 1, 1348}, {25, 84, 1, 1349}, {28, 85, 2, 1349}, {29, 86, 2, 1349}, {32, 87, 1, 1349}, {33, 88, 2, 1349}, {34, 89, 1, 1349}, {25, 85, 1, 1350}, {28, 87, 2, 1350}, {29, 88, 2, 1350}, {32, 90, 1, 1350}, {33, 91, 2, 1350}, {34, 92, 1, 1350}, {25, 86, 1, 1351}, {28, 88, 2, 1351}, {29, 89, 2, 1351}, {32, 91, 1, 1351}, {33, 92, 2, 1351}, {34, 93, 1, 1351}, {25, 87, 1, 1352}, {28, 90, 2, 1352}, {29, 91, 2, 1352}, {32, 94, 1, 1352}, {33, 95, 2, 1352}, {34, 96, 1, 1352}, {25, 88, 1, 1353}, {28, 91, 2, 1353}, {29, 92, 2, 1353}, {32, 95, 1, 1353}, {33, 96, 2, 1353}, {34, 97, 1, 1353}, {25, 89, 1, 1354}, {28, 92, 2, 1354}, {29, 93, 2, 1354}, {32, 96, 1, 1354}, {33, 97, 2, 1354}, {34, 98, 1, 1354}, {25, 90, 1, 1355}, {28, 94, 2, 1355}, {29, 95, 2, 1355}, {32, 99, 1, 1355}, {33, 100, 2, 1355}, {34, 101, 1, 1355}, {25, 91, 1, 1356}, {28, 95, 2, 1356}, {29, 96, 2, 1356}, {32, 100, 1, 1356}, {33, 101, 2, 1356}, {34, 102, 1, 1356}, {25, 92, 1, 1357}, {28, 96, 2, 1357}, {29, 97, 2, 1357}, {32, 101, 1, 1357}, {33, 102, 2, 1357}, {34, 103, 1, 1357}, {25, 93, 1, 1358}, {28, 97, 2, 1358}, {29, 98, 2, 1358}, {32, 102, 1, 1358}, {33, 103, 2, 1358}, {34, 104, 1, 1358}, {25, 94, 1, 1359}, {28, 99, 2, 1359}, {29, 100, 2, 1359}, {32, 105, 1, 1359}, {33, 106, 2, 1359}, {34, 107, 1, 1359}, {25, 95, 1, 1360}, {28, 100, 2, 1360}, {29, 101, 2, 1360}, {32, 106, 1, 1360}, {33, 107, 2, 1360}, {34, 108, 1, 1360}, {25, 96, 1, 1361}, {28, 101, 2, 1361}, {29, 102, 2, 1361}, {32, 107, 1, 1361}, {33, 108, 2, 1361}, {34, 109, 1, 1361}, {25, 97, 1, 1362}, {28, 102, 2, 1362}, {29, 103, 2, 1362}, {32, 108, 1, 1362}, {33, 109, 2, 1362}, {34, 110, 1, 1362}, {25, 98, 1, 1363}, {28, 103, 2, 1363}, {29, 104, 2, 1363}, {32, 109, 1, 1363}, {33, 110, 2, 1363}, {34, 111, 1, 1363}, {25, 99, 1, 1364}, {28, 105, 2, 1364}, {29, 106, 2, 1364}, {32, 112, 1, 1364}, {33, 113, 2, 1364}, {34, 114, 1, 1364}, {25, 100, 1, 1365}, {28, 106, 2, 1365}, {29, 107, 2, 1365}, {32, 113, 1, 1365}, {33, 114, 2, 1365}, {34, 115, 1, 1365}, {25, 101, 1, 1366}, {28, 107, 2, 1366}, {29, 108, 2, 1366}, {32, 114, 1, 1366}, {33, 115, 2, 1366}, {34, 116, 1, 1366}, {25, 102, 1, 1367}, {28, 108, 2, 1367}, {29, 109, 2, 1367}, {32, 115, 1, 1367}, {33, 116, 2, 1367}, {34, 117, 1, 1367}, {25, 103, 1, 1368}, {28, 109, 2, 1368}, {29, 110, 2, 1368}, {32, 116, 1, 1368}, {33, 117, 2, 1368}, {34, 118, 1, 1368}, {25, 104, 1, 1369}, {28, 110, 2, 1369}, {29, 111, 2, 1369}, {32, 117, 1, 1369}, {33, 118, 2, 1369}, {34, 119, 1, 1369}, {20, 20, 1, 1370}, {21, 21, 3, 1370}, {22, 22, 3, 1370}, {23, 23, 3, 1370}, {24, 24, 6, 1370}, {25, 25, 3, 1370}, {26, 26, 1, 1370}, {27, 27, 3, 1370}, {28, 28, 3, 1370}, {29, 29, 1, 1370}, {20, 21, 1, 1371}, {21, 23, 3, 1371}, {22, 24, 3, 1371}, {23, 26, 3, 1371}, {24, 27, 6, 1371}, {25, 28, 3, 1371}, {26, 30, 1, 1371}, {27, 31, 3, 1371}, {28, 32, 3, 1371}, {29, 33, 1, 1371}, {20, 22, 1, 1372}, {21, 24, 3, 1372}, {22, 25, 3, 1372}, {23, 27, 3, 1372}, {24, 28, 6, 1372}, {25, 29, 3, 1372}, {26, 31, 1, 1372}, {27, 32, 3, 1372}, {28, 33, 3, 1372}, {29, 34, 1, 1372}, {21, 21, 1, 1373}, {23, 23, 3, 1373}, {24, 24, 3, 1373}, {26, 26, 3, 1373}, {27, 27, 6, 1373}, {28, 28, 3, 1373}, {30, 30, 1, 1373}, {31, 31, 3, 1373}, {32, 32, 3, 1373}, {33, 33, 1, 1373}, {21, 22, 1, 1374}, {23, 24, 3, 1374}, {24, 25, 3, 1374}, {26, 27, 3, 1374}, {27, 28, 6, 1374}, {28, 29, 3, 1374}, {30, 31, 1, 1374}, {31, 32, 3, 1374}, {32, 33, 3, 1374}, {33, 34, 1, 1374}, {22, 22, 1, 1375}, {24, 24, 3, 1375}, {25, 25, 3, 1375}, {27, 27, 3, 1375}, {28, 28, 6, 1375}, {29, 29, 3, 1375}, {31, 31, 1, 1375}, {32, 32, 3, 1375}, {33, 33, 3, 1375}, {34, 34, 1, 1375}, {20, 35, 1, 1376}, {21, 36, 3, 1376}, {22, 37, 3, 1376}, {23, 38, 3, 1376}, {24, 39, 6, 1376}, {25, 40, 3, 1376}, {26, 41, 1, 1376}, {27, 42, 3, 1376}, {28, 43, 3, 1376}, {29, 44, 1, 1376}, {20, 36, 1, 1377}, {21, 38, 3, 1377}, {22, 39, 3, 1377}, {23, 41, 3, 1377}, {24, 42, 6, 1377}, {25, 43, 3, 1377}, {26, 45, 1, 1377}, {27, 46, 3, 1377}, {28, 47, 3, 1377}, {29, 48, 1, 1377}, {20, 37, 1, 1378}, {21, 39, 3, 1378}, {22, 40, 3, 1378}, {23, 42, 3, 1378}, {24, 43, 6, 1378}, {25, 44, 3, 1378}, {26, 46, 1, 1378}, {27, 47, 3, 1378}, {28, 48, 3, 1378}, {29, 49, 1, 1378}, {20, 38, 1, 1379}, {21, 41, 3, 1379}, {22, 42, 3, 1379}, {23, 45, 3, 1379}, {24, 46, 6, 1379}, {25, 47, 3, 1379}, {26, 50, 1, 1379}, {27, 51, 3, 1379}, {28, 52, 3, 1379}, {29, 53, 1, 1379}, {20, 39, 1, 1380}, {21, 42, 3, 1380}, {22, 43, 3, 1380}, {23, 46, 3, 1380}, {24, 47, 6, 1380}, {25, 48, 3, 1380}, {26, 51, 1, 1380}, {27, 52, 3, 1380}, {28, 53, 3, 1380}, {29, 54, 1, 1380}, {20, 40, 1, 1381}, {21, 43, 3, 1381}, {22, 44, 3, 1381}, {23, 47, 3, 1381}, {24, 48, 6, 1381}, {25, 49, 3, 1381}, {26, 52, 1, 1381}, {27, 53, 3, 1381}, {28, 54, 3, 1381}, {29, 55, 1, 1381}, {21, 35, 1, 1382}, {23, 36, 3, 1382}, {24, 37, 3, 1382}, {26, 38, 3, 1382}, {27, 39, 6, 1382}, {28, 40, 3, 1382}, {30, 41, 1, 1382}, {31, 42, 3, 1382}, {32, 43, 3, 1382}, {33, 44, 1, 1382}, {21, 36, 1, 1383}, {23, 38, 3, 1383}, {24, 39, 3, 1383}, {26, 41, 3, 1383}, {27, 42, 6, 1383}, {28, 43, 3, 1383}, {30, 45, 1, 1383}, {31, 46, 3, 1383}, {32, 47, 3, 1383}, {33, 48, 1, 1383}, {21, 37, 1, 1384}, {23, 39, 3, 1384}, {24, 40, 3, 1384}, {26, 42, 3, 1384}, {27, 43, 6, 1384}, {28, 44, 3, 1384}, {30, 46, 1, 1384}, {31, 47, 3, 1384}, {32, 48, 3, 1384}, {33, 49, 1, 1384}, {21, 38, 1, 1385}, {23, 41, 3, 1385}, {24, 42, 3, 1385}, {26, 45, 3, 1385}, {27, 46, 6, 1385}, {28, 47, 3, 1385}, {30, 50, 1, 1385}, {31, 51, 3, 1385}, {32, 52, 3, 1385}, {33, 53, 1, 1385}, {21, 39, 1, 1386}, {23, 42, 3, 1386}, {24, 43, 3, 1386}, {26, 46, 3, 1386}, {27, 47, 6, 1386}, {28, 48, 3, 1386}, {30, 51, 1, 1386}, {31, 52, 3,

1386}, {32, 53, 3, 1386}, {33, 54, 1, 1386}, {21, 40, 1, 1387}, {23, 43, 3, 1387}, {24, 44, 3, 1387}, {26, 47, 3, 1387}, {27, 48, 6, 1387}, {28, 49, 3, 1387}, {30, 52, 1, 1387}, {31, 53, 3, 1387}, {32, 54, 3, 1387}, {33, 55, 1, 1387}, {22, 35, 1, 1388}, {24, 36, 3, 1388}, {25, 37, 3, 1388}, {27, 38, 3, 1388}, {28, 39, 6, 1388}, {29, 40, 3, 1388}, {31, 41, 1, 1388}, {32, 42, 3, 1388}, {33, 43, 3, 1388}, {34, 44, 1, 1388}, {22, 36, 1, 1389}, {24, 38, 3, 1389}, {25, 39, 3, 1389}, {27, 41, 3, 1389}, {28, 42, 6, 1389}, {29, 43, 3, 1389}, {31, 45, 1, 1389}, {32, 46, 3, 1389}, {33, 47, 3, 1389}, {34, 48, 1, 1389}, {22, 37, 1, 1390}, {24, 39, 3, 1390}, {25, 40, 3, 1390}, {27, 42, 3, 1390}, {28, 43, 6, 1390}, {29, 44, 3, 1390}, {31, 46, 1, 1390}, {32, 47, 3, 1390}, {33, 48, 3, 1390}, {34, 49, 1, 1390}, {22, 38, 1, 1391}, {24, 41, 3, 1391}, {25, 42, 3, 1391}, {27, 45, 3, 1391}, {28, 46, 6, 1391}, {29, 47, 3, 1391}, {31, 50, 1, 1391}, {32, 51, 3, 1391}, {33, 52, 3, 1391}, {34, 53, 1, 1391}, {22, 39, 1, 1392}, {24, 42, 3, 1392}, {25, 43, 3, 1392}, {27, 46, 3, 1392}, {28, 47, 6, 1392}, {29, 48, 3, 1392}, {31, 51, 1, 1392}, {32, 52, 3, 1392}, {33, 53, 3, 1392}, {34, 54, 1, 1392}, {22, 40, 1, 1393}, {24, 43, 3, 1393}, {25, 44, 3, 1393}, {27, 47, 3, 1393}, {28, 48, 6, 1393}, {29, 49, 3, 1393}, {31, 52, 1, 1393}, {32, 53, 3, 1393}, {33, 54, 3, 1393}, {34, 55, 1, 1393}, {20, 56, 1, 1394}, {21, 57, 3, 1394}, {22, 58, 3, 1394}, {23, 59, 3, 1394}, {24, 60, 6, 1394}, {25, 61, 3, 1394}, {26, 62, 1, 1394}, {27, 63, 3, 1394}, {28, 64, 3, 1394}, {29, 65, 1, 1394}, {20, 57, 1, 1395}, {21, 59, 3, 1395}, {22, 60, 3, 1395}, {23, 62, 3, 1395}, {24, 63, 6, 1395}, {25, 64, 3, 1395}, {26, 66, 1, 1395}, {27, 67, 3, 1395}, {28, 68, 3, 1395}, {29, 69, 1, 1395}, {20, 58, 1, 1396}, {21, 60, 3, 1396}, {22, 61, 3, 1396}, {23, 63, 3, 1396}, {24, 64, 6, 1396}, {25, 65, 3, 1396}, {26, 67, 1, 1396}, {27, 68, 3, 1396}, {28, 69, 3, 1396}, {29, 70, 1, 1396}, {20, 59, 1, 1397}, {21, 62, 3, 1397}, {22, 63, 3, 1397}, {23, 66, 3, 1397}, {24, 67, 6, 1397}, {25, 68, 3, 1397}, {26, 71, 1, 1397}, {27, 72, 3, 1397}, {28, 73, 3, 1397}, {29, 74, 1, 1397}, {20, 60, 1, 1398}, {21, 63, 3, 1398}, {22, 64, 3, 1398}, {23, 67, 3, 1398}, {24, 68, 6, 1398}, {25, 69, 3, 1398}, {26, 72, 1, 1398}, {27, 73, 3, 1398}, {28, 74, 3, 1398}, {29, 75, 1, 1398}, {20, 61, 1, 1399}, {21, 64, 3, 1399}, {22, 65, 3, 1399}, {23, 68, 3, 1399}, {24, 69, 6, 1399}, {25, 70, 3, 1399}, {26, 73, 1, 1399}, {27, 74, 3, 1399}, {28, 75, 3, 1399}, {29, 76, 1, 1399}, {20, 62, 1, 1400}, {21, 66, 3, 1400}, {22, 67, 3, 1400}, {23, 71, 3, 1400}, {24, 72, 6, 1400}, {25, 73, 3, 1400}, {26, 77, 1, 1400}, {27, 78, 3, 1400}, {28, 79, 3, 1400}, {29, 80, 1, 1400}, {20, 63, 1, 1401}, {21, 67, 3, 1401}, {22, 68, 3, 1401}, {23, 72, 3, 1401}, {24, 73, 6, 1401}, {25, 74, 3, 1401}, {26, 78, 1, 1401}, {27, 79, 3, 1401}, {28, 80, 3, 1401}, {29, 81, 1, 1401}, {20, 64, 1, 1402}, {21, 68, 3, 1402}, {22, 69, 3, 1402}, {23, 73, 3, 1402}, {24, 74, 6, 1402}, {25, 75, 3, 1402}, {26, 79, 1, 1402}, {27, 80, 3, 1402}, {28, 81, 3, 1402}, {29, 82, 1, 1402}, {20, 65, 1, 1403}, {21, 69, 3, 1403}, {22, 70, 3, 1403}, {23, 74, 3, 1403}, {24, 75, 6, 1403}, {25, 76, 3, 1403}, {26, 80, 1, 1403}, {27, 81, 3, 1403}, {28, 82, 3, 1403}, {29, 83, 1, 1403}, {21, 56, 1, 1404}, {23, 57, 3, 1404}, {24, 58, 3, 1404}, {26, 59, 3, 1404}, {27, 60, 6, 1404}, {28, 61, 3, 1404}, {30, 62, 1, 1404}, {31, 63, 3, 1404}, {32, 64, 3, 1404}, {33, 65, 1, 1404}, {21, 57, 1, 1405}, {23, 59, 3, 1405}, {24, 60, 3, 1405}, {26, 62, 3, 1405}, {27, 63, 6, 1405}, {28, 64, 3, 1405}, {30, 66, 1, 1405}, {31, 67, 3, 1405}, {32, 68, 3, 1405}, {33, 69, 1, 1405}, {21, 58, 1, 1406}, {23, 60, 3, 1406}, {24, 61, 3, 1406}, {26, 63, 3, 1406}, {27, 64, 6, 1406}, {28, 65, 3, 1406}, {30, 67, 1, 1406}, {31, 68, 3, 1406}, {32, 69, 3, 1406}, {33, 70, 1, 1406}, {21, 59, 1, 1407}, {23, 62, 3, 1407}, {24, 63, 3, 1407}, {26, 66, 3, 1407}, {27, 67, 6, 1407}, {28, 68, 3, 1407}, {30, 71, 1, 1407}, {31, 72, 3, 1407}, {32, 73, 3, 1407}, {33, 74, 1, 1407}, {21, 60, 1, 1408}, {23, 63, 3, 1408}, {24, 64, 3, 1408}, {26, 67, 3, 1408}, {27, 68, 6, 1408}, {28, 69, 3, 1408}, {30, 72, 1, 1408}, {31, 73, 3, 1408}, {32, 74, 3, 1408}, {33, 75, 1, 1408}, {21, 61, 1, 1409}, {23, 64, 3, 1409}, {24, 65, 3, 1409}, {26, 68, 3, 1409}, {27, 69, 6, 1409}, {28, 70, 3, 1409}, {30, 73, 1, 1409}, {31, 74, 3, 1409}, {32, 75, 3, 1409}, {33, 76, 1, 1409}, {21, 62, 1, 1410}, {23, 66, 3, 1410}, {24, 67, 3, 1410}, {26, 71, 3, 1410}, {27, 72, 6, 1410}, {28, 73, 3, 1410}, {30, 77, 1, 1410}, {31, 78, 3, 1410}, {32, 79, 3, 1410}, {33, 80, 1, 1410}, {21, 63, 1, 1411}, {23, 67, 3, 1411}, {24, 68, 3, 1411}, {26, 72, 3, 1411}, {27, 73, 6, 1411}, {28, 74, 3, 1411}, {30, 78, 1, 1411}, {31, 79, 3, 1411}, {32, 80, 3, 1411}, {33, 81, 1, 1411}, {21, 64, 1, 1412}, {23, 68, 3, 1412}, {24, 69, 3, 1412}, {26, 73, 3, 1412}, {27, 74, 6, 1412}, {28, 75, 3, 1412}, {30, 79, 1, 1412}, {31, 80, 3, 1412}, {32, 81, 3, 1412}, {33, 82, 1, 1412}, {21, 65, 1, 1413}, {23, 69, 3, 1413}, {24, 70, 3, 1413}, {26, 74, 3, 1413}, {27, 75, 6, 1413}, {28, 76, 3, 1413}, {30, 80, 1, 1413}, {31, 81, 3, 1413}, {32, 82, 3, 1413}, {33, 83, 1, 1413}, {22, 56, 1, 1414}, {24, 57, 3, 1414}, {25, 58, 3, 1414}, {27, 59, 3, 1414}, {28, 60, 6, 1414}, {29, 61, 3, 1414}, {31, 62, 1, 1414}, {32, 63, 3, 1414}, {33, 64, 3, 1414}, {34, 65, 1, 1414}, {22, 57, 1, 1415}, {24, 59, 3, 1415}, {25, 60, 3, 1415}, {27, 62, 3, 1415}, {28, 63, 6, 1415}, {29, 64, 3, 1415}, {31, 66, 1, 1415}, {32, 67, 3, 1415}, {33, 68, 3, 1415}, {34, 69, 1, 1415}, {22, 58, 1, 1416}, {24, 60, 3, 1416}, {25, 61, 3, 1416}, {27, 63, 3, 1416}, {28, 64, 6, 1416}, {29, 65, 3, 1416}, {31, 67, 1, 1416}, {32, 68, 3, 1416}, {33, 69, 3, 1416}, {34, 70, 1, 1416}, {22, 59, 1, 1417}, {24, 62, 3, 1417}, {25, 63, 3, 1417}, {27, 66, 3, 1417}, {28, 67, 6, 1417}, {29, 68, 3, 1417}, {31, 71, 1, 1417}, {32, 72, 3, 1417}, {33, 73, 3, 1417}, {34, 74, 1, 1417}, {22, 60, 1, 1418}, {24, 63, 3, 1418}, {25, 64, 3, 1418}, {27, 67, 3, 1418}, {28, 68, 6, 1418}, {29, 69, 3, 1418}, {31, 72, 1, 1418}, {32, 73, 3, 1418}, {33, 74, 3, 1418}, {34, 75, 1,

1418}, {22, 61, 1, 1419}, {24, 64, 3, 1419}, {25, 65, 3, 1419}, {27, 68, 3, 1419}, {28,
69, 6, 1419}, {29, 70, 3, 1419}, {31, 73, 1, 1419}, {32, 74, 3, 1419}, {33, 75, 3, 1419},
{34, 76, 1, 1419}, {22, 62, 1, 1420}, {24, 66, 3, 1420}, {25, 67, 3, 1420}, {27, 71, 3,
1420}, {28, 72, 6, 1420}, {29, 73, 3, 1420}, {31, 77, 1, 1420}, {32, 78, 3, 1420}, {33,
79, 3, 1420}, {34, 80, 1, 1420}, {22, 63, 1, 1421}, {24, 67, 3, 1421}, {25, 68, 3, 1421},
{27, 72, 3, 1421}, {28, 73, 6, 1421}, {29, 74, 3, 1421}, {31, 78, 1, 1421}, {32, 79, 3,
1421}, {33, 80, 3, 1421}, {34, 81, 1, 1421}, {22, 64, 1, 1422}, {24, 68, 3, 1422}, {25,
69, 3, 1422}, {27, 73, 3, 1422}, {28, 74, 6, 1422}, {29, 75, 3, 1422}, {31, 79, 1, 1422},
{32, 80, 3, 1422}, {33, 81, 3, 1422}, {34, 82, 1, 1422}, {22, 65, 1, 1423}, {24, 69, 3,
1423}, {25, 70, 3, 1423}, {27, 74, 3, 1423}, {28, 75, 6, 1423}, {29, 76, 3, 1423}, {31,
80, 1, 1423}, {32, 81, 3, 1423}, {33, 82, 3, 1423}, {34, 83, 1, 1423}, {20, 84, 1, 1424},
{21, 85, 3, 1424}, {22, 86, 3, 1424}, {23, 87, 3, 1424}, {24, 88, 6, 1424}, {25, 89, 3,
1424}, {26, 90, 1, 1424}, {27, 91, 3, 1424}, {28, 92, 3, 1424}, {29, 93, 1, 1424}, {20,
85, 1, 1425}, {21, 87, 3, 1425}, {22, 88, 3, 1425}, {23, 90, 3, 1425}, {24, 91, 6, 1425},
{25, 92, 3, 1425}, {26, 94, 1, 1425}, {27, 95, 3, 1425}, {28, 96, 3, 1425}, {29, 97, 1,
1425}, {20, 86, 1, 1426}, {21, 88, 3, 1426}, {22, 89, 3, 1426}, {23, 91, 3, 1426}, {24,
92, 6, 1426}, {25, 93, 3, 1426}, {26, 95, 1, 1426}, {27, 96, 3, 1426}, {28, 97, 3, 1426},
{29, 98, 1, 1426}, {20, 87, 1, 1427}, {21, 90, 3, 1427}, {22, 91, 3, 1427}, {23, 94, 3,
1427}, {24, 95, 6, 1427}, {25, 96, 3, 1427}, {26, 99, 1, 1427}, {27, 100, 3, 1427}, {28,
101, 3, 1427}, {29, 102, 1, 1427}, {20, 88, 1, 1428}, {21, 91, 3, 1428}, {22, 92, 3,
1428}, {23, 95, 3, 1428}, {24, 96, 6, 1428}, {25, 97, 3, 1428}, {26, 100, 1, 1428}, {27,
101, 3, 1428}, {28, 102, 3, 1428}, {29, 103, 1, 1428}, {20, 89, 1, 1429}, {21, 92, 3,
1429}, {22, 93, 3, 1429}, {23, 96, 3, 1429}, {24, 97, 6, 1429}, {25, 98, 3, 1429}, {26,
101, 1, 1429}, {27, 102, 3, 1429}, {28, 103, 3, 1429}, {29, 104, 1, 1429}, {20, 90, 1,
1430}, {21, 94, 3, 1430}, {22, 95, 3, 1430}, {23, 99, 3, 1430}, {24, 100, 6, 1430}, {25,
101, 3, 1430}, {26, 105, 1, 1430}, {27, 106, 3, 1430}, {28, 107, 3, 1430}, {29, 108, 1,
1430}, {20, 91, 1, 1431}, {21, 95, 3, 1431}, {22, 96, 3, 1431}, {23, 100, 3, 1431}, {24,
101, 6, 1431}, {25, 102, 3, 1431}, {26, 106, 1, 1431}, {27, 107, 3, 1431}, {28, 108, 3,
1431}, {29, 109, 1, 1431}, {20, 92, 1, 1432}, {21, 96, 3, 1432}, {22, 97, 3, 1432}, {23,
101, 3, 1432}, {24, 102, 6, 1432}, {25, 103, 3, 1432}, {26, 107, 1, 1432}, {27, 108, 3,
1432}, {28, 109, 3, 1432}, {29, 110, 1, 1432}, {20, 93, 1, 1433}, {21, 97, 3, 1433}, {22,
98, 3, 1433}, {23, 102, 3, 1433}, {24, 103, 6, 1433}, {25, 104, 3, 1433}, {26, 108, 1,
1433}, {27, 109, 3, 1433}, {28, 110, 3, 1433}, {29, 111, 1, 1433}, {20, 94, 1, 1434}, {21,
99, 3, 1434}, {22, 100, 3, 1434}, {23, 105, 3, 1434}, {24, 106, 6, 1434}, {25, 107, 3,
1434}, {26, 112, 1, 1434}, {27, 113, 3, 1434}, {28, 114, 3, 1434}, {29, 115, 1, 1434},
{20, 95, 1, 1435}, {21, 100, 3, 1435}, {22, 101, 3, 1435}, {23, 106, 3, 1435}, {24, 107,
6, 1435}, {25, 108, 3, 1435}, {26, 113, 1, 1435}, {27, 114, 3, 1435}, {28, 115, 3, 1435},
{29, 116, 1, 1435}, {20, 96, 1, 1436}, {21, 101, 3, 1436}, {22, 102, 3, 1436}, {23, 107,
3, 1436}, {24, 108, 6, 1436}, {25, 109, 3, 1436}, {26, 114, 1, 1436}, {27, 115, 3, 1436},
{28, 116, 3, 1436}, {29, 117, 1, 1436}, {20, 97, 1, 1437}, {21, 102, 3, 1437}, {22, 103,
3, 1437}, {23, 108, 3, 1437}, {24, 109, 6, 1437}, {25, 110, 3, 1437}, {26, 115, 1, 1437},
{27, 116, 3, 1437}, {28, 117, 3, 1437}, {29, 118, 1, 1437}, {20, 98, 1, 1438}, {21, 103,
3, 1438}, {22, 104, 3, 1438}, {23, 109, 3, 1438}, {24, 110, 6, 1438}, {25, 111, 3, 1438},
{26, 116, 1, 1438}, {27, 117, 3, 1438}, {28, 118, 3, 1438}, {29, 119, 1, 1438}, {21, 84,
1, 1439}, {23, 85, 3, 1439}, {24, 86, 3, 1439}, {26, 87, 3, 1439}, {27, 88, 6, 1439}, {28,
89, 3, 1439}, {30, 90, 1, 1439}, {31, 91, 3, 1439}, {32, 92, 3, 1439}, {33, 93, 1, 1439},
{21, 85, 1, 1440}, {23, 87, 3, 1440}, {24, 88, 3, 1440}, {26, 90, 3, 1440}, {27, 91, 6,
1440}, {28, 92, 3, 1440}, {30, 94, 1, 1440}, {31, 95, 3, 1440}, {32, 96, 3, 1440}, {33,
97, 1, 1440}, {21, 86, 1, 1441}, {23, 88, 3, 1441}, {24, 89, 3, 1441}, {26, 91, 3, 1441},
{27, 92, 6, 1441}, {28, 93, 3, 1441}, {30, 95, 1, 1441}, {31, 96, 3, 1441}, {32, 97, 3,
1441}, {33, 98, 1, 1441}, {21, 87, 1, 1442}, {23, 90, 3, 1442}, {24, 91, 3, 1442}, {26,
94, 3, 1442}, {27, 95, 6, 1442}, {28, 96, 3, 1442}, {30, 99, 1, 1442}, {31, 100, 3, 1442},
{32, 101, 3, 1442}, {33, 102, 1, 1442}, {21, 88, 1, 1443}, {23, 91, 3, 1443}, {24, 92, 3,
1443}, {26, 95, 3, 1443}, {27, 96, 6, 1443}, {28, 97, 3, 1443}, {30, 100, 1, 1443}, {31,
101, 3, 1443}, {32, 102, 3, 1443}, {33, 103, 1, 1443}, {21, 89, 1, 1444}, {23, 92, 3,
1444}, {24, 93, 3, 1444}, {26, 96, 3, 1444}, {27, 97, 6, 1444}, {28, 98, 3, 1444}, {30,
101, 1, 1444}, {31, 102, 3, 1444}, {32, 103, 3, 1444}, {33, 104, 1, 1444}, {21, 90, 1,
1445}, {23, 94, 3, 1445}, {24, 95, 3, 1445}, {26, 99, 3, 1445}, {27, 100, 6, 1445}, {28,
101, 3, 1445}, {30, 105, 1, 1445}, {31, 106, 3, 1445}, {32, 107, 3, 1445}, {33, 108, 1,
1445}, {21, 91, 1, 1446}, {23, 95, 3, 1446}, {24, 96, 3, 1446}, {26, 100, 3, 1446}, {27,
101, 6, 1446}, {28, 102, 3, 1446}, {30, 106, 1, 1446}, {31, 107, 3, 1446}, {32, 108, 3,
1446}, {33, 109, 1, 1446}, {21, 92, 1, 1447}, {23, 96, 3, 1447}, {24, 97, 3, 1447}, {26,
101, 3, 1447}, {27, 102, 6, 1447}, {28, 103, 3, 1447}, {30, 107, 1, 1447}, {31, 108, 3,
1447}, {32, 109, 3, 1447}, {33, 110, 1, 1447}, {21, 93, 1, 1448}, {23, 97, 3, 1448}, {24,
98, 3, 1448}, {26, 102, 3, 1448}, {27, 103, 6, 1448}, {28, 104, 3, 1448}, {30, 108, 1,
1448}, {31, 109, 3, 1448}, {32, 110, 3, 1448}, {33, 111, 1, 1448}, {21, 94, 1, 1449}, {23,
99, 3, 1449}, {24, 100, 3, 1449}, {26, 105, 3, 1449}, {27, 106, 6, 1449}, {28, 107, 3,
1449}, {30, 112, 1, 1449}, {31, 113, 3, 1449}, {32, 114, 3, 1449}, {33, 115, 1, 1449},
{21, 95, 1, 1450}, {23, 100, 3, 1450}, {24, 101, 3, 1450}, {26, 106, 3, 1450}, {27, 107,

6, 1450}, {28, 108, 3, 1450}, {30, 113, 1, 1450}, {31, 114, 3, 1450}, {32, 115, 3, 1450},
{33, 116, 1, 1450}, {21, 96, 1, 1451}, {23, 101, 3, 1451}, {24, 102, 3, 1451}, {26, 107,
3, 1451}, {27, 108, 6, 1451}, {28, 109, 3, 1451}, {30, 114, 1, 1451}, {31, 115, 3, 1451},
{32, 116, 3, 1451}, {33, 117, 1, 1451}, {21, 97, 1, 1452}, {23, 102, 3, 1452}, {24, 103,
3, 1452}, {26, 108, 3, 1452}, {27, 109, 6, 1452}, {28, 110, 3, 1452}, {30, 115, 1, 1452},
{31, 116, 3, 1452}, {32, 117, 3, 1452}, {33, 118, 1, 1452}, {21, 98, 1, 1453}, {23, 103,
3, 1453}, {24, 104, 3, 1453}, {26, 109, 3, 1453}, {27, 110, 6, 1453}, {28, 111, 3, 1453},
{30, 116, 1, 1453}, {31, 117, 3, 1453}, {32, 118, 3, 1453}, {33, 119, 1, 1453}, {22, 84,
1, 1454}, {24, 85, 3, 1454}, {25, 86, 3, 1454}, {27, 87, 3, 1454}, {28, 88, 6, 1454}, {29,
89, 3, 1454}, {31, 90, 1, 1454}, {32, 91, 3, 1454}, {33, 92, 3, 1454}, {34, 93, 1, 1454},
{22, 85, 1, 1455}, {24, 87, 3, 1455}, {25, 88, 3, 1455}, {27, 90, 3, 1455}, {28, 91, 6,
1455}, {29, 92, 3, 1455}, {31, 94, 1, 1455}, {32, 95, 3, 1455}, {33, 96, 3, 1455}, {34,
97, 1, 1455}, {22, 86, 1, 1456}, {24, 88, 3, 1456}, {25, 89, 3, 1456}, {27, 91, 3, 1456},
{28, 92, 6, 1456}, {29, 93, 3, 1456}, {31, 95, 1, 1456}, {32, 96, 3, 1456}, {33, 97, 3,
1456}, {34, 98, 1, 1456}, {22, 87, 1, 1457}, {24, 90, 3, 1457}, {25, 91, 3, 1457}, {27,
94, 3, 1457}, {28, 95, 6, 1457}, {29, 96, 3, 1457}, {31, 99, 1, 1457}, {32, 100, 3, 1457},
{33, 101, 3, 1457}, {34, 102, 1, 1457}, {22, 88, 1, 1458}, {24, 91, 3, 1458}, {25, 92, 3,
1458}, {27, 95, 3, 1458}, {28, 96, 6, 1458}, {29, 97, 3, 1458}, {31, 100, 1, 1458}, {32,
101, 3, 1458}, {33, 102, 3, 1458}, {34, 103, 1, 1458}, {22, 89, 1, 1459}, {24, 92, 3,
1459}, {25, 93, 3, 1459}, {27, 96, 3, 1459}, {28, 97, 6, 1459}, {29, 98, 3, 1459}, {31,
101, 1, 1459}, {32, 102, 3, 1459}, {33, 103, 3, 1459}, {34, 104, 1, 1459}, {22, 90, 1,
1460}, {24, 94, 3, 1460}, {25, 95, 3, 1460}, {27, 99, 3, 1460}, {28, 100, 6, 1460}, {29,
101, 3, 1460}, {31, 105, 1, 1460}, {32, 106, 3, 1460}, {33, 107, 3, 1460}, {34, 108, 1,
1460}, {22, 91, 1, 1461}, {24, 95, 3, 1461}, {25, 96, 3, 1461}, {27, 100, 3, 1461}, {28,
101, 6, 1461}, {29, 102, 3, 1461}, {31, 106, 1, 1461}, {32, 107, 3, 1461}, {33, 108, 3,
1461}, {34, 109, 1, 1461}, {22, 92, 1, 1462}, {24, 96, 3, 1462}, {25, 97, 3, 1462}, {27,
101, 3, 1462}, {28, 102, 6, 1462}, {29, 103, 3, 1462}, {31, 107, 1, 1462}, {32, 108, 3,
1462}, {33, 109, 3, 1462}, {34, 110, 1, 1462}, {22, 93, 1, 1463}, {24, 97, 3, 1463}, {25,
98, 3, 1463}, {27, 102, 3, 1463}, {28, 103, 6, 1463}, {29, 104, 3, 1463}, {31, 108, 1,
1463}, {32, 109, 3, 1463}, {33, 110, 3, 1463}, {34, 111, 1, 1463}, {22, 94, 1, 1464}, {24,
99, 3, 1464}, {25, 100, 3, 1464}, {27, 105, 3, 1464}, {28, 106, 6, 1464}, {29, 107, 3,
1464}, {31, 112, 1, 1464}, {32, 113, 3, 1464}, {33, 114, 3, 1464}, {34, 115, 1, 1464},
{22, 95, 1, 1465}, {24, 100, 3, 1465}, {25, 101, 3, 1465}, {27, 106, 3, 1465}, {28, 107,
6, 1465}, {29, 108, 3, 1465}, {31, 113, 1, 1465}, {32, 114, 3, 1465}, {33, 115, 3, 1465},
{34, 116, 1, 1465}, {22, 96, 1, 1466}, {24, 101, 3, 1466}, {25, 102, 3, 1466}, {27, 107,
3, 1466}, {28, 108, 6, 1466}, {29, 109, 3, 1466}, {31, 114, 1, 1466}, {32, 115, 3, 1466},
{33, 116, 3, 1466}, {34, 117, 1, 1466}, {22, 97, 1, 1467}, {24, 102, 3, 1467}, {25, 103,
3, 1467}, {27, 108, 3, 1467}, {28, 109, 6, 1467}, {29, 110, 3, 1467}, {31, 115, 1, 1467},
{32, 116, 3, 1467}, {33, 117, 3, 1467}, {34, 118, 1, 1467}, {22, 98, 1, 1468}, {24, 103,
3, 1468}, {25, 104, 3, 1468}, {27, 109, 3, 1468}, {28, 110, 6, 1468}, {29, 111, 3, 1468},
{31, 116, 1, 1468}, {32, 117, 3, 1468}, {33, 118, 3, 1468}, {34, 119, 1, 1468}, {20, 120,
1, 1469}, {21, 121, 3, 1469}, {22, 122, 3, 1469}, {23, 123, 3, 1469}, {24, 124, 6, 1469},
{25, 125, 3, 1469}, {26, 126, 1, 1469}, {27, 127, 3, 1469}, {28, 128, 3, 1469}, {29, 129,
1, 1469}, {20, 121, 1, 1470}, {21, 123, 3, 1470}, {22, 124, 3, 1470}, {23, 126, 3, 1470},
{24, 127, 6, 1470}, {25, 128, 3, 1470}, {26, 130, 1, 1470}, {27, 131, 3, 1470}, {28, 132,
3, 1470}, {29, 133, 1, 1470}, {20, 122, 1, 1471}, {21, 124, 3, 1471}, {22, 125, 3, 1471},
{23, 127, 3, 1471}, {24, 128, 6, 1471}, {25, 129, 3, 1471}, {26, 131, 1, 1471}, {27, 132,
3, 1471}, {28, 133, 3, 1471}, {29, 134, 1, 1471}, {20, 123, 1, 1472}, {21, 126, 3, 1472},
{22, 127, 3, 1472}, {23, 130, 3, 1472}, {24, 131, 6, 1472}, {25, 132, 3, 1472}, {26, 135,
1, 1472}, {27, 136, 3, 1472}, {28, 137, 3, 1472}, {29, 138, 1, 1472}, {20, 124, 1, 1473},
{21, 127, 3, 1473}, {22, 128, 3, 1473}, {23, 131, 3, 1473}, {24, 132, 6, 1473}, {25, 133,
3, 1473}, {26, 136, 1, 1473}, {27, 137, 3, 1473}, {28, 138, 3, 1473}, {29, 139, 1, 1473},
{20, 125, 1, 1474}, {21, 128, 3, 1474}, {22, 129, 3, 1474}, {23, 132, 3, 1474}, {24, 133,
6, 1474}, {25, 134, 3, 1474}, {26, 137, 1, 1474}, {27, 138, 3, 1474}, {28, 139, 3, 1474},
{29, 140, 1, 1474}, {20, 126, 1, 1475}, {21, 130, 3, 1475}, {22, 131, 3, 1475}, {23, 135,
3, 1475}, {24, 136, 6, 1475}, {25, 137, 3, 1475}, {26, 141, 1, 1475}, {27, 142, 3, 1475},
{28, 143, 3, 1475}, {29, 144, 1, 1475}, {20, 127, 1, 1476}, {21, 131, 3, 1476}, {22, 132,
3, 1476}, {23, 136, 3, 1476}, {24, 137, 6, 1476}, {25, 138, 3, 1476}, {26, 142, 1, 1476},
{27, 143, 3, 1476}, {28, 144, 3, 1476}, {29, 145, 1, 1476}, {20, 128, 1, 1477}, {21, 132,
3, 1477}, {22, 133, 3, 1477}, {23, 137, 3, 1477}, {24, 138, 6, 1477}, {25, 139, 3, 1477},
{26, 143, 1, 1477}, {27, 144, 3, 1477}, {28, 145, 3, 1477}, {29, 146, 1, 1477}, {20, 129,
1, 1478}, {21, 133, 3, 1478}, {22, 134, 3, 1478}, {23, 138, 3, 1478}, {24, 139, 6, 1478},
{25, 140, 3, 1478}, {26, 144, 1, 1478}, {27, 145, 3, 1478}, {28, 146, 3, 1478}, {29, 147,
1, 1478}, {20, 130, 1, 1479}, {21, 135, 3, 1479}, {22, 136, 3, 1479}, {23, 141, 3, 1479},
{24, 142, 6, 1479}, {25, 143, 3, 1479}, {26, 148, 1, 1479}, {27, 149, 3, 1479}, {28, 150,
3, 1479}, {29, 151, 1, 1479}, {20, 131, 1, 1480}, {21, 136, 3, 1480}, {22, 137, 3, 1480},
{23, 142, 3, 1480}, {24, 143, 6, 1480}, {25, 144, 3, 1480}, {26, 149, 1, 1480}, {27, 150,
3, 1480}, {28, 151, 3, 1480}, {29, 152, 1, 1480}, {20, 132, 1, 1481}, {21, 137, 3, 1481},
{22, 138, 3, 1481}, {23, 143, 3, 1481}, {24, 144, 6, 1481}, {25, 145, 3, 1481}, {26, 150,

1, 1481}, {27, 151, 3, 1481}, {28, 152, 3, 1481}, {29, 153, 1, 1481}, {20, 133, 1, 1482}, {21, 138, 3, 1482}, {22, 139, 3, 1482}, {23, 144, 3, 1482}, {24, 145, 6, 1482}, {25, 146, 3, 1482}, {26, 151, 1, 1482}, {27, 152, 3, 1482}, {28, 153, 3, 1482}, {29, 154, 1, 1482}, {20, 134, 1, 1483}, {21, 139, 3, 1483}, {22, 140, 3, 1483}, {23, 145, 3, 1483}, {24, 146, 6, 1483}, {25, 147, 3, 1483}, {26, 152, 1, 1483}, {27, 153, 3, 1483}, {28, 154, 3, 1483}, {29, 155, 1, 1483}, {20, 135, 1, 1484}, {21, 141, 3, 1484}, {22, 142, 3, 1484}, {23, 148, 3, 1484}, {24, 149, 6, 1484}, {25, 150, 3, 1484}, {26, 156, 1, 1484}, {27, 157, 3, 1484}, {28, 158, 3, 1484}, {29, 159, 1, 1484}, {20, 136, 1, 1485}, {21, 142, 3, 1485}, {22, 143, 3, 1485}, {23, 149, 3, 1485}, {24, 150, 6, 1485}, {25, 151, 3, 1485}, {26, 157, 1, 1485}, {27, 158, 3, 1485}, {28, 159, 3, 1485}, {29, 160, 1, 1485}, {20, 137, 1, 1486}, {21, 143, 3, 1486}, {22, 144, 3, 1486}, {23, 150, 3, 1486}, {24, 151, 6, 1486}, {25, 152, 3, 1486}, {26, 158, 1, 1486}, {27, 159, 3, 1486}, {28, 160, 3, 1486}, {29, 161, 1, 1486}, {20, 138, 1, 1487}, {21, 144, 3, 1487}, {22, 145, 3, 1487}, {23, 151, 3, 1487}, {24, 152, 6, 1487}, {25, 153, 3, 1487}, {26, 159, 1, 1487}, {27, 160, 3, 1487}, {28, 161, 3, 1487}, {29, 162, 1, 1487}, {20, 139, 1, 1488}, {21, 145, 3, 1488}, {22, 146, 3, 1488}, {23, 152, 3, 1488}, {24, 153, 6, 1488}, {25, 154, 3, 1488}, {26, 160, 1, 1488}, {27, 161, 3, 1488}, {28, 162, 3, 1488}, {29, 163, 1, 1488}, {20, 140, 1, 1489}, {21, 146, 3, 1489}, {22, 147, 3, 1489}, {23, 153, 3, 1489}, {24, 154, 6, 1489}, {25, 155, 3, 1489}, {26, 161, 1, 1489}, {27, 162, 3, 1489}, {28, 163, 3, 1489}, {29, 164, 1, 1489}, {21, 120, 1, 1490}, {23, 121, 3, 1490}, {24, 122, 3, 1490}, {26, 123, 3, 1490}, {27, 124, 6, 1490}, {28, 125, 3, 1490}, {30, 126, 1, 1490}, {31, 127, 3, 1490}, {32, 128, 3, 1490}, {33, 129, 1, 1490}, {21, 121, 1, 1491}, {23, 123, 3, 1491}, {24, 124, 3, 1491}, {26, 126, 3, 1491}, {27, 127, 6, 1491}, {28, 128, 3, 1491}, {30, 130, 1, 1491}, {31, 131, 3, 1491}, {32, 132, 3, 1491}, {33, 133, 1, 1491}, {21, 122, 1, 1492}, {23, 124, 3, 1492}, {24, 125, 3, 1492}, {26, 127, 3, 1492}, {27, 128, 6, 1492}, {28, 129, 3, 1492}, {30, 131, 1, 1492}, {31, 132, 3, 1492}, {32, 133, 3, 1492}, {33, 134, 1, 1492}, {21, 123, 1, 1493}, {23, 126, 3, 1493}, {24, 127, 3, 1493}, {26, 130, 3, 1493}, {27, 131, 6, 1493}, {28, 132, 3, 1493}, {30, 135, 1, 1493}, {31, 136, 3, 1493}, {32, 137, 3, 1493}, {33, 138, 1, 1493}, {21, 124, 1, 1494}, {23, 127, 3, 1494}, {24, 128, 3, 1494}, {26, 131, 3, 1494}, {27, 132, 6, 1494}, {28, 133, 3, 1494}, {30, 136, 1, 1494}, {31, 137, 3, 1494}, {32, 138, 3, 1494}, {33, 139, 1, 1494}, {21, 125, 1, 1495}, {23, 128, 3, 1495}, {24, 129, 3, 1495}, {26, 132, 3, 1495}, {27, 133, 6, 1495}, {28, 134, 3, 1495}, {30, 137, 1, 1495}, {31, 138, 3, 1495}, {32, 139, 3, 1495}, {33, 140, 1, 1495}, {21, 126, 1, 1496}, {23, 130, 3, 1496}, {24, 131, 3, 1496}, {26, 135, 3, 1496}, {27, 136, 6, 1496}, {28, 137, 3, 1496}, {30, 141, 1, 1496}, {31, 142, 3, 1496}, {32, 143, 3, 1496}, {33, 144, 1, 1496}, {21, 127, 1, 1497}, {23, 131, 3, 1497}, {24, 132, 3, 1497}, {26, 136, 3, 1497}, {27, 137, 6, 1497}, {28, 138, 3, 1497}, {30, 142, 1, 1497}, {31, 143, 3, 1497}, {32, 144, 3, 1497}, {33, 145, 1, 1497}, {21, 128, 1, 1498}, {23, 132, 3, 1498}, {24, 133, 3, 1498}, {26, 137, 3, 1498}, {27, 138, 6, 1498}, {28, 139, 3, 1498}, {30, 143, 1, 1498}, {31, 144, 3, 1498}, {32, 145, 3, 1498}, {33, 146, 1, 1498}, {21, 129, 1, 1499}, {23, 133, 3, 1499}, {24, 134, 3, 1499}, {26, 138, 3, 1499}, {27, 139, 6, 1499}, {28, 140, 3, 1499}, {30, 144, 1, 1499}, {31, 145, 3, 1499}, {32, 146, 3, 1499}, {33, 147, 1, 1499}, {21, 130, 1, 1500}, {23, 135, 3, 1500}, {24, 136, 3, 1500}, {26, 141, 3, 1500}, {27, 142, 6, 1500}, {28, 143, 3, 1500}, {30, 148, 1, 1500}, {31, 149, 3, 1500}, {32, 150, 3, 1500}, {33, 151, 1, 1500}, {21, 131, 1, 1501}, {23, 136, 3, 1501}, {24, 137, 3, 1501}, {26, 142, 3, 1501}, {27, 143, 6, 1501}, {28, 144, 3, 1501}, {30, 149, 1, 1501}, {31, 150, 3, 1501}, {32, 151, 3, 1501}, {33, 152, 1, 1501}, {21, 132, 1, 1502}, {23, 137, 3, 1502}, {24, 138, 3, 1502}, {26, 143, 3, 1502}, {27, 144, 6, 1502}, {28, 145, 3, 1502}, {30, 150, 1, 1502}, {31, 151, 3, 1502}, {32, 152, 3, 1502}, {33, 153, 1, 1502}, {21, 133, 1, 1503}, {23, 138, 3, 1503}, {24, 139, 3, 1503}, {26, 144, 3, 1503}, {27, 145, 6, 1503}, {28, 146, 3, 1503}, {30, 151, 1, 1503}, {31, 152, 3, 1503}, {32, 153, 3, 1503}, {33, 154, 1, 1503}, {21, 134, 1, 1504}, {23, 139, 3, 1504}, {24, 140, 3, 1504}, {26, 145, 3, 1504}, {27, 146, 6, 1504}, {28, 147, 3, 1504}, {30, 152, 1, 1504}, {31, 153, 3, 1504}, {32, 154, 3, 1504}, {33, 155, 1, 1504}, {21, 135, 1, 1505}, {23, 141, 3, 1505}, {24, 142, 3, 1505}, {26, 148, 3, 1505}, {27, 149, 6, 1505}, {28, 150, 3, 1505}, {30, 156, 1, 1505}, {31, 157, 3, 1505}, {32, 158, 3, 1505}, {33, 159, 1, 1505}, {21, 136, 1, 1506}, {23, 142, 3, 1506}, {24, 143, 3, 1506}, {26, 149, 3, 1506}, {27, 150, 6, 1506}, {28, 151, 3, 1506}, {30, 157, 1, 1506}, {31, 158, 3, 1506}, {32, 159, 3, 1506}, {33, 160, 1, 1506}, {21, 137, 1, 1507}, {23, 143, 3, 1507}, {24, 144, 3, 1507}, {26, 150, 3, 1507}, {27, 151, 6, 1507}, {28, 152, 3, 1507}, {30, 158, 1, 1507}, {31, 159, 3, 1507}, {32, 160, 3, 1507}, {33, 161, 1, 1507}, {21, 138, 1, 1508}, {23, 144, 3, 1508}, {24, 145, 3, 1508}, {26, 151, 3, 1508}, {27, 152, 6, 1508}, {28, 153, 3, 1508}, {30, 159, 1, 1508}, {31, 160, 3, 1508}, {32, 161, 3, 1508}, {33, 162, 1, 1508}, {21, 139, 1, 1509}, {23, 145, 3, 1509}, {24, 146, 3, 1509}, {26, 152, 3, 1509}, {27, 153, 6, 1509}, {28, 154, 3, 1509}, {30, 160, 1, 1509}, {31, 161, 3, 1509}, {32, 162, 3, 1509}, {33, 163, 1, 1509}, {21, 140, 1, 1510}, {23, 146, 3, 1510}, {24, 147, 3, 1510}, {26, 153, 3, 1510}, {27, 154, 6, 1510}, {28, 155, 3, 1510}, {30, 161, 1, 1510}, {31, 162, 3, 1510}, {32, 163, 3, 1510}, {33, 164, 1, 1510}, {22, 120, 1, 1511}, {24, 121, 3, 1511}, {25, 122, 3, 1511}, {27, 123, 3, 1511}, {28, 124, 6, 1511}, {29, 125, 3, 1511}, {31, 126, 1, 1511}, {32, 127, 3, 1511}, {33, 128, 3, 1511}, {34, 129, 1, 1511}, {22, 121, 1, 1512}, {24, 123, 3, 1512}, {25, 124, 3, 1512}, {27, 126, 3, 1512}, {28, 127, 6, 1512}, {29, 128, 3, 1512}, {31, 130, 1, 1512},

{32, 131, 3, 1512}, {33, 132, 3, 1512}, {34, 133, 1, 1512}, {22, 122, 1, 1513}, {24, 124, 3, 1513}, {25, 125, 3, 1513}, {27, 127, 3, 1513}, {28, 128, 6, 1513}, {29, 129, 3, 1513}, {31, 131, 1, 1513}, {32, 132, 3, 1513}, {33, 133, 3, 1513}, {34, 134, 1, 1513}, {22, 123, 1, 1514}, {24, 126, 3, 1514}, {25, 127, 3, 1514}, {27, 130, 3, 1514}, {28, 131, 6, 1514}, {29, 132, 3, 1514}, {31, 135, 1, 1514}, {32, 136, 3, 1514}, {33, 137, 3, 1514}, {34, 138, 1, 1514}, {22, 124, 1, 1515}, {24, 127, 3, 1515}, {25, 128, 3, 1515}, {27, 131, 3, 1515}, {28, 132, 6, 1515}, {29, 133, 3, 1515}, {31, 136, 1, 1515}, {32, 137, 3, 1515}, {33, 138, 3, 1515}, {34, 139, 1, 1515}, {22, 125, 1, 1516}, {24, 128, 3, 1516}, {25, 129, 3, 1516}, {27, 132, 3, 1516}, {28, 133, 6, 1516}, {29, 134, 3, 1516}, {31, 137, 1, 1516}, {32, 138, 3, 1516}, {33, 139, 3, 1516}, {34, 140, 1, 1516}, {22, 126, 1, 1517}, {24, 130, 3, 1517}, {25, 131, 3, 1517}, {27, 135, 3, 1517}, {28, 136, 6, 1517}, {29, 137, 3, 1517}, {31, 141, 1, 1517}, {32, 142, 3, 1517}, {33, 143, 3, 1517}, {34, 144, 1, 1517}, {22, 127, 1, 1518}, {24, 131, 3, 1518}, {25, 132, 3, 1518}, {27, 136, 3, 1518}, {28, 137, 6, 1518}, {29, 138, 3, 1518}, {31, 142, 1, 1518}, {32, 143, 3, 1518}, {33, 144, 3, 1518}, {34, 145, 1, 1518}, {22, 128, 1, 1519}, {24, 132, 3, 1519}, {25, 133, 3, 1519}, {27, 137, 3, 1519}, {28, 138, 6, 1519}, {29, 139, 3, 1519}, {31, 143, 1, 1519}, {32, 144, 3, 1519}, {33, 145, 3, 1519}, {34, 146, 1, 1519}, {22, 129, 1, 1520}, {24, 133, 3, 1520}, {25, 134, 3, 1520}, {27, 138, 3, 1520}, {28, 139, 6, 1520}, {29, 140, 3, 1520}, {31, 144, 1, 1520}, {32, 145, 3, 1520}, {33, 146, 3, 1520}, {34, 147, 1, 1520}, {22, 130, 1, 1521}, {24, 135, 3, 1521}, {25, 136, 3, 1521}, {27, 141, 3, 1521}, {28, 142, 6, 1521}, {29, 143, 3, 1521}, {31, 148, 1, 1521}, {32, 149, 3, 1521}, {33, 150, 3, 1521}, {34, 151, 1, 1521}, {22, 131, 1, 1522}, {24, 136, 3, 1522}, {25, 137, 3, 1522}, {27, 142, 3, 1522}, {28, 143, 6, 1522}, {29, 144, 3, 1522}, {31, 149, 1, 1522}, {32, 150, 3, 1522}, {33, 151, 3, 1522}, {34, 152, 1, 1522}, {22, 132, 1, 1523}, {24, 137, 3, 1523}, {25, 138, 3, 1523}, {27, 143, 3, 1523}, {28, 144, 6, 1523}, {29, 145, 3, 1523}, {31, 150, 1, 1523}, {32, 151, 3, 1523}, {33, 152, 3, 1523}, {34, 153, 1, 1523}, {22, 133, 1, 1524}, {24, 138, 3, 1524}, {25, 139, 3, 1524}, {27, 144, 3, 1524}, {28, 145, 6, 1524}, {29, 146, 3, 1524}, {31, 151, 1, 1524}, {32, 152, 3, 1524}, {33, 153, 3, 1524}, {34, 154, 1, 1524}, {22, 134, 1, 1525}, {24, 139, 3, 1525}, {25, 140, 3, 1525}, {27, 145, 3, 1525}, {28, 146, 6, 1525}, {29, 147, 3, 1525}, {31, 152, 1, 1525}, {32, 153, 3, 1525}, {33, 154, 3, 1525}, {34, 155, 1, 1525}, {22, 135, 1, 1526}, {24, 141, 3, 1526}, {25, 142, 3, 1526}, {27, 148, 3, 1526}, {28, 149, 6, 1526}, {29, 150, 3, 1526}, {31, 156, 1, 1526}, {32, 157, 3, 1526}, {33, 158, 3, 1526}, {34, 159, 1, 1526}, {22, 136, 1, 1527}, {24, 142, 3, 1527}, {25, 143, 3, 1527}, {27, 149, 3, 1527}, {28, 150, 6, 1527}, {29, 151, 3, 1527}, {31, 157, 1, 1527}, {32, 158, 3, 1527}, {33, 159, 3, 1527}, {34, 160, 1, 1527}, {22, 137, 1, 1528}, {24, 143, 3, 1528}, {25, 144, 3, 1528}, {27, 150, 3, 1528}, {28, 151, 6, 1528}, {29, 152, 3, 1528}, {31, 158, 1, 1528}, {32, 159, 3, 1528}, {33, 160, 3, 1528}, {34, 161, 1, 1528}, {22, 138, 1, 1529}, {24, 144, 3, 1529}, {25, 145, 3, 1529}, {27, 151, 3, 1529}, {28, 152, 6, 1529}, {29, 153, 3, 1529}, {31, 159, 1, 1529}, {32, 160, 3, 1529}, {33, 161, 3, 1529}, {34, 162, 1, 1529}, {22, 139, 1, 1530}, {24, 145, 3, 1530}, {25, 146, 3, 1530}, {27, 152, 3, 1530}, {28, 153, 6, 1530}, {29, 154, 3, 1530}, {31, 160, 1, 1530}, {32, 161, 3, 1530}, {33, 162, 3, 1530}, {34, 163, 1, 1530}, {22, 140, 1, 1531}, {24, 146, 3, 1531}, {25, 147, 3, 1531}, {27, 153, 3, 1531}, {28, 154, 6, 1531}, {29, 155, 3, 1531}, {31, 161, 1, 1531}, {32, 162, 3, 1531}, {33, 163, 3, 1531}, {34, 164, 1, 1531}, {20, 20, 1, 1532}, {21, 21, 4, 1532}, {22, 22, 4, 1532}, {23, 23, 6, 1532}, {24, 24, 12, 1532}, {25, 25, 6, 1532}, {26, 26, 4, 1532}, {27, 27, 12, 1532}, {28, 28, 12, 1532}, {29, 29, 4, 1532}, {30, 30, 1, 1532}, {31, 31, 4, 1532}, {32, 32, 6, 1532}, {33, 33, 4, 1532}, {34, 34, 1, 1532}, {20, 35, 1, 1533}, {21, 36, 4, 1533}, {22, 37, 4, 1533}, {23, 38, 6, 1533}, {24, 39, 12, 1533}, {25, 40, 6, 1533}, {26, 41, 4, 1533}, {27, 42, 12, 1533}, {28, 43, 12, 1533}, {29, 44, 4, 1533}, {30, 45, 1, 1533}, {31, 46, 4, 1533}, {32, 47, 6, 1533}, {33, 48, 4, 1533}, {34, 49, 1, 1533}, {20, 36, 1, 1534}, {21, 38, 4, 1534}, {22, 39, 4, 1534}, {23, 41, 6, 1534}, {24, 42, 12, 1534}, {25, 43, 6, 1534}, {26, 45, 4, 1534}, {27, 46, 12, 1534}, {28, 47, 12, 1534}, {29, 48, 4, 1534}, {30, 50, 1, 1534}, {31, 51, 4, 1534}, {32, 52, 6, 1534}, {33, 53, 4, 1534}, {34, 54, 1, 1534}, {20, 37, 1, 1535}, {21, 39, 4, 1535}, {22, 40, 4, 1535}, {23, 42, 6, 1535}, {24, 43, 12, 1535}, {25, 44, 6, 1535}, {26, 46, 4, 1535}, {27, 47, 12, 1535}, {28, 48, 12, 1535}, {29, 49, 4, 1535}, {30, 51, 1, 1535}, {31, 52, 4, 1535}, {32, 53, 6, 1535}, {33, 54, 4, 1535}, {34, 55, 1, 1535}, {20, 56, 1, 1536}, {21, 57, 4, 1536}, {22, 58, 4, 1536}, {23, 59, 6, 1536}, {24, 60, 12, 1536}, {25, 61, 6, 1536}, {26, 62, 4, 1536}, {27, 63, 12, 1536}, {28, 64, 12, 1536}, {29, 65, 4, 1536}, {30, 66, 1, 1536}, {31, 67, 4, 1536}, {32, 68, 6, 1536}, {33, 69, 4, 1536}, {34, 70, 1, 1536}, {20, 57, 1, 1537}, {21, 59, 4, 1537}, {22, 60, 4, 1537}, {23, 62, 6, 1537}, {24, 63, 12, 1537}, {25, 64, 6, 1537}, {26, 66, 4, 1537}, {27, 67, 12, 1537}, {28, 68, 12, 1537}, {29, 69, 4, 1537}, {30, 71, 1, 1537}, {31, 72, 4, 1537}, {32, 73, 6, 1537}, {33, 74, 4, 1537}, {34, 75, 1, 1537}, {20, 58, 1, 1538}, {21, 60, 4, 1538}, {22, 61, 4, 1538}, {23, 63, 6, 1538}, {24, 64, 12, 1538}, {25, 65, 6, 1538}, {26, 67, 4, 1538}, {27, 68, 12, 1538}, {28, 69, 12, 1538}, {29, 70, 4, 1538}, {30, 72, 1, 1538}, {31, 73, 4, 1538}, {32, 74, 6, 1538}, {33, 75, 4, 1538}, {34, 76, 1, 1538}, {20, 59, 1, 1539}, {21, 62, 4, 1539}, {22, 63, 4, 1539}, {23, 66, 6, 1539}, {24, 67, 12, 1539}, {25, 68, 6, 1539}, {26, 71, 4, 1539}, {27, 72, 12, 1539}, {28, 73, 12, 1539}, {29, 74, 4, 1539}, {30, 77, 1, 1539}, {31, 78, 4, 1539}, {32, 79, 6, 1539}, {33, 80, 4, 1539}, {34, 81, 1, 1539}, {20, 60, 1, 1540}, {21,

63, 4, 1540}, {22, 64, 4, 1540}, {23, 67, 6, 1540}, {24, 68, 12, 1540}, {25, 69, 6, 1540}, {26, 72, 4, 1540}, {27, 73, 12, 1540}, {28, 74, 12, 1540}, {29, 75, 4, 1540}, {30, 78, 1, 1540}, {31, 79, 4, 1540}, {32, 80, 6, 1540}, {33, 81, 4, 1540}, {34, 82, 1, 1540}, {20, 61, 1, 1541}, {21, 64, 4, 1541}, {22, 65, 4, 1541}, {23, 68, 6, 1541}, {24, 69, 12, 1541}, {25, 70, 6, 1541}, {26, 73, 4, 1541}, {27, 74, 12, 1541}, {28, 75, 12, 1541}, {29, 76, 4, 1541}, {30, 79, 1, 1541}, {31, 80, 4, 1541}, {32, 81, 6, 1541}, {33, 82, 4, 1541}, {34, 83, 1, 1541}, {20, 84, 1, 1542}, {21, 85, 4, 1542}, {22, 86, 4, 1542}, {23, 87, 6, 1542}, {24, 88, 12, 1542}, {25, 89, 6, 1542}, {26, 90, 4, 1542}, {27, 91, 12, 1542}, {28, 92, 12, 1542}, {29, 93, 4, 1542}, {30, 94, 1, 1542}, {31, 95, 4, 1542}, {32, 96, 6, 1542}, {33, 97, 4, 1542}, {34, 98, 1, 1542}, {20, 85, 1, 1543}, {21, 87, 4, 1543}, {22, 88, 4, 1543}, {23, 90, 6, 1543}, {24, 91, 12, 1543}, {25, 92, 6, 1543}, {26, 94, 4, 1543}, {27, 95, 12, 1543}, {28, 96, 12, 1543}, {29, 97, 4, 1543}, {30, 99, 1, 1543}, {31, 100, 4, 1543}, {32, 101, 6, 1543}, {33, 102, 4, 1543}, {34, 103, 1, 1543}, {20, 86, 1, 1544}, {21, 88, 4, 1544}, {22, 89, 4, 1544}, {23, 91, 6, 1544}, {24, 92, 12, 1544}, {25, 93, 6, 1544}, {26, 95, 4, 1544}, {27, 96, 12, 1544}, {28, 97, 12, 1544}, {29, 98, 4, 1544}, {30, 100, 1, 1544}, {31, 101, 4, 1544}, {32, 102, 6, 1544}, {33, 103, 4, 1544}, {34, 104, 1, 1544}, {20, 87, 1, 1545}, {21, 90, 4, 1545}, {22, 91, 4, 1545}, {23, 94, 6, 1545}, {24, 95, 12, 1545}, {25, 96, 6, 1545}, {26, 99, 4, 1545}, {27, 100, 12, 1545}, {28, 101, 12, 1545}, {29, 102, 4, 1545}, {30, 105, 1, 1545}, {31, 106, 4, 1545}, {32, 107, 6, 1545}, {33, 108, 4, 1545}, {34, 109, 1, 1545}, {20, 88, 1, 1546}, {21, 91, 4, 1546}, {22, 92, 4, 1546}, {23, 95, 6, 1546}, {24, 96, 12, 1546}, {25, 97, 6, 1546}, {26, 100, 4, 1546}, {27, 101, 12, 1546}, {28, 102, 12, 1546}, {29, 103, 4, 1546}, {30, 106, 1, 1546}, {31, 107, 4, 1546}, {32, 108, 6, 1546}, {33, 109, 4, 1546}, {34, 110, 1, 1546}, {20, 89, 1, 1547}, {21, 92, 4, 1547}, {22, 93, 4, 1547}, {23, 96, 6, 1547}, {24, 97, 12, 1547}, {25, 98, 6, 1547}, {26, 101, 4, 1547}, {27, 102, 12, 1547}, {28, 103, 12, 1547}, {29, 104, 4, 1547}, {30, 107, 1, 1547}, {31, 108, 4, 1547}, {32, 109, 6, 1547}, {33, 110, 4, 1547}, {34, 111, 1, 1547}, {20, 90, 1, 1548}, {21, 94, 4, 1548}, {22, 95, 4, 1548}, {23, 99, 6, 1548}, {24, 100, 12, 1548}, {25, 101, 6, 1548}, {26, 105, 4, 1548}, {27, 106, 12, 1548}, {28, 107, 12, 1548}, {29, 108, 4, 1548}, {30, 112, 1, 1548}, {31, 113, 4, 1548}, {32, 114, 6, 1548}, {33, 115, 4, 1548}, {34, 116, 1, 1548}, {20, 91, 1, 1549}, {21, 95, 4, 1549}, {22, 96, 4, 1549}, {23, 100, 6, 1549}, {24, 101, 12, 1549}, {25, 102, 6, 1549}, {26, 106, 4, 1549}, {27, 107, 12, 1549}, {28, 108, 12, 1549}, {29, 109, 4, 1549}, {30, 113, 1, 1549}, {31, 114, 4, 1549}, {32, 115, 6, 1549}, {33, 116, 4, 1549}, {34, 117, 1, 1549}, {20, 92, 1, 1550}, {21, 96, 4, 1550}, {22, 97, 4, 1550}, {23, 101, 6, 1550}, {24, 102, 12, 1550}, {25, 103, 6, 1550}, {26, 107, 4, 1550}, {27, 108, 12, 1550}, {28, 109, 12, 1550}, {29, 110, 4, 1550}, {30, 114, 1, 1550}, {31, 115, 4, 1550}, {32, 116, 6, 1550}, {33, 117, 4, 1550}, {34, 118, 1, 1550}, {20, 93, 1, 1551}, {21, 97, 4, 1551}, {22, 98, 4, 1551}, {23, 102, 6, 1551}, {24, 103, 12, 1551}, {25, 104, 6, 1551}, {26, 108, 4, 1551}, {27, 109, 12, 1551}, {28, 110, 12, 1551}, {29, 111, 4, 1551}, {30, 115, 1, 1551}, {31, 116, 4, 1551}, {32, 117, 6, 1551}, {33, 118, 4, 1551}, {34, 119, 1, 1551}, {20, 120, 1, 1552}, {21, 121, 4, 1552}, {22, 122, 4, 1552}, {23, 123, 6, 1552}, {24, 124, 12, 1552}, {25, 125, 6, 1552}, {26, 126, 4, 1552}, {27, 127, 12, 1552}, {28, 128, 12, 1552}, {29, 129, 4, 1552}, {30, 130, 1, 1552}, {31, 131, 4, 1552}, {32, 132, 6, 1552}, {33, 133, 4, 1552}, {34, 134, 1, 1552}, {20, 121, 1, 1553}, {21, 123, 4, 1553}, {22, 124, 4, 1553}, {23, 126, 6, 1553}, {24, 127, 12, 1553}, {25, 128, 6, 1553}, {26, 130, 4, 1553}, {27, 131, 12, 1553}, {28, 132, 12, 1553}, {29, 133, 4, 1553}, {30, 135, 1, 1553}, {31, 136, 4, 1553}, {32, 137, 6, 1553}, {33, 138, 4, 1553}, {34, 139, 1, 1553}, {20, 122, 1, 1554}, {21, 124, 4, 1554}, {22, 125, 4, 1554}, {23, 127, 6, 1554}, {24, 128, 12, 1554}, {25, 129, 6, 1554}, {26, 131, 4, 1554}, {27, 132, 12, 1554}, {28, 133, 12, 1554}, {29, 134, 4, 1554}, {30, 136, 1, 1554}, {31, 137, 4, 1554}, {32, 138, 6, 1554}, {33, 139, 4, 1554}, {34, 140, 1, 1554}, {20, 123, 1, 1555}, {21, 126, 4, 1555}, {22, 127, 4, 1555}, {23, 130, 6, 1555}, {24, 131, 12, 1555}, {25, 132, 6, 1555}, {26, 135, 4, 1555}, {27, 136, 12, 1555}, {28, 137, 12, 1555}, {29, 138, 4, 1555}, {30, 141, 1, 1555}, {31, 142, 4, 1555}, {32, 143, 6, 1555}, {33, 144, 4, 1555}, {34, 145, 1, 1555}, {20, 124, 1, 1556}, {21, 127, 4, 1556}, {22, 128, 4, 1556}, {23, 131, 6, 1556}, {24, 132, 12, 1556}, {25, 133, 6, 1556}, {26, 136, 4, 1556}, {27, 137, 12, 1556}, {28, 138, 12, 1556}, {29, 139, 4, 1556}, {30, 142, 1, 1556}, {31, 143, 4, 1556}, {32, 144, 6, 1556}, {33, 145, 4, 1556}, {34, 146, 1, 1556}, {20, 125, 1, 1557}, {21, 128, 4, 1557}, {22, 129, 4, 1557}, {23, 132, 6, 1557}, {24, 133, 12, 1557}, {25, 134, 6, 1557}, {26, 137, 4, 1557}, {27, 138, 12, 1557}, {28, 139, 12, 1557}, {29, 140, 4, 1557}, {30, 143, 1, 1557}, {31, 144, 4, 1557}, {32, 145, 6, 1557}, {33, 146, 4, 1557}, {34, 147, 1, 1557}, {20, 126, 1, 1558}, {21, 130, 4, 1558}, {22, 131, 4, 1558}, {23, 135, 6, 1558}, {24, 136, 12, 1558}, {25, 137, 6, 1558}, {26, 141, 4, 1558}, {27, 142, 12, 1558}, {28, 143, 12, 1558}, {29, 144, 4, 1558}, {30, 148, 1, 1558}, {31, 149, 4, 1558}, {32, 150, 6, 1558}, {33, 151, 4, 1558}, {34, 152, 1, 1558}, {20, 127, 1, 1559}, {21, 131, 4, 1559}, {22, 132, 4, 1559}, {23, 136, 6, 1559}, {24, 137, 12, 1559}, {25, 138, 6, 1559}, {26, 142, 4, 1559}, {27, 143, 12, 1559}, {28, 144, 12, 1559}, {29, 145, 4, 1559}, {30, 149, 1, 1559}, {31, 150, 4, 1559}, {32, 151, 6, 1559}, {33, 152, 4, 1559}, {34, 153, 1, 1559}, {20, 128, 1, 1560}, {21, 132, 4, 1560}, {22, 133, 4, 1560}, {23, 137, 6, 1560}, {24, 138, 12, 1560}, {25, 139, 6, 1560}, {26, 143, 4, 1560}, {27, 144, 12, 1560}, {28,

145, 12, 1560}, {29, 146, 4, 1560}, {30, 150, 1, 1560}, {31, 151, 4, 1560}, {32, 152, 6, 1560}, {33, 153, 4, 1560}, {34, 154, 1, 1560}, {20, 129, 1, 1561}, {21, 133, 4, 1561}, {22, 134, 4, 1561}, {23, 138, 6, 1561}, {24, 139, 12, 1561}, {25, 140, 6, 1561}, {26, 144, 4, 1561}, {27, 145, 12, 1561}, {28, 146, 12, 1561}, {29, 147, 4, 1561}, {30, 151, 1, 1561}, {31, 152, 4, 1561}, {32, 153, 6, 1561}, {33, 154, 4, 1561}, {34, 155, 1, 1561}, {20, 130, 1, 1562}, {21, 135, 4, 1562}, {22, 136, 4, 1562}, {23, 141, 6, 1562}, {24, 142, 12, 1562}, {25, 143, 6, 1562}, {26, 148, 4, 1562}, {27, 149, 12, 1562}, {28, 150, 12, 1562}, {29, 151, 4, 1562}, {30, 156, 1, 1562}, {31, 157, 4, 1562}, {32, 158, 6, 1562}, {33, 159, 4, 1562}, {34, 160, 1, 1562}, {20, 131, 1, 1563}, {21, 136, 4, 1563}, {22, 137, 4, 1563}, {23, 142, 6, 1563}, {24, 143, 12, 1563}, {25, 144, 6, 1563}, {26, 149, 4, 1563}, {27, 150, 12, 1563}, {28, 151, 12, 1563}, {29, 152, 4, 1563}, {30, 157, 1, 1563}, {31, 158, 4, 1563}, {32, 159, 6, 1563}, {33, 160, 4, 1563}, {34, 161, 1, 1563}, {20, 132, 1, 1564}, {21, 137, 4, 1564}, {22, 138, 4, 1564}, {23, 143, 6, 1564}, {24, 144, 12, 1564}, {25, 145, 6, 1564}, {26, 150, 4, 1564}, {27, 151, 12, 1564}, {28, 152, 12, 1564}, {29, 153, 4, 1564}, {30, 158, 1, 1564}, {31, 159, 4, 1564}, {32, 160, 6, 1564}, {33, 161, 4, 1564}, {34, 162, 1, 1564}, {20, 133, 1, 1565}, {21, 138, 4, 1565}, {22, 139, 4, 1565}, {23, 144, 6, 1565}, {24, 145, 12, 1565}, {25, 146, 6, 1565}, {26, 151, 4, 1565}, {27, 152, 12, 1565}, {28, 153, 12, 1565}, {29, 154, 4, 1565}, {30, 159, 1, 1565}, {31, 160, 4, 1565}, {32, 161, 6, 1565}, {33, 162, 4, 1565}, {34, 163, 1, 1565}, {20, 134, 1, 1566}, {21, 139, 4, 1566}, {22, 140, 4, 1566}, {23, 145, 6, 1566}, {24, 146, 12, 1566}, {25, 147, 6, 1566}, {26, 152, 4, 1566}, {27, 153, 12, 1566}, {28, 154, 12, 1566}, {29, 155, 4, 1566}, {30, 160, 1, 1566}, {31, 161, 4, 1566}, {32, 162, 6, 1566}, {33, 163, 4, 1566}, {34, 164, 1, 1566}, {20, 165, 1, 1567}, {21, 166, 4, 1567}, {22, 167, 4, 1567}, {23, 168, 6, 1567}, {24, 169, 12, 1567}, {25, 170, 6, 1567}, {26, 171, 4, 1567}, {27, 172, 12, 1567}, {28, 173, 12, 1567}, {29, 174, 4, 1567}, {30, 175, 1, 1567}, {31, 176, 4, 1567}, {32, 177, 6, 1567}, {33, 178, 4, 1567}, {34, 179, 1, 1567}, {20, 166, 1, 1568}, {21, 168, 4, 1568}, {22, 169, 4, 1568}, {23, 171, 6, 1568}, {24, 172, 12, 1568}, {25, 173, 6, 1568}, {26, 175, 4, 1568}, {27, 176, 12, 1568}, {28, 177, 12, 1568}, {29, 178, 4, 1568}, {30, 180, 1, 1568}, {31, 181, 4, 1568}, {32, 182, 6, 1568}, {33, 183, 4, 1568}, {34, 184, 1, 1568}, {20, 167, 1, 1569}, {21, 169, 4, 1569}, {22, 170, 4, 1569}, {23, 172, 6, 1569}, {24, 173, 12, 1569}, {25, 174, 6, 1569}, {26, 176, 4, 1569}, {27, 177, 12, 1569}, {28, 178, 12, 1569}, {29, 179, 4, 1569}, {30, 181, 1, 1569}, {31, 182, 4, 1569}, {32, 183, 6, 1569}, {33, 184, 4, 1569}, {34, 185, 1, 1569}, {20, 168, 1, 1570}, {21, 171, 4, 1570}, {22, 172, 4, 1570}, {23, 175, 6, 1570}, {24, 176, 12, 1570}, {25, 177, 6, 1570}, {26, 180, 4, 1570}, {27, 181, 12, 1570}, {28, 182, 12, 1570}, {29, 183, 4, 1570}, {30, 186, 1, 1570}, {31, 187, 4, 1570}, {32, 188, 6, 1570}, {33, 189, 4, 1570}, {34, 190, 1, 1570}, {20, 169, 1, 1571}, {21, 172, 4, 1571}, {22, 173, 4, 1571}, {23, 176, 6, 1571}, {24, 177, 12, 1571}, {25, 178, 6, 1571}, {26, 181, 4, 1571}, {27, 182, 12, 1571}, {28, 183, 12, 1571}, {29, 184, 4, 1571}, {30, 187, 1, 1571}, {31, 188, 4, 1571}, {32, 189, 6, 1571}, {33, 190, 4, 1571}, {34, 191, 1, 1571}, {20, 170, 1, 1572}, {21, 173, 4, 1572}, {22, 174, 4, 1572}, {23, 177, 6, 1572}, {24, 178, 12, 1572}, {25, 179, 6, 1572}, {26, 182, 4, 1572}, {27, 183, 12, 1572}, {28, 184, 12, 1572}, {29, 185, 4, 1572}, {30, 188, 1, 1572}, {31, 189, 4, 1572}, {32, 190, 6, 1572}, {33, 191, 4, 1572}, {34, 192, 1, 1572}, {20, 171, 1, 1573}, {21, 175, 4, 1573}, {22, 176, 4, 1573}, {23, 180, 6, 1573}, {24, 181, 12, 1573}, {25, 182, 6, 1573}, {26, 186, 4, 1573}, {27, 187, 12, 1573}, {28, 188, 12, 1573}, {29, 189, 4, 1573}, {30, 193, 1, 1573}, {31, 194, 4, 1573}, {32, 195, 6, 1573}, {33, 196, 4, 1573}, {34, 197, 1, 1573}, {20, 172, 1, 1574}, {21, 176, 4, 1574}, {22, 177, 4, 1574}, {23, 181, 6, 1574}, {24, 182, 12, 1574}, {25, 183, 6, 1574}, {26, 187, 4, 1574}, {27, 188, 12, 1574}, {28, 189, 12, 1574}, {29, 190, 4, 1574}, {30, 194, 1, 1574}, {31, 195, 4, 1574}, {32, 196, 6, 1574}, {33, 197, 4, 1574}, {34, 198, 1, 1574}, {20, 173, 1, 1575}, {21, 177, 4, 1575}, {22, 178, 4, 1575}, {23, 182, 6, 1575}, {24, 183, 12, 1575}, {25, 184, 6, 1575}, {26, 188, 4, 1575}, {27, 189, 12, 1575}, {28, 190, 12, 1575}, {29, 191, 4, 1575}, {30, 195, 1, 1575}, {31, 196, 4, 1575}, {32, 197, 6, 1575}, {33, 198, 4, 1575}, {34, 199, 1, 1575}, {20, 174, 1, 1576}, {21, 178, 4, 1576}, {22, 179, 4, 1576}, {23, 183, 6, 1576}, {24, 184, 12, 1576}, {25, 185, 6, 1576}, {26, 189, 4, 1576}, {27, 190, 12, 1576}, {28, 191, 12, 1576}, {29, 192, 4, 1576}, {30, 196, 1, 1576}, {31, 197, 4, 1576}, {32, 198, 6, 1576}, {33, 199, 4, 1576}, {34, 200, 1, 1576}, {20, 175, 1, 1577}, {21, 180, 4, 1577}, {22, 181, 4, 1577}, {23, 186, 6, 1577}, {24, 187, 12, 1577}, {25, 188, 6, 1577}, {26, 193, 4, 1577}, {27, 194, 12, 1577}, {28, 195, 12, 1577}, {29, 196, 4, 1577}, {30, 201, 1, 1577}, {31, 202, 4, 1577}, {32, 203, 6, 1577}, {33, 204, 4, 1577}, {34, 205, 1, 1577}, {20, 176, 1, 1578}, {21, 181, 4, 1578}, {22, 182, 4, 1578}, {23, 187, 6, 1578}, {24, 188, 12, 1578}, {25, 189, 6, 1578}, {26, 194, 4, 1578}, {27, 195, 12, 1578}, {28, 196, 12, 1578}, {29, 197, 4, 1578}, {30, 202, 1, 1578}, {31, 203, 4, 1578}, {32, 204, 6, 1578}, {33, 205, 4, 1578}, {34, 206, 1, 1578}, {20, 177, 1, 1579}, {21, 182, 4, 1579}, {22, 183, 4, 1579}, {23, 188, 6, 1579}, {24, 189, 12, 1579}, {25, 190, 6, 1579}, {26, 195, 4, 1579}, {27, 196, 12, 1579}, {28, 197, 12, 1579}, {29, 198, 4, 1579}, {30, 203, 1, 1579}, {31, 204, 4, 1579}, {32, 205, 6, 1579}, {33, 206, 4, 1579}, {34, 207, 1, 1579}, {20, 178, 1, 1580}, {21, 183, 4, 1580}, {22, 184, 4, 1580}, {23, 189, 6, 1580}, {24, 190, 12, 1580}, {25, 191, 6, 1580}, {26, 196, 4, 1580}, {27, 197, 12, 1580}, {28, 198, 12,

1580}, {29, 199, 4, 1580}, {30, 204, 1, 1580}, {31, 205, 4, 1580}, {32, 206, 6, 1580}, {33, 207, 4, 1580}, {34, 208, 1, 1580}, {20, 179, 1, 1581}, {21, 184, 4, 1581}, {22, 185, 4, 1581}, {23, 190, 6, 1581}, {24, 191, 12, 1581}, {25, 192, 6, 1581}, {26, 197, 4, 1581}, {27, 198, 12, 1581}, {28, 199, 12, 1581}, {29, 200, 4, 1581}, {30, 205, 1, 1581}, {31, 206, 4, 1581}, {32, 207, 6, 1581}, {33, 208, 4, 1581}, {34, 209, 1, 1581}, {20, 180, 1, 1582}, {21, 186, 4, 1582}, {22, 187, 4, 1582}, {23, 193, 6, 1582}, {24, 194, 12, 1582}, {25, 195, 6, 1582}, {26, 201, 4, 1582}, {27, 202, 12, 1582}, {28, 203, 12, 1582}, {29, 204, 4, 1582}, {30, 210, 1, 1582}, {31, 211, 4, 1582}, {32, 212, 6, 1582}, {33, 213, 4, 1582}, {34, 214, 1, 1582}, {20, 181, 1, 1583}, {21, 187, 4, 1583}, {22, 188, 4, 1583}, {23, 194, 6, 1583}, {24, 195, 12, 1583}, {25, 196, 6, 1583}, {26, 202, 4, 1583}, {27, 203, 12, 1583}, {28, 204, 12, 1583}, {29, 205, 4, 1583}, {30, 211, 1, 1583}, {31, 212, 4, 1583}, {32, 213, 6, 1583}, {33, 214, 4, 1583}, {34, 215, 1, 1583}, {20, 182, 1, 1584}, {21, 188, 4, 1584}, {22, 189, 4, 1584}, {23, 195, 6, 1584}, {24, 196, 12, 1584}, {25, 197, 6, 1584}, {26, 203, 4, 1584}, {27, 204, 12, 1584}, {28, 205, 12, 1584}, {29, 206, 4, 1584}, {30, 212, 1, 1584}, {31, 213, 4, 1584}, {32, 214, 6, 1584}, {33, 215, 4, 1584}, {34, 216, 1, 1584}, {20, 183, 1, 1585}, {21, 189, 4, 1585}, {22, 190, 4, 1585}, {23, 196, 6, 1585}, {24, 197, 12, 1585}, {25, 198, 6, 1585}, {26, 204, 4, 1585}, {27, 205, 12, 1585}, {28, 206, 12, 1585}, {29, 207, 4, 1585}, {30, 213, 1, 1585}, {31, 214, 4, 1585}, {32, 215, 6, 1585}, {33, 216, 4, 1585}, {34, 217, 1, 1585}, {20, 184, 1, 1586}, {21, 190, 4, 1586}, {22, 191, 4, 1586}, {23, 197, 6, 1586}, {24, 198, 12, 1586}, {25, 199, 6, 1586}, {26, 205, 4, 1586}, {27, 206, 12, 1586}, {28, 207, 12, 1586}, {29, 208, 4, 1586}, {30, 214, 1, 1586}, {31, 215, 4, 1586}, {32, 216, 6, 1586}, {33, 217, 4, 1586}, {34, 218, 1, 1586}, {20, 185, 1, 1587}, {21, 191, 4, 1587}, {22, 192, 4, 1587}, {23, 198, 6, 1587}, {24, 199, 12, 1587}, {25, 200, 6, 1587}, {26, 206, 4, 1587}, {27, 207, 12, 1587}, {28, 208, 12, 1587}, {29, 209, 4, 1587}, {30, 215, 1, 1587}, {31, 216, 4, 1587}, {32, 217, 6, 1587}, {33, 218, 4, 1587}, {34, 219, 1, 1587}, {35, 35, 1, 1588}, {36, 36, 2, 1588}, {37, 37, 2, 1588}, {38, 38, 1, 1588}, {39, 39, 2, 1588}, {40, 40, 1, 1588}, {35, 36, 1, 1589}, {36, 38, 2, 1589}, {37, 39, 2, 1589}, {38, 41, 1, 1589}, {39, 42, 2, 1589}, {40, 43, 1, 1589}, {35, 37, 1, 1590}, {36, 39, 2, 1590}, {37, 40, 2, 1590}, {38, 42, 1, 1590}, {39, 43, 2, 1590}, {40, 44, 1, 1590}, {35, 38, 1, 1591}, {36, 41, 2, 1591}, {37, 42, 2, 1591}, {38, 45, 1, 1591}, {39, 46, 2, 1591}, {40, 47, 1, 1591}, {35, 39, 1, 1592}, {36, 42, 2, 1592}, {37, 43, 2, 1592}, {38, 46, 1, 1592}, {39, 47, 2, 1592}, {40, 48, 1, 1592}, {35, 40, 1, 1593}, {36, 43, 2, 1593}, {37, 44, 2, 1593}, {38, 47, 1, 1593}, {39, 48, 2, 1593}, {40, 49, 1, 1593}, {35, 41, 1, 1594}, {36, 45, 2, 1594}, {37, 46, 2, 1594}, {38, 50, 1, 1594}, {39, 51, 2, 1594}, {40, 52, 1, 1594}, {35, 42, 1, 1595}, {36, 46, 2, 1595}, {37, 47, 2, 1595}, {38, 51, 1, 1595}, {39, 52, 2, 1595}, {40, 53, 1, 1595}, {35, 43, 1, 1596}, {36, 47, 2, 1596}, {37, 48, 2, 1596}, {38, 52, 1, 1596}, {39, 53, 2, 1596}, {40, 54, 1, 1596}, {35, 44, 1, 1597}, {36, 48, 2, 1597}, {37, 49, 2, 1597}, {38, 53, 1, 1597}, {39, 54, 2, 1597}, {40, 55, 1, 1597}, {36, 36, 1, 1598}, {38, 38, 2, 1598}, {39, 39, 2, 1598}, {41, 41, 1, 1598}, {42, 42, 2, 1598}, {43, 43, 1, 1598}, {36, 37, 1, 1599}, {38, 39, 2, 1599}, {39, 40, 2, 1599}, {41, 42, 1, 1599}, {42, 43, 2, 1599}, {43, 44, 1, 1599}, {36, 38, 1, 1600}, {38, 41, 2, 1600}, {39, 42, 2, 1600}, {41, 45, 1, 1600}, {42, 46, 2, 1600}, {43, 47, 1, 1600}, {36, 39, 1, 1601}, {38, 42, 2, 1601}, {39, 43, 2, 1601}, {41, 46, 1, 1601}, {42, 47, 2, 1601}, {43, 48, 1, 1601}, {36, 40, 1, 1602}, {38, 43, 2, 1602}, {39, 44, 2, 1602}, {41, 47, 1, 1602}, {42, 48, 2, 1602}, {43, 49, 1, 1602}, {36, 41, 1, 1603}, {38, 45, 2, 1603}, {39, 46, 2, 1603}, {41, 50, 1, 1603}, {42, 51, 2, 1603}, {43, 52, 1, 1603}, {36, 42, 1, 1604}, {38, 46, 2, 1604}, {39, 47, 2, 1604}, {41, 51, 1, 1604}, {42, 52, 2, 1604}, {43, 53, 1, 1604}, {36, 43, 1, 1605}, {38, 47, 2, 1605}, {39, 48, 2, 1605}, {41, 52, 1, 1605}, {42, 53, 2, 1605}, {43, 54, 1, 1605}, {36, 44, 1, 1606}, {38, 48, 2, 1606}, {39, 49, 2, 1606}, {41, 53, 1, 1606}, {42, 54, 2, 1606}, {43, 55, 1, 1606}, {37, 37, 1, 1607}, {39, 39, 2, 1607}, {40, 40, 2, 1607}, {42, 42, 1, 1607}, {43, 43, 2, 1607}, {44, 44, 1, 1607}, {37, 38, 1, 1608}, {39, 41, 2, 1608}, {40, 42, 2, 1608}, {42, 45, 1, 1608}, {43, 46, 2, 1608}, {44, 47, 1, 1608}, {37, 39, 1, 1609}, {39, 42, 2, 1609}, {40, 43, 2, 1609}, {42, 46, 1, 1609}, {43, 47, 2, 1609}, {44, 48, 1, 1609}, {37, 40, 1, 1610}, {39, 43, 2, 1610}, {40, 44, 2, 1610}, {42, 47, 1, 1610}, {43, 48, 2, 1610}, {44, 49, 1, 1610}, {37, 41, 1, 1611}, {39, 45, 2, 1611}, {40, 46, 2, 1611}, {42, 50, 1, 1611}, {43, 51, 2, 1611}, {44, 52, 1, 1611}, {37, 42, 1, 1612}, {39, 46, 2, 1612}, {40, 47, 2, 1612}, {42, 51, 1, 1612}, {43, 52, 2, 1612}, {44, 53, 1, 1612}, {37, 43, 1, 1613}, {39, 47, 2, 1613}, {40, 48, 2, 1613}, {42, 52, 1, 1613}, {43, 53, 2, 1613}, {44, 54, 1, 1613}, {37, 44, 1, 1614}, {39, 48, 2, 1614}, {40, 49, 2, 1614}, {42, 53, 1, 1614}, {43, 54, 2, 1614}, {44, 55, 1, 1614}, {38, 38, 1, 1615}, {41, 41, 2, 1615}, {42, 42, 2, 1615}, {45, 45, 1, 1615}, {46, 46, 2, 1615}, {47, 47, 1, 1615}, {38, 39, 1, 1616}, {41, 42, 2, 1616}, {42, 43, 2, 1616}, {45, 46, 1, 1616}, {46, 47, 2, 1616}, {47, 48, 1, 1616}, {38, 40, 1, 1617}, {41, 43, 2, 1617}, {42, 44, 2, 1617}, {45, 47, 1, 1617}, {46, 48, 2, 1617}, {47, 49, 1, 1617}, {38, 41, 1, 1618}, {41, 45, 2, 1618}, {42, 46, 2, 1618}, {45, 50, 1, 1618}, {46, 51, 2, 1618}, {47, 52, 1, 1618}, {38, 42, 1, 1619}, {41, 46, 2, 1619}, {42, 47, 2, 1619}, {45, 51, 1, 1619}, {46, 52, 2, 1619}, {47, 53, 1, 1619}, {38, 43, 1, 1620}, {41, 47, 2, 1620}, {42, 48, 2, 1620}, {45, 52, 1, 1620}, {46, 53, 2, 1620}, {47, 54, 1, 1620}, {38, 44, 1, 1621}, {41, 48, 2, 1621}, {42, 49, 2, 1621}, {45, 53, 1, 1621}, {46, 54, 2, 1621},

{47, 55, 1, 1621}, {39, 39, 1, 1622}, {42, 42, 2, 1622}, {43, 43, 2, 1622}, {46, 46, 1, 1622}, {47, 47, 2, 1622}, {48, 48, 1, 1622}, {39, 40, 1, 1623}, {42, 43, 2, 1623}, {43, 44, 2, 1623}, {46, 47, 1, 1623}, {47, 48, 2, 1623}, {48, 49, 1, 1623}, {39, 41, 1, 1624}, {42, 45, 2, 1624}, {43, 46, 2, 1624}, {46, 50, 1, 1624}, {47, 51, 2, 1624}, {48, 52, 1, 1624}, {39, 42, 1, 1625}, {42, 46, 2, 1625}, {43, 47, 2, 1625}, {46, 51, 1, 1625}, {47, 52, 2, 1625}, {48, 53, 1, 1625}, {39, 43, 1, 1626}, {42, 47, 2, 1626}, {43, 48, 2, 1626}, {46, 52, 1, 1626}, {47, 53, 2, 1626}, {48, 54, 1, 1626}, {39, 44, 1, 1627}, {42, 48, 2, 1627}, {43, 49, 2, 1627}, {46, 53, 1, 1627}, {47, 54, 2, 1627}, {48, 55, 1, 1627}, {40, 40, 1, 1628}, {43, 43, 2, 1628}, {44, 44, 2, 1628}, {47, 47, 1, 1628}, {48, 48, 2, 1628}, {49, 49, 1, 1628}, {40, 41, 1, 1629}, {43, 45, 2, 1629}, {44, 46, 2, 1629}, {47, 50, 1, 1629}, {48, 51, 2, 1629}, {49, 52, 1, 1629}, {40, 42, 1, 1630}, {43, 46, 2, 1630}, {44, 47, 2, 1630}, {47, 51, 1, 1630}, {48, 52, 2, 1630}, {49, 53, 1, 1630}, {40, 43, 1, 1631}, {43, 47, 2, 1631}, {44, 48, 2, 1631}, {47, 52, 1, 1631}, {48, 53, 2, 1631}, {49, 54, 1, 1631}, {40, 44, 1, 1632}, {43, 48, 2, 1632}, {44, 49, 2, 1632}, {47, 53, 1, 1632}, {48, 54, 2, 1632}, {49, 55, 1, 1632}, {41, 41, 1, 1633}, {45, 45, 2, 1633}, {46, 46, 2, 1633}, {50, 50, 1, 1633}, {51, 51, 2, 1633}, {52, 52, 1, 1633}, {41, 42, 1, 1634}, {45, 46, 2, 1634}, {46, 47, 2, 1634}, {50, 51, 1, 1634}, {51, 52, 2, 1634}, {52, 53, 1, 1634}, {41, 43, 1, 1635}, {45, 47, 2, 1635}, {46, 48, 2, 1635}, {50, 52, 1, 1635}, {51, 53, 2, 1635}, {52, 54, 1, 1635}, {41, 44, 1, 1636}, {45, 48, 2, 1636}, {46, 49, 2, 1636}, {50, 53, 1, 1636}, {51, 54, 2, 1636}, {52, 55, 1, 1636}, {42, 42, 1, 1637}, {46, 46, 2, 1637}, {47, 47, 2, 1637}, {51, 51, 1, 1637}, {52, 52, 2, 1637}, {53, 53, 1, 1637}, {42, 43, 1, 1638}, {46, 47, 2, 1638}, {47, 48, 2, 1638}, {51, 52, 1, 1638}, {52, 53, 2, 1638}, {53, 54, 1, 1638}, {42, 44, 1, 1639}, {46, 48, 2, 1639}, {47, 49, 2, 1639}, {51, 53, 1, 1639}, {52, 54, 2, 1639}, {53, 55, 1, 1639}, {43, 43, 1, 1640}, {47, 47, 2, 1640}, {48, 48, 2, 1640}, {52, 52, 1, 1640}, {53, 53, 2, 1640}, {54, 54, 1, 1640}, {43, 44, 1, 1641}, {47, 48, 2, 1641}, {48, 49, 2, 1641}, {52, 53, 1, 1641}, {53, 54, 2, 1641}, {54, 55, 1, 1641}, {44, 44, 1, 1642}, {48, 48, 2, 1642}, {49, 49, 2, 1642}, {53, 53, 1, 1642}, {54, 54, 2, 1642}, {55, 55, 1, 1642}, {35, 56, 1, 1643}, {36, 57, 2, 1643}, {37, 58, 2, 1643}, {38, 59, 1, 1643}, {39, 60, 2, 1643}, {40, 61, 1, 1643}, {35, 57, 1, 1644}, {36, 59, 2, 1644}, {37, 60, 2, 1644}, {38, 62, 1, 1644}, {39, 63, 2, 1644}, {40, 64, 1, 1644}, {35, 58, 1, 1645}, {36, 60, 2, 1645}, {37, 61, 2, 1645}, {38, 63, 1, 1645}, {39, 64, 2, 1645}, {40, 65, 1, 1645}, {35, 59, 1, 1646}, {36, 62, 2, 1646}, {37, 63, 2, 1646}, {38, 66, 1, 1646}, {39, 67, 2, 1646}, {40, 68, 1, 1646}, {35, 60, 1, 1647}, {36, 63, 2, 1647}, {37, 64, 2, 1647}, {38, 67, 1, 1647}, {39, 68, 2, 1647}, {40, 69, 1, 1647}, {35, 61, 1, 1648}, {36, 64, 2, 1648}, {37, 65, 2, 1648}, {38, 68, 1, 1648}, {39, 69, 2, 1648}, {40, 70, 1, 1648}, {35, 62, 1, 1649}, {36, 66, 2, 1649}, {37, 67, 2, 1649}, {38, 71, 1, 1649}, {39, 72, 2, 1649}, {40, 73, 1, 1649}, {35, 63, 1, 1650}, {36, 67, 2, 1650}, {37, 68, 2, 1650}, {38, 72, 1, 1650}, {39, 73, 2, 1650}, {40, 74, 1, 1650}, {35, 64, 1, 1651}, {36, 68, 2, 1651}, {37, 69, 2, 1651}, {38, 73, 1, 1651}, {39, 74, 2, 1651}, {40, 75, 1, 1651}, {35, 65, 1, 1652}, {36, 69, 2, 1652}, {37, 70, 2, 1652}, {38, 74, 1, 1652}, {39, 75, 2, 1652}, {40, 76, 1, 1652}, {35, 66, 1, 1653}, {36, 71, 2, 1653}, {37, 72, 2, 1653}, {38, 77, 1, 1653}, {39, 78, 2, 1653}, {40, 79, 1, 1653}, {35, 67, 1, 1654}, {36, 72, 2, 1654}, {37, 73, 2, 1654}, {38, 78, 1, 1654}, {39, 79, 2, 1654}, {40, 80, 1, 1654}, {35, 68, 1, 1655}, {36, 73, 2, 1655}, {37, 74, 2, 1655}, {38, 79, 1, 1655}, {39, 80, 2, 1655}, {40, 81, 1, 1655}, {35, 69, 1, 1656}, {36, 74, 2, 1656}, {37, 75, 2, 1656}, {38, 80, 1, 1656}, {39, 81, 2, 1656}, {40, 82, 1, 1656}, {35, 70, 1, 1657}, {36, 75, 2, 1657}, {37, 76, 2, 1657}, {38, 81, 1, 1657}, {39, 82, 2, 1657}, {40, 83, 1, 1657}, {36, 56, 1, 1658}, {38, 57, 2, 1658}, {39, 58, 2, 1658}, {41, 59, 1, 1658}, {42, 60, 2, 1658}, {43, 61, 1, 1658}, {36, 57, 1, 1659}, {38, 59, 2, 1659}, {39, 60, 2, 1659}, {41, 62, 1, 1659}, {42, 63, 2, 1659}, {43, 64, 1, 1659}, {36, 58, 1, 1660}, {38, 60, 2, 1660}, {39, 61, 2, 1660}, {41, 63, 1, 1660}, {42, 64, 2, 1660}, {43, 65, 1, 1660}, {36, 59, 1, 1661}, {38, 62, 2, 1661}, {39, 63, 2, 1661}, {41, 66, 1, 1661}, {42, 67, 2, 1661}, {43, 68, 1, 1661}, {36, 60, 1, 1662}, {38, 63, 2, 1662}, {39, 64, 2, 1662}, {41, 67, 1, 1662}, {42, 68, 2, 1662}, {43, 69, 1, 1662}, {36, 61, 1, 1663}, {38, 64, 2, 1663}, {39, 65, 2, 1663}, {41, 68, 1, 1663}, {42, 69, 2, 1663}, {43, 70, 1, 1663}, {36, 62, 1, 1664}, {38, 66, 2, 1664}, {39, 67, 2, 1664}, {41, 71, 1, 1664}, {42, 72, 2, 1664}, {43, 73, 1, 1664}, {36, 63, 1, 1665}, {38, 67, 2, 1665}, {39, 68, 2, 1665}, {41, 72, 1, 1665}, {42, 73, 2, 1665}, {43, 74, 1, 1665}, {36, 64, 1, 1666}, {38, 68, 2, 1666}, {39, 69, 2, 1666}, {41, 73, 1, 1666}, {42, 74, 2, 1666}, {43, 75, 1, 1666}, {36, 65, 1, 1667}, {38, 69, 2, 1667}, {39, 70, 2, 1667}, {41, 74, 1, 1667}, {42, 75, 2, 1667}, {43, 76, 1, 1667}, {36, 66, 1, 1668}, {38, 71, 2, 1668}, {39, 72, 2, 1668}, {41, 77, 1, 1668}, {42, 78, 2, 1668}, {43, 79, 1, 1668}, {36, 67, 1, 1669}, {38, 72, 2, 1669}, {39, 73, 2, 1669}, {41, 78, 1, 1669}, {42, 79, 2, 1669}, {43, 80, 1, 1669}, {36, 68, 1, 1670}, {38, 73, 2, 1670}, {39, 74, 2, 1670}, {41, 79, 1, 1670}, {42, 80, 2, 1670}, {43, 81, 1, 1670}, {36, 69, 1, 1671}, {38, 74, 2, 1671}, {39, 75, 2, 1671}, {41, 80, 1, 1671}, {42, 81, 2, 1671}, {43, 82, 1, 1671}, {36, 70, 1, 1672}, {38, 75, 2, 1672}, {39, 76, 2, 1672}, {41, 81, 1, 1672}, {42, 82, 2, 1672}, {43, 83, 1, 1672}, {37, 56, 1, 1673}, {39, 57, 2, 1673}, {40, 58, 2, 1673}, {42, 59, 1, 1673}, {43, 60, 2, 1673}, {44, 61, 1, 1673}, {37, 57, 1, 1674}, {39, 59, 2, 1674}, {40, 60, 2, 1674}, {42, 62, 1, 1674}, {43, 63, 2, 1674}, {44, 64, 1, 1674}, {37, 58, 1, 1675}, {39, 60, 2, 1675}, {40, 61, 2, 1675},

{42, 63, 1, 1675}, {43, 64, 2, 1675}, {44, 65, 1, 1675}, {37, 59, 1, 1676}, {39, 62, 2, 1676}, {40, 63, 2, 1676}, {42, 66, 1, 1676}, {43, 67, 2, 1676}, {44, 68, 1, 1676}, {37, 60, 1, 1677}, {39, 63, 2, 1677}, {40, 64, 2, 1677}, {42, 67, 1, 1677}, {43, 68, 2, 1677}, {44, 69, 1, 1677}, {37, 61, 1, 1678}, {39, 64, 2, 1678}, {40, 65, 2, 1678}, {42, 68, 1, 1678}, {43, 69, 2, 1678}, {44, 70, 1, 1678}, {37, 62, 1, 1679}, {39, 66, 2, 1679}, {40, 67, 2, 1679}, {42, 71, 1, 1679}, {43, 72, 2, 1679}, {44, 73, 1, 1679}, {37, 63, 1, 1680}, {39, 67, 2, 1680}, {40, 68, 2, 1680}, {42, 72, 1, 1680}, {43, 73, 2, 1680}, {44, 74, 1, 1680}, {37, 64, 1, 1681}, {39, 68, 2, 1681}, {40, 69, 2, 1681}, {42, 73, 1, 1681}, {43, 74, 2, 1681}, {44, 75, 1, 1681}, {37, 65, 1, 1682}, {39, 69, 2, 1682}, {40, 70, 2, 1682}, {42, 74, 1, 1682}, {43, 75, 2, 1682}, {44, 76, 1, 1682}, {37, 66, 1, 1683}, {39, 71, 2, 1683}, {40, 72, 2, 1683}, {42, 77, 1, 1683}, {43, 78, 2, 1683}, {44, 79, 1, 1683}, {37, 67, 1, 1684}, {39, 72, 2, 1684}, {40, 73, 2, 1684}, {42, 78, 1, 1684}, {43, 79, 2, 1684}, {44, 80, 1, 1684}, {37, 68, 1, 1685}, {39, 73, 2, 1685}, {40, 74, 2, 1685}, {42, 79, 1, 1685}, {43, 80, 2, 1685}, {44, 81, 1, 1685}, {37, 69, 1, 1686}, {39, 74, 2, 1686}, {40, 75, 2, 1686}, {42, 80, 1, 1686}, {43, 81, 2, 1686}, {44, 82, 1, 1686}, {37, 70, 1, 1687}, {39, 75, 2, 1687}, {40, 76, 2, 1687}, {42, 81, 1, 1687}, {43, 82, 2, 1687}, {44, 83, 1, 1687}, {38, 56, 1, 1688}, {41, 57, 2, 1688}, {42, 58, 2, 1688}, {45, 59, 1, 1688}, {46, 60, 2, 1688}, {47, 61, 1, 1688}, {38, 57, 1, 1689}, {41, 59, 2, 1689}, {42, 60, 2, 1689}, {45, 62, 1, 1689}, {46, 63, 2, 1689}, {47, 64, 1, 1689}, {38, 58, 1, 1690}, {41, 60, 2, 1690}, {42, 61, 2, 1690}, {45, 63, 1, 1690}, {46, 64, 2, 1690}, {47, 65, 1, 1690}, {38, 59, 1, 1691}, {41, 62, 2, 1691}, {42, 63, 2, 1691}, {45, 66, 1, 1691}, {46, 67, 2, 1691}, {47, 68, 1, 1691}, {38, 60, 1, 1692}, {41, 63, 2, 1692}, {42, 64, 2, 1692}, {45, 67, 1, 1692}, {46, 68, 2, 1692}, {47, 69, 1, 1692}, {38, 61, 1, 1693}, {41, 64, 2, 1693}, {42, 65, 2, 1693}, {45, 68, 1, 1693}, {46, 69, 2, 1693}, {47, 70, 1, 1693}, {38, 62, 1, 1694}, {41, 66, 2, 1694}, {42, 67, 2, 1694}, {45, 71, 1, 1694}, {46, 72, 2, 1694}, {47, 73, 1, 1694}, {38, 63, 1, 1695}, {41, 67, 2, 1695}, {42, 68, 2, 1695}, {45, 72, 1, 1695}, {46, 73, 2, 1695}, {47, 74, 1, 1695}, {38, 64, 1, 1696}, {41, 68, 2, 1696}, {42, 69, 2, 1696}, {45, 73, 1, 1696}, {46, 74, 2, 1696}, {47, 75, 1, 1696}, {38, 65, 1, 1697}, {41, 69, 2, 1697}, {42, 70, 2, 1697}, {45, 74, 1, 1697}, {46, 75, 2, 1697}, {47, 76, 1, 1697}, {38, 66, 1, 1698}, {41, 71, 2, 1698}, {42, 72, 2, 1698}, {45, 77, 1, 1698}, {46, 78, 2, 1698}, {47, 79, 1, 1698}, {38, 67, 1, 1699}, {41, 72, 2, 1699}, {42, 73, 2, 1699}, {45, 78, 1, 1699}, {46, 79, 2, 1699}, {47, 80, 1, 1699}, {38, 68, 1, 1700}, {41, 73, 2, 1700}, {42, 74, 2, 1700}, {45, 79, 1, 1700}, {46, 80, 2, 1700}, {47, 81, 1, 1700}, {38, 69, 1, 1701}, {41, 74, 2, 1701}, {42, 75, 2, 1701}, {45, 80, 1, 1701}, {46, 81, 2, 1701}, {47, 82, 1, 1701}, {38, 70, 1, 1702}, {41, 75, 2, 1702}, {42, 76, 2, 1702}, {45, 81, 1, 1702}, {46, 82, 2, 1702}, {47, 83, 1, 1702}, {39, 56, 1, 1703}, {42, 57, 2, 1703}, {43, 58, 2, 1703}, {46, 59, 1, 1703}, {47, 60, 2, 1703}, {48, 61, 1, 1703}, {39, 57, 1, 1704}, {42, 59, 2, 1704}, {43, 60, 2, 1704}, {46, 62, 1, 1704}, {47, 63, 2, 1704}, {48, 64, 1, 1704}, {39, 58, 1, 1705}, {42, 60, 2, 1705}, {43, 61, 2, 1705}, {46, 63, 1, 1705}, {47, 64, 2, 1705}, {48, 65, 1, 1705}, {39, 59, 1, 1706}, {42, 62, 2, 1706}, {43, 63, 2, 1706}, {46, 66, 1, 1706}, {47, 67, 2, 1706}, {48, 68, 1, 1706}, {39, 60, 1, 1707}, {42, 63, 2, 1707}, {43, 64, 2, 1707}, {46, 67, 1, 1707}, {47, 68, 2, 1707}, {48, 69, 1, 1707}, {39, 61, 1, 1708}, {42, 64, 2, 1708}, {43, 65, 2, 1708}, {46, 68, 1, 1708}, {47, 69, 2, 1708}, {48, 70, 1, 1708}, {39, 62, 1, 1709}, {42, 66, 2, 1709}, {43, 67, 2, 1709}, {46, 71, 1, 1709}, {47, 72, 2, 1709}, {48, 73, 1, 1709}, {39, 63, 1, 1710}, {42, 67, 2, 1710}, {43, 68, 2, 1710}, {46, 72, 1, 1710}, {47, 73, 2, 1710}, {48, 74, 1, 1710}, {39, 64, 1, 1711}, {42, 68, 2, 1711}, {43, 69, 2, 1711}, {46, 73, 1, 1711}, {47, 74, 2, 1711}, {48, 75, 1, 1711}, {39, 65, 1, 1712}, {42, 69, 2, 1712}, {43, 70, 2, 1712}, {46, 74, 1, 1712}, {47, 75, 2, 1712}, {48, 76, 1, 1712}, {39, 66, 1, 1713}, {42, 71, 2, 1713}, {43, 72, 2, 1713}, {46, 77, 1, 1713}, {47, 78, 2, 1713}, {48, 79, 1, 1713}, {39, 67, 1, 1714}, {42, 72, 2, 1714}, {43, 73, 2, 1714}, {46, 78, 1, 1714}, {47, 79, 2, 1714}, {48, 80, 1, 1714}, {39, 68, 1, 1715}, {42, 73, 2, 1715}, {43, 74, 2, 1715}, {46, 79, 1, 1715}, {47, 80, 2, 1715}, {48, 81, 1, 1715}, {39, 69, 1, 1716}, {42, 74, 2, 1716}, {43, 75, 2, 1716}, {46, 80, 1, 1716}, {47, 81, 2, 1716}, {48, 82, 1, 1716}, {39, 70, 1, 1717}, {42, 75, 2, 1717}, {43, 76, 2, 1717}, {46, 81, 1, 1717}, {47, 82, 2, 1717}, {48, 83, 1, 1717}, {40, 56, 1, 1718}, {43, 57, 2, 1718}, {44, 58, 2, 1718}, {47, 59, 1, 1718}, {48, 60, 2, 1718}, {49, 61, 1, 1718}, {40, 57, 1, 1719}, {43, 59, 2, 1719}, {44, 60, 2, 1719}, {47, 62, 1, 1719}, {48, 63, 2, 1719}, {49, 64, 1, 1719}, {40, 58, 1, 1720}, {43, 60, 2, 1720}, {44, 61, 2, 1720}, {47, 63, 1, 1720}, {48, 64, 2, 1720}, {49, 65, 1, 1720}, {40, 59, 1, 1721}, {43, 62, 2, 1721}, {44, 63, 2, 1721}, {47, 66, 1, 1721}, {48, 67, 2, 1721}, {49, 68, 1, 1721}, {40, 60, 1, 1722}, {43, 63, 2, 1722}, {44, 64, 2, 1722}, {47, 67, 1, 1722}, {48, 68, 2, 1722}, {49, 69, 1, 1722}, {40, 61, 1, 1723}, {43, 64, 2, 1723}, {44, 65, 2, 1723}, {47, 68, 1, 1723}, {48, 69, 2, 1723}, {49, 70, 1, 1723}, {40, 62, 1, 1724}, {43, 66, 2, 1724}, {44, 67, 2, 1724}, {47, 71, 1, 1724}, {48, 72, 2, 1724}, {49, 73, 1, 1724}, {40, 63, 1, 1725}, {43, 67, 2, 1725}, {44, 68, 2, 1725}, {47, 72, 1, 1725}, {48, 73, 2, 1725}, {49, 74, 1, 1725}, {40, 64, 1, 1726}, {43, 68, 2, 1726}, {44, 69, 2, 1726}, {47, 73, 1, 1726}, {48, 74, 2, 1726}, {49, 75, 1, 1726}, {40, 65, 1, 1727}, {43, 69, 2, 1727}, {44, 70, 2, 1727}, {47, 74, 1, 1727}, {48, 75, 2, 1727}, {49, 76, 1, 1727}, {40, 66, 1, 1728}, {43, 71, 2, 1728}, {44, 72, 2, 1728}, {47, 77, 1, 1728}, {48, 78, 2, 1728}, {49, 79, 1, 1728}, {40, 67, 1, 1729},

{43, 72, 2, 1729}, {44, 73, 2, 1729}, {47, 78, 1, 1729}, {48, 79, 2, 1729}, {49, 80, 1, 1729}, {40, 68, 1, 1730}, {43, 73, 2, 1730}, {44, 74, 2, 1730}, {47, 79, 1, 1730}, {48, 80, 2, 1730}, {49, 81, 1, 1730}, {40, 69, 1, 1731}, {43, 74, 2, 1731}, {44, 75, 2, 1731}, {47, 80, 1, 1731}, {48, 81, 2, 1731}, {49, 82, 1, 1731}, {40, 70, 1, 1732}, {43, 75, 2, 1732}, {44, 76, 2, 1732}, {47, 81, 1, 1732}, {48, 82, 2, 1732}, {49, 83, 1, 1732}, {41, 56, 1, 1733}, {45, 57, 2, 1733}, {46, 58, 2, 1733}, {50, 59, 1, 1733}, {51, 60, 2, 1733}, {52, 61, 1, 1733}, {41, 57, 1, 1734}, {45, 59, 2, 1734}, {46, 60, 2, 1734}, {50, 62, 1, 1734}, {51, 63, 2, 1734}, {52, 64, 1, 1734}, {41, 58, 1, 1735}, {45, 60, 2, 1735}, {46, 61, 2, 1735}, {50, 63, 1, 1735}, {51, 64, 2, 1735}, {52, 65, 1, 1735}, {41, 59, 1, 1736}, {45, 62, 2, 1736}, {46, 63, 2, 1736}, {50, 66, 1, 1736}, {51, 67, 2, 1736}, {52, 68, 1, 1736}, {41, 60, 1, 1737}, {45, 63, 2, 1737}, {46, 64, 2, 1737}, {50, 67, 1, 1737}, {51, 68, 2, 1737}, {52, 69, 1, 1737}, {41, 61, 1, 1738}, {45, 64, 2, 1738}, {46, 65, 2, 1738}, {50, 68, 1, 1738}, {51, 69, 2, 1738}, {52, 70, 1, 1738}, {41, 62, 1, 1739}, {45, 66, 2, 1739}, {46, 67, 2, 1739}, {50, 71, 1, 1739}, {51, 72, 2, 1739}, {52, 73, 1, 1739}, {41, 63, 1, 1740}, {45, 67, 2, 1740}, {46, 68, 2, 1740}, {50, 72, 1, 1740}, {51, 73, 2, 1740}, {52, 74, 1, 1740}, {41, 64, 1, 1741}, {45, 68, 2, 1741}, {46, 69, 2, 1741}, {50, 73, 1, 1741}, {51, 74, 2, 1741}, {52, 75, 1, 1741}, {41, 65, 1, 1742}, {45, 69, 2, 1742}, {46, 70, 2, 1742}, {50, 74, 1, 1742}, {51, 75, 2, 1742}, {52, 76, 1, 1742}, {41, 66, 1, 1743}, {45, 71, 2, 1743}, {46, 72, 2, 1743}, {50, 77, 1, 1743}, {51, 78, 2, 1743}, {52, 79, 1, 1743}, {41, 67, 1, 1744}, {45, 72, 2, 1744}, {46, 73, 2, 1744}, {50, 78, 1, 1744}, {51, 79, 2, 1744}, {52, 80, 1, 1744}, {41, 68, 1, 1745}, {45, 73, 2, 1745}, {46, 74, 2, 1745}, {50, 79, 1, 1745}, {51, 80, 2, 1745}, {52, 81, 1, 1745}, {41, 69, 1, 1746}, {45, 74, 2, 1746}, {46, 75, 2, 1746}, {50, 80, 1, 1746}, {51, 81, 2, 1746}, {52, 82, 1, 1746}, {41, 70, 1, 1747}, {45, 75, 2, 1747}, {46, 76, 2, 1747}, {50, 81, 1, 1747}, {51, 82, 2, 1747}, {52, 83, 1, 1747}, {42, 56, 1, 1748}, {46, 57, 2, 1748}, {47, 58, 2, 1748}, {51, 59, 1, 1748}, {52, 60, 2, 1748}, {53, 61, 1, 1748}, {42, 57, 1, 1749}, {46, 59, 2, 1749}, {47, 60, 2, 1749}, {51, 62, 1, 1749}, {52, 63, 2, 1749}, {53, 64, 1, 1749}, {42, 58, 1, 1750}, {46, 60, 2, 1750}, {47, 61, 2, 1750}, {51, 63, 1, 1750}, {52, 64, 2, 1750}, {53, 65, 1, 1750}, {42, 59, 1, 1751}, {46, 62, 2, 1751}, {47, 63, 2, 1751}, {51, 66, 1, 1751}, {52, 67, 2, 1751}, {53, 68, 1, 1751}, {42, 60, 1, 1752}, {46, 63, 2, 1752}, {47, 64, 2, 1752}, {51, 67, 1, 1752}, {52, 68, 2, 1752}, {53, 69, 1, 1752}, {42, 61, 1, 1753}, {46, 64, 2, 1753}, {47, 65, 2, 1753}, {51, 68, 1, 1753}, {52, 69, 2, 1753}, {53, 70, 1, 1753}, {42, 62, 1, 1754}, {46, 66, 2, 1754}, {47, 67, 2, 1754}, {51, 71, 1, 1754}, {52, 72, 2, 1754}, {53, 73, 1, 1754}, {42, 63, 1, 1755}, {46, 67, 2, 1755}, {47, 68, 2, 1755}, {51, 72, 1, 1755}, {52, 73, 2, 1755}, {53, 74, 1, 1755}, {42, 64, 1, 1756}, {46, 68, 2, 1756}, {47, 69, 2, 1756}, {51, 73, 1, 1756}, {52, 74, 2, 1756}, {53, 75, 1, 1756}, {42, 65, 1, 1757}, {46, 69, 2, 1757}, {47, 70, 2, 1757}, {51, 74, 1, 1757}, {52, 75, 2, 1757}, {53, 76, 1, 1757}, {42, 66, 1, 1758}, {46, 71, 2, 1758}, {47, 72, 2, 1758}, {51, 77, 1, 1758}, {52, 78, 2, 1758}, {53, 79, 1, 1758}, {42, 67, 1, 1759}, {46, 72, 2, 1759}, {47, 73, 2, 1759}, {51, 78, 1, 1759}, {52, 79, 2, 1759}, {53, 80, 1, 1759}, {42, 68, 1, 1760}, {46, 73, 2, 1760}, {47, 74, 2, 1760}, {51, 79, 1, 1760}, {52, 80, 2, 1760}, {53, 81, 1, 1760}, {42, 69, 1, 1761}, {46, 74, 2, 1761}, {47, 75, 2, 1761}, {51, 80, 1, 1761}, {52, 81, 2, 1761}, {53, 82, 1, 1761}, {42, 70, 1, 1762}, {46, 75, 2, 1762}, {47, 76, 2, 1762}, {51, 81, 1, 1762}, {52, 82, 2, 1762}, {53, 83, 1, 1762}, {43, 56, 1, 1763}, {47, 57, 2, 1763}, {48, 58, 2, 1763}, {52, 59, 1, 1763}, {53, 60, 2, 1763}, {54, 61, 1, 1763}, {43, 57, 1, 1764}, {47, 59, 2, 1764}, {48, 60, 2, 1764}, {52, 62, 1, 1764}, {53, 63, 2, 1764}, {54, 64, 1, 1764}, {43, 58, 1, 1765}, {47, 60, 2, 1765}, {48, 61, 2, 1765}, {52, 63, 1, 1765}, {53, 64, 2, 1765}, {54, 65, 1, 1765}, {43, 59, 1, 1766}, {47, 62, 2, 1766}, {48, 63, 2, 1766}, {52, 66, 1, 1766}, {53, 67, 2, 1766}, {54, 68, 1, 1766}, {43, 60, 1, 1767}, {47, 63, 2, 1767}, {48, 64, 2, 1767}, {52, 67, 1, 1767}, {53, 68, 2, 1767}, {54, 69, 1, 1767}, {43, 61, 1, 1768}, {47, 64, 2, 1768}, {48, 65, 2, 1768}, {52, 68, 1, 1768}, {53, 69, 2, 1768}, {54, 70, 1, 1768}, {43, 62, 1, 1769}, {47, 66, 2, 1769}, {48, 67, 2, 1769}, {52, 71, 1, 1769}, {53, 72, 2, 1769}, {54, 73, 1, 1769}, {43, 63, 1, 1770}, {47, 67, 2, 1770}, {48, 68, 2, 1770}, {52, 72, 1, 1770}, {53, 73, 2, 1770}, {54, 74, 1, 1770}, {43, 64, 1, 1771}, {47, 68, 2, 1771}, {48, 69, 2, 1771}, {52, 73, 1, 1771}, {53, 74, 2, 1771}, {54, 75, 1, 1771}, {43, 65, 1, 1772}, {47, 69, 2, 1772}, {48, 70, 2, 1772}, {52, 74, 1, 1772}, {53, 75, 2, 1772}, {54, 76, 1, 1772}, {43, 66, 1, 1773}, {47, 71, 2, 1773}, {48, 72, 2, 1773}, {52, 77, 1, 1773}, {53, 78, 2, 1773}, {54, 79, 1, 1773}, {43, 67, 1, 1774}, {47, 72, 2, 1774}, {48, 73, 2, 1774}, {52, 78, 1, 1774}, {53, 79, 2, 1774}, {54, 80, 1, 1774}, {43, 68, 1, 1775}, {47, 73, 2, 1775}, {48, 74, 2, 1775}, {52, 79, 1, 1775}, {53, 80, 2, 1775}, {54, 81, 1, 1775}, {43, 69, 1, 1776}, {47, 74, 2, 1776}, {48, 75, 2, 1776}, {52, 80, 1, 1776}, {53, 81, 2, 1776}, {54, 82, 1, 1776}, {43, 70, 1, 1777}, {47, 75, 2, 1777}, {48, 76, 2, 1777}, {52, 81, 1, 1777}, {53, 82, 2, 1777}, {54, 83, 1, 1777}, {44, 56, 1, 1778}, {48, 57, 2, 1778}, {49, 58, 2, 1778}, {53, 59, 1, 1778}, {54, 60, 2, 1778}, {55, 61, 1, 1778}, {44, 57, 1, 1779}, {48, 59, 2, 1779}, {49, 60, 2, 1779}, {53, 62, 1, 1779}, {54, 63, 2, 1779}, {55, 64, 1, 1779}, {44, 58, 1, 1780}, {48, 60, 2, 1780}, {49, 61, 2, 1780}, {53, 63, 1, 1780}, {54, 64, 2, 1780}, {55, 65, 1, 1780}, {44, 59, 1, 1781}, {48, 62, 2, 1781}, {49, 63, 2, 1781}, {53, 66, 1, 1781}, {54, 67, 2, 1781}, {55, 68, 1, 1781}, {44, 60, 1, 1782}, {48, 63, 2, 1782}, {49, 64, 2, 1782}, {53, 67, 1, 1782}, {54, 68, 2, 1782},

{55, 69, 1, 1782}, {44, 61, 1, 1783}, {48, 64, 2, 1783}, {49, 65, 2, 1783}, {53, 68, 1, 1783}, {54, 69, 2, 1783}, {55, 70, 1, 1783}, {44, 62, 1, 1784}, {48, 66, 2, 1784}, {49, 67, 2, 1784}, {53, 71, 1, 1784}, {54, 72, 2, 1784}, {55, 73, 1, 1784}, {44, 63, 1, 1785}, {48, 67, 2, 1785}, {49, 68, 2, 1785}, {53, 72, 1, 1785}, {54, 73, 2, 1785}, {55, 74, 1, 1785}, {44, 64, 1, 1786}, {48, 68, 2, 1786}, {49, 69, 2, 1786}, {53, 73, 1, 1786}, {54, 74, 2, 1786}, {55, 75, 1, 1786}, {44, 65, 1, 1787}, {48, 69, 2, 1787}, {49, 70, 2, 1787}, {53, 74, 1, 1787}, {54, 75, 2, 1787}, {55, 76, 1, 1787}, {44, 66, 1, 1788}, {48, 71, 2, 1788}, {49, 72, 2, 1788}, {53, 77, 1, 1788}, {54, 78, 2, 1788}, {55, 79, 1, 1788}, {44, 67, 1, 1789}, {48, 72, 2, 1789}, {49, 73, 2, 1789}, {53, 78, 1, 1789}, {54, 79, 2, 1789}, {55, 80, 1, 1789}, {44, 68, 1, 1790}, {48, 73, 2, 1790}, {49, 74, 2, 1790}, {53, 79, 1, 1790}, {54, 80, 2, 1790}, {55, 81, 1, 1790}, {44, 69, 1, 1791}, {48, 74, 2, 1791}, {49, 75, 2, 1791}, {53, 80, 1, 1791}, {54, 81, 2, 1791}, {55, 82, 1, 1791}, {44, 70, 1, 1792}, {48, 75, 2, 1792}, {49, 76, 2, 1792}, {53, 81, 1, 1792}, {54, 82, 2, 1792}, {55, 83, 1, 1792}, {35, 35, 1, 1793}, {36, 36, 3, 1793}, {37, 37, 3, 1793}, {38, 38, 3, 1793}, {39, 39, 6, 1793}, {40, 40, 3, 1793}, {41, 41, 1, 1793}, {42, 42, 3, 1793}, {43, 43, 3, 1793}, {44, 44, 1, 1793}, {35, 36, 1, 1794}, {36, 38, 3, 1794}, {37, 39, 3, 1794}, {38, 41, 3, 1794}, {39, 42, 6, 1794}, {40, 43, 3, 1794}, {41, 45, 1, 1794}, {42, 46, 3, 1794}, {43, 47, 3, 1794}, {44, 48, 1, 1794}, {35, 37, 1, 1795}, {36, 39, 3, 1795}, {37, 40, 3, 1795}, {38, 42, 3, 1795}, {39, 43, 6, 1795}, {40, 44, 3, 1795}, {41, 46, 1, 1795}, {42, 47, 3, 1795}, {43, 48, 3, 1795}, {44, 49, 1, 1795}, {35, 38, 1, 1796}, {36, 41, 3, 1796}, {37, 42, 3, 1796}, {38, 45, 3, 1796}, {39, 46, 6, 1796}, {40, 47, 3, 1796}, {41, 50, 1, 1796}, {42, 51, 3, 1796}, {43, 52, 3, 1796}, {44, 53, 1, 1796}, {35, 39, 1, 1797}, {36, 42, 3, 1797}, {37, 43, 3, 1797}, {38, 46, 3, 1797}, {39, 47, 6, 1797}, {40, 48, 3, 1797}, {41, 51, 1, 1797}, {42, 52, 3, 1797}, {43, 53, 3, 1797}, {44, 54, 1, 1797}, {35, 40, 1, 1798}, {36, 43, 3, 1798}, {37, 44, 3, 1798}, {38, 47, 3, 1798}, {39, 48, 6, 1798}, {40, 49, 3, 1798}, {41, 52, 1, 1798}, {42, 53, 3, 1798}, {43, 54, 3, 1798}, {44, 55, 1, 1798}, {36, 36, 1, 1799}, {38, 38, 3, 1799}, {39, 39, 3, 1799}, {41, 41, 3, 1799}, {42, 42, 6, 1799}, {43, 43, 3, 1799}, {45, 45, 1, 1799}, {46, 46, 3, 1799}, {47, 47, 3, 1799}, {48, 48, 1, 1799}, {36, 37, 1, 1800}, {38, 39, 3, 1800}, {39, 40, 3, 1800}, {41, 42, 3, 1800}, {42, 43, 6, 1800}, {43, 44, 3, 1800}, {45, 46, 1, 1800}, {46, 47, 3, 1800}, {47, 48, 3, 1800}, {48, 49, 1, 1800}, {36, 38, 1, 1801}, {38, 41, 3, 1801}, {39, 42, 3, 1801}, {41, 45, 3, 1801}, {42, 46, 6, 1801}, {43, 47, 3, 1801}, {45, 50, 1, 1801}, {46, 51, 3, 1801}, {47, 52, 3, 1801}, {48, 53, 1, 1801}, {36, 39, 1, 1802}, {38, 42, 3, 1802}, {39, 43, 3, 1802}, {41, 46, 3, 1802}, {42, 47, 6, 1802}, {43, 48, 3, 1802}, {45, 51, 1, 1802}, {46, 52, 3, 1802}, {47, 53, 3, 1802}, {48, 54, 1, 1802}, {36, 40, 1, 1803}, {38, 43, 3, 1803}, {39, 44, 3, 1803}, {41, 47, 3, 1803}, {42, 48, 6, 1803}, {43, 49, 3, 1803}, {45, 52, 1, 1803}, {46, 53, 3, 1803}, {47, 54, 3, 1803}, {48, 55, 1, 1803}, {37, 37, 1, 1804}, {39, 39, 3, 1804}, {40, 40, 3, 1804}, {42, 42, 3, 1804}, {43, 43, 6, 1804}, {44, 44, 3, 1804}, {46, 46, 1, 1804}, {47, 47, 3, 1804}, {48, 48, 3, 1804}, {49, 49, 1, 1804}, {37, 38, 1, 1805}, {39, 41, 3, 1805}, {40, 42, 3, 1805}, {42, 45, 3, 1805}, {43, 46, 6, 1805}, {44, 47, 3, 1805}, {46, 50, 1, 1805}, {47, 51, 3, 1805}, {48, 52, 3, 1805}, {49, 53, 1, 1805}, {37, 39, 1, 1806}, {39, 42, 3, 1806}, {40, 43, 3, 1806}, {42, 46, 3, 1806}, {43, 47, 6, 1806}, {44, 48, 3, 1806}, {46, 51, 1, 1806}, {47, 52, 3, 1806}, {48, 53, 3, 1806}, {49, 54, 1, 1806}, {37, 40, 1, 1807}, {39, 43, 3, 1807}, {40, 44, 3, 1807}, {42, 47, 3, 1807}, {43, 48, 6, 1807}, {44, 49, 3, 1807}, {46, 52, 1, 1807}, {47, 53, 3, 1807}, {48, 54, 3, 1807}, {49, 55, 1, 1807}, {38, 38, 1, 1808}, {41, 41, 3, 1808}, {42, 42, 3, 1808}, {45, 45, 3, 1808}, {46, 46, 6, 1808}, {47, 47, 3, 1808}, {50, 50, 1, 1808}, {51, 51, 3, 1808}, {52, 52, 3, 1808}, {53, 53, 1, 1808}, {38, 39, 1, 1809}, {41, 42, 3, 1809}, {42, 43, 3, 1809}, {45, 46, 3, 1809}, {46, 47, 6, 1809}, {47, 48, 3, 1809}, {50, 51, 1, 1809}, {51, 52, 3, 1809}, {52, 53, 3, 1809}, {53, 54, 1, 1809}, {38, 40, 1, 1810}, {41, 43, 3, 1810}, {42, 44, 3, 1810}, {45, 47, 3, 1810}, {46, 48, 6, 1810}, {47, 49, 3, 1810}, {50, 52, 1, 1810}, {51, 53, 3, 1810}, {52, 54, 3, 1810}, {53, 55, 1, 1810}, {39, 39, 1, 1811}, {42, 42, 3, 1811}, {43, 43, 3, 1811}, {46, 46, 3, 1811}, {47, 47, 6, 1811}, {48, 48, 3, 1811}, {51, 51, 1, 1811}, {52, 52, 3, 1811}, {53, 53, 3, 1811}, {54, 54, 1, 1811}, {39, 40, 1, 1812}, {42, 43, 3, 1812}, {43, 44, 3, 1812}, {46, 47, 3, 1812}, {47, 48, 6, 1812}, {48, 49, 3, 1812}, {51, 52, 1, 1812}, {52, 53, 3, 1812}, {53, 54, 3, 1812}, {54, 55, 1, 1812}, {40, 40, 1, 1813}, {43, 43, 3, 1813}, {44, 44, 3, 1813}, {47, 47, 3, 1813}, {48, 48, 6, 1813}, {49, 49, 3, 1813}, {52, 52, 1, 1813}, {53, 53, 3, 1813}, {54, 54, 3, 1813}, {55, 55, 1, 1813}, {35, 56, 1, 1814}, {36, 57, 3, 1814}, {37, 58, 3, 1814}, {38, 59, 3, 1814}, {39, 60, 6, 1814}, {40, 61, 3, 1814}, {41, 62, 1, 1814}, {42, 63, 3, 1814}, {43, 64, 3, 1814}, {44, 65, 1, 1814}, {35, 57, 1, 1815}, {36, 59, 3, 1815}, {37, 60, 3, 1815}, {38, 62, 3, 1815}, {39, 63, 6, 1815}, {40, 64, 3, 1815}, {41, 66, 1, 1815}, {42, 67, 3, 1815}, {43, 68, 3, 1815}, {44, 69, 1, 1815}, {35, 58, 1, 1816}, {36, 60, 3, 1816}, {37, 61, 3, 1816}, {38, 63, 3, 1816}, {39, 64, 6, 1816}, {40, 65, 3, 1816}, {41, 67, 1, 1816}, {42, 68, 3, 1816}, {43, 69, 3, 1816}, {44, 70, 1, 1816}, {35, 59, 1, 1817}, {36, 62, 3, 1817}, {37, 63, 3, 1817}, {38, 66, 3, 1817}, {39, 67, 6, 1817}, {40, 68, 3, 1817}, {41, 71, 1, 1817}, {42, 72, 3, 1817}, {43, 73, 3, 1817}, {44, 74, 1, 1817}, {35, 60, 1, 1818}, {36, 63, 3, 1818}, {37, 64, 3, 1818}, {38, 67, 3, 1818}, {39, 68, 6, 1818}, {40, 69, 3, 1818}, {41, 72, 1, 1818}, {42, 73, 3, 1818}, {43, 74, 3, 1818}, {44, 75, 1, 1818}, {35, 61, 1, 1819},

{36, 64, 3, 1819}, {37, 65, 3, 1819}, {38, 68, 3, 1819}, {39, 69, 6, 1819}, {40, 70, 3,
1819}, {41, 73, 1, 1819}, {42, 74, 3, 1819}, {43, 75, 3, 1819}, {44, 76, 1, 1819}, {35,
62, 1, 1820}, {36, 66, 3, 1820}, {37, 67, 3, 1820}, {38, 71, 3, 1820}, {39, 72, 6, 1820},
{40, 73, 3, 1820}, {41, 77, 1, 1820}, {42, 78, 3, 1820}, {43, 79, 3, 1820}, {44, 80, 1,
1820}, {35, 63, 1, 1821}, {36, 67, 3, 1821}, {37, 68, 3, 1821}, {38, 72, 3, 1821}, {39,
73, 6, 1821}, {40, 74, 3, 1821}, {41, 78, 1, 1821}, {42, 79, 3, 1821}, {43, 80, 3, 1821},
{44, 81, 1, 1821}, {35, 64, 1, 1822}, {36, 68, 3, 1822}, {37, 69, 3, 1822}, {38, 73, 3,
1822}, {39, 74, 6, 1822}, {40, 75, 3, 1822}, {41, 79, 1, 1822}, {42, 80, 3, 1822}, {43,
81, 3, 1822}, {44, 82, 1, 1822}, {35, 65, 1, 1823}, {36, 69, 3, 1823}, {37, 70, 3, 1823},
{38, 74, 3, 1823}, {39, 75, 6, 1823}, {40, 76, 3, 1823}, {41, 80, 1, 1823}, {42, 81, 3,
1823}, {43, 82, 3, 1823}, {44, 83, 1, 1823}, {36, 56, 1, 1824}, {38, 57, 3, 1824}, {39,
58, 3, 1824}, {41, 59, 3, 1824}, {42, 60, 6, 1824}, {43, 61, 3, 1824}, {45, 62, 1, 1824},
{46, 63, 3, 1824}, {47, 64, 3, 1824}, {48, 65, 1, 1824}, {36, 57, 1, 1825}, {38, 59, 3,
1825}, {39, 60, 3, 1825}, {41, 62, 3, 1825}, {42, 63, 6, 1825}, {43, 64, 3, 1825}, {45,
66, 1, 1825}, {46, 67, 3, 1825}, {47, 68, 3, 1825}, {48, 69, 1, 1825}, {36, 58, 1, 1826},
{38, 60, 3, 1826}, {39, 61, 3, 1826}, {41, 63, 3, 1826}, {42, 64, 6, 1826}, {43, 65, 3,
1826}, {45, 67, 1, 1826}, {46, 68, 3, 1826}, {47, 69, 3, 1826}, {48, 70, 1, 1826}, {36,
59, 1, 1827}, {38, 62, 3, 1827}, {39, 63, 3, 1827}, {41, 66, 3, 1827}, {42, 67, 6, 1827},
{43, 68, 3, 1827}, {45, 71, 1, 1827}, {46, 72, 3, 1827}, {47, 73, 3, 1827}, {48, 74, 1,
1827}, {36, 60, 1, 1828}, {38, 63, 3, 1828}, {39, 64, 3, 1828}, {41, 67, 3, 1828}, {42,
68, 6, 1828}, {43, 69, 3, 1828}, {45, 72, 1, 1828}, {46, 73, 3, 1828}, {47, 74, 3, 1828},
{48, 75, 1, 1828}, {36, 61, 1, 1829}, {38, 64, 3, 1829}, {39, 65, 3, 1829}, {41, 68, 3,
1829}, {42, 69, 6, 1829}, {43, 70, 3, 1829}, {45, 73, 1, 1829}, {46, 74, 3, 1829}, {47,
75, 3, 1829}, {48, 76, 1, 1829}, {36, 62, 1, 1830}, {38, 66, 3, 1830}, {39, 67, 3, 1830},
{41, 71, 3, 1830}, {42, 72, 6, 1830}, {43, 73, 3, 1830}, {45, 77, 1, 1830}, {46, 78, 3,
1830}, {47, 79, 3, 1830}, {48, 80, 1, 1830}, {36, 63, 1, 1831}, {38, 67, 3, 1831}, {39,
68, 3, 1831}, {41, 72, 3, 1831}, {42, 73, 6, 1831}, {43, 74, 3, 1831}, {45, 78, 1, 1831},
{46, 79, 3, 1831}, {47, 80, 3, 1831}, {48, 81, 1, 1831}, {36, 64, 1, 1832}, {38, 68, 3,
1832}, {39, 69, 3, 1832}, {41, 73, 3, 1832}, {42, 74, 6, 1832}, {43, 75, 3, 1832}, {45,
79, 1, 1832}, {46, 80, 3, 1832}, {47, 81, 3, 1832}, {48, 82, 1, 1832}, {36, 65, 1, 1833},
{38, 69, 3, 1833}, {39, 70, 3, 1833}, {41, 74, 3, 1833}, {42, 75, 6, 1833}, {43, 76, 3,
1833}, {45, 80, 1, 1833}, {46, 81, 3, 1833}, {47, 82, 3, 1833}, {48, 83, 1, 1833}, {37,
56, 1, 1834}, {39, 57, 3, 1834}, {40, 58, 3, 1834}, {42, 59, 3, 1834}, {43, 60, 6, 1834},
{44, 61, 3, 1834}, {46, 62, 1, 1834}, {47, 63, 3, 1834}, {48, 64, 3, 1834}, {49, 65, 1,
1834}, {37, 57, 1, 1835}, {39, 59, 3, 1835}, {40, 60, 3, 1835}, {42, 62, 3, 1835}, {43,
63, 6, 1835}, {44, 64, 3, 1835}, {46, 66, 1, 1835}, {47, 67, 3, 1835}, {48, 68, 3, 1835},
{49, 69, 1, 1835}, {37, 58, 1, 1836}, {39, 60, 3, 1836}, {40, 61, 3, 1836}, {42, 63, 3,
1836}, {43, 64, 6, 1836}, {44, 65, 3, 1836}, {46, 67, 1, 1836}, {47, 68, 3, 1836}, {48,
69, 3, 1836}, {49, 70, 1, 1836}, {37, 59, 1, 1837}, {39, 62, 3, 1837}, {40, 63, 3, 1837},
{42, 66, 3, 1837}, {43, 67, 6, 1837}, {44, 68, 3, 1837}, {46, 71, 1, 1837}, {47, 72, 3,
1837}, {48, 73, 3, 1837}, {49, 74, 1, 1837}, {37, 60, 1, 1838}, {39, 63, 3, 1838}, {40,
64, 3, 1838}, {42, 67, 3, 1838}, {43, 68, 6, 1838}, {44, 69, 3, 1838}, {46, 72, 1, 1838},
{47, 73, 3, 1838}, {48, 74, 3, 1838}, {49, 75, 1, 1838}, {37, 61, 1, 1839}, {39, 64, 3,
1839}, {40, 65, 3, 1839}, {42, 68, 3, 1839}, {43, 69, 6, 1839}, {44, 70, 3, 1839}, {46,
73, 1, 1839}, {47, 74, 3, 1839}, {48, 75, 3, 1839}, {49, 76, 1, 1839}, {37, 62, 1, 1840},
{39, 66, 3, 1840}, {40, 67, 3, 1840}, {42, 71, 3, 1840}, {43, 72, 6, 1840}, {44, 73, 3,
1840}, {46, 77, 1, 1840}, {47, 78, 3, 1840}, {48, 79, 3, 1840}, {49, 80, 1, 1840}, {37,
63, 1, 1841}, {39, 67, 3, 1841}, {40, 68, 3, 1841}, {42, 72, 3, 1841}, {43, 73, 6, 1841},
{44, 74, 3, 1841}, {46, 78, 1, 1841}, {47, 79, 3, 1841}, {48, 80, 3, 1841}, {49, 81, 1,
1841}, {37, 64, 1, 1842}, {39, 68, 3, 1842}, {40, 69, 3, 1842}, {42, 73, 3, 1842}, {43,
74, 6, 1842}, {44, 75, 3, 1842}, {46, 79, 1, 1842}, {47, 80, 3, 1842}, {48, 81, 3, 1842},
{49, 82, 1, 1842}, {37, 65, 1, 1843}, {39, 69, 3, 1843}, {40, 70, 3, 1843}, {42, 74, 3,
1843}, {43, 75, 6, 1843}, {44, 76, 3, 1843}, {46, 80, 1, 1843}, {47, 81, 3, 1843}, {48,
82, 3, 1843}, {49, 83, 1, 1843}, {38, 56, 1, 1844}, {41, 57, 3, 1844}, {42, 58, 3, 1844},
{45, 59, 3, 1844}, {46, 60, 6, 1844}, {47, 61, 3, 1844}, {50, 62, 1, 1844}, {51, 63, 3,
1844}, {52, 64, 3, 1844}, {53, 65, 1, 1844}, {38, 57, 1, 1845}, {41, 59, 3, 1845}, {42,
60, 3, 1845}, {45, 62, 3, 1845}, {46, 63, 6, 1845}, {47, 64, 3, 1845}, {50, 66, 1, 1845},
{51, 67, 3, 1845}, {52, 68, 3, 1845}, {53, 69, 1, 1845}, {38, 58, 1, 1846}, {41, 60, 3,
1846}, {42, 61, 3, 1846}, {45, 63, 3, 1846}, {46, 64, 6, 1846}, {47, 65, 3, 1846}, {50,
67, 1, 1846}, {51, 68, 3, 1846}, {52, 69, 3, 1846}, {53, 70, 1, 1846}, {38, 59, 1, 1847},
{41, 62, 3, 1847}, {42, 63, 3, 1847}, {45, 66, 3, 1847}, {46, 67, 6, 1847}, {47, 68, 3,
1847}, {50, 71, 1, 1847}, {51, 72, 3, 1847}, {52, 73, 3, 1847}, {53, 74, 1, 1847}, {38,
60, 1, 1848}, {41, 63, 3, 1848}, {42, 64, 3, 1848}, {45, 67, 3, 1848}, {46, 68, 6, 1848},
{47, 69, 3, 1848}, {50, 72, 1, 1848}, {51, 73, 3, 1848}, {52, 74, 3, 1848}, {53, 75, 1,
1848}, {38, 61, 1, 1849}, {41, 64, 3, 1849}, {42, 65, 3, 1849}, {45, 68, 3, 1849}, {46,
69, 6, 1849}, {47, 70, 3, 1849}, {50, 73, 1, 1849}, {51, 74, 3, 1849}, {52, 75, 3, 1849},
{53, 76, 1, 1849}, {38, 62, 1, 1850}, {41, 66, 3, 1850}, {42, 67, 3, 1850}, {45, 71, 3,
1850}, {46, 72, 6, 1850}, {47, 73, 3, 1850}, {50, 77, 1, 1850}, {51, 78, 3, 1850}, {52,
79, 3, 1850}, {53, 80, 1, 1850}, {38, 63, 1, 1851}, {41, 67, 3, 1851}, {42, 68, 3, 1851},

{45, 72, 3, 1851}, {46, 73, 6, 1851}, {47, 74, 3, 1851}, {50, 78, 1, 1851}, {51, 79, 3, 1851}, {52, 80, 3, 1851}, {53, 81, 1, 1851}, {38, 64, 1, 1852}, {41, 68, 3, 1852}, {42, 69, 3, 1852}, {45, 73, 3, 1852}, {46, 74, 6, 1852}, {47, 75, 3, 1852}, {50, 79, 1, 1852}, {51, 80, 3, 1852}, {52, 81, 3, 1852}, {53, 82, 1, 1852}, {38, 65, 1, 1853}, {41, 69, 3, 1853}, {42, 70, 3, 1853}, {45, 74, 3, 1853}, {46, 75, 6, 1853}, {47, 76, 3, 1853}, {50, 80, 1, 1853}, {51, 81, 3, 1853}, {52, 82, 3, 1853}, {53, 83, 1, 1853}, {39, 56, 1, 1854}, {42, 57, 3, 1854}, {43, 58, 3, 1854}, {46, 59, 3, 1854}, {47, 60, 6, 1854}, {48, 61, 3, 1854}, {51, 62, 1, 1854}, {52, 63, 3, 1854}, {53, 64, 3, 1854}, {54, 65, 1, 1854}, {39, 57, 1, 1855}, {42, 59, 3, 1855}, {43, 60, 3, 1855}, {46, 62, 3, 1855}, {47, 63, 6, 1855}, {48, 64, 3, 1855}, {51, 66, 1, 1855}, {52, 67, 3, 1855}, {53, 68, 3, 1855}, {54, 69, 1, 1855}, {39, 58, 1, 1856}, {42, 60, 3, 1856}, {43, 61, 3, 1856}, {46, 63, 3, 1856}, {47, 64, 6, 1856}, {48, 65, 3, 1856}, {51, 67, 1, 1856}, {52, 68, 3, 1856}, {53, 69, 3, 1856}, {54, 70, 1, 1856}, {39, 59, 1, 1857}, {42, 62, 3, 1857}, {43, 63, 3, 1857}, {46, 66, 3, 1857}, {47, 67, 6, 1857}, {48, 68, 3, 1857}, {51, 71, 1, 1857}, {52, 72, 3, 1857}, {53, 73, 3, 1857}, {54, 74, 1, 1857}, {39, 60, 1, 1858}, {42, 63, 3, 1858}, {43, 64, 3, 1858}, {46, 67, 3, 1858}, {47, 68, 6, 1858}, {48, 69, 3, 1858}, {51, 72, 1, 1858}, {52, 73, 3, 1858}, {53, 74, 3, 1858}, {54, 75, 1, 1858}, {39, 61, 1, 1859}, {42, 64, 3, 1859}, {43, 65, 3, 1859}, {46, 68, 3, 1859}, {47, 69, 6, 1859}, {48, 70, 3, 1859}, {51, 73, 1, 1859}, {52, 74, 3, 1859}, {53, 75, 3, 1859}, {54, 76, 1, 1859}, {39, 62, 1, 1860}, {42, 66, 3, 1860}, {43, 67, 3, 1860}, {46, 71, 3, 1860}, {47, 72, 6, 1860}, {48, 73, 3, 1860}, {51, 77, 1, 1860}, {52, 78, 3, 1860}, {53, 79, 3, 1860}, {54, 80, 1, 1860}, {39, 63, 1, 1861}, {42, 67, 3, 1861}, {43, 68, 3, 1861}, {46, 72, 3, 1861}, {47, 73, 6, 1861}, {48, 74, 3, 1861}, {51, 78, 1, 1861}, {52, 79, 3, 1861}, {53, 80, 3, 1861}, {54, 81, 1, 1861}, {39, 64, 1, 1862}, {42, 68, 3, 1862}, {43, 69, 3, 1862}, {46, 73, 3, 1862}, {47, 74, 6, 1862}, {48, 75, 3, 1862}, {51, 79, 1, 1862}, {52, 80, 3, 1862}, {53, 81, 3, 1862}, {54, 82, 1, 1862}, {39, 65, 1, 1863}, {42, 69, 3, 1863}, {43, 70, 3, 1863}, {46, 74, 3, 1863}, {47, 75, 6, 1863}, {48, 76, 3, 1863}, {51, 80, 1, 1863}, {52, 81, 3, 1863}, {53, 82, 3, 1863}, {54, 83, 1, 1863}, {40, 56, 1, 1864}, {43, 57, 3, 1864}, {44, 58, 3, 1864}, {47, 59, 3, 1864}, {48, 60, 6, 1864}, {49, 61, 3, 1864}, {52, 62, 1, 1864}, {53, 63, 3, 1864}, {54, 64, 3, 1864}, {55, 65, 1, 1864}, {40, 57, 1, 1865}, {43, 59, 3, 1865}, {44, 60, 3, 1865}, {47, 62, 3, 1865}, {48, 63, 6, 1865}, {49, 64, 3, 1865}, {52, 66, 1, 1865}, {53, 67, 3, 1865}, {54, 68, 3, 1865}, {55, 69, 1, 1865}, {40, 58, 1, 1866}, {43, 60, 3, 1866}, {44, 61, 3, 1866}, {47, 63, 3, 1866}, {48, 64, 6, 1866}, {49, 65, 3, 1866}, {52, 67, 1, 1866}, {53, 68, 3, 1866}, {54, 69, 3, 1866}, {55, 70, 1, 1866}, {40, 59, 1, 1867}, {43, 62, 3, 1867}, {44, 63, 3, 1867}, {47, 66, 3, 1867}, {48, 67, 6, 1867}, {49, 68, 3, 1867}, {52, 71, 1, 1867}, {53, 72, 3, 1867}, {54, 73, 3, 1867}, {55, 74, 1, 1867}, {40, 60, 1, 1868}, {43, 63, 3, 1868}, {44, 64, 3, 1868}, {47, 67, 3, 1868}, {48, 68, 6, 1868}, {49, 69, 3, 1868}, {52, 72, 1, 1868}, {53, 73, 3, 1868}, {54, 74, 3, 1868}, {55, 75, 1, 1868}, {40, 61, 1, 1869}, {43, 64, 3, 1869}, {44, 65, 3, 1869}, {47, 68, 3, 1869}, {48, 69, 6, 1869}, {49, 70, 3, 1869}, {52, 73, 1, 1869}, {53, 74, 3, 1869}, {54, 75, 3, 1869}, {55, 76, 1, 1869}, {40, 62, 1, 1870}, {43, 66, 3, 1870}, {44, 67, 3, 1870}, {47, 71, 3, 1870}, {48, 72, 6, 1870}, {49, 73, 3, 1870}, {52, 77, 1, 1870}, {53, 78, 3, 1870}, {54, 79, 3, 1870}, {55, 80, 1, 1870}, {40, 63, 1, 1871}, {43, 67, 3, 1871}, {44, 68, 3, 1871}, {47, 72, 3, 1871}, {48, 73, 6, 1871}, {49, 74, 3, 1871}, {52, 78, 1, 1871}, {53, 79, 3, 1871}, {54, 80, 3, 1871}, {55, 81, 1, 1871}, {40, 64, 1, 1872}, {43, 68, 3, 1872}, {44, 69, 3, 1872}, {47, 73, 3, 1872}, {48, 74, 6, 1872}, {49, 75, 3, 1872}, {52, 79, 1, 1872}, {53, 80, 3, 1872}, {54, 81, 3, 1872}, {55, 82, 1, 1872}, {40, 65, 1, 1873}, {43, 69, 3, 1873}, {44, 70, 3, 1873}, {47, 74, 3, 1873}, {48, 75, 6, 1873}, {49, 76, 3, 1873}, {52, 80, 1, 1873}, {53, 81, 3, 1873}, {54, 82, 3, 1873}, {55, 83, 1, 1873}, {35, 84, 1, 1874}, {36, 85, 3, 1874}, {37, 86, 3, 1874}, {38, 87, 3, 1874}, {39, 88, 6, 1874}, {40, 89, 3, 1874}, {41, 90, 1, 1874}, {42, 91, 3, 1874}, {43, 92, 3, 1874}, {44, 93, 1, 1874}, {35, 85, 1, 1875}, {36, 87, 3, 1875}, {37, 88, 3, 1875}, {38, 90, 3, 1875}, {39, 91, 6, 1875}, {40, 92, 3, 1875}, {41, 94, 1, 1875}, {42, 95, 3, 1875}, {43, 96, 3, 1875}, {44, 97, 1, 1875}, {35, 86, 1, 1876}, {36, 88, 3, 1876}, {37, 89, 3, 1876}, {38, 91, 3, 1876}, {39, 92, 6, 1876}, {40, 93, 3, 1876}, {41, 95, 1, 1876}, {42, 96, 3, 1876}, {43, 97, 3, 1876}, {44, 98, 1, 1876}, {35, 87, 1, 1877}, {36, 90, 3, 1877}, {37, 91, 3, 1877}, {38, 94, 3, 1877}, {39, 95, 6, 1877}, {40, 96, 3, 1877}, {41, 99, 1, 1877}, {42, 100, 3, 1877}, {43, 101, 3, 1877}, {44, 102, 1, 1877}, {35, 88, 1, 1878}, {36, 91, 3, 1878}, {37, 92, 3, 1878}, {38, 95, 3, 1878}, {39, 96, 6, 1878}, {40, 97, 3, 1878}, {41, 100, 1, 1878}, {42, 101, 3, 1878}, {43, 102, 3, 1878}, {44, 103, 1, 1878}, {35, 89, 1, 1879}, {36, 92, 3, 1879}, {37, 93, 3, 1879}, {38, 96, 3, 1879}, {39, 97, 6, 1879}, {40, 98, 3, 1879}, {41, 101, 1, 1879}, {42, 102, 3, 1879}, {43, 103, 3, 1879}, {44, 104, 1, 1879}, {35, 90, 1, 1880}, {36, 94, 3, 1880}, {37, 95, 3, 1880}, {38, 99, 3, 1880}, {39, 100, 6, 1880}, {40, 101, 3, 1880}, {41, 105, 1, 1880}, {42, 106, 3, 1880}, {43, 107, 3, 1880}, {44, 108, 1, 1880}, {35, 91, 1, 1881}, {36, 95, 3, 1881}, {37, 96, 3, 1881}, {38, 100, 3, 1881}, {39, 101, 6, 1881}, {40, 102, 3, 1881}, {41, 106, 1, 1881}, {42, 107, 3, 1881}, {43, 108, 3, 1881}, {44, 109, 1, 1881}, {35, 92, 1, 1882}, {36, 96, 3, 1882}, {37, 97, 3, 1882}, {38, 101, 3, 1882}, {39, 102, 6, 1882}, {40, 103, 3, 1882}, {41, 107, 1, 1882}, {42, 108, 3, 1882}, {43, 109, 3, 1882}, {44, 110, 1, 1882}, {35, 93, 1, 1883}, {36, 97, 3, 1883}, {37, 98, 3, 1883}, {38,

102, 3, 1883}, {39, 103, 6, 1883}, {40, 104, 3, 1883}, {41, 108, 1, 1883}, {42, 109, 3,
1883}, {43, 110, 3, 1883}, {44, 111, 1, 1883}, {35, 94, 1, 1884}, {36, 99, 3, 1884}, {37,
100, 3, 1884}, {38, 105, 3, 1884}, {39, 106, 6, 1884}, {40, 107, 3, 1884}, {41, 112, 1,
1884}, {42, 113, 3, 1884}, {43, 114, 3, 1884}, {44, 115, 1, 1884}, {35, 95, 1, 1885}, {36,
100, 3, 1885}, {37, 101, 3, 1885}, {38, 106, 3, 1885}, {39, 107, 6, 1885}, {40, 108, 3,
1885}, {41, 113, 1, 1885}, {42, 114, 3, 1885}, {43, 115, 3, 1885}, {44, 116, 1, 1885},
{35, 96, 1, 1886}, {36, 101, 3, 1886}, {37, 102, 3, 1886}, {38, 107, 3, 1886}, {39, 108,
6, 1886}, {40, 109, 3, 1886}, {41, 114, 1, 1886}, {42, 115, 3, 1886}, {43, 116, 3, 1886},
{44, 117, 1, 1886}, {35, 97, 1, 1887}, {36, 102, 3, 1887}, {37, 103, 3, 1887}, {38, 108,
3, 1887}, {39, 109, 6, 1887}, {40, 110, 3, 1887}, {41, 115, 1, 1887}, {42, 116, 3, 1887},
{43, 117, 3, 1887}, {44, 118, 1, 1887}, {35, 98, 1, 1888}, {36, 103, 3, 1888}, {37, 104,
3, 1888}, {38, 109, 3, 1888}, {39, 110, 6, 1888}, {40, 111, 3, 1888}, {41, 116, 1, 1888},
{42, 117, 3, 1888}, {43, 118, 3, 1888}, {44, 119, 1, 1888}, {36, 84, 1, 1889}, {38, 85, 3,
1889}, {39, 86, 3, 1889}, {41, 87, 3, 1889}, {42, 88, 6, 1889}, {43, 89, 3, 1889}, {45,
90, 1, 1889}, {46, 91, 3, 1889}, {47, 92, 3, 1889}, {48, 93, 1, 1889}, {36, 85, 1, 1890},
{38, 87, 3, 1890}, {39, 88, 3, 1890}, {41, 90, 3, 1890}, {42, 91, 6, 1890}, {43, 92, 3,
1890}, {45, 94, 1, 1890}, {46, 95, 3, 1890}, {47, 96, 3, 1890}, {48, 97, 1, 1890}, {36,
86, 1, 1891}, {38, 88, 3, 1891}, {39, 89, 3, 1891}, {41, 91, 3, 1891}, {42, 92, 6, 1891},
{43, 93, 3, 1891}, {45, 95, 1, 1891}, {46, 96, 3, 1891}, {47, 97, 3, 1891}, {48, 98, 1,
1891}, {36, 87, 1, 1892}, {38, 90, 3, 1892}, {39, 91, 3, 1892}, {41, 94, 3, 1892}, {42,
95, 6, 1892}, {43, 96, 3, 1892}, {45, 99, 1, 1892}, {46, 100, 3, 1892}, {47, 101, 3,
1892}, {48, 102, 1, 1892}, {36, 88, 1, 1893}, {38, 91, 3, 1893}, {39, 92, 3, 1893}, {41,
95, 3, 1893}, {42, 96, 6, 1893}, {43, 97, 3, 1893}, {45, 100, 1, 1893}, {46, 101, 3,
1893}, {47, 102, 3, 1893}, {48, 103, 1, 1893}, {36, 89, 1, 1894}, {38, 92, 3, 1894}, {39,
93, 3, 1894}, {41, 96, 3, 1894}, {42, 97, 6, 1894}, {43, 98, 3, 1894}, {45, 101, 1, 1894},
{46, 102, 3, 1894}, {47, 103, 3, 1894}, {48, 104, 1, 1894}, {36, 90, 1, 1895}, {38, 94, 3,
1895}, {39, 95, 3, 1895}, {41, 99, 3, 1895}, {42, 100, 6, 1895}, {43, 101, 3, 1895}, {45,
105, 1, 1895}, {46, 106, 3, 1895}, {47, 107, 3, 1895}, {48, 108, 1, 1895}, {36, 91, 1,
1896}, {38, 95, 3, 1896}, {39, 96, 3, 1896}, {41, 100, 3, 1896}, {42, 101, 6, 1896}, {43,
102, 3, 1896}, {45, 106, 1, 1896}, {46, 107, 3, 1896}, {47, 108, 3, 1896}, {48, 109, 1,
1896}, {36, 92, 1, 1897}, {38, 96, 3, 1897}, {39, 97, 3, 1897}, {41, 101, 3, 1897}, {42,
102, 6, 1897}, {43, 103, 3, 1897}, {45, 107, 1, 1897}, {46, 108, 3, 1897}, {47, 109, 3,
1897}, {48, 110, 1, 1897}, {36, 93, 1, 1898}, {38, 97, 3, 1898}, {39, 98, 3, 1898}, {41,
102, 3, 1898}, {42, 103, 6, 1898}, {43, 104, 3, 1898}, {45, 108, 1, 1898}, {46, 109, 3,
1898}, {47, 110, 3, 1898}, {48, 111, 1, 1898}, {36, 94, 1, 1899}, {38, 99, 3, 1899}, {39,
100, 3, 1899}, {41, 105, 3, 1899}, {42, 106, 6, 1899}, {43, 107, 3, 1899}, {45, 112, 1,
1899}, {46, 113, 3, 1899}, {47, 114, 3, 1899}, {48, 115, 1, 1899}, {36, 95, 1, 1900}, {38,
100, 3, 1900}, {39, 101, 3, 1900}, {41, 106, 3, 1900}, {42, 107, 6, 1900}, {43, 108, 3,
1900}, {45, 113, 1, 1900}, {46, 114, 3, 1900}, {47, 115, 3, 1900}, {48, 116, 1, 1900},
{36, 96, 1, 1901}, {38, 101, 3, 1901}, {39, 102, 3, 1901}, {41, 107, 3, 1901}, {42, 108,
6, 1901}, {43, 109, 3, 1901}, {45, 114, 1, 1901}, {46, 115, 3, 1901}, {47, 116, 3, 1901},
{48, 117, 1, 1901}, {36, 97, 1, 1902}, {38, 102, 3, 1902}, {39, 103, 3, 1902}, {41, 108,
3, 1902}, {42, 109, 6, 1902}, {43, 110, 3, 1902}, {45, 115, 1, 1902}, {46, 116, 3, 1902},
{47, 117, 3, 1902}, {48, 118, 1, 1902}, {36, 98, 1, 1903}, {38, 103, 3, 1903}, {39, 104,
3, 1903}, {41, 109, 3, 1903}, {42, 110, 6, 1903}, {43, 111, 3, 1903}, {45, 116, 1, 1903},
{46, 117, 3, 1903}, {47, 118, 3, 1903}, {48, 119, 1, 1903}, {37, 84, 1, 1904}, {39, 85, 3,
1904}, {40, 86, 3, 1904}, {42, 87, 3, 1904}, {43, 88, 6, 1904}, {44, 89, 3, 1904}, {46,
90, 1, 1904}, {47, 91, 3, 1904}, {48, 92, 3, 1904}, {49, 93, 1, 1904}, {37, 85, 1, 1905},
{39, 87, 3, 1905}, {40, 88, 3, 1905}, {42, 90, 3, 1905}, {43, 91, 6, 1905}, {44, 92, 3,
1905}, {46, 94, 1, 1905}, {47, 95, 3, 1905}, {48, 96, 3, 1905}, {49, 97, 1, 1905}, {37,
86, 1, 1906}, {39, 88, 3, 1906}, {40, 89, 3, 1906}, {42, 91, 3, 1906}, {43, 92, 6, 1906},
{44, 93, 3, 1906}, {46, 95, 1, 1906}, {47, 96, 3, 1906}, {48, 97, 3, 1906}, {49, 98, 1,
1906}, {37, 87, 1, 1907}, {39, 90, 3, 1907}, {40, 91, 3, 1907}, {42, 94, 3, 1907}, {43,
95, 6, 1907}, {44, 96, 3, 1907}, {46, 99, 1, 1907}, {47, 100, 3, 1907}, {48, 101, 3,
1907}, {49, 102, 1, 1907}, {37, 88, 1, 1908}, {39, 91, 3, 1908}, {40, 92, 3, 1908}, {42,
95, 3, 1908}, {43, 96, 6, 1908}, {44, 97, 3, 1908}, {46, 100, 1, 1908}, {47, 101, 3,
1908}, {48, 102, 3, 1908}, {49, 103, 1, 1908}, {37, 89, 1, 1909}, {39, 92, 3, 1909}, {40,
93, 3, 1909}, {42, 96, 3, 1909}, {43, 97, 6, 1909}, {44, 98, 3, 1909}, {46, 101, 1, 1909},
{47, 102, 3, 1909}, {48, 103, 3, 1909}, {49, 104, 1, 1909}, {37, 90, 1, 1910}, {39, 94, 3,
1910}, {40, 95, 3, 1910}, {42, 99, 3, 1910}, {43, 100, 6, 1910}, {44, 101, 3, 1910}, {46,
105, 1, 1910}, {47, 106, 3, 1910}, {48, 107, 3, 1910}, {49, 108, 1, 1910}, {37, 91, 1,
1911}, {39, 95, 3, 1911}, {40, 96, 3, 1911}, {42, 100, 3, 1911}, {43, 101, 6, 1911}, {44,
102, 3, 1911}, {46, 106, 1, 1911}, {47, 107, 3, 1911}, {48, 108, 3, 1911}, {49, 109, 1,
1911}, {37, 92, 1, 1912}, {39, 96, 3, 1912}, {40, 97, 3, 1912}, {42, 101, 3, 1912}, {43,
102, 6, 1912}, {44, 103, 3, 1912}, {46, 107, 1, 1912}, {47, 108, 3, 1912}, {48, 109, 3,
1912}, {49, 110, 1, 1912}, {37, 93, 1, 1913}, {39, 97, 3, 1913}, {40, 98, 3, 1913}, {42,
102, 3, 1913}, {43, 103, 6, 1913}, {44, 104, 3, 1913}, {46, 108, 1, 1913}, {47, 109, 3,
1913}, {48, 110, 3, 1913}, {49, 111, 1, 1913}, {37, 94, 1, 1914}, {39, 99, 3, 1914}, {40,
100, 3, 1914}, {42, 105, 3, 1914}, {43, 106, 6, 1914}, {44, 107, 3, 1914}, {46, 112, 1,

1914}, {47, 113, 3, 1914}, {48, 114, 3, 1914}, {49, 115, 1, 1914}, {37, 95, 1, 1915}, {39, 100, 3, 1915}, {40, 101, 3, 1915}, {42, 106, 3, 1915}, {43, 107, 6, 1915}, {44, 108, 3, 1915}, {46, 113, 1, 1915}, {47, 114, 3, 1915}, {48, 115, 3, 1915}, {49, 116, 1, 1915}, {37, 96, 1, 1916}, {39, 101, 3, 1916}, {40, 102, 3, 1916}, {42, 107, 3, 1916}, {43, 108, 6, 1916}, {44, 109, 3, 1916}, {46, 114, 1, 1916}, {47, 115, 3, 1916}, {48, 116, 3, 1916}, {49, 117, 1, 1916}, {37, 97, 1, 1917}, {39, 102, 3, 1917}, {40, 103, 3, 1917}, {42, 108, 3, 1917}, {43, 109, 6, 1917}, {44, 110, 3, 1917}, {46, 115, 1, 1917}, {47, 116, 3, 1917}, {48, 117, 3, 1917}, {49, 118, 1, 1917}, {37, 98, 1, 1918}, {39, 103, 3, 1918}, {40, 104, 3, 1918}, {42, 109, 3, 1918}, {43, 110, 6, 1918}, {44, 111, 3, 1918}, {46, 116, 1, 1918}, {47, 117, 3, 1918}, {48, 118, 3, 1918}, {49, 119, 1, 1918}, {38, 84, 1, 1919}, {41, 85, 3, 1919}, {42, 86, 3, 1919}, {45, 87, 3, 1919}, {46, 88, 6, 1919}, {47, 89, 3, 1919}, {50, 90, 1, 1919}, {51, 91, 3, 1919}, {52, 92, 3, 1919}, {53, 93, 1, 1919}, {38, 85, 1, 1920}, {41, 87, 3, 1920}, {42, 88, 3, 1920}, {45, 90, 3, 1920}, {46, 91, 6, 1920}, {47, 92, 3, 1920}, {50, 94, 1, 1920}, {51, 95, 3, 1920}, {52, 96, 3, 1920}, {53, 97, 1, 1920}, {38, 86, 1, 1921}, {41, 88, 3, 1921}, {42, 89, 3, 1921}, {45, 91, 3, 1921}, {46, 92, 6, 1921}, {47, 93, 3, 1921}, {50, 95, 1, 1921}, {51, 96, 3, 1921}, {52, 97, 3, 1921}, {53, 98, 1, 1921}, {38, 87, 1, 1922}, {41, 90, 3, 1922}, {42, 91, 3, 1922}, {45, 94, 3, 1922}, {46, 95, 6, 1922}, {47, 96, 3, 1922}, {50, 99, 1, 1922}, {51, 100, 3, 1922}, {52, 101, 3, 1922}, {53, 102, 1, 1922}, {38, 88, 1, 1923}, {41, 91, 3, 1923}, {42, 92, 3, 1923}, {45, 95, 3, 1923}, {46, 96, 6, 1923}, {47, 97, 3, 1923}, {50, 100, 1, 1923}, {51, 101, 3, 1923}, {52, 102, 3, 1923}, {53, 103, 1, 1923}, {38, 89, 1, 1924}, {41, 92, 3, 1924}, {42, 93, 3, 1924}, {45, 96, 3, 1924}, {46, 97, 6, 1924}, {47, 98, 3, 1924}, {50, 101, 1, 1924}, {51, 102, 3, 1924}, {52, 103, 3, 1924}, {53, 104, 1, 1924}, {38, 90, 1, 1925}, {41, 94, 3, 1925}, {42, 95, 3, 1925}, {45, 99, 3, 1925}, {46, 100, 6, 1925}, {47, 101, 3, 1925}, {50, 105, 1, 1925}, {51, 106, 3, 1925}, {52, 107, 3, 1925}, {53, 108, 1, 1925}, {38, 91, 1, 1926}, {41, 95, 3, 1926}, {42, 96, 3, 1926}, {45, 100, 3, 1926}, {46, 101, 6, 1926}, {47, 102, 3, 1926}, {50, 106, 1, 1926}, {51, 107, 3, 1926}, {52, 108, 3, 1926}, {53, 109, 1, 1926}, {38, 92, 1, 1927}, {41, 96, 3, 1927}, {42, 97, 3, 1927}, {45, 101, 3, 1927}, {46, 102, 6, 1927}, {47, 103, 3, 1927}, {50, 107, 1, 1927}, {51, 108, 3, 1927}, {52, 109, 3, 1927}, {53, 110, 1, 1927}, {38, 93, 1, 1928}, {41, 97, 3, 1928}, {42, 98, 3, 1928}, {45, 102, 3, 1928}, {46, 103, 6, 1928}, {47, 104, 3, 1928}, {50, 108, 1, 1928}, {51, 109, 3, 1928}, {52, 110, 3, 1928}, {53, 111, 1, 1928}, {38, 94, 1, 1929}, {41, 99, 3, 1929}, {42, 100, 3, 1929}, {45, 105, 3, 1929}, {46, 106, 6, 1929}, {47, 107, 3, 1929}, {50, 112, 1, 1929}, {51, 113, 3, 1929}, {52, 114, 3, 1929}, {53, 115, 1, 1929}, {38, 95, 1, 1930}, {41, 100, 3, 1930}, {42, 101, 3, 1930}, {45, 106, 3, 1930}, {46, 107, 6, 1930}, {47, 108, 3, 1930}, {50, 113, 1, 1930}, {51, 114, 3, 1930}, {52, 115, 3, 1930}, {53, 116, 1, 1930}, {38, 96, 1, 1931}, {41, 101, 3, 1931}, {42, 102, 3, 1931}, {45, 107, 3, 1931}, {46, 108, 6, 1931}, {47, 109, 3, 1931}, {50, 114, 1, 1931}, {51, 115, 3, 1931}, {52, 116, 3, 1931}, {53, 117, 1, 1931}, {38, 97, 1, 1932}, {41, 102, 3, 1932}, {42, 103, 3, 1932}, {45, 108, 3, 1932}, {46, 109, 6, 1932}, {47, 110, 3, 1932}, {50, 115, 1, 1932}, {51, 116, 3, 1932}, {52, 117, 3, 1932}, {53, 118, 1, 1932}, {38, 98, 1, 1933}, {41, 103, 3, 1933}, {42, 104, 3, 1933}, {45, 109, 3, 1933}, {46, 110, 6, 1933}, {47, 111, 3, 1933}, {50, 116, 1, 1933}, {51, 117, 3, 1933}, {52, 118, 3, 1933}, {53, 119, 1, 1933}, {39, 84, 1, 1934}, {42, 85, 3, 1934}, {43, 86, 3, 1934}, {46, 87, 3, 1934}, {47, 88, 6, 1934}, {48, 89, 3, 1934}, {51, 90, 1, 1934}, {52, 91, 3, 1934}, {53, 92, 3, 1934}, {54, 93, 1, 1934}, {39, 85, 1, 1935}, {42, 87, 3, 1935}, {43, 88, 3, 1935}, {46, 90, 3, 1935}, {47, 91, 6, 1935}, {48, 92, 3, 1935}, {51, 94, 1, 1935}, {52, 95, 3, 1935}, {53, 96, 3, 1935}, {54, 97, 1, 1935}, {39, 86, 1, 1936}, {42, 88, 3, 1936}, {43, 89, 3, 1936}, {46, 91, 3, 1936}, {47, 92, 6, 1936}, {48, 93, 3, 1936}, {51, 95, 1, 1936}, {52, 96, 3, 1936}, {53, 97, 3, 1936}, {54, 98, 1, 1936}, {39, 87, 1, 1937}, {42, 90, 3, 1937}, {43, 91, 3, 1937}, {46, 94, 3, 1937}, {47, 95, 6, 1937}, {48, 96, 3, 1937}, {51, 99, 1, 1937}, {52, 100, 3, 1937}, {53, 101, 3, 1937}, {54, 102, 1, 1937}, {39, 88, 1, 1938}, {42, 91, 3, 1938}, {43, 92, 3, 1938}, {46, 95, 3, 1938}, {47, 96, 6, 1938}, {48, 97, 3, 1938}, {51, 100, 1, 1938}, {52, 101, 3, 1938}, {53, 102, 3, 1938}, {54, 103, 1, 1938}, {39, 89, 1, 1939}, {42, 92, 3, 1939}, {43, 93, 3, 1939}, {46, 96, 3, 1939}, {47, 97, 6, 1939}, {48, 98, 3, 1939}, {51, 101, 1, 1939}, {52, 102, 3, 1939}, {53, 103, 3, 1939}, {54, 104, 1, 1939}, {39, 90, 1, 1940}, {42, 94, 3, 1940}, {43, 95, 3, 1940}, {46, 99, 3, 1940}, {47, 100, 6, 1940}, {48, 101, 3, 1940}, {51, 105, 1, 1940}, {52, 106, 3, 1940}, {53, 107, 3, 1940}, {54, 108, 1, 1940}, {39, 91, 1, 1941}, {42, 95, 3, 1941}, {43, 96, 3, 1941}, {46, 100, 3, 1941}, {47, 101, 6, 1941}, {48, 102, 3, 1941}, {51, 106, 1, 1941}, {52, 107, 3, 1941}, {53, 108, 3, 1941}, {54, 109, 1, 1941}, {39, 92, 1, 1942}, {42, 96, 3, 1942}, {43, 97, 3, 1942}, {46, 101, 3, 1942}, {47, 102, 6, 1942}, {48, 103, 3, 1942}, {51, 107, 1, 1942}, {52, 108, 3, 1942}, {53, 109, 3, 1942}, {54, 110, 1, 1942}, {39, 93, 1, 1943}, {42, 97, 3, 1943}, {43, 98, 3, 1943}, {46, 102, 3, 1943}, {47, 103, 6, 1943}, {48, 104, 3, 1943}, {51, 108, 1, 1943}, {52, 109, 3, 1943}, {53, 110, 3, 1943}, {54, 111, 1, 1943}, {39, 94, 1, 1944}, {42, 99, 3, 1944}, {43, 100, 3, 1944}, {46, 105, 3, 1944}, {47, 106, 6, 1944}, {48, 107, 3, 1944}, {51, 112, 1, 1944}, {52, 113, 3, 1944}, {53, 114, 3, 1944}, {54, 115, 1, 1944}, {39, 95, 1, 1945}, {42, 100, 3, 1945}, {43, 101, 3, 1945}, {46, 106, 3, 1945}, {47, 107, 6, 1945}, {48, 108, 3, 1945}, {51, 113, 1, 1945}, {52, 114, 3, 1945}, {53, 115, 3, 1945}, {54, 116, 1, 1945},

{39, 96, 1, 1946}, {42, 101, 3, 1946}, {43, 102, 3, 1946}, {46, 107, 3, 1946}, {47, 108, 6, 1946}, {48, 109, 3, 1946}, {51, 114, 1, 1946}, {52, 115, 3, 1946}, {53, 116, 3, 1946}, {54, 117, 1, 1946}, {39, 97, 1, 1947}, {42, 102, 3, 1947}, {43, 103, 3, 1947}, {46, 108, 3, 1947}, {47, 109, 6, 1947}, {48, 110, 3, 1947}, {51, 115, 1, 1947}, {52, 116, 3, 1947}, {53, 117, 3, 1947}, {54, 118, 1, 1947}, {39, 98, 1, 1948}, {42, 103, 3, 1948}, {43, 104, 3, 1948}, {46, 109, 3, 1948}, {47, 110, 6, 1948}, {48, 111, 3, 1948}, {51, 116, 1, 1948}, {52, 117, 3, 1948}, {53, 118, 3, 1948}, {54, 119, 1, 1948}, {40, 84, 1, 1949}, {43, 85, 3, 1949}, {44, 86, 3, 1949}, {47, 87, 3, 1949}, {48, 88, 6, 1949}, {49, 89, 3, 1949}, {52, 90, 1, 1949}, {53, 91, 3, 1949}, {54, 92, 3, 1949}, {55, 93, 1, 1949}, {40, 85, 1, 1950}, {43, 87, 3, 1950}, {44, 88, 3, 1950}, {47, 90, 3, 1950}, {48, 91, 6, 1950}, {49, 92, 3, 1950}, {52, 94, 1, 1950}, {53, 95, 3, 1950}, {54, 96, 3, 1950}, {55, 97, 1, 1950}, {40, 86, 1, 1951}, {43, 88, 3, 1951}, {44, 89, 3, 1951}, {47, 91, 3, 1951}, {48, 92, 6, 1951}, {49, 93, 3, 1951}, {52, 95, 1, 1951}, {53, 96, 3, 1951}, {54, 97, 3, 1951}, {55, 98, 1, 1951}, {40, 87, 1, 1952}, {43, 90, 3, 1952}, {44, 91, 3, 1952}, {47, 94, 3, 1952}, {48, 95, 6, 1952}, {49, 96, 3, 1952}, {52, 99, 1, 1952}, {53, 100, 3, 1952}, {54, 101, 3, 1952}, {55, 102, 1, 1952}, {40, 88, 1, 1953}, {43, 91, 3, 1953}, {44, 92, 3, 1953}, {47, 95, 3, 1953}, {48, 96, 6, 1953}, {49, 97, 3, 1953}, {52, 100, 1, 1953}, {53, 101, 3, 1953}, {54, 102, 3, 1953}, {55, 103, 1, 1953}, {40, 89, 1, 1954}, {43, 92, 3, 1954}, {44, 93, 3, 1954}, {47, 96, 3, 1954}, {48, 97, 6, 1954}, {49, 98, 3, 1954}, {52, 101, 1, 1954}, {53, 102, 3, 1954}, {54, 103, 3, 1954}, {55, 104, 1, 1954}, {40, 90, 1, 1955}, {43, 94, 3, 1955}, {44, 95, 3, 1955}, {47, 99, 3, 1955}, {48, 100, 6, 1955}, {49, 101, 3, 1955}, {52, 105, 1, 1955}, {53, 106, 3, 1955}, {54, 107, 3, 1955}, {55, 108, 1, 1955}, {40, 91, 1, 1956}, {43, 95, 3, 1956}, {44, 96, 3, 1956}, {47, 100, 3, 1956}, {48, 101, 6, 1956}, {49, 102, 3, 1956}, {52, 106, 1, 1956}, {53, 107, 3, 1956}, {54, 108, 3, 1956}, {55, 109, 1, 1956}, {40, 92, 1, 1957}, {43, 96, 3, 1957}, {44, 97, 3, 1957}, {47, 101, 3, 1957}, {48, 102, 6, 1957}, {49, 103, 3, 1957}, {52, 107, 1, 1957}, {53, 108, 3, 1957}, {54, 109, 3, 1957}, {55, 110, 1, 1957}, {40, 93, 1, 1958}, {43, 97, 3, 1958}, {44, 98, 3, 1958}, {47, 102, 3, 1958}, {48, 103, 6, 1958}, {49, 104, 3, 1958}, {52, 108, 1, 1958}, {53, 109, 3, 1958}, {54, 110, 3, 1958}, {55, 111, 1, 1958}, {40, 94, 1, 1959}, {43, 99, 3, 1959}, {44, 100, 3, 1959}, {47, 105, 3, 1959}, {48, 106, 6, 1959}, {49, 107, 3, 1959}, {52, 112, 1, 1959}, {53, 113, 3, 1959}, {54, 114, 3, 1959}, {55, 115, 1, 1959}, {40, 95, 1, 1960}, {43, 100, 3, 1960}, {44, 101, 3, 1960}, {47, 106, 3, 1960}, {48, 107, 6, 1960}, {49, 108, 3, 1960}, {52, 113, 1, 1960}, {53, 114, 3, 1960}, {54, 115, 3, 1960}, {55, 116, 1, 1960}, {40, 96, 1, 1961}, {43, 101, 3, 1961}, {44, 102, 3, 1961}, {47, 107, 3, 1961}, {48, 108, 6, 1961}, {49, 109, 3, 1961}, {52, 114, 1, 1961}, {53, 115, 3, 1961}, {54, 116, 3, 1961}, {55, 117, 1, 1961}, {40, 97, 1, 1962}, {43, 102, 3, 1962}, {44, 103, 3, 1962}, {47, 108, 3, 1962}, {48, 109, 6, 1962}, {49, 110, 3, 1962}, {52, 115, 1, 1962}, {53, 116, 3, 1962}, {54, 117, 3, 1962}, {55, 118, 1, 1962}, {40, 98, 1, 1963}, {43, 103, 3, 1963}, {44, 104, 3, 1963}, {47, 109, 3, 1963}, {48, 110, 6, 1963}, {49, 111, 3, 1963}, {52, 116, 1, 1963}, {53, 117, 3, 1963}, {54, 118, 3, 1963}, {55, 119, 1, 1963}, {35, 35, 1, 1964}, {36, 36, 4, 1964}, {37, 37, 4, 1964}, {38, 38, 6, 1964}, {39, 39, 12, 1964}, {40, 40, 6, 1964}, {41, 41, 4, 1964}, {42, 42, 12, 1964}, {43, 43, 12, 1964}, {44, 44, 4, 1964}, {45, 45, 1, 1964}, {46, 46, 4, 1964}, {47, 47, 6, 1964}, {48, 48, 4, 1964}, {49, 49, 1, 1964}, {35, 36, 1, 1965}, {36, 38, 4, 1965}, {37, 39, 4, 1965}, {38, 41, 6, 1965}, {39, 42, 12, 1965}, {40, 43, 6, 1965}, {41, 45, 4, 1965}, {42, 46, 12, 1965}, {43, 47, 12, 1965}, {44, 48, 4, 1965}, {45, 50, 1, 1965}, {46, 51, 4, 1965}, {47, 52, 6, 1965}, {48, 53, 4, 1965}, {49, 54, 1, 1965}, {35, 37, 1, 1966}, {36, 39, 4, 1966}, {37, 40, 4, 1966}, {38, 42, 6, 1966}, {39, 43, 12, 1966}, {40, 44, 6, 1966}, {41, 46, 4, 1966}, {42, 47, 12, 1966}, {43, 48, 12, 1966}, {44, 49, 4, 1966}, {45, 51, 1, 1966}, {46, 52, 4, 1966}, {47, 53, 6, 1966}, {48, 54, 4, 1966}, {49, 55, 1, 1966}, {36, 36, 1, 1967}, {38, 38, 4, 1967}, {39, 39, 4, 1967}, {41, 41, 6, 1967}, {42, 42, 12, 1967}, {43, 43, 6, 1967}, {45, 45, 4, 1967}, {46, 46, 12, 1967}, {47, 47, 12, 1967}, {48, 48, 4, 1967}, {50, 50, 1, 1967}, {51, 51, 4, 1967}, {52, 52, 6, 1967}, {53, 53, 4, 1967}, {54, 54, 1, 1967}, {36, 37, 1, 1968}, {38, 39, 4, 1968}, {39, 40, 4, 1968}, {41, 42, 6, 1968}, {42, 43, 12, 1968}, {43, 44, 6, 1968}, {45, 46, 4, 1968}, {46, 47, 12, 1968}, {47, 48, 12, 1968}, {48, 49, 4, 1968}, {50, 51, 1, 1968}, {51, 52, 4, 1968}, {52, 53, 6, 1968}, {53, 54, 4, 1968}, {54, 55, 1, 1968}, {37, 37, 1, 1969}, {39, 39, 4, 1969}, {40, 40, 4, 1969}, {42, 42, 6, 1969}, {43, 43, 12, 1969}, {44, 44, 6, 1969}, {46, 46, 4, 1969}, {47, 47, 12, 1969}, {48, 48, 12, 1969}, {49, 49, 4, 1969}, {51, 51, 1, 1969}, {52, 52, 4, 1969}, {53, 53, 6, 1969}, {54, 54, 4, 1969}, {55, 55, 1, 1969}, {35, 56, 1, 1970}, {36, 57, 4, 1970}, {37, 58, 4, 1970}, {38, 59, 6, 1970}, {39, 60, 12, 1970}, {40, 61, 6, 1970}, {41, 62, 4, 1970}, {42, 63, 12, 1970}, {43, 64, 12, 1970}, {44, 65, 4, 1970}, {45, 66, 1, 1970}, {46, 67, 4, 1970}, {47, 68, 6, 1970}, {48, 69, 4, 1970}, {49, 70, 1, 1970}, {35, 57, 1, 1971}, {36, 59, 4, 1971}, {37, 60, 4, 1971}, {38, 62, 6, 1971}, {39, 63, 12, 1971}, {40, 64, 6, 1971}, {41, 66, 4, 1971}, {42, 67, 12, 1971}, {43, 68, 12, 1971}, {44, 69, 4, 1971}, {45, 71, 1, 1971}, {46, 72, 4, 1971}, {47, 73, 6, 1971}, {48, 74, 4, 1971}, {49, 75, 1, 1971}, {35, 58, 1, 1972}, {36, 60, 4, 1972}, {37, 61, 4, 1972}, {38, 63, 6, 1972}, {39, 64, 12, 1972}, {40, 65, 6, 1972}, {41, 67, 4, 1972}, {42, 68, 12, 1972}, {43, 69, 12, 1972}, {44, 70, 4, 1972}, {45, 72, 1, 1972}, {46, 73, 4, 1972}, {47, 74, 6, 1972}, {48, 75, 4, 1972}, {49, 76, 1, 1972}, {35, 59, 1, 1973}, {36,

62, 4, 1973}, {37, 63, 4, 1973}, {38, 66, 6, 1973}, {39, 67, 12, 1973}, {40, 68, 6, 1973}, {41, 71, 4, 1973}, {42, 72, 12, 1973}, {43, 73, 12, 1973}, {44, 74, 4, 1973}, {45, 77, 1, 1973}, {46, 78, 4, 1973}, {47, 79, 6, 1973}, {48, 80, 4, 1973}, {49, 81, 1, 1973}, {35, 60, 1, 1974}, {36, 63, 4, 1974}, {37, 64, 4, 1974}, {38, 67, 6, 1974}, {39, 68, 12, 1974}, {40, 69, 6, 1974}, {41, 72, 4, 1974}, {42, 73, 12, 1974}, {43, 74, 12, 1974}, {44, 75, 4, 1974}, {45, 78, 1, 1974}, {46, 79, 4, 1974}, {47, 80, 6, 1974}, {48, 81, 4, 1974}, {49, 82, 1, 1974}, {35, 61, 1, 1975}, {36, 64, 4, 1975}, {37, 65, 4, 1975}, {38, 68, 6, 1975}, {39, 69, 12, 1975}, {40, 70, 6, 1975}, {41, 73, 4, 1975}, {42, 74, 12, 1975}, {43, 75, 12, 1975}, {44, 76, 4, 1975}, {45, 79, 1, 1975}, {46, 80, 4, 1975}, {47, 81, 6, 1975}, {48, 82, 4, 1975}, {49, 83, 1, 1975}, {36, 56, 1, 1976}, {38, 57, 4, 1976}, {39, 58, 4, 1976}, {41, 59, 6, 1976}, {42, 60, 12, 1976}, {43, 61, 6, 1976}, {45, 62, 4, 1976}, {46, 63, 12, 1976}, {47, 64, 12, 1976}, {48, 65, 4, 1976}, {50, 66, 1, 1976}, {51, 67, 4, 1976}, {52, 68, 6, 1976}, {53, 69, 4, 1976}, {54, 70, 1, 1976}, {36, 57, 1, 1977}, {38, 59, 4, 1977}, {39, 60, 4, 1977}, {41, 62, 6, 1977}, {42, 63, 12, 1977}, {43, 64, 6, 1977}, {45, 66, 4, 1977}, {46, 67, 12, 1977}, {47, 68, 12, 1977}, {48, 69, 4, 1977}, {50, 71, 1, 1977}, {51, 72, 4, 1977}, {52, 73, 6, 1977}, {53, 74, 4, 1977}, {54, 75, 1, 1977}, {36, 58, 1, 1978}, {38, 60, 4, 1978}, {39, 61, 4, 1978}, {41, 63, 6, 1978}, {42, 64, 12, 1978}, {43, 65, 6, 1978}, {45, 67, 4, 1978}, {46, 68, 12, 1978}, {47, 69, 12, 1978}, {48, 70, 4, 1978}, {50, 72, 1, 1978}, {51, 73, 4, 1978}, {52, 74, 6, 1978}, {53, 75, 4, 1978}, {54, 76, 1, 1978}, {36, 59, 1, 1979}, {38, 62, 4, 1979}, {39, 63, 4, 1979}, {41, 66, 6, 1979}, {42, 67, 12, 1979}, {43, 68, 6, 1979}, {45, 71, 4, 1979}, {46, 72, 12, 1979}, {47, 73, 12, 1979}, {48, 74, 4, 1979}, {50, 77, 1, 1979}, {51, 78, 4, 1979}, {52, 79, 6, 1979}, {53, 80, 4, 1979}, {54, 81, 1, 1979}, {36, 60, 1, 1980}, {38, 63, 4, 1980}, {39, 64, 4, 1980}, {41, 67, 6, 1980}, {42, 68, 12, 1980}, {43, 69, 6, 1980}, {45, 72, 4, 1980}, {46, 73, 12, 1980}, {47, 74, 12, 1980}, {48, 75, 4, 1980}, {50, 78, 1, 1980}, {51, 79, 4, 1980}, {52, 80, 6, 1980}, {53, 81, 4, 1980}, {54, 82, 1, 1980}, {36, 61, 1, 1981}, {38, 64, 4, 1981}, {39, 65, 4, 1981}, {41, 68, 6, 1981}, {42, 69, 12, 1981}, {43, 70, 6, 1981}, {45, 73, 4, 1981}, {46, 74, 12, 1981}, {47, 75, 12, 1981}, {48, 76, 4, 1981}, {50, 79, 1, 1981}, {51, 80, 4, 1981}, {52, 81, 6, 1981}, {53, 82, 4, 1981}, {54, 83, 1, 1981}, {37, 56, 1, 1982}, {39, 57, 4, 1982}, {40, 58, 4, 1982}, {42, 59, 6, 1982}, {43, 60, 12, 1982}, {44, 61, 6, 1982}, {46, 62, 4, 1982}, {47, 63, 12, 1982}, {48, 64, 12, 1982}, {49, 65, 4, 1982}, {51, 66, 1, 1982}, {52, 67, 4, 1982}, {53, 68, 6, 1982}, {54, 69, 4, 1982}, {55, 70, 1, 1982}, {37, 57, 1, 1983}, {39, 59, 4, 1983}, {40, 60, 4, 1983}, {42, 62, 6, 1983}, {43, 63, 12, 1983}, {44, 64, 6, 1983}, {46, 66, 4, 1983}, {47, 67, 12, 1983}, {48, 68, 12, 1983}, {49, 69, 4, 1983}, {51, 71, 1, 1983}, {52, 72, 4, 1983}, {53, 73, 6, 1983}, {54, 74, 4, 1983}, {55, 75, 1, 1983}, {37, 58, 1, 1984}, {39, 60, 4, 1984}, {40, 61, 4, 1984}, {42, 63, 6, 1984}, {43, 64, 12, 1984}, {44, 65, 6, 1984}, {46, 67, 4, 1984}, {47, 68, 12, 1984}, {48, 69, 12, 1984}, {49, 70, 4, 1984}, {51, 72, 1, 1984}, {52, 73, 4, 1984}, {53, 74, 6, 1984}, {54, 75, 4, 1984}, {55, 76, 1, 1984}, {37, 59, 1, 1985}, {39, 62, 4, 1985}, {40, 63, 4, 1985}, {42, 66, 6, 1985}, {43, 67, 12, 1985}, {44, 68, 6, 1985}, {46, 71, 4, 1985}, {47, 72, 12, 1985}, {48, 73, 12, 1985}, {49, 74, 4, 1985}, {51, 77, 1, 1985}, {52, 78, 4, 1985}, {53, 79, 6, 1985}, {54, 80, 4, 1985}, {55, 81, 1, 1985}, {37, 60, 1, 1986}, {39, 63, 4, 1986}, {40, 64, 4, 1986}, {42, 67, 6, 1986}, {43, 68, 12, 1986}, {44, 69, 6, 1986}, {46, 72, 4, 1986}, {47, 73, 12, 1986}, {48, 74, 12, 1986}, {49, 75, 4, 1986}, {51, 78, 1, 1986}, {52, 79, 4, 1986}, {53, 80, 6, 1986}, {54, 81, 4, 1986}, {55, 82, 1, 1986}, {37, 61, 1, 1987}, {39, 64, 4, 1987}, {40, 65, 4, 1987}, {42, 68, 6, 1987}, {43, 69, 12, 1987}, {44, 70, 6, 1987}, {46, 73, 4, 1987}, {47, 74, 12, 1987}, {48, 75, 12, 1987}, {49, 76, 4, 1987}, {51, 79, 1, 1987}, {52, 80, 4, 1987}, {53, 81, 6, 1987}, {54, 82, 4, 1987}, {55, 83, 1, 1987}, {35, 84, 1, 1988}, {36, 85, 4, 1988}, {37, 86, 4, 1988}, {38, 87, 6, 1988}, {39, 88, 12, 1988}, {40, 89, 6, 1988}, {41, 90, 4, 1988}, {42, 91, 12, 1988}, {43, 92, 12, 1988}, {44, 93, 4, 1988}, {45, 94, 1, 1988}, {46, 95, 4, 1988}, {47, 96, 6, 1988}, {48, 97, 4, 1988}, {49, 98, 1, 1988}, {35, 85, 1, 1989}, {36, 87, 4, 1989}, {37, 88, 4, 1989}, {38, 90, 6, 1989}, {39, 91, 12, 1989}, {40, 92, 6, 1989}, {41, 94, 4, 1989}, {42, 95, 12, 1989}, {43, 96, 12, 1989}, {44, 97, 4, 1989}, {45, 99, 1, 1989}, {46, 100, 4, 1989}, {47, 101, 6, 1989}, {48, 102, 4, 1989}, {49, 103, 1, 1989}, {35, 86, 1, 1990}, {36, 88, 4, 1990}, {37, 89, 4, 1990}, {38, 91, 6, 1990}, {39, 92, 12, 1990}, {40, 93, 6, 1990}, {41, 95, 4, 1990}, {42, 96, 12, 1990}, {43, 97, 12, 1990}, {44, 98, 4, 1990}, {45, 100, 1, 1990}, {46, 101, 4, 1990}, {47, 102, 6, 1990}, {48, 103, 4, 1990}, {49, 104, 1, 1990}, {35, 87, 1, 1991}, {36, 90, 4, 1991}, {37, 91, 4, 1991}, {38, 94, 6, 1991}, {39, 95, 12, 1991}, {40, 96, 6, 1991}, {41, 99, 4, 1991}, {42, 100, 12, 1991}, {43, 101, 12, 1991}, {44, 102, 4, 1991}, {45, 105, 1, 1991}, {46, 106, 4, 1991}, {47, 107, 6, 1991}, {48, 108, 4, 1991}, {49, 109, 1, 1991}, {35, 88, 1, 1992}, {36, 91, 4, 1992}, {37, 92, 4, 1992}, {38, 95, 6, 1992}, {39, 96, 12, 1992}, {40, 97, 6, 1992}, {41, 100, 4, 1992}, {42, 101, 12, 1992}, {43, 102, 12, 1992}, {44, 103, 4, 1992}, {45, 106, 1, 1992}, {46, 107, 4, 1992}, {47, 108, 6, 1992}, {48, 109, 4, 1992}, {49, 110, 1, 1992}, {35, 89, 1, 1993}, {36, 92, 4, 1993}, {37, 93, 4, 1993}, {38, 96, 6, 1993}, {39, 97, 12, 1993}, {40, 98, 6, 1993}, {41, 101, 4, 1993}, {42, 102, 12, 1993}, {43, 103, 12, 1993}, {44, 104, 4, 1993}, {45, 107, 1, 1993}, {46, 108, 4, 1993}, {47, 109, 6, 1993}, {48, 110, 4, 1993}, {49, 111, 1, 1993}, {35, 90, 1, 1994}, {36, 94, 4, 1994}, {37, 95, 4, 1994}, {38, 99, 6, 1994}, {39, 100, 12, 1994}, {40, 101, 6,

1994}, {41, 105, 4, 1994}, {42, 106, 12, 1994}, {43, 107, 12, 1994}, {44, 108, 4, 1994},
{45, 112, 1, 1994}, {46, 113, 4, 1994}, {47, 114, 6, 1994}, {48, 115, 4, 1994}, {49, 116,
1, 1994}, {35, 91, 1, 1995}, {36, 95, 4, 1995}, {37, 96, 4, 1995}, {38, 100, 6, 1995},
{39, 101, 12, 1995}, {40, 102, 6, 1995}, {41, 106, 4, 1995}, {42, 107, 12, 1995}, {43,
108, 12, 1995}, {44, 109, 4, 1995}, {45, 113, 1, 1995}, {46, 114, 4, 1995}, {47, 115, 6,
1995}, {48, 116, 4, 1995}, {49, 117, 1, 1995}, {35, 92, 1, 1996}, {36, 96, 4, 1996}, {37,
97, 4, 1996}, {38, 101, 6, 1996}, {39, 102, 12, 1996}, {40, 103, 6, 1996}, {41, 107, 4,
1996}, {42, 108, 12, 1996}, {43, 109, 12, 1996}, {44, 110, 4, 1996}, {45, 114, 1, 1996},
{46, 115, 4, 1996}, {47, 116, 6, 1996}, {48, 117, 4, 1996}, {49, 118, 1, 1996}, {35, 93,
1, 1997}, {36, 97, 4, 1997}, {37, 98, 4, 1997}, {38, 102, 6, 1997}, {39, 103, 12, 1997},
{40, 104, 6, 1997}, {41, 108, 4, 1997}, {42, 109, 12, 1997}, {43, 110, 12, 1997}, {44,
111, 4, 1997}, {45, 115, 1, 1997}, {46, 116, 4, 1997}, {47, 117, 6, 1997}, {48, 118, 4,
1997}, {49, 119, 1, 1997}, {36, 84, 1, 1998}, {38, 85, 4, 1998}, {39, 86, 4, 1998}, {41,
87, 6, 1998}, {42, 88, 12, 1998}, {43, 89, 6, 1998}, {45, 90, 4, 1998}, {46, 91, 12,
1998}, {47, 92, 12, 1998}, {48, 93, 4, 1998}, {50, 94, 1, 1998}, {51, 95, 4, 1998}, {52,
96, 6, 1998}, {53, 97, 4, 1998}, {54, 98, 1, 1998}, {36, 85, 1, 1999}, {38, 87, 4, 1999},
{39, 88, 4, 1999}, {41, 90, 6, 1999}, {42, 91, 12, 1999}, {43, 92, 6, 1999}, {45, 94, 4,
1999}, {46, 95, 12, 1999}, {47, 96, 12, 1999}, {48, 97, 4, 1999}, {50, 99, 1, 1999}, {51,
100, 4, 1999}, {52, 101, 6, 1999}, {53, 102, 4, 1999}, {54, 103, 1, 1999}, {36, 86, 1,
2000}, {38, 88, 4, 2000}, {39, 89, 4, 2000}, {41, 91, 6, 2000}, {42, 92, 12, 2000}, {43,
93, 6, 2000}, {45, 95, 4, 2000}, {46, 96, 12, 2000}, {47, 97, 12, 2000}, {48, 98, 4,
2000}, {50, 100, 1, 2000}, {51, 101, 4, 2000}, {52, 102, 6, 2000}, {53, 103, 4, 2000},
{54, 104, 1, 2000}, {36, 87, 1, 2001}, {38, 90, 4, 2001}, {39, 91, 4, 2001}, {41, 94, 6,
2001}, {42, 95, 12, 2001}, {43, 96, 6, 2001}, {45, 99, 4, 2001}, {46, 100, 12, 2001}, {47,
101, 12, 2001}, {48, 102, 4, 2001}, {50, 105, 1, 2001}, {51, 106, 4, 2001}, {52, 107, 6,
2001}, {53, 108, 4, 2001}, {54, 109, 1, 2001}, {36, 88, 1, 2002}, {38, 91, 4, 2002}, {39,
92, 4, 2002}, {41, 95, 6, 2002}, {42, 96, 12, 2002}, {43, 97, 6, 2002}, {45, 100, 4,
2002}, {46, 101, 12, 2002}, {47, 102, 12, 2002}, {48, 103, 4, 2002}, {50, 106, 1, 2002},
{51, 107, 4, 2002}, {52, 108, 6, 2002}, {53, 109, 4, 2002}, {54, 110, 1, 2002}, {36, 89,
1, 2003}, {38, 92, 4, 2003}, {39, 93, 4, 2003}, {41, 96, 6, 2003}, {42, 97, 12, 2003},
{43, 98, 6, 2003}, {45, 101, 4, 2003}, {46, 102, 12, 2003}, {47, 103, 12, 2003}, {48, 104,
4, 2003}, {50, 107, 1, 2003}, {51, 108, 4, 2003}, {52, 109, 6, 2003}, {53, 110, 4, 2003},
{54, 111, 1, 2003}, {36, 90, 1, 2004}, {38, 94, 4, 2004}, {39, 95, 4, 2004}, {41, 99, 6,
2004}, {42, 100, 12, 2004}, {43, 101, 6, 2004}, {45, 105, 4, 2004}, {46, 106, 12, 2004},
{47, 107, 12, 2004}, {48, 108, 4, 2004}, {50, 112, 1, 2004}, {51, 113, 4, 2004}, {52, 114,
6, 2004}, {53, 115, 4, 2004}, {54, 116, 1, 2004}, {36, 91, 1, 2005}, {38, 95, 4, 2005},
{39, 96, 4, 2005}, {41, 100, 6, 2005}, {42, 101, 12, 2005}, {43, 102, 6, 2005}, {45, 106,
4, 2005}, {46, 107, 12, 2005}, {47, 108, 12, 2005}, {48, 109, 4, 2005}, {50, 113, 1,
2005}, {51, 114, 4, 2005}, {52, 115, 6, 2005}, {53, 116, 4, 2005}, {54, 117, 1, 2005},
{36, 92, 1, 2006}, {38, 96, 4, 2006}, {39, 97, 4, 2006}, {41, 101, 6, 2006}, {42, 102, 12,
2006}, {43, 103, 6, 2006}, {45, 107, 4, 2006}, {46, 108, 12, 2006}, {47, 109, 12, 2006},
{48, 110, 4, 2006}, {50, 114, 1, 2006}, {51, 115, 4, 2006}, {52, 116, 6, 2006}, {53, 117,
4, 2006}, {54, 118, 1, 2006}, {36, 93, 1, 2007}, {38, 97, 4, 2007}, {39, 98, 4, 2007},
{41, 102, 6, 2007}, {42, 103, 12, 2007}, {43, 104, 6, 2007}, {45, 108, 4, 2007}, {46, 109,
12, 2007}, {47, 110, 12, 2007}, {48, 111, 4, 2007}, {50, 115, 1, 2007}, {51, 116, 4,
2007}, {52, 117, 6, 2007}, {53, 118, 4, 2007}, {54, 119, 1, 2007}, {37, 84, 1, 2008}, {39,
85, 4, 2008}, {40, 86, 4, 2008}, {42, 87, 6, 2008}, {43, 88, 12, 2008}, {44, 89, 6, 2008},
{46, 90, 4, 2008}, {47, 91, 12, 2008}, {48, 92, 12, 2008}, {49, 93, 4, 2008}, {51, 94, 1,
2008}, {52, 95, 4, 2008}, {53, 96, 6, 2008}, {54, 97, 4, 2008}, {55, 98, 1, 2008}, {37,
85, 1, 2009}, {39, 87, 4, 2009}, {40, 88, 4, 2009}, {42, 90, 6, 2009}, {43, 91, 12, 2009},
{44, 92, 6, 2009}, {46, 94, 4, 2009}, {47, 95, 12, 2009}, {48, 96, 12, 2009}, {49, 97, 4,
2009}, {51, 99, 1, 2009}, {52, 100, 4, 2009}, {53, 101, 6, 2009}, {54, 102, 4, 2009}, {55,
103, 1, 2009}, {37, 86, 1, 2010}, {39, 88, 4, 2010}, {40, 89, 4, 2010}, {42, 91, 6, 2010},
{43, 92, 12, 2010}, {44, 93, 6, 2010}, {46, 95, 4, 2010}, {47, 96, 12, 2010}, {48, 97, 12,
2010}, {49, 98, 4, 2010}, {51, 100, 1, 2010}, {52, 101, 4, 2010}, {53, 102, 6, 2010}, {54,
103, 4, 2010}, {55, 104, 1, 2010}, {37, 87, 1, 2011}, {39, 90, 4, 2011}, {40, 91, 4,
2011}, {42, 94, 6, 2011}, {43, 95, 12, 2011}, {44, 96, 6, 2011}, {46, 99, 4, 2011}, {47,
100, 12, 2011}, {48, 101, 12, 2011}, {49, 102, 4, 2011}, {51, 105, 1, 2011}, {52, 106, 4,
2011}, {53, 107, 6, 2011}, {54, 108, 4, 2011}, {55, 109, 1, 2011}, {37, 88, 1, 2012}, {39,
91, 4, 2012}, {40, 92, 4, 2012}, {42, 95, 6, 2012}, {43, 96, 12, 2012}, {44, 97, 6, 2012},
{46, 100, 4, 2012}, {47, 101, 12, 2012}, {48, 102, 12, 2012}, {49, 103, 4, 2012}, {51,
106, 1, 2012}, {52, 107, 4, 2012}, {53, 108, 6, 2012}, {54, 109, 4, 2012}, {55, 110, 1,
2012}, {37, 89, 1, 2013}, {39, 92, 4, 2013}, {40, 93, 4, 2013}, {42, 96, 6, 2013}, {43,
97, 12, 2013}, {44, 98, 6, 2013}, {46, 101, 4, 2013}, {47, 102, 12, 2013}, {48, 103, 12,
2013}, {49, 104, 4, 2013}, {51, 107, 1, 2013}, {52, 108, 4, 2013}, {53, 109, 6, 2013},
{54, 110, 4, 2013}, {55, 111, 1, 2013}, {37, 90, 1, 2014}, {39, 94, 4, 2014}, {40, 95, 4,
2014}, {42, 99, 6, 2014}, {43, 100, 12, 2014}, {44, 101, 6, 2014}, {46, 105, 4, 2014},
{47, 106, 12, 2014}, {48, 107, 12, 2014}, {49, 108, 4, 2014}, {51, 112, 1, 2014}, {52,
113, 4, 2014}, {53, 114, 6, 2014}, {54, 115, 4, 2014}, {55, 116, 1, 2014}, {37, 91, 1,

2015}, {39, 95, 4, 2015}, {40, 96, 4, 2015}, {42, 100, 6, 2015}, {43, 101, 12, 2015}, {44, 102, 6, 2015}, {46, 106, 4, 2015}, {47, 107, 12, 2015}, {48, 108, 12, 2015}, {49, 109, 4, 2015}, {51, 113, 1, 2015}, {52, 114, 4, 2015}, {53, 115, 6, 2015}, {54, 116, 4, 2015}, {55, 117, 1, 2015}, {37, 92, 1, 2016}, {39, 96, 4, 2016}, {40, 97, 4, 2016}, {42, 101, 6, 2016}, {43, 102, 12, 2016}, {44, 103, 6, 2016}, {46, 107, 4, 2016}, {47, 108, 12, 2016}, {48, 109, 12, 2016}, {49, 110, 4, 2016}, {51, 114, 1, 2016}, {52, 115, 4, 2016}, {53, 116, 6, 2016}, {54, 117, 4, 2016}, {55, 118, 1, 2016}, {37, 93, 1, 2017}, {39, 97, 4, 2017}, {40, 98, 4, 2017}, {42, 102, 6, 2017}, {43, 103, 12, 2017}, {44, 104, 6, 2017}, {46, 108, 4, 2017}, {47, 109, 12, 2017}, {48, 110, 12, 2017}, {49, 111, 4, 2017}, {51, 115, 1, 2017}, {52, 116, 4, 2017}, {53, 117, 6, 2017}, {54, 118, 4, 2017}, {55, 119, 1, 2017}, {35, 120, 1, 2018}, {36, 121, 4, 2018}, {37, 122, 4, 2018}, {38, 123, 6, 2018}, {39, 124, 12, 2018}, {40, 125, 6, 2018}, {41, 126, 4, 2018}, {42, 127, 12, 2018}, {43, 128, 12, 2018}, {44, 129, 4, 2018}, {45, 130, 1, 2018}, {46, 131, 4, 2018}, {47, 132, 6, 2018}, {48, 133, 4, 2018}, {49, 134, 1, 2018}, {35, 121, 1, 2019}, {36, 123, 4, 2019}, {37, 124, 4, 2019}, {38, 126, 6, 2019}, {39, 127, 12, 2019}, {40, 128, 6, 2019}, {41, 130, 4, 2019}, {42, 131, 12, 2019}, {43, 132, 12, 2019}, {44, 133, 4, 2019}, {45, 135, 1, 2019}, {46, 136, 4, 2019}, {47, 137, 6, 2019}, {48, 138, 4, 2019}, {49, 139, 1, 2019}, {35, 122, 1, 2020}, {36, 124, 4, 2020}, {37, 125, 4, 2020}, {38, 127, 6, 2020}, {39, 128, 12, 2020}, {40, 129, 6, 2020}, {41, 131, 4, 2020}, {42, 132, 12, 2020}, {43, 133, 12, 2020}, {44, 134, 4, 2020}, {45, 136, 1, 2020}, {46, 137, 4, 2020}, {47, 138, 6, 2020}, {48, 139, 4, 2020}, {49, 140, 1, 2020}, {35, 123, 1, 2021}, {36, 126, 4, 2021}, {37, 127, 4, 2021}, {38, 130, 6, 2021}, {39, 131, 12, 2021}, {40, 132, 6, 2021}, {41, 135, 4, 2021}, {42, 136, 12, 2021}, {43, 137, 12, 2021}, {44, 138, 4, 2021}, {45, 141, 1, 2021}, {46, 142, 4, 2021}, {47, 143, 6, 2021}, {48, 144, 4, 2021}, {49, 145, 1, 2021}, {35, 124, 1, 2022}, {36, 127, 4, 2022}, {37, 128, 4, 2022}, {38, 131, 6, 2022}, {39, 132, 12, 2022}, {40, 133, 6, 2022}, {41, 136, 4, 2022}, {42, 137, 12, 2022}, {43, 138, 12, 2022}, {44, 139, 4, 2022}, {45, 142, 1, 2022}, {46, 143, 4, 2022}, {47, 144, 6, 2022}, {48, 145, 4, 2022}, {49, 146, 1, 2022}, {35, 125, 1, 2023}, {36, 128, 4, 2023}, {37, 129, 4, 2023}, {38, 132, 6, 2023}, {39, 133, 12, 2023}, {40, 134, 6, 2023}, {41, 137, 4, 2023}, {42, 138, 12, 2023}, {43, 139, 12, 2023}, {44, 140, 4, 2023}, {45, 143, 1, 2023}, {46, 144, 4, 2023}, {47, 145, 6, 2023}, {48, 146, 4, 2023}, {49, 147, 1, 2023}, {35, 126, 1, 2024}, {36, 130, 4, 2024}, {37, 131, 4, 2024}, {38, 135, 6, 2024}, {39, 136, 12, 2024}, {40, 137, 6, 2024}, {41, 141, 4, 2024}, {42, 142, 12, 2024}, {43, 143, 12, 2024}, {44, 144, 4, 2024}, {45, 148, 1, 2024}, {46, 149, 4, 2024}, {47, 150, 6, 2024}, {48, 151, 4, 2024}, {49, 152, 1, 2024}, {35, 127, 1, 2025}, {36, 131, 4, 2025}, {37, 132, 4, 2025}, {38, 136, 6, 2025}, {39, 137, 12, 2025}, {40, 138, 6, 2025}, {41, 142, 4, 2025}, {42, 143, 12, 2025}, {43, 144, 12, 2025}, {44, 145, 4, 2025}, {45, 149, 1, 2025}, {46, 150, 4, 2025}, {47, 151, 6, 2025}, {48, 152, 4, 2025}, {49, 153, 1, 2025}, {35, 128, 1, 2026}, {36, 132, 4, 2026}, {37, 133, 4, 2026}, {38, 137, 6, 2026}, {39, 138, 12, 2026}, {40, 139, 6, 2026}, {41, 143, 4, 2026}, {42, 144, 12, 2026}, {43, 145, 12, 2026}, {44, 146, 4, 2026}, {45, 150, 1, 2026}, {46, 151, 4, 2026}, {47, 152, 6, 2026}, {48, 153, 4, 2026}, {49, 154, 1, 2026}, {35, 129, 1, 2027}, {36, 133, 4, 2027}, {37, 134, 4, 2027}, {38, 138, 6, 2027}, {39, 139, 12, 2027}, {40, 140, 6, 2027}, {41, 144, 4, 2027}, {42, 145, 12, 2027}, {43, 146, 12, 2027}, {44, 147, 4, 2027}, {45, 151, 1, 2027}, {46, 152, 4, 2027}, {47, 153, 6, 2027}, {48, 154, 4, 2027}, {49, 155, 1, 2027}, {35, 130, 1, 2028}, {36, 135, 4, 2028}, {37, 136, 4, 2028}, {38, 141, 6, 2028}, {39, 142, 12, 2028}, {40, 143, 6, 2028}, {41, 148, 4, 2028}, {42, 149, 12, 2028}, {43, 150, 12, 2028}, {44, 151, 4, 2028}, {45, 156, 1, 2028}, {46, 157, 4, 2028}, {47, 158, 6, 2028}, {48, 159, 4, 2028}, {49, 160, 1, 2028}, {35, 131, 1, 2029}, {36, 136, 4, 2029}, {37, 137, 4, 2029}, {38, 142, 6, 2029}, {39, 143, 12, 2029}, {40, 144, 6, 2029}, {41, 149, 4, 2029}, {42, 150, 12, 2029}, {43, 151, 12, 2029}, {44, 152, 4, 2029}, {45, 157, 1, 2029}, {46, 158, 4, 2029}, {47, 159, 6, 2029}, {48, 160, 4, 2029}, {49, 161, 1, 2029}, {35, 132, 1, 2030}, {36, 137, 4, 2030}, {37, 138, 4, 2030}, {38, 143, 6, 2030}, {39, 144, 12, 2030}, {40, 145, 6, 2030}, {41, 150, 4, 2030}, {42, 151, 12, 2030}, {43, 152, 12, 2030}, {44, 153, 4, 2030}, {45, 158, 1, 2030}, {46, 159, 4, 2030}, {47, 160, 6, 2030}, {48, 161, 4, 2030}, {49, 162, 1, 2030}, {35, 133, 1, 2031}, {36, 138, 4, 2031}, {37, 139, 4, 2031}, {38, 144, 6, 2031}, {39, 145, 12, 2031}, {40, 146, 6, 2031}, {41, 151, 4, 2031}, {42, 152, 12, 2031}, {43, 153, 12, 2031}, {44, 154, 4, 2031}, {45, 159, 1, 2031}, {46, 160, 4, 2031}, {47, 161, 6, 2031}, {48, 162, 4, 2031}, {49, 163, 1, 2031}, {35, 134, 1, 2032}, {36, 139, 4, 2032}, {37, 140, 4, 2032}, {38, 145, 6, 2032}, {39, 146, 12, 2032}, {40, 147, 6, 2032}, {41, 152, 4, 2032}, {42, 153, 12, 2032}, {43, 154, 12, 2032}, {44, 155, 4, 2032}, {45, 160, 1, 2032}, {46, 161, 4, 2032}, {47, 162, 6, 2032}, {48, 163, 4, 2032}, {49, 164, 1, 2032}, {36, 120, 1, 2033}, {38, 121, 4, 2033}, {39, 122, 4, 2033}, {41, 123, 6, 2033}, {42, 124, 12, 2033}, {43, 125, 6, 2033}, {45, 126, 4, 2033}, {46, 127, 12, 2033}, {47, 128, 12, 2033}, {48, 129, 4, 2033}, {50, 130, 1, 2033}, {51, 131, 4, 2033}, {52, 132, 6, 2033}, {53, 133, 4, 2033}, {54, 134, 1, 2033}, {36, 121, 1, 2034}, {38, 123, 4, 2034}, {39, 124, 4, 2034}, {41, 126, 6, 2034}, {42, 127, 12, 2034}, {43, 128, 6, 2034}, {45, 130, 4, 2034}, {46, 131, 12, 2034}, {47, 132, 12, 2034}, {48, 133, 4, 2034}, {50, 135, 1, 2034}, {51, 136, 4, 2034}, {52, 137, 6, 2034}, {53, 138, 4, 2034}, {54, 139, 1, 2034}, {36, 122, 1, 2035}, {38, 124, 4, 2035},

{39, 125, 4, 2035}, {41, 127, 6, 2035}, {42, 128, 12, 2035}, {43, 129, 6, 2035}, {45, 131, 4, 2035}, {46, 132, 12, 2035}, {47, 133, 12, 2035}, {48, 134, 4, 2035}, {50, 136, 1, 2035}, {51, 137, 4, 2035}, {52, 138, 6, 2035}, {53, 139, 4, 2035}, {54, 140, 1, 2035}, {36, 123, 1, 2036}, {38, 126, 4, 2036}, {39, 127, 4, 2036}, {41, 130, 6, 2036}, {42, 131, 12, 2036}, {43, 132, 6, 2036}, {45, 135, 4, 2036}, {46, 136, 12, 2036}, {47, 137, 12, 2036}, {48, 138, 4, 2036}, {50, 141, 1, 2036}, {51, 142, 4, 2036}, {52, 143, 6, 2036}, {53, 144, 4, 2036}, {54, 145, 1, 2036}, {36, 124, 1, 2037}, {38, 127, 4, 2037}, {39, 128, 4, 2037}, {41, 131, 6, 2037}, {42, 132, 12, 2037}, {43, 133, 6, 2037}, {45, 136, 4, 2037}, {46, 137, 12, 2037}, {47, 138, 12, 2037}, {48, 139, 4, 2037}, {50, 142, 1, 2037}, {51, 143, 4, 2037}, {52, 144, 6, 2037}, {53, 145, 4, 2037}, {54, 146, 1, 2037}, {36, 125, 1, 2038}, {38, 128, 4, 2038}, {39, 129, 4, 2038}, {41, 132, 6, 2038}, {42, 133, 12, 2038}, {43, 134, 6, 2038}, {45, 137, 4, 2038}, {46, 138, 12, 2038}, {47, 139, 12, 2038}, {48, 140, 4, 2038}, {50, 143, 1, 2038}, {51, 144, 4, 2038}, {52, 145, 6, 2038}, {53, 146, 4, 2038}, {54, 147, 1, 2038}, {36, 126, 1, 2039}, {38, 130, 4, 2039}, {39, 131, 4, 2039}, {41, 135, 6, 2039}, {42, 136, 12, 2039}, {43, 137, 6, 2039}, {45, 141, 4, 2039}, {46, 142, 12, 2039}, {47, 143, 12, 2039}, {48, 144, 4, 2039}, {50, 148, 1, 2039}, {51, 149, 4, 2039}, {52, 150, 6, 2039}, {53, 151, 4, 2039}, {54, 152, 1, 2039}, {36, 127, 1, 2040}, {38, 131, 4, 2040}, {39, 132, 4, 2040}, {41, 136, 6, 2040}, {42, 137, 12, 2040}, {43, 138, 6, 2040}, {45, 142, 4, 2040}, {46, 143, 12, 2040}, {47, 144, 12, 2040}, {48, 145, 4, 2040}, {50, 149, 1, 2040}, {51, 150, 4, 2040}, {52, 151, 6, 2040}, {53, 152, 4, 2040}, {54, 153, 1, 2040}, {36, 128, 1, 2041}, {38, 132, 4, 2041}, {39, 133, 4, 2041}, {41, 137, 6, 2041}, {42, 138, 12, 2041}, {43, 139, 6, 2041}, {45, 143, 4, 2041}, {46, 144, 12, 2041}, {47, 145, 12, 2041}, {48, 146, 4, 2041}, {50, 150, 1, 2041}, {51, 151, 4, 2041}, {52, 152, 6, 2041}, {53, 153, 4, 2041}, {54, 154, 1, 2041}, {36, 129, 1, 2042}, {38, 133, 4, 2042}, {39, 134, 4, 2042}, {41, 138, 6, 2042}, {42, 139, 12, 2042}, {43, 140, 6, 2042}, {45, 144, 4, 2042}, {46, 145, 12, 2042}, {47, 146, 12, 2042}, {48, 147, 4, 2042}, {50, 151, 1, 2042}, {51, 152, 4, 2042}, {52, 153, 6, 2042}, {53, 154, 4, 2042}, {54, 155, 1, 2042}, {36, 130, 1, 2043}, {38, 135, 4, 2043}, {39, 136, 4, 2043}, {41, 141, 6, 2043}, {42, 142, 12, 2043}, {43, 143, 6, 2043}, {45, 148, 4, 2043}, {46, 149, 12, 2043}, {47, 150, 12, 2043}, {48, 151, 4, 2043}, {50, 156, 1, 2043}, {51, 157, 4, 2043}, {52, 158, 6, 2043}, {53, 159, 4, 2043}, {54, 160, 1, 2043}, {36, 131, 1, 2044}, {38, 136, 4, 2044}, {39, 137, 4, 2044}, {41, 142, 6, 2044}, {42, 143, 12, 2044}, {43, 144, 6, 2044}, {45, 149, 4, 2044}, {46, 150, 12, 2044}, {47, 151, 12, 2044}, {48, 152, 4, 2044}, {50, 157, 1, 2044}, {51, 158, 4, 2044}, {52, 159, 6, 2044}, {53, 160, 4, 2044}, {54, 161, 1, 2044}, {36, 132, 1, 2045}, {38, 137, 4, 2045}, {39, 138, 4, 2045}, {41, 143, 6, 2045}, {42, 144, 12, 2045}, {43, 145, 6, 2045}, {45, 150, 4, 2045}, {46, 151, 12, 2045}, {47, 152, 12, 2045}, {48, 153, 4, 2045}, {50, 158, 1, 2045}, {51, 159, 4, 2045}, {52, 160, 6, 2045}, {53, 161, 4, 2045}, {54, 162, 1, 2045}, {36, 133, 1, 2046}, {38, 138, 4, 2046}, {39, 139, 4, 2046}, {41, 144, 6, 2046}, {42, 145, 12, 2046}, {43, 146, 6, 2046}, {45, 151, 4, 2046}, {46, 152, 12, 2046}, {47, 153, 12, 2046}, {48, 154, 4, 2046}, {50, 159, 1, 2046}, {51, 160, 4, 2046}, {52, 161, 6, 2046}, {53, 162, 4, 2046}, {54, 163, 1, 2046}, {36, 134, 1, 2047}, {38, 139, 4, 2047}, {39, 140, 4, 2047}, {41, 145, 6, 2047}, {42, 146, 12, 2047}, {43, 147, 6, 2047}, {45, 152, 4, 2047}, {46, 153, 12, 2047}, {47, 154, 12, 2047}, {48, 155, 4, 2047}, {50, 160, 1, 2047}, {51, 161, 4, 2047}, {52, 162, 6, 2047}, {53, 163, 4, 2047}, {54, 164, 1, 2047}, {37, 120, 1, 2048}, {39, 121, 4, 2048}, {40, 122, 4, 2048}, {42, 123, 6, 2048}, {43, 124, 12, 2048}, {44, 125, 6, 2048}, {46, 126, 4, 2048}, {47, 127, 12, 2048}, {48, 128, 12, 2048}, {49, 129, 4, 2048}, {51, 130, 1, 2048}, {52, 131, 4, 2048}, {53, 132, 6, 2048}, {54, 133, 4, 2048}, {55, 134, 1, 2048}, {37, 121, 1, 2049}, {39, 123, 4, 2049}, {40, 124, 4, 2049}, {42, 126, 6, 2049}, {43, 127, 12, 2049}, {44, 128, 6, 2049}, {46, 130, 4, 2049}, {47, 131, 12, 2049}, {48, 132, 12, 2049}, {49, 133, 4, 2049}, {51, 135, 1, 2049}, {52, 136, 4, 2049}, {53, 137, 6, 2049}, {54, 138, 4, 2049}, {55, 139, 1, 2049}, {37, 122, 1, 2050}, {39, 124, 4, 2050}, {40, 125, 4, 2050}, {42, 127, 6, 2050}, {43, 128, 12, 2050}, {44, 129, 6, 2050}, {46, 131, 4, 2050}, {47, 132, 12, 2050}, {48, 133, 12, 2050}, {49, 134, 4, 2050}, {51, 136, 1, 2050}, {52, 137, 4, 2050}, {53, 138, 6, 2050}, {54, 139, 4, 2050}, {55, 140, 1, 2050}, {37, 123, 1, 2051}, {39, 126, 4, 2051}, {40, 127, 4, 2051}, {42, 130, 6, 2051}, {43, 131, 12, 2051}, {44, 132, 6, 2051}, {46, 135, 4, 2051}, {47, 136, 12, 2051}, {48, 137, 12, 2051}, {49, 138, 4, 2051}, {51, 141, 1, 2051}, {52, 142, 4, 2051}, {53, 143, 6, 2051}, {54, 144, 4, 2051}, {55, 145, 1, 2051}, {37, 124, 1, 2052}, {39, 127, 4, 2052}, {40, 128, 4, 2052}, {42, 131, 6, 2052}, {43, 132, 12, 2052}, {44, 133, 6, 2052}, {46, 136, 4, 2052}, {47, 137, 12, 2052}, {48, 138, 12, 2052}, {49, 139, 4, 2052}, {51, 142, 1, 2052}, {52, 143, 4, 2052}, {53, 144, 6, 2052}, {54, 145, 4, 2052}, {55, 146, 1, 2052}, {37, 125, 1, 2053}, {39, 128, 4, 2053}, {40, 129, 4, 2053}, {42, 132, 6, 2053}, {43, 133, 12, 2053}, {44, 134, 6, 2053}, {46, 137, 4, 2053}, {47, 138, 12, 2053}, {48, 139, 12, 2053}, {49, 140, 4, 2053}, {51, 143, 1, 2053}, {52, 144, 4, 2053}, {53, 145, 6, 2053}, {54, 146, 4, 2053}, {55, 147, 1, 2053}, {37, 126, 1, 2054}, {39, 130, 4, 2054}, {40, 131, 4, 2054}, {42, 135, 6, 2054}, {43, 136, 12, 2054}, {44, 137, 6, 2054}, {46, 141, 4, 2054}, {47, 142, 12, 2054}, {48, 143, 12, 2054}, {49, 144, 4, 2054}, {51, 148, 1, 2054}, {52, 149, 4, 2054}, {53, 150, 6, 2054}, {54, 151, 4, 2054}, {55, 152, 1, 2054}, {37, 127, 1, 2055}, {39, 131, 4, 2055}, {40, 132,

4, 2055}, {42, 136, 6, 2055}, {43, 137, 12, 2055}, {44, 138, 6, 2055}, {46, 142, 4, 2055}, {47, 143, 12, 2055}, {48, 144, 12, 2055}, {49, 145, 4, 2055}, {51, 149, 1, 2055}, {52, 150, 4, 2055}, {53, 151, 6, 2055}, {54, 152, 4, 2055}, {55, 153, 1, 2055}, {37, 128, 1, 2056}, {39, 132, 4, 2056}, {40, 133, 4, 2056}, {42, 137, 6, 2056}, {43, 138, 12, 2056}, {44, 139, 6, 2056}, {46, 143, 4, 2056}, {47, 144, 12, 2056}, {48, 145, 12, 2056}, {49, 146, 4, 2056}, {51, 150, 1, 2056}, {52, 151, 4, 2056}, {53, 152, 6, 2056}, {54, 153, 4, 2056}, {55, 154, 1, 2056}, {37, 129, 1, 2057}, {39, 133, 4, 2057}, {40, 134, 4, 2057}, {42, 138, 6, 2057}, {43, 139, 12, 2057}, {44, 140, 6, 2057}, {46, 144, 4, 2057}, {47, 145, 12, 2057}, {48, 146, 12, 2057}, {49, 147, 4, 2057}, {51, 151, 1, 2057}, {52, 152, 4, 2057}, {53, 153, 6, 2057}, {54, 154, 4, 2057}, {55, 155, 1, 2057}, {37, 130, 1, 2058}, {39, 135, 4, 2058}, {40, 136, 4, 2058}, {42, 141, 6, 2058}, {43, 142, 12, 2058}, {44, 143, 6, 2058}, {46, 148, 4, 2058}, {47, 149, 12, 2058}, {48, 150, 12, 2058}, {49, 151, 4, 2058}, {51, 156, 1, 2058}, {52, 157, 4, 2058}, {53, 158, 6, 2058}, {54, 159, 4, 2058}, {55, 160, 1, 2058}, {37, 131, 1, 2059}, {39, 136, 4, 2059}, {40, 137, 4, 2059}, {42, 142, 6, 2059}, {43, 143, 12, 2059}, {44, 144, 6, 2059}, {46, 149, 4, 2059}, {47, 150, 12, 2059}, {48, 151, 12, 2059}, {49, 152, 4, 2059}, {51, 157, 1, 2059}, {52, 158, 4, 2059}, {53, 159, 6, 2059}, {54, 160, 4, 2059}, {55, 161, 1, 2059}, {37, 132, 1, 2060}, {39, 137, 4, 2060}, {40, 138, 4, 2060}, {42, 143, 6, 2060}, {43, 144, 12, 2060}, {44, 145, 6, 2060}, {46, 150, 4, 2060}, {47, 151, 12, 2060}, {48, 152, 12, 2060}, {49, 153, 4, 2060}, {51, 158, 1, 2060}, {52, 159, 4, 2060}, {53, 160, 6, 2060}, {54, 161, 4, 2060}, {55, 162, 1, 2060}, {37, 133, 1, 2061}, {39, 138, 4, 2061}, {40, 139, 4, 2061}, {42, 144, 6, 2061}, {43, 145, 12, 2061}, {44, 146, 6, 2061}, {46, 151, 4, 2061}, {47, 152, 12, 2061}, {48, 153, 12, 2061}, {49, 154, 4, 2061}, {51, 159, 1, 2061}, {52, 160, 4, 2061}, {53, 161, 6, 2061}, {54, 162, 4, 2061}, {55, 163, 1, 2061}, {37, 134, 1, 2062}, {39, 139, 4, 2062}, {40, 140, 4, 2062}, {42, 145, 6, 2062}, {43, 146, 12, 2062}, {44, 147, 6, 2062}, {46, 152, 4, 2062}, {47, 153, 12, 2062}, {48, 154, 12, 2062}, {49, 155, 4, 2062}, {51, 160, 1, 2062}, {52, 161, 4, 2062}, {53, 162, 6, 2062}, {54, 163, 4, 2062}, {55, 164, 1, 2062}, {35, 35, 1, 2063}, {36, 36, 5, 2063}, {37, 37, 5, 2063}, {38, 38, 10, 2063}, {39, 39, 20, 2063}, {40, 40, 10, 2063}, {41, 41, 10, 2063}, {42, 42, 30, 2063}, {43, 43, 30, 2063}, {44, 44, 10, 2063}, {45, 45, 5, 2063}, {46, 46, 20, 2063}, {47, 47, 30, 2063}, {48, 48, 20, 2063}, {49, 49, 5, 2063}, {50, 50, 1, 2063}, {51, 51, 5, 2063}, {52, 52, 10, 2063}, {53, 53, 10, 2063}, {54, 54, 5, 2063}, {55, 55, 1, 2063}, {35, 56, 1, 2064}, {36, 57, 5, 2064}, {37, 58, 5, 2064}, {38, 59, 10, 2064}, {39, 60, 20, 2064}, {40, 61, 10, 2064}, {41, 62, 10, 2064}, {42, 63, 30, 2064}, {43, 64, 30, 2064}, {44, 65, 10, 2064}, {45, 66, 5, 2064}, {46, 67, 20, 2064}, {47, 68, 30, 2064}, {48, 69, 20, 2064}, {49, 70, 5, 2064}, {50, 71, 1, 2064}, {51, 72, 5, 2064}, {52, 73, 10, 2064}, {53, 74, 10, 2064}, {54, 75, 5, 2064}, {55, 76, 1, 2064}, {35, 57, 1, 2065}, {36, 59, 5, 2065}, {37, 60, 5, 2065}, {38, 62, 10, 2065}, {39, 63, 20, 2065}, {40, 64, 10, 2065}, {41, 66, 10, 2065}, {42, 67, 30, 2065}, {43, 68, 30, 2065}, {44, 69, 10, 2065}, {45, 71, 5, 2065}, {46, 72, 20, 2065}, {47, 73, 30, 2065}, {48, 74, 20, 2065}, {49, 75, 5, 2065}, {50, 77, 1, 2065}, {51, 78, 5, 2065}, {52, 79, 10, 2065}, {53, 80, 10, 2065}, {54, 81, 5, 2065}, {55, 82, 1, 2065}, {35, 58, 1, 2066}, {36, 60, 5, 2066}, {37, 61, 5, 2066}, {38, 63, 10, 2066}, {39, 64, 20, 2066}, {40, 65, 10, 2066}, {41, 67, 10, 2066}, {42, 68, 30, 2066}, {43, 69, 30, 2066}, {44, 70, 10, 2066}, {45, 72, 5, 2066}, {46, 73, 20, 2066}, {47, 74, 30, 2066}, {48, 75, 20, 2066}, {49, 76, 5, 2066}, {50, 78, 1, 2066}, {51, 79, 5, 2066}, {52, 80, 10, 2066}, {53, 81, 10, 2066}, {54, 82, 5, 2066}, {55, 83, 1, 2066}, {35, 84, 1, 2067}, {36, 85, 5, 2067}, {37, 86, 5, 2067}, {38, 87, 10, 2067}, {39, 88, 20, 2067}, {40, 89, 10, 2067}, {41, 90, 10, 2067}, {42, 91, 30, 2067}, {43, 92, 30, 2067}, {44, 93, 10, 2067}, {45, 94, 5, 2067}, {46, 95, 20, 2067}, {47, 96, 30, 2067}, {48, 97, 20, 2067}, {49, 98, 5, 2067}, {50, 99, 1, 2067}, {51, 100, 5, 2067}, {52, 101, 10, 2067}, {53, 102, 10, 2067}, {54, 103, 5, 2067}, {55, 104, 1, 2067}, {35, 85, 1, 2068}, {36, 87, 5, 2068}, {37, 88, 5, 2068}, {38, 90, 10, 2068}, {39, 91, 20, 2068}, {40, 92, 10, 2068}, {41, 94, 10, 2068}, {42, 95, 30, 2068}, {43, 96, 30, 2068}, {44, 97, 10, 2068}, {45, 99, 5, 2068}, {46, 100, 20, 2068}, {47, 101, 30, 2068}, {48, 102, 20, 2068}, {49, 103, 5, 2068}, {50, 105, 1, 2068}, {51, 106, 5, 2068}, {52, 107, 10, 2068}, {53, 108, 10, 2068}, {54, 109, 5, 2068}, {55, 110, 1, 2068}, {35, 86, 1, 2069}, {36, 88, 5, 2069}, {37, 89, 5, 2069}, {38, 91, 10, 2069}, {39, 92, 20, 2069}, {40, 93, 10, 2069}, {41, 95, 10, 2069}, {42, 96, 30, 2069}, {43, 97, 30, 2069}, {44, 98, 10, 2069}, {45, 100, 5, 2069}, {46, 101, 20, 2069}, {47, 102, 30, 2069}, {48, 103, 20, 2069}, {49, 104, 5, 2069}, {50, 106, 1, 2069}, {51, 107, 5, 2069}, {52, 108, 10, 2069}, {53, 109, 10, 2069}, {54, 110, 5, 2069}, {55, 111, 1, 2069}, {35, 87, 1, 2070}, {36, 90, 5, 2070}, {37, 91, 5, 2070}, {38, 94, 10, 2070}, {39, 95, 20, 2070}, {40, 96, 10, 2070}, {41, 99, 10, 2070}, {42, 100, 30, 2070}, {43, 101, 30, 2070}, {44, 102, 10, 2070}, {45, 105, 5, 2070}, {46, 106, 20, 2070}, {47, 107, 30, 2070}, {48, 108, 20, 2070}, {49, 109, 5, 2070}, {50, 112, 1, 2070}, {51, 113, 5, 2070}, {52, 114, 10, 2070}, {53, 115, 10, 2070}, {54, 116, 5, 2070}, {55, 117, 1, 2070}, {35, 88, 1, 2071}, {36, 91, 5, 2071}, {37, 92, 5, 2071}, {38, 95, 10, 2071}, {39, 96, 20, 2071}, {40, 97, 10, 2071}, {41, 100, 10, 2071}, {42, 101, 30, 2071}, {43, 102, 30, 2071}, {44, 103, 10, 2071}, {45, 106, 5, 2071}, {46, 107, 20, 2071}, {47, 108, 30, 2071}, {48, 109, 20, 2071}, {49, 110, 5, 2071}, {50, 113, 1, 2071}, {51, 114, 5, 2071}, {52, 115, 10, 2071}, {53, 116, 10, 2071}, {54, 117, 5,

2071}, {55, 118, 1, 2071}, {35, 89, 1, 2072}, {36, 92, 5, 2072}, {37, 93, 5, 2072}, {38, 96, 10, 2072}, {39, 97, 20, 2072}, {40, 98, 10, 2072}, {41, 101, 10, 2072}, {42, 102, 30, 2072}, {43, 103, 30, 2072}, {44, 104, 10, 2072}, {45, 107, 5, 2072}, {46, 108, 20, 2072}, {47, 109, 30, 2072}, {48, 110, 20, 2072}, {49, 111, 5, 2072}, {50, 114, 1, 2072}, {51, 115, 5, 2072}, {52, 116, 10, 2072}, {53, 117, 10, 2072}, {54, 118, 5, 2072}, {55, 119, 1, 2072}, {35, 120, 1, 2073}, {36, 121, 5, 2073}, {37, 122, 5, 2073}, {38, 123, 10, 2073}, {39, 124, 20, 2073}, {40, 125, 10, 2073}, {41, 126, 10, 2073}, {42, 127, 30, 2073}, {43, 128, 30, 2073}, {44, 129, 10, 2073}, {45, 130, 5, 2073}, {46, 131, 20, 2073}, {47, 132, 30, 2073}, {48, 133, 20, 2073}, {49, 134, 5, 2073}, {50, 135, 1, 2073}, {51, 136, 5, 2073}, {52, 137, 10, 2073}, {53, 138, 10, 2073}, {54, 139, 5, 2073}, {55, 140, 1, 2073}, {35, 121, 1, 2074}, {36, 123, 5, 2074}, {37, 124, 5, 2074}, {38, 126, 10, 2074}, {39, 127, 20, 2074}, {40, 128, 10, 2074}, {41, 130, 10, 2074}, {42, 131, 30, 2074}, {43, 132, 30, 2074}, {44, 133, 10, 2074}, {45, 135, 5, 2074}, {46, 136, 20, 2074}, {47, 137, 30, 2074}, {48, 138, 20, 2074}, {49, 139, 5, 2074}, {50, 141, 1, 2074}, {51, 142, 5, 2074}, {52, 143, 10, 2074}, {53, 144, 10, 2074}, {54, 145, 5, 2074}, {55, 146, 1, 2074}, {35, 122, 1, 2075}, {36, 124, 5, 2075}, {37, 125, 5, 2075}, {38, 127, 10, 2075}, {39, 128, 20, 2075}, {40, 129, 10, 2075}, {41, 131, 10, 2075}, {42, 132, 30, 2075}, {43, 133, 30, 2075}, {44, 134, 10, 2075}, {45, 136, 5, 2075}, {46, 137, 20, 2075}, {47, 138, 30, 2075}, {48, 139, 20, 2075}, {49, 140, 5, 2075}, {50, 142, 1, 2075}, {51, 143, 5, 2075}, {52, 144, 10, 2075}, {53, 145, 10, 2075}, {54, 146, 5, 2075}, {55, 147, 1, 2075}, {35, 123, 1, 2076}, {36, 126, 5, 2076}, {37, 127, 5, 2076}, {38, 130, 10, 2076}, {39, 131, 20, 2076}, {40, 132, 10, 2076}, {41, 135, 10, 2076}, {42, 136, 30, 2076}, {43, 137, 30, 2076}, {44, 138, 10, 2076}, {45, 141, 5, 2076}, {46, 142, 20, 2076}, {47, 143, 30, 2076}, {48, 144, 20, 2076}, {49, 145, 5, 2076}, {50, 148, 1, 2076}, {51, 149, 5, 2076}, {52, 150, 10, 2076}, {53, 151, 10, 2076}, {54, 152, 5, 2076}, {55, 153, 1, 2076}, {35, 124, 1, 2077}, {36, 127, 5, 2077}, {37, 128, 5, 2077}, {38, 131, 10, 2077}, {39, 132, 20, 2077}, {40, 133, 10, 2077}, {41, 136, 10, 2077}, {42, 137, 30, 2077}, {43, 138, 30, 2077}, {44, 139, 10, 2077}, {45, 142, 5, 2077}, {46, 143, 20, 2077}, {47, 144, 30, 2077}, {48, 145, 20, 2077}, {49, 146, 5, 2077}, {50, 149, 1, 2077}, {51, 150, 5, 2077}, {52, 151, 10, 2077}, {53, 152, 10, 2077}, {54, 153, 5, 2077}, {55, 154, 1, 2077}, {35, 125, 1, 2078}, {36, 128, 5, 2078}, {37, 129, 5, 2078}, {38, 132, 10, 2078}, {39, 133, 20, 2078}, {40, 134, 10, 2078}, {41, 137, 10, 2078}, {42, 138, 30, 2078}, {43, 139, 30, 2078}, {44, 140, 10, 2078}, {45, 143, 5, 2078}, {46, 144, 20, 2078}, {47, 145, 30, 2078}, {48, 146, 20, 2078}, {49, 147, 5, 2078}, {50, 150, 1, 2078}, {51, 151, 5, 2078}, {52, 152, 10, 2078}, {53, 153, 10, 2078}, {54, 154, 5, 2078}, {55, 155, 1, 2078}, {35, 126, 1, 2079}, {36, 130, 5, 2079}, {37, 131, 5, 2079}, {38, 135, 10, 2079}, {39, 136, 20, 2079}, {40, 137, 10, 2079}, {41, 141, 10, 2079}, {42, 142, 30, 2079}, {43, 143, 30, 2079}, {44, 144, 10, 2079}, {45, 148, 5, 2079}, {46, 149, 20, 2079}, {47, 150, 30, 2079}, {48, 151, 20, 2079}, {49, 152, 5, 2079}, {50, 156, 1, 2079}, {51, 157, 5, 2079}, {52, 158, 10, 2079}, {53, 159, 10, 2079}, {54, 160, 5, 2079}, {55, 161, 1, 2079}, {35, 127, 1, 2080}, {36, 131, 5, 2080}, {37, 132, 5, 2080}, {38, 136, 10, 2080}, {39, 137, 20, 2080}, {40, 138, 10, 2080}, {41, 142, 10, 2080}, {42, 143, 30, 2080}, {43, 144, 30, 2080}, {44, 145, 10, 2080}, {45, 149, 5, 2080}, {46, 150, 20, 2080}, {47, 151, 30, 2080}, {48, 152, 20, 2080}, {49, 153, 5, 2080}, {50, 157, 1, 2080}, {51, 158, 5, 2080}, {52, 159, 10, 2080}, {53, 160, 10, 2080}, {54, 161, 5, 2080}, {55, 162, 1, 2080}, {35, 128, 1, 2081}, {36, 132, 5, 2081}, {37, 133, 5, 2081}, {38, 137, 10, 2081}, {39, 138, 20, 2081}, {40, 139, 10, 2081}, {41, 143, 10, 2081}, {42, 144, 30, 2081}, {43, 145, 30, 2081}, {44, 146, 10, 2081}, {45, 150, 5, 2081}, {46, 151, 20, 2081}, {47, 152, 30, 2081}, {48, 153, 20, 2081}, {49, 154, 5, 2081}, {50, 158, 1, 2081}, {51, 159, 5, 2081}, {52, 160, 10, 2081}, {53, 161, 10, 2081}, {54, 162, 5, 2081}, {55, 163, 1, 2081}, {35, 129, 1, 2082}, {36, 133, 5, 2082}, {37, 134, 5, 2082}, {38, 138, 10, 2082}, {39, 139, 20, 2082}, {40, 140, 10, 2082}, {41, 144, 10, 2082}, {42, 145, 30, 2082}, {43, 146, 30, 2082}, {44, 147, 10, 2082}, {45, 151, 5, 2082}, {46, 152, 20, 2082}, {47, 153, 30, 2082}, {48, 154, 20, 2082}, {49, 155, 5, 2082}, {50, 159, 1, 2082}, {51, 160, 5, 2082}, {52, 161, 10, 2082}, {53, 162, 10, 2082}, {54, 163, 5, 2082}, {55, 164, 1, 2082}, {35, 165, 1, 2083}, {36, 166, 5, 2083}, {37, 167, 5, 2083}, {38, 168, 10, 2083}, {39, 169, 20, 2083}, {40, 170, 10, 2083}, {41, 171, 10, 2083}, {42, 172, 30, 2083}, {43, 173, 30, 2083}, {44, 174, 10, 2083}, {45, 175, 5, 2083}, {46, 176, 20, 2083}, {47, 177, 30, 2083}, {48, 178, 20, 2083}, {49, 179, 5, 2083}, {50, 180, 1, 2083}, {51, 181, 5, 2083}, {52, 182, 10, 2083}, {53, 183, 10, 2083}, {54, 184, 5, 2083}, {55, 185, 1, 2083}, {35, 166, 1, 2084}, {36, 168, 5, 2084}, {37, 169, 5, 2084}, {38, 171, 10, 2084}, {39, 172, 20, 2084}, {40, 173, 10, 2084}, {41, 175, 10, 2084}, {42, 176, 30, 2084}, {43, 177, 30, 2084}, {44, 178, 10, 2084}, {45, 180, 5, 2084}, {46, 181, 20, 2084}, {47, 182, 30, 2084}, {48, 183, 20, 2084}, {49, 184, 5, 2084}, {50, 186, 1, 2084}, {51, 187, 5, 2084}, {52, 188, 10, 2084}, {53, 189, 10, 2084}, {54, 190, 5, 2084}, {55, 191, 1, 2084}, {35, 167, 1, 2085}, {36, 169, 5, 2085}, {37, 170, 5, 2085}, {38, 172, 10, 2085}, {39, 173, 20, 2085}, {40, 174, 10, 2085}, {41, 176, 10, 2085}, {42, 177, 30, 2085}, {43, 178, 30, 2085}, {44, 179, 10, 2085}, {45, 181, 5, 2085}, {46, 182, 20, 2085}, {47, 183, 30, 2085}, {48, 184, 20, 2085}, {49, 185, 5, 2085}, {50, 187, 1, 2085}, {51, 188, 5, 2085}, {52, 189, 10, 2085}, {53, 190, 10, 2085}, {54, 191, 5, 2085}, {55, 192, 1, 2085}, {35, 168, 1, 2086}, {36, 171,

5, 2086}, {37, 172, 5, 2086}, {38, 175, 10, 2086}, {39, 176, 20, 2086}, {40, 177, 10,
2086}, {41, 180, 10, 2086}, {42, 181, 30, 2086}, {43, 182, 30, 2086}, {44, 183, 10, 2086},
{45, 186, 5, 2086}, {46, 187, 20, 2086}, {47, 188, 30, 2086}, {48, 189, 20, 2086}, {49,
190, 5, 2086}, {50, 193, 1, 2086}, {51, 194, 5, 2086}, {52, 195, 10, 2086}, {53, 196, 10,
2086}, {54, 197, 5, 2086}, {55, 198, 1, 2086}, {35, 169, 1, 2087}, {36, 172, 5, 2087},
{37, 173, 5, 2087}, {38, 176, 10, 2087}, {39, 177, 20, 2087}, {40, 178, 10, 2087}, {41,
181, 10, 2087}, {42, 182, 30, 2087}, {43, 183, 30, 2087}, {44, 184, 10, 2087}, {45, 187,
5, 2087}, {46, 188, 20, 2087}, {47, 189, 30, 2087}, {48, 190, 20, 2087}, {49, 191, 5,
2087}, {50, 194, 1, 2087}, {51, 195, 5, 2087}, {52, 196, 10, 2087}, {53, 197, 10, 2087},
{54, 198, 5, 2087}, {55, 199, 1, 2087}, {35, 170, 1, 2088}, {36, 173, 5, 2088}, {37, 174,
5, 2088}, {38, 177, 10, 2088}, {39, 178, 20, 2088}, {40, 179, 10, 2088}, {41, 182, 10,
2088}, {42, 183, 30, 2088}, {43, 184, 30, 2088}, {44, 185, 10, 2088}, {45, 188, 5, 2088},
{46, 189, 20, 2088}, {47, 190, 30, 2088}, {48, 191, 20, 2088}, {49, 192, 5, 2088}, {50,
195, 1, 2088}, {51, 196, 5, 2088}, {52, 197, 10, 2088}, {53, 198, 10, 2088}, {54, 199, 5,
2088}, {55, 200, 1, 2088}, {35, 171, 1, 2089}, {36, 175, 5, 2089}, {37, 176, 5, 2089},
{38, 180, 10, 2089}, {39, 181, 20, 2089}, {40, 182, 10, 2089}, {41, 186, 10, 2089}, {42,
187, 30, 2089}, {43, 188, 30, 2089}, {44, 189, 10, 2089}, {45, 193, 5, 2089}, {46, 194,
20, 2089}, {47, 195, 30, 2089}, {48, 196, 20, 2089}, {49, 197, 5, 2089}, {50, 201, 1,
2089}, {51, 202, 5, 2089}, {52, 203, 10, 2089}, {53, 204, 10, 2089}, {54, 205, 5, 2089},
{55, 206, 1, 2089}, {35, 172, 1, 2090}, {36, 176, 5, 2090}, {37, 177, 5, 2090}, {38, 181,
10, 2090}, {39, 182, 20, 2090}, {40, 183, 10, 2090}, {41, 187, 10, 2090}, {42, 188, 30,
2090}, {43, 189, 30, 2090}, {44, 190, 10, 2090}, {45, 194, 5, 2090}, {46, 195, 20, 2090},
{47, 196, 30, 2090}, {48, 197, 20, 2090}, {49, 198, 5, 2090}, {50, 202, 1, 2090}, {51,
203, 5, 2090}, {52, 204, 10, 2090}, {53, 205, 10, 2090}, {54, 206, 5, 2090}, {55, 207, 1,
2090}, {35, 173, 1, 2091}, {36, 177, 5, 2091}, {37, 178, 5, 2091}, {38, 182, 10, 2091},
{39, 183, 20, 2091}, {40, 184, 10, 2091}, {41, 188, 10, 2091}, {42, 189, 30, 2091}, {43,
190, 30, 2091}, {44, 191, 10, 2091}, {45, 195, 5, 2091}, {46, 196, 20, 2091}, {47, 197,
30, 2091}, {48, 198, 20, 2091}, {49, 199, 5, 2091}, {50, 203, 1, 2091}, {51, 204, 5,
2091}, {52, 205, 10, 2091}, {53, 206, 10, 2091}, {54, 207, 5, 2091}, {55, 208, 1, 2091},
{35, 174, 1, 2092}, {36, 178, 5, 2092}, {37, 179, 5, 2092}, {38, 183, 10, 2092}, {39, 184,
20, 2092}, {40, 185, 10, 2092}, {41, 189, 10, 2092}, {42, 190, 30, 2092}, {43, 191, 30,
2092}, {44, 192, 10, 2092}, {45, 196, 5, 2092}, {46, 197, 20, 2092}, {47, 198, 30, 2092},
{48, 199, 20, 2092}, {49, 200, 5, 2092}, {50, 204, 1, 2092}, {51, 205, 5, 2092}, {52, 206,
10, 2092}, {53, 207, 10, 2092}, {54, 208, 5, 2092}, {55, 209, 1, 2092}, {35, 175, 1,
2093}, {36, 180, 5, 2093}, {37, 181, 5, 2093}, {38, 186, 10, 2093}, {39, 187, 20, 2093},
{40, 188, 10, 2093}, {41, 193, 10, 2093}, {42, 194, 30, 2093}, {43, 195, 30, 2093}, {44,
196, 10, 2093}, {45, 201, 5, 2093}, {46, 202, 20, 2093}, {47, 203, 30, 2093}, {48, 204,
20, 2093}, {49, 205, 5, 2093}, {50, 210, 1, 2093}, {51, 211, 5, 2093}, {52, 212, 10,
2093}, {53, 213, 10, 2093}, {54, 214, 5, 2093}, {55, 215, 1, 2093}, {35, 176, 1, 2094},
{36, 181, 5, 2094}, {37, 182, 5, 2094}, {38, 187, 10, 2094}, {39, 188, 20, 2094}, {40,
189, 10, 2094}, {41, 194, 10, 2094}, {42, 195, 30, 2094}, {43, 196, 30, 2094}, {44, 197,
10, 2094}, {45, 202, 5, 2094}, {46, 203, 20, 2094}, {47, 204, 30, 2094}, {48, 205, 20,
2094}, {49, 206, 5, 2094}, {50, 211, 1, 2094}, {51, 212, 5, 2094}, {52, 213, 10, 2094},
{53, 214, 10, 2094}, {54, 215, 5, 2094}, {55, 216, 1, 2094}, {35, 177, 1, 2095}, {36, 182,
5, 2095}, {37, 183, 5, 2095}, {38, 188, 10, 2095}, {39, 189, 20, 2095}, {40, 190, 10,
2095}, {41, 195, 10, 2095}, {42, 196, 30, 2095}, {43, 197, 30, 2095}, {44, 198, 10, 2095},
{45, 203, 5, 2095}, {46, 204, 20, 2095}, {47, 205, 30, 2095}, {48, 206, 20, 2095}, {49,
207, 5, 2095}, {50, 212, 1, 2095}, {51, 213, 5, 2095}, {52, 214, 10, 2095}, {53, 215, 10,
2095}, {54, 216, 5, 2095}, {55, 217, 1, 2095}, {35, 178, 1, 2096}, {36, 183, 5, 2096},
{37, 184, 5, 2096}, {38, 189, 10, 2096}, {39, 190, 20, 2096}, {40, 191, 10, 2096}, {41,
196, 10, 2096}, {42, 197, 30, 2096}, {43, 198, 30, 2096}, {44, 199, 10, 2096}, {45, 204,
5, 2096}, {46, 205, 20, 2096}, {47, 206, 30, 2096}, {48, 207, 20, 2096}, {49, 208, 5,
2096}, {50, 213, 1, 2096}, {51, 214, 5, 2096}, {52, 215, 10, 2096}, {53, 216, 10, 2096},
{54, 217, 5, 2096}, {55, 218, 1, 2096}, {35, 179, 1, 2097}, {36, 184, 5, 2097}, {37, 185,
5, 2097}, {38, 190, 10, 2097}, {39, 191, 20, 2097}, {40, 192, 10, 2097}, {41, 197, 10,
2097}, {42, 198, 30, 2097}, {43, 199, 30, 2097}, {44, 200, 10, 2097}, {45, 205, 5, 2097},
{46, 206, 20, 2097}, {47, 207, 30, 2097}, {48, 208, 20, 2097}, {49, 209, 5, 2097}, {50,
214, 1, 2097}, {51, 215, 5, 2097}, {52, 216, 10, 2097}, {53, 217, 10, 2097}, {54, 218, 5,
2097}, {55, 219, 1, 2097}, {35, 220, 1, 2098}, {36, 221, 5, 2098}, {37, 222, 5, 2098},
{38, 223, 10, 2098}, {39, 224, 20, 2098}, {40, 225, 10, 2098}, {41, 226, 10, 2098}, {42,
227, 30, 2098}, {43, 228, 30, 2098}, {44, 229, 10, 2098}, {45, 230, 5, 2098}, {46, 231,
20, 2098}, {47, 232, 30, 2098}, {48, 233, 20, 2098}, {49, 234, 5, 2098}, {50, 235, 1,
2098}, {51, 236, 5, 2098}, {52, 237, 10, 2098}, {53, 238, 10, 2098}, {54, 239, 5, 2098},
{55, 240, 1, 2098}, {35, 221, 1, 2099}, {36, 223, 5, 2099}, {37, 224, 5, 2099}, {38, 226,
10, 2099}, {39, 227, 20, 2099}, {40, 228, 10, 2099}, {41, 230, 10, 2099}, {42, 231, 30,
2099}, {43, 232, 30, 2099}, {44, 233, 10, 2099}, {45, 235, 5, 2099}, {46, 236, 20, 2099},
{47, 237, 30, 2099}, {48, 238, 20, 2099}, {49, 239, 5, 2099}, {50, 241, 1, 2099}, {51,
242, 5, 2099}, {52, 243, 10, 2099}, {53, 244, 10, 2099}, {54, 245, 5, 2099}, {55, 246, 1,
2099}, {35, 222, 1, 2100}, {36, 224, 5, 2100}, {37, 225, 5, 2100}, {38, 227, 10, 2100},

{39, 228, 20, 2100}, {40, 229, 10, 2100}, {41, 231, 10, 2100}, {42, 232, 30, 2100}, {43, 233, 30, 2100}, {44, 234, 10, 2100}, {45, 236, 5, 2100}, {46, 237, 20, 2100}, {47, 238, 30, 2100}, {48, 239, 20, 2100}, {49, 240, 5, 2100}, {50, 242, 1, 2100}, {51, 243, 5, 2100}, {52, 244, 10, 2100}, {53, 245, 10, 2100}, {54, 246, 5, 2100}, {55, 247, 1, 2100}, {35, 223, 1, 2101}, {36, 226, 5, 2101}, {37, 227, 5, 2101}, {38, 230, 10, 2101}, {39, 231, 20, 2101}, {40, 232, 10, 2101}, {41, 235, 10, 2101}, {42, 236, 30, 2101}, {43, 237, 30, 2101}, {44, 238, 10, 2101}, {45, 241, 5, 2101}, {46, 242, 20, 2101}, {47, 243, 30, 2101}, {48, 244, 20, 2101}, {49, 245, 5, 2101}, {50, 248, 1, 2101}, {51, 249, 5, 2101}, {52, 250, 10, 2101}, {53, 251, 10, 2101}, {54, 252, 5, 2101}, {55, 253, 1, 2101}, {35, 224, 1, 2102}, {36, 227, 5, 2102}, {37, 228, 5, 2102}, {38, 231, 10, 2102}, {39, 232, 20, 2102}, {40, 233, 10, 2102}, {41, 236, 10, 2102}, {42, 237, 30, 2102}, {43, 238, 30, 2102}, {44, 239, 10, 2102}, {45, 242, 5, 2102}, {46, 243, 20, 2102}, {47, 244, 30, 2102}, {48, 245, 20, 2102}, {49, 246, 5, 2102}, {50, 249, 1, 2102}, {51, 250, 5, 2102}, {52, 251, 10, 2102}, {53, 252, 10, 2102}, {54, 253, 5, 2102}, {55, 254, 1, 2102}, {35, 225, 1, 2103}, {36, 228, 5, 2103}, {37, 229, 5, 2103}, {38, 232, 10, 2103}, {39, 233, 20, 2103}, {40, 234, 10, 2103}, {41, 237, 10, 2103}, {42, 238, 30, 2103}, {43, 239, 30, 2103}, {44, 240, 10, 2103}, {45, 243, 5, 2103}, {46, 244, 20, 2103}, {47, 245, 30, 2103}, {48, 246, 20, 2103}, {49, 247, 5, 2103}, {50, 250, 1, 2103}, {51, 251, 5, 2103}, {52, 252, 10, 2103}, {53, 253, 10, 2103}, {54, 254, 5, 2103}, {55, 255, 1, 2103}, {35, 226, 1, 2104}, {36, 230, 5, 2104}, {37, 231, 5, 2104}, {38, 235, 10, 2104}, {39, 236, 20, 2104}, {40, 237, 10, 2104}, {41, 241, 10, 2104}, {42, 242, 30, 2104}, {43, 243, 30, 2104}, {44, 244, 10, 2104}, {45, 248, 5, 2104}, {46, 249, 20, 2104}, {47, 250, 30, 2104}, {48, 251, 20, 2104}, {49, 252, 5, 2104}, {50, 256, 1, 2104}, {51, 257, 5, 2104}, {52, 258, 10, 2104}, {53, 259, 10, 2104}, {54, 260, 5, 2104}, {55, 261, 1, 2104}, {35, 227, 1, 2105}, {36, 231, 5, 2105}, {37, 232, 5, 2105}, {38, 236, 10, 2105}, {39, 237, 20, 2105}, {40, 238, 10, 2105}, {41, 242, 10, 2105}, {42, 243, 30, 2105}, {43, 244, 30, 2105}, {44, 245, 10, 2105}, {45, 249, 5, 2105}, {46, 250, 20, 2105}, {47, 251, 30, 2105}, {48, 252, 20, 2105}, {49, 253, 5, 2105}, {50, 257, 1, 2105}, {51, 258, 5, 2105}, {52, 259, 10, 2105}, {53, 260, 10, 2105}, {54, 261, 5, 2105}, {55, 262, 1, 2105}, {35, 228, 1, 2106}, {36, 232, 5, 2106}, {37, 233, 5, 2106}, {38, 237, 10, 2106}, {39, 238, 20, 2106}, {40, 239, 10, 2106}, {41, 243, 10, 2106}, {42, 244, 30, 2106}, {43, 245, 30, 2106}, {44, 246, 10, 2106}, {45, 250, 5, 2106}, {46, 251, 20, 2106}, {47, 252, 30, 2106}, {48, 253, 20, 2106}, {49, 254, 5, 2106}, {50, 258, 1, 2106}, {51, 259, 5, 2106}, {52, 260, 10, 2106}, {53, 261, 10, 2106}, {54, 262, 5, 2106}, {55, 263, 1, 2106}, {35, 229, 1, 2107}, {36, 233, 5, 2107}, {37, 234, 5, 2107}, {38, 238, 10, 2107}, {39, 239, 20, 2107}, {40, 240, 10, 2107}, {41, 244, 10, 2107}, {42, 245, 30, 2107}, {43, 246, 30, 2107}, {44, 247, 10, 2107}, {45, 251, 5, 2107}, {46, 252, 20, 2107}, {47, 253, 30, 2107}, {48, 254, 20, 2107}, {49, 255, 5, 2107}, {50, 259, 1, 2107}, {51, 260, 5, 2107}, {52, 261, 10, 2107}, {53, 262, 10, 2107}, {54, 263, 5, 2107}, {55, 264, 1, 2107}, {35, 230, 1, 2108}, {36, 235, 5, 2108}, {37, 236, 5, 2108}, {38, 241, 10, 2108}, {39, 242, 20, 2108}, {40, 243, 10, 2108}, {41, 248, 10, 2108}, {42, 249, 30, 2108}, {43, 250, 30, 2108}, {44, 251, 10, 2108}, {45, 256, 5, 2108}, {46, 257, 20, 2108}, {47, 258, 30, 2108}, {48, 259, 20, 2108}, {49, 260, 5, 2108}, {50, 265, 1, 2108}, {51, 266, 5, 2108}, {52, 267, 10, 2108}, {53, 268, 10, 2108}, {54, 269, 5, 2108}, {55, 270, 1, 2108}, {35, 231, 1, 2109}, {36, 236, 5, 2109}, {37, 237, 5, 2109}, {38, 242, 10, 2109}, {39, 243, 20, 2109}, {40, 244, 10, 2109}, {41, 249, 10, 2109}, {42, 250, 30, 2109}, {43, 251, 30, 2109}, {44, 252, 10, 2109}, {45, 257, 5, 2109}, {46, 258, 20, 2109}, {47, 259, 30, 2109}, {48, 260, 20, 2109}, {49, 261, 5, 2109}, {50, 266, 1, 2109}, {51, 267, 5, 2109}, {52, 268, 10, 2109}, {53, 269, 10, 2109}, {54, 270, 5, 2109}, {55, 271, 1, 2109}, {35, 232, 1, 2110}, {36, 237, 5, 2110}, {37, 238, 5, 2110}, {38, 243, 10, 2110}, {39, 244, 20, 2110}, {40, 245, 10, 2110}, {41, 250, 10, 2110}, {42, 251, 30, 2110}, {43, 252, 30, 2110}, {44, 253, 10, 2110}, {45, 258, 5, 2110}, {46, 259, 20, 2110}, {47, 260, 30, 2110}, {48, 261, 20, 2110}, {49, 262, 5, 2110}, {50, 267, 1, 2110}, {51, 268, 5, 2110}, {52, 269, 10, 2110}, {53, 270, 10, 2110}, {54, 271, 5, 2110}, {55, 272, 1, 2110}, {35, 233, 1, 2111}, {36, 238, 5, 2111}, {37, 239, 5, 2111}, {38, 244, 10, 2111}, {39, 245, 20, 2111}, {40, 246, 10, 2111}, {41, 251, 10, 2111}, {42, 252, 30, 2111}, {43, 253, 30, 2111}, {44, 254, 10, 2111}, {45, 259, 5, 2111}, {46, 260, 20, 2111}, {47, 261, 30, 2111}, {48, 262, 20, 2111}, {49, 263, 5, 2111}, {50, 268, 1, 2111}, {51, 269, 5, 2111}, {52, 270, 10, 2111}, {53, 271, 10, 2111}, {54, 272, 5, 2111}, {55, 273, 1, 2111}, {35, 234, 1, 2112}, {36, 239, 5, 2112}, {37, 240, 5, 2112}, {38, 245, 10, 2112}, {39, 246, 20, 2112}, {40, 247, 10, 2112}, {41, 252, 10, 2112}, {42, 253, 30, 2112}, {43, 254, 30, 2112}, {44, 255, 10, 2112}, {45, 260, 5, 2112}, {46, 261, 20, 2112}, {47, 262, 30, 2112}, {48, 263, 20, 2112}, {49, 264, 5, 2112}, {50, 269, 1, 2112}, {51, 270, 5, 2112}, {52, 271, 10, 2112}, {53, 272, 10, 2112}, {54, 273, 5, 2112}, {55, 274, 1, 2112}, {35, 235, 1, 2113}, {36, 241, 5, 2113}, {37, 242, 5, 2113}, {38, 248, 10, 2113}, {39, 249, 20, 2113}, {40, 250, 10, 2113}, {41, 256, 10, 2113}, {42, 257, 30, 2113}, {43, 258, 30, 2113}, {44, 259, 10, 2113}, {45, 265, 5, 2113}, {46, 266, 20, 2113}, {47, 267, 30, 2113}, {48, 268, 20, 2113}, {49, 269, 5, 2113}, {50, 275, 1, 2113}, {51, 276, 5, 2113}, {52, 277, 10, 2113}, {53, 278, 10, 2113}, {54, 279, 5, 2113}, {55, 280, 1, 2113}, {35, 236, 1, 2114}, {36, 242, 5, 2114}, {37, 243, 5, 2114}, {38, 249, 10, 2114}, {39, 250, 20, 2114}, {40, 251, 10, 2114}, {41,

257, 10, 2114}, {42, 258, 30, 2114}, {43, 259, 30, 2114}, {44, 260, 10, 2114}, {45, 266, 5, 2114}, {46, 267, 20, 2114}, {47, 268, 30, 2114}, {48, 269, 20, 2114}, {49, 270, 5, 2114}, {50, 276, 1, 2114}, {51, 277, 5, 2114}, {52, 278, 10, 2114}, {53, 279, 10, 2114}, {54, 280, 5, 2114}, {55, 281, 1, 2114}, {35, 237, 1, 2115}, {36, 243, 5, 2115}, {37, 244, 5, 2115}, {38, 250, 10, 2115}, {39, 251, 20, 2115}, {40, 252, 10, 2115}, {41, 258, 10, 2115}, {42, 259, 30, 2115}, {43, 260, 30, 2115}, {44, 261, 10, 2115}, {45, 267, 5, 2115}, {46, 268, 20, 2115}, {47, 269, 30, 2115}, {48, 270, 20, 2115}, {49, 271, 5, 2115}, {50, 277, 1, 2115}, {51, 278, 5, 2115}, {52, 279, 10, 2115}, {53, 280, 10, 2115}, {54, 281, 5, 2115}, {55, 282, 1, 2115}, {35, 238, 1, 2116}, {36, 244, 5, 2116}, {37, 245, 5, 2116}, {38, 251, 10, 2116}, {39, 252, 20, 2116}, {40, 253, 10, 2116}, {41, 259, 10, 2116}, {42, 260, 30, 2116}, {43, 261, 30, 2116}, {44, 262, 10, 2116}, {45, 268, 5, 2116}, {46, 269, 20, 2116}, {47, 270, 30, 2116}, {48, 271, 20, 2116}, {49, 272, 5, 2116}, {50, 278, 1, 2116}, {51, 279, 5, 2116}, {52, 280, 10, 2116}, {53, 281, 10, 2116}, {54, 282, 5, 2116}, {55, 283, 1, 2116}, {35, 239, 1, 2117}, {36, 245, 5, 2117}, {37, 246, 5, 2117}, {38, 252, 10, 2117}, {39, 253, 20, 2117}, {40, 254, 10, 2117}, {41, 260, 10, 2117}, {42, 261, 30, 2117}, {43, 262, 30, 2117}, {44, 263, 10, 2117}, {45, 269, 5, 2117}, {46, 270, 20, 2117}, {47, 271, 30, 2117}, {48, 272, 20, 2117}, {49, 273, 5, 2117}, {50, 279, 1, 2117}, {51, 280, 5, 2117}, {52, 281, 10, 2117}, {53, 282, 10, 2117}, {54, 283, 5, 2117}, {55, 284, 1, 2117}, {35, 240, 1, 2118}, {36, 246, 5, 2118}, {37, 247, 5, 2118}, {38, 253, 10, 2118}, {39, 254, 20, 2118}, {40, 255, 10, 2118}, {41, 261, 10, 2118}, {42, 262, 30, 2118}, {43, 263, 30, 2118}, {44, 264, 10, 2118}, {45, 270, 5, 2118}, {46, 271, 20, 2118}, {47, 272, 30, 2118}, {48, 273, 20, 2118}, {49, 274, 5, 2118}, {50, 280, 1, 2118}, {51, 281, 5, 2118}, {52, 282, 10, 2118}, {53, 283, 10, 2118}, {54, 284, 5, 2118}, {55, 285, 1, 2118}, {56, 56, 1, 2119}, {57, 57, 3, 2119}, {58, 58, 3, 2119}, {59, 59, 3, 2119}, {60, 60, 6, 2119}, {61, 61, 3, 2119}, {62, 62, 1, 2119}, {63, 63, 3, 2119}, {64, 64, 3, 2119}, {65, 65, 1, 2119}, {56, 57, 1, 2120}, {57, 59, 3, 2120}, {58, 60, 3, 2120}, {59, 62, 3, 2120}, {60, 63, 6, 2120}, {61, 64, 3, 2120}, {62, 66, 1, 2120}, {63, 67, 3, 2120}, {64, 68, 3, 2120}, {65, 69, 1, 2120}, {56, 58, 1, 2121}, {57, 60, 3, 2121}, {58, 61, 3, 2121}, {59, 63, 3, 2121}, {60, 64, 6, 2121}, {61, 65, 3, 2121}, {62, 67, 1, 2121}, {63, 68, 3, 2121}, {64, 69, 3, 2121}, {65, 70, 1, 2121}, {56, 59, 1, 2122}, {57, 62, 3, 2122}, {58, 63, 3, 2122}, {59, 66, 3, 2122}, {60, 67, 6, 2122}, {61, 68, 3, 2122}, {62, 71, 1, 2122}, {63, 72, 3, 2122}, {64, 73, 3, 2122}, {65, 74, 1, 2122}, {56, 60, 1, 2123}, {57, 63, 3, 2123}, {58, 64, 3, 2123}, {59, 67, 3, 2123}, {60, 68, 6, 2123}, {61, 69, 3, 2123}, {62, 72, 1, 2123}, {63, 73, 3, 2123}, {64, 74, 3, 2123}, {65, 75, 1, 2123}, {56, 61, 1, 2124}, {57, 64, 3, 2124}, {58, 65, 3, 2124}, {59, 68, 3, 2124}, {60, 69, 6, 2124}, {61, 70, 3, 2124}, {62, 73, 1, 2124}, {63, 74, 3, 2124}, {64, 75, 3, 2124}, {65, 76, 1, 2124}, {56, 62, 1, 2125}, {57, 66, 3, 2125}, {58, 67, 3, 2125}, {59, 71, 3, 2125}, {60, 72, 6, 2125}, {61, 73, 3, 2125}, {62, 77, 1, 2125}, {63, 78, 3, 2125}, {64, 79, 3, 2125}, {65, 80, 1, 2125}, {56, 63, 1, 2126}, {57, 67, 3, 2126}, {58, 68, 3, 2126}, {59, 72, 3, 2126}, {60, 73, 6, 2126}, {61, 74, 3, 2126}, {62, 78, 1, 2126}, {63, 79, 3, 2126}, {64, 80, 3, 2126}, {65, 81, 1, 2126}, {56, 64, 1, 2127}, {57, 68, 3, 2127}, {58, 69, 3, 2127}, {59, 73, 3, 2127}, {60, 74, 6, 2127}, {61, 75, 3, 2127}, {62, 79, 1, 2127}, {63, 80, 3, 2127}, {64, 81, 3, 2127}, {65, 82, 1, 2127}, {56, 65, 1, 2128}, {57, 69, 3, 2128}, {58, 70, 3, 2128}, {59, 74, 3, 2128}, {60, 75, 6, 2128}, {61, 76, 3, 2128}, {62, 80, 1, 2128}, {63, 81, 3, 2128}, {64, 82, 3, 2128}, {65, 83, 1, 2128}, {57, 57, 1, 2129}, {59, 59, 3, 2129}, {60, 60, 3, 2129}, {62, 62, 3, 2129}, {63, 63, 6, 2129}, {64, 64, 3, 2129}, {66, 66, 1, 2129}, {67, 67, 3, 2129}, {68, 68, 3, 2129}, {69, 69, 1, 2129}, {57, 58, 1, 2130}, {59, 60, 3, 2130}, {60, 61, 3, 2130}, {62, 63, 3, 2130}, {63, 64, 6, 2130}, {64, 65, 3, 2130}, {66, 67, 1, 2130}, {67, 68, 3, 2130}, {68, 69, 3, 2130}, {69, 70, 1, 2130}, {57, 59, 1, 2131}, {59, 62, 3, 2131}, {60, 63, 3, 2131}, {62, 66, 3, 2131}, {63, 67, 6, 2131}, {64, 68, 3, 2131}, {66, 71, 1, 2131}, {67, 72, 3, 2131}, {68, 73, 3, 2131}, {69, 74, 1, 2131}, {57, 60, 1, 2132}, {59, 63, 3, 2132}, {60, 64, 3, 2132}, {62, 67, 3, 2132}, {63, 68, 6, 2132}, {64, 69, 3, 2132}, {66, 72, 1, 2132}, {67, 73, 3, 2132}, {68, 74, 3, 2132}, {69, 75, 1, 2132}, {57, 61, 1, 2133}, {59, 64, 3, 2133}, {60, 65, 3, 2133}, {62, 68, 3, 2133}, {63, 69, 6, 2133}, {64, 70, 3, 2133}, {66, 73, 1, 2133}, {67, 74, 3, 2133}, {68, 75, 3, 2133}, {69, 76, 1, 2133}, {57, 62, 1, 2134}, {59, 66, 3, 2134}, {60, 67, 3, 2134}, {62, 71, 3, 2134}, {63, 72, 6, 2134}, {64, 73, 3, 2134}, {66, 77, 1, 2134}, {67, 78, 3, 2134}, {68, 79, 3, 2134}, {69, 80, 1, 2134}, {57, 63, 1, 2135}, {59, 67, 3, 2135}, {60, 68, 3, 2135}, {62, 72, 3, 2135}, {63, 73, 6, 2135}, {64, 74, 3, 2135}, {66, 78, 1, 2135}, {67, 79, 3, 2135}, {68, 80, 3, 2135}, {69, 81, 1, 2135}, {57, 64, 1, 2136}, {59, 68, 3, 2136}, {60, 69, 3, 2136}, {62, 73, 3, 2136}, {63, 74, 6, 2136}, {64, 75, 3, 2136}, {66, 79, 1, 2136}, {67, 80, 3, 2136}, {68, 81, 3, 2136}, {69, 82, 1, 2136}, {57, 65, 1, 2137}, {59, 69, 3, 2137}, {60, 70, 3, 2137}, {62, 74, 3, 2137}, {63, 75, 6, 2137}, {64, 76, 3, 2137}, {66, 80, 1, 2137}, {67, 81, 3, 2137}, {68, 82, 3, 2137}, {69, 83, 1, 2137}, {58, 58, 1, 2138}, {60, 60, 3, 2138}, {61, 61, 3, 2138}, {63, 63, 3, 2138}, {64, 64, 6, 2138}, {65, 65, 3, 2138}, {67, 67, 1, 2138}, {68, 68, 3, 2138}, {69, 69, 3, 2138}, {70, 70, 1, 2138}, {58, 59, 1, 2139}, {60, 62, 3, 2139}, {61, 63, 3, 2139}, {63, 66, 3, 2139}, {64, 67, 6, 2139}, {65, 68, 3, 2139}, {67, 71, 1, 2139}, {68, 72, 3, 2139}, {69, 73, 3, 2139}, {70, 74, 1, 2139}, {58, 60, 1, 2140}, {60, 63, 3, 2140}, {61, 64, 3, 2140}, {63, 67, 3, 2140}, {64, 68, 6,

2140}, {65, 69, 3, 2140}, {67, 72, 1, 2140}, {68, 73, 3, 2140}, {69, 74, 3, 2140}, {70, 75, 1, 2140}, {58, 61, 1, 2141}, {60, 64, 3, 2141}, {61, 65, 3, 2141}, {63, 68, 3, 2141}, {64, 69, 6, 2141}, {65, 70, 3, 2141}, {67, 73, 1, 2141}, {68, 74, 3, 2141}, {69, 75, 3, 2141}, {70, 76, 1, 2141}, {58, 62, 1, 2142}, {60, 66, 3, 2142}, {61, 67, 3, 2142}, {63, 71, 3, 2142}, {64, 72, 6, 2142}, {65, 73, 3, 2142}, {67, 77, 1, 2142}, {68, 78, 3, 2142}, {69, 79, 3, 2142}, {70, 80, 1, 2142}, {58, 63, 1, 2143}, {60, 67, 3, 2143}, {61, 68, 3, 2143}, {63, 72, 3, 2143}, {64, 73, 6, 2143}, {65, 74, 3, 2143}, {67, 78, 1, 2143}, {68, 79, 3, 2143}, {69, 80, 3, 2143}, {70, 81, 1, 2143}, {58, 64, 1, 2144}, {60, 68, 3, 2144}, {61, 69, 3, 2144}, {63, 73, 3, 2144}, {64, 74, 6, 2144}, {65, 75, 3, 2144}, {67, 79, 1, 2144}, {68, 80, 3, 2144}, {69, 81, 3, 2144}, {70, 82, 1, 2144}, {58, 65, 1, 2145}, {60, 69, 3, 2145}, {61, 70, 3, 2145}, {63, 74, 3, 2145}, {64, 75, 6, 2145}, {65, 76, 3, 2145}, {67, 80, 1, 2145}, {68, 81, 3, 2145}, {69, 82, 3, 2145}, {70, 83, 1, 2145}, {59, 59, 1, 2146}, {62, 62, 3, 2146}, {63, 63, 3, 2146}, {66, 66, 3, 2146}, {67, 67, 6, 2146}, {68, 68, 3, 2146}, {71, 71, 1, 2146}, {72, 72, 3, 2146}, {73, 73, 3, 2146}, {74, 74, 1, 2146}, {59, 60, 1, 2147}, {62, 63, 3, 2147}, {63, 64, 3, 2147}, {66, 67, 3, 2147}, {67, 68, 6, 2147}, {68, 69, 3, 2147}, {71, 72, 1, 2147}, {72, 73, 3, 2147}, {73, 74, 3, 2147}, {74, 75, 1, 2147}, {59, 61, 1, 2148}, {62, 64, 3, 2148}, {63, 65, 3, 2148}, {66, 68, 3, 2148}, {67, 69, 6, 2148}, {68, 70, 3, 2148}, {71, 73, 1, 2148}, {72, 74, 3, 2148}, {73, 75, 3, 2148}, {74, 76, 1, 2148}, {59, 62, 1, 2149}, {62, 66, 3, 2149}, {63, 67, 3, 2149}, {66, 71, 3, 2149}, {67, 72, 6, 2149}, {68, 73, 3, 2149}, {71, 77, 1, 2149}, {72, 78, 3, 2149}, {73, 79, 3, 2149}, {74, 80, 1, 2149}, {59, 63, 1, 2150}, {62, 67, 3, 2150}, {63, 68, 3, 2150}, {66, 72, 3, 2150}, {67, 73, 6, 2150}, {68, 74, 3, 2150}, {71, 78, 1, 2150}, {72, 79, 3, 2150}, {73, 80, 3, 2150}, {74, 81, 1, 2150}, {59, 64, 1, 2151}, {62, 68, 3, 2151}, {63, 69, 3, 2151}, {66, 73, 3, 2151}, {67, 74, 6, 2151}, {68, 75, 3, 2151}, {71, 79, 1, 2151}, {72, 80, 3, 2151}, {73, 81, 3, 2151}, {74, 82, 1, 2151}, {59, 65, 1, 2152}, {62, 69, 3, 2152}, {63, 70, 3, 2152}, {66, 74, 3, 2152}, {67, 75, 6, 2152}, {68, 76, 3, 2152}, {71, 80, 1, 2152}, {72, 81, 3, 2152}, {73, 82, 3, 2152}, {74, 83, 1, 2152}, {60, 60, 1, 2153}, {63, 63, 3, 2153}, {64, 64, 3, 2153}, {67, 67, 3, 2153}, {68, 68, 6, 2153}, {69, 69, 3, 2153}, {72, 72, 1, 2153}, {73, 73, 3, 2153}, {74, 74, 3, 2153}, {75, 75, 1, 2153}, {60, 61, 1, 2154}, {63, 64, 3, 2154}, {64, 65, 3, 2154}, {67, 68, 3, 2154}, {68, 69, 6, 2154}, {69, 70, 3, 2154}, {72, 73, 1, 2154}, {73, 74, 3, 2154}, {74, 75, 3, 2154}, {75, 76, 1, 2154}, {60, 62, 1, 2155}, {63, 66, 3, 2155}, {64, 67, 3, 2155}, {67, 71, 3, 2155}, {68, 72, 6, 2155}, {69, 73, 3, 2155}, {72, 77, 1, 2155}, {73, 78, 3, 2155}, {74, 79, 3, 2155}, {75, 80, 1, 2155}, {60, 63, 1, 2156}, {63, 67, 3, 2156}, {64, 68, 3, 2156}, {67, 72, 3, 2156}, {68, 73, 6, 2156}, {69, 74, 3, 2156}, {72, 78, 1, 2156}, {73, 79, 3, 2156}, {74, 80, 3, 2156}, {75, 81, 1, 2156}, {60, 64, 1, 2157}, {63, 68, 3, 2157}, {64, 69, 3, 2157}, {67, 73, 3, 2157}, {68, 74, 6, 2157}, {69, 75, 3, 2157}, {72, 79, 1, 2157}, {73, 80, 3, 2157}, {74, 81, 3, 2157}, {75, 82, 1, 2157}, {60, 65, 1, 2158}, {63, 69, 3, 2158}, {64, 70, 3, 2158}, {67, 74, 3, 2158}, {68, 75, 6, 2158}, {69, 76, 3, 2158}, {72, 80, 1, 2158}, {73, 81, 3, 2158}, {74, 82, 3, 2158}, {75, 83, 1, 2158}, {61, 61, 1, 2159}, {64, 64, 3, 2159}, {65, 65, 3, 2159}, {68, 68, 3, 2159}, {69, 69, 6, 2159}, {70, 70, 3, 2159}, {73, 73, 1, 2159}, {74, 74, 3, 2159}, {75, 75, 3, 2159}, {76, 76, 1, 2159}, {61, 62, 1, 2160}, {64, 66, 3, 2160}, {65, 67, 3, 2160}, {68, 71, 3, 2160}, {69, 72, 6, 2160}, {70, 73, 3, 2160}, {73, 77, 1, 2160}, {74, 78, 3, 2160}, {75, 79, 3, 2160}, {76, 80, 1, 2160}, {61, 63, 1, 2161}, {64, 67, 3, 2161}, {65, 68, 3, 2161}, {68, 72, 3, 2161}, {69, 73, 6, 2161}, {70, 74, 3, 2161}, {73, 78, 1, 2161}, {74, 79, 3, 2161}, {75, 80, 3, 2161}, {76, 81, 1, 2161}, {61, 64, 1, 2162}, {64, 68, 3, 2162}, {65, 69, 3, 2162}, {68, 73, 3, 2162}, {69, 74, 6, 2162}, {70, 75, 3, 2162}, {73, 79, 1, 2162}, {74, 80, 3, 2162}, {75, 81, 3, 2162}, {76, 82, 1, 2162}, {61, 65, 1, 2163}, {64, 69, 3, 2163}, {65, 70, 3, 2163}, {68, 74, 3, 2163}, {69, 75, 6, 2163}, {70, 76, 3, 2163}, {73, 80, 1, 2163}, {74, 81, 3, 2163}, {75, 82, 3, 2163}, {76, 83, 1, 2163}, {62, 62, 1, 2164}, {66, 66, 3, 2164}, {67, 67, 3, 2164}, {71, 71, 3, 2164}, {72, 72, 6, 2164}, {73, 73, 3, 2164}, {77, 77, 1, 2164}, {78, 78, 3, 2164}, {79, 79, 3, 2164}, {80, 80, 1, 2164}, {62, 63, 1, 2165}, {66, 67, 3, 2165}, {67, 68, 3, 2165}, {71, 72, 3, 2165}, {72, 73, 6, 2165}, {73, 74, 3, 2165}, {77, 78, 1, 2165}, {78, 79, 3, 2165}, {79, 80, 3, 2165}, {80, 81, 1, 2165}, {62, 64, 1, 2166}, {66, 68, 3, 2166}, {67, 69, 3, 2166}, {71, 73, 3, 2166}, {72, 74, 6, 2166}, {73, 75, 3, 2166}, {77, 79, 1, 2166}, {78, 80, 3, 2166}, {79, 81, 3, 2166}, {80, 82, 1, 2166}, {62, 65, 1, 2167}, {66, 69, 3, 2167}, {67, 70, 3, 2167}, {71, 74, 3, 2167}, {72, 75, 6, 2167}, {73, 76, 3, 2167}, {77, 80, 1, 2167}, {78, 81, 3, 2167}, {79, 82, 3, 2167}, {80, 83, 1, 2167}, {63, 63, 1, 2168}, {67, 67, 3, 2168}, {68, 68, 3, 2168}, {72, 72, 3, 2168}, {73, 73, 6, 2168}, {74, 74, 3, 2168}, {78, 78, 1, 2168}, {79, 79, 3, 2168}, {80, 80, 3, 2168}, {81, 81, 1, 2168}, {63, 64, 1, 2169}, {67, 68, 3, 2169}, {68, 69, 3, 2169}, {72, 73, 3, 2169}, {73, 74, 6, 2169}, {74, 75, 3, 2169}, {78, 79, 1, 2169}, {79, 80, 3, 2169}, {80, 81, 3, 2169}, {81, 82, 1, 2169}, {63, 65, 1, 2170}, {67, 69, 3, 2170}, {68, 70, 3, 2170}, {72, 74, 3, 2170}, {73, 75, 6, 2170}, {74, 76, 3, 2170}, {78, 80, 1, 2170}, {79, 81, 3, 2170}, {80, 82, 3, 2170}, {81, 83, 1, 2170}, {64, 64, 1, 2171}, {68, 68, 3, 2171}, {69, 69, 3, 2171}, {73, 73, 3, 2171}, {74, 74, 6, 2171}, {75, 75, 3, 2171}, {79, 79, 1, 2171}, {80, 80, 3, 2171}, {81, 81, 3, 2171}, {82, 82, 1, 2171}, {64, 65, 1, 2172}, {68, 69, 3, 2172}, {69, 70, 3, 2172}, {73, 74, 3, 2172}, {74, 75, 6, 2172}, {75, 76, 3, 2172}, {79, 80, 1,

2172}, {80, 81, 3, 2172}, {81, 82, 3, 2172}, {82, 83, 1, 2172}, {65, 65, 1, 2173}, {69,
69, 3, 2173}, {70, 70, 3, 2173}, {74, 74, 3, 2173}, {75, 75, 6, 2173}, {76, 76, 3, 2173},
{80, 80, 1, 2173}, {81, 81, 3, 2173}, {82, 82, 3, 2173}, {83, 83, 1, 2173}, {56, 56, 1,
2174}, {57, 57, 4, 2174}, {58, 58, 4, 2174}, {59, 59, 6, 2174}, {60, 60, 12, 2174}, {61,
61, 6, 2174}, {62, 62, 4, 2174}, {63, 63, 12, 2174}, {64, 64, 12, 2174}, {65, 65, 4,
2174}, {66, 66, 1, 2174}, {67, 67, 4, 2174}, {68, 68, 6, 2174}, {69, 69, 4, 2174}, {70,
70, 1, 2174}, {56, 57, 1, 2175}, {57, 59, 4, 2175}, {58, 60, 4, 2175}, {59, 62, 6, 2175},
{60, 63, 12, 2175}, {61, 64, 6, 2175}, {62, 66, 4, 2175}, {63, 67, 12, 2175}, {64, 68, 12,
2175}, {65, 69, 4, 2175}, {66, 71, 1, 2175}, {67, 72, 4, 2175}, {68, 73, 6, 2175}, {69,
74, 4, 2175}, {70, 75, 1, 2175}, {56, 58, 1, 2176}, {57, 60, 4, 2176}, {58, 61, 4, 2176},
{59, 63, 6, 2176}, {60, 64, 12, 2176}, {61, 65, 6, 2176}, {62, 67, 4, 2176}, {63, 68, 12,
2176}, {64, 69, 12, 2176}, {65, 70, 4, 2176}, {66, 72, 1, 2176}, {67, 73, 4, 2176}, {68,
74, 6, 2176}, {69, 75, 4, 2176}, {70, 76, 1, 2176}, {56, 59, 1, 2177}, {57, 62, 4, 2177},
{58, 63, 4, 2177}, {59, 66, 6, 2177}, {60, 67, 12, 2177}, {61, 68, 6, 2177}, {62, 71, 4,
2177}, {63, 72, 12, 2177}, {64, 73, 12, 2177}, {65, 74, 4, 2177}, {66, 77, 1, 2177}, {67,
78, 4, 2177}, {68, 79, 6, 2177}, {69, 80, 4, 2177}, {70, 81, 1, 2177}, {56, 60, 1, 2178},
{57, 63, 4, 2178}, {58, 64, 4, 2178}, {59, 67, 6, 2178}, {60, 68, 12, 2178}, {61, 69, 6,
2178}, {62, 72, 4, 2178}, {63, 73, 12, 2178}, {64, 74, 12, 2178}, {65, 75, 4, 2178}, {66,
78, 1, 2178}, {67, 79, 4, 2178}, {68, 80, 6, 2178}, {69, 81, 4, 2178}, {70, 82, 1, 2178},
{56, 61, 1, 2179}, {57, 64, 4, 2179}, {58, 65, 4, 2179}, {59, 68, 6, 2179}, {60, 69, 12,
2179}, {61, 70, 6, 2179}, {62, 73, 4, 2179}, {63, 74, 12, 2179}, {64, 75, 12, 2179}, {65,
76, 4, 2179}, {66, 79, 1, 2179}, {67, 80, 4, 2179}, {68, 81, 6, 2179}, {69, 82, 4, 2179},
{70, 83, 1, 2179}, {57, 57, 1, 2180}, {59, 59, 4, 2180}, {60, 60, 4, 2180}, {62, 62, 6,
2180}, {63, 63, 12, 2180}, {64, 64, 6, 2180}, {66, 66, 4, 2180}, {67, 67, 12, 2180}, {68,
68, 12, 2180}, {69, 69, 4, 2180}, {71, 71, 1, 2180}, {72, 72, 4, 2180}, {73, 73, 6, 2180},
{74, 74, 4, 2180}, {75, 75, 1, 2180}, {57, 58, 1, 2181}, {59, 60, 4, 2181}, {60, 61, 4,
2181}, {62, 63, 6, 2181}, {63, 64, 12, 2181}, {64, 65, 6, 2181}, {66, 67, 4, 2181}, {67,
68, 12, 2181}, {68, 69, 12, 2181}, {69, 70, 4, 2181}, {71, 72, 1, 2181}, {72, 73, 4,
2181}, {73, 74, 6, 2181}, {74, 75, 4, 2181}, {75, 76, 1, 2181}, {57, 59, 1, 2182}, {59,
62, 4, 2182}, {60, 63, 4, 2182}, {62, 66, 6, 2182}, {63, 67, 12, 2182}, {64, 68, 6, 2182},
{66, 71, 4, 2182}, {67, 72, 12, 2182}, {68, 73, 12, 2182}, {69, 74, 4, 2182}, {71, 77, 1,
2182}, {72, 78, 4, 2182}, {73, 79, 6, 2182}, {74, 80, 4, 2182}, {75, 81, 1, 2182}, {57,
60, 1, 2183}, {59, 63, 4, 2183}, {60, 64, 4, 2183}, {62, 67, 6, 2183}, {63, 68, 12, 2183},
{64, 69, 6, 2183}, {66, 72, 4, 2183}, {67, 73, 12, 2183}, {68, 74, 12, 2183}, {69, 75, 4,
2183}, {71, 78, 1, 2183}, {72, 79, 4, 2183}, {73, 80, 6, 2183}, {74, 81, 4, 2183}, {75,
82, 1, 2183}, {57, 61, 1, 2184}, {59, 64, 4, 2184}, {60, 65, 4, 2184}, {62, 68, 6, 2184},
{63, 69, 12, 2184}, {64, 70, 6, 2184}, {66, 73, 4, 2184}, {67, 74, 12, 2184}, {68, 75, 12,
2184}, {69, 76, 4, 2184}, {71, 79, 1, 2184}, {72, 80, 4, 2184}, {73, 81, 6, 2184}, {74,
82, 4, 2184}, {75, 83, 1, 2184}, {58, 58, 1, 2185}, {60, 60, 4, 2185}, {61, 61, 4, 2185},
{63, 63, 6, 2185}, {64, 64, 12, 2185}, {65, 65, 6, 2185}, {67, 67, 4, 2185}, {68, 68, 12,
2185}, {69, 69, 12, 2185}, {70, 70, 4, 2185}, {72, 72, 1, 2185}, {73, 73, 4, 2185}, {74,
74, 6, 2185}, {75, 75, 4, 2185}, {76, 76, 1, 2185}, {58, 59, 1, 2186}, {60, 62, 4, 2186},
{61, 63, 4, 2186}, {63, 66, 6, 2186}, {64, 67, 12, 2186}, {65, 68, 6, 2186}, {67, 71, 4,
2186}, {68, 72, 12, 2186}, {69, 73, 12, 2186}, {70, 74, 4, 2186}, {72, 77, 1, 2186}, {73,
78, 4, 2186}, {74, 79, 6, 2186}, {75, 80, 4, 2186}, {76, 81, 1, 2186}, {58, 60, 1, 2187},
{60, 63, 4, 2187}, {61, 64, 4, 2187}, {63, 67, 6, 2187}, {64, 68, 12, 2187}, {65, 69, 6,
2187}, {67, 72, 4, 2187}, {68, 73, 12, 2187}, {69, 74, 12, 2187}, {70, 75, 4, 2187}, {72,
78, 1, 2187}, {73, 79, 4, 2187}, {74, 80, 6, 2187}, {75, 81, 4, 2187}, {76, 82, 1, 2187},
{58, 61, 1, 2188}, {60, 64, 4, 2188}, {61, 65, 4, 2188}, {63, 68, 6, 2188}, {64, 69, 12,
2188}, {65, 70, 6, 2188}, {67, 73, 4, 2188}, {68, 74, 12, 2188}, {69, 75, 12, 2188}, {70,
76, 4, 2188}, {72, 79, 1, 2188}, {73, 80, 4, 2188}, {74, 81, 6, 2188}, {75, 82, 4, 2188},
{76, 83, 1, 2188}, {59, 59, 1, 2189}, {62, 62, 4, 2189}, {63, 63, 4, 2189}, {66, 66, 6,
2189}, {67, 67, 12, 2189}, {68, 68, 6, 2189}, {71, 71, 4, 2189}, {72, 72, 12, 2189}, {73,
73, 12, 2189}, {74, 74, 4, 2189}, {77, 77, 1, 2189}, {78, 78, 4, 2189}, {79, 79, 6, 2189},
{80, 80, 4, 2189}, {81, 81, 1, 2189}, {59, 60, 1, 2190}, {62, 63, 4, 2190}, {63, 64, 4,
2190}, {66, 67, 6, 2190}, {67, 68, 12, 2190}, {68, 69, 6, 2190}, {71, 72, 4, 2190}, {72,
73, 12, 2190}, {73, 74, 12, 2190}, {74, 75, 4, 2190}, {77, 78, 1, 2190}, {78, 79, 4,
2190}, {79, 80, 6, 2190}, {80, 81, 4, 2190}, {81, 82, 1, 2190}, {59, 61, 1, 2191}, {62,
64, 4, 2191}, {63, 65, 4, 2191}, {66, 68, 6, 2191}, {67, 69, 12, 2191}, {68, 70, 6, 2191},
{71, 73, 4, 2191}, {72, 74, 12, 2191}, {73, 75, 12, 2191}, {74, 76, 4, 2191}, {77, 79, 1,
2191}, {78, 80, 4, 2191}, {79, 81, 6, 2191}, {80, 82, 4, 2191}, {81, 83, 1, 2191}, {60,
60, 1, 2192}, {63, 63, 4, 2192}, {64, 64, 4, 2192}, {67, 67, 6, 2192}, {68, 68, 12, 2192},
{69, 69, 6, 2192}, {72, 72, 4, 2192}, {73, 73, 12, 2192}, {74, 74, 12, 2192}, {75, 75, 4,
2192}, {78, 78, 1, 2192}, {79, 79, 4, 2192}, {80, 80, 6, 2192}, {81, 81, 4, 2192}, {82,
82, 1, 2192}, {60, 61, 1, 2193}, {63, 64, 4, 2193}, {64, 65, 4, 2193}, {67, 68, 6, 2193},
{68, 69, 12, 2193}, {69, 70, 6, 2193}, {72, 73, 4, 2193}, {73, 74, 12, 2193}, {74, 75, 12,
2193}, {75, 76, 4, 2193}, {78, 79, 1, 2193}, {79, 80, 4, 2193}, {80, 81, 6, 2193}, {81,
82, 4, 2193}, {82, 83, 1, 2193}, {61, 61, 1, 2194}, {64, 64, 4, 2194}, {65, 65, 4, 2194},
{68, 68, 6, 2194}, {69, 69, 12, 2194}, {70, 70, 6, 2194}, {73, 73, 4, 2194}, {74, 74, 12,

2194}, {75, 75, 12, 2194}, {76, 76, 4, 2194}, {79, 79, 1, 2194}, {80, 80, 4, 2194}, {81, 81, 6, 2194}, {82, 82, 4, 2194}, {83, 83, 1, 2194}, {56, 84, 1, 2195}, {57, 85, 4, 2195}, {58, 86, 4, 2195}, {59, 87, 6, 2195}, {60, 88, 12, 2195}, {61, 89, 6, 2195}, {62, 90, 4, 2195}, {63, 91, 12, 2195}, {64, 92, 12, 2195}, {65, 93, 4, 2195}, {66, 94, 1, 2195}, {67, 95, 4, 2195}, {68, 96, 6, 2195}, {69, 97, 4, 2195}, {70, 98, 1, 2195}, {56, 85, 1, 2196}, {57, 87, 4, 2196}, {58, 88, 4, 2196}, {59, 90, 6, 2196}, {60, 91, 12, 2196}, {61, 92, 6, 2196}, {62, 94, 4, 2196}, {63, 95, 12, 2196}, {64, 96, 12, 2196}, {65, 97, 4, 2196}, {66, 99, 1, 2196}, {67, 100, 4, 2196}, {68, 101, 6, 2196}, {69, 102, 4, 2196}, {70, 103, 1, 2196}, {56, 86, 1, 2197}, {57, 88, 4, 2197}, {58, 89, 4, 2197}, {59, 91, 6, 2197}, {60, 92, 12, 2197}, {61, 93, 6, 2197}, {62, 95, 4, 2197}, {63, 96, 12, 2197}, {64, 97, 12, 2197}, {65, 98, 4, 2197}, {66, 100, 1, 2197}, {67, 101, 4, 2197}, {68, 102, 6, 2197}, {69, 103, 4, 2197}, {70, 104, 1, 2197}, {56, 87, 1, 2198}, {57, 90, 4, 2198}, {58, 91, 4, 2198}, {59, 94, 6, 2198}, {60, 95, 12, 2198}, {61, 96, 6, 2198}, {62, 99, 4, 2198}, {63, 100, 12, 2198}, {64, 101, 12, 2198}, {65, 102, 4, 2198}, {66, 105, 1, 2198}, {67, 106, 4, 2198}, {68, 107, 6, 2198}, {69, 108, 4, 2198}, {70, 109, 1, 2198}, {56, 88, 1, 2199}, {57, 91, 4, 2199}, {58, 92, 4, 2199}, {59, 95, 6, 2199}, {60, 96, 12, 2199}, {61, 97, 6, 2199}, {62, 100, 4, 2199}, {63, 101, 12, 2199}, {64, 102, 12, 2199}, {65, 103, 4, 2199}, {66, 106, 1, 2199}, {67, 107, 4, 2199}, {68, 108, 6, 2199}, {69, 109, 4, 2199}, {70, 110, 1, 2199}, {56, 89, 1, 2200}, {57, 92, 4, 2200}, {58, 93, 4, 2200}, {59, 96, 6, 2200}, {60, 97, 12, 2200}, {61, 98, 6, 2200}, {62, 101, 4, 2200}, {63, 102, 12, 2200}, {64, 103, 12, 2200}, {65, 104, 4, 2200}, {66, 107, 1, 2200}, {67, 108, 4, 2200}, {68, 109, 6, 2200}, {69, 110, 4, 2200}, {70, 111, 1, 2200}, {56, 90, 1, 2201}, {57, 94, 4, 2201}, {58, 95, 4, 2201}, {59, 99, 6, 2201}, {60, 100, 12, 2201}, {61, 101, 6, 2201}, {62, 105, 4, 2201}, {63, 106, 12, 2201}, {64, 107, 12, 2201}, {65, 108, 4, 2201}, {66, 112, 1, 2201}, {67, 113, 4, 2201}, {68, 114, 6, 2201}, {69, 115, 4, 2201}, {70, 116, 1, 2201}, {56, 91, 1, 2202}, {57, 95, 4, 2202}, {58, 96, 4, 2202}, {59, 100, 6, 2202}, {60, 101, 12, 2202}, {61, 102, 6, 2202}, {62, 106, 4, 2202}, {63, 107, 12, 2202}, {64, 108, 12, 2202}, {65, 109, 4, 2202}, {66, 113, 1, 2202}, {67, 114, 4, 2202}, {68, 115, 6, 2202}, {69, 116, 4, 2202}, {70, 117, 1, 2202}, {56, 92, 1, 2203}, {57, 96, 4, 2203}, {58, 97, 4, 2203}, {59, 101, 6, 2203}, {60, 102, 12, 2203}, {61, 103, 6, 2203}, {62, 107, 4, 2203}, {63, 108, 12, 2203}, {64, 109, 12, 2203}, {65, 110, 4, 2203}, {66, 114, 1, 2203}, {67, 115, 4, 2203}, {68, 116, 6, 2203}, {69, 117, 4, 2203}, {70, 118, 1, 2203}, {56, 93, 1, 2204}, {57, 97, 4, 2204}, {58, 98, 4, 2204}, {59, 102, 6, 2204}, {60, 103, 12, 2204}, {61, 104, 6, 2204}, {62, 108, 4, 2204}, {63, 109, 12, 2204}, {64, 110, 12, 2204}, {65, 111, 4, 2204}, {66, 115, 1, 2204}, {67, 116, 4, 2204}, {68, 117, 6, 2204}, {69, 118, 4, 2204}, {70, 119, 1, 2204}, {57, 84, 1, 2205}, {59, 85, 4, 2205}, {60, 86, 4, 2205}, {62, 87, 6, 2205}, {63, 88, 12, 2205}, {64, 89, 6, 2205}, {66, 90, 4, 2205}, {67, 91, 12, 2205}, {68, 92, 12, 2205}, {69, 93, 4, 2205}, {71, 94, 1, 2205}, {72, 95, 4, 2205}, {73, 96, 6, 2205}, {74, 97, 4, 2205}, {75, 98, 1, 2205}, {57, 85, 1, 2206}, {59, 87, 4, 2206}, {60, 88, 4, 2206}, {62, 90, 6, 2206}, {63, 91, 12, 2206}, {64, 92, 6, 2206}, {66, 94, 4, 2206}, {67, 95, 12, 2206}, {68, 96, 12, 2206}, {69, 97, 4, 2206}, {71, 99, 1, 2206}, {72, 100, 4, 2206}, {73, 101, 6, 2206}, {74, 102, 4, 2206}, {75, 103, 1, 2206}, {57, 86, 1, 2207}, {59, 88, 4, 2207}, {60, 89, 4, 2207}, {62, 91, 6, 2207}, {63, 92, 12, 2207}, {64, 93, 6, 2207}, {66, 95, 4, 2207}, {67, 96, 12, 2207}, {68, 97, 12, 2207}, {69, 98, 4, 2207}, {71, 100, 1, 2207}, {72, 101, 4, 2207}, {73, 102, 6, 2207}, {74, 103, 4, 2207}, {75, 104, 1, 2207}, {57, 87, 1, 2208}, {59, 90, 4, 2208}, {60, 91, 4, 2208}, {62, 94, 6, 2208}, {63, 95, 12, 2208}, {64, 96, 6, 2208}, {66, 99, 4, 2208}, {67, 100, 12, 2208}, {68, 101, 12, 2208}, {69, 102, 4, 2208}, {71, 105, 1, 2208}, {72, 106, 4, 2208}, {73, 107, 6, 2208}, {74, 108, 4, 2208}, {75, 109, 1, 2208}, {57, 88, 1, 2209}, {59, 91, 4, 2209}, {60, 92, 4, 2209}, {62, 95, 6, 2209}, {63, 96, 12, 2209}, {64, 97, 6, 2209}, {66, 100, 4, 2209}, {67, 101, 12, 2209}, {68, 102, 12, 2209}, {69, 103, 4, 2209}, {71, 106, 1, 2209}, {72, 107, 4, 2209}, {73, 108, 6, 2209}, {74, 109, 4, 2209}, {75, 110, 1, 2209}, {57, 89, 1, 2210}, {59, 92, 4, 2210}, {60, 93, 4, 2210}, {62, 96, 6, 2210}, {63, 97, 12, 2210}, {64, 98, 6, 2210}, {66, 101, 4, 2210}, {67, 102, 12, 2210}, {68, 103, 12, 2210}, {69, 104, 4, 2210}, {71, 107, 1, 2210}, {72, 108, 4, 2210}, {73, 109, 6, 2210}, {74, 110, 4, 2210}, {75, 111, 1, 2210}, {57, 90, 1, 2211}, {59, 94, 4, 2211}, {60, 95, 4, 2211}, {62, 99, 6, 2211}, {63, 100, 12, 2211}, {64, 101, 6, 2211}, {66, 105, 4, 2211}, {67, 106, 12, 2211}, {68, 107, 12, 2211}, {69, 108, 4, 2211}, {71, 112, 1, 2211}, {72, 113, 4, 2211}, {73, 114, 6, 2211}, {74, 115, 4, 2211}, {75, 116, 1, 2211}, {57, 91, 1, 2212}, {59, 95, 4, 2212}, {60, 96, 4, 2212}, {62, 100, 6, 2212}, {63, 101, 12, 2212}, {64, 102, 6, 2212}, {66, 106, 4, 2212}, {67, 107, 12, 2212}, {68, 108, 12, 2212}, {69, 109, 4, 2212}, {71, 113, 1, 2212}, {72, 114, 4, 2212}, {73, 115, 6, 2212}, {74, 116, 4, 2212}, {75, 117, 1, 2212}, {57, 92, 1, 2213}, {59, 96, 4, 2213}, {60, 97, 4, 2213}, {62, 101, 6, 2213}, {63, 102, 12, 2213}, {64, 103, 6, 2213}, {66, 107, 4, 2213}, {67, 108, 12, 2213}, {68, 109, 12, 2213}, {69, 110, 4, 2213}, {71, 114, 1, 2213}, {72, 115, 4, 2213}, {73, 116, 6, 2213}, {74, 117, 4, 2213}, {75, 118, 1, 2213}, {57, 93, 1, 2214}, {59, 97, 4, 2214}, {60, 98, 4, 2214}, {62, 102, 6, 2214}, {63, 103, 12, 2214}, {64, 104, 6, 2214}, {66, 108, 4, 2214}, {67, 109, 12, 2214}, {68, 110, 12, 2214}, {69, 111, 4, 2214}, {71, 115, 1, 2214}, {72, 116, 4, 2214}, {73, 117, 6, 2214}, {74, 118, 4, 2214}, {75, 119, 1, 2214}, {58, 84, 1, 2215}, {60, 85, 4, 2215}, {61, 86, 4, 2215}, {63,

87, 6, 2215}, {64, 88, 12, 2215}, {65, 89, 6, 2215}, {67, 90, 4, 2215}, {68, 91, 12, 2215}, {69, 92, 12, 2215}, {70, 93, 4, 2215}, {72, 94, 1, 2215}, {73, 95, 4, 2215}, {74, 96, 6, 2215}, {75, 97, 4, 2215}, {76, 98, 1, 2215}, {58, 85, 1, 2216}, {60, 87, 4, 2216}, {61, 88, 4, 2216}, {63, 90, 6, 2216}, {64, 91, 12, 2216}, {65, 92, 6, 2216}, {67, 94, 4, 2216}, {68, 95, 12, 2216}, {69, 96, 12, 2216}, {70, 97, 4, 2216}, {72, 99, 1, 2216}, {73, 100, 4, 2216}, {74, 101, 6, 2216}, {75, 102, 4, 2216}, {76, 103, 1, 2216}, {58, 86, 1, 2217}, {60, 88, 4, 2217}, {61, 89, 4, 2217}, {63, 91, 6, 2217}, {64, 92, 12, 2217}, {65, 93, 6, 2217}, {67, 95, 4, 2217}, {68, 96, 12, 2217}, {69, 97, 12, 2217}, {70, 98, 4, 2217}, {72, 100, 1, 2217}, {73, 101, 4, 2217}, {74, 102, 6, 2217}, {75, 103, 4, 2217}, {76, 104, 1, 2217}, {58, 87, 1, 2218}, {60, 90, 4, 2218}, {61, 91, 4, 2218}, {63, 94, 6, 2218}, {64, 95, 12, 2218}, {65, 96, 6, 2218}, {67, 99, 4, 2218}, {68, 100, 12, 2218}, {69, 101, 12, 2218}, {70, 102, 4, 2218}, {72, 105, 1, 2218}, {73, 106, 4, 2218}, {74, 107, 6, 2218}, {75, 108, 4, 2218}, {76, 109, 1, 2218}, {58, 88, 1, 2219}, {60, 91, 4, 2219}, {61, 92, 4, 2219}, {63, 95, 6, 2219}, {64, 96, 12, 2219}, {65, 97, 6, 2219}, {67, 100, 4, 2219}, {68, 101, 12, 2219}, {69, 102, 12, 2219}, {70, 103, 4, 2219}, {72, 106, 1, 2219}, {73, 107, 4, 2219}, {74, 108, 6, 2219}, {75, 109, 4, 2219}, {76, 110, 1, 2219}, {58, 89, 1, 2220}, {60, 92, 4, 2220}, {61, 93, 4, 2220}, {63, 96, 6, 2220}, {64, 97, 12, 2220}, {65, 98, 6, 2220}, {67, 101, 4, 2220}, {68, 102, 12, 2220}, {69, 103, 12, 2220}, {70, 104, 4, 2220}, {72, 107, 1, 2220}, {73, 108, 4, 2220}, {74, 109, 6, 2220}, {75, 110, 4, 2220}, {76, 111, 1, 2220}, {58, 90, 1, 2221}, {60, 94, 4, 2221}, {61, 95, 4, 2221}, {63, 99, 6, 2221}, {64, 100, 12, 2221}, {65, 101, 6, 2221}, {67, 105, 4, 2221}, {68, 106, 12, 2221}, {69, 107, 12, 2221}, {70, 108, 4, 2221}, {72, 112, 1, 2221}, {73, 113, 4, 2221}, {74, 114, 6, 2221}, {75, 115, 4, 2221}, {76, 116, 1, 2221}, {58, 91, 1, 2222}, {60, 95, 4, 2222}, {61, 96, 4, 2222}, {63, 100, 6, 2222}, {64, 101, 12, 2222}, {65, 102, 6, 2222}, {67, 106, 4, 2222}, {68, 107, 12, 2222}, {69, 108, 12, 2222}, {70, 109, 4, 2222}, {72, 113, 1, 2222}, {73, 114, 4, 2222}, {74, 115, 6, 2222}, {75, 116, 4, 2222}, {76, 117, 1, 2222}, {58, 92, 1, 2223}, {60, 96, 4, 2223}, {61, 97, 4, 2223}, {63, 101, 6, 2223}, {64, 102, 12, 2223}, {65, 103, 6, 2223}, {67, 107, 4, 2223}, {68, 108, 12, 2223}, {69, 109, 12, 2223}, {70, 110, 4, 2223}, {72, 114, 1, 2223}, {73, 115, 4, 2223}, {74, 116, 6, 2223}, {75, 117, 4, 2223}, {76, 118, 1, 2223}, {58, 93, 1, 2224}, {60, 97, 4, 2224}, {61, 98, 4, 2224}, {63, 102, 6, 2224}, {64, 103, 12, 2224}, {65, 104, 6, 2224}, {67, 108, 4, 2224}, {68, 109, 12, 2224}, {69, 110, 12, 2224}, {70, 111, 4, 2224}, {72, 115, 1, 2224}, {73, 116, 4, 2224}, {74, 117, 6, 2224}, {75, 118, 4, 2224}, {76, 119, 1, 2224}, {59, 84, 1, 2225}, {62, 85, 4, 2225}, {63, 86, 4, 2225}, {66, 87, 6, 2225}, {67, 88, 12, 2225}, {68, 89, 6, 2225}, {71, 90, 4, 2225}, {72, 91, 12, 2225}, {73, 92, 12, 2225}, {74, 93, 4, 2225}, {77, 94, 1, 2225}, {78, 95, 4, 2225}, {79, 96, 6, 2225}, {80, 97, 4, 2225}, {81, 98, 1, 2225}, {59, 85, 1, 2226}, {62, 87, 4, 2226}, {63, 88, 4, 2226}, {66, 90, 6, 2226}, {67, 91, 12, 2226}, {68, 92, 6, 2226}, {71, 94, 4, 2226}, {72, 95, 12, 2226}, {73, 96, 12, 2226}, {74, 97, 4, 2226}, {77, 99, 1, 2226}, {78, 100, 4, 2226}, {79, 101, 6, 2226}, {80, 102, 4, 2226}, {81, 103, 1, 2226}, {59, 86, 1, 2227}, {62, 88, 4, 2227}, {63, 89, 4, 2227}, {66, 91, 6, 2227}, {67, 92, 12, 2227}, {68, 93, 6, 2227}, {71, 95, 4, 2227}, {72, 96, 12, 2227}, {73, 97, 12, 2227}, {74, 98, 4, 2227}, {77, 100, 1, 2227}, {78, 101, 4, 2227}, {79, 102, 6, 2227}, {80, 103, 4, 2227}, {81, 104, 1, 2227}, {59, 87, 1, 2228}, {62, 90, 4, 2228}, {63, 91, 4, 2228}, {66, 94, 6, 2228}, {67, 95, 12, 2228}, {68, 96, 6, 2228}, {71, 99, 4, 2228}, {72, 100, 12, 2228}, {73, 101, 12, 2228}, {74, 102, 4, 2228}, {77, 105, 1, 2228}, {78, 106, 4, 2228}, {79, 107, 6, 2228}, {80, 108, 4, 2228}, {81, 109, 1, 2228}, {59, 88, 1, 2229}, {62, 91, 4, 2229}, {63, 92, 4, 2229}, {66, 95, 6, 2229}, {67, 96, 12, 2229}, {68, 97, 6, 2229}, {71, 100, 4, 2229}, {72, 101, 12, 2229}, {73, 102, 12, 2229}, {74, 103, 4, 2229}, {77, 106, 1, 2229}, {78, 107, 4, 2229}, {79, 108, 6, 2229}, {80, 109, 4, 2229}, {81, 110, 1, 2229}, {59, 89, 1, 2230}, {62, 92, 4, 2230}, {63, 93, 4, 2230}, {66, 96, 6, 2230}, {67, 97, 12, 2230}, {68, 98, 6, 2230}, {71, 101, 4, 2230}, {72, 102, 12, 2230}, {73, 103, 12, 2230}, {74, 104, 4, 2230}, {77, 107, 1, 2230}, {78, 108, 4, 2230}, {79, 109, 6, 2230}, {80, 110, 4, 2230}, {81, 111, 1, 2230}, {59, 90, 1, 2231}, {62, 94, 4, 2231}, {63, 95, 4, 2231}, {66, 99, 6, 2231}, {67, 100, 12, 2231}, {68, 101, 6, 2231}, {71, 105, 4, 2231}, {72, 106, 12, 2231}, {73, 107, 12, 2231}, {74, 108, 4, 2231}, {77, 112, 1, 2231}, {78, 113, 4, 2231}, {79, 114, 6, 2231}, {80, 115, 4, 2231}, {81, 116, 1, 2231}, {59, 91, 1, 2232}, {62, 95, 4, 2232}, {63, 96, 4, 2232}, {66, 100, 6, 2232}, {67, 101, 12, 2232}, {68, 102, 6, 2232}, {71, 106, 4, 2232}, {72, 107, 12, 2232}, {73, 108, 12, 2232}, {74, 109, 4, 2232}, {77, 113, 1, 2232}, {78, 114, 4, 2232}, {79, 115, 6, 2232}, {80, 116, 4, 2232}, {81, 117, 1, 2232}, {59, 92, 1, 2233}, {62, 96, 4, 2233}, {63, 97, 4, 2233}, {66, 101, 6, 2233}, {67, 102, 12, 2233}, {68, 103, 6, 2233}, {71, 107, 4, 2233}, {72, 108, 12, 2233}, {73, 109, 12, 2233}, {74, 110, 4, 2233}, {77, 114, 1, 2233}, {78, 115, 4, 2233}, {79, 116, 6, 2233}, {80, 117, 4, 2233}, {81, 118, 1, 2233}, {59, 93, 1, 2234}, {62, 97, 4, 2234}, {63, 98, 4, 2234}, {66, 102, 6, 2234}, {67, 103, 12, 2234}, {68, 104, 6, 2234}, {71, 108, 4, 2234}, {72, 109, 12, 2234}, {73, 110, 12, 2234}, {74, 111, 4, 2234}, {77, 115, 1, 2234}, {78, 116, 4, 2234}, {79, 117, 6, 2234}, {80, 118, 4, 2234}, {81, 119, 1, 2234}, {60, 84, 1, 2235}, {63, 85, 4, 2235}, {64, 86, 4, 2235}, {67, 87, 6, 2235}, {68, 88, 12, 2235}, {69, 89, 6, 2235}, {72, 90, 4, 2235}, {73, 91, 12, 2235}, {74, 92, 12, 2235}, {75, 93, 4, 2235}, {78, 94, 1, 2235}, {79, 95, 4, 2235}, {80, 96, 6, 2235}, {81, 97, 4, 2235},

{82, 98, 1, 2235}, {60, 85, 1, 2236}, {63, 87, 4, 2236}, {64, 88, 4, 2236}, {67, 90, 6,
2236}, {68, 91, 12, 2236}, {69, 92, 6, 2236}, {72, 94, 4, 2236}, {73, 95, 12, 2236}, {74,
96, 12, 2236}, {75, 97, 4, 2236}, {78, 99, 1, 2236}, {79, 100, 4, 2236}, {80, 101, 6,
2236}, {81, 102, 4, 2236}, {82, 103, 1, 2236}, {60, 86, 1, 2237}, {63, 88, 4, 2237}, {64,
89, 4, 2237}, {67, 91, 6, 2237}, {68, 92, 12, 2237}, {69, 93, 6, 2237}, {72, 95, 4, 2237},
{73, 96, 12, 2237}, {74, 97, 12, 2237}, {75, 98, 4, 2237}, {78, 100, 1, 2237}, {79, 101,
4, 2237}, {80, 102, 6, 2237}, {81, 103, 4, 2237}, {82, 104, 1, 2237}, {60, 87, 1, 2238},
{63, 90, 4, 2238}, {64, 91, 4, 2238}, {67, 94, 6, 2238}, {68, 95, 12, 2238}, {69, 96, 6,
2238}, {72, 99, 4, 2238}, {73, 100, 12, 2238}, {74, 101, 12, 2238}, {75, 102, 4, 2238},
{78, 105, 1, 2238}, {79, 106, 4, 2238}, {80, 107, 6, 2238}, {81, 108, 4, 2238}, {82, 109,
1, 2238}, {60, 88, 1, 2239}, {63, 91, 4, 2239}, {64, 92, 4, 2239}, {67, 95, 6, 2239}, {68,
96, 12, 2239}, {69, 97, 6, 2239}, {72, 100, 4, 2239}, {73, 101, 12, 2239}, {74, 102, 12,
2239}, {75, 103, 4, 2239}, {78, 106, 1, 2239}, {79, 107, 4, 2239}, {80, 108, 6, 2239},
{81, 109, 4, 2239}, {82, 110, 1, 2239}, {60, 89, 1, 2240}, {63, 92, 4, 2240}, {64, 93, 4,
2240}, {67, 96, 6, 2240}, {68, 97, 12, 2240}, {69, 98, 6, 2240}, {72, 101, 4, 2240}, {73,
102, 12, 2240}, {74, 103, 12, 2240}, {75, 104, 4, 2240}, {78, 107, 1, 2240}, {79, 108, 4,
2240}, {80, 109, 6, 2240}, {81, 110, 4, 2240}, {82, 111, 1, 2240}, {60, 90, 1, 2241}, {63,
94, 4, 2241}, {64, 95, 4, 2241}, {67, 99, 6, 2241}, {68, 100, 12, 2241}, {69, 101, 6,
2241}, {72, 105, 4, 2241}, {73, 106, 12, 2241}, {74, 107, 12, 2241}, {75, 108, 4, 2241},
{78, 112, 1, 2241}, {79, 113, 4, 2241}, {80, 114, 6, 2241}, {81, 115, 4, 2241}, {82, 116,
1, 2241}, {60, 91, 1, 2242}, {63, 95, 4, 2242}, {64, 96, 4, 2242}, {67, 100, 6, 2242},
{68, 101, 12, 2242}, {69, 102, 6, 2242}, {72, 106, 4, 2242}, {73, 107, 12, 2242}, {74,
108, 12, 2242}, {75, 109, 4, 2242}, {78, 113, 1, 2242}, {79, 114, 4, 2242}, {80, 115, 6,
2242}, {81, 116, 4, 2242}, {82, 117, 1, 2242}, {60, 92, 1, 2243}, {63, 96, 4, 2243}, {64,
97, 4, 2243}, {67, 101, 6, 2243}, {68, 102, 12, 2243}, {69, 103, 6, 2243}, {72, 107, 4,
2243}, {73, 108, 12, 2243}, {74, 109, 12, 2243}, {75, 110, 4, 2243}, {78, 114, 1, 2243},
{79, 115, 4, 2243}, {80, 116, 6, 2243}, {81, 117, 4, 2243}, {82, 118, 1, 2243}, {60, 93,
1, 2244}, {63, 97, 4, 2244}, {64, 98, 4, 2244}, {67, 102, 6, 2244}, {68, 103, 12, 2244},
{69, 104, 6, 2244}, {72, 108, 4, 2244}, {73, 109, 12, 2244}, {74, 110, 12, 2244}, {75,
111, 4, 2244}, {78, 115, 1, 2244}, {79, 116, 4, 2244}, {80, 117, 6, 2244}, {81, 118, 4,
2244}, {82, 119, 1, 2244}, {61, 84, 1, 2245}, {64, 85, 4, 2245}, {65, 86, 4, 2245}, {68,
87, 6, 2245}, {69, 88, 12, 2245}, {70, 89, 6, 2245}, {73, 90, 4, 2245}, {74, 91, 12,
2245}, {75, 92, 12, 2245}, {76, 93, 4, 2245}, {79, 94, 1, 2245}, {80, 95, 4, 2245}, {81,
96, 6, 2245}, {82, 97, 4, 2245}, {83, 98, 1, 2245}, {61, 85, 1, 2246}, {64, 87, 4, 2246},
{65, 88, 4, 2246}, {68, 90, 6, 2246}, {69, 91, 12, 2246}, {70, 92, 6, 2246}, {73, 94, 4,
2246}, {74, 95, 12, 2246}, {75, 96, 12, 2246}, {76, 97, 4, 2246}, {79, 99, 1, 2246}, {80,
100, 4, 2246}, {81, 101, 6, 2246}, {82, 102, 4, 2246}, {83, 103, 1, 2246}, {61, 86, 1,
2247}, {64, 88, 4, 2247}, {65, 89, 4, 2247}, {68, 91, 6, 2247}, {69, 92, 12, 2247}, {70,
93, 6, 2247}, {73, 95, 4, 2247}, {74, 96, 12, 2247}, {75, 97, 12, 2247}, {76, 98, 4,
2247}, {79, 100, 1, 2247}, {80, 101, 4, 2247}, {81, 102, 6, 2247}, {82, 103, 4, 2247},
{83, 104, 1, 2247}, {61, 87, 1, 2248}, {64, 90, 4, 2248}, {65, 91, 4, 2248}, {68, 94, 6,
2248}, {69, 95, 12, 2248}, {70, 96, 6, 2248}, {73, 99, 4, 2248}, {74, 100, 12, 2248}, {75,
101, 12, 2248}, {76, 102, 4, 2248}, {79, 105, 1, 2248}, {80, 106, 4, 2248}, {81, 107, 6,
2248}, {82, 108, 4, 2248}, {83, 109, 1, 2248}, {61, 88, 1, 2249}, {64, 91, 4, 2249}, {65,
92, 4, 2249}, {68, 95, 6, 2249}, {69, 96, 12, 2249}, {70, 97, 6, 2249}, {73, 100, 4,
2249}, {74, 101, 12, 2249}, {75, 102, 12, 2249}, {76, 103, 4, 2249}, {79, 106, 1, 2249},
{80, 107, 4, 2249}, {81, 108, 6, 2249}, {82, 109, 4, 2249}, {83, 110, 1, 2249}, {61, 89,
1, 2250}, {64, 92, 4, 2250}, {65, 93, 4, 2250}, {68, 96, 6, 2250}, {69, 97, 12, 2250},
{70, 98, 6, 2250}, {73, 101, 4, 2250}, {74, 102, 12, 2250}, {75, 103, 12, 2250}, {76, 104,
4, 2250}, {79, 107, 1, 2250}, {80, 108, 4, 2250}, {81, 109, 6, 2250}, {82, 110, 4, 2250},
{83, 111, 1, 2250}, {61, 90, 1, 2251}, {64, 94, 4, 2251}, {65, 95, 4, 2251}, {68, 99, 6,
2251}, {69, 100, 12, 2251}, {70, 101, 6, 2251}, {73, 105, 4, 2251}, {74, 106, 12, 2251},
{75, 107, 12, 2251}, {76, 108, 4, 2251}, {79, 112, 1, 2251}, {80, 113, 4, 2251}, {81, 114,
6, 2251}, {82, 115, 4, 2251}, {83, 116, 1, 2251}, {61, 91, 1, 2252}, {64, 95, 4, 2252},
{65, 96, 4, 2252}, {68, 100, 6, 2252}, {69, 101, 12, 2252}, {70, 102, 6, 2252}, {73, 106,
4, 2252}, {74, 107, 12, 2252}, {75, 108, 12, 2252}, {76, 109, 4, 2252}, {79, 113, 1,
2252}, {80, 114, 4, 2252}, {81, 115, 6, 2252}, {82, 116, 4, 2252}, {83, 117, 1, 2252},
{61, 92, 1, 2253}, {64, 96, 4, 2253}, {65, 97, 4, 2253}, {68, 101, 6, 2253}, {69, 102, 12,
2253}, {70, 103, 6, 2253}, {73, 107, 4, 2253}, {74, 108, 12, 2253}, {75, 109, 12, 2253},
{76, 110, 4, 2253}, {79, 114, 1, 2253}, {80, 115, 4, 2253}, {81, 116, 6, 2253}, {82, 117,
4, 2253}, {83, 118, 1, 2253}, {61, 93, 1, 2254}, {64, 97, 4, 2254}, {65, 98, 4, 2254},
{68, 102, 6, 2254}, {69, 103, 12, 2254}, {70, 104, 6, 2254}, {73, 108, 4, 2254}, {74, 109,
12, 2254}, {75, 110, 12, 2254}, {76, 111, 4, 2254}, {79, 115, 1, 2254}, {80, 116, 4,
2254}, {81, 117, 6, 2254}, {82, 118, 4, 2254}, {83, 119, 1, 2254}, {56, 120, 1, 2255},
{57, 121, 4, 2255}, {58, 122, 4, 2255}, {59, 123, 6, 2255}, {60, 124, 12, 2255}, {61, 125,
6, 2255}, {62, 126, 4, 2255}, {63, 127, 12, 2255}, {64, 128, 12, 2255}, {65, 129, 4,
2255}, {66, 130, 1, 2255}, {67, 131, 4, 2255}, {68, 132, 6, 2255}, {69, 133, 4, 2255},
{70, 134, 1, 2255}, {56, 121, 1, 2256}, {57, 123, 4, 2256}, {58, 124, 4, 2256}, {59, 126,
6, 2256}, {60, 127, 12, 2256}, {61, 128, 6, 2256}, {62, 130, 4, 2256}, {63, 131, 12,

2256}, {64, 132, 12, 2256}, {65, 133, 4, 2256}, {66, 135, 1, 2256}, {67, 136, 4, 2256}, {68, 137, 6, 2256}, {69, 138, 4, 2256}, {70, 139, 1, 2256}, {56, 122, 1, 2257}, {57, 124, 4, 2257}, {58, 125, 4, 2257}, {59, 127, 6, 2257}, {60, 128, 12, 2257}, {61, 129, 6, 2257}, {62, 131, 4, 2257}, {63, 132, 12, 2257}, {64, 133, 12, 2257}, {65, 134, 4, 2257}, {66, 136, 1, 2257}, {67, 137, 4, 2257}, {68, 138, 6, 2257}, {69, 139, 4, 2257}, {70, 140, 1, 2257}, {56, 123, 1, 2258}, {57, 126, 4, 2258}, {58, 127, 4, 2258}, {59, 130, 6, 2258}, {60, 131, 12, 2258}, {61, 132, 6, 2258}, {62, 135, 4, 2258}, {63, 136, 12, 2258}, {64, 137, 12, 2258}, {65, 138, 4, 2258}, {66, 141, 1, 2258}, {67, 142, 4, 2258}, {68, 143, 6, 2258}, {69, 144, 4, 2258}, {70, 145, 1, 2258}, {56, 124, 1, 2259}, {57, 127, 4, 2259}, {58, 128, 4, 2259}, {59, 131, 6, 2259}, {60, 132, 12, 2259}, {61, 133, 6, 2259}, {62, 136, 4, 2259}, {63, 137, 12, 2259}, {64, 138, 12, 2259}, {65, 139, 4, 2259}, {66, 142, 1, 2259}, {67, 143, 4, 2259}, {68, 144, 6, 2259}, {69, 145, 4, 2259}, {70, 146, 1, 2259}, {56, 125, 1, 2260}, {57, 128, 4, 2260}, {58, 129, 4, 2260}, {59, 132, 6, 2260}, {60, 133, 12, 2260}, {61, 134, 6, 2260}, {62, 137, 4, 2260}, {63, 138, 12, 2260}, {64, 139, 12, 2260}, {65, 140, 4, 2260}, {66, 143, 1, 2260}, {67, 144, 4, 2260}, {68, 145, 6, 2260}, {69, 146, 4, 2260}, {70, 147, 1, 2260}, {56, 126, 1, 2261}, {57, 130, 4, 2261}, {58, 131, 4, 2261}, {59, 135, 6, 2261}, {60, 136, 12, 2261}, {61, 137, 6, 2261}, {62, 141, 4, 2261}, {63, 142, 12, 2261}, {64, 143, 12, 2261}, {65, 144, 4, 2261}, {66, 148, 1, 2261}, {67, 149, 4, 2261}, {68, 150, 6, 2261}, {69, 151, 4, 2261}, {70, 152, 1, 2261}, {56, 127, 1, 2262}, {57, 131, 4, 2262}, {58, 132, 4, 2262}, {59, 136, 6, 2262}, {60, 137, 12, 2262}, {61, 138, 6, 2262}, {62, 142, 4, 2262}, {63, 143, 12, 2262}, {64, 144, 12, 2262}, {65, 145, 4, 2262}, {66, 149, 1, 2262}, {67, 150, 4, 2262}, {68, 151, 6, 2262}, {69, 152, 4, 2262}, {70, 153, 1, 2262}, {56, 128, 1, 2263}, {57, 132, 4, 2263}, {58, 133, 4, 2263}, {59, 137, 6, 2263}, {60, 138, 12, 2263}, {61, 139, 6, 2263}, {62, 143, 4, 2263}, {63, 144, 12, 2263}, {64, 145, 12, 2263}, {65, 146, 4, 2263}, {66, 150, 1, 2263}, {67, 151, 4, 2263}, {68, 152, 6, 2263}, {69, 153, 4, 2263}, {70, 154, 1, 2263}, {56, 129, 1, 2264}, {57, 133, 4, 2264}, {58, 134, 4, 2264}, {59, 138, 6, 2264}, {60, 139, 12, 2264}, {61, 140, 6, 2264}, {62, 144, 4, 2264}, {63, 145, 12, 2264}, {64, 146, 12, 2264}, {65, 147, 4, 2264}, {66, 151, 1, 2264}, {67, 152, 4, 2264}, {68, 153, 6, 2264}, {69, 154, 4, 2264}, {70, 155, 1, 2264}, {56, 130, 1, 2265}, {57, 135, 4, 2265}, {58, 136, 4, 2265}, {59, 141, 6, 2265}, {60, 142, 12, 2265}, {61, 143, 6, 2265}, {62, 148, 4, 2265}, {63, 149, 12, 2265}, {64, 150, 12, 2265}, {65, 151, 4, 2265}, {66, 156, 1, 2265}, {67, 157, 4, 2265}, {68, 158, 6, 2265}, {69, 159, 4, 2265}, {70, 160, 1, 2265}, {56, 131, 1, 2266}, {57, 136, 4, 2266}, {58, 137, 4, 2266}, {59, 142, 6, 2266}, {60, 143, 12, 2266}, {61, 144, 6, 2266}, {62, 149, 4, 2266}, {63, 150, 12, 2266}, {64, 151, 12, 2266}, {65, 152, 4, 2266}, {66, 157, 1, 2266}, {67, 158, 4, 2266}, {68, 159, 6, 2266}, {69, 160, 4, 2266}, {70, 161, 1, 2266}, {56, 132, 1, 2267}, {57, 137, 4, 2267}, {58, 138, 4, 2267}, {59, 143, 6, 2267}, {60, 144, 12, 2267}, {61, 145, 6, 2267}, {62, 150, 4, 2267}, {63, 151, 12, 2267}, {64, 152, 12, 2267}, {65, 153, 4, 2267}, {66, 158, 1, 2267}, {67, 159, 4, 2267}, {68, 160, 6, 2267}, {69, 161, 4, 2267}, {70, 162, 1, 2267}, {56, 133, 1, 2268}, {57, 138, 4, 2268}, {58, 139, 4, 2268}, {59, 144, 6, 2268}, {60, 145, 12, 2268}, {61, 146, 6, 2268}, {62, 151, 4, 2268}, {63, 152, 12, 2268}, {64, 153, 12, 2268}, {65, 154, 4, 2268}, {66, 159, 1, 2268}, {67, 160, 4, 2268}, {68, 161, 6, 2268}, {69, 162, 4, 2268}, {70, 163, 1, 2268}, {56, 134, 1, 2269}, {57, 139, 4, 2269}, {58, 140, 4, 2269}, {59, 145, 6, 2269}, {60, 146, 12, 2269}, {61, 147, 6, 2269}, {62, 152, 4, 2269}, {63, 153, 12, 2269}, {64, 154, 12, 2269}, {65, 155, 4, 2269}, {66, 160, 1, 2269}, {67, 161, 4, 2269}, {68, 162, 6, 2269}, {69, 163, 4, 2269}, {70, 164, 1, 2269}, {57, 120, 1, 2270}, {59, 121, 4, 2270}, {60, 122, 4, 2270}, {62, 123, 6, 2270}, {63, 124, 12, 2270}, {64, 125, 6, 2270}, {66, 126, 4, 2270}, {67, 127, 12, 2270}, {68, 128, 12, 2270}, {69, 129, 4, 2270}, {71, 130, 1, 2270}, {72, 131, 4, 2270}, {73, 132, 6, 2270}, {74, 133, 4, 2270}, {75, 134, 1, 2270}, {57, 121, 1, 2271}, {59, 123, 4, 2271}, {60, 124, 4, 2271}, {62, 126, 6, 2271}, {63, 127, 12, 2271}, {64, 128, 6, 2271}, {66, 130, 4, 2271}, {67, 131, 12, 2271}, {68, 132, 12, 2271}, {69, 133, 4, 2271}, {71, 135, 1, 2271}, {72, 136, 4, 2271}, {73, 137, 6, 2271}, {74, 138, 4, 2271}, {75, 139, 1, 2271}, {57, 122, 1, 2272}, {59, 124, 4, 2272}, {60, 125, 4, 2272}, {62, 127, 6, 2272}, {63, 128, 12, 2272}, {64, 129, 6, 2272}, {66, 131, 4, 2272}, {67, 132, 12, 2272}, {68, 133, 12, 2272}, {69, 134, 4, 2272}, {71, 136, 1, 2272}, {72, 137, 4, 2272}, {73, 138, 6, 2272}, {74, 139, 4, 2272}, {75, 140, 1, 2272}, {57, 123, 1, 2273}, {59, 126, 4, 2273}, {60, 127, 4, 2273}, {62, 130, 6, 2273}, {63, 131, 12, 2273}, {64, 132, 6, 2273}, {66, 135, 4, 2273}, {67, 136, 12, 2273}, {68, 137, 12, 2273}, {69, 138, 4, 2273}, {71, 141, 1, 2273}, {72, 142, 4, 2273}, {73, 143, 6, 2273}, {74, 144, 4, 2273}, {75, 145, 1, 2273}, {57, 124, 1, 2274}, {59, 127, 4, 2274}, {60, 128, 4, 2274}, {62, 131, 6, 2274}, {63, 132, 12, 2274}, {64, 133, 6, 2274}, {66, 136, 4, 2274}, {67, 137, 12, 2274}, {68, 138, 12, 2274}, {69, 139, 4, 2274}, {71, 142, 1, 2274}, {72, 143, 4, 2274}, {73, 144, 6, 2274}, {74, 145, 4, 2274}, {75, 146, 1, 2274}, {57, 125, 1, 2275}, {59, 128, 4, 2275}, {60, 129, 4, 2275}, {62, 132, 6, 2275}, {63, 133, 12, 2275}, {64, 134, 6, 2275}, {66, 137, 4, 2275}, {67, 138, 12, 2275}, {68, 139, 12, 2275}, {69, 140, 4, 2275}, {71, 143, 1, 2275}, {72, 144, 4, 2275}, {73, 145, 6, 2275}, {74, 146, 4, 2275}, {75, 147, 1, 2275}, {57, 126, 1, 2276}, {59, 130, 4, 2276}, {60, 131, 4, 2276}, {62, 135, 6, 2276}, {63, 136, 12, 2276}, {64, 137, 6, 2276}, {66, 141, 4, 2276}, {67, 142, 12, 2276}, {68,

143, 12, 2276}, {69, 144, 4, 2276}, {71, 148, 1, 2276}, {72, 149, 4, 2276}, {73, 150, 6, 2276}, {74, 151, 4, 2276}, {75, 152, 1, 2276}, {57, 127, 1, 2277}, {59, 131, 4, 2277}, {60, 132, 4, 2277}, {62, 136, 6, 2277}, {63, 137, 12, 2277}, {64, 138, 6, 2277}, {66, 142, 4, 2277}, {67, 143, 12, 2277}, {68, 144, 12, 2277}, {69, 145, 4, 2277}, {71, 149, 1, 2277}, {72, 150, 4, 2277}, {73, 151, 6, 2277}, {74, 152, 4, 2277}, {75, 153, 1, 2277}, {57, 128, 1, 2278}, {59, 132, 4, 2278}, {60, 133, 4, 2278}, {62, 137, 6, 2278}, {63, 138, 12, 2278}, {64, 139, 6, 2278}, {66, 143, 4, 2278}, {67, 144, 12, 2278}, {68, 145, 12, 2278}, {69, 146, 4, 2278}, {71, 150, 1, 2278}, {72, 151, 4, 2278}, {73, 152, 6, 2278}, {74, 153, 4, 2278}, {75, 154, 1, 2278}, {57, 129, 1, 2279}, {59, 133, 4, 2279}, {60, 134, 4, 2279}, {62, 138, 6, 2279}, {63, 139, 12, 2279}, {64, 140, 6, 2279}, {66, 144, 4, 2279}, {67, 145, 12, 2279}, {68, 146, 12, 2279}, {69, 147, 4, 2279}, {71, 151, 1, 2279}, {72, 152, 4, 2279}, {73, 153, 6, 2279}, {74, 154, 4, 2279}, {75, 155, 1, 2279}, {57, 130, 1, 2280}, {59, 135, 4, 2280}, {60, 136, 4, 2280}, {62, 141, 6, 2280}, {63, 142, 12, 2280}, {64, 143, 6, 2280}, {66, 148, 4, 2280}, {67, 149, 12, 2280}, {68, 150, 12, 2280}, {69, 151, 4, 2280}, {71, 156, 1, 2280}, {72, 157, 4, 2280}, {73, 158, 6, 2280}, {74, 159, 4, 2280}, {75, 160, 1, 2280}, {57, 131, 1, 2281}, {59, 136, 4, 2281}, {60, 137, 4, 2281}, {62, 142, 6, 2281}, {63, 143, 12, 2281}, {64, 144, 6, 2281}, {66, 149, 4, 2281}, {67, 150, 12, 2281}, {68, 151, 12, 2281}, {69, 152, 4, 2281}, {71, 157, 1, 2281}, {72, 158, 4, 2281}, {73, 159, 6, 2281}, {74, 160, 4, 2281}, {75, 161, 1, 2281}, {57, 132, 1, 2282}, {59, 137, 4, 2282}, {60, 138, 4, 2282}, {62, 143, 6, 2282}, {63, 144, 12, 2282}, {64, 145, 6, 2282}, {66, 150, 4, 2282}, {67, 151, 12, 2282}, {68, 152, 12, 2282}, {69, 153, 4, 2282}, {71, 158, 1, 2282}, {72, 159, 4, 2282}, {73, 160, 6, 2282}, {74, 161, 4, 2282}, {75, 162, 1, 2282}, {57, 133, 1, 2283}, {59, 138, 4, 2283}, {60, 139, 4, 2283}, {62, 144, 6, 2283}, {63, 145, 12, 2283}, {64, 146, 6, 2283}, {66, 151, 4, 2283}, {67, 152, 12, 2283}, {68, 153, 12, 2283}, {69, 154, 4, 2283}, {71, 159, 1, 2283}, {72, 160, 4, 2283}, {73, 161, 6, 2283}, {74, 162, 4, 2283}, {75, 163, 1, 2283}, {57, 134, 1, 2284}, {59, 139, 4, 2284}, {60, 140, 4, 2284}, {62, 145, 6, 2284}, {63, 146, 12, 2284}, {64, 147, 6, 2284}, {66, 152, 4, 2284}, {67, 153, 12, 2284}, {68, 154, 12, 2284}, {69, 155, 4, 2284}, {71, 160, 1, 2284}, {72, 161, 4, 2284}, {73, 162, 6, 2284}, {74, 163, 4, 2284}, {75, 164, 1, 2284}, {58, 120, 1, 2285}, {60, 121, 4, 2285}, {61, 122, 4, 2285}, {63, 123, 6, 2285}, {64, 124, 12, 2285}, {65, 125, 6, 2285}, {67, 126, 4, 2285}, {68, 127, 12, 2285}, {69, 128, 12, 2285}, {70, 129, 4, 2285}, {72, 130, 1, 2285}, {73, 131, 4, 2285}, {74, 132, 6, 2285}, {75, 133, 4, 2285}, {76, 134, 1, 2285}, {58, 121, 1, 2286}, {60, 123, 4, 2286}, {61, 124, 4, 2286}, {63, 126, 6, 2286}, {64, 127, 12, 2286}, {65, 128, 6, 2286}, {67, 130, 4, 2286}, {68, 131, 12, 2286}, {69, 132, 12, 2286}, {70, 133, 4, 2286}, {72, 135, 1, 2286}, {73, 136, 4, 2286}, {74, 137, 6, 2286}, {75, 138, 4, 2286}, {76, 139, 1, 2286}, {58, 122, 1, 2287}, {60, 124, 4, 2287}, {61, 125, 4, 2287}, {63, 127, 6, 2287}, {64, 128, 12, 2287}, {65, 129, 6, 2287}, {67, 131, 4, 2287}, {68, 132, 12, 2287}, {69, 133, 12, 2287}, {70, 134, 4, 2287}, {72, 136, 1, 2287}, {73, 137, 4, 2287}, {74, 138, 6, 2287}, {75, 139, 4, 2287}, {76, 140, 1, 2287}, {58, 123, 1, 2288}, {60, 126, 4, 2288}, {61, 127, 4, 2288}, {63, 130, 6, 2288}, {64, 131, 12, 2288}, {65, 132, 6, 2288}, {67, 135, 4, 2288}, {68, 136, 12, 2288}, {69, 137, 12, 2288}, {70, 138, 4, 2288}, {72, 141, 1, 2288}, {73, 142, 4, 2288}, {74, 143, 6, 2288}, {75, 144, 4, 2288}, {76, 145, 1, 2288}, {58, 124, 1, 2289}, {60, 127, 4, 2289}, {61, 128, 4, 2289}, {63, 131, 6, 2289}, {64, 132, 12, 2289}, {65, 133, 6, 2289}, {67, 136, 4, 2289}, {68, 137, 12, 2289}, {69, 138, 12, 2289}, {70, 139, 4, 2289}, {72, 142, 1, 2289}, {73, 143, 4, 2289}, {74, 144, 6, 2289}, {75, 145, 4, 2289}, {76, 146, 1, 2289}, {58, 125, 1, 2290}, {60, 128, 4, 2290}, {61, 129, 4, 2290}, {63, 132, 6, 2290}, {64, 133, 12, 2290}, {65, 134, 6, 2290}, {67, 137, 4, 2290}, {68, 138, 12, 2290}, {69, 139, 12, 2290}, {70, 140, 4, 2290}, {72, 143, 1, 2290}, {73, 144, 4, 2290}, {74, 145, 6, 2290}, {75, 146, 4, 2290}, {76, 147, 1, 2290}, {58, 126, 1, 2291}, {60, 130, 4, 2291}, {61, 131, 4, 2291}, {63, 135, 6, 2291}, {64, 136, 12, 2291}, {65, 137, 6, 2291}, {67, 141, 4, 2291}, {68, 142, 12, 2291}, {69, 143, 12, 2291}, {70, 144, 4, 2291}, {72, 148, 1, 2291}, {73, 149, 4, 2291}, {74, 150, 6, 2291}, {75, 151, 4, 2291}, {76, 152, 1, 2291}, {58, 127, 1, 2292}, {60, 131, 4, 2292}, {61, 132, 4, 2292}, {63, 136, 6, 2292}, {64, 137, 12, 2292}, {65, 138, 6, 2292}, {67, 142, 4, 2292}, {68, 143, 12, 2292}, {69, 144, 12, 2292}, {70, 145, 4, 2292}, {72, 149, 1, 2292}, {73, 150, 4, 2292}, {74, 151, 6, 2292}, {75, 152, 4, 2292}, {76, 153, 1, 2292}, {58, 128, 1, 2293}, {60, 132, 4, 2293}, {61, 133, 4, 2293}, {63, 137, 6, 2293}, {64, 138, 12, 2293}, {65, 139, 6, 2293}, {67, 143, 4, 2293}, {68, 144, 12, 2293}, {69, 145, 12, 2293}, {70, 146, 4, 2293}, {72, 150, 1, 2293}, {73, 151, 4, 2293}, {74, 152, 6, 2293}, {75, 153, 4, 2293}, {76, 154, 1, 2293}, {58, 129, 1, 2294}, {60, 133, 4, 2294}, {61, 134, 4, 2294}, {63, 138, 6, 2294}, {64, 139, 12, 2294}, {65, 140, 6, 2294}, {67, 144, 4, 2294}, {68, 145, 12, 2294}, {69, 146, 12, 2294}, {70, 147, 4, 2294}, {72, 151, 1, 2294}, {73, 152, 4, 2294}, {74, 153, 6, 2294}, {75, 154, 4, 2294}, {76, 155, 1, 2294}, {58, 130, 1, 2295}, {60, 135, 4, 2295}, {61, 136, 4, 2295}, {63, 141, 6, 2295}, {64, 142, 12, 2295}, {65, 143, 6, 2295}, {67, 148, 4, 2295}, {68, 149, 12, 2295}, {69, 150, 12, 2295}, {70, 151, 4, 2295}, {72, 156, 1, 2295}, {73, 157, 4, 2295}, {74, 158, 6, 2295}, {75, 159, 4, 2295}, {76, 160, 1, 2295}, {58, 131, 1, 2296}, {60, 136, 4, 2296}, {61, 137, 4, 2296}, {63, 142, 6, 2296}, {64, 143, 12, 2296}, {65, 144, 6, 2296}, {67, 149, 4, 2296}, {68, 150, 12, 2296}, {69, 151, 12,

2296}, {70, 152, 4, 2296}, {72, 157, 1, 2296}, {73, 158, 4, 2296}, {74, 159, 6, 2296}, {75, 160, 4, 2296}, {76, 161, 1, 2296}, {58, 132, 1, 2297}, {60, 137, 4, 2297}, {61, 138, 4, 2297}, {63, 143, 6, 2297}, {64, 144, 12, 2297}, {65, 145, 6, 2297}, {67, 150, 4, 2297}, {68, 151, 12, 2297}, {69, 152, 12, 2297}, {70, 153, 4, 2297}, {72, 158, 1, 2297}, {73, 159, 4, 2297}, {74, 160, 6, 2297}, {75, 161, 4, 2297}, {76, 162, 1, 2297}, {58, 133, 1, 2298}, {60, 138, 4, 2298}, {61, 139, 4, 2298}, {63, 144, 6, 2298}, {64, 145, 12, 2298}, {65, 146, 6, 2298}, {67, 151, 4, 2298}, {68, 152, 12, 2298}, {69, 153, 12, 2298}, {70, 154, 4, 2298}, {72, 159, 1, 2298}, {73, 160, 4, 2298}, {74, 161, 6, 2298}, {75, 162, 4, 2298}, {76, 163, 1, 2298}, {58, 134, 1, 2299}, {60, 139, 4, 2299}, {61, 140, 4, 2299}, {63, 145, 6, 2299}, {64, 146, 12, 2299}, {65, 147, 6, 2299}, {67, 152, 4, 2299}, {68, 153, 12, 2299}, {69, 154, 12, 2299}, {70, 155, 4, 2299}, {72, 160, 1, 2299}, {73, 161, 4, 2299}, {74, 162, 6, 2299}, {75, 163, 4, 2299}, {76, 164, 1, 2299}, {59, 120, 1, 2300}, {62, 121, 4, 2300}, {63, 122, 4, 2300}, {66, 123, 6, 2300}, {67, 124, 12, 2300}, {68, 125, 6, 2300}, {71, 126, 4, 2300}, {72, 127, 12, 2300}, {73, 128, 12, 2300}, {74, 129, 4, 2300}, {77, 130, 1, 2300}, {78, 131, 4, 2300}, {79, 132, 6, 2300}, {80, 133, 4, 2300}, {81, 134, 1, 2300}, {59, 121, 1, 2301}, {62, 123, 4, 2301}, {63, 124, 4, 2301}, {66, 126, 6, 2301}, {67, 127, 12, 2301}, {68, 128, 6, 2301}, {71, 130, 4, 2301}, {72, 131, 12, 2301}, {73, 132, 12, 2301}, {74, 133, 4, 2301}, {77, 135, 1, 2301}, {78, 136, 4, 2301}, {79, 137, 6, 2301}, {80, 138, 4, 2301}, {81, 139, 1, 2301}, {59, 122, 1, 2302}, {62, 124, 4, 2302}, {63, 125, 4, 2302}, {66, 127, 6, 2302}, {67, 128, 12, 2302}, {68, 129, 6, 2302}, {71, 131, 4, 2302}, {72, 132, 12, 2302}, {73, 133, 12, 2302}, {74, 134, 4, 2302}, {77, 136, 1, 2302}, {78, 137, 4, 2302}, {79, 138, 6, 2302}, {80, 139, 4, 2302}, {81, 140, 1, 2302}, {59, 123, 1, 2303}, {62, 126, 4, 2303}, {63, 127, 4, 2303}, {66, 130, 6, 2303}, {67, 131, 12, 2303}, {68, 132, 6, 2303}, {71, 135, 4, 2303}, {72, 136, 12, 2303}, {73, 137, 12, 2303}, {74, 138, 4, 2303}, {77, 141, 1, 2303}, {78, 142, 4, 2303}, {79, 143, 6, 2303}, {80, 144, 4, 2303}, {81, 145, 1, 2303}, {59, 124, 1, 2304}, {62, 127, 4, 2304}, {63, 128, 4, 2304}, {66, 131, 6, 2304}, {67, 132, 12, 2304}, {68, 133, 6, 2304}, {71, 136, 4, 2304}, {72, 137, 12, 2304}, {73, 138, 12, 2304}, {74, 139, 4, 2304}, {77, 142, 1, 2304}, {78, 143, 4, 2304}, {79, 144, 6, 2304}, {80, 145, 4, 2304}, {81, 146, 1, 2304}, {59, 125, 1, 2305}, {62, 128, 4, 2305}, {63, 129, 4, 2305}, {66, 132, 6, 2305}, {67, 133, 12, 2305}, {68, 134, 6, 2305}, {71, 137, 4, 2305}, {72, 138, 12, 2305}, {73, 139, 12, 2305}, {74, 140, 4, 2305}, {77, 143, 1, 2305}, {78, 144, 4, 2305}, {79, 145, 6, 2305}, {80, 146, 4, 2305}, {81, 147, 1, 2305}, {59, 126, 1, 2306}, {62, 130, 4, 2306}, {63, 131, 4, 2306}, {66, 135, 6, 2306}, {67, 136, 12, 2306}, {68, 137, 6, 2306}, {71, 141, 4, 2306}, {72, 142, 12, 2306}, {73, 143, 12, 2306}, {74, 144, 4, 2306}, {77, 148, 1, 2306}, {78, 149, 4, 2306}, {79, 150, 6, 2306}, {80, 151, 4, 2306}, {81, 152, 1, 2306}, {59, 127, 1, 2307}, {62, 131, 4, 2307}, {63, 132, 4, 2307}, {66, 136, 6, 2307}, {67, 137, 12, 2307}, {68, 138, 6, 2307}, {71, 142, 4, 2307}, {72, 143, 12, 2307}, {73, 144, 12, 2307}, {74, 145, 4, 2307}, {77, 149, 1, 2307}, {78, 150, 4, 2307}, {79, 151, 6, 2307}, {80, 152, 4, 2307}, {81, 153, 1, 2307}, {59, 128, 1, 2308}, {62, 132, 4, 2308}, {63, 133, 4, 2308}, {66, 137, 6, 2308}, {67, 138, 12, 2308}, {68, 139, 6, 2308}, {71, 143, 4, 2308}, {72, 144, 12, 2308}, {73, 145, 12, 2308}, {74, 146, 4, 2308}, {77, 150, 1, 2308}, {78, 151, 4, 2308}, {79, 152, 6, 2308}, {80, 153, 4, 2308}, {81, 154, 1, 2308}, {59, 129, 1, 2309}, {62, 133, 4, 2309}, {63, 134, 4, 2309}, {66, 138, 6, 2309}, {67, 139, 12, 2309}, {68, 140, 6, 2309}, {71, 144, 4, 2309}, {72, 145, 12, 2309}, {73, 146, 12, 2309}, {74, 147, 4, 2309}, {77, 151, 1, 2309}, {78, 152, 4, 2309}, {79, 153, 6, 2309}, {80, 154, 4, 2309}, {81, 155, 1, 2309}, {59, 130, 1, 2310}, {62, 135, 4, 2310}, {63, 136, 4, 2310}, {66, 141, 6, 2310}, {67, 142, 12, 2310}, {68, 143, 6, 2310}, {71, 148, 4, 2310}, {72, 149, 12, 2310}, {73, 150, 12, 2310}, {74, 151, 4, 2310}, {77, 156, 1, 2310}, {78, 157, 4, 2310}, {79, 158, 6, 2310}, {80, 159, 4, 2310}, {81, 160, 1, 2310}, {59, 131, 1, 2311}, {62, 136, 4, 2311}, {63, 137, 4, 2311}, {66, 142, 6, 2311}, {67, 143, 12, 2311}, {68, 144, 6, 2311}, {71, 149, 4, 2311}, {72, 150, 12, 2311}, {73, 151, 12, 2311}, {74, 152, 4, 2311}, {77, 157, 1, 2311}, {78, 158, 4, 2311}, {79, 159, 6, 2311}, {80, 160, 4, 2311}, {81, 161, 1, 2311}, {59, 132, 1, 2312}, {62, 137, 4, 2312}, {63, 138, 4, 2312}, {66, 143, 6, 2312}, {67, 144, 12, 2312}, {68, 145, 6, 2312}, {71, 150, 4, 2312}, {72, 151, 12, 2312}, {73, 152, 12, 2312}, {74, 153, 4, 2312}, {77, 158, 1, 2312}, {78, 159, 4, 2312}, {79, 160, 6, 2312}, {80, 161, 4, 2312}, {81, 162, 1, 2312}, {59, 133, 1, 2313}, {62, 138, 4, 2313}, {63, 139, 4, 2313}, {66, 144, 6, 2313}, {67, 145, 12, 2313}, {68, 146, 6, 2313}, {71, 151, 4, 2313}, {72, 152, 12, 2313}, {73, 153, 12, 2313}, {74, 154, 4, 2313}, {77, 159, 1, 2313}, {78, 160, 4, 2313}, {79, 161, 6, 2313}, {80, 162, 4, 2313}, {81, 163, 1, 2313}, {59, 134, 1, 2314}, {62, 139, 4, 2314}, {63, 140, 4, 2314}, {66, 145, 6, 2314}, {67, 146, 12, 2314}, {68, 147, 6, 2314}, {71, 152, 4, 2314}, {72, 153, 12, 2314}, {73, 154, 12, 2314}, {74, 155, 4, 2314}, {77, 160, 1, 2314}, {78, 161, 4, 2314}, {79, 162, 6, 2314}, {80, 163, 4, 2314}, {81, 164, 1, 2314}, {60, 120, 1, 2315}, {63, 121, 4, 2315}, {64, 122, 4, 2315}, {67, 123, 6, 2315}, {68, 124, 12, 2315}, {69, 125, 6, 2315}, {72, 126, 4, 2315}, {73, 127, 12, 2315}, {74, 128, 12, 2315}, {75, 129, 4, 2315}, {78, 130, 1, 2315}, {79, 131, 4, 2315}, {80, 132, 6, 2315}, {81, 133, 4, 2315}, {82, 134, 1, 2315}, {60, 121, 1, 2316}, {63, 123, 4, 2316}, {64, 124, 4, 2316}, {67, 126, 6, 2316}, {68, 127, 12, 2316}, {69, 128, 6, 2316}, {72, 130, 4, 2316}, {73, 131, 12, 2316}, {74, 132, 12, 2316}, {75,

133, 4, 2316}, {78, 135, 1, 2316}, {79, 136, 4, 2316}, {80, 137, 6, 2316}, {81, 138, 4, 2316}, {82, 139, 1, 2316}, {60, 122, 1, 2317}, {63, 124, 4, 2317}, {64, 125, 4, 2317}, {67, 127, 6, 2317}, {68, 128, 12, 2317}, {69, 129, 6, 2317}, {72, 131, 4, 2317}, {73, 132, 12, 2317}, {74, 133, 12, 2317}, {75, 134, 4, 2317}, {78, 136, 1, 2317}, {79, 137, 4, 2317}, {80, 138, 6, 2317}, {81, 139, 4, 2317}, {82, 140, 1, 2317}, {60, 123, 1, 2318}, {63, 126, 4, 2318}, {64, 127, 4, 2318}, {67, 130, 6, 2318}, {68, 131, 12, 2318}, {69, 132, 6, 2318}, {72, 135, 4, 2318}, {73, 136, 12, 2318}, {74, 137, 12, 2318}, {75, 138, 4, 2318}, {78, 141, 1, 2318}, {79, 142, 4, 2318}, {80, 143, 6, 2318}, {81, 144, 4, 2318}, {82, 145, 1, 2318}, {60, 124, 1, 2319}, {63, 127, 4, 2319}, {64, 128, 4, 2319}, {67, 131, 6, 2319}, {68, 132, 12, 2319}, {69, 133, 6, 2319}, {72, 136, 4, 2319}, {73, 137, 12, 2319}, {74, 138, 12, 2319}, {75, 139, 4, 2319}, {78, 142, 1, 2319}, {79, 143, 4, 2319}, {80, 144, 6, 2319}, {81, 145, 4, 2319}, {82, 146, 1, 2319}, {60, 125, 1, 2320}, {63, 128, 4, 2320}, {64, 129, 4, 2320}, {67, 132, 6, 2320}, {68, 133, 12, 2320}, {69, 134, 6, 2320}, {72, 137, 4, 2320}, {73, 138, 12, 2320}, {74, 139, 12, 2320}, {75, 140, 4, 2320}, {78, 143, 1, 2320}, {79, 144, 4, 2320}, {80, 145, 6, 2320}, {81, 146, 4, 2320}, {82, 147, 1, 2320}, {60, 126, 1, 2321}, {63, 130, 4, 2321}, {64, 131, 4, 2321}, {67, 135, 6, 2321}, {68, 136, 12, 2321}, {69, 137, 6, 2321}, {72, 141, 4, 2321}, {73, 142, 12, 2321}, {74, 143, 12, 2321}, {75, 144, 4, 2321}, {78, 148, 1, 2321}, {79, 149, 4, 2321}, {80, 150, 6, 2321}, {81, 151, 4, 2321}, {82, 152, 1, 2321}, {60, 127, 1, 2322}, {63, 131, 4, 2322}, {64, 132, 4, 2322}, {67, 136, 6, 2322}, {68, 137, 12, 2322}, {69, 138, 6, 2322}, {72, 142, 4, 2322}, {73, 143, 12, 2322}, {74, 144, 12, 2322}, {75, 145, 4, 2322}, {78, 149, 1, 2322}, {79, 150, 4, 2322}, {80, 151, 6, 2322}, {81, 152, 4, 2322}, {82, 153, 1, 2322}, {60, 128, 1, 2323}, {63, 132, 4, 2323}, {64, 133, 4, 2323}, {67, 137, 6, 2323}, {68, 138, 12, 2323}, {69, 139, 6, 2323}, {72, 143, 4, 2323}, {73, 144, 12, 2323}, {74, 145, 12, 2323}, {75, 146, 4, 2323}, {78, 150, 1, 2323}, {79, 151, 4, 2323}, {80, 152, 6, 2323}, {81, 153, 4, 2323}, {82, 154, 1, 2323}, {60, 129, 1, 2324}, {63, 133, 4, 2324}, {64, 134, 4, 2324}, {67, 138, 6, 2324}, {68, 139, 12, 2324}, {69, 140, 6, 2324}, {72, 144, 4, 2324}, {73, 145, 12, 2324}, {74, 146, 12, 2324}, {75, 147, 4, 2324}, {78, 151, 1, 2324}, {79, 152, 4, 2324}, {80, 153, 6, 2324}, {81, 154, 4, 2324}, {82, 155, 1, 2324}, {60, 130, 1, 2325}, {63, 135, 4, 2325}, {64, 136, 4, 2325}, {67, 141, 6, 2325}, {68, 142, 12, 2325}, {69, 143, 6, 2325}, {72, 148, 4, 2325}, {73, 149, 12, 2325}, {74, 150, 12, 2325}, {75, 151, 4, 2325}, {78, 156, 1, 2325}, {79, 157, 4, 2325}, {80, 158, 6, 2325}, {81, 159, 4, 2325}, {82, 160, 1, 2325}, {60, 131, 1, 2326}, {63, 136, 4, 2326}, {64, 137, 4, 2326}, {67, 142, 6, 2326}, {68, 143, 12, 2326}, {69, 144, 6, 2326}, {72, 149, 4, 2326}, {73, 150, 12, 2326}, {74, 151, 12, 2326}, {75, 152, 4, 2326}, {78, 157, 1, 2326}, {79, 158, 4, 2326}, {80, 159, 6, 2326}, {81, 160, 4, 2326}, {82, 161, 1, 2326}, {60, 132, 1, 2327}, {63, 137, 4, 2327}, {64, 138, 4, 2327}, {67, 143, 6, 2327}, {68, 144, 12, 2327}, {69, 145, 6, 2327}, {72, 150, 4, 2327}, {73, 151, 12, 2327}, {74, 152, 12, 2327}, {75, 153, 4, 2327}, {78, 158, 1, 2327}, {79, 159, 4, 2327}, {80, 160, 6, 2327}, {81, 161, 4, 2327}, {82, 162, 1, 2327}, {60, 133, 1, 2328}, {63, 138, 4, 2328}, {64, 139, 4, 2328}, {67, 144, 6, 2328}, {68, 145, 12, 2328}, {69, 146, 6, 2328}, {72, 151, 4, 2328}, {73, 152, 12, 2328}, {74, 153, 12, 2328}, {75, 154, 4, 2328}, {78, 159, 1, 2328}, {79, 160, 4, 2328}, {80, 161, 6, 2328}, {81, 162, 4, 2328}, {82, 163, 1, 2328}, {60, 134, 1, 2329}, {63, 139, 4, 2329}, {64, 140, 4, 2329}, {67, 145, 6, 2329}, {68, 146, 12, 2329}, {69, 147, 6, 2329}, {72, 152, 4, 2329}, {73, 153, 12, 2329}, {74, 154, 12, 2329}, {75, 155, 4, 2329}, {78, 160, 1, 2329}, {79, 161, 4, 2329}, {80, 162, 6, 2329}, {81, 163, 4, 2329}, {82, 164, 1, 2329}, {61, 120, 1, 2330}, {64, 121, 4, 2330}, {65, 122, 4, 2330}, {68, 123, 6, 2330}, {69, 124, 12, 2330}, {70, 125, 6, 2330}, {73, 126, 4, 2330}, {74, 127, 12, 2330}, {75, 128, 12, 2330}, {76, 129, 4, 2330}, {79, 130, 1, 2330}, {80, 131, 4, 2330}, {81, 132, 6, 2330}, {82, 133, 4, 2330}, {83, 134, 1, 2330}, {61, 121, 1, 2331}, {64, 123, 4, 2331}, {65, 124, 4, 2331}, {68, 126, 6, 2331}, {69, 127, 12, 2331}, {70, 128, 6, 2331}, {73, 130, 4, 2331}, {74, 131, 12, 2331}, {75, 132, 12, 2331}, {76, 133, 4, 2331}, {79, 135, 1, 2331}, {80, 136, 4, 2331}, {81, 137, 6, 2331}, {82, 138, 4, 2331}, {83, 139, 1, 2331}, {61, 122, 1, 2332}, {64, 124, 4, 2332}, {65, 125, 4, 2332}, {68, 127, 6, 2332}, {69, 128, 12, 2332}, {70, 129, 6, 2332}, {73, 131, 4, 2332}, {74, 132, 12, 2332}, {75, 133, 12, 2332}, {76, 134, 4, 2332}, {79, 136, 1, 2332}, {80, 137, 4, 2332}, {81, 138, 6, 2332}, {82, 139, 4, 2332}, {83, 140, 1, 2332}, {61, 123, 1, 2333}, {64, 126, 4, 2333}, {65, 127, 4, 2333}, {68, 130, 6, 2333}, {69, 131, 12, 2333}, {70, 132, 6, 2333}, {73, 135, 4, 2333}, {74, 136, 12, 2333}, {75, 137, 12, 2333}, {76, 138, 4, 2333}, {79, 141, 1, 2333}, {80, 142, 4, 2333}, {81, 143, 6, 2333}, {82, 144, 4, 2333}, {83, 145, 1, 2333}, {61, 124, 1, 2334}, {64, 127, 4, 2334}, {65, 128, 4, 2334}, {68, 131, 6, 2334}, {69, 132, 12, 2334}, {70, 133, 6, 2334}, {73, 136, 4, 2334}, {74, 137, 12, 2334}, {75, 138, 12, 2334}, {76, 139, 4, 2334}, {79, 142, 1, 2334}, {80, 143, 4, 2334}, {81, 144, 6, 2334}, {82, 145, 4, 2334}, {83, 146, 1, 2334}, {61, 125, 1, 2335}, {64, 128, 4, 2335}, {65, 129, 4, 2335}, {68, 132, 6, 2335}, {69, 133, 12, 2335}, {70, 134, 6, 2335}, {73, 137, 4, 2335}, {74, 138, 12, 2335}, {75, 139, 12, 2335}, {76, 140, 4, 2335}, {79, 143, 1, 2335}, {80, 144, 4, 2335}, {81, 145, 6, 2335}, {82, 146, 4, 2335}, {83, 147, 1, 2335}, {61, 126, 1, 2336}, {64, 130, 4, 2336}, {65, 131, 4, 2336}, {68, 135, 6, 2336}, {69, 136, 12, 2336}, {70, 137, 6, 2336}, {73, 141, 4, 2336}, {74, 142, 12, 2336}, {75, 143, 12, 2336}, {76, 144, 4,

2336}, {79, 148, 1, 2336}, {80, 149, 4, 2336}, {81, 150, 6, 2336}, {82, 151, 4, 2336}, {83, 152, 1, 2336}, {61, 127, 1, 2337}, {64, 131, 4, 2337}, {65, 132, 4, 2337}, {68, 136, 6, 2337}, {69, 137, 12, 2337}, {70, 138, 6, 2337}, {73, 142, 4, 2337}, {74, 143, 12, 2337}, {75, 144, 12, 2337}, {76, 145, 4, 2337}, {79, 149, 1, 2337}, {80, 150, 4, 2337}, {81, 151, 6, 2337}, {82, 152, 4, 2337}, {83, 153, 1, 2337}, {61, 128, 1, 2338}, {64, 132, 4, 2338}, {65, 133, 4, 2338}, {68, 137, 6, 2338}, {69, 138, 12, 2338}, {70, 139, 6, 2338}, {73, 143, 4, 2338}, {74, 144, 12, 2338}, {75, 145, 12, 2338}, {76, 146, 4, 2338}, {79, 150, 1, 2338}, {80, 151, 4, 2338}, {81, 152, 6, 2338}, {82, 153, 4, 2338}, {83, 154, 1, 2338}, {61, 129, 1, 2339}, {64, 133, 4, 2339}, {65, 134, 4, 2339}, {68, 138, 6, 2339}, {69, 139, 12, 2339}, {70, 140, 6, 2339}, {73, 144, 4, 2339}, {74, 145, 12, 2339}, {75, 146, 12, 2339}, {76, 147, 4, 2339}, {79, 151, 1, 2339}, {80, 152, 4, 2339}, {81, 153, 6, 2339}, {82, 154, 4, 2339}, {83, 155, 1, 2339}, {61, 130, 1, 2340}, {64, 135, 4, 2340}, {65, 136, 4, 2340}, {68, 141, 6, 2340}, {69, 142, 12, 2340}, {70, 143, 6, 2340}, {73, 148, 4, 2340}, {74, 149, 12, 2340}, {75, 150, 12, 2340}, {76, 151, 4, 2340}, {79, 156, 1, 2340}, {80, 157, 4, 2340}, {81, 158, 6, 2340}, {82, 159, 4, 2340}, {83, 160, 1, 2340}, {61, 131, 1, 2341}, {64, 136, 4, 2341}, {65, 137, 4, 2341}, {68, 142, 6, 2341}, {69, 143, 12, 2341}, {70, 144, 6, 2341}, {73, 149, 4, 2341}, {74, 150, 12, 2341}, {75, 151, 12, 2341}, {76, 152, 4, 2341}, {79, 157, 1, 2341}, {80, 158, 4, 2341}, {81, 159, 6, 2341}, {82, 160, 4, 2341}, {83, 161, 1, 2341}, {61, 132, 1, 2342}, {64, 137, 4, 2342}, {65, 138, 4, 2342}, {68, 143, 6, 2342}, {69, 144, 12, 2342}, {70, 145, 6, 2342}, {73, 150, 4, 2342}, {74, 151, 12, 2342}, {75, 152, 12, 2342}, {76, 153, 4, 2342}, {79, 158, 1, 2342}, {80, 159, 4, 2342}, {81, 160, 6, 2342}, {82, 161, 4, 2342}, {83, 162, 1, 2342}, {61, 133, 1, 2343}, {64, 138, 4, 2343}, {65, 139, 4, 2343}, {68, 144, 6, 2343}, {69, 145, 12, 2343}, {70, 146, 6, 2343}, {73, 151, 4, 2343}, {74, 152, 12, 2343}, {75, 153, 12, 2343}, {76, 154, 4, 2343}, {79, 159, 1, 2343}, {80, 160, 4, 2343}, {81, 161, 6, 2343}, {82, 162, 4, 2343}, {83, 163, 1, 2343}, {61, 134, 1, 2344}, {64, 139, 4, 2344}, {65, 140, 4, 2344}, {68, 145, 6, 2344}, {69, 146, 12, 2344}, {70, 147, 6, 2344}, {73, 152, 4, 2344}, {74, 153, 12, 2344}, {75, 154, 12, 2344}, {76, 155, 4, 2344}, {79, 160, 1, 2344}, {80, 161, 4, 2344}, {81, 162, 6, 2344}, {82, 163, 4, 2344}, {83, 164, 1, 2344}, {56, 56, 1, 2345}, {57, 57, 5, 2345}, {58, 58, 5, 2345}, {59, 59, 10, 2345}, {60, 60, 20, 2345}, {61, 61, 10, 2345}, {62, 62, 10, 2345}, {63, 63, 30, 2345}, {64, 64, 30, 2345}, {65, 65, 10, 2345}, {66, 66, 5, 2345}, {67, 67, 20, 2345}, {68, 68, 30, 2345}, {69, 69, 20, 2345}, {70, 70, 5, 2345}, {71, 71, 1, 2345}, {72, 72, 5, 2345}, {73, 73, 10, 2345}, {74, 74, 10, 2345}, {75, 75, 5, 2345}, {76, 76, 1, 2345}, {56, 57, 1, 2346}, {57, 59, 5, 2346}, {58, 60, 5, 2346}, {59, 62, 10, 2346}, {60, 63, 20, 2346}, {61, 64, 10, 2346}, {62, 66, 10, 2346}, {63, 67, 30, 2346}, {64, 68, 30, 2346}, {65, 69, 10, 2346}, {66, 71, 5, 2346}, {67, 72, 20, 2346}, {68, 73, 30, 2346}, {69, 74, 20, 2346}, {70, 75, 5, 2346}, {71, 77, 1, 2346}, {72, 78, 5, 2346}, {73, 79, 10, 2346}, {74, 80, 10, 2346}, {75, 81, 5, 2346}, {76, 82, 1, 2346}, {56, 58, 1, 2347}, {57, 60, 5, 2347}, {58, 61, 5, 2347}, {59, 63, 10, 2347}, {60, 64, 20, 2347}, {61, 65, 10, 2347}, {62, 67, 10, 2347}, {63, 68, 30, 2347}, {64, 69, 30, 2347}, {65, 70, 10, 2347}, {66, 72, 5, 2347}, {67, 73, 20, 2347}, {68, 74, 30, 2347}, {69, 75, 20, 2347}, {70, 76, 5, 2347}, {71, 78, 1, 2347}, {72, 79, 5, 2347}, {73, 80, 10, 2347}, {74, 81, 10, 2347}, {75, 82, 5, 2347}, {76, 83, 1, 2347}, {57, 57, 1, 2348}, {59, 59, 5, 2348}, {60, 60, 5, 2348}, {62, 62, 10, 2348}, {63, 63, 20, 2348}, {64, 64, 10, 2348}, {66, 66, 10, 2348}, {67, 67, 30, 2348}, {68, 68, 30, 2348}, {69, 69, 10, 2348}, {71, 71, 5, 2348}, {72, 72, 20, 2348}, {73, 73, 30, 2348}, {74, 74, 20, 2348}, {75, 75, 5, 2348}, {77, 77, 1, 2348}, {78, 78, 5, 2348}, {79, 79, 10, 2348}, {80, 80, 10, 2348}, {81, 81, 5, 2348}, {82, 82, 1, 2348}, {57, 58, 1, 2349}, {59, 60, 5, 2349}, {60, 61, 5, 2349}, {62, 63, 10, 2349}, {63, 64, 20, 2349}, {64, 65, 10, 2349}, {66, 67, 10, 2349}, {67, 68, 30, 2349}, {68, 69, 30, 2349}, {69, 70, 10, 2349}, {71, 72, 5, 2349}, {72, 73, 20, 2349}, {73, 74, 30, 2349}, {74, 75, 20, 2349}, {75, 76, 5, 2349}, {77, 78, 1, 2349}, {78, 79, 5, 2349}, {79, 80, 10, 2349}, {80, 81, 10, 2349}, {81, 82, 5, 2349}, {82, 83, 1, 2349}, {58, 58, 1, 2350}, {60, 60, 5, 2350}, {61, 61, 5, 2350}, {63, 63, 10, 2350}, {64, 64, 20, 2350}, {65, 65, 10, 2350}, {67, 67, 10, 2350}, {68, 68, 30, 2350}, {69, 69, 30, 2350}, {70, 70, 10, 2350}, {72, 72, 5, 2350}, {73, 73, 20, 2350}, {74, 74, 30, 2350}, {75, 75, 20, 2350}, {76, 76, 5, 2350}, {78, 78, 1, 2350}, {79, 79, 5, 2350}, {80, 80, 10, 2350}, {81, 81, 10, 2350}, {82, 82, 5, 2350}, {83, 83, 1, 2350}, {56, 84, 1, 2351}, {57, 85, 5, 2351}, {58, 86, 5, 2351}, {59, 87, 10, 2351}, {60, 88, 20, 2351}, {61, 89, 10, 2351}, {62, 90, 10, 2351}, {63, 91, 30, 2351}, {64, 92, 30, 2351}, {65, 93, 10, 2351}, {66, 94, 5, 2351}, {67, 95, 20, 2351}, {68, 96, 30, 2351}, {69, 97, 20, 2351}, {70, 98, 5, 2351}, {71, 99, 1, 2351}, {72, 100, 5, 2351}, {73, 101, 10, 2351}, {74, 102, 10, 2351}, {75, 103, 5, 2351}, {76, 104, 1, 2351}, {56, 85, 1, 2352}, {57, 87, 5, 2352}, {58, 88, 5, 2352}, {59, 90, 10, 2352}, {60, 91, 20, 2352}, {61, 92, 10, 2352}, {62, 94, 10, 2352}, {63, 95, 30, 2352}, {64, 96, 30, 2352}, {65, 97, 10, 2352}, {66, 99, 5, 2352}, {67, 100, 20, 2352}, {68, 101, 30, 2352}, {69, 102, 20, 2352}, {70, 103, 5, 2352}, {71, 105, 1, 2352}, {72, 106, 5, 2352}, {73, 107, 10, 2352}, {74, 108, 10, 2352}, {75, 109, 5, 2352}, {76, 110, 1, 2352}, {56, 86, 1, 2353}, {57, 88, 5, 2353}, {58, 89, 5, 2353}, {59, 91, 10, 2353}, {60, 92, 20, 2353}, {61, 93, 10, 2353}, {62, 95, 10, 2353}, {63, 96, 30, 2353}, {64, 97, 30, 2353}, {65, 98, 10, 2353}, {66, 100, 5, 2353}, {67, 101, 20, 2353}, {68, 102, 30, 2353},

{69, 103, 20, 2353}, {70, 104, 5, 2353}, {71, 106, 1, 2353}, {72, 107, 5, 2353}, {73, 108, 10, 2353}, {74, 109, 10, 2353}, {75, 110, 5, 2353}, {76, 111, 1, 2353}, {56, 87, 1, 2354}, {57, 90, 5, 2354}, {58, 91, 5, 2354}, {59, 94, 10, 2354}, {60, 95, 20, 2354}, {61, 96, 10, 2354}, {62, 99, 10, 2354}, {63, 100, 30, 2354}, {64, 101, 30, 2354}, {65, 102, 10, 2354}, {66, 105, 5, 2354}, {67, 106, 20, 2354}, {68, 107, 30, 2354}, {69, 108, 20, 2354}, {70, 109, 5, 2354}, {71, 112, 1, 2354}, {72, 113, 5, 2354}, {73, 114, 10, 2354}, {74, 115, 10, 2354}, {75, 116, 5, 2354}, {76, 117, 1, 2354}, {56, 88, 1, 2355}, {57, 91, 5, 2355}, {58, 92, 5, 2355}, {59, 95, 10, 2355}, {60, 96, 20, 2355}, {61, 97, 10, 2355}, {62, 100, 10, 2355}, {63, 101, 30, 2355}, {64, 102, 30, 2355}, {65, 103, 10, 2355}, {66, 106, 5, 2355}, {67, 107, 20, 2355}, {68, 108, 30, 2355}, {69, 109, 20, 2355}, {70, 110, 5, 2355}, {71, 113, 1, 2355}, {72, 114, 5, 2355}, {73, 115, 10, 2355}, {74, 116, 10, 2355}, {75, 117, 5, 2355}, {76, 118, 1, 2355}, {56, 89, 1, 2356}, {57, 92, 5, 2356}, {58, 93, 5, 2356}, {59, 96, 10, 2356}, {60, 97, 20, 2356}, {61, 98, 10, 2356}, {62, 101, 10, 2356}, {63, 102, 30, 2356}, {64, 103, 30, 2356}, {65, 104, 10, 2356}, {66, 107, 5, 2356}, {67, 108, 20, 2356}, {68, 109, 30, 2356}, {69, 110, 20, 2356}, {70, 111, 5, 2356}, {71, 114, 1, 2356}, {72, 115, 5, 2356}, {73, 116, 10, 2356}, {74, 117, 10, 2356}, {75, 118, 5, 2356}, {76, 119, 1, 2356}, {57, 84, 1, 2357}, {59, 85, 5, 2357}, {60, 86, 5, 2357}, {62, 87, 10, 2357}, {63, 88, 20, 2357}, {64, 89, 10, 2357}, {66, 90, 10, 2357}, {67, 91, 30, 2357}, {68, 92, 30, 2357}, {69, 93, 10, 2357}, {71, 94, 5, 2357}, {72, 95, 20, 2357}, {73, 96, 30, 2357}, {74, 97, 20, 2357}, {75, 98, 5, 2357}, {77, 99, 1, 2357}, {78, 100, 5, 2357}, {79, 101, 10, 2357}, {80, 102, 10, 2357}, {81, 103, 5, 2357}, {82, 104, 1, 2357}, {57, 85, 1, 2358}, {59, 87, 5, 2358}, {60, 88, 5, 2358}, {62, 90, 10, 2358}, {63, 91, 20, 2358}, {64, 92, 10, 2358}, {66, 94, 10, 2358}, {67, 95, 30, 2358}, {68, 96, 30, 2358}, {69, 97, 10, 2358}, {71, 99, 5, 2358}, {72, 100, 20, 2358}, {73, 101, 30, 2358}, {74, 102, 20, 2358}, {75, 103, 5, 2358}, {77, 105, 1, 2358}, {78, 106, 5, 2358}, {79, 107, 10, 2358}, {80, 108, 10, 2358}, {81, 109, 5, 2358}, {82, 110, 1, 2358}, {57, 86, 1, 2359}, {59, 88, 5, 2359}, {60, 89, 5, 2359}, {62, 91, 10, 2359}, {63, 92, 20, 2359}, {64, 93, 10, 2359}, {66, 95, 10, 2359}, {67, 96, 30, 2359}, {68, 97, 30, 2359}, {69, 98, 10, 2359}, {71, 100, 5, 2359}, {72, 101, 20, 2359}, {73, 102, 30, 2359}, {74, 103, 20, 2359}, {75, 104, 5, 2359}, {77, 106, 1, 2359}, {78, 107, 5, 2359}, {79, 108, 10, 2359}, {80, 109, 10, 2359}, {81, 110, 5, 2359}, {82, 111, 1, 2359}, {57, 87, 1, 2360}, {59, 90, 5, 2360}, {60, 91, 5, 2360}, {62, 94, 10, 2360}, {63, 95, 20, 2360}, {64, 96, 10, 2360}, {66, 99, 10, 2360}, {67, 100, 30, 2360}, {68, 101, 30, 2360}, {69, 102, 10, 2360}, {71, 105, 5, 2360}, {72, 106, 20, 2360}, {73, 107, 30, 2360}, {74, 108, 20, 2360}, {75, 109, 5, 2360}, {77, 112, 1, 2360}, {78, 113, 5, 2360}, {79, 114, 10, 2360}, {80, 115, 10, 2360}, {81, 116, 5, 2360}, {82, 117, 1, 2360}, {57, 88, 1, 2361}, {59, 91, 5, 2361}, {60, 92, 5, 2361}, {62, 95, 10, 2361}, {63, 96, 20, 2361}, {64, 97, 10, 2361}, {66, 100, 10, 2361}, {67, 101, 30, 2361}, {68, 102, 30, 2361}, {69, 103, 10, 2361}, {71, 106, 5, 2361}, {72, 107, 20, 2361}, {73, 108, 30, 2361}, {74, 109, 20, 2361}, {75, 110, 5, 2361}, {77, 113, 1, 2361}, {78, 114, 5, 2361}, {79, 115, 10, 2361}, {80, 116, 10, 2361}, {81, 117, 5, 2361}, {82, 118, 1, 2361}, {57, 89, 1, 2362}, {59, 92, 5, 2362}, {60, 93, 5, 2362}, {62, 96, 10, 2362}, {63, 97, 20, 2362}, {64, 98, 10, 2362}, {66, 101, 10, 2362}, {67, 102, 30, 2362}, {68, 103, 30, 2362}, {69, 104, 10, 2362}, {71, 107, 5, 2362}, {72, 108, 20, 2362}, {73, 109, 30, 2362}, {74, 110, 20, 2362}, {75, 111, 5, 2362}, {77, 114, 1, 2362}, {78, 115, 5, 2362}, {79, 116, 10, 2362}, {80, 117, 10, 2362}, {81, 118, 5, 2362}, {82, 119, 1, 2362}, {58, 84, 1, 2363}, {60, 85, 5, 2363}, {61, 86, 5, 2363}, {63, 87, 10, 2363}, {64, 88, 20, 2363}, {65, 89, 10, 2363}, {67, 90, 10, 2363}, {68, 91, 30, 2363}, {69, 92, 30, 2363}, {70, 93, 10, 2363}, {72, 94, 5, 2363}, {73, 95, 20, 2363}, {74, 96, 30, 2363}, {75, 97, 20, 2363}, {76, 98, 5, 2363}, {78, 99, 1, 2363}, {79, 100, 5, 2363}, {80, 101, 10, 2363}, {81, 102, 10, 2363}, {82, 103, 5, 2363}, {83, 104, 1, 2363}, {58, 85, 1, 2364}, {60, 87, 5, 2364}, {61, 88, 5, 2364}, {63, 90, 10, 2364}, {64, 91, 20, 2364}, {65, 92, 10, 2364}, {67, 94, 10, 2364}, {68, 95, 30, 2364}, {69, 96, 30, 2364}, {70, 97, 10, 2364}, {72, 99, 5, 2364}, {73, 100, 20, 2364}, {74, 101, 30, 2364}, {75, 102, 20, 2364}, {76, 103, 5, 2364}, {78, 105, 1, 2364}, {79, 106, 5, 2364}, {80, 107, 10, 2364}, {81, 108, 10, 2364}, {82, 109, 5, 2364}, {83, 110, 1, 2364}, {58, 86, 1, 2365}, {60, 88, 5, 2365}, {61, 89, 5, 2365}, {63, 91, 10, 2365}, {64, 92, 20, 2365}, {65, 93, 10, 2365}, {67, 95, 10, 2365}, {68, 96, 30, 2365}, {69, 97, 30, 2365}, {70, 98, 10, 2365}, {72, 100, 5, 2365}, {73, 101, 20, 2365}, {74, 102, 30, 2365}, {75, 103, 20, 2365}, {76, 104, 5, 2365}, {78, 106, 1, 2365}, {79, 107, 5, 2365}, {80, 108, 10, 2365}, {81, 109, 10, 2365}, {82, 110, 5, 2365}, {83, 111, 1, 2365}, {58, 87, 1, 2366}, {60, 90, 5, 2366}, {61, 91, 5, 2366}, {63, 94, 10, 2366}, {64, 95, 20, 2366}, {65, 96, 10, 2366}, {67, 99, 10, 2366}, {68, 100, 30, 2366}, {69, 101, 30, 2366}, {70, 102, 10, 2366}, {72, 105, 5, 2366}, {73, 106, 20, 2366}, {74, 107, 30, 2366}, {75, 108, 20, 2366}, {76, 109, 5, 2366}, {78, 112, 1, 2366}, {79, 113, 5, 2366}, {80, 114, 10, 2366}, {81, 115, 10, 2366}, {82, 116, 5, 2366}, {83, 117, 1, 2366}, {58, 88, 1, 2367}, {60, 91, 5, 2367}, {61, 92, 5, 2367}, {63, 95, 10, 2367}, {64, 96, 20, 2367}, {65, 97, 10, 2367}, {67, 100, 10, 2367}, {68, 101, 30, 2367}, {69, 102, 30, 2367}, {70, 103, 10, 2367}, {72, 106, 5, 2367}, {73, 107, 20, 2367}, {74, 108, 30, 2367}, {75, 109, 20, 2367}, {76, 110, 5, 2367}, {78, 113, 1, 2367}, {79, 114, 5, 2367}, {80, 115, 10, 2367}, {81, 116, 10, 2367}, {82, 117, 5, 2367}, {83, 118, 1, 2367}, {58, 89, 1, 2368}, {60, 92, 5, 2368}, {61, 93, 5, 2368}, {63,

96, 10, 2368}, {64, 97, 20, 2368}, {65, 98, 10, 2368}, {67, 101, 10, 2368}, {68, 102, 30, 2368}, {69, 103, 30, 2368}, {70, 104, 10, 2368}, {72, 107, 5, 2368}, {73, 108, 20, 2368}, {74, 109, 30, 2368}, {75, 110, 20, 2368}, {76, 111, 5, 2368}, {78, 114, 1, 2368}, {79, 115, 5, 2368}, {80, 116, 10, 2368}, {81, 117, 10, 2368}, {82, 118, 5, 2368}, {83, 119, 1, 2368}, {56, 120, 1, 2369}, {57, 121, 5, 2369}, {58, 122, 5, 2369}, {59, 123, 10, 2369}, {60, 124, 20, 2369}, {61, 125, 10, 2369}, {62, 126, 10, 2369}, {63, 127, 30, 2369}, {64, 128, 30, 2369}, {65, 129, 10, 2369}, {66, 130, 5, 2369}, {67, 131, 20, 2369}, {68, 132, 30, 2369}, {69, 133, 20, 2369}, {70, 134, 5, 2369}, {71, 135, 1, 2369}, {72, 136, 5, 2369}, {73, 137, 10, 2369}, {74, 138, 10, 2369}, {75, 139, 5, 2369}, {76, 140, 1, 2369}, {56, 121, 1, 2370}, {57, 123, 5, 2370}, {58, 124, 5, 2370}, {59, 126, 10, 2370}, {60, 127, 20, 2370}, {61, 128, 10, 2370}, {62, 130, 10, 2370}, {63, 131, 30, 2370}, {64, 132, 30, 2370}, {65, 133, 10, 2370}, {66, 135, 5, 2370}, {67, 136, 20, 2370}, {68, 137, 30, 2370}, {69, 138, 20, 2370}, {70, 139, 5, 2370}, {71, 141, 1, 2370}, {72, 142, 5, 2370}, {73, 143, 10, 2370}, {74, 144, 10, 2370}, {75, 145, 5, 2370}, {76, 146, 1, 2370}, {56, 122, 1, 2371}, {57, 124, 5, 2371}, {58, 125, 5, 2371}, {59, 127, 10, 2371}, {60, 128, 20, 2371}, {61, 129, 10, 2371}, {62, 131, 10, 2371}, {63, 132, 30, 2371}, {64, 133, 30, 2371}, {65, 134, 10, 2371}, {66, 136, 5, 2371}, {67, 137, 20, 2371}, {68, 138, 30, 2371}, {69, 139, 20, 2371}, {70, 140, 5, 2371}, {71, 142, 1, 2371}, {72, 143, 5, 2371}, {73, 144, 10, 2371}, {74, 145, 10, 2371}, {75, 146, 5, 2371}, {76, 147, 1, 2371}, {56, 123, 1, 2372}, {57, 126, 5, 2372}, {58, 127, 5, 2372}, {59, 130, 10, 2372}, {60, 131, 20, 2372}, {61, 132, 10, 2372}, {62, 135, 10, 2372}, {63, 136, 30, 2372}, {64, 137, 30, 2372}, {65, 138, 10, 2372}, {66, 141, 5, 2372}, {67, 142, 20, 2372}, {68, 143, 30, 2372}, {69, 144, 20, 2372}, {70, 145, 5, 2372}, {71, 148, 1, 2372}, {72, 149, 5, 2372}, {73, 150, 10, 2372}, {74, 151, 10, 2372}, {75, 152, 5, 2372}, {76, 153, 1, 2372}, {56, 124, 1, 2373}, {57, 127, 5, 2373}, {58, 128, 5, 2373}, {59, 131, 10, 2373}, {60, 132, 20, 2373}, {61, 133, 10, 2373}, {62, 136, 10, 2373}, {63, 137, 30, 2373}, {64, 138, 30, 2373}, {65, 139, 10, 2373}, {66, 142, 5, 2373}, {67, 143, 20, 2373}, {68, 144, 30, 2373}, {69, 145, 20, 2373}, {70, 146, 5, 2373}, {71, 149, 1, 2373}, {72, 150, 5, 2373}, {73, 151, 10, 2373}, {74, 152, 10, 2373}, {75, 153, 5, 2373}, {76, 154, 1, 2373}, {56, 125, 1, 2374}, {57, 128, 5, 2374}, {58, 129, 5, 2374}, {59, 132, 10, 2374}, {60, 133, 20, 2374}, {61, 134, 10, 2374}, {62, 137, 10, 2374}, {63, 138, 30, 2374}, {64, 139, 30, 2374}, {65, 140, 10, 2374}, {66, 143, 5, 2374}, {67, 144, 20, 2374}, {68, 145, 30, 2374}, {69, 146, 20, 2374}, {70, 147, 5, 2374}, {71, 150, 1, 2374}, {72, 151, 5, 2374}, {73, 152, 10, 2374}, {74, 153, 10, 2374}, {75, 154, 5, 2374}, {76, 155, 1, 2374}, {56, 126, 1, 2375}, {57, 130, 5, 2375}, {58, 131, 5, 2375}, {59, 135, 10, 2375}, {60, 136, 20, 2375}, {61, 137, 10, 2375}, {62, 141, 10, 2375}, {63, 142, 30, 2375}, {64, 143, 30, 2375}, {65, 144, 10, 2375}, {66, 148, 5, 2375}, {67, 149, 20, 2375}, {68, 150, 30, 2375}, {69, 151, 20, 2375}, {70, 152, 5, 2375}, {71, 156, 1, 2375}, {72, 157, 5, 2375}, {73, 158, 10, 2375}, {74, 159, 10, 2375}, {75, 160, 5, 2375}, {76, 161, 1, 2375}, {56, 127, 1, 2376}, {57, 131, 5, 2376}, {58, 132, 5, 2376}, {59, 136, 10, 2376}, {60, 137, 20, 2376}, {61, 138, 10, 2376}, {62, 142, 10, 2376}, {63, 143, 30, 2376}, {64, 144, 30, 2376}, {65, 145, 10, 2376}, {66, 149, 5, 2376}, {67, 150, 20, 2376}, {68, 151, 30, 2376}, {69, 152, 20, 2376}, {70, 153, 5, 2376}, {71, 157, 1, 2376}, {72, 158, 5, 2376}, {73, 159, 10, 2376}, {74, 160, 10, 2376}, {75, 161, 5, 2376}, {76, 162, 1, 2376}, {56, 128, 1, 2377}, {57, 132, 5, 2377}, {58, 133, 5, 2377}, {59, 137, 10, 2377}, {60, 138, 20, 2377}, {61, 139, 10, 2377}, {62, 143, 10, 2377}, {63, 144, 30, 2377}, {64, 145, 30, 2377}, {65, 146, 10, 2377}, {66, 150, 5, 2377}, {67, 151, 20, 2377}, {68, 152, 30, 2377}, {69, 153, 20, 2377}, {70, 154, 5, 2377}, {71, 158, 1, 2377}, {72, 159, 5, 2377}, {73, 160, 10, 2377}, {74, 161, 10, 2377}, {75, 162, 5, 2377}, {76, 163, 1, 2377}, {56, 129, 1, 2378}, {57, 133, 5, 2378}, {58, 134, 5, 2378}, {59, 138, 10, 2378}, {60, 139, 20, 2378}, {61, 140, 10, 2378}, {62, 144, 10, 2378}, {63, 145, 30, 2378}, {64, 146, 30, 2378}, {65, 147, 10, 2378}, {66, 151, 5, 2378}, {67, 152, 20, 2378}, {68, 153, 30, 2378}, {69, 154, 20, 2378}, {70, 155, 5, 2378}, {71, 159, 1, 2378}, {72, 160, 5, 2378}, {73, 161, 10, 2378}, {74, 162, 10, 2378}, {75, 163, 5, 2378}, {76, 164, 1, 2378}, {57, 120, 1, 2379}, {59, 121, 5, 2379}, {60, 122, 5, 2379}, {62, 123, 10, 2379}, {63, 124, 20, 2379}, {64, 125, 10, 2379}, {66, 126, 10, 2379}, {67, 127, 30, 2379}, {68, 128, 30, 2379}, {69, 129, 10, 2379}, {71, 130, 5, 2379}, {72, 131, 20, 2379}, {73, 132, 30, 2379}, {74, 133, 20, 2379}, {75, 134, 5, 2379}, {77, 135, 1, 2379}, {78, 136, 5, 2379}, {79, 137, 10, 2379}, {80, 138, 10, 2379}, {81, 139, 5, 2379}, {82, 140, 1, 2379}, {57, 121, 1, 2380}, {59, 123, 5, 2380}, {60, 124, 5, 2380}, {62, 126, 10, 2380}, {63, 127, 20, 2380}, {64, 128, 10, 2380}, {66, 130, 10, 2380}, {67, 131, 30, 2380}, {68, 132, 30, 2380}, {69, 133, 10, 2380}, {71, 135, 5, 2380}, {72, 136, 20, 2380}, {73, 137, 30, 2380}, {74, 138, 20, 2380}, {75, 139, 5, 2380}, {77, 141, 1, 2380}, {78, 142, 5, 2380}, {79, 143, 10, 2380}, {80, 144, 10, 2380}, {81, 145, 5, 2380}, {82, 146, 1, 2380}, {57, 122, 1, 2381}, {59, 124, 5, 2381}, {60, 125, 5, 2381}, {62, 127, 10, 2381}, {63, 128, 20, 2381}, {64, 129, 10, 2381}, {66, 131, 10, 2381}, {67, 132, 30, 2381}, {68, 133, 30, 2381}, {69, 134, 10, 2381}, {71, 136, 5, 2381}, {72, 137, 20, 2381}, {73, 138, 30, 2381}, {74, 139, 20, 2381}, {75, 140, 5, 2381}, {77, 142, 1, 2381}, {78, 143, 5, 2381}, {79, 144, 10, 2381}, {80, 145, 10, 2381}, {81, 146, 5, 2381}, {82, 147, 1, 2381}, {57, 123, 1, 2382}, {59, 126, 5, 2382}, {60, 127, 5, 2382}, {62, 130, 10, 2382}, {63, 131, 20, 2382}, {64, 132, 10,

2382}, {66, 135, 10, 2382}, {67, 136, 30, 2382}, {68, 137, 30, 2382}, {69, 138, 10, 2382}, {71, 141, 5, 2382}, {72, 142, 20, 2382}, {73, 143, 30, 2382}, {74, 144, 20, 2382}, {75, 145, 5, 2382}, {77, 148, 1, 2382}, {78, 149, 5, 2382}, {79, 150, 10, 2382}, {80, 151, 10, 2382}, {81, 152, 5, 2382}, {82, 153, 1, 2382}, {57, 124, 1, 2383}, {59, 127, 5, 2383}, {60, 128, 5, 2383}, {62, 131, 10, 2383}, {63, 132, 20, 2383}, {64, 133, 10, 2383}, {66, 136, 10, 2383}, {67, 137, 30, 2383}, {68, 138, 30, 2383}, {69, 139, 10, 2383}, {71, 142, 5, 2383}, {72, 143, 20, 2383}, {73, 144, 30, 2383}, {74, 145, 20, 2383}, {75, 146, 5, 2383}, {77, 149, 1, 2383}, {78, 150, 5, 2383}, {79, 151, 10, 2383}, {80, 152, 10, 2383}, {81, 153, 5, 2383}, {82, 154, 1, 2383}, {57, 125, 1, 2384}, {59, 128, 5, 2384}, {60, 129, 5, 2384}, {62, 132, 10, 2384}, {63, 133, 20, 2384}, {64, 134, 10, 2384}, {66, 137, 10, 2384}, {67, 138, 30, 2384}, {68, 139, 30, 2384}, {69, 140, 10, 2384}, {71, 143, 5, 2384}, {72, 144, 20, 2384}, {73, 145, 30, 2384}, {74, 146, 20, 2384}, {75, 147, 5, 2384}, {77, 150, 1, 2384}, {78, 151, 5, 2384}, {79, 152, 10, 2384}, {80, 153, 10, 2384}, {81, 154, 5, 2384}, {82, 155, 1, 2384}, {57, 126, 1, 2385}, {59, 130, 5, 2385}, {60, 131, 5, 2385}, {62, 135, 10, 2385}, {63, 136, 20, 2385}, {64, 137, 10, 2385}, {66, 141, 10, 2385}, {67, 142, 30, 2385}, {68, 143, 30, 2385}, {69, 144, 10, 2385}, {71, 148, 5, 2385}, {72, 149, 20, 2385}, {73, 150, 30, 2385}, {74, 151, 20, 2385}, {75, 152, 5, 2385}, {77, 156, 1, 2385}, {78, 157, 5, 2385}, {79, 158, 10, 2385}, {80, 159, 10, 2385}, {81, 160, 5, 2385}, {82, 161, 1, 2385}, {57, 127, 1, 2386}, {59, 131, 5, 2386}, {60, 132, 5, 2386}, {62, 136, 10, 2386}, {63, 137, 20, 2386}, {64, 138, 10, 2386}, {66, 142, 10, 2386}, {67, 143, 30, 2386}, {68, 144, 30, 2386}, {69, 145, 10, 2386}, {71, 149, 5, 2386}, {72, 150, 20, 2386}, {73, 151, 30, 2386}, {74, 152, 20, 2386}, {75, 153, 5, 2386}, {77, 157, 1, 2386}, {78, 158, 5, 2386}, {79, 159, 10, 2386}, {80, 160, 10, 2386}, {81, 161, 5, 2386}, {82, 162, 1, 2386}, {57, 128, 1, 2387}, {59, 132, 5, 2387}, {60, 133, 5, 2387}, {62, 137, 10, 2387}, {63, 138, 20, 2387}, {64, 139, 10, 2387}, {66, 143, 10, 2387}, {67, 144, 30, 2387}, {68, 145, 30, 2387}, {69, 146, 10, 2387}, {71, 150, 5, 2387}, {72, 151, 20, 2387}, {73, 152, 30, 2387}, {74, 153, 20, 2387}, {75, 154, 5, 2387}, {77, 158, 1, 2387}, {78, 159, 5, 2387}, {79, 160, 10, 2387}, {80, 161, 10, 2387}, {81, 162, 5, 2387}, {82, 163, 1, 2387}, {57, 129, 1, 2388}, {59, 133, 5, 2388}, {60, 134, 5, 2388}, {62, 138, 10, 2388}, {63, 139, 20, 2388}, {64, 140, 10, 2388}, {66, 144, 10, 2388}, {67, 145, 30, 2388}, {68, 146, 30, 2388}, {69, 147, 10, 2388}, {71, 151, 5, 2388}, {72, 152, 20, 2388}, {73, 153, 30, 2388}, {74, 154, 20, 2388}, {75, 155, 5, 2388}, {77, 159, 1, 2388}, {78, 160, 5, 2388}, {79, 161, 10, 2388}, {80, 162, 10, 2388}, {81, 163, 5, 2388}, {82, 164, 1, 2388}, {58, 120, 1, 2389}, {60, 121, 5, 2389}, {61, 122, 5, 2389}, {63, 123, 10, 2389}, {64, 124, 20, 2389}, {65, 125, 10, 2389}, {67, 126, 10, 2389}, {68, 127, 30, 2389}, {69, 128, 30, 2389}, {70, 129, 10, 2389}, {72, 130, 5, 2389}, {73, 131, 20, 2389}, {74, 132, 30, 2389}, {75, 133, 20, 2389}, {76, 134, 5, 2389}, {78, 135, 1, 2389}, {79, 136, 5, 2389}, {80, 137, 10, 2389}, {81, 138, 10, 2389}, {82, 139, 5, 2389}, {83, 140, 1, 2389}, {58, 121, 1, 2390}, {60, 123, 5, 2390}, {61, 124, 5, 2390}, {63, 126, 10, 2390}, {64, 127, 20, 2390}, {65, 128, 10, 2390}, {67, 130, 10, 2390}, {68, 131, 30, 2390}, {69, 132, 30, 2390}, {70, 133, 10, 2390}, {72, 135, 5, 2390}, {73, 136, 20, 2390}, {74, 137, 30, 2390}, {75, 138, 20, 2390}, {76, 139, 5, 2390}, {78, 141, 1, 2390}, {79, 142, 5, 2390}, {80, 143, 10, 2390}, {81, 144, 10, 2390}, {82, 145, 5, 2390}, {83, 146, 1, 2390}, {58, 122, 1, 2391}, {60, 124, 5, 2391}, {61, 125, 5, 2391}, {63, 127, 10, 2391}, {64, 128, 20, 2391}, {65, 129, 10, 2391}, {67, 131, 10, 2391}, {68, 132, 30, 2391}, {69, 133, 30, 2391}, {70, 134, 10, 2391}, {72, 136, 5, 2391}, {73, 137, 20, 2391}, {74, 138, 30, 2391}, {75, 139, 20, 2391}, {76, 140, 5, 2391}, {78, 142, 1, 2391}, {79, 143, 5, 2391}, {80, 144, 10, 2391}, {81, 145, 10, 2391}, {82, 146, 5, 2391}, {83, 147, 1, 2391}, {58, 123, 1, 2392}, {60, 126, 5, 2392}, {61, 127, 5, 2392}, {63, 130, 10, 2392}, {64, 131, 20, 2392}, {65, 132, 10, 2392}, {67, 135, 10, 2392}, {68, 136, 30, 2392}, {69, 137, 30, 2392}, {70, 138, 10, 2392}, {72, 141, 5, 2392}, {73, 142, 20, 2392}, {74, 143, 30, 2392}, {75, 144, 20, 2392}, {76, 145, 5, 2392}, {78, 148, 1, 2392}, {79, 149, 5, 2392}, {80, 150, 10, 2392}, {81, 151, 10, 2392}, {82, 152, 5, 2392}, {83, 153, 1, 2392}, {58, 124, 1, 2393}, {60, 127, 5, 2393}, {61, 128, 5, 2393}, {63, 131, 10, 2393}, {64, 132, 20, 2393}, {65, 133, 10, 2393}, {67, 136, 10, 2393}, {68, 137, 30, 2393}, {69, 138, 30, 2393}, {70, 139, 10, 2393}, {72, 142, 5, 2393}, {73, 143, 20, 2393}, {74, 144, 30, 2393}, {75, 145, 20, 2393}, {76, 146, 5, 2393}, {78, 149, 1, 2393}, {79, 150, 5, 2393}, {80, 151, 10, 2393}, {81, 152, 10, 2393}, {82, 153, 5, 2393}, {83, 154, 1, 2393}, {58, 125, 1, 2394}, {60, 128, 5, 2394}, {61, 129, 5, 2394}, {63, 132, 10, 2394}, {64, 133, 20, 2394}, {65, 134, 10, 2394}, {67, 137, 10, 2394}, {68, 138, 30, 2394}, {69, 139, 30, 2394}, {70, 140, 10, 2394}, {72, 143, 5, 2394}, {73, 144, 20, 2394}, {74, 145, 30, 2394}, {75, 146, 20, 2394}, {76, 147, 5, 2394}, {78, 150, 1, 2394}, {79, 151, 5, 2394}, {80, 152, 10, 2394}, {81, 153, 10, 2394}, {82, 154, 5, 2394}, {83, 155, 1, 2394}, {58, 126, 1, 2395}, {60, 130, 5, 2395}, {61, 131, 5, 2395}, {63, 135, 10, 2395}, {64, 136, 20, 2395}, {65, 137, 10, 2395}, {67, 141, 10, 2395}, {68, 142, 30, 2395}, {69, 143, 30, 2395}, {70, 144, 10, 2395}, {72, 148, 5, 2395}, {73, 149, 20, 2395}, {74, 150, 30, 2395}, {75, 151, 20, 2395}, {76, 152, 5, 2395}, {78, 156, 1, 2395}, {79, 157, 5, 2395}, {80, 158, 10, 2395}, {81, 159, 10, 2395}, {82, 160, 5, 2395}, {83, 161, 1, 2395}, {58, 127, 1, 2396}, {60, 131, 5, 2396}, {61, 132, 5, 2396}, {63, 136, 10, 2396}, {64, 137, 20, 2396}, {65, 138, 10, 2396}, {67, 142, 10, 2396}, {68, 143, 30, 2396}, {69,

144, 30, 2396}, {70, 145, 10, 2396}, {72, 149, 5, 2396}, {73, 150, 20, 2396}, {74, 151, 30, 2396}, {75, 152, 20, 2396}, {76, 153, 5, 2396}, {78, 157, 1, 2396}, {79, 158, 5, 2396}, {80, 159, 10, 2396}, {81, 160, 10, 2396}, {82, 161, 5, 2396}, {83, 162, 1, 2396}, {58, 128, 1, 2397}, {60, 132, 5, 2397}, {61, 133, 5, 2397}, {63, 137, 10, 2397}, {64, 138, 20, 2397}, {65, 139, 10, 2397}, {67, 143, 10, 2397}, {68, 144, 30, 2397}, {69, 145, 30, 2397}, {70, 146, 10, 2397}, {72, 150, 5, 2397}, {73, 151, 20, 2397}, {74, 152, 30, 2397}, {75, 153, 20, 2397}, {76, 154, 5, 2397}, {78, 158, 1, 2397}, {79, 159, 5, 2397}, {80, 160, 10, 2397}, {81, 161, 10, 2397}, {82, 162, 5, 2397}, {83, 163, 1, 2397}, {58, 129, 1, 2398}, {60, 133, 5, 2398}, {61, 134, 5, 2398}, {63, 138, 10, 2398}, {64, 139, 20, 2398}, {65, 140, 10, 2398}, {67, 144, 10, 2398}, {68, 145, 30, 2398}, {69, 146, 30, 2398}, {70, 147, 10, 2398}, {72, 151, 5, 2398}, {73, 152, 20, 2398}, {74, 153, 30, 2398}, {75, 154, 20, 2398}, {76, 155, 5, 2398}, {78, 159, 1, 2398}, {79, 160, 5, 2398}, {80, 161, 10, 2398}, {81, 162, 10, 2398}, {82, 163, 5, 2398}, {83, 164, 1, 2398}, {56, 165, 1, 2399}, {57, 166, 5, 2399}, {58, 167, 5, 2399}, {59, 168, 10, 2399}, {60, 169, 20, 2399}, {61, 170, 10, 2399}, {62, 171, 10, 2399}, {63, 172, 30, 2399}, {64, 173, 30, 2399}, {65, 174, 10, 2399}, {66, 175, 5, 2399}, {67, 176, 20, 2399}, {68, 177, 30, 2399}, {69, 178, 20, 2399}, {70, 179, 5, 2399}, {71, 180, 1, 2399}, {72, 181, 5, 2399}, {73, 182, 10, 2399}, {74, 183, 10, 2399}, {75, 184, 5, 2399}, {76, 185, 1, 2399}, {56, 166, 1, 2400}, {57, 168, 5, 2400}, {58, 169, 5, 2400}, {59, 171, 10, 2400}, {60, 172, 20, 2400}, {61, 173, 10, 2400}, {62, 175, 10, 2400}, {63, 176, 30, 2400}, {64, 177, 30, 2400}, {65, 178, 10, 2400}, {66, 180, 5, 2400}, {67, 181, 20, 2400}, {68, 182, 30, 2400}, {69, 183, 20, 2400}, {70, 184, 5, 2400}, {71, 186, 1, 2400}, {72, 187, 5, 2400}, {73, 188, 10, 2400}, {74, 189, 10, 2400}, {75, 190, 5, 2400}, {76, 191, 1, 2400}, {56, 167, 1, 2401}, {57, 169, 5, 2401}, {58, 170, 5, 2401}, {59, 172, 10, 2401}, {60, 173, 20, 2401}, {61, 174, 10, 2401}, {62, 176, 10, 2401}, {63, 177, 30, 2401}, {64, 178, 30, 2401}, {65, 179, 10, 2401}, {66, 181, 5, 2401}, {67, 182, 20, 2401}, {68, 183, 30, 2401}, {69, 184, 20, 2401}, {70, 185, 5, 2401}, {71, 187, 1, 2401}, {72, 188, 5, 2401}, {73, 189, 10, 2401}, {74, 190, 10, 2401}, {75, 191, 5, 2401}, {76, 192, 1, 2401}, {56, 168, 1, 2402}, {57, 171, 5, 2402}, {58, 172, 5, 2402}, {59, 175, 10, 2402}, {60, 176, 20, 2402}, {61, 177, 10, 2402}, {62, 180, 10, 2402}, {63, 181, 30, 2402}, {64, 182, 30, 2402}, {65, 183, 10, 2402}, {66, 186, 5, 2402}, {67, 187, 20, 2402}, {68, 188, 30, 2402}, {69, 189, 20, 2402}, {70, 190, 5, 2402}, {71, 193, 1, 2402}, {72, 194, 5, 2402}, {73, 195, 10, 2402}, {74, 196, 10, 2402}, {75, 197, 5, 2402}, {76, 198, 1, 2402}, {56, 169, 1, 2403}, {57, 172, 5, 2403}, {58, 173, 5, 2403}, {59, 176, 10, 2403}, {60, 177, 20, 2403}, {61, 178, 10, 2403}, {62, 181, 10, 2403}, {63, 182, 30, 2403}, {64, 183, 30, 2403}, {65, 184, 10, 2403}, {66, 187, 5, 2403}, {67, 188, 20, 2403}, {68, 189, 30, 2403}, {69, 190, 20, 2403}, {70, 191, 5, 2403}, {71, 194, 1, 2403}, {72, 195, 5, 2403}, {73, 196, 10, 2403}, {74, 197, 10, 2403}, {75, 198, 5, 2403}, {76, 199, 1, 2403}, {56, 170, 1, 2404}, {57, 173, 5, 2404}, {58, 174, 5, 2404}, {59, 177, 10, 2404}, {60, 178, 20, 2404}, {61, 179, 10, 2404}, {62, 182, 10, 2404}, {63, 183, 30, 2404}, {64, 184, 30, 2404}, {65, 185, 10, 2404}, {66, 188, 5, 2404}, {67, 189, 20, 2404}, {68, 190, 30, 2404}, {69, 191, 20, 2404}, {70, 192, 5, 2404}, {71, 195, 1, 2404}, {72, 196, 5, 2404}, {73, 197, 10, 2404}, {74, 198, 10, 2404}, {75, 199, 5, 2404}, {76, 200, 1, 2404}, {56, 171, 1, 2405}, {57, 175, 5, 2405}, {58, 176, 5, 2405}, {59, 180, 10, 2405}, {60, 181, 20, 2405}, {61, 182, 10, 2405}, {62, 186, 10, 2405}, {63, 187, 30, 2405}, {64, 188, 30, 2405}, {65, 189, 10, 2405}, {66, 193, 5, 2405}, {67, 194, 20, 2405}, {68, 195, 30, 2405}, {69, 196, 20, 2405}, {70, 197, 5, 2405}, {71, 201, 1, 2405}, {72, 202, 5, 2405}, {73, 203, 10, 2405}, {74, 204, 10, 2405}, {75, 205, 5, 2405}, {76, 206, 1, 2405}, {56, 172, 1, 2406}, {57, 176, 5, 2406}, {58, 177, 5, 2406}, {59, 181, 10, 2406}, {60, 182, 20, 2406}, {61, 183, 10, 2406}, {62, 187, 10, 2406}, {63, 188, 30, 2406}, {64, 189, 30, 2406}, {65, 190, 10, 2406}, {66, 194, 5, 2406}, {67, 195, 20, 2406}, {68, 196, 30, 2406}, {69, 197, 20, 2406}, {70, 198, 5, 2406}, {71, 202, 1, 2406}, {72, 203, 5, 2406}, {73, 204, 10, 2406}, {74, 205, 10, 2406}, {75, 206, 5, 2406}, {76, 207, 1, 2406}, {56, 173, 1, 2407}, {57, 177, 5, 2407}, {58, 178, 5, 2407}, {59, 182, 10, 2407}, {60, 183, 20, 2407}, {61, 184, 10, 2407}, {62, 188, 10, 2407}, {63, 189, 30, 2407}, {64, 190, 30, 2407}, {65, 191, 10, 2407}, {66, 195, 5, 2407}, {67, 196, 20, 2407}, {68, 197, 30, 2407}, {69, 198, 20, 2407}, {70, 199, 5, 2407}, {71, 203, 1, 2407}, {72, 204, 5, 2407}, {73, 205, 10, 2407}, {74, 206, 10, 2407}, {75, 207, 5, 2407}, {76, 208, 1, 2407}, {56, 174, 1, 2408}, {57, 178, 5, 2408}, {58, 179, 5, 2408}, {59, 183, 10, 2408}, {60, 184, 20, 2408}, {61, 185, 10, 2408}, {62, 189, 10, 2408}, {63, 190, 30, 2408}, {64, 191, 30, 2408}, {65, 192, 10, 2408}, {66, 196, 5, 2408}, {67, 197, 20, 2408}, {68, 198, 30, 2408}, {69, 199, 20, 2408}, {70, 200, 5, 2408}, {71, 204, 1, 2408}, {72, 205, 5, 2408}, {73, 206, 10, 2408}, {74, 207, 10, 2408}, {75, 208, 5, 2408}, {76, 209, 1, 2408}, {56, 175, 1, 2409}, {57, 180, 5, 2409}, {58, 181, 5, 2409}, {59, 186, 10, 2409}, {60, 187, 20, 2409}, {61, 188, 10, 2409}, {62, 193, 10, 2409}, {63, 194, 30, 2409}, {64, 195, 30, 2409}, {65, 196, 10, 2409}, {66, 201, 5, 2409}, {67, 202, 20, 2409}, {68, 203, 30, 2409}, {69, 204, 20, 2409}, {70, 205, 5, 2409}, {71, 210, 1, 2409}, {72, 211, 5, 2409}, {73, 212, 10, 2409}, {74, 213, 10, 2409}, {75, 214, 5, 2409}, {76, 215, 1, 2409}, {56, 176, 1, 2410}, {57, 181, 5, 2410}, {58, 182, 5, 2410}, {59, 187, 10, 2410}, {60, 188, 20, 2410}, {61, 189, 10, 2410}, {62, 194, 10, 2410}, {63, 195, 30, 2410}, {64, 196, 30, 2410}, {65, 197, 10, 2410}, {66, 202,

5, 2410}, {67, 203, 20, 2410}, {68, 204, 30, 2410}, {69, 205, 20, 2410}, {70, 206, 5,
2410}, {71, 211, 1, 2410}, {72, 212, 5, 2410}, {73, 213, 10, 2410}, {74, 214, 10, 2410},
{75, 215, 5, 2410}, {76, 216, 1, 2410}, {56, 177, 1, 2411}, {57, 182, 5, 2411}, {58, 183,
5, 2411}, {59, 188, 10, 2411}, {60, 189, 20, 2411}, {61, 190, 10, 2411}, {62, 195, 10,
2411}, {63, 196, 30, 2411}, {64, 197, 30, 2411}, {65, 198, 10, 2411}, {66, 203, 5, 2411},
{67, 204, 20, 2411}, {68, 205, 30, 2411}, {69, 206, 20, 2411}, {70, 207, 5, 2411}, {71,
212, 1, 2411}, {72, 213, 5, 2411}, {73, 214, 10, 2411}, {74, 215, 10, 2411}, {75, 216, 5,
2411}, {76, 217, 1, 2411}, {56, 178, 1, 2412}, {57, 183, 5, 2412}, {58, 184, 5, 2412},
{59, 189, 10, 2412}, {60, 190, 20, 2412}, {61, 191, 10, 2412}, {62, 196, 10, 2412}, {63,
197, 30, 2412}, {64, 198, 30, 2412}, {65, 199, 10, 2412}, {66, 204, 5, 2412}, {67, 205,
20, 2412}, {68, 206, 30, 2412}, {69, 207, 20, 2412}, {70, 208, 5, 2412}, {71, 213, 1,
2412}, {72, 214, 5, 2412}, {73, 215, 10, 2412}, {74, 216, 10, 2412}, {75, 217, 5, 2412},
{76, 218, 1, 2412}, {56, 179, 1, 2413}, {57, 184, 5, 2413}, {58, 185, 5, 2413}, {59, 190,
10, 2413}, {60, 191, 20, 2413}, {61, 192, 10, 2413}, {62, 197, 10, 2413}, {63, 198, 30,
2413}, {64, 199, 30, 2413}, {65, 200, 10, 2413}, {66, 205, 5, 2413}, {67, 206, 20, 2413},
{68, 207, 30, 2413}, {69, 208, 20, 2413}, {70, 209, 5, 2413}, {71, 214, 1, 2413}, {72,
215, 5, 2413}, {73, 216, 10, 2413}, {74, 217, 10, 2413}, {75, 218, 5, 2413}, {76, 219, 1,
2413}, {57, 165, 1, 2414}, {59, 166, 5, 2414}, {60, 167, 5, 2414}, {62, 168, 10, 2414},
{63, 169, 20, 2414}, {64, 170, 10, 2414}, {66, 171, 10, 2414}, {67, 172, 30, 2414}, {68,
173, 30, 2414}, {69, 174, 10, 2414}, {71, 175, 5, 2414}, {72, 176, 20, 2414}, {73, 177,
30, 2414}, {74, 178, 20, 2414}, {75, 179, 5, 2414}, {77, 180, 1, 2414}, {78, 181, 5,
2414}, {79, 182, 10, 2414}, {80, 183, 10, 2414}, {81, 184, 5, 2414}, {82, 185, 1, 2414},
{57, 166, 1, 2415}, {59, 168, 5, 2415}, {60, 169, 5, 2415}, {62, 171, 10, 2415}, {63, 172,
20, 2415}, {64, 173, 10, 2415}, {66, 175, 10, 2415}, {67, 176, 30, 2415}, {68, 177, 30,
2415}, {69, 178, 10, 2415}, {71, 180, 5, 2415}, {72, 181, 20, 2415}, {73, 182, 30, 2415},
{74, 183, 20, 2415}, {75, 184, 5, 2415}, {77, 186, 1, 2415}, {78, 187, 5, 2415}, {79, 188,
10, 2415}, {80, 189, 10, 2415}, {81, 190, 5, 2415}, {82, 191, 1, 2415}, {57, 167, 1,
2416}, {59, 169, 5, 2416}, {60, 170, 5, 2416}, {62, 172, 10, 2416}, {63, 173, 20, 2416},
{64, 174, 10, 2416}, {66, 176, 10, 2416}, {67, 177, 30, 2416}, {68, 178, 30, 2416}, {69,
179, 10, 2416}, {71, 181, 5, 2416}, {72, 182, 20, 2416}, {73, 183, 30, 2416}, {74, 184,
20, 2416}, {75, 185, 5, 2416}, {77, 187, 1, 2416}, {78, 188, 5, 2416}, {79, 189, 10,
2416}, {80, 190, 10, 2416}, {81, 191, 5, 2416}, {82, 192, 1, 2416}, {57, 168, 1, 2417},
{59, 171, 5, 2417}, {60, 172, 5, 2417}, {62, 175, 10, 2417}, {63, 176, 20, 2417}, {64,
177, 10, 2417}, {66, 180, 10, 2417}, {67, 181, 30, 2417}, {68, 182, 30, 2417}, {69, 183,
10, 2417}, {71, 186, 5, 2417}, {72, 187, 20, 2417}, {73, 188, 30, 2417}, {74, 189, 20,
2417}, {75, 190, 5, 2417}, {77, 193, 1, 2417}, {78, 194, 5, 2417}, {79, 195, 10, 2417},
{80, 196, 10, 2417}, {81, 197, 5, 2417}, {82, 198, 1, 2417}, {57, 169, 1, 2418}, {59, 172,
5, 2418}, {60, 173, 5, 2418}, {62, 176, 10, 2418}, {63, 177, 20, 2418}, {64, 178, 10,
2418}, {66, 181, 10, 2418}, {67, 182, 30, 2418}, {68, 183, 30, 2418}, {69, 184, 10, 2418},
{71, 187, 5, 2418}, {72, 188, 20, 2418}, {73, 189, 30, 2418}, {74, 190, 20, 2418}, {75,
191, 5, 2418}, {77, 194, 1, 2418}, {78, 195, 5, 2418}, {79, 196, 10, 2418}, {80, 197, 10,
2418}, {81, 198, 5, 2418}, {82, 199, 1, 2418}, {57, 170, 1, 2419}, {59, 173, 5, 2419},
{60, 174, 5, 2419}, {62, 177, 10, 2419}, {63, 178, 20, 2419}, {64, 179, 10, 2419}, {66,
182, 10, 2419}, {67, 183, 30, 2419}, {68, 184, 30, 2419}, {69, 185, 10, 2419}, {71, 188,
5, 2419}, {72, 189, 20, 2419}, {73, 190, 30, 2419}, {74, 191, 20, 2419}, {75, 192, 5,
2419}, {77, 195, 1, 2419}, {78, 196, 5, 2419}, {79, 197, 10, 2419}, {80, 198, 10, 2419},
{81, 199, 5, 2419}, {82, 200, 1, 2419}, {57, 171, 1, 2420}, {59, 175, 5, 2420}, {60, 176,
5, 2420}, {62, 180, 10, 2420}, {63, 181, 20, 2420}, {64, 182, 10, 2420}, {66, 186, 10,
2420}, {67, 187, 30, 2420}, {68, 188, 30, 2420}, {69, 189, 10, 2420}, {71, 193, 5, 2420},
{72, 194, 20, 2420}, {73, 195, 30, 2420}, {74, 196, 20, 2420}, {75, 197, 5, 2420}, {77,
201, 1, 2420}, {78, 202, 5, 2420}, {79, 203, 10, 2420}, {80, 204, 10, 2420}, {81, 205, 5,
2420}, {82, 206, 1, 2420}, {57, 172, 1, 2421}, {59, 176, 5, 2421}, {60, 177, 5, 2421},
{62, 181, 10, 2421}, {63, 182, 20, 2421}, {64, 183, 10, 2421}, {66, 187, 10, 2421}, {67,
188, 30, 2421}, {68, 189, 30, 2421}, {69, 190, 10, 2421}, {71, 194, 5, 2421}, {72, 195,
20, 2421}, {73, 196, 30, 2421}, {74, 197, 20, 2421}, {75, 198, 5, 2421}, {77, 202, 1,
2421}, {78, 203, 5, 2421}, {79, 204, 10, 2421}, {80, 205, 10, 2421}, {81, 206, 5, 2421},
{82, 207, 1, 2421}, {57, 173, 1, 2422}, {59, 177, 5, 2422}, {60, 178, 5, 2422}, {62, 182,
10, 2422}, {63, 183, 20, 2422}, {64, 184, 10, 2422}, {66, 188, 10, 2422}, {67, 189, 30,
2422}, {68, 190, 30, 2422}, {69, 191, 10, 2422}, {71, 195, 5, 2422}, {72, 196, 20, 2422},
{73, 197, 30, 2422}, {74, 198, 20, 2422}, {75, 199, 5, 2422}, {77, 203, 1, 2422}, {78,
204, 5, 2422}, {79, 205, 10, 2422}, {80, 206, 10, 2422}, {81, 207, 5, 2422}, {82, 208, 1,
2422}, {57, 174, 1, 2423}, {59, 178, 5, 2423}, {60, 179, 5, 2423}, {62, 183, 10, 2423},
{63, 184, 20, 2423}, {64, 185, 10, 2423}, {66, 189, 10, 2423}, {67, 190, 30, 2423}, {68,
191, 30, 2423}, {69, 192, 10, 2423}, {71, 196, 5, 2423}, {72, 197, 20, 2423}, {73, 198,
30, 2423}, {74, 199, 20, 2423}, {75, 200, 5, 2423}, {77, 204, 1, 2423}, {78, 205, 5,
2423}, {79, 206, 10, 2423}, {80, 207, 10, 2423}, {81, 208, 5, 2423}, {82, 209, 1, 2423},
{57, 175, 1, 2424}, {59, 180, 5, 2424}, {60, 181, 5, 2424}, {62, 186, 10, 2424}, {63, 187,
20, 2424}, {64, 188, 10, 2424}, {66, 193, 10, 2424}, {67, 194, 30, 2424}, {68, 195, 30,
2424}, {69, 196, 10, 2424}, {71, 201, 5, 2424}, {72, 202, 20, 2424}, {73, 203, 30, 2424},

{74, 204, 20, 2424}, {75, 205, 5, 2424}, {77, 210, 1, 2424}, {78, 211, 5, 2424}, {79, 212, 10, 2424}, {80, 213, 10, 2424}, {81, 214, 5, 2424}, {82, 215, 1, 2424}, {57, 176, 1, 2425}, {59, 181, 5, 2425}, {60, 182, 5, 2425}, {62, 187, 10, 2425}, {63, 188, 20, 2425}, {64, 189, 10, 2425}, {66, 194, 10, 2425}, {67, 195, 30, 2425}, {68, 196, 30, 2425}, {69, 197, 10, 2425}, {71, 202, 5, 2425}, {72, 203, 20, 2425}, {73, 204, 30, 2425}, {74, 205, 20, 2425}, {75, 206, 5, 2425}, {77, 211, 1, 2425}, {78, 212, 5, 2425}, {79, 213, 10, 2425}, {80, 214, 10, 2425}, {81, 215, 5, 2425}, {82, 216, 1, 2425}, {57, 177, 1, 2426}, {59, 182, 5, 2426}, {60, 183, 5, 2426}, {62, 188, 10, 2426}, {63, 189, 20, 2426}, {64, 190, 10, 2426}, {66, 195, 10, 2426}, {67, 196, 30, 2426}, {68, 197, 30, 2426}, {69, 198, 10, 2426}, {71, 203, 5, 2426}, {72, 204, 20, 2426}, {73, 205, 30, 2426}, {74, 206, 20, 2426}, {75, 207, 5, 2426}, {77, 212, 1, 2426}, {78, 213, 5, 2426}, {79, 214, 10, 2426}, {80, 215, 10, 2426}, {81, 216, 5, 2426}, {82, 217, 1, 2426}, {57, 178, 1, 2427}, {59, 183, 5, 2427}, {60, 184, 5, 2427}, {62, 189, 10, 2427}, {63, 190, 20, 2427}, {64, 191, 10, 2427}, {66, 196, 10, 2427}, {67, 197, 30, 2427}, {68, 198, 30, 2427}, {69, 199, 10, 2427}, {71, 204, 5, 2427}, {72, 205, 20, 2427}, {73, 206, 30, 2427}, {74, 207, 20, 2427}, {75, 208, 5, 2427}, {77, 213, 1, 2427}, {78, 214, 5, 2427}, {79, 215, 10, 2427}, {80, 216, 10, 2427}, {81, 217, 5, 2427}, {82, 218, 1, 2427}, {57, 179, 1, 2428}, {59, 184, 5, 2428}, {60, 185, 5, 2428}, {62, 190, 10, 2428}, {63, 191, 20, 2428}, {64, 192, 10, 2428}, {66, 197, 10, 2428}, {67, 198, 30, 2428}, {68, 199, 30, 2428}, {69, 200, 10, 2428}, {71, 205, 5, 2428}, {72, 206, 20, 2428}, {73, 207, 30, 2428}, {74, 208, 20, 2428}, {75, 209, 5, 2428}, {77, 214, 1, 2428}, {78, 215, 5, 2428}, {79, 216, 10, 2428}, {80, 217, 10, 2428}, {81, 218, 5, 2428}, {82, 219, 1, 2428}, {58, 165, 1, 2429}, {60, 166, 5, 2429}, {61, 167, 5, 2429}, {63, 168, 10, 2429}, {64, 169, 20, 2429}, {65, 170, 10, 2429}, {67, 171, 10, 2429}, {68, 172, 30, 2429}, {69, 173, 30, 2429}, {70, 174, 10, 2429}, {72, 175, 5, 2429}, {73, 176, 20, 2429}, {74, 177, 30, 2429}, {75, 178, 20, 2429}, {76, 179, 5, 2429}, {78, 180, 1, 2429}, {79, 181, 5, 2429}, {80, 182, 10, 2429}, {81, 183, 10, 2429}, {82, 184, 5, 2429}, {83, 185, 1, 2429}, {58, 166, 1, 2430}, {60, 168, 5, 2430}, {61, 169, 5, 2430}, {63, 171, 10, 2430}, {64, 172, 20, 2430}, {65, 173, 10, 2430}, {67, 175, 10, 2430}, {68, 176, 30, 2430}, {69, 177, 30, 2430}, {70, 178, 10, 2430}, {72, 180, 5, 2430}, {73, 181, 20, 2430}, {74, 182, 30, 2430}, {75, 183, 20, 2430}, {76, 184, 5, 2430}, {78, 186, 1, 2430}, {79, 187, 5, 2430}, {80, 188, 10, 2430}, {81, 189, 10, 2430}, {82, 190, 5, 2430}, {83, 191, 1, 2430}, {58, 167, 1, 2431}, {60, 169, 5, 2431}, {61, 170, 5, 2431}, {63, 172, 10, 2431}, {64, 173, 20, 2431}, {65, 174, 10, 2431}, {67, 176, 10, 2431}, {68, 177, 30, 2431}, {69, 178, 30, 2431}, {70, 179, 10, 2431}, {72, 181, 5, 2431}, {73, 182, 20, 2431}, {74, 183, 30, 2431}, {75, 184, 20, 2431}, {76, 185, 5, 2431}, {78, 187, 1, 2431}, {79, 188, 5, 2431}, {80, 189, 10, 2431}, {81, 190, 10, 2431}, {82, 191, 5, 2431}, {83, 192, 1, 2431}, {58, 168, 1, 2432}, {60, 171, 5, 2432}, {61, 172, 5, 2432}, {63, 175, 10, 2432}, {64, 176, 20, 2432}, {65, 177, 10, 2432}, {67, 180, 10, 2432}, {68, 181, 30, 2432}, {69, 182, 30, 2432}, {70, 183, 10, 2432}, {72, 186, 5, 2432}, {73, 187, 20, 2432}, {74, 188, 30, 2432}, {75, 189, 20, 2432}, {76, 190, 5, 2432}, {78, 193, 1, 2432}, {79, 194, 5, 2432}, {80, 195, 10, 2432}, {81, 196, 10, 2432}, {82, 197, 5, 2432}, {83, 198, 1, 2432}, {58, 169, 1, 2433}, {60, 172, 5, 2433}, {61, 173, 5, 2433}, {63, 176, 10, 2433}, {64, 177, 20, 2433}, {65, 178, 10, 2433}, {67, 181, 10, 2433}, {68, 182, 30, 2433}, {69, 183, 30, 2433}, {70, 184, 10, 2433}, {72, 187, 5, 2433}, {73, 188, 20, 2433}, {74, 189, 30, 2433}, {75, 190, 20, 2433}, {76, 191, 5, 2433}, {78, 194, 1, 2433}, {79, 195, 5, 2433}, {80, 196, 10, 2433}, {81, 197, 10, 2433}, {82, 198, 5, 2433}, {83, 199, 1, 2433}, {58, 170, 1, 2434}, {60, 173, 5, 2434}, {61, 174, 5, 2434}, {63, 177, 10, 2434}, {64, 178, 20, 2434}, {65, 179, 10, 2434}, {67, 182, 10, 2434}, {68, 183, 30, 2434}, {69, 184, 30, 2434}, {70, 185, 10, 2434}, {72, 188, 5, 2434}, {73, 189, 20, 2434}, {74, 190, 30, 2434}, {75, 191, 20, 2434}, {76, 192, 5, 2434}, {78, 195, 1, 2434}, {79, 196, 5, 2434}, {80, 197, 10, 2434}, {81, 198, 10, 2434}, {82, 199, 5, 2434}, {83, 200, 1, 2434}, {58, 171, 1, 2435}, {60, 175, 5, 2435}, {61, 176, 5, 2435}, {63, 180, 10, 2435}, {64, 181, 20, 2435}, {65, 182, 10, 2435}, {67, 186, 10, 2435}, {68, 187, 30, 2435}, {69, 188, 30, 2435}, {70, 189, 10, 2435}, {72, 193, 5, 2435}, {73, 194, 20, 2435}, {74, 195, 30, 2435}, {75, 196, 20, 2435}, {76, 197, 5, 2435}, {78, 201, 1, 2435}, {79, 202, 5, 2435}, {80, 203, 10, 2435}, {81, 204, 10, 2435}, {82, 205, 5, 2435}, {83, 206, 1, 2435}, {58, 172, 1, 2436}, {60, 176, 5, 2436}, {61, 177, 5, 2436}, {63, 181, 10, 2436}, {64, 182, 20, 2436}, {65, 183, 10, 2436}, {67, 187, 10, 2436}, {68, 188, 30, 2436}, {69, 189, 30, 2436}, {70, 190, 10, 2436}, {72, 194, 5, 2436}, {73, 195, 20, 2436}, {74, 196, 30, 2436}, {75, 197, 20, 2436}, {76, 198, 5, 2436}, {78, 202, 1, 2436}, {79, 203, 5, 2436}, {80, 204, 10, 2436}, {81, 205, 10, 2436}, {82, 206, 5, 2436}, {83, 207, 1, 2436}, {58, 173, 1, 2437}, {60, 177, 5, 2437}, {61, 178, 5, 2437}, {63, 182, 10, 2437}, {64, 183, 20, 2437}, {65, 184, 10, 2437}, {67, 188, 10, 2437}, {68, 189, 30, 2437}, {69, 190, 30, 2437}, {70, 191, 10, 2437}, {72, 195, 5, 2437}, {73, 196, 20, 2437}, {74, 197, 30, 2437}, {75, 198, 20, 2437}, {76, 199, 5, 2437}, {78, 203, 1, 2437}, {79, 204, 5, 2437}, {80, 205, 10, 2437}, {81, 206, 10, 2437}, {82, 207, 5, 2437}, {83, 208, 1, 2437}, {58, 174, 1, 2438}, {60, 178, 5, 2438}, {61, 179, 5, 2438}, {63, 183, 10, 2438}, {64, 184, 20, 2438}, {65, 185, 10, 2438}, {67, 189, 10, 2438}, {68, 190, 30, 2438}, {69, 191, 30, 2438}, {70, 192, 10, 2438}, {72, 196, 5, 2438}, {73, 197, 20, 2438}, {74, 198, 30, 2438}, {75, 199, 20, 2438}, {76, 200, 5, 2438}, {78,

204, 1, 2438}, {79, 205, 5, 2438}, {80, 206, 10, 2438}, {81, 207, 10, 2438}, {82, 208, 5, 2438}, {83, 209, 1, 2438}, {58, 175, 1, 2439}, {60, 180, 5, 2439}, {61, 181, 5, 2439}, {63, 186, 10, 2439}, {64, 187, 20, 2439}, {65, 188, 10, 2439}, {67, 193, 10, 2439}, {68, 194, 30, 2439}, {69, 195, 30, 2439}, {70, 196, 10, 2439}, {72, 201, 5, 2439}, {73, 202, 20, 2439}, {74, 203, 30, 2439}, {75, 204, 20, 2439}, {76, 205, 5, 2439}, {78, 210, 1, 2439}, {79, 211, 5, 2439}, {80, 212, 10, 2439}, {81, 213, 10, 2439}, {82, 214, 5, 2439}, {83, 215, 1, 2439}, {58, 176, 1, 2440}, {60, 181, 5, 2440}, {61, 182, 5, 2440}, {63, 187, 10, 2440}, {64, 188, 20, 2440}, {65, 189, 10, 2440}, {67, 194, 10, 2440}, {68, 195, 30, 2440}, {69, 196, 30, 2440}, {70, 197, 10, 2440}, {72, 202, 5, 2440}, {73, 203, 20, 2440}, {74, 204, 30, 2440}, {75, 205, 20, 2440}, {76, 206, 5, 2440}, {78, 211, 1, 2440}, {79, 212, 5, 2440}, {80, 213, 10, 2440}, {81, 214, 10, 2440}, {82, 215, 5, 2440}, {83, 216, 1, 2440}, {58, 177, 1, 2441}, {60, 182, 5, 2441}, {61, 183, 5, 2441}, {63, 188, 10, 2441}, {64, 189, 20, 2441}, {65, 190, 10, 2441}, {67, 195, 10, 2441}, {68, 196, 30, 2441}, {69, 197, 30, 2441}, {70, 198, 10, 2441}, {72, 203, 5, 2441}, {73, 204, 20, 2441}, {74, 205, 30, 2441}, {75, 206, 20, 2441}, {76, 207, 5, 2441}, {78, 212, 1, 2441}, {79, 213, 5, 2441}, {80, 214, 10, 2441}, {81, 215, 10, 2441}, {82, 216, 5, 2441}, {83, 217, 1, 2441}, {58, 178, 1, 2442}, {60, 183, 5, 2442}, {61, 184, 5, 2442}, {63, 189, 10, 2442}, {64, 190, 20, 2442}, {65, 191, 10, 2442}, {67, 196, 10, 2442}, {68, 197, 30, 2442}, {69, 198, 30, 2442}, {70, 199, 10, 2442}, {72, 204, 5, 2442}, {73, 205, 20, 2442}, {74, 206, 30, 2442}, {75, 207, 20, 2442}, {76, 208, 5, 2442}, {78, 213, 1, 2442}, {79, 214, 5, 2442}, {80, 215, 10, 2442}, {81, 216, 10, 2442}, {82, 217, 5, 2442}, {83, 218, 1, 2442}, {58, 179, 1, 2443}, {60, 184, 5, 2443}, {61, 185, 5, 2443}, {63, 190, 10, 2443}, {64, 191, 20, 2443}, {65, 192, 10, 2443}, {67, 197, 10, 2443}, {68, 198, 30, 2443}, {69, 199, 30, 2443}, {70, 200, 10, 2443}, {72, 205, 5, 2443}, {73, 206, 20, 2443}, {74, 207, 30, 2443}, {75, 208, 20, 2443}, {76, 209, 5, 2443}, {78, 214, 1, 2443}, {79, 215, 5, 2443}, {80, 216, 10, 2443}, {81, 217, 10, 2443}, {82, 218, 5, 2443}, {83, 219, 1, 2443}, {56, 56, 1, 2444}, {57, 57, 6, 2444}, {58, 58, 6, 2444}, {59, 59, 15, 2444}, {60, 60, 30, 2444}, {61, 61, 15, 2444}, {62, 62, 20, 2444}, {63, 63, 60, 2444}, {64, 64, 60, 2444}, {65, 65, 20, 2444}, {66, 66, 15, 2444}, {67, 67, 60, 2444}, {68, 68, 90, 2444}, {69, 69, 60, 2444}, {70, 70, 15, 2444}, {71, 71, 6, 2444}, {72, 72, 30, 2444}, {73, 73, 60, 2444}, {74, 74, 60, 2444}, {75, 75, 30, 2444}, {76, 76, 6, 2444}, {77, 77, 1, 2444}, {78, 78, 6, 2444}, {79, 79, 15, 2444}, {80, 80, 20, 2444}, {81, 81, 15, 2444}, {82, 82, 6, 2444}, {83, 83, 1, 2444}, {56, 84, 1, 2445}, {57, 85, 6, 2445}, {58, 86, 6, 2445}, {59, 87, 15, 2445}, {60, 88, 30, 2445}, {61, 89, 15, 2445}, {62, 90, 20, 2445}, {63, 91, 60, 2445}, {64, 92, 60, 2445}, {65, 93, 20, 2445}, {66, 94, 15, 2445}, {67, 95, 60, 2445}, {68, 96, 90, 2445}, {69, 97, 60, 2445}, {70, 98, 15, 2445}, {71, 99, 6, 2445}, {72, 100, 30, 2445}, {73, 101, 60, 2445}, {74, 102, 60, 2445}, {75, 103, 30, 2445}, {76, 104, 6, 2445}, {77, 105, 1, 2445}, {78, 106, 6, 2445}, {79, 107, 15, 2445}, {80, 108, 20, 2445}, {81, 109, 15, 2445}, {82, 110, 6, 2445}, {83, 111, 1, 2445}, {56, 85, 1, 2446}, {57, 87, 6, 2446}, {58, 88, 6, 2446}, {59, 90, 15, 2446}, {60, 91, 30, 2446}, {61, 92, 15, 2446}, {62, 94, 20, 2446}, {63, 95, 60, 2446}, {64, 96, 60, 2446}, {65, 97, 20, 2446}, {66, 99, 15, 2446}, {67, 100, 60, 2446}, {68, 101, 90, 2446}, {69, 102, 60, 2446}, {70, 103, 15, 2446}, {71, 105, 6, 2446}, {72, 106, 30, 2446}, {73, 107, 60, 2446}, {74, 108, 60, 2446}, {75, 109, 30, 2446}, {76, 110, 6, 2446}, {77, 112, 1, 2446}, {78, 113, 6, 2446}, {79, 114, 15, 2446}, {80, 115, 20, 2446}, {81, 116, 15, 2446}, {82, 117, 6, 2446}, {83, 118, 1, 2446}, {56, 86, 1, 2447}, {57, 88, 6, 2447}, {58, 89, 6, 2447}, {59, 91, 15, 2447}, {60, 92, 30, 2447}, {61, 93, 15, 2447}, {62, 95, 20, 2447}, {63, 96, 60, 2447}, {64, 97, 60, 2447}, {65, 98, 20, 2447}, {66, 100, 15, 2447}, {67, 101, 60, 2447}, {68, 102, 90, 2447}, {69, 103, 60, 2447}, {70, 104, 15, 2447}, {71, 106, 6, 2447}, {72, 107, 30, 2447}, {73, 108, 60, 2447}, {74, 109, 60, 2447}, {75, 110, 30, 2447}, {76, 111, 6, 2447}, {77, 113, 1, 2447}, {78, 114, 6, 2447}, {79, 115, 15, 2447}, {80, 116, 20, 2447}, {81, 117, 15, 2447}, {82, 118, 6, 2447}, {83, 119, 1, 2447}, {56, 120, 1, 2448}, {57, 121, 6, 2448}, {58, 122, 6, 2448}, {59, 123, 15, 2448}, {60, 124, 30, 2448}, {61, 125, 15, 2448}, {62, 126, 20, 2448}, {63, 127, 60, 2448}, {64, 128, 60, 2448}, {65, 129, 20, 2448}, {66, 130, 15, 2448}, {67, 131, 60, 2448}, {68, 132, 90, 2448}, {69, 133, 60, 2448}, {70, 134, 15, 2448}, {71, 135, 6, 2448}, {72, 136, 30, 2448}, {73, 137, 60, 2448}, {74, 138, 60, 2448}, {75, 139, 30, 2448}, {76, 140, 6, 2448}, {77, 141, 1, 2448}, {78, 142, 6, 2448}, {79, 143, 15, 2448}, {80, 144, 20, 2448}, {81, 145, 15, 2448}, {82, 146, 6, 2448}, {83, 147, 1, 2448}, {56, 121, 1, 2449}, {57, 123, 6, 2449}, {58, 124, 6, 2449}, {59, 126, 15, 2449}, {60, 127, 30, 2449}, {61, 128, 15, 2449}, {62, 130, 20, 2449}, {63, 131, 60, 2449}, {64, 132, 60, 2449}, {65, 133, 20, 2449}, {66, 135, 15, 2449}, {67, 136, 60, 2449}, {68, 137, 90, 2449}, {69, 138, 60, 2449}, {70, 139, 15, 2449}, {71, 141, 6, 2449}, {72, 142, 30, 2449}, {73, 143, 60, 2449}, {74, 144, 60, 2449}, {75, 145, 30, 2449}, {76, 146, 6, 2449}, {77, 148, 1, 2449}, {78, 149, 6, 2449}, {79, 150, 15, 2449}, {80, 151, 20, 2449}, {81, 152, 15, 2449}, {82, 153, 6, 2449}, {83, 154, 1, 2449}, {56, 122, 1, 2450}, {57, 124, 6, 2450}, {58, 125, 6, 2450}, {59, 127, 15, 2450}, {60, 128, 30, 2450}, {61, 129, 15, 2450}, {62, 131, 20, 2450}, {63, 132, 60, 2450}, {64, 133, 60, 2450}, {65, 134, 20, 2450}, {66, 136, 15, 2450}, {67, 137, 60, 2450}, {68, 138, 90, 2450}, {69, 139, 60, 2450}, {70, 140, 15, 2450}, {71, 142, 6, 2450}, {72, 143, 30, 2450}, {73, 144, 60, 2450}, {74, 145, 60, 2450}, {75, 146, 30, 2450},

{76, 147, 6, 2450}, {77, 149, 1, 2450}, {78, 150, 6, 2450}, {79, 151, 15, 2450}, {80, 152, 20, 2450}, {81, 153, 15, 2450}, {82, 154, 6, 2450}, {83, 155, 1, 2450}, {56, 123, 1, 2451}, {57, 126, 6, 2451}, {58, 127, 6, 2451}, {59, 130, 15, 2451}, {60, 131, 30, 2451}, {61, 132, 15, 2451}, {62, 135, 20, 2451}, {63, 136, 60, 2451}, {64, 137, 60, 2451}, {65, 138, 20, 2451}, {66, 141, 15, 2451}, {67, 142, 60, 2451}, {68, 143, 90, 2451}, {69, 144, 60, 2451}, {70, 145, 15, 2451}, {71, 148, 6, 2451}, {72, 149, 30, 2451}, {73, 150, 60, 2451}, {74, 151, 60, 2451}, {75, 152, 30, 2451}, {76, 153, 6, 2451}, {77, 156, 1, 2451}, {78, 157, 6, 2451}, {79, 158, 15, 2451}, {80, 159, 20, 2451}, {81, 160, 15, 2451}, {82, 161, 6, 2451}, {83, 162, 1, 2451}, {56, 124, 1, 2452}, {57, 127, 6, 2452}, {58, 128, 6, 2452}, {59, 131, 15, 2452}, {60, 132, 30, 2452}, {61, 133, 15, 2452}, {62, 136, 20, 2452}, {63, 137, 60, 2452}, {64, 138, 60, 2452}, {65, 139, 20, 2452}, {66, 142, 15, 2452}, {67, 143, 60, 2452}, {68, 144, 90, 2452}, {69, 145, 60, 2452}, {70, 146, 15, 2452}, {71, 149, 6, 2452}, {72, 150, 30, 2452}, {73, 151, 60, 2452}, {74, 152, 60, 2452}, {75, 153, 30, 2452}, {76, 154, 6, 2452}, {77, 157, 1, 2452}, {78, 158, 6, 2452}, {79, 159, 15, 2452}, {80, 160, 20, 2452}, {81, 161, 15, 2452}, {82, 162, 6, 2452}, {83, 163, 1, 2452}, {56, 125, 1, 2453}, {57, 128, 6, 2453}, {58, 129, 6, 2453}, {59, 132, 15, 2453}, {60, 133, 30, 2453}, {61, 134, 15, 2453}, {62, 137, 20, 2453}, {63, 138, 60, 2453}, {64, 139, 60, 2453}, {65, 140, 20, 2453}, {66, 143, 15, 2453}, {67, 144, 60, 2453}, {68, 145, 90, 2453}, {69, 146, 60, 2453}, {70, 147, 15, 2453}, {71, 150, 6, 2453}, {72, 151, 30, 2453}, {73, 152, 60, 2453}, {74, 153, 60, 2453}, {75, 154, 30, 2453}, {76, 155, 6, 2453}, {77, 158, 1, 2453}, {78, 159, 6, 2453}, {79, 160, 15, 2453}, {80, 161, 20, 2453}, {81, 162, 15, 2453}, {82, 163, 6, 2453}, {83, 164, 1, 2453}, {56, 165, 1, 2454}, {57, 166, 6, 2454}, {58, 167, 6, 2454}, {59, 168, 15, 2454}, {60, 169, 30, 2454}, {61, 170, 15, 2454}, {62, 171, 20, 2454}, {63, 172, 60, 2454}, {64, 173, 60, 2454}, {65, 174, 20, 2454}, {66, 175, 15, 2454}, {67, 176, 60, 2454}, {68, 177, 90, 2454}, {69, 178, 60, 2454}, {70, 179, 15, 2454}, {71, 180, 6, 2454}, {72, 181, 30, 2454}, {73, 182, 60, 2454}, {74, 183, 60, 2454}, {75, 184, 30, 2454}, {76, 185, 6, 2454}, {77, 186, 1, 2454}, {78, 187, 6, 2454}, {79, 188, 15, 2454}, {80, 189, 20, 2454}, {81, 190, 15, 2454}, {82, 191, 6, 2454}, {83, 192, 1, 2454}, {56, 166, 1, 2455}, {57, 168, 6, 2455}, {58, 169, 6, 2455}, {59, 171, 15, 2455}, {60, 172, 30, 2455}, {61, 173, 15, 2455}, {62, 175, 20, 2455}, {63, 176, 60, 2455}, {64, 177, 60, 2455}, {65, 178, 20, 2455}, {66, 180, 15, 2455}, {67, 181, 60, 2455}, {68, 182, 90, 2455}, {69, 183, 60, 2455}, {70, 184, 15, 2455}, {71, 186, 6, 2455}, {72, 187, 30, 2455}, {73, 188, 60, 2455}, {74, 189, 60, 2455}, {75, 190, 30, 2455}, {76, 191, 6, 2455}, {77, 193, 1, 2455}, {78, 194, 6, 2455}, {79, 195, 15, 2455}, {80, 196, 20, 2455}, {81, 197, 15, 2455}, {82, 198, 6, 2455}, {83, 199, 1, 2455}, {56, 167, 1, 2456}, {57, 169, 6, 2456}, {58, 170, 6, 2456}, {59, 172, 15, 2456}, {60, 173, 30, 2456}, {61, 174, 15, 2456}, {62, 176, 20, 2456}, {63, 177, 60, 2456}, {64, 178, 60, 2456}, {65, 179, 20, 2456}, {66, 181, 15, 2456}, {67, 182, 60, 2456}, {68, 183, 90, 2456}, {69, 184, 60, 2456}, {70, 185, 15, 2456}, {71, 187, 6, 2456}, {72, 188, 30, 2456}, {73, 189, 60, 2456}, {74, 190, 60, 2456}, {75, 191, 30, 2456}, {76, 192, 6, 2456}, {77, 194, 1, 2456}, {78, 195, 6, 2456}, {79, 196, 15, 2456}, {80, 197, 20, 2456}, {81, 198, 15, 2456}, {82, 199, 6, 2456}, {83, 200, 1, 2456}, {56, 168, 1, 2457}, {57, 171, 6, 2457}, {58, 172, 6, 2457}, {59, 175, 15, 2457}, {60, 176, 30, 2457}, {61, 177, 15, 2457}, {62, 180, 20, 2457}, {63, 181, 60, 2457}, {64, 182, 60, 2457}, {65, 183, 20, 2457}, {66, 186, 15, 2457}, {67, 187, 60, 2457}, {68, 188, 90, 2457}, {69, 189, 60, 2457}, {70, 190, 15, 2457}, {71, 193, 6, 2457}, {72, 194, 30, 2457}, {73, 195, 60, 2457}, {74, 196, 60, 2457}, {75, 197, 30, 2457}, {76, 198, 6, 2457}, {77, 201, 1, 2457}, {78, 202, 6, 2457}, {79, 203, 15, 2457}, {80, 204, 20, 2457}, {81, 205, 15, 2457}, {82, 206, 6, 2457}, {83, 207, 1, 2457}, {56, 169, 1, 2458}, {57, 172, 6, 2458}, {58, 173, 6, 2458}, {59, 176, 15, 2458}, {60, 177, 30, 2458}, {61, 178, 15, 2458}, {62, 181, 20, 2458}, {63, 182, 60, 2458}, {64, 183, 60, 2458}, {65, 184, 20, 2458}, {66, 187, 15, 2458}, {67, 188, 60, 2458}, {68, 189, 90, 2458}, {69, 190, 60, 2458}, {70, 191, 15, 2458}, {71, 194, 6, 2458}, {72, 195, 30, 2458}, {73, 196, 60, 2458}, {74, 197, 60, 2458}, {75, 198, 30, 2458}, {76, 199, 6, 2458}, {77, 202, 1, 2458}, {78, 203, 6, 2458}, {79, 204, 15, 2458}, {80, 205, 20, 2458}, {81, 206, 15, 2458}, {82, 207, 6, 2458}, {83, 208, 1, 2458}, {56, 170, 1, 2459}, {57, 173, 6, 2459}, {58, 174, 6, 2459}, {59, 177, 15, 2459}, {60, 178, 30, 2459}, {61, 179, 15, 2459}, {62, 182, 20, 2459}, {63, 183, 60, 2459}, {64, 184, 60, 2459}, {65, 185, 20, 2459}, {66, 188, 15, 2459}, {67, 189, 60, 2459}, {68, 190, 90, 2459}, {69, 191, 60, 2459}, {70, 192, 15, 2459}, {71, 195, 6, 2459}, {72, 196, 30, 2459}, {73, 197, 60, 2459}, {74, 198, 60, 2459}, {75, 199, 30, 2459}, {76, 200, 6, 2459}, {77, 203, 1, 2459}, {78, 204, 6, 2459}, {79, 205, 15, 2459}, {80, 206, 20, 2459}, {81, 207, 15, 2459}, {82, 208, 6, 2459}, {83, 209, 1, 2459}, {56, 171, 1, 2460}, {57, 175, 6, 2460}, {58, 176, 6, 2460}, {59, 180, 15, 2460}, {60, 181, 30, 2460}, {61, 182, 15, 2460}, {62, 186, 20, 2460}, {63, 187, 60, 2460}, {64, 188, 60, 2460}, {65, 189, 20, 2460}, {66, 193, 15, 2460}, {67, 194, 60, 2460}, {68, 195, 90, 2460}, {69, 196, 60, 2460}, {70, 197, 15, 2460}, {71, 201, 6, 2460}, {72, 202, 30, 2460}, {73, 203, 60, 2460}, {74, 204, 60, 2460}, {75, 205, 30, 2460}, {76, 206, 6, 2460}, {77, 210, 1, 2460}, {78, 211, 6, 2460}, {79, 212, 15, 2460}, {80, 213, 20, 2460}, {81, 214, 15, 2460}, {82, 215, 6, 2460}, {83, 216, 1, 2460}, {56, 172, 1, 2461}, {57, 176, 6, 2461}, {58, 177, 6, 2461}, {59, 181, 15, 2461}, {60, 182, 30, 2461}, {61, 183, 15, 2461}, {62, 187, 20, 2461}, {63, 188, 60, 2461}, {64, 189, 60,

2461}, {65, 190, 20, 2461}, {66, 194, 15, 2461}, {67, 195, 60, 2461}, {68, 196, 90, 2461},
{69, 197, 60, 2461}, {70, 198, 15, 2461}, {71, 202, 6, 2461}, {72, 203, 30, 2461}, {73,
204, 60, 2461}, {74, 205, 60, 2461}, {75, 206, 30, 2461}, {76, 207, 6, 2461}, {77, 211, 1,
2461}, {78, 212, 6, 2461}, {79, 213, 15, 2461}, {80, 214, 20, 2461}, {81, 215, 15, 2461},
{82, 216, 6, 2461}, {83, 217, 1, 2461}, {56, 173, 1, 2462}, {57, 177, 6, 2462}, {58, 178,
6, 2462}, {59, 182, 15, 2462}, {60, 183, 30, 2462}, {61, 184, 15, 2462}, {62, 188, 20,
2462}, {63, 189, 60, 2462}, {64, 190, 60, 2462}, {65, 191, 20, 2462}, {66, 195, 15, 2462},
{67, 196, 60, 2462}, {68, 197, 90, 2462}, {69, 198, 60, 2462}, {70, 199, 15, 2462}, {71,
203, 6, 2462}, {72, 204, 30, 2462}, {73, 205, 60, 2462}, {74, 206, 60, 2462}, {75, 207,
30, 2462}, {76, 208, 6, 2462}, {77, 212, 1, 2462}, {78, 213, 6, 2462}, {79, 214, 15,
2462}, {80, 215, 20, 2462}, {81, 216, 15, 2462}, {82, 217, 6, 2462}, {83, 218, 1, 2462},
{56, 174, 1, 2463}, {57, 178, 6, 2463}, {58, 179, 6, 2463}, {59, 183, 15, 2463}, {60, 184,
30, 2463}, {61, 185, 15, 2463}, {62, 189, 20, 2463}, {63, 190, 60, 2463}, {64, 191, 60,
2463}, {65, 192, 20, 2463}, {66, 196, 15, 2463}, {67, 197, 60, 2463}, {68, 198, 90, 2463},
{69, 199, 60, 2463}, {70, 200, 15, 2463}, {71, 204, 6, 2463}, {72, 205, 30, 2463}, {73,
206, 60, 2463}, {74, 207, 60, 2463}, {75, 208, 30, 2463}, {76, 209, 6, 2463}, {77, 213, 1,
2463}, {78, 214, 6, 2463}, {79, 215, 15, 2463}, {80, 216, 20, 2463}, {81, 217, 15, 2463},
{82, 218, 6, 2463}, {83, 219, 1, 2463}, {56, 220, 1, 2464}, {57, 221, 6, 2464}, {58, 222,
6, 2464}, {59, 223, 15, 2464}, {60, 224, 30, 2464}, {61, 225, 15, 2464}, {62, 226, 20,
2464}, {63, 227, 60, 2464}, {64, 228, 60, 2464}, {65, 229, 20, 2464}, {66, 230, 15, 2464},
{67, 231, 60, 2464}, {68, 232, 90, 2464}, {69, 233, 60, 2464}, {70, 234, 15, 2464}, {71,
235, 6, 2464}, {72, 236, 30, 2464}, {73, 237, 60, 2464}, {74, 238, 60, 2464}, {75, 239,
30, 2464}, {76, 240, 6, 2464}, {77, 241, 1, 2464}, {78, 242, 6, 2464}, {79, 243, 15,
2464}, {80, 244, 20, 2464}, {81, 245, 15, 2464}, {82, 246, 6, 2464}, {83, 247, 1, 2464},
{56, 221, 1, 2465}, {57, 223, 6, 2465}, {58, 224, 6, 2465}, {59, 226, 15, 2465}, {60, 227,
30, 2465}, {61, 228, 15, 2465}, {62, 230, 20, 2465}, {63, 231, 60, 2465}, {64, 232, 60,
2465}, {65, 233, 20, 2465}, {66, 235, 15, 2465}, {67, 236, 60, 2465}, {68, 237, 90, 2465},
{69, 238, 60, 2465}, {70, 239, 15, 2465}, {71, 241, 6, 2465}, {72, 242, 30, 2465}, {73,
243, 60, 2465}, {74, 244, 60, 2465}, {75, 245, 30, 2465}, {76, 246, 6, 2465}, {77, 248, 1,
2465}, {78, 249, 6, 2465}, {79, 250, 15, 2465}, {80, 251, 20, 2465}, {81, 252, 15, 2465},
{82, 253, 6, 2465}, {83, 254, 1, 2465}, {56, 222, 1, 2466}, {57, 224, 6, 2466}, {58, 225,
6, 2466}, {59, 227, 15, 2466}, {60, 228, 30, 2466}, {61, 229, 15, 2466}, {62, 231, 20,
2466}, {63, 232, 60, 2466}, {64, 233, 60, 2466}, {65, 234, 20, 2466}, {66, 236, 15, 2466},
{67, 237, 60, 2466}, {68, 238, 90, 2466}, {69, 239, 60, 2466}, {70, 240, 15, 2466}, {71,
242, 6, 2466}, {72, 243, 30, 2466}, {73, 244, 60, 2466}, {74, 245, 60, 2466}, {75, 246,
30, 2466}, {76, 247, 6, 2466}, {77, 249, 1, 2466}, {78, 250, 6, 2466}, {79, 251, 15,
2466}, {80, 252, 20, 2466}, {81, 253, 15, 2466}, {82, 254, 6, 2466}, {83, 255, 1, 2466},
{56, 223, 1, 2467}, {57, 226, 6, 2467}, {58, 227, 6, 2467}, {59, 230, 15, 2467}, {60, 231,
30, 2467}, {61, 232, 15, 2467}, {62, 235, 20, 2467}, {63, 236, 60, 2467}, {64, 237, 60,
2467}, {65, 238, 20, 2467}, {66, 241, 15, 2467}, {67, 242, 60, 2467}, {68, 243, 90, 2467},
{69, 244, 60, 2467}, {70, 245, 15, 2467}, {71, 248, 6, 2467}, {72, 249, 30, 2467}, {73,
250, 60, 2467}, {74, 251, 60, 2467}, {75, 252, 30, 2467}, {76, 253, 6, 2467}, {77, 256, 1,
2467}, {78, 257, 6, 2467}, {79, 258, 15, 2467}, {80, 259, 20, 2467}, {81, 260, 15, 2467},
{82, 261, 6, 2467}, {83, 262, 1, 2467}, {56, 224, 1, 2468}, {57, 227, 6, 2468}, {58, 228,
6, 2468}, {59, 231, 15, 2468}, {60, 232, 30, 2468}, {61, 233, 15, 2468}, {62, 236, 20,
2468}, {63, 237, 60, 2468}, {64, 238, 60, 2468}, {65, 239, 20, 2468}, {66, 242, 15, 2468},
{67, 243, 60, 2468}, {68, 244, 90, 2468}, {69, 245, 60, 2468}, {70, 246, 15, 2468}, {71,
249, 6, 2468}, {72, 250, 30, 2468}, {73, 251, 60, 2468}, {74, 252, 60, 2468}, {75, 253,
30, 2468}, {76, 254, 6, 2468}, {77, 257, 1, 2468}, {78, 258, 6, 2468}, {79, 259, 15,
2468}, {80, 260, 20, 2468}, {81, 261, 15, 2468}, {82, 262, 6, 2468}, {83, 263, 1, 2468},
{56, 225, 1, 2469}, {57, 228, 6, 2469}, {58, 229, 6, 2469}, {59, 232, 15, 2469}, {60, 233,
30, 2469}, {61, 234, 15, 2469}, {62, 237, 20, 2469}, {63, 238, 60, 2469}, {64, 239, 60,
2469}, {65, 240, 20, 2469}, {66, 243, 15, 2469}, {67, 244, 60, 2469}, {68, 245, 90, 2469},
{69, 246, 60, 2469}, {70, 247, 15, 2469}, {71, 250, 6, 2469}, {72, 251, 30, 2469}, {73,
252, 60, 2469}, {74, 253, 60, 2469}, {75, 254, 30, 2469}, {76, 255, 6, 2469}, {77, 258, 1,
2469}, {78, 259, 6, 2469}, {79, 260, 15, 2469}, {80, 261, 20, 2469}, {81, 262, 15, 2469},
{82, 263, 6, 2469}, {83, 264, 1, 2469}, {56, 226, 1, 2470}, {57, 230, 6, 2470}, {58, 231,
6, 2470}, {59, 235, 15, 2470}, {60, 236, 30, 2470}, {61, 237, 15, 2470}, {62, 241, 20,
2470}, {63, 242, 60, 2470}, {64, 243, 60, 2470}, {65, 244, 20, 2470}, {66, 248, 15, 2470},
{67, 249, 60, 2470}, {68, 250, 90, 2470}, {69, 251, 60, 2470}, {70, 252, 15, 2470}, {71,
256, 6, 2470}, {72, 257, 30, 2470}, {73, 258, 60, 2470}, {74, 259, 60, 2470}, {75, 260,
30, 2470}, {76, 261, 6, 2470}, {77, 265, 1, 2470}, {78, 266, 6, 2470}, {79, 267, 15,
2470}, {80, 268, 20, 2470}, {81, 269, 15, 2470}, {82, 270, 6, 2470}, {83, 271, 1, 2470},
{56, 227, 1, 2471}, {57, 231, 6, 2471}, {58, 232, 6, 2471}, {59, 236, 15, 2471}, {60, 237,
30, 2471}, {61, 238, 15, 2471}, {62, 242, 20, 2471}, {63, 243, 60, 2471}, {64, 244, 60,
2471}, {65, 245, 20, 2471}, {66, 249, 15, 2471}, {67, 250, 60, 2471}, {68, 251, 90, 2471},
{69, 252, 60, 2471}, {70, 253, 15, 2471}, {71, 257, 6, 2471}, {72, 258, 30, 2471}, {73,
259, 60, 2471}, {74, 260, 60, 2471}, {75, 261, 30, 2471}, {76, 262, 6, 2471}, {77, 266, 1,
2471}, {78, 267, 6, 2471}, {79, 268, 15, 2471}, {80, 269, 20, 2471}, {81, 270, 15, 2471},

{82, 271, 6, 2471}, {83, 272, 1, 2471}, {56, 228, 1, 2472}, {57, 232, 6, 2472}, {58, 233,
6, 2472}, {59, 237, 15, 2472}, {60, 238, 30, 2472}, {61, 239, 15, 2472}, {62, 243, 20,
2472}, {63, 244, 60, 2472}, {64, 245, 60, 2472}, {65, 246, 20, 2472}, {66, 250, 15, 2472},
{67, 251, 60, 2472}, {68, 252, 90, 2472}, {69, 253, 60, 2472}, {70, 254, 15, 2472}, {71,
258, 6, 2472}, {72, 259, 30, 2472}, {73, 260, 60, 2472}, {74, 261, 60, 2472}, {75, 262,
30, 2472}, {76, 263, 6, 2472}, {77, 267, 1, 2472}, {78, 268, 6, 2472}, {79, 269, 15,
2472}, {80, 270, 20, 2472}, {81, 271, 15, 2472}, {82, 272, 6, 2472}, {83, 273, 1, 2472},
{56, 229, 1, 2473}, {57, 233, 6, 2473}, {58, 234, 6, 2473}, {59, 238, 15, 2473}, {60, 239,
30, 2473}, {61, 240, 15, 2473}, {62, 244, 20, 2473}, {63, 245, 60, 2473}, {64, 246, 60,
2473}, {65, 247, 20, 2473}, {66, 251, 15, 2473}, {67, 252, 60, 2473}, {68, 253, 90, 2473},
{69, 254, 60, 2473}, {70, 255, 15, 2473}, {71, 259, 6, 2473}, {72, 260, 30, 2473}, {73,
261, 60, 2473}, {74, 262, 60, 2473}, {75, 263, 30, 2473}, {76, 264, 6, 2473}, {77, 268, 1,
2473}, {78, 269, 6, 2473}, {79, 270, 15, 2473}, {80, 271, 20, 2473}, {81, 272, 15, 2473},
{82, 273, 6, 2473}, {83, 274, 1, 2473}, {56, 230, 1, 2474}, {57, 235, 6, 2474}, {58, 236,
6, 2474}, {59, 241, 15, 2474}, {60, 242, 30, 2474}, {61, 243, 15, 2474}, {62, 248, 20,
2474}, {63, 249, 60, 2474}, {64, 250, 60, 2474}, {65, 251, 20, 2474}, {66, 256, 15, 2474},
{67, 257, 60, 2474}, {68, 258, 90, 2474}, {69, 259, 60, 2474}, {70, 260, 15, 2474}, {71,
265, 6, 2474}, {72, 266, 30, 2474}, {73, 267, 60, 2474}, {74, 268, 60, 2474}, {75, 269,
30, 2474}, {76, 270, 6, 2474}, {77, 275, 1, 2474}, {78, 276, 6, 2474}, {79, 277, 15,
2474}, {80, 278, 20, 2474}, {81, 279, 15, 2474}, {82, 280, 6, 2474}, {83, 281, 1, 2474},
{56, 231, 1, 2475}, {57, 236, 6, 2475}, {58, 237, 6, 2475}, {59, 242, 15, 2475}, {60, 243,
30, 2475}, {61, 244, 15, 2475}, {62, 249, 20, 2475}, {63, 250, 60, 2475}, {64, 251, 60,
2475}, {65, 252, 20, 2475}, {66, 257, 15, 2475}, {67, 258, 60, 2475}, {68, 259, 90, 2475},
{69, 260, 60, 2475}, {70, 261, 15, 2475}, {71, 266, 6, 2475}, {72, 267, 30, 2475}, {73,
268, 60, 2475}, {74, 269, 60, 2475}, {75, 270, 30, 2475}, {76, 271, 6, 2475}, {77, 276, 1,
2475}, {78, 277, 6, 2475}, {79, 278, 15, 2475}, {80, 279, 20, 2475}, {81, 280, 15, 2475},
{82, 281, 6, 2475}, {83, 282, 1, 2475}, {56, 232, 1, 2476}, {57, 237, 6, 2476}, {58, 238,
6, 2476}, {59, 243, 15, 2476}, {60, 244, 30, 2476}, {61, 245, 15, 2476}, {62, 250, 20,
2476}, {63, 251, 60, 2476}, {64, 252, 60, 2476}, {65, 253, 20, 2476}, {66, 258, 15, 2476},
{67, 259, 60, 2476}, {68, 260, 90, 2476}, {69, 261, 60, 2476}, {70, 262, 15, 2476}, {71,
267, 6, 2476}, {72, 268, 30, 2476}, {73, 269, 60, 2476}, {74, 270, 60, 2476}, {75, 271,
30, 2476}, {76, 272, 6, 2476}, {77, 277, 1, 2476}, {78, 278, 6, 2476}, {79, 279, 15,
2476}, {80, 280, 20, 2476}, {81, 281, 15, 2476}, {82, 282, 6, 2476}, {83, 283, 1, 2476},
{56, 233, 1, 2477}, {57, 238, 6, 2477}, {58, 239, 6, 2477}, {59, 244, 15, 2477}, {60, 245,
30, 2477}, {61, 246, 15, 2477}, {62, 251, 20, 2477}, {63, 252, 60, 2477}, {64, 253, 60,
2477}, {65, 254, 20, 2477}, {66, 259, 15, 2477}, {67, 260, 60, 2477}, {68, 261, 90, 2477},
{69, 262, 60, 2477}, {70, 263, 15, 2477}, {71, 268, 6, 2477}, {72, 269, 30, 2477}, {73,
270, 60, 2477}, {74, 271, 60, 2477}, {75, 272, 30, 2477}, {76, 273, 6, 2477}, {77, 278, 1,
2477}, {78, 279, 6, 2477}, {79, 280, 15, 2477}, {80, 281, 20, 2477}, {81, 282, 15, 2477},
{82, 283, 6, 2477}, {83, 284, 1, 2477}, {56, 234, 1, 2478}, {57, 239, 6, 2478}, {58, 240,
6, 2478}, {59, 245, 15, 2478}, {60, 246, 30, 2478}, {61, 247, 15, 2478}, {62, 252, 20,
2478}, {63, 253, 60, 2478}, {64, 254, 60, 2478}, {65, 255, 20, 2478}, {66, 260, 15, 2478},
{67, 261, 60, 2478}, {68, 262, 90, 2478}, {69, 263, 60, 2478}, {70, 264, 15, 2478}, {71,
269, 6, 2478}, {72, 270, 30, 2478}, {73, 271, 60, 2478}, {74, 272, 60, 2478}, {75, 273,
30, 2478}, {76, 274, 6, 2478}, {77, 279, 1, 2478}, {78, 280, 6, 2478}, {79, 281, 15,
2478}, {80, 282, 20, 2478}, {81, 283, 15, 2478}, {82, 284, 6, 2478}, {83, 285, 1, 2478},
{84, 84, 1, 2479}, {85, 85, 4, 2479}, {86, 86, 4, 2479}, {87, 87, 6, 2479}, {88, 88, 12,
2479}, {89, 89, 6, 2479}, {90, 90, 4, 2479}, {91, 91, 12, 2479}, {92, 92, 12, 2479}, {93,
93, 4, 2479}, {94, 94, 1, 2479}, {95, 95, 4, 2479}, {96, 96, 6, 2479}, {97, 97, 4, 2479},
{98, 98, 1, 2479}, {84, 85, 1, 2480}, {85, 87, 4, 2480}, {86, 88, 4, 2480}, {87, 90, 6,
2480}, {88, 91, 12, 2480}, {89, 92, 6, 2480}, {90, 94, 4, 2480}, {91, 95, 12, 2480}, {92,
96, 12, 2480}, {93, 97, 4, 2480}, {94, 99, 1, 2480}, {95, 100, 4, 2480}, {96, 101, 6,
2480}, {97, 102, 4, 2480}, {98, 103, 1, 2480}, {84, 86, 1, 2481}, {85, 88, 4, 2481}, {86,
89, 4, 2481}, {87, 91, 6, 2481}, {88, 92, 12, 2481}, {89, 93, 6, 2481}, {90, 95, 4, 2481},
{91, 96, 12, 2481}, {92, 97, 12, 2481}, {93, 98, 4, 2481}, {94, 100, 1, 2481}, {95, 101,
4, 2481}, {96, 102, 6, 2481}, {97, 103, 4, 2481}, {98, 104, 1, 2481}, {84, 87, 1, 2482},
{85, 90, 4, 2482}, {86, 91, 4, 2482}, {87, 94, 6, 2482}, {88, 95, 12, 2482}, {89, 96, 6,
2482}, {90, 99, 4, 2482}, {91, 100, 12, 2482}, {92, 101, 12, 2482}, {93, 102, 4, 2482},
{94, 105, 1, 2482}, {95, 106, 4, 2482}, {96, 107, 6, 2482}, {97, 108, 4, 2482}, {98, 109,
1, 2482}, {84, 88, 1, 2483}, {85, 91, 4, 2483}, {86, 92, 4, 2483}, {87, 95, 6, 2483}, {88,
96, 12, 2483}, {89, 97, 6, 2483}, {90, 100, 4, 2483}, {91, 101, 12, 2483}, {92, 102, 12,
2483}, {93, 103, 4, 2483}, {94, 106, 1, 2483}, {95, 107, 4, 2483}, {96, 108, 6, 2483},
{97, 109, 4, 2483}, {98, 110, 1, 2483}, {84, 89, 1, 2484}, {85, 92, 4, 2484}, {86, 93, 4,
2484}, {87, 96, 6, 2484}, {88, 97, 12, 2484}, {89, 98, 6, 2484}, {90, 101, 4, 2484}, {91,
102, 12, 2484}, {92, 103, 12, 2484}, {93, 104, 4, 2484}, {94, 107, 1, 2484}, {95, 108, 4,
2484}, {96, 109, 6, 2484}, {97, 110, 4, 2484}, {98, 111, 1, 2484}, {84, 90, 1, 2485}, {85,
94, 4, 2485}, {86, 95, 4, 2485}, {87, 99, 6, 2485}, {88, 100, 12, 2485}, {89, 101, 6,
2485}, {90, 105, 4, 2485}, {91, 106, 12, 2485}, {92, 107, 12, 2485}, {93, 108, 4, 2485},
{94, 112, 1, 2485}, {95, 113, 4, 2485}, {96, 114, 6, 2485}, {97, 115, 4, 2485}, {98, 116,

1, 2485}, {84, 91, 1, 2486}, {85, 95, 4, 2486}, {86, 96, 4, 2486}, {87, 100, 6, 2486}, {88, 101, 12, 2486}, {89, 102, 6, 2486}, {90, 106, 4, 2486}, {91, 107, 12, 2486}, {92, 108, 12, 2486}, {93, 109, 4, 2486}, {94, 113, 1, 2486}, {95, 114, 4, 2486}, {96, 115, 6, 2486}, {97, 116, 4, 2486}, {98, 117, 1, 2486}, {84, 92, 1, 2487}, {85, 96, 4, 2487}, {86, 97, 4, 2487}, {87, 101, 6, 2487}, {88, 102, 12, 2487}, {89, 103, 6, 2487}, {90, 107, 4, 2487}, {91, 108, 12, 2487}, {92, 109, 12, 2487}, {93, 110, 4, 2487}, {94, 114, 1, 2487}, {95, 115, 4, 2487}, {96, 116, 6, 2487}, {97, 117, 4, 2487}, {98, 118, 1, 2487}, {84, 93, 1, 2488}, {85, 97, 4, 2488}, {86, 98, 4, 2488}, {87, 102, 6, 2488}, {88, 103, 12, 2488}, {89, 104, 6, 2488}, {90, 108, 4, 2488}, {91, 109, 12, 2488}, {92, 110, 12, 2488}, {93, 111, 4, 2488}, {94, 115, 1, 2488}, {95, 116, 4, 2488}, {96, 117, 6, 2488}, {97, 118, 4, 2488}, {98, 119, 1, 2488}, {85, 85, 1, 2489}, {87, 87, 4, 2489}, {88, 88, 4, 2489}, {90, 90, 6, 2489}, {91, 91, 12, 2489}, {92, 92, 6, 2489}, {94, 94, 4, 2489}, {95, 95, 12, 2489}, {96, 96, 12, 2489}, {97, 97, 4, 2489}, {99, 99, 1, 2489}, {100, 100, 4, 2489}, {101, 101, 6, 2489}, {102, 102, 4, 2489}, {103, 103, 1, 2489}, {85, 86, 1, 2490}, {87, 88, 4, 2490}, {88, 89, 4, 2490}, {90, 91, 6, 2490}, {91, 92, 12, 2490}, {92, 93, 6, 2490}, {94, 95, 4, 2490}, {95, 96, 12, 2490}, {96, 97, 12, 2490}, {97, 98, 4, 2490}, {99, 100, 1, 2490}, {100, 101, 4, 2490}, {101, 102, 6, 2490}, {102, 103, 4, 2490}, {103, 104, 1, 2490}, {85, 87, 1, 2491}, {87, 90, 4, 2491}, {88, 91, 4, 2491}, {90, 94, 6, 2491}, {91, 95, 12, 2491}, {92, 96, 6, 2491}, {94, 99, 4, 2491}, {95, 100, 12, 2491}, {96, 101, 12, 2491}, {97, 102, 4, 2491}, {99, 105, 1, 2491}, {100, 106, 4, 2491}, {101, 107, 6, 2491}, {102, 108, 4, 2491}, {103, 109, 1, 2491}, {85, 88, 1, 2492}, {87, 91, 4, 2492}, {88, 92, 4, 2492}, {90, 95, 6, 2492}, {91, 96, 12, 2492}, {92, 97, 6, 2492}, {94, 100, 4, 2492}, {95, 101, 12, 2492}, {96, 102, 12, 2492}, {97, 103, 4, 2492}, {99, 106, 1, 2492}, {100, 107, 4, 2492}, {101, 108, 6, 2492}, {102, 109, 4, 2492}, {103, 110, 1, 2492}, {85, 89, 1, 2493}, {87, 92, 4, 2493}, {88, 93, 4, 2493}, {90, 96, 6, 2493}, {91, 97, 12, 2493}, {92, 98, 6, 2493}, {94, 101, 4, 2493}, {95, 102, 12, 2493}, {96, 103, 12, 2493}, {97, 104, 4, 2493}, {99, 107, 1, 2493}, {100, 108, 4, 2493}, {101, 109, 6, 2493}, {102, 110, 4, 2493}, {103, 111, 1, 2493}, {85, 90, 1, 2494}, {87, 94, 4, 2494}, {88, 95, 4, 2494}, {90, 99, 6, 2494}, {91, 100, 12, 2494}, {92, 101, 6, 2494}, {94, 105, 4, 2494}, {95, 106, 12, 2494}, {96, 107, 12, 2494}, {97, 108, 4, 2494}, {99, 112, 1, 2494}, {100, 113, 4, 2494}, {101, 114, 6, 2494}, {102, 115, 4, 2494}, {103, 116, 1, 2494}, {85, 91, 1, 2495}, {87, 95, 4, 2495}, {88, 96, 4, 2495}, {90, 100, 6, 2495}, {91, 101, 12, 2495}, {92, 102, 6, 2495}, {94, 106, 4, 2495}, {95, 107, 12, 2495}, {96, 108, 12, 2495}, {97, 109, 4, 2495}, {99, 113, 1, 2495}, {100, 114, 4, 2495}, {101, 115, 6, 2495}, {102, 116, 4, 2495}, {103, 117, 1, 2495}, {85, 92, 1, 2496}, {87, 96, 4, 2496}, {88, 97, 4, 2496}, {90, 101, 6, 2496}, {91, 102, 12, 2496}, {92, 103, 6, 2496}, {94, 107, 4, 2496}, {95, 108, 12, 2496}, {96, 109, 12, 2496}, {97, 110, 4, 2496}, {99, 114, 1, 2496}, {100, 115, 4, 2496}, {101, 116, 6, 2496}, {102, 117, 4, 2496}, {103, 118, 1, 2496}, {85, 93, 1, 2497}, {87, 97, 4, 2497}, {88, 98, 4, 2497}, {90, 102, 6, 2497}, {91, 103, 12, 2497}, {92, 104, 6, 2497}, {94, 108, 4, 2497}, {95, 109, 12, 2497}, {96, 110, 12, 2497}, {97, 111, 4, 2497}, {99, 115, 1, 2497}, {100, 116, 4, 2497}, {101, 117, 6, 2497}, {102, 118, 4, 2497}, {103, 119, 1, 2497}, {86, 86, 1, 2498}, {88, 88, 4, 2498}, {89, 89, 4, 2498}, {91, 91, 6, 2498}, {92, 92, 12, 2498}, {93, 93, 6, 2498}, {95, 95, 4, 2498}, {96, 96, 12, 2498}, {97, 97, 12, 2498}, {98, 98, 4, 2498}, {100, 100, 1, 2498}, {101, 101, 4, 2498}, {102, 102, 6, 2498}, {103, 103, 4, 2498}, {104, 104, 1, 2498}, {86, 87, 1, 2499}, {88, 90, 4, 2499}, {89, 91, 4, 2499}, {91, 94, 6, 2499}, {92, 95, 12, 2499}, {93, 96, 6, 2499}, {95, 99, 4, 2499}, {96, 100, 12, 2499}, {97, 101, 12, 2499}, {98, 102, 4, 2499}, {100, 105, 1, 2499}, {101, 106, 4, 2499}, {102, 107, 6, 2499}, {103, 108, 4, 2499}, {104, 109, 1, 2499}, {86, 88, 1, 2500}, {88, 91, 4, 2500}, {89, 92, 4, 2500}, {91, 95, 6, 2500}, {92, 96, 12, 2500}, {93, 97, 6, 2500}, {95, 100, 4, 2500}, {96, 101, 12, 2500}, {97, 102, 12, 2500}, {98, 103, 4, 2500}, {100, 106, 1, 2500}, {101, 107, 4, 2500}, {102, 108, 6, 2500}, {103, 109, 4, 2500}, {104, 110, 1, 2500}, {86, 89, 1, 2501}, {88, 92, 4, 2501}, {89, 93, 4, 2501}, {91, 96, 6, 2501}, {92, 97, 12, 2501}, {93, 98, 6, 2501}, {95, 101, 4, 2501}, {96, 102, 12, 2501}, {97, 103, 12, 2501}, {98, 104, 4, 2501}, {100, 107, 1, 2501}, {101, 108, 4, 2501}, {102, 109, 6, 2501}, {103, 110, 4, 2501}, {104, 111, 1, 2501}, {86, 90, 1, 2502}, {88, 94, 4, 2502}, {89, 95, 4, 2502}, {91, 99, 6, 2502}, {92, 100, 12, 2502}, {93, 101, 6, 2502}, {95, 105, 4, 2502}, {96, 106, 12, 2502}, {97, 107, 12, 2502}, {98, 108, 4, 2502}, {100, 112, 1, 2502}, {101, 113, 4, 2502}, {102, 114, 6, 2502}, {103, 115, 4, 2502}, {104, 116, 1, 2502}, {86, 91, 1, 2503}, {88, 95, 4, 2503}, {89, 96, 4, 2503}, {91, 100, 6, 2503}, {92, 101, 12, 2503}, {93, 102, 6, 2503}, {95, 106, 4, 2503}, {96, 107, 12, 2503}, {97, 108, 12, 2503}, {98, 109, 4, 2503}, {100, 113, 1, 2503}, {101, 114, 4, 2503}, {102, 115, 6, 2503}, {103, 116, 4, 2503}, {104, 117, 1, 2503}, {86, 92, 1, 2504}, {88, 96, 4, 2504}, {89, 97, 4, 2504}, {91, 101, 6, 2504}, {92, 102, 12, 2504}, {93, 103, 6, 2504}, {95, 107, 4, 2504}, {96, 108, 12, 2504}, {97, 109, 12, 2504}, {98, 110, 4, 2504}, {100, 114, 1, 2504}, {101, 115, 4, 2504}, {102, 116, 6, 2504}, {103, 117, 4, 2504}, {104, 118, 1, 2504}, {86, 93, 1, 2505}, {88, 97, 4, 2505}, {89, 98, 4, 2505}, {91, 102, 6, 2505}, {92, 103, 12, 2505}, {93, 104, 6, 2505}, {95, 108, 4, 2505}, {96, 109, 12, 2505}, {97, 110, 12, 2505}, {98, 111, 4, 2505}, {100, 115, 1, 2505}, {101, 116, 4, 2505}, {102, 117, 6, 2505}, {103, 118, 4, 2505}, {104, 119, 1, 2505}, {87, 87, 1, 2506}, {90, 90, 4, 2506}, {91, 91, 4, 2506}, {94, 94, 6, 2506}, {95, 95, 12,

2506}, {96, 96, 6, 2506}, {99, 99, 4, 2506}, {100, 100, 12, 2506}, {101, 101, 12, 2506}, {102, 102, 4, 2506}, {105, 105, 1, 2506}, {106, 106, 4, 2506}, {107, 107, 6, 2506}, {108, 108, 4, 2506}, {109, 109, 1, 2506}, {87, 88, 1, 2507}, {90, 91, 4, 2507}, {91, 92, 4, 2507}, {94, 95, 6, 2507}, {95, 96, 12, 2507}, {96, 97, 6, 2507}, {99, 100, 4, 2507}, {100, 101, 12, 2507}, {101, 102, 12, 2507}, {102, 103, 4, 2507}, {105, 106, 1, 2507}, {106, 107, 4, 2507}, {107, 108, 6, 2507}, {108, 109, 4, 2507}, {109, 110, 1, 2507}, {87, 89, 1, 2508}, {90, 92, 4, 2508}, {91, 93, 4, 2508}, {94, 96, 6, 2508}, {95, 97, 12, 2508}, {96, 98, 6, 2508}, {99, 101, 4, 2508}, {100, 102, 12, 2508}, {101, 103, 12, 2508}, {102, 104, 4, 2508}, {105, 107, 1, 2508}, {106, 108, 4, 2508}, {107, 109, 6, 2508}, {108, 110, 4, 2508}, {109, 111, 1, 2508}, {87, 90, 1, 2509}, {90, 94, 4, 2509}, {91, 95, 4, 2509}, {94, 99, 6, 2509}, {95, 100, 12, 2509}, {96, 101, 6, 2509}, {99, 105, 4, 2509}, {100, 106, 12, 2509}, {101, 107, 12, 2509}, {102, 108, 4, 2509}, {105, 112, 1, 2509}, {106, 113, 4, 2509}, {107, 114, 6, 2509}, {108, 115, 4, 2509}, {109, 116, 1, 2509}, {87, 91, 1, 2510}, {90, 95, 4, 2510}, {91, 96, 4, 2510}, {94, 100, 6, 2510}, {95, 101, 12, 2510}, {96, 102, 6, 2510}, {99, 106, 4, 2510}, {100, 107, 12, 2510}, {101, 108, 12, 2510}, {102, 109, 4, 2510}, {105, 113, 1, 2510}, {106, 114, 4, 2510}, {107, 115, 6, 2510}, {108, 116, 4, 2510}, {109, 117, 1, 2510}, {87, 92, 1, 2511}, {90, 96, 4, 2511}, {91, 97, 4, 2511}, {94, 101, 6, 2511}, {95, 102, 12, 2511}, {96, 103, 6, 2511}, {99, 107, 4, 2511}, {100, 108, 12, 2511}, {101, 109, 12, 2511}, {102, 110, 4, 2511}, {105, 114, 1, 2511}, {106, 115, 4, 2511}, {107, 116, 6, 2511}, {108, 117, 4, 2511}, {109, 118, 1, 2511}, {87, 93, 1, 2512}, {90, 97, 4, 2512}, {91, 98, 4, 2512}, {94, 102, 6, 2512}, {95, 103, 12, 2512}, {96, 104, 6, 2512}, {99, 108, 4, 2512}, {100, 109, 12, 2512}, {101, 110, 12, 2512}, {102, 111, 4, 2512}, {105, 115, 1, 2512}, {106, 116, 4, 2512}, {107, 117, 6, 2512}, {108, 118, 4, 2512}, {109, 119, 1, 2512}, {88, 88, 1, 2513}, {91, 91, 4, 2513}, {92, 92, 4, 2513}, {95, 95, 6, 2513}, {96, 96, 12, 2513}, {97, 97, 6, 2513}, {100, 100, 4, 2513}, {101, 101, 12, 2513}, {102, 102, 12, 2513}, {103, 103, 4, 2513}, {106, 106, 1, 2513}, {107, 107, 4, 2513}, {108, 108, 6, 2513}, {109, 109, 4, 2513}, {110, 110, 1, 2513}, {88, 89, 1, 2514}, {91, 92, 4, 2514}, {92, 93, 4, 2514}, {95, 96, 6, 2514}, {96, 97, 12, 2514}, {97, 98, 6, 2514}, {100, 101, 4, 2514}, {101, 102, 12, 2514}, {102, 103, 12, 2514}, {103, 104, 4, 2514}, {106, 107, 1, 2514}, {107, 108, 4, 2514}, {108, 109, 6, 2514}, {109, 110, 4, 2514}, {110, 111, 1, 2514}, {88, 90, 1, 2515}, {91, 94, 4, 2515}, {92, 95, 4, 2515}, {95, 99, 6, 2515}, {96, 100, 12, 2515}, {97, 101, 6, 2515}, {100, 105, 4, 2515}, {101, 106, 12, 2515}, {102, 107, 12, 2515}, {103, 108, 4, 2515}, {106, 112, 1, 2515}, {107, 113, 4, 2515}, {108, 114, 6, 2515}, {109, 115, 4, 2515}, {110, 116, 1, 2515}, {88, 91, 1, 2516}, {91, 95, 4, 2516}, {92, 96, 4, 2516}, {95, 100, 6, 2516}, {96, 101, 12, 2516}, {97, 102, 6, 2516}, {100, 106, 4, 2516}, {101, 107, 12, 2516}, {102, 108, 12, 2516}, {103, 109, 4, 2516}, {106, 113, 1, 2516}, {107, 114, 4, 2516}, {108, 115, 6, 2516}, {109, 116, 4, 2516}, {110, 117, 1, 2516}, {88, 92, 1, 2517}, {91, 96, 4, 2517}, {92, 97, 4, 2517}, {95, 101, 6, 2517}, {96, 102, 12, 2517}, {97, 103, 6, 2517}, {100, 107, 4, 2517}, {101, 108, 12, 2517}, {102, 109, 12, 2517}, {103, 110, 4, 2517}, {106, 114, 1, 2517}, {107, 115, 4, 2517}, {108, 116, 6, 2517}, {109, 117, 4, 2517}, {110, 118, 1, 2517}, {88, 93, 1, 2518}, {91, 97, 4, 2518}, {92, 98, 4, 2518}, {95, 102, 6, 2518}, {96, 103, 12, 2518}, {97, 104, 6, 2518}, {100, 108, 4, 2518}, {101, 109, 12, 2518}, {102, 110, 12, 2518}, {103, 111, 4, 2518}, {106, 115, 1, 2518}, {107, 116, 4, 2518}, {108, 117, 6, 2518}, {109, 118, 4, 2518}, {110, 119, 1, 2518}, {89, 89, 1, 2519}, {92, 92, 4, 2519}, {93, 93, 4, 2519}, {96, 96, 6, 2519}, {97, 97, 12, 2519}, {98, 98, 6, 2519}, {101, 101, 4, 2519}, {102, 102, 12, 2519}, {103, 103, 12, 2519}, {104, 104, 4, 2519}, {107, 107, 1, 2519}, {108, 108, 4, 2519}, {109, 109, 6, 2519}, {110, 110, 4, 2519}, {111, 111, 1, 2519}, {89, 90, 1, 2520}, {92, 94, 4, 2520}, {93, 95, 4, 2520}, {96, 99, 6, 2520}, {97, 100, 12, 2520}, {98, 101, 6, 2520}, {101, 105, 4, 2520}, {102, 106, 12, 2520}, {103, 107, 12, 2520}, {104, 108, 4, 2520}, {107, 112, 1, 2520}, {108, 113, 4, 2520}, {109, 114, 6, 2520}, {110, 115, 4, 2520}, {111, 116, 1, 2520}, {89, 91, 1, 2521}, {92, 95, 4, 2521}, {93, 96, 4, 2521}, {96, 100, 6, 2521}, {97, 101, 12, 2521}, {98, 102, 6, 2521}, {101, 106, 4, 2521}, {102, 107, 12, 2521}, {103, 108, 12, 2521}, {104, 109, 4, 2521}, {107, 113, 1, 2521}, {108, 114, 4, 2521}, {109, 115, 6, 2521}, {110, 116, 4, 2521}, {111, 117, 1, 2521}, {89, 92, 1, 2522}, {92, 96, 4, 2522}, {93, 97, 4, 2522}, {96, 101, 6, 2522}, {97, 102, 12, 2522}, {98, 103, 6, 2522}, {101, 107, 4, 2522}, {102, 108, 12, 2522}, {103, 109, 12, 2522}, {104, 110, 4, 2522}, {107, 114, 1, 2522}, {108, 115, 4, 2522}, {109, 116, 6, 2522}, {110, 117, 4, 2522}, {111, 118, 1, 2522}, {89, 93, 1, 2523}, {92, 97, 4, 2523}, {93, 98, 4, 2523}, {96, 102, 6, 2523}, {97, 103, 12, 2523}, {98, 104, 6, 2523}, {101, 108, 4, 2523}, {102, 109, 12, 2523}, {103, 110, 12, 2523}, {104, 111, 4, 2523}, {107, 115, 1, 2523}, {108, 116, 4, 2523}, {109, 117, 6, 2523}, {110, 118, 4, 2523}, {111, 119, 1, 2523}, {90, 90, 1, 2524}, {94, 94, 4, 2524}, {95, 95, 4, 2524}, {99, 99, 6, 2524}, {100, 100, 12, 2524}, {101, 101, 6, 2524}, {105, 105, 4, 2524}, {106, 106, 12, 2524}, {107, 107, 12, 2524}, {108, 108, 4, 2524}, {112, 112, 1, 2524}, {113, 113, 4, 2524}, {114, 114, 6, 2524}, {115, 115, 4, 2524}, {116, 116, 1, 2524}, {90, 91, 1, 2525}, {94, 95, 4, 2525}, {95, 96, 4, 2525}, {99, 100, 6, 2525}, {100, 101, 12, 2525}, {101, 102, 6, 2525}, {105, 106, 4, 2525}, {106, 107, 12, 2525}, {107, 108, 12, 2525}, {108, 109, 4, 2525}, {112, 113, 1, 2525}, {113, 114, 4, 2525}, {114, 115, 6, 2525}, {115, 116, 4, 2525}, {116, 117, 1, 2525}, {90, 92, 1, 2526}, {94, 96, 4, 2526}, {95, 97,

4, 2526}, {99, 101, 6, 2526}, {100, 102, 12, 2526}, {101, 103, 6, 2526}, {105, 107, 4, 2526}, {106, 108, 12, 2526}, {107, 109, 12, 2526}, {108, 110, 4, 2526}, {112, 114, 1, 2526}, {113, 115, 4, 2526}, {114, 116, 6, 2526}, {115, 117, 4, 2526}, {116, 118, 1, 2526}, {90, 93, 1, 2527}, {94, 97, 4, 2527}, {95, 98, 4, 2527}, {99, 102, 6, 2527}, {100, 103, 12, 2527}, {101, 104, 6, 2527}, {105, 108, 4, 2527}, {106, 109, 12, 2527}, {107, 110, 12, 2527}, {108, 111, 4, 2527}, {112, 115, 1, 2527}, {113, 116, 4, 2527}, {114, 117, 6, 2527}, {115, 118, 4, 2527}, {116, 119, 1, 2527}, {91, 91, 1, 2528}, {95, 95, 4, 2528}, {96, 96, 4, 2528}, {100, 100, 6, 2528}, {101, 101, 12, 2528}, {102, 102, 6, 2528}, {106, 106, 4, 2528}, {107, 107, 12, 2528}, {108, 108, 12, 2528}, {109, 109, 4, 2528}, {113, 113, 1, 2528}, {114, 114, 4, 2528}, {115, 115, 6, 2528}, {116, 116, 4, 2528}, {117, 117, 1, 2528}, {91, 92, 1, 2529}, {95, 96, 4, 2529}, {96, 97, 4, 2529}, {100, 101, 6, 2529}, {101, 102, 12, 2529}, {102, 103, 6, 2529}, {106, 107, 4, 2529}, {107, 108, 12, 2529}, {108, 109, 12, 2529}, {109, 110, 4, 2529}, {113, 114, 1, 2529}, {114, 115, 4, 2529}, {115, 116, 6, 2529}, {116, 117, 4, 2529}, {117, 118, 1, 2529}, {91, 93, 1, 2530}, {95, 97, 4, 2530}, {96, 98, 4, 2530}, {100, 102, 6, 2530}, {101, 103, 12, 2530}, {102, 104, 6, 2530}, {106, 108, 4, 2530}, {107, 109, 12, 2530}, {108, 110, 12, 2530}, {109, 111, 4, 2530}, {113, 115, 1, 2530}, {114, 116, 4, 2530}, {115, 117, 6, 2530}, {116, 118, 4, 2530}, {117, 119, 1, 2530}, {92, 92, 1, 2531}, {96, 96, 4, 2531}, {97, 97, 4, 2531}, {101, 101, 6, 2531}, {102, 102, 12, 2531}, {103, 103, 6, 2531}, {107, 107, 4, 2531}, {108, 108, 12, 2531}, {109, 109, 12, 2531}, {110, 110, 4, 2531}, {114, 114, 1, 2531}, {115, 115, 4, 2531}, {116, 116, 6, 2531}, {117, 117, 4, 2531}, {118, 118, 1, 2531}, {92, 93, 1, 2532}, {96, 97, 4, 2532}, {97, 98, 4, 2532}, {101, 102, 6, 2532}, {102, 103, 12, 2532}, {103, 104, 6, 2532}, {107, 108, 4, 2532}, {108, 109, 12, 2532}, {109, 110, 12, 2532}, {110, 111, 4, 2532}, {114, 115, 1, 2532}, {115, 116, 4, 2532}, {116, 117, 6, 2532}, {117, 118, 4, 2532}, {118, 119, 1, 2532}, {93, 93, 1, 2533}, {97, 97, 4, 2533}, {98, 98, 4, 2533}, {102, 102, 6, 2533}, {103, 103, 12, 2533}, {104, 104, 6, 2533}, {108, 108, 4, 2533}, {109, 109, 12, 2533}, {110, 110, 12, 2533}, {111, 111, 4, 2533}, {115, 115, 1, 2533}, {116, 116, 4, 2533}, {117, 117, 6, 2533}, {118, 118, 4, 2533}, {119, 119, 1, 2533}, {84, 84, 1, 2534}, {85, 85, 5, 2534}, {86, 86, 5, 2534}, {87, 87, 10, 2534}, {88, 88, 20, 2534}, {89, 89, 10, 2534}, {90, 90, 10, 2534}, {91, 91, 30, 2534}, {92, 92, 30, 2534}, {93, 93, 10, 2534}, {94, 94, 5, 2534}, {95, 95, 20, 2534}, {96, 96, 30, 2534}, {97, 97, 20, 2534}, {98, 98, 5, 2534}, {99, 99, 1, 2534}, {100, 100, 5, 2534}, {101, 101, 10, 2534}, {102, 102, 10, 2534}, {103, 103, 5, 2534}, {104, 104, 1, 2534}, {84, 85, 1, 2535}, {85, 87, 5, 2535}, {86, 88, 5, 2535}, {87, 90, 10, 2535}, {88, 91, 20, 2535}, {89, 92, 10, 2535}, {90, 94, 10, 2535}, {91, 95, 30, 2535}, {92, 96, 30, 2535}, {93, 97, 10, 2535}, {94, 99, 5, 2535}, {95, 100, 20, 2535}, {96, 101, 30, 2535}, {97, 102, 20, 2535}, {98, 103, 5, 2535}, {99, 105, 1, 2535}, {100, 106, 5, 2535}, {101, 107, 10, 2535}, {102, 108, 10, 2535}, {103, 109, 5, 2535}, {104, 110, 1, 2535}, {84, 86, 1, 2536}, {85, 88, 5, 2536}, {86, 89, 5, 2536}, {87, 91, 10, 2536}, {88, 92, 20, 2536}, {89, 93, 10, 2536}, {90, 95, 10, 2536}, {91, 96, 30, 2536}, {92, 97, 30, 2536}, {93, 98, 10, 2536}, {94, 100, 5, 2536}, {95, 101, 20, 2536}, {96, 102, 30, 2536}, {97, 103, 20, 2536}, {98, 104, 5, 2536}, {99, 106, 1, 2536}, {100, 107, 5, 2536}, {101, 108, 10, 2536}, {102, 109, 10, 2536}, {103, 110, 5, 2536}, {104, 111, 1, 2536}, {84, 87, 1, 2537}, {85, 90, 5, 2537}, {86, 91, 5, 2537}, {87, 94, 10, 2537}, {88, 95, 20, 2537}, {89, 96, 10, 2537}, {90, 99, 10, 2537}, {91, 100, 30, 2537}, {92, 101, 30, 2537}, {93, 102, 10, 2537}, {94, 105, 5, 2537}, {95, 106, 20, 2537}, {96, 107, 30, 2537}, {97, 108, 20, 2537}, {98, 109, 5, 2537}, {99, 112, 1, 2537}, {100, 113, 5, 2537}, {101, 114, 10, 2537}, {102, 115, 10, 2537}, {103, 116, 5, 2537}, {104, 117, 1, 2537}, {84, 88, 1, 2538}, {85, 91, 5, 2538}, {86, 92, 5, 2538}, {87, 95, 10, 2538}, {88, 96, 20, 2538}, {89, 97, 10, 2538}, {90, 100, 10, 2538}, {91, 101, 30, 2538}, {92, 102, 30, 2538}, {93, 103, 10, 2538}, {94, 106, 5, 2538}, {95, 107, 20, 2538}, {96, 108, 30, 2538}, {97, 109, 20, 2538}, {98, 110, 5, 2538}, {99, 113, 1, 2538}, {100, 114, 5, 2538}, {101, 115, 10, 2538}, {102, 116, 10, 2538}, {103, 117, 5, 2538}, {104, 118, 1, 2538}, {84, 89, 1, 2539}, {85, 92, 5, 2539}, {86, 93, 5, 2539}, {87, 96, 10, 2539}, {88, 97, 20, 2539}, {89, 98, 10, 2539}, {90, 101, 10, 2539}, {91, 102, 30, 2539}, {92, 103, 30, 2539}, {93, 104, 10, 2539}, {94, 107, 5, 2539}, {95, 108, 20, 2539}, {96, 109, 30, 2539}, {97, 110, 20, 2539}, {98, 111, 5, 2539}, {99, 114, 1, 2539}, {100, 115, 5, 2539}, {101, 116, 10, 2539}, {102, 117, 10, 2539}, {103, 118, 5, 2539}, {104, 119, 1, 2539}, {85, 85, 1, 2540}, {87, 87, 5, 2540}, {88, 88, 5, 2540}, {90, 90, 10, 2540}, {91, 91, 20, 2540}, {92, 92, 10, 2540}, {94, 94, 10, 2540}, {95, 95, 30, 2540}, {96, 96, 30, 2540}, {97, 97, 10, 2540}, {99, 99, 5, 2540}, {100, 100, 20, 2540}, {101, 101, 30, 2540}, {102, 102, 20, 2540}, {103, 103, 5, 2540}, {105, 105, 1, 2540}, {106, 106, 5, 2540}, {107, 107, 10, 2540}, {108, 108, 10, 2540}, {109, 109, 5, 2540}, {110, 110, 1, 2540}, {85, 86, 1, 2541}, {87, 88, 5, 2541}, {88, 89, 5, 2541}, {90, 91, 10, 2541}, {91, 92, 20, 2541}, {92, 93, 10, 2541}, {94, 95, 10, 2541}, {95, 96, 30, 2541}, {96, 97, 30, 2541}, {97, 98, 10, 2541}, {99, 100, 5, 2541}, {100, 101, 20, 2541}, {101, 102, 30, 2541}, {102, 103, 20, 2541}, {103, 104, 5, 2541}, {105, 106, 1, 2541}, {106, 107, 5, 2541}, {107, 108, 10, 2541}, {108, 109, 10, 2541}, {109, 110, 5, 2541}, {110, 111, 1, 2541}, {85, 87, 1, 2542}, {87, 90, 5, 2542}, {88, 91, 5, 2542}, {90, 94, 10, 2542}, {91, 95, 20, 2542}, {92, 96, 10, 2542}, {94, 99, 10, 2542}, {95, 100, 30, 2542}, {96, 101, 30, 2542}, {97, 102, 10, 2542}, {99, 105, 5, 2542}, {100, 106, 20, 2542}, {101,

107, 30, 2542}, {102, 108, 20, 2542}, {103, 109, 5, 2542}, {105, 112, 1, 2542}, {106, 113, 5, 2542}, {107, 114, 10, 2542}, {108, 115, 10, 2542}, {109, 116, 5, 2542}, {110, 117, 1, 2542}, {85, 88, 1, 2543}, {87, 91, 5, 2543}, {88, 92, 5, 2543}, {90, 95, 10, 2543}, {91, 96, 20, 2543}, {92, 97, 10, 2543}, {94, 100, 10, 2543}, {95, 101, 30, 2543}, {96, 102, 30, 2543}, {97, 103, 10, 2543}, {99, 106, 5, 2543}, {100, 107, 20, 2543}, {101, 108, 30, 2543}, {102, 109, 20, 2543}, {103, 110, 5, 2543}, {105, 113, 1, 2543}, {106, 114, 5, 2543}, {107, 115, 10, 2543}, {108, 116, 10, 2543}, {109, 117, 5, 2543}, {110, 118, 1, 2543}, {85, 89, 1, 2544}, {87, 92, 5, 2544}, {88, 93, 5, 2544}, {90, 96, 10, 2544}, {91, 97, 20, 2544}, {92, 98, 10, 2544}, {94, 101, 10, 2544}, {95, 102, 30, 2544}, {96, 103, 30, 2544}, {97, 104, 10, 2544}, {99, 107, 5, 2544}, {100, 108, 20, 2544}, {101, 109, 30, 2544}, {102, 110, 20, 2544}, {103, 111, 5, 2544}, {105, 114, 1, 2544}, {106, 115, 5, 2544}, {107, 116, 10, 2544}, {108, 117, 10, 2544}, {109, 118, 5, 2544}, {110, 119, 1, 2544}, {86, 86, 1, 2545}, {88, 88, 5, 2545}, {89, 89, 5, 2545}, {91, 91, 10, 2545}, {92, 92, 20, 2545}, {93, 93, 10, 2545}, {95, 95, 10, 2545}, {96, 96, 30, 2545}, {97, 97, 30, 2545}, {98, 98, 10, 2545}, {100, 100, 5, 2545}, {101, 101, 20, 2545}, {102, 102, 30, 2545}, {103, 103, 20, 2545}, {104, 104, 5, 2545}, {106, 106, 1, 2545}, {107, 107, 5, 2545}, {108, 108, 10, 2545}, {109, 109, 10, 2545}, {110, 110, 5, 2545}, {111, 111, 1, 2545}, {86, 87, 1, 2546}, {88, 90, 5, 2546}, {89, 91, 5, 2546}, {91, 94, 10, 2546}, {92, 95, 20, 2546}, {93, 96, 10, 2546}, {95, 99, 10, 2546}, {96, 100, 30, 2546}, {97, 101, 30, 2546}, {98, 102, 10, 2546}, {100, 105, 5, 2546}, {101, 106, 20, 2546}, {102, 107, 30, 2546}, {103, 108, 20, 2546}, {104, 109, 5, 2546}, {106, 112, 1, 2546}, {107, 113, 5, 2546}, {108, 114, 10, 2546}, {109, 115, 10, 2546}, {110, 116, 5, 2546}, {111, 117, 1, 2546}, {86, 88, 1, 2547}, {88, 91, 5, 2547}, {89, 92, 5, 2547}, {91, 95, 10, 2547}, {92, 96, 20, 2547}, {93, 97, 10, 2547}, {95, 100, 10, 2547}, {96, 101, 30, 2547}, {97, 102, 30, 2547}, {98, 103, 10, 2547}, {100, 106, 5, 2547}, {101, 107, 20, 2547}, {102, 108, 30, 2547}, {103, 109, 20, 2547}, {104, 110, 5, 2547}, {106, 113, 1, 2547}, {107, 114, 5, 2547}, {108, 115, 10, 2547}, {109, 116, 10, 2547}, {110, 117, 5, 2547}, {111, 118, 1, 2547}, {86, 89, 1, 2548}, {88, 92, 5, 2548}, {89, 93, 5, 2548}, {91, 96, 10, 2548}, {92, 97, 20, 2548}, {93, 98, 10, 2548}, {95, 101, 10, 2548}, {96, 102, 30, 2548}, {97, 103, 30, 2548}, {98, 104, 10, 2548}, {100, 107, 5, 2548}, {101, 108, 20, 2548}, {102, 109, 30, 2548}, {103, 110, 20, 2548}, {104, 111, 5, 2548}, {106, 114, 1, 2548}, {107, 115, 5, 2548}, {108, 116, 10, 2548}, {109, 117, 10, 2548}, {110, 118, 5, 2548}, {111, 119, 1, 2548}, {87, 87, 1, 2549}, {90, 90, 5, 2549}, {91, 91, 5, 2549}, {94, 94, 10, 2549}, {95, 95, 20, 2549}, {96, 96, 10, 2549}, {99, 99, 10, 2549}, {100, 100, 30, 2549}, {101, 101, 30, 2549}, {102, 102, 10, 2549}, {105, 105, 5, 2549}, {106, 106, 20, 2549}, {107, 107, 30, 2549}, {108, 108, 20, 2549}, {109, 109, 5, 2549}, {112, 112, 1, 2549}, {113, 113, 5, 2549}, {114, 114, 10, 2549}, {115, 115, 10, 2549}, {116, 116, 5, 2549}, {117, 117, 1, 2549}, {87, 88, 1, 2550}, {90, 91, 5, 2550}, {91, 92, 5, 2550}, {94, 95, 10, 2550}, {95, 96, 20, 2550}, {96, 97, 10, 2550}, {99, 100, 10, 2550}, {100, 101, 30, 2550}, {101, 102, 30, 2550}, {102, 103, 10, 2550}, {105, 106, 5, 2550}, {106, 107, 20, 2550}, {107, 108, 30, 2550}, {108, 109, 20, 2550}, {109, 110, 5, 2550}, {112, 113, 1, 2550}, {113, 114, 5, 2550}, {114, 115, 10, 2550}, {115, 116, 10, 2550}, {116, 117, 5, 2550}, {117, 118, 1, 2550}, {87, 89, 1, 2551}, {90, 92, 5, 2551}, {91, 93, 5, 2551}, {94, 96, 10, 2551}, {95, 97, 20, 2551}, {96, 98, 10, 2551}, {99, 101, 10, 2551}, {100, 102, 30, 2551}, {101, 103, 30, 2551}, {102, 104, 10, 2551}, {105, 107, 5, 2551}, {106, 108, 20, 2551}, {107, 109, 30, 2551}, {108, 110, 20, 2551}, {109, 111, 5, 2551}, {112, 114, 1, 2551}, {113, 115, 5, 2551}, {114, 116, 10, 2551}, {115, 117, 10, 2551}, {116, 118, 5, 2551}, {117, 119, 1, 2551}, {88, 88, 1, 2552}, {91, 91, 5, 2552}, {92, 92, 5, 2552}, {95, 95, 10, 2552}, {96, 96, 20, 2552}, {97, 97, 10, 2552}, {100, 100, 10, 2552}, {101, 101, 30, 2552}, {102, 102, 30, 2552}, {103, 103, 10, 2552}, {106, 106, 5, 2552}, {107, 107, 20, 2552}, {108, 108, 30, 2552}, {109, 109, 20, 2552}, {110, 110, 5, 2552}, {113, 113, 1, 2552}, {114, 114, 5, 2552}, {115, 115, 10, 2552}, {116, 116, 10, 2552}, {117, 117, 5, 2552}, {118, 118, 1, 2552}, {88, 89, 1, 2553}, {91, 92, 5, 2553}, {92, 93, 5, 2553}, {95, 96, 10, 2553}, {96, 97, 20, 2553}, {97, 98, 10, 2553}, {100, 101, 10, 2553}, {101, 102, 30, 2553}, {102, 103, 30, 2553}, {103, 104, 10, 2553}, {106, 107, 5, 2553}, {107, 108, 20, 2553}, {108, 109, 30, 2553}, {109, 110, 20, 2553}, {110, 111, 5, 2553}, {113, 114, 1, 2553}, {114, 115, 5, 2553}, {115, 116, 10, 2553}, {116, 117, 10, 2553}, {117, 118, 5, 2553}, {118, 119, 1, 2553}, {89, 89, 1, 2554}, {92, 92, 5, 2554}, {93, 93, 5, 2554}, {96, 96, 10, 2554}, {97, 97, 20, 2554}, {98, 98, 10, 2554}, {101, 101, 10, 2554}, {102, 102, 30, 2554}, {103, 103, 30, 2554}, {104, 104, 10, 2554}, {107, 107, 5, 2554}, {108, 108, 20, 2554}, {109, 109, 30, 2554}, {110, 110, 20, 2554}, {111, 111, 5, 2554}, {114, 114, 1, 2554}, {115, 115, 5, 2554}, {116, 116, 10, 2554}, {117, 117, 10, 2554}, {118, 118, 5, 2554}, {119, 119, 1, 2554}, {84, 120, 1, 2555}, {85, 121, 5, 2555}, {86, 122, 5, 2555}, {87, 123, 10, 2555}, {88, 124, 20, 2555}, {89, 125, 10, 2555}, {90, 126, 10, 2555}, {91, 127, 30, 2555}, {92, 128, 30, 2555}, {93, 129, 10, 2555}, {94, 130, 5, 2555}, {95, 131, 20, 2555}, {96, 132, 30, 2555}, {97, 133, 20, 2555}, {98, 134, 5, 2555}, {99, 135, 1, 2555}, {100, 136, 5, 2555}, {101, 137, 10, 2555}, {102, 138, 10, 2555}, {103, 139, 5, 2555}, {104, 140, 1, 2555}, {84, 121, 1, 2556}, {85, 123, 5, 2556}, {86, 124, 5, 2556}, {87, 126, 10, 2556}, {88, 127, 20, 2556}, {89, 128, 10, 2556}, {90, 130, 10, 2556}, {91, 131, 30, 2556}, {92,

132, 30, 2556}, {93, 133, 10, 2556}, {94, 135, 5, 2556}, {95, 136, 20, 2556}, {96, 137, 30, 2556}, {97, 138, 20, 2556}, {98, 139, 5, 2556}, {99, 141, 1, 2556}, {100, 142, 5, 2556}, {101, 143, 10, 2556}, {102, 144, 10, 2556}, {103, 145, 5, 2556}, {104, 146, 1, 2556}, {84, 122, 1, 2557}, {85, 124, 5, 2557}, {86, 125, 5, 2557}, {87, 127, 10, 2557}, {88, 128, 20, 2557}, {89, 129, 10, 2557}, {90, 131, 10, 2557}, {91, 132, 30, 2557}, {92, 133, 30, 2557}, {93, 134, 10, 2557}, {94, 136, 5, 2557}, {95, 137, 20, 2557}, {96, 138, 30, 2557}, {97, 139, 20, 2557}, {98, 140, 5, 2557}, {99, 142, 1, 2557}, {100, 143, 5, 2557}, {101, 144, 10, 2557}, {102, 145, 10, 2557}, {103, 146, 5, 2557}, {104, 147, 1, 2557}, {84, 123, 1, 2558}, {85, 126, 5, 2558}, {86, 127, 5, 2558}, {87, 130, 10, 2558}, {88, 131, 20, 2558}, {89, 132, 10, 2558}, {90, 135, 10, 2558}, {91, 136, 30, 2558}, {92, 137, 30, 2558}, {93, 138, 10, 2558}, {94, 141, 5, 2558}, {95, 142, 20, 2558}, {96, 143, 30, 2558}, {97, 144, 20, 2558}, {98, 145, 5, 2558}, {99, 148, 1, 2558}, {100, 149, 5, 2558}, {101, 150, 10, 2558}, {102, 151, 10, 2558}, {103, 152, 5, 2558}, {104, 153, 1, 2558}, {84, 124, 1, 2559}, {85, 127, 5, 2559}, {86, 128, 5, 2559}, {87, 131, 10, 2559}, {88, 132, 20, 2559}, {89, 133, 10, 2559}, {90, 136, 10, 2559}, {91, 137, 30, 2559}, {92, 138, 30, 2559}, {93, 139, 10, 2559}, {94, 142, 5, 2559}, {95, 143, 20, 2559}, {96, 144, 30, 2559}, {97, 145, 20, 2559}, {98, 146, 5, 2559}, {99, 149, 1, 2559}, {100, 150, 5, 2559}, {101, 151, 10, 2559}, {102, 152, 10, 2559}, {103, 153, 5, 2559}, {104, 154, 1, 2559}, {84, 125, 1, 2560}, {85, 128, 5, 2560}, {86, 129, 5, 2560}, {87, 132, 10, 2560}, {88, 133, 20, 2560}, {89, 134, 10, 2560}, {90, 137, 10, 2560}, {91, 138, 30, 2560}, {92, 139, 30, 2560}, {93, 140, 10, 2560}, {94, 143, 5, 2560}, {95, 144, 20, 2560}, {96, 145, 30, 2560}, {97, 146, 20, 2560}, {98, 147, 5, 2560}, {99, 150, 1, 2560}, {100, 151, 5, 2560}, {101, 152, 10, 2560}, {102, 153, 10, 2560}, {103, 154, 5, 2560}, {104, 155, 1, 2560}, {84, 126, 1, 2561}, {85, 130, 5, 2561}, {86, 131, 5, 2561}, {87, 135, 10, 2561}, {88, 136, 20, 2561}, {89, 137, 10, 2561}, {90, 141, 10, 2561}, {91, 142, 30, 2561}, {92, 143, 30, 2561}, {93, 144, 10, 2561}, {94, 148, 5, 2561}, {95, 149, 20, 2561}, {96, 150, 30, 2561}, {97, 151, 20, 2561}, {98, 152, 5, 2561}, {99, 156, 1, 2561}, {100, 157, 5, 2561}, {101, 158, 10, 2561}, {102, 159, 10, 2561}, {103, 160, 5, 2561}, {104, 161, 1, 2561}, {84, 127, 1, 2562}, {85, 131, 5, 2562}, {86, 132, 5, 2562}, {87, 136, 10, 2562}, {88, 137, 20, 2562}, {89, 138, 10, 2562}, {90, 142, 10, 2562}, {91, 143, 30, 2562}, {92, 144, 30, 2562}, {93, 145, 10, 2562}, {94, 149, 5, 2562}, {95, 150, 20, 2562}, {96, 151, 30, 2562}, {97, 152, 20, 2562}, {98, 153, 5, 2562}, {99, 157, 1, 2562}, {100, 158, 5, 2562}, {101, 159, 10, 2562}, {102, 160, 10, 2562}, {103, 161, 5, 2562}, {104, 162, 1, 2562}, {84, 128, 1, 2563}, {85, 132, 5, 2563}, {86, 133, 5, 2563}, {87, 137, 10, 2563}, {88, 138, 20, 2563}, {89, 139, 10, 2563}, {90, 143, 10, 2563}, {91, 144, 30, 2563}, {92, 145, 30, 2563}, {93, 146, 10, 2563}, {94, 150, 5, 2563}, {95, 151, 20, 2563}, {96, 152, 30, 2563}, {97, 153, 20, 2563}, {98, 154, 5, 2563}, {99, 158, 1, 2563}, {100, 159, 5, 2563}, {101, 160, 10, 2563}, {102, 161, 10, 2563}, {103, 162, 5, 2563}, {104, 163, 1, 2563}, {84, 129, 1, 2564}, {85, 133, 5, 2564}, {86, 134, 5, 2564}, {87, 138, 10, 2564}, {88, 139, 20, 2564}, {89, 140, 10, 2564}, {90, 144, 10, 2564}, {91, 145, 30, 2564}, {92, 146, 30, 2564}, {93, 147, 10, 2564}, {94, 151, 5, 2564}, {95, 152, 20, 2564}, {96, 153, 30, 2564}, {97, 154, 20, 2564}, {98, 155, 5, 2564}, {99, 159, 1, 2564}, {100, 160, 5, 2564}, {101, 161, 10, 2564}, {102, 162, 10, 2564}, {103, 163, 5, 2564}, {104, 164, 1, 2564}, {85, 120, 1, 2565}, {87, 121, 5, 2565}, {88, 122, 5, 2565}, {90, 123, 10, 2565}, {91, 124, 20, 2565}, {92, 125, 10, 2565}, {94, 126, 10, 2565}, {95, 127, 30, 2565}, {96, 128, 30, 2565}, {97, 129, 10, 2565}, {99, 130, 5, 2565}, {100, 131, 20, 2565}, {101, 132, 30, 2565}, {102, 133, 20, 2565}, {103, 134, 5, 2565}, {105, 135, 1, 2565}, {106, 136, 5, 2565}, {107, 137, 10, 2565}, {108, 138, 10, 2565}, {109, 139, 5, 2565}, {110, 140, 1, 2565}, {85, 121, 1, 2566}, {87, 123, 5, 2566}, {88, 124, 5, 2566}, {90, 126, 10, 2566}, {91, 127, 20, 2566}, {92, 128, 10, 2566}, {94, 130, 10, 2566}, {95, 131, 30, 2566}, {96, 132, 30, 2566}, {97, 133, 10, 2566}, {99, 135, 5, 2566}, {100, 136, 20, 2566}, {101, 137, 30, 2566}, {102, 138, 20, 2566}, {103, 139, 5, 2566}, {105, 141, 1, 2566}, {106, 142, 5, 2566}, {107, 143, 10, 2566}, {108, 144, 10, 2566}, {109, 145, 5, 2566}, {110, 146, 1, 2566}, {85, 122, 1, 2567}, {87, 124, 5, 2567}, {88, 125, 5, 2567}, {90, 127, 10, 2567}, {91, 128, 20, 2567}, {92, 129, 10, 2567}, {94, 131, 10, 2567}, {95, 132, 30, 2567}, {96, 133, 30, 2567}, {97, 134, 10, 2567}, {99, 136, 5, 2567}, {100, 137, 20, 2567}, {101, 138, 30, 2567}, {102, 139, 20, 2567}, {103, 140, 5, 2567}, {105, 142, 1, 2567}, {106, 143, 5, 2567}, {107, 144, 10, 2567}, {108, 145, 10, 2567}, {109, 146, 5, 2567}, {110, 147, 1, 2567}, {85, 123, 1, 2568}, {87, 126, 5, 2568}, {88, 127, 5, 2568}, {90, 130, 10, 2568}, {91, 131, 20, 2568}, {92, 132, 10, 2568}, {94, 135, 10, 2568}, {95, 136, 30, 2568}, {96, 137, 30, 2568}, {97, 138, 10, 2568}, {99, 141, 5, 2568}, {100, 142, 20, 2568}, {101, 143, 30, 2568}, {102, 144, 20, 2568}, {103, 145, 5, 2568}, {105, 148, 1, 2568}, {106, 149, 5, 2568}, {107, 150, 10, 2568}, {108, 151, 10, 2568}, {109, 152, 5, 2568}, {110, 153, 1, 2568}, {85, 124, 1, 2569}, {87, 127, 5, 2569}, {88, 128, 5, 2569}, {90, 131, 10, 2569}, {91, 132, 20, 2569}, {92, 133, 10, 2569}, {94, 136, 10, 2569}, {95, 137, 30, 2569}, {96, 138, 30, 2569}, {97, 139, 10, 2569}, {99, 142, 5, 2569}, {100, 143, 20, 2569}, {101, 144, 30, 2569}, {102, 145, 20, 2569}, {103, 146, 5, 2569}, {105, 149, 1, 2569}, {106, 150, 5, 2569}, {107, 151, 10, 2569}, {108, 152, 10, 2569}, {109, 153, 5, 2569}, {110, 154, 1, 2569}, {85, 125, 1, 2570}, {87, 128, 5, 2570}, {88, 129, 5, 2570}, {90, 132, 10, 2570},

{91, 133, 20, 2570}, {92, 134, 10, 2570}, {94, 137, 10, 2570}, {95, 138, 30, 2570}, {96, 139, 30, 2570}, {97, 140, 10, 2570}, {99, 143, 5, 2570}, {100, 144, 20, 2570}, {101, 145, 30, 2570}, {102, 146, 20, 2570}, {103, 147, 5, 2570}, {105, 150, 1, 2570}, {106, 151, 5, 2570}, {107, 152, 10, 2570}, {108, 153, 10, 2570}, {109, 154, 5, 2570}, {110, 155, 1, 2570}, {85, 126, 1, 2571}, {87, 130, 5, 2571}, {88, 131, 5, 2571}, {90, 135, 10, 2571}, {91, 136, 20, 2571}, {92, 137, 10, 2571}, {94, 141, 10, 2571}, {95, 142, 30, 2571}, {96, 143, 30, 2571}, {97, 144, 10, 2571}, {99, 148, 5, 2571}, {100, 149, 20, 2571}, {101, 150, 30, 2571}, {102, 151, 20, 2571}, {103, 152, 5, 2571}, {105, 156, 1, 2571}, {106, 157, 5, 2571}, {107, 158, 10, 2571}, {108, 159, 10, 2571}, {109, 160, 5, 2571}, {110, 161, 1, 2571}, {85, 127, 1, 2572}, {87, 131, 5, 2572}, {88, 132, 5, 2572}, {90, 136, 10, 2572}, {91, 137, 20, 2572}, {92, 138, 10, 2572}, {94, 142, 10, 2572}, {95, 143, 30, 2572}, {96, 144, 30, 2572}, {97, 145, 10, 2572}, {99, 149, 5, 2572}, {100, 150, 20, 2572}, {101, 151, 30, 2572}, {102, 152, 20, 2572}, {103, 153, 5, 2572}, {105, 157, 1, 2572}, {106, 158, 5, 2572}, {107, 159, 10, 2572}, {108, 160, 10, 2572}, {109, 161, 5, 2572}, {110, 162, 1, 2572}, {85, 128, 1, 2573}, {87, 132, 5, 2573}, {88, 133, 5, 2573}, {90, 137, 10, 2573}, {91, 138, 20, 2573}, {92, 139, 10, 2573}, {94, 143, 10, 2573}, {95, 144, 30, 2573}, {96, 145, 30, 2573}, {97, 146, 10, 2573}, {99, 150, 5, 2573}, {100, 151, 20, 2573}, {101, 152, 30, 2573}, {102, 153, 20, 2573}, {103, 154, 5, 2573}, {105, 158, 1, 2573}, {106, 159, 5, 2573}, {107, 160, 10, 2573}, {108, 161, 10, 2573}, {109, 162, 5, 2573}, {110, 163, 1, 2573}, {85, 129, 1, 2574}, {87, 133, 5, 2574}, {88, 134, 5, 2574}, {90, 138, 10, 2574}, {91, 139, 20, 2574}, {92, 140, 10, 2574}, {94, 144, 10, 2574}, {95, 145, 30, 2574}, {96, 146, 30, 2574}, {97, 147, 10, 2574}, {99, 151, 5, 2574}, {100, 152, 20, 2574}, {101, 153, 30, 2574}, {102, 154, 20, 2574}, {103, 155, 5, 2574}, {105, 159, 1, 2574}, {106, 160, 5, 2574}, {107, 161, 10, 2574}, {108, 162, 10, 2574}, {109, 163, 5, 2574}, {110, 164, 1, 2574}, {86, 120, 1, 2575}, {88, 121, 5, 2575}, {89, 122, 5, 2575}, {91, 123, 10, 2575}, {92, 124, 20, 2575}, {93, 125, 10, 2575}, {95, 126, 10, 2575}, {96, 127, 30, 2575}, {97, 128, 30, 2575}, {98, 129, 10, 2575}, {100, 130, 5, 2575}, {101, 131, 20, 2575}, {102, 132, 30, 2575}, {103, 133, 20, 2575}, {104, 134, 5, 2575}, {106, 135, 1, 2575}, {107, 136, 5, 2575}, {108, 137, 10, 2575}, {109, 138, 10, 2575}, {110, 139, 5, 2575}, {111, 140, 1, 2575}, {86, 121, 1, 2576}, {88, 123, 5, 2576}, {89, 124, 5, 2576}, {91, 126, 10, 2576}, {92, 127, 20, 2576}, {93, 128, 10, 2576}, {95, 130, 10, 2576}, {96, 131, 30, 2576}, {97, 132, 30, 2576}, {98, 133, 10, 2576}, {100, 135, 5, 2576}, {101, 136, 20, 2576}, {102, 137, 30, 2576}, {103, 138, 20, 2576}, {104, 139, 5, 2576}, {106, 141, 1, 2576}, {107, 142, 5, 2576}, {108, 143, 10, 2576}, {109, 144, 10, 2576}, {110, 145, 5, 2576}, {111, 146, 1, 2576}, {86, 122, 1, 2577}, {88, 124, 5, 2577}, {89, 125, 5, 2577}, {91, 127, 10, 2577}, {92, 128, 20, 2577}, {93, 129, 10, 2577}, {95, 131, 10, 2577}, {96, 132, 30, 2577}, {97, 133, 30, 2577}, {98, 134, 10, 2577}, {100, 136, 5, 2577}, {101, 137, 20, 2577}, {102, 138, 30, 2577}, {103, 139, 20, 2577}, {104, 140, 5, 2577}, {106, 142, 1, 2577}, {107, 143, 5, 2577}, {108, 144, 10, 2577}, {109, 145, 10, 2577}, {110, 146, 5, 2577}, {111, 147, 1, 2577}, {86, 123, 1, 2578}, {88, 126, 5, 2578}, {89, 127, 5, 2578}, {91, 130, 10, 2578}, {92, 131, 20, 2578}, {93, 132, 10, 2578}, {95, 135, 10, 2578}, {96, 136, 30, 2578}, {97, 137, 30, 2578}, {98, 138, 10, 2578}, {100, 141, 5, 2578}, {101, 142, 20, 2578}, {102, 143, 30, 2578}, {103, 144, 20, 2578}, {104, 145, 5, 2578}, {106, 148, 1, 2578}, {107, 149, 5, 2578}, {108, 150, 10, 2578}, {109, 151, 10, 2578}, {110, 152, 5, 2578}, {111, 153, 1, 2578}, {86, 124, 1, 2579}, {88, 127, 5, 2579}, {89, 128, 5, 2579}, {91, 131, 10, 2579}, {92, 132, 20, 2579}, {93, 133, 10, 2579}, {95, 136, 10, 2579}, {96, 137, 30, 2579}, {97, 138, 30, 2579}, {98, 139, 10, 2579}, {100, 142, 5, 2579}, {101, 143, 20, 2579}, {102, 144, 30, 2579}, {103, 145, 20, 2579}, {104, 146, 5, 2579}, {106, 149, 1, 2579}, {107, 150, 5, 2579}, {108, 151, 10, 2579}, {109, 152, 10, 2579}, {110, 153, 5, 2579}, {111, 154, 1, 2579}, {86, 125, 1, 2580}, {88, 128, 5, 2580}, {89, 129, 5, 2580}, {91, 132, 10, 2580}, {92, 133, 20, 2580}, {93, 134, 10, 2580}, {95, 137, 10, 2580}, {96, 138, 30, 2580}, {97, 139, 30, 2580}, {98, 140, 10, 2580}, {100, 143, 5, 2580}, {101, 144, 20, 2580}, {102, 145, 30, 2580}, {103, 146, 20, 2580}, {104, 147, 5, 2580}, {106, 150, 1, 2580}, {107, 151, 5, 2580}, {108, 152, 10, 2580}, {109, 153, 10, 2580}, {110, 154, 5, 2580}, {111, 155, 1, 2580}, {86, 126, 1, 2581}, {88, 130, 5, 2581}, {89, 131, 5, 2581}, {91, 135, 10, 2581}, {92, 136, 20, 2581}, {93, 137, 10, 2581}, {95, 141, 10, 2581}, {96, 142, 30, 2581}, {97, 143, 30, 2581}, {98, 144, 10, 2581}, {100, 148, 5, 2581}, {101, 149, 20, 2581}, {102, 150, 30, 2581}, {103, 151, 20, 2581}, {104, 152, 5, 2581}, {106, 156, 1, 2581}, {107, 157, 5, 2581}, {108, 158, 10, 2581}, {109, 159, 10, 2581}, {110, 160, 5, 2581}, {111, 161, 1, 2581}, {86, 127, 1, 2582}, {88, 131, 5, 2582}, {89, 132, 5, 2582}, {91, 136, 10, 2582}, {92, 137, 20, 2582}, {93, 138, 10, 2582}, {95, 142, 10, 2582}, {96, 143, 30, 2582}, {97, 144, 30, 2582}, {98, 145, 10, 2582}, {100, 149, 5, 2582}, {101, 150, 20, 2582}, {102, 151, 30, 2582}, {103, 152, 20, 2582}, {104, 153, 5, 2582}, {106, 157, 1, 2582}, {107, 158, 5, 2582}, {108, 159, 10, 2582}, {109, 160, 10, 2582}, {110, 161, 5, 2582}, {111, 162, 1, 2582}, {86, 128, 1, 2583}, {88, 132, 5, 2583}, {89, 133, 5, 2583}, {91, 137, 10, 2583}, {92, 138, 20, 2583}, {93, 139, 10, 2583}, {95, 143, 10, 2583}, {96, 144, 30, 2583}, {97, 145, 30, 2583}, {98, 146, 10, 2583}, {100, 150, 5, 2583}, {101, 151, 20, 2583}, {102, 152, 30, 2583}, {103, 153, 20, 2583}, {104, 154, 5, 2583}, {106, 158, 1, 2583}, {107, 159, 5, 2583}, {108, 160, 10, 2583}, {109, 161, 10, 2583}, {110, 162, 5, 2583}, {111, 163, 1,

2583}, {86, 129, 1, 2584}, {88, 133, 5, 2584}, {89, 134, 5, 2584}, {91, 138, 10, 2584}, {92, 139, 20, 2584}, {93, 140, 10, 2584}, {95, 144, 10, 2584}, {96, 145, 30, 2584}, {97, 146, 30, 2584}, {98, 147, 10, 2584}, {100, 151, 5, 2584}, {101, 152, 20, 2584}, {102, 153, 30, 2584}, {103, 154, 20, 2584}, {104, 155, 5, 2584}, {106, 159, 1, 2584}, {107, 160, 5, 2584}, {108, 161, 10, 2584}, {109, 162, 10, 2584}, {110, 163, 5, 2584}, {111, 164, 1, 2584}, {87, 120, 1, 2585}, {90, 121, 5, 2585}, {91, 122, 5, 2585}, {94, 123, 10, 2585}, {95, 124, 20, 2585}, {96, 125, 10, 2585}, {99, 126, 10, 2585}, {100, 127, 30, 2585}, {101, 128, 30, 2585}, {102, 129, 10, 2585}, {105, 130, 5, 2585}, {106, 131, 20, 2585}, {107, 132, 30, 2585}, {108, 133, 20, 2585}, {109, 134, 5, 2585}, {112, 135, 1, 2585}, {113, 136, 5, 2585}, {114, 137, 10, 2585}, {115, 138, 10, 2585}, {116, 139, 5, 2585}, {117, 140, 1, 2585}, {87, 121, 1, 2586}, {90, 123, 5, 2586}, {91, 124, 5, 2586}, {94, 126, 10, 2586}, {95, 127, 20, 2586}, {96, 128, 10, 2586}, {99, 130, 10, 2586}, {100, 131, 30, 2586}, {101, 132, 30, 2586}, {102, 133, 10, 2586}, {105, 135, 5, 2586}, {106, 136, 20, 2586}, {107, 137, 30, 2586}, {108, 138, 20, 2586}, {109, 139, 5, 2586}, {112, 141, 1, 2586}, {113, 142, 5, 2586}, {114, 143, 10, 2586}, {115, 144, 10, 2586}, {116, 145, 5, 2586}, {117, 146, 1, 2586}, {87, 122, 1, 2587}, {90, 124, 5, 2587}, {91, 125, 5, 2587}, {94, 127, 10, 2587}, {95, 128, 20, 2587}, {96, 129, 10, 2587}, {99, 131, 10, 2587}, {100, 132, 30, 2587}, {101, 133, 30, 2587}, {102, 134, 10, 2587}, {105, 136, 5, 2587}, {106, 137, 20, 2587}, {107, 138, 30, 2587}, {108, 139, 20, 2587}, {109, 140, 5, 2587}, {112, 142, 1, 2587}, {113, 143, 5, 2587}, {114, 144, 10, 2587}, {115, 145, 10, 2587}, {116, 146, 5, 2587}, {117, 147, 1, 2587}, {87, 123, 1, 2588}, {90, 126, 5, 2588}, {91, 127, 5, 2588}, {94, 130, 10, 2588}, {95, 131, 20, 2588}, {96, 132, 10, 2588}, {99, 135, 10, 2588}, {100, 136, 30, 2588}, {101, 137, 30, 2588}, {102, 138, 10, 2588}, {105, 141, 5, 2588}, {106, 142, 20, 2588}, {107, 143, 30, 2588}, {108, 144, 20, 2588}, {109, 145, 5, 2588}, {112, 148, 1, 2588}, {113, 149, 5, 2588}, {114, 150, 10, 2588}, {115, 151, 10, 2588}, {116, 152, 5, 2588}, {117, 153, 1, 2588}, {87, 124, 1, 2589}, {90, 127, 5, 2589}, {91, 128, 5, 2589}, {94, 131, 10, 2589}, {95, 132, 20, 2589}, {96, 133, 10, 2589}, {99, 136, 10, 2589}, {100, 137, 30, 2589}, {101, 138, 30, 2589}, {102, 139, 10, 2589}, {105, 142, 5, 2589}, {106, 143, 20, 2589}, {107, 144, 30, 2589}, {108, 145, 20, 2589}, {109, 146, 5, 2589}, {112, 149, 1, 2589}, {113, 150, 5, 2589}, {114, 151, 10, 2589}, {115, 152, 10, 2589}, {116, 153, 5, 2589}, {117, 154, 1, 2589}, {87, 125, 1, 2590}, {90, 128, 5, 2590}, {91, 129, 5, 2590}, {94, 132, 10, 2590}, {95, 133, 20, 2590}, {96, 134, 10, 2590}, {99, 137, 10, 2590}, {100, 138, 30, 2590}, {101, 139, 30, 2590}, {102, 140, 10, 2590}, {105, 143, 5, 2590}, {106, 144, 20, 2590}, {107, 145, 30, 2590}, {108, 146, 20, 2590}, {109, 147, 5, 2590}, {112, 150, 1, 2590}, {113, 151, 5, 2590}, {114, 152, 10, 2590}, {115, 153, 10, 2590}, {116, 154, 5, 2590}, {117, 155, 1, 2590}, {87, 126, 1, 2591}, {90, 130, 5, 2591}, {91, 131, 5, 2591}, {94, 135, 10, 2591}, {95, 136, 20, 2591}, {96, 137, 10, 2591}, {99, 141, 10, 2591}, {100, 142, 30, 2591}, {101, 143, 30, 2591}, {102, 144, 10, 2591}, {105, 148, 5, 2591}, {106, 149, 20, 2591}, {107, 150, 30, 2591}, {108, 151, 20, 2591}, {109, 152, 5, 2591}, {112, 156, 1, 2591}, {113, 157, 5, 2591}, {114, 158, 10, 2591}, {115, 159, 10, 2591}, {116, 160, 5, 2591}, {117, 161, 1, 2591}, {87, 127, 1, 2592}, {90, 131, 5, 2592}, {91, 132, 5, 2592}, {94, 136, 10, 2592}, {95, 137, 20, 2592}, {96, 138, 10, 2592}, {99, 142, 10, 2592}, {100, 143, 30, 2592}, {101, 144, 30, 2592}, {102, 145, 10, 2592}, {105, 149, 5, 2592}, {106, 150, 20, 2592}, {107, 151, 30, 2592}, {108, 152, 20, 2592}, {109, 153, 5, 2592}, {112, 157, 1, 2592}, {113, 158, 5, 2592}, {114, 159, 10, 2592}, {115, 160, 10, 2592}, {116, 161, 5, 2592}, {117, 162, 1, 2592}, {87, 128, 1, 2593}, {90, 132, 5, 2593}, {91, 133, 5, 2593}, {94, 137, 10, 2593}, {95, 138, 20, 2593}, {96, 139, 10, 2593}, {99, 143, 10, 2593}, {100, 144, 30, 2593}, {101, 145, 30, 2593}, {102, 146, 10, 2593}, {105, 150, 5, 2593}, {106, 151, 20, 2593}, {107, 152, 30, 2593}, {108, 153, 20, 2593}, {109, 154, 5, 2593}, {112, 158, 1, 2593}, {113, 159, 5, 2593}, {114, 160, 10, 2593}, {115, 161, 10, 2593}, {116, 162, 5, 2593}, {117, 163, 1, 2593}, {87, 129, 1, 2594}, {90, 133, 5, 2594}, {91, 134, 5, 2594}, {94, 138, 10, 2594}, {95, 139, 20, 2594}, {96, 140, 10, 2594}, {99, 144, 10, 2594}, {100, 145, 30, 2594}, {101, 146, 30, 2594}, {102, 147, 10, 2594}, {105, 151, 5, 2594}, {106, 152, 20, 2594}, {107, 153, 30, 2594}, {108, 154, 20, 2594}, {109, 155, 5, 2594}, {112, 159, 1, 2594}, {113, 160, 5, 2594}, {114, 161, 10, 2594}, {115, 162, 10, 2594}, {116, 163, 5, 2594}, {117, 164, 1, 2594}, {88, 120, 1, 2595}, {91, 121, 5, 2595}, {92, 122, 5, 2595}, {95, 123, 10, 2595}, {96, 124, 20, 2595}, {97, 125, 10, 2595}, {100, 126, 10, 2595}, {101, 127, 30, 2595}, {102, 128, 30, 2595}, {103, 129, 10, 2595}, {106, 130, 5, 2595}, {107, 131, 20, 2595}, {108, 132, 30, 2595}, {109, 133, 20, 2595}, {110, 134, 5, 2595}, {113, 135, 1, 2595}, {114, 136, 5, 2595}, {115, 137, 10, 2595}, {116, 138, 10, 2595}, {117, 139, 5, 2595}, {118, 140, 1, 2595}, {88, 121, 1, 2596}, {91, 123, 5, 2596}, {92, 124, 5, 2596}, {95, 126, 10, 2596}, {96, 127, 20, 2596}, {97, 128, 10, 2596}, {100, 130, 10, 2596}, {101, 131, 30, 2596}, {102, 132, 30, 2596}, {103, 133, 10, 2596}, {106, 135, 5, 2596}, {107, 136, 20, 2596}, {108, 137, 30, 2596}, {109, 138, 20, 2596}, {110, 139, 5, 2596}, {113, 141, 1, 2596}, {114, 142, 5, 2596}, {115, 143, 10, 2596}, {116, 144, 10, 2596}, {117, 145, 5, 2596}, {118, 146, 1, 2596}, {88, 122, 1, 2597}, {91, 124, 5, 2597}, {92, 125, 5, 2597}, {95, 127, 10, 2597}, {96, 128, 20, 2597}, {97, 129, 10, 2597}, {100, 131, 10, 2597}, {101, 132, 30, 2597}, {102, 133, 30, 2597}, {103, 134, 10, 2597}, {106, 136, 5, 2597}, {107, 137, 20, 2597}, {108, 138, 30, 2597}, {109, 139, 20, 2597}, {110, 140, 5, 2597}, {113,

142, 1, 2597}, {114, 143, 5, 2597}, {115, 144, 10, 2597}, {116, 145, 10, 2597}, {117, 146, 5, 2597}, {118, 147, 1, 2597}, {88, 123, 1, 2598}, {91, 126, 5, 2598}, {92, 127, 5, 2598}, {95, 130, 10, 2598}, {96, 131, 20, 2598}, {97, 132, 10, 2598}, {100, 135, 10, 2598}, {101, 136, 30, 2598}, {102, 137, 30, 2598}, {103, 138, 10, 2598}, {106, 141, 5, 2598}, {107, 142, 20, 2598}, {108, 143, 30, 2598}, {109, 144, 20, 2598}, {110, 145, 5, 2598}, {113, 148, 1, 2598}, {114, 149, 5, 2598}, {115, 150, 10, 2598}, {116, 151, 10, 2598}, {117, 152, 5, 2598}, {118, 153, 1, 2598}, {88, 124, 1, 2599}, {91, 127, 5, 2599}, {92, 128, 5, 2599}, {95, 131, 10, 2599}, {96, 132, 20, 2599}, {97, 133, 10, 2599}, {100, 136, 10, 2599}, {101, 137, 30, 2599}, {102, 138, 30, 2599}, {103, 139, 10, 2599}, {106, 142, 5, 2599}, {107, 143, 20, 2599}, {108, 144, 30, 2599}, {109, 145, 20, 2599}, {110, 146, 5, 2599}, {113, 149, 1, 2599}, {114, 150, 5, 2599}, {115, 151, 10, 2599}, {116, 152, 10, 2599}, {117, 153, 5, 2599}, {118, 154, 1, 2599}, {88, 125, 1, 2600}, {91, 128, 5, 2600}, {92, 129, 5, 2600}, {95, 132, 10, 2600}, {96, 133, 20, 2600}, {97, 134, 10, 2600}, {100, 137, 10, 2600}, {101, 138, 30, 2600}, {102, 139, 30, 2600}, {103, 140, 10, 2600}, {106, 143, 5, 2600}, {107, 144, 20, 2600}, {108, 145, 30, 2600}, {109, 146, 20, 2600}, {110, 147, 5, 2600}, {113, 150, 1, 2600}, {114, 151, 5, 2600}, {115, 152, 10, 2600}, {116, 153, 10, 2600}, {117, 154, 5, 2600}, {118, 155, 1, 2600}, {88, 126, 1, 2601}, {91, 130, 5, 2601}, {92, 131, 5, 2601}, {95, 135, 10, 2601}, {96, 136, 20, 2601}, {97, 137, 10, 2601}, {100, 141, 10, 2601}, {101, 142, 30, 2601}, {102, 143, 30, 2601}, {103, 144, 10, 2601}, {106, 148, 5, 2601}, {107, 149, 20, 2601}, {108, 150, 30, 2601}, {109, 151, 20, 2601}, {110, 152, 5, 2601}, {113, 156, 1, 2601}, {114, 157, 5, 2601}, {115, 158, 10, 2601}, {116, 159, 10, 2601}, {117, 160, 5, 2601}, {118, 161, 1, 2601}, {88, 127, 1, 2602}, {91, 131, 5, 2602}, {92, 132, 5, 2602}, {95, 136, 10, 2602}, {96, 137, 20, 2602}, {97, 138, 10, 2602}, {100, 142, 10, 2602}, {101, 143, 30, 2602}, {102, 144, 30, 2602}, {103, 145, 10, 2602}, {106, 149, 5, 2602}, {107, 150, 20, 2602}, {108, 151, 30, 2602}, {109, 152, 20, 2602}, {110, 153, 5, 2602}, {113, 157, 1, 2602}, {114, 158, 5, 2602}, {115, 159, 10, 2602}, {116, 160, 10, 2602}, {117, 161, 5, 2602}, {118, 162, 1, 2602}, {88, 128, 1, 2603}, {91, 132, 5, 2603}, {92, 133, 5, 2603}, {95, 137, 10, 2603}, {96, 138, 20, 2603}, {97, 139, 10, 2603}, {100, 143, 10, 2603}, {101, 144, 30, 2603}, {102, 145, 30, 2603}, {103, 146, 10, 2603}, {106, 150, 5, 2603}, {107, 151, 20, 2603}, {108, 152, 30, 2603}, {109, 153, 20, 2603}, {110, 154, 5, 2603}, {113, 158, 1, 2603}, {114, 159, 5, 2603}, {115, 160, 10, 2603}, {116, 161, 10, 2603}, {117, 162, 5, 2603}, {118, 163, 1, 2603}, {88, 129, 1, 2604}, {91, 133, 5, 2604}, {92, 134, 5, 2604}, {95, 138, 10, 2604}, {96, 139, 20, 2604}, {97, 140, 10, 2604}, {100, 144, 10, 2604}, {101, 145, 30, 2604}, {102, 146, 30, 2604}, {103, 147, 10, 2604}, {106, 151, 5, 2604}, {107, 152, 20, 2604}, {108, 153, 30, 2604}, {109, 154, 20, 2604}, {110, 155, 5, 2604}, {113, 159, 1, 2604}, {114, 160, 5, 2604}, {115, 161, 10, 2604}, {116, 162, 10, 2604}, {117, 163, 5, 2604}, {118, 164, 1, 2604}, {89, 120, 1, 2605}, {92, 121, 5, 2605}, {93, 122, 5, 2605}, {96, 123, 10, 2605}, {97, 124, 20, 2605}, {98, 125, 10, 2605}, {101, 126, 10, 2605}, {102, 127, 30, 2605}, {103, 128, 30, 2605}, {104, 129, 10, 2605}, {107, 130, 5, 2605}, {108, 131, 20, 2605}, {109, 132, 30, 2605}, {110, 133, 20, 2605}, {111, 134, 5, 2605}, {114, 135, 1, 2605}, {115, 136, 5, 2605}, {116, 137, 10, 2605}, {117, 138, 10, 2605}, {118, 139, 5, 2605}, {119, 140, 1, 2605}, {89, 121, 1, 2606}, {92, 123, 5, 2606}, {93, 124, 5, 2606}, {96, 126, 10, 2606}, {97, 127, 20, 2606}, {98, 128, 10, 2606}, {101, 130, 10, 2606}, {102, 131, 30, 2606}, {103, 132, 30, 2606}, {104, 133, 10, 2606}, {107, 135, 5, 2606}, {108, 136, 20, 2606}, {109, 137, 30, 2606}, {110, 138, 20, 2606}, {111, 139, 5, 2606}, {114, 141, 1, 2606}, {115, 142, 5, 2606}, {116, 143, 10, 2606}, {117, 144, 10, 2606}, {118, 145, 5, 2606}, {119, 146, 1, 2606}, {89, 122, 1, 2607}, {92, 124, 5, 2607}, {93, 125, 5, 2607}, {96, 127, 10, 2607}, {97, 128, 20, 2607}, {98, 129, 10, 2607}, {101, 131, 10, 2607}, {102, 132, 30, 2607}, {103, 133, 30, 2607}, {104, 134, 10, 2607}, {107, 136, 5, 2607}, {108, 137, 20, 2607}, {109, 138, 30, 2607}, {110, 139, 20, 2607}, {111, 140, 5, 2607}, {114, 142, 1, 2607}, {115, 143, 5, 2607}, {116, 144, 10, 2607}, {117, 145, 10, 2607}, {118, 146, 5, 2607}, {119, 147, 1, 2607}, {89, 123, 1, 2608}, {92, 126, 5, 2608}, {93, 127, 5, 2608}, {96, 130, 10, 2608}, {97, 131, 20, 2608}, {98, 132, 10, 2608}, {101, 135, 10, 2608}, {102, 136, 30, 2608}, {103, 137, 30, 2608}, {104, 138, 10, 2608}, {107, 141, 5, 2608}, {108, 142, 20, 2608}, {109, 143, 30, 2608}, {110, 144, 20, 2608}, {111, 145, 5, 2608}, {114, 148, 1, 2608}, {115, 149, 5, 2608}, {116, 150, 10, 2608}, {117, 151, 10, 2608}, {118, 152, 5, 2608}, {119, 153, 1, 2608}, {89, 124, 1, 2609}, {92, 127, 5, 2609}, {93, 128, 5, 2609}, {96, 131, 10, 2609}, {97, 132, 20, 2609}, {98, 133, 10, 2609}, {101, 136, 10, 2609}, {102, 137, 30, 2609}, {103, 138, 30, 2609}, {104, 139, 10, 2609}, {107, 142, 5, 2609}, {108, 143, 20, 2609}, {109, 144, 30, 2609}, {110, 145, 20, 2609}, {111, 146, 5, 2609}, {114, 149, 1, 2609}, {115, 150, 5, 2609}, {116, 151, 10, 2609}, {117, 152, 10, 2609}, {118, 153, 5, 2609}, {119, 154, 1, 2609}, {89, 125, 1, 2610}, {92, 128, 5, 2610}, {93, 129, 5, 2610}, {96, 132, 10, 2610}, {97, 133, 20, 2610}, {98, 134, 10, 2610}, {101, 137, 10, 2610}, {102, 138, 30, 2610}, {103, 139, 30, 2610}, {104, 140, 10, 2610}, {107, 143, 5, 2610}, {108, 144, 20, 2610}, {109, 145, 30, 2610}, {110, 146, 20, 2610}, {111, 147, 5, 2610}, {114, 150, 1, 2610}, {115, 151, 5, 2610}, {116, 152, 10, 2610}, {117, 153, 10, 2610}, {118, 154, 5, 2610}, {119, 155, 1, 2610}, {89, 126, 1, 2611}, {92, 130, 5, 2611}, {93, 131, 5, 2611}, {96, 135, 10, 2611}, {97, 136, 20, 2611}, {98, 137, 10, 2611}, {101, 141, 10, 2611}, {102, 142, 30, 2611}, {103, 143, 30, 2611}, {104, 144, 10, 2611}, {107, 148, 5, 2611}, {108,

149, 20, 2611}, {109, 150, 30, 2611}, {110, 151, 20, 2611}, {111, 152, 5, 2611}, {114, 156, 1, 2611}, {115, 157, 5, 2611}, {116, 158, 10, 2611}, {117, 159, 10, 2611}, {118, 160, 5, 2611}, {119, 161, 1, 2611}, {89, 127, 1, 2612}, {92, 131, 5, 2612}, {93, 132, 5, 2612}, {96, 136, 10, 2612}, {97, 137, 20, 2612}, {98, 138, 10, 2612}, {101, 142, 10, 2612}, {102, 143, 30, 2612}, {103, 144, 30, 2612}, {104, 145, 10, 2612}, {107, 149, 5, 2612}, {108, 150, 20, 2612}, {109, 151, 30, 2612}, {110, 152, 20, 2612}, {111, 153, 5, 2612}, {114, 157, 1, 2612}, {115, 158, 5, 2612}, {116, 159, 10, 2612}, {117, 160, 10, 2612}, {118, 161, 5, 2612}, {119, 162, 1, 2612}, {89, 128, 1, 2613}, {92, 132, 5, 2613}, {93, 133, 5, 2613}, {96, 137, 10, 2613}, {97, 138, 20, 2613}, {98, 139, 10, 2613}, {101, 143, 10, 2613}, {102, 144, 30, 2613}, {103, 145, 30, 2613}, {104, 146, 10, 2613}, {107, 150, 5, 2613}, {108, 151, 20, 2613}, {109, 152, 30, 2613}, {110, 153, 20, 2613}, {111, 154, 5, 2613}, {114, 158, 1, 2613}, {115, 159, 5, 2613}, {116, 160, 10, 2613}, {117, 161, 10, 2613}, {118, 162, 5, 2613}, {119, 163, 1, 2613}, {89, 129, 1, 2614}, {92, 133, 5, 2614}, {93, 134, 5, 2614}, {96, 138, 10, 2614}, {97, 139, 20, 2614}, {98, 140, 10, 2614}, {101, 144, 10, 2614}, {102, 145, 30, 2614}, {103, 146, 30, 2614}, {104, 147, 10, 2614}, {107, 151, 5, 2614}, {108, 152, 20, 2614}, {109, 153, 30, 2614}, {110, 154, 20, 2614}, {111, 155, 5, 2614}, {114, 159, 1, 2614}, {115, 160, 5, 2614}, {116, 161, 10, 2614}, {117, 162, 10, 2614}, {118, 163, 5, 2614}, {119, 164, 1, 2614}, {84, 84, 1, 2615}, {85, 85, 6, 2615}, {86, 86, 6, 2615}, {87, 87, 15, 2615}, {88, 88, 30, 2615}, {89, 89, 15, 2615}, {90, 90, 20, 2615}, {91, 91, 60, 2615}, {92, 92, 60, 2615}, {93, 93, 20, 2615}, {94, 94, 15, 2615}, {95, 95, 60, 2615}, {96, 96, 90, 2615}, {97, 97, 60, 2615}, {98, 98, 15, 2615}, {99, 99, 6, 2615}, {100, 100, 30, 2615}, {101, 101, 60, 2615}, {102, 102, 60, 2615}, {103, 103, 30, 2615}, {104, 104, 6, 2615}, {105, 105, 1, 2615}, {106, 106, 6, 2615}, {107, 107, 15, 2615}, {108, 108, 20, 2615}, {109, 109, 15, 2615}, {110, 110, 6, 2615}, {111, 111, 1, 2615}, {84, 85, 1, 2616}, {85, 87, 6, 2616}, {86, 88, 6, 2616}, {87, 90, 15, 2616}, {88, 91, 30, 2616}, {89, 92, 15, 2616}, {90, 94, 20, 2616}, {91, 95, 60, 2616}, {92, 96, 60, 2616}, {93, 97, 20, 2616}, {94, 99, 15, 2616}, {95, 100, 60, 2616}, {96, 101, 90, 2616}, {97, 102, 60, 2616}, {98, 103, 15, 2616}, {99, 105, 6, 2616}, {100, 106, 30, 2616}, {101, 107, 60, 2616}, {102, 108, 60, 2616}, {103, 109, 30, 2616}, {104, 110, 6, 2616}, {105, 112, 1, 2616}, {106, 113, 6, 2616}, {107, 114, 15, 2616}, {108, 115, 20, 2616}, {109, 116, 15, 2616}, {110, 117, 6, 2616}, {111, 118, 1, 2616}, {84, 86, 1, 2617}, {85, 88, 6, 2617}, {86, 89, 6, 2617}, {87, 91, 15, 2617}, {88, 92, 30, 2617}, {89, 93, 15, 2617}, {90, 95, 20, 2617}, {91, 96, 60, 2617}, {92, 97, 60, 2617}, {93, 98, 20, 2617}, {94, 100, 15, 2617}, {95, 101, 60, 2617}, {96, 102, 90, 2617}, {97, 103, 60, 2617}, {98, 104, 15, 2617}, {99, 106, 6, 2617}, {100, 107, 30, 2617}, {101, 108, 60, 2617}, {102, 109, 60, 2617}, {103, 110, 30, 2617}, {104, 111, 6, 2617}, {105, 113, 1, 2617}, {106, 114, 6, 2617}, {107, 115, 15, 2617}, {108, 116, 20, 2617}, {109, 117, 15, 2617}, {110, 118, 6, 2617}, {111, 119, 1, 2617}, {85, 85, 1, 2618}, {87, 87, 6, 2618}, {88, 88, 6, 2618}, {90, 90, 15, 2618}, {91, 91, 30, 2618}, {92, 92, 15, 2618}, {94, 94, 20, 2618}, {95, 95, 60, 2618}, {96, 96, 60, 2618}, {97, 97, 20, 2618}, {99, 99, 15, 2618}, {100, 100, 60, 2618}, {101, 101, 90, 2618}, {102, 102, 60, 2618}, {103, 103, 15, 2618}, {105, 105, 6, 2618}, {106, 106, 30, 2618}, {107, 107, 60, 2618}, {108, 108, 60, 2618}, {109, 109, 30, 2618}, {110, 110, 6, 2618}, {112, 112, 1, 2618}, {113, 113, 6, 2618}, {114, 114, 15, 2618}, {115, 115, 20, 2618}, {116, 116, 15, 2618}, {117, 117, 6, 2618}, {118, 118, 1, 2618}, {85, 86, 1, 2619}, {87, 88, 6, 2619}, {88, 89, 6, 2619}, {90, 91, 15, 2619}, {91, 92, 30, 2619}, {92, 93, 15, 2619}, {94, 95, 20, 2619}, {95, 96, 60, 2619}, {96, 97, 60, 2619}, {97, 98, 20, 2619}, {99, 100, 15, 2619}, {100, 101, 60, 2619}, {101, 102, 90, 2619}, {102, 103, 60, 2619}, {103, 104, 15, 2619}, {105, 106, 6, 2619}, {106, 107, 30, 2619}, {107, 108, 60, 2619}, {108, 109, 60, 2619}, {109, 110, 30, 2619}, {110, 111, 6, 2619}, {112, 113, 1, 2619}, {113, 114, 6, 2619}, {114, 115, 15, 2619}, {115, 116, 20, 2619}, {116, 117, 15, 2619}, {117, 118, 6, 2619}, {118, 119, 1, 2619}, {86, 86, 1, 2620}, {88, 88, 6, 2620}, {89, 89, 6, 2620}, {91, 91, 15, 2620}, {92, 92, 30, 2620}, {93, 93, 15, 2620}, {95, 95, 20, 2620}, {96, 96, 60, 2620}, {97, 97, 60, 2620}, {98, 98, 20, 2620}, {100, 100, 15, 2620}, {101, 101, 60, 2620}, {102, 102, 90, 2620}, {103, 103, 60, 2620}, {104, 104, 15, 2620}, {106, 106, 6, 2620}, {107, 107, 30, 2620}, {108, 108, 60, 2620}, {109, 109, 60, 2620}, {110, 110, 30, 2620}, {111, 111, 6, 2620}, {113, 113, 1, 2620}, {114, 114, 6, 2620}, {115, 115, 15, 2620}, {116, 116, 20, 2620}, {117, 117, 15, 2620}, {118, 118, 6, 2620}, {119, 119, 1, 2620}, {84, 120, 1, 2621}, {85, 121, 6, 2621}, {86, 122, 6, 2621}, {87, 123, 15, 2621}, {88, 124, 30, 2621}, {89, 125, 15, 2621}, {90, 126, 20, 2621}, {91, 127, 60, 2621}, {92, 128, 60, 2621}, {93, 129, 20, 2621}, {94, 130, 15, 2621}, {95, 131, 60, 2621}, {96, 132, 90, 2621}, {97, 133, 60, 2621}, {98, 134, 15, 2621}, {99, 135, 6, 2621}, {100, 136, 30, 2621}, {101, 137, 60, 2621}, {102, 138, 60, 2621}, {103, 139, 30, 2621}, {104, 140, 6, 2621}, {105, 141, 1, 2621}, {106, 142, 6, 2621}, {107, 143, 15, 2621}, {108, 144, 20, 2621}, {109, 145, 15, 2621}, {110, 146, 6, 2621}, {111, 147, 1, 2621}, {84, 121, 1, 2622}, {85, 123, 6, 2622}, {86, 124, 6, 2622}, {87, 126, 15, 2622}, {88, 127, 30, 2622}, {89, 128, 15, 2622}, {90, 130, 20, 2622}, {91, 131, 60, 2622}, {92, 132, 60, 2622}, {93, 133, 20, 2622}, {94, 135, 15, 2622}, {95, 136, 60, 2622}, {96, 137, 90, 2622}, {97, 138, 60, 2622}, {98, 139, 15, 2622}, {99, 141, 6, 2622}, {100, 142, 30, 2622}, {101, 143, 60, 2622}, {102, 144, 60, 2622}, {103, 145, 30, 2622}, {104, 146, 6, 2622}, {105, 148, 1, 2622}, {106, 149, 6,

2622}, {107, 150, 15, 2622}, {108, 151, 20, 2622}, {109, 152, 15, 2622}, {110, 153, 6, 2622}, {111, 154, 1, 2622}, {84, 122, 1, 2623}, {85, 124, 6, 2623}, {86, 125, 6, 2623}, {87, 127, 15, 2623}, {88, 128, 30, 2623}, {89, 129, 15, 2623}, {90, 131, 20, 2623}, {91, 132, 60, 2623}, {92, 133, 60, 2623}, {93, 134, 20, 2623}, {94, 136, 15, 2623}, {95, 137, 60, 2623}, {96, 138, 90, 2623}, {97, 139, 60, 2623}, {98, 140, 15, 2623}, {99, 142, 6, 2623}, {100, 143, 30, 2623}, {101, 144, 60, 2623}, {102, 145, 60, 2623}, {103, 146, 30, 2623}, {104, 147, 6, 2623}, {105, 149, 1, 2623}, {106, 150, 6, 2623}, {107, 151, 15, 2623}, {108, 152, 20, 2623}, {109, 153, 15, 2623}, {110, 154, 6, 2623}, {111, 155, 1, 2623}, {84, 123, 1, 2624}, {85, 126, 6, 2624}, {86, 127, 6, 2624}, {87, 130, 15, 2624}, {88, 131, 30, 2624}, {89, 132, 15, 2624}, {90, 135, 20, 2624}, {91, 136, 60, 2624}, {92, 137, 60, 2624}, {93, 138, 20, 2624}, {94, 141, 15, 2624}, {95, 142, 60, 2624}, {96, 143, 90, 2624}, {97, 144, 60, 2624}, {98, 145, 15, 2624}, {99, 148, 6, 2624}, {100, 149, 30, 2624}, {101, 150, 60, 2624}, {102, 151, 60, 2624}, {103, 152, 30, 2624}, {104, 153, 6, 2624}, {105, 156, 1, 2624}, {106, 157, 6, 2624}, {107, 158, 15, 2624}, {108, 159, 20, 2624}, {109, 160, 15, 2624}, {110, 161, 6, 2624}, {111, 162, 1, 2624}, {84, 124, 1, 2625}, {85, 127, 6, 2625}, {86, 128, 6, 2625}, {87, 131, 15, 2625}, {88, 132, 30, 2625}, {89, 133, 15, 2625}, {90, 136, 20, 2625}, {91, 137, 60, 2625}, {92, 138, 60, 2625}, {93, 139, 20, 2625}, {94, 142, 15, 2625}, {95, 143, 60, 2625}, {96, 144, 90, 2625}, {97, 145, 60, 2625}, {98, 146, 15, 2625}, {99, 149, 6, 2625}, {100, 150, 30, 2625}, {101, 151, 60, 2625}, {102, 152, 60, 2625}, {103, 153, 30, 2625}, {104, 154, 6, 2625}, {105, 157, 1, 2625}, {106, 158, 6, 2625}, {107, 159, 15, 2625}, {108, 160, 20, 2625}, {109, 161, 15, 2625}, {110, 162, 6, 2625}, {111, 163, 1, 2625}, {84, 125, 1, 2626}, {85, 128, 6, 2626}, {86, 129, 6, 2626}, {87, 132, 15, 2626}, {88, 133, 30, 2626}, {89, 134, 15, 2626}, {90, 137, 20, 2626}, {91, 138, 60, 2626}, {92, 139, 60, 2626}, {93, 140, 20, 2626}, {94, 143, 15, 2626}, {95, 144, 60, 2626}, {96, 145, 90, 2626}, {97, 146, 60, 2626}, {98, 147, 15, 2626}, {99, 150, 6, 2626}, {100, 151, 30, 2626}, {101, 152, 60, 2626}, {102, 153, 60, 2626}, {103, 154, 30, 2626}, {104, 155, 6, 2626}, {105, 158, 1, 2626}, {106, 159, 6, 2626}, {107, 160, 15, 2626}, {108, 161, 20, 2626}, {109, 162, 15, 2626}, {110, 163, 6, 2626}, {111, 164, 1, 2626}, {85, 120, 1, 2627}, {87, 121, 6, 2627}, {88, 122, 6, 2627}, {90, 123, 15, 2627}, {91, 124, 30, 2627}, {92, 125, 15, 2627}, {94, 126, 20, 2627}, {95, 127, 60, 2627}, {96, 128, 60, 2627}, {97, 129, 20, 2627}, {99, 130, 15, 2627}, {100, 131, 60, 2627}, {101, 132, 90, 2627}, {102, 133, 60, 2627}, {103, 134, 15, 2627}, {105, 135, 6, 2627}, {106, 136, 30, 2627}, {107, 137, 60, 2627}, {108, 138, 60, 2627}, {109, 139, 30, 2627}, {110, 140, 6, 2627}, {112, 141, 1, 2627}, {113, 142, 6, 2627}, {114, 143, 15, 2627}, {115, 144, 20, 2627}, {116, 145, 15, 2627}, {117, 146, 6, 2627}, {118, 147, 1, 2627}, {85, 121, 1, 2628}, {87, 123, 6, 2628}, {88, 124, 6, 2628}, {90, 126, 15, 2628}, {91, 127, 30, 2628}, {92, 128, 15, 2628}, {94, 130, 20, 2628}, {95, 131, 60, 2628}, {96, 132, 60, 2628}, {97, 133, 20, 2628}, {99, 135, 15, 2628}, {100, 136, 60, 2628}, {101, 137, 90, 2628}, {102, 138, 60, 2628}, {103, 139, 15, 2628}, {105, 141, 6, 2628}, {106, 142, 30, 2628}, {107, 143, 60, 2628}, {108, 144, 60, 2628}, {109, 145, 30, 2628}, {110, 146, 6, 2628}, {112, 148, 1, 2628}, {113, 149, 6, 2628}, {114, 150, 15, 2628}, {115, 151, 20, 2628}, {116, 152, 15, 2628}, {117, 153, 6, 2628}, {118, 154, 1, 2628}, {85, 122, 1, 2629}, {87, 124, 6, 2629}, {88, 125, 6, 2629}, {90, 127, 15, 2629}, {91, 128, 30, 2629}, {92, 129, 15, 2629}, {94, 131, 20, 2629}, {95, 132, 60, 2629}, {96, 133, 60, 2629}, {97, 134, 20, 2629}, {99, 136, 15, 2629}, {100, 137, 60, 2629}, {101, 138, 90, 2629}, {102, 139, 60, 2629}, {103, 140, 15, 2629}, {105, 142, 6, 2629}, {106, 143, 30, 2629}, {107, 144, 60, 2629}, {108, 145, 60, 2629}, {109, 146, 30, 2629}, {110, 147, 6, 2629}, {112, 149, 1, 2629}, {113, 150, 6, 2629}, {114, 151, 15, 2629}, {115, 152, 20, 2629}, {116, 153, 15, 2629}, {117, 154, 6, 2629}, {118, 155, 1, 2629}, {85, 123, 1, 2630}, {87, 126, 6, 2630}, {88, 127, 6, 2630}, {90, 130, 15, 2630}, {91, 131, 30, 2630}, {92, 132, 15, 2630}, {94, 135, 20, 2630}, {95, 136, 60, 2630}, {96, 137, 60, 2630}, {97, 138, 20, 2630}, {99, 141, 15, 2630}, {100, 142, 60, 2630}, {101, 143, 90, 2630}, {102, 144, 60, 2630}, {103, 145, 15, 2630}, {105, 148, 6, 2630}, {106, 149, 30, 2630}, {107, 150, 60, 2630}, {108, 151, 60, 2630}, {109, 152, 30, 2630}, {110, 153, 6, 2630}, {112, 156, 1, 2630}, {113, 157, 6, 2630}, {114, 158, 15, 2630}, {115, 159, 20, 2630}, {116, 160, 15, 2630}, {117, 161, 6, 2630}, {118, 162, 1, 2630}, {85, 124, 1, 2631}, {87, 127, 6, 2631}, {88, 128, 6, 2631}, {90, 131, 15, 2631}, {91, 132, 30, 2631}, {92, 133, 15, 2631}, {94, 136, 20, 2631}, {95, 137, 60, 2631}, {96, 138, 60, 2631}, {97, 139, 20, 2631}, {99, 142, 15, 2631}, {100, 143, 60, 2631}, {101, 144, 90, 2631}, {102, 145, 60, 2631}, {103, 146, 15, 2631}, {105, 149, 6, 2631}, {106, 150, 30, 2631}, {107, 151, 60, 2631}, {108, 152, 60, 2631}, {109, 153, 30, 2631}, {110, 154, 6, 2631}, {112, 157, 1, 2631}, {113, 158, 6, 2631}, {114, 159, 15, 2631}, {115, 160, 20, 2631}, {116, 161, 15, 2631}, {117, 162, 6, 2631}, {118, 163, 1, 2631}, {85, 125, 1, 2632}, {87, 128, 6, 2632}, {88, 129, 6, 2632}, {90, 132, 15, 2632}, {91, 133, 30, 2632}, {92, 134, 15, 2632}, {94, 137, 20, 2632}, {95, 138, 60, 2632}, {96, 139, 60, 2632}, {97, 140, 20, 2632}, {99, 143, 15, 2632}, {100, 144, 60, 2632}, {101, 145, 90, 2632}, {102, 146, 60, 2632}, {103, 147, 15, 2632}, {105, 150, 6, 2632}, {106, 151, 30, 2632}, {107, 152, 60, 2632}, {108, 153, 60, 2632}, {109, 154, 30, 2632}, {110, 155, 6, 2632}, {112, 158, 1, 2632}, {113, 159, 6, 2632}, {114, 160, 15, 2632}, {115, 161, 20, 2632}, {116, 162, 15, 2632}, {117, 163, 6, 2632}, {118, 164, 1, 2632}, {86, 120, 1, 2633},

{88, 121, 6, 2633}, {89, 122, 6, 2633}, {91, 123, 15, 2633}, {92, 124, 30, 2633}, {93, 125, 15, 2633}, {95, 126, 20, 2633}, {96, 127, 60, 2633}, {97, 128, 60, 2633}, {98, 129, 20, 2633}, {100, 130, 15, 2633}, {101, 131, 60, 2633}, {102, 132, 90, 2633}, {103, 133, 60, 2633}, {104, 134, 15, 2633}, {106, 135, 6, 2633}, {107, 136, 30, 2633}, {108, 137, 60, 2633}, {109, 138, 60, 2633}, {110, 139, 30, 2633}, {111, 140, 6, 2633}, {113, 141, 1, 2633}, {114, 142, 6, 2633}, {115, 143, 15, 2633}, {116, 144, 20, 2633}, {117, 145, 15, 2633}, {118, 146, 6, 2633}, {119, 147, 1, 2633}, {86, 121, 1, 2634}, {88, 123, 6, 2634}, {89, 124, 6, 2634}, {91, 126, 15, 2634}, {92, 127, 30, 2634}, {93, 128, 15, 2634}, {95, 130, 20, 2634}, {96, 131, 60, 2634}, {97, 132, 60, 2634}, {98, 133, 20, 2634}, {100, 135, 15, 2634}, {101, 136, 60, 2634}, {102, 137, 90, 2634}, {103, 138, 60, 2634}, {104, 139, 15, 2634}, {106, 141, 6, 2634}, {107, 142, 30, 2634}, {108, 143, 60, 2634}, {109, 144, 60, 2634}, {110, 145, 30, 2634}, {111, 146, 6, 2634}, {113, 148, 1, 2634}, {114, 149, 6, 2634}, {115, 150, 15, 2634}, {116, 151, 20, 2634}, {117, 152, 15, 2634}, {118, 153, 6, 2634}, {119, 154, 1, 2634}, {86, 122, 1, 2635}, {88, 124, 6, 2635}, {89, 125, 6, 2635}, {91, 127, 15, 2635}, {92, 128, 30, 2635}, {93, 129, 15, 2635}, {95, 131, 20, 2635}, {96, 132, 60, 2635}, {97, 133, 60, 2635}, {98, 134, 20, 2635}, {100, 136, 15, 2635}, {101, 137, 60, 2635}, {102, 138, 90, 2635}, {103, 139, 60, 2635}, {104, 140, 15, 2635}, {106, 142, 6, 2635}, {107, 143, 30, 2635}, {108, 144, 60, 2635}, {109, 145, 60, 2635}, {110, 146, 30, 2635}, {111, 147, 6, 2635}, {113, 149, 1, 2635}, {114, 150, 6, 2635}, {115, 151, 15, 2635}, {116, 152, 20, 2635}, {117, 153, 15, 2635}, {118, 154, 6, 2635}, {119, 155, 1, 2635}, {86, 123, 1, 2636}, {88, 126, 6, 2636}, {89, 127, 6, 2636}, {91, 130, 15, 2636}, {92, 131, 30, 2636}, {93, 132, 15, 2636}, {95, 135, 20, 2636}, {96, 136, 60, 2636}, {97, 137, 60, 2636}, {98, 138, 20, 2636}, {100, 141, 15, 2636}, {101, 142, 60, 2636}, {102, 143, 90, 2636}, {103, 144, 60, 2636}, {104, 145, 15, 2636}, {106, 148, 6, 2636}, {107, 149, 30, 2636}, {108, 150, 60, 2636}, {109, 151, 60, 2636}, {110, 152, 30, 2636}, {111, 153, 6, 2636}, {113, 156, 1, 2636}, {114, 157, 6, 2636}, {115, 158, 15, 2636}, {116, 159, 20, 2636}, {117, 160, 15, 2636}, {118, 161, 6, 2636}, {119, 162, 1, 2636}, {86, 124, 1, 2637}, {88, 127, 6, 2637}, {89, 128, 6, 2637}, {91, 131, 15, 2637}, {92, 132, 30, 2637}, {93, 133, 15, 2637}, {95, 136, 20, 2637}, {96, 137, 60, 2637}, {97, 138, 60, 2637}, {98, 139, 20, 2637}, {100, 142, 15, 2637}, {101, 143, 60, 2637}, {102, 144, 90, 2637}, {103, 145, 60, 2637}, {104, 146, 15, 2637}, {106, 149, 6, 2637}, {107, 150, 30, 2637}, {108, 151, 60, 2637}, {109, 152, 60, 2637}, {110, 153, 30, 2637}, {111, 154, 6, 2637}, {113, 157, 1, 2637}, {114, 158, 6, 2637}, {115, 159, 15, 2637}, {116, 160, 20, 2637}, {117, 161, 15, 2637}, {118, 162, 6, 2637}, {119, 163, 1, 2637}, {86, 125, 1, 2638}, {88, 128, 6, 2638}, {89, 129, 6, 2638}, {91, 132, 15, 2638}, {92, 133, 30, 2638}, {93, 134, 15, 2638}, {95, 137, 20, 2638}, {96, 138, 60, 2638}, {97, 139, 60, 2638}, {98, 140, 20, 2638}, {100, 143, 15, 2638}, {101, 144, 60, 2638}, {102, 145, 90, 2638}, {103, 146, 60, 2638}, {104, 147, 15, 2638}, {106, 150, 6, 2638}, {107, 151, 30, 2638}, {108, 152, 60, 2638}, {109, 153, 60, 2638}, {110, 154, 30, 2638}, {111, 155, 6, 2638}, {113, 158, 1, 2638}, {114, 159, 6, 2638}, {115, 160, 15, 2638}, {116, 161, 20, 2638}, {117, 162, 15, 2638}, {118, 163, 6, 2638}, {119, 164, 1, 2638}, {84, 165, 1, 2639}, {85, 166, 6, 2639}, {86, 167, 6, 2639}, {87, 168, 15, 2639}, {88, 169, 30, 2639}, {89, 170, 15, 2639}, {90, 171, 20, 2639}, {91, 172, 60, 2639}, {92, 173, 60, 2639}, {93, 174, 20, 2639}, {94, 175, 15, 2639}, {95, 176, 60, 2639}, {96, 177, 90, 2639}, {97, 178, 60, 2639}, {98, 179, 15, 2639}, {99, 180, 6, 2639}, {100, 181, 30, 2639}, {101, 182, 60, 2639}, {102, 183, 60, 2639}, {103, 184, 30, 2639}, {104, 185, 6, 2639}, {105, 186, 1, 2639}, {106, 187, 6, 2639}, {107, 188, 15, 2639}, {108, 189, 20, 2639}, {109, 190, 15, 2639}, {110, 191, 6, 2639}, {111, 192, 1, 2639}, {84, 166, 1, 2640}, {85, 168, 6, 2640}, {86, 169, 6, 2640}, {87, 171, 15, 2640}, {88, 172, 30, 2640}, {89, 173, 15, 2640}, {90, 175, 20, 2640}, {91, 176, 60, 2640}, {92, 177, 60, 2640}, {93, 178, 20, 2640}, {94, 180, 15, 2640}, {95, 181, 60, 2640}, {96, 182, 90, 2640}, {97, 183, 60, 2640}, {98, 184, 15, 2640}, {99, 186, 6, 2640}, {100, 187, 30, 2640}, {101, 188, 60, 2640}, {102, 189, 60, 2640}, {103, 190, 30, 2640}, {104, 191, 6, 2640}, {105, 193, 1, 2640}, {106, 194, 6, 2640}, {107, 195, 15, 2640}, {108, 196, 20, 2640}, {109, 197, 15, 2640}, {110, 198, 6, 2640}, {111, 199, 1, 2640}, {84, 167, 1, 2641}, {85, 169, 6, 2641}, {86, 170, 6, 2641}, {87, 172, 15, 2641}, {88, 173, 30, 2641}, {89, 174, 15, 2641}, {90, 176, 20, 2641}, {91, 177, 60, 2641}, {92, 178, 60, 2641}, {93, 179, 20, 2641}, {94, 181, 15, 2641}, {95, 182, 60, 2641}, {96, 183, 90, 2641}, {97, 184, 60, 2641}, {98, 185, 15, 2641}, {99, 187, 6, 2641}, {100, 188, 30, 2641}, {101, 189, 60, 2641}, {102, 190, 60, 2641}, {103, 191, 30, 2641}, {104, 192, 6, 2641}, {105, 194, 1, 2641}, {106, 195, 6, 2641}, {107, 196, 15, 2641}, {108, 197, 20, 2641}, {109, 198, 15, 2641}, {110, 199, 6, 2641}, {111, 200, 1, 2641}, {84, 168, 1, 2642}, {85, 171, 6, 2642}, {86, 172, 6, 2642}, {87, 175, 15, 2642}, {88, 176, 30, 2642}, {89, 177, 15, 2642}, {90, 180, 20, 2642}, {91, 181, 60, 2642}, {92, 182, 60, 2642}, {93, 183, 20, 2642}, {94, 186, 15, 2642}, {95, 187, 60, 2642}, {96, 188, 90, 2642}, {97, 189, 60, 2642}, {98, 190, 15, 2642}, {99, 193, 6, 2642}, {100, 194, 30, 2642}, {101, 195, 60, 2642}, {102, 196, 60, 2642}, {103, 197, 30, 2642}, {104, 198, 6, 2642}, {105, 201, 1, 2642}, {106, 202, 6, 2642}, {107, 203, 15, 2642}, {108, 204, 20, 2642}, {109, 205, 15, 2642}, {110, 206, 6, 2642}, {111, 207, 1, 2642}, {84, 169, 1, 2643}, {85, 172, 6, 2643}, {86, 173, 6, 2643}, {87, 176, 15, 2643}, {88, 177, 30, 2643}, {89, 178, 15, 2643}, {90, 181, 20, 2643}, {91,

182, 60, 2643}, {92, 183, 60, 2643}, {93, 184, 20, 2643}, {94, 187, 15, 2643}, {95, 188, 60, 2643}, {96, 189, 90, 2643}, {97, 190, 60, 2643}, {98, 191, 15, 2643}, {99, 194, 6, 2643}, {100, 195, 30, 2643}, {101, 196, 60, 2643}, {102, 197, 60, 2643}, {103, 198, 30, 2643}, {104, 199, 6, 2643}, {105, 202, 1, 2643}, {106, 203, 6, 2643}, {107, 204, 15, 2643}, {108, 205, 20, 2643}, {109, 206, 15, 2643}, {110, 207, 6, 2643}, {111, 208, 1, 2643}, {84, 170, 1, 2644}, {85, 173, 6, 2644}, {86, 174, 6, 2644}, {87, 177, 15, 2644}, {88, 178, 30, 2644}, {89, 179, 15, 2644}, {90, 182, 20, 2644}, {91, 183, 60, 2644}, {92, 184, 60, 2644}, {93, 185, 20, 2644}, {94, 188, 15, 2644}, {95, 189, 60, 2644}, {96, 190, 90, 2644}, {97, 191, 60, 2644}, {98, 192, 15, 2644}, {99, 195, 6, 2644}, {100, 196, 30, 2644}, {101, 197, 60, 2644}, {102, 198, 60, 2644}, {103, 199, 30, 2644}, {104, 200, 6, 2644}, {105, 203, 1, 2644}, {106, 204, 6, 2644}, {107, 205, 15, 2644}, {108, 206, 20, 2644}, {109, 207, 15, 2644}, {110, 208, 6, 2644}, {111, 209, 1, 2644}, {84, 171, 1, 2645}, {85, 175, 6, 2645}, {86, 176, 6, 2645}, {87, 180, 15, 2645}, {88, 181, 30, 2645}, {89, 182, 15, 2645}, {90, 186, 20, 2645}, {91, 187, 60, 2645}, {92, 188, 60, 2645}, {93, 189, 20, 2645}, {94, 193, 15, 2645}, {95, 194, 60, 2645}, {96, 195, 90, 2645}, {97, 196, 60, 2645}, {98, 197, 15, 2645}, {99, 201, 6, 2645}, {100, 202, 30, 2645}, {101, 203, 60, 2645}, {102, 204, 60, 2645}, {103, 205, 30, 2645}, {104, 206, 6, 2645}, {105, 210, 1, 2645}, {106, 211, 6, 2645}, {107, 212, 15, 2645}, {108, 213, 20, 2645}, {109, 214, 15, 2645}, {110, 215, 6, 2645}, {111, 216, 1, 2645}, {84, 172, 1, 2646}, {85, 176, 6, 2646}, {86, 177, 6, 2646}, {87, 181, 15, 2646}, {88, 182, 30, 2646}, {89, 183, 15, 2646}, {90, 187, 20, 2646}, {91, 188, 60, 2646}, {92, 189, 60, 2646}, {93, 190, 20, 2646}, {94, 194, 15, 2646}, {95, 195, 60, 2646}, {96, 196, 90, 2646}, {97, 197, 60, 2646}, {98, 198, 15, 2646}, {99, 202, 6, 2646}, {100, 203, 30, 2646}, {101, 204, 60, 2646}, {102, 205, 60, 2646}, {103, 206, 30, 2646}, {104, 207, 6, 2646}, {105, 211, 1, 2646}, {106, 212, 6, 2646}, {107, 213, 15, 2646}, {108, 214, 20, 2646}, {109, 215, 15, 2646}, {110, 216, 6, 2646}, {111, 217, 1, 2646}, {84, 173, 1, 2647}, {85, 177, 6, 2647}, {86, 178, 6, 2647}, {87, 182, 15, 2647}, {88, 183, 30, 2647}, {89, 184, 15, 2647}, {90, 188, 20, 2647}, {91, 189, 60, 2647}, {92, 190, 60, 2647}, {93, 191, 20, 2647}, {94, 195, 15, 2647}, {95, 196, 60, 2647}, {96, 197, 90, 2647}, {97, 198, 60, 2647}, {98, 199, 15, 2647}, {99, 203, 6, 2647}, {100, 204, 30, 2647}, {101, 205, 60, 2647}, {102, 206, 60, 2647}, {103, 207, 30, 2647}, {104, 208, 6, 2647}, {105, 212, 1, 2647}, {106, 213, 6, 2647}, {107, 214, 15, 2647}, {108, 215, 20, 2647}, {109, 216, 15, 2647}, {110, 217, 6, 2647}, {111, 218, 1, 2647}, {84, 174, 1, 2648}, {85, 178, 6, 2648}, {86, 179, 6, 2648}, {87, 183, 15, 2648}, {88, 184, 30, 2648}, {89, 185, 15, 2648}, {90, 189, 20, 2648}, {91, 190, 60, 2648}, {92, 191, 60, 2648}, {93, 192, 20, 2648}, {94, 196, 15, 2648}, {95, 197, 60, 2648}, {96, 198, 90, 2648}, {97, 199, 60, 2648}, {98, 200, 15, 2648}, {99, 204, 6, 2648}, {100, 205, 30, 2648}, {101, 206, 60, 2648}, {102, 207, 60, 2648}, {103, 208, 30, 2648}, {104, 209, 6, 2648}, {105, 213, 1, 2648}, {106, 214, 6, 2648}, {107, 215, 15, 2648}, {108, 216, 20, 2648}, {109, 217, 15, 2648}, {110, 218, 6, 2648}, {111, 219, 1, 2648}, {85, 165, 1, 2649}, {87, 166, 6, 2649}, {88, 167, 6, 2649}, {90, 168, 15, 2649}, {91, 169, 30, 2649}, {92, 170, 15, 2649}, {94, 171, 20, 2649}, {95, 172, 60, 2649}, {96, 173, 60, 2649}, {97, 174, 20, 2649}, {99, 175, 15, 2649}, {100, 176, 60, 2649}, {101, 177, 90, 2649}, {102, 178, 60, 2649}, {103, 179, 15, 2649}, {105, 180, 6, 2649}, {106, 181, 30, 2649}, {107, 182, 60, 2649}, {108, 183, 60, 2649}, {109, 184, 30, 2649}, {110, 185, 6, 2649}, {112, 186, 1, 2649}, {113, 187, 6, 2649}, {114, 188, 15, 2649}, {115, 189, 20, 2649}, {116, 190, 15, 2649}, {117, 191, 6, 2649}, {118, 192, 1, 2649}, {85, 166, 1, 2650}, {87, 168, 6, 2650}, {88, 169, 6, 2650}, {90, 171, 15, 2650}, {91, 172, 30, 2650}, {92, 173, 15, 2650}, {94, 175, 20, 2650}, {95, 176, 60, 2650}, {96, 177, 60, 2650}, {97, 178, 20, 2650}, {99, 180, 15, 2650}, {100, 181, 60, 2650}, {101, 182, 90, 2650}, {102, 183, 60, 2650}, {103, 184, 15, 2650}, {105, 186, 6, 2650}, {106, 187, 30, 2650}, {107, 188, 60, 2650}, {108, 189, 60, 2650}, {109, 190, 30, 2650}, {110, 191, 6, 2650}, {112, 193, 1, 2650}, {113, 194, 6, 2650}, {114, 195, 15, 2650}, {115, 196, 20, 2650}, {116, 197, 15, 2650}, {117, 198, 6, 2650}, {118, 199, 1, 2650}, {85, 167, 1, 2651}, {87, 169, 6, 2651}, {88, 170, 6, 2651}, {90, 172, 15, 2651}, {91, 173, 30, 2651}, {92, 174, 15, 2651}, {94, 176, 20, 2651}, {95, 177, 60, 2651}, {96, 178, 60, 2651}, {97, 179, 20, 2651}, {99, 181, 15, 2651}, {100, 182, 60, 2651}, {101, 183, 90, 2651}, {102, 184, 60, 2651}, {103, 185, 15, 2651}, {105, 187, 6, 2651}, {106, 188, 30, 2651}, {107, 189, 60, 2651}, {108, 190, 60, 2651}, {109, 191, 30, 2651}, {110, 192, 6, 2651}, {112, 194, 1, 2651}, {113, 195, 6, 2651}, {114, 196, 15, 2651}, {115, 197, 20, 2651}, {116, 198, 15, 2651}, {117, 199, 6, 2651}, {118, 200, 1, 2651}, {85, 168, 1, 2652}, {87, 171, 6, 2652}, {88, 172, 6, 2652}, {90, 175, 15, 2652}, {91, 176, 30, 2652}, {92, 177, 15, 2652}, {94, 180, 20, 2652}, {95, 181, 60, 2652}, {96, 182, 60, 2652}, {97, 183, 20, 2652}, {99, 186, 15, 2652}, {100, 187, 60, 2652}, {101, 188, 90, 2652}, {102, 189, 60, 2652}, {103, 190, 15, 2652}, {105, 193, 6, 2652}, {106, 194, 30, 2652}, {107, 195, 60, 2652}, {108, 196, 60, 2652}, {109, 197, 30, 2652}, {110, 198, 6, 2652}, {112, 201, 1, 2652}, {113, 202, 6, 2652}, {114, 203, 15, 2652}, {115, 204, 20, 2652}, {116, 205, 15, 2652}, {117, 206, 6, 2652}, {118, 207, 1, 2652}, {85, 169, 1, 2653}, {87, 172, 6, 2653}, {88, 173, 6, 2653}, {90, 176, 15, 2653}, {91, 177, 30, 2653}, {92, 178, 15, 2653}, {94, 181, 20, 2653}, {95, 182, 60, 2653}, {96, 183, 60, 2653}, {97, 184, 20, 2653}, {99, 187, 15, 2653}, {100, 188, 60, 2653}, {101, 189, 90, 2653}, {102, 190, 60,

2653}, {103, 191, 15, 2653}, {105, 194, 6, 2653}, {106, 195, 30, 2653}, {107, 196, 60, 2653}, {108, 197, 60, 2653}, {109, 198, 30, 2653}, {110, 199, 6, 2653}, {112, 202, 1, 2653}, {113, 203, 6, 2653}, {114, 204, 15, 2653}, {115, 205, 20, 2653}, {116, 206, 15, 2653}, {117, 207, 6, 2653}, {118, 208, 1, 2653}, {85, 170, 1, 2654}, {87, 173, 6, 2654}, {88, 174, 6, 2654}, {90, 177, 15, 2654}, {91, 178, 30, 2654}, {92, 179, 15, 2654}, {94, 182, 20, 2654}, {95, 183, 60, 2654}, {96, 184, 60, 2654}, {97, 185, 20, 2654}, {99, 188, 15, 2654}, {100, 189, 60, 2654}, {101, 190, 90, 2654}, {102, 191, 60, 2654}, {103, 192, 15, 2654}, {105, 195, 6, 2654}, {106, 196, 30, 2654}, {107, 197, 60, 2654}, {108, 198, 60, 2654}, {109, 199, 30, 2654}, {110, 200, 6, 2654}, {112, 203, 1, 2654}, {113, 204, 6, 2654}, {114, 205, 15, 2654}, {115, 206, 20, 2654}, {116, 207, 15, 2654}, {117, 208, 6, 2654}, {118, 209, 1, 2654}, {85, 171, 1, 2655}, {87, 175, 6, 2655}, {88, 176, 6, 2655}, {90, 180, 15, 2655}, {91, 181, 30, 2655}, {92, 182, 15, 2655}, {94, 186, 20, 2655}, {95, 187, 60, 2655}, {96, 188, 60, 2655}, {97, 189, 20, 2655}, {99, 193, 15, 2655}, {100, 194, 60, 2655}, {101, 195, 90, 2655}, {102, 196, 60, 2655}, {103, 197, 15, 2655}, {105, 201, 6, 2655}, {106, 202, 30, 2655}, {107, 203, 60, 2655}, {108, 204, 60, 2655}, {109, 205, 30, 2655}, {110, 206, 6, 2655}, {112, 210, 1, 2655}, {113, 211, 6, 2655}, {114, 212, 15, 2655}, {115, 213, 20, 2655}, {116, 214, 15, 2655}, {117, 215, 6, 2655}, {118, 216, 1, 2655}, {85, 172, 1, 2656}, {87, 176, 6, 2656}, {88, 177, 6, 2656}, {90, 181, 15, 2656}, {91, 182, 30, 2656}, {92, 183, 15, 2656}, {94, 187, 20, 2656}, {95, 188, 60, 2656}, {96, 189, 60, 2656}, {97, 190, 20, 2656}, {99, 194, 15, 2656}, {100, 195, 60, 2656}, {101, 196, 90, 2656}, {102, 197, 60, 2656}, {103, 198, 15, 2656}, {105, 202, 6, 2656}, {106, 203, 30, 2656}, {107, 204, 60, 2656}, {108, 205, 60, 2656}, {109, 206, 30, 2656}, {110, 207, 6, 2656}, {112, 211, 1, 2656}, {113, 212, 6, 2656}, {114, 213, 15, 2656}, {115, 214, 20, 2656}, {116, 215, 15, 2656}, {117, 216, 6, 2656}, {118, 217, 1, 2656}, {85, 173, 1, 2657}, {87, 177, 6, 2657}, {88, 178, 6, 2657}, {90, 182, 15, 2657}, {91, 183, 30, 2657}, {92, 184, 15, 2657}, {94, 188, 20, 2657}, {95, 189, 60, 2657}, {96, 190, 60, 2657}, {97, 191, 20, 2657}, {99, 195, 15, 2657}, {100, 196, 60, 2657}, {101, 197, 90, 2657}, {102, 198, 60, 2657}, {103, 199, 15, 2657}, {105, 203, 6, 2657}, {106, 204, 30, 2657}, {107, 205, 60, 2657}, {108, 206, 60, 2657}, {109, 207, 30, 2657}, {110, 208, 6, 2657}, {112, 212, 1, 2657}, {113, 213, 6, 2657}, {114, 214, 15, 2657}, {115, 215, 20, 2657}, {116, 216, 15, 2657}, {117, 217, 6, 2657}, {118, 218, 1, 2657}, {85, 174, 1, 2658}, {87, 178, 6, 2658}, {88, 179, 6, 2658}, {90, 183, 15, 2658}, {91, 184, 30, 2658}, {92, 185, 15, 2658}, {94, 189, 20, 2658}, {95, 190, 60, 2658}, {96, 191, 60, 2658}, {97, 192, 20, 2658}, {99, 196, 15, 2658}, {100, 197, 60, 2658}, {101, 198, 90, 2658}, {102, 199, 60, 2658}, {103, 200, 15, 2658}, {105, 204, 6, 2658}, {106, 205, 30, 2658}, {107, 206, 60, 2658}, {108, 207, 60, 2658}, {109, 208, 30, 2658}, {110, 209, 6, 2658}, {112, 213, 1, 2658}, {113, 214, 6, 2658}, {114, 215, 15, 2658}, {115, 216, 20, 2658}, {116, 217, 15, 2658}, {117, 218, 6, 2658}, {118, 219, 1, 2658}, {86, 165, 1, 2659}, {88, 166, 6, 2659}, {89, 167, 6, 2659}, {91, 168, 15, 2659}, {92, 169, 30, 2659}, {93, 170, 15, 2659}, {95, 171, 20, 2659}, {96, 172, 60, 2659}, {97, 173, 60, 2659}, {98, 174, 20, 2659}, {100, 175, 15, 2659}, {101, 176, 60, 2659}, {102, 177, 90, 2659}, {103, 178, 60, 2659}, {104, 179, 15, 2659}, {106, 180, 6, 2659}, {107, 181, 30, 2659}, {108, 182, 60, 2659}, {109, 183, 60, 2659}, {110, 184, 30, 2659}, {111, 185, 6, 2659}, {113, 186, 1, 2659}, {114, 187, 6, 2659}, {115, 188, 15, 2659}, {116, 189, 20, 2659}, {117, 190, 15, 2659}, {118, 191, 6, 2659}, {119, 192, 1, 2659}, {86, 166, 1, 2660}, {88, 168, 6, 2660}, {89, 169, 6, 2660}, {91, 171, 15, 2660}, {92, 172, 30, 2660}, {93, 173, 15, 2660}, {95, 175, 20, 2660}, {96, 176, 60, 2660}, {97, 177, 60, 2660}, {98, 178, 20, 2660}, {100, 180, 15, 2660}, {101, 181, 60, 2660}, {102, 182, 90, 2660}, {103, 183, 60, 2660}, {104, 184, 15, 2660}, {106, 186, 6, 2660}, {107, 187, 30, 2660}, {108, 188, 60, 2660}, {109, 189, 60, 2660}, {110, 190, 30, 2660}, {111, 191, 6, 2660}, {113, 193, 1, 2660}, {114, 194, 6, 2660}, {115, 195, 15, 2660}, {116, 196, 20, 2660}, {117, 197, 15, 2660}, {118, 198, 6, 2660}, {119, 199, 1, 2660}, {86, 167, 1, 2661}, {88, 169, 6, 2661}, {89, 170, 6, 2661}, {91, 172, 15, 2661}, {92, 173, 30, 2661}, {93, 174, 15, 2661}, {95, 176, 20, 2661}, {96, 177, 60, 2661}, {97, 178, 60, 2661}, {98, 179, 20, 2661}, {100, 181, 15, 2661}, {101, 182, 60, 2661}, {102, 183, 90, 2661}, {103, 184, 60, 2661}, {104, 185, 15, 2661}, {106, 187, 6, 2661}, {107, 188, 30, 2661}, {108, 189, 60, 2661}, {109, 190, 60, 2661}, {110, 191, 30, 2661}, {111, 192, 6, 2661}, {113, 194, 1, 2661}, {114, 195, 6, 2661}, {115, 196, 15, 2661}, {116, 197, 20, 2661}, {117, 198, 15, 2661}, {118, 199, 6, 2661}, {119, 200, 1, 2661}, {86, 168, 1, 2662}, {88, 171, 6, 2662}, {89, 172, 6, 2662}, {91, 175, 15, 2662}, {92, 176, 30, 2662}, {93, 177, 15, 2662}, {95, 180, 20, 2662}, {96, 181, 60, 2662}, {97, 182, 60, 2662}, {98, 183, 20, 2662}, {100, 186, 15, 2662}, {101, 187, 60, 2662}, {102, 188, 90, 2662}, {103, 189, 60, 2662}, {104, 190, 15, 2662}, {106, 193, 6, 2662}, {107, 194, 30, 2662}, {108, 195, 60, 2662}, {109, 196, 60, 2662}, {110, 197, 30, 2662}, {111, 198, 6, 2662}, {113, 201, 1, 2662}, {114, 202, 6, 2662}, {115, 203, 15, 2662}, {116, 204, 20, 2662}, {117, 205, 15, 2662}, {118, 206, 6, 2662}, {119, 207, 1, 2662}, {86, 169, 1, 2663}, {88, 172, 6, 2663}, {89, 173, 6, 2663}, {91, 176, 15, 2663}, {92, 177, 30, 2663}, {93, 178, 15, 2663}, {95, 181, 20, 2663}, {96, 182, 60, 2663}, {97, 183, 60, 2663}, {98, 184, 20, 2663}, {100, 187, 15, 2663}, {101, 188, 60, 2663}, {102, 189, 90, 2663}, {103, 190, 60, 2663}, {104, 191, 15, 2663}, {106, 194, 6, 2663}, {107, 195, 30, 2663}, {108, 196, 60, 2663}, {109, 197, 60, 2663}, {110, 198, 30,

2663}, {111, 199, 6, 2663}, {113, 202, 1, 2663}, {114, 203, 6, 2663}, {115, 204, 15, 2663}, {116, 205, 20, 2663}, {117, 206, 15, 2663}, {118, 207, 6, 2663}, {119, 208, 1, 2663}, {86, 170, 1, 2664}, {88, 173, 6, 2664}, {89, 174, 6, 2664}, {91, 177, 15, 2664}, {92, 178, 30, 2664}, {93, 179, 15, 2664}, {95, 182, 20, 2664}, {96, 183, 60, 2664}, {97, 184, 60, 2664}, {98, 185, 20, 2664}, {100, 188, 15, 2664}, {101, 189, 60, 2664}, {102, 190, 90, 2664}, {103, 191, 60, 2664}, {104, 192, 15, 2664}, {106, 195, 6, 2664}, {107, 196, 30, 2664}, {108, 197, 60, 2664}, {109, 198, 60, 2664}, {110, 199, 30, 2664}, {111, 200, 6, 2664}, {113, 203, 1, 2664}, {114, 204, 6, 2664}, {115, 205, 15, 2664}, {116, 206, 20, 2664}, {117, 207, 15, 2664}, {118, 208, 6, 2664}, {119, 209, 1, 2664}, {86, 171, 1, 2665}, {88, 175, 6, 2665}, {89, 176, 6, 2665}, {91, 180, 15, 2665}, {92, 181, 30, 2665}, {93, 182, 15, 2665}, {95, 186, 20, 2665}, {96, 187, 60, 2665}, {97, 188, 60, 2665}, {98, 189, 20, 2665}, {100, 193, 15, 2665}, {101, 194, 60, 2665}, {102, 195, 90, 2665}, {103, 196, 60, 2665}, {104, 197, 15, 2665}, {106, 201, 6, 2665}, {107, 202, 30, 2665}, {108, 203, 60, 2665}, {109, 204, 60, 2665}, {110, 205, 30, 2665}, {111, 206, 6, 2665}, {113, 210, 1, 2665}, {114, 211, 6, 2665}, {115, 212, 15, 2665}, {116, 213, 20, 2665}, {117, 214, 15, 2665}, {118, 215, 6, 2665}, {119, 216, 1, 2665}, {86, 172, 1, 2666}, {88, 176, 6, 2666}, {89, 177, 6, 2666}, {91, 181, 15, 2666}, {92, 182, 30, 2666}, {93, 183, 15, 2666}, {95, 187, 20, 2666}, {96, 188, 60, 2666}, {97, 189, 60, 2666}, {98, 190, 20, 2666}, {100, 194, 15, 2666}, {101, 195, 60, 2666}, {102, 196, 90, 2666}, {103, 197, 60, 2666}, {104, 198, 15, 2666}, {106, 202, 6, 2666}, {107, 203, 30, 2666}, {108, 204, 60, 2666}, {109, 205, 60, 2666}, {110, 206, 30, 2666}, {111, 207, 6, 2666}, {113, 211, 1, 2666}, {114, 212, 6, 2666}, {115, 213, 15, 2666}, {116, 214, 20, 2666}, {117, 215, 15, 2666}, {118, 216, 6, 2666}, {119, 217, 1, 2666}, {86, 173, 1, 2667}, {88, 177, 6, 2667}, {89, 178, 6, 2667}, {91, 182, 15, 2667}, {92, 183, 30, 2667}, {93, 184, 15, 2667}, {95, 188, 20, 2667}, {96, 189, 60, 2667}, {97, 190, 60, 2667}, {98, 191, 20, 2667}, {100, 195, 15, 2667}, {101, 196, 60, 2667}, {102, 197, 90, 2667}, {103, 198, 60, 2667}, {104, 199, 15, 2667}, {106, 203, 6, 2667}, {107, 204, 30, 2667}, {108, 205, 60, 2667}, {109, 206, 60, 2667}, {110, 207, 30, 2667}, {111, 208, 6, 2667}, {113, 212, 1, 2667}, {114, 213, 6, 2667}, {115, 214, 15, 2667}, {116, 215, 20, 2667}, {117, 216, 15, 2667}, {118, 217, 6, 2667}, {119, 218, 1, 2667}, {86, 174, 1, 2668}, {88, 178, 6, 2668}, {89, 179, 6, 2668}, {91, 183, 15, 2668}, {92, 184, 30, 2668}, {93, 185, 15, 2668}, {95, 189, 20, 2668}, {96, 190, 60, 2668}, {97, 191, 60, 2668}, {98, 192, 20, 2668}, {100, 196, 15, 2668}, {101, 197, 60, 2668}, {102, 198, 90, 2668}, {103, 199, 60, 2668}, {104, 200, 15, 2668}, {106, 204, 6, 2668}, {107, 205, 30, 2668}, {108, 206, 60, 2668}, {109, 207, 60, 2668}, {110, 208, 30, 2668}, {111, 209, 6, 2668}, {113, 213, 1, 2668}, {114, 214, 6, 2668}, {115, 215, 15, 2668}, {116, 216, 20, 2668}, {117, 217, 15, 2668}, {118, 218, 6, 2668}, {119, 219, 1, 2668}, {84, 84, 1, 2669}, {85, 85, 7, 2669}, {86, 86, 7, 2669}, {87, 87, 21, 2669}, {88, 88, 42, 2669}, {89, 89, 21, 2669}, {90, 90, 35, 2669}, {91, 91, 105, 2669}, {92, 92, 105, 2669}, {93, 93, 35, 2669}, {94, 94, 35, 2669}, {95, 95, 140, 2669}, {96, 96, 210, 2669}, {97, 97, 140, 2669}, {98, 98, 35, 2669}, {99, 99, 21, 2669}, {100, 100, 105, 2669}, {101, 101, 210, 2669}, {102, 102, 210, 2669}, {103, 103, 105, 2669}, {104, 104, 21, 2669}, {105, 105, 7, 2669}, {106, 106, 42, 2669}, {107, 107, 105, 2669}, {108, 108, 140, 2669}, {109, 109, 105, 2669}, {110, 110, 42, 2669}, {111, 111, 7, 2669}, {112, 112, 1, 2669}, {113, 113, 7, 2669}, {114, 114, 21, 2669}, {115, 115, 35, 2669}, {116, 116, 35, 2669}, {117, 117, 21, 2669}, {118, 118, 7, 2669}, {119, 119, 1, 2669}, {84, 120, 1, 2670}, {85, 121, 7, 2670}, {86, 122, 7, 2670}, {87, 123, 21, 2670}, {88, 124, 42, 2670}, {89, 125, 21, 2670}, {90, 126, 35, 2670}, {91, 127, 105, 2670}, {92, 128, 105, 2670}, {93, 129, 35, 2670}, {94, 130, 35, 2670}, {95, 131, 140, 2670}, {96, 132, 210, 2670}, {97, 133, 140, 2670}, {98, 134, 35, 2670}, {99, 135, 21, 2670}, {100, 136, 105, 2670}, {101, 137, 210, 2670}, {102, 138, 210, 2670}, {103, 139, 105, 2670}, {104, 140, 21, 2670}, {105, 141, 7, 2670}, {106, 142, 42, 2670}, {107, 143, 105, 2670}, {108, 144, 140, 2670}, {109, 145, 105, 2670}, {110, 146, 42, 2670}, {111, 147, 7, 2670}, {112, 148, 1, 2670}, {113, 149, 7, 2670}, {114, 150, 21, 2670}, {115, 151, 35, 2670}, {116, 152, 35, 2670}, {117, 153, 21, 2670}, {118, 154, 7, 2670}, {119, 155, 1, 2670}, {84, 121, 1, 2671}, {85, 123, 7, 2671}, {86, 124, 7, 2671}, {87, 126, 21, 2671}, {88, 127, 42, 2671}, {89, 128, 21, 2671}, {90, 130, 35, 2671}, {91, 131, 105, 2671}, {92, 132, 105, 2671}, {93, 133, 35, 2671}, {94, 135, 35, 2671}, {95, 136, 140, 2671}, {96, 137, 210, 2671}, {97, 138, 140, 2671}, {98, 139, 35, 2671}, {99, 141, 21, 2671}, {100, 142, 105, 2671}, {101, 143, 210, 2671}, {102, 144, 210, 2671}, {103, 145, 105, 2671}, {104, 146, 21, 2671}, {105, 148, 7, 2671}, {106, 149, 42, 2671}, {107, 150, 105, 2671}, {108, 151, 140, 2671}, {109, 152, 105, 2671}, {110, 153, 42, 2671}, {111, 154, 7, 2671}, {112, 156, 1, 2671}, {113, 157, 7, 2671}, {114, 158, 21, 2671}, {115, 159, 35, 2671}, {116, 160, 35, 2671}, {117, 161, 21, 2671}, {118, 162, 7, 2671}, {119, 163, 1, 2671}, {84, 122, 1, 2672}, {85, 124, 7, 2672}, {86, 125, 7, 2672}, {87, 127, 21, 2672}, {88, 128, 42, 2672}, {89, 129, 21, 2672}, {90, 131, 35, 2672}, {91, 132, 105, 2672}, {92, 133, 105, 2672}, {93, 134, 35, 2672}, {94, 136, 35, 2672}, {95, 137, 140, 2672}, {96, 138, 210, 2672}, {97, 139, 140, 2672}, {98, 140, 35, 2672}, {99, 142, 21, 2672}, {100, 143, 105, 2672}, {101, 144, 210, 2672}, {102, 145, 210, 2672}, {103, 146, 105, 2672}, {104, 147, 21, 2672}, {105, 149, 7, 2672}, {106, 150, 42, 2672}, {107, 151, 105, 2672}, {108, 152, 140, 2672}, {109, 153, 105, 2672}, {110, 154, 42, 2672}, {111, 155, 7, 2672}, {112, 157, 1, 2672}, {113, 158, 7,

2672}, {114, 159, 21, 2672}, {115, 160, 35, 2672}, {116, 161, 35, 2672}, {117, 162, 21, 2672}, {118, 163, 7, 2672}, {119, 164, 1, 2672}, {84, 165, 1, 2673}, {85, 166, 7, 2673}, {86, 167, 7, 2673}, {87, 168, 21, 2673}, {88, 169, 42, 2673}, {89, 170, 21, 2673}, {90, 171, 35, 2673}, {91, 172, 105, 2673}, {92, 173, 105, 2673}, {93, 174, 35, 2673}, {94, 175, 35, 2673}, {95, 176, 140, 2673}, {96, 177, 210, 2673}, {97, 178, 140, 2673}, {98, 179, 35, 2673}, {99, 180, 21, 2673}, {100, 181, 105, 2673}, {101, 182, 210, 2673}, {102, 183, 210, 2673}, {103, 184, 105, 2673}, {104, 185, 21, 2673}, {105, 186, 7, 2673}, {106, 187, 42, 2673}, {107, 188, 105, 2673}, {108, 189, 140, 2673}, {109, 190, 105, 2673}, {110, 191, 42, 2673}, {111, 192, 7, 2673}, {112, 193, 1, 2673}, {113, 194, 7, 2673}, {114, 195, 21, 2673}, {115, 196, 35, 2673}, {116, 197, 35, 2673}, {117, 198, 21, 2673}, {118, 199, 7, 2673}, {119, 200, 1, 2673}, {84, 166, 1, 2674}, {85, 168, 7, 2674}, {86, 169, 7, 2674}, {87, 171, 21, 2674}, {88, 172, 42, 2674}, {89, 173, 21, 2674}, {90, 175, 35, 2674}, {91, 176, 105, 2674}, {92, 177, 105, 2674}, {93, 178, 35, 2674}, {94, 180, 35, 2674}, {95, 181, 140, 2674}, {96, 182, 210, 2674}, {97, 183, 140, 2674}, {98, 184, 35, 2674}, {99, 186, 21, 2674}, {100, 187, 105, 2674}, {101, 188, 210, 2674}, {102, 189, 210, 2674}, {103, 190, 105, 2674}, {104, 191, 21, 2674}, {105, 193, 7, 2674}, {106, 194, 42, 2674}, {107, 195, 105, 2674}, {108, 196, 140, 2674}, {109, 197, 105, 2674}, {110, 198, 42, 2674}, {111, 199, 7, 2674}, {112, 201, 1, 2674}, {113, 202, 7, 2674}, {114, 203, 21, 2674}, {115, 204, 35, 2674}, {116, 205, 35, 2674}, {117, 206, 21, 2674}, {118, 207, 7, 2674}, {119, 208, 1, 2674}, {84, 167, 1, 2675}, {85, 169, 7, 2675}, {86, 170, 7, 2675}, {87, 172, 21, 2675}, {88, 173, 42, 2675}, {89, 174, 21, 2675}, {90, 176, 35, 2675}, {91, 177, 105, 2675}, {92, 178, 105, 2675}, {93, 179, 35, 2675}, {94, 181, 35, 2675}, {95, 182, 140, 2675}, {96, 183, 210, 2675}, {97, 184, 140, 2675}, {98, 185, 35, 2675}, {99, 187, 21, 2675}, {100, 188, 105, 2675}, {101, 189, 210, 2675}, {102, 190, 210, 2675}, {103, 191, 105, 2675}, {104, 192, 21, 2675}, {105, 194, 7, 2675}, {106, 195, 42, 2675}, {107, 196, 105, 2675}, {108, 197, 140, 2675}, {109, 198, 105, 2675}, {110, 199, 42, 2675}, {111, 200, 7, 2675}, {112, 202, 1, 2675}, {113, 203, 7, 2675}, {114, 204, 21, 2675}, {115, 205, 35, 2675}, {116, 206, 35, 2675}, {117, 207, 21, 2675}, {118, 208, 7, 2675}, {119, 209, 1, 2675}, {84, 168, 1, 2676}, {85, 171, 7, 2676}, {86, 172, 7, 2676}, {87, 175, 21, 2676}, {88, 176, 42, 2676}, {89, 177, 21, 2676}, {90, 180, 35, 2676}, {91, 181, 105, 2676}, {92, 182, 105, 2676}, {93, 183, 35, 2676}, {94, 186, 35, 2676}, {95, 187, 140, 2676}, {96, 188, 210, 2676}, {97, 189, 140, 2676}, {98, 190, 35, 2676}, {99, 193, 21, 2676}, {100, 194, 105, 2676}, {101, 195, 210, 2676}, {102, 196, 210, 2676}, {103, 197, 105, 2676}, {104, 198, 21, 2676}, {105, 201, 7, 2676}, {106, 202, 42, 2676}, {107, 203, 105, 2676}, {108, 204, 140, 2676}, {109, 205, 105, 2676}, {110, 206, 42, 2676}, {111, 207, 7, 2676}, {112, 210, 1, 2676}, {113, 211, 7, 2676}, {114, 212, 21, 2676}, {115, 213, 35, 2676}, {116, 214, 35, 2676}, {117, 215, 21, 2676}, {118, 216, 7, 2676}, {119, 217, 1, 2676}, {84, 169, 1, 2677}, {85, 172, 7, 2677}, {86, 173, 7, 2677}, {87, 176, 21, 2677}, {88, 177, 42, 2677}, {89, 178, 21, 2677}, {90, 181, 35, 2677}, {91, 182, 105, 2677}, {92, 183, 105, 2677}, {93, 184, 35, 2677}, {94, 187, 35, 2677}, {95, 188, 140, 2677}, {96, 189, 210, 2677}, {97, 190, 140, 2677}, {98, 191, 35, 2677}, {99, 194, 21, 2677}, {100, 195, 105, 2677}, {101, 196, 210, 2677}, {102, 197, 210, 2677}, {103, 198, 105, 2677}, {104, 199, 21, 2677}, {105, 202, 7, 2677}, {106, 203, 42, 2677}, {107, 204, 105, 2677}, {108, 205, 140, 2677}, {109, 206, 105, 2677}, {110, 207, 42, 2677}, {111, 208, 7, 2677}, {112, 211, 1, 2677}, {113, 212, 7, 2677}, {114, 213, 21, 2677}, {115, 214, 35, 2677}, {116, 215, 35, 2677}, {117, 216, 21, 2677}, {118, 217, 7, 2677}, {119, 218, 1, 2677}, {84, 170, 1, 2678}, {85, 173, 7, 2678}, {86, 174, 7, 2678}, {87, 177, 21, 2678}, {88, 178, 42, 2678}, {89, 179, 21, 2678}, {90, 182, 35, 2678}, {91, 183, 105, 2678}, {92, 184, 105, 2678}, {93, 185, 35, 2678}, {94, 188, 35, 2678}, {95, 189, 140, 2678}, {96, 190, 210, 2678}, {97, 191, 140, 2678}, {98, 192, 35, 2678}, {99, 195, 21, 2678}, {100, 196, 105, 2678}, {101, 197, 210, 2678}, {102, 198, 210, 2678}, {103, 199, 105, 2678}, {104, 200, 21, 2678}, {105, 203, 7, 2678}, {106, 204, 42, 2678}, {107, 205, 105, 2678}, {108, 206, 140, 2678}, {109, 207, 105, 2678}, {110, 208, 42, 2678}, {111, 209, 7, 2678}, {112, 212, 1, 2678}, {113, 213, 7, 2678}, {114, 214, 21, 2678}, {115, 215, 35, 2678}, {116, 216, 35, 2678}, {117, 217, 21, 2678}, {118, 218, 7, 2678}, {119, 219, 1, 2678}, {84, 220, 1, 2679}, {85, 221, 7, 2679}, {86, 222, 7, 2679}, {87, 223, 21, 2679}, {88, 224, 42, 2679}, {89, 225, 21, 2679}, {90, 226, 35, 2679}, {91, 227, 105, 2679}, {92, 228, 105, 2679}, {93, 229, 35, 2679}, {94, 230, 35, 2679}, {95, 231, 140, 2679}, {96, 232, 210, 2679}, {97, 233, 140, 2679}, {98, 234, 35, 2679}, {99, 235, 21, 2679}, {100, 236, 105, 2679}, {101, 237, 210, 2679}, {102, 238, 210, 2679}, {103, 239, 105, 2679}, {104, 240, 21, 2679}, {105, 241, 7, 2679}, {106, 242, 42, 2679}, {107, 243, 105, 2679}, {108, 244, 140, 2679}, {109, 245, 105, 2679}, {110, 246, 42, 2679}, {111, 247, 7, 2679}, {112, 248, 1, 2679}, {113, 249, 7, 2679}, {114, 250, 21, 2679}, {115, 251, 35, 2679}, {116, 252, 35, 2679}, {117, 253, 21, 2679}, {118, 254, 7, 2679}, {119, 255, 1, 2679}, {84, 221, 1, 2680}, {85, 223, 7, 2680}, {86, 224, 7, 2680}, {87, 226, 21, 2680}, {88, 227, 42, 2680}, {89, 228, 21, 2680}, {90, 230, 35, 2680}, {91, 231, 105, 2680}, {92, 232, 105, 2680}, {93, 233, 35, 2680}, {94, 235, 35, 2680}, {95, 236, 140, 2680}, {96, 237, 210, 2680}, {97, 238, 140, 2680}, {98, 239, 35, 2680}, {99, 241, 21, 2680}, {100, 242, 105, 2680}, {101, 243, 210, 2680}, {102, 244, 210, 2680}, {103, 245, 105, 2680}, {104, 246, 21, 2680}, {105, 248, 7, 2680}, {106, 249, 42, 2680}, {107, 250, 105, 2680}, {108, 251, 140, 2680}, {109, 252,

105, 2680}, {110, 253, 42, 2680}, {111, 254, 7, 2680}, {112, 256, 1, 2680}, {113, 257, 7, 2680}, {114, 258, 21, 2680}, {115, 259, 35, 2680}, {116, 260, 35, 2680}, {117, 261, 21, 2680}, {118, 262, 7, 2680}, {119, 263, 1, 2680}, {84, 222, 1, 2681}, {85, 224, 7, 2681}, {86, 225, 7, 2681}, {87, 227, 21, 2681}, {88, 228, 42, 2681}, {89, 229, 21, 2681}, {90, 231, 35, 2681}, {91, 232, 105, 2681}, {92, 233, 105, 2681}, {93, 234, 35, 2681}, {94, 236, 35, 2681}, {95, 237, 140, 2681}, {96, 238, 210, 2681}, {97, 239, 140, 2681}, {98, 240, 35, 2681}, {99, 242, 21, 2681}, {100, 243, 105, 2681}, {101, 244, 210, 2681}, {102, 245, 210, 2681}, {103, 246, 105, 2681}, {104, 247, 21, 2681}, {105, 249, 7, 2681}, {106, 250, 42, 2681}, {107, 251, 105, 2681}, {108, 252, 140, 2681}, {109, 253, 105, 2681}, {110, 254, 42, 2681}, {111, 255, 7, 2681}, {112, 257, 1, 2681}, {113, 258, 7, 2681}, {114, 259, 21, 2681}, {115, 260, 35, 2681}, {116, 261, 35, 2681}, {117, 262, 21, 2681}, {118, 263, 7, 2681}, {119, 264, 1, 2681}, {84, 223, 1, 2682}, {85, 226, 7, 2682}, {86, 227, 7, 2682}, {87, 230, 21, 2682}, {88, 231, 42, 2682}, {89, 232, 21, 2682}, {90, 235, 35, 2682}, {91, 236, 105, 2682}, {92, 237, 105, 2682}, {93, 238, 35, 2682}, {94, 241, 35, 2682}, {95, 242, 140, 2682}, {96, 243, 210, 2682}, {97, 244, 140, 2682}, {98, 245, 35, 2682}, {99, 248, 21, 2682}, {100, 249, 105, 2682}, {101, 250, 210, 2682}, {102, 251, 210, 2682}, {103, 252, 105, 2682}, {104, 253, 21, 2682}, {105, 256, 7, 2682}, {106, 257, 42, 2682}, {107, 258, 105, 2682}, {108, 259, 140, 2682}, {109, 260, 105, 2682}, {110, 261, 42, 2682}, {111, 262, 7, 2682}, {112, 265, 1, 2682}, {113, 266, 7, 2682}, {114, 267, 21, 2682}, {115, 268, 35, 2682}, {116, 269, 35, 2682}, {117, 270, 21, 2682}, {118, 271, 7, 2682}, {119, 272, 1, 2682}, {84, 224, 1, 2683}, {85, 227, 7, 2683}, {86, 228, 7, 2683}, {87, 231, 21, 2683}, {88, 232, 42, 2683}, {89, 233, 21, 2683}, {90, 236, 35, 2683}, {91, 237, 105, 2683}, {92, 238, 105, 2683}, {93, 239, 35, 2683}, {94, 242, 35, 2683}, {95, 243, 140, 2683}, {96, 244, 210, 2683}, {97, 245, 140, 2683}, {98, 246, 35, 2683}, {99, 249, 21, 2683}, {100, 250, 105, 2683}, {101, 251, 210, 2683}, {102, 252, 210, 2683}, {103, 253, 105, 2683}, {104, 254, 21, 2683}, {105, 257, 7, 2683}, {106, 258, 42, 2683}, {107, 259, 105, 2683}, {108, 260, 140, 2683}, {109, 261, 105, 2683}, {110, 262, 42, 2683}, {111, 263, 7, 2683}, {112, 266, 1, 2683}, {113, 267, 7, 2683}, {114, 268, 21, 2683}, {115, 269, 35, 2683}, {116, 270, 35, 2683}, {117, 271, 21, 2683}, {118, 272, 7, 2683}, {119, 273, 1, 2683}, {84, 225, 1, 2684}, {85, 228, 7, 2684}, {86, 229, 7, 2684}, {87, 232, 21, 2684}, {88, 233, 42, 2684}, {89, 234, 21, 2684}, {90, 237, 35, 2684}, {91, 238, 105, 2684}, {92, 239, 105, 2684}, {93, 240, 35, 2684}, {94, 243, 35, 2684}, {95, 244, 140, 2684}, {96, 245, 210, 2684}, {97, 246, 140, 2684}, {98, 247, 35, 2684}, {99, 250, 21, 2684}, {100, 251, 105, 2684}, {101, 252, 210, 2684}, {102, 253, 210, 2684}, {103, 254, 105, 2684}, {104, 255, 21, 2684}, {105, 258, 7, 2684}, {106, 259, 42, 2684}, {107, 260, 105, 2684}, {108, 261, 140, 2684}, {109, 262, 105, 2684}, {110, 263, 42, 2684}, {111, 264, 7, 2684}, {112, 267, 1, 2684}, {113, 268, 7, 2684}, {114, 269, 21, 2684}, {115, 270, 35, 2684}, {116, 271, 35, 2684}, {117, 272, 21, 2684}, {118, 273, 7, 2684}, {119, 274, 1, 2684}, {84, 226, 1, 2685}, {85, 230, 7, 2685}, {86, 231, 7, 2685}, {87, 235, 21, 2685}, {88, 236, 42, 2685}, {89, 237, 21, 2685}, {90, 241, 35, 2685}, {91, 242, 105, 2685}, {92, 243, 105, 2685}, {93, 244, 35, 2685}, {94, 248, 35, 2685}, {95, 249, 140, 2685}, {96, 250, 210, 2685}, {97, 251, 140, 2685}, {98, 252, 35, 2685}, {99, 256, 21, 2685}, {100, 257, 105, 2685}, {101, 258, 210, 2685}, {102, 259, 210, 2685}, {103, 260, 105, 2685}, {104, 261, 21, 2685}, {105, 265, 7, 2685}, {106, 266, 42, 2685}, {107, 267, 105, 2685}, {108, 268, 140, 2685}, {109, 269, 105, 2685}, {110, 270, 42, 2685}, {111, 271, 7, 2685}, {112, 275, 1, 2685}, {113, 276, 7, 2685}, {114, 277, 21, 2685}, {115, 278, 35, 2685}, {116, 279, 35, 2685}, {117, 280, 21, 2685}, {118, 281, 7, 2685}, {119, 282, 1, 2685}, {84, 227, 1, 2686}, {85, 231, 7, 2686}, {86, 232, 7, 2686}, {87, 236, 21, 2686}, {88, 237, 42, 2686}, {89, 238, 21, 2686}, {90, 242, 35, 2686}, {91, 243, 105, 2686}, {92, 244, 105, 2686}, {93, 245, 35, 2686}, {94, 249, 35, 2686}, {95, 250, 140, 2686}, {96, 251, 210, 2686}, {97, 252, 140, 2686}, {98, 253, 35, 2686}, {99, 257, 21, 2686}, {100, 258, 105, 2686}, {101, 259, 210, 2686}, {102, 260, 210, 2686}, {103, 261, 105, 2686}, {104, 262, 21, 2686}, {105, 266, 7, 2686}, {106, 267, 42, 2686}, {107, 268, 105, 2686}, {108, 269, 140, 2686}, {109, 270, 105, 2686}, {110, 271, 42, 2686}, {111, 272, 7, 2686}, {112, 276, 1, 2686}, {113, 277, 7, 2686}, {114, 278, 21, 2686}, {115, 279, 35, 2686}, {116, 280, 35, 2686}, {117, 281, 21, 2686}, {118, 282, 7, 2686}, {119, 283, 1, 2686}, {84, 228, 1, 2687}, {85, 232, 7, 2687}, {86, 233, 7, 2687}, {87, 237, 21, 2687}, {88, 238, 42, 2687}, {89, 239, 21, 2687}, {90, 243, 35, 2687}, {91, 244, 105, 2687}, {92, 245, 105, 2687}, {93, 246, 35, 2687}, {94, 250, 35, 2687}, {95, 251, 140, 2687}, {96, 252, 210, 2687}, {97, 253, 140, 2687}, {98, 254, 35, 2687}, {99, 258, 21, 2687}, {100, 259, 105, 2687}, {101, 260, 210, 2687}, {102, 261, 210, 2687}, {103, 262, 105, 2687}, {104, 263, 21, 2687}, {105, 267, 7, 2687}, {106, 268, 42, 2687}, {107, 269, 105, 2687}, {108, 270, 140, 2687}, {109, 271, 105, 2687}, {110, 272, 42, 2687}, {111, 273, 7, 2687}, {112, 277, 1, 2687}, {113, 278, 7, 2687}, {114, 279, 21, 2687}, {115, 280, 35, 2687}, {116, 281, 35, 2687}, {117, 282, 21, 2687}, {118, 283, 7, 2687}, {119, 284, 1, 2687}, {84, 229, 1, 2688}, {85, 233, 7, 2688}, {86, 234, 7, 2688}, {87, 238, 21, 2688}, {88, 239, 42, 2688}, {89, 240, 21, 2688}, {90, 244, 35, 2688}, {91, 245, 105, 2688}, {92, 246, 105, 2688}, {93, 247, 35, 2688}, {94, 251, 35, 2688}, {95, 252, 140, 2688}, {96, 253, 210, 2688}, {97, 254, 140, 2688}, {98, 255, 35, 2688}, {99, 259, 21, 2688}, {100, 260, 105, 2688}, {101, 261, 210, 2688}, {102, 262, 210, 2688}, {103, 263, 105, 2688}, {104, 264, 21, 2688}, {105, 268,

7, 2688}, {106, 269, 42, 2688}, {107, 270, 105, 2688}, {108, 271, 140, 2688}, {109, 272, 105, 2688}, {110, 273, 42, 2688}, {111, 274, 7, 2688}, {112, 278, 1, 2688}, {113, 279, 7, 2688}, {114, 280, 21, 2688}, {115, 281, 35, 2688}, {116, 282, 35, 2688}, {117, 283, 21, 2688}, {118, 284, 7, 2688}, {119, 285, 1, 2688}, {120, 120, 1, 2689}, {121, 121, 6, 2689}, {122, 122, 6, 2689}, {123, 123, 15, 2689}, {124, 124, 30, 2689}, {125, 125, 15, 2689}, {126, 126, 20, 2689}, {127, 127, 60, 2689}, {128, 128, 60, 2689}, {129, 129, 20, 2689}, {130, 130, 15, 2689}, {131, 131, 60, 2689}, {132, 132, 90, 2689}, {133, 133, 60, 2689}, {134, 134, 15, 2689}, {135, 135, 6, 2689}, {136, 136, 30, 2689}, {137, 137, 60, 2689}, {138, 138, 60, 2689}, {139, 139, 30, 2689}, {140, 140, 6, 2689}, {141, 141, 1, 2689}, {142, 142, 6, 2689}, {143, 143, 15, 2689}, {144, 144, 20, 2689}, {145, 145, 15, 2689}, {146, 146, 6, 2689}, {147, 147, 1, 2689}, {120, 121, 1, 2690}, {121, 123, 6, 2690}, {122, 124, 6, 2690}, {123, 126, 15, 2690}, {124, 127, 30, 2690}, {125, 128, 15, 2690}, {126, 130, 20, 2690}, {127, 131, 60, 2690}, {128, 132, 60, 2690}, {129, 133, 20, 2690}, {130, 135, 15, 2690}, {131, 136, 60, 2690}, {132, 137, 90, 2690}, {133, 138, 60, 2690}, {134, 139, 15, 2690}, {135, 141, 6, 2690}, {136, 142, 30, 2690}, {137, 143, 60, 2690}, {138, 144, 60, 2690}, {139, 145, 30, 2690}, {140, 146, 6, 2690}, {141, 148, 1, 2690}, {142, 149, 6, 2690}, {143, 150, 15, 2690}, {144, 151, 20, 2690}, {145, 152, 15, 2690}, {146, 153, 6, 2690}, {147, 154, 1, 2690}, {120, 122, 1, 2691}, {121, 124, 6, 2691}, {122, 125, 6, 2691}, {123, 127, 15, 2691}, {124, 128, 30, 2691}, {125, 129, 15, 2691}, {126, 131, 20, 2691}, {127, 132, 60, 2691}, {128, 133, 60, 2691}, {129, 134, 20, 2691}, {130, 136, 15, 2691}, {131, 137, 60, 2691}, {132, 138, 90, 2691}, {133, 139, 60, 2691}, {134, 140, 15, 2691}, {135, 142, 6, 2691}, {136, 143, 30, 2691}, {137, 144, 60, 2691}, {138, 145, 60, 2691}, {139, 146, 30, 2691}, {140, 147, 6, 2691}, {141, 149, 1, 2691}, {142, 150, 6, 2691}, {143, 151, 15, 2691}, {144, 152, 20, 2691}, {145, 153, 15, 2691}, {146, 154, 6, 2691}, {147, 155, 1, 2691}, {120, 123, 1, 2692}, {121, 126, 6, 2692}, {122, 127, 6, 2692}, {123, 130, 15, 2692}, {124, 131, 30, 2692}, {125, 132, 15, 2692}, {126, 135, 20, 2692}, {127, 136, 60, 2692}, {128, 137, 60, 2692}, {129, 138, 20, 2692}, {130, 141, 15, 2692}, {131, 142, 60, 2692}, {132, 143, 90, 2692}, {133, 144, 60, 2692}, {134, 145, 15, 2692}, {135, 148, 6, 2692}, {136, 149, 30, 2692}, {137, 150, 60, 2692}, {138, 151, 60, 2692}, {139, 152, 30, 2692}, {140, 153, 6, 2692}, {141, 156, 1, 2692}, {142, 157, 6, 2692}, {143, 158, 15, 2692}, {144, 159, 20, 2692}, {145, 160, 15, 2692}, {146, 161, 6, 2692}, {147, 162, 1, 2692}, {120, 124, 1, 2693}, {121, 127, 6, 2693}, {122, 128, 6, 2693}, {123, 131, 15, 2693}, {124, 132, 30, 2693}, {125, 133, 15, 2693}, {126, 136, 20, 2693}, {127, 137, 60, 2693}, {128, 138, 60, 2693}, {129, 139, 20, 2693}, {130, 142, 15, 2693}, {131, 143, 60, 2693}, {132, 144, 90, 2693}, {133, 145, 60, 2693}, {134, 146, 15, 2693}, {135, 149, 6, 2693}, {136, 150, 30, 2693}, {137, 151, 60, 2693}, {138, 152, 60, 2693}, {139, 153, 30, 2693}, {140, 154, 6, 2693}, {141, 157, 1, 2693}, {142, 158, 6, 2693}, {143, 159, 15, 2693}, {144, 160, 20, 2693}, {145, 161, 15, 2693}, {146, 162, 6, 2693}, {147, 163, 1, 2693}, {120, 125, 1, 2694}, {121, 128, 6, 2694}, {122, 129, 6, 2694}, {123, 132, 15, 2694}, {124, 133, 30, 2694}, {125, 134, 15, 2694}, {126, 137, 20, 2694}, {127, 138, 60, 2694}, {128, 139, 60, 2694}, {129, 140, 20, 2694}, {130, 143, 15, 2694}, {131, 144, 60, 2694}, {132, 145, 90, 2694}, {133, 146, 60, 2694}, {134, 147, 15, 2694}, {135, 150, 6, 2694}, {136, 151, 30, 2694}, {137, 152, 60, 2694}, {138, 153, 60, 2694}, {139, 154, 30, 2694}, {140, 155, 6, 2694}, {141, 158, 1, 2694}, {142, 159, 6, 2694}, {143, 160, 15, 2694}, {144, 161, 20, 2694}, {145, 162, 15, 2694}, {146, 163, 6, 2694}, {147, 164, 1, 2694}, {121, 121, 1, 2695}, {123, 123, 6, 2695}, {124, 124, 6, 2695}, {126, 126, 15, 2695}, {127, 127, 30, 2695}, {128, 128, 15, 2695}, {130, 130, 20, 2695}, {131, 131, 60, 2695}, {132, 132, 60, 2695}, {133, 133, 20, 2695}, {135, 135, 15, 2695}, {136, 136, 60, 2695}, {137, 137, 90, 2695}, {138, 138, 60, 2695}, {139, 139, 15, 2695}, {141, 141, 6, 2695}, {142, 142, 30, 2695}, {143, 143, 60, 2695}, {144, 144, 60, 2695}, {145, 145, 30, 2695}, {146, 146, 6, 2695}, {148, 148, 1, 2695}, {149, 149, 6, 2695}, {150, 150, 15, 2695}, {151, 151, 20, 2695}, {152, 152, 15, 2695}, {153, 153, 6, 2695}, {154, 154, 1, 2695}, {121, 122, 1, 2696}, {123, 124, 6, 2696}, {124, 125, 6, 2696}, {126, 127, 15, 2696}, {127, 128, 30, 2696}, {128, 129, 15, 2696}, {130, 131, 20, 2696}, {131, 132, 60, 2696}, {132, 133, 60, 2696}, {133, 134, 20, 2696}, {135, 136, 15, 2696}, {136, 137, 60, 2696}, {137, 138, 90, 2696}, {138, 139, 60, 2696}, {139, 140, 15, 2696}, {141, 142, 6, 2696}, {142, 143, 30, 2696}, {143, 144, 60, 2696}, {144, 145, 60, 2696}, {145, 146, 30, 2696}, {146, 147, 6, 2696}, {148, 149, 1, 2696}, {149, 150, 6, 2696}, {150, 151, 15, 2696}, {151, 152, 20, 2696}, {152, 153, 15, 2696}, {153, 154, 6, 2696}, {154, 155, 1, 2696}, {121, 123, 1, 2697}, {123, 126, 6, 2697}, {124, 127, 6, 2697}, {126, 130, 15, 2697}, {127, 131, 30, 2697}, {128, 132, 15, 2697}, {130, 135, 20, 2697}, {131, 136, 60, 2697}, {132, 137, 60, 2697}, {133, 138, 20, 2697}, {135, 141, 15, 2697}, {136, 142, 60, 2697}, {137, 143, 90, 2697}, {138, 144, 60, 2697}, {139, 145, 15, 2697}, {141, 148, 6, 2697}, {142, 149, 30, 2697}, {143, 150, 60, 2697}, {144, 151, 60, 2697}, {145, 152, 30, 2697}, {146, 153, 6, 2697}, {148, 156, 1, 2697}, {149, 157, 6, 2697}, {150, 158, 15, 2697}, {151, 159, 20, 2697}, {152, 160, 15, 2697}, {153, 161, 6, 2697}, {154, 162, 1, 2697}, {121, 124, 1, 2698}, {123, 127, 6, 2698}, {124, 128, 6, 2698}, {126, 131, 15, 2698}, {127, 132, 30, 2698}, {128, 133, 15, 2698}, {130, 136, 20, 2698}, {131, 137, 60, 2698}, {132, 138, 60, 2698}, {133, 139, 20, 2698}, {135, 142, 15, 2698}, {136, 143, 60,

2698}, {137, 144, 90, 2698}, {138, 145, 60, 2698}, {139, 146, 15, 2698}, {141, 149, 6,
2698}, {142, 150, 30, 2698}, {143, 151, 60, 2698}, {144, 152, 60, 2698}, {145, 153, 30,
2698}, {146, 154, 6, 2698}, {148, 157, 1, 2698}, {149, 158, 6, 2698}, {150, 159, 15,
2698}, {151, 160, 20, 2698}, {152, 161, 15, 2698}, {153, 162, 6, 2698}, {154, 163, 1,
2698}, {121, 125, 1, 2699}, {123, 128, 6, 2699}, {124, 129, 6, 2699}, {126, 132, 15,
2699}, {127, 133, 30, 2699}, {128, 134, 15, 2699}, {130, 137, 20, 2699}, {131, 138, 60,
2699}, {132, 139, 60, 2699}, {133, 140, 20, 2699}, {135, 143, 15, 2699}, {136, 144, 60,
2699}, {137, 145, 90, 2699}, {138, 146, 60, 2699}, {139, 147, 15, 2699}, {141, 150, 6,
2699}, {142, 151, 30, 2699}, {143, 152, 60, 2699}, {144, 153, 60, 2699}, {145, 154, 30,
2699}, {146, 155, 6, 2699}, {148, 158, 1, 2699}, {149, 159, 6, 2699}, {150, 160, 15,
2699}, {151, 161, 20, 2699}, {152, 162, 15, 2699}, {153, 163, 6, 2699}, {154, 164, 1,
2699}, {122, 122, 1, 2700}, {124, 124, 6, 2700}, {125, 125, 6, 2700}, {127, 127, 15,
2700}, {128, 128, 30, 2700}, {129, 129, 15, 2700}, {131, 131, 20, 2700}, {132, 132, 60,
2700}, {133, 133, 60, 2700}, {134, 134, 20, 2700}, {136, 136, 15, 2700}, {137, 137, 60,
2700}, {138, 138, 90, 2700}, {139, 139, 60, 2700}, {140, 140, 15, 2700}, {142, 142, 6,
2700}, {143, 143, 30, 2700}, {144, 144, 60, 2700}, {145, 145, 60, 2700}, {146, 146, 30,
2700}, {147, 147, 6, 2700}, {149, 149, 1, 2700}, {150, 150, 6, 2700}, {151, 151, 15,
2700}, {152, 152, 20, 2700}, {153, 153, 15, 2700}, {154, 154, 6, 2700}, {155, 155, 1,
2700}, {122, 123, 1, 2701}, {124, 126, 6, 2701}, {125, 127, 6, 2701}, {127, 130, 15,
2701}, {128, 131, 30, 2701}, {129, 132, 15, 2701}, {131, 135, 20, 2701}, {132, 136, 60,
2701}, {133, 137, 60, 2701}, {134, 138, 20, 2701}, {136, 141, 15, 2701}, {137, 142, 60,
2701}, {138, 143, 90, 2701}, {139, 144, 60, 2701}, {140, 145, 15, 2701}, {142, 148, 6,
2701}, {143, 149, 30, 2701}, {144, 150, 60, 2701}, {145, 151, 60, 2701}, {146, 152, 30,
2701}, {147, 153, 6, 2701}, {149, 156, 1, 2701}, {150, 157, 6, 2701}, {151, 158, 15,
2701}, {152, 159, 20, 2701}, {153, 160, 15, 2701}, {154, 161, 6, 2701}, {155, 162, 1,
2701}, {122, 124, 1, 2702}, {124, 127, 6, 2702}, {125, 128, 6, 2702}, {127, 131, 15,
2702}, {128, 132, 30, 2702}, {129, 133, 15, 2702}, {131, 136, 20, 2702}, {132, 137, 60,
2702}, {133, 138, 60, 2702}, {134, 139, 20, 2702}, {136, 142, 15, 2702}, {137, 143, 60,
2702}, {138, 144, 90, 2702}, {139, 145, 60, 2702}, {140, 146, 15, 2702}, {142, 149, 6,
2702}, {143, 150, 30, 2702}, {144, 151, 60, 2702}, {145, 152, 60, 2702}, {146, 153, 30,
2702}, {147, 154, 6, 2702}, {149, 157, 1, 2702}, {150, 158, 6, 2702}, {151, 159, 15,
2702}, {152, 160, 20, 2702}, {153, 161, 15, 2702}, {154, 162, 6, 2702}, {155, 163, 1,
2702}, {122, 125, 1, 2703}, {124, 128, 6, 2703}, {125, 129, 6, 2703}, {127, 132, 15,
2703}, {128, 133, 30, 2703}, {129, 134, 15, 2703}, {131, 137, 20, 2703}, {132, 138, 60,
2703}, {133, 139, 60, 2703}, {134, 140, 20, 2703}, {136, 143, 15, 2703}, {137, 144, 60,
2703}, {138, 145, 90, 2703}, {139, 146, 60, 2703}, {140, 147, 15, 2703}, {142, 150, 6,
2703}, {143, 151, 30, 2703}, {144, 152, 60, 2703}, {145, 153, 60, 2703}, {146, 154, 30,
2703}, {147, 155, 6, 2703}, {149, 158, 1, 2703}, {150, 159, 6, 2703}, {151, 160, 15,
2703}, {152, 161, 20, 2703}, {153, 162, 15, 2703}, {154, 163, 6, 2703}, {155, 164, 1,
2703}, {123, 123, 1, 2704}, {126, 126, 6, 2704}, {127, 127, 6, 2704}, {130, 130, 15,
2704}, {131, 131, 30, 2704}, {132, 132, 15, 2704}, {135, 135, 20, 2704}, {136, 136, 60,
2704}, {137, 137, 60, 2704}, {138, 138, 20, 2704}, {141, 141, 15, 2704}, {142, 142, 60,
2704}, {143, 143, 90, 2704}, {144, 144, 60, 2704}, {145, 145, 15, 2704}, {148, 148, 6,
2704}, {149, 149, 30, 2704}, {150, 150, 60, 2704}, {151, 151, 60, 2704}, {152, 152, 30,
2704}, {153, 153, 6, 2704}, {156, 156, 1, 2704}, {157, 157, 6, 2704}, {158, 158, 15,
2704}, {159, 159, 20, 2704}, {160, 160, 15, 2704}, {161, 161, 6, 2704}, {162, 162, 1,
2704}, {123, 124, 1, 2705}, {126, 127, 6, 2705}, {127, 128, 6, 2705}, {130, 131, 15,
2705}, {131, 132, 30, 2705}, {132, 133, 15, 2705}, {135, 136, 20, 2705}, {136, 137, 60,
2705}, {137, 138, 60, 2705}, {138, 139, 20, 2705}, {141, 142, 15, 2705}, {142, 143, 60,
2705}, {143, 144, 90, 2705}, {144, 145, 60, 2705}, {145, 146, 15, 2705}, {148, 149, 6,
2705}, {149, 150, 30, 2705}, {150, 151, 60, 2705}, {151, 152, 60, 2705}, {152, 153, 30,
2705}, {153, 154, 6, 2705}, {156, 157, 1, 2705}, {157, 158, 6, 2705}, {158, 159, 15,
2705}, {159, 160, 20, 2705}, {160, 161, 15, 2705}, {161, 162, 6, 2705}, {162, 163, 1,
2705}, {123, 125, 1, 2706}, {126, 128, 6, 2706}, {127, 129, 6, 2706}, {130, 132, 15,
2706}, {131, 133, 30, 2706}, {132, 134, 15, 2706}, {135, 137, 20, 2706}, {136, 138, 60,
2706}, {137, 139, 60, 2706}, {138, 140, 20, 2706}, {141, 143, 15, 2706}, {142, 144, 60,
2706}, {143, 145, 90, 2706}, {144, 146, 60, 2706}, {145, 147, 15, 2706}, {148, 150, 6,
2706}, {149, 151, 30, 2706}, {150, 152, 60, 2706}, {151, 153, 60, 2706}, {152, 154, 30,
2706}, {153, 155, 6, 2706}, {156, 158, 1, 2706}, {157, 159, 6, 2706}, {158, 160, 15,
2706}, {159, 161, 20, 2706}, {160, 162, 15, 2706}, {161, 163, 6, 2706}, {162, 164, 1,
2706}, {124, 124, 1, 2707}, {127, 127, 6, 2707}, {128, 128, 6, 2707}, {131, 131, 15,
2707}, {132, 132, 30, 2707}, {133, 133, 15, 2707}, {136, 136, 20, 2707}, {137, 137, 60,
2707}, {138, 138, 60, 2707}, {139, 139, 20, 2707}, {142, 142, 15, 2707}, {143, 143, 60,
2707}, {144, 144, 90, 2707}, {145, 145, 60, 2707}, {146, 146, 15, 2707}, {149, 149, 6,
2707}, {150, 150, 30, 2707}, {151, 151, 60, 2707}, {152, 152, 60, 2707}, {153, 153, 30,
2707}, {154, 154, 6, 2707}, {157, 157, 1, 2707}, {158, 158, 6, 2707}, {159, 159, 15,
2707}, {160, 160, 20, 2707}, {161, 161, 15, 2707}, {162, 162, 6, 2707}, {163, 163, 1,
2707}, {124, 125, 1, 2708}, {127, 128, 6, 2708}, {128, 129, 6, 2708}, {131, 132, 15,
2708}, {132, 133, 30, 2708}, {133, 134, 15, 2708}, {136, 137, 20, 2708}, {137, 138, 60,

2708}, {138, 139, 60, 2708}, {139, 140, 20, 2708}, {142, 143, 15, 2708}, {143, 144, 60, 2708}, {144, 145, 90, 2708}, {145, 146, 60, 2708}, {146, 147, 15, 2708}, {149, 150, 6, 2708}, {150, 151, 30, 2708}, {151, 152, 60, 2708}, {152, 153, 60, 2708}, {153, 154, 30, 2708}, {154, 155, 6, 2708}, {157, 158, 1, 2708}, {158, 159, 6, 2708}, {159, 160, 15, 2708}, {160, 161, 20, 2708}, {161, 162, 15, 2708}, {162, 163, 6, 2708}, {163, 164, 1, 2708}, {125, 125, 1, 2709}, {128, 128, 6, 2709}, {129, 129, 6, 2709}, {132, 132, 15, 2709}, {133, 133, 30, 2709}, {134, 134, 15, 2709}, {137, 137, 20, 2709}, {138, 138, 60, 2709}, {139, 139, 60, 2709}, {140, 140, 20, 2709}, {143, 143, 15, 2709}, {144, 144, 60, 2709}, {145, 145, 90, 2709}, {146, 146, 60, 2709}, {147, 147, 15, 2709}, {150, 150, 6, 2709}, {151, 151, 30, 2709}, {152, 152, 60, 2709}, {153, 153, 60, 2709}, {154, 154, 30, 2709}, {155, 155, 6, 2709}, {158, 158, 1, 2709}, {159, 159, 6, 2709}, {160, 160, 15, 2709}, {161, 161, 20, 2709}, {162, 162, 15, 2709}, {163, 163, 6, 2709}, {164, 164, 1, 2709}, {120, 120, 1, 2710}, {121, 121, 7, 2710}, {122, 122, 7, 2710}, {123, 123, 21, 2710}, {124, 124, 42, 2710}, {125, 125, 21, 2710}, {126, 126, 35, 2710}, {127, 127, 105, 2710}, {128, 128, 105, 2710}, {129, 129, 35, 2710}, {130, 130, 35, 2710}, {131, 131, 140, 2710}, {132, 132, 210, 2710}, {133, 133, 140, 2710}, {134, 134, 35, 2710}, {135, 135, 21, 2710}, {136, 136, 105, 2710}, {137, 137, 210, 2710}, {138, 138, 210, 2710}, {139, 139, 105, 2710}, {140, 140, 21, 2710}, {141, 141, 7, 2710}, {142, 142, 42, 2710}, {143, 143, 105, 2710}, {144, 144, 140, 2710}, {145, 145, 105, 2710}, {146, 146, 42, 2710}, {147, 147, 7, 2710}, {148, 148, 1, 2710}, {149, 149, 7, 2710}, {150, 150, 21, 2710}, {151, 151, 35, 2710}, {152, 152, 35, 2710}, {153, 153, 21, 2710}, {154, 154, 7, 2710}, {155, 155, 1, 2710}, {120, 121, 1, 2711}, {121, 123, 7, 2711}, {122, 124, 7, 2711}, {123, 126, 21, 2711}, {124, 127, 42, 2711}, {125, 128, 21, 2711}, {126, 130, 35, 2711}, {127, 131, 105, 2711}, {128, 132, 105, 2711}, {129, 133, 35, 2711}, {130, 135, 35, 2711}, {131, 136, 140, 2711}, {132, 137, 210, 2711}, {133, 138, 140, 2711}, {134, 139, 35, 2711}, {135, 141, 21, 2711}, {136, 142, 105, 2711}, {137, 143, 210, 2711}, {138, 144, 210, 2711}, {139, 145, 105, 2711}, {140, 146, 21, 2711}, {141, 148, 7, 2711}, {142, 149, 42, 2711}, {143, 150, 105, 2711}, {144, 151, 140, 2711}, {145, 152, 105, 2711}, {146, 153, 42, 2711}, {147, 154, 7, 2711}, {148, 156, 1, 2711}, {149, 157, 7, 2711}, {150, 158, 21, 2711}, {151, 159, 35, 2711}, {152, 160, 35, 2711}, {153, 161, 21, 2711}, {154, 162, 7, 2711}, {155, 163, 1, 2711}, {120, 122, 1, 2712}, {121, 124, 7, 2712}, {122, 125, 7, 2712}, {123, 127, 21, 2712}, {124, 128, 42, 2712}, {125, 129, 21, 2712}, {126, 131, 35, 2712}, {127, 132, 105, 2712}, {128, 133, 105, 2712}, {129, 134, 35, 2712}, {130, 136, 35, 2712}, {131, 137, 140, 2712}, {132, 138, 210, 2712}, {133, 139, 140, 2712}, {134, 140, 35, 2712}, {135, 142, 21, 2712}, {136, 143, 105, 2712}, {137, 144, 210, 2712}, {138, 145, 210, 2712}, {139, 146, 105, 2712}, {140, 147, 21, 2712}, {141, 149, 7, 2712}, {142, 150, 42, 2712}, {143, 151, 105, 2712}, {144, 152, 140, 2712}, {145, 153, 105, 2712}, {146, 154, 42, 2712}, {147, 155, 7, 2712}, {148, 157, 1, 2712}, {149, 158, 7, 2712}, {150, 159, 21, 2712}, {151, 160, 35, 2712}, {152, 161, 35, 2712}, {153, 162, 21, 2712}, {154, 163, 7, 2712}, {155, 164, 1, 2712}, {121, 121, 1, 2713}, {123, 123, 7, 2713}, {124, 124, 7, 2713}, {126, 126, 21, 2713}, {127, 127, 42, 2713}, {128, 128, 21, 2713}, {130, 130, 35, 2713}, {131, 131, 105, 2713}, {132, 132, 105, 2713}, {133, 133, 35, 2713}, {135, 135, 35, 2713}, {136, 136, 140, 2713}, {137, 137, 210, 2713}, {138, 138, 140, 2713}, {139, 139, 35, 2713}, {141, 141, 21, 2713}, {142, 142, 105, 2713}, {143, 143, 210, 2713}, {144, 144, 210, 2713}, {145, 145, 105, 2713}, {146, 146, 21, 2713}, {148, 148, 7, 2713}, {149, 149, 42, 2713}, {150, 150, 105, 2713}, {151, 151, 140, 2713}, {152, 152, 105, 2713}, {153, 153, 42, 2713}, {154, 154, 7, 2713}, {156, 156, 1, 2713}, {157, 157, 7, 2713}, {158, 158, 21, 2713}, {159, 159, 35, 2713}, {160, 160, 35, 2713}, {161, 161, 21, 2713}, {162, 162, 7, 2713}, {163, 163, 1, 2713}, {121, 122, 1, 2714}, {123, 124, 7, 2714}, {124, 125, 7, 2714}, {126, 127, 21, 2714}, {127, 128, 42, 2714}, {128, 129, 21, 2714}, {130, 131, 35, 2714}, {131, 132, 105, 2714}, {132, 133, 105, 2714}, {133, 134, 35, 2714}, {135, 136, 35, 2714}, {136, 137, 140, 2714}, {137, 138, 210, 2714}, {138, 139, 140, 2714}, {139, 140, 35, 2714}, {141, 142, 21, 2714}, {142, 143, 105, 2714}, {143, 144, 210, 2714}, {144, 145, 210, 2714}, {145, 146, 105, 2714}, {146, 147, 21, 2714}, {148, 149, 7, 2714}, {149, 150, 42, 2714}, {150, 151, 105, 2714}, {151, 152, 140, 2714}, {152, 153, 105, 2714}, {153, 154, 42, 2714}, {154, 155, 7, 2714}, {156, 157, 1, 2714}, {157, 158, 7, 2714}, {158, 159, 21, 2714}, {159, 160, 35, 2714}, {160, 161, 35, 2714}, {161, 162, 21, 2714}, {162, 163, 7, 2714}, {163, 164, 1, 2714}, {122, 122, 1, 2715}, {124, 124, 7, 2715}, {125, 125, 7, 2715}, {127, 127, 21, 2715}, {128, 128, 42, 2715}, {129, 129, 21, 2715}, {131, 131, 35, 2715}, {132, 132, 105, 2715}, {133, 133, 105, 2715}, {134, 134, 35, 2715}, {136, 136, 35, 2715}, {137, 137, 140, 2715}, {138, 138, 210, 2715}, {139, 139, 140, 2715}, {140, 140, 35, 2715}, {142, 142, 21, 2715}, {143, 143, 105, 2715}, {144, 144, 210, 2715}, {145, 145, 210, 2715}, {146, 146, 105, 2715}, {147, 147, 21, 2715}, {149, 149, 7, 2715}, {150, 150, 42, 2715}, {151, 151, 105, 2715}, {152, 152, 140, 2715}, {153, 153, 105, 2715}, {154, 154, 42, 2715}, {155, 155, 7, 2715}, {157, 157, 1, 2715}, {158, 158, 7, 2715}, {159, 159, 21, 2715}, {160, 160, 35, 2715}, {161, 161, 35, 2715}, {162, 162, 21, 2715}, {163, 163, 7, 2715}, {164, 164, 1, 2715}, {120, 165, 1, 2716}, {121, 166, 7, 2716}, {122, 167, 7, 2716}, {123, 168, 21, 2716}, {124, 169, 42, 2716}, {125, 170, 21, 2716}, {126, 171, 35, 2716}, {127, 172, 105, 2716}, {128, 173, 105, 2716}, {129, 174, 35, 2716}, {130, 175, 35, 2716}, {131, 176, 140,

2716}, {132, 177, 210, 2716}, {133, 178, 140, 2716}, {134, 179, 35, 2716}, {135, 180, 21, 2716}, {136, 181, 105, 2716}, {137, 182, 210, 2716}, {138, 183, 210, 2716}, {139, 184, 105, 2716}, {140, 185, 21, 2716}, {141, 186, 7, 2716}, {142, 187, 42, 2716}, {143, 188, 105, 2716}, {144, 189, 140, 2716}, {145, 190, 105, 2716}, {146, 191, 42, 2716}, {147, 192, 7, 2716}, {148, 193, 1, 2716}, {149, 194, 7, 2716}, {150, 195, 21, 2716}, {151, 196, 35, 2716}, {152, 197, 35, 2716}, {153, 198, 21, 2716}, {154, 199, 7, 2716}, {155, 200, 1, 2716}, {120, 166, 1, 2717}, {121, 168, 7, 2717}, {122, 169, 7, 2717}, {123, 171, 21, 2717}, {124, 172, 42, 2717}, {125, 173, 21, 2717}, {126, 175, 35, 2717}, {127, 176, 105, 2717}, {128, 177, 105, 2717}, {129, 178, 35, 2717}, {130, 180, 35, 2717}, {131, 181, 140, 2717}, {132, 182, 210, 2717}, {133, 183, 140, 2717}, {134, 184, 35, 2717}, {135, 186, 21, 2717}, {136, 187, 105, 2717}, {137, 188, 210, 2717}, {138, 189, 210, 2717}, {139, 190, 105, 2717}, {140, 191, 21, 2717}, {141, 193, 7, 2717}, {142, 194, 42, 2717}, {143, 195, 105, 2717}, {144, 196, 140, 2717}, {145, 197, 105, 2717}, {146, 198, 42, 2717}, {147, 199, 7, 2717}, {148, 201, 1, 2717}, {149, 202, 7, 2717}, {150, 203, 21, 2717}, {151, 204, 35, 2717}, {152, 205, 35, 2717}, {153, 206, 21, 2717}, {154, 207, 7, 2717}, {155, 208, 1, 2717}, {120, 167, 1, 2718}, {121, 169, 7, 2718}, {122, 170, 7, 2718}, {123, 172, 21, 2718}, {124, 173, 42, 2718}, {125, 174, 21, 2718}, {126, 176, 35, 2718}, {127, 177, 105, 2718}, {128, 178, 105, 2718}, {129, 179, 35, 2718}, {130, 181, 35, 2718}, {131, 182, 140, 2718}, {132, 183, 210, 2718}, {133, 184, 140, 2718}, {134, 185, 35, 2718}, {135, 187, 21, 2718}, {136, 188, 105, 2718}, {137, 189, 210, 2718}, {138, 190, 210, 2718}, {139, 191, 105, 2718}, {140, 192, 21, 2718}, {141, 194, 7, 2718}, {142, 195, 42, 2718}, {143, 196, 105, 2718}, {144, 197, 140, 2718}, {145, 198, 105, 2718}, {146, 199, 42, 2718}, {147, 200, 7, 2718}, {148, 202, 1, 2718}, {149, 203, 7, 2718}, {150, 204, 21, 2718}, {151, 205, 35, 2718}, {152, 206, 35, 2718}, {153, 207, 21, 2718}, {154, 208, 7, 2718}, {155, 209, 1, 2718}, {120, 168, 1, 2719}, {121, 171, 7, 2719}, {122, 172, 7, 2719}, {123, 175, 21, 2719}, {124, 176, 42, 2719}, {125, 177, 21, 2719}, {126, 180, 35, 2719}, {127, 181, 105, 2719}, {128, 182, 105, 2719}, {129, 183, 35, 2719}, {130, 186, 35, 2719}, {131, 187, 140, 2719}, {132, 188, 210, 2719}, {133, 189, 140, 2719}, {134, 190, 35, 2719}, {135, 193, 21, 2719}, {136, 194, 105, 2719}, {137, 195, 210, 2719}, {138, 196, 210, 2719}, {139, 197, 105, 2719}, {140, 198, 21, 2719}, {141, 201, 7, 2719}, {142, 202, 42, 2719}, {143, 203, 105, 2719}, {144, 204, 140, 2719}, {145, 205, 105, 2719}, {146, 206, 42, 2719}, {147, 207, 7, 2719}, {148, 210, 1, 2719}, {149, 211, 7, 2719}, {150, 212, 21, 2719}, {151, 213, 35, 2719}, {152, 214, 35, 2719}, {153, 215, 21, 2719}, {154, 216, 7, 2719}, {155, 217, 1, 2719}, {120, 169, 1, 2720}, {121, 172, 7, 2720}, {122, 173, 7, 2720}, {123, 176, 21, 2720}, {124, 177, 42, 2720}, {125, 178, 21, 2720}, {126, 181, 35, 2720}, {127, 182, 105, 2720}, {128, 183, 105, 2720}, {129, 184, 35, 2720}, {130, 187, 35, 2720}, {131, 188, 140, 2720}, {132, 189, 210, 2720}, {133, 190, 140, 2720}, {134, 191, 35, 2720}, {135, 194, 21, 2720}, {136, 195, 105, 2720}, {137, 196, 210, 2720}, {138, 197, 210, 2720}, {139, 198, 105, 2720}, {140, 199, 21, 2720}, {141, 202, 7, 2720}, {142, 203, 42, 2720}, {143, 204, 105, 2720}, {144, 205, 140, 2720}, {145, 206, 105, 2720}, {146, 207, 42, 2720}, {147, 208, 7, 2720}, {148, 211, 1, 2720}, {149, 212, 7, 2720}, {150, 213, 21, 2720}, {151, 214, 35, 2720}, {152, 215, 35, 2720}, {153, 216, 21, 2720}, {154, 217, 7, 2720}, {155, 218, 1, 2720}, {120, 170, 1, 2721}, {121, 173, 7, 2721}, {122, 174, 7, 2721}, {123, 177, 21, 2721}, {124, 178, 42, 2721}, {125, 179, 21, 2721}, {126, 182, 35, 2721}, {127, 183, 105, 2721}, {128, 184, 105, 2721}, {129, 185, 35, 2721}, {130, 188, 35, 2721}, {131, 189, 140, 2721}, {132, 190, 210, 2721}, {133, 191, 140, 2721}, {134, 192, 35, 2721}, {135, 195, 21, 2721}, {136, 196, 105, 2721}, {137, 197, 210, 2721}, {138, 198, 210, 2721}, {139, 199, 105, 2721}, {140, 200, 21, 2721}, {141, 203, 7, 2721}, {142, 204, 42, 2721}, {143, 205, 105, 2721}, {144, 206, 140, 2721}, {145, 207, 105, 2721}, {146, 208, 42, 2721}, {147, 209, 7, 2721}, {148, 212, 1, 2721}, {149, 213, 7, 2721}, {150, 214, 21, 2721}, {151, 215, 35, 2721}, {152, 216, 35, 2721}, {153, 217, 21, 2721}, {154, 218, 7, 2721}, {155, 219, 1, 2721}, {121, 165, 1, 2722}, {123, 166, 7, 2722}, {124, 167, 7, 2722}, {126, 168, 21, 2722}, {127, 169, 42, 2722}, {128, 170, 21, 2722}, {130, 171, 35, 2722}, {131, 172, 105, 2722}, {132, 173, 105, 2722}, {133, 174, 35, 2722}, {135, 175, 35, 2722}, {136, 176, 140, 2722}, {137, 177, 210, 2722}, {138, 178, 140, 2722}, {139, 179, 35, 2722}, {141, 180, 21, 2722}, {142, 181, 105, 2722}, {143, 182, 210, 2722}, {144, 183, 210, 2722}, {145, 184, 105, 2722}, {146, 185, 21, 2722}, {148, 186, 7, 2722}, {149, 187, 42, 2722}, {150, 188, 105, 2722}, {151, 189, 140, 2722}, {152, 190, 105, 2722}, {153, 191, 42, 2722}, {154, 192, 7, 2722}, {156, 193, 1, 2722}, {157, 194, 7, 2722}, {158, 195, 21, 2722}, {159, 196, 35, 2722}, {160, 197, 35, 2722}, {161, 198, 21, 2722}, {162, 199, 7, 2722}, {163, 200, 1, 2722}, {121, 166, 1, 2723}, {123, 168, 7, 2723}, {124, 169, 7, 2723}, {126, 171, 21, 2723}, {127, 172, 42, 2723}, {128, 173, 21, 2723}, {130, 175, 35, 2723}, {131, 176, 105, 2723}, {132, 177, 105, 2723}, {133, 178, 35, 2723}, {135, 180, 35, 2723}, {136, 181, 140, 2723}, {137, 182, 210, 2723}, {138, 183, 140, 2723}, {139, 184, 35, 2723}, {141, 186, 21, 2723}, {142, 187, 105, 2723}, {143, 188, 210, 2723}, {144, 189, 210, 2723}, {145, 190, 105, 2723}, {146, 191, 21, 2723}, {148, 193, 7, 2723}, {149, 194, 42, 2723}, {150, 195, 105, 2723}, {151, 196, 140, 2723}, {152, 197, 105, 2723}, {153, 198, 42, 2723}, {154, 199, 7, 2723}, {156, 201, 1, 2723}, {157, 202, 7, 2723}, {158, 203, 21, 2723}, {159, 204, 35, 2723}, {160, 205, 35, 2723}, {161, 206, 21, 2723}, {162, 207, 7, 2723}, {163, 208, 1,

2723}, {121, 167, 1, 2724}, {123, 169, 7, 2724}, {124, 170, 7, 2724}, {126, 172, 21, 2724}, {127, 173, 42, 2724}, {128, 174, 21, 2724}, {130, 176, 35, 2724}, {131, 177, 105, 2724}, {132, 178, 105, 2724}, {133, 179, 35, 2724}, {135, 181, 35, 2724}, {136, 182, 140, 2724}, {137, 183, 210, 2724}, {138, 184, 140, 2724}, {139, 185, 35, 2724}, {141, 187, 21, 2724}, {142, 188, 105, 2724}, {143, 189, 210, 2724}, {144, 190, 210, 2724}, {145, 191, 105, 2724}, {146, 192, 21, 2724}, {148, 194, 7, 2724}, {149, 195, 42, 2724}, {150, 196, 105, 2724}, {151, 197, 140, 2724}, {152, 198, 105, 2724}, {153, 199, 42, 2724}, {154, 200, 7, 2724}, {156, 202, 1, 2724}, {157, 203, 7, 2724}, {158, 204, 21, 2724}, {159, 205, 35, 2724}, {160, 206, 35, 2724}, {161, 207, 21, 2724}, {162, 208, 7, 2724}, {163, 209, 1, 2724}, {121, 168, 1, 2725}, {123, 171, 7, 2725}, {124, 172, 7, 2725}, {126, 175, 21, 2725}, {127, 176, 42, 2725}, {128, 177, 21, 2725}, {130, 180, 35, 2725}, {131, 181, 105, 2725}, {132, 182, 105, 2725}, {133, 183, 35, 2725}, {135, 186, 35, 2725}, {136, 187, 140, 2725}, {137, 188, 210, 2725}, {138, 189, 140, 2725}, {139, 190, 35, 2725}, {141, 193, 21, 2725}, {142, 194, 105, 2725}, {143, 195, 210, 2725}, {144, 196, 210, 2725}, {145, 197, 105, 2725}, {146, 198, 21, 2725}, {148, 201, 7, 2725}, {149, 202, 42, 2725}, {150, 203, 105, 2725}, {151, 204, 140, 2725}, {152, 205, 105, 2725}, {153, 206, 42, 2725}, {154, 207, 7, 2725}, {156, 210, 1, 2725}, {157, 211, 7, 2725}, {158, 212, 21, 2725}, {159, 213, 35, 2725}, {160, 214, 35, 2725}, {161, 215, 21, 2725}, {162, 216, 7, 2725}, {163, 217, 1, 2725}, {121, 169, 1, 2726}, {123, 172, 7, 2726}, {124, 173, 7, 2726}, {126, 176, 21, 2726}, {127, 177, 42, 2726}, {128, 178, 21, 2726}, {130, 181, 35, 2726}, {131, 182, 105, 2726}, {132, 183, 105, 2726}, {133, 184, 35, 2726}, {135, 187, 35, 2726}, {136, 188, 140, 2726}, {137, 189, 210, 2726}, {138, 190, 140, 2726}, {139, 191, 35, 2726}, {141, 194, 21, 2726}, {142, 195, 105, 2726}, {143, 196, 210, 2726}, {144, 197, 210, 2726}, {145, 198, 105, 2726}, {146, 199, 21, 2726}, {148, 202, 7, 2726}, {149, 203, 42, 2726}, {150, 204, 105, 2726}, {151, 205, 140, 2726}, {152, 206, 105, 2726}, {153, 207, 42, 2726}, {154, 208, 7, 2726}, {156, 211, 1, 2726}, {157, 212, 7, 2726}, {158, 213, 21, 2726}, {159, 214, 35, 2726}, {160, 215, 35, 2726}, {161, 216, 21, 2726}, {162, 217, 7, 2726}, {163, 218, 1, 2726}, {121, 170, 1, 2727}, {123, 173, 7, 2727}, {124, 174, 7, 2727}, {126, 177, 21, 2727}, {127, 178, 42, 2727}, {128, 179, 21, 2727}, {130, 182, 35, 2727}, {131, 183, 105, 2727}, {132, 184, 105, 2727}, {133, 185, 35, 2727}, {135, 188, 35, 2727}, {136, 189, 140, 2727}, {137, 190, 210, 2727}, {138, 191, 140, 2727}, {139, 192, 35, 2727}, {141, 195, 21, 2727}, {142, 196, 105, 2727}, {143, 197, 210, 2727}, {144, 198, 210, 2727}, {145, 199, 105, 2727}, {146, 200, 21, 2727}, {148, 203, 7, 2727}, {149, 204, 42, 2727}, {150, 205, 105, 2727}, {151, 206, 140, 2727}, {152, 207, 105, 2727}, {153, 208, 42, 2727}, {154, 209, 7, 2727}, {156, 212, 1, 2727}, {157, 213, 7, 2727}, {158, 214, 21, 2727}, {159, 215, 35, 2727}, {160, 216, 35, 2727}, {161, 217, 21, 2727}, {162, 218, 7, 2727}, {163, 219, 1, 2727}, {122, 165, 1, 2728}, {124, 166, 7, 2728}, {125, 167, 7, 2728}, {127, 168, 21, 2728}, {128, 169, 42, 2728}, {129, 170, 21, 2728}, {131, 171, 35, 2728}, {132, 172, 105, 2728}, {133, 173, 105, 2728}, {134, 174, 35, 2728}, {136, 175, 35, 2728}, {137, 176, 140, 2728}, {138, 177, 210, 2728}, {139, 178, 140, 2728}, {140, 179, 35, 2728}, {142, 180, 21, 2728}, {143, 181, 105, 2728}, {144, 182, 210, 2728}, {145, 183, 210, 2728}, {146, 184, 105, 2728}, {147, 185, 21, 2728}, {149, 186, 7, 2728}, {150, 187, 42, 2728}, {151, 188, 105, 2728}, {152, 189, 140, 2728}, {153, 190, 105, 2728}, {154, 191, 42, 2728}, {155, 192, 7, 2728}, {157, 193, 1, 2728}, {158, 194, 7, 2728}, {159, 195, 21, 2728}, {160, 196, 35, 2728}, {161, 197, 35, 2728}, {162, 198, 21, 2728}, {163, 199, 7, 2728}, {164, 200, 1, 2728}, {122, 166, 1, 2729}, {124, 168, 7, 2729}, {125, 169, 7, 2729}, {127, 171, 21, 2729}, {128, 172, 42, 2729}, {129, 173, 21, 2729}, {131, 175, 35, 2729}, {132, 176, 105, 2729}, {133, 177, 105, 2729}, {134, 178, 35, 2729}, {136, 180, 35, 2729}, {137, 181, 140, 2729}, {138, 182, 210, 2729}, {139, 183, 140, 2729}, {140, 184, 35, 2729}, {142, 186, 21, 2729}, {143, 187, 105, 2729}, {144, 188, 210, 2729}, {145, 189, 210, 2729}, {146, 190, 105, 2729}, {147, 191, 21, 2729}, {149, 193, 7, 2729}, {150, 194, 42, 2729}, {151, 195, 105, 2729}, {152, 196, 140, 2729}, {153, 197, 105, 2729}, {154, 198, 42, 2729}, {155, 199, 7, 2729}, {157, 201, 1, 2729}, {158, 202, 7, 2729}, {159, 203, 21, 2729}, {160, 204, 35, 2729}, {161, 205, 35, 2729}, {162, 206, 21, 2729}, {163, 207, 7, 2729}, {164, 208, 1, 2729}, {122, 167, 1, 2730}, {124, 169, 7, 2730}, {125, 170, 7, 2730}, {127, 172, 21, 2730}, {128, 173, 42, 2730}, {129, 174, 21, 2730}, {131, 176, 35, 2730}, {132, 177, 105, 2730}, {133, 178, 105, 2730}, {134, 179, 35, 2730}, {136, 181, 35, 2730}, {137, 182, 140, 2730}, {138, 183, 210, 2730}, {139, 184, 140, 2730}, {140, 185, 35, 2730}, {142, 187, 21, 2730}, {143, 188, 105, 2730}, {144, 189, 210, 2730}, {145, 190, 210, 2730}, {146, 191, 105, 2730}, {147, 192, 21, 2730}, {149, 194, 7, 2730}, {150, 195, 42, 2730}, {151, 196, 105, 2730}, {152, 197, 140, 2730}, {153, 198, 105, 2730}, {154, 199, 42, 2730}, {155, 200, 7, 2730}, {157, 202, 1, 2730}, {158, 203, 7, 2730}, {159, 204, 21, 2730}, {160, 205, 35, 2730}, {161, 206, 35, 2730}, {162, 207, 21, 2730}, {163, 208, 7, 2730}, {164, 209, 1, 2730}, {122, 168, 1, 2731}, {124, 171, 7, 2731}, {125, 172, 7, 2731}, {127, 175, 21, 2731}, {128, 176, 42, 2731}, {129, 177, 21, 2731}, {131, 180, 35, 2731}, {132, 181, 105, 2731}, {133, 182, 105, 2731}, {134, 183, 35, 2731}, {136, 186, 35, 2731}, {137, 187, 140, 2731}, {138, 188, 210, 2731}, {139, 189, 140, 2731}, {140, 190, 35, 2731}, {142, 193, 21, 2731}, {143, 194, 105, 2731}, {144, 195, 210, 2731}, {145, 196, 210, 2731}, {146, 197, 105, 2731}, {147, 198, 21, 2731}, {149, 201, 7, 2731}, {150, 202, 42, 2731}, {151, 203,

105, 2731}, {152, 204, 140, 2731}, {153, 205, 105, 2731}, {154, 206, 42, 2731}, {155, 207, 7, 2731}, {157, 210, 1, 2731}, {158, 211, 7, 2731}, {159, 212, 21, 2731}, {160, 213, 35, 2731}, {161, 214, 35, 2731}, {162, 215, 21, 2731}, {163, 216, 7, 2731}, {164, 217, 1, 2731}, {122, 169, 1, 2732}, {124, 172, 7, 2732}, {125, 173, 7, 2732}, {127, 176, 21, 2732}, {128, 177, 42, 2732}, {129, 178, 21, 2732}, {131, 181, 35, 2732}, {132, 182, 105, 2732}, {133, 183, 105, 2732}, {134, 184, 35, 2732}, {136, 187, 35, 2732}, {137, 188, 140, 2732}, {138, 189, 210, 2732}, {139, 190, 140, 2732}, {140, 191, 35, 2732}, {142, 194, 21, 2732}, {143, 195, 105, 2732}, {144, 196, 210, 2732}, {145, 197, 210, 2732}, {146, 198, 105, 2732}, {147, 199, 21, 2732}, {149, 202, 7, 2732}, {150, 203, 42, 2732}, {151, 204, 105, 2732}, {152, 205, 140, 2732}, {153, 206, 105, 2732}, {154, 207, 42, 2732}, {155, 208, 7, 2732}, {157, 211, 1, 2732}, {158, 212, 7, 2732}, {159, 213, 21, 2732}, {160, 214, 35, 2732}, {161, 215, 35, 2732}, {162, 216, 21, 2732}, {163, 217, 7, 2732}, {164, 218, 1, 2732}, {122, 170, 1, 2733}, {124, 173, 7, 2733}, {125, 174, 7, 2733}, {127, 177, 21, 2733}, {128, 178, 42, 2733}, {129, 179, 21, 2733}, {131, 182, 35, 2733}, {132, 183, 105, 2733}, {133, 184, 105, 2733}, {134, 185, 35, 2733}, {136, 188, 35, 2733}, {137, 189, 140, 2733}, {138, 190, 210, 2733}, {139, 191, 140, 2733}, {140, 192, 35, 2733}, {142, 195, 21, 2733}, {143, 196, 105, 2733}, {144, 197, 210, 2733}, {145, 198, 210, 2733}, {146, 199, 105, 2733}, {147, 200, 21, 2733}, {149, 203, 7, 2733}, {150, 204, 42, 2733}, {151, 205, 105, 2733}, {152, 206, 140, 2733}, {153, 207, 105, 2733}, {154, 208, 42, 2733}, {155, 209, 7, 2733}, {157, 212, 1, 2733}, {158, 213, 7, 2733}, {159, 214, 21, 2733}, {160, 215, 35, 2733}, {161, 216, 35, 2733}, {162, 217, 21, 2733}, {163, 218, 7, 2733}, {164, 219, 1, 2733}, {120, 120, 1, 2734}, {121, 121, 8, 2734}, {122, 122, 8, 2734}, {123, 123, 28, 2734}, {124, 124, 56, 2734}, {125, 125, 28, 2734}, {126, 126, 56, 2734}, {127, 127, 168, 2734}, {128, 128, 168, 2734}, {129, 129, 56, 2734}, {130, 130, 70, 2734}, {131, 131, 280, 2734}, {132, 132, 420, 2734}, {133, 133, 280, 2734}, {134, 134, 70, 2734}, {135, 135, 56, 2734}, {136, 136, 280, 2734}, {137, 137, 560, 2734}, {138, 138, 560, 2734}, {139, 139, 280, 2734}, {140, 140, 56, 2734}, {141, 141, 28, 2734}, {142, 142, 168, 2734}, {143, 143, 420, 2734}, {144, 144, 560, 2734}, {145, 145, 420, 2734}, {146, 146, 168, 2734}, {147, 147, 28, 2734}, {148, 148, 8, 2734}, {149, 149, 56, 2734}, {150, 150, 168, 2734}, {151, 151, 280, 2734}, {152, 152, 280, 2734}, {153, 153, 168, 2734}, {154, 154, 56, 2734}, {155, 155, 8, 2734}, {156, 156, 1, 2734}, {157, 157, 8, 2734}, {158, 158, 28, 2734}, {159, 159, 56, 2734}, {160, 160, 70, 2734}, {161, 161, 56, 2734}, {162, 162, 28, 2734}, {163, 163, 8, 2734}, {164, 164, 1, 2734}, {120, 165, 1, 2735}, {121, 166, 8, 2735}, {122, 167, 8, 2735}, {123, 168, 28, 2735}, {124, 169, 56, 2735}, {125, 170, 28, 2735}, {126, 171, 56, 2735}, {127, 172, 168, 2735}, {128, 173, 168, 2735}, {129, 174, 56, 2735}, {130, 175, 70, 2735}, {131, 176, 280, 2735}, {132, 177, 420, 2735}, {133, 178, 280, 2735}, {134, 179, 70, 2735}, {135, 180, 56, 2735}, {136, 181, 280, 2735}, {137, 182, 560, 2735}, {138, 183, 560, 2735}, {139, 184, 280, 2735}, {140, 185, 56, 2735}, {141, 186, 28, 2735}, {142, 187, 168, 2735}, {143, 188, 420, 2735}, {144, 189, 560, 2735}, {145, 190, 420, 2735}, {146, 191, 168, 2735}, {147, 192, 28, 2735}, {148, 193, 8, 2735}, {149, 194, 56, 2735}, {150, 195, 168, 2735}, {151, 196, 280, 2735}, {152, 197, 280, 2735}, {153, 198, 168, 2735}, {154, 199, 56, 2735}, {155, 200, 8, 2735}, {156, 201, 1, 2735}, {157, 202, 8, 2735}, {158, 203, 28, 2735}, {159, 204, 56, 2735}, {160, 205, 70, 2735}, {161, 206, 56, 2735}, {162, 207, 28, 2735}, {163, 208, 8, 2735}, {164, 209, 1, 2735}, {120, 166, 1, 2736}, {121, 168, 8, 2736}, {122, 169, 8, 2736}, {123, 171, 28, 2736}, {124, 172, 56, 2736}, {125, 173, 28, 2736}, {126, 175, 56, 2736}, {127, 176, 168, 2736}, {128, 177, 168, 2736}, {129, 178, 56, 2736}, {130, 180, 70, 2736}, {131, 181, 280, 2736}, {132, 182, 420, 2736}, {133, 183, 280, 2736}, {134, 184, 70, 2736}, {135, 186, 56, 2736}, {136, 187, 280, 2736}, {137, 188, 560, 2736}, {138, 189, 560, 2736}, {139, 190, 280, 2736}, {140, 191, 56, 2736}, {141, 193, 28, 2736}, {142, 194, 168, 2736}, {143, 195, 420, 2736}, {144, 196, 560, 2736}, {145, 197, 420, 2736}, {146, 198, 168, 2736}, {147, 199, 28, 2736}, {148, 201, 8, 2736}, {149, 202, 56, 2736}, {150, 203, 168, 2736}, {151, 204, 280, 2736}, {152, 205, 280, 2736}, {153, 206, 168, 2736}, {154, 207, 56, 2736}, {155, 208, 8, 2736}, {156, 210, 1, 2736}, {157, 211, 8, 2736}, {158, 212, 28, 2736}, {159, 213, 56, 2736}, {160, 214, 70, 2736}, {161, 215, 56, 2736}, {162, 216, 28, 2736}, {163, 217, 8, 2736}, {164, 218, 1, 2736}, {120, 167, 1, 2737}, {121, 169, 8, 2737}, {122, 170, 8, 2737}, {123, 172, 28, 2737}, {124, 173, 56, 2737}, {125, 174, 28, 2737}, {126, 176, 56, 2737}, {127, 177, 168, 2737}, {128, 178, 168, 2737}, {129, 179, 56, 2737}, {130, 181, 70, 2737}, {131, 182, 280, 2737}, {132, 183, 420, 2737}, {133, 184, 280, 2737}, {134, 185, 70, 2737}, {135, 187, 56, 2737}, {136, 188, 280, 2737}, {137, 189, 560, 2737}, {138, 190, 560, 2737}, {139, 191, 280, 2737}, {140, 192, 56, 2737}, {141, 194, 28, 2737}, {142, 195, 168, 2737}, {143, 196, 420, 2737}, {144, 197, 560, 2737}, {145, 198, 420, 2737}, {146, 199, 168, 2737}, {147, 200, 28, 2737}, {148, 202, 8, 2737}, {149, 203, 56, 2737}, {150, 204, 168, 2737}, {151, 205, 280, 2737}, {152, 206, 280, 2737}, {153, 207, 168, 2737}, {154, 208, 56, 2737}, {155, 209, 8, 2737}, {156, 211, 1, 2737}, {157, 212, 8, 2737}, {158, 213, 28, 2737}, {159, 214, 56, 2737}, {160, 215, 70, 2737}, {161, 216, 56, 2737}, {162, 217, 28, 2737}, {163, 218, 8, 2737}, {164, 219, 1, 2737}, {120, 220, 1, 2738}, {121, 221, 8, 2738}, {122, 222, 8, 2738}, {123, 223, 28, 2738}, {124, 224, 56, 2738}, {125, 225, 28, 2738}, {126, 226, 56, 2738}, {127, 227, 168, 2738}, {128, 228, 168, 2738}, {129, 229, 56, 2738}, {130, 230, 70, 2738}, {131, 231, 280,

2738}, {132, 232, 420, 2738}, {133, 233, 280, 2738}, {134, 234, 70, 2738}, {135, 235, 56, 2738}, {136, 236, 280, 2738}, {137, 237, 560, 2738}, {138, 238, 560, 2738}, {139, 239, 280, 2738}, {140, 240, 56, 2738}, {141, 241, 28, 2738}, {142, 242, 168, 2738}, {143, 243, 420, 2738}, {144, 244, 560, 2738}, {145, 245, 420, 2738}, {146, 246, 168, 2738}, {147, 247, 28, 2738}, {148, 248, 8, 2738}, {149, 249, 56, 2738}, {150, 250, 168, 2738}, {151, 251, 280, 2738}, {152, 252, 280, 2738}, {153, 253, 168, 2738}, {154, 254, 56, 2738}, {155, 255, 8, 2738}, {156, 256, 1, 2738}, {157, 257, 8, 2738}, {158, 258, 28, 2738}, {159, 259, 56, 2738}, {160, 260, 70, 2738}, {161, 261, 56, 2738}, {162, 262, 28, 2738}, {163, 263, 8, 2738}, {164, 264, 1, 2738}, {120, 221, 1, 2739}, {121, 223, 8, 2739}, {122, 224, 8, 2739}, {123, 226, 28, 2739}, {124, 227, 56, 2739}, {125, 228, 28, 2739}, {126, 230, 56, 2739}, {127, 231, 168, 2739}, {128, 232, 168, 2739}, {129, 233, 56, 2739}, {130, 235, 70, 2739}, {131, 236, 280, 2739}, {132, 237, 420, 2739}, {133, 238, 280, 2739}, {134, 239, 70, 2739}, {135, 241, 56, 2739}, {136, 242, 280, 2739}, {137, 243, 560, 2739}, {138, 244, 560, 2739}, {139, 245, 280, 2739}, {140, 246, 56, 2739}, {141, 248, 28, 2739}, {142, 249, 168, 2739}, {143, 250, 420, 2739}, {144, 251, 560, 2739}, {145, 252, 420, 2739}, {146, 253, 168, 2739}, {147, 254, 28, 2739}, {148, 256, 8, 2739}, {149, 257, 56, 2739}, {150, 258, 168, 2739}, {151, 259, 280, 2739}, {152, 260, 280, 2739}, {153, 261, 168, 2739}, {154, 262, 56, 2739}, {155, 263, 8, 2739}, {156, 265, 1, 2739}, {157, 266, 8, 2739}, {158, 267, 28, 2739}, {159, 268, 56, 2739}, {160, 269, 70, 2739}, {161, 270, 56, 2739}, {162, 271, 28, 2739}, {163, 272, 8, 2739}, {164, 273, 1, 2739}, {120, 222, 1, 2740}, {121, 224, 8, 2740}, {122, 225, 8, 2740}, {123, 227, 28, 2740}, {124, 228, 56, 2740}, {125, 229, 28, 2740}, {126, 231, 56, 2740}, {127, 232, 168, 2740}, {128, 233, 168, 2740}, {129, 234, 56, 2740}, {130, 236, 70, 2740}, {131, 237, 280, 2740}, {132, 238, 420, 2740}, {133, 239, 280, 2740}, {134, 240, 70, 2740}, {135, 242, 56, 2740}, {136, 243, 280, 2740}, {137, 244, 560, 2740}, {138, 245, 560, 2740}, {139, 246, 280, 2740}, {140, 247, 56, 2740}, {141, 249, 28, 2740}, {142, 250, 168, 2740}, {143, 251, 420, 2740}, {144, 252, 560, 2740}, {145, 253, 420, 2740}, {146, 254, 168, 2740}, {147, 255, 28, 2740}, {148, 257, 8, 2740}, {149, 258, 56, 2740}, {150, 259, 168, 2740}, {151, 260, 280, 2740}, {152, 261, 280, 2740}, {153, 262, 168, 2740}, {154, 263, 56, 2740}, {155, 264, 8, 2740}, {156, 266, 1, 2740}, {157, 267, 8, 2740}, {158, 268, 28, 2740}, {159, 269, 56, 2740}, {160, 270, 70, 2740}, {161, 271, 56, 2740}, {162, 272, 28, 2740}, {163, 273, 8, 2740}, {164, 274, 1, 2740}, {120, 223, 1, 2741}, {121, 226, 8, 2741}, {122, 227, 8, 2741}, {123, 230, 28, 2741}, {124, 231, 56, 2741}, {125, 232, 28, 2741}, {126, 235, 56, 2741}, {127, 236, 168, 2741}, {128, 237, 168, 2741}, {129, 238, 56, 2741}, {130, 241, 70, 2741}, {131, 242, 280, 2741}, {132, 243, 420, 2741}, {133, 244, 280, 2741}, {134, 245, 70, 2741}, {135, 248, 56, 2741}, {136, 249, 280, 2741}, {137, 250, 560, 2741}, {138, 251, 560, 2741}, {139, 252, 280, 2741}, {140, 253, 56, 2741}, {141, 256, 28, 2741}, {142, 257, 168, 2741}, {143, 258, 420, 2741}, {144, 259, 560, 2741}, {145, 260, 420, 2741}, {146, 261, 168, 2741}, {147, 262, 28, 2741}, {148, 265, 8, 2741}, {149, 266, 56, 2741}, {150, 267, 168, 2741}, {151, 268, 280, 2741}, {152, 269, 280, 2741}, {153, 270, 168, 2741}, {154, 271, 56, 2741}, {155, 272, 8, 2741}, {156, 275, 1, 2741}, {157, 276, 8, 2741}, {158, 277, 28, 2741}, {159, 278, 56, 2741}, {160, 279, 70, 2741}, {161, 280, 56, 2741}, {162, 281, 28, 2741}, {163, 282, 8, 2741}, {164, 283, 1, 2741}, {120, 224, 1, 2742}, {121, 227, 8, 2742}, {122, 228, 8, 2742}, {123, 231, 28, 2742}, {124, 232, 56, 2742}, {125, 233, 28, 2742}, {126, 236, 56, 2742}, {127, 237, 168, 2742}, {128, 238, 168, 2742}, {129, 239, 56, 2742}, {130, 242, 70, 2742}, {131, 243, 280, 2742}, {132, 244, 420, 2742}, {133, 245, 280, 2742}, {134, 246, 70, 2742}, {135, 249, 56, 2742}, {136, 250, 280, 2742}, {137, 251, 560, 2742}, {138, 252, 560, 2742}, {139, 253, 280, 2742}, {140, 254, 56, 2742}, {141, 257, 28, 2742}, {142, 258, 168, 2742}, {143, 259, 420, 2742}, {144, 260, 560, 2742}, {145, 261, 420, 2742}, {146, 262, 168, 2742}, {147, 263, 28, 2742}, {148, 266, 8, 2742}, {149, 267, 56, 2742}, {150, 268, 168, 2742}, {151, 269, 280, 2742}, {152, 270, 280, 2742}, {153, 271, 168, 2742}, {154, 272, 56, 2742}, {155, 273, 8, 2742}, {156, 276, 1, 2742}, {157, 277, 8, 2742}, {158, 278, 28, 2742}, {159, 279, 56, 2742}, {160, 280, 70, 2742}, {161, 281, 56, 2742}, {162, 282, 28, 2742}, {163, 283, 8, 2742}, {164, 284, 1, 2742}, {120, 225, 1, 2743}, {121, 228, 8, 2743}, {122, 229, 8, 2743}, {123, 232, 28, 2743}, {124, 233, 56, 2743}, {125, 234, 28, 2743}, {126, 237, 56, 2743}, {127, 238, 168, 2743}, {128, 239, 168, 2743}, {129, 240, 56, 2743}, {130, 243, 70, 2743}, {131, 244, 280, 2743}, {132, 245, 420, 2743}, {133, 246, 280, 2743}, {134, 247, 70, 2743}, {135, 250, 56, 2743}, {136, 251, 280, 2743}, {137, 252, 560, 2743}, {138, 253, 560, 2743}, {139, 254, 280, 2743}, {140, 255, 56, 2743}, {141, 258, 28, 2743}, {142, 259, 168, 2743}, {143, 260, 420, 2743}, {144, 261, 560, 2743}, {145, 262, 420, 2743}, {146, 263, 168, 2743}, {147, 264, 28, 2743}, {148, 267, 8, 2743}, {149, 268, 56, 2743}, {150, 269, 168, 2743}, {151, 270, 280, 2743}, {152, 271, 280, 2743}, {153, 272, 168, 2743}, {154, 273, 56, 2743}, {155, 274, 8, 2743}, {156, 277, 1, 2743}, {157, 278, 8, 2743}, {158, 279, 28, 2743}, {159, 280, 56, 2743}, {160, 281, 70, 2743}, {161, 282, 56, 2743}, {162, 283, 28, 2743}, {163, 284, 8, 2743}, {164, 285, 1, 2743}, {165, 165, 1, 2744}, {166, 166, 8, 2744}, {167, 167, 8, 2744}, {168, 168, 28, 2744}, {169, 169, 56, 2744}, {170, 170, 28, 2744}, {171, 171, 56, 2744}, {172, 172, 168, 2744}, {173, 173, 168, 2744}, {174, 174, 56, 2744}, {175, 175, 70, 2744}, {176, 176, 280, 2744}, {177, 177, 420, 2744}, {178, 178, 280, 2744}, {179, 179, 70, 2744}, {180, 180, 56, 2744}, {181, 181, 280, 2744}, {182, 182, 560, 2744},

{183, 183, 560, 2744}, {184, 184, 280, 2744}, {185, 185, 56, 2744}, {186, 186, 28, 2744}, {187, 187, 168, 2744}, {188, 188, 420, 2744}, {189, 189, 560, 2744}, {190, 190, 420, 2744}, {191, 191, 168, 2744}, {192, 192, 28, 2744}, {193, 193, 8, 2744}, {194, 194, 56, 2744}, {195, 195, 168, 2744}, {196, 196, 280, 2744}, {197, 197, 280, 2744}, {198, 198, 168, 2744}, {199, 199, 56, 2744}, {200, 200, 8, 2744}, {201, 201, 1, 2744}, {202, 202, 8, 2744}, {203, 203, 28, 2744}, {204, 204, 56, 2744}, {205, 205, 70, 2744}, {206, 206, 56, 2744}, {207, 207, 28, 2744}, {208, 208, 8, 2744}, {209, 209, 1, 2744}, {165, 166, 1, 2745}, {166, 168, 8, 2745}, {167, 169, 8, 2745}, {168, 171, 28, 2745}, {169, 172, 56, 2745}, {170, 173, 28, 2745}, {171, 175, 56, 2745}, {172, 176, 168, 2745}, {173, 177, 168, 2745}, {174, 178, 56, 2745}, {175, 180, 70, 2745}, {176, 181, 280, 2745}, {177, 182, 420, 2745}, {178, 183, 280, 2745}, {179, 184, 70, 2745}, {180, 186, 56, 2745}, {181, 187, 280, 2745}, {182, 188, 560, 2745}, {183, 189, 560, 2745}, {184, 190, 280, 2745}, {185, 191, 56, 2745}, {186, 193, 28, 2745}, {187, 194, 168, 2745}, {188, 195, 420, 2745}, {189, 196, 560, 2745}, {190, 197, 420, 2745}, {191, 198, 168, 2745}, {192, 199, 28, 2745}, {193, 201, 8, 2745}, {194, 202, 56, 2745}, {195, 203, 168, 2745}, {196, 204, 280, 2745}, {197, 205, 280, 2745}, {198, 206, 168, 2745}, {199, 207, 56, 2745}, {200, 208, 8, 2745}, {201, 210, 1, 2745}, {202, 211, 8, 2745}, {203, 212, 28, 2745}, {204, 213, 56, 2745}, {205, 214, 70, 2745}, {206, 215, 56, 2745}, {207, 216, 28, 2745}, {208, 217, 8, 2745}, {209, 218, 1, 2745}, {165, 167, 1, 2746}, {166, 169, 8, 2746}, {167, 170, 8, 2746}, {168, 172, 28, 2746}, {169, 173, 56, 2746}, {170, 174, 28, 2746}, {171, 176, 56, 2746}, {172, 177, 168, 2746}, {173, 178, 168, 2746}, {174, 179, 56, 2746}, {175, 181, 70, 2746}, {176, 182, 280, 2746}, {177, 183, 420, 2746}, {178, 184, 280, 2746}, {179, 185, 70, 2746}, {180, 187, 56, 2746}, {181, 188, 280, 2746}, {182, 189, 560, 2746}, {183, 190, 560, 2746}, {184, 191, 280, 2746}, {185, 192, 56, 2746}, {186, 194, 28, 2746}, {187, 195, 168, 2746}, {188, 196, 420, 2746}, {189, 197, 560, 2746}, {190, 198, 420, 2746}, {191, 199, 168, 2746}, {192, 200, 28, 2746}, {193, 202, 8, 2746}, {194, 203, 56, 2746}, {195, 204, 168, 2746}, {196, 205, 280, 2746}, {197, 206, 280, 2746}, {198, 207, 168, 2746}, {199, 208, 56, 2746}, {200, 209, 8, 2746}, {201, 211, 1, 2746}, {202, 212, 8, 2746}, {203, 213, 28, 2746}, {204, 214, 56, 2746}, {205, 215, 70, 2746}, {206, 216, 56, 2746}, {207, 217, 28, 2746}, {208, 218, 8, 2746}, {209, 219, 1, 2746}, {166, 166, 1, 2747}, {168, 168, 8, 2747}, {169, 169, 8, 2747}, {171, 171, 28, 2747}, {172, 172, 56, 2747}, {173, 173, 28, 2747}, {175, 175, 56, 2747}, {176, 176, 168, 2747}, {177, 177, 168, 2747}, {178, 178, 56, 2747}, {180, 180, 70, 2747}, {181, 181, 280, 2747}, {182, 182, 420, 2747}, {183, 183, 280, 2747}, {184, 184, 70, 2747}, {186, 186, 56, 2747}, {187, 187, 280, 2747}, {188, 188, 560, 2747}, {189, 189, 560, 2747}, {190, 190, 280, 2747}, {191, 191, 56, 2747}, {193, 193, 28, 2747}, {194, 194, 168, 2747}, {195, 195, 420, 2747}, {196, 196, 560, 2747}, {197, 197, 420, 2747}, {198, 198, 168, 2747}, {199, 199, 28, 2747}, {201, 201, 8, 2747}, {202, 202, 56, 2747}, {203, 203, 168, 2747}, {204, 204, 280, 2747}, {205, 205, 280, 2747}, {206, 206, 168, 2747}, {207, 207, 56, 2747}, {208, 208, 8, 2747}, {210, 210, 1, 2747}, {211, 211, 8, 2747}, {212, 212, 28, 2747}, {213, 213, 56, 2747}, {214, 214, 70, 2747}, {215, 215, 56, 2747}, {216, 216, 28, 2747}, {217, 217, 8, 2747}, {218, 218, 1, 2747}, {166, 167, 1, 2748}, {168, 169, 8, 2748}, {169, 170, 8, 2748}, {171, 172, 28, 2748}, {172, 173, 56, 2748}, {173, 174, 28, 2748}, {175, 176, 56, 2748}, {176, 177, 168, 2748}, {177, 178, 168, 2748}, {178, 179, 56, 2748}, {180, 181, 70, 2748}, {181, 182, 280, 2748}, {182, 183, 420, 2748}, {183, 184, 280, 2748}, {184, 185, 70, 2748}, {186, 187, 56, 2748}, {187, 188, 280, 2748}, {188, 189, 560, 2748}, {189, 190, 560, 2748}, {190, 191, 280, 2748}, {191, 192, 56, 2748}, {193, 194, 28, 2748}, {194, 195, 168, 2748}, {195, 196, 420, 2748}, {196, 197, 560, 2748}, {197, 198, 420, 2748}, {198, 199, 168, 2748}, {199, 200, 28, 2748}, {201, 202, 8, 2748}, {202, 203, 56, 2748}, {203, 204, 168, 2748}, {204, 205, 280, 2748}, {205, 206, 280, 2748}, {206, 207, 168, 2748}, {207, 208, 56, 2748}, {208, 209, 8, 2748}, {210, 211, 1, 2748}, {211, 212, 8, 2748}, {212, 213, 28, 2748}, {213, 214, 56, 2748}, {214, 215, 70, 2748}, {215, 216, 56, 2748}, {216, 217, 28, 2748}, {217, 218, 8, 2748}, {218, 219, 1, 2748}, {167, 167, 1, 2749}, {169, 169, 8, 2749}, {170, 170, 8, 2749}, {172, 172, 28, 2749}, {173, 173, 56, 2749}, {174, 174, 28, 2749}, {176, 176, 56, 2749}, {177, 177, 168, 2749}, {178, 178, 168, 2749}, {179, 179, 56, 2749}, {181, 181, 70, 2749}, {182, 182, 280, 2749}, {183, 183, 420, 2749}, {184, 184, 280, 2749}, {185, 185, 70, 2749}, {187, 187, 56, 2749}, {188, 188, 280, 2749}, {189, 189, 560, 2749}, {190, 190, 560, 2749}, {191, 191, 280, 2749}, {192, 192, 56, 2749}, {194, 194, 28, 2749}, {195, 195, 168, 2749}, {196, 196, 420, 2749}, {197, 197, 560, 2749}, {198, 198, 420, 2749}, {199, 199, 168, 2749}, {200, 200, 28, 2749}, {202, 202, 8, 2749}, {203, 203, 56, 2749}, {204, 204, 168, 2749}, {205, 205, 280, 2749}, {206, 206, 280, 2749}, {207, 207, 168, 2749}, {208, 208, 56, 2749}, {209, 209, 8, 2749}, {211, 211, 1, 2749}, {212, 212, 8, 2749}, {213, 213, 28, 2749}, {214, 214, 56, 2749}, {215, 215, 70, 2749}, {216, 216, 56, 2749}, {217, 217, 28, 2749}, {218, 218, 8, 2749}, {219, 219, 1, 2749}, {165, 165, 1, 2750}, {166, 166, 9, 2750}, {167, 167, 9, 2750}, {168, 168, 36, 2750}, {169, 169, 72, 2750}, {170, 170, 36, 2750}, {171, 171, 84, 2750}, {172, 172, 252, 2750}, {173, 173, 252, 2750}, {174, 174, 84, 2750}, {175, 175, 126, 2750}, {176, 176, 504, 2750}, {177, 177, 756, 2750}, {178, 178, 504, 2750}, {179, 179, 126, 2750}, {180, 180, 126, 2750}, {181, 181, 630, 2750}, {182, 182, 1260, 2750}, {183, 183, 1260, 2750}, {184, 184, 630, 2750}, {185, 185, 126, 2750}, {186, 186, 84, 2750}, {187, 187, 504, 2750}, {188,

188, 1260, 2750}, {189, 189, 1680, 2750}, {190, 190, 1260, 2750}, {191, 191, 504, 2750}, {192, 192, 84, 2750}, {193, 193, 36, 2750}, {194, 194, 252, 2750}, {195, 195, 756, 2750}, {196, 196, 1260, 2750}, {197, 197, 1260, 2750}, {198, 198, 756, 2750}, {199, 199, 252, 2750}, {200, 200, 36, 2750}, {201, 201, 9, 2750}, {202, 202, 72, 2750}, {203, 203, 252, 2750}, {204, 204, 504, 2750}, {205, 205, 630, 2750}, {206, 206, 504, 2750}, {207, 207, 252, 2750}, {208, 208, 72, 2750}, {209, 209, 9, 2750}, {210, 210, 1, 2750}, {211, 211, 9, 2750}, {212, 212, 36, 2750}, {213, 213, 84, 2750}, {214, 214, 126, 2750}, {215, 215, 126, 2750}, {216, 216, 84, 2750}, {217, 217, 36, 2750}, {218, 218, 9, 2750}, {219, 219, 1, 2750}, {165, 220, 1, 2751}, {166, 221, 9, 2751}, {167, 222, 9, 2751}, {168, 223, 36, 2751}, {169, 224, 72, 2751}, {170, 225, 36, 2751}, {171, 226, 84, 2751}, {172, 227, 252, 2751}, {173, 228, 252, 2751}, {174, 229, 84, 2751}, {175, 230, 126, 2751}, {176, 231, 504, 2751}, {177, 232, 756, 2751}, {178, 233, 504, 2751}, {179, 234, 126, 2751}, {180, 235, 126, 2751}, {181, 236, 630, 2751}, {182, 237, 1260, 2751}, {183, 238, 1260, 2751}, {184, 239, 630, 2751}, {185, 240, 126, 2751}, {186, 241, 84, 2751}, {187, 242, 504, 2751}, {188, 243, 1260, 2751}, {189, 244, 1680, 2751}, {190, 245, 1260, 2751}, {191, 246, 504, 2751}, {192, 247, 84, 2751}, {193, 248, 36, 2751}, {194, 249, 252, 2751}, {195, 250, 756, 2751}, {196, 251, 1260, 2751}, {197, 252, 1260, 2751}, {198, 253, 756, 2751}, {199, 254, 252, 2751}, {200, 255, 36, 2751}, {201, 256, 9, 2751}, {202, 257, 72, 2751}, {203, 258, 252, 2751}, {204, 259, 504, 2751}, {205, 260, 630, 2751}, {206, 261, 504, 2751}, {207, 262, 252, 2751}, {208, 263, 72, 2751}, {209, 264, 9, 2751}, {210, 265, 1, 2751}, {211, 266, 9, 2751}, {212, 267, 36, 2751}, {213, 268, 84, 2751}, {214, 269, 126, 2751}, {215, 270, 126, 2751}, {216, 271, 84, 2751}, {217, 272, 36, 2751}, {218, 273, 9, 2751}, {219, 274, 1, 2751}, {165, 221, 1, 2752}, {166, 223, 9, 2752}, {167, 224, 9, 2752}, {168, 226, 36, 2752}, {169, 227, 72, 2752}, {170, 228, 36, 2752}, {171, 230, 84, 2752}, {172, 231, 252, 2752}, {173, 232, 252, 2752}, {174, 233, 84, 2752}, {175, 235, 126, 2752}, {176, 236, 504, 2752}, {177, 237, 756, 2752}, {178, 238, 504, 2752}, {179, 239, 126, 2752}, {180, 241, 126, 2752}, {181, 242, 630, 2752}, {182, 243, 1260, 2752}, {183, 244, 1260, 2752}, {184, 245, 630, 2752}, {185, 246, 126, 2752}, {186, 248, 84, 2752}, {187, 249, 504, 2752}, {188, 250, 1260, 2752}, {189, 251, 1680, 2752}, {190, 252, 1260, 2752}, {191, 253, 504, 2752}, {192, 254, 84, 2752}, {193, 256, 36, 2752}, {194, 257, 252, 2752}, {195, 258, 756, 2752}, {196, 259, 1260, 2752}, {197, 260, 1260, 2752}, {198, 261, 756, 2752}, {199, 262, 252, 2752}, {200, 263, 36, 2752}, {201, 265, 9, 2752}, {202, 266, 72, 2752}, {203, 267, 252, 2752}, {204, 268, 504, 2752}, {205, 269, 630, 2752}, {206, 270, 504, 2752}, {207, 271, 252, 2752}, {208, 272, 72, 2752}, {209, 273, 9, 2752}, {210, 275, 1, 2752}, {211, 276, 9, 2752}, {212, 277, 36, 2752}, {213, 278, 84, 2752}, {214, 279, 126, 2752}, {215, 280, 126, 2752}, {216, 281, 84, 2752}, {217, 282, 36, 2752}, {218, 283, 9, 2752}, {219, 284, 1, 2752}, {165, 222, 1, 2753}, {166, 224, 9, 2753}, {167, 225, 9, 2753}, {168, 227, 36, 2753}, {169, 228, 72, 2753}, {170, 229, 36, 2753}, {171, 231, 84, 2753}, {172, 232, 252, 2753}, {173, 233, 252, 2753}, {174, 234, 84, 2753}, {175, 236, 126, 2753}, {176, 237, 504, 2753}, {177, 238, 756, 2753}, {178, 239, 504, 2753}, {179, 240, 126, 2753}, {180, 242, 126, 2753}, {181, 243, 630, 2753}, {182, 244, 1260, 2753}, {183, 245, 1260, 2753}, {184, 246, 630, 2753}, {185, 247, 126, 2753}, {186, 249, 84, 2753}, {187, 250, 504, 2753}, {188, 251, 1260, 2753}, {189, 252, 1680, 2753}, {190, 253, 1260, 2753}, {191, 254, 504, 2753}, {192, 255, 84, 2753}, {193, 257, 36, 2753}, {194, 258, 252, 2753}, {195, 259, 756, 2753}, {196, 260, 1260, 2753}, {197, 261, 1260, 2753}, {198, 262, 756, 2753}, {199, 263, 252, 2753}, {200, 264, 36, 2753}, {201, 266, 9, 2753}, {202, 267, 72, 2753}, {203, 268, 252, 2753}, {204, 269, 504, 2753}, {205, 270, 630, 2753}, {206, 271, 504, 2753}, {207, 272, 252, 2753}, {208, 273, 72, 2753}, {209, 274, 9, 2753}, {210, 276, 1, 2753}, {211, 277, 9, 2753}, {212, 278, 36, 2753}, {213, 279, 84, 2753}, {214, 280, 126, 2753}, {215, 281, 126, 2753}, {216, 282, 84, 2753}, {217, 283, 36, 2753}, {218, 284, 9, 2753}, {219, 285, 1, 2753}, {220, 220, 1, 2754}, {221, 221, 10, 2754}, {222, 222, 10, 2754}, {223, 223, 45, 2754}, {224, 224, 90, 2754}, {225, 225, 45, 2754}, {226, 226, 120, 2754}, {227, 227, 360, 2754}, {228, 228, 360, 2754}, {229, 229, 120, 2754}, {230, 230, 210, 2754}, {231, 231, 840, 2754}, {232, 232, 1260, 2754}, {233, 233, 840, 2754}, {234, 234, 210, 2754}, {235, 235, 252, 2754}, {236, 236, 1260, 2754}, {237, 237, 2520, 2754}, {238, 238, 2520, 2754}, {239, 239, 1260, 2754}, {240, 240, 252, 2754}, {241, 241, 210, 2754}, {242, 242, 1260, 2754}, {243, 243, 3150, 2754}, {244, 244, 4200, 2754}, {245, 245, 3150, 2754}, {246, 246, 1260, 2754}, {247, 247, 210, 2754}, {248, 248, 120, 2754}, {249, 249, 840, 2754}, {250, 250, 2520, 2754}, {251, 251, 4200, 2754}, {252, 252, 4200, 2754}, {253, 253, 2520, 2754}, {254, 254, 840, 2754}, {255, 255, 120, 2754}, {256, 256, 45, 2754}, {257, 257, 360, 2754}, {258, 258, 1260, 2754}, {259, 259, 2520, 2754}, {260, 260, 3150, 2754}, {261, 261, 2520, 2754}, {262, 262, 1260, 2754}, {263, 263, 360, 2754}, {264, 264, 45, 2754}, {265, 265, 10, 2754}, {266, 266, 90, 2754}, {267, 267, 360, 2754}, {268, 268, 840, 2754}, {269, 269, 1260, 2754}, {270, 270, 1260, 2754}, {271, 271, 840, 2754}, {272, 272, 360, 2754}, {273, 273, 90, 2754}, {274, 274, 10, 2754}, {275, 275, 1, 2754}, {276, 276, 10, 2754}, {277, 277, 45, 2754}, {278, 278, 120, 2754}, {279, 279, 210, 2754}, {280, 280, 252, 2754}, {281, 281, 210, 2754}, {282, 282, 120, 2754}, {283, 283, 45, 2754}, {284, 284, 10, 2754}, {285, 285, 1, 2754}, {0, 286, 1, 2755}, {1, 287, 1, 2756}, {2, 288, 1, 2756}, {3, 289, 1, 2756}, {1, 290, 1, 2757}, {2, 291, 1, 2757}, {3, 292, 1, 2757}, {1, 291, 1, 2758}, {2, 293, 1,

2758}, {3, 294, 1, 2758}, {1, 292, 1, 2759}, {2, 294, 1, 2759}, {3, 295, 1, 2759}, {1, 296, 1, 2760}, {2, 297, 1, 2760}, {3, 298, 1, 2760}, {1, 297, 1, 2761}, {2, 299, 1, 2761}, {3, 300, 1, 2761}, {1, 298, 1, 2762}, {2, 300, 1, 2762}, {3, 301, 1, 2762}, {1, 299, 1, 2763}, {2, 302, 1, 2763}, {3, 303, 1, 2763}, {1, 300, 1, 2764}, {2, 303, 1, 2764}, {3, 304, 1, 2764}, {1, 301, 1, 2765}, {2, 304, 1, 2765}, {3, 305, 1, 2765}, {4, 287, 1, 2766}, {5, 288, 1, 2766}, {6, 289, 1, 2766}, {5, 287, 1, 2767}, {7, 288, 1, 2767}, {8, 289, 1, 2767}, {6, 287, 1, 2768}, {8, 288, 1, 2768}, {9, 289, 1, 2768}, {4, 290, 1, 2769}, {5, 291, 1, 2769}, {6, 292, 1, 2769}, {4, 291, 1, 2770}, {5, 293, 1, 2770}, {6, 294, 1, 2770}, {4, 292, 1, 2771}, {5, 294, 1, 2771}, {6, 295, 1, 2771}, {5, 290, 1, 2772}, {7, 291, 1, 2772}, {8, 292, 1, 2772}, {5, 291, 1, 2773}, {7, 293, 1, 2773}, {8, 294, 1, 2773}, {5, 292, 1, 2774}, {7, 294, 1, 2774}, {8, 295, 1, 2774}, {6, 290, 1, 2775}, {8, 291, 1, 2775}, {9, 292, 1, 2775}, {6, 291, 1, 2776}, {8, 293, 1, 2776}, {9, 294, 1, 2776}, {6, 292, 1, 2777}, {8, 294, 1, 2777}, {9, 295, 1, 2777}, {4, 296, 1, 2778}, {5, 297, 1, 2778}, {6, 298, 1, 2778}, {4, 297, 1, 2779}, {5, 299, 1, 2779}, {6, 300, 1, 2779}, {4, 298, 1, 2780}, {5, 300, 1, 2780}, {6, 301, 1, 2780}, {4, 299, 1, 2781}, {5, 302, 1, 2781}, {6, 303, 1, 2781}, {4, 300, 1, 2782}, {5, 303, 1, 2782}, {6, 304, 1, 2782}, {4, 301, 1, 2783}, {5, 304, 1, 2783}, {6, 305, 1, 2783}, {5, 296, 1, 2784}, {7, 297, 1, 2784}, {8, 298, 1, 2784}, {5, 297, 1, 2785}, {7, 299, 1, 2785}, {8, 300, 1, 2785}, {5, 298, 1, 2786}, {7, 300, 1, 2786}, {8, 301, 1, 2786}, {5, 299, 1, 2787}, {7, 302, 1, 2787}, {8, 303, 1, 2787}, {5, 300, 1, 2788}, {7, 303, 1, 2788}, {8, 304, 1, 2788}, {5, 301, 1, 2789}, {7, 304, 1, 2789}, {8, 305, 1, 2789}, {6, 296, 1, 2790}, {8, 297, 1, 2790}, {9, 298, 1, 2790}, {6, 297, 1, 2791}, {8, 299, 1, 2791}, {9, 300, 1, 2791}, {6, 298, 1, 2792}, {8, 300, 1, 2792}, {9, 301, 1, 2792}, {6, 299, 1, 2793}, {8, 302, 1, 2793}, {9, 303, 1, 2793}, {6, 300, 1, 2794}, {8, 303, 1, 2794}, {9, 304, 1, 2794}, {6, 301, 1, 2795}, {8, 304, 1, 2795}, {9, 305, 1, 2795}, {4, 306, 1, 2796}, {5, 307, 1, 2796}, {6, 308, 1, 2796}, {4, 307, 1, 2797}, {5, 309, 1, 2797}, {6, 310, 1, 2797}, {4, 308, 1, 2798}, {5, 310, 1, 2798}, {6, 311, 1, 2798}, {4, 309, 1, 2799}, {5, 312, 1, 2799}, {6, 313, 1, 2799}, {4, 310, 1, 2800}, {5, 313, 1, 2800}, {6, 314, 1, 2800}, {4, 311, 1, 2801}, {5, 314, 1, 2801}, {6, 315, 1, 2801}, {4, 312, 1, 2802}, {5, 316, 1, 2802}, {6, 317, 1, 2802}, {4, 313, 1, 2803}, {5, 317, 1, 2803}, {6, 318, 1, 2803}, {4, 314, 1, 2804}, {5, 318, 1, 2804}, {6, 319, 1, 2804}, {4, 315, 1, 2805}, {5, 319, 1, 2805}, {6, 320, 1, 2805}, {5, 306, 1, 2806}, {7, 307, 1, 2806}, {8, 308, 1, 2806}, {5, 307, 1, 2807}, {7, 309, 1, 2807}, {8, 310, 1, 2807}, {5, 308, 1, 2808}, {7, 310, 1, 2808}, {8, 311, 1, 2808}, {5, 309, 1, 2809}, {7, 312, 1, 2809}, {8, 313, 1, 2809}, {5, 310, 1, 2810}, {7, 313, 1, 2810}, {8, 314, 1, 2810}, {5, 311, 1, 2811}, {7, 314, 1, 2811}, {8, 315, 1, 2811}, {5, 312, 1, 2812}, {7, 316, 1, 2812}, {8, 317, 1, 2812}, {5, 313, 1, 2813}, {7, 317, 1, 2813}, {8, 318, 1, 2813}, {5, 314, 1, 2814}, {7, 318, 1, 2814}, {8, 319, 1, 2814}, {5, 315, 1, 2815}, {7, 319, 1, 2815}, {8, 320, 1, 2815}, {6, 306, 1, 2816}, {8, 307, 1, 2816}, {9, 308, 1, 2816}, {6, 307, 1, 2817}, {8, 309, 1, 2817}, {9, 310, 1, 2817}, {6, 308, 1, 2818}, {8, 310, 1, 2818}, {9, 311, 1, 2818}, {6, 309, 1, 2819}, {8, 312, 1, 2819}, {9, 313, 1, 2819}, {6, 310, 1, 2820}, {8, 313, 1, 2820}, {9, 314, 1, 2820}, {6, 311, 1, 2821}, {8, 314, 1, 2821}, {9, 315, 1, 2821}, {6, 312, 1, 2822}, {8, 316, 1, 2822}, {9, 317, 1, 2822}, {6, 313, 1, 2823}, {8, 317, 1, 2823}, {9, 318, 1, 2823}, {6, 314, 1, 2824}, {8, 318, 1, 2824}, {9, 319, 1, 2824}, {6, 315, 1, 2825}, {8, 319, 1, 2825}, {9, 320, 1, 2825}, {4, 290, 1, 2826}, {5, 291, 2, 2826}, {6, 292, 2, 2826}, {7, 293, 1, 2826}, {8, 294, 2, 2826}, {9, 295, 1, 2826}, {4, 296, 1, 2827}, {5, 297, 2, 2827}, {6, 298, 2, 2827}, {7, 299, 1, 2827}, {8, 300, 2, 2827}, {9, 301, 1, 2827}, {4, 297, 1, 2828}, {5, 299, 2, 2828}, {6, 300, 2, 2828}, {7, 302, 1, 2828}, {8, 303, 2, 2828}, {9, 304, 1, 2828}, {4, 298, 1, 2829}, {5, 300, 2, 2829}, {6, 301, 2, 2829}, {7, 303, 1, 2829}, {8, 304, 2, 2829}, {9, 305, 1, 2829}, {4, 306, 1, 2830}, {5, 307, 2, 2830}, {6, 308, 2, 2830}, {7, 309, 1, 2830}, {8, 310, 2, 2830}, {9, 311, 1, 2830}, {4, 307, 1, 2831}, {5, 309, 2, 2831}, {6, 310, 2, 2831}, {7, 312, 1, 2831}, {8, 313, 2, 2831}, {9, 314, 1, 2831}, {4, 308, 1, 2832}, {5, 310, 2, 2832}, {6, 311, 2, 2832}, {7, 313, 1, 2832}, {8, 314, 2, 2832}, {9, 315, 1, 2832}, {4, 309, 1, 2833}, {5, 312, 2, 2833}, {6, 313, 2, 2833}, {7, 316, 1, 2833}, {8, 317, 2, 2833}, {9, 318, 1, 2833}, {4, 310, 1, 2834}, {5, 313, 2, 2834}, {6, 314, 2, 2834}, {7, 317, 1, 2834}, {8, 318, 2, 2834}, {9, 319, 1, 2834}, {4, 311, 1, 2835}, {5, 314, 2, 2835}, {6, 315, 2, 2835}, {7, 318, 1, 2835}, {8, 319, 2, 2835}, {9, 320, 1, 2835}, {4, 321, 1, 2836}, {5, 322, 2, 2836}, {6, 323, 2, 2836}, {7, 324, 1, 2836}, {8, 325, 2, 2836}, {9, 326, 1, 2836}, {4, 322, 1, 2837}, {5, 324, 2, 2837}, {6, 325, 2, 2837}, {7, 327, 1, 2837}, {8, 328, 2, 2837}, {9, 329, 1, 2837}, {4, 323, 1, 2838}, {5, 325, 2, 2838}, {6, 326, 2, 2838}, {7, 328, 1, 2838}, {8, 329, 2, 2838}, {9, 330, 1, 2838}, {4, 324, 1, 2839}, {5, 327, 2, 2839}, {6, 328, 2, 2839}, {7, 331, 1, 2839}, {8, 332, 2, 2839}, {9, 333, 1, 2839}, {4, 325, 1, 2840}, {5, 328, 2, 2840}, {6, 329, 2, 2840}, {7, 332, 1, 2840}, {8, 333, 2, 2840}, {9, 334, 1, 2840}, {4, 326, 1, 2841}, {5, 329, 2, 2841}, {6, 330, 2, 2841}, {7, 333, 1, 2841}, {8, 334, 2, 2841}, {9, 335, 1, 2841}, {4, 327, 1, 2842}, {5, 331, 2, 2842}, {6, 332, 2, 2842}, {7, 336, 1, 2842}, {8, 337, 2, 2842}, {9, 338, 1, 2842}, {4, 328, 1, 2843}, {5, 332, 2, 2843}, {6, 333, 2, 2843}, {7, 337, 1, 2843}, {8, 338, 2, 2843}, {9, 339, 1, 2843}, {4, 329, 1, 2844}, {5, 333, 2, 2844}, {6, 334, 2, 2844}, {7, 338, 1, 2844}, {8, 339, 2, 2844}, {9, 340, 1, 2844}, {4, 330, 1, 2845}, {5, 334, 2, 2845}, {6, 335, 2, 2845}, {7, 339, 1, 2845}, {8, 340, 2, 2845}, {9, 341, 1,

2845}, {4, 342, 1, 2846}, {5, 343, 2, 2846}, {6, 344, 2, 2846}, {7, 345, 1, 2846}, {8, 346, 2, 2846}, {9, 347, 1, 2846}, {4, 343, 1, 2847}, {5, 345, 2, 2847}, {6, 346, 2, 2847}, {7, 348, 1, 2847}, {8, 349, 2, 2847}, {9, 350, 1, 2847}, {4, 344, 1, 2848}, {5, 346, 2, 2848}, {6, 347, 2, 2848}, {7, 349, 1, 2848}, {8, 350, 2, 2848}, {9, 351, 1, 2848}, {4, 345, 1, 2849}, {5, 348, 2, 2849}, {6, 349, 2, 2849}, {7, 352, 1, 2849}, {8, 353, 2, 2849}, {9, 354, 1, 2849}, {4, 346, 1, 2850}, {5, 349, 2, 2850}, {6, 350, 2, 2850}, {7, 353, 1, 2850}, {8, 354, 2, 2850}, {9, 355, 1, 2850}, {4, 347, 1, 2851}, {5, 350, 2, 2851}, {6, 351, 2, 2851}, {7, 354, 1, 2851}, {8, 355, 2, 2851}, {9, 356, 1, 2851}, {4, 348, 1, 2852}, {5, 352, 2, 2852}, {6, 353, 2, 2852}, {7, 357, 1, 2852}, {8, 358, 2, 2852}, {9, 359, 1, 2852}, {4, 349, 1, 2853}, {5, 353, 2, 2853}, {6, 354, 2, 2853}, {7, 358, 1, 2853}, {8, 359, 2, 2853}, {9, 360, 1, 2853}, {4, 350, 1, 2854}, {5, 354, 2, 2854}, {6, 355, 2, 2854}, {7, 359, 1, 2854}, {8, 360, 2, 2854}, {9, 361, 1, 2854}, {4, 351, 1, 2855}, {5, 355, 2, 2855}, {6, 356, 2, 2855}, {7, 360, 1, 2855}, {8, 361, 2, 2855}, {9, 362, 1, 2855}, {4, 352, 1, 2856}, {5, 357, 2, 2856}, {6, 358, 2, 2856}, {7, 363, 1, 2856}, {8, 364, 2, 2856}, {9, 365, 1, 2856}, {4, 353, 1, 2857}, {5, 358, 2, 2857}, {6, 359, 2, 2857}, {7, 364, 1, 2857}, {8, 365, 2, 2857}, {9, 366, 1, 2857}, {4, 354, 1, 2858}, {5, 359, 2, 2858}, {6, 360, 2, 2858}, {7, 365, 1, 2858}, {8, 366, 2, 2858}, {9, 367, 1, 2858}, {4, 355, 1, 2859}, {5, 360, 2, 2859}, {6, 361, 2, 2859}, {7, 366, 1, 2859}, {8, 367, 2, 2859}, {9, 368, 1, 2859}, {4, 356, 1, 2860}, {5, 361, 2, 2860}, {6, 362, 2, 2860}, {7, 367, 1, 2860}, {8, 368, 2, 2860}, {9, 369, 1, 2860}, {10, 287, 1, 2861}, {11, 288, 1, 2861}, {12, 289, 1, 2861}, {11, 287, 1, 2862}, {13, 288, 1, 2862}, {14, 289, 1, 2862}, {12, 287, 1, 2863}, {14, 288, 1, 2863}, {15, 289, 1, 2863}, {13, 287, 1, 2864}, {16, 288, 1, 2864}, {17, 289, 1, 2864}, {14, 287, 1, 2865}, {17, 288, 1, 2865}, {18, 289, 1, 2865}, {15, 287, 1, 2866}, {18, 288, 1, 2866}, {19, 289, 1, 2866}, {10, 290, 1, 2867}, {11, 291, 1, 2867}, {12, 292, 1, 2867}, {10, 291, 1, 2868}, {11, 293, 1, 2868}, {12, 294, 1, 2868}, {10, 292, 1, 2869}, {11, 294, 1, 2869}, {12, 295, 1, 2869}, {11, 290, 1, 2870}, {13, 291, 1, 2870}, {14, 292, 1, 2870}, {11, 291, 1, 2871}, {13, 293, 1, 2871}, {14, 294, 1, 2871}, {11, 292, 1, 2872}, {13, 294, 1, 2872}, {14, 295, 1, 2872}, {12, 290, 1, 2873}, {14, 291, 1, 2873}, {15, 292, 1, 2873}, {12, 291, 1, 2874}, {14, 293, 1, 2874}, {15, 294, 1, 2874}, {12, 292, 1, 2875}, {14, 294, 1, 2875}, {15, 295, 1, 2875}, {13, 290, 1, 2876}, {16, 291, 1, 2876}, {17, 292, 1, 2876}, {13, 291, 1, 2877}, {16, 293, 1, 2877}, {17, 294, 1, 2877}, {13, 292, 1, 2878}, {16, 294, 1, 2878}, {17, 295, 1, 2878}, {14, 290, 1, 2879}, {17, 291, 1, 2879}, {18, 292, 1, 2879}, {14, 291, 1, 2880}, {17, 293, 1, 2880}, {18, 294, 1, 2880}, {14, 292, 1, 2881}, {17, 294, 1, 2881}, {18, 295, 1, 2881}, {15, 290, 1, 2882}, {18, 291, 1, 2882}, {19, 292, 1, 2882}, {15, 291, 1, 2883}, {18, 293, 1, 2883}, {19, 294, 1, 2883}, {15, 292, 1, 2884}, {18, 294, 1, 2884}, {19, 295, 1, 2884}, {10, 296, 1, 2885}, {11, 297, 1, 2885}, {12, 298, 1, 2885}, {10, 297, 1, 2886}, {11, 299, 1, 2886}, {12, 300, 1, 2886}, {10, 298, 1, 2887}, {11, 300, 1, 2887}, {12, 301, 1, 2887}, {10, 299, 1, 2888}, {11, 302, 1, 2888}, {12, 303, 1, 2888}, {10, 300, 1, 2889}, {11, 303, 1, 2889}, {12, 304, 1, 2889}, {10, 301, 1, 2890}, {11, 304, 1, 2890}, {12, 305, 1, 2890}, {11, 296, 1, 2891}, {13, 297, 1, 2891}, {14, 298, 1, 2891}, {11, 297, 1, 2892}, {13, 299, 1, 2892}, {14, 300, 1, 2892}, {11, 298, 1, 2893}, {13, 300, 1, 2893}, {14, 301, 1, 2893}, {11, 299, 1, 2894}, {13, 302, 1, 2894}, {14, 303, 1, 2894}, {11, 300, 1, 2895}, {13, 303, 1, 2895}, {14, 304, 1, 2895}, {11, 301, 1, 2896}, {13, 304, 1, 2896}, {14, 305, 1, 2896}, {12, 296, 1, 2897}, {14, 297, 1, 2897}, {15, 298, 1, 2897}, {12, 297, 1, 2898}, {14, 299, 1, 2898}, {15, 300, 1, 2898}, {12, 298, 1, 2899}, {14, 300, 1, 2899}, {15, 301, 1, 2899}, {12, 299, 1, 2900}, {14, 302, 1, 2900}, {15, 303, 1, 2900}, {12, 300, 1, 2901}, {14, 303, 1, 2901}, {15, 304, 1, 2901}, {12, 301, 1, 2902}, {14, 304, 1, 2902}, {15, 305, 1, 2902}, {13, 296, 1, 2903}, {16, 297, 1, 2903}, {17, 298, 1, 2903}, {13, 297, 1, 2904}, {16, 299, 1, 2904}, {17, 300, 1, 2904}, {13, 298, 1, 2905}, {16, 300, 1, 2905}, {17, 301, 1, 2905}, {13, 299, 1, 2906}, {16, 302, 1, 2906}, {17, 303, 1, 2906}, {13, 300, 1, 2907}, {16, 303, 1, 2907}, {17, 304, 1, 2907}, {13, 301, 1, 2908}, {16, 304, 1, 2908}, {17, 305, 1, 2908}, {14, 296, 1, 2909}, {17, 297, 1, 2909}, {18, 298, 1, 2909}, {14, 297, 1, 2910}, {17, 299, 1, 2910}, {18, 300, 1, 2910}, {14, 298, 1, 2911}, {17, 300, 1, 2911}, {18, 301, 1, 2911}, {14, 299, 1, 2912}, {17, 302, 1, 2912}, {18, 303, 1, 2912}, {14, 300, 1, 2913}, {17, 303, 1, 2913}, {18, 304, 1, 2913}, {14, 301, 1, 2914}, {17, 304, 1, 2914}, {18, 305, 1, 2914}, {15, 296, 1, 2915}, {18, 297, 1, 2915}, {19, 298, 1, 2915}, {15, 297, 1, 2916}, {18, 299, 1, 2916}, {19, 300, 1, 2916}, {15, 298, 1, 2917}, {18, 300, 1, 2917}, {19, 301, 1, 2917}, {15, 299, 1, 2918}, {18, 302, 1, 2918}, {19, 303, 1, 2918}, {15, 300, 1, 2919}, {18, 303, 1, 2919}, {19, 304, 1, 2919}, {15, 301, 1, 2920}, {18, 304, 1, 2920}, {19, 305, 1, 2920}, {10, 290, 1, 2921}, {11, 291, 2, 2921}, {12, 292, 2, 2921}, {13, 293, 1, 2921}, {14, 294, 2, 2921}, {15, 295, 1, 2921}, {11, 290, 1, 2922}, {13, 291, 2, 2922}, {14, 292, 2, 2922}, {16, 293, 1, 2922}, {17, 294, 2, 2922}, {18, 295, 1, 2922}, {12, 290, 1, 2923}, {14, 291, 2, 2923}, {15, 292, 2, 2923}, {17, 293, 1, 2923}, {18, 294, 2, 2923}, {19, 295, 1, 2923}, {10, 296, 1, 2924}, {11, 297, 2, 2924}, {12, 298, 2, 2924}, {13, 299, 1, 2924}, {14, 300, 2, 2924}, {15, 301, 1, 2924}, {10, 297, 1, 2925}, {11, 299, 2, 2925}, {12, 300, 2, 2925}, {13, 302, 1, 2925}, {14, 303, 2, 2925}, {15, 304, 1, 2925}, {10, 298, 1, 2926}, {11, 300, 2, 2926}, {12, 301, 2, 2926}, {13, 303, 1, 2926}, {14, 304, 2, 2926}, {15, 305, 1, 2926}, {11, 296, 1, 2927}, {13, 297, 2, 2927}, {14, 298, 2, 2927}, {16, 299, 1, 2927}, {17, 300, 2, 2927}, {18, 301, 1, 2927}, {11, 297, 1, 2928},

{13, 299, 2, 2928}, {14, 300, 2, 2928}, {16, 302, 1, 2928}, {17, 303, 2, 2928}, {18, 304, 1, 2928}, {11, 298, 1, 2929}, {13, 300, 2, 2929}, {14, 301, 2, 2929}, {16, 303, 1, 2929}, {17, 304, 2, 2929}, {18, 305, 1, 2929}, {12, 296, 1, 2930}, {14, 297, 2, 2930}, {15, 298, 2, 2930}, {17, 299, 1, 2930}, {18, 300, 2, 2930}, {19, 301, 1, 2930}, {12, 297, 1, 2931}, {14, 299, 2, 2931}, {15, 300, 2, 2931}, {17, 302, 1, 2931}, {18, 303, 2, 2931}, {19, 304, 1, 2931}, {12, 298, 1, 2932}, {14, 300, 2, 2932}, {15, 301, 2, 2932}, {17, 303, 1, 2932}, {18, 304, 2, 2932}, {19, 305, 1, 2932}, {10, 306, 1, 2933}, {11, 307, 2, 2933}, {12, 308, 2, 2933}, {13, 309, 1, 2933}, {14, 310, 2, 2933}, {15, 311, 1, 2933}, {10, 307, 1, 2934}, {11, 309, 2, 2934}, {12, 310, 2, 2934}, {13, 312, 1, 2934}, {14, 313, 2, 2934}, {15, 314, 1, 2934}, {10, 308, 1, 2935}, {11, 310, 2, 2935}, {12, 311, 2, 2935}, {13, 313, 1, 2935}, {14, 314, 2, 2935}, {15, 315, 1, 2935}, {10, 309, 1, 2936}, {11, 312, 2, 2936}, {12, 313, 2, 2936}, {13, 316, 1, 2936}, {14, 317, 2, 2936}, {15, 318, 1, 2936}, {10, 310, 1, 2937}, {11, 313, 2, 2937}, {12, 314, 2, 2937}, {13, 317, 1, 2937}, {14, 318, 2, 2937}, {15, 319, 1, 2937}, {10, 311, 1, 2938}, {11, 314, 2, 2938}, {12, 315, 2, 2938}, {13, 318, 1, 2938}, {14, 319, 2, 2938}, {15, 320, 1, 2938}, {11, 306, 1, 2939}, {13, 307, 2, 2939}, {14, 308, 2, 2939}, {16, 309, 1, 2939}, {17, 310, 2, 2939}, {18, 311, 1, 2939}, {11, 307, 1, 2940}, {13, 309, 2, 2940}, {14, 310, 2, 2940}, {16, 312, 1, 2940}, {17, 313, 2, 2940}, {18, 314, 1, 2940}, {11, 308, 1, 2941}, {13, 310, 2, 2941}, {14, 311, 2, 2941}, {16, 313, 1, 2941}, {17, 314, 2, 2941}, {18, 315, 1, 2941}, {11, 309, 1, 2942}, {13, 312, 2, 2942}, {14, 313, 2, 2942}, {16, 316, 1, 2942}, {17, 317, 2, 2942}, {18, 318, 1, 2942}, {11, 310, 1, 2943}, {13, 313, 2, 2943}, {14, 314, 2, 2943}, {16, 317, 1, 2943}, {17, 318, 2, 2943}, {18, 319, 1, 2943}, {11, 311, 1, 2944}, {13, 314, 2, 2944}, {14, 315, 2, 2944}, {16, 318, 1, 2944}, {17, 319, 2, 2944}, {18, 320, 1, 2944}, {12, 306, 1, 2945}, {14, 307, 2, 2945}, {15, 308, 2, 2945}, {17, 309, 1, 2945}, {18, 310, 2, 2945}, {19, 311, 1, 2945}, {12, 307, 1, 2946}, {14, 309, 2, 2946}, {15, 310, 2, 2946}, {17, 312, 1, 2946}, {18, 313, 2, 2946}, {19, 314, 1, 2946}, {12, 308, 1, 2947}, {14, 310, 2, 2947}, {15, 311, 2, 2947}, {17, 313, 1, 2947}, {18, 314, 2, 2947}, {19, 315, 1, 2947}, {12, 309, 1, 2948}, {14, 312, 2, 2948}, {15, 313, 2, 2948}, {17, 316, 1, 2948}, {18, 317, 2, 2948}, {19, 318, 1, 2948}, {12, 310, 1, 2949}, {14, 313, 2, 2949}, {15, 314, 2, 2949}, {17, 317, 1, 2949}, {18, 318, 2, 2949}, {19, 319, 1, 2949}, {12, 311, 1, 2950}, {14, 314, 2, 2950}, {15, 315, 2, 2950}, {17, 318, 1, 2950}, {18, 319, 2, 2950}, {19, 320, 1, 2950}, {10, 321, 1, 2951}, {11, 322, 2, 2951}, {12, 323, 2, 2951}, {13, 324, 1, 2951}, {14, 325, 2, 2951}, {15, 326, 1, 2951}, {10, 322, 1, 2952}, {11, 324, 2, 2952}, {12, 325, 2, 2952}, {13, 327, 1, 2952}, {14, 328, 2, 2952}, {15, 329, 1, 2952}, {10, 323, 1, 2953}, {11, 325, 2, 2953}, {12, 326, 2, 2953}, {13, 328, 1, 2953}, {14, 329, 2, 2953}, {15, 330, 1, 2953}, {10, 324, 1, 2954}, {11, 327, 2, 2954}, {12, 328, 2, 2954}, {13, 331, 1, 2954}, {14, 332, 2, 2954}, {15, 333, 1, 2954}, {10, 325, 1, 2955}, {11, 328, 2, 2955}, {12, 329, 2, 2955}, {13, 332, 1, 2955}, {14, 333, 2, 2955}, {15, 334, 1, 2955}, {10, 326, 1, 2956}, {11, 329, 2, 2956}, {12, 330, 2, 2956}, {13, 333, 1, 2956}, {14, 334, 2, 2956}, {15, 335, 1, 2956}, {10, 327, 1, 2957}, {11, 331, 2, 2957}, {12, 332, 2, 2957}, {13, 336, 1, 2957}, {14, 337, 2, 2957}, {15, 338, 1, 2957}, {10, 328, 1, 2958}, {11, 332, 2, 2958}, {12, 333, 2, 2958}, {13, 337, 1, 2958}, {14, 338, 2, 2958}, {15, 339, 1, 2958}, {10, 329, 1, 2959}, {11, 333, 2, 2959}, {12, 334, 2, 2959}, {13, 338, 1, 2959}, {14, 339, 2, 2959}, {15, 340, 1, 2959}, {10, 330, 1, 2960}, {11, 334, 2, 2960}, {12, 335, 2, 2960}, {13, 339, 1, 2960}, {14, 340, 2, 2960}, {15, 341, 1, 2960}, {11, 321, 1, 2961}, {13, 322, 2, 2961}, {14, 323, 2, 2961}, {16, 324, 1, 2961}, {17, 325, 2, 2961}, {18, 326, 1, 2961}, {11, 322, 1, 2962}, {13, 324, 2, 2962}, {14, 325, 2, 2962}, {16, 327, 1, 2962}, {17, 328, 2, 2962}, {18, 329, 1, 2962}, {11, 323, 1, 2963}, {13, 325, 2, 2963}, {14, 326, 2, 2963}, {16, 328, 1, 2963}, {17, 329, 2, 2963}, {18, 330, 1, 2963}, {11, 324, 1, 2964}, {13, 327, 2, 2964}, {14, 328, 2, 2964}, {16, 331, 1, 2964}, {17, 332, 2, 2964}, {18, 333, 1, 2964}, {11, 325, 1, 2965}, {13, 328, 2, 2965}, {14, 329, 2, 2965}, {16, 332, 1, 2965}, {17, 333, 2, 2965}, {18, 334, 1, 2965}, {11, 326, 1, 2966}, {13, 329, 2, 2966}, {14, 330, 2, 2966}, {16, 333, 1, 2966}, {17, 334, 2, 2966}, {18, 335, 1, 2966}, {11, 327, 1, 2967}, {13, 331, 2, 2967}, {14, 332, 2, 2967}, {16, 336, 1, 2967}, {17, 337, 2, 2967}, {18, 338, 1, 2967}, {11, 328, 1, 2968}, {13, 332, 2, 2968}, {14, 333, 2, 2968}, {16, 337, 1, 2968}, {17, 338, 2, 2968}, {18, 339, 1, 2968}, {11, 329, 1, 2969}, {13, 333, 2, 2969}, {14, 334, 2, 2969}, {16, 338, 1, 2969}, {17, 339, 2, 2969}, {18, 340, 1, 2969}, {11, 330, 1, 2970}, {13, 334, 2, 2970}, {14, 335, 2, 2970}, {16, 339, 1, 2970}, {17, 340, 2, 2970}, {18, 341, 1, 2970}, {12, 321, 1, 2971}, {14, 322, 2, 2971}, {15, 323, 2, 2971}, {17, 324, 1, 2971}, {18, 325, 2, 2971}, {19, 326, 1, 2971}, {12, 322, 1, 2972}, {14, 324, 2, 2972}, {15, 325, 2, 2972}, {17, 327, 1, 2972}, {18, 328, 2, 2972}, {19, 329, 1, 2972}, {12, 323, 1, 2973}, {14, 325, 2, 2973}, {15, 326, 2, 2973}, {17, 328, 1, 2973}, {18, 329, 2, 2973}, {19, 330, 1, 2973}, {12, 324, 1, 2974}, {14, 327, 2, 2974}, {15, 328, 2, 2974}, {17, 331, 1, 2974}, {18, 332, 2, 2974}, {19, 333, 1, 2974}, {12, 325, 1, 2975}, {14, 328, 2, 2975}, {15, 329, 2, 2975}, {17, 332, 1, 2975}, {18, 333, 2, 2975}, {19, 334, 1, 2975}, {12, 326, 1, 2976}, {14, 329, 2, 2976}, {15, 330, 2, 2976}, {17, 333, 1, 2976}, {18, 334, 2, 2976}, {19, 335, 1, 2976}, {12, 327, 1, 2977}, {14, 331, 2, 2977}, {15, 332, 2, 2977}, {17, 336, 1, 2977}, {18, 337, 2, 2977}, {19, 338, 1, 2977}, {12, 328, 1, 2978}, {14, 332, 2, 2978}, {15, 333, 2, 2978}, {17, 337, 1, 2978}, {18, 338, 2, 2978}, {19, 339, 1, 2978}, {12, 329, 1, 2979}, {14, 333, 2, 2979}, {15, 334, 2, 2979}, {17, 338, 1, 2979}, {18, 339, 2, 2979}, {19, 340,

1, 2979}, {12, 330, 1, 2980}, {14, 334, 2, 2980}, {15, 335, 2, 2980}, {17, 339, 1, 2980},
{18, 340, 2, 2980}, {19, 341, 1, 2980}, {10, 342, 1, 2981}, {11, 343, 2, 2981}, {12, 344,
2, 2981}, {13, 345, 1, 2981}, {14, 346, 2, 2981}, {15, 347, 1, 2981}, {10, 343, 1, 2982},
{11, 345, 2, 2982}, {12, 346, 2, 2982}, {13, 348, 1, 2982}, {14, 349, 2, 2982}, {15, 350,
1, 2982}, {10, 344, 1, 2983}, {11, 346, 2, 2983}, {12, 347, 2, 2983}, {13, 349, 1, 2983},
{14, 350, 2, 2983}, {15, 351, 1, 2983}, {10, 345, 1, 2984}, {11, 348, 2, 2984}, {12, 349,
2, 2984}, {13, 352, 1, 2984}, {14, 353, 2, 2984}, {15, 354, 1, 2984}, {10, 346, 1, 2985},
{11, 349, 2, 2985}, {12, 350, 2, 2985}, {13, 353, 1, 2985}, {14, 354, 2, 2985}, {15, 355,
1, 2985}, {10, 347, 1, 2986}, {11, 350, 2, 2986}, {12, 351, 2, 2986}, {13, 354, 1, 2986},
{14, 355, 2, 2986}, {15, 356, 1, 2986}, {10, 348, 1, 2987}, {11, 352, 2, 2987}, {12, 353,
2, 2987}, {13, 357, 1, 2987}, {14, 358, 2, 2987}, {15, 359, 1, 2987}, {10, 349, 1, 2988},
{11, 353, 2, 2988}, {12, 354, 2, 2988}, {13, 358, 1, 2988}, {14, 359, 2, 2988}, {15, 360,
1, 2988}, {10, 350, 1, 2989}, {11, 354, 2, 2989}, {12, 355, 2, 2989}, {13, 359, 1, 2989},
{14, 360, 2, 2989}, {15, 361, 1, 2989}, {10, 351, 1, 2990}, {11, 355, 2, 2990}, {12, 356,
2, 2990}, {13, 360, 1, 2990}, {14, 361, 2, 2990}, {15, 362, 1, 2990}, {10, 352, 1, 2991},
{11, 357, 2, 2991}, {12, 358, 2, 2991}, {13, 363, 1, 2991}, {14, 364, 2, 2991}, {15, 365,
1, 2991}, {10, 353, 1, 2992}, {11, 358, 2, 2992}, {12, 359, 2, 2992}, {13, 364, 1, 2992},
{14, 365, 2, 2992}, {15, 366, 1, 2992}, {10, 354, 1, 2993}, {11, 359, 2, 2993}, {12, 360,
2, 2993}, {13, 365, 1, 2993}, {14, 366, 2, 2993}, {15, 367, 1, 2993}, {10, 355, 1, 2994},
{11, 360, 2, 2994}, {12, 361, 2, 2994}, {13, 366, 1, 2994}, {14, 367, 2, 2994}, {15, 368,
1, 2994}, {10, 356, 1, 2995}, {11, 361, 2, 2995}, {12, 362, 2, 2995}, {13, 367, 1, 2995},
{14, 368, 2, 2995}, {15, 369, 1, 2995}, {11, 342, 1, 2996}, {13, 343, 2, 2996}, {14, 344,
2, 2996}, {16, 345, 1, 2996}, {17, 346, 2, 2996}, {18, 347, 1, 2996}, {11, 343, 1, 2997},
{13, 345, 2, 2997}, {14, 346, 2, 2997}, {16, 348, 1, 2997}, {17, 349, 2, 2997}, {18, 350,
1, 2997}, {11, 344, 1, 2998}, {13, 346, 2, 2998}, {14, 347, 2, 2998}, {16, 349, 1, 2998},
{17, 350, 2, 2998}, {18, 351, 1, 2998}, {11, 345, 1, 2999}, {13, 348, 2, 2999}, {14, 349,
2, 2999}, {16, 352, 1, 2999}, {17, 353, 2, 2999}, {18, 354, 1, 2999}, {11, 346, 1, 3000},
{13, 349, 2, 3000}, {14, 350, 2, 3000}, {16, 353, 1, 3000}, {17, 354, 2, 3000}, {18, 355,
1, 3000}, {11, 347, 1, 3001}, {13, 350, 2, 3001}, {14, 351, 2, 3001}, {16, 354, 1, 3001},
{17, 355, 2, 3001}, {18, 356, 1, 3001}, {11, 348, 1, 3002}, {13, 352, 2, 3002}, {14, 353,
2, 3002}, {16, 357, 1, 3002}, {17, 358, 2, 3002}, {18, 359, 1, 3002}, {11, 349, 1, 3003},
{13, 353, 2, 3003}, {14, 354, 2, 3003}, {16, 358, 1, 3003}, {17, 359, 2, 3003}, {18, 360,
1, 3003}, {11, 350, 1, 3004}, {13, 354, 2, 3004}, {14, 355, 2, 3004}, {16, 359, 1, 3004},
{17, 360, 2, 3004}, {18, 361, 1, 3004}, {11, 351, 1, 3005}, {13, 355, 2, 3005}, {14, 356,
2, 3005}, {16, 360, 1, 3005}, {17, 361, 2, 3005}, {18, 362, 1, 3005}, {11, 352, 1, 3006},
{13, 357, 2, 3006}, {14, 358, 2, 3006}, {16, 363, 1, 3006}, {17, 364, 2, 3006}, {18, 365,
1, 3006}, {11, 353, 1, 3007}, {13, 358, 2, 3007}, {14, 359, 2, 3007}, {16, 364, 1, 3007},
{17, 365, 2, 3007}, {18, 366, 1, 3007}, {11, 354, 1, 3008}, {13, 359, 2, 3008}, {14, 360,
2, 3008}, {16, 365, 1, 3008}, {17, 366, 2, 3008}, {18, 367, 1, 3008}, {11, 355, 1, 3009},
{13, 360, 2, 3009}, {14, 361, 2, 3009}, {16, 366, 1, 3009}, {17, 367, 2, 3009}, {18, 368,
1, 3009}, {11, 356, 1, 3010}, {13, 361, 2, 3010}, {14, 362, 2, 3010}, {16, 367, 1, 3010},
{17, 368, 2, 3010}, {18, 369, 1, 3010}, {12, 342, 1, 3011}, {14, 343, 2, 3011}, {15, 344,
2, 3011}, {17, 345, 1, 3011}, {18, 346, 2, 3011}, {19, 347, 1, 3011}, {12, 343, 1, 3012},
{14, 345, 2, 3012}, {15, 346, 2, 3012}, {17, 348, 1, 3012}, {18, 349, 2, 3012}, {19, 350,
1, 3012}, {12, 344, 1, 3013}, {14, 346, 2, 3013}, {15, 347, 2, 3013}, {17, 349, 1, 3013},
{18, 350, 2, 3013}, {19, 351, 1, 3013}, {12, 345, 1, 3014}, {14, 348, 2, 3014}, {15, 349,
2, 3014}, {17, 352, 1, 3014}, {18, 353, 2, 3014}, {19, 354, 1, 3014}, {12, 346, 1, 3015},
{14, 349, 2, 3015}, {15, 350, 2, 3015}, {17, 353, 1, 3015}, {18, 354, 2, 3015}, {19, 355,
1, 3015}, {12, 347, 1, 3016}, {14, 350, 2, 3016}, {15, 351, 2, 3016}, {17, 354, 1, 3016},
{18, 355, 2, 3016}, {19, 356, 1, 3016}, {12, 348, 1, 3017}, {14, 352, 2, 3017}, {15, 353,
2, 3017}, {17, 357, 1, 3017}, {18, 358, 2, 3017}, {19, 359, 1, 3017}, {12, 349, 1, 3018},
{14, 353, 2, 3018}, {15, 354, 2, 3018}, {17, 358, 1, 3018}, {18, 359, 2, 3018}, {19, 360,
1, 3018}, {12, 350, 1, 3019}, {14, 354, 2, 3019}, {15, 355, 2, 3019}, {17, 359, 1, 3019},
{18, 360, 2, 3019}, {19, 361, 1, 3019}, {12, 351, 1, 3020}, {14, 355, 2, 3020}, {15, 356,
2, 3020}, {17, 360, 1, 3020}, {18, 361, 2, 3020}, {19, 362, 1, 3020}, {12, 352, 1, 3021},
{14, 357, 2, 3021}, {15, 358, 2, 3021}, {17, 363, 1, 3021}, {18, 364, 2, 3021}, {19, 365,
1, 3021}, {12, 353, 1, 3022}, {14, 358, 2, 3022}, {15, 359, 2, 3022}, {17, 364, 1, 3022},
{18, 365, 2, 3022}, {19, 366, 1, 3022}, {12, 354, 1, 3023}, {14, 359, 2, 3023}, {15, 360,
2, 3023}, {17, 365, 1, 3023}, {18, 366, 2, 3023}, {19, 367, 1, 3023}, {12, 355, 1, 3024},
{14, 360, 2, 3024}, {15, 361, 2, 3024}, {17, 366, 1, 3024}, {18, 367, 2, 3024}, {19, 368,
1, 3024}, {12, 356, 1, 3025}, {14, 361, 2, 3025}, {15, 362, 2, 3025}, {17, 367, 1, 3025},
{18, 368, 2, 3025}, {19, 369, 1, 3025}, {10, 296, 1, 3026}, {11, 297, 3, 3026}, {12, 298,
3, 3026}, {13, 299, 3, 3026}, {14, 300, 6, 3026}, {15, 301, 3, 3026}, {16, 302, 1, 3026},
{17, 303, 3, 3026}, {18, 304, 3, 3026}, {19, 305, 1, 3026}, {10, 306, 1, 3027}, {11, 307,
3, 3027}, {12, 308, 3, 3027}, {13, 309, 3, 3027}, {14, 310, 6, 3027}, {15, 311, 3, 3027},
{16, 312, 1, 3027}, {17, 313, 3, 3027}, {18, 314, 3, 3027}, {19, 315, 1, 3027}, {10, 307,
1, 3028}, {11, 309, 3, 3028}, {12, 310, 3, 3028}, {13, 312, 3, 3028}, {14, 313, 6, 3028},
{15, 314, 3, 3028}, {16, 316, 1, 3028}, {17, 317, 3, 3028}, {18, 318, 3, 3028}, {19, 319,
1, 3028}, {10, 308, 1, 3029}, {11, 310, 3, 3029}, {12, 311, 3, 3029}, {13, 313, 3, 3029},

{14, 314, 6, 3029}, {15, 315, 3, 3029}, {16, 317, 1, 3029}, {17, 318, 3, 3029}, {18, 319, 3, 3029}, {19, 320, 1, 3029}, {10, 321, 1, 3030}, {11, 322, 3, 3030}, {12, 323, 3, 3030}, {13, 324, 3, 3030}, {14, 325, 6, 3030}, {15, 326, 3, 3030}, {16, 327, 1, 3030}, {17, 328, 3, 3030}, {18, 329, 3, 3030}, {19, 330, 1, 3030}, {10, 322, 1, 3031}, {11, 324, 3, 3031}, {12, 325, 3, 3031}, {13, 327, 3, 3031}, {14, 328, 6, 3031}, {15, 329, 3, 3031}, {16, 331, 1, 3031}, {17, 332, 3, 3031}, {18, 333, 3, 3031}, {19, 334, 1, 3031}, {10, 323, 1, 3032}, {11, 325, 3, 3032}, {12, 326, 3, 3032}, {13, 328, 3, 3032}, {14, 329, 6, 3032}, {15, 330, 3, 3032}, {16, 332, 1, 3032}, {17, 333, 3, 3032}, {18, 334, 3, 3032}, {19, 335, 1, 3032}, {10, 324, 1, 3033}, {11, 327, 3, 3033}, {12, 328, 3, 3033}, {13, 331, 3, 3033}, {14, 332, 6, 3033}, {15, 333, 3, 3033}, {16, 336, 1, 3033}, {17, 337, 3, 3033}, {18, 338, 3, 3033}, {19, 339, 1, 3033}, {10, 325, 1, 3034}, {11, 328, 3, 3034}, {12, 329, 3, 3034}, {13, 332, 3, 3034}, {14, 333, 6, 3034}, {15, 334, 3, 3034}, {16, 337, 1, 3034}, {17, 338, 3, 3034}, {18, 339, 3, 3034}, {19, 340, 1, 3034}, {10, 326, 1, 3035}, {11, 329, 3, 3035}, {12, 330, 3, 3035}, {13, 333, 3, 3035}, {14, 334, 6, 3035}, {15, 335, 3, 3035}, {16, 338, 1, 3035}, {17, 339, 3, 3035}, {18, 340, 3, 3035}, {19, 341, 1, 3035}, {10, 342, 1, 3036}, {11, 343, 3, 3036}, {12, 344, 3, 3036}, {13, 345, 3, 3036}, {14, 346, 6, 3036}, {15, 347, 3, 3036}, {16, 348, 1, 3036}, {17, 349, 3, 3036}, {18, 350, 3, 3036}, {19, 351, 1, 3036}, {10, 343, 1, 3037}, {11, 345, 3, 3037}, {12, 346, 3, 3037}, {13, 348, 3, 3037}, {14, 349, 6, 3037}, {15, 350, 3, 3037}, {16, 352, 1, 3037}, {17, 353, 3, 3037}, {18, 354, 3, 3037}, {19, 355, 1, 3037}, {10, 344, 1, 3038}, {11, 346, 3, 3038}, {12, 347, 3, 3038}, {13, 349, 3, 3038}, {14, 350, 6, 3038}, {15, 351, 3, 3038}, {16, 353, 1, 3038}, {17, 354, 3, 3038}, {18, 355, 3, 3038}, {19, 356, 1, 3038}, {10, 345, 1, 3039}, {11, 348, 3, 3039}, {12, 349, 3, 3039}, {13, 352, 3, 3039}, {14, 353, 6, 3039}, {15, 354, 3, 3039}, {16, 357, 1, 3039}, {17, 358, 3, 3039}, {18, 359, 3, 3039}, {19, 360, 1, 3039}, {10, 346, 1, 3040}, {11, 349, 3, 3040}, {12, 350, 3, 3040}, {13, 353, 3, 3040}, {14, 354, 6, 3040}, {15, 355, 3, 3040}, {16, 358, 1, 3040}, {17, 359, 3, 3040}, {18, 360, 3, 3040}, {19, 361, 1, 3040}, {10, 347, 1, 3041}, {11, 350, 3, 3041}, {12, 351, 3, 3041}, {13, 354, 3, 3041}, {14, 355, 6, 3041}, {15, 356, 3, 3041}, {16, 359, 1, 3041}, {17, 360, 3, 3041}, {18, 361, 3, 3041}, {19, 362, 1, 3041}, {10, 348, 1, 3042}, {11, 352, 3, 3042}, {12, 353, 3, 3042}, {13, 357, 3, 3042}, {14, 358, 6, 3042}, {15, 359, 3, 3042}, {16, 363, 1, 3042}, {17, 364, 3, 3042}, {18, 365, 3, 3042}, {19, 366, 1, 3042}, {10, 349, 1, 3043}, {11, 353, 3, 3043}, {12, 354, 3, 3043}, {13, 358, 3, 3043}, {14, 359, 6, 3043}, {15, 360, 3, 3043}, {16, 364, 1, 3043}, {17, 365, 3, 3043}, {18, 366, 3, 3043}, {19, 367, 1, 3043}, {10, 350, 1, 3044}, {11, 354, 3, 3044}, {12, 355, 3, 3044}, {13, 359, 3, 3044}, {14, 360, 6, 3044}, {15, 361, 3, 3044}, {16, 365, 1, 3044}, {17, 366, 3, 3044}, {18, 367, 3, 3044}, {19, 368, 1, 3044}, {10, 351, 1, 3045}, {11, 355, 3, 3045}, {12, 356, 3, 3045}, {13, 360, 3, 3045}, {14, 361, 6, 3045}, {15, 362, 3, 3045}, {16, 366, 1, 3045}, {17, 367, 3, 3045}, {18, 368, 3, 3045}, {19, 369, 1, 3045}, {10, 370, 1, 3046}, {11, 371, 3, 3046}, {12, 372, 3, 3046}, {13, 373, 3, 3046}, {14, 374, 6, 3046}, {15, 375, 3, 3046}, {16, 376, 1, 3046}, {17, 377, 3, 3046}, {18, 378, 3, 3046}, {19, 379, 1, 3046}, {10, 371, 1, 3047}, {11, 373, 3, 3047}, {12, 374, 3, 3047}, {13, 376, 3, 3047}, {14, 377, 6, 3047}, {15, 378, 3, 3047}, {16, 380, 1, 3047}, {17, 381, 3, 3047}, {18, 382, 3, 3047}, {19, 383, 1, 3047}, {10, 372, 1, 3048}, {11, 374, 3, 3048}, {12, 375, 3, 3048}, {13, 377, 3, 3048}, {14, 378, 6, 3048}, {15, 379, 3, 3048}, {16, 381, 1, 3048}, {17, 382, 3, 3048}, {18, 383, 3, 3048}, {19, 384, 1, 3048}, {10, 373, 1, 3049}, {11, 376, 3, 3049}, {12, 377, 3, 3049}, {13, 380, 3, 3049}, {14, 381, 6, 3049}, {15, 382, 3, 3049}, {16, 385, 1, 3049}, {17, 386, 3, 3049}, {18, 387, 3, 3049}, {19, 388, 1, 3049}, {10, 374, 1, 3050}, {11, 377, 3, 3050}, {12, 378, 3, 3050}, {13, 381, 3, 3050}, {14, 382, 6, 3050}, {15, 383, 3, 3050}, {16, 386, 1, 3050}, {17, 387, 3, 3050}, {18, 388, 3, 3050}, {19, 389, 1, 3050}, {10, 375, 1, 3051}, {11, 378, 3, 3051}, {12, 379, 3, 3051}, {13, 382, 3, 3051}, {14, 383, 6, 3051}, {15, 384, 3, 3051}, {16, 387, 1, 3051}, {17, 388, 3, 3051}, {18, 389, 3, 3051}, {19, 390, 1, 3051}, {10, 376, 1, 3052}, {11, 380, 3, 3052}, {12, 381, 3, 3052}, {13, 385, 3, 3052}, {14, 386, 6, 3052}, {15, 387, 3, 3052}, {16, 391, 1, 3052}, {17, 392, 3, 3052}, {18, 393, 3, 3052}, {19, 394, 1, 3052}, {10, 377, 1, 3053}, {11, 381, 3, 3053}, {12, 382, 3, 3053}, {13, 386, 3, 3053}, {14, 387, 6, 3053}, {15, 388, 3, 3053}, {16, 392, 1, 3053}, {17, 393, 3, 3053}, {18, 394, 3, 3053}, {19, 395, 1, 3053}, {10, 378, 1, 3054}, {11, 382, 3, 3054}, {12, 383, 3, 3054}, {13, 387, 3, 3054}, {14, 388, 6, 3054}, {15, 389, 3, 3054}, {16, 393, 1, 3054}, {17, 394, 3, 3054}, {18, 395, 3, 3054}, {19, 396, 1, 3054}, {10, 379, 1, 3055}, {11, 383, 3, 3055}, {12, 384, 3, 3055}, {13, 388, 3, 3055}, {14, 389, 6, 3055}, {15, 390, 3, 3055}, {16, 394, 1, 3055}, {17, 395, 3, 3055}, {18, 396, 3, 3055}, {19, 397, 1, 3055}, {10, 380, 1, 3056}, {11, 385, 3, 3056}, {12, 386, 3, 3056}, {13, 391, 3, 3056}, {14, 392, 6, 3056}, {15, 393, 3, 3056}, {16, 398, 1, 3056}, {17, 399, 3, 3056}, {18, 400, 3, 3056}, {19, 401, 1, 3056}, {10, 381, 1, 3057}, {11, 386, 3, 3057}, {12, 387, 3, 3057}, {13, 392, 3, 3057}, {14, 393, 6, 3057}, {15, 394, 3, 3057}, {16, 399, 1, 3057}, {17, 400, 3, 3057}, {18, 401, 3, 3057}, {19, 402, 1, 3057}, {10, 382, 1, 3058}, {11, 387, 3, 3058}, {12, 388, 3, 3058}, {13, 393, 3, 3058}, {14, 394, 6, 3058}, {15, 395, 3, 3058}, {16, 400, 1, 3058}, {17, 401, 3, 3058}, {18, 402, 3, 3058}, {19, 403, 1, 3058}, {10, 383, 1, 3059}, {11, 388, 3, 3059}, {12, 389, 3, 3059}, {13, 394, 3, 3059}, {14, 395, 6, 3059}, {15, 396, 3, 3059}, {16, 401, 1, 3059}, {17, 402, 3, 3059}, {18, 403, 3, 3059}, {19, 404, 1, 3059}, {10, 384, 1, 3060}, {11, 389, 3, 3060}, {12, 390, 3, 3060}, {13, 395, 3, 3060}, {14, 396,

6, 3060}, {15, 397, 3, 3060}, {16, 402, 1, 3060}, {17, 403, 3, 3060}, {18, 404, 3, 3060},
{19, 405, 1, 3060}, {20, 290, 1, 3061}, {21, 291, 2, 3061}, {22, 292, 2, 3061}, {23, 293,
1, 3061}, {24, 294, 2, 3061}, {25, 295, 1, 3061}, {21, 290, 1, 3062}, {23, 291, 2, 3062},
{24, 292, 2, 3062}, {26, 293, 1, 3062}, {27, 294, 2, 3062}, {28, 295, 1, 3062}, {22, 290,
1, 3063}, {24, 291, 2, 3063}, {25, 292, 2, 3063}, {27, 293, 1, 3063}, {28, 294, 2, 3063},
{29, 295, 1, 3063}, {23, 290, 1, 3064}, {26, 291, 2, 3064}, {27, 292, 2, 3064}, {30, 293,
1, 3064}, {31, 294, 2, 3064}, {32, 295, 1, 3064}, {24, 290, 1, 3065}, {27, 291, 2, 3065},
{28, 292, 2, 3065}, {31, 293, 1, 3065}, {32, 294, 2, 3065}, {33, 295, 1, 3065}, {25, 290,
1, 3066}, {28, 291, 2, 3066}, {29, 292, 2, 3066}, {32, 293, 1, 3066}, {33, 294, 2, 3066},
{34, 295, 1, 3066}, {20, 296, 1, 3067}, {21, 297, 2, 3067}, {22, 298, 2, 3067}, {23, 299,
1, 3067}, {24, 300, 2, 3067}, {25, 301, 1, 3067}, {20, 297, 1, 3068}, {21, 299, 2, 3068},
{22, 300, 2, 3068}, {23, 302, 1, 3068}, {24, 303, 2, 3068}, {25, 304, 1, 3068}, {20, 298,
1, 3069}, {21, 300, 2, 3069}, {22, 301, 2, 3069}, {23, 303, 1, 3069}, {24, 304, 2, 3069},
{25, 305, 1, 3069}, {21, 296, 1, 3070}, {23, 297, 2, 3070}, {24, 298, 2, 3070}, {26, 299,
1, 3070}, {27, 300, 2, 3070}, {28, 301, 1, 3070}, {21, 297, 1, 3071}, {23, 299, 2, 3071},
{24, 300, 2, 3071}, {26, 302, 1, 3071}, {27, 303, 2, 3071}, {28, 304, 1, 3071}, {21, 298,
1, 3072}, {23, 300, 2, 3072}, {24, 301, 2, 3072}, {26, 303, 1, 3072}, {27, 304, 2, 3072},
{28, 305, 1, 3072}, {22, 296, 1, 3073}, {24, 297, 2, 3073}, {25, 298, 2, 3073}, {27, 299,
1, 3073}, {28, 300, 2, 3073}, {29, 301, 1, 3073}, {22, 297, 1, 3074}, {24, 299, 2, 3074},
{25, 300, 2, 3074}, {27, 302, 1, 3074}, {28, 303, 2, 3074}, {29, 304, 1, 3074}, {22, 298,
1, 3075}, {24, 300, 2, 3075}, {25, 301, 2, 3075}, {27, 303, 1, 3075}, {28, 304, 2, 3075},
{29, 305, 1, 3075}, {23, 296, 1, 3076}, {26, 297, 2, 3076}, {27, 298, 2, 3076}, {30, 299,
1, 3076}, {31, 300, 2, 3076}, {32, 301, 1, 3076}, {23, 297, 1, 3077}, {26, 299, 2, 3077},
{27, 300, 2, 3077}, {30, 302, 1, 3077}, {31, 303, 2, 3077}, {32, 304, 1, 3077}, {23, 298,
1, 3078}, {26, 300, 2, 3078}, {27, 301, 2, 3078}, {30, 303, 1, 3078}, {31, 304, 2, 3078},
{32, 305, 1, 3078}, {24, 296, 1, 3079}, {27, 297, 2, 3079}, {28, 298, 2, 3079}, {31, 299,
1, 3079}, {32, 300, 2, 3079}, {33, 301, 1, 3079}, {24, 297, 1, 3080}, {27, 299, 2, 3080},
{28, 300, 2, 3080}, {31, 302, 1, 3080}, {32, 303, 2, 3080}, {33, 304, 1, 3080}, {24, 298,
1, 3081}, {27, 300, 2, 3081}, {28, 301, 2, 3081}, {31, 303, 1, 3081}, {32, 304, 2, 3081},
{33, 305, 1, 3081}, {25, 296, 1, 3082}, {28, 297, 2, 3082}, {29, 298, 2, 3082}, {32, 299,
1, 3082}, {33, 300, 2, 3082}, {34, 301, 1, 3082}, {25, 297, 1, 3083}, {28, 299, 2, 3083},
{29, 300, 2, 3083}, {32, 302, 1, 3083}, {33, 303, 2, 3083}, {34, 304, 1, 3083}, {25, 298,
1, 3084}, {28, 300, 2, 3084}, {29, 301, 2, 3084}, {32, 303, 1, 3084}, {33, 304, 2, 3084},
{34, 305, 1, 3084}, {20, 306, 1, 3085}, {21, 307, 2, 3085}, {22, 308, 2, 3085}, {23, 309,
1, 3085}, {24, 310, 2, 3085}, {25, 311, 1, 3085}, {20, 307, 1, 3086}, {21, 309, 2, 3086},
{22, 310, 2, 3086}, {23, 312, 1, 3086}, {24, 313, 2, 3086}, {25, 314, 1, 3086}, {20, 308,
1, 3087}, {21, 310, 2, 3087}, {22, 311, 2, 3087}, {23, 313, 1, 3087}, {24, 314, 2, 3087},
{25, 315, 1, 3087}, {20, 309, 1, 3088}, {21, 312, 2, 3088}, {22, 313, 2, 3088}, {23, 316,
1, 3088}, {24, 317, 2, 3088}, {25, 318, 1, 3088}, {20, 310, 1, 3089}, {21, 313, 2, 3089},
{22, 314, 2, 3089}, {23, 317, 1, 3089}, {24, 318, 2, 3089}, {25, 319, 1, 3089}, {20, 311,
1, 3090}, {21, 314, 2, 3090}, {22, 315, 2, 3090}, {23, 318, 1, 3090}, {24, 319, 2, 3090},
{25, 320, 1, 3090}, {21, 306, 1, 3091}, {23, 307, 2, 3091}, {24, 308, 2, 3091}, {26, 309,
1, 3091}, {27, 310, 2, 3091}, {28, 311, 1, 3091}, {21, 307, 1, 3092}, {23, 309, 2, 3092},
{24, 310, 2, 3092}, {26, 312, 1, 3092}, {27, 313, 2, 3092}, {28, 314, 1, 3092}, {21, 308,
1, 3093}, {23, 310, 2, 3093}, {24, 311, 2, 3093}, {26, 313, 1, 3093}, {27, 314, 2, 3093},
{28, 315, 1, 3093}, {21, 309, 1, 3094}, {23, 312, 2, 3094}, {24, 313, 2, 3094}, {26, 316,
1, 3094}, {27, 317, 2, 3094}, {28, 318, 1, 3094}, {21, 310, 1, 3095}, {23, 313, 2, 3095},
{24, 314, 2, 3095}, {26, 317, 1, 3095}, {27, 318, 2, 3095}, {28, 319, 1, 3095}, {21, 311,
1, 3096}, {23, 314, 2, 3096}, {24, 315, 2, 3096}, {26, 318, 1, 3096}, {27, 319, 2, 3096},
{28, 320, 1, 3096}, {22, 306, 1, 3097}, {24, 307, 2, 3097}, {25, 308, 2, 3097}, {27, 309,
1, 3097}, {28, 310, 2, 3097}, {29, 311, 1, 3097}, {22, 307, 1, 3098}, {24, 309, 2, 3098},
{25, 310, 2, 3098}, {27, 312, 1, 3098}, {28, 313, 2, 3098}, {29, 314, 1, 3098}, {22, 308,
1, 3099}, {24, 310, 2, 3099}, {25, 311, 2, 3099}, {27, 313, 1, 3099}, {28, 314, 2, 3099},
{29, 315, 1, 3099}, {22, 309, 1, 3100}, {24, 312, 2, 3100}, {25, 313, 2, 3100}, {27, 316,
1, 3100}, {28, 317, 2, 3100}, {29, 318, 1, 3100}, {22, 310, 1, 3101}, {24, 313, 2, 3101},
{25, 314, 2, 3101}, {27, 317, 1, 3101}, {28, 318, 2, 3101}, {29, 319, 1, 3101}, {22, 311,
1, 3102}, {24, 314, 2, 3102}, {25, 315, 2, 3102}, {27, 318, 1, 3102}, {28, 319, 2, 3102},
{29, 320, 1, 3102}, {23, 306, 1, 3103}, {26, 307, 2, 3103}, {27, 308, 2, 3103}, {30, 309,
1, 3103}, {31, 310, 2, 3103}, {32, 311, 1, 3103}, {23, 307, 1, 3104}, {26, 309, 2, 3104},
{27, 310, 2, 3104}, {30, 312, 1, 3104}, {31, 313, 2, 3104}, {32, 314, 1, 3104}, {23, 308,
1, 3105}, {26, 310, 2, 3105}, {27, 311, 2, 3105}, {30, 313, 1, 3105}, {31, 314, 2, 3105},
{32, 315, 1, 3105}, {23, 309, 1, 3106}, {26, 312, 2, 3106}, {27, 313, 2, 3106}, {30, 316,
1, 3106}, {31, 317, 2, 3106}, {32, 318, 1, 3106}, {23, 310, 1, 3107}, {26, 313, 2, 3107},
{27, 314, 2, 3107}, {30, 317, 1, 3107}, {31, 318, 2, 3107}, {32, 319, 1, 3107}, {23, 311,
1, 3108}, {26, 314, 2, 3108}, {27, 315, 2, 3108}, {30, 318, 1, 3108}, {31, 319, 2, 3108},
{32, 320, 1, 3108}, {24, 306, 1, 3109}, {27, 307, 2, 3109}, {28, 308, 2, 3109}, {31, 309,
1, 3109}, {32, 310, 2, 3109}, {33, 311, 1, 3109}, {24, 307, 1, 3110}, {27, 309, 2, 3110},
{28, 310, 2, 3110}, {31, 312, 1, 3110}, {32, 313, 2, 3110}, {33, 314, 1, 3110}, {24, 308,
1, 3111}, {27, 310, 2, 3111}, {28, 311, 2, 3111}, {31, 313, 1, 3111}, {32, 314, 2, 3111},

{33, 315, 1, 3111}, {24, 309, 1, 3112}, {27, 312, 2, 3112}, {28, 313, 2, 3112}, {31, 316, 1, 3112}, {32, 317, 2, 3112}, {33, 318, 1, 3112}, {24, 310, 1, 3113}, {27, 313, 2, 3113}, {28, 314, 2, 3113}, {31, 317, 1, 3113}, {32, 318, 2, 3113}, {33, 319, 1, 3113}, {24, 311, 1, 3114}, {27, 314, 2, 3114}, {28, 315, 2, 3114}, {31, 318, 1, 3114}, {32, 319, 2, 3114}, {33, 320, 1, 3114}, {25, 306, 1, 3115}, {28, 307, 2, 3115}, {29, 308, 2, 3115}, {32, 309, 1, 3115}, {33, 310, 2, 3115}, {34, 311, 1, 3115}, {25, 307, 1, 3116}, {28, 309, 2, 3116}, {29, 310, 2, 3116}, {32, 312, 1, 3116}, {33, 313, 2, 3116}, {34, 314, 1, 3116}, {25, 308, 1, 3117}, {28, 310, 2, 3117}, {29, 311, 2, 3117}, {32, 313, 1, 3117}, {33, 314, 2, 3117}, {34, 315, 1, 3117}, {25, 309, 1, 3118}, {28, 312, 2, 3118}, {29, 313, 2, 3118}, {32, 316, 1, 3118}, {33, 317, 2, 3118}, {34, 318, 1, 3118}, {25, 310, 1, 3119}, {28, 313, 2, 3119}, {29, 314, 2, 3119}, {32, 317, 1, 3119}, {33, 318, 2, 3119}, {34, 319, 1, 3119}, {25, 311, 1, 3120}, {28, 314, 2, 3120}, {29, 315, 2, 3120}, {32, 318, 1, 3120}, {33, 319, 2, 3120}, {34, 320, 1, 3120}, {20, 321, 1, 3121}, {21, 322, 2, 3121}, {22, 323, 2, 3121}, {23, 324, 1, 3121}, {24, 325, 2, 3121}, {25, 326, 1, 3121}, {20, 322, 1, 3122}, {21, 324, 2, 3122}, {22, 325, 2, 3122}, {23, 327, 1, 3122}, {24, 328, 2, 3122}, {25, 329, 1, 3122}, {20, 323, 1, 3123}, {21, 325, 2, 3123}, {22, 326, 2, 3123}, {23, 328, 1, 3123}, {24, 329, 2, 3123}, {25, 330, 1, 3123}, {20, 324, 1, 3124}, {21, 327, 2, 3124}, {22, 328, 2, 3124}, {23, 331, 1, 3124}, {24, 332, 2, 3124}, {25, 333, 1, 3124}, {20, 325, 1, 3125}, {21, 328, 2, 3125}, {22, 329, 2, 3125}, {23, 332, 1, 3125}, {24, 333, 2, 3125}, {25, 334, 1, 3125}, {20, 326, 1, 3126}, {21, 329, 2, 3126}, {22, 330, 2, 3126}, {23, 333, 1, 3126}, {24, 334, 2, 3126}, {25, 335, 1, 3126}, {20, 327, 1, 3127}, {21, 331, 2, 3127}, {22, 332, 2, 3127}, {23, 336, 1, 3127}, {24, 337, 2, 3127}, {25, 338, 1, 3127}, {20, 328, 1, 3128}, {21, 332, 2, 3128}, {22, 333, 2, 3128}, {23, 337, 1, 3128}, {24, 338, 2, 3128}, {25, 339, 1, 3128}, {20, 329, 1, 3129}, {21, 333, 2, 3129}, {22, 334, 2, 3129}, {23, 338, 1, 3129}, {24, 339, 2, 3129}, {25, 340, 1, 3129}, {20, 330, 1, 3130}, {21, 334, 2, 3130}, {22, 335, 2, 3130}, {23, 339, 1, 3130}, {24, 340, 2, 3130}, {25, 341, 1, 3130}, {21, 321, 1, 3131}, {23, 322, 2, 3131}, {24, 323, 2, 3131}, {26, 324, 1, 3131}, {27, 325, 2, 3131}, {28, 326, 1, 3131}, {21, 322, 1, 3132}, {23, 324, 2, 3132}, {24, 325, 2, 3132}, {26, 327, 1, 3132}, {27, 328, 2, 3132}, {28, 329, 1, 3132}, {21, 323, 1, 3133}, {23, 325, 2, 3133}, {24, 326, 2, 3133}, {26, 328, 1, 3133}, {27, 329, 2, 3133}, {28, 330, 1, 3133}, {21, 324, 1, 3134}, {23, 327, 2, 3134}, {24, 328, 2, 3134}, {26, 331, 1, 3134}, {27, 332, 2, 3134}, {28, 333, 1, 3134}, {21, 325, 1, 3135}, {23, 328, 2, 3135}, {24, 329, 2, 3135}, {26, 332, 1, 3135}, {27, 333, 2, 3135}, {28, 334, 1, 3135}, {21, 326, 1, 3136}, {23, 329, 2, 3136}, {24, 330, 2, 3136}, {26, 333, 1, 3136}, {27, 334, 2, 3136}, {28, 335, 1, 3136}, {21, 327, 1, 3137}, {23, 331, 2, 3137}, {24, 332, 2, 3137}, {26, 336, 1, 3137}, {27, 337, 2, 3137}, {28, 338, 1, 3137}, {21, 328, 1, 3138}, {23, 332, 2, 3138}, {24, 333, 2, 3138}, {26, 337, 1, 3138}, {27, 338, 2, 3138}, {28, 339, 1, 3138}, {21, 329, 1, 3139}, {23, 333, 2, 3139}, {24, 334, 2, 3139}, {26, 338, 1, 3139}, {27, 339, 2, 3139}, {28, 340, 1, 3139}, {21, 330, 1, 3140}, {23, 334, 2, 3140}, {24, 335, 2, 3140}, {26, 339, 1, 3140}, {27, 340, 2, 3140}, {28, 341, 1, 3140}, {22, 321, 1, 3141}, {24, 322, 2, 3141}, {25, 323, 2, 3141}, {27, 324, 1, 3141}, {28, 325, 2, 3141}, {29, 326, 1, 3141}, {22, 322, 1, 3142}, {24, 324, 2, 3142}, {25, 325, 2, 3142}, {27, 327, 1, 3142}, {28, 328, 2, 3142}, {29, 329, 1, 3142}, {22, 323, 1, 3143}, {24, 325, 2, 3143}, {25, 326, 2, 3143}, {27, 328, 1, 3143}, {28, 329, 2, 3143}, {29, 330, 1, 3143}, {22, 324, 1, 3144}, {24, 327, 2, 3144}, {25, 328, 2, 3144}, {27, 331, 1, 3144}, {28, 332, 2, 3144}, {29, 333, 1, 3144}, {22, 325, 1, 3145}, {24, 328, 2, 3145}, {25, 329, 2, 3145}, {27, 332, 1, 3145}, {28, 333, 2, 3145}, {29, 334, 1, 3145}, {22, 326, 1, 3146}, {24, 329, 2, 3146}, {25, 330, 2, 3146}, {27, 333, 1, 3146}, {28, 334, 2, 3146}, {29, 335, 1, 3146}, {22, 327, 1, 3147}, {24, 331, 2, 3147}, {25, 332, 2, 3147}, {27, 336, 1, 3147}, {28, 337, 2, 3147}, {29, 338, 1, 3147}, {22, 328, 1, 3148}, {24, 332, 2, 3148}, {25, 333, 2, 3148}, {27, 337, 1, 3148}, {28, 338, 2, 3148}, {29, 339, 1, 3148}, {22, 329, 1, 3149}, {24, 333, 2, 3149}, {25, 334, 2, 3149}, {27, 338, 1, 3149}, {28, 339, 2, 3149}, {29, 340, 1, 3149}, {22, 330, 1, 3150}, {24, 334, 2, 3150}, {25, 335, 2, 3150}, {27, 339, 1, 3150}, {28, 340, 2, 3150}, {29, 341, 1, 3150}, {23, 321, 1, 3151}, {26, 322, 2, 3151}, {27, 323, 2, 3151}, {30, 324, 1, 3151}, {31, 325, 2, 3151}, {32, 326, 1, 3151}, {23, 322, 1, 3152}, {26, 324, 2, 3152}, {27, 325, 2, 3152}, {30, 327, 1, 3152}, {31, 328, 2, 3152}, {32, 329, 1, 3152}, {23, 323, 1, 3153}, {26, 325, 2, 3153}, {27, 326, 2, 3153}, {30, 328, 1, 3153}, {31, 329, 2, 3153}, {32, 330, 1, 3153}, {23, 324, 1, 3154}, {26, 327, 2, 3154}, {27, 328, 2, 3154}, {30, 331, 1, 3154}, {31, 332, 2, 3154}, {32, 333, 1, 3154}, {23, 325, 1, 3155}, {26, 328, 2, 3155}, {27, 329, 2, 3155}, {30, 332, 1, 3155}, {31, 333, 2, 3155}, {32, 334, 1, 3155}, {23, 326, 1, 3156}, {26, 329, 2, 3156}, {27, 330, 2, 3156}, {30, 333, 1, 3156}, {31, 334, 2, 3156}, {32, 335, 1, 3156}, {23, 327, 1, 3157}, {26, 331, 2, 3157}, {27, 332, 2, 3157}, {30, 336, 1, 3157}, {31, 337, 2, 3157}, {32, 338, 1, 3157}, {23, 328, 1, 3158}, {26, 332, 2, 3158}, {27, 333, 2, 3158}, {30, 337, 1, 3158}, {31, 338, 2, 3158}, {32, 339, 1, 3158}, {23, 329, 1, 3159}, {26, 333, 2, 3159}, {27, 334, 2, 3159}, {30, 338, 1, 3159}, {31, 339, 2, 3159}, {32, 340, 1, 3159}, {23, 330, 1, 3160}, {26, 334, 2, 3160}, {27, 335, 2, 3160}, {30, 339, 1, 3160}, {31, 340, 2, 3160}, {32, 341, 1, 3160}, {24, 321, 1, 3161}, {27, 322, 2, 3161}, {28, 323, 2, 3161}, {31, 324, 1, 3161}, {32, 325, 2, 3161}, {33, 326, 1, 3161}, {24, 322, 1, 3162}, {27, 324, 2, 3162}, {28, 325, 2, 3162}, {31, 327, 1, 3162}, {32, 328, 2, 3162}, {33, 329, 1, 3162}, {24, 323, 1, 3163}, {27, 325, 2, 3163}, {28, 326, 2, 3163}, {31, 328,

1, 3163}, {32, 329, 2, 3163}, {33, 330, 1, 3163}, {24, 324, 1, 3164}, {27, 327, 2, 3164}, {28, 328, 2, 3164}, {31, 331, 1, 3164}, {32, 332, 2, 3164}, {33, 333, 1, 3164}, {24, 325, 1, 3165}, {27, 328, 2, 3165}, {28, 329, 2, 3165}, {31, 332, 1, 3165}, {32, 333, 2, 3165}, {33, 334, 1, 3165}, {24, 326, 1, 3166}, {27, 329, 2, 3166}, {28, 330, 2, 3166}, {31, 333, 1, 3166}, {32, 334, 2, 3166}, {33, 335, 1, 3166}, {24, 327, 1, 3167}, {27, 331, 2, 3167}, {28, 332, 2, 3167}, {31, 336, 1, 3167}, {32, 337, 2, 3167}, {33, 338, 1, 3167}, {24, 328, 1, 3168}, {27, 332, 2, 3168}, {28, 333, 2, 3168}, {31, 337, 1, 3168}, {32, 338, 2, 3168}, {33, 339, 1, 3168}, {24, 329, 1, 3169}, {27, 333, 2, 3169}, {28, 334, 2, 3169}, {31, 338, 1, 3169}, {32, 339, 2, 3169}, {33, 340, 1, 3169}, {24, 330, 1, 3170}, {27, 334, 2, 3170}, {28, 335, 2, 3170}, {31, 339, 1, 3170}, {32, 340, 2, 3170}, {33, 341, 1, 3170}, {25, 321, 1, 3171}, {28, 322, 2, 3171}, {29, 323, 2, 3171}, {32, 324, 1, 3171}, {33, 325, 2, 3171}, {34, 326, 1, 3171}, {25, 322, 1, 3172}, {28, 324, 2, 3172}, {29, 325, 2, 3172}, {32, 327, 1, 3172}, {33, 328, 2, 3172}, {34, 329, 1, 3172}, {25, 323, 1, 3173}, {28, 325, 2, 3173}, {29, 326, 2, 3173}, {32, 328, 1, 3173}, {33, 329, 2, 3173}, {34, 330, 1, 3173}, {25, 324, 1, 3174}, {28, 327, 2, 3174}, {29, 328, 2, 3174}, {32, 331, 1, 3174}, {33, 332, 2, 3174}, {34, 333, 1, 3174}, {25, 325, 1, 3175}, {28, 328, 2, 3175}, {29, 329, 2, 3175}, {32, 332, 1, 3175}, {33, 333, 2, 3175}, {34, 334, 1, 3175}, {25, 326, 1, 3176}, {28, 329, 2, 3176}, {29, 330, 2, 3176}, {32, 333, 1, 3176}, {33, 334, 2, 3176}, {34, 335, 1, 3176}, {25, 327, 1, 3177}, {28, 331, 2, 3177}, {29, 332, 2, 3177}, {32, 336, 1, 3177}, {33, 337, 2, 3177}, {34, 338, 1, 3177}, {25, 328, 1, 3178}, {28, 332, 2, 3178}, {29, 333, 2, 3178}, {32, 337, 1, 3178}, {33, 338, 2, 3178}, {34, 339, 1, 3178}, {25, 329, 1, 3179}, {28, 333, 2, 3179}, {29, 334, 2, 3179}, {32, 338, 1, 3179}, {33, 339, 2, 3179}, {34, 340, 1, 3179}, {25, 330, 1, 3180}, {28, 334, 2, 3180}, {29, 335, 2, 3180}, {32, 339, 1, 3180}, {33, 340, 2, 3180}, {34, 341, 1, 3180}, {20, 296, 1, 3181}, {21, 297, 3, 3181}, {22, 298, 3, 3181}, {23, 299, 3, 3181}, {24, 300, 6, 3181}, {25, 301, 3, 3181}, {26, 302, 1, 3181}, {27, 303, 3, 3181}, {28, 304, 3, 3181}, {29, 305, 1, 3181}, {21, 296, 1, 3182}, {23, 297, 3, 3182}, {24, 298, 3, 3182}, {26, 299, 3, 3182}, {27, 300, 6, 3182}, {28, 301, 3, 3182}, {30, 302, 1, 3182}, {31, 303, 3, 3182}, {32, 304, 3, 3182}, {33, 305, 1, 3182}, {22, 296, 1, 3183}, {24, 297, 3, 3183}, {25, 298, 3, 3183}, {27, 299, 3, 3183}, {28, 300, 6, 3183}, {29, 301, 3, 3183}, {31, 302, 1, 3183}, {32, 303, 3, 3183}, {33, 304, 3, 3183}, {34, 305, 1, 3183}, {20, 306, 1, 3184}, {21, 307, 3, 3184}, {22, 308, 3, 3184}, {23, 309, 3, 3184}, {24, 310, 6, 3184}, {25, 311, 3, 3184}, {26, 312, 1, 3184}, {27, 313, 3, 3184}, {28, 314, 3, 3184}, {29, 315, 1, 3184}, {20, 307, 1, 3185}, {21, 309, 3, 3185}, {22, 310, 3, 3185}, {23, 312, 3, 3185}, {24, 313, 6, 3185}, {25, 314, 3, 3185}, {26, 316, 1, 3185}, {27, 317, 3, 3185}, {28, 318, 3, 3185}, {29, 319, 1, 3185}, {20, 308, 1, 3186}, {21, 310, 3, 3186}, {22, 311, 3, 3186}, {23, 313, 3, 3186}, {24, 314, 6, 3186}, {25, 315, 3, 3186}, {26, 317, 1, 3186}, {27, 318, 3, 3186}, {28, 319, 3, 3186}, {29, 320, 1, 3186}, {21, 306, 1, 3187}, {23, 307, 3, 3187}, {24, 308, 3, 3187}, {26, 309, 3, 3187}, {27, 310, 6, 3187}, {28, 311, 3, 3187}, {30, 312, 1, 3187}, {31, 313, 3, 3187}, {32, 314, 3, 3187}, {33, 315, 1, 3187}, {21, 307, 1, 3188}, {23, 309, 3, 3188}, {24, 310, 3, 3188}, {26, 312, 3, 3188}, {27, 313, 6, 3188}, {28, 314, 3, 3188}, {30, 316, 1, 3188}, {31, 317, 3, 3188}, {32, 318, 3, 3188}, {33, 319, 1, 3188}, {21, 308, 1, 3189}, {23, 310, 3, 3189}, {24, 311, 3, 3189}, {26, 313, 3, 3189}, {27, 314, 6, 3189}, {28, 315, 3, 3189}, {30, 317, 1, 3189}, {31, 318, 3, 3189}, {32, 319, 3, 3189}, {33, 320, 1, 3189}, {22, 306, 1, 3190}, {24, 307, 3, 3190}, {25, 308, 3, 3190}, {27, 309, 3, 3190}, {28, 310, 6, 3190}, {29, 311, 3, 3190}, {31, 312, 1, 3190}, {32, 313, 3, 3190}, {33, 314, 3, 3190}, {34, 315, 1, 3190}, {22, 307, 1, 3191}, {24, 309, 3, 3191}, {25, 310, 3, 3191}, {27, 312, 3, 3191}, {28, 313, 6, 3191}, {29, 314, 3, 3191}, {31, 316, 1, 3191}, {32, 317, 3, 3191}, {33, 318, 3, 3191}, {34, 319, 1, 3191}, {22, 308, 1, 3192}, {24, 310, 3, 3192}, {25, 311, 3, 3192}, {27, 313, 3, 3192}, {28, 314, 6, 3192}, {29, 315, 3, 3192}, {31, 317, 1, 3192}, {32, 318, 3, 3192}, {33, 319, 3, 3192}, {34, 320, 1, 3192}, {20, 321, 1, 3193}, {21, 322, 3, 3193}, {22, 323, 3, 3193}, {23, 324, 3, 3193}, {24, 325, 6, 3193}, {25, 326, 3, 3193}, {26, 327, 1, 3193}, {27, 328, 3, 3193}, {28, 329, 3, 3193}, {29, 330, 1, 3193}, {20, 322, 1, 3194}, {21, 324, 3, 3194}, {22, 325, 3, 3194}, {23, 327, 3, 3194}, {24, 328, 6, 3194}, {25, 329, 3, 3194}, {26, 331, 1, 3194}, {27, 332, 3, 3194}, {28, 333, 3, 3194}, {29, 334, 1, 3194}, {20, 323, 1, 3195}, {21, 325, 3, 3195}, {22, 326, 3, 3195}, {23, 328, 3, 3195}, {24, 329, 6, 3195}, {25, 330, 3, 3195}, {26, 332, 1, 3195}, {27, 333, 3, 3195}, {28, 334, 3, 3195}, {29, 335, 1, 3195}, {20, 324, 1, 3196}, {21, 327, 3, 3196}, {22, 328, 3, 3196}, {23, 331, 3, 3196}, {24, 332, 6, 3196}, {25, 333, 3, 3196}, {26, 336, 1, 3196}, {27, 337, 3, 3196}, {28, 338, 3, 3196}, {29, 339, 1, 3196}, {20, 325, 1, 3197}, {21, 328, 3, 3197}, {22, 329, 3, 3197}, {23, 332, 3, 3197}, {24, 333, 6, 3197}, {25, 334, 3, 3197}, {26, 337, 1, 3197}, {27, 338, 3, 3197}, {28, 339, 3, 3197}, {29, 340, 1, 3197}, {20, 326, 1, 3198}, {21, 329, 3, 3198}, {22, 330, 3, 3198}, {23, 333, 3, 3198}, {24, 334, 6, 3198}, {25, 335, 3, 3198}, {26, 338, 1, 3198}, {27, 339, 3, 3198}, {28, 340, 3, 3198}, {29, 341, 1, 3198}, {21, 321, 1, 3199}, {23, 322, 3, 3199}, {24, 323, 3, 3199}, {26, 324, 3, 3199}, {27, 325, 6, 3199}, {28, 326, 3, 3199}, {30, 327, 1, 3199}, {31, 328, 3, 3199}, {32, 329, 3, 3199}, {33, 330, 1, 3199}, {21, 322, 1, 3200}, {23, 324, 3, 3200}, {24, 325, 3, 3200}, {26, 327, 3, 3200}, {27, 328, 6, 3200}, {28, 329, 3, 3200}, {30, 331, 1, 3200}, {31, 332, 3, 3200}, {32, 333, 3, 3200}, {33, 334, 1, 3200}, {21, 323, 1, 3201}, {23, 325, 3, 3201}, {24, 326, 3, 3201}, {26, 328, 3, 3201}, {27, 329, 6, 3201}, {28, 330, 3, 3201},

{30, 332, 1, 3201}, {31, 333, 3, 3201}, {32, 334, 3, 3201}, {33, 335, 1, 3201}, {21, 324, 1, 3202}, {23, 327, 3, 3202}, {24, 328, 3, 3202}, {26, 331, 3, 3202}, {27, 332, 6, 3202}, {28, 333, 3, 3202}, {30, 336, 1, 3202}, {31, 337, 3, 3202}, {32, 338, 3, 3202}, {33, 339, 1, 3202}, {21, 325, 1, 3203}, {23, 328, 3, 3203}, {24, 329, 3, 3203}, {26, 332, 3, 3203}, {27, 333, 6, 3203}, {28, 334, 3, 3203}, {30, 337, 1, 3203}, {31, 338, 3, 3203}, {32, 339, 3, 3203}, {33, 340, 1, 3203}, {21, 326, 1, 3204}, {23, 329, 3, 3204}, {24, 330, 3, 3204}, {26, 333, 3, 3204}, {27, 334, 6, 3204}, {28, 335, 3, 3204}, {30, 338, 1, 3204}, {31, 339, 3, 3204}, {32, 340, 3, 3204}, {33, 341, 1, 3204}, {22, 321, 1, 3205}, {24, 322, 3, 3205}, {25, 323, 3, 3205}, {27, 324, 3, 3205}, {28, 325, 6, 3205}, {29, 326, 3, 3205}, {31, 327, 1, 3205}, {32, 328, 3, 3205}, {33, 329, 3, 3205}, {34, 330, 1, 3205}, {22, 322, 1, 3206}, {24, 324, 3, 3206}, {25, 325, 3, 3206}, {27, 327, 3, 3206}, {28, 328, 6, 3206}, {29, 329, 3, 3206}, {31, 331, 1, 3206}, {32, 332, 3, 3206}, {33, 333, 3, 3206}, {34, 334, 1, 3206}, {22, 323, 1, 3207}, {24, 325, 3, 3207}, {25, 326, 3, 3207}, {27, 328, 3, 3207}, {28, 329, 6, 3207}, {29, 330, 3, 3207}, {31, 332, 1, 3207}, {32, 333, 3, 3207}, {33, 334, 3, 3207}, {34, 335, 1, 3207}, {22, 324, 1, 3208}, {24, 327, 3, 3208}, {25, 328, 3, 3208}, {27, 331, 3, 3208}, {28, 332, 6, 3208}, {29, 333, 3, 3208}, {31, 336, 1, 3208}, {32, 337, 3, 3208}, {33, 338, 3, 3208}, {34, 339, 1, 3208}, {22, 325, 1, 3209}, {24, 328, 3, 3209}, {25, 329, 3, 3209}, {27, 332, 3, 3209}, {28, 333, 6, 3209}, {29, 334, 3, 3209}, {31, 337, 1, 3209}, {32, 338, 3, 3209}, {33, 339, 3, 3209}, {34, 340, 1, 3209}, {22, 326, 1, 3210}, {24, 329, 3, 3210}, {25, 330, 3, 3210}, {27, 333, 3, 3210}, {28, 334, 6, 3210}, {29, 335, 3, 3210}, {31, 338, 1, 3210}, {32, 339, 3, 3210}, {33, 340, 3, 3210}, {34, 341, 1, 3210}, {20, 342, 1, 3211}, {21, 343, 3, 3211}, {22, 344, 3, 3211}, {23, 345, 3, 3211}, {24, 346, 6, 3211}, {25, 347, 3, 3211}, {26, 348, 1, 3211}, {27, 349, 3, 3211}, {28, 350, 3, 3211}, {29, 351, 1, 3211}, {20, 343, 1, 3212}, {21, 345, 3, 3212}, {22, 346, 3, 3212}, {23, 348, 3, 3212}, {24, 349, 6, 3212}, {25, 350, 3, 3212}, {26, 352, 1, 3212}, {27, 353, 3, 3212}, {28, 354, 3, 3212}, {29, 355, 1, 3212}, {20, 344, 1, 3213}, {21, 346, 3, 3213}, {22, 347, 3, 3213}, {23, 349, 3, 3213}, {24, 350, 6, 3213}, {25, 351, 3, 3213}, {26, 353, 1, 3213}, {27, 354, 3, 3213}, {28, 355, 3, 3213}, {29, 356, 1, 3213}, {20, 345, 1, 3214}, {21, 348, 3, 3214}, {22, 349, 3, 3214}, {23, 352, 3, 3214}, {24, 353, 6, 3214}, {25, 354, 3, 3214}, {26, 357, 1, 3214}, {27, 358, 3, 3214}, {28, 359, 3, 3214}, {29, 360, 1, 3214}, {20, 346, 1, 3215}, {21, 349, 3, 3215}, {22, 350, 3, 3215}, {23, 353, 3, 3215}, {24, 354, 6, 3215}, {25, 355, 3, 3215}, {26, 358, 1, 3215}, {27, 359, 3, 3215}, {28, 360, 3, 3215}, {29, 361, 1, 3215}, {20, 347, 1, 3216}, {21, 350, 3, 3216}, {22, 351, 3, 3216}, {23, 354, 3, 3216}, {24, 355, 6, 3216}, {25, 356, 3, 3216}, {26, 359, 1, 3216}, {27, 360, 3, 3216}, {28, 361, 3, 3216}, {29, 362, 1, 3216}, {20, 348, 1, 3217}, {21, 352, 3, 3217}, {22, 353, 3, 3217}, {23, 357, 3, 3217}, {24, 358, 6, 3217}, {25, 359, 3, 3217}, {26, 363, 1, 3217}, {27, 364, 3, 3217}, {28, 365, 3, 3217}, {29, 366, 1, 3217}, {20, 349, 1, 3218}, {21, 353, 3, 3218}, {22, 354, 3, 3218}, {23, 358, 3, 3218}, {24, 359, 6, 3218}, {25, 360, 3, 3218}, {26, 364, 1, 3218}, {27, 365, 3, 3218}, {28, 366, 3, 3218}, {29, 367, 1, 3218}, {20, 350, 1, 3219}, {21, 354, 3, 3219}, {22, 355, 3, 3219}, {23, 359, 3, 3219}, {24, 360, 6, 3219}, {25, 361, 3, 3219}, {26, 365, 1, 3219}, {27, 366, 3, 3219}, {28, 367, 3, 3219}, {29, 368, 1, 3219}, {20, 351, 1, 3220}, {21, 355, 3, 3220}, {22, 356, 3, 3220}, {23, 360, 3, 3220}, {24, 361, 6, 3220}, {25, 362, 3, 3220}, {26, 366, 1, 3220}, {27, 367, 3, 3220}, {28, 368, 3, 3220}, {29, 369, 1, 3220}, {21, 342, 1, 3221}, {23, 343, 3, 3221}, {24, 344, 3, 3221}, {26, 345, 3, 3221}, {27, 346, 6, 3221}, {28, 347, 3, 3221}, {30, 348, 1, 3221}, {31, 349, 3, 3221}, {32, 350, 3, 3221}, {33, 351, 1, 3221}, {21, 343, 1, 3222}, {23, 345, 3, 3222}, {24, 346, 3, 3222}, {26, 348, 3, 3222}, {27, 349, 6, 3222}, {28, 350, 3, 3222}, {30, 352, 1, 3222}, {31, 353, 3, 3222}, {32, 354, 3, 3222}, {33, 355, 1, 3222}, {21, 344, 1, 3223}, {23, 346, 3, 3223}, {24, 347, 3, 3223}, {26, 349, 3, 3223}, {27, 350, 6, 3223}, {28, 351, 3, 3223}, {30, 353, 1, 3223}, {31, 354, 3, 3223}, {32, 355, 3, 3223}, {33, 356, 1, 3223}, {21, 345, 1, 3224}, {23, 348, 3, 3224}, {24, 349, 3, 3224}, {26, 352, 3, 3224}, {27, 353, 6, 3224}, {28, 354, 3, 3224}, {30, 357, 1, 3224}, {31, 358, 3, 3224}, {32, 359, 3, 3224}, {33, 360, 1, 3224}, {21, 346, 1, 3225}, {23, 349, 3, 3225}, {24, 350, 3, 3225}, {26, 353, 3, 3225}, {27, 354, 6, 3225}, {28, 355, 3, 3225}, {30, 358, 1, 3225}, {31, 359, 3, 3225}, {32, 360, 3, 3225}, {33, 361, 1, 3225}, {21, 347, 1, 3226}, {23, 350, 3, 3226}, {24, 351, 3, 3226}, {26, 354, 3, 3226}, {27, 355, 6, 3226}, {28, 356, 3, 3226}, {30, 359, 1, 3226}, {31, 360, 3, 3226}, {32, 361, 3, 3226}, {33, 362, 1, 3226}, {21, 348, 1, 3227}, {23, 352, 3, 3227}, {24, 353, 3, 3227}, {26, 357, 3, 3227}, {27, 358, 6, 3227}, {28, 359, 3, 3227}, {30, 363, 1, 3227}, {31, 364, 3, 3227}, {32, 365, 3, 3227}, {33, 366, 1, 3227}, {21, 349, 1, 3228}, {23, 353, 3, 3228}, {24, 354, 3, 3228}, {26, 358, 3, 3228}, {27, 359, 6, 3228}, {28, 360, 3, 3228}, {30, 364, 1, 3228}, {31, 365, 3, 3228}, {32, 366, 3, 3228}, {33, 367, 1, 3228}, {21, 350, 1, 3229}, {23, 354, 3, 3229}, {24, 355, 3, 3229}, {26, 359, 3, 3229}, {27, 360, 6, 3229}, {28, 361, 3, 3229}, {30, 365, 1, 3229}, {31, 366, 3, 3229}, {32, 367, 3, 3229}, {33, 368, 1, 3229}, {21, 351, 1, 3230}, {23, 355, 3, 3230}, {24, 356, 3, 3230}, {26, 360, 3, 3230}, {27, 361, 6, 3230}, {28, 362, 3, 3230}, {30, 366, 1, 3230}, {31, 367, 3, 3230}, {32, 368, 3, 3230}, {33, 369, 1, 3230}, {22, 342, 1, 3231}, {24, 343, 3, 3231}, {25, 344, 3, 3231}, {27, 345, 3, 3231}, {28, 346, 6, 3231}, {29, 347, 3, 3231}, {31, 348, 1, 3231}, {32, 349, 3, 3231}, {33, 350, 3, 3231}, {34, 351, 1, 3231}, {22, 343, 1, 3232}, {24, 345, 3, 3232}, {25, 346, 3, 3232}, {27, 348, 3, 3232}, {28, 349, 6, 3232}, {29, 350, 3, 3232}, {31, 352,

1, 3232}, {32, 353, 3, 3232}, {33, 354, 3, 3232}, {34, 355, 1, 3232}, {22, 344, 1, 3233}, {24, 346, 3, 3233}, {25, 347, 3, 3233}, {27, 349, 3, 3233}, {28, 350, 6, 3233}, {29, 351, 3, 3233}, {31, 353, 1, 3233}, {32, 354, 3, 3233}, {33, 355, 3, 3233}, {34, 356, 1, 3233}, {22, 345, 1, 3234}, {24, 348, 3, 3234}, {25, 349, 3, 3234}, {27, 352, 3, 3234}, {28, 353, 6, 3234}, {29, 354, 3, 3234}, {31, 357, 1, 3234}, {32, 358, 3, 3234}, {33, 359, 3, 3234}, {34, 360, 1, 3234}, {22, 346, 1, 3235}, {24, 349, 3, 3235}, {25, 350, 3, 3235}, {27, 353, 3, 3235}, {28, 354, 6, 3235}, {29, 355, 3, 3235}, {31, 358, 1, 3235}, {32, 359, 3, 3235}, {33, 360, 3, 3235}, {34, 361, 1, 3235}, {22, 347, 1, 3236}, {24, 350, 3, 3236}, {25, 351, 3, 3236}, {27, 354, 3, 3236}, {28, 355, 6, 3236}, {29, 356, 3, 3236}, {31, 359, 1, 3236}, {32, 360, 3, 3236}, {33, 361, 3, 3236}, {34, 362, 1, 3236}, {22, 348, 1, 3237}, {24, 352, 3, 3237}, {25, 353, 3, 3237}, {27, 357, 3, 3237}, {28, 358, 6, 3237}, {29, 359, 3, 3237}, {31, 363, 1, 3237}, {32, 364, 3, 3237}, {33, 365, 3, 3237}, {34, 366, 1, 3237}, {22, 349, 1, 3238}, {24, 353, 3, 3238}, {25, 354, 3, 3238}, {27, 358, 3, 3238}, {28, 359, 6, 3238}, {29, 360, 3, 3238}, {31, 364, 1, 3238}, {32, 365, 3, 3238}, {33, 366, 3, 3238}, {34, 367, 1, 3238}, {22, 350, 1, 3239}, {24, 354, 3, 3239}, {25, 355, 3, 3239}, {27, 359, 3, 3239}, {28, 360, 6, 3239}, {29, 361, 3, 3239}, {31, 365, 1, 3239}, {32, 366, 3, 3239}, {33, 367, 3, 3239}, {34, 368, 1, 3239}, {22, 351, 1, 3240}, {24, 355, 3, 3240}, {25, 356, 3, 3240}, {27, 360, 3, 3240}, {28, 361, 6, 3240}, {29, 362, 3, 3240}, {31, 366, 1, 3240}, {32, 367, 3, 3240}, {33, 368, 3, 3240}, {34, 369, 1, 3240}, {20, 306, 1, 3241}, {21, 307, 4, 3241}, {22, 308, 4, 3241}, {23, 309, 6, 3241}, {24, 310, 12, 3241}, {25, 311, 6, 3241}, {26, 312, 4, 3241}, {27, 313, 12, 3241}, {28, 314, 12, 3241}, {29, 315, 4, 3241}, {30, 316, 1, 3241}, {31, 317, 4, 3241}, {32, 318, 6, 3241}, {33, 319, 4, 3241}, {34, 320, 1, 3241}, {20, 321, 1, 3242}, {21, 322, 4, 3242}, {22, 323, 4, 3242}, {23, 324, 6, 3242}, {24, 325, 12, 3242}, {25, 326, 6, 3242}, {26, 327, 4, 3242}, {27, 328, 12, 3242}, {28, 329, 12, 3242}, {29, 330, 4, 3242}, {30, 331, 1, 3242}, {31, 332, 4, 3242}, {32, 333, 6, 3242}, {33, 334, 4, 3242}, {34, 335, 1, 3242}, {20, 322, 1, 3243}, {21, 324, 4, 3243}, {22, 325, 4, 3243}, {23, 327, 6, 3243}, {24, 328, 12, 3243}, {25, 329, 6, 3243}, {26, 331, 4, 3243}, {27, 332, 12, 3243}, {28, 333, 12, 3243}, {29, 334, 4, 3243}, {30, 336, 1, 3243}, {31, 337, 4, 3243}, {32, 338, 6, 3243}, {33, 339, 4, 3243}, {34, 340, 1, 3243}, {20, 323, 1, 3244}, {21, 325, 4, 3244}, {22, 326, 4, 3244}, {23, 328, 6, 3244}, {24, 329, 12, 3244}, {25, 330, 6, 3244}, {26, 332, 4, 3244}, {27, 333, 12, 3244}, {28, 334, 12, 3244}, {29, 335, 4, 3244}, {30, 337, 1, 3244}, {31, 338, 4, 3244}, {32, 339, 6, 3244}, {33, 340, 4, 3244}, {34, 341, 1, 3244}, {20, 342, 1, 3245}, {21, 343, 4, 3245}, {22, 344, 4, 3245}, {23, 345, 6, 3245}, {24, 346, 12, 3245}, {25, 347, 6, 3245}, {26, 348, 4, 3245}, {27, 349, 12, 3245}, {28, 350, 12, 3245}, {29, 351, 4, 3245}, {30, 352, 1, 3245}, {31, 353, 4, 3245}, {32, 354, 6, 3245}, {33, 355, 4, 3245}, {34, 356, 1, 3245}, {20, 343, 1, 3246}, {21, 345, 4, 3246}, {22, 346, 4, 3246}, {23, 348, 6, 3246}, {24, 349, 12, 3246}, {25, 350, 6, 3246}, {26, 352, 4, 3246}, {27, 353, 12, 3246}, {28, 354, 12, 3246}, {29, 355, 4, 3246}, {30, 357, 1, 3246}, {31, 358, 4, 3246}, {32, 359, 6, 3246}, {33, 360, 4, 3246}, {34, 361, 1, 3246}, {20, 344, 1, 3247}, {21, 346, 4, 3247}, {22, 347, 4, 3247}, {23, 349, 6, 3247}, {24, 350, 12, 3247}, {25, 351, 6, 3247}, {26, 353, 4, 3247}, {27, 354, 12, 3247}, {28, 355, 12, 3247}, {29, 356, 4, 3247}, {30, 358, 1, 3247}, {31, 359, 4, 3247}, {32, 360, 6, 3247}, {33, 361, 4, 3247}, {34, 362, 1, 3247}, {20, 345, 1, 3248}, {21, 348, 4, 3248}, {22, 349, 4, 3248}, {23, 352, 6, 3248}, {24, 353, 12, 3248}, {25, 354, 6, 3248}, {26, 357, 4, 3248}, {27, 358, 12, 3248}, {28, 359, 12, 3248}, {29, 360, 4, 3248}, {30, 363, 1, 3248}, {31, 364, 4, 3248}, {32, 365, 6, 3248}, {33, 366, 4, 3248}, {34, 367, 1, 3248}, {20, 346, 1, 3249}, {21, 349, 4, 3249}, {22, 350, 4, 3249}, {23, 353, 6, 3249}, {24, 354, 12, 3249}, {25, 355, 6, 3249}, {26, 358, 4, 3249}, {27, 359, 12, 3249}, {28, 360, 12, 3249}, {29, 361, 4, 3249}, {30, 364, 1, 3249}, {31, 365, 4, 3249}, {32, 366, 6, 3249}, {33, 367, 4, 3249}, {34, 368, 1, 3249}, {20, 347, 1, 3250}, {21, 350, 4, 3250}, {22, 351, 4, 3250}, {23, 354, 6, 3250}, {24, 355, 12, 3250}, {25, 356, 6, 3250}, {26, 359, 4, 3250}, {27, 360, 12, 3250}, {28, 361, 12, 3250}, {29, 362, 4, 3250}, {30, 365, 1, 3250}, {31, 366, 4, 3250}, {32, 367, 6, 3250}, {33, 368, 4, 3250}, {34, 369, 1, 3250}, {20, 370, 1, 3251}, {21, 371, 4, 3251}, {22, 372, 4, 3251}, {23, 373, 6, 3251}, {24, 374, 12, 3251}, {25, 375, 6, 3251}, {26, 376, 4, 3251}, {27, 377, 12, 3251}, {28, 378, 12, 3251}, {29, 379, 4, 3251}, {30, 380, 1, 3251}, {31, 381, 4, 3251}, {32, 382, 6, 3251}, {33, 383, 4, 3251}, {34, 384, 1, 3251}, {20, 371, 1, 3252}, {21, 373, 4, 3252}, {22, 374, 4, 3252}, {23, 376, 6, 3252}, {24, 377, 12, 3252}, {25, 378, 6, 3252}, {26, 380, 4, 3252}, {27, 381, 12, 3252}, {28, 382, 12, 3252}, {29, 383, 4, 3252}, {30, 385, 1, 3252}, {31, 386, 4, 3252}, {32, 387, 6, 3252}, {33, 388, 4, 3252}, {34, 389, 1, 3252}, {20, 372, 1, 3253}, {21, 374, 4, 3253}, {22, 375, 4, 3253}, {23, 377, 6, 3253}, {24, 378, 12, 3253}, {25, 379, 6, 3253}, {26, 381, 4, 3253}, {27, 382, 12, 3253}, {28, 383, 12, 3253}, {29, 384, 4, 3253}, {30, 386, 1, 3253}, {31, 387, 4, 3253}, {32, 388, 6, 3253}, {33, 389, 4, 3253}, {34, 390, 1, 3253}, {20, 373, 1, 3254}, {21, 376, 4, 3254}, {22, 377, 4, 3254}, {23, 380, 6, 3254}, {24, 381, 12, 3254}, {25, 382, 6, 3254}, {26, 385, 4, 3254}, {27, 386, 12, 3254}, {28, 387, 12, 3254}, {29, 388, 4, 3254}, {30, 391, 1, 3254}, {31, 392, 4, 3254}, {32, 393, 6, 3254}, {33, 394, 4, 3254}, {34, 395, 1, 3254}, {20, 374, 1, 3255}, {21, 377, 4, 3255}, {22, 378, 4, 3255}, {23, 381, 6, 3255}, {24, 382, 12, 3255}, {25, 383, 6, 3255}, {26, 386, 4, 3255}, {27, 387, 12, 3255}, {28, 388, 12, 3255}, {29, 389, 4,

3255}, {30, 392, 1, 3255}, {31, 393, 4, 3255}, {32, 394, 6, 3255}, {33, 395, 4, 3255}, {34, 396, 1, 3255}, {20, 375, 1, 3256}, {21, 378, 4, 3256}, {22, 379, 4, 3256}, {23, 382, 6, 3256}, {24, 383, 12, 3256}, {25, 384, 6, 3256}, {26, 387, 4, 3256}, {27, 388, 12, 3256}, {28, 389, 12, 3256}, {29, 390, 4, 3256}, {30, 393, 1, 3256}, {31, 394, 4, 3256}, {32, 395, 6, 3256}, {33, 396, 4, 3256}, {34, 397, 1, 3256}, {20, 376, 1, 3257}, {21, 380, 4, 3257}, {22, 381, 4, 3257}, {23, 385, 6, 3257}, {24, 386, 12, 3257}, {25, 387, 6, 3257}, {26, 391, 4, 3257}, {27, 392, 12, 3257}, {28, 393, 12, 3257}, {29, 394, 4, 3257}, {30, 398, 1, 3257}, {31, 399, 4, 3257}, {32, 400, 6, 3257}, {33, 401, 4, 3257}, {34, 402, 1, 3257}, {20, 377, 1, 3258}, {21, 381, 4, 3258}, {22, 382, 4, 3258}, {23, 386, 6, 3258}, {24, 387, 12, 3258}, {25, 388, 6, 3258}, {26, 392, 4, 3258}, {27, 393, 12, 3258}, {28, 394, 12, 3258}, {29, 395, 4, 3258}, {30, 399, 1, 3258}, {31, 400, 4, 3258}, {32, 401, 6, 3258}, {33, 402, 4, 3258}, {34, 403, 1, 3258}, {20, 378, 1, 3259}, {21, 382, 4, 3259}, {22, 383, 4, 3259}, {23, 387, 6, 3259}, {24, 388, 12, 3259}, {25, 389, 6, 3259}, {26, 393, 4, 3259}, {27, 394, 12, 3259}, {28, 395, 12, 3259}, {29, 396, 4, 3259}, {30, 400, 1, 3259}, {31, 401, 4, 3259}, {32, 402, 6, 3259}, {33, 403, 4, 3259}, {34, 404, 1, 3259}, {20, 379, 1, 3260}, {21, 383, 4, 3260}, {22, 384, 4, 3260}, {23, 388, 6, 3260}, {24, 389, 12, 3260}, {25, 390, 6, 3260}, {26, 394, 4, 3260}, {27, 395, 12, 3260}, {28, 396, 12, 3260}, {29, 397, 4, 3260}, {30, 401, 1, 3260}, {31, 402, 4, 3260}, {32, 403, 6, 3260}, {33, 404, 4, 3260}, {34, 405, 1, 3260}, {20, 406, 1, 3261}, {21, 407, 4, 3261}, {22, 408, 4, 3261}, {23, 409, 6, 3261}, {24, 410, 12, 3261}, {25, 411, 6, 3261}, {26, 412, 4, 3261}, {27, 413, 12, 3261}, {28, 414, 12, 3261}, {29, 415, 4, 3261}, {30, 416, 1, 3261}, {31, 417, 4, 3261}, {32, 418, 6, 3261}, {33, 419, 4, 3261}, {34, 420, 1, 3261}, {20, 407, 1, 3262}, {21, 409, 4, 3262}, {22, 410, 4, 3262}, {23, 412, 6, 3262}, {24, 413, 12, 3262}, {25, 414, 6, 3262}, {26, 416, 4, 3262}, {27, 417, 12, 3262}, {28, 418, 12, 3262}, {29, 419, 4, 3262}, {30, 421, 1, 3262}, {31, 422, 4, 3262}, {32, 423, 6, 3262}, {33, 424, 4, 3262}, {34, 425, 1, 3262}, {20, 408, 1, 3263}, {21, 410, 4, 3263}, {22, 411, 4, 3263}, {23, 413, 6, 3263}, {24, 414, 12, 3263}, {25, 415, 6, 3263}, {26, 417, 4, 3263}, {27, 418, 12, 3263}, {28, 419, 12, 3263}, {29, 420, 4, 3263}, {30, 422, 1, 3263}, {31, 423, 4, 3263}, {32, 424, 6, 3263}, {33, 425, 4, 3263}, {34, 426, 1, 3263}, {20, 409, 1, 3264}, {21, 412, 4, 3264}, {22, 413, 4, 3264}, {23, 416, 6, 3264}, {24, 417, 12, 3264}, {25, 418, 6, 3264}, {26, 421, 4, 3264}, {27, 422, 12, 3264}, {28, 423, 12, 3264}, {29, 424, 4, 3264}, {30, 427, 1, 3264}, {31, 428, 4, 3264}, {32, 429, 6, 3264}, {33, 430, 4, 3264}, {34, 431, 1, 3264}, {20, 410, 1, 3265}, {21, 413, 4, 3265}, {22, 414, 4, 3265}, {23, 417, 6, 3265}, {24, 418, 12, 3265}, {25, 419, 6, 3265}, {26, 422, 4, 3265}, {27, 423, 12, 3265}, {28, 424, 12, 3265}, {29, 425, 4, 3265}, {30, 428, 1, 3265}, {31, 429, 4, 3265}, {32, 430, 6, 3265}, {33, 431, 4, 3265}, {34, 432, 1, 3265}, {20, 411, 1, 3266}, {21, 414, 4, 3266}, {22, 415, 4, 3266}, {23, 418, 6, 3266}, {24, 419, 12, 3266}, {25, 420, 6, 3266}, {26, 423, 4, 3266}, {27, 424, 12, 3266}, {28, 425, 12, 3266}, {29, 426, 4, 3266}, {30, 429, 1, 3266}, {31, 430, 4, 3266}, {32, 431, 6, 3266}, {33, 432, 4, 3266}, {34, 433, 1, 3266}, {20, 412, 1, 3267}, {21, 416, 4, 3267}, {22, 417, 4, 3267}, {23, 421, 6, 3267}, {24, 422, 12, 3267}, {25, 423, 6, 3267}, {26, 427, 4, 3267}, {27, 428, 12, 3267}, {28, 429, 12, 3267}, {29, 430, 4, 3267}, {30, 434, 1, 3267}, {31, 435, 4, 3267}, {32, 436, 6, 3267}, {33, 437, 4, 3267}, {34, 438, 1, 3267}, {20, 413, 1, 3268}, {21, 417, 4, 3268}, {22, 418, 4, 3268}, {23, 422, 6, 3268}, {24, 423, 12, 3268}, {25, 424, 6, 3268}, {26, 428, 4, 3268}, {27, 429, 12, 3268}, {28, 430, 12, 3268}, {29, 431, 4, 3268}, {30, 435, 1, 3268}, {31, 436, 4, 3268}, {32, 437, 6, 3268}, {33, 438, 4, 3268}, {34, 439, 1, 3268}, {20, 414, 1, 3269}, {21, 418, 4, 3269}, {22, 419, 4, 3269}, {23, 423, 6, 3269}, {24, 424, 12, 3269}, {25, 425, 6, 3269}, {26, 429, 4, 3269}, {27, 430, 12, 3269}, {28, 431, 12, 3269}, {29, 432, 4, 3269}, {30, 436, 1, 3269}, {31, 437, 4, 3269}, {32, 438, 6, 3269}, {33, 439, 4, 3269}, {34, 440, 1, 3269}, {20, 415, 1, 3270}, {21, 419, 4, 3270}, {22, 420, 4, 3270}, {23, 424, 6, 3270}, {24, 425, 12, 3270}, {25, 426, 6, 3270}, {26, 430, 4, 3270}, {27, 431, 12, 3270}, {28, 432, 12, 3270}, {29, 433, 4, 3270}, {30, 437, 1, 3270}, {31, 438, 4, 3270}, {32, 439, 6, 3270}, {33, 440, 4, 3270}, {34, 441, 1, 3270}, {20, 416, 1, 3271}, {21, 421, 4, 3271}, {22, 422, 4, 3271}, {23, 427, 6, 3271}, {24, 428, 12, 3271}, {25, 429, 6, 3271}, {26, 434, 4, 3271}, {27, 435, 12, 3271}, {28, 436, 12, 3271}, {29, 437, 4, 3271}, {30, 442, 1, 3271}, {31, 443, 4, 3271}, {32, 444, 6, 3271}, {33, 445, 4, 3271}, {34, 446, 1, 3271}, {20, 417, 1, 3272}, {21, 422, 4, 3272}, {22, 423, 4, 3272}, {23, 428, 6, 3272}, {24, 429, 12, 3272}, {25, 430, 6, 3272}, {26, 435, 4, 3272}, {27, 436, 12, 3272}, {28, 437, 12, 3272}, {29, 438, 4, 3272}, {30, 443, 1, 3272}, {31, 444, 4, 3272}, {32, 445, 6, 3272}, {33, 446, 4, 3272}, {34, 447, 1, 3272}, {20, 418, 1, 3273}, {21, 423, 4, 3273}, {22, 424, 4, 3273}, {23, 429, 6, 3273}, {24, 430, 12, 3273}, {25, 431, 6, 3273}, {26, 436, 4, 3273}, {27, 437, 12, 3273}, {28, 438, 12, 3273}, {29, 439, 4, 3273}, {30, 444, 1, 3273}, {31, 445, 4, 3273}, {32, 446, 6, 3273}, {33, 447, 4, 3273}, {34, 448, 1, 3273}, {20, 419, 1, 3274}, {21, 424, 4, 3274}, {22, 425, 4, 3274}, {23, 430, 6, 3274}, {24, 431, 12, 3274}, {25, 432, 6, 3274}, {26, 437, 4, 3274}, {27, 438, 12, 3274}, {28, 439, 12, 3274}, {29, 440, 4, 3274}, {30, 445, 1, 3274}, {31, 446, 4, 3274}, {32, 447, 6, 3274}, {33, 448, 4, 3274}, {34, 449, 1, 3274}, {20, 420, 1, 3275}, {21, 425, 4, 3275}, {22, 426, 4, 3275}, {23, 431, 6, 3275}, {24, 432, 12, 3275}, {25, 433, 6, 3275}, {26, 438, 4, 3275}, {27, 439, 12, 3275}, {28, 440, 12, 3275}, {29, 441, 4, 3275}, {30,

446, 1, 3275}, {31, 447, 4, 3275}, {32, 448, 6, 3275}, {33, 449, 4, 3275}, {34, 450, 1,
3275}, {35, 290, 1, 3276}, {36, 291, 2, 3276}, {37, 292, 2, 3276}, {38, 293, 1, 3276},
{39, 294, 2, 3276}, {40, 295, 1, 3276}, {36, 290, 1, 3277}, {38, 291, 2, 3277}, {39, 292,
2, 3277}, {41, 293, 1, 3277}, {42, 294, 2, 3277}, {43, 295, 1, 3277}, {37, 290, 1, 3278},
{39, 291, 2, 3278}, {40, 292, 2, 3278}, {42, 293, 1, 3278}, {43, 294, 2, 3278}, {44, 295,
1, 3278}, {38, 290, 1, 3279}, {41, 291, 2, 3279}, {42, 292, 2, 3279}, {45, 293, 1, 3279},
{46, 294, 2, 3279}, {47, 295, 1, 3279}, {39, 290, 1, 3280}, {42, 291, 2, 3280}, {43, 292,
2, 3280}, {46, 293, 1, 3280}, {47, 294, 2, 3280}, {48, 295, 1, 3280}, {40, 290, 1, 3281},
{43, 291, 2, 3281}, {44, 292, 2, 3281}, {47, 293, 1, 3281}, {48, 294, 2, 3281}, {49, 295,
1, 3281}, {41, 290, 1, 3282}, {45, 291, 2, 3282}, {46, 292, 2, 3282}, {50, 293, 1, 3282},
{51, 294, 2, 3282}, {52, 295, 1, 3282}, {42, 290, 1, 3283}, {46, 291, 2, 3283}, {47, 292,
2, 3283}, {51, 293, 1, 3283}, {52, 294, 2, 3283}, {53, 295, 1, 3283}, {43, 290, 1, 3284},
{47, 291, 2, 3284}, {48, 292, 2, 3284}, {52, 293, 1, 3284}, {53, 294, 2, 3284}, {54, 295,
1, 3284}, {44, 290, 1, 3285}, {48, 291, 2, 3285}, {49, 292, 2, 3285}, {53, 293, 1, 3285},
{54, 294, 2, 3285}, {55, 295, 1, 3285}, {35, 296, 1, 3286}, {36, 297, 2, 3286}, {37, 298,
2, 3286}, {38, 299, 1, 3286}, {39, 300, 2, 3286}, {40, 301, 1, 3286}, {35, 297, 1, 3287},
{36, 299, 2, 3287}, {37, 300, 2, 3287}, {38, 302, 1, 3287}, {39, 303, 2, 3287}, {40, 304,
1, 3287}, {35, 298, 1, 3288}, {36, 300, 2, 3288}, {37, 301, 2, 3288}, {38, 303, 1, 3288},
{39, 304, 2, 3288}, {40, 305, 1, 3288}, {36, 296, 1, 3289}, {38, 297, 2, 3289}, {39, 298,
2, 3289}, {41, 299, 1, 3289}, {42, 300, 2, 3289}, {43, 301, 1, 3289}, {36, 297, 1, 3290},
{38, 299, 2, 3290}, {39, 300, 2, 3290}, {41, 302, 1, 3290}, {42, 303, 2, 3290}, {43, 304,
1, 3290}, {36, 298, 1, 3291}, {38, 300, 2, 3291}, {39, 301, 2, 3291}, {41, 303, 1, 3291},
{42, 304, 2, 3291}, {43, 305, 1, 3291}, {37, 296, 1, 3292}, {39, 297, 2, 3292}, {40, 298,
2, 3292}, {42, 299, 1, 3292}, {43, 300, 2, 3292}, {44, 301, 1, 3292}, {37, 297, 1, 3293},
{39, 299, 2, 3293}, {40, 300, 2, 3293}, {42, 302, 1, 3293}, {43, 303, 2, 3293}, {44, 304,
1, 3293}, {37, 298, 1, 3294}, {39, 300, 2, 3294}, {40, 301, 2, 3294}, {42, 303, 1, 3294},
{43, 304, 2, 3294}, {44, 305, 1, 3294}, {38, 296, 1, 3295}, {41, 297, 2, 3295}, {42, 298,
2, 3295}, {45, 299, 1, 3295}, {46, 300, 2, 3295}, {47, 301, 1, 3295}, {38, 297, 1, 3296},
{41, 299, 2, 3296}, {42, 300, 2, 3296}, {45, 302, 1, 3296}, {46, 303, 2, 3296}, {47, 304,
1, 3296}, {38, 298, 1, 3297}, {41, 300, 2, 3297}, {42, 301, 2, 3297}, {45, 303, 1, 3297},
{46, 304, 2, 3297}, {47, 305, 1, 3297}, {39, 296, 1, 3298}, {42, 297, 2, 3298}, {43, 298,
2, 3298}, {46, 299, 1, 3298}, {47, 300, 2, 3298}, {48, 301, 1, 3298}, {39, 297, 1, 3299},
{42, 299, 2, 3299}, {43, 300, 2, 3299}, {46, 302, 1, 3299}, {47, 303, 2, 3299}, {48, 304,
1, 3299}, {39, 298, 1, 3300}, {42, 300, 2, 3300}, {43, 301, 2, 3300}, {46, 303, 1, 3300},
{47, 304, 2, 3300}, {48, 305, 1, 3300}, {40, 296, 1, 3301}, {43, 297, 2, 3301}, {44, 298,
2, 3301}, {47, 299, 1, 3301}, {48, 300, 2, 3301}, {49, 301, 1, 3301}, {40, 297, 1, 3302},
{43, 299, 2, 3302}, {44, 300, 2, 3302}, {47, 302, 1, 3302}, {48, 303, 2, 3302}, {49, 304,
1, 3302}, {40, 298, 1, 3303}, {43, 300, 2, 3303}, {44, 301, 2, 3303}, {47, 303, 1, 3303},
{48, 304, 2, 3303}, {49, 305, 1, 3303}, {41, 296, 1, 3304}, {45, 297, 2, 3304}, {46, 298,
2, 3304}, {50, 299, 1, 3304}, {51, 300, 2, 3304}, {52, 301, 1, 3304}, {41, 297, 1, 3305},
{45, 299, 2, 3305}, {46, 300, 2, 3305}, {50, 302, 1, 3305}, {51, 303, 2, 3305}, {52, 304,
1, 3305}, {41, 298, 1, 3306}, {45, 300, 2, 3306}, {46, 301, 2, 3306}, {50, 303, 1, 3306},
{51, 304, 2, 3306}, {52, 305, 1, 3306}, {42, 296, 1, 3307}, {46, 297, 2, 3307}, {47, 298,
2, 3307}, {51, 299, 1, 3307}, {52, 300, 2, 3307}, {53, 301, 1, 3307}, {42, 297, 1, 3308},
{46, 299, 2, 3308}, {47, 300, 2, 3308}, {51, 302, 1, 3308}, {52, 303, 2, 3308}, {53, 304,
1, 3308}, {42, 298, 1, 3309}, {46, 300, 2, 3309}, {47, 301, 2, 3309}, {51, 303, 1, 3309},
{52, 304, 2, 3309}, {53, 305, 1, 3309}, {43, 296, 1, 3310}, {47, 297, 2, 3310}, {48, 298,
2, 3310}, {52, 299, 1, 3310}, {53, 300, 2, 3310}, {54, 301, 1, 3310}, {43, 297, 1, 3311},
{47, 299, 2, 3311}, {48, 300, 2, 3311}, {52, 302, 1, 3311}, {53, 303, 2, 3311}, {54, 304,
1, 3311}, {43, 298, 1, 3312}, {47, 300, 2, 3312}, {48, 301, 2, 3312}, {52, 303, 1, 3312},
{53, 304, 2, 3312}, {54, 305, 1, 3312}, {44, 296, 1, 3313}, {48, 297, 2, 3313}, {49, 298,
2, 3313}, {53, 299, 1, 3313}, {54, 300, 2, 3313}, {55, 301, 1, 3313}, {44, 297, 1, 3314},
{48, 299, 2, 3314}, {49, 300, 2, 3314}, {53, 302, 1, 3314}, {54, 303, 2, 3314}, {55, 304,
1, 3314}, {44, 298, 1, 3315}, {48, 300, 2, 3315}, {49, 301, 2, 3315}, {53, 303, 1, 3315},
{54, 304, 2, 3315}, {55, 305, 1, 3315}, {35, 306, 1, 3316}, {36, 307, 2, 3316}, {37, 308,
2, 3316}, {38, 309, 1, 3316}, {39, 310, 2, 3316}, {40, 311, 1, 3316}, {35, 307, 1, 3317},
{36, 309, 2, 3317}, {37, 310, 2, 3317}, {38, 312, 1, 3317}, {39, 313, 2, 3317}, {40, 314,
1, 3317}, {35, 308, 1, 3318}, {36, 310, 2, 3318}, {37, 311, 2, 3318}, {38, 313, 1, 3318},
{39, 314, 2, 3318}, {40, 315, 1, 3318}, {35, 309, 1, 3319}, {36, 312, 2, 3319}, {37, 313,
2, 3319}, {38, 316, 1, 3319}, {39, 317, 2, 3319}, {40, 318, 1, 3319}, {35, 310, 1, 3320},
{36, 313, 2, 3320}, {37, 314, 2, 3320}, {38, 317, 1, 3320}, {39, 318, 2, 3320}, {40, 319,
1, 3320}, {35, 311, 1, 3321}, {36, 314, 2, 3321}, {37, 315, 2, 3321}, {38, 318, 1, 3321},
{39, 319, 2, 3321}, {40, 320, 1, 3321}, {36, 306, 1, 3322}, {38, 307, 2, 3322}, {39, 308,
2, 3322}, {41, 309, 1, 3322}, {42, 310, 2, 3322}, {43, 311, 1, 3322}, {36, 307, 1, 3323},
{38, 309, 2, 3323}, {39, 310, 2, 3323}, {41, 312, 1, 3323}, {42, 313, 2, 3323}, {43, 314,
1, 3323}, {36, 308, 1, 3324}, {38, 310, 2, 3324}, {39, 311, 2, 3324}, {41, 313, 1, 3324},
{42, 314, 2, 3324}, {43, 315, 1, 3324}, {36, 309, 1, 3325}, {38, 312, 2, 3325}, {39, 313,
2, 3325}, {41, 316, 1, 3325}, {42, 317, 2, 3325}, {43, 318, 1, 3325}, {36, 310, 1, 3326},
{38, 313, 2, 3326}, {39, 314, 2, 3326}, {41, 317, 1, 3326}, {42, 318, 2, 3326}, {43, 319,

1, 3326}, {36, 311, 1, 3327}, {38, 314, 2, 3327}, {39, 315, 2, 3327}, {41, 318, 1, 3327},
{42, 319, 2, 3327}, {43, 320, 1, 3327}, {37, 306, 1, 3328}, {39, 307, 2, 3328}, {40, 308,
2, 3328}, {42, 309, 1, 3328}, {43, 310, 2, 3328}, {44, 311, 1, 3328}, {37, 307, 1, 3329},
{39, 309, 2, 3329}, {40, 310, 2, 3329}, {42, 312, 1, 3329}, {43, 313, 2, 3329}, {44, 314,
1, 3329}, {37, 308, 1, 3330}, {39, 310, 2, 3330}, {40, 311, 2, 3330}, {42, 313, 1, 3330},
{43, 314, 2, 3330}, {44, 315, 1, 3330}, {37, 309, 1, 3331}, {39, 312, 2, 3331}, {40, 313,
2, 3331}, {42, 316, 1, 3331}, {43, 317, 2, 3331}, {44, 318, 1, 3331}, {37, 310, 1, 3332},
{39, 313, 2, 3332}, {40, 314, 2, 3332}, {42, 317, 1, 3332}, {43, 318, 2, 3332}, {44, 319,
1, 3332}, {37, 311, 1, 3333}, {39, 314, 2, 3333}, {40, 315, 2, 3333}, {42, 318, 1, 3333},
{43, 319, 2, 3333}, {44, 320, 1, 3333}, {38, 306, 1, 3334}, {41, 307, 2, 3334}, {42, 308,
2, 3334}, {45, 309, 1, 3334}, {46, 310, 2, 3334}, {47, 311, 1, 3334}, {38, 307, 1, 3335},
{41, 309, 2, 3335}, {42, 310, 2, 3335}, {45, 312, 1, 3335}, {46, 313, 2, 3335}, {47, 314,
1, 3335}, {38, 308, 1, 3336}, {41, 310, 2, 3336}, {42, 311, 2, 3336}, {45, 313, 1, 3336},
{46, 314, 2, 3336}, {47, 315, 1, 3336}, {38, 309, 1, 3337}, {41, 312, 2, 3337}, {42, 313,
2, 3337}, {45, 316, 1, 3337}, {46, 317, 2, 3337}, {47, 318, 1, 3337}, {38, 310, 1, 3338},
{41, 313, 2, 3338}, {42, 314, 2, 3338}, {45, 317, 1, 3338}, {46, 318, 2, 3338}, {47, 319,
1, 3338}, {38, 311, 1, 3339}, {41, 314, 2, 3339}, {42, 315, 2, 3339}, {45, 318, 1, 3339},
{46, 319, 2, 3339}, {47, 320, 1, 3339}, {39, 306, 1, 3340}, {42, 307, 2, 3340}, {43, 308,
2, 3340}, {46, 309, 1, 3340}, {47, 310, 2, 3340}, {48, 311, 1, 3340}, {39, 307, 1, 3341},
{42, 309, 2, 3341}, {43, 310, 2, 3341}, {46, 312, 1, 3341}, {47, 313, 2, 3341}, {48, 314,
1, 3341}, {39, 308, 1, 3342}, {42, 310, 2, 3342}, {43, 311, 2, 3342}, {46, 313, 1, 3342},
{47, 314, 2, 3342}, {48, 315, 1, 3342}, {39, 309, 1, 3343}, {42, 312, 2, 3343}, {43, 313,
2, 3343}, {46, 316, 1, 3343}, {47, 317, 2, 3343}, {48, 318, 1, 3343}, {39, 310, 1, 3344},
{42, 313, 2, 3344}, {43, 314, 2, 3344}, {46, 317, 1, 3344}, {47, 318, 2, 3344}, {48, 319,
1, 3344}, {39, 311, 1, 3345}, {42, 314, 2, 3345}, {43, 315, 2, 3345}, {46, 318, 1, 3345},
{47, 319, 2, 3345}, {48, 320, 1, 3345}, {40, 306, 1, 3346}, {43, 307, 2, 3346}, {44, 308,
2, 3346}, {47, 309, 1, 3346}, {48, 310, 2, 3346}, {49, 311, 1, 3346}, {40, 307, 1, 3347},
{43, 309, 2, 3347}, {44, 310, 2, 3347}, {47, 312, 1, 3347}, {48, 313, 2, 3347}, {49, 314,
1, 3347}, {40, 308, 1, 3348}, {43, 310, 2, 3348}, {44, 311, 2, 3348}, {47, 313, 1, 3348},
{48, 314, 2, 3348}, {49, 315, 1, 3348}, {40, 309, 1, 3349}, {43, 312, 2, 3349}, {44, 313,
2, 3349}, {47, 316, 1, 3349}, {48, 317, 2, 3349}, {49, 318, 1, 3349}, {40, 310, 1, 3350},
{43, 313, 2, 3350}, {44, 314, 2, 3350}, {47, 317, 1, 3350}, {48, 318, 2, 3350}, {49, 319,
1, 3350}, {40, 311, 1, 3351}, {43, 314, 2, 3351}, {44, 315, 2, 3351}, {47, 318, 1, 3351},
{48, 319, 2, 3351}, {49, 320, 1, 3351}, {41, 306, 1, 3352}, {45, 307, 2, 3352}, {46, 308,
2, 3352}, {50, 309, 1, 3352}, {51, 310, 2, 3352}, {52, 311, 1, 3352}, {41, 307, 1, 3353},
{45, 309, 2, 3353}, {46, 310, 2, 3353}, {50, 312, 1, 3353}, {51, 313, 2, 3353}, {52, 314,
1, 3353}, {41, 308, 1, 3354}, {45, 310, 2, 3354}, {46, 311, 2, 3354}, {50, 313, 1, 3354},
{51, 314, 2, 3354}, {52, 315, 1, 3354}, {41, 309, 1, 3355}, {45, 312, 2, 3355}, {46, 313,
2, 3355}, {50, 316, 1, 3355}, {51, 317, 2, 3355}, {52, 318, 1, 3355}, {41, 310, 1, 3356},
{45, 313, 2, 3356}, {46, 314, 2, 3356}, {50, 317, 1, 3356}, {51, 318, 2, 3356}, {52, 319,
1, 3356}, {41, 311, 1, 3357}, {45, 314, 2, 3357}, {46, 315, 2, 3357}, {50, 318, 1, 3357},
{51, 319, 2, 3357}, {52, 320, 1, 3357}, {42, 306, 1, 3358}, {46, 307, 2, 3358}, {47, 308,
2, 3358}, {51, 309, 1, 3358}, {52, 310, 2, 3358}, {53, 311, 1, 3358}, {42, 307, 1, 3359},
{46, 309, 2, 3359}, {47, 310, 2, 3359}, {51, 312, 1, 3359}, {52, 313, 2, 3359}, {53, 314,
1, 3359}, {42, 308, 1, 3360}, {46, 310, 2, 3360}, {47, 311, 2, 3360}, {51, 313, 1, 3360},
{52, 314, 2, 3360}, {53, 315, 1, 3360}, {42, 309, 1, 3361}, {46, 312, 2, 3361}, {47, 313,
2, 3361}, {51, 316, 1, 3361}, {52, 317, 2, 3361}, {53, 318, 1, 3361}, {42, 310, 1, 3362},
{46, 313, 2, 3362}, {47, 314, 2, 3362}, {51, 317, 1, 3362}, {52, 318, 2, 3362}, {53, 319,
1, 3362}, {42, 311, 1, 3363}, {46, 314, 2, 3363}, {47, 315, 2, 3363}, {51, 318, 1, 3363},
{52, 319, 2, 3363}, {53, 320, 1, 3363}, {43, 306, 1, 3364}, {47, 307, 2, 3364}, {48, 308,
2, 3364}, {52, 309, 1, 3364}, {53, 310, 2, 3364}, {54, 311, 1, 3364}, {43, 307, 1, 3365},
{47, 309, 2, 3365}, {48, 310, 2, 3365}, {52, 312, 1, 3365}, {53, 313, 2, 3365}, {54, 314,
1, 3365}, {43, 308, 1, 3366}, {47, 310, 2, 3366}, {48, 311, 2, 3366}, {52, 313, 1, 3366},
{53, 314, 2, 3366}, {54, 315, 1, 3366}, {43, 309, 1, 3367}, {47, 312, 2, 3367}, {48, 313,
2, 3367}, {52, 316, 1, 3367}, {53, 317, 2, 3367}, {54, 318, 1, 3367}, {43, 310, 1, 3368},
{47, 313, 2, 3368}, {48, 314, 2, 3368}, {52, 317, 1, 3368}, {53, 318, 2, 3368}, {54, 319,
1, 3368}, {43, 311, 1, 3369}, {47, 314, 2, 3369}, {48, 315, 2, 3369}, {52, 318, 1, 3369},
{53, 319, 2, 3369}, {54, 320, 1, 3369}, {44, 306, 1, 3370}, {48, 307, 2, 3370}, {49, 308,
2, 3370}, {53, 309, 1, 3370}, {54, 310, 2, 3370}, {55, 311, 1, 3370}, {44, 307, 1, 3371},
{48, 309, 2, 3371}, {49, 310, 2, 3371}, {53, 312, 1, 3371}, {54, 313, 2, 3371}, {55, 314,
1, 3371}, {44, 308, 1, 3372}, {48, 310, 2, 3372}, {49, 311, 2, 3372}, {53, 313, 1, 3372},
{54, 314, 2, 3372}, {55, 315, 1, 3372}, {44, 309, 1, 3373}, {48, 312, 2, 3373}, {49, 313,
2, 3373}, {53, 316, 1, 3373}, {54, 317, 2, 3373}, {55, 318, 1, 3373}, {44, 310, 1, 3374},
{48, 313, 2, 3374}, {49, 314, 2, 3374}, {53, 317, 1, 3374}, {54, 318, 2, 3374}, {55, 319,
1, 3374}, {44, 311, 1, 3375}, {48, 314, 2, 3375}, {49, 315, 2, 3375}, {53, 318, 1, 3375},
{54, 319, 2, 3375}, {55, 320, 1, 3375}, {35, 296, 1, 3376}, {36, 297, 3, 3376}, {37, 298,
3, 3376}, {38, 299, 3, 3376}, {39, 300, 6, 3376}, {40, 301, 3, 3376}, {41, 302, 1, 3376},
{42, 303, 3, 3376}, {43, 304, 3, 3376}, {44, 305, 1, 3376}, {36, 296, 1, 3377}, {38, 297,
3, 3377}, {39, 298, 3, 3377}, {41, 299, 3, 3377}, {42, 300, 6, 3377}, {43, 301, 3, 3377},

{45, 302, 1, 3377}, {46, 303, 3, 3377}, {47, 304, 3, 3377}, {48, 305, 1, 3377}, {37, 296, 1, 3378}, {39, 297, 3, 3378}, {40, 298, 3, 3378}, {42, 299, 3, 3378}, {43, 300, 6, 3378}, {44, 301, 3, 3378}, {46, 302, 1, 3378}, {47, 303, 3, 3378}, {48, 304, 3, 3378}, {49, 305, 1, 3378}, {38, 296, 1, 3379}, {41, 297, 3, 3379}, {42, 298, 3, 3379}, {45, 299, 3, 3379}, {46, 300, 6, 3379}, {47, 301, 3, 3379}, {50, 302, 1, 3379}, {51, 303, 3, 3379}, {52, 304, 3, 3379}, {53, 305, 1, 3379}, {39, 296, 1, 3380}, {42, 297, 3, 3380}, {43, 298, 3, 3380}, {46, 299, 3, 3380}, {47, 300, 6, 3380}, {48, 301, 3, 3380}, {51, 302, 1, 3380}, {52, 303, 3, 3380}, {53, 304, 3, 3380}, {54, 305, 1, 3380}, {40, 296, 1, 3381}, {43, 297, 3, 3381}, {44, 298, 3, 3381}, {47, 299, 3, 3381}, {48, 300, 6, 3381}, {49, 301, 3, 3381}, {52, 302, 1, 3381}, {53, 303, 3, 3381}, {54, 304, 3, 3381}, {55, 305, 1, 3381}, {35, 306, 1, 3382}, {36, 307, 3, 3382}, {37, 308, 3, 3382}, {38, 309, 3, 3382}, {39, 310, 6, 3382}, {40, 311, 3, 3382}, {41, 312, 1, 3382}, {42, 313, 3, 3382}, {43, 314, 3, 3382}, {44, 315, 1, 3382}, {35, 307, 1, 3383}, {36, 309, 3, 3383}, {37, 310, 3, 3383}, {38, 312, 3, 3383}, {39, 313, 6, 3383}, {40, 314, 3, 3383}, {41, 316, 1, 3383}, {42, 317, 3, 3383}, {43, 318, 3, 3383}, {44, 319, 1, 3383}, {35, 308, 1, 3384}, {36, 310, 3, 3384}, {37, 311, 3, 3384}, {38, 313, 3, 3384}, {39, 314, 6, 3384}, {40, 315, 3, 3384}, {41, 317, 1, 3384}, {42, 318, 3, 3384}, {43, 319, 3, 3384}, {44, 320, 1, 3384}, {36, 306, 1, 3385}, {38, 307, 3, 3385}, {39, 308, 3, 3385}, {41, 309, 3, 3385}, {42, 310, 6, 3385}, {43, 311, 3, 3385}, {45, 312, 1, 3385}, {46, 313, 3, 3385}, {47, 314, 3, 3385}, {48, 315, 1, 3385}, {36, 307, 1, 3386}, {38, 309, 3, 3386}, {39, 310, 3, 3386}, {41, 312, 3, 3386}, {42, 313, 6, 3386}, {43, 314, 3, 3386}, {45, 316, 1, 3386}, {46, 317, 3, 3386}, {47, 318, 3, 3386}, {48, 319, 1, 3386}, {36, 308, 1, 3387}, {38, 310, 3, 3387}, {39, 311, 3, 3387}, {41, 313, 3, 3387}, {42, 314, 6, 3387}, {43, 315, 3, 3387}, {45, 317, 1, 3387}, {46, 318, 3, 3387}, {47, 319, 3, 3387}, {48, 320, 1, 3387}, {37, 306, 1, 3388}, {39, 307, 3, 3388}, {40, 308, 3, 3388}, {42, 309, 3, 3388}, {43, 310, 6, 3388}, {44, 311, 3, 3388}, {46, 312, 1, 3388}, {47, 313, 3, 3388}, {48, 314, 3, 3388}, {49, 315, 1, 3388}, {37, 307, 1, 3389}, {39, 309, 3, 3389}, {40, 310, 3, 3389}, {42, 312, 3, 3389}, {43, 313, 6, 3389}, {44, 314, 3, 3389}, {46, 316, 1, 3389}, {47, 317, 3, 3389}, {48, 318, 3, 3389}, {49, 319, 1, 3389}, {37, 308, 1, 3390}, {39, 310, 3, 3390}, {40, 311, 3, 3390}, {42, 313, 3, 3390}, {43, 314, 6, 3390}, {44, 315, 3, 3390}, {46, 317, 1, 3390}, {47, 318, 3, 3390}, {48, 319, 3, 3390}, {49, 320, 1, 3390}, {38, 306, 1, 3391}, {41, 307, 3, 3391}, {42, 308, 3, 3391}, {45, 309, 3, 3391}, {46, 310, 6, 3391}, {47, 311, 3, 3391}, {50, 312, 1, 3391}, {51, 313, 3, 3391}, {52, 314, 3, 3391}, {53, 315, 1, 3391}, {38, 307, 1, 3392}, {41, 309, 3, 3392}, {42, 310, 3, 3392}, {45, 312, 3, 3392}, {46, 313, 6, 3392}, {47, 314, 3, 3392}, {50, 316, 1, 3392}, {51, 317, 3, 3392}, {52, 318, 3, 3392}, {53, 319, 1, 3392}, {38, 308, 1, 3393}, {41, 310, 3, 3393}, {42, 311, 3, 3393}, {45, 313, 3, 3393}, {46, 314, 6, 3393}, {47, 315, 3, 3393}, {50, 317, 1, 3393}, {51, 318, 3, 3393}, {52, 319, 3, 3393}, {53, 320, 1, 3393}, {39, 306, 1, 3394}, {42, 307, 3, 3394}, {43, 308, 3, 3394}, {46, 309, 3, 3394}, {47, 310, 6, 3394}, {48, 311, 3, 3394}, {51, 312, 1, 3394}, {52, 313, 3, 3394}, {53, 314, 3, 3394}, {54, 315, 1, 3394}, {39, 307, 1, 3395}, {42, 309, 3, 3395}, {43, 310, 3, 3395}, {46, 312, 3, 3395}, {47, 313, 6, 3395}, {48, 314, 3, 3395}, {51, 316, 1, 3395}, {52, 317, 3, 3395}, {53, 318, 3, 3395}, {54, 319, 1, 3395}, {39, 308, 1, 3396}, {42, 310, 3, 3396}, {43, 311, 3, 3396}, {46, 313, 3, 3396}, {47, 314, 6, 3396}, {48, 315, 3, 3396}, {51, 317, 1, 3396}, {52, 318, 3, 3396}, {53, 319, 3, 3396}, {54, 320, 1, 3396}, {40, 306, 1, 3397}, {43, 307, 3, 3397}, {44, 308, 3, 3397}, {47, 309, 3, 3397}, {48, 310, 6, 3397}, {49, 311, 3, 3397}, {52, 312, 1, 3397}, {53, 313, 3, 3397}, {54, 314, 3, 3397}, {55, 315, 1, 3397}, {40, 307, 1, 3398}, {43, 309, 3, 3398}, {44, 310, 3, 3398}, {47, 312, 3, 3398}, {48, 313, 6, 3398}, {49, 314, 3, 3398}, {52, 316, 1, 3398}, {53, 317, 3, 3398}, {54, 318, 3, 3398}, {55, 319, 1, 3398}, {40, 308, 1, 3399}, {43, 310, 3, 3399}, {44, 311, 3, 3399}, {47, 313, 3, 3399}, {48, 314, 6, 3399}, {49, 315, 3, 3399}, {52, 317, 1, 3399}, {53, 318, 3, 3399}, {54, 319, 3, 3399}, {55, 320, 1, 3399}, {35, 321, 1, 3400}, {36, 322, 3, 3400}, {37, 323, 3, 3400}, {38, 324, 3, 3400}, {39, 325, 6, 3400}, {40, 326, 3, 3400}, {41, 327, 1, 3400}, {42, 328, 3, 3400}, {43, 329, 3, 3400}, {44, 330, 1, 3400}, {35, 322, 1, 3401}, {36, 324, 3, 3401}, {37, 325, 3, 3401}, {38, 327, 3, 3401}, {39, 328, 6, 3401}, {40, 329, 3, 3401}, {41, 331, 1, 3401}, {42, 332, 3, 3401}, {43, 333, 3, 3401}, {44, 334, 1, 3401}, {35, 323, 1, 3402}, {36, 325, 3, 3402}, {37, 326, 3, 3402}, {38, 328, 3, 3402}, {39, 329, 6, 3402}, {40, 330, 3, 3402}, {41, 332, 1, 3402}, {42, 333, 3, 3402}, {43, 334, 3, 3402}, {44, 335, 1, 3402}, {35, 324, 1, 3403}, {36, 327, 3, 3403}, {37, 328, 3, 3403}, {38, 331, 3, 3403}, {39, 332, 6, 3403}, {40, 333, 3, 3403}, {41, 336, 1, 3403}, {42, 337, 3, 3403}, {43, 338, 3, 3403}, {44, 339, 1, 3403}, {35, 325, 1, 3404}, {36, 328, 3, 3404}, {37, 329, 3, 3404}, {38, 332, 3, 3404}, {39, 333, 6, 3404}, {40, 334, 3, 3404}, {41, 337, 1, 3404}, {42, 338, 3, 3404}, {43, 339, 3, 3404}, {44, 340, 1, 3404}, {35, 326, 1, 3405}, {36, 329, 3, 3405}, {37, 330, 3, 3405}, {38, 333, 3, 3405}, {39, 334, 6, 3405}, {40, 335, 3, 3405}, {41, 338, 1, 3405}, {42, 339, 3, 3405}, {43, 340, 3, 3405}, {44, 341, 1, 3405}, {36, 321, 1, 3406}, {38, 322, 3, 3406}, {39, 323, 3, 3406}, {41, 324, 3, 3406}, {42, 325, 6, 3406}, {43, 326, 3, 3406}, {45, 327, 1, 3406}, {46, 328, 3, 3406}, {47, 329, 3, 3406}, {48, 330, 1, 3406}, {36, 322, 1, 3407}, {38, 324, 3, 3407}, {39, 325, 3, 3407}, {41, 327, 3, 3407}, {42, 328, 6, 3407}, {43, 329, 3, 3407}, {45, 331, 1, 3407}, {46, 332, 3, 3407}, {47, 333, 3, 3407}, {48, 334, 1, 3407}, {36, 323, 1, 3408}, {38, 325, 3, 3408}, {39, 326, 3, 3408}, {41, 328, 3, 3408}, {42, 329, 6, 3408}, {43, 330, 3, 3408}, {45, 332,

1, 3408}, {46, 333, 3, 3408}, {47, 334, 3, 3408}, {48, 335, 1, 3408}, {36, 324, 1, 3409}, {38, 327, 3, 3409}, {39, 328, 3, 3409}, {41, 331, 3, 3409}, {42, 332, 6, 3409}, {43, 333, 3, 3409}, {45, 336, 1, 3409}, {46, 337, 3, 3409}, {47, 338, 3, 3409}, {48, 339, 1, 3409}, {36, 325, 1, 3410}, {38, 328, 3, 3410}, {39, 329, 3, 3410}, {41, 332, 3, 3410}, {42, 333, 6, 3410}, {43, 334, 3, 3410}, {45, 337, 1, 3410}, {46, 338, 3, 3410}, {47, 339, 3, 3410}, {48, 340, 1, 3410}, {36, 326, 1, 3411}, {38, 329, 3, 3411}, {39, 330, 3, 3411}, {41, 333, 3, 3411}, {42, 334, 6, 3411}, {43, 335, 3, 3411}, {45, 338, 1, 3411}, {46, 339, 3, 3411}, {47, 340, 3, 3411}, {48, 341, 1, 3411}, {37, 321, 1, 3412}, {39, 322, 3, 3412}, {40, 323, 3, 3412}, {42, 324, 3, 3412}, {43, 325, 6, 3412}, {44, 326, 3, 3412}, {46, 327, 1, 3412}, {47, 328, 3, 3412}, {48, 329, 3, 3412}, {49, 330, 1, 3412}, {37, 322, 1, 3413}, {39, 324, 3, 3413}, {40, 325, 3, 3413}, {42, 327, 3, 3413}, {43, 328, 6, 3413}, {44, 329, 3, 3413}, {46, 331, 1, 3413}, {47, 332, 3, 3413}, {48, 333, 3, 3413}, {49, 334, 1, 3413}, {37, 323, 1, 3414}, {39, 325, 3, 3414}, {40, 326, 3, 3414}, {42, 328, 3, 3414}, {43, 329, 6, 3414}, {44, 330, 3, 3414}, {46, 332, 1, 3414}, {47, 333, 3, 3414}, {48, 334, 3, 3414}, {49, 335, 1, 3414}, {37, 324, 1, 3415}, {39, 327, 3, 3415}, {40, 328, 3, 3415}, {42, 331, 3, 3415}, {43, 332, 6, 3415}, {44, 333, 3, 3415}, {46, 336, 1, 3415}, {47, 337, 3, 3415}, {48, 338, 3, 3415}, {49, 339, 1, 3415}, {37, 325, 1, 3416}, {39, 328, 3, 3416}, {40, 329, 3, 3416}, {42, 332, 3, 3416}, {43, 333, 6, 3416}, {44, 334, 3, 3416}, {46, 337, 1, 3416}, {47, 338, 3, 3416}, {48, 339, 3, 3416}, {49, 340, 1, 3416}, {37, 326, 1, 3417}, {39, 329, 3, 3417}, {40, 330, 3, 3417}, {42, 333, 3, 3417}, {43, 334, 6, 3417}, {44, 335, 3, 3417}, {46, 338, 1, 3417}, {47, 339, 3, 3417}, {48, 340, 3, 3417}, {49, 341, 1, 3417}, {38, 321, 1, 3418}, {41, 322, 3, 3418}, {42, 323, 3, 3418}, {45, 324, 3, 3418}, {46, 325, 6, 3418}, {47, 326, 3, 3418}, {50, 327, 1, 3418}, {51, 328, 3, 3418}, {52, 329, 3, 3418}, {53, 330, 1, 3418}, {38, 322, 1, 3419}, {41, 324, 3, 3419}, {42, 325, 3, 3419}, {45, 327, 3, 3419}, {46, 328, 6, 3419}, {47, 329, 3, 3419}, {50, 331, 1, 3419}, {51, 332, 3, 3419}, {52, 333, 3, 3419}, {53, 334, 1, 3419}, {38, 323, 1, 3420}, {41, 325, 3, 3420}, {42, 326, 3, 3420}, {45, 328, 3, 3420}, {46, 329, 6, 3420}, {47, 330, 3, 3420}, {50, 332, 1, 3420}, {51, 333, 3, 3420}, {52, 334, 3, 3420}, {53, 335, 1, 3420}, {38, 324, 1, 3421}, {41, 327, 3, 3421}, {42, 328, 3, 3421}, {45, 331, 3, 3421}, {46, 332, 6, 3421}, {47, 333, 3, 3421}, {50, 336, 1, 3421}, {51, 337, 3, 3421}, {52, 338, 3, 3421}, {53, 339, 1, 3421}, {38, 325, 1, 3422}, {41, 328, 3, 3422}, {42, 329, 3, 3422}, {45, 332, 3, 3422}, {46, 333, 6, 3422}, {47, 334, 3, 3422}, {50, 337, 1, 3422}, {51, 338, 3, 3422}, {52, 339, 3, 3422}, {53, 340, 1, 3422}, {38, 326, 1, 3423}, {41, 329, 3, 3423}, {42, 330, 3, 3423}, {45, 333, 3, 3423}, {46, 334, 6, 3423}, {47, 335, 3, 3423}, {50, 338, 1, 3423}, {51, 339, 3, 3423}, {52, 340, 3, 3423}, {53, 341, 1, 3423}, {39, 321, 1, 3424}, {42, 322, 3, 3424}, {43, 323, 3, 3424}, {46, 324, 3, 3424}, {47, 325, 6, 3424}, {48, 326, 3, 3424}, {51, 327, 1, 3424}, {52, 328, 3, 3424}, {53, 329, 3, 3424}, {54, 330, 1, 3424}, {39, 322, 1, 3425}, {42, 324, 3, 3425}, {43, 325, 3, 3425}, {46, 327, 3, 3425}, {47, 328, 6, 3425}, {48, 329, 3, 3425}, {51, 331, 1, 3425}, {52, 332, 3, 3425}, {53, 333, 3, 3425}, {54, 334, 1, 3425}, {39, 323, 1, 3426}, {42, 325, 3, 3426}, {43, 326, 3, 3426}, {46, 328, 3, 3426}, {47, 329, 6, 3426}, {48, 330, 3, 3426}, {51, 332, 1, 3426}, {52, 333, 3, 3426}, {53, 334, 3, 3426}, {54, 335, 1, 3426}, {39, 324, 1, 3427}, {42, 327, 3, 3427}, {43, 328, 3, 3427}, {46, 331, 3, 3427}, {47, 332, 6, 3427}, {48, 333, 3, 3427}, {51, 336, 1, 3427}, {52, 337, 3, 3427}, {53, 338, 3, 3427}, {54, 339, 1, 3427}, {39, 325, 1, 3428}, {42, 328, 3, 3428}, {43, 329, 3, 3428}, {46, 332, 3, 3428}, {47, 333, 6, 3428}, {48, 334, 3, 3428}, {51, 337, 1, 3428}, {52, 338, 3, 3428}, {53, 339, 3, 3428}, {54, 340, 1, 3428}, {39, 326, 1, 3429}, {42, 329, 3, 3429}, {43, 330, 3, 3429}, {46, 333, 3, 3429}, {47, 334, 6, 3429}, {48, 335, 3, 3429}, {51, 338, 1, 3429}, {52, 339, 3, 3429}, {53, 340, 3, 3429}, {54, 341, 1, 3429}, {40, 321, 1, 3430}, {43, 322, 3, 3430}, {44, 323, 3, 3430}, {47, 324, 3, 3430}, {48, 325, 6, 3430}, {49, 326, 3, 3430}, {52, 327, 1, 3430}, {53, 328, 3, 3430}, {54, 329, 3, 3430}, {55, 330, 1, 3430}, {40, 322, 1, 3431}, {43, 324, 3, 3431}, {44, 325, 3, 3431}, {47, 327, 3, 3431}, {48, 328, 6, 3431}, {49, 329, 3, 3431}, {52, 331, 1, 3431}, {53, 332, 3, 3431}, {54, 333, 3, 3431}, {55, 334, 1, 3431}, {40, 323, 1, 3432}, {43, 325, 3, 3432}, {44, 326, 3, 3432}, {47, 328, 3, 3432}, {48, 329, 6, 3432}, {49, 330, 3, 3432}, {52, 332, 1, 3432}, {53, 333, 3, 3432}, {54, 334, 3, 3432}, {55, 335, 1, 3432}, {40, 324, 1, 3433}, {43, 327, 3, 3433}, {44, 328, 3, 3433}, {47, 331, 3, 3433}, {48, 332, 6, 3433}, {49, 333, 3, 3433}, {52, 336, 1, 3433}, {53, 337, 3, 3433}, {54, 338, 3, 3433}, {55, 339, 1, 3433}, {40, 325, 1, 3434}, {43, 328, 3, 3434}, {44, 329, 3, 3434}, {47, 332, 3, 3434}, {48, 333, 6, 3434}, {49, 334, 3, 3434}, {52, 337, 1, 3434}, {53, 338, 3, 3434}, {54, 339, 3, 3434}, {55, 340, 1, 3434}, {40, 326, 1, 3435}, {43, 329, 3, 3435}, {44, 330, 3, 3435}, {47, 333, 3, 3435}, {48, 334, 6, 3435}, {49, 335, 3, 3435}, {52, 338, 1, 3435}, {53, 339, 3, 3435}, {54, 340, 3, 3435}, {55, 341, 1, 3435}, {35, 342, 1, 3436}, {36, 343, 3, 3436}, {37, 344, 3, 3436}, {38, 345, 3, 3436}, {39, 346, 6, 3436}, {40, 347, 3, 3436}, {41, 348, 1, 3436}, {42, 349, 3, 3436}, {43, 350, 3, 3436}, {44, 351, 1, 3436}, {35, 343, 1, 3437}, {36, 345, 3, 3437}, {37, 346, 3, 3437}, {38, 348, 3, 3437}, {39, 349, 6, 3437}, {40, 350, 3, 3437}, {41, 352, 1, 3437}, {42, 353, 3, 3437}, {43, 354, 3, 3437}, {44, 355, 1, 3437}, {35, 344, 1, 3438}, {36, 346, 3, 3438}, {37, 347, 3, 3438}, {38, 349, 3, 3438}, {39, 350, 6, 3438}, {40, 351, 3, 3438}, {41, 353, 1, 3438}, {42, 354, 3, 3438}, {43, 355, 3, 3438}, {44, 356, 1, 3438}, {35, 345, 1, 3439}, {36, 348, 3, 3439}, {37, 349, 3, 3439}, {38, 352, 3, 3439}, {39, 353, 6, 3439}, {40, 354, 3, 3439}, {41, 357, 1, 3439},

{42, 358, 3, 3439}, {43, 359, 3, 3439}, {44, 360, 1, 3439}, {35, 346, 1, 3440}, {36, 349, 3, 3440}, {37, 350, 3, 3440}, {38, 353, 3, 3440}, {39, 354, 6, 3440}, {40, 355, 3, 3440}, {41, 358, 1, 3440}, {42, 359, 3, 3440}, {43, 360, 3, 3440}, {44, 361, 1, 3440}, {35, 347, 1, 3441}, {36, 350, 3, 3441}, {37, 351, 3, 3441}, {38, 354, 3, 3441}, {39, 355, 6, 3441}, {40, 356, 3, 3441}, {41, 359, 1, 3441}, {42, 360, 3, 3441}, {43, 361, 3, 3441}, {44, 362, 1, 3441}, {35, 348, 1, 3442}, {36, 352, 3, 3442}, {37, 353, 3, 3442}, {38, 357, 3, 3442}, {39, 358, 6, 3442}, {40, 359, 3, 3442}, {41, 363, 1, 3442}, {42, 364, 3, 3442}, {43, 365, 3, 3442}, {44, 366, 1, 3442}, {35, 349, 1, 3443}, {36, 353, 3, 3443}, {37, 354, 3, 3443}, {38, 358, 3, 3443}, {39, 359, 6, 3443}, {40, 360, 3, 3443}, {41, 364, 1, 3443}, {42, 365, 3, 3443}, {43, 366, 3, 3443}, {44, 367, 1, 3443}, {35, 350, 1, 3444}, {36, 354, 3, 3444}, {37, 355, 3, 3444}, {38, 359, 3, 3444}, {39, 360, 6, 3444}, {40, 361, 3, 3444}, {41, 365, 1, 3444}, {42, 366, 3, 3444}, {43, 367, 3, 3444}, {44, 368, 1, 3444}, {35, 351, 1, 3445}, {36, 355, 3, 3445}, {37, 356, 3, 3445}, {38, 360, 3, 3445}, {39, 361, 6, 3445}, {40, 362, 3, 3445}, {41, 366, 1, 3445}, {42, 367, 3, 3445}, {43, 368, 3, 3445}, {44, 369, 1, 3445}, {36, 342, 1, 3446}, {38, 343, 3, 3446}, {39, 344, 3, 3446}, {41, 345, 3, 3446}, {42, 346, 6, 3446}, {43, 347, 3, 3446}, {45, 348, 1, 3446}, {46, 349, 3, 3446}, {47, 350, 3, 3446}, {48, 351, 1, 3446}, {36, 343, 1, 3447}, {38, 345, 3, 3447}, {39, 346, 3, 3447}, {41, 348, 3, 3447}, {42, 349, 6, 3447}, {43, 350, 3, 3447}, {45, 352, 1, 3447}, {46, 353, 3, 3447}, {47, 354, 3, 3447}, {48, 355, 1, 3447}, {36, 344, 1, 3448}, {38, 346, 3, 3448}, {39, 347, 3, 3448}, {41, 349, 3, 3448}, {42, 350, 6, 3448}, {43, 351, 3, 3448}, {45, 353, 1, 3448}, {46, 354, 3, 3448}, {47, 355, 3, 3448}, {48, 356, 1, 3448}, {36, 345, 1, 3449}, {38, 348, 3, 3449}, {39, 349, 3, 3449}, {41, 352, 3, 3449}, {42, 353, 6, 3449}, {43, 354, 3, 3449}, {45, 357, 1, 3449}, {46, 358, 3, 3449}, {47, 359, 3, 3449}, {48, 360, 1, 3449}, {36, 346, 1, 3450}, {38, 349, 3, 3450}, {39, 350, 3, 3450}, {41, 353, 3, 3450}, {42, 354, 6, 3450}, {43, 355, 3, 3450}, {45, 358, 1, 3450}, {46, 359, 3, 3450}, {47, 360, 3, 3450}, {48, 361, 1, 3450}, {36, 347, 1, 3451}, {38, 350, 3, 3451}, {39, 351, 3, 3451}, {41, 354, 3, 3451}, {42, 355, 6, 3451}, {43, 356, 3, 3451}, {45, 359, 1, 3451}, {46, 360, 3, 3451}, {47, 361, 3, 3451}, {48, 362, 1, 3451}, {36, 348, 1, 3452}, {38, 352, 3, 3452}, {39, 353, 3, 3452}, {41, 357, 3, 3452}, {42, 358, 6, 3452}, {43, 359, 3, 3452}, {45, 363, 1, 3452}, {46, 364, 3, 3452}, {47, 365, 3, 3452}, {48, 366, 1, 3452}, {36, 349, 1, 3453}, {38, 353, 3, 3453}, {39, 354, 3, 3453}, {41, 358, 3, 3453}, {42, 359, 6, 3453}, {43, 360, 3, 3453}, {45, 364, 1, 3453}, {46, 365, 3, 3453}, {47, 366, 3, 3453}, {48, 367, 1, 3453}, {36, 350, 1, 3454}, {38, 354, 3, 3454}, {39, 355, 3, 3454}, {41, 359, 3, 3454}, {42, 360, 6, 3454}, {43, 361, 3, 3454}, {45, 365, 1, 3454}, {46, 366, 3, 3454}, {47, 367, 3, 3454}, {48, 368, 1, 3454}, {36, 351, 1, 3455}, {38, 355, 3, 3455}, {39, 356, 3, 3455}, {41, 360, 3, 3455}, {42, 361, 6, 3455}, {43, 362, 3, 3455}, {45, 366, 1, 3455}, {46, 367, 3, 3455}, {47, 368, 3, 3455}, {48, 369, 1, 3455}, {37, 342, 1, 3456}, {39, 343, 3, 3456}, {40, 344, 3, 3456}, {42, 345, 3, 3456}, {43, 346, 6, 3456}, {44, 347, 3, 3456}, {46, 348, 1, 3456}, {47, 349, 3, 3456}, {48, 350, 3, 3456}, {49, 351, 1, 3456}, {37, 343, 1, 3457}, {39, 345, 3, 3457}, {40, 346, 3, 3457}, {42, 348, 3, 3457}, {43, 349, 6, 3457}, {44, 350, 3, 3457}, {46, 352, 1, 3457}, {47, 353, 3, 3457}, {48, 354, 3, 3457}, {49, 355, 1, 3457}, {37, 344, 1, 3458}, {39, 346, 3, 3458}, {40, 347, 3, 3458}, {42, 349, 3, 3458}, {43, 350, 6, 3458}, {44, 351, 3, 3458}, {46, 353, 1, 3458}, {47, 354, 3, 3458}, {48, 355, 3, 3458}, {49, 356, 1, 3458}, {37, 345, 1, 3459}, {39, 348, 3, 3459}, {40, 349, 3, 3459}, {42, 352, 3, 3459}, {43, 353, 6, 3459}, {44, 354, 3, 3459}, {46, 357, 1, 3459}, {47, 358, 3, 3459}, {48, 359, 3, 3459}, {49, 360, 1, 3459}, {37, 346, 1, 3460}, {39, 349, 3, 3460}, {40, 350, 3, 3460}, {42, 353, 3, 3460}, {43, 354, 6, 3460}, {44, 355, 3, 3460}, {46, 358, 1, 3460}, {47, 359, 3, 3460}, {48, 360, 3, 3460}, {49, 361, 1, 3460}, {37, 347, 1, 3461}, {39, 350, 3, 3461}, {40, 351, 3, 3461}, {42, 354, 3, 3461}, {43, 355, 6, 3461}, {44, 356, 3, 3461}, {46, 359, 1, 3461}, {47, 360, 3, 3461}, {48, 361, 3, 3461}, {49, 362, 1, 3461}, {37, 348, 1, 3462}, {39, 352, 3, 3462}, {40, 353, 3, 3462}, {42, 357, 3, 3462}, {43, 358, 6, 3462}, {44, 359, 3, 3462}, {46, 363, 1, 3462}, {47, 364, 3, 3462}, {48, 365, 3, 3462}, {49, 366, 1, 3462}, {37, 349, 1, 3463}, {39, 353, 3, 3463}, {40, 354, 3, 3463}, {42, 358, 3, 3463}, {43, 359, 6, 3463}, {44, 360, 3, 3463}, {46, 364, 1, 3463}, {47, 365, 3, 3463}, {48, 366, 3, 3463}, {49, 367, 1, 3463}, {37, 350, 1, 3464}, {39, 354, 3, 3464}, {40, 355, 3, 3464}, {42, 359, 3, 3464}, {43, 360, 6, 3464}, {44, 361, 3, 3464}, {46, 365, 1, 3464}, {47, 366, 3, 3464}, {48, 367, 3, 3464}, {49, 368, 1, 3464}, {37, 351, 1, 3465}, {39, 355, 3, 3465}, {40, 356, 3, 3465}, {42, 360, 3, 3465}, {43, 361, 6, 3465}, {44, 362, 3, 3465}, {46, 366, 1, 3465}, {47, 367, 3, 3465}, {48, 368, 3, 3465}, {49, 369, 1, 3465}, {38, 342, 1, 3466}, {41, 343, 3, 3466}, {42, 344, 3, 3466}, {45, 345, 3, 3466}, {46, 346, 6, 3466}, {47, 347, 3, 3466}, {50, 348, 1, 3466}, {51, 349, 3, 3466}, {52, 350, 3, 3466}, {53, 351, 1, 3466}, {38, 343, 1, 3467}, {41, 345, 3, 3467}, {42, 346, 3, 3467}, {45, 348, 3, 3467}, {46, 349, 6, 3467}, {47, 350, 3, 3467}, {50, 352, 1, 3467}, {51, 353, 3, 3467}, {52, 354, 3, 3467}, {53, 355, 1, 3467}, {38, 344, 1, 3468}, {41, 346, 3, 3468}, {42, 347, 3, 3468}, {45, 349, 3, 3468}, {46, 350, 6, 3468}, {47, 351, 3, 3468}, {50, 353, 1, 3468}, {51, 354, 3, 3468}, {52, 355, 3, 3468}, {53, 356, 1, 3468}, {38, 345, 1, 3469}, {41, 348, 3, 3469}, {42, 349, 3, 3469}, {45, 352, 3, 3469}, {46, 353, 6, 3469}, {47, 354, 3, 3469}, {50, 357, 1, 3469}, {51, 358, 3, 3469}, {52, 359, 3, 3469}, {53, 360, 1, 3469}, {38, 346, 1, 3470}, {41, 349, 3, 3470}, {42, 350, 3, 3470}, {45, 353, 3, 3470}, {46, 354, 6, 3470}, {47, 355, 3, 3470}, {50, 358, 1, 3470}, {51, 359,

3, 3470}, {52, 360, 3, 3470}, {53, 361, 1, 3470}, {38, 347, 1, 3471}, {41, 350, 3, 3471}, {42, 351, 3, 3471}, {45, 354, 3, 3471}, {46, 355, 6, 3471}, {47, 356, 3, 3471}, {50, 359, 1, 3471}, {51, 360, 3, 3471}, {52, 361, 3, 3471}, {53, 362, 1, 3471}, {38, 348, 1, 3472}, {41, 352, 3, 3472}, {42, 353, 3, 3472}, {45, 357, 3, 3472}, {46, 358, 6, 3472}, {47, 359, 3, 3472}, {50, 363, 1, 3472}, {51, 364, 3, 3472}, {52, 365, 3, 3472}, {53, 366, 1, 3472}, {38, 349, 1, 3473}, {41, 353, 3, 3473}, {42, 354, 3, 3473}, {45, 358, 3, 3473}, {46, 359, 6, 3473}, {47, 360, 3, 3473}, {50, 364, 1, 3473}, {51, 365, 3, 3473}, {52, 366, 3, 3473}, {53, 367, 1, 3473}, {38, 350, 1, 3474}, {41, 354, 3, 3474}, {42, 355, 3, 3474}, {45, 359, 3, 3474}, {46, 360, 6, 3474}, {47, 361, 3, 3474}, {50, 365, 1, 3474}, {51, 366, 3, 3474}, {52, 367, 3, 3474}, {53, 368, 1, 3474}, {38, 351, 1, 3475}, {41, 355, 3, 3475}, {42, 356, 3, 3475}, {45, 360, 3, 3475}, {46, 361, 6, 3475}, {47, 362, 3, 3475}, {50, 366, 1, 3475}, {51, 367, 3, 3475}, {52, 368, 3, 3475}, {53, 369, 1, 3475}, {39, 342, 1, 3476}, {42, 343, 3, 3476}, {43, 344, 3, 3476}, {46, 345, 3, 3476}, {47, 346, 6, 3476}, {48, 347, 3, 3476}, {51, 348, 1, 3476}, {52, 349, 3, 3476}, {53, 350, 3, 3476}, {54, 351, 1, 3476}, {39, 343, 1, 3477}, {42, 345, 3, 3477}, {43, 346, 3, 3477}, {46, 348, 3, 3477}, {47, 349, 6, 3477}, {48, 350, 3, 3477}, {51, 352, 1, 3477}, {52, 353, 3, 3477}, {53, 354, 3, 3477}, {54, 355, 1, 3477}, {39, 344, 1, 3478}, {42, 346, 3, 3478}, {43, 347, 3, 3478}, {46, 349, 3, 3478}, {47, 350, 6, 3478}, {48, 351, 3, 3478}, {51, 353, 1, 3478}, {52, 354, 3, 3478}, {53, 355, 3, 3478}, {54, 356, 1, 3478}, {39, 345, 1, 3479}, {42, 348, 3, 3479}, {43, 349, 3, 3479}, {46, 352, 3, 3479}, {47, 353, 6, 3479}, {48, 354, 3, 3479}, {51, 357, 1, 3479}, {52, 358, 3, 3479}, {53, 359, 3, 3479}, {54, 360, 1, 3479}, {39, 346, 1, 3480}, {42, 349, 3, 3480}, {43, 350, 3, 3480}, {46, 353, 3, 3480}, {47, 354, 6, 3480}, {48, 355, 3, 3480}, {51, 358, 1, 3480}, {52, 359, 3, 3480}, {53, 360, 3, 3480}, {54, 361, 1, 3480}, {39, 347, 1, 3481}, {42, 350, 3, 3481}, {43, 351, 3, 3481}, {46, 354, 3, 3481}, {47, 355, 6, 3481}, {48, 356, 3, 3481}, {51, 359, 1, 3481}, {52, 360, 3, 3481}, {53, 361, 3, 3481}, {54, 362, 1, 3481}, {39, 348, 1, 3482}, {42, 352, 3, 3482}, {43, 353, 3, 3482}, {46, 357, 3, 3482}, {47, 358, 6, 3482}, {48, 359, 3, 3482}, {51, 363, 1, 3482}, {52, 364, 3, 3482}, {53, 365, 3, 3482}, {54, 366, 1, 3482}, {39, 349, 1, 3483}, {42, 353, 3, 3483}, {43, 354, 3, 3483}, {46, 358, 3, 3483}, {47, 359, 6, 3483}, {48, 360, 3, 3483}, {51, 364, 1, 3483}, {52, 365, 3, 3483}, {53, 366, 3, 3483}, {54, 367, 1, 3483}, {39, 350, 1, 3484}, {42, 354, 3, 3484}, {43, 355, 3, 3484}, {46, 359, 3, 3484}, {47, 360, 6, 3484}, {48, 361, 3, 3484}, {51, 365, 1, 3484}, {52, 366, 3, 3484}, {53, 367, 3, 3484}, {54, 368, 1, 3484}, {39, 351, 1, 3485}, {42, 355, 3, 3485}, {43, 356, 3, 3485}, {46, 360, 3, 3485}, {47, 361, 6, 3485}, {48, 362, 3, 3485}, {51, 366, 1, 3485}, {52, 367, 3, 3485}, {53, 368, 3, 3485}, {54, 369, 1, 3485}, {40, 342, 1, 3486}, {43, 343, 3, 3486}, {44, 344, 3, 3486}, {47, 345, 3, 3486}, {48, 346, 6, 3486}, {49, 347, 3, 3486}, {52, 348, 1, 3486}, {53, 349, 3, 3486}, {54, 350, 3, 3486}, {55, 351, 1, 3486}, {40, 343, 1, 3487}, {43, 345, 3, 3487}, {44, 346, 3, 3487}, {47, 348, 3, 3487}, {48, 349, 6, 3487}, {49, 350, 3, 3487}, {52, 352, 1, 3487}, {53, 353, 3, 3487}, {54, 354, 3, 3487}, {55, 355, 1, 3487}, {40, 344, 1, 3488}, {43, 346, 3, 3488}, {44, 347, 3, 3488}, {47, 349, 3, 3488}, {48, 350, 6, 3488}, {49, 351, 3, 3488}, {52, 353, 1, 3488}, {53, 354, 3, 3488}, {54, 355, 3, 3488}, {55, 356, 1, 3488}, {40, 345, 1, 3489}, {43, 348, 3, 3489}, {44, 349, 3, 3489}, {47, 352, 3, 3489}, {48, 353, 6, 3489}, {49, 354, 3, 3489}, {52, 357, 1, 3489}, {53, 358, 3, 3489}, {54, 359, 3, 3489}, {55, 360, 1, 3489}, {40, 346, 1, 3490}, {43, 349, 3, 3490}, {44, 350, 3, 3490}, {47, 353, 3, 3490}, {48, 354, 6, 3490}, {49, 355, 3, 3490}, {52, 358, 1, 3490}, {53, 359, 3, 3490}, {54, 360, 3, 3490}, {55, 361, 1, 3490}, {40, 347, 1, 3491}, {43, 350, 3, 3491}, {44, 351, 3, 3491}, {47, 354, 3, 3491}, {48, 355, 6, 3491}, {49, 356, 3, 3491}, {52, 359, 1, 3491}, {53, 360, 3, 3491}, {54, 361, 3, 3491}, {55, 362, 1, 3491}, {40, 348, 1, 3492}, {43, 352, 3, 3492}, {44, 353, 3, 3492}, {47, 357, 3, 3492}, {48, 358, 6, 3492}, {49, 359, 3, 3492}, {52, 363, 1, 3492}, {53, 364, 3, 3492}, {54, 365, 3, 3492}, {55, 366, 1, 3492}, {40, 349, 1, 3493}, {43, 353, 3, 3493}, {44, 354, 3, 3493}, {47, 358, 3, 3493}, {48, 359, 6, 3493}, {49, 360, 3, 3493}, {52, 364, 1, 3493}, {53, 365, 3, 3493}, {54, 366, 3, 3493}, {55, 367, 1, 3493}, {40, 350, 1, 3494}, {43, 354, 3, 3494}, {44, 355, 3, 3494}, {47, 359, 3, 3494}, {48, 360, 6, 3494}, {49, 361, 3, 3494}, {52, 365, 1, 3494}, {53, 366, 3, 3494}, {54, 367, 3, 3494}, {55, 368, 1, 3494}, {40, 351, 1, 3495}, {43, 355, 3, 3495}, {44, 356, 3, 3495}, {47, 360, 3, 3495}, {48, 361, 6, 3495}, {49, 362, 3, 3495}, {52, 366, 1, 3495}, {53, 367, 3, 3495}, {54, 368, 3, 3495}, {55, 369, 1, 3495}, {35, 306, 1, 3496}, {36, 307, 4, 3496}, {37, 308, 4, 3496}, {38, 309, 6, 3496}, {39, 310, 12, 3496}, {40, 311, 6, 3496}, {41, 312, 4, 3496}, {42, 313, 12, 3496}, {43, 314, 12, 3496}, {44, 315, 4, 3496}, {45, 316, 1, 3496}, {46, 317, 4, 3496}, {47, 318, 6, 3496}, {48, 319, 4, 3496}, {49, 320, 1, 3496}, {36, 306, 1, 3497}, {38, 307, 4, 3497}, {39, 308, 4, 3497}, {41, 309, 6, 3497}, {42, 310, 12, 3497}, {43, 311, 6, 3497}, {45, 312, 4, 3497}, {46, 313, 12, 3497}, {47, 314, 12, 3497}, {48, 315, 4, 3497}, {50, 316, 1, 3497}, {51, 317, 4, 3497}, {52, 318, 6, 3497}, {53, 319, 4, 3497}, {54, 320, 1, 3497}, {37, 306, 1, 3498}, {39, 307, 4, 3498}, {40, 308, 4, 3498}, {42, 309, 6, 3498}, {43, 310, 12, 3498}, {44, 311, 6, 3498}, {46, 312, 4, 3498}, {47, 313, 12, 3498}, {48, 314, 12, 3498}, {49, 315, 4, 3498}, {51, 316, 1, 3498}, {52, 317, 4, 3498}, {53, 318, 6, 3498}, {54, 319, 4, 3498}, {55, 320, 1, 3498}, {35, 321, 1, 3499}, {36, 322, 4, 3499}, {37, 323, 4, 3499}, {38, 324, 6, 3499}, {39, 325, 12, 3499}, {40, 326, 6, 3499}, {41, 327, 4, 3499}, {42, 328, 12, 3499}, {43, 329, 12, 3499}, {44, 330, 4, 3499}, {45, 331, 1, 3499}, {46,

332, 4, 3499}, {47, 333, 6, 3499}, {48, 334, 4, 3499}, {49, 335, 1, 3499}, {35, 322, 1, 3500}, {36, 324, 4, 3500}, {37, 325, 4, 3500}, {38, 327, 6, 3500}, {39, 328, 12, 3500}, {40, 329, 6, 3500}, {41, 331, 4, 3500}, {42, 332, 12, 3500}, {43, 333, 12, 3500}, {44, 334, 4, 3500}, {45, 336, 1, 3500}, {46, 337, 4, 3500}, {47, 338, 6, 3500}, {48, 339, 4, 3500}, {49, 340, 1, 3500}, {35, 323, 1, 3501}, {36, 325, 4, 3501}, {37, 326, 4, 3501}, {38, 328, 6, 3501}, {39, 329, 12, 3501}, {40, 330, 6, 3501}, {41, 332, 4, 3501}, {42, 333, 12, 3501}, {43, 334, 12, 3501}, {44, 335, 4, 3501}, {45, 337, 1, 3501}, {46, 338, 4, 3501}, {47, 339, 6, 3501}, {48, 340, 4, 3501}, {49, 341, 1, 3501}, {36, 321, 1, 3502}, {38, 322, 4, 3502}, {39, 323, 4, 3502}, {41, 324, 6, 3502}, {42, 325, 12, 3502}, {43, 326, 6, 3502}, {45, 327, 4, 3502}, {46, 328, 12, 3502}, {47, 329, 12, 3502}, {48, 330, 4, 3502}, {50, 331, 1, 3502}, {51, 332, 4, 3502}, {52, 333, 6, 3502}, {53, 334, 4, 3502}, {54, 335, 1, 3502}, {36, 322, 1, 3503}, {38, 324, 4, 3503}, {39, 325, 4, 3503}, {41, 327, 6, 3503}, {42, 328, 12, 3503}, {43, 329, 6, 3503}, {45, 331, 4, 3503}, {46, 332, 12, 3503}, {47, 333, 12, 3503}, {48, 334, 4, 3503}, {50, 336, 1, 3503}, {51, 337, 4, 3503}, {52, 338, 6, 3503}, {53, 339, 4, 3503}, {54, 340, 1, 3503}, {36, 323, 1, 3504}, {38, 325, 4, 3504}, {39, 326, 4, 3504}, {41, 328, 6, 3504}, {42, 329, 12, 3504}, {43, 330, 6, 3504}, {45, 332, 4, 3504}, {46, 333, 12, 3504}, {47, 334, 12, 3504}, {48, 335, 4, 3504}, {50, 337, 1, 3504}, {51, 338, 4, 3504}, {52, 339, 6, 3504}, {53, 340, 4, 3504}, {54, 341, 1, 3504}, {37, 321, 1, 3505}, {39, 322, 4, 3505}, {40, 323, 4, 3505}, {42, 324, 6, 3505}, {43, 325, 12, 3505}, {44, 326, 6, 3505}, {46, 327, 4, 3505}, {47, 328, 12, 3505}, {48, 329, 12, 3505}, {49, 330, 4, 3505}, {51, 331, 1, 3505}, {52, 332, 4, 3505}, {53, 333, 6, 3505}, {54, 334, 4, 3505}, {55, 335, 1, 3505}, {37, 322, 1, 3506}, {39, 324, 4, 3506}, {40, 325, 4, 3506}, {42, 327, 6, 3506}, {43, 328, 12, 3506}, {44, 329, 6, 3506}, {46, 331, 4, 3506}, {47, 332, 12, 3506}, {48, 333, 12, 3506}, {49, 334, 4, 3506}, {51, 336, 1, 3506}, {52, 337, 4, 3506}, {53, 338, 6, 3506}, {54, 339, 4, 3506}, {55, 340, 1, 3506}, {37, 323, 1, 3507}, {39, 325, 4, 3507}, {40, 326, 4, 3507}, {42, 328, 6, 3507}, {43, 329, 12, 3507}, {44, 330, 6, 3507}, {46, 332, 4, 3507}, {47, 333, 12, 3507}, {48, 334, 12, 3507}, {49, 335, 4, 3507}, {51, 337, 1, 3507}, {52, 338, 4, 3507}, {53, 339, 6, 3507}, {54, 340, 4, 3507}, {55, 341, 1, 3507}, {35, 342, 1, 3508}, {36, 343, 4, 3508}, {37, 344, 4, 3508}, {38, 345, 6, 3508}, {39, 346, 12, 3508}, {40, 347, 6, 3508}, {41, 348, 4, 3508}, {42, 349, 12, 3508}, {43, 350, 12, 3508}, {44, 351, 4, 3508}, {45, 352, 1, 3508}, {46, 353, 4, 3508}, {47, 354, 6, 3508}, {48, 355, 4, 3508}, {49, 356, 1, 3508}, {35, 343, 1, 3509}, {36, 345, 4, 3509}, {37, 346, 4, 3509}, {38, 348, 6, 3509}, {39, 349, 12, 3509}, {40, 350, 6, 3509}, {41, 352, 4, 3509}, {42, 353, 12, 3509}, {43, 354, 12, 3509}, {44, 355, 4, 3509}, {45, 357, 1, 3509}, {46, 358, 4, 3509}, {47, 359, 6, 3509}, {48, 360, 4, 3509}, {49, 361, 1, 3509}, {35, 344, 1, 3510}, {36, 346, 4, 3510}, {37, 347, 4, 3510}, {38, 349, 6, 3510}, {39, 350, 12, 3510}, {40, 351, 6, 3510}, {41, 353, 4, 3510}, {42, 354, 12, 3510}, {43, 355, 12, 3510}, {44, 356, 4, 3510}, {45, 358, 1, 3510}, {46, 359, 4, 3510}, {47, 360, 6, 3510}, {48, 361, 4, 3510}, {49, 362, 1, 3510}, {35, 345, 1, 3511}, {36, 348, 4, 3511}, {37, 349, 4, 3511}, {38, 352, 6, 3511}, {39, 353, 12, 3511}, {40, 354, 6, 3511}, {41, 357, 4, 3511}, {42, 358, 12, 3511}, {43, 359, 12, 3511}, {44, 360, 4, 3511}, {45, 363, 1, 3511}, {46, 364, 4, 3511}, {47, 365, 6, 3511}, {48, 366, 4, 3511}, {49, 367, 1, 3511}, {35, 346, 1, 3512}, {36, 349, 4, 3512}, {37, 350, 4, 3512}, {38, 353, 6, 3512}, {39, 354, 12, 3512}, {40, 355, 6, 3512}, {41, 358, 4, 3512}, {42, 359, 12, 3512}, {43, 360, 12, 3512}, {44, 361, 4, 3512}, {45, 364, 1, 3512}, {46, 365, 4, 3512}, {47, 366, 6, 3512}, {48, 367, 4, 3512}, {49, 368, 1, 3512}, {35, 347, 1, 3513}, {36, 350, 4, 3513}, {37, 351, 4, 3513}, {38, 354, 6, 3513}, {39, 355, 12, 3513}, {40, 356, 6, 3513}, {41, 359, 4, 3513}, {42, 360, 12, 3513}, {43, 361, 12, 3513}, {44, 362, 4, 3513}, {45, 365, 1, 3513}, {46, 366, 4, 3513}, {47, 367, 6, 3513}, {48, 368, 4, 3513}, {49, 369, 1, 3513}, {36, 342, 1, 3514}, {38, 343, 4, 3514}, {39, 344, 4, 3514}, {41, 345, 6, 3514}, {42, 346, 12, 3514}, {43, 347, 6, 3514}, {45, 348, 4, 3514}, {46, 349, 12, 3514}, {47, 350, 12, 3514}, {48, 351, 4, 3514}, {50, 352, 1, 3514}, {51, 353, 4, 3514}, {52, 354, 6, 3514}, {53, 355, 4, 3514}, {54, 356, 1, 3514}, {36, 343, 1, 3515}, {38, 345, 4, 3515}, {39, 346, 4, 3515}, {41, 348, 6, 3515}, {42, 349, 12, 3515}, {43, 350, 6, 3515}, {45, 352, 4, 3515}, {46, 353, 12, 3515}, {47, 354, 12, 3515}, {48, 355, 4, 3515}, {50, 357, 1, 3515}, {51, 358, 4, 3515}, {52, 359, 6, 3515}, {53, 360, 4, 3515}, {54, 361, 1, 3515}, {36, 344, 1, 3516}, {38, 346, 4, 3516}, {39, 347, 4, 3516}, {41, 349, 6, 3516}, {42, 350, 12, 3516}, {43, 351, 6, 3516}, {45, 353, 4, 3516}, {46, 354, 12, 3516}, {47, 355, 12, 3516}, {48, 356, 4, 3516}, {50, 358, 1, 3516}, {51, 359, 4, 3516}, {52, 360, 6, 3516}, {53, 361, 4, 3516}, {54, 362, 1, 3516}, {36, 345, 1, 3517}, {38, 348, 4, 3517}, {39, 349, 4, 3517}, {41, 352, 6, 3517}, {42, 353, 12, 3517}, {43, 354, 6, 3517}, {45, 357, 4, 3517}, {46, 358, 12, 3517}, {47, 359, 12, 3517}, {48, 360, 4, 3517}, {50, 363, 1, 3517}, {51, 364, 4, 3517}, {52, 365, 6, 3517}, {53, 366, 4, 3517}, {54, 367, 1, 3517}, {36, 346, 1, 3518}, {38, 349, 4, 3518}, {39, 350, 4, 3518}, {41, 353, 6, 3518}, {42, 354, 12, 3518}, {43, 355, 6, 3518}, {45, 358, 4, 3518}, {46, 359, 12, 3518}, {47, 360, 12, 3518}, {48, 361, 4, 3518}, {50, 364, 1, 3518}, {51, 365, 4, 3518}, {52, 366, 6, 3518}, {53, 367, 4, 3518}, {54, 368, 1, 3518}, {36, 347, 1, 3519}, {38, 350, 4, 3519}, {39, 351, 4, 3519}, {41, 354, 6, 3519}, {42, 355, 12, 3519}, {43, 356, 6, 3519}, {45, 359, 4, 3519}, {46, 360, 12, 3519}, {47, 361, 12, 3519}, {48, 362, 4, 3519}, {50, 365, 1, 3519}, {51, 366, 4,

3519}, {52, 367, 6, 3519}, {53, 368, 4, 3519}, {54, 369, 1, 3519}, {37, 342, 1, 3520}, {39, 343, 4, 3520}, {40, 344, 4, 3520}, {42, 345, 6, 3520}, {43, 346, 12, 3520}, {44, 347, 6, 3520}, {46, 348, 4, 3520}, {47, 349, 12, 3520}, {48, 350, 12, 3520}, {49, 351, 4, 3520}, {51, 352, 1, 3520}, {52, 353, 4, 3520}, {53, 354, 6, 3520}, {54, 355, 4, 3520}, {55, 356, 1, 3520}, {37, 343, 1, 3521}, {39, 345, 4, 3521}, {40, 346, 4, 3521}, {42, 348, 6, 3521}, {43, 349, 12, 3521}, {44, 350, 6, 3521}, {46, 352, 4, 3521}, {47, 353, 12, 3521}, {48, 354, 12, 3521}, {49, 355, 4, 3521}, {51, 357, 1, 3521}, {52, 358, 4, 3521}, {53, 359, 6, 3521}, {54, 360, 4, 3521}, {55, 361, 1, 3521}, {37, 344, 1, 3522}, {39, 346, 4, 3522}, {40, 347, 4, 3522}, {42, 349, 6, 3522}, {43, 350, 12, 3522}, {44, 351, 6, 3522}, {46, 353, 4, 3522}, {47, 354, 12, 3522}, {48, 355, 12, 3522}, {49, 356, 4, 3522}, {51, 358, 1, 3522}, {52, 359, 4, 3522}, {53, 360, 6, 3522}, {54, 361, 4, 3522}, {55, 362, 1, 3522}, {37, 345, 1, 3523}, {39, 348, 4, 3523}, {40, 349, 4, 3523}, {42, 352, 6, 3523}, {43, 353, 12, 3523}, {44, 354, 6, 3523}, {46, 357, 4, 3523}, {47, 358, 12, 3523}, {48, 359, 12, 3523}, {49, 360, 4, 3523}, {51, 363, 1, 3523}, {52, 364, 4, 3523}, {53, 365, 6, 3523}, {54, 366, 4, 3523}, {55, 367, 1, 3523}, {37, 346, 1, 3524}, {39, 349, 4, 3524}, {40, 350, 4, 3524}, {42, 353, 6, 3524}, {43, 354, 12, 3524}, {44, 355, 6, 3524}, {46, 358, 4, 3524}, {47, 359, 12, 3524}, {48, 360, 12, 3524}, {49, 361, 4, 3524}, {51, 364, 1, 3524}, {52, 365, 4, 3524}, {53, 366, 6, 3524}, {54, 367, 4, 3524}, {55, 368, 1, 3524}, {37, 347, 1, 3525}, {39, 350, 4, 3525}, {40, 351, 4, 3525}, {42, 354, 6, 3525}, {43, 355, 12, 3525}, {44, 356, 6, 3525}, {46, 359, 4, 3525}, {47, 360, 12, 3525}, {48, 361, 12, 3525}, {49, 362, 4, 3525}, {51, 365, 1, 3525}, {52, 366, 4, 3525}, {53, 367, 6, 3525}, {54, 368, 4, 3525}, {55, 369, 1, 3525}, {35, 370, 1, 3526}, {36, 371, 4, 3526}, {37, 372, 4, 3526}, {38, 373, 6, 3526}, {39, 374, 12, 3526}, {40, 375, 6, 3526}, {41, 376, 4, 3526}, {42, 377, 12, 3526}, {43, 378, 12, 3526}, {44, 379, 4, 3526}, {45, 380, 1, 3526}, {46, 381, 4, 3526}, {47, 382, 6, 3526}, {48, 383, 4, 3526}, {49, 384, 1, 3526}, {35, 371, 1, 3527}, {36, 373, 4, 3527}, {37, 374, 4, 3527}, {38, 376, 6, 3527}, {39, 377, 12, 3527}, {40, 378, 6, 3527}, {41, 380, 4, 3527}, {42, 381, 12, 3527}, {43, 382, 12, 3527}, {44, 383, 4, 3527}, {45, 385, 1, 3527}, {46, 386, 4, 3527}, {47, 387, 6, 3527}, {48, 388, 4, 3527}, {49, 389, 1, 3527}, {35, 372, 1, 3528}, {36, 374, 4, 3528}, {37, 375, 4, 3528}, {38, 377, 6, 3528}, {39, 378, 12, 3528}, {40, 379, 6, 3528}, {41, 381, 4, 3528}, {42, 382, 12, 3528}, {43, 383, 12, 3528}, {44, 384, 4, 3528}, {45, 386, 1, 3528}, {46, 387, 4, 3528}, {47, 388, 6, 3528}, {48, 389, 4, 3528}, {49, 390, 1, 3528}, {35, 373, 1, 3529}, {36, 376, 4, 3529}, {37, 377, 4, 3529}, {38, 380, 6, 3529}, {39, 381, 12, 3529}, {40, 382, 6, 3529}, {41, 385, 4, 3529}, {42, 386, 12, 3529}, {43, 387, 12, 3529}, {44, 388, 4, 3529}, {45, 391, 1, 3529}, {46, 392, 4, 3529}, {47, 393, 6, 3529}, {48, 394, 4, 3529}, {49, 395, 1, 3529}, {35, 374, 1, 3530}, {36, 377, 4, 3530}, {37, 378, 4, 3530}, {38, 381, 6, 3530}, {39, 382, 12, 3530}, {40, 383, 6, 3530}, {41, 386, 4, 3530}, {42, 387, 12, 3530}, {43, 388, 12, 3530}, {44, 389, 4, 3530}, {45, 392, 1, 3530}, {46, 393, 4, 3530}, {47, 394, 6, 3530}, {48, 395, 4, 3530}, {49, 396, 1, 3530}, {35, 375, 1, 3531}, {36, 378, 4, 3531}, {37, 379, 4, 3531}, {38, 382, 6, 3531}, {39, 383, 12, 3531}, {40, 384, 6, 3531}, {41, 387, 4, 3531}, {42, 388, 12, 3531}, {43, 389, 12, 3531}, {44, 390, 4, 3531}, {45, 393, 1, 3531}, {46, 394, 4, 3531}, {47, 395, 6, 3531}, {48, 396, 4, 3531}, {49, 397, 1, 3531}, {35, 376, 1, 3532}, {36, 380, 4, 3532}, {37, 381, 4, 3532}, {38, 385, 6, 3532}, {39, 386, 12, 3532}, {40, 387, 6, 3532}, {41, 391, 4, 3532}, {42, 392, 12, 3532}, {43, 393, 12, 3532}, {44, 394, 4, 3532}, {45, 398, 1, 3532}, {46, 399, 4, 3532}, {47, 400, 6, 3532}, {48, 401, 4, 3532}, {49, 402, 1, 3532}, {35, 377, 1, 3533}, {36, 381, 4, 3533}, {37, 382, 4, 3533}, {38, 386, 6, 3533}, {39, 387, 12, 3533}, {40, 388, 6, 3533}, {41, 392, 4, 3533}, {42, 393, 12, 3533}, {43, 394, 12, 3533}, {44, 395, 4, 3533}, {45, 399, 1, 3533}, {46, 400, 4, 3533}, {47, 401, 6, 3533}, {48, 402, 4, 3533}, {49, 403, 1, 3533}, {35, 378, 1, 3534}, {36, 382, 4, 3534}, {37, 383, 4, 3534}, {38, 387, 6, 3534}, {39, 388, 12, 3534}, {40, 389, 6, 3534}, {41, 393, 4, 3534}, {42, 394, 12, 3534}, {43, 395, 12, 3534}, {44, 396, 4, 3534}, {45, 400, 1, 3534}, {46, 401, 4, 3534}, {47, 402, 6, 3534}, {48, 403, 4, 3534}, {49, 404, 1, 3534}, {35, 379, 1, 3535}, {36, 383, 4, 3535}, {37, 384, 4, 3535}, {38, 388, 6, 3535}, {39, 389, 12, 3535}, {40, 390, 6, 3535}, {41, 394, 4, 3535}, {42, 395, 12, 3535}, {43, 396, 12, 3535}, {44, 397, 4, 3535}, {45, 401, 1, 3535}, {46, 402, 4, 3535}, {47, 403, 6, 3535}, {48, 404, 4, 3535}, {49, 405, 1, 3535}, {36, 370, 1, 3536}, {38, 371, 4, 3536}, {39, 372, 4, 3536}, {41, 373, 6, 3536}, {42, 374, 12, 3536}, {43, 375, 6, 3536}, {45, 376, 4, 3536}, {46, 377, 12, 3536}, {47, 378, 12, 3536}, {48, 379, 4, 3536}, {50, 380, 1, 3536}, {51, 381, 4, 3536}, {52, 382, 6, 3536}, {53, 383, 4, 3536}, {54, 384, 1, 3536}, {36, 371, 1, 3537}, {38, 373, 4, 3537}, {39, 374, 4, 3537}, {41, 376, 6, 3537}, {42, 377, 12, 3537}, {43, 378, 6, 3537}, {45, 380, 4, 3537}, {46, 381, 12, 3537}, {47, 382, 12, 3537}, {48, 383, 4, 3537}, {50, 385, 1, 3537}, {51, 386, 4, 3537}, {52, 387, 6, 3537}, {53, 388, 4, 3537}, {54, 389, 1, 3537}, {36, 372, 1, 3538}, {38, 374, 4, 3538}, {39, 375, 4, 3538}, {41, 377, 6, 3538}, {42, 378, 12, 3538}, {43, 379, 6, 3538}, {45, 381, 4, 3538}, {46, 382, 12, 3538}, {47, 383, 12, 3538}, {48, 384, 4, 3538}, {50, 386, 1, 3538}, {51, 387, 4, 3538}, {52, 388, 6, 3538}, {53, 389, 4, 3538}, {54, 390, 1, 3538}, {36, 373, 1, 3539}, {38, 376, 4, 3539}, {39, 377, 4, 3539}, {41, 380, 6, 3539}, {42, 381, 12, 3539}, {43, 382, 6, 3539}, {45, 385, 4, 3539}, {46, 386, 12, 3539}, {47, 387, 12, 3539}, {48, 388, 4, 3539}, {50, 391, 1, 3539}, {51, 392, 4, 3539},

{52, 393, 6, 3539}, {53, 394, 4, 3539}, {54, 395, 1, 3539}, {36, 374, 1, 3540}, {38, 377, 4, 3540}, {39, 378, 4, 3540}, {41, 381, 6, 3540}, {42, 382, 12, 3540}, {43, 383, 6, 3540}, {45, 386, 4, 3540}, {46, 387, 12, 3540}, {47, 388, 12, 3540}, {48, 389, 4, 3540}, {50, 392, 1, 3540}, {51, 393, 4, 3540}, {52, 394, 6, 3540}, {53, 395, 4, 3540}, {54, 396, 1, 3540}, {36, 375, 1, 3541}, {38, 378, 4, 3541}, {39, 379, 4, 3541}, {41, 382, 6, 3541}, {42, 383, 12, 3541}, {43, 384, 6, 3541}, {45, 387, 4, 3541}, {46, 388, 12, 3541}, {47, 389, 12, 3541}, {48, 390, 4, 3541}, {50, 393, 1, 3541}, {51, 394, 4, 3541}, {52, 395, 6, 3541}, {53, 396, 4, 3541}, {54, 397, 1, 3541}, {36, 376, 1, 3542}, {38, 380, 4, 3542}, {39, 381, 4, 3542}, {41, 385, 6, 3542}, {42, 386, 12, 3542}, {43, 387, 6, 3542}, {45, 391, 4, 3542}, {46, 392, 12, 3542}, {47, 393, 12, 3542}, {48, 394, 4, 3542}, {50, 398, 1, 3542}, {51, 399, 4, 3542}, {52, 400, 6, 3542}, {53, 401, 4, 3542}, {54, 402, 1, 3542}, {36, 377, 1, 3543}, {38, 381, 4, 3543}, {39, 382, 4, 3543}, {41, 386, 6, 3543}, {42, 387, 12, 3543}, {43, 388, 6, 3543}, {45, 392, 4, 3543}, {46, 393, 12, 3543}, {47, 394, 12, 3543}, {48, 395, 4, 3543}, {50, 399, 1, 3543}, {51, 400, 4, 3543}, {52, 401, 6, 3543}, {53, 402, 4, 3543}, {54, 403, 1, 3543}, {36, 378, 1, 3544}, {38, 382, 4, 3544}, {39, 383, 4, 3544}, {41, 387, 6, 3544}, {42, 388, 12, 3544}, {43, 389, 6, 3544}, {45, 393, 4, 3544}, {46, 394, 12, 3544}, {47, 395, 12, 3544}, {48, 396, 4, 3544}, {50, 400, 1, 3544}, {51, 401, 4, 3544}, {52, 402, 6, 3544}, {53, 403, 4, 3544}, {54, 404, 1, 3544}, {36, 379, 1, 3545}, {38, 383, 4, 3545}, {39, 384, 4, 3545}, {41, 388, 6, 3545}, {42, 389, 12, 3545}, {43, 390, 6, 3545}, {45, 394, 4, 3545}, {46, 395, 12, 3545}, {47, 396, 12, 3545}, {48, 397, 4, 3545}, {50, 401, 1, 3545}, {51, 402, 4, 3545}, {52, 403, 6, 3545}, {53, 404, 4, 3545}, {54, 405, 1, 3545}, {37, 370, 1, 3546}, {39, 371, 4, 3546}, {40, 372, 4, 3546}, {42, 373, 6, 3546}, {43, 374, 12, 3546}, {44, 375, 6, 3546}, {46, 376, 4, 3546}, {47, 377, 12, 3546}, {48, 378, 12, 3546}, {49, 379, 4, 3546}, {51, 380, 1, 3546}, {52, 381, 4, 3546}, {53, 382, 6, 3546}, {54, 383, 4, 3546}, {55, 384, 1, 3546}, {37, 371, 1, 3547}, {39, 373, 4, 3547}, {40, 374, 4, 3547}, {42, 376, 6, 3547}, {43, 377, 12, 3547}, {44, 378, 6, 3547}, {46, 380, 4, 3547}, {47, 381, 12, 3547}, {48, 382, 12, 3547}, {49, 383, 4, 3547}, {51, 385, 1, 3547}, {52, 386, 4, 3547}, {53, 387, 6, 3547}, {54, 388, 4, 3547}, {55, 389, 1, 3547}, {37, 372, 1, 3548}, {39, 374, 4, 3548}, {40, 375, 4, 3548}, {42, 377, 6, 3548}, {43, 378, 12, 3548}, {44, 379, 6, 3548}, {46, 381, 4, 3548}, {47, 382, 12, 3548}, {48, 383, 12, 3548}, {49, 384, 4, 3548}, {51, 386, 1, 3548}, {52, 387, 4, 3548}, {53, 388, 6, 3548}, {54, 389, 4, 3548}, {55, 390, 1, 3548}, {37, 373, 1, 3549}, {39, 376, 4, 3549}, {40, 377, 4, 3549}, {42, 380, 6, 3549}, {43, 381, 12, 3549}, {44, 382, 6, 3549}, {46, 385, 4, 3549}, {47, 386, 12, 3549}, {48, 387, 12, 3549}, {49, 388, 4, 3549}, {51, 391, 1, 3549}, {52, 392, 4, 3549}, {53, 393, 6, 3549}, {54, 394, 4, 3549}, {55, 395, 1, 3549}, {37, 374, 1, 3550}, {39, 377, 4, 3550}, {40, 378, 4, 3550}, {42, 381, 6, 3550}, {43, 382, 12, 3550}, {44, 383, 6, 3550}, {46, 386, 4, 3550}, {47, 387, 12, 3550}, {48, 388, 12, 3550}, {49, 389, 4, 3550}, {51, 392, 1, 3550}, {52, 393, 4, 3550}, {53, 394, 6, 3550}, {54, 395, 4, 3550}, {55, 396, 1, 3550}, {37, 375, 1, 3551}, {39, 378, 4, 3551}, {40, 379, 4, 3551}, {42, 382, 6, 3551}, {43, 383, 12, 3551}, {44, 384, 6, 3551}, {46, 387, 4, 3551}, {47, 388, 12, 3551}, {48, 389, 12, 3551}, {49, 390, 4, 3551}, {51, 393, 1, 3551}, {52, 394, 4, 3551}, {53, 395, 6, 3551}, {54, 396, 4, 3551}, {55, 397, 1, 3551}, {37, 376, 1, 3552}, {39, 380, 4, 3552}, {40, 381, 4, 3552}, {42, 385, 6, 3552}, {43, 386, 12, 3552}, {44, 387, 6, 3552}, {46, 391, 4, 3552}, {47, 392, 12, 3552}, {48, 393, 12, 3552}, {49, 394, 4, 3552}, {51, 398, 1, 3552}, {52, 399, 4, 3552}, {53, 400, 6, 3552}, {54, 401, 4, 3552}, {55, 402, 1, 3552}, {37, 377, 1, 3553}, {39, 381, 4, 3553}, {40, 382, 4, 3553}, {42, 386, 6, 3553}, {43, 387, 12, 3553}, {44, 388, 6, 3553}, {46, 392, 4, 3553}, {47, 393, 12, 3553}, {48, 394, 12, 3553}, {49, 395, 4, 3553}, {51, 399, 1, 3553}, {52, 400, 4, 3553}, {53, 401, 6, 3553}, {54, 402, 4, 3553}, {55, 403, 1, 3553}, {37, 378, 1, 3554}, {39, 382, 4, 3554}, {40, 383, 4, 3554}, {42, 387, 6, 3554}, {43, 388, 12, 3554}, {44, 389, 6, 3554}, {46, 393, 4, 3554}, {47, 394, 12, 3554}, {48, 395, 12, 3554}, {49, 396, 4, 3554}, {51, 400, 1, 3554}, {52, 401, 4, 3554}, {53, 402, 6, 3554}, {54, 403, 4, 3554}, {55, 404, 1, 3554}, {37, 379, 1, 3555}, {39, 383, 4, 3555}, {40, 384, 4, 3555}, {42, 388, 6, 3555}, {43, 389, 12, 3555}, {44, 390, 6, 3555}, {46, 394, 4, 3555}, {47, 395, 12, 3555}, {48, 396, 12, 3555}, {49, 397, 4, 3555}, {51, 401, 1, 3555}, {52, 402, 4, 3555}, {53, 403, 6, 3555}, {54, 404, 4, 3555}, {55, 405, 1, 3555}, {35, 321, 1, 3556}, {36, 322, 5, 3556}, {37, 323, 5, 3556}, {38, 324, 10, 3556}, {39, 325, 20, 3556}, {40, 326, 10, 3556}, {41, 327, 10, 3556}, {42, 328, 30, 3556}, {43, 329, 30, 3556}, {44, 330, 10, 3556}, {45, 331, 5, 3556}, {46, 332, 20, 3556}, {47, 333, 30, 3556}, {48, 334, 20, 3556}, {49, 335, 5, 3556}, {50, 336, 1, 3556}, {51, 337, 5, 3556}, {52, 338, 10, 3556}, {53, 339, 10, 3556}, {54, 340, 5, 3556}, {55, 341, 1, 3556}, {35, 342, 1, 3557}, {36, 343, 5, 3557}, {37, 344, 5, 3557}, {38, 345, 10, 3557}, {39, 346, 20, 3557}, {40, 347, 10, 3557}, {41, 348, 10, 3557}, {42, 349, 30, 3557}, {43, 350, 30, 3557}, {44, 351, 10, 3557}, {45, 352, 5, 3557}, {46, 353, 20, 3557}, {47, 354, 30, 3557}, {48, 355, 20, 3557}, {49, 356, 5, 3557}, {50, 357, 1, 3557}, {51, 358, 5, 3557}, {52, 359, 10, 3557}, {53, 360, 10, 3557}, {54, 361, 5, 3557}, {55, 362, 1, 3557}, {35, 343, 1, 3558}, {36, 345, 5, 3558}, {37, 346, 5, 3558}, {38, 348, 10, 3558}, {39, 349, 20, 3558}, {40, 350, 10, 3558}, {41, 352, 10, 3558}, {42, 353, 30, 3558}, {43, 354, 30, 3558}, {44, 355, 10, 3558}, {45, 357, 5, 3558}, {46, 358, 20, 3558}, {47, 359, 30, 3558}, {48, 360, 20, 3558}, {49, 361, 5,

3558}, {50, 363, 1, 3558}, {51, 364, 5, 3558}, {52, 365, 10, 3558}, {53, 366, 10, 3558}, {54, 367, 5, 3558}, {55, 368, 1, 3558}, {35, 344, 1, 3559}, {36, 346, 5, 3559}, {37, 347, 5, 3559}, {38, 349, 10, 3559}, {39, 350, 20, 3559}, {40, 351, 10, 3559}, {41, 353, 10, 3559}, {42, 354, 30, 3559}, {43, 355, 30, 3559}, {44, 356, 10, 3559}, {45, 358, 5, 3559}, {46, 359, 20, 3559}, {47, 360, 30, 3559}, {48, 361, 20, 3559}, {49, 362, 5, 3559}, {50, 364, 1, 3559}, {51, 365, 5, 3559}, {52, 366, 10, 3559}, {53, 367, 10, 3559}, {54, 368, 5, 3559}, {55, 369, 1, 3559}, {35, 370, 1, 3560}, {36, 371, 5, 3560}, {37, 372, 5, 3560}, {38, 373, 10, 3560}, {39, 374, 20, 3560}, {40, 375, 10, 3560}, {41, 376, 10, 3560}, {42, 377, 30, 3560}, {43, 378, 30, 3560}, {44, 379, 10, 3560}, {45, 380, 5, 3560}, {46, 381, 20, 3560}, {47, 382, 30, 3560}, {48, 383, 20, 3560}, {49, 384, 5, 3560}, {50, 385, 1, 3560}, {51, 386, 5, 3560}, {52, 387, 10, 3560}, {53, 388, 10, 3560}, {54, 389, 5, 3560}, {55, 390, 1, 3560}, {35, 371, 1, 3561}, {36, 373, 5, 3561}, {37, 374, 5, 3561}, {38, 376, 10, 3561}, {39, 377, 20, 3561}, {40, 378, 10, 3561}, {41, 380, 10, 3561}, {42, 381, 30, 3561}, {43, 382, 30, 3561}, {44, 383, 10, 3561}, {45, 385, 5, 3561}, {46, 386, 20, 3561}, {47, 387, 30, 3561}, {48, 388, 20, 3561}, {49, 389, 5, 3561}, {50, 391, 1, 3561}, {51, 392, 5, 3561}, {52, 393, 10, 3561}, {53, 394, 10, 3561}, {54, 395, 5, 3561}, {55, 396, 1, 3561}, {35, 372, 1, 3562}, {36, 374, 5, 3562}, {37, 375, 5, 3562}, {38, 377, 10, 3562}, {39, 378, 20, 3562}, {40, 379, 10, 3562}, {41, 381, 10, 3562}, {42, 382, 30, 3562}, {43, 383, 30, 3562}, {44, 384, 10, 3562}, {45, 386, 5, 3562}, {46, 387, 20, 3562}, {47, 388, 30, 3562}, {48, 389, 20, 3562}, {49, 390, 5, 3562}, {50, 392, 1, 3562}, {51, 393, 5, 3562}, {52, 394, 10, 3562}, {53, 395, 10, 3562}, {54, 396, 5, 3562}, {55, 397, 1, 3562}, {35, 373, 1, 3563}, {36, 376, 5, 3563}, {37, 377, 5, 3563}, {38, 380, 10, 3563}, {39, 381, 20, 3563}, {40, 382, 10, 3563}, {41, 385, 10, 3563}, {42, 386, 30, 3563}, {43, 387, 30, 3563}, {44, 388, 10, 3563}, {45, 391, 5, 3563}, {46, 392, 20, 3563}, {47, 393, 30, 3563}, {48, 394, 20, 3563}, {49, 395, 5, 3563}, {50, 398, 1, 3563}, {51, 399, 5, 3563}, {52, 400, 10, 3563}, {53, 401, 10, 3563}, {54, 402, 5, 3563}, {55, 403, 1, 3563}, {35, 374, 1, 3564}, {36, 377, 5, 3564}, {37, 378, 5, 3564}, {38, 381, 10, 3564}, {39, 382, 20, 3564}, {40, 383, 10, 3564}, {41, 386, 10, 3564}, {42, 387, 30, 3564}, {43, 388, 30, 3564}, {44, 389, 10, 3564}, {45, 392, 5, 3564}, {46, 393, 20, 3564}, {47, 394, 30, 3564}, {48, 395, 20, 3564}, {49, 396, 5, 3564}, {50, 399, 1, 3564}, {51, 400, 5, 3564}, {52, 401, 10, 3564}, {53, 402, 10, 3564}, {54, 403, 5, 3564}, {55, 404, 1, 3564}, {35, 375, 1, 3565}, {36, 378, 5, 3565}, {37, 379, 5, 3565}, {38, 382, 10, 3565}, {39, 383, 20, 3565}, {40, 384, 10, 3565}, {41, 387, 10, 3565}, {42, 388, 30, 3565}, {43, 389, 30, 3565}, {44, 390, 10, 3565}, {45, 393, 5, 3565}, {46, 394, 20, 3565}, {47, 395, 30, 3565}, {48, 396, 20, 3565}, {49, 397, 5, 3565}, {50, 400, 1, 3565}, {51, 401, 5, 3565}, {52, 402, 10, 3565}, {53, 403, 10, 3565}, {54, 404, 5, 3565}, {55, 405, 1, 3565}, {35, 406, 1, 3566}, {36, 407, 5, 3566}, {37, 408, 5, 3566}, {38, 409, 10, 3566}, {39, 410, 20, 3566}, {40, 411, 10, 3566}, {41, 412, 10, 3566}, {42, 413, 30, 3566}, {43, 414, 30, 3566}, {44, 415, 10, 3566}, {45, 416, 5, 3566}, {46, 417, 20, 3566}, {47, 418, 30, 3566}, {48, 419, 20, 3566}, {49, 420, 5, 3566}, {50, 421, 1, 3566}, {51, 422, 5, 3566}, {52, 423, 10, 3566}, {53, 424, 10, 3566}, {54, 425, 5, 3566}, {55, 426, 1, 3566}, {35, 407, 1, 3567}, {36, 409, 5, 3567}, {37, 410, 5, 3567}, {38, 412, 10, 3567}, {39, 413, 20, 3567}, {40, 414, 10, 3567}, {41, 416, 10, 3567}, {42, 417, 30, 3567}, {43, 418, 30, 3567}, {44, 419, 10, 3567}, {45, 421, 5, 3567}, {46, 422, 20, 3567}, {47, 423, 30, 3567}, {48, 424, 20, 3567}, {49, 425, 5, 3567}, {50, 427, 1, 3567}, {51, 428, 5, 3567}, {52, 429, 10, 3567}, {53, 430, 10, 3567}, {54, 431, 5, 3567}, {55, 432, 1, 3567}, {35, 408, 1, 3568}, {36, 410, 5, 3568}, {37, 411, 5, 3568}, {38, 413, 10, 3568}, {39, 414, 20, 3568}, {40, 415, 10, 3568}, {41, 417, 10, 3568}, {42, 418, 30, 3568}, {43, 419, 30, 3568}, {44, 420, 10, 3568}, {45, 422, 5, 3568}, {46, 423, 20, 3568}, {47, 424, 30, 3568}, {48, 425, 20, 3568}, {49, 426, 5, 3568}, {50, 428, 1, 3568}, {51, 429, 5, 3568}, {52, 430, 10, 3568}, {53, 431, 10, 3568}, {54, 432, 5, 3568}, {55, 433, 1, 3568}, {35, 409, 1, 3569}, {36, 412, 5, 3569}, {37, 413, 5, 3569}, {38, 416, 10, 3569}, {39, 417, 20, 3569}, {40, 418, 10, 3569}, {41, 421, 10, 3569}, {42, 422, 30, 3569}, {43, 423, 30, 3569}, {44, 424, 10, 3569}, {45, 427, 5, 3569}, {46, 428, 20, 3569}, {47, 429, 30, 3569}, {48, 430, 20, 3569}, {49, 431, 5, 3569}, {50, 434, 1, 3569}, {51, 435, 5, 3569}, {52, 436, 10, 3569}, {53, 437, 10, 3569}, {54, 438, 5, 3569}, {55, 439, 1, 3569}, {35, 410, 1, 3570}, {36, 413, 5, 3570}, {37, 414, 5, 3570}, {38, 417, 10, 3570}, {39, 418, 20, 3570}, {40, 419, 10, 3570}, {41, 422, 10, 3570}, {42, 423, 30, 3570}, {43, 424, 30, 3570}, {44, 425, 10, 3570}, {45, 428, 5, 3570}, {46, 429, 20, 3570}, {47, 430, 30, 3570}, {48, 431, 20, 3570}, {49, 432, 5, 3570}, {50, 435, 1, 3570}, {51, 436, 5, 3570}, {52, 437, 10, 3570}, {53, 438, 10, 3570}, {54, 439, 5, 3570}, {55, 440, 1, 3570}, {35, 411, 1, 3571}, {36, 414, 5, 3571}, {37, 415, 5, 3571}, {38, 418, 10, 3571}, {39, 419, 20, 3571}, {40, 420, 10, 3571}, {41, 423, 10, 3571}, {42, 424, 30, 3571}, {43, 425, 30, 3571}, {44, 426, 10, 3571}, {45, 429, 5, 3571}, {46, 430, 20, 3571}, {47, 431, 30, 3571}, {48, 432, 20, 3571}, {49, 433, 5, 3571}, {50, 436, 1, 3571}, {51, 437, 5, 3571}, {52, 438, 10, 3571}, {53, 439, 10, 3571}, {54, 440, 5, 3571}, {55, 441, 1, 3571}, {35, 412, 1, 3572}, {36, 416, 5, 3572}, {37, 417, 5, 3572}, {38, 421, 10, 3572}, {39, 422, 20, 3572}, {40, 423, 10, 3572}, {41, 427, 10, 3572}, {42, 428, 30, 3572}, {43, 429, 30, 3572}, {44, 430, 10, 3572}, {45, 434, 5, 3572}, {46, 435, 20, 3572}, {47, 436, 30, 3572}, {48, 437, 20, 3572}, {49, 438, 5, 3572}, {50, 442, 1, 3572}, {51, 443, 5, 3572}, {52, 444,

10, 3572}, {53, 445, 10, 3572}, {54, 446, 5, 3572}, {55, 447, 1, 3572}, {35, 413, 1,
3573}, {36, 417, 5, 3573}, {37, 418, 5, 3573}, {38, 422, 10, 3573}, {39, 423, 20, 3573},
{40, 424, 10, 3573}, {41, 428, 10, 3573}, {42, 429, 30, 3573}, {43, 430, 30, 3573}, {44,
431, 10, 3573}, {45, 435, 5, 3573}, {46, 436, 20, 3573}, {47, 437, 30, 3573}, {48, 438,
20, 3573}, {49, 439, 5, 3573}, {50, 443, 1, 3573}, {51, 444, 5, 3573}, {52, 445, 10,
3573}, {53, 446, 10, 3573}, {54, 447, 5, 3573}, {55, 448, 1, 3573}, {35, 414, 1, 3574},
{36, 418, 5, 3574}, {37, 419, 5, 3574}, {38, 423, 10, 3574}, {39, 424, 20, 3574}, {40,
425, 10, 3574}, {41, 429, 10, 3574}, {42, 430, 30, 3574}, {43, 431, 30, 3574}, {44, 432,
10, 3574}, {45, 436, 5, 3574}, {46, 437, 20, 3574}, {47, 438, 30, 3574}, {48, 439, 20,
3574}, {49, 440, 5, 3574}, {50, 444, 1, 3574}, {51, 445, 5, 3574}, {52, 446, 10, 3574},
{53, 447, 10, 3574}, {54, 448, 5, 3574}, {55, 449, 1, 3574}, {35, 415, 1, 3575}, {36, 419,
5, 3575}, {37, 420, 5, 3575}, {38, 424, 10, 3575}, {39, 425, 20, 3575}, {40, 426, 10,
3575}, {41, 430, 10, 3575}, {42, 431, 30, 3575}, {43, 432, 30, 3575}, {44, 433, 10, 3575},
{45, 437, 5, 3575}, {46, 438, 20, 3575}, {47, 439, 30, 3575}, {48, 440, 20, 3575}, {49,
441, 5, 3575}, {50, 445, 1, 3575}, {51, 446, 5, 3575}, {52, 447, 10, 3575}, {53, 448, 10,
3575}, {54, 449, 5, 3575}, {55, 450, 1, 3575}, {56, 290, 1, 3576}, {57, 291, 2, 3576},
{58, 292, 2, 3576}, {59, 293, 1, 3576}, {60, 294, 2, 3576}, {61, 295, 1, 3576}, {57, 290,
1, 3577}, {59, 291, 2, 3577}, {60, 292, 2, 3577}, {62, 293, 1, 3577}, {63, 294, 2, 3577},
{64, 295, 1, 3577}, {58, 290, 1, 3578}, {60, 291, 2, 3578}, {61, 292, 2, 3578}, {63, 293,
1, 3578}, {64, 294, 2, 3578}, {65, 295, 1, 3578}, {59, 290, 1, 3579}, {62, 291, 2, 3579},
{63, 292, 2, 3579}, {66, 293, 1, 3579}, {67, 294, 2, 3579}, {68, 295, 1, 3579}, {60, 290,
1, 3580}, {63, 291, 2, 3580}, {64, 292, 2, 3580}, {67, 293, 1, 3580}, {68, 294, 2, 3580},
{69, 295, 1, 3580}, {61, 290, 1, 3581}, {64, 291, 2, 3581}, {65, 292, 2, 3581}, {68, 293,
1, 3581}, {69, 294, 2, 3581}, {70, 295, 1, 3581}, {62, 290, 1, 3582}, {66, 291, 2, 3582},
{67, 292, 2, 3582}, {71, 293, 1, 3582}, {72, 294, 2, 3582}, {73, 295, 1, 3582}, {63, 290,
1, 3583}, {67, 291, 2, 3583}, {68, 292, 2, 3583}, {72, 293, 1, 3583}, {73, 294, 2, 3583},
{74, 295, 1, 3583}, {64, 290, 1, 3584}, {68, 291, 2, 3584}, {69, 292, 2, 3584}, {73, 293,
1, 3584}, {74, 294, 2, 3584}, {75, 295, 1, 3584}, {65, 290, 1, 3585}, {69, 291, 2, 3585},
{70, 292, 2, 3585}, {74, 293, 1, 3585}, {75, 294, 2, 3585}, {76, 295, 1, 3585}, {66, 290,
1, 3586}, {71, 291, 2, 3586}, {72, 292, 2, 3586}, {77, 293, 1, 3586}, {78, 294, 2, 3586},
{79, 295, 1, 3586}, {67, 290, 1, 3587}, {72, 291, 2, 3587}, {73, 292, 2, 3587}, {78, 293,
1, 3587}, {79, 294, 2, 3587}, {80, 295, 1, 3587}, {68, 290, 1, 3588}, {73, 291, 2, 3588},
{74, 292, 2, 3588}, {79, 293, 1, 3588}, {80, 294, 2, 3588}, {81, 295, 1, 3588}, {69, 290,
1, 3589}, {74, 291, 2, 3589}, {75, 292, 2, 3589}, {80, 293, 1, 3589}, {81, 294, 2, 3589},
{82, 295, 1, 3589}, {70, 290, 1, 3590}, {75, 291, 2, 3590}, {76, 292, 2, 3590}, {81, 293,
1, 3590}, {82, 294, 2, 3590}, {83, 295, 1, 3590}, {56, 296, 1, 3591}, {57, 297, 2, 3591},
{58, 298, 2, 3591}, {59, 299, 1, 3591}, {60, 300, 2, 3591}, {61, 301, 1, 3591}, {56, 297,
1, 3592}, {57, 299, 2, 3592}, {58, 300, 2, 3592}, {59, 302, 1, 3592}, {60, 303, 2, 3592},
{61, 304, 1, 3592}, {56, 298, 1, 3593}, {57, 300, 2, 3593}, {58, 301, 2, 3593}, {59, 303,
1, 3593}, {60, 304, 2, 3593}, {61, 305, 1, 3593}, {57, 296, 1, 3594}, {59, 297, 2, 3594},
{60, 298, 2, 3594}, {62, 299, 1, 3594}, {63, 300, 2, 3594}, {64, 301, 1, 3594}, {57, 297,
1, 3595}, {59, 299, 2, 3595}, {60, 300, 2, 3595}, {62, 302, 1, 3595}, {63, 303, 2, 3595},
{64, 304, 1, 3595}, {57, 298, 1, 3596}, {59, 300, 2, 3596}, {60, 301, 2, 3596}, {62, 303,
1, 3596}, {63, 304, 2, 3596}, {64, 305, 1, 3596}, {58, 296, 1, 3597}, {60, 297, 2, 3597},
{61, 298, 2, 3597}, {63, 299, 1, 3597}, {64, 300, 2, 3597}, {65, 301, 1, 3597}, {58, 297,
1, 3598}, {60, 299, 2, 3598}, {61, 300, 2, 3598}, {63, 302, 1, 3598}, {64, 303, 2, 3598},
{65, 304, 1, 3598}, {58, 298, 1, 3599}, {60, 300, 2, 3599}, {61, 301, 2, 3599}, {63, 303,
1, 3599}, {64, 304, 2, 3599}, {65, 305, 1, 3599}, {59, 296, 1, 3600}, {62, 297, 2, 3600},
{63, 298, 2, 3600}, {66, 299, 1, 3600}, {67, 300, 2, 3600}, {68, 301, 1, 3600}, {59, 297,
1, 3601}, {62, 299, 2, 3601}, {63, 300, 2, 3601}, {66, 302, 1, 3601}, {67, 303, 2, 3601},
{68, 304, 1, 3601}, {59, 298, 1, 3602}, {62, 300, 2, 3602}, {63, 301, 2, 3602}, {66, 303,
1, 3602}, {67, 304, 2, 3602}, {68, 305, 1, 3602}, {60, 296, 1, 3603}, {63, 297, 2, 3603},
{64, 298, 2, 3603}, {67, 299, 1, 3603}, {68, 300, 2, 3603}, {69, 301, 1, 3603}, {60, 297,
1, 3604}, {63, 299, 2, 3604}, {64, 300, 2, 3604}, {67, 302, 1, 3604}, {68, 303, 2, 3604},
{69, 304, 1, 3604}, {60, 298, 1, 3605}, {63, 300, 2, 3605}, {64, 301, 2, 3605}, {67, 303,
1, 3605}, {68, 304, 2, 3605}, {69, 305, 1, 3605}, {61, 296, 1, 3606}, {64, 297, 2, 3606},
{65, 298, 2, 3606}, {68, 299, 1, 3606}, {69, 300, 2, 3606}, {70, 301, 1, 3606}, {61, 297,
1, 3607}, {64, 299, 2, 3607}, {65, 300, 2, 3607}, {68, 302, 1, 3607}, {69, 303, 2, 3607},
{70, 304, 1, 3607}, {61, 298, 1, 3608}, {64, 300, 2, 3608}, {65, 301, 2, 3608}, {68, 303,
1, 3608}, {69, 304, 2, 3608}, {70, 305, 1, 3608}, {62, 296, 1, 3609}, {66, 297, 2, 3609},
{67, 298, 2, 3609}, {71, 299, 1, 3609}, {72, 300, 2, 3609}, {73, 301, 1, 3609}, {62, 297,
1, 3610}, {66, 299, 2, 3610}, {67, 300, 2, 3610}, {71, 302, 1, 3610}, {72, 303, 2, 3610},
{73, 304, 1, 3610}, {62, 298, 1, 3611}, {66, 300, 2, 3611}, {67, 301, 2, 3611}, {71, 303,
1, 3611}, {72, 304, 2, 3611}, {73, 305, 1, 3611}, {63, 296, 1, 3612}, {67, 297, 2, 3612},
{68, 298, 2, 3612}, {72, 299, 1, 3612}, {73, 300, 2, 3612}, {74, 301, 1, 3612}, {63, 297,
1, 3613}, {67, 299, 2, 3613}, {68, 300, 2, 3613}, {72, 302, 1, 3613}, {73, 303, 2, 3613},
{74, 304, 1, 3613}, {63, 298, 1, 3614}, {67, 300, 2, 3614}, {68, 301, 2, 3614}, {72, 303,
1, 3614}, {73, 304, 2, 3614}, {74, 305, 1, 3614}, {64, 296, 1, 3615}, {68, 297, 2, 3615},
{69, 298, 2, 3615}, {73, 299, 1, 3615}, {74, 300, 2, 3615}, {75, 301, 1, 3615}, {64, 297,

1, 3616}, {68, 299, 2, 3616}, {69, 300, 2, 3616}, {73, 302, 1, 3616}, {74, 303, 2, 3616}, {75, 304, 1, 3616}, {64, 298, 1, 3617}, {68, 300, 2, 3617}, {69, 301, 2, 3617}, {73, 303, 1, 3617}, {74, 304, 2, 3617}, {75, 305, 1, 3617}, {65, 296, 1, 3618}, {69, 297, 2, 3618}, {70, 298, 2, 3618}, {74, 299, 1, 3618}, {75, 300, 2, 3618}, {76, 301, 1, 3618}, {65, 297, 1, 3619}, {69, 299, 2, 3619}, {70, 300, 2, 3619}, {74, 302, 1, 3619}, {75, 303, 2, 3619}, {76, 304, 1, 3619}, {65, 298, 1, 3620}, {69, 300, 2, 3620}, {70, 301, 2, 3620}, {74, 303, 1, 3620}, {75, 304, 2, 3620}, {76, 305, 1, 3620}, {66, 296, 1, 3621}, {71, 297, 2, 3621}, {72, 298, 2, 3621}, {77, 299, 1, 3621}, {78, 300, 2, 3621}, {79, 301, 1, 3621}, {66, 297, 1, 3622}, {71, 299, 2, 3622}, {72, 300, 2, 3622}, {77, 302, 1, 3622}, {78, 303, 2, 3622}, {79, 304, 1, 3622}, {66, 298, 1, 3623}, {71, 300, 2, 3623}, {72, 301, 2, 3623}, {77, 303, 1, 3623}, {78, 304, 2, 3623}, {79, 305, 1, 3623}, {67, 296, 1, 3624}, {72, 297, 2, 3624}, {73, 298, 2, 3624}, {78, 299, 1, 3624}, {79, 300, 2, 3624}, {80, 301, 1, 3624}, {67, 297, 1, 3625}, {72, 299, 2, 3625}, {73, 300, 2, 3625}, {78, 302, 1, 3625}, {79, 303, 2, 3625}, {80, 304, 1, 3625}, {67, 298, 1, 3626}, {72, 300, 2, 3626}, {73, 301, 2, 3626}, {78, 303, 1, 3626}, {79, 304, 2, 3626}, {80, 305, 1, 3626}, {68, 296, 1, 3627}, {73, 297, 2, 3627}, {74, 298, 2, 3627}, {79, 299, 1, 3627}, {80, 300, 2, 3627}, {81, 301, 1, 3627}, {68, 297, 1, 3628}, {73, 299, 2, 3628}, {74, 300, 2, 3628}, {79, 302, 1, 3628}, {80, 303, 2, 3628}, {81, 304, 1, 3628}, {68, 298, 1, 3629}, {73, 300, 2, 3629}, {74, 301, 2, 3629}, {79, 303, 1, 3629}, {80, 304, 2, 3629}, {81, 305, 1, 3629}, {69, 296, 1, 3630}, {74, 297, 2, 3630}, {75, 298, 2, 3630}, {80, 299, 1, 3630}, {81, 300, 2, 3630}, {82, 301, 1, 3630}, {69, 297, 1, 3631}, {74, 299, 2, 3631}, {75, 300, 2, 3631}, {80, 302, 1, 3631}, {81, 303, 2, 3631}, {82, 304, 1, 3631}, {69, 298, 1, 3632}, {74, 300, 2, 3632}, {75, 301, 2, 3632}, {80, 303, 1, 3632}, {81, 304, 2, 3632}, {82, 305, 1, 3632}, {70, 296, 1, 3633}, {75, 297, 2, 3633}, {76, 298, 2, 3633}, {81, 299, 1, 3633}, {82, 300, 2, 3633}, {83, 301, 1, 3633}, {70, 297, 1, 3634}, {75, 299, 2, 3634}, {76, 300, 2, 3634}, {81, 302, 1, 3634}, {82, 303, 2, 3634}, {83, 304, 1, 3634}, {70, 298, 1, 3635}, {75, 300, 2, 3635}, {76, 301, 2, 3635}, {81, 303, 1, 3635}, {82, 304, 2, 3635}, {83, 305, 1, 3635}, {56, 296, 1, 3636}, {57, 297, 3, 3636}, {58, 298, 3, 3636}, {59, 299, 3, 3636}, {60, 300, 6, 3636}, {61, 301, 3, 3636}, {62, 302, 1, 3636}, {63, 303, 3, 3636}, {64, 304, 3, 3636}, {65, 305, 1, 3636}, {57, 296, 1, 3637}, {59, 297, 3, 3637}, {60, 298, 3, 3637}, {62, 299, 3, 3637}, {63, 300, 6, 3637}, {64, 301, 3, 3637}, {66, 302, 1, 3637}, {67, 303, 3, 3637}, {68, 304, 3, 3637}, {69, 305, 1, 3637}, {58, 296, 1, 3638}, {60, 297, 3, 3638}, {61, 298, 3, 3638}, {63, 299, 3, 3638}, {64, 300, 6, 3638}, {65, 301, 3, 3638}, {67, 302, 1, 3638}, {68, 303, 3, 3638}, {69, 304, 3, 3638}, {70, 305, 1, 3638}, {59, 296, 1, 3639}, {62, 297, 3, 3639}, {63, 298, 3, 3639}, {66, 299, 3, 3639}, {67, 300, 6, 3639}, {68, 301, 3, 3639}, {71, 302, 1, 3639}, {72, 303, 3, 3639}, {73, 304, 3, 3639}, {74, 305, 1, 3639}, {60, 296, 1, 3640}, {63, 297, 3, 3640}, {64, 298, 3, 3640}, {67, 299, 3, 3640}, {68, 300, 6, 3640}, {69, 301, 3, 3640}, {72, 302, 1, 3640}, {73, 303, 3, 3640}, {74, 304, 3, 3640}, {75, 305, 1, 3640}, {61, 296, 1, 3641}, {64, 297, 3, 3641}, {65, 298, 3, 3641}, {68, 299, 3, 3641}, {69, 300, 6, 3641}, {70, 301, 3, 3641}, {73, 302, 1, 3641}, {74, 303, 3, 3641}, {75, 304, 3, 3641}, {76, 305, 1, 3641}, {62, 296, 1, 3642}, {66, 297, 3, 3642}, {67, 298, 3, 3642}, {71, 299, 3, 3642}, {72, 300, 6, 3642}, {73, 301, 3, 3642}, {77, 302, 1, 3642}, {78, 303, 3, 3642}, {79, 304, 3, 3642}, {80, 305, 1, 3642}, {63, 296, 1, 3643}, {67, 297, 3, 3643}, {68, 298, 3, 3643}, {72, 299, 3, 3643}, {73, 300, 6, 3643}, {74, 301, 3, 3643}, {78, 302, 1, 3643}, {79, 303, 3, 3643}, {80, 304, 3, 3643}, {81, 305, 1, 3643}, {64, 296, 1, 3644}, {68, 297, 3, 3644}, {69, 298, 3, 3644}, {73, 299, 3, 3644}, {74, 300, 6, 3644}, {75, 301, 3, 3644}, {79, 302, 1, 3644}, {80, 303, 3, 3644}, {81, 304, 3, 3644}, {82, 305, 1, 3644}, {65, 296, 1, 3645}, {69, 297, 3, 3645}, {70, 298, 3, 3645}, {74, 299, 3, 3645}, {75, 300, 6, 3645}, {76, 301, 3, 3645}, {80, 302, 1, 3645}, {81, 303, 3, 3645}, {82, 304, 3, 3645}, {83, 305, 1, 3645}, {56, 306, 1, 3646}, {57, 307, 3, 3646}, {58, 308, 3, 3646}, {59, 309, 3, 3646}, {60, 310, 6, 3646}, {61, 311, 3, 3646}, {62, 312, 1, 3646}, {63, 313, 3, 3646}, {64, 314, 3, 3646}, {65, 315, 1, 3646}, {56, 307, 1, 3647}, {57, 309, 3, 3647}, {58, 310, 3, 3647}, {59, 312, 3, 3647}, {60, 313, 6, 3647}, {61, 314, 3, 3647}, {62, 316, 1, 3647}, {63, 317, 3, 3647}, {64, 318, 3, 3647}, {65, 319, 1, 3647}, {56, 308, 1, 3648}, {57, 310, 3, 3648}, {58, 311, 3, 3648}, {59, 313, 3, 3648}, {60, 314, 6, 3648}, {61, 315, 3, 3648}, {62, 317, 1, 3648}, {63, 318, 3, 3648}, {64, 319, 3, 3648}, {65, 320, 1, 3648}, {57, 306, 1, 3649}, {59, 307, 3, 3649}, {60, 308, 3, 3649}, {62, 309, 3, 3649}, {63, 310, 6, 3649}, {64, 311, 3, 3649}, {66, 312, 1, 3649}, {67, 313, 3, 3649}, {68, 314, 3, 3649}, {69, 315, 1, 3649}, {57, 307, 1, 3650}, {59, 309, 3, 3650}, {60, 310, 3, 3650}, {62, 312, 3, 3650}, {63, 313, 6, 3650}, {64, 314, 3, 3650}, {66, 316, 1, 3650}, {67, 317, 3, 3650}, {68, 318, 3, 3650}, {69, 319, 1, 3650}, {57, 308, 1, 3651}, {59, 310, 3, 3651}, {60, 311, 3, 3651}, {62, 313, 3, 3651}, {63, 314, 6, 3651}, {64, 315, 3, 3651}, {66, 317, 1, 3651}, {67, 318, 3, 3651}, {68, 319, 3, 3651}, {69, 320, 1, 3651}, {58, 306, 1, 3652}, {60, 307, 3, 3652}, {61, 308, 3, 3652}, {63, 309, 3, 3652}, {64, 310, 6, 3652}, {65, 311, 3, 3652}, {67, 312, 1, 3652}, {68, 313, 3, 3652}, {69, 314, 3, 3652}, {70, 315, 1, 3652}, {58, 307, 1, 3653}, {60, 309, 3, 3653}, {61, 310, 3, 3653}, {63, 312, 3, 3653}, {64, 313, 6, 3653}, {65, 314, 3, 3653}, {67, 316, 1, 3653}, {68, 317, 3, 3653}, {69, 318, 3, 3653}, {70, 319, 1, 3653}, {58, 308, 1, 3654}, {60, 310, 3, 3654}, {61, 311, 3, 3654}, {63, 313, 3, 3654}, {64, 314, 6, 3654}, {65, 315, 3, 3654}, {67, 317, 1, 3654}, {68, 318, 3, 3654}, {69, 319, 3, 3654}, {70, 320, 1, 3654}, {59, 306, 1, 3655},

{62, 307, 3, 3655}, {63, 308, 3, 3655}, {66, 309, 3, 3655}, {67, 310, 6, 3655}, {68, 311, 3, 3655}, {71, 312, 1, 3655}, {72, 313, 3, 3655}, {73, 314, 3, 3655}, {74, 315, 1, 3655}, {59, 307, 1, 3656}, {62, 309, 3, 3656}, {63, 310, 3, 3656}, {66, 312, 3, 3656}, {67, 313, 6, 3656}, {68, 314, 3, 3656}, {71, 316, 1, 3656}, {72, 317, 3, 3656}, {73, 318, 3, 3656}, {74, 319, 1, 3656}, {59, 308, 1, 3657}, {62, 310, 3, 3657}, {63, 311, 3, 3657}, {66, 313, 3, 3657}, {67, 314, 6, 3657}, {68, 315, 3, 3657}, {71, 317, 1, 3657}, {72, 318, 3, 3657}, {73, 319, 3, 3657}, {74, 320, 1, 3657}, {60, 306, 1, 3658}, {63, 307, 3, 3658}, {64, 308, 3, 3658}, {67, 309, 3, 3658}, {68, 310, 6, 3658}, {69, 311, 3, 3658}, {72, 312, 1, 3658}, {73, 313, 3, 3658}, {74, 314, 3, 3658}, {75, 315, 1, 3658}, {60, 307, 1, 3659}, {63, 309, 3, 3659}, {64, 310, 3, 3659}, {67, 312, 3, 3659}, {68, 313, 6, 3659}, {69, 314, 3, 3659}, {72, 316, 1, 3659}, {73, 317, 3, 3659}, {74, 318, 3, 3659}, {75, 319, 1, 3659}, {60, 308, 1, 3660}, {63, 310, 3, 3660}, {64, 311, 3, 3660}, {67, 313, 3, 3660}, {68, 314, 6, 3660}, {69, 315, 3, 3660}, {72, 317, 1, 3660}, {73, 318, 3, 3660}, {74, 319, 3, 3660}, {75, 320, 1, 3660}, {61, 306, 1, 3661}, {64, 307, 3, 3661}, {65, 308, 3, 3661}, {68, 309, 3, 3661}, {69, 310, 6, 3661}, {70, 311, 3, 3661}, {73, 312, 1, 3661}, {74, 313, 3, 3661}, {75, 314, 3, 3661}, {76, 315, 1, 3661}, {61, 307, 1, 3662}, {64, 309, 3, 3662}, {65, 310, 3, 3662}, {68, 312, 3, 3662}, {69, 313, 6, 3662}, {70, 314, 3, 3662}, {73, 316, 1, 3662}, {74, 317, 3, 3662}, {75, 318, 3, 3662}, {76, 319, 1, 3662}, {61, 308, 1, 3663}, {64, 310, 3, 3663}, {65, 311, 3, 3663}, {68, 313, 3, 3663}, {69, 314, 6, 3663}, {70, 315, 3, 3663}, {73, 317, 1, 3663}, {74, 318, 3, 3663}, {75, 319, 3, 3663}, {76, 320, 1, 3663}, {62, 306, 1, 3664}, {66, 307, 3, 3664}, {67, 308, 3, 3664}, {71, 309, 3, 3664}, {72, 310, 6, 3664}, {73, 311, 3, 3664}, {77, 312, 1, 3664}, {78, 313, 3, 3664}, {79, 314, 3, 3664}, {80, 315, 1, 3664}, {62, 307, 1, 3665}, {66, 309, 3, 3665}, {67, 310, 3, 3665}, {71, 312, 3, 3665}, {72, 313, 6, 3665}, {73, 314, 3, 3665}, {77, 316, 1, 3665}, {78, 317, 3, 3665}, {79, 318, 3, 3665}, {80, 319, 1, 3665}, {62, 308, 1, 3666}, {66, 310, 3, 3666}, {67, 311, 3, 3666}, {71, 313, 3, 3666}, {72, 314, 6, 3666}, {73, 315, 3, 3666}, {77, 317, 1, 3666}, {78, 318, 3, 3666}, {79, 319, 3, 3666}, {80, 320, 1, 3666}, {63, 306, 1, 3667}, {67, 307, 3, 3667}, {68, 308, 3, 3667}, {72, 309, 3, 3667}, {73, 310, 6, 3667}, {74, 311, 3, 3667}, {78, 312, 1, 3667}, {79, 313, 3, 3667}, {80, 314, 3, 3667}, {81, 315, 1, 3667}, {63, 307, 1, 3668}, {67, 309, 3, 3668}, {68, 310, 3, 3668}, {72, 312, 3, 3668}, {73, 313, 6, 3668}, {74, 314, 3, 3668}, {78, 316, 1, 3668}, {79, 317, 3, 3668}, {80, 318, 3, 3668}, {81, 319, 1, 3668}, {63, 308, 1, 3669}, {67, 310, 3, 3669}, {68, 311, 3, 3669}, {72, 313, 3, 3669}, {73, 314, 6, 3669}, {74, 315, 3, 3669}, {78, 317, 1, 3669}, {79, 318, 3, 3669}, {80, 319, 3, 3669}, {81, 320, 1, 3669}, {64, 306, 1, 3670}, {68, 307, 3, 3670}, {69, 308, 3, 3670}, {73, 309, 3, 3670}, {74, 310, 6, 3670}, {75, 311, 3, 3670}, {79, 312, 1, 3670}, {80, 313, 3, 3670}, {81, 314, 3, 3670}, {82, 315, 1, 3670}, {64, 307, 1, 3671}, {68, 309, 3, 3671}, {69, 310, 3, 3671}, {73, 312, 3, 3671}, {74, 313, 6, 3671}, {75, 314, 3, 3671}, {79, 316, 1, 3671}, {80, 317, 3, 3671}, {81, 318, 3, 3671}, {82, 319, 1, 3671}, {64, 308, 1, 3672}, {68, 310, 3, 3672}, {69, 311, 3, 3672}, {73, 313, 3, 3672}, {74, 314, 6, 3672}, {75, 315, 3, 3672}, {79, 317, 1, 3672}, {80, 318, 3, 3672}, {81, 319, 3, 3672}, {82, 320, 1, 3672}, {65, 306, 1, 3673}, {69, 307, 3, 3673}, {70, 308, 3, 3673}, {74, 309, 3, 3673}, {75, 310, 6, 3673}, {76, 311, 3, 3673}, {80, 312, 1, 3673}, {81, 313, 3, 3673}, {82, 314, 3, 3673}, {83, 315, 1, 3673}, {65, 307, 1, 3674}, {69, 309, 3, 3674}, {70, 310, 3, 3674}, {74, 312, 3, 3674}, {75, 313, 6, 3674}, {76, 314, 3, 3674}, {80, 316, 1, 3674}, {81, 317, 3, 3674}, {82, 318, 3, 3674}, {83, 319, 1, 3674}, {65, 308, 1, 3675}, {69, 310, 3, 3675}, {70, 311, 3, 3675}, {74, 313, 3, 3675}, {75, 314, 6, 3675}, {76, 315, 3, 3675}, {80, 317, 1, 3675}, {81, 318, 3, 3675}, {82, 319, 3, 3675}, {83, 320, 1, 3675}, {56, 321, 1, 3676}, {57, 322, 3, 3676}, {58, 323, 3, 3676}, {59, 324, 3, 3676}, {60, 325, 6, 3676}, {61, 326, 3, 3676}, {62, 327, 1, 3676}, {63, 328, 3, 3676}, {64, 329, 3, 3676}, {65, 330, 1, 3676}, {56, 322, 1, 3677}, {57, 324, 3, 3677}, {58, 325, 3, 3677}, {59, 327, 3, 3677}, {60, 328, 6, 3677}, {61, 329, 3, 3677}, {62, 331, 1, 3677}, {63, 332, 3, 3677}, {64, 333, 3, 3677}, {65, 334, 1, 3677}, {56, 323, 1, 3678}, {57, 325, 3, 3678}, {58, 326, 3, 3678}, {59, 328, 3, 3678}, {60, 329, 6, 3678}, {61, 330, 3, 3678}, {62, 332, 1, 3678}, {63, 333, 3, 3678}, {64, 334, 3, 3678}, {65, 335, 1, 3678}, {56, 324, 1, 3679}, {57, 327, 3, 3679}, {58, 328, 3, 3679}, {59, 331, 3, 3679}, {60, 332, 6, 3679}, {61, 333, 3, 3679}, {62, 336, 1, 3679}, {63, 337, 3, 3679}, {64, 338, 3, 3679}, {65, 339, 1, 3679}, {56, 325, 1, 3680}, {57, 328, 3, 3680}, {58, 329, 3, 3680}, {59, 332, 3, 3680}, {60, 333, 6, 3680}, {61, 334, 3, 3680}, {62, 337, 1, 3680}, {63, 338, 3, 3680}, {64, 339, 3, 3680}, {65, 340, 1, 3680}, {56, 326, 1, 3681}, {57, 329, 3, 3681}, {58, 330, 3, 3681}, {59, 333, 3, 3681}, {60, 334, 6, 3681}, {61, 335, 3, 3681}, {62, 338, 1, 3681}, {63, 339, 3, 3681}, {64, 340, 3, 3681}, {65, 341, 1, 3681}, {57, 321, 1, 3682}, {59, 322, 3, 3682}, {60, 323, 3, 3682}, {62, 324, 3, 3682}, {63, 325, 6, 3682}, {64, 326, 3, 3682}, {66, 327, 1, 3682}, {67, 328, 3, 3682}, {68, 329, 3, 3682}, {69, 330, 1, 3682}, {57, 322, 1, 3683}, {59, 324, 3, 3683}, {60, 325, 3, 3683}, {62, 327, 3, 3683}, {63, 328, 6, 3683}, {64, 329, 3, 3683}, {66, 331, 1, 3683}, {67, 332, 3, 3683}, {68, 333, 3, 3683}, {69, 334, 1, 3683}, {57, 323, 1, 3684}, {59, 325, 3, 3684}, {60, 326, 3, 3684}, {62, 328, 3, 3684}, {63, 329, 6, 3684}, {64, 330, 3, 3684}, {66, 332, 1, 3684}, {67, 333, 3, 3684}, {68, 334, 3, 3684}, {69, 335, 1, 3684}, {57, 324, 1, 3685}, {59, 327, 3, 3685}, {60, 328, 3, 3685}, {62, 331, 3, 3685}, {63, 332, 6, 3685}, {64, 333, 3, 3685}, {66, 336, 1, 3685}, {67, 337, 3, 3685}, {68, 338, 3, 3685}, {69, 339, 1, 3685}, {57, 325, 1, 3686}, {59, 328,

3, 3686}, {60, 329, 3, 3686}, {62, 332, 3, 3686}, {63, 333, 6, 3686}, {64, 334, 3, 3686},
{66, 337, 1, 3686}, {67, 338, 3, 3686}, {68, 339, 3, 3686}, {69, 340, 1, 3686}, {57, 326,
1, 3687}, {59, 329, 3, 3687}, {60, 330, 3, 3687}, {62, 333, 3, 3687}, {63, 334, 6, 3687},
{64, 335, 3, 3687}, {66, 338, 1, 3687}, {67, 339, 3, 3687}, {68, 340, 3, 3687}, {69, 341,
1, 3687}, {58, 321, 1, 3688}, {60, 322, 3, 3688}, {61, 323, 3, 3688}, {63, 324, 3, 3688},
{64, 325, 6, 3688}, {65, 326, 3, 3688}, {67, 327, 1, 3688}, {68, 328, 3, 3688}, {69, 329,
3, 3688}, {70, 330, 1, 3688}, {58, 322, 1, 3689}, {60, 324, 3, 3689}, {61, 325, 3, 3689},
{63, 327, 3, 3689}, {64, 328, 6, 3689}, {65, 329, 3, 3689}, {67, 331, 1, 3689}, {68, 332,
3, 3689}, {69, 333, 3, 3689}, {70, 334, 1, 3689}, {58, 323, 1, 3690}, {60, 325, 3, 3690},
{61, 326, 3, 3690}, {63, 328, 3, 3690}, {64, 329, 6, 3690}, {65, 330, 3, 3690}, {67, 332,
1, 3690}, {68, 333, 3, 3690}, {69, 334, 3, 3690}, {70, 335, 1, 3690}, {58, 324, 1, 3691},
{60, 327, 3, 3691}, {61, 328, 3, 3691}, {63, 331, 3, 3691}, {64, 332, 6, 3691}, {65, 333,
3, 3691}, {67, 336, 1, 3691}, {68, 337, 3, 3691}, {69, 338, 3, 3691}, {70, 339, 1, 3691},
{58, 325, 1, 3692}, {60, 328, 3, 3692}, {61, 329, 3, 3692}, {63, 332, 3, 3692}, {64, 333,
6, 3692}, {65, 334, 3, 3692}, {67, 337, 1, 3692}, {68, 338, 3, 3692}, {69, 339, 3, 3692},
{70, 340, 1, 3692}, {58, 326, 1, 3693}, {60, 329, 3, 3693}, {61, 330, 3, 3693}, {63, 333,
3, 3693}, {64, 334, 6, 3693}, {65, 335, 3, 3693}, {67, 338, 1, 3693}, {68, 339, 3, 3693},
{69, 340, 3, 3693}, {70, 341, 1, 3693}, {59, 321, 1, 3694}, {62, 322, 3, 3694}, {63, 323,
3, 3694}, {66, 324, 3, 3694}, {67, 325, 6, 3694}, {68, 326, 3, 3694}, {71, 327, 1, 3694},
{72, 328, 3, 3694}, {73, 329, 3, 3694}, {74, 330, 1, 3694}, {59, 322, 1, 3695}, {62, 324,
3, 3695}, {63, 325, 3, 3695}, {66, 327, 3, 3695}, {67, 328, 6, 3695}, {68, 329, 3, 3695},
{71, 331, 1, 3695}, {72, 332, 3, 3695}, {73, 333, 3, 3695}, {74, 334, 1, 3695}, {59, 323,
1, 3696}, {62, 325, 3, 3696}, {63, 326, 3, 3696}, {66, 328, 3, 3696}, {67, 329, 6, 3696},
{68, 330, 3, 3696}, {71, 332, 1, 3696}, {72, 333, 3, 3696}, {73, 334, 3, 3696}, {74, 335,
1, 3696}, {59, 324, 1, 3697}, {62, 327, 3, 3697}, {63, 328, 3, 3697}, {66, 331, 3, 3697},
{67, 332, 6, 3697}, {68, 333, 3, 3697}, {71, 336, 1, 3697}, {72, 337, 3, 3697}, {73, 338,
3, 3697}, {74, 339, 1, 3697}, {59, 325, 1, 3698}, {62, 328, 3, 3698}, {63, 329, 3, 3698},
{66, 332, 3, 3698}, {67, 333, 6, 3698}, {68, 334, 3, 3698}, {71, 337, 1, 3698}, {72, 338,
3, 3698}, {73, 339, 3, 3698}, {74, 340, 1, 3698}, {59, 326, 1, 3699}, {62, 329, 3, 3699},
{63, 330, 3, 3699}, {66, 333, 3, 3699}, {67, 334, 6, 3699}, {68, 335, 3, 3699}, {71, 338,
1, 3699}, {72, 339, 3, 3699}, {73, 340, 3, 3699}, {74, 341, 1, 3699}, {60, 321, 1, 3700},
{63, 322, 3, 3700}, {64, 323, 3, 3700}, {67, 324, 3, 3700}, {68, 325, 6, 3700}, {69, 326,
3, 3700}, {72, 327, 1, 3700}, {73, 328, 3, 3700}, {74, 329, 3, 3700}, {75, 330, 1, 3700},
{60, 322, 1, 3701}, {63, 324, 3, 3701}, {64, 325, 3, 3701}, {67, 327, 3, 3701}, {68, 328,
6, 3701}, {69, 329, 3, 3701}, {72, 331, 1, 3701}, {73, 332, 3, 3701}, {74, 333, 3, 3701},
{75, 334, 1, 3701}, {60, 323, 1, 3702}, {63, 325, 3, 3702}, {64, 326, 3, 3702}, {67, 328,
3, 3702}, {68, 329, 6, 3702}, {69, 330, 3, 3702}, {72, 332, 1, 3702}, {73, 333, 3, 3702},
{74, 334, 3, 3702}, {75, 335, 1, 3702}, {60, 324, 1, 3703}, {63, 327, 3, 3703}, {64, 328,
3, 3703}, {67, 331, 3, 3703}, {68, 332, 6, 3703}, {69, 333, 3, 3703}, {72, 336, 1, 3703},
{73, 337, 3, 3703}, {74, 338, 3, 3703}, {75, 339, 1, 3703}, {60, 325, 1, 3704}, {63, 328,
3, 3704}, {64, 329, 3, 3704}, {67, 332, 3, 3704}, {68, 333, 6, 3704}, {69, 334, 3, 3704},
{72, 337, 1, 3704}, {73, 338, 3, 3704}, {74, 339, 3, 3704}, {75, 340, 1, 3704}, {60, 326,
1, 3705}, {63, 329, 3, 3705}, {64, 330, 3, 3705}, {67, 333, 3, 3705}, {68, 334, 6, 3705},
{69, 335, 3, 3705}, {72, 338, 1, 3705}, {73, 339, 3, 3705}, {74, 340, 3, 3705}, {75, 341,
1, 3705}, {61, 321, 1, 3706}, {64, 322, 3, 3706}, {65, 323, 3, 3706}, {68, 324, 3, 3706},
{69, 325, 6, 3706}, {70, 326, 3, 3706}, {73, 327, 1, 3706}, {74, 328, 3, 3706}, {75, 329,
3, 3706}, {76, 330, 1, 3706}, {61, 322, 1, 3707}, {64, 324, 3, 3707}, {65, 325, 3, 3707},
{68, 327, 3, 3707}, {69, 328, 6, 3707}, {70, 329, 3, 3707}, {73, 331, 1, 3707}, {74, 332,
3, 3707}, {75, 333, 3, 3707}, {76, 334, 1, 3707}, {61, 323, 1, 3708}, {64, 325, 3, 3708},
{65, 326, 3, 3708}, {68, 328, 3, 3708}, {69, 329, 6, 3708}, {70, 330, 3, 3708}, {73, 332,
1, 3708}, {74, 333, 3, 3708}, {75, 334, 3, 3708}, {76, 335, 1, 3708}, {61, 324, 1, 3709},
{64, 327, 3, 3709}, {65, 328, 3, 3709}, {68, 331, 3, 3709}, {69, 332, 6, 3709}, {70, 333,
3, 3709}, {73, 336, 1, 3709}, {74, 337, 3, 3709}, {75, 338, 3, 3709}, {76, 339, 1, 3709},
{61, 325, 1, 3710}, {64, 328, 3, 3710}, {65, 329, 3, 3710}, {68, 332, 3, 3710}, {69, 333,
6, 3710}, {70, 334, 3, 3710}, {73, 337, 1, 3710}, {74, 338, 3, 3710}, {75, 339, 3, 3710},
{76, 340, 1, 3710}, {61, 326, 1, 3711}, {64, 329, 3, 3711}, {65, 330, 3, 3711}, {68, 333,
3, 3711}, {69, 334, 6, 3711}, {70, 335, 3, 3711}, {73, 338, 1, 3711}, {74, 339, 3, 3711},
{75, 340, 3, 3711}, {76, 341, 1, 3711}, {62, 321, 1, 3712}, {66, 322, 3, 3712}, {67, 323,
3, 3712}, {71, 324, 3, 3712}, {72, 325, 6, 3712}, {73, 326, 3, 3712}, {77, 327, 1, 3712},
{78, 328, 3, 3712}, {79, 329, 3, 3712}, {80, 330, 1, 3712}, {62, 322, 1, 3713}, {66, 324,
3, 3713}, {67, 325, 3, 3713}, {71, 327, 3, 3713}, {72, 328, 6, 3713}, {73, 329, 3, 3713},
{77, 331, 1, 3713}, {78, 332, 3, 3713}, {79, 333, 3, 3713}, {80, 334, 1, 3713}, {62, 323,
1, 3714}, {66, 325, 3, 3714}, {67, 326, 3, 3714}, {71, 328, 3, 3714}, {72, 329, 6, 3714},
{73, 330, 3, 3714}, {77, 332, 1, 3714}, {78, 333, 3, 3714}, {79, 334, 3, 3714}, {80, 335,
1, 3714}, {62, 324, 1, 3715}, {66, 327, 3, 3715}, {67, 328, 3, 3715}, {71, 331, 3, 3715},
{72, 332, 6, 3715}, {73, 333, 3, 3715}, {77, 336, 1, 3715}, {78, 337, 3, 3715}, {79, 338,
3, 3715}, {80, 339, 1, 3715}, {62, 325, 1, 3716}, {66, 328, 3, 3716}, {67, 329, 3, 3716},
{71, 332, 3, 3716}, {72, 333, 6, 3716}, {73, 334, 3, 3716}, {77, 337, 1, 3716}, {78, 338,
3, 3716}, {79, 339, 3, 3716}, {80, 340, 1, 3716}, {62, 326, 1, 3717}, {66, 329, 3, 3717},

{67, 330, 3, 3717}, {71, 333, 3, 3717}, {72, 334, 6, 3717}, {73, 335, 3, 3717}, {77, 338, 1, 3717}, {78, 339, 3, 3717}, {79, 340, 3, 3717}, {80, 341, 1, 3717}, {63, 321, 1, 3718}, {67, 322, 3, 3718}, {68, 323, 3, 3718}, {72, 324, 3, 3718}, {73, 325, 6, 3718}, {74, 326, 3, 3718}, {78, 327, 1, 3718}, {79, 328, 3, 3718}, {80, 329, 3, 3718}, {81, 330, 1, 3718}, {63, 322, 1, 3719}, {67, 324, 3, 3719}, {68, 325, 3, 3719}, {72, 327, 3, 3719}, {73, 328, 6, 3719}, {74, 329, 3, 3719}, {78, 331, 1, 3719}, {79, 332, 3, 3719}, {80, 333, 3, 3719}, {81, 334, 1, 3719}, {63, 323, 1, 3720}, {67, 325, 3, 3720}, {68, 326, 3, 3720}, {72, 328, 3, 3720}, {73, 329, 6, 3720}, {74, 330, 3, 3720}, {78, 332, 1, 3720}, {79, 333, 3, 3720}, {80, 334, 3, 3720}, {81, 335, 1, 3720}, {63, 324, 1, 3721}, {67, 327, 3, 3721}, {68, 328, 3, 3721}, {72, 331, 3, 3721}, {73, 332, 6, 3721}, {74, 333, 3, 3721}, {78, 336, 1, 3721}, {79, 337, 3, 3721}, {80, 338, 3, 3721}, {81, 339, 1, 3721}, {63, 325, 1, 3722}, {67, 328, 3, 3722}, {68, 329, 3, 3722}, {72, 332, 3, 3722}, {73, 333, 6, 3722}, {74, 334, 3, 3722}, {78, 337, 1, 3722}, {79, 338, 3, 3722}, {80, 339, 3, 3722}, {81, 340, 1, 3722}, {63, 326, 1, 3723}, {67, 329, 3, 3723}, {68, 330, 3, 3723}, {72, 333, 3, 3723}, {73, 334, 6, 3723}, {74, 335, 3, 3723}, {78, 338, 1, 3723}, {79, 339, 3, 3723}, {80, 340, 3, 3723}, {81, 341, 1, 3723}, {64, 321, 1, 3724}, {68, 322, 3, 3724}, {69, 323, 3, 3724}, {73, 324, 3, 3724}, {74, 325, 6, 3724}, {75, 326, 3, 3724}, {79, 327, 1, 3724}, {80, 328, 3, 3724}, {81, 329, 3, 3724}, {82, 330, 1, 3724}, {64, 322, 1, 3725}, {68, 324, 3, 3725}, {69, 325, 3, 3725}, {73, 327, 3, 3725}, {74, 328, 6, 3725}, {75, 329, 3, 3725}, {79, 331, 1, 3725}, {80, 332, 3, 3725}, {81, 333, 3, 3725}, {82, 334, 1, 3725}, {64, 323, 1, 3726}, {68, 325, 3, 3726}, {69, 326, 3, 3726}, {73, 328, 3, 3726}, {74, 329, 6, 3726}, {75, 330, 3, 3726}, {79, 332, 1, 3726}, {80, 333, 3, 3726}, {81, 334, 3, 3726}, {82, 335, 1, 3726}, {64, 324, 1, 3727}, {68, 327, 3, 3727}, {69, 328, 3, 3727}, {73, 331, 3, 3727}, {74, 332, 6, 3727}, {75, 333, 3, 3727}, {79, 336, 1, 3727}, {80, 337, 3, 3727}, {81, 338, 3, 3727}, {82, 339, 1, 3727}, {64, 325, 1, 3728}, {68, 328, 3, 3728}, {69, 329, 3, 3728}, {73, 332, 3, 3728}, {74, 333, 6, 3728}, {75, 334, 3, 3728}, {79, 337, 1, 3728}, {80, 338, 3, 3728}, {81, 339, 3, 3728}, {82, 340, 1, 3728}, {64, 326, 1, 3729}, {68, 329, 3, 3729}, {69, 330, 3, 3729}, {73, 333, 3, 3729}, {74, 334, 6, 3729}, {75, 335, 3, 3729}, {79, 338, 1, 3729}, {80, 339, 3, 3729}, {81, 340, 3, 3729}, {82, 341, 1, 3729}, {65, 321, 1, 3730}, {69, 322, 3, 3730}, {70, 323, 3, 3730}, {74, 324, 3, 3730}, {75, 325, 6, 3730}, {76, 326, 3, 3730}, {80, 327, 1, 3730}, {81, 328, 3, 3730}, {82, 329, 3, 3730}, {83, 330, 1, 3730}, {65, 322, 1, 3731}, {69, 324, 3, 3731}, {70, 325, 3, 3731}, {74, 327, 3, 3731}, {75, 328, 6, 3731}, {76, 329, 3, 3731}, {80, 331, 1, 3731}, {81, 332, 3, 3731}, {82, 333, 3, 3731}, {83, 334, 1, 3731}, {65, 323, 1, 3732}, {69, 325, 3, 3732}, {70, 326, 3, 3732}, {74, 328, 3, 3732}, {75, 329, 6, 3732}, {76, 330, 3, 3732}, {80, 332, 1, 3732}, {81, 333, 3, 3732}, {82, 334, 3, 3732}, {83, 335, 1, 3732}, {65, 324, 1, 3733}, {69, 327, 3, 3733}, {70, 328, 3, 3733}, {74, 331, 3, 3733}, {75, 332, 6, 3733}, {76, 333, 3, 3733}, {80, 336, 1, 3733}, {81, 337, 3, 3733}, {82, 338, 3, 3733}, {83, 339, 1, 3733}, {65, 325, 1, 3734}, {69, 328, 3, 3734}, {70, 329, 3, 3734}, {74, 332, 3, 3734}, {75, 333, 6, 3734}, {76, 334, 3, 3734}, {80, 337, 1, 3734}, {81, 338, 3, 3734}, {82, 339, 3, 3734}, {83, 340, 1, 3734}, {65, 326, 1, 3735}, {69, 329, 3, 3735}, {70, 330, 3, 3735}, {74, 333, 3, 3735}, {75, 334, 6, 3735}, {76, 335, 3, 3735}, {80, 338, 1, 3735}, {81, 339, 3, 3735}, {82, 340, 3, 3735}, {83, 341, 1, 3735}, {56, 306, 1, 3736}, {57, 307, 4, 3736}, {58, 308, 4, 3736}, {59, 309, 6, 3736}, {60, 310, 12, 3736}, {61, 311, 6, 3736}, {62, 312, 4, 3736}, {63, 313, 12, 3736}, {64, 314, 12, 3736}, {65, 315, 4, 3736}, {66, 316, 1, 3736}, {67, 317, 4, 3736}, {68, 318, 6, 3736}, {69, 319, 4, 3736}, {70, 320, 1, 3736}, {57, 306, 1, 3737}, {59, 307, 4, 3737}, {60, 308, 4, 3737}, {62, 309, 6, 3737}, {63, 310, 12, 3737}, {64, 311, 6, 3737}, {66, 312, 4, 3737}, {67, 313, 12, 3737}, {68, 314, 12, 3737}, {69, 315, 4, 3737}, {71, 316, 1, 3737}, {72, 317, 4, 3737}, {73, 318, 6, 3737}, {74, 319, 4, 3737}, {75, 320, 1, 3737}, {58, 306, 1, 3738}, {60, 307, 4, 3738}, {61, 308, 4, 3738}, {63, 309, 6, 3738}, {64, 310, 12, 3738}, {65, 311, 6, 3738}, {67, 312, 4, 3738}, {68, 313, 12, 3738}, {69, 314, 12, 3738}, {70, 315, 4, 3738}, {72, 316, 1, 3738}, {73, 317, 4, 3738}, {74, 318, 6, 3738}, {75, 319, 4, 3738}, {76, 320, 1, 3738}, {59, 306, 1, 3739}, {62, 307, 4, 3739}, {63, 308, 4, 3739}, {66, 309, 6, 3739}, {67, 310, 12, 3739}, {68, 311, 6, 3739}, {71, 312, 4, 3739}, {72, 313, 12, 3739}, {73, 314, 12, 3739}, {74, 315, 4, 3739}, {77, 316, 1, 3739}, {78, 317, 4, 3739}, {79, 318, 6, 3739}, {80, 319, 4, 3739}, {81, 320, 1, 3739}, {60, 306, 1, 3740}, {63, 307, 4, 3740}, {64, 308, 4, 3740}, {67, 309, 6, 3740}, {68, 310, 12, 3740}, {69, 311, 6, 3740}, {72, 312, 4, 3740}, {73, 313, 12, 3740}, {74, 314, 12, 3740}, {75, 315, 4, 3740}, {78, 316, 1, 3740}, {79, 317, 4, 3740}, {80, 318, 6, 3740}, {81, 319, 4, 3740}, {82, 320, 1, 3740}, {61, 306, 1, 3741}, {64, 307, 4, 3741}, {65, 308, 4, 3741}, {68, 309, 6, 3741}, {69, 310, 12, 3741}, {70, 311, 6, 3741}, {73, 312, 4, 3741}, {74, 313, 12, 3741}, {75, 314, 12, 3741}, {76, 315, 4, 3741}, {79, 316, 1, 3741}, {80, 317, 4, 3741}, {81, 318, 6, 3741}, {82, 319, 4, 3741}, {83, 320, 1, 3741}, {56, 321, 1, 3742}, {57, 322, 4, 3742}, {58, 323, 4, 3742}, {59, 324, 6, 3742}, {60, 325, 12, 3742}, {61, 326, 6, 3742}, {62, 327, 4, 3742}, {63, 328, 12, 3742}, {64, 329, 12, 3742}, {65, 330, 4, 3742}, {66, 331, 1, 3742}, {67, 332, 4, 3742}, {68, 333, 6, 3742}, {69, 334, 4, 3742}, {70, 335, 1, 3742}, {56, 322, 1, 3743}, {57, 324, 4, 3743}, {58, 325, 4, 3743}, {59, 327, 6, 3743}, {60, 328, 12, 3743}, {61, 329, 6, 3743}, {62, 331, 4, 3743}, {63, 332, 12, 3743}, {64, 333, 12, 3743}, {65, 334, 4, 3743}, {66, 336, 1, 3743}, {67, 337, 4, 3743}, {68, 338, 6, 3743}, {69, 339, 4,

3743}, {70, 340, 1, 3743}, {56, 323, 1, 3744}, {57, 325, 4, 3744}, {58, 326, 4, 3744},
{59, 328, 6, 3744}, {60, 329, 12, 3744}, {61, 330, 6, 3744}, {62, 332, 4, 3744}, {63, 333,
12, 3744}, {64, 334, 12, 3744}, {65, 335, 4, 3744}, {66, 337, 1, 3744}, {67, 338, 4,
3744}, {68, 339, 6, 3744}, {69, 340, 4, 3744}, {70, 341, 1, 3744}, {57, 321, 1, 3745},
{59, 322, 4, 3745}, {60, 323, 4, 3745}, {62, 324, 6, 3745}, {63, 325, 12, 3745}, {64, 326,
6, 3745}, {66, 327, 4, 3745}, {67, 328, 12, 3745}, {68, 329, 12, 3745}, {69, 330, 4,
3745}, {71, 331, 1, 3745}, {72, 332, 4, 3745}, {73, 333, 6, 3745}, {74, 334, 4, 3745},
{75, 335, 1, 3745}, {57, 322, 1, 3746}, {59, 324, 4, 3746}, {60, 325, 4, 3746}, {62, 327,
6, 3746}, {63, 328, 12, 3746}, {64, 329, 6, 3746}, {66, 331, 4, 3746}, {67, 332, 12,
3746}, {68, 333, 12, 3746}, {69, 334, 4, 3746}, {71, 336, 1, 3746}, {72, 337, 4, 3746},
{73, 338, 6, 3746}, {74, 339, 4, 3746}, {75, 340, 1, 3746}, {57, 323, 1, 3747}, {59, 325,
4, 3747}, {60, 326, 4, 3747}, {62, 328, 6, 3747}, {63, 329, 12, 3747}, {64, 330, 6, 3747},
{66, 332, 4, 3747}, {67, 333, 12, 3747}, {68, 334, 12, 3747}, {69, 335, 4, 3747}, {71,
337, 1, 3747}, {72, 338, 4, 3747}, {73, 339, 6, 3747}, {74, 340, 4, 3747}, {75, 341, 1,
3747}, {58, 321, 1, 3748}, {60, 322, 4, 3748}, {61, 323, 4, 3748}, {63, 324, 6, 3748},
{64, 325, 12, 3748}, {65, 326, 6, 3748}, {67, 327, 4, 3748}, {68, 328, 12, 3748}, {69,
329, 12, 3748}, {70, 330, 4, 3748}, {72, 331, 1, 3748}, {73, 332, 4, 3748}, {74, 333, 6,
3748}, {75, 334, 4, 3748}, {76, 335, 1, 3748}, {58, 322, 1, 3749}, {60, 324, 4, 3749},
{61, 325, 4, 3749}, {63, 327, 6, 3749}, {64, 328, 12, 3749}, {65, 329, 6, 3749}, {67, 331,
4, 3749}, {68, 332, 12, 3749}, {69, 333, 12, 3749}, {70, 334, 4, 3749}, {72, 336, 1,
3749}, {73, 337, 4, 3749}, {74, 338, 6, 3749}, {75, 339, 4, 3749}, {76, 340, 1, 3749},
{58, 323, 1, 3750}, {60, 325, 4, 3750}, {61, 326, 4, 3750}, {63, 328, 6, 3750}, {64, 329,
12, 3750}, {65, 330, 6, 3750}, {67, 332, 4, 3750}, {68, 333, 12, 3750}, {69, 334, 12,
3750}, {70, 335, 4, 3750}, {72, 337, 1, 3750}, {73, 338, 4, 3750}, {74, 339, 6, 3750},
{75, 340, 4, 3750}, {76, 341, 1, 3750}, {59, 321, 1, 3751}, {62, 322, 4, 3751}, {63, 323,
4, 3751}, {66, 324, 6, 3751}, {67, 325, 12, 3751}, {68, 326, 6, 3751}, {71, 327, 4, 3751},
{72, 328, 12, 3751}, {73, 329, 12, 3751}, {74, 330, 4, 3751}, {77, 331, 1, 3751}, {78,
332, 4, 3751}, {79, 333, 6, 3751}, {80, 334, 4, 3751}, {81, 335, 1, 3751}, {59, 322, 1,
3752}, {62, 324, 4, 3752}, {63, 325, 4, 3752}, {66, 327, 6, 3752}, {67, 328, 12, 3752},
{68, 329, 6, 3752}, {71, 331, 4, 3752}, {72, 332, 12, 3752}, {73, 333, 12, 3752}, {74,
334, 4, 3752}, {77, 336, 1, 3752}, {78, 337, 4, 3752}, {79, 338, 6, 3752}, {80, 339, 4,
3752}, {81, 340, 1, 3752}, {59, 323, 1, 3753}, {62, 325, 4, 3753}, {63, 326, 4, 3753},
{66, 328, 6, 3753}, {67, 329, 12, 3753}, {68, 330, 6, 3753}, {71, 332, 4, 3753}, {72, 333,
12, 3753}, {73, 334, 12, 3753}, {74, 335, 4, 3753}, {77, 337, 1, 3753}, {78, 338, 4,
3753}, {79, 339, 6, 3753}, {80, 340, 4, 3753}, {81, 341, 1, 3753}, {60, 321, 1, 3754},
{63, 322, 4, 3754}, {64, 323, 4, 3754}, {67, 324, 6, 3754}, {68, 325, 12, 3754}, {69, 326,
6, 3754}, {72, 327, 4, 3754}, {73, 328, 12, 3754}, {74, 329, 12, 3754}, {75, 330, 4,
3754}, {78, 331, 1, 3754}, {79, 332, 4, 3754}, {80, 333, 6, 3754}, {81, 334, 4, 3754},
{82, 335, 1, 3754}, {60, 322, 1, 3755}, {63, 324, 4, 3755}, {64, 325, 4, 3755}, {67, 327,
6, 3755}, {68, 328, 12, 3755}, {69, 329, 6, 3755}, {72, 331, 4, 3755}, {73, 332, 12,
3755}, {74, 333, 12, 3755}, {75, 334, 4, 3755}, {78, 336, 1, 3755}, {79, 337, 4, 3755},
{80, 338, 6, 3755}, {81, 339, 4, 3755}, {82, 340, 1, 3755}, {60, 323, 1, 3756}, {63, 325,
4, 3756}, {64, 326, 4, 3756}, {67, 328, 6, 3756}, {68, 329, 12, 3756}, {69, 330, 6, 3756},
{72, 332, 4, 3756}, {73, 333, 12, 3756}, {74, 334, 12, 3756}, {75, 335, 4, 3756}, {78,
337, 1, 3756}, {79, 338, 4, 3756}, {80, 339, 6, 3756}, {81, 340, 4, 3756}, {82, 341, 1,
3756}, {61, 321, 1, 3757}, {64, 322, 4, 3757}, {65, 323, 4, 3757}, {68, 324, 6, 3757},
{69, 325, 12, 3757}, {70, 326, 6, 3757}, {73, 327, 4, 3757}, {74, 328, 12, 3757}, {75,
329, 12, 3757}, {76, 330, 4, 3757}, {79, 331, 1, 3757}, {80, 332, 4, 3757}, {81, 333, 6,
3757}, {82, 334, 4, 3757}, {83, 335, 1, 3757}, {61, 322, 1, 3758}, {64, 324, 4, 3758},
{65, 325, 4, 3758}, {68, 327, 6, 3758}, {69, 328, 12, 3758}, {70, 329, 6, 3758}, {73, 331,
4, 3758}, {74, 332, 12, 3758}, {75, 333, 12, 3758}, {76, 334, 4, 3758}, {79, 336, 1,
3758}, {80, 337, 4, 3758}, {81, 338, 6, 3758}, {82, 339, 4, 3758}, {83, 340, 1, 3758},
{61, 323, 1, 3759}, {64, 325, 4, 3759}, {65, 326, 4, 3759}, {68, 328, 6, 3759}, {69, 329,
12, 3759}, {70, 330, 6, 3759}, {73, 332, 4, 3759}, {74, 333, 12, 3759}, {75, 334, 12,
3759}, {76, 335, 4, 3759}, {79, 337, 1, 3759}, {80, 338, 4, 3759}, {81, 339, 6, 3759},
{82, 340, 4, 3759}, {83, 341, 1, 3759}, {56, 342, 1, 3760}, {57, 343, 4, 3760}, {58, 344,
4, 3760}, {59, 345, 6, 3760}, {60, 346, 12, 3760}, {61, 347, 6, 3760}, {62, 348, 4, 3760},
{63, 349, 12, 3760}, {64, 350, 12, 3760}, {65, 351, 4, 3760}, {66, 352, 1, 3760}, {67,
353, 4, 3760}, {68, 354, 6, 3760}, {69, 355, 4, 3760}, {70, 356, 1, 3760}, {56, 343, 1,
3761}, {57, 345, 4, 3761}, {58, 346, 4, 3761}, {59, 348, 6, 3761}, {60, 349, 12, 3761},
{61, 350, 6, 3761}, {62, 352, 4, 3761}, {63, 353, 12, 3761}, {64, 354, 12, 3761}, {65,
355, 4, 3761}, {66, 357, 1, 3761}, {67, 358, 4, 3761}, {68, 359, 6, 3761}, {69, 360, 4,
3761}, {70, 361, 1, 3761}, {56, 344, 1, 3762}, {57, 346, 4, 3762}, {58, 347, 4, 3762},
{59, 349, 6, 3762}, {60, 350, 12, 3762}, {61, 351, 6, 3762}, {62, 353, 4, 3762}, {63, 354,
12, 3762}, {64, 355, 12, 3762}, {65, 356, 4, 3762}, {66, 358, 1, 3762}, {67, 359, 4,
3762}, {68, 360, 6, 3762}, {69, 361, 4, 3762}, {70, 362, 1, 3762}, {56, 345, 1, 3763},
{57, 348, 4, 3763}, {58, 349, 4, 3763}, {59, 352, 6, 3763}, {60, 353, 12, 3763}, {61, 354,
6, 3763}, {62, 357, 4, 3763}, {63, 358, 12, 3763}, {64, 359, 12, 3763}, {65, 360, 4,
3763}, {66, 363, 1, 3763}, {67, 364, 4, 3763}, {68, 365, 6, 3763}, {69, 366, 4, 3763},

{70, 367, 1, 3763}, {56, 346, 1, 3764}, {57, 349, 4, 3764}, {58, 350, 4, 3764}, {59, 353, 6, 3764}, {60, 354, 12, 3764}, {61, 355, 6, 3764}, {62, 358, 4, 3764}, {63, 359, 12, 3764}, {64, 360, 12, 3764}, {65, 361, 4, 3764}, {66, 364, 1, 3764}, {67, 365, 4, 3764}, {68, 366, 6, 3764}, {69, 367, 4, 3764}, {70, 368, 1, 3764}, {56, 347, 1, 3765}, {57, 350, 4, 3765}, {58, 351, 4, 3765}, {59, 354, 6, 3765}, {60, 355, 12, 3765}, {61, 356, 6, 3765}, {62, 359, 4, 3765}, {63, 360, 12, 3765}, {64, 361, 12, 3765}, {65, 362, 4, 3765}, {66, 365, 1, 3765}, {67, 366, 4, 3765}, {68, 367, 6, 3765}, {69, 368, 4, 3765}, {70, 369, 1, 3765}, {57, 342, 1, 3766}, {59, 343, 4, 3766}, {60, 344, 4, 3766}, {62, 345, 6, 3766}, {63, 346, 12, 3766}, {64, 347, 6, 3766}, {66, 348, 4, 3766}, {67, 349, 12, 3766}, {68, 350, 12, 3766}, {69, 351, 4, 3766}, {71, 352, 1, 3766}, {72, 353, 4, 3766}, {73, 354, 6, 3766}, {74, 355, 4, 3766}, {75, 356, 1, 3766}, {57, 343, 1, 3767}, {59, 345, 4, 3767}, {60, 346, 4, 3767}, {62, 348, 6, 3767}, {63, 349, 12, 3767}, {64, 350, 6, 3767}, {66, 352, 4, 3767}, {67, 353, 12, 3767}, {68, 354, 12, 3767}, {69, 355, 4, 3767}, {71, 357, 1, 3767}, {72, 358, 4, 3767}, {73, 359, 6, 3767}, {74, 360, 4, 3767}, {75, 361, 1, 3767}, {57, 344, 1, 3768}, {59, 346, 4, 3768}, {60, 347, 4, 3768}, {62, 349, 6, 3768}, {63, 350, 12, 3768}, {64, 351, 6, 3768}, {66, 353, 4, 3768}, {67, 354, 12, 3768}, {68, 355, 12, 3768}, {69, 356, 4, 3768}, {71, 358, 1, 3768}, {72, 359, 4, 3768}, {73, 360, 6, 3768}, {74, 361, 4, 3768}, {75, 362, 1, 3768}, {57, 345, 1, 3769}, {59, 348, 4, 3769}, {60, 349, 4, 3769}, {62, 352, 6, 3769}, {63, 353, 12, 3769}, {64, 354, 6, 3769}, {66, 357, 4, 3769}, {67, 358, 12, 3769}, {68, 359, 12, 3769}, {69, 360, 4, 3769}, {71, 363, 1, 3769}, {72, 364, 4, 3769}, {73, 365, 6, 3769}, {74, 366, 4, 3769}, {75, 367, 1, 3769}, {57, 346, 1, 3770}, {59, 349, 4, 3770}, {60, 350, 4, 3770}, {62, 353, 6, 3770}, {63, 354, 12, 3770}, {64, 355, 6, 3770}, {66, 358, 4, 3770}, {67, 359, 12, 3770}, {68, 360, 12, 3770}, {69, 361, 4, 3770}, {71, 364, 1, 3770}, {72, 365, 4, 3770}, {73, 366, 6, 3770}, {74, 367, 4, 3770}, {75, 368, 1, 3770}, {57, 347, 1, 3771}, {59, 350, 4, 3771}, {60, 351, 4, 3771}, {62, 354, 6, 3771}, {63, 355, 12, 3771}, {64, 356, 6, 3771}, {66, 359, 4, 3771}, {67, 360, 12, 3771}, {68, 361, 12, 3771}, {69, 362, 4, 3771}, {71, 365, 1, 3771}, {72, 366, 4, 3771}, {73, 367, 6, 3771}, {74, 368, 4, 3771}, {75, 369, 1, 3771}, {58, 342, 1, 3772}, {60, 343, 4, 3772}, {61, 344, 4, 3772}, {63, 345, 6, 3772}, {64, 346, 12, 3772}, {65, 347, 6, 3772}, {67, 348, 4, 3772}, {68, 349, 12, 3772}, {69, 350, 12, 3772}, {70, 351, 4, 3772}, {72, 352, 1, 3772}, {73, 353, 4, 3772}, {74, 354, 6, 3772}, {75, 355, 4, 3772}, {76, 356, 1, 3772}, {58, 343, 1, 3773}, {60, 345, 4, 3773}, {61, 346, 4, 3773}, {63, 348, 6, 3773}, {64, 349, 12, 3773}, {65, 350, 6, 3773}, {67, 352, 4, 3773}, {68, 353, 12, 3773}, {69, 354, 12, 3773}, {70, 355, 4, 3773}, {72, 357, 1, 3773}, {73, 358, 4, 3773}, {74, 359, 6, 3773}, {75, 360, 4, 3773}, {76, 361, 1, 3773}, {58, 344, 1, 3774}, {60, 346, 4, 3774}, {61, 347, 4, 3774}, {63, 349, 6, 3774}, {64, 350, 12, 3774}, {65, 351, 6, 3774}, {67, 353, 4, 3774}, {68, 354, 12, 3774}, {69, 355, 12, 3774}, {70, 356, 4, 3774}, {72, 358, 1, 3774}, {73, 359, 4, 3774}, {74, 360, 6, 3774}, {75, 361, 4, 3774}, {76, 362, 1, 3774}, {58, 345, 1, 3775}, {60, 348, 4, 3775}, {61, 349, 4, 3775}, {63, 352, 6, 3775}, {64, 353, 12, 3775}, {65, 354, 6, 3775}, {67, 357, 4, 3775}, {68, 358, 12, 3775}, {69, 359, 12, 3775}, {70, 360, 4, 3775}, {72, 363, 1, 3775}, {73, 364, 4, 3775}, {74, 365, 6, 3775}, {75, 366, 4, 3775}, {76, 367, 1, 3775}, {58, 346, 1, 3776}, {60, 349, 4, 3776}, {61, 350, 4, 3776}, {63, 353, 6, 3776}, {64, 354, 12, 3776}, {65, 355, 6, 3776}, {67, 358, 4, 3776}, {68, 359, 12, 3776}, {69, 360, 12, 3776}, {70, 361, 4, 3776}, {72, 364, 1, 3776}, {73, 365, 4, 3776}, {74, 366, 6, 3776}, {75, 367, 4, 3776}, {76, 368, 1, 3776}, {58, 347, 1, 3777}, {60, 350, 4, 3777}, {61, 351, 4, 3777}, {63, 354, 6, 3777}, {64, 355, 12, 3777}, {65, 356, 6, 3777}, {67, 359, 4, 3777}, {68, 360, 12, 3777}, {69, 361, 12, 3777}, {70, 362, 4, 3777}, {72, 365, 1, 3777}, {73, 366, 4, 3777}, {74, 367, 6, 3777}, {75, 368, 4, 3777}, {76, 369, 1, 3777}, {59, 342, 1, 3778}, {62, 343, 4, 3778}, {63, 344, 4, 3778}, {66, 345, 6, 3778}, {67, 346, 12, 3778}, {68, 347, 6, 3778}, {71, 348, 4, 3778}, {72, 349, 12, 3778}, {73, 350, 12, 3778}, {74, 351, 4, 3778}, {77, 352, 1, 3778}, {78, 353, 4, 3778}, {79, 354, 6, 3778}, {80, 355, 4, 3778}, {81, 356, 1, 3778}, {59, 343, 1, 3779}, {62, 345, 4, 3779}, {63, 346, 4, 3779}, {66, 348, 6, 3779}, {67, 349, 12, 3779}, {68, 350, 6, 3779}, {71, 352, 4, 3779}, {72, 353, 12, 3779}, {73, 354, 12, 3779}, {74, 355, 4, 3779}, {77, 357, 1, 3779}, {78, 358, 4, 3779}, {79, 359, 6, 3779}, {80, 360, 4, 3779}, {81, 361, 1, 3779}, {59, 344, 1, 3780}, {62, 346, 4, 3780}, {63, 347, 4, 3780}, {66, 349, 6, 3780}, {67, 350, 12, 3780}, {68, 351, 6, 3780}, {71, 353, 4, 3780}, {72, 354, 12, 3780}, {73, 355, 12, 3780}, {74, 356, 4, 3780}, {77, 358, 1, 3780}, {78, 359, 4, 3780}, {79, 360, 6, 3780}, {80, 361, 4, 3780}, {81, 362, 1, 3780}, {59, 345, 1, 3781}, {62, 348, 4, 3781}, {63, 349, 4, 3781}, {66, 352, 6, 3781}, {67, 353, 12, 3781}, {68, 354, 6, 3781}, {71, 357, 4, 3781}, {72, 358, 12, 3781}, {73, 359, 12, 3781}, {74, 360, 4, 3781}, {77, 363, 1, 3781}, {78, 364, 4, 3781}, {79, 365, 6, 3781}, {80, 366, 4, 3781}, {81, 367, 1, 3781}, {59, 346, 1, 3782}, {62, 349, 4, 3782}, {63, 350, 4, 3782}, {66, 353, 6, 3782}, {67, 354, 12, 3782}, {68, 355, 6, 3782}, {71, 358, 4, 3782}, {72, 359, 12, 3782}, {73, 360, 12, 3782}, {74, 361, 4, 3782}, {77, 364, 1, 3782}, {78, 365, 4, 3782}, {79, 366, 6, 3782}, {80, 367, 4, 3782}, {81, 368, 1, 3782}, {59, 347, 1, 3783}, {62, 350, 4, 3783}, {63, 351, 4, 3783}, {66, 354, 6, 3783}, {67, 355, 12, 3783}, {68, 356, 6, 3783}, {71, 359, 4, 3783}, {72, 360, 12, 3783}, {73, 361, 12, 3783}, {74, 362, 4, 3783}, {77, 365, 1, 3783}, {78, 366, 4, 3783}, {79, 367, 6, 3783}, {80, 368, 4, 3783}, {81, 369, 1,

3783}, {60, 342, 1, 3784}, {63, 343, 4, 3784}, {64, 344, 4, 3784}, {67, 345, 6, 3784}, {68, 346, 12, 3784}, {69, 347, 6, 3784}, {72, 348, 4, 3784}, {73, 349, 12, 3784}, {74, 350, 12, 3784}, {75, 351, 4, 3784}, {78, 352, 1, 3784}, {79, 353, 4, 3784}, {80, 354, 6, 3784}, {81, 355, 4, 3784}, {82, 356, 1, 3784}, {60, 343, 1, 3785}, {63, 345, 4, 3785}, {64, 346, 4, 3785}, {67, 348, 6, 3785}, {68, 349, 12, 3785}, {69, 350, 6, 3785}, {72, 352, 4, 3785}, {73, 353, 12, 3785}, {74, 354, 12, 3785}, {75, 355, 4, 3785}, {78, 357, 1, 3785}, {79, 358, 4, 3785}, {80, 359, 6, 3785}, {81, 360, 4, 3785}, {82, 361, 1, 3785}, {60, 344, 1, 3786}, {63, 346, 4, 3786}, {64, 347, 4, 3786}, {67, 349, 6, 3786}, {68, 350, 12, 3786}, {69, 351, 6, 3786}, {72, 353, 4, 3786}, {73, 354, 12, 3786}, {74, 355, 12, 3786}, {75, 356, 4, 3786}, {78, 358, 1, 3786}, {79, 359, 4, 3786}, {80, 360, 6, 3786}, {81, 361, 4, 3786}, {82, 362, 1, 3786}, {60, 345, 1, 3787}, {63, 348, 4, 3787}, {64, 349, 4, 3787}, {67, 352, 6, 3787}, {68, 353, 12, 3787}, {69, 354, 6, 3787}, {72, 357, 4, 3787}, {73, 358, 12, 3787}, {74, 359, 12, 3787}, {75, 360, 4, 3787}, {78, 363, 1, 3787}, {79, 364, 4, 3787}, {80, 365, 6, 3787}, {81, 366, 4, 3787}, {82, 367, 1, 3787}, {60, 346, 1, 3788}, {63, 349, 4, 3788}, {64, 350, 4, 3788}, {67, 353, 6, 3788}, {68, 354, 12, 3788}, {69, 355, 6, 3788}, {72, 358, 4, 3788}, {73, 359, 12, 3788}, {74, 360, 12, 3788}, {75, 361, 4, 3788}, {78, 364, 1, 3788}, {79, 365, 4, 3788}, {80, 366, 6, 3788}, {81, 367, 4, 3788}, {82, 368, 1, 3788}, {60, 347, 1, 3789}, {63, 350, 4, 3789}, {64, 351, 4, 3789}, {67, 354, 6, 3789}, {68, 355, 12, 3789}, {69, 356, 6, 3789}, {72, 359, 4, 3789}, {73, 360, 12, 3789}, {74, 361, 12, 3789}, {75, 362, 4, 3789}, {78, 365, 1, 3789}, {79, 366, 4, 3789}, {80, 367, 6, 3789}, {81, 368, 4, 3789}, {82, 369, 1, 3789}, {61, 342, 1, 3790}, {64, 343, 4, 3790}, {65, 344, 4, 3790}, {68, 345, 6, 3790}, {69, 346, 12, 3790}, {70, 347, 6, 3790}, {73, 348, 4, 3790}, {74, 349, 12, 3790}, {75, 350, 12, 3790}, {76, 351, 4, 3790}, {79, 352, 1, 3790}, {80, 353, 4, 3790}, {81, 354, 6, 3790}, {82, 355, 4, 3790}, {83, 356, 1, 3790}, {61, 343, 1, 3791}, {64, 345, 4, 3791}, {65, 346, 4, 3791}, {68, 348, 6, 3791}, {69, 349, 12, 3791}, {70, 350, 6, 3791}, {73, 352, 4, 3791}, {74, 353, 12, 3791}, {75, 354, 12, 3791}, {76, 355, 4, 3791}, {79, 357, 1, 3791}, {80, 358, 4, 3791}, {81, 359, 6, 3791}, {82, 360, 4, 3791}, {83, 361, 1, 3791}, {61, 344, 1, 3792}, {64, 346, 4, 3792}, {65, 347, 4, 3792}, {68, 349, 6, 3792}, {69, 350, 12, 3792}, {70, 351, 6, 3792}, {73, 353, 4, 3792}, {74, 354, 12, 3792}, {75, 355, 12, 3792}, {76, 356, 4, 3792}, {79, 358, 1, 3792}, {80, 359, 4, 3792}, {81, 360, 6, 3792}, {82, 361, 4, 3792}, {83, 362, 1, 3792}, {61, 345, 1, 3793}, {64, 348, 4, 3793}, {65, 349, 4, 3793}, {68, 352, 6, 3793}, {69, 353, 12, 3793}, {70, 354, 6, 3793}, {73, 357, 4, 3793}, {74, 358, 12, 3793}, {75, 359, 12, 3793}, {76, 360, 4, 3793}, {79, 363, 1, 3793}, {80, 364, 4, 3793}, {81, 365, 6, 3793}, {82, 366, 4, 3793}, {83, 367, 1, 3793}, {61, 346, 1, 3794}, {64, 349, 4, 3794}, {65, 350, 4, 3794}, {68, 353, 6, 3794}, {69, 354, 12, 3794}, {70, 355, 6, 3794}, {73, 358, 4, 3794}, {74, 359, 12, 3794}, {75, 360, 12, 3794}, {76, 361, 4, 3794}, {79, 364, 1, 3794}, {80, 365, 4, 3794}, {81, 366, 6, 3794}, {82, 367, 4, 3794}, {83, 368, 1, 3794}, {61, 347, 1, 3795}, {64, 350, 4, 3795}, {65, 351, 4, 3795}, {68, 354, 6, 3795}, {69, 355, 12, 3795}, {70, 356, 6, 3795}, {73, 359, 4, 3795}, {74, 360, 12, 3795}, {75, 361, 12, 3795}, {76, 362, 4, 3795}, {79, 365, 1, 3795}, {80, 366, 4, 3795}, {81, 367, 6, 3795}, {82, 368, 4, 3795}, {83, 369, 1, 3795}, {56, 321, 1, 3796}, {57, 322, 5, 3796}, {58, 323, 5, 3796}, {59, 324, 10, 3796}, {60, 325, 20, 3796}, {61, 326, 10, 3796}, {62, 327, 10, 3796}, {63, 328, 30, 3796}, {64, 329, 30, 3796}, {65, 330, 10, 3796}, {66, 331, 5, 3796}, {67, 332, 20, 3796}, {68, 333, 30, 3796}, {69, 334, 20, 3796}, {70, 335, 5, 3796}, {71, 336, 1, 3796}, {72, 337, 5, 3796}, {73, 338, 10, 3796}, {74, 339, 10, 3796}, {75, 340, 5, 3796}, {76, 341, 1, 3796}, {57, 321, 1, 3797}, {59, 322, 5, 3797}, {60, 323, 5, 3797}, {62, 324, 10, 3797}, {63, 325, 20, 3797}, {64, 326, 10, 3797}, {66, 327, 10, 3797}, {67, 328, 30, 3797}, {68, 329, 30, 3797}, {69, 330, 10, 3797}, {71, 331, 5, 3797}, {72, 332, 20, 3797}, {73, 333, 30, 3797}, {74, 334, 20, 3797}, {75, 335, 5, 3797}, {77, 336, 1, 3797}, {78, 337, 5, 3797}, {79, 338, 10, 3797}, {80, 339, 10, 3797}, {81, 340, 5, 3797}, {82, 341, 1, 3797}, {58, 321, 1, 3798}, {60, 322, 5, 3798}, {61, 323, 5, 3798}, {63, 324, 10, 3798}, {64, 325, 20, 3798}, {65, 326, 10, 3798}, {67, 327, 10, 3798}, {68, 328, 30, 3798}, {69, 329, 30, 3798}, {70, 330, 10, 3798}, {72, 331, 5, 3798}, {73, 332, 20, 3798}, {74, 333, 30, 3798}, {75, 334, 20, 3798}, {76, 335, 5, 3798}, {78, 336, 1, 3798}, {79, 337, 5, 3798}, {80, 338, 10, 3798}, {81, 339, 10, 3798}, {82, 340, 5, 3798}, {83, 341, 1, 3798}, {56, 342, 1, 3799}, {57, 343, 5, 3799}, {58, 344, 5, 3799}, {59, 345, 10, 3799}, {60, 346, 20, 3799}, {61, 347, 10, 3799}, {62, 348, 10, 3799}, {63, 349, 30, 3799}, {64, 350, 30, 3799}, {65, 351, 10, 3799}, {66, 352, 5, 3799}, {67, 353, 20, 3799}, {68, 354, 30, 3799}, {69, 355, 20, 3799}, {70, 356, 5, 3799}, {71, 357, 1, 3799}, {72, 358, 5, 3799}, {73, 359, 10, 3799}, {74, 360, 10, 3799}, {75, 361, 5, 3799}, {76, 362, 1, 3799}, {56, 343, 1, 3800}, {57, 345, 5, 3800}, {58, 346, 5, 3800}, {59, 348, 10, 3800}, {60, 349, 20, 3800}, {61, 350, 10, 3800}, {62, 352, 10, 3800}, {63, 353, 30, 3800}, {64, 354, 30, 3800}, {65, 355, 10, 3800}, {66, 357, 5, 3800}, {67, 358, 20, 3800}, {68, 359, 30, 3800}, {69, 360, 20, 3800}, {70, 361, 5, 3800}, {71, 363, 1, 3800}, {72, 364, 5, 3800}, {73, 365, 10, 3800}, {74, 366, 10, 3800}, {75, 367, 5, 3800}, {76, 368, 1, 3800}, {56, 344, 1, 3801}, {57, 346, 5, 3801}, {58, 347, 5, 3801}, {59, 349, 10, 3801}, {60, 350, 20, 3801}, {61, 351, 10, 3801}, {62, 353, 10, 3801}, {63, 354, 30, 3801}, {64, 355, 30, 3801}, {65, 356, 10, 3801}, {66, 358, 5, 3801}, {67, 359, 20, 3801}, {68, 360, 30, 3801}, {69, 361,

20, 3801}, {70, 362, 5, 3801}, {71, 364, 1, 3801}, {72, 365, 5, 3801}, {73, 366, 10, 3801}, {74, 367, 10, 3801}, {75, 368, 5, 3801}, {76, 369, 1, 3801}, {57, 342, 1, 3802}, {59, 343, 5, 3802}, {60, 344, 5, 3802}, {62, 345, 10, 3802}, {63, 346, 20, 3802}, {64, 347, 10, 3802}, {66, 348, 10, 3802}, {67, 349, 30, 3802}, {68, 350, 30, 3802}, {69, 351, 10, 3802}, {71, 352, 5, 3802}, {72, 353, 20, 3802}, {73, 354, 30, 3802}, {74, 355, 20, 3802}, {75, 356, 5, 3802}, {77, 357, 1, 3802}, {78, 358, 5, 3802}, {79, 359, 10, 3802}, {80, 360, 10, 3802}, {81, 361, 5, 3802}, {82, 362, 1, 3802}, {57, 343, 1, 3803}, {59, 345, 5, 3803}, {60, 346, 5, 3803}, {62, 348, 10, 3803}, {63, 349, 20, 3803}, {64, 350, 10, 3803}, {66, 352, 10, 3803}, {67, 353, 30, 3803}, {68, 354, 30, 3803}, {69, 355, 10, 3803}, {71, 357, 5, 3803}, {72, 358, 20, 3803}, {73, 359, 30, 3803}, {74, 360, 20, 3803}, {75, 361, 5, 3803}, {77, 363, 1, 3803}, {78, 364, 5, 3803}, {79, 365, 10, 3803}, {80, 366, 10, 3803}, {81, 367, 5, 3803}, {82, 368, 1, 3803}, {57, 344, 1, 3804}, {59, 346, 5, 3804}, {60, 347, 5, 3804}, {62, 349, 10, 3804}, {63, 350, 20, 3804}, {64, 351, 10, 3804}, {66, 353, 10, 3804}, {67, 354, 30, 3804}, {68, 355, 30, 3804}, {69, 356, 10, 3804}, {71, 358, 5, 3804}, {72, 359, 20, 3804}, {73, 360, 30, 3804}, {74, 361, 20, 3804}, {75, 362, 5, 3804}, {77, 364, 1, 3804}, {78, 365, 5, 3804}, {79, 366, 10, 3804}, {80, 367, 10, 3804}, {81, 368, 5, 3804}, {82, 369, 1, 3804}, {58, 342, 1, 3805}, {60, 343, 5, 3805}, {61, 344, 5, 3805}, {63, 345, 10, 3805}, {64, 346, 20, 3805}, {65, 347, 10, 3805}, {67, 348, 10, 3805}, {68, 349, 30, 3805}, {69, 350, 30, 3805}, {70, 351, 10, 3805}, {72, 352, 5, 3805}, {73, 353, 20, 3805}, {74, 354, 30, 3805}, {75, 355, 20, 3805}, {76, 356, 5, 3805}, {78, 357, 1, 3805}, {79, 358, 5, 3805}, {80, 359, 10, 3805}, {81, 360, 10, 3805}, {82, 361, 5, 3805}, {83, 362, 1, 3805}, {58, 343, 1, 3806}, {60, 345, 5, 3806}, {61, 346, 5, 3806}, {63, 348, 10, 3806}, {64, 349, 20, 3806}, {65, 350, 10, 3806}, {67, 352, 10, 3806}, {68, 353, 30, 3806}, {69, 354, 30, 3806}, {70, 355, 10, 3806}, {72, 357, 5, 3806}, {73, 358, 20, 3806}, {74, 359, 30, 3806}, {75, 360, 20, 3806}, {76, 361, 5, 3806}, {78, 363, 1, 3806}, {79, 364, 5, 3806}, {80, 365, 10, 3806}, {81, 366, 10, 3806}, {82, 367, 5, 3806}, {83, 368, 1, 3806}, {58, 344, 1, 3807}, {60, 346, 5, 3807}, {61, 347, 5, 3807}, {63, 349, 10, 3807}, {64, 350, 20, 3807}, {65, 351, 10, 3807}, {67, 353, 10, 3807}, {68, 354, 30, 3807}, {69, 355, 30, 3807}, {70, 356, 10, 3807}, {72, 358, 5, 3807}, {73, 359, 20, 3807}, {74, 360, 30, 3807}, {75, 361, 20, 3807}, {76, 362, 5, 3807}, {78, 364, 1, 3807}, {79, 365, 5, 3807}, {80, 366, 10, 3807}, {81, 367, 10, 3807}, {82, 368, 5, 3807}, {83, 369, 1, 3807}, {56, 370, 1, 3808}, {57, 371, 5, 3808}, {58, 372, 5, 3808}, {59, 373, 10, 3808}, {60, 374, 20, 3808}, {61, 375, 10, 3808}, {62, 376, 10, 3808}, {63, 377, 30, 3808}, {64, 378, 30, 3808}, {65, 379, 10, 3808}, {66, 380, 5, 3808}, {67, 381, 20, 3808}, {68, 382, 30, 3808}, {69, 383, 20, 3808}, {70, 384, 5, 3808}, {71, 385, 1, 3808}, {72, 386, 5, 3808}, {73, 387, 10, 3808}, {74, 388, 10, 3808}, {75, 389, 5, 3808}, {76, 390, 1, 3808}, {56, 371, 1, 3809}, {57, 373, 5, 3809}, {58, 374, 5, 3809}, {59, 376, 10, 3809}, {60, 377, 20, 3809}, {61, 378, 10, 3809}, {62, 380, 10, 3809}, {63, 381, 30, 3809}, {64, 382, 30, 3809}, {65, 383, 10, 3809}, {66, 385, 5, 3809}, {67, 386, 20, 3809}, {68, 387, 30, 3809}, {69, 388, 20, 3809}, {70, 389, 5, 3809}, {71, 391, 1, 3809}, {72, 392, 5, 3809}, {73, 393, 10, 3809}, {74, 394, 10, 3809}, {75, 395, 5, 3809}, {76, 396, 1, 3809}, {56, 372, 1, 3810}, {57, 374, 5, 3810}, {58, 375, 5, 3810}, {59, 377, 10, 3810}, {60, 378, 20, 3810}, {61, 379, 10, 3810}, {62, 381, 10, 3810}, {63, 382, 30, 3810}, {64, 383, 30, 3810}, {65, 384, 10, 3810}, {66, 386, 5, 3810}, {67, 387, 20, 3810}, {68, 388, 30, 3810}, {69, 389, 20, 3810}, {70, 390, 5, 3810}, {71, 392, 1, 3810}, {72, 393, 5, 3810}, {73, 394, 10, 3810}, {74, 395, 10, 3810}, {75, 396, 5, 3810}, {76, 397, 1, 3810}, {56, 373, 1, 3811}, {57, 376, 5, 3811}, {58, 377, 5, 3811}, {59, 380, 10, 3811}, {60, 381, 20, 3811}, {61, 382, 10, 3811}, {62, 385, 10, 3811}, {63, 386, 30, 3811}, {64, 387, 30, 3811}, {65, 388, 10, 3811}, {66, 391, 5, 3811}, {67, 392, 20, 3811}, {68, 393, 30, 3811}, {69, 394, 20, 3811}, {70, 395, 5, 3811}, {71, 398, 1, 3811}, {72, 399, 5, 3811}, {73, 400, 10, 3811}, {74, 401, 10, 3811}, {75, 402, 5, 3811}, {76, 403, 1, 3811}, {56, 374, 1, 3812}, {57, 377, 5, 3812}, {58, 378, 5, 3812}, {59, 381, 10, 3812}, {60, 382, 20, 3812}, {61, 383, 10, 3812}, {62, 386, 10, 3812}, {63, 387, 30, 3812}, {64, 388, 30, 3812}, {65, 389, 10, 3812}, {66, 392, 5, 3812}, {67, 393, 20, 3812}, {68, 394, 30, 3812}, {69, 395, 20, 3812}, {70, 396, 5, 3812}, {71, 399, 1, 3812}, {72, 400, 5, 3812}, {73, 401, 10, 3812}, {74, 402, 10, 3812}, {75, 403, 5, 3812}, {76, 404, 1, 3812}, {56, 375, 1, 3813}, {57, 378, 5, 3813}, {58, 379, 5, 3813}, {59, 382, 10, 3813}, {60, 383, 20, 3813}, {61, 384, 10, 3813}, {62, 387, 10, 3813}, {63, 388, 30, 3813}, {64, 389, 30, 3813}, {65, 390, 10, 3813}, {66, 393, 5, 3813}, {67, 394, 20, 3813}, {68, 395, 30, 3813}, {69, 396, 20, 3813}, {70, 397, 5, 3813}, {71, 400, 1, 3813}, {72, 401, 5, 3813}, {73, 402, 10, 3813}, {74, 403, 10, 3813}, {75, 404, 5, 3813}, {76, 405, 1, 3813}, {57, 370, 1, 3814}, {59, 371, 5, 3814}, {60, 372, 5, 3814}, {62, 373, 10, 3814}, {63, 374, 20, 3814}, {64, 375, 10, 3814}, {66, 376, 10, 3814}, {67, 377, 30, 3814}, {68, 378, 30, 3814}, {69, 379, 10, 3814}, {71, 380, 5, 3814}, {72, 381, 20, 3814}, {73, 382, 30, 3814}, {74, 383, 20, 3814}, {75, 384, 5, 3814}, {77, 385, 1, 3814}, {78, 386, 5, 3814}, {79, 387, 10, 3814}, {80, 388, 10, 3814}, {81, 389, 5, 3814}, {82, 390, 1, 3814}, {57, 371, 1, 3815}, {59, 373, 5, 3815}, {60, 374, 5, 3815}, {62, 376, 10, 3815}, {63, 377, 20, 3815}, {64, 378, 10, 3815}, {66, 380, 10, 3815}, {67, 381, 30, 3815}, {68, 382, 30, 3815}, {69, 383, 10, 3815}, {71, 385, 5, 3815}, {72, 386, 20, 3815}, {73, 387, 30, 3815}, {74, 388, 20, 3815}, {75, 389, 5, 3815}, {77, 391, 1,

3815}, {78, 392, 5, 3815}, {79, 393, 10, 3815}, {80, 394, 10, 3815}, {81, 395, 5, 3815}, {82, 396, 1, 3815}, {57, 372, 1, 3816}, {59, 374, 5, 3816}, {60, 375, 5, 3816}, {62, 377, 10, 3816}, {63, 378, 20, 3816}, {64, 379, 10, 3816}, {66, 381, 10, 3816}, {67, 382, 30, 3816}, {68, 383, 30, 3816}, {69, 384, 10, 3816}, {71, 386, 5, 3816}, {72, 387, 20, 3816}, {73, 388, 30, 3816}, {74, 389, 20, 3816}, {75, 390, 5, 3816}, {77, 392, 1, 3816}, {78, 393, 5, 3816}, {79, 394, 10, 3816}, {80, 395, 10, 3816}, {81, 396, 5, 3816}, {82, 397, 1, 3816}, {57, 373, 1, 3817}, {59, 376, 5, 3817}, {60, 377, 5, 3817}, {62, 380, 10, 3817}, {63, 381, 20, 3817}, {64, 382, 10, 3817}, {66, 385, 10, 3817}, {67, 386, 30, 3817}, {68, 387, 30, 3817}, {69, 388, 10, 3817}, {71, 391, 5, 3817}, {72, 392, 20, 3817}, {73, 393, 30, 3817}, {74, 394, 20, 3817}, {75, 395, 5, 3817}, {77, 398, 1, 3817}, {78, 399, 5, 3817}, {79, 400, 10, 3817}, {80, 401, 10, 3817}, {81, 402, 5, 3817}, {82, 403, 1, 3817}, {57, 374, 1, 3818}, {59, 377, 5, 3818}, {60, 378, 5, 3818}, {62, 381, 10, 3818}, {63, 382, 20, 3818}, {64, 383, 10, 3818}, {66, 386, 10, 3818}, {67, 387, 30, 3818}, {68, 388, 30, 3818}, {69, 389, 10, 3818}, {71, 392, 5, 3818}, {72, 393, 20, 3818}, {73, 394, 30, 3818}, {74, 395, 20, 3818}, {75, 396, 5, 3818}, {77, 399, 1, 3818}, {78, 400, 5, 3818}, {79, 401, 10, 3818}, {80, 402, 10, 3818}, {81, 403, 5, 3818}, {82, 404, 1, 3818}, {57, 375, 1, 3819}, {59, 378, 5, 3819}, {60, 379, 5, 3819}, {62, 382, 10, 3819}, {63, 383, 20, 3819}, {64, 384, 10, 3819}, {66, 387, 10, 3819}, {67, 388, 30, 3819}, {68, 389, 30, 3819}, {69, 390, 10, 3819}, {71, 393, 5, 3819}, {72, 394, 20, 3819}, {73, 395, 30, 3819}, {74, 396, 20, 3819}, {75, 397, 5, 3819}, {77, 400, 1, 3819}, {78, 401, 5, 3819}, {79, 402, 10, 3819}, {80, 403, 10, 3819}, {81, 404, 5, 3819}, {82, 405, 1, 3819}, {58, 370, 1, 3820}, {60, 371, 5, 3820}, {61, 372, 5, 3820}, {63, 373, 10, 3820}, {64, 374, 20, 3820}, {65, 375, 10, 3820}, {67, 376, 10, 3820}, {68, 377, 30, 3820}, {69, 378, 30, 3820}, {70, 379, 10, 3820}, {72, 380, 5, 3820}, {73, 381, 20, 3820}, {74, 382, 30, 3820}, {75, 383, 20, 3820}, {76, 384, 5, 3820}, {78, 385, 1, 3820}, {79, 386, 5, 3820}, {80, 387, 10, 3820}, {81, 388, 10, 3820}, {82, 389, 5, 3820}, {83, 390, 1, 3820}, {58, 371, 1, 3821}, {60, 373, 5, 3821}, {61, 374, 5, 3821}, {63, 376, 10, 3821}, {64, 377, 20, 3821}, {65, 378, 10, 3821}, {67, 380, 10, 3821}, {68, 381, 30, 3821}, {69, 382, 30, 3821}, {70, 383, 10, 3821}, {72, 385, 5, 3821}, {73, 386, 20, 3821}, {74, 387, 30, 3821}, {75, 388, 20, 3821}, {76, 389, 5, 3821}, {78, 391, 1, 3821}, {79, 392, 5, 3821}, {80, 393, 10, 3821}, {81, 394, 10, 3821}, {82, 395, 5, 3821}, {83, 396, 1, 3821}, {58, 372, 1, 3822}, {60, 374, 5, 3822}, {61, 375, 5, 3822}, {63, 377, 10, 3822}, {64, 378, 20, 3822}, {65, 379, 10, 3822}, {67, 381, 10, 3822}, {68, 382, 30, 3822}, {69, 383, 30, 3822}, {70, 384, 10, 3822}, {72, 386, 5, 3822}, {73, 387, 20, 3822}, {74, 388, 30, 3822}, {75, 389, 20, 3822}, {76, 390, 5, 3822}, {78, 392, 1, 3822}, {79, 393, 5, 3822}, {80, 394, 10, 3822}, {81, 395, 10, 3822}, {82, 396, 5, 3822}, {83, 397, 1, 3822}, {58, 373, 1, 3823}, {60, 376, 5, 3823}, {61, 377, 5, 3823}, {63, 380, 10, 3823}, {64, 381, 20, 3823}, {65, 382, 10, 3823}, {67, 385, 10, 3823}, {68, 386, 30, 3823}, {69, 387, 30, 3823}, {70, 388, 10, 3823}, {72, 391, 5, 3823}, {73, 392, 20, 3823}, {74, 393, 30, 3823}, {75, 394, 20, 3823}, {76, 395, 5, 3823}, {78, 398, 1, 3823}, {79, 399, 5, 3823}, {80, 400, 10, 3823}, {81, 401, 10, 3823}, {82, 402, 5, 3823}, {83, 403, 1, 3823}, {58, 374, 1, 3824}, {60, 377, 5, 3824}, {61, 378, 5, 3824}, {63, 381, 10, 3824}, {64, 382, 20, 3824}, {65, 383, 10, 3824}, {67, 386, 10, 3824}, {68, 387, 30, 3824}, {69, 388, 30, 3824}, {70, 389, 10, 3824}, {72, 392, 5, 3824}, {73, 393, 20, 3824}, {74, 394, 30, 3824}, {75, 395, 20, 3824}, {76, 396, 5, 3824}, {78, 399, 1, 3824}, {79, 400, 5, 3824}, {80, 401, 10, 3824}, {81, 402, 10, 3824}, {82, 403, 5, 3824}, {83, 404, 1, 3824}, {58, 375, 1, 3825}, {60, 378, 5, 3825}, {61, 379, 5, 3825}, {63, 382, 10, 3825}, {64, 383, 20, 3825}, {65, 384, 10, 3825}, {67, 387, 10, 3825}, {68, 388, 30, 3825}, {69, 389, 30, 3825}, {70, 390, 10, 3825}, {72, 393, 5, 3825}, {73, 394, 20, 3825}, {74, 395, 30, 3825}, {75, 396, 20, 3825}, {76, 397, 5, 3825}, {78, 400, 1, 3825}, {79, 401, 5, 3825}, {80, 402, 10, 3825}, {81, 403, 10, 3825}, {82, 404, 5, 3825}, {83, 405, 1, 3825}, {56, 342, 1, 3826}, {57, 343, 6, 3826}, {58, 344, 6, 3826}, {59, 345, 15, 3826}, {60, 346, 30, 3826}, {61, 347, 15, 3826}, {62, 348, 20, 3826}, {63, 349, 60, 3826}, {64, 350, 60, 3826}, {65, 351, 20, 3826}, {66, 352, 15, 3826}, {67, 353, 60, 3826}, {68, 354, 90, 3826}, {69, 355, 60, 3826}, {70, 356, 15, 3826}, {71, 357, 6, 3826}, {72, 358, 30, 3826}, {73, 359, 60, 3826}, {74, 360, 60, 3826}, {75, 361, 30, 3826}, {76, 362, 6, 3826}, {77, 363, 1, 3826}, {78, 364, 6, 3826}, {79, 365, 15, 3826}, {80, 366, 20, 3826}, {81, 367, 15, 3826}, {82, 368, 6, 3826}, {83, 369, 1, 3826}, {56, 370, 1, 3827}, {57, 371, 6, 3827}, {58, 372, 6, 3827}, {59, 373, 15, 3827}, {60, 374, 30, 3827}, {61, 375, 15, 3827}, {62, 376, 20, 3827}, {63, 377, 60, 3827}, {64, 378, 60, 3827}, {65, 379, 20, 3827}, {66, 380, 15, 3827}, {67, 381, 60, 3827}, {68, 382, 90, 3827}, {69, 383, 60, 3827}, {70, 384, 15, 3827}, {71, 385, 6, 3827}, {72, 386, 30, 3827}, {73, 387, 60, 3827}, {74, 388, 60, 3827}, {75, 389, 30, 3827}, {76, 390, 6, 3827}, {77, 391, 1, 3827}, {78, 392, 6, 3827}, {79, 393, 15, 3827}, {80, 394, 20, 3827}, {81, 395, 15, 3827}, {82, 396, 6, 3827}, {83, 397, 1, 3827}, {56, 371, 1, 3828}, {57, 373, 6, 3828}, {58, 374, 6, 3828}, {59, 376, 15, 3828}, {60, 377, 30, 3828}, {61, 378, 15, 3828}, {62, 380, 20, 3828}, {63, 381, 60, 3828}, {64, 382, 60, 3828}, {65, 383, 20, 3828}, {66, 385, 15, 3828}, {67, 386, 60, 3828}, {68, 387, 90, 3828}, {69, 388, 60, 3828}, {70, 389, 15, 3828}, {71, 391, 6, 3828}, {72, 392, 30, 3828}, {73, 393, 60, 3828}, {74, 394, 60, 3828}, {75, 395, 30, 3828}, {76, 396, 6, 3828}, {77, 398, 1, 3828}, {78, 399, 6, 3828}, {79, 400, 15, 3828}, {80, 401, 20, 3828},

{81, 402, 15, 3828}, {82, 403, 6, 3828}, {83, 404, 1, 3828}, {56, 372, 1, 3829}, {57, 374, 6, 3829}, {58, 375, 6, 3829}, {59, 377, 15, 3829}, {60, 378, 30, 3829}, {61, 379, 15, 3829}, {62, 381, 20, 3829}, {63, 382, 60, 3829}, {64, 383, 60, 3829}, {65, 384, 20, 3829}, {66, 386, 15, 3829}, {67, 387, 60, 3829}, {68, 388, 90, 3829}, {69, 389, 60, 3829}, {70, 390, 15, 3829}, {71, 392, 6, 3829}, {72, 393, 30, 3829}, {73, 394, 60, 3829}, {74, 395, 60, 3829}, {75, 396, 30, 3829}, {76, 397, 6, 3829}, {77, 399, 1, 3829}, {78, 400, 6, 3829}, {79, 401, 15, 3829}, {80, 402, 20, 3829}, {81, 403, 15, 3829}, {82, 404, 6, 3829}, {83, 405, 1, 3829}, {56, 406, 1, 3830}, {57, 407, 6, 3830}, {58, 408, 6, 3830}, {59, 409, 15, 3830}, {60, 410, 30, 3830}, {61, 411, 15, 3830}, {62, 412, 20, 3830}, {63, 413, 60, 3830}, {64, 414, 60, 3830}, {65, 415, 20, 3830}, {66, 416, 15, 3830}, {67, 417, 60, 3830}, {68, 418, 90, 3830}, {69, 419, 60, 3830}, {70, 420, 15, 3830}, {71, 421, 6, 3830}, {72, 422, 30, 3830}, {73, 423, 60, 3830}, {74, 424, 60, 3830}, {75, 425, 30, 3830}, {76, 426, 6, 3830}, {77, 427, 1, 3830}, {78, 428, 6, 3830}, {79, 429, 15, 3830}, {80, 430, 20, 3830}, {81, 431, 15, 3830}, {82, 432, 6, 3830}, {83, 433, 1, 3830}, {56, 407, 1, 3831}, {57, 409, 6, 3831}, {58, 410, 6, 3831}, {59, 412, 15, 3831}, {60, 413, 30, 3831}, {61, 414, 15, 3831}, {62, 416, 20, 3831}, {63, 417, 60, 3831}, {64, 418, 60, 3831}, {65, 419, 20, 3831}, {66, 421, 15, 3831}, {67, 422, 60, 3831}, {68, 423, 90, 3831}, {69, 424, 60, 3831}, {70, 425, 15, 3831}, {71, 427, 6, 3831}, {72, 428, 30, 3831}, {73, 429, 60, 3831}, {74, 430, 60, 3831}, {75, 431, 30, 3831}, {76, 432, 6, 3831}, {77, 434, 1, 3831}, {78, 435, 6, 3831}, {79, 436, 15, 3831}, {80, 437, 20, 3831}, {81, 438, 15, 3831}, {82, 439, 6, 3831}, {83, 440, 1, 3831}, {56, 408, 1, 3832}, {57, 410, 6, 3832}, {58, 411, 6, 3832}, {59, 413, 15, 3832}, {60, 414, 30, 3832}, {61, 415, 15, 3832}, {62, 417, 20, 3832}, {63, 418, 60, 3832}, {64, 419, 60, 3832}, {65, 420, 20, 3832}, {66, 422, 15, 3832}, {67, 423, 60, 3832}, {68, 424, 90, 3832}, {69, 425, 60, 3832}, {70, 426, 15, 3832}, {71, 428, 6, 3832}, {72, 429, 30, 3832}, {73, 430, 60, 3832}, {74, 431, 60, 3832}, {75, 432, 30, 3832}, {76, 433, 6, 3832}, {77, 435, 1, 3832}, {78, 436, 6, 3832}, {79, 437, 15, 3832}, {80, 438, 20, 3832}, {81, 439, 15, 3832}, {82, 440, 6, 3832}, {83, 441, 1, 3832}, {56, 409, 1, 3833}, {57, 412, 6, 3833}, {58, 413, 6, 3833}, {59, 416, 15, 3833}, {60, 417, 30, 3833}, {61, 418, 15, 3833}, {62, 421, 20, 3833}, {63, 422, 60, 3833}, {64, 423, 60, 3833}, {65, 424, 20, 3833}, {66, 427, 15, 3833}, {67, 428, 60, 3833}, {68, 429, 90, 3833}, {69, 430, 60, 3833}, {70, 431, 15, 3833}, {71, 434, 6, 3833}, {72, 435, 30, 3833}, {73, 436, 60, 3833}, {74, 437, 60, 3833}, {75, 438, 30, 3833}, {76, 439, 6, 3833}, {77, 442, 1, 3833}, {78, 443, 6, 3833}, {79, 444, 15, 3833}, {80, 445, 20, 3833}, {81, 446, 15, 3833}, {82, 447, 6, 3833}, {83, 448, 1, 3833}, {56, 410, 1, 3834}, {57, 413, 6, 3834}, {58, 414, 6, 3834}, {59, 417, 15, 3834}, {60, 418, 30, 3834}, {61, 419, 15, 3834}, {62, 422, 20, 3834}, {63, 423, 60, 3834}, {64, 424, 60, 3834}, {65, 425, 20, 3834}, {66, 428, 15, 3834}, {67, 429, 60, 3834}, {68, 430, 90, 3834}, {69, 431, 60, 3834}, {70, 432, 15, 3834}, {71, 435, 6, 3834}, {72, 436, 30, 3834}, {73, 437, 60, 3834}, {74, 438, 60, 3834}, {75, 439, 30, 3834}, {76, 440, 6, 3834}, {77, 443, 1, 3834}, {78, 444, 6, 3834}, {79, 445, 15, 3834}, {80, 446, 20, 3834}, {81, 447, 15, 3834}, {82, 448, 6, 3834}, {83, 449, 1, 3834}, {56, 411, 1, 3835}, {57, 414, 6, 3835}, {58, 415, 6, 3835}, {59, 418, 15, 3835}, {60, 419, 30, 3835}, {61, 420, 15, 3835}, {62, 423, 20, 3835}, {63, 424, 60, 3835}, {64, 425, 60, 3835}, {65, 426, 20, 3835}, {66, 429, 15, 3835}, {67, 430, 60, 3835}, {68, 431, 90, 3835}, {69, 432, 60, 3835}, {70, 433, 15, 3835}, {71, 436, 6, 3835}, {72, 437, 30, 3835}, {73, 438, 60, 3835}, {74, 439, 60, 3835}, {75, 440, 30, 3835}, {76, 441, 6, 3835}, {77, 444, 1, 3835}, {78, 445, 6, 3835}, {79, 446, 15, 3835}, {80, 447, 20, 3835}, {81, 448, 15, 3835}, {82, 449, 6, 3835}, {83, 450, 1, 3835}, {84, 296, 1, 3836}, {85, 297, 3, 3836}, {86, 298, 3, 3836}, {87, 299, 3, 3836}, {88, 300, 6, 3836}, {89, 301, 3, 3836}, {90, 302, 1, 3836}, {91, 303, 3, 3836}, {92, 304, 3, 3836}, {93, 305, 1, 3836}, {85, 296, 1, 3837}, {87, 297, 3, 3837}, {88, 298, 3, 3837}, {90, 299, 3, 3837}, {91, 300, 6, 3837}, {92, 301, 3, 3837}, {94, 302, 1, 3837}, {95, 303, 3, 3837}, {96, 304, 3, 3837}, {97, 305, 1, 3837}, {86, 296, 1, 3838}, {88, 297, 3, 3838}, {89, 298, 3, 3838}, {91, 299, 3, 3838}, {92, 300, 6, 3838}, {93, 301, 3, 3838}, {95, 302, 1, 3838}, {96, 303, 3, 3838}, {97, 304, 3, 3838}, {98, 305, 1, 3838}, {87, 296, 1, 3839}, {90, 297, 3, 3839}, {91, 298, 3, 3839}, {94, 299, 3, 3839}, {95, 300, 6, 3839}, {96, 301, 3, 3839}, {99, 302, 1, 3839}, {100, 303, 3, 3839}, {101, 304, 3, 3839}, {102, 305, 1, 3839}, {88, 296, 1, 3840}, {91, 297, 3, 3840}, {92, 298, 3, 3840}, {95, 299, 3, 3840}, {96, 300, 6, 3840}, {97, 301, 3, 3840}, {100, 302, 1, 3840}, {101, 303, 3, 3840}, {102, 304, 3, 3840}, {103, 305, 1, 3840}, {89, 296, 1, 3841}, {92, 297, 3, 3841}, {93, 298, 3, 3841}, {96, 299, 3, 3841}, {97, 300, 6, 3841}, {98, 301, 3, 3841}, {101, 302, 1, 3841}, {102, 303, 3, 3841}, {103, 304, 3, 3841}, {104, 305, 1, 3841}, {90, 296, 1, 3842}, {94, 297, 3, 3842}, {95, 298, 3, 3842}, {99, 299, 3, 3842}, {100, 300, 6, 3842}, {101, 301, 3, 3842}, {105, 302, 1, 3842}, {106, 303, 3, 3842}, {107, 304, 3, 3842}, {108, 305, 1, 3842}, {91, 296, 1, 3843}, {95, 297, 3, 3843}, {96, 298, 3, 3843}, {100, 299, 3, 3843}, {101, 300, 6, 3843}, {102, 301, 3, 3843}, {106, 302, 1, 3843}, {107, 303, 3, 3843}, {108, 304, 3, 3843}, {109, 305, 1, 3843}, {92, 296, 1, 3844}, {96, 297, 3, 3844}, {97, 298, 3, 3844}, {101, 299, 3, 3844}, {102, 300, 6, 3844}, {103, 301, 3, 3844}, {107, 302, 1, 3844}, {108, 303, 3, 3844}, {109, 304, 3, 3844}, {110, 305, 1, 3844}, {93, 296, 1, 3845}, {97, 297, 3, 3845}, {98, 298, 3, 3845}, {102, 299, 3, 3845}, {103, 300, 6, 3845}, {104, 301, 3, 3845}, {108, 302, 1, 3845}, {109, 303, 3, 3845}, {110, 304, 3, 3845},

{111, 305, 1, 3845}, {94, 296, 1, 3846}, {99, 297, 3, 3846}, {100, 298, 3, 3846}, {105, 299, 3, 3846}, {106, 300, 6, 3846}, {107, 301, 3, 3846}, {112, 302, 1, 3846}, {113, 303, 3, 3846}, {114, 304, 3, 3846}, {115, 305, 1, 3846}, {95, 296, 1, 3847}, {100, 297, 3, 3847}, {101, 298, 3, 3847}, {106, 299, 3, 3847}, {107, 300, 6, 3847}, {108, 301, 3, 3847}, {113, 302, 1, 3847}, {114, 303, 3, 3847}, {115, 304, 3, 3847}, {116, 305, 1, 3847}, {96, 296, 1, 3848}, {101, 297, 3, 3848}, {102, 298, 3, 3848}, {107, 299, 3, 3848}, {108, 300, 6, 3848}, {109, 301, 3, 3848}, {114, 302, 1, 3848}, {115, 303, 3, 3848}, {116, 304, 3, 3848}, {117, 305, 1, 3848}, {97, 296, 1, 3849}, {102, 297, 3, 3849}, {103, 298, 3, 3849}, {108, 299, 3, 3849}, {109, 300, 6, 3849}, {110, 301, 3, 3849}, {115, 302, 1, 3849}, {116, 303, 3, 3849}, {117, 304, 3, 3849}, {118, 305, 1, 3849}, {98, 296, 1, 3850}, {103, 297, 3, 3850}, {104, 298, 3, 3850}, {109, 299, 3, 3850}, {110, 300, 6, 3850}, {111, 301, 3, 3850}, {116, 302, 1, 3850}, {117, 303, 3, 3850}, {118, 304, 3, 3850}, {119, 305, 1, 3850}, {84, 306, 1, 3851}, {85, 307, 4, 3851}, {86, 308, 4, 3851}, {87, 309, 6, 3851}, {88, 310, 12, 3851}, {89, 311, 6, 3851}, {90, 312, 4, 3851}, {91, 313, 12, 3851}, {92, 314, 12, 3851}, {93, 315, 4, 3851}, {94, 316, 1, 3851}, {95, 317, 4, 3851}, {96, 318, 6, 3851}, {97, 319, 4, 3851}, {98, 320, 1, 3851}, {85, 306, 1, 3852}, {87, 307, 4, 3852}, {88, 308, 4, 3852}, {90, 309, 6, 3852}, {91, 310, 12, 3852}, {92, 311, 6, 3852}, {94, 312, 4, 3852}, {95, 313, 12, 3852}, {96, 314, 12, 3852}, {97, 315, 4, 3852}, {99, 316, 1, 3852}, {100, 317, 4, 3852}, {101, 318, 6, 3852}, {102, 319, 4, 3852}, {103, 320, 1, 3852}, {86, 306, 1, 3853}, {88, 307, 4, 3853}, {89, 308, 4, 3853}, {91, 309, 6, 3853}, {92, 310, 12, 3853}, {93, 311, 6, 3853}, {95, 312, 4, 3853}, {96, 313, 12, 3853}, {97, 314, 12, 3853}, {98, 315, 4, 3853}, {100, 316, 1, 3853}, {101, 317, 4, 3853}, {102, 318, 6, 3853}, {103, 319, 4, 3853}, {104, 320, 1, 3853}, {87, 306, 1, 3854}, {90, 307, 4, 3854}, {91, 308, 4, 3854}, {94, 309, 6, 3854}, {95, 310, 12, 3854}, {96, 311, 6, 3854}, {99, 312, 4, 3854}, {100, 313, 12, 3854}, {101, 314, 12, 3854}, {102, 315, 4, 3854}, {105, 316, 1, 3854}, {106, 317, 4, 3854}, {107, 318, 6, 3854}, {108, 319, 4, 3854}, {109, 320, 1, 3854}, {88, 306, 1, 3855}, {91, 307, 4, 3855}, {92, 308, 4, 3855}, {95, 309, 6, 3855}, {96, 310, 12, 3855}, {97, 311, 6, 3855}, {100, 312, 4, 3855}, {101, 313, 12, 3855}, {102, 314, 12, 3855}, {103, 315, 4, 3855}, {106, 316, 1, 3855}, {107, 317, 4, 3855}, {108, 318, 6, 3855}, {109, 319, 4, 3855}, {110, 320, 1, 3855}, {89, 306, 1, 3856}, {92, 307, 4, 3856}, {93, 308, 4, 3856}, {96, 309, 6, 3856}, {97, 310, 12, 3856}, {98, 311, 6, 3856}, {101, 312, 4, 3856}, {102, 313, 12, 3856}, {103, 314, 12, 3856}, {104, 315, 4, 3856}, {107, 316, 1, 3856}, {108, 317, 4, 3856}, {109, 318, 6, 3856}, {110, 319, 4, 3856}, {111, 320, 1, 3856}, {90, 306, 1, 3857}, {94, 307, 4, 3857}, {95, 308, 4, 3857}, {99, 309, 6, 3857}, {100, 310, 12, 3857}, {101, 311, 6, 3857}, {105, 312, 4, 3857}, {106, 313, 12, 3857}, {107, 314, 12, 3857}, {108, 315, 4, 3857}, {112, 316, 1, 3857}, {113, 317, 4, 3857}, {114, 318, 6, 3857}, {115, 319, 4, 3857}, {116, 320, 1, 3857}, {91, 306, 1, 3858}, {95, 307, 4, 3858}, {96, 308, 4, 3858}, {100, 309, 6, 3858}, {101, 310, 12, 3858}, {102, 311, 6, 3858}, {106, 312, 4, 3858}, {107, 313, 12, 3858}, {108, 314, 12, 3858}, {109, 315, 4, 3858}, {113, 316, 1, 3858}, {114, 317, 4, 3858}, {115, 318, 6, 3858}, {116, 319, 4, 3858}, {117, 320, 1, 3858}, {92, 306, 1, 3859}, {96, 307, 4, 3859}, {97, 308, 4, 3859}, {101, 309, 6, 3859}, {102, 310, 12, 3859}, {103, 311, 6, 3859}, {107, 312, 4, 3859}, {108, 313, 12, 3859}, {109, 314, 12, 3859}, {110, 315, 4, 3859}, {114, 316, 1, 3859}, {115, 317, 4, 3859}, {116, 318, 6, 3859}, {117, 319, 4, 3859}, {118, 320, 1, 3859}, {93, 306, 1, 3860}, {97, 307, 4, 3860}, {98, 308, 4, 3860}, {102, 309, 6, 3860}, {103, 310, 12, 3860}, {104, 311, 6, 3860}, {108, 312, 4, 3860}, {109, 313, 12, 3860}, {110, 314, 12, 3860}, {111, 315, 4, 3860}, {115, 316, 1, 3860}, {116, 317, 4, 3860}, {117, 318, 6, 3860}, {118, 319, 4, 3860}, {119, 320, 1, 3860}, {84, 321, 1, 3861}, {85, 322, 4, 3861}, {86, 323, 4, 3861}, {87, 324, 6, 3861}, {88, 325, 12, 3861}, {89, 326, 6, 3861}, {90, 327, 4, 3861}, {91, 328, 12, 3861}, {92, 329, 12, 3861}, {93, 330, 4, 3861}, {94, 331, 1, 3861}, {95, 332, 4, 3861}, {96, 333, 6, 3861}, {97, 334, 4, 3861}, {98, 335, 1, 3861}, {84, 322, 1, 3862}, {85, 324, 4, 3862}, {86, 325, 4, 3862}, {87, 327, 6, 3862}, {88, 328, 12, 3862}, {89, 329, 6, 3862}, {90, 331, 4, 3862}, {91, 332, 12, 3862}, {92, 333, 12, 3862}, {93, 334, 4, 3862}, {94, 336, 1, 3862}, {95, 337, 4, 3862}, {96, 338, 6, 3862}, {97, 339, 4, 3862}, {98, 340, 1, 3862}, {84, 323, 1, 3863}, {85, 325, 4, 3863}, {86, 326, 4, 3863}, {87, 328, 6, 3863}, {88, 329, 12, 3863}, {89, 330, 6, 3863}, {90, 332, 4, 3863}, {91, 333, 12, 3863}, {92, 334, 12, 3863}, {93, 335, 4, 3863}, {94, 337, 1, 3863}, {95, 338, 4, 3863}, {96, 339, 6, 3863}, {97, 340, 4, 3863}, {98, 341, 1, 3863}, {85, 321, 1, 3864}, {87, 322, 4, 3864}, {88, 323, 4, 3864}, {90, 324, 6, 3864}, {91, 325, 12, 3864}, {92, 326, 6, 3864}, {94, 327, 4, 3864}, {95, 328, 12, 3864}, {96, 329, 12, 3864}, {97, 330, 4, 3864}, {99, 331, 1, 3864}, {100, 332, 4, 3864}, {101, 333, 6, 3864}, {102, 334, 4, 3864}, {103, 335, 1, 3864}, {85, 322, 1, 3865}, {87, 324, 4, 3865}, {88, 325, 4, 3865}, {90, 327, 6, 3865}, {91, 328, 12, 3865}, {92, 329, 6, 3865}, {94, 331, 4, 3865}, {95, 332, 12, 3865}, {96, 333, 12, 3865}, {97, 334, 4, 3865}, {99, 336, 1, 3865}, {100, 337, 4, 3865}, {101, 338, 6, 3865}, {102, 339, 4, 3865}, {103, 340, 1, 3865}, {85, 323, 1, 3866}, {87, 325, 4, 3866}, {88, 326, 4, 3866}, {90, 328, 6, 3866}, {91, 329, 12, 3866}, {92, 330, 6, 3866}, {94, 332, 4, 3866}, {95, 333, 12, 3866}, {96, 334, 12, 3866}, {97, 335, 4, 3866}, {99, 337, 1, 3866}, {100, 338, 4, 3866}, {101, 339, 6, 3866}, {102, 340, 4, 3866}, {103, 341, 1, 3866}, {86, 321, 1, 3867}, {88, 322, 4, 3867}, {89, 323, 4, 3867}, {91, 324, 6, 3867}, {92, 325, 12, 3867}, {93, 326, 6,

3867}, {95, 327, 4, 3867}, {96, 328, 12, 3867}, {97, 329, 12, 3867}, {98, 330, 4, 3867},
{100, 331, 1, 3867}, {101, 332, 4, 3867}, {102, 333, 6, 3867}, {103, 334, 4, 3867}, {104,
335, 1, 3867}, {86, 322, 1, 3868}, {88, 324, 4, 3868}, {89, 325, 4, 3868}, {91, 327, 6,
3868}, {92, 328, 12, 3868}, {93, 329, 6, 3868}, {95, 331, 4, 3868}, {96, 332, 12, 3868},
{97, 333, 12, 3868}, {98, 334, 4, 3868}, {100, 336, 1, 3868}, {101, 337, 4, 3868}, {102,
338, 6, 3868}, {103, 339, 4, 3868}, {104, 340, 1, 3868}, {86, 323, 1, 3869}, {88, 325, 4,
3869}, {89, 326, 4, 3869}, {91, 328, 6, 3869}, {92, 329, 12, 3869}, {93, 330, 6, 3869},
{95, 332, 4, 3869}, {96, 333, 12, 3869}, {97, 334, 12, 3869}, {98, 335, 4, 3869}, {100,
337, 1, 3869}, {101, 338, 4, 3869}, {102, 339, 6, 3869}, {103, 340, 4, 3869}, {104, 341,
1, 3869}, {87, 321, 1, 3870}, {90, 322, 4, 3870}, {91, 323, 4, 3870}, {94, 324, 6, 3870},
{95, 325, 12, 3870}, {96, 326, 6, 3870}, {99, 327, 4, 3870}, {100, 328, 12, 3870}, {101,
329, 12, 3870}, {102, 330, 4, 3870}, {105, 331, 1, 3870}, {106, 332, 4, 3870}, {107, 333,
6, 3870}, {108, 334, 4, 3870}, {109, 335, 1, 3870}, {87, 322, 1, 3871}, {90, 324, 4,
3871}, {91, 325, 4, 3871}, {94, 327, 6, 3871}, {95, 328, 12, 3871}, {96, 329, 6, 3871},
{99, 331, 4, 3871}, {100, 332, 12, 3871}, {101, 333, 12, 3871}, {102, 334, 4, 3871}, {105,
336, 1, 3871}, {106, 337, 4, 3871}, {107, 338, 6, 3871}, {108, 339, 4, 3871}, {109, 340,
1, 3871}, {87, 323, 1, 3872}, {90, 325, 4, 3872}, {91, 326, 4, 3872}, {94, 328, 6, 3872},
{95, 329, 12, 3872}, {96, 330, 6, 3872}, {99, 332, 4, 3872}, {100, 333, 12, 3872}, {101,
334, 12, 3872}, {102, 335, 4, 3872}, {105, 337, 1, 3872}, {106, 338, 4, 3872}, {107, 339,
6, 3872}, {108, 340, 4, 3872}, {109, 341, 1, 3872}, {88, 321, 1, 3873}, {91, 322, 4,
3873}, {92, 323, 4, 3873}, {95, 324, 6, 3873}, {96, 325, 12, 3873}, {97, 326, 6, 3873},
{100, 327, 4, 3873}, {101, 328, 12, 3873}, {102, 329, 12, 3873}, {103, 330, 4, 3873},
{106, 331, 1, 3873}, {107, 332, 4, 3873}, {108, 333, 6, 3873}, {109, 334, 4, 3873}, {110,
335, 1, 3873}, {88, 322, 1, 3874}, {91, 324, 4, 3874}, {92, 325, 4, 3874}, {95, 327, 6,
3874}, {96, 328, 12, 3874}, {97, 329, 6, 3874}, {100, 331, 4, 3874}, {101, 332, 12, 3874},
{102, 333, 12, 3874}, {103, 334, 4, 3874}, {106, 336, 1, 3874}, {107, 337, 4, 3874}, {108,
338, 6, 3874}, {109, 339, 4, 3874}, {110, 340, 1, 3874}, {88, 323, 1, 3875}, {91, 325, 4,
3875}, {92, 326, 4, 3875}, {95, 328, 6, 3875}, {96, 329, 12, 3875}, {97, 330, 6, 3875},
{100, 332, 4, 3875}, {101, 333, 12, 3875}, {102, 334, 12, 3875}, {103, 335, 4, 3875},
{106, 337, 1, 3875}, {107, 338, 4, 3875}, {108, 339, 6, 3875}, {109, 340, 4, 3875}, {110,
341, 1, 3875}, {89, 321, 1, 3876}, {92, 322, 4, 3876}, {93, 323, 4, 3876}, {96, 324, 6,
3876}, {97, 325, 12, 3876}, {98, 326, 6, 3876}, {101, 327, 4, 3876}, {102, 328, 12, 3876},
{103, 329, 12, 3876}, {104, 330, 4, 3876}, {107, 331, 1, 3876}, {108, 332, 4, 3876}, {109,
333, 6, 3876}, {110, 334, 4, 3876}, {111, 335, 1, 3876}, {89, 322, 1, 3877}, {92, 324, 4,
3877}, {93, 325, 4, 3877}, {96, 327, 6, 3877}, {97, 328, 12, 3877}, {98, 329, 6, 3877},
{101, 331, 4, 3877}, {102, 332, 12, 3877}, {103, 333, 12, 3877}, {104, 334, 4, 3877},
{107, 336, 1, 3877}, {108, 337, 4, 3877}, {109, 338, 6, 3877}, {110, 339, 4, 3877}, {111,
340, 1, 3877}, {89, 323, 1, 3878}, {92, 325, 4, 3878}, {93, 326, 4, 3878}, {96, 328, 6,
3878}, {97, 329, 12, 3878}, {98, 330, 6, 3878}, {101, 332, 4, 3878}, {102, 333, 12, 3878},
{103, 334, 12, 3878}, {104, 335, 4, 3878}, {107, 337, 1, 3878}, {108, 338, 4, 3878}, {109,
339, 6, 3878}, {110, 340, 4, 3878}, {111, 341, 1, 3878}, {90, 321, 1, 3879}, {94, 322, 4,
3879}, {95, 323, 4, 3879}, {99, 324, 6, 3879}, {100, 325, 12, 3879}, {101, 326, 6, 3879},
{105, 327, 4, 3879}, {106, 328, 12, 3879}, {107, 329, 12, 3879}, {108, 330, 4, 3879},
{112, 331, 1, 3879}, {113, 332, 4, 3879}, {114, 333, 6, 3879}, {115, 334, 4, 3879}, {116,
335, 1, 3879}, {90, 322, 1, 3880}, {94, 324, 4, 3880}, {95, 325, 4, 3880}, {99, 327, 6,
3880}, {100, 328, 12, 3880}, {101, 329, 6, 3880}, {105, 331, 4, 3880}, {106, 332, 12,
3880}, {107, 333, 12, 3880}, {108, 334, 4, 3880}, {112, 336, 1, 3880}, {113, 337, 4,
3880}, {114, 338, 6, 3880}, {115, 339, 4, 3880}, {116, 340, 1, 3880}, {90, 323, 1, 3881},
{94, 325, 4, 3881}, {95, 326, 4, 3881}, {99, 328, 6, 3881}, {100, 329, 12, 3881}, {101,
330, 6, 3881}, {105, 332, 4, 3881}, {106, 333, 12, 3881}, {107, 334, 12, 3881}, {108, 335,
4, 3881}, {112, 337, 1, 3881}, {113, 338, 4, 3881}, {114, 339, 6, 3881}, {115, 340, 4,
3881}, {116, 341, 1, 3881}, {91, 321, 1, 3882}, {95, 322, 4, 3882}, {96, 323, 4, 3882},
{100, 324, 6, 3882}, {101, 325, 12, 3882}, {102, 326, 6, 3882}, {106, 327, 4, 3882}, {107,
328, 12, 3882}, {108, 329, 12, 3882}, {109, 330, 4, 3882}, {113, 331, 1, 3882}, {114, 332,
4, 3882}, {115, 333, 6, 3882}, {116, 334, 4, 3882}, {117, 335, 1, 3882}, {91, 322, 1,
3883}, {95, 324, 4, 3883}, {96, 325, 4, 3883}, {100, 327, 6, 3883}, {101, 328, 12, 3883},
{102, 329, 6, 3883}, {106, 331, 4, 3883}, {107, 332, 12, 3883}, {108, 333, 12, 3883},
{109, 334, 4, 3883}, {113, 336, 1, 3883}, {114, 337, 4, 3883}, {115, 338, 6, 3883}, {116,
339, 4, 3883}, {117, 340, 1, 3883}, {91, 323, 1, 3884}, {95, 325, 4, 3884}, {96, 326, 4,
3884}, {100, 328, 6, 3884}, {101, 329, 12, 3884}, {102, 330, 6, 3884}, {106, 332, 4,
3884}, {107, 333, 12, 3884}, {108, 334, 12, 3884}, {109, 335, 4, 3884}, {113, 337, 1,
3884}, {114, 338, 4, 3884}, {115, 339, 6, 3884}, {116, 340, 4, 3884}, {117, 341, 1, 3884},
{92, 321, 1, 3885}, {96, 322, 4, 3885}, {97, 323, 4, 3885}, {101, 324, 6, 3885}, {102,
325, 12, 3885}, {103, 326, 6, 3885}, {107, 327, 4, 3885}, {108, 328, 12, 3885}, {109, 329,
12, 3885}, {110, 330, 4, 3885}, {114, 331, 1, 3885}, {115, 332, 4, 3885}, {116, 333, 6,
3885}, {117, 334, 4, 3885}, {118, 335, 1, 3885}, {92, 322, 1, 3886}, {96, 324, 4, 3886},
{97, 325, 4, 3886}, {101, 327, 6, 3886}, {102, 328, 12, 3886}, {103, 329, 6, 3886}, {107,
331, 4, 3886}, {108, 332, 12, 3886}, {109, 333, 12, 3886}, {110, 334, 4, 3886}, {114, 336,
1, 3886}, {115, 337, 4, 3886}, {116, 338, 6, 3886}, {117, 339, 4, 3886}, {118, 340, 1,

3886}, {92, 323, 1, 3887}, {96, 325, 4, 3887}, {97, 326, 4, 3887}, {101, 328, 6, 3887}, {102, 329, 12, 3887}, {103, 330, 6, 3887}, {107, 332, 4, 3887}, {108, 333, 12, 3887}, {109, 334, 12, 3887}, {110, 335, 4, 3887}, {114, 337, 1, 3887}, {115, 338, 4, 3887}, {116, 339, 6, 3887}, {117, 340, 4, 3887}, {118, 341, 1, 3887}, {93, 321, 1, 3888}, {97, 322, 4, 3888}, {98, 323, 4, 3888}, {102, 324, 6, 3888}, {103, 325, 12, 3888}, {104, 326, 6, 3888}, {108, 327, 4, 3888}, {109, 328, 12, 3888}, {110, 329, 12, 3888}, {111, 330, 4, 3888}, {115, 331, 1, 3888}, {116, 332, 4, 3888}, {117, 333, 6, 3888}, {118, 334, 4, 3888}, {119, 335, 1, 3888}, {93, 322, 1, 3889}, {97, 324, 4, 3889}, {98, 325, 4, 3889}, {102, 327, 6, 3889}, {103, 328, 12, 3889}, {104, 329, 6, 3889}, {108, 331, 4, 3889}, {109, 332, 12, 3889}, {110, 333, 12, 3889}, {111, 334, 4, 3889}, {115, 336, 1, 3889}, {116, 337, 4, 3889}, {117, 338, 6, 3889}, {118, 339, 4, 3889}, {119, 340, 1, 3889}, {93, 323, 1, 3890}, {97, 325, 4, 3890}, {98, 326, 4, 3890}, {102, 328, 6, 3890}, {103, 329, 12, 3890}, {104, 330, 6, 3890}, {108, 332, 4, 3890}, {109, 333, 12, 3890}, {110, 334, 12, 3890}, {111, 335, 4, 3890}, {115, 337, 1, 3890}, {116, 338, 4, 3890}, {117, 339, 6, 3890}, {118, 340, 4, 3890}, {119, 341, 1, 3890}, {84, 321, 1, 3891}, {85, 322, 5, 3891}, {86, 323, 5, 3891}, {87, 324, 10, 3891}, {88, 325, 20, 3891}, {89, 326, 10, 3891}, {90, 327, 10, 3891}, {91, 328, 30, 3891}, {92, 329, 30, 3891}, {93, 330, 10, 3891}, {94, 331, 5, 3891}, {95, 332, 20, 3891}, {96, 333, 30, 3891}, {97, 334, 20, 3891}, {98, 335, 5, 3891}, {99, 336, 1, 3891}, {100, 337, 5, 3891}, {101, 338, 10, 3891}, {102, 339, 10, 3891}, {103, 340, 5, 3891}, {104, 341, 1, 3891}, {85, 321, 1, 3892}, {87, 322, 5, 3892}, {88, 323, 5, 3892}, {90, 324, 10, 3892}, {91, 325, 20, 3892}, {92, 326, 10, 3892}, {94, 327, 10, 3892}, {95, 328, 30, 3892}, {96, 329, 30, 3892}, {97, 330, 10, 3892}, {99, 331, 5, 3892}, {100, 332, 20, 3892}, {101, 333, 30, 3892}, {102, 334, 20, 3892}, {103, 335, 5, 3892}, {105, 336, 1, 3892}, {106, 337, 5, 3892}, {107, 338, 10, 3892}, {108, 339, 10, 3892}, {109, 340, 5, 3892}, {110, 341, 1, 3892}, {86, 321, 1, 3893}, {88, 322, 5, 3893}, {89, 323, 5, 3893}, {91, 324, 10, 3893}, {92, 325, 20, 3893}, {93, 326, 10, 3893}, {95, 327, 10, 3893}, {96, 328, 30, 3893}, {97, 329, 30, 3893}, {98, 330, 10, 3893}, {100, 331, 5, 3893}, {101, 332, 20, 3893}, {102, 333, 30, 3893}, {103, 334, 20, 3893}, {104, 335, 5, 3893}, {106, 336, 1, 3893}, {107, 337, 5, 3893}, {108, 338, 10, 3893}, {109, 339, 10, 3893}, {110, 340, 5, 3893}, {111, 341, 1, 3893}, {87, 321, 1, 3894}, {90, 322, 5, 3894}, {91, 323, 5, 3894}, {94, 324, 10, 3894}, {95, 325, 20, 3894}, {96, 326, 10, 3894}, {99, 327, 10, 3894}, {100, 328, 30, 3894}, {101, 329, 30, 3894}, {102, 330, 10, 3894}, {105, 331, 5, 3894}, {106, 332, 20, 3894}, {107, 333, 30, 3894}, {108, 334, 20, 3894}, {109, 335, 5, 3894}, {112, 336, 1, 3894}, {113, 337, 5, 3894}, {114, 338, 10, 3894}, {115, 339, 10, 3894}, {116, 340, 5, 3894}, {117, 341, 1, 3894}, {88, 321, 1, 3895}, {91, 322, 5, 3895}, {92, 323, 5, 3895}, {95, 324, 10, 3895}, {96, 325, 20, 3895}, {97, 326, 10, 3895}, {100, 327, 10, 3895}, {101, 328, 30, 3895}, {102, 329, 30, 3895}, {103, 330, 10, 3895}, {106, 331, 5, 3895}, {107, 332, 20, 3895}, {108, 333, 30, 3895}, {109, 334, 20, 3895}, {110, 335, 5, 3895}, {113, 336, 1, 3895}, {114, 337, 5, 3895}, {115, 338, 10, 3895}, {116, 339, 10, 3895}, {117, 340, 5, 3895}, {118, 341, 1, 3895}, {89, 321, 1, 3896}, {92, 322, 5, 3896}, {93, 323, 5, 3896}, {96, 324, 10, 3896}, {97, 325, 20, 3896}, {98, 326, 10, 3896}, {101, 327, 10, 3896}, {102, 328, 30, 3896}, {103, 329, 30, 3896}, {104, 330, 10, 3896}, {107, 331, 5, 3896}, {108, 332, 20, 3896}, {109, 333, 30, 3896}, {110, 334, 20, 3896}, {111, 335, 5, 3896}, {114, 336, 1, 3896}, {115, 337, 5, 3896}, {116, 338, 10, 3896}, {117, 339, 10, 3896}, {118, 340, 5, 3896}, {119, 341, 1, 3896}, {84, 342, 1, 3897}, {85, 343, 5, 3897}, {86, 344, 5, 3897}, {87, 345, 10, 3897}, {88, 346, 20, 3897}, {89, 347, 10, 3897}, {90, 348, 10, 3897}, {91, 349, 30, 3897}, {92, 350, 30, 3897}, {93, 351, 10, 3897}, {94, 352, 5, 3897}, {95, 353, 20, 3897}, {96, 354, 30, 3897}, {97, 355, 20, 3897}, {98, 356, 5, 3897}, {99, 357, 1, 3897}, {100, 358, 5, 3897}, {101, 359, 10, 3897}, {102, 360, 10, 3897}, {103, 361, 5, 3897}, {104, 362, 1, 3897}, {84, 343, 1, 3898}, {85, 345, 5, 3898}, {86, 346, 5, 3898}, {87, 348, 10, 3898}, {88, 349, 20, 3898}, {89, 350, 10, 3898}, {90, 352, 10, 3898}, {91, 353, 30, 3898}, {92, 354, 30, 3898}, {93, 355, 10, 3898}, {94, 357, 5, 3898}, {95, 358, 20, 3898}, {96, 359, 30, 3898}, {97, 360, 20, 3898}, {98, 361, 5, 3898}, {99, 363, 1, 3898}, {100, 364, 5, 3898}, {101, 365, 10, 3898}, {102, 366, 10, 3898}, {103, 367, 5, 3898}, {104, 368, 1, 3898}, {84, 344, 1, 3899}, {85, 346, 5, 3899}, {86, 347, 5, 3899}, {87, 349, 10, 3899}, {88, 350, 20, 3899}, {89, 351, 10, 3899}, {90, 353, 10, 3899}, {91, 354, 30, 3899}, {92, 355, 30, 3899}, {93, 356, 10, 3899}, {94, 358, 5, 3899}, {95, 359, 20, 3899}, {96, 360, 30, 3899}, {97, 361, 20, 3899}, {98, 362, 5, 3899}, {99, 364, 1, 3899}, {100, 365, 5, 3899}, {101, 366, 10, 3899}, {102, 367, 10, 3899}, {103, 368, 5, 3899}, {104, 369, 1, 3899}, {85, 342, 1, 3900}, {87, 343, 5, 3900}, {88, 344, 5, 3900}, {90, 345, 10, 3900}, {91, 346, 20, 3900}, {92, 347, 10, 3900}, {94, 348, 10, 3900}, {95, 349, 30, 3900}, {96, 350, 30, 3900}, {97, 351, 10, 3900}, {99, 352, 5, 3900}, {100, 353, 20, 3900}, {101, 354, 30, 3900}, {102, 355, 20, 3900}, {103, 356, 5, 3900}, {105, 357, 1, 3900}, {106, 358, 5, 3900}, {107, 359, 10, 3900}, {108, 360, 10, 3900}, {109, 361, 5, 3900}, {110, 362, 1, 3900}, {85, 343, 1, 3901}, {87, 345, 5, 3901}, {88, 346, 5, 3901}, {90, 348, 10, 3901}, {91, 349, 20, 3901}, {92, 350, 10, 3901}, {94, 352, 10, 3901}, {95, 353, 30, 3901}, {96, 354, 30, 3901}, {97, 355, 10, 3901}, {99, 357, 5, 3901}, {100, 358, 20, 3901}, {101, 359, 30, 3901}, {102, 360, 20, 3901}, {103, 361, 5, 3901}, {105, 363, 1, 3901}, {106, 364, 5, 3901}, {107, 365, 10, 3901}, {108, 366, 10, 3901}, {109, 367, 5,

3901}, {110, 368, 1, 3901}, {85, 344, 1, 3902}, {87, 346, 5, 3902}, {88, 347, 5, 3902}, {90, 349, 10, 3902}, {91, 350, 20, 3902}, {92, 351, 10, 3902}, {94, 353, 10, 3902}, {95, 354, 30, 3902}, {96, 355, 30, 3902}, {97, 356, 10, 3902}, {99, 358, 5, 3902}, {100, 359, 20, 3902}, {101, 360, 30, 3902}, {102, 361, 20, 3902}, {103, 362, 5, 3902}, {105, 364, 1, 3902}, {106, 365, 5, 3902}, {107, 366, 10, 3902}, {108, 367, 10, 3902}, {109, 368, 5, 3902}, {110, 369, 1, 3902}, {86, 342, 1, 3903}, {88, 343, 5, 3903}, {89, 344, 5, 3903}, {91, 345, 10, 3903}, {92, 346, 20, 3903}, {93, 347, 10, 3903}, {95, 348, 10, 3903}, {96, 349, 30, 3903}, {97, 350, 30, 3903}, {98, 351, 10, 3903}, {100, 352, 5, 3903}, {101, 353, 20, 3903}, {102, 354, 30, 3903}, {103, 355, 20, 3903}, {104, 356, 5, 3903}, {106, 357, 1, 3903}, {107, 358, 5, 3903}, {108, 359, 10, 3903}, {109, 360, 10, 3903}, {110, 361, 5, 3903}, {111, 362, 1, 3903}, {86, 343, 1, 3904}, {88, 345, 5, 3904}, {89, 346, 5, 3904}, {91, 348, 10, 3904}, {92, 349, 20, 3904}, {93, 350, 10, 3904}, {95, 352, 10, 3904}, {96, 353, 30, 3904}, {97, 354, 30, 3904}, {98, 355, 10, 3904}, {100, 357, 5, 3904}, {101, 358, 20, 3904}, {102, 359, 30, 3904}, {103, 360, 20, 3904}, {104, 361, 5, 3904}, {106, 363, 1, 3904}, {107, 364, 5, 3904}, {108, 365, 10, 3904}, {109, 366, 10, 3904}, {110, 367, 5, 3904}, {111, 368, 1, 3904}, {86, 344, 1, 3905}, {88, 346, 5, 3905}, {89, 347, 5, 3905}, {91, 349, 10, 3905}, {92, 350, 20, 3905}, {93, 351, 10, 3905}, {95, 353, 10, 3905}, {96, 354, 30, 3905}, {97, 355, 30, 3905}, {98, 356, 10, 3905}, {100, 358, 5, 3905}, {101, 359, 20, 3905}, {102, 360, 30, 3905}, {103, 361, 20, 3905}, {104, 362, 5, 3905}, {106, 364, 1, 3905}, {107, 365, 5, 3905}, {108, 366, 10, 3905}, {109, 367, 10, 3905}, {110, 368, 5, 3905}, {111, 369, 1, 3905}, {87, 342, 1, 3906}, {90, 343, 5, 3906}, {91, 344, 5, 3906}, {94, 345, 10, 3906}, {95, 346, 20, 3906}, {96, 347, 10, 3906}, {99, 348, 10, 3906}, {100, 349, 30, 3906}, {101, 350, 30, 3906}, {102, 351, 10, 3906}, {105, 352, 5, 3906}, {106, 353, 20, 3906}, {107, 354, 30, 3906}, {108, 355, 20, 3906}, {109, 356, 5, 3906}, {112, 357, 1, 3906}, {113, 358, 5, 3906}, {114, 359, 10, 3906}, {115, 360, 10, 3906}, {116, 361, 5, 3906}, {117, 362, 1, 3906}, {87, 343, 1, 3907}, {90, 345, 5, 3907}, {91, 346, 5, 3907}, {94, 348, 10, 3907}, {95, 349, 20, 3907}, {96, 350, 10, 3907}, {99, 352, 10, 3907}, {100, 353, 30, 3907}, {101, 354, 30, 3907}, {102, 355, 10, 3907}, {105, 357, 5, 3907}, {106, 358, 20, 3907}, {107, 359, 30, 3907}, {108, 360, 20, 3907}, {109, 361, 5, 3907}, {112, 363, 1, 3907}, {113, 364, 5, 3907}, {114, 365, 10, 3907}, {115, 366, 10, 3907}, {116, 367, 5, 3907}, {117, 368, 1, 3907}, {87, 344, 1, 3908}, {90, 346, 5, 3908}, {91, 347, 5, 3908}, {94, 349, 10, 3908}, {95, 350, 20, 3908}, {96, 351, 10, 3908}, {99, 353, 10, 3908}, {100, 354, 30, 3908}, {101, 355, 30, 3908}, {102, 356, 10, 3908}, {105, 358, 5, 3908}, {106, 359, 20, 3908}, {107, 360, 30, 3908}, {108, 361, 20, 3908}, {109, 362, 5, 3908}, {112, 364, 1, 3908}, {113, 365, 5, 3908}, {114, 366, 10, 3908}, {115, 367, 10, 3908}, {116, 368, 5, 3908}, {117, 369, 1, 3908}, {88, 342, 1, 3909}, {91, 343, 5, 3909}, {92, 344, 5, 3909}, {95, 345, 10, 3909}, {96, 346, 20, 3909}, {97, 347, 10, 3909}, {100, 348, 10, 3909}, {101, 349, 30, 3909}, {102, 350, 30, 3909}, {103, 351, 10, 3909}, {106, 352, 5, 3909}, {107, 353, 20, 3909}, {108, 354, 30, 3909}, {109, 355, 20, 3909}, {110, 356, 5, 3909}, {113, 357, 1, 3909}, {114, 358, 5, 3909}, {115, 359, 10, 3909}, {116, 360, 10, 3909}, {117, 361, 5, 3909}, {118, 362, 1, 3909}, {88, 343, 1, 3910}, {91, 345, 5, 3910}, {92, 346, 5, 3910}, {95, 348, 10, 3910}, {96, 349, 20, 3910}, {97, 350, 10, 3910}, {100, 352, 10, 3910}, {101, 353, 30, 3910}, {102, 354, 30, 3910}, {103, 355, 10, 3910}, {106, 357, 5, 3910}, {107, 358, 20, 3910}, {108, 359, 30, 3910}, {109, 360, 20, 3910}, {110, 361, 5, 3910}, {113, 363, 1, 3910}, {114, 364, 5, 3910}, {115, 365, 10, 3910}, {116, 366, 10, 3910}, {117, 367, 5, 3910}, {118, 368, 1, 3910}, {88, 344, 1, 3911}, {91, 346, 5, 3911}, {92, 347, 5, 3911}, {95, 349, 10, 3911}, {96, 350, 20, 3911}, {97, 351, 10, 3911}, {100, 353, 10, 3911}, {101, 354, 30, 3911}, {102, 355, 30, 3911}, {103, 356, 10, 3911}, {106, 358, 5, 3911}, {107, 359, 20, 3911}, {108, 360, 30, 3911}, {109, 361, 20, 3911}, {110, 362, 5, 3911}, {113, 364, 1, 3911}, {114, 365, 5, 3911}, {115, 366, 10, 3911}, {116, 367, 10, 3911}, {117, 368, 5, 3911}, {118, 369, 1, 3911}, {89, 342, 1, 3912}, {92, 343, 5, 3912}, {93, 344, 5, 3912}, {96, 345, 10, 3912}, {97, 346, 20, 3912}, {98, 347, 10, 3912}, {101, 348, 10, 3912}, {102, 349, 30, 3912}, {103, 350, 30, 3912}, {104, 351, 10, 3912}, {107, 352, 5, 3912}, {108, 353, 20, 3912}, {109, 354, 30, 3912}, {110, 355, 20, 3912}, {111, 356, 5, 3912}, {114, 357, 1, 3912}, {115, 358, 5, 3912}, {116, 359, 10, 3912}, {117, 360, 10, 3912}, {118, 361, 5, 3912}, {119, 362, 1, 3912}, {89, 343, 1, 3913}, {92, 345, 5, 3913}, {93, 346, 5, 3913}, {96, 348, 10, 3913}, {97, 349, 20, 3913}, {98, 350, 10, 3913}, {101, 352, 10, 3913}, {102, 353, 30, 3913}, {103, 354, 30, 3913}, {104, 355, 10, 3913}, {107, 357, 5, 3913}, {108, 358, 20, 3913}, {109, 359, 30, 3913}, {110, 360, 20, 3913}, {111, 361, 5, 3913}, {114, 363, 1, 3913}, {115, 364, 5, 3913}, {116, 365, 10, 3913}, {117, 366, 10, 3913}, {118, 367, 5, 3913}, {119, 368, 1, 3913}, {89, 344, 1, 3914}, {92, 346, 5, 3914}, {93, 347, 5, 3914}, {96, 349, 10, 3914}, {97, 350, 20, 3914}, {98, 351, 10, 3914}, {101, 353, 10, 3914}, {102, 354, 30, 3914}, {103, 355, 30, 3914}, {104, 356, 10, 3914}, {107, 358, 5, 3914}, {108, 359, 20, 3914}, {109, 360, 30, 3914}, {110, 361, 20, 3914}, {111, 362, 5, 3914}, {114, 364, 1, 3914}, {115, 365, 5, 3914}, {116, 366, 10, 3914}, {117, 367, 10, 3914}, {118, 368, 5, 3914}, {119, 369, 1, 3914}, {84, 342, 1, 3915}, {85, 343, 6, 3915}, {86, 344, 6, 3915}, {87, 345, 15, 3915}, {88, 346, 30, 3915}, {89, 347, 15, 3915}, {90, 348, 20, 3915}, {91, 349, 60, 3915}, {92, 350, 60, 3915}, {93, 351, 20, 3915}, {94, 352, 15, 3915}, {95, 353, 60, 3915}, {96, 354, 90, 3915}, {97, 355, 60, 3915}, {98, 356, 15, 3915}, {99, 357, 6,

3915}, {100, 358, 30, 3915}, {101, 359, 60, 3915}, {102, 360, 60, 3915}, {103, 361, 30, 3915}, {104, 362, 6, 3915}, {105, 363, 1, 3915}, {106, 364, 6, 3915}, {107, 365, 15, 3915}, {108, 366, 20, 3915}, {109, 367, 15, 3915}, {110, 368, 6, 3915}, {111, 369, 1, 3915}, {85, 342, 1, 3916}, {87, 343, 6, 3916}, {88, 344, 6, 3916}, {90, 345, 15, 3916}, {91, 346, 30, 3916}, {92, 347, 15, 3916}, {94, 348, 20, 3916}, {95, 349, 60, 3916}, {96, 350, 60, 3916}, {97, 351, 20, 3916}, {99, 352, 15, 3916}, {100, 353, 60, 3916}, {101, 354, 90, 3916}, {102, 355, 60, 3916}, {103, 356, 15, 3916}, {105, 357, 6, 3916}, {106, 358, 30, 3916}, {107, 359, 60, 3916}, {108, 360, 60, 3916}, {109, 361, 30, 3916}, {110, 362, 6, 3916}, {112, 363, 1, 3916}, {113, 364, 6, 3916}, {114, 365, 15, 3916}, {115, 366, 20, 3916}, {116, 367, 15, 3916}, {117, 368, 6, 3916}, {118, 369, 1, 3916}, {86, 342, 1, 3917}, {88, 343, 6, 3917}, {89, 344, 6, 3917}, {91, 345, 15, 3917}, {92, 346, 30, 3917}, {93, 347, 15, 3917}, {95, 348, 20, 3917}, {96, 349, 60, 3917}, {97, 350, 60, 3917}, {98, 351, 20, 3917}, {100, 352, 15, 3917}, {101, 353, 60, 3917}, {102, 354, 90, 3917}, {103, 355, 60, 3917}, {104, 356, 15, 3917}, {106, 357, 6, 3917}, {107, 358, 30, 3917}, {108, 359, 60, 3917}, {109, 360, 60, 3917}, {110, 361, 30, 3917}, {111, 362, 6, 3917}, {113, 363, 1, 3917}, {114, 364, 6, 3917}, {115, 365, 15, 3917}, {116, 366, 20, 3917}, {117, 367, 15, 3917}, {118, 368, 6, 3917}, {119, 369, 1, 3917}, {84, 370, 1, 3918}, {85, 371, 6, 3918}, {86, 372, 6, 3918}, {87, 373, 15, 3918}, {88, 374, 30, 3918}, {89, 375, 15, 3918}, {90, 376, 20, 3918}, {91, 377, 60, 3918}, {92, 378, 60, 3918}, {93, 379, 20, 3918}, {94, 380, 15, 3918}, {95, 381, 60, 3918}, {96, 382, 90, 3918}, {97, 383, 60, 3918}, {98, 384, 15, 3918}, {99, 385, 6, 3918}, {100, 386, 30, 3918}, {101, 387, 60, 3918}, {102, 388, 60, 3918}, {103, 389, 30, 3918}, {104, 390, 6, 3918}, {105, 391, 1, 3918}, {106, 392, 6, 3918}, {107, 393, 15, 3918}, {108, 394, 20, 3918}, {109, 395, 15, 3918}, {110, 396, 6, 3918}, {111, 397, 1, 3918}, {84, 371, 1, 3919}, {85, 373, 6, 3919}, {86, 374, 6, 3919}, {87, 376, 15, 3919}, {88, 377, 30, 3919}, {89, 378, 15, 3919}, {90, 380, 20, 3919}, {91, 381, 60, 3919}, {92, 382, 60, 3919}, {93, 383, 20, 3919}, {94, 385, 15, 3919}, {95, 386, 60, 3919}, {96, 387, 90, 3919}, {97, 388, 60, 3919}, {98, 389, 15, 3919}, {99, 391, 6, 3919}, {100, 392, 30, 3919}, {101, 393, 60, 3919}, {102, 394, 60, 3919}, {103, 395, 30, 3919}, {104, 396, 6, 3919}, {105, 398, 1, 3919}, {106, 399, 6, 3919}, {107, 400, 15, 3919}, {108, 401, 20, 3919}, {109, 402, 15, 3919}, {110, 403, 6, 3919}, {111, 404, 1, 3919}, {84, 372, 1, 3920}, {85, 374, 6, 3920}, {86, 375, 6, 3920}, {87, 377, 15, 3920}, {88, 378, 30, 3920}, {89, 379, 15, 3920}, {90, 381, 20, 3920}, {91, 382, 60, 3920}, {92, 383, 60, 3920}, {93, 384, 20, 3920}, {94, 386, 15, 3920}, {95, 387, 60, 3920}, {96, 388, 90, 3920}, {97, 389, 60, 3920}, {98, 390, 15, 3920}, {99, 392, 6, 3920}, {100, 393, 30, 3920}, {101, 394, 60, 3920}, {102, 395, 60, 3920}, {103, 396, 30, 3920}, {104, 397, 6, 3920}, {105, 399, 1, 3920}, {106, 400, 6, 3920}, {107, 401, 15, 3920}, {108, 402, 20, 3920}, {109, 403, 15, 3920}, {110, 404, 6, 3920}, {111, 405, 1, 3920}, {85, 370, 1, 3921}, {87, 371, 6, 3921}, {88, 372, 6, 3921}, {90, 373, 15, 3921}, {91, 374, 30, 3921}, {92, 375, 15, 3921}, {94, 376, 20, 3921}, {95, 377, 60, 3921}, {96, 378, 60, 3921}, {97, 379, 20, 3921}, {99, 380, 15, 3921}, {100, 381, 60, 3921}, {101, 382, 90, 3921}, {102, 383, 60, 3921}, {103, 384, 15, 3921}, {105, 385, 6, 3921}, {106, 386, 30, 3921}, {107, 387, 60, 3921}, {108, 388, 60, 3921}, {109, 389, 30, 3921}, {110, 390, 6, 3921}, {112, 391, 1, 3921}, {113, 392, 6, 3921}, {114, 393, 15, 3921}, {115, 394, 20, 3921}, {116, 395, 15, 3921}, {117, 396, 6, 3921}, {118, 397, 1, 3921}, {85, 371, 1, 3922}, {87, 373, 6, 3922}, {88, 374, 6, 3922}, {90, 376, 15, 3922}, {91, 377, 30, 3922}, {92, 378, 15, 3922}, {94, 380, 20, 3922}, {95, 381, 60, 3922}, {96, 382, 60, 3922}, {97, 383, 20, 3922}, {99, 385, 15, 3922}, {100, 386, 60, 3922}, {101, 387, 90, 3922}, {102, 388, 60, 3922}, {103, 389, 15, 3922}, {105, 391, 6, 3922}, {106, 392, 30, 3922}, {107, 393, 60, 3922}, {108, 394, 60, 3922}, {109, 395, 30, 3922}, {110, 396, 6, 3922}, {112, 398, 1, 3922}, {113, 399, 6, 3922}, {114, 400, 15, 3922}, {115, 401, 20, 3922}, {116, 402, 15, 3922}, {117, 403, 6, 3922}, {118, 404, 1, 3922}, {85, 372, 1, 3923}, {87, 374, 6, 3923}, {88, 375, 6, 3923}, {90, 377, 15, 3923}, {91, 378, 30, 3923}, {92, 379, 15, 3923}, {94, 381, 20, 3923}, {95, 382, 60, 3923}, {96, 383, 60, 3923}, {97, 384, 20, 3923}, {99, 386, 15, 3923}, {100, 387, 60, 3923}, {101, 388, 90, 3923}, {102, 389, 60, 3923}, {103, 390, 15, 3923}, {105, 392, 6, 3923}, {106, 393, 30, 3923}, {107, 394, 60, 3923}, {108, 395, 60, 3923}, {109, 396, 30, 3923}, {110, 397, 6, 3923}, {112, 399, 1, 3923}, {113, 400, 6, 3923}, {114, 401, 15, 3923}, {115, 402, 20, 3923}, {116, 403, 15, 3923}, {117, 404, 6, 3923}, {118, 405, 1, 3923}, {86, 370, 1, 3924}, {88, 371, 6, 3924}, {89, 372, 6, 3924}, {91, 373, 15, 3924}, {92, 374, 30, 3924}, {93, 375, 15, 3924}, {95, 376, 20, 3924}, {96, 377, 60, 3924}, {97, 378, 60, 3924}, {98, 379, 20, 3924}, {100, 380, 15, 3924}, {101, 381, 60, 3924}, {102, 382, 90, 3924}, {103, 383, 60, 3924}, {104, 384, 15, 3924}, {106, 385, 6, 3924}, {107, 386, 30, 3924}, {108, 387, 60, 3924}, {109, 388, 60, 3924}, {110, 389, 30, 3924}, {111, 390, 6, 3924}, {113, 391, 1, 3924}, {114, 392, 6, 3924}, {115, 393, 15, 3924}, {116, 394, 20, 3924}, {117, 395, 15, 3924}, {118, 396, 6, 3924}, {119, 397, 1, 3924}, {86, 371, 1, 3925}, {88, 373, 6, 3925}, {89, 374, 6, 3925}, {91, 376, 15, 3925}, {92, 377, 30, 3925}, {93, 378, 15, 3925}, {95, 380, 20, 3925}, {96, 381, 60, 3925}, {97, 382, 60, 3925}, {98, 383, 20, 3925}, {100, 385, 15, 3925}, {101, 386, 60, 3925}, {102, 387, 90, 3925}, {103, 388, 60, 3925}, {104, 389, 15, 3925}, {106, 391, 6, 3925}, {107, 392, 30, 3925}, {108, 393, 60, 3925}, {109, 394, 60, 3925}, {110, 395, 30, 3925}, {111, 396, 6, 3925}, {113,

398, 1, 3925}, {114, 399, 6, 3925}, {115, 400, 15, 3925}, {116, 401, 20, 3925}, {117, 402, 15, 3925}, {118, 403, 6, 3925}, {119, 404, 1, 3925}, {86, 372, 1, 3926}, {88, 374, 6, 3926}, {89, 375, 6, 3926}, {91, 377, 15, 3926}, {92, 378, 30, 3926}, {93, 379, 15, 3926}, {95, 381, 20, 3926}, {96, 382, 60, 3926}, {97, 383, 60, 3926}, {98, 384, 20, 3926}, {100, 386, 15, 3926}, {101, 387, 60, 3926}, {102, 388, 90, 3926}, {103, 389, 60, 3926}, {104, 390, 15, 3926}, {106, 392, 6, 3926}, {107, 393, 30, 3926}, {108, 394, 60, 3926}, {109, 395, 60, 3926}, {110, 396, 30, 3926}, {111, 397, 6, 3926}, {113, 399, 1, 3926}, {114, 400, 6, 3926}, {115, 401, 15, 3926}, {116, 402, 20, 3926}, {117, 403, 15, 3926}, {118, 404, 6, 3926}, {119, 405, 1, 3926}, {84, 370, 1, 3927}, {85, 371, 7, 3927}, {86, 372, 7, 3927}, {87, 373, 21, 3927}, {88, 374, 42, 3927}, {89, 375, 21, 3927}, {90, 376, 35, 3927}, {91, 377, 105, 3927}, {92, 378, 105, 3927}, {93, 379, 35, 3927}, {94, 380, 35, 3927}, {95, 381, 140, 3927}, {96, 382, 210, 3927}, {97, 383, 140, 3927}, {98, 384, 35, 3927}, {99, 385, 21, 3927}, {100, 386, 105, 3927}, {101, 387, 210, 3927}, {102, 388, 210, 3927}, {103, 389, 105, 3927}, {104, 390, 21, 3927}, {105, 391, 7, 3927}, {106, 392, 42, 3927}, {107, 393, 105, 3927}, {108, 394, 140, 3927}, {109, 395, 105, 3927}, {110, 396, 42, 3927}, {111, 397, 7, 3927}, {112, 398, 1, 3927}, {113, 399, 7, 3927}, {114, 400, 21, 3927}, {115, 401, 35, 3927}, {116, 402, 35, 3927}, {117, 403, 21, 3927}, {118, 404, 7, 3927}, {119, 405, 1, 3927}, {84, 406, 1, 3928}, {85, 407, 7, 3928}, {86, 408, 7, 3928}, {87, 409, 21, 3928}, {88, 410, 42, 3928}, {89, 411, 21, 3928}, {90, 412, 35, 3928}, {91, 413, 105, 3928}, {92, 414, 105, 3928}, {93, 415, 35, 3928}, {94, 416, 35, 3928}, {95, 417, 140, 3928}, {96, 418, 210, 3928}, {97, 419, 140, 3928}, {98, 420, 35, 3928}, {99, 421, 21, 3928}, {100, 422, 105, 3928}, {101, 423, 210, 3928}, {102, 424, 210, 3928}, {103, 425, 105, 3928}, {104, 426, 21, 3928}, {105, 427, 7, 3928}, {106, 428, 42, 3928}, {107, 429, 105, 3928}, {108, 430, 140, 3928}, {109, 431, 105, 3928}, {110, 432, 42, 3928}, {111, 433, 7, 3928}, {112, 434, 1, 3928}, {113, 435, 7, 3928}, {114, 436, 21, 3928}, {115, 437, 35, 3928}, {116, 438, 35, 3928}, {117, 439, 21, 3928}, {118, 440, 7, 3928}, {119, 441, 1, 3928}, {84, 407, 1, 3929}, {85, 409, 7, 3929}, {86, 410, 7, 3929}, {87, 412, 21, 3929}, {88, 413, 42, 3929}, {89, 414, 21, 3929}, {90, 416, 35, 3929}, {91, 417, 105, 3929}, {92, 418, 105, 3929}, {93, 419, 35, 3929}, {94, 421, 35, 3929}, {95, 422, 140, 3929}, {96, 423, 210, 3929}, {97, 424, 140, 3929}, {98, 425, 35, 3929}, {99, 427, 21, 3929}, {100, 428, 105, 3929}, {101, 429, 210, 3929}, {102, 430, 210, 3929}, {103, 431, 105, 3929}, {104, 432, 21, 3929}, {105, 434, 7, 3929}, {106, 435, 42, 3929}, {107, 436, 105, 3929}, {108, 437, 140, 3929}, {109, 438, 105, 3929}, {110, 439, 42, 3929}, {111, 440, 7, 3929}, {112, 442, 1, 3929}, {113, 443, 7, 3929}, {114, 444, 21, 3929}, {115, 445, 35, 3929}, {116, 446, 35, 3929}, {117, 447, 21, 3929}, {118, 448, 7, 3929}, {119, 449, 1, 3929}, {84, 408, 1, 3930}, {85, 410, 7, 3930}, {86, 411, 7, 3930}, {87, 413, 21, 3930}, {88, 414, 42, 3930}, {89, 415, 21, 3930}, {90, 417, 35, 3930}, {91, 418, 105, 3930}, {92, 419, 105, 3930}, {93, 420, 35, 3930}, {94, 422, 35, 3930}, {95, 423, 140, 3930}, {96, 424, 210, 3930}, {97, 425, 140, 3930}, {98, 426, 35, 3930}, {99, 428, 21, 3930}, {100, 429, 105, 3930}, {101, 430, 210, 3930}, {102, 431, 210, 3930}, {103, 432, 105, 3930}, {104, 433, 21, 3930}, {105, 435, 7, 3930}, {106, 436, 42, 3930}, {107, 437, 105, 3930}, {108, 438, 140, 3930}, {109, 439, 105, 3930}, {110, 440, 42, 3930}, {111, 441, 7, 3930}, {112, 443, 1, 3930}, {113, 444, 7, 3930}, {114, 445, 21, 3930}, {115, 446, 35, 3930}, {116, 447, 35, 3930}, {117, 448, 21, 3930}, {118, 449, 7, 3930}, {119, 450, 1, 3930}, {120, 306, 1, 3931}, {121, 307, 4, 3931}, {122, 308, 4, 3931}, {123, 309, 6, 3931}, {124, 310, 12, 3931}, {125, 311, 6, 3931}, {126, 312, 4, 3931}, {127, 313, 12, 3931}, {128, 314, 12, 3931}, {129, 315, 4, 3931}, {130, 316, 1, 3931}, {131, 317, 4, 3931}, {132, 318, 6, 3931}, {133, 319, 4, 3931}, {134, 320, 1, 3931}, {121, 306, 1, 3932}, {123, 307, 4, 3932}, {124, 308, 4, 3932}, {126, 309, 6, 3932}, {127, 310, 12, 3932}, {128, 311, 6, 3932}, {130, 312, 4, 3932}, {131, 313, 12, 3932}, {132, 314, 12, 3932}, {133, 315, 4, 3932}, {135, 316, 1, 3932}, {136, 317, 4, 3932}, {137, 318, 6, 3932}, {138, 319, 4, 3932}, {139, 320, 1, 3932}, {122, 306, 1, 3933}, {124, 307, 4, 3933}, {125, 308, 4, 3933}, {127, 309, 6, 3933}, {128, 310, 12, 3933}, {129, 311, 6, 3933}, {131, 312, 4, 3933}, {132, 313, 12, 3933}, {133, 314, 12, 3933}, {134, 315, 4, 3933}, {136, 316, 1, 3933}, {137, 317, 4, 3933}, {138, 318, 6, 3933}, {139, 319, 4, 3933}, {140, 320, 1, 3933}, {123, 306, 1, 3934}, {126, 307, 4, 3934}, {127, 308, 4, 3934}, {130, 309, 6, 3934}, {131, 310, 12, 3934}, {132, 311, 6, 3934}, {135, 312, 4, 3934}, {136, 313, 12, 3934}, {137, 314, 12, 3934}, {138, 315, 4, 3934}, {141, 316, 1, 3934}, {142, 317, 4, 3934}, {143, 318, 6, 3934}, {144, 319, 4, 3934}, {145, 320, 1, 3934}, {124, 306, 1, 3935}, {127, 307, 4, 3935}, {128, 308, 4, 3935}, {131, 309, 6, 3935}, {132, 310, 12, 3935}, {133, 311, 6, 3935}, {136, 312, 4, 3935}, {137, 313, 12, 3935}, {138, 314, 12, 3935}, {139, 315, 4, 3935}, {142, 316, 1, 3935}, {143, 317, 4, 3935}, {144, 318, 6, 3935}, {145, 319, 4, 3935}, {146, 320, 1, 3935}, {125, 306, 1, 3936}, {128, 307, 4, 3936}, {129, 308, 4, 3936}, {132, 309, 6, 3936}, {133, 310, 12, 3936}, {134, 311, 6, 3936}, {137, 312, 4, 3936}, {138, 313, 12, 3936}, {139, 314, 12, 3936}, {140, 315, 4, 3936}, {143, 316, 1, 3936}, {144, 317, 4, 3936}, {145, 318, 6, 3936}, {146, 319, 4, 3936}, {147, 320, 1, 3936}, {126, 306, 1, 3937}, {130, 307, 4, 3937}, {131, 308, 4, 3937}, {135, 309, 6, 3937}, {136, 310, 12, 3937}, {137, 311, 6, 3937}, {141, 312, 4, 3937}, {142, 313, 12, 3937}, {143, 314, 12, 3937}, {144, 315, 4, 3937}, {148, 316, 1, 3937}, {149, 317, 4, 3937}, {150, 318, 6, 3937}, {151, 319, 4, 3937}, {152, 320, 1, 3937}, {127, 306, 1, 3938}, {131, 307, 4, 3938}, {132, 308, 4, 3938}, {136,

309, 6, 3938}, {137, 310, 12, 3938}, {138, 311, 6, 3938}, {142, 312, 4, 3938}, {143, 313, 12, 3938}, {144, 314, 12, 3938}, {145, 315, 4, 3938}, {149, 316, 1, 3938}, {150, 317, 4, 3938}, {151, 318, 6, 3938}, {152, 319, 4, 3938}, {153, 320, 1, 3938}, {128, 306, 1, 3939}, {132, 307, 4, 3939}, {133, 308, 4, 3939}, {137, 309, 6, 3939}, {138, 310, 12, 3939}, {139, 311, 6, 3939}, {143, 312, 4, 3939}, {144, 313, 12, 3939}, {145, 314, 12, 3939}, {146, 315, 4, 3939}, {150, 316, 1, 3939}, {151, 317, 4, 3939}, {152, 318, 6, 3939}, {153, 319, 4, 3939}, {154, 320, 1, 3939}, {129, 306, 1, 3940}, {133, 307, 4, 3940}, {134, 308, 4, 3940}, {138, 309, 6, 3940}, {139, 310, 12, 3940}, {140, 311, 6, 3940}, {144, 312, 4, 3940}, {145, 313, 12, 3940}, {146, 314, 12, 3940}, {147, 315, 4, 3940}, {151, 316, 1, 3940}, {152, 317, 4, 3940}, {153, 318, 6, 3940}, {154, 319, 4, 3940}, {155, 320, 1, 3940}, {130, 306, 1, 3941}, {135, 307, 4, 3941}, {136, 308, 4, 3941}, {141, 309, 6, 3941}, {142, 310, 12, 3941}, {143, 311, 6, 3941}, {148, 312, 4, 3941}, {149, 313, 12, 3941}, {150, 314, 12, 3941}, {151, 315, 4, 3941}, {156, 316, 1, 3941}, {157, 317, 4, 3941}, {158, 318, 6, 3941}, {159, 319, 4, 3941}, {160, 320, 1, 3941}, {131, 306, 1, 3942}, {136, 307, 4, 3942}, {137, 308, 4, 3942}, {142, 309, 6, 3942}, {143, 310, 12, 3942}, {144, 311, 6, 3942}, {149, 312, 4, 3942}, {150, 313, 12, 3942}, {151, 314, 12, 3942}, {152, 315, 4, 3942}, {157, 316, 1, 3942}, {158, 317, 4, 3942}, {159, 318, 6, 3942}, {160, 319, 4, 3942}, {161, 320, 1, 3942}, {132, 306, 1, 3943}, {137, 307, 4, 3943}, {138, 308, 4, 3943}, {143, 309, 6, 3943}, {144, 310, 12, 3943}, {145, 311, 6, 3943}, {150, 312, 4, 3943}, {151, 313, 12, 3943}, {152, 314, 12, 3943}, {153, 315, 4, 3943}, {158, 316, 1, 3943}, {159, 317, 4, 3943}, {160, 318, 6, 3943}, {161, 319, 4, 3943}, {162, 320, 1, 3943}, {133, 306, 1, 3944}, {138, 307, 4, 3944}, {139, 308, 4, 3944}, {144, 309, 6, 3944}, {145, 310, 12, 3944}, {146, 311, 6, 3944}, {151, 312, 4, 3944}, {152, 313, 12, 3944}, {153, 314, 12, 3944}, {154, 315, 4, 3944}, {159, 316, 1, 3944}, {160, 317, 4, 3944}, {161, 318, 6, 3944}, {162, 319, 4, 3944}, {163, 320, 1, 3944}, {134, 306, 1, 3945}, {139, 307, 4, 3945}, {140, 308, 4, 3945}, {145, 309, 6, 3945}, {146, 310, 12, 3945}, {147, 311, 6, 3945}, {152, 312, 4, 3945}, {153, 313, 12, 3945}, {154, 314, 12, 3945}, {155, 315, 4, 3945}, {160, 316, 1, 3945}, {161, 317, 4, 3945}, {162, 318, 6, 3945}, {163, 319, 4, 3945}, {164, 320, 1, 3945}, {120, 321, 1, 3946}, {121, 322, 5, 3946}, {122, 323, 5, 3946}, {123, 324, 10, 3946}, {124, 325, 20, 3946}, {125, 326, 10, 3946}, {126, 327, 10, 3946}, {127, 328, 30, 3946}, {128, 329, 30, 3946}, {129, 330, 10, 3946}, {130, 331, 5, 3946}, {131, 332, 20, 3946}, {132, 333, 30, 3946}, {133, 334, 20, 3946}, {134, 335, 5, 3946}, {135, 336, 1, 3946}, {136, 337, 5, 3946}, {137, 338, 10, 3946}, {138, 339, 10, 3946}, {139, 340, 5, 3946}, {140, 341, 1, 3946}, {121, 321, 1, 3947}, {123, 322, 5, 3947}, {124, 323, 5, 3947}, {126, 324, 10, 3947}, {127, 325, 20, 3947}, {128, 326, 10, 3947}, {130, 327, 10, 3947}, {131, 328, 30, 3947}, {132, 329, 30, 3947}, {133, 330, 10, 3947}, {135, 331, 5, 3947}, {136, 332, 20, 3947}, {137, 333, 30, 3947}, {138, 334, 20, 3947}, {139, 335, 5, 3947}, {141, 336, 1, 3947}, {142, 337, 5, 3947}, {143, 338, 10, 3947}, {144, 339, 10, 3947}, {145, 340, 5, 3947}, {146, 341, 1, 3947}, {122, 321, 1, 3948}, {124, 322, 5, 3948}, {125, 323, 5, 3948}, {127, 324, 10, 3948}, {128, 325, 20, 3948}, {129, 326, 10, 3948}, {131, 327, 10, 3948}, {132, 328, 30, 3948}, {133, 329, 30, 3948}, {134, 330, 10, 3948}, {136, 331, 5, 3948}, {137, 332, 20, 3948}, {138, 333, 30, 3948}, {139, 334, 20, 3948}, {140, 335, 5, 3948}, {142, 336, 1, 3948}, {143, 337, 5, 3948}, {144, 338, 10, 3948}, {145, 339, 10, 3948}, {146, 340, 5, 3948}, {147, 341, 1, 3948}, {123, 321, 1, 3949}, {126, 322, 5, 3949}, {127, 323, 5, 3949}, {130, 324, 10, 3949}, {131, 325, 20, 3949}, {132, 326, 10, 3949}, {135, 327, 10, 3949}, {136, 328, 30, 3949}, {137, 329, 30, 3949}, {138, 330, 10, 3949}, {141, 331, 5, 3949}, {142, 332, 20, 3949}, {143, 333, 30, 3949}, {144, 334, 20, 3949}, {145, 335, 5, 3949}, {148, 336, 1, 3949}, {149, 337, 5, 3949}, {150, 338, 10, 3949}, {151, 339, 10, 3949}, {152, 340, 5, 3949}, {153, 341, 1, 3949}, {124, 321, 1, 3950}, {127, 322, 5, 3950}, {128, 323, 5, 3950}, {131, 324, 10, 3950}, {132, 325, 20, 3950}, {133, 326, 10, 3950}, {136, 327, 10, 3950}, {137, 328, 30, 3950}, {138, 329, 30, 3950}, {139, 330, 10, 3950}, {142, 331, 5, 3950}, {143, 332, 20, 3950}, {144, 333, 30, 3950}, {145, 334, 20, 3950}, {146, 335, 5, 3950}, {149, 336, 1, 3950}, {150, 337, 5, 3950}, {151, 338, 10, 3950}, {152, 339, 10, 3950}, {153, 340, 5, 3950}, {154, 341, 1, 3950}, {125, 321, 1, 3951}, {128, 322, 5, 3951}, {129, 323, 5, 3951}, {132, 324, 10, 3951}, {133, 325, 20, 3951}, {134, 326, 10, 3951}, {137, 327, 10, 3951}, {138, 328, 30, 3951}, {139, 329, 30, 3951}, {140, 330, 10, 3951}, {143, 331, 5, 3951}, {144, 332, 20, 3951}, {145, 333, 30, 3951}, {146, 334, 20, 3951}, {147, 335, 5, 3951}, {150, 336, 1, 3951}, {151, 337, 5, 3951}, {152, 338, 10, 3951}, {153, 339, 10, 3951}, {154, 340, 5, 3951}, {155, 341, 1, 3951}, {126, 321, 1, 3952}, {130, 322, 5, 3952}, {131, 323, 5, 3952}, {135, 324, 10, 3952}, {136, 325, 20, 3952}, {137, 326, 10, 3952}, {141, 327, 10, 3952}, {142, 328, 30, 3952}, {143, 329, 30, 3952}, {144, 330, 10, 3952}, {148, 331, 5, 3952}, {149, 332, 20, 3952}, {150, 333, 30, 3952}, {151, 334, 20, 3952}, {152, 335, 5, 3952}, {156, 336, 1, 3952}, {157, 337, 5, 3952}, {158, 338, 10, 3952}, {159, 339, 10, 3952}, {160, 340, 5, 3952}, {161, 341, 1, 3952}, {127, 321, 1, 3953}, {131, 322, 5, 3953}, {132, 323, 5, 3953}, {136, 324, 10, 3953}, {137, 325, 20, 3953}, {138, 326, 10, 3953}, {142, 327, 10, 3953}, {143, 328, 30, 3953}, {144, 329, 30, 3953}, {145, 330, 10, 3953}, {149, 331, 5, 3953}, {150, 332, 20, 3953}, {151, 333, 30, 3953}, {152, 334, 20, 3953}, {153, 335, 5, 3953}, {157, 336, 1, 3953}, {158, 337, 5, 3953}, {159, 338, 10, 3953}, {160, 339, 10, 3953}, {161, 340, 5,

3953}, {162, 341, 1, 3953}, {128, 321, 1, 3954}, {132, 322, 5, 3954}, {133, 323, 5, 3954}, {137, 324, 10, 3954}, {138, 325, 20, 3954}, {139, 326, 10, 3954}, {143, 327, 10, 3954}, {144, 328, 30, 3954}, {145, 329, 30, 3954}, {146, 330, 10, 3954}, {150, 331, 5, 3954}, {151, 332, 20, 3954}, {152, 333, 30, 3954}, {153, 334, 20, 3954}, {154, 335, 5, 3954}, {158, 336, 1, 3954}, {159, 337, 5, 3954}, {160, 338, 10, 3954}, {161, 339, 10, 3954}, {162, 340, 5, 3954}, {163, 341, 1, 3954}, {129, 321, 1, 3955}, {133, 322, 5, 3955}, {134, 323, 5, 3955}, {138, 324, 10, 3955}, {139, 325, 20, 3955}, {140, 326, 10, 3955}, {144, 327, 10, 3955}, {145, 328, 30, 3955}, {146, 329, 30, 3955}, {147, 330, 10, 3955}, {151, 331, 5, 3955}, {152, 332, 20, 3955}, {153, 333, 30, 3955}, {154, 334, 20, 3955}, {155, 335, 5, 3955}, {159, 336, 1, 3955}, {160, 337, 5, 3955}, {161, 338, 10, 3955}, {162, 339, 10, 3955}, {163, 340, 5, 3955}, {164, 341, 1, 3955}, {120, 342, 1, 3956}, {121, 343, 6, 3956}, {122, 344, 6, 3956}, {123, 345, 15, 3956}, {124, 346, 30, 3956}, {125, 347, 15, 3956}, {126, 348, 20, 3956}, {127, 349, 60, 3956}, {128, 350, 60, 3956}, {129, 351, 20, 3956}, {130, 352, 15, 3956}, {131, 353, 60, 3956}, {132, 354, 90, 3956}, {133, 355, 60, 3956}, {134, 356, 15, 3956}, {135, 357, 6, 3956}, {136, 358, 30, 3956}, {137, 359, 60, 3956}, {138, 360, 60, 3956}, {139, 361, 30, 3956}, {140, 362, 6, 3956}, {141, 363, 1, 3956}, {142, 364, 6, 3956}, {143, 365, 15, 3956}, {144, 366, 20, 3956}, {145, 367, 15, 3956}, {146, 368, 6, 3956}, {147, 369, 1, 3956}, {121, 342, 1, 3957}, {123, 343, 6, 3957}, {124, 344, 6, 3957}, {126, 345, 15, 3957}, {127, 346, 30, 3957}, {128, 347, 15, 3957}, {130, 348, 20, 3957}, {131, 349, 60, 3957}, {132, 350, 60, 3957}, {133, 351, 20, 3957}, {135, 352, 15, 3957}, {136, 353, 60, 3957}, {137, 354, 90, 3957}, {138, 355, 60, 3957}, {139, 356, 15, 3957}, {141, 357, 6, 3957}, {142, 358, 30, 3957}, {143, 359, 60, 3957}, {144, 360, 60, 3957}, {145, 361, 30, 3957}, {146, 362, 6, 3957}, {148, 363, 1, 3957}, {149, 364, 6, 3957}, {150, 365, 15, 3957}, {151, 366, 20, 3957}, {152, 367, 15, 3957}, {153, 368, 6, 3957}, {154, 369, 1, 3957}, {122, 342, 1, 3958}, {124, 343, 6, 3958}, {125, 344, 6, 3958}, {127, 345, 15, 3958}, {128, 346, 30, 3958}, {129, 347, 15, 3958}, {131, 348, 20, 3958}, {132, 349, 60, 3958}, {133, 350, 60, 3958}, {134, 351, 20, 3958}, {136, 352, 15, 3958}, {137, 353, 60, 3958}, {138, 354, 90, 3958}, {139, 355, 60, 3958}, {140, 356, 15, 3958}, {142, 357, 6, 3958}, {143, 358, 30, 3958}, {144, 359, 60, 3958}, {145, 360, 60, 3958}, {146, 361, 30, 3958}, {147, 362, 6, 3958}, {149, 363, 1, 3958}, {150, 364, 6, 3958}, {151, 365, 15, 3958}, {152, 366, 20, 3958}, {153, 367, 15, 3958}, {154, 368, 6, 3958}, {155, 369, 1, 3958}, {123, 342, 1, 3959}, {126, 343, 6, 3959}, {127, 344, 6, 3959}, {130, 345, 15, 3959}, {131, 346, 30, 3959}, {132, 347, 15, 3959}, {135, 348, 20, 3959}, {136, 349, 60, 3959}, {137, 350, 60, 3959}, {138, 351, 20, 3959}, {141, 352, 15, 3959}, {142, 353, 60, 3959}, {143, 354, 90, 3959}, {144, 355, 60, 3959}, {145, 356, 15, 3959}, {148, 357, 6, 3959}, {149, 358, 30, 3959}, {150, 359, 60, 3959}, {151, 360, 60, 3959}, {152, 361, 30, 3959}, {153, 362, 6, 3959}, {156, 363, 1, 3959}, {157, 364, 6, 3959}, {158, 365, 15, 3959}, {159, 366, 20, 3959}, {160, 367, 15, 3959}, {161, 368, 6, 3959}, {162, 369, 1, 3959}, {124, 342, 1, 3960}, {127, 343, 6, 3960}, {128, 344, 6, 3960}, {131, 345, 15, 3960}, {132, 346, 30, 3960}, {133, 347, 15, 3960}, {136, 348, 20, 3960}, {137, 349, 60, 3960}, {138, 350, 60, 3960}, {139, 351, 20, 3960}, {142, 352, 15, 3960}, {143, 353, 60, 3960}, {144, 354, 90, 3960}, {145, 355, 60, 3960}, {146, 356, 15, 3960}, {149, 357, 6, 3960}, {150, 358, 30, 3960}, {151, 359, 60, 3960}, {152, 360, 60, 3960}, {153, 361, 30, 3960}, {154, 362, 6, 3960}, {157, 363, 1, 3960}, {158, 364, 6, 3960}, {159, 365, 15, 3960}, {160, 366, 20, 3960}, {161, 367, 15, 3960}, {162, 368, 6, 3960}, {163, 369, 1, 3960}, {125, 342, 1, 3961}, {128, 343, 6, 3961}, {129, 344, 6, 3961}, {132, 345, 15, 3961}, {133, 346, 30, 3961}, {134, 347, 15, 3961}, {137, 348, 20, 3961}, {138, 349, 60, 3961}, {139, 350, 60, 3961}, {140, 351, 20, 3961}, {143, 352, 15, 3961}, {144, 353, 60, 3961}, {145, 354, 90, 3961}, {146, 355, 60, 3961}, {147, 356, 15, 3961}, {150, 357, 6, 3961}, {151, 358, 30, 3961}, {152, 359, 60, 3961}, {153, 360, 60, 3961}, {154, 361, 30, 3961}, {155, 362, 6, 3961}, {158, 363, 1, 3961}, {159, 364, 6, 3961}, {160, 365, 15, 3961}, {161, 366, 20, 3961}, {162, 367, 15, 3961}, {163, 368, 6, 3961}, {164, 369, 1, 3961}, {120, 370, 1, 3962}, {121, 371, 7, 3962}, {122, 372, 7, 3962}, {123, 373, 21, 3962}, {124, 374, 42, 3962}, {125, 375, 21, 3962}, {126, 376, 35, 3962}, {127, 377, 105, 3962}, {128, 378, 105, 3962}, {129, 379, 35, 3962}, {130, 380, 35, 3962}, {131, 381, 140, 3962}, {132, 382, 210, 3962}, {133, 383, 140, 3962}, {134, 384, 35, 3962}, {135, 385, 21, 3962}, {136, 386, 105, 3962}, {137, 387, 210, 3962}, {138, 388, 210, 3962}, {139, 389, 105, 3962}, {140, 390, 21, 3962}, {141, 391, 7, 3962}, {142, 392, 42, 3962}, {143, 393, 105, 3962}, {144, 394, 140, 3962}, {145, 395, 105, 3962}, {146, 396, 42, 3962}, {147, 397, 7, 3962}, {148, 398, 1, 3962}, {149, 399, 7, 3962}, {150, 400, 21, 3962}, {151, 401, 35, 3962}, {152, 402, 35, 3962}, {153, 403, 21, 3962}, {154, 404, 7, 3962}, {155, 405, 1, 3962}, {121, 370, 1, 3963}, {123, 371, 7, 3963}, {124, 372, 7, 3963}, {126, 373, 21, 3963}, {127, 374, 42, 3963}, {128, 375, 21, 3963}, {130, 376, 35, 3963}, {131, 377, 105, 3963}, {132, 378, 105, 3963}, {133, 379, 35, 3963}, {135, 380, 35, 3963}, {136, 381, 140, 3963}, {137, 382, 210, 3963}, {138, 383, 140, 3963}, {139, 384, 35, 3963}, {141, 385, 21, 3963}, {142, 386, 105, 3963}, {143, 387, 210, 3963}, {144, 388, 210, 3963}, {145, 389, 105, 3963}, {146, 390, 21, 3963}, {148, 391, 7, 3963}, {149, 392, 42, 3963}, {150, 393, 105, 3963}, {151, 394, 140, 3963}, {152, 395, 105, 3963}, {153, 396, 42, 3963}, {154, 397, 7, 3963}, {156, 398, 1, 3963}, {157, 399, 7, 3963}, {158, 400, 21, 3963}, {159, 401, 35,

3963}, {160, 402, 35, 3963}, {161, 403, 21, 3963}, {162, 404, 7, 3963}, {163, 405, 1, 3963}, {122, 370, 1, 3964}, {124, 371, 7, 3964}, {125, 372, 7, 3964}, {127, 373, 21, 3964}, {128, 374, 42, 3964}, {129, 375, 21, 3964}, {131, 376, 35, 3964}, {132, 377, 105, 3964}, {133, 378, 105, 3964}, {134, 379, 35, 3964}, {136, 380, 35, 3964}, {137, 381, 140, 3964}, {138, 382, 210, 3964}, {139, 383, 140, 3964}, {140, 384, 35, 3964}, {142, 385, 21, 3964}, {143, 386, 105, 3964}, {144, 387, 210, 3964}, {145, 388, 210, 3964}, {146, 389, 105, 3964}, {147, 390, 21, 3964}, {149, 391, 7, 3964}, {150, 392, 42, 3964}, {151, 393, 105, 3964}, {152, 394, 140, 3964}, {153, 395, 105, 3964}, {154, 396, 42, 3964}, {155, 397, 7, 3964}, {157, 398, 1, 3964}, {158, 399, 7, 3964}, {159, 400, 21, 3964}, {160, 401, 35, 3964}, {161, 402, 35, 3964}, {162, 403, 21, 3964}, {163, 404, 7, 3964}, {164, 405, 1, 3964}, {120, 406, 1, 3965}, {121, 407, 8, 3965}, {122, 408, 8, 3965}, {123, 409, 28, 3965}, {124, 410, 56, 3965}, {125, 411, 28, 3965}, {126, 412, 56, 3965}, {127, 413, 168, 3965}, {128, 414, 168, 3965}, {129, 415, 56, 3965}, {130, 416, 70, 3965}, {131, 417, 280, 3965}, {132, 418, 420, 3965}, {133, 419, 280, 3965}, {134, 420, 70, 3965}, {135, 421, 56, 3965}, {136, 422, 280, 3965}, {137, 423, 560, 3965}, {138, 424, 560, 3965}, {139, 425, 280, 3965}, {140, 426, 56, 3965}, {141, 427, 28, 3965}, {142, 428, 168, 3965}, {143, 429, 420, 3965}, {144, 430, 560, 3965}, {145, 431, 420, 3965}, {146, 432, 168, 3965}, {147, 433, 28, 3965}, {148, 434, 8, 3965}, {149, 435, 56, 3965}, {150, 436, 168, 3965}, {151, 437, 280, 3965}, {152, 438, 280, 3965}, {153, 439, 168, 3965}, {154, 440, 56, 3965}, {155, 441, 8, 3965}, {156, 442, 1, 3965}, {157, 443, 8, 3965}, {158, 444, 28, 3965}, {159, 445, 56, 3965}, {160, 446, 70, 3965}, {161, 447, 56, 3965}, {162, 448, 28, 3965}, {163, 449, 8, 3965}, {164, 450, 1, 3965}, {165, 451, 1, 3966}, {166, 452, 9, 3966}, {167, 453, 9, 3966}, {168, 454, 36, 3966}, {169, 455, 72, 3966}, {170, 456, 36, 3966}, {171, 457, 84, 3966}, {172, 458, 252, 3966}, {173, 459, 252, 3966}, {174, 460, 84, 3966}, {175, 461, 126, 3966}, {176, 462, 504, 3966}, {177, 463, 756, 3966}, {178, 464, 504, 3966}, {179, 465, 126, 3966}, {180, 466, 126, 3966}, {181, 467, 630, 3966}, {182, 468, 1260, 3966}, {183, 469, 1260, 3966}, {184, 470, 630, 3966}, {185, 471, 126, 3966}, {186, 472, 84, 3966}, {187, 473, 504, 3966}, {188, 474, 1260, 3966}, {189, 475, 1680, 3966}, {190, 476, 1260, 3966}, {191, 477, 504, 3966}, {192, 478, 84, 3966}, {193, 479, 36, 3966}, {194, 480, 252, 3966}, {195, 481, 756, 3966}, {196, 482, 1260, 3966}, {197, 483, 1260, 3966}, {198, 484, 756, 3966}, {199, 485, 252, 3966}, {200, 486, 36, 3966}, {201, 487, 9, 3966}, {202, 488, 72, 3966}, {203, 489, 252, 3966}, {204, 490, 504, 3966}, {205, 491, 630, 3966}, {206, 492, 504, 3966}, {207, 493, 252, 3966}, {208, 494, 72, 3966}, {209, 495, 9, 3966}, {210, 496, 1, 3966}, {211, 497, 9, 3966}, {212, 498, 36, 3966}, {213, 499, 84, 3966}, {214, 500, 126, 3966}, {215, 501, 126, 3966}, {216, 502, 84, 3966}, {217, 503, 36, 3966}, {218, 504, 9, 3966}, {219, 505, 1, 3966}, {0, 506, 1, 3967}, {1, 507, 1, 3968}, {2, 508, 1, 3968}, {3, 509, 1, 3968}, {1, 510, 1, 3969}, {2, 511, 1, 3969}, {3, 512, 1, 3969}, {1, 511, 1, 3970}, {2, 513, 1, 3970}, {3, 514, 1, 3970}, {1, 512, 1, 3971}, {2, 514, 1, 3971}, {3, 515, 1, 3971}, {1, 516, 1, 3972}, {2, 517, 1, 3972}, {3, 518, 1, 3972}, {1, 517, 1, 3973}, {2, 519, 1, 3973}, {3, 520, 1, 3973}, {1, 518, 1, 3974}, {2, 520, 1, 3974}, {3, 521, 1, 3974}, {1, 519, 1, 3975}, {2, 522, 1, 3975}, {3, 523, 1, 3975}, {1, 520, 1, 3976}, {2, 523, 1, 3976}, {3, 524, 1, 3976}, {1, 521, 1, 3977}, {2, 524, 1, 3977}, {3, 525, 1, 3977}, {4, 507, 1, 3978}, {5, 508, 1, 3978}, {6, 509, 1, 3978}, {5, 507, 1, 3979}, {7, 508, 1, 3979}, {8, 509, 1, 3979}, {6, 507, 1, 3980}, {8, 508, 1, 3980}, {9, 509, 1, 3980}, {4, 510, 1, 3981}, {5, 511, 1, 3981}, {6, 512, 1, 3981}, {4, 511, 1, 3982}, {5, 513, 1, 3982}, {6, 514, 1, 3982}, {4, 512, 1, 3983}, {5, 514, 1, 3983}, {6, 515, 1, 3983}, {5, 510, 1, 3984}, {7, 511, 1, 3984}, {8, 512, 1, 3984}, {5, 511, 1, 3985}, {7, 513, 1, 3985}, {8, 514, 1, 3985}, {5, 512, 1, 3986}, {7, 514, 1, 3986}, {8, 515, 1, 3986}, {6, 510, 1, 3987}, {8, 511, 1, 3987}, {9, 512, 1, 3987}, {6, 511, 1, 3988}, {8, 513, 1, 3988}, {9, 514, 1, 3988}, {6, 512, 1, 3989}, {8, 514, 1, 3989}, {9, 515, 1, 3989}, {4, 516, 1, 3990}, {5, 517, 1, 3990}, {6, 518, 1, 3990}, {4, 517, 1, 3991}, {5, 519, 1, 3991}, {6, 520, 1, 3991}, {4, 518, 1, 3992}, {5, 520, 1, 3992}, {6, 521, 1, 3992}, {4, 519, 1, 3993}, {5, 522, 1, 3993}, {6, 523, 1, 3993}, {4, 520, 1, 3994}, {5, 523, 1, 3994}, {6, 524, 1, 3994}, {4, 521, 1, 3995}, {5, 524, 1, 3995}, {6, 525, 1, 3995}, {5, 516, 1, 3996}, {7, 517, 1, 3996}, {8, 518, 1, 3996}, {5, 517, 1, 3997}, {7, 519, 1, 3997}, {8, 520, 1, 3997}, {5, 518, 1, 3998}, {7, 520, 1, 3998}, {8, 521, 1, 3998}, {5, 519, 1, 3999}, {7, 522, 1, 3999}, {8, 523, 1, 3999}, {5, 520, 1, 4000}, {7, 523, 1, 4000}, {8, 524, 1, 4000}, {5, 521, 1, 4001}, {7, 524, 1, 4001}, {8, 525, 1, 4001}, {6, 516, 1, 4002}, {8, 517, 1, 4002}, {9, 518, 1, 4002}, {6, 517, 1, 4003}, {8, 519, 1, 4003}, {9, 520, 1, 4003}, {6, 518, 1, 4004}, {8, 520, 1, 4004}, {9, 521, 1, 4004}, {6, 519, 1, 4005}, {8, 522, 1, 4005}, {9, 523, 1, 4005}, {6, 520, 1, 4006}, {8, 523, 1, 4006}, {9, 524, 1, 4006}, {6, 521, 1, 4007}, {8, 524, 1, 4007}, {9, 525, 1, 4007}, {4, 526, 1, 4008}, {5, 527, 1, 4008}, {6, 528, 1, 4008}, {4, 527, 1, 4009}, {5, 529, 1, 4009}, {6, 530, 1, 4009}, {4, 528, 1, 4010}, {5, 530, 1, 4010}, {6, 531, 1, 4010}, {4, 529, 1, 4011}, {5, 532, 1, 4011}, {6, 533, 1, 4011}, {4, 530, 1, 4012}, {5, 533, 1, 4012}, {6, 534, 1, 4012}, {4, 531, 1, 4013}, {5, 534, 1, 4013}, {6, 535, 1, 4013}, {4, 532, 1, 4014}, {5, 536, 1, 4014}, {6, 537, 1, 4014}, {4, 533, 1, 4015}, {5, 537, 1, 4015}, {6, 538, 1, 4015}, {4, 534, 1, 4016}, {5, 538, 1, 4016}, {6, 539, 1, 4016}, {4, 535, 1, 4017}, {5, 539, 1, 4017}, {6, 540, 1, 4017}, {5, 526, 1, 4018}, {7, 527, 1, 4018}, {8, 528, 1, 4018}, {5, 527, 1, 4019}, {7, 529, 1, 4019}, {8, 530, 1, 4019}, {5,

528, 1, 4020}, {7, 530, 1, 4020}, {8, 531, 1, 4020}, {5, 529, 1, 4021}, {7, 532, 1, 4021}, {8, 533, 1, 4021}, {5, 530, 1, 4022}, {7, 533, 1, 4022}, {8, 534, 1, 4022}, {5, 531, 1, 4023}, {7, 534, 1, 4023}, {8, 535, 1, 4023}, {5, 532, 1, 4024}, {7, 536, 1, 4024}, {8, 537, 1, 4024}, {5, 533, 1, 4025}, {7, 537, 1, 4025}, {8, 538, 1, 4025}, {5, 534, 1, 4026}, {7, 538, 1, 4026}, {8, 539, 1, 4026}, {5, 535, 1, 4027}, {7, 539, 1, 4027}, {8, 540, 1, 4027}, {6, 526, 1, 4028}, {8, 527, 1, 4028}, {9, 528, 1, 4028}, {6, 527, 1, 4029}, {8, 529, 1, 4029}, {9, 530, 1, 4029}, {6, 528, 1, 4030}, {8, 530, 1, 4030}, {9, 531, 1, 4030}, {6, 529, 1, 4031}, {8, 532, 1, 4031}, {9, 533, 1, 4031}, {6, 530, 1, 4032}, {8, 533, 1, 4032}, {9, 534, 1, 4032}, {6, 531, 1, 4033}, {8, 534, 1, 4033}, {9, 535, 1, 4033}, {6, 532, 1, 4034}, {8, 536, 1, 4034}, {9, 537, 1, 4034}, {6, 533, 1, 4035}, {8, 537, 1, 4035}, {9, 538, 1, 4035}, {6, 534, 1, 4036}, {8, 538, 1, 4036}, {9, 539, 1, 4036}, {6, 535, 1, 4037}, {8, 539, 1, 4037}, {9, 540, 1, 4037}, {4, 510, 1, 4038}, {5, 511, 2, 4038}, {6, 512, 2, 4038}, {7, 513, 1, 4038}, {8, 514, 2, 4038}, {9, 515, 1, 4038}, {4, 516, 1, 4039}, {5, 517, 2, 4039}, {6, 518, 2, 4039}, {7, 519, 1, 4039}, {8, 520, 2, 4039}, {9, 521, 1, 4039}, {4, 517, 1, 4040}, {5, 519, 2, 4040}, {6, 520, 2, 4040}, {7, 522, 1, 4040}, {8, 523, 2, 4040}, {9, 524, 1, 4040}, {4, 518, 1, 4041}, {5, 520, 2, 4041}, {6, 521, 2, 4041}, {7, 523, 1, 4041}, {8, 524, 2, 4041}, {9, 525, 1, 4041}, {4, 526, 1, 4042}, {5, 527, 2, 4042}, {6, 528, 2, 4042}, {7, 529, 1, 4042}, {8, 530, 2, 4042}, {9, 531, 1, 4042}, {4, 527, 1, 4043}, {5, 529, 2, 4043}, {6, 530, 2, 4043}, {7, 532, 1, 4043}, {8, 533, 2, 4043}, {9, 534, 1, 4043}, {4, 528, 1, 4044}, {5, 530, 2, 4044}, {6, 531, 2, 4044}, {7, 533, 1, 4044}, {8, 534, 2, 4044}, {9, 535, 1, 4044}, {4, 529, 1, 4045}, {5, 532, 2, 4045}, {6, 533, 2, 4045}, {7, 536, 1, 4045}, {8, 537, 2, 4045}, {9, 538, 1, 4045}, {4, 530, 1, 4046}, {5, 533, 2, 4046}, {6, 534, 2, 4046}, {7, 537, 1, 4046}, {8, 538, 2, 4046}, {9, 539, 1, 4046}, {4, 531, 1, 4047}, {5, 534, 2, 4047}, {6, 535, 2, 4047}, {7, 538, 1, 4047}, {8, 539, 2, 4047}, {9, 540, 1, 4047}, {4, 541, 1, 4048}, {5, 542, 2, 4048}, {6, 543, 2, 4048}, {7, 544, 1, 4048}, {8, 545, 2, 4048}, {9, 546, 1, 4048}, {4, 542, 1, 4049}, {5, 544, 2, 4049}, {6, 545, 2, 4049}, {7, 547, 1, 4049}, {8, 548, 2, 4049}, {9, 549, 1, 4049}, {4, 543, 1, 4050}, {5, 545, 2, 4050}, {6, 546, 2, 4050}, {7, 548, 1, 4050}, {8, 549, 2, 4050}, {9, 550, 1, 4050}, {4, 544, 1, 4051}, {5, 547, 2, 4051}, {6, 548, 2, 4051}, {7, 551, 1, 4051}, {8, 552, 2, 4051}, {9, 553, 1, 4051}, {4, 545, 1, 4052}, {5, 548, 2, 4052}, {6, 549, 2, 4052}, {7, 552, 1, 4052}, {8, 553, 2, 4052}, {9, 554, 1, 4052}, {4, 546, 1, 4053}, {5, 549, 2, 4053}, {6, 550, 2, 4053}, {7, 553, 1, 4053}, {8, 554, 2, 4053}, {9, 555, 1, 4053}, {4, 547, 1, 4054}, {5, 551, 2, 4054}, {6, 552, 2, 4054}, {7, 556, 1, 4054}, {8, 557, 2, 4054}, {9, 558, 1, 4054}, {4, 548, 1, 4055}, {5, 552, 2, 4055}, {6, 553, 2, 4055}, {7, 557, 1, 4055}, {8, 558, 2, 4055}, {9, 559, 1, 4055}, {4, 549, 1, 4056}, {5, 553, 2, 4056}, {6, 554, 2, 4056}, {7, 558, 1, 4056}, {8, 559, 2, 4056}, {9, 560, 1, 4056}, {4, 550, 1, 4057}, {5, 554, 2, 4057}, {6, 555, 2, 4057}, {7, 559, 1, 4057}, {8, 560, 2, 4057}, {9, 561, 1, 4057}, {10, 507, 1, 4058}, {11, 508, 1, 4058}, {12, 509, 1, 4058}, {11, 507, 1, 4059}, {13, 508, 1, 4059}, {14, 509, 1, 4059}, {12, 507, 1, 4060}, {14, 508, 1, 4060}, {15, 509, 1, 4060}, {13, 507, 1, 4061}, {16, 508, 1, 4061}, {17, 509, 1, 4061}, {14, 507, 1, 4062}, {17, 508, 1, 4062}, {18, 509, 1, 4062}, {15, 507, 1, 4063}, {18, 508, 1, 4063}, {19, 509, 1, 4063}, {10, 510, 1, 4064}, {11, 511, 1, 4064}, {12, 512, 1, 4064}, {10, 511, 1, 4065}, {11, 513, 1, 4065}, {12, 514, 1, 4065}, {10, 512, 1, 4066}, {11, 514, 1, 4066}, {12, 515, 1, 4066}, {11, 510, 1, 4067}, {13, 511, 1, 4067}, {14, 512, 1, 4067}, {11, 511, 1, 4068}, {13, 513, 1, 4068}, {14, 514, 1, 4068}, {11, 512, 1, 4069}, {13, 514, 1, 4069}, {14, 515, 1, 4069}, {12, 510, 1, 4070}, {14, 511, 1, 4070}, {15, 512, 1, 4070}, {12, 511, 1, 4071}, {14, 513, 1, 4071}, {15, 514, 1, 4071}, {12, 512, 1, 4072}, {14, 514, 1, 4072}, {15, 515, 1, 4072}, {13, 510, 1, 4073}, {16, 511, 1, 4073}, {17, 512, 1, 4073}, {13, 511, 1, 4074}, {16, 513, 1, 4074}, {17, 514, 1, 4074}, {13, 512, 1, 4075}, {16, 514, 1, 4075}, {17, 515, 1, 4075}, {14, 510, 1, 4076}, {17, 511, 1, 4076}, {18, 512, 1, 4076}, {14, 511, 1, 4077}, {17, 513, 1, 4077}, {18, 514, 1, 4077}, {14, 512, 1, 4078}, {17, 514, 1, 4078}, {18, 515, 1, 4078}, {15, 510, 1, 4079}, {18, 511, 1, 4079}, {19, 512, 1, 4079}, {15, 511, 1, 4080}, {18, 513, 1, 4080}, {19, 514, 1, 4080}, {15, 512, 1, 4081}, {18, 514, 1, 4081}, {19, 515, 1, 4081}, {10, 516, 1, 4082}, {11, 517, 1, 4082}, {12, 518, 1, 4082}, {10, 517, 1, 4083}, {11, 519, 1, 4083}, {12, 520, 1, 4083}, {10, 518, 1, 4084}, {11, 520, 1, 4084}, {12, 521, 1, 4084}, {10, 519, 1, 4085}, {11, 522, 1, 4085}, {12, 523, 1, 4085}, {10, 520, 1, 4086}, {11, 523, 1, 4086}, {12, 524, 1, 4086}, {10, 521, 1, 4087}, {11, 524, 1, 4087}, {12, 525, 1, 4087}, {11, 516, 1, 4088}, {13, 517, 1, 4088}, {14, 518, 1, 4088}, {11, 517, 1, 4089}, {13, 519, 1, 4089}, {14, 520, 1, 4089}, {11, 518, 1, 4090}, {13, 520, 1, 4090}, {14, 521, 1, 4090}, {11, 519, 1, 4091}, {13, 522, 1, 4091}, {14, 523, 1, 4091}, {11, 520, 1, 4092}, {13, 523, 1, 4092}, {14, 524, 1, 4092}, {11, 521, 1, 4093}, {13, 524, 1, 4093}, {14, 525, 1, 4093}, {12, 516, 1, 4094}, {14, 517, 1, 4094}, {15, 518, 1, 4094}, {12, 517, 1, 4095}, {14, 519, 1, 4095}, {15, 520, 1, 4095}, {12, 518, 1, 4096}, {14, 520, 1, 4096}, {15, 521, 1, 4096}, {12, 519, 1, 4097}, {14, 522, 1, 4097}, {15, 523, 1, 4097}, {12, 520, 1, 4098}, {14, 523, 1, 4098}, {15, 524, 1, 4098}, {12, 521, 1, 4099}, {14, 524, 1, 4099}, {15, 525, 1, 4099}, {13, 516, 1, 4100}, {16, 517, 1, 4100}, {17, 518, 1, 4100}, {13, 517, 1, 4101}, {16, 519, 1, 4101}, {17, 520, 1, 4101}, {13, 518, 1, 4102}, {16, 520, 1, 4102}, {17, 521, 1, 4102}, {13, 519, 1, 4103}, {16, 522, 1, 4103}, {17, 523, 1, 4103}, {13, 520, 1, 4104}, {16, 523, 1, 4104}, {17, 524, 1, 4104}, {13, 521, 1, 4105}, {16, 524, 1, 4105},

{17, 525, 1, 4105}, {14, 516, 1, 4106}, {17, 517, 1, 4106}, {18, 518, 1, 4106}, {14, 517, 1, 4107}, {17, 519, 1, 4107}, {18, 520, 1, 4107}, {14, 518, 1, 4108}, {17, 520, 1, 4108}, {18, 521, 1, 4108}, {14, 519, 1, 4109}, {17, 522, 1, 4109}, {18, 523, 1, 4109}, {14, 520, 1, 4110}, {17, 523, 1, 4110}, {18, 524, 1, 4110}, {14, 521, 1, 4111}, {17, 524, 1, 4111}, {18, 525, 1, 4111}, {15, 516, 1, 4112}, {18, 517, 1, 4112}, {19, 518, 1, 4112}, {15, 517, 1, 4113}, {18, 519, 1, 4113}, {19, 520, 1, 4113}, {15, 518, 1, 4114}, {18, 520, 1, 4114}, {19, 521, 1, 4114}, {15, 519, 1, 4115}, {18, 522, 1, 4115}, {19, 523, 1, 4115}, {15, 520, 1, 4116}, {18, 523, 1, 4116}, {19, 524, 1, 4116}, {15, 521, 1, 4117}, {18, 524, 1, 4117}, {19, 525, 1, 4117}, {10, 510, 1, 4118}, {11, 511, 2, 4118}, {12, 512, 2, 4118}, {13, 513, 1, 4118}, {14, 514, 2, 4118}, {15, 515, 1, 4118}, {11, 510, 1, 4119}, {13, 511, 2, 4119}, {14, 512, 2, 4119}, {16, 513, 1, 4119}, {17, 514, 2, 4119}, {18, 515, 1, 4119}, {12, 510, 1, 4120}, {14, 511, 2, 4120}, {15, 512, 2, 4120}, {17, 513, 1, 4120}, {18, 514, 2, 4120}, {19, 515, 1, 4120}, {10, 516, 1, 4121}, {11, 517, 2, 4121}, {12, 518, 2, 4121}, {13, 519, 1, 4121}, {14, 520, 2, 4121}, {15, 521, 1, 4121}, {10, 517, 1, 4122}, {11, 519, 2, 4122}, {12, 520, 2, 4122}, {13, 522, 1, 4122}, {14, 523, 2, 4122}, {15, 524, 1, 4122}, {10, 518, 1, 4123}, {11, 520, 2, 4123}, {12, 521, 2, 4123}, {13, 523, 1, 4123}, {14, 524, 2, 4123}, {15, 525, 1, 4123}, {11, 516, 1, 4124}, {13, 517, 2, 4124}, {14, 518, 2, 4124}, {16, 519, 1, 4124}, {17, 520, 2, 4124}, {18, 521, 1, 4124}, {11, 517, 1, 4125}, {13, 519, 2, 4125}, {14, 520, 2, 4125}, {16, 522, 1, 4125}, {17, 523, 2, 4125}, {18, 524, 1, 4125}, {11, 518, 1, 4126}, {13, 520, 2, 4126}, {14, 521, 2, 4126}, {16, 523, 1, 4126}, {17, 524, 2, 4126}, {18, 525, 1, 4126}, {12, 516, 1, 4127}, {14, 517, 2, 4127}, {15, 518, 2, 4127}, {17, 519, 1, 4127}, {18, 520, 2, 4127}, {19, 521, 1, 4127}, {12, 517, 1, 4128}, {14, 519, 2, 4128}, {15, 520, 2, 4128}, {17, 522, 1, 4128}, {18, 523, 2, 4128}, {19, 524, 1, 4128}, {12, 518, 1, 4129}, {14, 520, 2, 4129}, {15, 521, 2, 4129}, {17, 523, 1, 4129}, {18, 524, 2, 4129}, {19, 525, 1, 4129}, {10, 526, 1, 4130}, {11, 527, 2, 4130}, {12, 528, 2, 4130}, {13, 529, 1, 4130}, {14, 530, 2, 4130}, {15, 531, 1, 4130}, {10, 527, 1, 4131}, {11, 529, 2, 4131}, {12, 530, 2, 4131}, {13, 532, 1, 4131}, {14, 533, 2, 4131}, {15, 534, 1, 4131}, {10, 528, 1, 4132}, {11, 530, 2, 4132}, {12, 531, 2, 4132}, {13, 533, 1, 4132}, {14, 534, 2, 4132}, {15, 535, 1, 4132}, {10, 529, 1, 4133}, {11, 532, 2, 4133}, {12, 533, 2, 4133}, {13, 536, 1, 4133}, {14, 537, 2, 4133}, {15, 538, 1, 4133}, {10, 530, 1, 4134}, {11, 533, 2, 4134}, {12, 534, 2, 4134}, {13, 537, 1, 4134}, {14, 538, 2, 4134}, {15, 539, 1, 4134}, {10, 531, 1, 4135}, {11, 534, 2, 4135}, {12, 535, 2, 4135}, {13, 538, 1, 4135}, {14, 539, 2, 4135}, {15, 540, 1, 4135}, {11, 526, 1, 4136}, {13, 527, 2, 4136}, {14, 528, 2, 4136}, {16, 529, 1, 4136}, {17, 530, 2, 4136}, {18, 531, 1, 4136}, {11, 527, 1, 4137}, {13, 529, 2, 4137}, {14, 530, 2, 4137}, {16, 532, 1, 4137}, {17, 533, 2, 4137}, {18, 534, 1, 4137}, {11, 528, 1, 4138}, {13, 530, 2, 4138}, {14, 531, 2, 4138}, {16, 533, 1, 4138}, {17, 534, 2, 4138}, {18, 535, 1, 4138}, {11, 529, 1, 4139}, {13, 532, 2, 4139}, {14, 533, 2, 4139}, {16, 536, 1, 4139}, {17, 537, 2, 4139}, {18, 538, 1, 4139}, {11, 530, 1, 4140}, {13, 533, 2, 4140}, {14, 534, 2, 4140}, {16, 537, 1, 4140}, {17, 538, 2, 4140}, {18, 539, 1, 4140}, {11, 531, 1, 4141}, {13, 534, 2, 4141}, {14, 535, 2, 4141}, {16, 538, 1, 4141}, {17, 539, 2, 4141}, {18, 540, 1, 4141}, {12, 526, 1, 4142}, {14, 527, 2, 4142}, {15, 528, 2, 4142}, {17, 529, 1, 4142}, {18, 530, 2, 4142}, {19, 531, 1, 4142}, {12, 527, 1, 4143}, {14, 529, 2, 4143}, {15, 530, 2, 4143}, {17, 532, 1, 4143}, {18, 533, 2, 4143}, {19, 534, 1, 4143}, {12, 528, 1, 4144}, {14, 530, 2, 4144}, {15, 531, 2, 4144}, {17, 533, 1, 4144}, {18, 534, 2, 4144}, {19, 535, 1, 4144}, {12, 529, 1, 4145}, {14, 532, 2, 4145}, {15, 533, 2, 4145}, {17, 536, 1, 4145}, {18, 537, 2, 4145}, {19, 538, 1, 4145}, {12, 530, 1, 4146}, {14, 533, 2, 4146}, {15, 534, 2, 4146}, {17, 537, 1, 4146}, {18, 538, 2, 4146}, {19, 539, 1, 4146}, {12, 531, 1, 4147}, {14, 534, 2, 4147}, {15, 535, 2, 4147}, {17, 538, 1, 4147}, {18, 539, 2, 4147}, {19, 540, 1, 4147}, {10, 516, 1, 4148}, {11, 517, 3, 4148}, {12, 518, 3, 4148}, {13, 519, 3, 4148}, {14, 520, 6, 4148}, {15, 521, 3, 4148}, {16, 522, 1, 4148}, {17, 523, 3, 4148}, {18, 524, 3, 4148}, {19, 525, 1, 4148}, {10, 526, 1, 4149}, {11, 527, 3, 4149}, {12, 528, 3, 4149}, {13, 529, 3, 4149}, {14, 530, 6, 4149}, {15, 531, 3, 4149}, {16, 532, 1, 4149}, {17, 533, 3, 4149}, {18, 534, 3, 4149}, {19, 535, 1, 4149}, {10, 527, 1, 4150}, {11, 529, 3, 4150}, {12, 530, 3, 4150}, {13, 532, 3, 4150}, {14, 533, 6, 4150}, {15, 534, 3, 4150}, {16, 536, 1, 4150}, {17, 537, 3, 4150}, {18, 538, 3, 4150}, {19, 539, 1, 4150}, {10, 528, 1, 4151}, {11, 530, 3, 4151}, {12, 531, 3, 4151}, {13, 533, 3, 4151}, {14, 534, 6, 4151}, {15, 535, 3, 4151}, {16, 537, 1, 4151}, {17, 538, 3, 4151}, {18, 539, 3, 4151}, {19, 540, 1, 4151}, {10, 541, 1, 4152}, {11, 542, 3, 4152}, {12, 543, 3, 4152}, {13, 544, 3, 4152}, {14, 545, 6, 4152}, {15, 546, 3, 4152}, {16, 547, 1, 4152}, {17, 548, 3, 4152}, {18, 549, 3, 4152}, {19, 550, 1, 4152}, {10, 542, 1, 4153}, {11, 544, 3, 4153}, {12, 545, 3, 4153}, {13, 547, 3, 4153}, {14, 548, 6, 4153}, {15, 549, 3, 4153}, {16, 551, 1, 4153}, {17, 552, 3, 4153}, {18, 553, 3, 4153}, {19, 554, 1, 4153}, {10, 543, 1, 4154}, {11, 545, 3, 4154}, {12, 546, 3, 4154}, {13, 548, 3, 4154}, {14, 549, 6, 4154}, {15, 550, 3, 4154}, {16, 552, 1, 4154}, {17, 553, 3, 4154}, {18, 554, 3, 4154}, {19, 555, 1, 4154}, {10, 544, 1, 4155}, {11, 547, 3, 4155}, {12, 548, 3, 4155}, {13, 551, 3, 4155}, {14, 552, 6, 4155}, {15, 553, 3, 4155}, {16, 556, 1, 4155}, {17, 557, 3, 4155}, {18, 558, 3, 4155}, {19, 559, 1, 4155}, {10, 545, 1, 4156}, {11, 548, 3, 4156}, {12, 549, 3, 4156}, {13, 552, 3, 4156}, {14, 553, 6, 4156}, {15, 554, 3, 4156}, {16, 557, 1, 4156}, {17, 558, 3, 4156}, {18, 559, 3, 4156}, {19, 560, 1, 4156}, {10, 546, 1, 4157}, {11, 549, 3, 4157}, {12, 550, 3, 4157}, {13, 553,

3, 4157}, {14, 554, 6, 4157}, {15, 555, 3, 4157}, {16, 558, 1, 4157}, {17, 559, 3, 4157}, {18, 560, 3, 4157}, {19, 561, 1, 4157}, {10, 562, 1, 4158}, {11, 563, 3, 4158}, {12, 564, 3, 4158}, {13, 565, 3, 4158}, {14, 566, 6, 4158}, {15, 567, 3, 4158}, {16, 568, 1, 4158}, {17, 569, 3, 4158}, {18, 570, 3, 4158}, {19, 571, 1, 4158}, {10, 563, 1, 4159}, {11, 565, 3, 4159}, {12, 566, 3, 4159}, {13, 568, 3, 4159}, {14, 569, 6, 4159}, {15, 570, 3, 4159}, {16, 572, 1, 4159}, {17, 573, 3, 4159}, {18, 574, 3, 4159}, {19, 575, 1, 4159}, {10, 564, 1, 4160}, {11, 566, 3, 4160}, {12, 567, 3, 4160}, {13, 569, 3, 4160}, {14, 570, 6, 4160}, {15, 571, 3, 4160}, {16, 573, 1, 4160}, {17, 574, 3, 4160}, {18, 575, 3, 4160}, {19, 576, 1, 4160}, {10, 565, 1, 4161}, {11, 568, 3, 4161}, {12, 569, 3, 4161}, {13, 572, 3, 4161}, {14, 573, 6, 4161}, {15, 574, 3, 4161}, {16, 577, 1, 4161}, {17, 578, 3, 4161}, {18, 579, 3, 4161}, {19, 580, 1, 4161}, {10, 566, 1, 4162}, {11, 569, 3, 4162}, {12, 570, 3, 4162}, {13, 573, 3, 4162}, {14, 574, 6, 4162}, {15, 575, 3, 4162}, {16, 578, 1, 4162}, {17, 579, 3, 4162}, {18, 580, 3, 4162}, {19, 581, 1, 4162}, {10, 567, 1, 4163}, {11, 570, 3, 4163}, {12, 571, 3, 4163}, {13, 574, 3, 4163}, {14, 575, 6, 4163}, {15, 576, 3, 4163}, {16, 579, 1, 4163}, {17, 580, 3, 4163}, {18, 581, 3, 4163}, {19, 582, 1, 4163}, {10, 568, 1, 4164}, {11, 572, 3, 4164}, {12, 573, 3, 4164}, {13, 577, 3, 4164}, {14, 578, 6, 4164}, {15, 579, 3, 4164}, {16, 583, 1, 4164}, {17, 584, 3, 4164}, {18, 585, 3, 4164}, {19, 586, 1, 4164}, {10, 569, 1, 4165}, {11, 573, 3, 4165}, {12, 574, 3, 4165}, {13, 578, 3, 4165}, {14, 579, 6, 4165}, {15, 580, 3, 4165}, {16, 584, 1, 4165}, {17, 585, 3, 4165}, {18, 586, 3, 4165}, {19, 587, 1, 4165}, {10, 570, 1, 4166}, {11, 574, 3, 4166}, {12, 575, 3, 4166}, {13, 579, 3, 4166}, {14, 580, 6, 4166}, {15, 581, 3, 4166}, {16, 585, 1, 4166}, {17, 586, 3, 4166}, {18, 587, 3, 4166}, {19, 588, 1, 4166}, {10, 571, 1, 4167}, {11, 575, 3, 4167}, {12, 576, 3, 4167}, {13, 580, 3, 4167}, {14, 581, 6, 4167}, {15, 582, 3, 4167}, {16, 586, 1, 4167}, {17, 587, 3, 4167}, {18, 588, 3, 4167}, {19, 589, 1, 4167}, {20, 510, 1, 4168}, {21, 511, 2, 4168}, {22, 512, 2, 4168}, {23, 513, 1, 4168}, {24, 514, 2, 4168}, {25, 515, 1, 4168}, {21, 510, 1, 4169}, {23, 511, 2, 4169}, {24, 512, 2, 4169}, {26, 513, 1, 4169}, {27, 514, 2, 4169}, {28, 515, 1, 4169}, {22, 510, 1, 4170}, {24, 511, 2, 4170}, {25, 512, 2, 4170}, {27, 513, 1, 4170}, {28, 514, 2, 4170}, {29, 515, 1, 4170}, {23, 510, 1, 4171}, {26, 511, 2, 4171}, {27, 512, 2, 4171}, {30, 513, 1, 4171}, {31, 514, 2, 4171}, {32, 515, 1, 4171}, {24, 510, 1, 4172}, {27, 511, 2, 4172}, {28, 512, 2, 4172}, {31, 513, 1, 4172}, {32, 514, 2, 4172}, {33, 515, 1, 4172}, {25, 510, 1, 4173}, {28, 511, 2, 4173}, {29, 512, 2, 4173}, {32, 513, 1, 4173}, {33, 514, 2, 4173}, {34, 515, 1, 4173}, {20, 516, 1, 4174}, {21, 517, 2, 4174}, {22, 518, 2, 4174}, {23, 519, 1, 4174}, {24, 520, 2, 4174}, {25, 521, 1, 4174}, {20, 517, 1, 4175}, {21, 519, 2, 4175}, {22, 520, 2, 4175}, {23, 522, 1, 4175}, {24, 523, 2, 4175}, {25, 524, 1, 4175}, {20, 518, 1, 4176}, {21, 520, 2, 4176}, {22, 521, 2, 4176}, {23, 523, 1, 4176}, {24, 524, 2, 4176}, {25, 525, 1, 4176}, {21, 516, 1, 4177}, {23, 517, 2, 4177}, {24, 518, 2, 4177}, {26, 519, 1, 4177}, {27, 520, 2, 4177}, {28, 521, 1, 4177}, {21, 517, 1, 4178}, {23, 519, 2, 4178}, {24, 520, 2, 4178}, {26, 522, 1, 4178}, {27, 523, 2, 4178}, {28, 524, 1, 4178}, {21, 518, 1, 4179}, {23, 520, 2, 4179}, {24, 521, 2, 4179}, {26, 523, 1, 4179}, {27, 524, 2, 4179}, {28, 525, 1, 4179}, {22, 516, 1, 4180}, {24, 517, 2, 4180}, {25, 518, 2, 4180}, {27, 519, 1, 4180}, {28, 520, 2, 4180}, {29, 521, 1, 4180}, {22, 517, 1, 4181}, {24, 519, 2, 4181}, {25, 520, 2, 4181}, {27, 522, 1, 4181}, {28, 523, 2, 4181}, {29, 524, 1, 4181}, {22, 518, 1, 4182}, {24, 520, 2, 4182}, {25, 521, 2, 4182}, {27, 523, 1, 4182}, {28, 524, 2, 4182}, {29, 525, 1, 4182}, {23, 516, 1, 4183}, {26, 517, 2, 4183}, {27, 518, 2, 4183}, {30, 519, 1, 4183}, {31, 520, 2, 4183}, {32, 521, 1, 4183}, {23, 517, 1, 4184}, {26, 519, 2, 4184}, {27, 520, 2, 4184}, {30, 522, 1, 4184}, {31, 523, 2, 4184}, {32, 524, 1, 4184}, {23, 518, 1, 4185}, {26, 520, 2, 4185}, {27, 521, 2, 4185}, {30, 523, 1, 4185}, {31, 524, 2, 4185}, {32, 525, 1, 4185}, {24, 516, 1, 4186}, {27, 517, 2, 4186}, {28, 518, 2, 4186}, {31, 519, 1, 4186}, {32, 520, 2, 4186}, {33, 521, 1, 4186}, {24, 517, 1, 4187}, {27, 519, 2, 4187}, {28, 520, 2, 4187}, {31, 522, 1, 4187}, {32, 523, 2, 4187}, {33, 524, 1, 4187}, {24, 518, 1, 4188}, {27, 520, 2, 4188}, {28, 521, 2, 4188}, {31, 523, 1, 4188}, {32, 524, 2, 4188}, {33, 525, 1, 4188}, {25, 516, 1, 4189}, {28, 517, 2, 4189}, {29, 518, 2, 4189}, {32, 519, 1, 4189}, {33, 520, 2, 4189}, {34, 521, 1, 4189}, {25, 517, 1, 4190}, {28, 519, 2, 4190}, {29, 520, 2, 4190}, {32, 522, 1, 4190}, {33, 523, 2, 4190}, {34, 524, 1, 4190}, {25, 518, 1, 4191}, {28, 520, 2, 4191}, {29, 521, 2, 4191}, {32, 523, 1, 4191}, {33, 524, 2, 4191}, {34, 525, 1, 4191}, {20, 526, 1, 4192}, {21, 527, 2, 4192}, {22, 528, 2, 4192}, {23, 529, 1, 4192}, {24, 530, 2, 4192}, {25, 531, 1, 4192}, {20, 527, 1, 4193}, {21, 529, 2, 4193}, {22, 530, 2, 4193}, {23, 532, 1, 4193}, {24, 533, 2, 4193}, {25, 534, 1, 4193}, {20, 528, 1, 4194}, {21, 530, 2, 4194}, {22, 531, 2, 4194}, {23, 533, 1, 4194}, {24, 534, 2, 4194}, {25, 535, 1, 4194}, {20, 529, 1, 4195}, {21, 532, 2, 4195}, {22, 533, 2, 4195}, {23, 536, 1, 4195}, {24, 537, 2, 4195}, {25, 538, 1, 4195}, {20, 530, 1, 4196}, {21, 533, 2, 4196}, {22, 534, 2, 4196}, {23, 537, 1, 4196}, {24, 538, 2, 4196}, {25, 539, 1, 4196}, {20, 531, 1, 4197}, {21, 534, 2, 4197}, {22, 535, 2, 4197}, {23, 538, 1, 4197}, {24, 539, 2, 4197}, {25, 540, 1, 4197}, {21, 526, 1, 4198}, {23, 527, 2, 4198}, {24, 528, 2, 4198}, {26, 529, 1, 4198}, {27, 530, 2, 4198}, {28, 531, 1, 4198}, {21, 527, 1, 4199}, {23, 529, 2, 4199}, {24, 530, 2, 4199}, {26, 532, 1, 4199}, {27, 533, 2, 4199}, {28, 534, 1, 4199}, {21, 528, 1, 4200}, {23, 530, 2, 4200}, {24, 531, 2, 4200}, {26, 533, 1, 4200}, {27, 534, 2, 4200}, {28, 535, 1, 4200}, {21, 529, 1, 4201}, {23, 532, 2, 4201}, {24, 533, 2, 4201}, {26, 536, 1, 4201}, {27, 537, 2, 4201}, {28, 538, 1, 4201},

{21, 530, 1, 4202}, {23, 533, 2, 4202}, {24, 534, 2, 4202}, {26, 537, 1, 4202}, {27, 538, 2, 4202}, {28, 539, 1, 4202}, {21, 531, 1, 4203}, {23, 534, 2, 4203}, {24, 535, 2, 4203}, {26, 538, 1, 4203}, {27, 539, 2, 4203}, {28, 540, 1, 4203}, {22, 526, 1, 4204}, {24, 527, 2, 4204}, {25, 528, 2, 4204}, {27, 529, 1, 4204}, {28, 530, 2, 4204}, {29, 531, 1, 4204}, {22, 527, 1, 4205}, {24, 529, 2, 4205}, {25, 530, 2, 4205}, {27, 532, 1, 4205}, {28, 533, 2, 4205}, {29, 534, 1, 4205}, {22, 528, 1, 4206}, {24, 530, 2, 4206}, {25, 531, 2, 4206}, {27, 533, 1, 4206}, {28, 534, 2, 4206}, {29, 535, 1, 4206}, {22, 529, 1, 4207}, {24, 532, 2, 4207}, {25, 533, 2, 4207}, {27, 536, 1, 4207}, {28, 537, 2, 4207}, {29, 538, 1, 4207}, {22, 530, 1, 4208}, {24, 533, 2, 4208}, {25, 534, 2, 4208}, {27, 537, 1, 4208}, {28, 538, 2, 4208}, {29, 539, 1, 4208}, {22, 531, 1, 4209}, {24, 534, 2, 4209}, {25, 535, 2, 4209}, {27, 538, 1, 4209}, {28, 539, 2, 4209}, {29, 540, 1, 4209}, {23, 526, 1, 4210}, {26, 527, 2, 4210}, {27, 528, 2, 4210}, {30, 529, 1, 4210}, {31, 530, 2, 4210}, {32, 531, 1, 4210}, {23, 527, 1, 4211}, {26, 529, 2, 4211}, {27, 530, 2, 4211}, {30, 532, 1, 4211}, {31, 533, 2, 4211}, {32, 534, 1, 4211}, {23, 528, 1, 4212}, {26, 530, 2, 4212}, {27, 531, 2, 4212}, {30, 533, 1, 4212}, {31, 534, 2, 4212}, {32, 535, 1, 4212}, {23, 529, 1, 4213}, {26, 532, 2, 4213}, {27, 533, 2, 4213}, {30, 536, 1, 4213}, {31, 537, 2, 4213}, {32, 538, 1, 4213}, {23, 530, 1, 4214}, {26, 533, 2, 4214}, {27, 534, 2, 4214}, {30, 537, 1, 4214}, {31, 538, 2, 4214}, {32, 539, 1, 4214}, {23, 531, 1, 4215}, {26, 534, 2, 4215}, {27, 535, 2, 4215}, {30, 538, 1, 4215}, {31, 539, 2, 4215}, {32, 540, 1, 4215}, {24, 526, 1, 4216}, {27, 527, 2, 4216}, {28, 528, 2, 4216}, {31, 529, 1, 4216}, {32, 530, 2, 4216}, {33, 531, 1, 4216}, {24, 527, 1, 4217}, {27, 529, 2, 4217}, {28, 530, 2, 4217}, {31, 532, 1, 4217}, {32, 533, 2, 4217}, {33, 534, 1, 4217}, {24, 528, 1, 4218}, {27, 530, 2, 4218}, {28, 531, 2, 4218}, {31, 533, 1, 4218}, {32, 534, 2, 4218}, {33, 535, 1, 4218}, {24, 529, 1, 4219}, {27, 532, 2, 4219}, {28, 533, 2, 4219}, {31, 536, 1, 4219}, {32, 537, 2, 4219}, {33, 538, 1, 4219}, {24, 530, 1, 4220}, {27, 533, 2, 4220}, {28, 534, 2, 4220}, {31, 537, 1, 4220}, {32, 538, 2, 4220}, {33, 539, 1, 4220}, {24, 531, 1, 4221}, {27, 534, 2, 4221}, {28, 535, 2, 4221}, {31, 538, 1, 4221}, {32, 539, 2, 4221}, {33, 540, 1, 4221}, {25, 526, 1, 4222}, {28, 527, 2, 4222}, {29, 528, 2, 4222}, {32, 529, 1, 4222}, {33, 530, 2, 4222}, {34, 531, 1, 4222}, {25, 527, 1, 4223}, {28, 529, 2, 4223}, {29, 530, 2, 4223}, {32, 532, 1, 4223}, {33, 533, 2, 4223}, {34, 534, 1, 4223}, {25, 528, 1, 4224}, {28, 530, 2, 4224}, {29, 531, 2, 4224}, {32, 533, 1, 4224}, {33, 534, 2, 4224}, {34, 535, 1, 4224}, {25, 529, 1, 4225}, {28, 532, 2, 4225}, {29, 533, 2, 4225}, {32, 536, 1, 4225}, {33, 537, 2, 4225}, {34, 538, 1, 4225}, {25, 530, 1, 4226}, {28, 533, 2, 4226}, {29, 534, 2, 4226}, {32, 537, 1, 4226}, {33, 538, 2, 4226}, {34, 539, 1, 4226}, {25, 531, 1, 4227}, {28, 534, 2, 4227}, {29, 535, 2, 4227}, {32, 538, 1, 4227}, {33, 539, 2, 4227}, {34, 540, 1, 4227}, {20, 516, 1, 4228}, {21, 517, 3, 4228}, {22, 518, 3, 4228}, {23, 519, 3, 4228}, {24, 520, 6, 4228}, {25, 521, 3, 4228}, {26, 522, 1, 4228}, {27, 523, 3, 4228}, {28, 524, 3, 4228}, {29, 525, 1, 4228}, {21, 516, 1, 4229}, {23, 517, 3, 4229}, {24, 518, 3, 4229}, {26, 519, 3, 4229}, {27, 520, 6, 4229}, {28, 521, 3, 4229}, {30, 522, 1, 4229}, {31, 523, 3, 4229}, {32, 524, 3, 4229}, {33, 525, 1, 4229}, {22, 516, 1, 4230}, {24, 517, 3, 4230}, {25, 518, 3, 4230}, {27, 519, 3, 4230}, {28, 520, 6, 4230}, {29, 521, 3, 4230}, {31, 522, 1, 4230}, {32, 523, 3, 4230}, {33, 524, 3, 4230}, {34, 525, 1, 4230}, {20, 526, 1, 4231}, {21, 527, 3, 4231}, {22, 528, 3, 4231}, {23, 529, 3, 4231}, {24, 530, 6, 4231}, {25, 531, 3, 4231}, {26, 532, 1, 4231}, {27, 533, 3, 4231}, {28, 534, 3, 4231}, {29, 535, 1, 4231}, {20, 527, 1, 4232}, {21, 529, 3, 4232}, {22, 530, 3, 4232}, {23, 532, 3, 4232}, {24, 533, 6, 4232}, {25, 534, 3, 4232}, {26, 536, 1, 4232}, {27, 537, 3, 4232}, {28, 538, 3, 4232}, {29, 539, 1, 4232}, {20, 528, 1, 4233}, {21, 530, 3, 4233}, {22, 531, 3, 4233}, {23, 533, 3, 4233}, {24, 534, 6, 4233}, {25, 535, 3, 4233}, {26, 537, 1, 4233}, {27, 538, 3, 4233}, {28, 539, 3, 4233}, {29, 540, 1, 4233}, {21, 526, 1, 4234}, {23, 527, 3, 4234}, {24, 528, 3, 4234}, {26, 529, 3, 4234}, {27, 530, 6, 4234}, {28, 531, 3, 4234}, {30, 532, 1, 4234}, {31, 533, 3, 4234}, {32, 534, 3, 4234}, {33, 535, 1, 4234}, {21, 527, 1, 4235}, {23, 529, 3, 4235}, {24, 530, 3, 4235}, {26, 532, 3, 4235}, {27, 533, 6, 4235}, {28, 534, 3, 4235}, {30, 536, 1, 4235}, {31, 537, 3, 4235}, {32, 538, 3, 4235}, {33, 539, 1, 4235}, {21, 528, 1, 4236}, {23, 530, 3, 4236}, {24, 531, 3, 4236}, {26, 533, 3, 4236}, {27, 534, 6, 4236}, {28, 535, 3, 4236}, {30, 537, 1, 4236}, {31, 538, 3, 4236}, {32, 539, 3, 4236}, {33, 540, 1, 4236}, {22, 526, 1, 4237}, {24, 527, 3, 4237}, {25, 528, 3, 4237}, {27, 529, 3, 4237}, {28, 530, 6, 4237}, {29, 531, 3, 4237}, {31, 532, 1, 4237}, {32, 533, 3, 4237}, {33, 534, 3, 4237}, {34, 535, 1, 4237}, {22, 527, 1, 4238}, {24, 529, 3, 4238}, {25, 530, 3, 4238}, {27, 532, 3, 4238}, {28, 533, 6, 4238}, {29, 534, 3, 4238}, {31, 536, 1, 4238}, {32, 537, 3, 4238}, {33, 538, 3, 4238}, {34, 539, 1, 4238}, {22, 528, 1, 4239}, {24, 530, 3, 4239}, {25, 531, 3, 4239}, {27, 533, 3, 4239}, {28, 534, 6, 4239}, {29, 535, 3, 4239}, {31, 537, 1, 4239}, {32, 538, 3, 4239}, {33, 539, 3, 4239}, {34, 540, 1, 4239}, {20, 541, 1, 4240}, {21, 542, 3, 4240}, {22, 543, 3, 4240}, {23, 544, 3, 4240}, {24, 545, 6, 4240}, {25, 546, 3, 4240}, {26, 547, 1, 4240}, {27, 548, 3, 4240}, {28, 549, 3, 4240}, {29, 550, 1, 4240}, {20, 542, 1, 4241}, {21, 544, 3, 4241}, {22, 545, 3, 4241}, {23, 547, 3, 4241}, {24, 548, 6, 4241}, {25, 549, 3, 4241}, {26, 551, 1, 4241}, {27, 552, 3, 4241}, {28, 553, 3, 4241}, {29, 554, 1, 4241}, {20, 543, 1, 4242}, {21, 545, 3, 4242}, {22, 546, 3, 4242}, {23, 548, 3, 4242}, {24, 549, 6, 4242}, {25, 550, 3, 4242}, {26, 552, 1, 4242}, {27, 553, 3, 4242}, {28, 554, 3, 4242}, {29, 555, 1, 4242}, {20, 544, 1, 4243}, {21, 547, 3, 4243}, {22, 548, 3, 4243}, {23, 551, 3, 4243}, {24, 552,

6, 4243}, {25, 553, 3, 4243}, {26, 556, 1, 4243}, {27, 557, 3, 4243}, {28, 558, 3, 4243}, {29, 559, 1, 4243}, {20, 545, 1, 4244}, {21, 548, 3, 4244}, {22, 549, 3, 4244}, {23, 552, 3, 4244}, {24, 553, 6, 4244}, {25, 554, 3, 4244}, {26, 557, 1, 4244}, {27, 558, 3, 4244}, {28, 559, 3, 4244}, {29, 560, 1, 4244}, {20, 546, 1, 4245}, {21, 549, 3, 4245}, {22, 550, 3, 4245}, {23, 553, 3, 4245}, {24, 554, 6, 4245}, {25, 555, 3, 4245}, {26, 558, 1, 4245}, {27, 559, 3, 4245}, {28, 560, 3, 4245}, {29, 561, 1, 4245}, {21, 541, 1, 4246}, {23, 542, 3, 4246}, {24, 543, 3, 4246}, {26, 544, 3, 4246}, {27, 545, 6, 4246}, {28, 546, 3, 4246}, {30, 547, 1, 4246}, {31, 548, 3, 4246}, {32, 549, 3, 4246}, {33, 550, 1, 4246}, {21, 542, 1, 4247}, {23, 544, 3, 4247}, {24, 545, 3, 4247}, {26, 547, 3, 4247}, {27, 548, 6, 4247}, {28, 549, 3, 4247}, {30, 551, 1, 4247}, {31, 552, 3, 4247}, {32, 553, 3, 4247}, {33, 554, 1, 4247}, {21, 543, 1, 4248}, {23, 545, 3, 4248}, {24, 546, 3, 4248}, {26, 548, 3, 4248}, {27, 549, 6, 4248}, {28, 550, 3, 4248}, {30, 552, 1, 4248}, {31, 553, 3, 4248}, {32, 554, 3, 4248}, {33, 555, 1, 4248}, {21, 544, 1, 4249}, {23, 547, 3, 4249}, {24, 548, 3, 4249}, {26, 551, 3, 4249}, {27, 552, 6, 4249}, {28, 553, 3, 4249}, {30, 556, 1, 4249}, {31, 557, 3, 4249}, {32, 558, 3, 4249}, {33, 559, 1, 4249}, {21, 545, 1, 4250}, {23, 548, 3, 4250}, {24, 549, 3, 4250}, {26, 552, 3, 4250}, {27, 553, 6, 4250}, {28, 554, 3, 4250}, {30, 557, 1, 4250}, {31, 558, 3, 4250}, {32, 559, 3, 4250}, {33, 560, 1, 4250}, {21, 546, 1, 4251}, {23, 549, 3, 4251}, {24, 550, 3, 4251}, {26, 553, 3, 4251}, {27, 554, 6, 4251}, {28, 555, 3, 4251}, {30, 558, 1, 4251}, {31, 559, 3, 4251}, {32, 560, 3, 4251}, {33, 561, 1, 4251}, {22, 541, 1, 4252}, {24, 542, 3, 4252}, {25, 543, 3, 4252}, {27, 544, 3, 4252}, {28, 545, 6, 4252}, {29, 546, 3, 4252}, {31, 547, 1, 4252}, {32, 548, 3, 4252}, {33, 549, 3, 4252}, {34, 550, 1, 4252}, {22, 542, 1, 4253}, {24, 544, 3, 4253}, {25, 545, 3, 4253}, {27, 547, 3, 4253}, {28, 548, 6, 4253}, {29, 549, 3, 4253}, {31, 551, 1, 4253}, {32, 552, 3, 4253}, {33, 553, 3, 4253}, {34, 554, 1, 4253}, {22, 543, 1, 4254}, {24, 545, 3, 4254}, {25, 546, 3, 4254}, {27, 548, 3, 4254}, {28, 549, 6, 4254}, {29, 550, 3, 4254}, {31, 552, 1, 4254}, {32, 553, 3, 4254}, {33, 554, 3, 4254}, {34, 555, 1, 4254}, {22, 544, 1, 4255}, {24, 547, 3, 4255}, {25, 548, 3, 4255}, {27, 551, 3, 4255}, {28, 552, 6, 4255}, {29, 553, 3, 4255}, {31, 556, 1, 4255}, {32, 557, 3, 4255}, {33, 558, 3, 4255}, {34, 559, 1, 4255}, {22, 545, 1, 4256}, {24, 548, 3, 4256}, {25, 549, 3, 4256}, {27, 552, 3, 4256}, {28, 553, 6, 4256}, {29, 554, 3, 4256}, {31, 557, 1, 4256}, {32, 558, 3, 4256}, {33, 559, 3, 4256}, {34, 560, 1, 4256}, {22, 546, 1, 4257}, {24, 549, 3, 4257}, {25, 550, 3, 4257}, {27, 553, 3, 4257}, {28, 554, 6, 4257}, {29, 555, 3, 4257}, {31, 558, 1, 4257}, {32, 559, 3, 4257}, {33, 560, 3, 4257}, {34, 561, 1, 4257}, {20, 526, 1, 4258}, {21, 527, 4, 4258}, {22, 528, 4, 4258}, {23, 529, 6, 4258}, {24, 530, 12, 4258}, {25, 531, 6, 4258}, {26, 532, 4, 4258}, {27, 533, 12, 4258}, {28, 534, 12, 4258}, {29, 535, 4, 4258}, {30, 536, 1, 4258}, {31, 537, 4, 4258}, {32, 538, 6, 4258}, {33, 539, 4, 4258}, {34, 540, 1, 4258}, {20, 541, 1, 4259}, {21, 542, 4, 4259}, {22, 543, 4, 4259}, {23, 544, 6, 4259}, {24, 545, 12, 4259}, {25, 546, 6, 4259}, {26, 547, 4, 4259}, {27, 548, 12, 4259}, {28, 549, 12, 4259}, {29, 550, 4, 4259}, {30, 551, 1, 4259}, {31, 552, 4, 4259}, {32, 553, 6, 4259}, {33, 554, 4, 4259}, {34, 555, 1, 4259}, {20, 542, 1, 4260}, {21, 544, 4, 4260}, {22, 545, 4, 4260}, {23, 547, 6, 4260}, {24, 548, 12, 4260}, {25, 549, 6, 4260}, {26, 551, 4, 4260}, {27, 552, 12, 4260}, {28, 553, 12, 4260}, {29, 554, 4, 4260}, {30, 556, 1, 4260}, {31, 557, 4, 4260}, {32, 558, 6, 4260}, {33, 559, 4, 4260}, {34, 560, 1, 4260}, {20, 543, 1, 4261}, {21, 545, 4, 4261}, {22, 546, 4, 4261}, {23, 548, 6, 4261}, {24, 549, 12, 4261}, {25, 550, 6, 4261}, {26, 552, 4, 4261}, {27, 553, 12, 4261}, {28, 554, 12, 4261}, {29, 555, 4, 4261}, {30, 557, 1, 4261}, {31, 558, 4, 4261}, {32, 559, 6, 4261}, {33, 560, 4, 4261}, {34, 561, 1, 4261}, {20, 562, 1, 4262}, {21, 563, 4, 4262}, {22, 564, 4, 4262}, {23, 565, 6, 4262}, {24, 566, 12, 4262}, {25, 567, 6, 4262}, {26, 568, 4, 4262}, {27, 569, 12, 4262}, {28, 570, 12, 4262}, {29, 571, 4, 4262}, {30, 572, 1, 4262}, {31, 573, 4, 4262}, {32, 574, 6, 4262}, {33, 575, 4, 4262}, {34, 576, 1, 4262}, {20, 563, 1, 4263}, {21, 565, 4, 4263}, {22, 566, 4, 4263}, {23, 568, 6, 4263}, {24, 569, 12, 4263}, {25, 570, 6, 4263}, {26, 572, 4, 4263}, {27, 573, 12, 4263}, {28, 574, 12, 4263}, {29, 575, 4, 4263}, {30, 577, 1, 4263}, {31, 578, 4, 4263}, {32, 579, 6, 4263}, {33, 580, 4, 4263}, {34, 581, 1, 4263}, {20, 564, 1, 4264}, {21, 566, 4, 4264}, {22, 567, 4, 4264}, {23, 569, 6, 4264}, {24, 570, 12, 4264}, {25, 571, 6, 4264}, {26, 573, 4, 4264}, {27, 574, 12, 4264}, {28, 575, 12, 4264}, {29, 576, 4, 4264}, {30, 578, 1, 4264}, {31, 579, 4, 4264}, {32, 580, 6, 4264}, {33, 581, 4, 4264}, {34, 582, 1, 4264}, {20, 565, 1, 4265}, {21, 568, 4, 4265}, {22, 569, 4, 4265}, {23, 572, 6, 4265}, {24, 573, 12, 4265}, {25, 574, 6, 4265}, {26, 577, 4, 4265}, {27, 578, 12, 4265}, {28, 579, 12, 4265}, {29, 580, 4, 4265}, {30, 583, 1, 4265}, {31, 584, 4, 4265}, {32, 585, 6, 4265}, {33, 586, 4, 4265}, {34, 587, 1, 4265}, {20, 566, 1, 4266}, {21, 569, 4, 4266}, {22, 570, 4, 4266}, {23, 573, 6, 4266}, {24, 574, 12, 4266}, {25, 575, 6, 4266}, {26, 578, 4, 4266}, {27, 579, 12, 4266}, {28, 580, 12, 4266}, {29, 581, 4, 4266}, {30, 584, 1, 4266}, {31, 585, 4, 4266}, {32, 586, 6, 4266}, {33, 587, 4, 4266}, {34, 588, 1, 4266}, {20, 567, 1, 4267}, {21, 570, 4, 4267}, {22, 571, 4, 4267}, {23, 574, 6, 4267}, {24, 575, 12, 4267}, {25, 576, 6, 4267}, {26, 579, 4, 4267}, {27, 580, 12, 4267}, {28, 581, 12, 4267}, {29, 582, 4, 4267}, {30, 585, 1, 4267}, {31, 586, 4, 4267}, {32, 587, 6, 4267}, {33, 588, 4, 4267}, {34, 589, 1, 4267}, {35, 510, 1, 4268}, {36, 511, 2, 4268}, {37, 512, 2, 4268}, {38, 513, 1, 4268}, {39, 514, 2, 4268}, {40, 515, 1, 4268}, {36, 510, 1, 4269}, {38, 511, 2, 4269}, {39, 512, 2, 4269}, {41, 513, 1, 4269},

{42, 514, 2, 4269}, {43, 515, 1, 4269}, {37, 510, 1, 4270}, {39, 511, 2, 4270}, {40, 512, 2, 4270}, {42, 513, 1, 4270}, {43, 514, 2, 4270}, {44, 515, 1, 4270}, {38, 510, 1, 4271}, {41, 511, 2, 4271}, {42, 512, 2, 4271}, {45, 513, 1, 4271}, {46, 514, 2, 4271}, {47, 515, 1, 4271}, {39, 510, 1, 4272}, {42, 511, 2, 4272}, {43, 512, 2, 4272}, {46, 513, 1, 4272}, {47, 514, 2, 4272}, {48, 515, 1, 4272}, {40, 510, 1, 4273}, {43, 511, 2, 4273}, {44, 512, 2, 4273}, {47, 513, 1, 4273}, {48, 514, 2, 4273}, {49, 515, 1, 4273}, {41, 510, 1, 4274}, {45, 511, 2, 4274}, {46, 512, 2, 4274}, {50, 513, 1, 4274}, {51, 514, 2, 4274}, {52, 515, 1, 4274}, {42, 510, 1, 4275}, {46, 511, 2, 4275}, {47, 512, 2, 4275}, {51, 513, 1, 4275}, {52, 514, 2, 4275}, {53, 515, 1, 4275}, {43, 510, 1, 4276}, {47, 511, 2, 4276}, {48, 512, 2, 4276}, {52, 513, 1, 4276}, {53, 514, 2, 4276}, {54, 515, 1, 4276}, {44, 510, 1, 4277}, {48, 511, 2, 4277}, {49, 512, 2, 4277}, {53, 513, 1, 4277}, {54, 514, 2, 4277}, {55, 515, 1, 4277}, {35, 516, 1, 4278}, {36, 517, 3, 4278}, {37, 518, 3, 4278}, {38, 519, 3, 4278}, {39, 520, 6, 4278}, {40, 521, 3, 4278}, {41, 522, 1, 4278}, {42, 523, 3, 4278}, {43, 524, 3, 4278}, {44, 525, 1, 4278}, {36, 516, 1, 4279}, {38, 517, 3, 4279}, {39, 518, 3, 4279}, {41, 519, 3, 4279}, {42, 520, 6, 4279}, {43, 521, 3, 4279}, {45, 522, 1, 4279}, {46, 523, 3, 4279}, {47, 524, 3, 4279}, {48, 525, 1, 4279}, {37, 516, 1, 4280}, {39, 517, 3, 4280}, {40, 518, 3, 4280}, {42, 519, 3, 4280}, {43, 520, 6, 4280}, {44, 521, 3, 4280}, {46, 522, 1, 4280}, {47, 523, 3, 4280}, {48, 524, 3, 4280}, {49, 525, 1, 4280}, {38, 516, 1, 4281}, {41, 517, 3, 4281}, {42, 518, 3, 4281}, {45, 519, 3, 4281}, {46, 520, 6, 4281}, {47, 521, 3, 4281}, {50, 522, 1, 4281}, {51, 523, 3, 4281}, {52, 524, 3, 4281}, {53, 525, 1, 4281}, {39, 516, 1, 4282}, {42, 517, 3, 4282}, {43, 518, 3, 4282}, {46, 519, 3, 4282}, {47, 520, 6, 4282}, {48, 521, 3, 4282}, {51, 522, 1, 4282}, {52, 523, 3, 4282}, {53, 524, 3, 4282}, {54, 525, 1, 4282}, {40, 516, 1, 4283}, {43, 517, 3, 4283}, {44, 518, 3, 4283}, {47, 519, 3, 4283}, {48, 520, 6, 4283}, {49, 521, 3, 4283}, {52, 522, 1, 4283}, {53, 523, 3, 4283}, {54, 524, 3, 4283}, {55, 525, 1, 4283}, {35, 526, 1, 4284}, {36, 527, 3, 4284}, {37, 528, 3, 4284}, {38, 529, 3, 4284}, {39, 530, 6, 4284}, {40, 531, 3, 4284}, {41, 532, 1, 4284}, {42, 533, 3, 4284}, {43, 534, 3, 4284}, {44, 535, 1, 4284}, {35, 527, 1, 4285}, {36, 529, 3, 4285}, {37, 530, 3, 4285}, {38, 532, 3, 4285}, {39, 533, 6, 4285}, {40, 534, 3, 4285}, {41, 536, 1, 4285}, {42, 537, 3, 4285}, {43, 538, 3, 4285}, {44, 539, 1, 4285}, {35, 528, 1, 4286}, {36, 530, 3, 4286}, {37, 531, 3, 4286}, {38, 533, 3, 4286}, {39, 534, 6, 4286}, {40, 535, 3, 4286}, {41, 537, 1, 4286}, {42, 538, 3, 4286}, {43, 539, 3, 4286}, {44, 540, 1, 4286}, {36, 526, 1, 4287}, {38, 527, 3, 4287}, {39, 528, 3, 4287}, {41, 529, 3, 4287}, {42, 530, 6, 4287}, {43, 531, 3, 4287}, {45, 532, 1, 4287}, {46, 533, 3, 4287}, {47, 534, 3, 4287}, {48, 535, 1, 4287}, {36, 527, 1, 4288}, {38, 529, 3, 4288}, {39, 530, 3, 4288}, {41, 532, 3, 4288}, {42, 533, 6, 4288}, {43, 534, 3, 4288}, {45, 536, 1, 4288}, {46, 537, 3, 4288}, {47, 538, 3, 4288}, {48, 539, 1, 4288}, {36, 528, 1, 4289}, {38, 530, 3, 4289}, {39, 531, 3, 4289}, {41, 533, 3, 4289}, {42, 534, 6, 4289}, {43, 535, 3, 4289}, {45, 537, 1, 4289}, {46, 538, 3, 4289}, {47, 539, 3, 4289}, {48, 540, 1, 4289}, {37, 526, 1, 4290}, {39, 527, 3, 4290}, {40, 528, 3, 4290}, {42, 529, 3, 4290}, {43, 530, 6, 4290}, {44, 531, 3, 4290}, {46, 532, 1, 4290}, {47, 533, 3, 4290}, {48, 534, 3, 4290}, {49, 535, 1, 4290}, {37, 527, 1, 4291}, {39, 529, 3, 4291}, {40, 530, 3, 4291}, {42, 532, 3, 4291}, {43, 533, 6, 4291}, {44, 534, 3, 4291}, {46, 536, 1, 4291}, {47, 537, 3, 4291}, {48, 538, 3, 4291}, {49, 539, 1, 4291}, {37, 528, 1, 4292}, {39, 530, 3, 4292}, {40, 531, 3, 4292}, {42, 533, 3, 4292}, {43, 534, 6, 4292}, {44, 535, 3, 4292}, {46, 537, 1, 4292}, {47, 538, 3, 4292}, {48, 539, 3, 4292}, {49, 540, 1, 4292}, {38, 526, 1, 4293}, {41, 527, 3, 4293}, {42, 528, 3, 4293}, {45, 529, 3, 4293}, {46, 530, 6, 4293}, {47, 531, 3, 4293}, {50, 532, 1, 4293}, {51, 533, 3, 4293}, {52, 534, 3, 4293}, {53, 535, 1, 4293}, {38, 527, 1, 4294}, {41, 529, 3, 4294}, {42, 530, 3, 4294}, {45, 532, 3, 4294}, {46, 533, 6, 4294}, {47, 534, 3, 4294}, {50, 536, 1, 4294}, {51, 537, 3, 4294}, {52, 538, 3, 4294}, {53, 539, 1, 4294}, {38, 528, 1, 4295}, {41, 530, 3, 4295}, {42, 531, 3, 4295}, {45, 533, 3, 4295}, {46, 534, 6, 4295}, {47, 535, 3, 4295}, {50, 537, 1, 4295}, {51, 538, 3, 4295}, {52, 539, 3, 4295}, {53, 540, 1, 4295}, {39, 526, 1, 4296}, {42, 527, 3, 4296}, {43, 528, 3, 4296}, {46, 529, 3, 4296}, {47, 530, 6, 4296}, {48, 531, 3, 4296}, {51, 532, 1, 4296}, {52, 533, 3, 4296}, {53, 534, 3, 4296}, {54, 535, 1, 4296}, {39, 527, 1, 4297}, {42, 529, 3, 4297}, {43, 530, 3, 4297}, {46, 532, 3, 4297}, {47, 533, 6, 4297}, {48, 534, 3, 4297}, {51, 536, 1, 4297}, {52, 537, 3, 4297}, {53, 538, 3, 4297}, {54, 539, 1, 4297}, {39, 528, 1, 4298}, {42, 530, 3, 4298}, {43, 531, 3, 4298}, {46, 533, 3, 4298}, {47, 534, 6, 4298}, {48, 535, 3, 4298}, {51, 537, 1, 4298}, {52, 538, 3, 4298}, {53, 539, 3, 4298}, {54, 540, 1, 4298}, {40, 526, 1, 4299}, {43, 527, 3, 4299}, {44, 528, 3, 4299}, {47, 529, 3, 4299}, {48, 530, 6, 4299}, {49, 531, 3, 4299}, {52, 532, 1, 4299}, {53, 533, 3, 4299}, {54, 534, 3, 4299}, {55, 535, 1, 4299}, {40, 527, 1, 4300}, {43, 529, 3, 4300}, {44, 530, 3, 4300}, {47, 532, 3, 4300}, {48, 533, 6, 4300}, {49, 534, 3, 4300}, {52, 536, 1, 4300}, {53, 537, 3, 4300}, {54, 538, 3, 4300}, {55, 539, 1, 4300}, {40, 528, 1, 4301}, {43, 530, 3, 4301}, {44, 531, 3, 4301}, {47, 533, 3, 4301}, {48, 534, 6, 4301}, {49, 535, 3, 4301}, {52, 537, 1, 4301}, {53, 538, 3, 4301}, {54, 539, 3, 4301}, {55, 540, 1, 4301}, {35, 526, 1, 4302}, {36, 527, 4, 4302}, {37, 528, 4, 4302}, {38, 529, 6, 4302}, {39, 530, 12, 4302}, {40, 531, 6, 4302}, {41, 532, 4, 4302}, {42, 533, 12, 4302}, {43, 534, 12, 4302}, {44, 535, 4, 4302}, {45, 536, 1, 4302}, {46, 537, 4, 4302}, {47, 538, 6, 4302}, {48, 539, 4, 4302}, {49, 540, 1, 4302}, {36, 526, 1, 4303}, {38, 527, 4, 4303}, {39, 528, 4, 4303}, {41, 529, 6, 4303}, {42, 530, 12, 4303},

{43, 531, 6, 4303}, {45, 532, 4, 4303}, {46, 533, 12, 4303}, {47, 534, 12, 4303}, {48, 535, 4, 4303}, {50, 536, 1, 4303}, {51, 537, 4, 4303}, {52, 538, 6, 4303}, {53, 539, 4, 4303}, {54, 540, 1, 4303}, {37, 526, 1, 4304}, {39, 527, 4, 4304}, {40, 528, 4, 4304}, {42, 529, 6, 4304}, {43, 530, 12, 4304}, {44, 531, 6, 4304}, {46, 532, 4, 4304}, {47, 533, 12, 4304}, {48, 534, 12, 4304}, {49, 535, 4, 4304}, {51, 536, 1, 4304}, {52, 537, 4, 4304}, {53, 538, 6, 4304}, {54, 539, 4, 4304}, {55, 540, 1, 4304}, {35, 541, 1, 4305}, {36, 542, 4, 4305}, {37, 543, 4, 4305}, {38, 544, 6, 4305}, {39, 545, 12, 4305}, {40, 546, 6, 4305}, {41, 547, 4, 4305}, {42, 548, 12, 4305}, {43, 549, 12, 4305}, {44, 550, 4, 4305}, {45, 551, 1, 4305}, {46, 552, 4, 4305}, {47, 553, 6, 4305}, {48, 554, 4, 4305}, {49, 555, 1, 4305}, {35, 542, 1, 4306}, {36, 544, 4, 4306}, {37, 545, 4, 4306}, {38, 547, 6, 4306}, {39, 548, 12, 4306}, {40, 549, 6, 4306}, {41, 551, 4, 4306}, {42, 552, 12, 4306}, {43, 553, 12, 4306}, {44, 554, 4, 4306}, {45, 556, 1, 4306}, {46, 557, 4, 4306}, {47, 558, 6, 4306}, {48, 559, 4, 4306}, {49, 560, 1, 4306}, {35, 543, 1, 4307}, {36, 545, 4, 4307}, {37, 546, 4, 4307}, {38, 548, 6, 4307}, {39, 549, 12, 4307}, {40, 550, 6, 4307}, {41, 552, 4, 4307}, {42, 553, 12, 4307}, {43, 554, 12, 4307}, {44, 555, 4, 4307}, {45, 557, 1, 4307}, {46, 558, 4, 4307}, {47, 559, 6, 4307}, {48, 560, 4, 4307}, {49, 561, 1, 4307}, {36, 541, 1, 4308}, {38, 542, 4, 4308}, {39, 543, 4, 4308}, {41, 544, 6, 4308}, {42, 545, 12, 4308}, {43, 546, 6, 4308}, {45, 547, 4, 4308}, {46, 548, 12, 4308}, {47, 549, 12, 4308}, {48, 550, 4, 4308}, {50, 551, 1, 4308}, {51, 552, 4, 4308}, {52, 553, 6, 4308}, {53, 554, 4, 4308}, {54, 555, 1, 4308}, {36, 542, 1, 4309}, {38, 544, 4, 4309}, {39, 545, 4, 4309}, {41, 547, 6, 4309}, {42, 548, 12, 4309}, {43, 549, 6, 4309}, {45, 551, 4, 4309}, {46, 552, 12, 4309}, {47, 553, 12, 4309}, {48, 554, 4, 4309}, {50, 556, 1, 4309}, {51, 557, 4, 4309}, {52, 558, 6, 4309}, {53, 559, 4, 4309}, {54, 560, 1, 4309}, {36, 543, 1, 4310}, {38, 545, 4, 4310}, {39, 546, 4, 4310}, {41, 548, 6, 4310}, {42, 549, 12, 4310}, {43, 550, 6, 4310}, {45, 552, 4, 4310}, {46, 553, 12, 4310}, {47, 554, 12, 4310}, {48, 555, 4, 4310}, {50, 557, 1, 4310}, {51, 558, 4, 4310}, {52, 559, 6, 4310}, {53, 560, 4, 4310}, {54, 561, 1, 4310}, {37, 541, 1, 4311}, {39, 542, 4, 4311}, {40, 543, 4, 4311}, {42, 544, 6, 4311}, {43, 545, 12, 4311}, {44, 546, 6, 4311}, {46, 547, 4, 4311}, {47, 548, 12, 4311}, {48, 549, 12, 4311}, {49, 550, 4, 4311}, {51, 551, 1, 4311}, {52, 552, 4, 4311}, {53, 553, 6, 4311}, {54, 554, 4, 4311}, {55, 555, 1, 4311}, {37, 542, 1, 4312}, {39, 544, 4, 4312}, {40, 545, 4, 4312}, {42, 547, 6, 4312}, {43, 548, 12, 4312}, {44, 549, 6, 4312}, {46, 551, 4, 4312}, {47, 552, 12, 4312}, {48, 553, 12, 4312}, {49, 554, 4, 4312}, {51, 556, 1, 4312}, {52, 557, 4, 4312}, {53, 558, 6, 4312}, {54, 559, 4, 4312}, {55, 560, 1, 4312}, {37, 543, 1, 4313}, {39, 545, 4, 4313}, {40, 546, 4, 4313}, {42, 548, 6, 4313}, {43, 549, 12, 4313}, {44, 550, 6, 4313}, {46, 552, 4, 4313}, {47, 553, 12, 4313}, {48, 554, 12, 4313}, {49, 555, 4, 4313}, {51, 557, 1, 4313}, {52, 558, 4, 4313}, {53, 559, 6, 4313}, {54, 560, 4, 4313}, {55, 561, 1, 4313}, {35, 541, 1, 4314}, {36, 542, 5, 4314}, {37, 543, 5, 4314}, {38, 544, 10, 4314}, {39, 545, 20, 4314}, {40, 546, 10, 4314}, {41, 547, 10, 4314}, {42, 548, 30, 4314}, {43, 549, 30, 4314}, {44, 550, 10, 4314}, {45, 551, 5, 4314}, {46, 552, 20, 4314}, {47, 553, 30, 4314}, {48, 554, 20, 4314}, {49, 555, 5, 4314}, {50, 556, 1, 4314}, {51, 557, 5, 4314}, {52, 558, 10, 4314}, {53, 559, 10, 4314}, {54, 560, 5, 4314}, {55, 561, 1, 4314}, {35, 562, 1, 4315}, {36, 563, 5, 4315}, {37, 564, 5, 4315}, {38, 565, 10, 4315}, {39, 566, 20, 4315}, {40, 567, 10, 4315}, {41, 568, 10, 4315}, {42, 569, 30, 4315}, {43, 570, 30, 4315}, {44, 571, 10, 4315}, {45, 572, 5, 4315}, {46, 573, 20, 4315}, {47, 574, 30, 4315}, {48, 575, 20, 4315}, {49, 576, 5, 4315}, {50, 577, 1, 4315}, {51, 578, 5, 4315}, {52, 579, 10, 4315}, {53, 580, 10, 4315}, {54, 581, 5, 4315}, {55, 582, 1, 4315}, {35, 563, 1, 4316}, {36, 565, 5, 4316}, {37, 566, 5, 4316}, {38, 568, 10, 4316}, {39, 569, 20, 4316}, {40, 570, 10, 4316}, {41, 572, 10, 4316}, {42, 573, 30, 4316}, {43, 574, 30, 4316}, {44, 575, 10, 4316}, {45, 577, 5, 4316}, {46, 578, 20, 4316}, {47, 579, 30, 4316}, {48, 580, 20, 4316}, {49, 581, 5, 4316}, {50, 583, 1, 4316}, {51, 584, 5, 4316}, {52, 585, 10, 4316}, {53, 586, 10, 4316}, {54, 587, 5, 4316}, {55, 588, 1, 4316}, {35, 564, 1, 4317}, {36, 566, 5, 4317}, {37, 567, 5, 4317}, {38, 569, 10, 4317}, {39, 570, 20, 4317}, {40, 571, 10, 4317}, {41, 573, 10, 4317}, {42, 574, 30, 4317}, {43, 575, 30, 4317}, {44, 576, 10, 4317}, {45, 578, 5, 4317}, {46, 579, 20, 4317}, {47, 580, 30, 4317}, {48, 581, 20, 4317}, {49, 582, 5, 4317}, {50, 584, 1, 4317}, {51, 585, 5, 4317}, {52, 586, 10, 4317}, {53, 587, 10, 4317}, {54, 588, 5, 4317}, {55, 589, 1, 4317}, {56, 516, 1, 4318}, {57, 517, 3, 4318}, {58, 518, 3, 4318}, {59, 519, 3, 4318}, {60, 520, 6, 4318}, {61, 521, 3, 4318}, {62, 522, 1, 4318}, {63, 523, 3, 4318}, {64, 524, 3, 4318}, {65, 525, 1, 4318}, {57, 516, 1, 4319}, {59, 517, 3, 4319}, {60, 518, 3, 4319}, {62, 519, 3, 4319}, {63, 520, 6, 4319}, {64, 521, 3, 4319}, {66, 522, 1, 4319}, {67, 523, 3, 4319}, {68, 524, 3, 4319}, {69, 525, 1, 4319}, {58, 516, 1, 4320}, {60, 517, 3, 4320}, {61, 518, 3, 4320}, {63, 519, 3, 4320}, {64, 520, 6, 4320}, {65, 521, 3, 4320}, {67, 522, 1, 4320}, {68, 523, 3, 4320}, {69, 524, 3, 4320}, {70, 525, 1, 4320}, {59, 516, 1, 4321}, {62, 517, 3, 4321}, {63, 518, 3, 4321}, {66, 519, 3, 4321}, {67, 520, 6, 4321}, {68, 521, 3, 4321}, {71, 522, 1, 4321}, {72, 523, 3, 4321}, {73, 524, 3, 4321}, {74, 525, 1, 4321}, {60, 516, 1, 4322}, {63, 517, 3, 4322}, {64, 518, 3, 4322}, {67, 519, 3, 4322}, {68, 520, 6, 4322}, {69, 521, 3, 4322}, {72, 522, 1, 4322}, {73, 523, 3, 4322}, {74, 524, 3, 4322}, {75, 525, 1, 4322}, {61, 516, 1, 4323}, {64, 517, 3, 4323}, {65, 518, 3, 4323}, {68, 519, 3, 4323}, {69, 520, 6, 4323}, {70, 521, 3, 4323}, {73, 522, 1, 4323},

{74, 523, 3, 4323}, {75, 524, 3, 4323}, {76, 525, 1, 4323}, {62, 516, 1, 4324}, {66, 517, 3, 4324}, {67, 518, 3, 4324}, {71, 519, 3, 4324}, {72, 520, 6, 4324}, {73, 521, 3, 4324}, {77, 522, 1, 4324}, {78, 523, 3, 4324}, {79, 524, 3, 4324}, {80, 525, 1, 4324}, {63, 516, 1, 4325}, {67, 517, 3, 4325}, {68, 518, 3, 4325}, {72, 519, 3, 4325}, {73, 520, 6, 4325}, {74, 521, 3, 4325}, {78, 522, 1, 4325}, {79, 523, 3, 4325}, {80, 524, 3, 4325}, {81, 525, 1, 4325}, {64, 516, 1, 4326}, {68, 517, 3, 4326}, {69, 518, 3, 4326}, {73, 519, 3, 4326}, {74, 520, 6, 4326}, {75, 521, 3, 4326}, {79, 522, 1, 4326}, {80, 523, 3, 4326}, {81, 524, 3, 4326}, {82, 525, 1, 4326}, {65, 516, 1, 4327}, {69, 517, 3, 4327}, {70, 518, 3, 4327}, {74, 519, 3, 4327}, {75, 520, 6, 4327}, {76, 521, 3, 4327}, {80, 522, 1, 4327}, {81, 523, 3, 4327}, {82, 524, 3, 4327}, {83, 525, 1, 4327}, {56, 526, 1, 4328}, {57, 527, 4, 4328}, {58, 528, 4, 4328}, {59, 529, 6, 4328}, {60, 530, 12, 4328}, {61, 531, 6, 4328}, {62, 532, 4, 4328}, {63, 533, 12, 4328}, {64, 534, 12, 4328}, {65, 535, 4, 4328}, {66, 536, 1, 4328}, {67, 537, 4, 4328}, {68, 538, 6, 4328}, {69, 539, 4, 4328}, {70, 540, 1, 4328}, {57, 526, 1, 4329}, {59, 527, 4, 4329}, {60, 528, 4, 4329}, {62, 529, 6, 4329}, {63, 530, 12, 4329}, {64, 531, 6, 4329}, {66, 532, 4, 4329}, {67, 533, 12, 4329}, {68, 534, 12, 4329}, {69, 535, 4, 4329}, {71, 536, 1, 4329}, {72, 537, 4, 4329}, {73, 538, 6, 4329}, {74, 539, 4, 4329}, {75, 540, 1, 4329}, {58, 526, 1, 4330}, {60, 527, 4, 4330}, {61, 528, 4, 4330}, {63, 529, 6, 4330}, {64, 530, 12, 4330}, {65, 531, 6, 4330}, {67, 532, 4, 4330}, {68, 533, 12, 4330}, {69, 534, 12, 4330}, {70, 535, 4, 4330}, {72, 536, 1, 4330}, {73, 537, 4, 4330}, {74, 538, 6, 4330}, {75, 539, 4, 4330}, {76, 540, 1, 4330}, {59, 526, 1, 4331}, {62, 527, 4, 4331}, {63, 528, 4, 4331}, {66, 529, 6, 4331}, {67, 530, 12, 4331}, {68, 531, 6, 4331}, {71, 532, 4, 4331}, {72, 533, 12, 4331}, {73, 534, 12, 4331}, {74, 535, 4, 4331}, {77, 536, 1, 4331}, {78, 537, 4, 4331}, {79, 538, 6, 4331}, {80, 539, 4, 4331}, {81, 540, 1, 4331}, {60, 526, 1, 4332}, {63, 527, 4, 4332}, {64, 528, 4, 4332}, {67, 529, 6, 4332}, {68, 530, 12, 4332}, {69, 531, 6, 4332}, {72, 532, 4, 4332}, {73, 533, 12, 4332}, {74, 534, 12, 4332}, {75, 535, 4, 4332}, {78, 536, 1, 4332}, {79, 537, 4, 4332}, {80, 538, 6, 4332}, {81, 539, 4, 4332}, {82, 540, 1, 4332}, {61, 526, 1, 4333}, {64, 527, 4, 4333}, {65, 528, 4, 4333}, {68, 529, 6, 4333}, {69, 530, 12, 4333}, {70, 531, 6, 4333}, {73, 532, 4, 4333}, {74, 533, 12, 4333}, {75, 534, 12, 4333}, {76, 535, 4, 4333}, {79, 536, 1, 4333}, {80, 537, 4, 4333}, {81, 538, 6, 4333}, {82, 539, 4, 4333}, {83, 540, 1, 4333}, {56, 541, 1, 4334}, {57, 542, 5, 4334}, {58, 543, 5, 4334}, {59, 544, 10, 4334}, {60, 545, 20, 4334}, {61, 546, 10, 4334}, {62, 547, 10, 4334}, {63, 548, 30, 4334}, {64, 549, 30, 4334}, {65, 550, 10, 4334}, {66, 551, 5, 4334}, {67, 552, 20, 4334}, {68, 553, 30, 4334}, {69, 554, 20, 4334}, {70, 555, 5, 4334}, {71, 556, 1, 4334}, {72, 557, 5, 4334}, {73, 558, 10, 4334}, {74, 559, 10, 4334}, {75, 560, 5, 4334}, {76, 561, 1, 4334}, {57, 541, 1, 4335}, {59, 542, 5, 4335}, {60, 543, 5, 4335}, {62, 544, 10, 4335}, {63, 545, 20, 4335}, {64, 546, 10, 4335}, {66, 547, 10, 4335}, {67, 548, 30, 4335}, {68, 549, 30, 4335}, {69, 550, 10, 4335}, {71, 551, 5, 4335}, {72, 552, 20, 4335}, {73, 553, 30, 4335}, {74, 554, 20, 4335}, {75, 555, 5, 4335}, {77, 556, 1, 4335}, {78, 557, 5, 4335}, {79, 558, 10, 4335}, {80, 559, 10, 4335}, {81, 560, 5, 4335}, {82, 561, 1, 4335}, {58, 541, 1, 4336}, {60, 542, 5, 4336}, {61, 543, 5, 4336}, {63, 544, 10, 4336}, {64, 545, 20, 4336}, {65, 546, 10, 4336}, {67, 547, 10, 4336}, {68, 548, 30, 4336}, {69, 549, 30, 4336}, {70, 550, 10, 4336}, {72, 551, 5, 4336}, {73, 552, 20, 4336}, {74, 553, 30, 4336}, {75, 554, 20, 4336}, {76, 555, 5, 4336}, {78, 556, 1, 4336}, {79, 557, 5, 4336}, {80, 558, 10, 4336}, {81, 559, 10, 4336}, {82, 560, 5, 4336}, {83, 561, 1, 4336}, {56, 562, 1, 4337}, {57, 563, 6, 4337}, {58, 564, 6, 4337}, {59, 565, 15, 4337}, {60, 566, 30, 4337}, {61, 567, 15, 4337}, {62, 568, 20, 4337}, {63, 569, 60, 4337}, {64, 570, 60, 4337}, {65, 571, 20, 4337}, {66, 572, 15, 4337}, {67, 573, 60, 4337}, {68, 574, 90, 4337}, {69, 575, 60, 4337}, {70, 576, 15, 4337}, {71, 577, 6, 4337}, {72, 578, 30, 4337}, {73, 579, 60, 4337}, {74, 580, 60, 4337}, {75, 581, 30, 4337}, {76, 582, 6, 4337}, {77, 583, 1, 4337}, {78, 584, 6, 4337}, {79, 585, 15, 4337}, {80, 586, 20, 4337}, {81, 587, 15, 4337}, {82, 588, 6, 4337}, {83, 589, 1, 4337}, {84, 590, 1, 4338}, {85, 591, 7, 4338}, {86, 592, 7, 4338}, {87, 593, 21, 4338}, {88, 594, 42, 4338}, {89, 595, 21, 4338}, {90, 596, 35, 4338}, {91, 597, 105, 4338}, {92, 598, 105, 4338}, {93, 599, 35, 4338}, {94, 600, 35, 4338}, {95, 601, 140, 4338}, {96, 602, 210, 4338}, {97, 603, 140, 4338}, {98, 604, 35, 4338}, {99, 605, 21, 4338}, {100, 606, 105, 4338}, {101, 607, 210, 4338}, {102, 608, 210, 4338}, {103, 609, 105, 4338}, {104, 610, 21, 4338}, {105, 611, 7, 4338}, {106, 612, 42, 4338}, {107, 613, 105, 4338}, {108, 614, 140, 4338}, {109, 615, 105, 4338}, {110, 616, 42, 4338}, {111, 617, 7, 4338}, {112, 618, 1, 4338}, {113, 619, 7, 4338}, {114, 620, 21, 4338}, {115, 621, 35, 4338}, {116, 622, 35, 4338}, {117, 623, 21, 4338}, {118, 624, 7, 4338}, {119, 625, 1, 4338}, {0, 626, 1, 4339}, {1, 627, 1, 4340}, {2, 628, 1, 4340}, {3, 629, 1, 4340}, {1, 630, 1, 4341}, {2, 631, 1, 4341}, {3, 632, 1, 4341}, {1, 631, 1, 4342}, {2, 633, 1, 4342}, {3, 634, 1, 4342}, {1, 632, 1, 4343}, {2, 634, 1, 4343}, {3, 635, 1, 4343}, {1, 636, 1, 4344}, {2, 637, 1, 4344}, {3, 638, 1, 4344}, {1, 637, 1, 4345}, {2, 639, 1, 4345}, {3, 640, 1, 4345}, {1, 638, 1, 4346}, {2, 640, 1, 4346}, {3, 641, 1, 4346}, {1, 639, 1, 4347}, {2, 642, 1, 4347}, {3, 643, 1, 4347}, {1, 640, 1, 4348}, {2, 643, 1, 4348}, {3, 644, 1, 4348}, {1, 641, 1, 4349}, {2, 644, 1, 4349}, {3, 645, 1, 4349}, {4, 627, 1, 4350}, {5, 628, 1, 4350}, {6, 629, 1, 4350}, {5, 627, 1, 4351}, {7, 628, 1, 4351}, {8, 629, 1, 4351}, {6, 627, 1, 4352}, {8, 628, 1, 4352}, {9, 629, 1, 4352}, {4, 630, 1, 4353}, {5, 631, 1,

4353}, {6, 632, 1, 4353}, {4, 631, 1, 4354}, {5, 633, 1, 4354}, {6, 634, 1, 4354}, {4, 632, 1, 4355}, {5, 634, 1, 4355}, {6, 635, 1, 4355}, {5, 630, 1, 4356}, {7, 631, 1, 4356}, {8, 632, 1, 4356}, {5, 631, 1, 4357}, {7, 633, 1, 4357}, {8, 634, 1, 4357}, {5, 632, 1, 4358}, {7, 634, 1, 4358}, {8, 635, 1, 4358}, {6, 630, 1, 4359}, {8, 631, 1, 4359}, {9, 632, 1, 4359}, {6, 631, 1, 4360}, {8, 633, 1, 4360}, {9, 634, 1, 4360}, {6, 632, 1, 4361}, {8, 634, 1, 4361}, {9, 635, 1, 4361}, {4, 630, 1, 4362}, {5, 631, 2, 4362}, {6, 632, 2, 4362}, {7, 633, 1, 4362}, {8, 634, 2, 4362}, {9, 635, 1, 4362}, {4, 636, 1, 4363}, {5, 637, 2, 4363}, {6, 638, 2, 4363}, {7, 639, 1, 4363}, {8, 640, 2, 4363}, {9, 641, 1, 4363}, {4, 637, 1, 4364}, {5, 639, 2, 4364}, {6, 640, 2, 4364}, {7, 642, 1, 4364}, {8, 643, 2, 4364}, {9, 644, 1, 4364}, {4, 638, 1, 4365}, {5, 640, 2, 4365}, {6, 641, 2, 4365}, {7, 643, 1, 4365}, {8, 644, 2, 4365}, {9, 645, 1, 4365}, {4, 646, 1, 4366}, {5, 647, 2, 4366}, {6, 648, 2, 4366}, {7, 649, 1, 4366}, {8, 650, 2, 4366}, {9, 651, 1, 4366}, {4, 647, 1, 4367}, {5, 649, 2, 4367}, {6, 650, 2, 4367}, {7, 652, 1, 4367}, {8, 653, 2, 4367}, {9, 654, 1, 4367}, {4, 648, 1, 4368}, {5, 650, 2, 4368}, {6, 651, 2, 4368}, {7, 653, 1, 4368}, {8, 654, 2, 4368}, {9, 655, 1, 4368}, {4, 649, 1, 4369}, {5, 652, 2, 4369}, {6, 653, 2, 4369}, {7, 656, 1, 4369}, {8, 657, 2, 4369}, {9, 658, 1, 4369}, {4, 650, 1, 4370}, {5, 653, 2, 4370}, {6, 654, 2, 4370}, {7, 657, 1, 4370}, {8, 658, 2, 4370}, {9, 659, 1, 4370}, {4, 651, 1, 4371}, {5, 654, 2, 4371}, {6, 655, 2, 4371}, {7, 658, 1, 4371}, {8, 659, 2, 4371}, {9, 660, 1, 4371}, {10, 627, 1, 4372}, {11, 628, 1, 4372}, {12, 629, 1, 4372}, {11, 627, 1, 4373}, {13, 628, 1, 4373}, {14, 629, 1, 4373}, {12, 627, 1, 4374}, {14, 628, 1, 4374}, {15, 629, 1, 4374}, {13, 627, 1, 4375}, {16, 628, 1, 4375}, {17, 629, 1, 4375}, {14, 627, 1, 4376}, {17, 628, 1, 4376}, {18, 629, 1, 4376}, {15, 627, 1, 4377}, {18, 628, 1, 4377}, {19, 629, 1, 4377}, {10, 630, 1, 4378}, {11, 631, 2, 4378}, {12, 632, 2, 4378}, {13, 633, 1, 4378}, {14, 634, 2, 4378}, {15, 635, 1, 4378}, {11, 630, 1, 4379}, {13, 631, 2, 4379}, {14, 632, 2, 4379}, {16, 633, 1, 4379}, {17, 634, 2, 4379}, {18, 635, 1, 4379}, {12, 630, 1, 4380}, {14, 631, 2, 4380}, {15, 632, 2, 4380}, {17, 633, 1, 4380}, {18, 634, 2, 4380}, {19, 635, 1, 4380}, {10, 636, 1, 4381}, {11, 637, 2, 4381}, {12, 638, 2, 4381}, {13, 639, 1, 4381}, {14, 640, 2, 4381}, {15, 641, 1, 4381}, {10, 637, 1, 4382}, {11, 639, 2, 4382}, {12, 640, 2, 4382}, {13, 642, 1, 4382}, {14, 643, 2, 4382}, {15, 644, 1, 4382}, {10, 638, 1, 4383}, {11, 640, 2, 4383}, {12, 641, 2, 4383}, {13, 643, 1, 4383}, {14, 644, 2, 4383}, {15, 645, 1, 4383}, {11, 636, 1, 4384}, {13, 637, 2, 4384}, {14, 638, 2, 4384}, {16, 639, 1, 4384}, {17, 640, 2, 4384}, {18, 641, 1, 4384}, {11, 637, 1, 4385}, {13, 639, 2, 4385}, {14, 640, 2, 4385}, {16, 642, 1, 4385}, {17, 643, 2, 4385}, {18, 644, 1, 4385}, {11, 638, 1, 4386}, {13, 640, 2, 4386}, {14, 641, 2, 4386}, {16, 643, 1, 4386}, {17, 644, 2, 4386}, {18, 645, 1, 4386}, {12, 636, 1, 4387}, {14, 637, 2, 4387}, {15, 638, 2, 4387}, {17, 639, 1, 4387}, {18, 640, 2, 4387}, {19, 641, 1, 4387}, {12, 637, 1, 4388}, {14, 639, 2, 4388}, {15, 640, 2, 4388}, {17, 642, 1, 4388}, {18, 643, 2, 4388}, {19, 644, 1, 4388}, {12, 638, 1, 4389}, {14, 640, 2, 4389}, {15, 641, 2, 4389}, {17, 643, 1, 4389}, {18, 644, 2, 4389}, {19, 645, 1, 4389}, {10, 636, 1, 4390}, {11, 637, 3, 4390}, {12, 638, 3, 4390}, {13, 639, 3, 4390}, {14, 640, 6, 4390}, {15, 641, 3, 4390}, {16, 642, 1, 4390}, {17, 643, 3, 4390}, {18, 644, 3, 4390}, {19, 645, 1, 4390}, {10, 646, 1, 4391}, {11, 647, 3, 4391}, {12, 648, 3, 4391}, {13, 649, 3, 4391}, {14, 650, 6, 4391}, {15, 651, 3, 4391}, {16, 652, 1, 4391}, {17, 653, 3, 4391}, {18, 654, 3, 4391}, {19, 655, 1, 4391}, {10, 647, 1, 4392}, {11, 649, 3, 4392}, {12, 650, 3, 4392}, {13, 652, 3, 4392}, {14, 653, 6, 4392}, {15, 654, 3, 4392}, {16, 656, 1, 4392}, {17, 657, 3, 4392}, {18, 658, 3, 4392}, {19, 659, 1, 4392}, {10, 648, 1, 4393}, {11, 650, 3, 4393}, {12, 651, 3, 4393}, {13, 653, 3, 4393}, {14, 654, 6, 4393}, {15, 655, 3, 4393}, {16, 657, 1, 4393}, {17, 658, 3, 4393}, {18, 659, 3, 4393}, {19, 660, 1, 4393}, {20, 630, 1, 4394}, {21, 631, 2, 4394}, {22, 632, 2, 4394}, {23, 633, 1, 4394}, {24, 634, 2, 4394}, {25, 635, 1, 4394}, {21, 630, 1, 4395}, {23, 631, 2, 4395}, {24, 632, 2, 4395}, {26, 633, 1, 4395}, {27, 634, 2, 4395}, {28, 635, 1, 4395}, {22, 630, 1, 4396}, {24, 631, 2, 4396}, {25, 632, 2, 4396}, {27, 633, 1, 4396}, {28, 634, 2, 4396}, {29, 635, 1, 4396}, {23, 630, 1, 4397}, {26, 631, 2, 4397}, {27, 632, 2, 4397}, {30, 633, 1, 4397}, {31, 634, 2, 4397}, {32, 635, 1, 4397}, {24, 630, 1, 4398}, {27, 631, 2, 4398}, {28, 632, 2, 4398}, {31, 633, 1, 4398}, {32, 634, 2, 4398}, {33, 635, 1, 4398}, {25, 630, 1, 4399}, {28, 631, 2, 4399}, {29, 632, 2, 4399}, {32, 633, 1, 4399}, {33, 634, 2, 4399}, {34, 635, 1, 4399}, {20, 636, 1, 4400}, {21, 637, 3, 4400}, {22, 638, 3, 4400}, {23, 639, 3, 4400}, {24, 640, 6, 4400}, {25, 641, 3, 4400}, {26, 642, 1, 4400}, {27, 643, 3, 4400}, {28, 644, 3, 4400}, {29, 645, 1, 4400}, {21, 636, 1, 4401}, {23, 637, 3, 4401}, {24, 638, 3, 4401}, {26, 639, 3, 4401}, {27, 640, 6, 4401}, {28, 641, 3, 4401}, {30, 642, 1, 4401}, {31, 643, 3, 4401}, {32, 644, 3, 4401}, {33, 645, 1, 4401}, {22, 636, 1, 4402}, {24, 637, 3, 4402}, {25, 638, 3, 4402}, {27, 639, 3, 4402}, {28, 640, 6, 4402}, {29, 641, 3, 4402}, {31, 642, 1, 4402}, {32, 643, 3, 4402}, {33, 644, 3, 4402}, {34, 645, 1, 4402}, {20, 646, 1, 4403}, {21, 647, 4, 4403}, {22, 648, 4, 4403}, {23, 649, 6, 4403}, {24, 650, 12, 4403}, {25, 651, 6, 4403}, {26, 652, 4, 4403}, {27, 653, 12, 4403}, {28, 654, 12, 4403}, {29, 655, 4, 4403}, {30, 656, 1, 4403}, {31, 657, 4, 4403}, {32, 658, 6, 4403}, {33, 659, 4, 4403}, {34, 660, 1, 4403}, {35, 661, 1, 4404}, {36, 662, 5, 4404}, {37, 663, 5, 4404}, {38, 664, 10, 4404}, {39, 665, 20, 4404}, {40, 666, 10, 4404}, {41, 667, 10, 4404}, {42, 668, 30, 4404}, {43, 669, 30, 4404}, {44, 670, 10, 4404}, {45, 671, 5, 4404}, {46, 672, 20, 4404}, {47, 673, 30, 4404}, {48, 674, 20, 4404}, {49, 675, 5, 4404}, {50, 676, 1,

4404}, {51, 677, 5, 4404}, {52, 678, 10, 4404}, {53, 679, 10, 4404}, {54, 680, 5, 4404}, {55, 681, 1, 4404}, {0, 682, 1, 4405}, {1, 683, 1, 4406}, {2, 684, 1, 4406}, {3, 685, 1, 4406}, {1, 686, 1, 4407}, {2, 687, 1, 4407}, {3, 688, 1, 4407}, {1, 687, 1, 4408}, {2, 689, 1, 4408}, {3, 690, 1, 4408}, {1, 688, 1, 4409}, {2, 690, 1, 4409}, {3, 691, 1, 4409}, {4, 683, 1, 4410}, {5, 684, 1, 4410}, {6, 685, 1, 4410}, {5, 683, 1, 4411}, {7, 684, 1, 4411}, {8, 685, 1, 4411}, {6, 683, 1, 4412}, {8, 684, 1, 4412}, {9, 685, 1, 4412}, {4, 686, 1, 4413}, {5, 687, 2, 4413}, {6, 688, 2, 4413}, {7, 689, 1, 4413}, {8, 690, 2, 4413}, {9, 691, 1, 4413}, {10, 692, 1, 4414}, {11, 693, 3, 4414}, {12, 694, 3, 4414}, {13, 695, 3, 4414}, {14, 696, 6, 4414}, {15, 697, 3, 4414}, {16, 698, 1, 4414}, {17, 699, 3, 4414}, {18, 700, 3, 4414}, {19, 701, 1, 4414}, {0, 702, 1, 4415}, {1, 703, 1, 4416}, {2, 704, 1, 4416}, {3, 705, 1, 4416}, {286, 286, 1, 4417}, {287, 287, 1, 4418}, {288, 288, 1, 4418}, {289, 289, 1, 4418}, {287, 290, 1, 4419}, {288, 291, 1, 4419}, {289, 292, 1, 4419}, {287, 291, 1, 4420}, {288, 293, 1, 4420}, {289, 294, 1, 4420}, {287, 292, 1, 4421}, {288, 294, 1, 4421}, {289, 295, 1, 4421}, {287, 296, 1, 4422}, {288, 297, 1, 4422}, {289, 298, 1, 4422}, {287, 297, 1, 4423}, {288, 299, 1, 4423}, {289, 300, 1, 4423}, {287, 298, 1, 4424}, {288, 300, 1, 4424}, {289, 301, 1, 4424}, {287, 299, 1, 4425}, {288, 302, 1, 4425}, {289, 303, 1, 4425}, {287, 300, 1, 4426}, {288, 303, 1, 4426}, {289, 304, 1, 4426}, {287, 301, 1, 4427}, {288, 304, 1, 4427}, {289, 305, 1, 4427}, {290, 290, 1, 4428}, {291, 291, 1, 4428}, {292, 292, 1, 4428}, {290, 291, 1, 4429}, {291, 293, 1, 4429}, {292, 294, 1, 4429}, {290, 292, 1, 4430}, {291, 294, 1, 4430}, {292, 295, 1, 4430}, {291, 291, 1, 4431}, {293, 293, 1, 4431}, {294, 294, 1, 4431}, {291, 292, 1, 4432}, {293, 294, 1, 4432}, {294, 295, 1, 4432}, {292, 292, 1, 4433}, {294, 294, 1, 4433}, {295, 295, 1, 4433}, {290, 296, 1, 4434}, {291, 297, 1, 4434}, {292, 298, 1, 4434}, {290, 297, 1, 4435}, {291, 299, 1, 4435}, {292, 300, 1, 4435}, {290, 298, 1, 4436}, {291, 300, 1, 4436}, {292, 301, 1, 4436}, {290, 299, 1, 4437}, {291, 302, 1, 4437}, {292, 303, 1, 4437}, {290, 300, 1, 4438}, {291, 303, 1, 4438}, {292, 304, 1, 4438}, {290, 301, 1, 4439}, {291, 304, 1, 4439}, {292, 305, 1, 4439}, {291, 296, 1, 4440}, {293, 297, 1, 4440}, {294, 298, 1, 4440}, {291, 297, 1, 4441}, {293, 299, 1, 4441}, {294, 300, 1, 4441}, {291, 298, 1, 4442}, {293, 300, 1, 4442}, {294, 301, 1, 4442}, {291, 299, 1, 4443}, {293, 302, 1, 4443}, {294, 303, 1, 4443}, {291, 300, 1, 4444}, {293, 303, 1, 4444}, {294, 304, 1, 4444}, {291, 301, 1, 4445}, {293, 304, 1, 4445}, {294, 305, 1, 4445}, {292, 296, 1, 4446}, {294, 297, 1, 4446}, {295, 298, 1, 4446}, {292, 297, 1, 4447}, {294, 299, 1, 4447}, {295, 300, 1, 4447}, {292, 298, 1, 4448}, {294, 300, 1, 4448}, {295, 301, 1, 4448}, {292, 299, 1, 4449}, {294, 302, 1, 4449}, {295, 303, 1, 4449}, {292, 300, 1, 4450}, {294, 303, 1, 4450}, {295, 304, 1, 4450}, {292, 301, 1, 4451}, {294, 304, 1, 4451}, {295, 305, 1, 4451}, {290, 306, 1, 4452}, {291, 307, 1, 4452}, {292, 308, 1, 4452}, {290, 307, 1, 4453}, {291, 309, 1, 4453}, {292, 310, 1, 4453}, {290, 308, 1, 4454}, {291, 310, 1, 4454}, {292, 311, 1, 4454}, {290, 309, 1, 4455}, {291, 312, 1, 4455}, {292, 313, 1, 4455}, {290, 310, 1, 4456}, {291, 313, 1, 4456}, {292, 314, 1, 4456}, {290, 311, 1, 4457}, {291, 314, 1, 4457}, {292, 315, 1, 4457}, {290, 312, 1, 4458}, {291, 316, 1, 4458}, {292, 317, 1, 4458}, {290, 313, 1, 4459}, {291, 317, 1, 4459}, {292, 318, 1, 4459}, {290, 314, 1, 4460}, {291, 318, 1, 4460}, {292, 319, 1, 4460}, {290, 315, 1, 4461}, {291, 319, 1, 4461}, {292, 320, 1, 4461}, {291, 306, 1, 4462}, {293, 307, 1, 4462}, {294, 308, 1, 4462}, {291, 307, 1, 4463}, {293, 309, 1, 4463}, {294, 310, 1, 4463}, {291, 308, 1, 4464}, {293, 310, 1, 4464}, {294, 311, 1, 4464}, {291, 309, 1, 4465}, {293, 312, 1, 4465}, {294, 313, 1, 4465}, {291, 310, 1, 4466}, {293, 313, 1, 4466}, {294, 314, 1, 4466}, {291, 311, 1, 4467}, {293, 314, 1, 4467}, {294, 315, 1, 4467}, {291, 312, 1, 4468}, {293, 316, 1, 4468}, {294, 317, 1, 4468}, {291, 313, 1, 4469}, {293, 317, 1, 4469}, {294, 318, 1, 4469}, {291, 314, 1, 4470}, {293, 318, 1, 4470}, {294, 319, 1, 4470}, {291, 315, 1, 4471}, {293, 319, 1, 4471}, {294, 320, 1, 4471}, {292, 306, 1, 4472}, {294, 307, 1, 4472}, {295, 308, 1, 4472}, {292, 307, 1, 4473}, {294, 309, 1, 4473}, {295, 310, 1, 4473}, {292, 308, 1, 4474}, {294, 310, 1, 4474}, {295, 311, 1, 4474}, {292, 309, 1, 4475}, {294, 312, 1, 4475}, {295, 313, 1, 4475}, {292, 310, 1, 4476}, {294, 313, 1, 4476}, {295, 314, 1, 4476}, {292, 311, 1, 4477}, {294, 314, 1, 4477}, {295, 315, 1, 4477}, {292, 312, 1, 4478}, {294, 316, 1, 4478}, {295, 317, 1, 4478}, {292, 313, 1, 4479}, {294, 317, 1, 4479}, {295, 318, 1, 4479}, {292, 314, 1, 4480}, {294, 318, 1, 4480}, {295, 319, 1, 4480}, {292, 315, 1, 4481}, {294, 319, 1, 4481}, {295, 320, 1, 4481}, {290, 290, 1, 4482}, {291, 291, 2, 4482}, {292, 292, 2, 4482}, {293, 293, 1, 4482}, {294, 294, 2, 4482}, {295, 295, 1, 4482}, {290, 296, 1, 4483}, {291, 297, 2, 4483}, {292, 298, 2, 4483}, {293, 299, 1, 4483}, {294, 300, 2, 4483}, {295, 301, 1, 4483}, {290, 297, 1, 4484}, {291, 299, 2, 4484}, {292, 300, 2, 4484}, {293, 302, 1, 4484}, {294, 303, 2, 4484}, {295, 304, 1, 4484}, {290, 298, 1, 4485}, {291, 300, 2, 4485}, {292, 301, 2, 4485}, {293, 303, 1, 4485}, {294, 304, 2, 4485}, {295, 305, 1, 4485}, {290, 306, 1, 4486}, {291, 307, 2, 4486}, {292, 308, 2, 4486}, {293, 309, 1, 4486}, {294, 310, 2, 4486}, {295, 311, 1, 4486}, {290, 307, 1, 4487}, {291, 309, 2, 4487}, {292, 310, 2, 4487}, {293, 312, 1, 4487}, {294, 313, 2, 4487}, {295, 314, 1, 4487}, {290, 308, 1, 4488}, {291, 310, 2, 4488}, {292, 311, 2, 4488}, {293, 313, 1, 4488}, {294, 314, 2, 4488}, {295, 315, 1, 4488}, {290, 309, 1, 4489}, {291, 312, 2, 4489}, {292, 313, 2, 4489}, {293, 316, 1, 4489}, {294, 317, 2, 4489}, {295, 318, 1, 4489}, {290, 310, 1, 4490}, {291, 313, 2, 4490}, {292, 314, 2, 4490}, {293, 317, 1, 4490}, {294, 318, 2, 4490}, {295, 319, 1, 4490}, {290, 311, 1, 4491}, {291, 314, 2, 4491}, {292, 315,

2, 4491}, {293, 318, 1, 4491}, {294, 319, 2, 4491}, {295, 320, 1, 4491}, {290, 321, 1,
4492}, {291, 322, 2, 4492}, {292, 323, 2, 4492}, {293, 324, 1, 4492}, {294, 325, 2, 4492},
{295, 326, 1, 4492}, {290, 322, 1, 4493}, {291, 324, 2, 4493}, {292, 325, 2, 4493}, {293,
327, 1, 4493}, {294, 328, 2, 4493}, {295, 329, 1, 4493}, {290, 323, 1, 4494}, {291, 325,
2, 4494}, {292, 326, 2, 4494}, {293, 328, 1, 4494}, {294, 329, 2, 4494}, {295, 330, 1,
4494}, {290, 324, 1, 4495}, {291, 327, 2, 4495}, {292, 328, 2, 4495}, {293, 331, 1, 4495},
{294, 332, 2, 4495}, {295, 333, 1, 4495}, {290, 325, 1, 4496}, {291, 328, 2, 4496}, {292,
329, 2, 4496}, {293, 332, 1, 4496}, {294, 333, 2, 4496}, {295, 334, 1, 4496}, {290, 326,
1, 4497}, {291, 329, 2, 4497}, {292, 330, 2, 4497}, {293, 333, 1, 4497}, {294, 334, 2,
4497}, {295, 335, 1, 4497}, {290, 327, 1, 4498}, {291, 331, 2, 4498}, {292, 332, 2, 4498},
{293, 336, 1, 4498}, {294, 337, 2, 4498}, {295, 338, 1, 4498}, {290, 328, 1, 4499}, {291,
332, 2, 4499}, {292, 333, 2, 4499}, {293, 337, 1, 4499}, {294, 338, 2, 4499}, {295, 339,
1, 4499}, {290, 329, 1, 4500}, {291, 333, 2, 4500}, {292, 334, 2, 4500}, {293, 338, 1,
4500}, {294, 339, 2, 4500}, {295, 340, 1, 4500}, {290, 330, 1, 4501}, {291, 334, 2, 4501},
{292, 335, 2, 4501}, {293, 339, 1, 4501}, {294, 340, 2, 4501}, {295, 341, 1, 4501}, {296,
296, 1, 4502}, {297, 297, 1, 4502}, {298, 298, 1, 4502}, {296, 297, 1, 4503}, {297, 299,
1, 4503}, {298, 300, 1, 4503}, {296, 298, 1, 4504}, {297, 300, 1, 4504}, {298, 301, 1,
4504}, {296, 299, 1, 4505}, {297, 302, 1, 4505}, {298, 303, 1, 4505}, {296, 300, 1, 4506},
{297, 303, 1, 4506}, {298, 304, 1, 4506}, {296, 301, 1, 4507}, {297, 304, 1, 4507}, {298,
305, 1, 4507}, {297, 297, 1, 4508}, {299, 299, 1, 4508}, {300, 300, 1, 4508}, {297, 298,
1, 4509}, {299, 300, 1, 4509}, {300, 301, 1, 4509}, {297, 299, 1, 4510}, {299, 302, 1,
4510}, {300, 303, 1, 4510}, {297, 300, 1, 4511}, {299, 303, 1, 4511}, {300, 304, 1, 4511},
{297, 301, 1, 4512}, {299, 304, 1, 4512}, {300, 305, 1, 4512}, {298, 298, 1, 4513}, {300,
300, 1, 4513}, {301, 301, 1, 4513}, {298, 299, 1, 4514}, {300, 302, 1, 4514}, {301, 303,
1, 4514}, {298, 300, 1, 4515}, {300, 303, 1, 4515}, {301, 304, 1, 4515}, {298, 301, 1,
4516}, {300, 304, 1, 4516}, {301, 305, 1, 4516}, {299, 299, 1, 4517}, {302, 302, 1, 4517},
{303, 303, 1, 4517}, {299, 300, 1, 4518}, {302, 303, 1, 4518}, {303, 304, 1, 4518}, {299,
301, 1, 4519}, {302, 304, 1, 4519}, {303, 305, 1, 4519}, {300, 300, 1, 4520}, {303, 303,
1, 4520}, {304, 304, 1, 4520}, {300, 301, 1, 4521}, {303, 304, 1, 4521}, {304, 305, 1,
4521}, {301, 301, 1, 4522}, {304, 304, 1, 4522}, {305, 305, 1, 4522}, {296, 296, 1, 4523},
{297, 297, 2, 4523}, {298, 298, 2, 4523}, {299, 299, 1, 4523}, {300, 300, 2, 4523}, {301,
301, 1, 4523}, {296, 297, 1, 4524}, {297, 299, 2, 4524}, {298, 300, 2, 4524}, {299, 302,
1, 4524}, {300, 303, 2, 4524}, {301, 304, 1, 4524}, {296, 298, 1, 4525}, {297, 300, 2,
4525}, {298, 301, 2, 4525}, {299, 303, 1, 4525}, {300, 304, 2, 4525}, {301, 305, 1, 4525},
{297, 297, 1, 4526}, {299, 299, 2, 4526}, {300, 300, 2, 4526}, {302, 302, 1, 4526}, {303,
303, 2, 4526}, {304, 304, 1, 4526}, {297, 298, 1, 4527}, {299, 300, 2, 4527}, {300, 301,
2, 4527}, {302, 303, 1, 4527}, {303, 304, 2, 4527}, {304, 305, 1, 4527}, {298, 298, 1,
4528}, {300, 300, 2, 4528}, {301, 301, 2, 4528}, {303, 303, 1, 4528}, {304, 304, 2, 4528},
{305, 305, 1, 4528}, {296, 306, 1, 4529}, {297, 307, 2, 4529}, {298, 308, 2, 4529}, {299,
309, 1, 4529}, {300, 310, 2, 4529}, {301, 311, 1, 4529}, {296, 307, 1, 4530}, {297, 309,
2, 4530}, {298, 310, 2, 4530}, {299, 312, 1, 4530}, {300, 313, 2, 4530}, {301, 314, 1,
4530}, {296, 308, 1, 4531}, {297, 310, 2, 4531}, {298, 311, 2, 4531}, {299, 313, 1, 4531},
{300, 314, 2, 4531}, {301, 315, 1, 4531}, {296, 309, 1, 4532}, {297, 312, 2, 4532}, {298,
313, 2, 4532}, {299, 316, 1, 4532}, {300, 317, 2, 4532}, {301, 318, 1, 4532}, {296, 310,
1, 4533}, {297, 313, 2, 4533}, {298, 314, 2, 4533}, {299, 317, 1, 4533}, {300, 318, 2,
4533}, {301, 319, 1, 4533}, {296, 311, 1, 4534}, {297, 314, 2, 4534}, {298, 315, 2, 4534},
{299, 318, 1, 4534}, {300, 319, 2, 4534}, {301, 320, 1, 4534}, {297, 306, 1, 4535}, {299,
307, 2, 4535}, {300, 308, 2, 4535}, {302, 309, 1, 4535}, {303, 310, 2, 4535}, {304, 311,
1, 4535}, {297, 307, 1, 4536}, {299, 309, 2, 4536}, {300, 310, 2, 4536}, {302, 312, 1,
4536}, {303, 313, 2, 4536}, {304, 314, 1, 4536}, {297, 308, 1, 4537}, {299, 310, 2, 4537},
{300, 311, 2, 4537}, {302, 313, 1, 4537}, {303, 314, 2, 4537}, {304, 315, 1, 4537}, {297,
309, 1, 4538}, {299, 312, 2, 4538}, {300, 313, 2, 4538}, {302, 316, 1, 4538}, {303, 317,
2, 4538}, {304, 318, 1, 4538}, {297, 310, 1, 4539}, {299, 313, 2, 4539}, {300, 314, 2,
4539}, {302, 317, 1, 4539}, {303, 318, 2, 4539}, {304, 319, 1, 4539}, {297, 311, 1, 4540},
{299, 314, 2, 4540}, {300, 315, 2, 4540}, {302, 318, 1, 4540}, {303, 319, 2, 4540}, {304,
320, 1, 4540}, {298, 306, 1, 4541}, {300, 307, 2, 4541}, {301, 308, 2, 4541}, {303, 309,
1, 4541}, {304, 310, 2, 4541}, {305, 311, 1, 4541}, {298, 307, 1, 4542}, {300, 309, 2,
4542}, {301, 310, 2, 4542}, {303, 312, 1, 4542}, {304, 313, 2, 4542}, {305, 314, 1, 4542},
{298, 308, 1, 4543}, {300, 310, 2, 4543}, {301, 311, 2, 4543}, {303, 313, 1, 4543}, {304,
314, 2, 4543}, {305, 315, 1, 4543}, {298, 309, 1, 4544}, {300, 312, 2, 4544}, {301, 313,
2, 4544}, {303, 316, 1, 4544}, {304, 317, 2, 4544}, {305, 318, 1, 4544}, {298, 310, 1,
4545}, {300, 313, 2, 4545}, {301, 314, 2, 4545}, {303, 317, 1, 4545}, {304, 318, 2, 4545},
{305, 319, 1, 4545}, {298, 311, 1, 4546}, {300, 314, 2, 4546}, {301, 315, 2, 4546}, {303,
318, 1, 4546}, {304, 319, 2, 4546}, {305, 320, 1, 4546}, {296, 296, 1, 4547}, {297, 297,
3, 4547}, {298, 298, 3, 4547}, {299, 299, 3, 4547}, {300, 300, 6, 4547}, {301, 301, 3,
4547}, {302, 302, 1, 4547}, {303, 303, 3, 4547}, {304, 304, 3, 4547}, {305, 305, 1, 4547},
{296, 306, 1, 4548}, {297, 307, 3, 4548}, {298, 308, 3, 4548}, {299, 309, 3, 4548}, {300,
310, 6, 4548}, {301, 311, 3, 4548}, {302, 312, 1, 4548}, {303, 313, 3, 4548}, {304, 314,
3, 4548}, {305, 315, 1, 4548}, {296, 307, 1, 4549}, {297, 309, 3, 4549}, {298, 310, 3,

4549}, {299, 312, 3, 4549}, {300, 313, 6, 4549}, {301, 314, 3, 4549}, {302, 316, 1, 4549}, {303, 317, 3, 4549}, {304, 318, 3, 4549}, {305, 319, 1, 4549}, {296, 308, 1, 4550}, {297, 310, 3, 4550}, {298, 311, 3, 4550}, {299, 313, 3, 4550}, {300, 314, 6, 4550}, {301, 315, 3, 4550}, {302, 317, 1, 4550}, {303, 318, 3, 4550}, {304, 319, 3, 4550}, {305, 320, 1, 4550}, {296, 321, 1, 4551}, {297, 322, 3, 4551}, {298, 323, 3, 4551}, {299, 324, 3, 4551}, {300, 325, 6, 4551}, {301, 326, 3, 4551}, {302, 327, 1, 4551}, {303, 328, 3, 4551}, {304, 329, 3, 4551}, {305, 330, 1, 4551}, {296, 322, 1, 4552}, {297, 324, 3, 4552}, {298, 325, 3, 4552}, {299, 327, 3, 4552}, {300, 328, 6, 4552}, {301, 329, 3, 4552}, {302, 331, 1, 4552}, {303, 332, 3, 4552}, {304, 333, 3, 4552}, {305, 334, 1, 4552}, {296, 323, 1, 4553}, {297, 325, 3, 4553}, {298, 326, 3, 4553}, {299, 328, 3, 4553}, {300, 329, 6, 4553}, {301, 330, 3, 4553}, {302, 332, 1, 4553}, {303, 333, 3, 4553}, {304, 334, 3, 4553}, {305, 335, 1, 4553}, {296, 324, 1, 4554}, {297, 327, 3, 4554}, {298, 328, 3, 4554}, {299, 331, 3, 4554}, {300, 332, 6, 4554}, {301, 333, 3, 4554}, {302, 336, 1, 4554}, {303, 337, 3, 4554}, {304, 338, 3, 4554}, {305, 339, 1, 4554}, {296, 325, 1, 4555}, {297, 328, 3, 4555}, {298, 329, 3, 4555}, {299, 332, 3, 4555}, {300, 333, 6, 4555}, {301, 334, 3, 4555}, {302, 337, 1, 4555}, {303, 338, 3, 4555}, {304, 339, 3, 4555}, {305, 340, 1, 4555}, {296, 326, 1, 4556}, {297, 329, 3, 4556}, {298, 330, 3, 4556}, {299, 333, 3, 4556}, {300, 334, 6, 4556}, {301, 335, 3, 4556}, {302, 338, 1, 4556}, {303, 339, 3, 4556}, {304, 340, 3, 4556}, {305, 341, 1, 4556}, {296, 342, 1, 4557}, {297, 343, 3, 4557}, {298, 344, 3, 4557}, {299, 345, 3, 4557}, {300, 346, 6, 4557}, {301, 347, 3, 4557}, {302, 348, 1, 4557}, {303, 349, 3, 4557}, {304, 350, 3, 4557}, {305, 351, 1, 4557}, {296, 343, 1, 4558}, {297, 345, 3, 4558}, {298, 346, 3, 4558}, {299, 348, 3, 4558}, {300, 349, 6, 4558}, {301, 350, 3, 4558}, {302, 352, 1, 4558}, {303, 353, 3, 4558}, {304, 354, 3, 4558}, {305, 355, 1, 4558}, {296, 344, 1, 4559}, {297, 346, 3, 4559}, {298, 347, 3, 4559}, {299, 349, 3, 4559}, {300, 350, 6, 4559}, {301, 351, 3, 4559}, {302, 353, 1, 4559}, {303, 354, 3, 4559}, {304, 355, 3, 4559}, {305, 356, 1, 4559}, {296, 345, 1, 4560}, {297, 348, 3, 4560}, {298, 349, 3, 4560}, {299, 352, 3, 4560}, {300, 353, 6, 4560}, {301, 354, 3, 4560}, {302, 357, 1, 4560}, {303, 358, 3, 4560}, {304, 359, 3, 4560}, {305, 360, 1, 4560}, {296, 346, 1, 4561}, {297, 349, 3, 4561}, {298, 350, 3, 4561}, {299, 353, 3, 4561}, {300, 354, 6, 4561}, {301, 355, 3, 4561}, {302, 358, 1, 4561}, {303, 359, 3, 4561}, {304, 360, 3, 4561}, {305, 361, 1, 4561}, {296, 347, 1, 4562}, {297, 350, 3, 4562}, {298, 351, 3, 4562}, {299, 354, 3, 4562}, {300, 355, 6, 4562}, {301, 356, 3, 4562}, {302, 359, 1, 4562}, {303, 360, 3, 4562}, {304, 361, 3, 4562}, {305, 362, 1, 4562}, {296, 348, 1, 4563}, {297, 352, 3, 4563}, {298, 353, 3, 4563}, {299, 357, 3, 4563}, {300, 358, 6, 4563}, {301, 359, 3, 4563}, {302, 363, 1, 4563}, {303, 364, 3, 4563}, {304, 365, 3, 4563}, {305, 366, 1, 4563}, {296, 349, 1, 4564}, {297, 353, 3, 4564}, {298, 354, 3, 4564}, {299, 358, 3, 4564}, {300, 359, 6, 4564}, {301, 360, 3, 4564}, {302, 364, 1, 4564}, {303, 365, 3, 4564}, {304, 366, 3, 4564}, {305, 367, 1, 4564}, {296, 350, 1, 4565}, {297, 354, 3, 4565}, {298, 355, 3, 4565}, {299, 359, 3, 4565}, {300, 360, 6, 4565}, {301, 361, 3, 4565}, {302, 365, 1, 4565}, {303, 366, 3, 4565}, {304, 367, 3, 4565}, {305, 368, 1, 4565}, {296, 351, 1, 4566}, {297, 355, 3, 4566}, {298, 356, 3, 4566}, {299, 360, 3, 4566}, {300, 361, 6, 4566}, {301, 362, 3, 4566}, {302, 366, 1, 4566}, {303, 367, 3, 4566}, {304, 368, 3, 4566}, {305, 369, 1, 4566}, {306, 306, 1, 4567}, {307, 307, 2, 4567}, {308, 308, 2, 4567}, {309, 309, 1, 4567}, {310, 310, 2, 4567}, {311, 311, 1, 4567}, {306, 307, 1, 4568}, {307, 309, 2, 4568}, {308, 310, 2, 4568}, {309, 312, 1, 4568}, {310, 313, 2, 4568}, {311, 314, 1, 4568}, {306, 308, 1, 4569}, {307, 310, 2, 4569}, {308, 311, 2, 4569}, {309, 313, 1, 4569}, {310, 314, 2, 4569}, {311, 315, 1, 4569}, {306, 309, 1, 4570}, {307, 312, 2, 4570}, {308, 313, 2, 4570}, {309, 316, 1, 4570}, {310, 317, 2, 4570}, {311, 318, 1, 4570}, {306, 310, 1, 4571}, {307, 313, 2, 4571}, {308, 314, 2, 4571}, {309, 317, 1, 4571}, {310, 318, 2, 4571}, {311, 319, 1, 4571}, {306, 311, 1, 4572}, {307, 314, 2, 4572}, {308, 315, 2, 4572}, {309, 318, 1, 4572}, {310, 319, 2, 4572}, {311, 320, 1, 4572}, {307, 307, 1, 4573}, {309, 309, 2, 4573}, {310, 310, 2, 4573}, {312, 312, 1, 4573}, {313, 313, 2, 4573}, {314, 314, 1, 4573}, {307, 308, 1, 4574}, {309, 310, 2, 4574}, {310, 311, 2, 4574}, {312, 313, 1, 4574}, {313, 314, 2, 4574}, {314, 315, 1, 4574}, {307, 309, 1, 4575}, {309, 312, 2, 4575}, {310, 313, 2, 4575}, {312, 316, 1, 4575}, {313, 317, 2, 4575}, {314, 318, 1, 4575}, {307, 310, 1, 4576}, {309, 313, 2, 4576}, {310, 314, 2, 4576}, {312, 317, 1, 4576}, {313, 318, 2, 4576}, {314, 319, 1, 4576}, {307, 311, 1, 4577}, {309, 314, 2, 4577}, {310, 315, 2, 4577}, {312, 318, 1, 4577}, {313, 319, 2, 4577}, {314, 320, 1, 4577}, {308, 308, 1, 4578}, {310, 310, 2, 4578}, {311, 311, 2, 4578}, {313, 313, 1, 4578}, {314, 314, 2, 4578}, {315, 315, 1, 4578}, {308, 309, 1, 4579}, {310, 312, 2, 4579}, {311, 313, 2, 4579}, {313, 316, 1, 4579}, {314, 317, 2, 4579}, {315, 318, 1, 4579}, {308, 310, 1, 4580}, {310, 313, 2, 4580}, {311, 314, 2, 4580}, {313, 317, 1, 4580}, {314, 318, 2, 4580}, {315, 319, 1, 4580}, {308, 311, 1, 4581}, {310, 314, 2, 4581}, {311, 315, 2, 4581}, {313, 318, 1, 4581}, {314, 319, 2, 4581}, {315, 320, 1, 4581}, {309, 309, 1, 4582}, {312, 312, 2, 4582}, {313, 313, 2, 4582}, {316, 316, 1, 4582}, {317, 317, 2, 4582}, {318, 318, 1, 4582}, {309, 310, 1, 4583}, {312, 313, 2, 4583}, {313, 314, 2, 4583}, {316, 317, 1, 4583}, {317, 318, 2, 4583}, {318, 319, 1, 4583}, {309, 311, 1, 4584}, {312, 314, 2, 4584}, {313, 315, 2, 4584}, {316, 318, 1, 4584}, {317, 319, 2, 4584}, {318, 320, 1, 4584}, {310, 310, 1, 4585}, {313, 313, 2, 4585}, {314, 314, 2, 4585}, {317, 317, 1, 4585}, {318, 318, 2, 4585}, {319, 319, 1, 4585}, {310, 311, 1, 4586}, {313, 314, 2, 4586},

{314, 315, 2, 4586}, {317, 318, 1, 4586}, {318, 319, 2, 4586}, {319, 320, 1, 4586}, {311, 311, 1, 4587}, {314, 314, 2, 4587}, {315, 315, 2, 4587}, {318, 318, 1, 4587}, {319, 319, 2, 4587}, {320, 320, 1, 4587}, {306, 306, 1, 4588}, {307, 307, 3, 4588}, {308, 308, 3, 4588}, {309, 309, 3, 4588}, {310, 310, 6, 4588}, {311, 311, 3, 4588}, {312, 312, 1, 4588}, {313, 313, 3, 4588}, {314, 314, 3, 4588}, {315, 315, 1, 4588}, {306, 307, 1, 4589}, {307, 309, 3, 4589}, {308, 310, 3, 4589}, {309, 312, 3, 4589}, {310, 313, 6, 4589}, {311, 314, 3, 4589}, {312, 316, 1, 4589}, {313, 317, 3, 4589}, {314, 318, 3, 4589}, {315, 319, 1, 4589}, {306, 308, 1, 4590}, {307, 310, 3, 4590}, {308, 311, 3, 4590}, {309, 313, 3, 4590}, {310, 314, 6, 4590}, {311, 315, 3, 4590}, {312, 317, 1, 4590}, {313, 318, 3, 4590}, {314, 319, 3, 4590}, {315, 320, 1, 4590}, {307, 307, 1, 4591}, {309, 309, 3, 4591}, {310, 310, 3, 4591}, {312, 312, 3, 4591}, {313, 313, 6, 4591}, {314, 314, 3, 4591}, {316, 316, 1, 4591}, {317, 317, 3, 4591}, {318, 318, 3, 4591}, {319, 319, 1, 4591}, {307, 308, 1, 4592}, {309, 310, 3, 4592}, {310, 311, 3, 4592}, {312, 313, 3, 4592}, {313, 314, 6, 4592}, {314, 315, 3, 4592}, {316, 317, 1, 4592}, {317, 318, 3, 4592}, {318, 319, 3, 4592}, {319, 320, 1, 4592}, {308, 308, 1, 4593}, {310, 310, 3, 4593}, {311, 311, 3, 4593}, {313, 313, 3, 4593}, {314, 314, 6, 4593}, {315, 315, 3, 4593}, {317, 317, 1, 4593}, {318, 318, 3, 4593}, {319, 319, 3, 4593}, {320, 320, 1, 4593}, {306, 321, 1, 4594}, {307, 322, 3, 4594}, {308, 323, 3, 4594}, {309, 324, 3, 4594}, {310, 325, 6, 4594}, {311, 326, 3, 4594}, {312, 327, 1, 4594}, {313, 328, 3, 4594}, {314, 329, 3, 4594}, {315, 330, 1, 4594}, {306, 322, 1, 4595}, {307, 324, 3, 4595}, {308, 325, 3, 4595}, {309, 327, 3, 4595}, {310, 328, 6, 4595}, {311, 329, 3, 4595}, {312, 331, 1, 4595}, {313, 332, 3, 4595}, {314, 333, 3, 4595}, {315, 334, 1, 4595}, {306, 323, 1, 4596}, {307, 325, 3, 4596}, {308, 326, 3, 4596}, {309, 328, 3, 4596}, {310, 329, 6, 4596}, {311, 330, 3, 4596}, {312, 332, 1, 4596}, {313, 333, 3, 4596}, {314, 334, 3, 4596}, {315, 335, 1, 4596}, {306, 324, 1, 4597}, {307, 327, 3, 4597}, {308, 328, 3, 4597}, {309, 331, 3, 4597}, {310, 332, 6, 4597}, {311, 333, 3, 4597}, {312, 336, 1, 4597}, {313, 337, 3, 4597}, {314, 338, 3, 4597}, {315, 339, 1, 4597}, {306, 325, 1, 4598}, {307, 328, 3, 4598}, {308, 329, 3, 4598}, {309, 332, 3, 4598}, {310, 333, 6, 4598}, {311, 334, 3, 4598}, {312, 337, 1, 4598}, {313, 338, 3, 4598}, {314, 339, 3, 4598}, {315, 340, 1, 4598}, {306, 326, 1, 4599}, {307, 329, 3, 4599}, {308, 330, 3, 4599}, {309, 333, 3, 4599}, {310, 334, 6, 4599}, {311, 335, 3, 4599}, {312, 338, 1, 4599}, {313, 339, 3, 4599}, {314, 340, 3, 4599}, {315, 341, 1, 4599}, {307, 321, 1, 4600}, {309, 322, 3, 4600}, {310, 323, 3, 4600}, {312, 324, 3, 4600}, {313, 325, 6, 4600}, {314, 326, 3, 4600}, {316, 327, 1, 4600}, {317, 328, 3, 4600}, {318, 329, 3, 4600}, {319, 330, 1, 4600}, {307, 322, 1, 4601}, {309, 324, 3, 4601}, {310, 325, 3, 4601}, {312, 327, 3, 4601}, {313, 328, 6, 4601}, {314, 329, 3, 4601}, {316, 331, 1, 4601}, {317, 332, 3, 4601}, {318, 333, 3, 4601}, {319, 334, 1, 4601}, {307, 323, 1, 4602}, {309, 325, 3, 4602}, {310, 326, 3, 4602}, {312, 328, 3, 4602}, {313, 329, 6, 4602}, {314, 330, 3, 4602}, {316, 332, 1, 4602}, {317, 333, 3, 4602}, {318, 334, 3, 4602}, {319, 335, 1, 4602}, {307, 324, 1, 4603}, {309, 327, 3, 4603}, {310, 328, 3, 4603}, {312, 331, 3, 4603}, {313, 332, 6, 4603}, {314, 333, 3, 4603}, {316, 336, 1, 4603}, {317, 337, 3, 4603}, {318, 338, 3, 4603}, {319, 339, 1, 4603}, {307, 325, 1, 4604}, {309, 328, 3, 4604}, {310, 329, 3, 4604}, {312, 332, 3, 4604}, {313, 333, 6, 4604}, {314, 334, 3, 4604}, {316, 337, 1, 4604}, {317, 338, 3, 4604}, {318, 339, 3, 4604}, {319, 340, 1, 4604}, {307, 326, 1, 4605}, {309, 329, 3, 4605}, {310, 330, 3, 4605}, {312, 333, 3, 4605}, {313, 334, 6, 4605}, {314, 335, 3, 4605}, {316, 338, 1, 4605}, {317, 339, 3, 4605}, {318, 340, 3, 4605}, {319, 341, 1, 4605}, {308, 321, 1, 4606}, {310, 322, 3, 4606}, {311, 323, 3, 4606}, {313, 324, 3, 4606}, {314, 325, 6, 4606}, {315, 326, 3, 4606}, {317, 327, 1, 4606}, {318, 328, 3, 4606}, {319, 329, 3, 4606}, {320, 330, 1, 4606}, {308, 322, 1, 4607}, {310, 324, 3, 4607}, {311, 325, 3, 4607}, {313, 327, 3, 4607}, {314, 328, 6, 4607}, {315, 329, 3, 4607}, {317, 331, 1, 4607}, {318, 332, 3, 4607}, {319, 333, 3, 4607}, {320, 334, 1, 4607}, {308, 323, 1, 4608}, {310, 325, 3, 4608}, {311, 326, 3, 4608}, {313, 328, 3, 4608}, {314, 329, 6, 4608}, {315, 330, 3, 4608}, {317, 332, 1, 4608}, {318, 333, 3, 4608}, {319, 334, 3, 4608}, {320, 335, 1, 4608}, {308, 324, 1, 4609}, {310, 327, 3, 4609}, {311, 328, 3, 4609}, {313, 331, 3, 4609}, {314, 332, 6, 4609}, {315, 333, 3, 4609}, {317, 336, 1, 4609}, {318, 337, 3, 4609}, {319, 338, 3, 4609}, {320, 339, 1, 4609}, {308, 325, 1, 4610}, {310, 328, 3, 4610}, {311, 329, 3, 4610}, {313, 332, 3, 4610}, {314, 333, 6, 4610}, {315, 334, 3, 4610}, {317, 337, 1, 4610}, {318, 338, 3, 4610}, {319, 339, 3, 4610}, {320, 340, 1, 4610}, {308, 326, 1, 4611}, {310, 329, 3, 4611}, {311, 330, 3, 4611}, {313, 333, 3, 4611}, {314, 334, 6, 4611}, {315, 335, 3, 4611}, {317, 338, 1, 4611}, {318, 339, 3, 4611}, {319, 340, 3, 4611}, {320, 341, 1, 4611}, {306, 306, 1, 4612}, {307, 307, 4, 4612}, {308, 308, 4, 4612}, {309, 309, 6, 4612}, {310, 310, 12, 4612}, {311, 311, 6, 4612}, {312, 312, 4, 4612}, {313, 313, 12, 4612}, {314, 314, 12, 4612}, {315, 315, 4, 4612}, {316, 316, 1, 4612}, {317, 317, 4, 4612}, {318, 318, 6, 4612}, {319, 319, 4, 4612}, {320, 320, 1, 4612}, {306, 321, 1, 4613}, {307, 322, 4, 4613}, {308, 323, 4, 4613}, {309, 324, 6, 4613}, {310, 325, 12, 4613}, {311, 326, 6, 4613}, {312, 327, 4, 4613}, {313, 328, 12, 4613}, {314, 329, 12, 4613}, {315, 330, 4, 4613}, {316, 331, 1, 4613}, {317, 332, 4, 4613}, {318, 333, 6, 4613}, {319, 334, 4, 4613}, {320, 335, 1, 4613}, {306, 322, 1, 4614}, {307, 324, 4, 4614}, {308, 325, 4, 4614}, {309, 327, 6, 4614}, {310, 328, 12, 4614}, {311, 329, 6, 4614}, {312, 331, 4, 4614}, {313, 332, 12, 4614}, {314, 333, 12, 4614}, {315, 334, 4, 4614}, {316, 336, 1, 4614}, {317, 337, 4, 4614}, {318, 338, 6,

4614}, {319, 339, 4, 4614}, {320, 340, 1, 4614}, {306, 323, 1, 4615}, {307, 325, 4, 4615}, {308, 326, 4, 4615}, {309, 328, 6, 4615}, {310, 329, 12, 4615}, {311, 330, 6, 4615}, {312, 332, 4, 4615}, {313, 333, 12, 4615}, {314, 334, 12, 4615}, {315, 335, 4, 4615}, {316, 337, 1, 4615}, {317, 338, 4, 4615}, {318, 339, 6, 4615}, {319, 340, 4, 4615}, {320, 341, 1, 4615}, {306, 342, 1, 4616}, {307, 343, 4, 4616}, {308, 344, 4, 4616}, {309, 345, 6, 4616}, {310, 346, 12, 4616}, {311, 347, 6, 4616}, {312, 348, 4, 4616}, {313, 349, 12, 4616}, {314, 350, 12, 4616}, {315, 351, 4, 4616}, {316, 352, 1, 4616}, {317, 353, 4, 4616}, {318, 354, 6, 4616}, {319, 355, 4, 4616}, {320, 356, 1, 4616}, {306, 343, 1, 4617}, {307, 345, 4, 4617}, {308, 346, 4, 4617}, {309, 348, 6, 4617}, {310, 349, 12, 4617}, {311, 350, 6, 4617}, {312, 352, 4, 4617}, {313, 353, 12, 4617}, {314, 354, 12, 4617}, {315, 355, 4, 4617}, {316, 357, 1, 4617}, {317, 358, 4, 4617}, {318, 359, 6, 4617}, {319, 360, 4, 4617}, {320, 361, 1, 4617}, {306, 344, 1, 4618}, {307, 346, 4, 4618}, {308, 347, 4, 4618}, {309, 349, 6, 4618}, {310, 350, 12, 4618}, {311, 351, 6, 4618}, {312, 353, 4, 4618}, {313, 354, 12, 4618}, {314, 355, 12, 4618}, {315, 356, 4, 4618}, {316, 358, 1, 4618}, {317, 359, 4, 4618}, {318, 360, 6, 4618}, {319, 361, 4, 4618}, {320, 362, 1, 4618}, {306, 345, 1, 4619}, {307, 348, 4, 4619}, {308, 349, 4, 4619}, {309, 352, 6, 4619}, {310, 353, 12, 4619}, {311, 354, 6, 4619}, {312, 357, 4, 4619}, {313, 358, 12, 4619}, {314, 359, 12, 4619}, {315, 360, 4, 4619}, {316, 363, 1, 4619}, {317, 364, 4, 4619}, {318, 365, 6, 4619}, {319, 366, 4, 4619}, {320, 367, 1, 4619}, {306, 346, 1, 4620}, {307, 349, 4, 4620}, {308, 350, 4, 4620}, {309, 353, 6, 4620}, {310, 354, 12, 4620}, {311, 355, 6, 4620}, {312, 358, 4, 4620}, {313, 359, 12, 4620}, {314, 360, 12, 4620}, {315, 361, 4, 4620}, {316, 364, 1, 4620}, {317, 365, 4, 4620}, {318, 366, 6, 4620}, {319, 367, 4, 4620}, {320, 368, 1, 4620}, {306, 347, 1, 4621}, {307, 350, 4, 4621}, {308, 351, 4, 4621}, {309, 354, 6, 4621}, {310, 355, 12, 4621}, {311, 356, 6, 4621}, {312, 359, 4, 4621}, {313, 360, 12, 4621}, {314, 361, 12, 4621}, {315, 362, 4, 4621}, {316, 365, 1, 4621}, {317, 366, 4, 4621}, {318, 367, 6, 4621}, {319, 368, 4, 4621}, {320, 369, 1, 4621}, {321, 321, 1, 4622}, {322, 322, 4, 4622}, {323, 323, 4, 4622}, {324, 324, 6, 4622}, {325, 325, 12, 4622}, {326, 326, 6, 4622}, {327, 327, 4, 4622}, {328, 328, 12, 4622}, {329, 329, 12, 4622}, {330, 330, 4, 4622}, {331, 331, 1, 4622}, {332, 332, 4, 4622}, {333, 333, 6, 4622}, {334, 334, 4, 4622}, {335, 335, 1, 4622}, {321, 322, 1, 4623}, {322, 324, 4, 4623}, {323, 325, 4, 4623}, {324, 327, 6, 4623}, {325, 328, 12, 4623}, {326, 329, 6, 4623}, {327, 331, 4, 4623}, {328, 332, 12, 4623}, {329, 333, 12, 4623}, {330, 334, 4, 4623}, {331, 336, 1, 4623}, {332, 337, 4, 4623}, {333, 338, 6, 4623}, {334, 339, 4, 4623}, {335, 340, 1, 4623}, {321, 323, 1, 4624}, {322, 325, 4, 4624}, {323, 326, 4, 4624}, {324, 328, 6, 4624}, {325, 329, 12, 4624}, {326, 330, 6, 4624}, {327, 332, 4, 4624}, {328, 333, 12, 4624}, {329, 334, 12, 4624}, {330, 335, 4, 4624}, {331, 337, 1, 4624}, {332, 338, 4, 4624}, {333, 339, 6, 4624}, {334, 340, 4, 4624}, {335, 341, 1, 4624}, {322, 322, 1, 4625}, {324, 324, 4, 4625}, {325, 325, 4, 4625}, {327, 327, 6, 4625}, {328, 328, 12, 4625}, {329, 329, 6, 4625}, {331, 331, 4, 4625}, {332, 332, 12, 4625}, {333, 333, 12, 4625}, {334, 334, 4, 4625}, {336, 336, 1, 4625}, {337, 337, 4, 4625}, {338, 338, 6, 4625}, {339, 339, 4, 4625}, {340, 340, 1, 4625}, {322, 323, 1, 4626}, {324, 325, 4, 4626}, {325, 326, 4, 4626}, {327, 328, 6, 4626}, {328, 329, 12, 4626}, {329, 330, 6, 4626}, {331, 332, 4, 4626}, {332, 333, 12, 4626}, {333, 334, 12, 4626}, {334, 335, 4, 4626}, {336, 337, 1, 4626}, {337, 338, 4, 4626}, {338, 339, 6, 4626}, {339, 340, 4, 4626}, {340, 341, 1, 4626}, {323, 323, 1, 4627}, {325, 325, 4, 4627}, {326, 326, 4, 4627}, {328, 328, 6, 4627}, {329, 329, 12, 4627}, {330, 330, 6, 4627}, {332, 332, 4, 4627}, {333, 333, 12, 4627}, {334, 334, 12, 4627}, {335, 335, 4, 4627}, {337, 337, 1, 4627}, {338, 338, 4, 4627}, {339, 339, 6, 4627}, {340, 340, 4, 4627}, {341, 341, 1, 4627}, {321, 321, 1, 4628}, {322, 322, 5, 4628}, {323, 323, 5, 4628}, {324, 324, 10, 4628}, {325, 325, 20, 4628}, {326, 326, 10, 4628}, {327, 327, 10, 4628}, {328, 328, 30, 4628}, {329, 329, 30, 4628}, {330, 330, 10, 4628}, {331, 331, 5, 4628}, {332, 332, 20, 4628}, {333, 333, 30, 4628}, {334, 334, 20, 4628}, {335, 335, 5, 4628}, {336, 336, 1, 4628}, {337, 337, 5, 4628}, {338, 338, 10, 4628}, {339, 339, 10, 4628}, {340, 340, 5, 4628}, {341, 341, 1, 4628}, {321, 342, 1, 4629}, {322, 343, 5, 4629}, {323, 344, 5, 4629}, {324, 345, 10, 4629}, {325, 346, 20, 4629}, {326, 347, 10, 4629}, {327, 348, 10, 4629}, {328, 349, 30, 4629}, {329, 350, 30, 4629}, {330, 351, 10, 4629}, {331, 352, 5, 4629}, {332, 353, 20, 4629}, {333, 354, 30, 4629}, {334, 355, 20, 4629}, {335, 356, 5, 4629}, {336, 357, 1, 4629}, {337, 358, 5, 4629}, {338, 359, 10, 4629}, {339, 360, 10, 4629}, {340, 361, 5, 4629}, {341, 362, 1, 4629}, {321, 343, 1, 4630}, {322, 345, 5, 4630}, {323, 346, 5, 4630}, {324, 348, 10, 4630}, {325, 349, 20, 4630}, {326, 350, 10, 4630}, {327, 352, 10, 4630}, {328, 353, 30, 4630}, {329, 354, 30, 4630}, {330, 355, 10, 4630}, {331, 357, 5, 4630}, {332, 358, 20, 4630}, {333, 359, 30, 4630}, {334, 360, 20, 4630}, {335, 361, 5, 4630}, {336, 363, 1, 4630}, {337, 364, 5, 4630}, {338, 365, 10, 4630}, {339, 366, 10, 4630}, {340, 367, 5, 4630}, {341, 368, 1, 4630}, {321, 344, 1, 4631}, {322, 346, 5, 4631}, {323, 347, 5, 4631}, {324, 349, 10, 4631}, {325, 350, 20, 4631}, {326, 351, 10, 4631}, {327, 353, 10, 4631}, {328, 354, 30, 4631}, {329, 355, 30, 4631}, {330, 356, 10, 4631}, {331, 358, 5, 4631}, {332, 359, 20, 4631}, {333, 360, 30, 4631}, {334, 361, 20, 4631}, {335, 362, 5, 4631}, {336, 364, 1, 4631}, {337, 365, 5, 4631}, {338, 366, 10, 4631}, {339, 367, 10, 4631}, {340, 368, 5, 4631}, {341, 369, 1, 4631}, {342, 342, 1, 4632}, {343, 343, 6, 4632}, {344, 344, 6, 4632}, {345, 345, 15, 4632}, {346, 346, 30, 4632}, {347, 347, 15, 4632}, {348,

348, 20, 4632}, {349, 349, 60, 4632}, {350, 350, 60, 4632}, {351, 351, 20, 4632}, {352, 352, 15, 4632}, {353, 353, 60, 4632}, {354, 354, 90, 4632}, {355, 355, 60, 4632}, {356, 356, 15, 4632}, {357, 357, 6, 4632}, {358, 358, 30, 4632}, {359, 359, 60, 4632}, {360, 360, 60, 4632}, {361, 361, 30, 4632}, {362, 362, 6, 4632}, {363, 363, 1, 4632}, {364, 364, 6, 4632}, {365, 365, 15, 4632}, {366, 366, 20, 4632}, {367, 367, 15, 4632}, {368, 368, 6, 4632}, {369, 369, 1, 4632}, {370, 370, 1, 4633}, {371, 371, 7, 4633}, {372, 372, 7, 4633}, {373, 373, 21, 4633}, {374, 374, 42, 4633}, {375, 375, 21, 4633}, {376, 376, 35, 4633}, {377, 377, 105, 4633}, {378, 378, 105, 4633}, {379, 379, 35, 4633}, {380, 380, 35, 4633}, {381, 381, 140, 4633}, {382, 382, 210, 4633}, {383, 383, 140, 4633}, {384, 384, 35, 4633}, {385, 385, 21, 4633}, {386, 386, 105, 4633}, {387, 387, 210, 4633}, {388, 388, 210, 4633}, {389, 389, 105, 4633}, {390, 390, 21, 4633}, {391, 391, 7, 4633}, {392, 392, 42, 4633}, {393, 393, 105, 4633}, {394, 394, 140, 4633}, {395, 395, 105, 4633}, {396, 396, 42, 4633}, {397, 397, 7, 4633}, {398, 398, 1, 4633}, {399, 399, 7, 4633}, {400, 400, 21, 4633}, {401, 401, 35, 4633}, {402, 402, 35, 4633}, {403, 403, 21, 4633}, {404, 404, 7, 4633}, {405, 405, 1, 4633}, {286, 506, 1, 4634}, {287, 507, 1, 4635}, {288, 508, 1, 4635}, {289, 509, 1, 4635}, {287, 510, 1, 4636}, {288, 511, 1, 4636}, {289, 512, 1, 4636}, {287, 511, 1, 4637}, {288, 513, 1, 4637}, {289, 514, 1, 4637}, {287, 512, 1, 4638}, {288, 514, 1, 4638}, {289, 515, 1, 4638}, {287, 516, 1, 4639}, {288, 517, 1, 4639}, {289, 518, 1, 4639}, {287, 517, 1, 4640}, {288, 519, 1, 4640}, {289, 520, 1, 4640}, {287, 518, 1, 4641}, {288, 520, 1, 4641}, {289, 521, 1, 4641}, {287, 519, 1, 4642}, {288, 522, 1, 4642}, {289, 523, 1, 4642}, {287, 520, 1, 4643}, {288, 523, 1, 4643}, {289, 524, 1, 4643}, {287, 521, 1, 4644}, {288, 524, 1, 4644}, {289, 525, 1, 4644}, {290, 507, 1, 4645}, {291, 508, 1, 4645}, {292, 509, 1, 4645}, {291, 507, 1, 4646}, {293, 508, 1, 4646}, {294, 509, 1, 4646}, {292, 507, 1, 4647}, {294, 508, 1, 4647}, {295, 509, 1, 4647}, {290, 510, 1, 4648}, {291, 511, 1, 4648}, {292, 512, 1, 4648}, {290, 511, 1, 4649}, {291, 513, 1, 4649}, {292, 514, 1, 4649}, {290, 512, 1, 4650}, {291, 514, 1, 4650}, {292, 515, 1, 4650}, {291, 510, 1, 4651}, {293, 511, 1, 4651}, {294, 512, 1, 4651}, {291, 511, 1, 4652}, {293, 513, 1, 4652}, {294, 514, 1, 4652}, {291, 512, 1, 4653}, {293, 514, 1, 4653}, {294, 515, 1, 4653}, {292, 510, 1, 4654}, {294, 511, 1, 4654}, {295, 512, 1, 4654}, {292, 511, 1, 4655}, {294, 513, 1, 4655}, {295, 514, 1, 4655}, {292, 512, 1, 4656}, {294, 514, 1, 4656}, {295, 515, 1, 4656}, {290, 510, 1, 4657}, {291, 511, 2, 4657}, {292, 512, 2, 4657}, {293, 513, 1, 4657}, {294, 514, 2, 4657}, {295, 515, 1, 4657}, {290, 516, 1, 4658}, {291, 517, 2, 4658}, {292, 518, 2, 4658}, {293, 519, 1, 4658}, {294, 520, 2, 4658}, {295, 521, 1, 4658}, {290, 517, 1, 4659}, {291, 519, 2, 4659}, {292, 520, 2, 4659}, {293, 522, 1, 4659}, {294, 523, 2, 4659}, {295, 524, 1, 4659}, {290, 518, 1, 4660}, {291, 520, 2, 4660}, {292, 521, 2, 4660}, {293, 523, 1, 4660}, {294, 524, 2, 4660}, {295, 525, 1, 4660}, {290, 526, 1, 4661}, {291, 527, 2, 4661}, {292, 528, 2, 4661}, {293, 529, 1, 4661}, {294, 530, 2, 4661}, {295, 531, 1, 4661}, {290, 527, 1, 4662}, {291, 529, 2, 4662}, {292, 530, 2, 4662}, {293, 532, 1, 4662}, {294, 533, 2, 4662}, {295, 534, 1, 4662}, {290, 528, 1, 4663}, {291, 530, 2, 4663}, {292, 531, 2, 4663}, {293, 533, 1, 4663}, {294, 534, 2, 4663}, {295, 535, 1, 4663}, {290, 529, 1, 4664}, {291, 532, 2, 4664}, {292, 533, 2, 4664}, {293, 536, 1, 4664}, {294, 537, 2, 4664}, {295, 538, 1, 4664}, {290, 530, 1, 4665}, {291, 533, 2, 4665}, {292, 534, 2, 4665}, {293, 537, 1, 4665}, {294, 538, 2, 4665}, {295, 539, 1, 4665}, {290, 531, 1, 4666}, {291, 534, 2, 4666}, {292, 535, 2, 4666}, {293, 538, 1, 4666}, {294, 539, 2, 4666}, {295, 540, 1, 4666}, {296, 507, 1, 4667}, {297, 508, 1, 4667}, {298, 509, 1, 4667}, {297, 507, 1, 4668}, {299, 508, 1, 4668}, {300, 509, 1, 4668}, {298, 507, 1, 4669}, {300, 508, 1, 4669}, {301, 509, 1, 4669}, {299, 507, 1, 4670}, {302, 508, 1, 4670}, {303, 509, 1, 4670}, {300, 507, 1, 4671}, {303, 508, 1, 4671}, {304, 509, 1, 4671}, {301, 507, 1, 4672}, {304, 508, 1, 4672}, {305, 509, 1, 4672}, {296, 510, 1, 4673}, {297, 511, 2, 4673}, {298, 512, 2, 4673}, {299, 513, 1, 4673}, {300, 514, 2, 4673}, {301, 515, 1, 4673}, {297, 510, 1, 4674}, {299, 511, 2, 4674}, {300, 512, 2, 4674}, {302, 513, 1, 4674}, {303, 514, 2, 4674}, {304, 515, 1, 4674}, {298, 510, 1, 4675}, {300, 511, 2, 4675}, {301, 512, 2, 4675}, {303, 513, 1, 4675}, {304, 514, 2, 4675}, {305, 515, 1, 4675}, {296, 516, 1, 4676}, {297, 517, 2, 4676}, {298, 518, 2, 4676}, {299, 519, 1, 4676}, {300, 520, 2, 4676}, {301, 521, 1, 4676}, {296, 517, 1, 4677}, {297, 519, 2, 4677}, {298, 520, 2, 4677}, {299, 522, 1, 4677}, {300, 523, 2, 4677}, {301, 524, 1, 4677}, {296, 518, 1, 4678}, {297, 520, 2, 4678}, {298, 521, 2, 4678}, {299, 523, 1, 4678}, {300, 524, 2, 4678}, {301, 525, 1, 4678}, {297, 516, 1, 4679}, {299, 517, 2, 4679}, {300, 518, 2, 4679}, {302, 519, 1, 4679}, {303, 520, 2, 4679}, {304, 521, 1, 4679}, {297, 517, 1, 4680}, {299, 519, 2, 4680}, {300, 520, 2, 4680}, {302, 522, 1, 4680}, {303, 523, 2, 4680}, {304, 524, 1, 4680}, {297, 518, 1, 4681}, {299, 520, 2, 4681}, {300, 521, 2, 4681}, {302, 523, 1, 4681}, {303, 524, 2, 4681}, {304, 525, 1, 4681}, {298, 516, 1, 4682}, {300, 517, 2, 4682}, {301, 518, 2, 4682}, {303, 519, 1, 4682}, {304, 520, 2, 4682}, {305, 521, 1, 4682}, {298, 517, 1, 4683}, {300, 519, 2, 4683}, {301, 520, 2, 4683}, {303, 522, 1, 4683}, {304, 523, 2, 4683}, {305, 524, 1, 4683}, {298, 518, 1, 4684}, {300, 520, 2, 4684}, {301, 521, 2, 4684}, {303, 523, 1, 4684}, {304, 524, 2, 4684}, {305, 525, 1, 4684}, {296, 516, 1, 4685}, {297, 517, 3, 4685}, {298, 518, 3, 4685}, {299, 519, 3, 4685}, {300, 520, 6, 4685}, {301, 521, 3, 4685}, {302, 522, 1, 4685}, {303, 523, 3, 4685}, {304, 524, 3, 4685}, {305, 525, 1, 4685}, {296, 526, 1, 4686}, {297, 527, 3, 4686}, {298, 528, 3, 4686}, {299, 529, 3, 4686}, {300, 530, 6, 4686}, {301, 531, 3, 4686},

{302, 532, 1, 4686}, {303, 533, 3, 4686}, {304, 534, 3, 4686}, {305, 535, 1, 4686}, {296,
527, 1, 4687}, {297, 529, 3, 4687}, {298, 530, 3, 4687}, {299, 532, 3, 4687}, {300, 533,
6, 4687}, {301, 534, 3, 4687}, {302, 536, 1, 4687}, {303, 537, 3, 4687}, {304, 538, 3,
4687}, {305, 539, 1, 4687}, {296, 528, 1, 4688}, {297, 530, 3, 4688}, {298, 531, 3, 4688},
{299, 533, 3, 4688}, {300, 534, 6, 4688}, {301, 535, 3, 4688}, {302, 537, 1, 4688}, {303,
538, 3, 4688}, {304, 539, 3, 4688}, {305, 540, 1, 4688}, {306, 510, 1, 4689}, {307, 511,
2, 4689}, {308, 512, 2, 4689}, {309, 513, 1, 4689}, {310, 514, 2, 4689}, {311, 515, 1,
4689}, {307, 510, 1, 4690}, {309, 511, 2, 4690}, {310, 512, 2, 4690}, {312, 513, 1, 4690},
{313, 514, 2, 4690}, {314, 515, 1, 4690}, {308, 510, 1, 4691}, {310, 511, 2, 4691}, {311,
512, 2, 4691}, {313, 513, 1, 4691}, {314, 514, 2, 4691}, {315, 515, 1, 4691}, {309, 510,
1, 4692}, {312, 511, 2, 4692}, {313, 512, 2, 4692}, {316, 513, 1, 4692}, {317, 514, 2,
4692}, {318, 515, 1, 4692}, {310, 510, 1, 4693}, {313, 511, 2, 4693}, {314, 512, 2, 4693},
{317, 513, 1, 4693}, {318, 514, 2, 4693}, {319, 515, 1, 4693}, {311, 510, 1, 4694}, {314,
511, 2, 4694}, {315, 512, 2, 4694}, {318, 513, 1, 4694}, {319, 514, 2, 4694}, {320, 515,
1, 4694}, {306, 516, 1, 4695}, {307, 517, 3, 4695}, {308, 518, 3, 4695}, {309, 519, 3,
4695}, {310, 520, 6, 4695}, {311, 521, 3, 4695}, {312, 522, 1, 4695}, {313, 523, 3, 4695},
{314, 524, 3, 4695}, {315, 525, 1, 4695}, {307, 516, 1, 4696}, {309, 517, 3, 4696}, {310,
518, 3, 4696}, {312, 519, 3, 4696}, {313, 520, 6, 4696}, {314, 521, 3, 4696}, {316, 522,
1, 4696}, {317, 523, 3, 4696}, {318, 524, 3, 4696}, {319, 525, 1, 4696}, {308, 516, 1,
4697}, {310, 517, 3, 4697}, {311, 518, 3, 4697}, {313, 519, 3, 4697}, {314, 520, 6, 4697},
{315, 521, 3, 4697}, {317, 522, 1, 4697}, {318, 523, 3, 4697}, {319, 524, 3, 4697}, {320,
525, 1, 4697}, {306, 526, 1, 4698}, {307, 527, 4, 4698}, {308, 528, 4, 4698}, {309, 529,
6, 4698}, {310, 530, 12, 4698}, {311, 531, 6, 4698}, {312, 532, 4, 4698}, {313, 533, 12,
4698}, {314, 534, 12, 4698}, {315, 535, 4, 4698}, {316, 536, 1, 4698}, {317, 537, 4,
4698}, {318, 538, 6, 4698}, {319, 539, 4, 4698}, {320, 540, 1, 4698}, {321, 541, 1, 4699},
{322, 542, 5, 4699}, {323, 543, 5, 4699}, {324, 544, 10, 4699}, {325, 545, 20, 4699},
{326, 546, 10, 4699}, {327, 547, 10, 4699}, {328, 548, 30, 4699}, {329, 549, 30, 4699},
{330, 550, 10, 4699}, {331, 551, 5, 4699}, {332, 552, 20, 4699}, {333, 553, 30, 4699},
{334, 554, 20, 4699}, {335, 555, 5, 4699}, {336, 556, 1, 4699}, {337, 557, 5, 4699}, {338,
558, 10, 4699}, {339, 559, 10, 4699}, {340, 560, 5, 4699}, {341, 561, 1, 4699}, {286, 626,
1, 4700}, {287, 627, 1, 4701}, {288, 628, 1, 4701}, {289, 629, 1, 4701}, {287, 630, 1,
4702}, {288, 631, 1, 4702}, {289, 632, 1, 4702}, {287, 631, 1, 4703}, {288, 633, 1, 4703},
{289, 634, 1, 4703}, {287, 632, 1, 4704}, {288, 634, 1, 4704}, {289, 635, 1, 4704}, {290,
627, 1, 4705}, {291, 628, 1, 4705}, {292, 629, 1, 4705}, {291, 627, 1, 4706}, {293, 628,
1, 4706}, {294, 629, 1, 4706}, {292, 627, 1, 4707}, {294, 628, 1, 4707}, {295, 629, 1,
4707}, {290, 630, 1, 4708}, {291, 631, 2, 4708}, {292, 632, 2, 4708}, {293, 633, 1, 4708},
{294, 634, 2, 4708}, {295, 635, 1, 4708}, {296, 636, 1, 4709}, {297, 637, 3, 4709}, {298,
638, 3, 4709}, {299, 639, 3, 4709}, {300, 640, 6, 4709}, {301, 641, 3, 4709}, {302, 642,
1, 4709}, {303, 643, 3, 4709}, {304, 644, 3, 4709}, {305, 645, 1, 4709}, {286, 682, 1,
4710}, {287, 683, 1, 4711}, {288, 684, 1, 4711}, {289, 685, 1, 4711}, {506, 506, 1, 4712},
{507, 507, 1, 4713}, {508, 508, 1, 4713}, {509, 509, 1, 4713}, {507, 510, 1, 4714}, {508,
511, 1, 4714}, {509, 512, 1, 4714}, {507, 511, 1, 4715}, {508, 513, 1, 4715}, {509, 514,
1, 4715}, {507, 512, 1, 4716}, {508, 514, 1, 4716}, {509, 515, 1, 4716}, {510, 510, 1,
4717}, {511, 511, 2, 4717}, {512, 512, 2, 4717}, {513, 513, 1, 4717}, {514, 514, 2, 4717},
{515, 515, 1, 4717}, {516, 516, 1, 4718}, {517, 517, 3, 4718}, {518, 518, 3, 4718}, {519,
519, 3, 4718}, {520, 520, 6, 4718}, {521, 521, 3, 4718}, {522, 522, 1, 4718}, {523, 523,
3, 4718}, {524, 524, 3, 4718}, {525, 525, 1, 4718}, {506, 626, 1, 4719}, {507, 627, 1,
4720}, {508, 628, 1, 4720}, {509, 629, 1, 4720}, {0, 706, 1, 4721}, {0, 707, 1, 4722}, {0,
771, 1, 4723}, {0, 989, 1, 4724}, {0, 1532, 1, 4725}, {0, 2063, 1, 4726}, {0, 2444, 1,
4727}, {0, 2669, 1, 4728}, {0, 2734, 1, 4729}, {0, 2750, 1, 4730}, {0, 2754, 1, 4731}, {1,
708, 1, 4732}, {2, 709, 1, 4732}, {3, 710, 1, 4732}, {1, 711, 1, 4733}, {2, 712, 1, 4733},
{3, 713, 1, 4733}, {1, 712, 1, 4734}, {2, 714, 1, 4734}, {3, 715, 1, 4734}, {1, 713, 1,
4735}, {2, 715, 1, 4735}, {3, 716, 1, 4735}, {1, 717, 1, 4736}, {2, 718, 1, 4736}, {3,
719, 1, 4736}, {1, 718, 1, 4737}, {2, 720, 1, 4737}, {3, 721, 1, 4737}, {1, 719, 1, 4738},
{2, 721, 1, 4738}, {3, 722, 1, 4738}, {1, 772, 1, 4739}, {2, 773, 1, 4739}, {3, 774, 1,
4739}, {1, 775, 1, 4740}, {2, 776, 1, 4740}, {3, 777, 1, 4740}, {1, 776, 1, 4741}, {2,
778, 1, 4741}, {3, 779, 1, 4741}, {1, 777, 1, 4742}, {2, 779, 1, 4742}, {3, 780, 1, 4742},
{1, 827, 1, 4743}, {2, 828, 1, 4743}, {3, 829, 1, 4743}, {1, 828, 1, 4744}, {2, 830, 1,
4744}, {3, 831, 1, 4744}, {1, 829, 1, 4745}, {2, 831, 1, 4745}, {3, 832, 1, 4745}, {1,
990, 1, 4746}, {2, 991, 1, 4746}, {3, 992, 1, 4746}, {1, 993, 1, 4747}, {2, 994, 1, 4747},
{3, 995, 1, 4747}, {1, 994, 1, 4748}, {2, 996, 1, 4748}, {3, 997, 1, 4748}, {1, 995, 1,
4749}, {2, 997, 1, 4749}, {3, 998, 1, 4749}, {1, 1370, 1, 4750}, {2, 1371, 1, 4750}, {3,
1372, 1, 4750}, {1, 1371, 1, 4751}, {2, 1373, 1, 4751}, {3, 1374, 1, 4751}, {1, 1372, 1,
4752}, {2, 1374, 1, 4752}, {3, 1375, 1, 4752}, {1, 1533, 1, 4753}, {2, 1534, 1, 4753}, {3,
1535, 1, 4753}, {1, 1536, 1, 4754}, {2, 1537, 1, 4754}, {3, 1538, 1, 4754}, {1, 1537, 1,
4755}, {2, 1539, 1, 4755}, {3, 1540, 1, 4755}, {1, 1538, 1, 4756}, {2, 1540, 1, 4756}, {3,
1541, 1, 4756}, {1, 1964, 1, 4757}, {2, 1965, 1, 4757}, {3, 1966, 1, 4757}, {1, 1965, 1,
4758}, {2, 1967, 1, 4758}, {3, 1968, 1, 4758}, {1, 1966, 1, 4759}, {2, 1968, 1, 4759}, {3,
1969, 1, 4759}, {1, 2064, 1, 4760}, {2, 2065, 1, 4760}, {3, 2066, 1, 4760}, {1, 2067, 1,

4761}, {2, 2068, 1, 4761}, {3, 2069, 1, 4761}, {1, 2068, 1, 4762}, {2, 2070, 1, 4762}, {3, 2071, 1, 4762}, {1, 2069, 1, 4763}, {2, 2071, 1, 4763}, {3, 2072, 1, 4763}, {1, 2345, 1, 4764}, {2, 2346, 1, 4764}, {3, 2347, 1, 4764}, {1, 2346, 1, 4765}, {2, 2348, 1, 4765}, {3, 2349, 1, 4765}, {1, 2347, 1, 4766}, {2, 2349, 1, 4766}, {3, 2350, 1, 4766}, {1, 2445, 1, 4767}, {2, 2446, 1, 4767}, {3, 2447, 1, 4767}, {1, 2448, 1, 4768}, {2, 2449, 1, 4768}, {3, 2450, 1, 4768}, {1, 2449, 1, 4769}, {2, 2451, 1, 4769}, {3, 2452, 1, 4769}, {1, 2450, 1, 4770}, {2, 2452, 1, 4770}, {3, 2453, 1, 4770}, {1, 2615, 1, 4771}, {2, 2616, 1, 4771}, {3, 2617, 1, 4771}, {1, 2616, 1, 4772}, {2, 2618, 1, 4772}, {3, 2619, 1, 4772}, {1, 2617, 1, 4773}, {2, 2619, 1, 4773}, {3, 2620, 1, 4773}, {1, 2670, 1, 4774}, {2, 2671, 1, 4774}, {3, 2672, 1, 4774}, {1, 2673, 1, 4775}, {2, 2674, 1, 4775}, {3, 2675, 1, 4775}, {1, 2674, 1, 4776}, {2, 2676, 1, 4776}, {3, 2677, 1, 4776}, {1, 2675, 1, 4777}, {2, 2677, 1, 4777}, {3, 2678, 1, 4777}, {1, 2710, 1, 4778}, {2, 2711, 1, 4778}, {3, 2712, 1, 4778}, {1, 2711, 1, 4779}, {2, 2713, 1, 4779}, {3, 2714, 1, 4779}, {1, 2712, 1, 4780}, {2, 2714, 1, 4780}, {3, 2715, 1, 4780}, {1, 2735, 1, 4781}, {2, 2736, 1, 4781}, {3, 2737, 1, 4781}, {1, 2751, 1, 4782}, {2, 2752, 1, 4782}, {3, 2753, 1, 4782}, {4, 717, 1, 4783}, {5, 718, 1, 4783}, {6, 719, 1, 4783}, {4, 718, 1, 4784}, {5, 720, 1, 4784}, {6, 721, 1, 4784}, {4, 719, 1, 4785}, {5, 721, 1, 4785}, {6, 722, 1, 4785}, {5, 717, 1, 4786}, {7, 718, 1, 4786}, {8, 719, 1, 4786}, {5, 718, 1, 4787}, {7, 720, 1, 4787}, {8, 721, 1, 4787}, {5, 719, 1, 4788}, {7, 721, 1, 4788}, {8, 722, 1, 4788}, {6, 717, 1, 4789}, {8, 718, 1, 4789}, {9, 719, 1, 4789}, {6, 718, 1, 4790}, {8, 720, 1, 4790}, {9, 721, 1, 4790}, {6, 719, 1, 4791}, {8, 721, 1, 4791}, {9, 722, 1, 4791}, {4, 772, 1, 4792}, {5, 773, 1, 4792}, {6, 774, 1, 4792}, {5, 772, 1, 4793}, {7, 773, 1, 4793}, {8, 774, 1, 4793}, {6, 772, 1, 4794}, {8, 773, 1, 4794}, {9, 774, 1, 4794}, {4, 827, 1, 4795}, {5, 828, 1, 4795}, {6, 829, 1, 4795}, {4, 828, 1, 4796}, {5, 830, 1, 4796}, {6, 831, 1, 4796}, {4, 829, 1, 4797}, {5, 831, 1, 4797}, {6, 832, 1, 4797}, {5, 827, 1, 4798}, {7, 828, 1, 4798}, {8, 829, 1, 4798}, {5, 828, 1, 4799}, {7, 830, 1, 4799}, {8, 831, 1, 4799}, {5, 829, 1, 4800}, {7, 831, 1, 4800}, {8, 832, 1, 4800}, {6, 827, 1, 4801}, {8, 828, 1, 4801}, {9, 829, 1, 4801}, {6, 828, 1, 4802}, {8, 830, 1, 4802}, {9, 831, 1, 4802}, {6, 829, 1, 4803}, {8, 831, 1, 4803}, {9, 832, 1, 4803}, {4, 990, 1, 4804}, {5, 991, 1, 4804}, {6, 992, 1, 4804}, {5, 990, 1, 4805}, {7, 991, 1, 4805}, {8, 992, 1, 4805}, {6, 990, 1, 4806}, {8, 991, 1, 4806}, {9, 992, 1, 4806}, {4, 993, 1, 4807}, {5, 994, 1, 4807}, {6, 995, 1, 4807}, {4, 994, 1, 4808}, {5, 996, 1, 4808}, {6, 997, 1, 4808}, {4, 995, 1, 4809}, {5, 997, 1, 4809}, {6, 998, 1, 4809}, {5, 993, 1, 4810}, {7, 994, 1, 4810}, {8, 995, 1, 4810}, {5, 994, 1, 4811}, {7, 996, 1, 4811}, {8, 997, 1, 4811}, {5, 995, 1, 4812}, {7, 997, 1, 4812}, {8, 998, 1, 4812}, {6, 993, 1, 4813}, {8, 994, 1, 4813}, {9, 995, 1, 4813}, {6, 994, 1, 4814}, {8, 996, 1, 4814}, {9, 997, 1, 4814}, {6, 995, 1, 4815}, {8, 997, 1, 4815}, {9, 998, 1, 4815}, {4, 1370, 1, 4816}, {5, 1371, 1, 4816}, {6, 1372, 1, 4816}, {4, 1371, 1, 4817}, {5, 1373, 1, 4817}, {6, 1374, 1, 4817}, {4, 1372, 1, 4818}, {5, 1374, 1, 4818}, {6, 1375, 1, 4818}, {5, 1370, 1, 4819}, {7, 1371, 1, 4819}, {8, 1372, 1, 4819}, {5, 1371, 1, 4820}, {7, 1373, 1, 4820}, {8, 1374, 1, 4820}, {5, 1372, 1, 4821}, {7, 1374, 1, 4821}, {8, 1375, 1, 4821}, {6, 1370, 1, 4822}, {8, 1371, 1, 4822}, {9, 1372, 1, 4822}, {6, 1371, 1, 4823}, {8, 1373, 1, 4823}, {9, 1374, 1, 4823}, {6, 1372, 1, 4824}, {8, 1374, 1, 4824}, {9, 1375, 1, 4824}, {4, 1533, 1, 4825}, {5, 1534, 1, 4825}, {6, 1535, 1, 4825}, {5, 1533, 1, 4826}, {7, 1534, 1, 4826}, {8, 1535, 1, 4826}, {6, 1533, 1, 4827}, {8, 1534, 1, 4827}, {9, 1535, 1, 4827}, {4, 1536, 1, 4828}, {5, 1537, 1, 4828}, {6, 1538, 1, 4828}, {4, 1537, 1, 4829}, {5, 1539, 1, 4829}, {6, 1540, 1, 4829}, {4, 1538, 1, 4830}, {5, 1540, 1, 4830}, {6, 1541, 1, 4830}, {5, 1536, 1, 4831}, {7, 1537, 1, 4831}, {8, 1538, 1, 4831}, {5, 1537, 1, 4832}, {7, 1539, 1, 4832}, {8, 1540, 1, 4832}, {5, 1538, 1, 4833}, {7, 1540, 1, 4833}, {8, 1541, 1, 4833}, {6, 1536, 1, 4834}, {8, 1537, 1, 4834}, {9, 1538, 1, 4834}, {6, 1537, 1, 4835}, {8, 1539, 1, 4835}, {9, 1540, 1, 4835}, {6, 1538, 1, 4836}, {8, 1540, 1, 4836}, {9, 1541, 1, 4836}, {4, 1964, 1, 4837}, {5, 1965, 1, 4837}, {6, 1966, 1, 4837}, {4, 1965, 1, 4838}, {5, 1967, 1, 4838}, {6, 1968, 1, 4838}, {4, 1966, 1, 4839}, {5, 1968, 1, 4839}, {6, 1969, 1, 4839}, {5, 1964, 1, 4840}, {7, 1965, 1, 4840}, {8, 1966, 1, 4840}, {5, 1965, 1, 4841}, {7, 1967, 1, 4841}, {8, 1968, 1, 4841}, {5, 1966, 1, 4842}, {7, 1968, 1, 4842}, {8, 1969, 1, 4842}, {6, 1964, 1, 4843}, {8, 1965, 1, 4843}, {9, 1966, 1, 4843}, {6, 1965, 1, 4844}, {8, 1967, 1, 4844}, {9, 1968, 1, 4844}, {6, 1966, 1, 4845}, {8, 1968, 1, 4845}, {9, 1969, 1, 4845}, {4, 2064, 1, 4846}, {5, 2065, 1, 4846}, {6, 2066, 1, 4846}, {5, 2064, 1, 4847}, {7, 2065, 1, 4847}, {8, 2066, 1, 4847}, {6, 2064, 1, 4848}, {8, 2065, 1, 4848}, {9, 2066, 1, 4848}, {4, 2067, 1, 4849}, {5, 2068, 1, 4849}, {6, 2069, 1, 4849}, {4, 2068, 1, 4850}, {5, 2070, 1, 4850}, {6, 2071, 1, 4850}, {4, 2069, 1, 4851}, {5, 2071, 1, 4851}, {6, 2072, 1, 4851}, {5, 2067, 1, 4852}, {7, 2068, 1, 4852}, {8, 2069, 1, 4852}, {5, 2068, 1, 4853}, {7, 2070, 1, 4853}, {8, 2071, 1, 4853}, {5, 2069, 1, 4854}, {7, 2071, 1, 4854}, {8, 2072, 1, 4854}, {6, 2067, 1, 4855}, {8, 2068, 1, 4855}, {9, 2069, 1, 4855}, {6, 2068, 1, 4856}, {8, 2070, 1, 4856}, {9, 2071, 1, 4856}, {6, 2069, 1, 4857}, {8, 2071, 1, 4857}, {9, 2072, 1, 4857}, {4, 2345, 1, 4858}, {5, 2346, 1, 4858}, {6, 2347, 1, 4858}, {4, 2346, 1, 4859}, {5, 2348, 1, 4859}, {6, 2349, 1, 4859}, {4, 2347, 1, 4860}, {5, 2349, 1, 4860}, {6, 2350, 1, 4860}, {5, 2345, 1, 4861}, {7, 2346, 1, 4861}, {8, 2347, 1, 4861}, {5, 2346, 1, 4862}, {7, 2348, 1, 4862}, {8, 2349, 1, 4862}, {5, 2347, 1, 4863}, {7, 2349, 1, 4863}, {8, 2350, 1, 4863}, {6, 2345, 1, 4864}, {8, 2346, 1, 4864}, {9, 2347, 1, 4864}, {6, 2346, 1, 4865}, {8, 2348, 1, 4865}, {9, 2349, 1,

4865}, {6, 2347, 1, 4866}, {8, 2349, 1, 4866}, {9, 2350, 1, 4866}, {4, 2445, 1, 4867}, {5, 2446, 1, 4867}, {6, 2447, 1, 4867}, {5, 2445, 1, 4868}, {7, 2446, 1, 4868}, {8, 2447, 1, 4868}, {6, 2445, 1, 4869}, {8, 2446, 1, 4869}, {9, 2447, 1, 4869}, {4, 2448, 1, 4870}, {5, 2449, 1, 4870}, {6, 2450, 1, 4870}, {4, 2449, 1, 4871}, {5, 2451, 1, 4871}, {6, 2452, 1, 4871}, {4, 2450, 1, 4872}, {5, 2452, 1, 4872}, {6, 2453, 1, 4872}, {5, 2448, 1, 4873}, {7, 2449, 1, 4873}, {8, 2450, 1, 4873}, {5, 2449, 1, 4874}, {7, 2451, 1, 4874}, {8, 2452, 1, 4874}, {5, 2450, 1, 4875}, {7, 2452, 1, 4875}, {8, 2453, 1, 4875}, {6, 2448, 1, 4876}, {8, 2449, 1, 4876}, {9, 2450, 1, 4876}, {6, 2449, 1, 4877}, {8, 2451, 1, 4877}, {9, 2452, 1, 4877}, {6, 2450, 1, 4878}, {8, 2452, 1, 4878}, {9, 2453, 1, 4878}, {4, 2615, 1, 4879}, {5, 2616, 1, 4879}, {6, 2617, 1, 4879}, {4, 2616, 1, 4880}, {5, 2618, 1, 4880}, {6, 2619, 1, 4880}, {4, 2617, 1, 4881}, {5, 2619, 1, 4881}, {6, 2620, 1, 4881}, {5, 2615, 1, 4882}, {7, 2616, 1, 4882}, {8, 2617, 1, 4882}, {5, 2616, 1, 4883}, {7, 2618, 1, 4883}, {8, 2619, 1, 4883}, {5, 2617, 1, 4884}, {7, 2619, 1, 4884}, {8, 2620, 1, 4884}, {6, 2615, 1, 4885}, {8, 2616, 1, 4885}, {9, 2617, 1, 4885}, {6, 2616, 1, 4886}, {8, 2618, 1, 4886}, {9, 2619, 1, 4886}, {6, 2617, 1, 4887}, {8, 2619, 1, 4887}, {9, 2620, 1, 4887}, {4, 2670, 1, 4888}, {5, 2671, 1, 4888}, {6, 2672, 1, 4888}, {5, 2670, 1, 4889}, {7, 2671, 1, 4889}, {8, 2672, 1, 4889}, {6, 2670, 1, 4890}, {8, 2671, 1, 4890}, {9, 2672, 1, 4890}, {4, 775, 1, 4891}, {5, 776, 2, 4891}, {6, 777, 2, 4891}, {7, 778, 1, 4891}, {8, 779, 2, 4891}, {9, 780, 1, 4891}, {4, 781, 1, 4892}, {5, 782, 2, 4892}, {6, 783, 2, 4892}, {7, 784, 1, 4892}, {8, 785, 2, 4892}, {9, 786, 1, 4892}, {4, 782, 1, 4893}, {5, 784, 2, 4893}, {6, 785, 2, 4893}, {7, 787, 1, 4893}, {8, 788, 2, 4893}, {9, 789, 1, 4893}, {4, 783, 1, 4894}, {5, 785, 2, 4894}, {6, 786, 2, 4894}, {7, 788, 1, 4894}, {8, 789, 2, 4894}, {9, 790, 1, 4894}, {4, 791, 1, 4895}, {5, 792, 2, 4895}, {6, 793, 2, 4895}, {7, 794, 1, 4895}, {8, 795, 2, 4895}, {9, 796, 1, 4895}, {4, 792, 1, 4896}, {5, 794, 2, 4896}, {6, 795, 2, 4896}, {7, 797, 1, 4896}, {8, 798, 2, 4896}, {9, 799, 1, 4896}, {4, 793, 1, 4897}, {5, 795, 2, 4897}, {6, 796, 2, 4897}, {7, 798, 1, 4897}, {8, 799, 2, 4897}, {9, 800, 1, 4897}, {4, 794, 1, 4898}, {5, 797, 2, 4898}, {6, 798, 2, 4898}, {7, 801, 1, 4898}, {8, 802, 2, 4898}, {9, 803, 1, 4898}, {4, 795, 1, 4899}, {5, 798, 2, 4899}, {6, 799, 2, 4899}, {7, 802, 1, 4899}, {8, 803, 2, 4899}, {9, 804, 1, 4899}, {4, 796, 1, 4900}, {5, 799, 2, 4900}, {6, 800, 2, 4900}, {7, 803, 1, 4900}, {8, 804, 2, 4900}, {9, 805, 1, 4900}, {4, 833, 1, 4901}, {5, 834, 2, 4901}, {6, 835, 2, 4901}, {7, 836, 1, 4901}, {8, 837, 2, 4901}, {9, 838, 1, 4901}, {4, 839, 1, 4902}, {5, 840, 2, 4902}, {6, 841, 2, 4902}, {7, 842, 1, 4902}, {8, 843, 2, 4902}, {9, 844, 1, 4902}, {4, 845, 1, 4903}, {5, 846, 2, 4903}, {6, 847, 2, 4903}, {7, 848, 1, 4903}, {8, 849, 2, 4903}, {9, 850, 1, 4903}, {4, 993, 1, 4904}, {5, 994, 2, 4904}, {6, 995, 2, 4904}, {7, 996, 1, 4904}, {8, 997, 2, 4904}, {9, 998, 1, 4904}, {4, 999, 1, 4905}, {5, 1000, 2, 4905}, {6, 1001, 2, 4905}, {7, 1002, 1, 4905}, {8, 1003, 2, 4905}, {9, 1004, 1, 4905}, {4, 1000, 1, 4906}, {5, 1002, 2, 4906}, {6, 1003, 2, 4906}, {7, 1005, 1, 4906}, {8, 1006, 2, 4906}, {9, 1007, 1, 4906}, {4, 1001, 1, 4907}, {5, 1003, 2, 4907}, {6, 1004, 2, 4907}, {7, 1006, 1, 4907}, {8, 1007, 2, 4907}, {9, 1008, 1, 4907}, {4, 1009, 1, 4908}, {5, 1010, 2, 4908}, {6, 1011, 2, 4908}, {7, 1012, 1, 4908}, {8, 1013, 2, 4908}, {9, 1014, 1, 4908}, {4, 1010, 1, 4909}, {5, 1012, 2, 4909}, {6, 1013, 2, 4909}, {7, 1015, 1, 4909}, {8, 1016, 2, 4909}, {9, 1017, 1, 4909}, {4, 1011, 1, 4910}, {5, 1013, 2, 4910}, {6, 1014, 2, 4910}, {7, 1016, 1, 4910}, {8, 1017, 2, 4910}, {9, 1018, 1, 4910}, {4, 1012, 1, 4911}, {5, 1015, 2, 4911}, {6, 1016, 2, 4911}, {7, 1019, 1, 4911}, {8, 1020, 2, 4911}, {9, 1021, 1, 4911}, {4, 1013, 1, 4912}, {5, 1016, 2, 4912}, {6, 1017, 2, 4912}, {7, 1020, 1, 4912}, {8, 1021, 2, 4912}, {9, 1022, 1, 4912}, {4, 1014, 1, 4913}, {5, 1017, 2, 4913}, {6, 1018, 2, 4913}, {7, 1021, 1, 4913}, {8, 1022, 2, 4913}, {9, 1023, 1, 4913}, {4, 1073, 1, 4914}, {5, 1074, 2, 4914}, {6, 1075, 2, 4914}, {7, 1076, 1, 4914}, {8, 1077, 2, 4914}, {9, 1078, 1, 4914}, {4, 1074, 1, 4915}, {5, 1079, 2, 4915}, {6, 1080, 2, 4915}, {7, 1081, 1, 4915}, {8, 1082, 2, 4915}, {9, 1083, 1, 4915}, {4, 1075, 1, 4916}, {5, 1080, 2, 4916}, {6, 1084, 2, 4916}, {7, 1085, 1, 4916}, {8, 1086, 2, 4916}, {9, 1087, 1, 4916}, {4, 1076, 1, 4917}, {5, 1081, 2, 4917}, {6, 1085, 2, 4917}, {7, 1088, 1, 4917}, {8, 1089, 2, 4917}, {9, 1090, 1, 4917}, {4, 1077, 1, 4918}, {5, 1082, 2, 4918}, {6, 1086, 2, 4918}, {7, 1089, 1, 4918}, {8, 1091, 2, 4918}, {9, 1092, 1, 4918}, {4, 1078, 1, 4919}, {5, 1083, 2, 4919}, {6, 1087, 2, 4919}, {7, 1090, 1, 4919}, {8, 1092, 2, 4919}, {9, 1093, 1, 4919}, {4, 1376, 1, 4920}, {5, 1377, 2, 4920}, {6, 1378, 2, 4920}, {7, 1379, 1, 4920}, {8, 1380, 2, 4920}, {9, 1381, 1, 4920}, {4, 1382, 1, 4921}, {5, 1383, 2, 4921}, {6, 1384, 2, 4921}, {7, 1385, 1, 4921}, {8, 1386, 2, 4921}, {9, 1387, 1, 4921}, {4, 1388, 1, 4922}, {5, 1389, 2, 4922}, {6, 1390, 2, 4922}, {7, 1391, 1, 4922}, {8, 1392, 2, 4922}, {9, 1393, 1, 4922}, {4, 1536, 1, 4923}, {5, 1537, 2, 4923}, {6, 1538, 2, 4923}, {7, 1539, 1, 4923}, {8, 1540, 2, 4923}, {9, 1541, 1, 4923}, {4, 1542, 1, 4924}, {5, 1543, 2, 4924}, {6, 1544, 2, 4924}, {7, 1545, 1, 4924}, {8, 1546, 2, 4924}, {9, 1547, 1, 4924}, {4, 1543, 1, 4925}, {5, 1545, 2, 4925}, {6, 1546, 2, 4925}, {7, 1548, 1, 4925}, {8, 1549, 2, 4925}, {9, 1550, 1, 4925}, {4, 1544, 1, 4926}, {5, 1546, 2, 4926}, {6, 1547, 2, 4926}, {7, 1549, 1, 4926}, {8, 1550, 2, 4926}, {9, 1551, 1, 4926}, {4, 1552, 1, 4927}, {5, 1553, 2, 4927}, {6, 1554, 2, 4927}, {7, 1555, 1, 4927}, {8, 1556, 2, 4927}, {9, 1557, 1, 4927}, {4, 1553, 1, 4928}, {5, 1555, 2, 4928}, {6, 1556, 2, 4928}, {7, 1558, 1, 4928}, {8, 1559, 2, 4928}, {9, 1560, 1, 4928}, {4, 1554, 1, 4929}, {5, 1556, 2, 4929}, {6, 1557, 2, 4929}, {7, 1559, 1, 4929}, {8, 1560, 2, 4929}, {9, 1561, 1, 4929}, {4, 1555, 1, 4930}, {5, 1558, 2, 4930}, {6, 1559, 2, 4930}, {7, 1562, 1, 4930}, {8,

1563, 2, 4930}, {9, 1564, 1, 4930}, {4, 1556, 1, 4931}, {5, 1559, 2, 4931}, {6, 1560, 2, 4931}, {7, 1563, 1, 4931}, {8, 1564, 2, 4931}, {9, 1565, 1, 4931}, {4, 1557, 1, 4932}, {5, 1560, 2, 4932}, {6, 1561, 2, 4932}, {7, 1564, 1, 4932}, {8, 1565, 2, 4932}, {9, 1566, 1, 4932}, {4, 1793, 1, 4933}, {5, 1794, 2, 4933}, {6, 1795, 2, 4933}, {7, 1796, 1, 4933}, {8, 1797, 2, 4933}, {9, 1798, 1, 4933}, {4, 1794, 1, 4934}, {5, 1799, 2, 4934}, {6, 1800, 2, 4934}, {7, 1801, 1, 4934}, {8, 1802, 2, 4934}, {9, 1803, 1, 4934}, {4, 1795, 1, 4935}, {5, 1800, 2, 4935}, {6, 1804, 2, 4935}, {7, 1805, 1, 4935}, {8, 1806, 2, 4935}, {9, 1807, 1, 4935}, {4, 1796, 1, 4936}, {5, 1801, 2, 4936}, {6, 1805, 2, 4936}, {7, 1808, 1, 4936}, {8, 1809, 2, 4936}, {9, 1810, 1, 4936}, {4, 1797, 1, 4937}, {5, 1802, 2, 4937}, {6, 1806, 2, 4937}, {7, 1809, 1, 4937}, {8, 1811, 2, 4937}, {9, 1812, 1, 4937}, {4, 1798, 1, 4938}, {5, 1803, 2, 4938}, {6, 1807, 2, 4938}, {7, 1810, 1, 4938}, {8, 1812, 2, 4938}, {9, 1813, 1, 4938}, {4, 1970, 1, 4939}, {5, 1971, 2, 4939}, {6, 1972, 2, 4939}, {7, 1973, 1, 4939}, {8, 1974, 2, 4939}, {9, 1975, 1, 4939}, {4, 1976, 1, 4940}, {5, 1977, 2, 4940}, {6, 1978, 2, 4940}, {7, 1979, 1, 4940}, {8, 1980, 2, 4940}, {9, 1981, 1, 4940}, {4, 1982, 1, 4941}, {5, 1983, 2, 4941}, {6, 1984, 2, 4941}, {7, 1985, 1, 4941}, {8, 1986, 2, 4941}, {9, 1987, 1, 4941}, {4, 2067, 1, 4942}, {5, 2068, 2, 4942}, {6, 2069, 2, 4942}, {7, 2070, 1, 4942}, {8, 2071, 2, 4942}, {9, 2072, 1, 4942}, {4, 2073, 1, 4943}, {5, 2074, 2, 4943}, {6, 2075, 2, 4943}, {7, 2076, 1, 4943}, {8, 2077, 2, 4943}, {9, 2078, 1, 4943}, {4, 2074, 1, 4944}, {5, 2076, 2, 4944}, {6, 2077, 2, 4944}, {7, 2079, 1, 4944}, {8, 2080, 2, 4944}, {9, 2081, 1, 4944}, {4, 2075, 1, 4945}, {5, 2077, 2, 4945}, {6, 2078, 2, 4945}, {7, 2080, 1, 4945}, {8, 2081, 2, 4945}, {9, 2082, 1, 4945}, {4, 2083, 1, 4946}, {5, 2084, 2, 4946}, {6, 2085, 2, 4946}, {7, 2086, 1, 4946}, {8, 2087, 2, 4946}, {9, 2088, 1, 4946}, {4, 2084, 1, 4947}, {5, 2086, 2, 4947}, {6, 2087, 2, 4947}, {7, 2089, 1, 4947}, {8, 2090, 2, 4947}, {9, 2091, 1, 4947}, {4, 2085, 1, 4948}, {5, 2087, 2, 4948}, {6, 2088, 2, 4948}, {7, 2090, 1, 4948}, {8, 2091, 2, 4948}, {9, 2092, 1, 4948}, {4, 2086, 1, 4949}, {5, 2089, 2, 4949}, {6, 2090, 2, 4949}, {7, 2093, 1, 4949}, {8, 2094, 2, 4949}, {9, 2095, 1, 4949}, {4, 2087, 1, 4950}, {5, 2090, 2, 4950}, {6, 2091, 2, 4950}, {7, 2094, 1, 4950}, {8, 2095, 2, 4950}, {9, 2096, 1, 4950}, {4, 2088, 1, 4951}, {5, 2091, 2, 4951}, {6, 2092, 2, 4951}, {7, 2095, 1, 4951}, {8, 2096, 2, 4951}, {9, 2097, 1, 4951}, {4, 2174, 1, 4952}, {5, 2175, 2, 4952}, {6, 2176, 2, 4952}, {7, 2177, 1, 4952}, {8, 2178, 2, 4952}, {9, 2179, 1, 4952}, {4, 2175, 1, 4953}, {5, 2180, 2, 4953}, {6, 2181, 2, 4953}, {7, 2182, 1, 4953}, {8, 2183, 2, 4953}, {9, 2184, 1, 4953}, {4, 2176, 1, 4954}, {5, 2181, 2, 4954}, {6, 2185, 2, 4954}, {7, 2186, 1, 4954}, {8, 2187, 2, 4954}, {9, 2188, 1, 4954}, {4, 2177, 1, 4955}, {5, 2182, 2, 4955}, {6, 2186, 2, 4955}, {7, 2189, 1, 4955}, {8, 2190, 2, 4955}, {9, 2191, 1, 4955}, {4, 2178, 1, 4956}, {5, 2183, 2, 4956}, {6, 2187, 2, 4956}, {7, 2190, 1, 4956}, {8, 2192, 2, 4956}, {9, 2193, 1, 4956}, {4, 2179, 1, 4957}, {5, 2184, 2, 4957}, {6, 2188, 2, 4957}, {7, 2191, 1, 4957}, {8, 2193, 2, 4957}, {9, 2194, 1, 4957}, {4, 2351, 1, 4958}, {5, 2352, 2, 4958}, {6, 2353, 2, 4958}, {7, 2354, 1, 4958}, {8, 2355, 2, 4958}, {9, 2356, 1, 4958}, {4, 2357, 1, 4959}, {5, 2358, 2, 4959}, {6, 2359, 2, 4959}, {7, 2360, 1, 4959}, {8, 2361, 2, 4959}, {9, 2362, 1, 4959}, {4, 2363, 1, 4960}, {5, 2364, 2, 4960}, {6, 2365, 2, 4960}, {7, 2366, 1, 4960}, {8, 2367, 2, 4960}, {9, 2368, 1, 4960}, {4, 2448, 1, 4961}, {5, 2449, 2, 4961}, {6, 2450, 2, 4961}, {7, 2451, 1, 4961}, {8, 2452, 2, 4961}, {9, 2453, 1, 4961}, {4, 2454, 1, 4962}, {5, 2455, 2, 4962}, {6, 2456, 2, 4962}, {7, 2457, 1, 4962}, {8, 2458, 2, 4962}, {9, 2459, 1, 4962}, {4, 2455, 1, 4963}, {5, 2457, 2, 4963}, {6, 2458, 2, 4963}, {7, 2460, 1, 4963}, {8, 2461, 2, 4963}, {9, 2462, 1, 4963}, {4, 2456, 1, 4964}, {5, 2458, 2, 4964}, {6, 2459, 2, 4964}, {7, 2461, 1, 4964}, {8, 2462, 2, 4964}, {9, 2463, 1, 4964}, {4, 2534, 1, 4965}, {5, 2535, 2, 4965}, {6, 2536, 2, 4965}, {7, 2537, 1, 4965}, {8, 2538, 2, 4965}, {9, 2539, 1, 4965}, {4, 2535, 1, 4966}, {5, 2540, 2, 4966}, {6, 2541, 2, 4966}, {7, 2542, 1, 4966}, {8, 2543, 2, 4966}, {9, 2544, 1, 4966}, {4, 2536, 1, 4967}, {5, 2541, 2, 4967}, {6, 2545, 2, 4967}, {7, 2546, 1, 4967}, {8, 2547, 2, 4967}, {9, 2548, 1, 4967}, {4, 2537, 1, 4968}, {5, 2542, 2, 4968}, {6, 2546, 2, 4968}, {7, 2549, 1, 4968}, {8, 2550, 2, 4968}, {9, 2551, 1, 4968}, {4, 2538, 1, 4969}, {5, 2543, 2, 4969}, {6, 2547, 2, 4969}, {7, 2550, 1, 4969}, {8, 2552, 2, 4969}, {9, 2553, 1, 4969}, {4, 2539, 1, 4970}, {5, 2544, 2, 4970}, {6, 2548, 2, 4970}, {7, 2551, 1, 4970}, {8, 2553, 2, 4970}, {9, 2554, 1, 4970}, {4, 2621, 1, 4971}, {5, 2622, 2, 4971}, {6, 2623, 2, 4971}, {7, 2624, 1, 4971}, {8, 2625, 2, 4971}, {9, 2626, 1, 4971}, {4, 2627, 1, 4972}, {5, 2628, 2, 4972}, {6, 2629, 2, 4972}, {7, 2630, 1, 4972}, {8, 2631, 2, 4972}, {9, 2632, 1, 4972}, {4, 2633, 1, 4973}, {5, 2634, 2, 4973}, {6, 2635, 2, 4973}, {7, 2636, 1, 4973}, {8, 2637, 2, 4973}, {9, 2638, 1, 4973}, {4, 2673, 1, 4974}, {5, 2674, 2, 4974}, {6, 2675, 2, 4974}, {7, 2676, 1, 4974}, {8, 2677, 2, 4974}, {9, 2678, 1, 4974}, {4, 2738, 1, 4975}, {5, 2739, 2, 4975}, {6, 2740, 2, 4975}, {7, 2741, 1, 4975}, {8, 2742, 2, 4975}, {9, 2743, 1, 4975}, {4, 717, 1, 4976}, {5, 718, 2, 4976}, {6, 719, 2, 4976}, {7, 720, 1, 4976}, {8, 721, 2, 4976}, {9, 722, 1, 4976}, {4, 723, 1, 4977}, {5, 724, 1, 4977}, {5, 729, 1, 4977}, {6, 725, 1, 4977}, {6, 735, 1, 4977}, {7, 730, 1, 4977}, {8, 731, 1, 4977}, {8, 736, 1, 4977}, {9, 737, 1, 4977}, {4, 724, 1, 4978}, {5, 726, 1, 4978}, {5, 730, 1, 4978}, {6, 727, 1, 4978}, {6, 736, 1, 4978}, {7, 732, 1, 4978}, {8, 733, 1, 4978}, {8, 738, 1, 4978}, {9, 739, 1, 4978}, {4, 725, 1, 4979}, {5, 727, 1, 4979}, {5, 731, 1, 4979}, {6, 728, 1, 4979}, {6, 737, 1, 4979}, {7, 733, 1, 4979}, {8, 734, 1, 4979}, {8, 739, 1, 4979}, {9, 740, 1, 4979}, {4, 741, 1, 4980}, {5, 742, 1, 4980}, {5, 751, 1, 4980}, {6, 743, 1, 4980}, {6, 761, 1, 4980}, {7, 752, 1, 4980}, {8, 753, 1, 4980},

{8, 762, 1, 4980}, {9, 763, 1, 4980}, {4, 742, 1, 4981}, {5, 744, 1, 4981}, {5, 752, 1, 4981}, {6, 745, 1, 4981}, {6, 762, 1, 4981}, {7, 754, 1, 4981}, {8, 755, 1, 4981}, {8, 764, 1, 4981}, {9, 765, 1, 4981}, {4, 743, 1, 4982}, {5, 745, 1, 4982}, {5, 753, 1, 4982}, {6, 746, 1, 4982}, {6, 763, 1, 4982}, {7, 755, 1, 4982}, {8, 756, 1, 4982}, {8, 765, 1, 4982}, {9, 766, 1, 4982}, {4, 744, 1, 4983}, {5, 747, 1, 4983}, {5, 754, 1, 4983}, {6, 748, 1, 4983}, {6, 764, 1, 4983}, {7, 757, 1, 4983}, {8, 758, 1, 4983}, {8, 767, 1, 4983}, {9, 768, 1, 4983}, {4, 745, 1, 4984}, {5, 748, 1, 4984}, {5, 755, 1, 4984}, {6, 749, 1, 4984}, {6, 765, 1, 4984}, {7, 758, 1, 4984}, {8, 759, 1, 4984}, {8, 768, 1, 4984}, {9, 769, 1, 4984}, {4, 746, 1, 4985}, {5, 749, 1, 4985}, {5, 756, 1, 4985}, {6, 750, 1, 4985}, {6, 766, 1, 4985}, {7, 759, 1, 4985}, {8, 760, 1, 4985}, {8, 769, 1, 4985}, {9, 770, 1, 4985}, {4, 806, 1, 4986}, {5, 807, 2, 4986}, {6, 808, 2, 4986}, {7, 812, 1, 4986}, {8, 813, 2, 4986}, {9, 817, 1, 4986}, {4, 807, 1, 4987}, {5, 809, 1, 4987}, {5, 812, 1, 4987}, {6, 810, 1, 4987}, {6, 813, 1, 4987}, {7, 814, 1, 4987}, {8, 815, 1, 4987}, {8, 818, 1, 4987}, {9, 819, 1, 4987}, {4, 808, 1, 4988}, {5, 810, 1, 4988}, {5, 813, 1, 4988}, {6, 811, 1, 4988}, {6, 817, 1, 4988}, {7, 815, 1, 4988}, {8, 816, 1, 4988}, {8, 819, 1, 4988}, {9, 820, 1, 4988}, {4, 812, 1, 4989}, {5, 814, 2, 4989}, {6, 815, 2, 4989}, {7, 821, 1, 4989}, {8, 822, 2, 4989}, {9, 824, 1, 4989}, {4, 813, 1, 4990}, {5, 815, 1, 4990}, {5, 818, 1, 4990}, {6, 816, 1, 4990}, {6, 819, 1, 4990}, {7, 822, 1, 4990}, {8, 823, 1, 4990}, {8, 824, 1, 4990}, {9, 825, 1, 4990}, {4, 817, 1, 4991}, {5, 819, 2, 4991}, {6, 820, 2, 4991}, {7, 824, 1, 4991}, {8, 825, 2, 4991}, {9, 826, 1, 4991}, {4, 827, 1, 4992}, {5, 828, 2, 4992}, {6, 829, 2, 4992}, {7, 830, 1, 4992}, {8, 831, 2, 4992}, {9, 832, 1, 4992}, {4, 833, 1, 4993}, {5, 834, 1, 4993}, {5, 839, 1, 4993}, {6, 835, 1, 4993}, {6, 845, 1, 4993}, {7, 840, 1, 4993}, {8, 841, 1, 4993}, {8, 846, 1, 4993}, {9, 847, 1, 4993}, {4, 834, 1, 4994}, {5, 836, 1, 4994}, {5, 840, 1, 4994}, {6, 837, 1, 4994}, {6, 846, 1, 4994}, {7, 842, 1, 4994}, {8, 843, 1, 4994}, {8, 848, 1, 4994}, {9, 849, 1, 4994}, {4, 835, 1, 4995}, {5, 837, 1, 4995}, {5, 841, 1, 4995}, {6, 838, 1, 4995}, {6, 847, 1, 4995}, {7, 843, 1, 4995}, {8, 844, 1, 4995}, {8, 849, 1, 4995}, {9, 850, 1, 4995}, {4, 851, 1, 4996}, {5, 852, 1, 4996}, {5, 861, 1, 4996}, {6, 853, 1, 4996}, {6, 871, 1, 4996}, {7, 862, 1, 4996}, {8, 863, 1, 4996}, {8, 872, 1, 4996}, {9, 873, 1, 4996}, {4, 852, 1, 4997}, {5, 854, 1, 4997}, {5, 862, 1, 4997}, {6, 855, 1, 4997}, {6, 872, 1, 4997}, {7, 864, 1, 4997}, {8, 865, 1, 4997}, {8, 874, 1, 4997}, {9, 875, 1, 4997}, {4, 853, 1, 4998}, {5, 855, 1, 4998}, {5, 863, 1, 4998}, {6, 856, 1, 4998}, {6, 873, 1, 4998}, {7, 865, 1, 4998}, {8, 866, 1, 4998}, {8, 875, 1, 4998}, {9, 876, 1, 4998}, {4, 854, 1, 4999}, {5, 857, 1, 4999}, {5, 864, 1, 4999}, {6, 858, 1, 4999}, {6, 874, 1, 4999}, {7, 867, 1, 4999}, {8, 868, 1, 4999}, {8, 877, 1, 4999}, {9, 878, 1, 4999}, {4, 855, 1, 5000}, {5, 858, 1, 5000}, {5, 865, 1, 5000}, {6, 859, 1, 5000}, {6, 875, 1, 5000}, {7, 868, 1, 5000}, {8, 869, 1, 5000}, {8, 878, 1, 5000}, {9, 879, 1, 5000}, {4, 856, 1, 5001}, {5, 859, 1, 5001}, {5, 866, 1, 5001}, {6, 860, 1, 5001}, {6, 876, 1, 5001}, {7, 869, 1, 5001}, {8, 870, 1, 5001}, {8, 879, 1, 5001}, {9, 880, 1, 5001}, {4, 1073, 1, 5002}, {5, 1074, 2, 5002}, {6, 1075, 2, 5002}, {7, 1079, 1, 5002}, {8, 1080, 2, 5002}, {9, 1084, 1, 5002}, {4, 1074, 1, 5003}, {5, 1076, 1, 5003}, {5, 1079, 1, 5003}, {6, 1077, 1, 5003}, {6, 1080, 1, 5003}, {7, 1081, 1, 5003}, {8, 1082, 1, 5003}, {8, 1085, 1, 5003}, {9, 1086, 1, 5003}, {4, 1075, 1, 5004}, {5, 1077, 1, 5004}, {5, 1080, 1, 5004}, {6, 1078, 1, 5004}, {6, 1084, 1, 5004}, {7, 1082, 1, 5004}, {8, 1083, 1, 5004}, {8, 1086, 1, 5004}, {9, 1087, 1, 5004}, {4, 1079, 1, 5005}, {5, 1081, 2, 5005}, {6, 1082, 2, 5005}, {7, 1088, 1, 5005}, {8, 1089, 2, 5005}, {9, 1091, 1, 5005}, {4, 1080, 1, 5006}, {5, 1082, 1, 5006}, {5, 1085, 1, 5006}, {6, 1083, 1, 5006}, {6, 1086, 1, 5006}, {7, 1089, 1, 5006}, {8, 1090, 1, 5006}, {8, 1091, 1, 5006}, {9, 1092, 1, 5006}, {4, 1084, 1, 5007}, {5, 1086, 2, 5007}, {6, 1087, 2, 5007}, {7, 1091, 1, 5007}, {8, 1092, 2, 5007}, {9, 1093, 1, 5007}, {4, 1370, 1, 5008}, {5, 1371, 2, 5008}, {6, 1372, 2, 5008}, {7, 1373, 1, 5008}, {8, 1374, 2, 5008}, {9, 1375, 1, 5008}, {4, 1376, 1, 5009}, {5, 1377, 1, 5009}, {5, 1382, 1, 5009}, {6, 1378, 1, 5009}, {6, 1388, 1, 5009}, {7, 1383, 1, 5009}, {8, 1384, 1, 5009}, {8, 1389, 1, 5009}, {9, 1390, 1, 5009}, {4, 1377, 1, 5010}, {5, 1379, 1, 5010}, {5, 1383, 1, 5010}, {6, 1380, 1, 5010}, {6, 1389, 1, 5010}, {7, 1385, 1, 5010}, {8, 1386, 1, 5010}, {8, 1391, 1, 5010}, {9, 1392, 1, 5010}, {4, 1378, 1, 5011}, {5, 1380, 1, 5011}, {5, 1384, 1, 5011}, {6, 1381, 1, 5011}, {6, 1390, 1, 5011}, {7, 1386, 1, 5011}, {8, 1387, 1, 5011}, {8, 1392, 1, 5011}, {9, 1393, 1, 5011}, {4, 1394, 1, 5012}, {5, 1395, 1, 5012}, {5, 1404, 1, 5012}, {6, 1396, 1, 5012}, {6, 1414, 1, 5012}, {7, 1405, 1, 5012}, {8, 1406, 1, 5012}, {8, 1415, 1, 5012}, {9, 1416, 1, 5012}, {4, 1395, 1, 5013}, {5, 1397, 1, 5013}, {5, 1405, 1, 5013}, {6, 1398, 1, 5013}, {6, 1415, 1, 5013}, {7, 1407, 1, 5013}, {8, 1408, 1, 5013}, {8, 1417, 1, 5013}, {9, 1418, 1, 5013}, {4, 1396, 1, 5014}, {5, 1398, 1, 5014}, {5, 1406, 1, 5014}, {6, 1399, 1, 5014}, {6, 1416, 1, 5014}, {7, 1408, 1, 5014}, {8, 1409, 1, 5014}, {8, 1418, 1, 5014}, {9, 1419, 1, 5014}, {4, 1397, 1, 5015}, {5, 1400, 1, 5015}, {5, 1407, 1, 5015}, {6, 1401, 1, 5015}, {6, 1417, 1, 5015}, {7, 1410, 1, 5015}, {8, 1411, 1, 5015}, {8, 1420, 1, 5015}, {9, 1421, 1, 5015}, {4, 1398, 1, 5016}, {5, 1401, 1, 5016}, {5, 1408, 1, 5016}, {6, 1402, 1, 5016}, {6, 1418, 1, 5016}, {7, 1411, 1, 5016}, {8, 1412, 1, 5016}, {8, 1421, 1, 5016}, {9, 1422, 1, 5016}, {4, 1399, 1, 5017}, {5, 1402, 1, 5017}, {5, 1409, 1, 5017}, {6, 1403, 1, 5017}, {6, 1419, 1, 5017}, {7, 1412, 1, 5017}, {8, 1413, 1, 5017}, {8, 1422, 1, 5017}, {9, 1423, 1, 5017}, {4, 1793, 1, 5018}, {5, 1794, 2, 5018}, {6, 1795, 2, 5018}, {7, 1799, 1, 5018}, {8, 1800, 2, 5018}, {9, 1804, 1,

5018}, {4, 1794, 1, 5019}, {5, 1796, 1, 5019}, {5, 1799, 1, 5019}, {6, 1797, 1, 5019}, {6,
1800, 1, 5019}, {7, 1801, 1, 5019}, {8, 1802, 1, 5019}, {8, 1805, 1, 5019}, {9, 1806, 1,
5019}, {4, 1795, 1, 5020}, {5, 1797, 1, 5020}, {5, 1800, 1, 5020}, {6, 1798, 1, 5020}, {6,
1804, 1, 5020}, {7, 1802, 1, 5020}, {8, 1803, 1, 5020}, {8, 1806, 1, 5020}, {9, 1807, 1,
5020}, {4, 1799, 1, 5021}, {5, 1801, 2, 5021}, {6, 1802, 2, 5021}, {7, 1808, 1, 5021}, {8,
1809, 2, 5021}, {9, 1811, 1, 5021}, {4, 1800, 1, 5022}, {5, 1802, 1, 5022}, {5, 1805, 1,
5022}, {6, 1803, 1, 5022}, {6, 1806, 1, 5022}, {7, 1809, 1, 5022}, {8, 1810, 1, 5022}, {8,
1811, 1, 5022}, {9, 1812, 1, 5022}, {4, 1804, 1, 5023}, {5, 1806, 2, 5023}, {6, 1807, 2,
5023}, {7, 1811, 1, 5023}, {8, 1812, 2, 5023}, {9, 1813, 1, 5023}, {4, 1964, 1, 5024}, {5,
1965, 2, 5024}, {6, 1966, 2, 5024}, {7, 1967, 1, 5024}, {8, 1968, 2, 5024}, {9, 1969, 1,
5024}, {4, 1970, 1, 5025}, {5, 1971, 1, 5025}, {5, 1976, 1, 5025}, {6, 1972, 1, 5025}, {6,
1982, 1, 5025}, {7, 1977, 1, 5025}, {8, 1978, 1, 5025}, {8, 1983, 1, 5025}, {9, 1984, 1,
5025}, {4, 1971, 1, 5026}, {5, 1973, 1, 5026}, {5, 1977, 1, 5026}, {6, 1974, 1, 5026}, {6,
1983, 1, 5026}, {7, 1979, 1, 5026}, {8, 1980, 1, 5026}, {8, 1985, 1, 5026}, {9, 1986, 1,
5026}, {4, 1972, 1, 5027}, {5, 1974, 1, 5027}, {5, 1978, 1, 5027}, {6, 1975, 1, 5027}, {6,
1984, 1, 5027}, {7, 1980, 1, 5027}, {8, 1981, 1, 5027}, {8, 1986, 1, 5027}, {9, 1987, 1,
5027}, {4, 1988, 1, 5028}, {5, 1989, 1, 5028}, {5, 1998, 1, 5028}, {6, 1990, 1, 5028}, {6,
2008, 1, 5028}, {7, 1999, 1, 5028}, {8, 2000, 1, 5028}, {8, 2009, 1, 5028}, {9, 2010, 1,
5028}, {4, 1989, 1, 5029}, {5, 1991, 1, 5029}, {5, 1999, 1, 5029}, {6, 1992, 1, 5029}, {6,
2009, 1, 5029}, {7, 2001, 1, 5029}, {8, 2002, 1, 5029}, {8, 2011, 1, 5029}, {9, 2012, 1,
5029}, {4, 1990, 1, 5030}, {5, 1992, 1, 5030}, {5, 2000, 1, 5030}, {6, 1993, 1, 5030}, {6,
2010, 1, 5030}, {7, 2002, 1, 5030}, {8, 2003, 1, 5030}, {8, 2012, 1, 5030}, {9, 2013, 1,
5030}, {4, 1991, 1, 5031}, {5, 1994, 1, 5031}, {5, 2001, 1, 5031}, {6, 1995, 1, 5031}, {6,
2011, 1, 5031}, {7, 2004, 1, 5031}, {8, 2005, 1, 5031}, {8, 2014, 1, 5031}, {9, 2015, 1,
5031}, {4, 1992, 1, 5032}, {5, 1995, 1, 5032}, {5, 2002, 1, 5032}, {6, 1996, 1, 5032}, {6,
2012, 1, 5032}, {7, 2005, 1, 5032}, {8, 2006, 1, 5032}, {8, 2015, 1, 5032}, {9, 2016, 1,
5032}, {4, 1993, 1, 5033}, {5, 1996, 1, 5033}, {5, 2003, 1, 5033}, {6, 1997, 1, 5033}, {6,
2013, 1, 5033}, {7, 2006, 1, 5033}, {8, 2007, 1, 5033}, {8, 2016, 1, 5033}, {9, 2017, 1,
5033}, {4, 2174, 1, 5034}, {5, 2175, 2, 5034}, {6, 2176, 2, 5034}, {7, 2180, 1, 5034}, {8,
2181, 2, 5034}, {9, 2185, 1, 5034}, {4, 2175, 1, 5035}, {5, 2177, 1, 5035}, {5, 2180, 1,
5035}, {6, 2178, 1, 5035}, {6, 2181, 1, 5035}, {7, 2182, 1, 5035}, {8, 2183, 1, 5035}, {8,
2186, 1, 5035}, {9, 2187, 1, 5035}, {4, 2176, 1, 5036}, {5, 2178, 1, 5036}, {5, 2181, 1,
5036}, {6, 2179, 1, 5036}, {6, 2185, 1, 5036}, {7, 2183, 1, 5036}, {8, 2184, 1, 5036}, {8,
2187, 1, 5036}, {9, 2188, 1, 5036}, {4, 2180, 1, 5037}, {5, 2182, 2, 5037}, {6, 2183, 2,
5037}, {7, 2189, 1, 5037}, {8, 2190, 2, 5037}, {9, 2192, 1, 5037}, {4, 2181, 1, 5038}, {5,
2183, 1, 5038}, {5, 2186, 1, 5038}, {6, 2184, 1, 5038}, {6, 2187, 1, 5038}, {7, 2190, 1,
5038}, {8, 2191, 1, 5038}, {8, 2192, 1, 5038}, {9, 2193, 1, 5038}, {4, 2185, 1, 5039}, {5,
2187, 2, 5039}, {6, 2188, 2, 5039}, {7, 2192, 1, 5039}, {8, 2193, 2, 5039}, {9, 2194, 1,
5039}, {4, 2345, 1, 5040}, {5, 2346, 2, 5040}, {6, 2347, 2, 5040}, {7, 2348, 1, 5040}, {8,
2349, 2, 5040}, {9, 2350, 1, 5040}, {4, 2351, 1, 5041}, {5, 2352, 1, 5041}, {5, 2357, 1,
5041}, {6, 2353, 1, 5041}, {6, 2363, 1, 5041}, {7, 2358, 1, 5041}, {8, 2359, 1, 5041}, {8,
2364, 1, 5041}, {9, 2365, 1, 5041}, {4, 2352, 1, 5042}, {5, 2354, 1, 5042}, {5, 2358, 1,
5042}, {6, 2355, 1, 5042}, {6, 2364, 1, 5042}, {7, 2360, 1, 5042}, {8, 2361, 1, 5042}, {8,
2366, 1, 5042}, {9, 2367, 1, 5042}, {4, 2353, 1, 5043}, {5, 2355, 1, 5043}, {5, 2359, 1,
5043}, {6, 2356, 1, 5043}, {6, 2365, 1, 5043}, {7, 2361, 1, 5043}, {8, 2362, 1, 5043}, {8,
2367, 1, 5043}, {9, 2368, 1, 5043}, {4, 2369, 1, 5044}, {5, 2370, 1, 5044}, {5, 2379, 1,
5044}, {6, 2371, 1, 5044}, {6, 2389, 1, 5044}, {7, 2380, 1, 5044}, {8, 2381, 1, 5044}, {8,
2390, 1, 5044}, {9, 2391, 1, 5044}, {4, 2370, 1, 5045}, {5, 2372, 1, 5045}, {5, 2380, 1,
5045}, {6, 2373, 1, 5045}, {6, 2390, 1, 5045}, {7, 2382, 1, 5045}, {8, 2383, 1, 5045}, {8,
2392, 1, 5045}, {9, 2393, 1, 5045}, {4, 2371, 1, 5046}, {5, 2373, 1, 5046}, {5, 2381, 1,
5046}, {6, 2374, 1, 5046}, {6, 2391, 1, 5046}, {7, 2383, 1, 5046}, {8, 2384, 1, 5046}, {8,
2393, 1, 5046}, {9, 2394, 1, 5046}, {4, 2372, 1, 5047}, {5, 2375, 1, 5047}, {5, 2382, 1,
5047}, {6, 2376, 1, 5047}, {6, 2392, 1, 5047}, {7, 2385, 1, 5047}, {8, 2386, 1, 5047}, {8,
2395, 1, 5047}, {9, 2396, 1, 5047}, {4, 2373, 1, 5048}, {5, 2376, 1, 5048}, {5, 2383, 1,
5048}, {6, 2377, 1, 5048}, {6, 2393, 1, 5048}, {7, 2386, 1, 5048}, {8, 2387, 1, 5048}, {8,
2396, 1, 5048}, {9, 2397, 1, 5048}, {4, 2374, 1, 5049}, {5, 2377, 1, 5049}, {5, 2384, 1,
5049}, {6, 2378, 1, 5049}, {6, 2394, 1, 5049}, {7, 2387, 1, 5049}, {8, 2388, 1, 5049}, {8,
2397, 1, 5049}, {9, 2398, 1, 5049}, {4, 2534, 1, 5050}, {5, 2535, 2, 5050}, {6, 2536, 2,
5050}, {7, 2540, 1, 5050}, {8, 2541, 2, 5050}, {9, 2545, 1, 5050}, {4, 2535, 1, 5051}, {5,
2537, 1, 5051}, {5, 2540, 1, 5051}, {6, 2538, 1, 5051}, {6, 2541, 1, 5051}, {7, 2542, 1,
5051}, {8, 2543, 1, 5051}, {8, 2546, 1, 5051}, {9, 2547, 1, 5051}, {4, 2536, 1, 5052}, {5,
2538, 1, 5052}, {5, 2541, 1, 5052}, {6, 2539, 1, 5052}, {6, 2545, 1, 5052}, {7, 2543, 1,
5052}, {8, 2544, 1, 5052}, {8, 2547, 1, 5052}, {9, 2548, 1, 5052}, {4, 2540, 1, 5053}, {5,
2542, 2, 5053}, {6, 2543, 2, 5053}, {7, 2549, 1, 5053}, {8, 2550, 2, 5053}, {9, 2552, 1,
5053}, {4, 2541, 1, 5054}, {5, 2543, 1, 5054}, {5, 2546, 1, 5054}, {6, 2544, 1, 5054}, {6,
2547, 1, 5054}, {7, 2550, 1, 5054}, {8, 2551, 1, 5054}, {8, 2552, 1, 5054}, {9, 2553, 1,
5054}, {4, 2545, 1, 5055}, {5, 2547, 2, 5055}, {6, 2548, 2, 5055}, {7, 2552, 1, 5055}, {8,
2553, 2, 5055}, {9, 2554, 1, 5055}, {4, 2615, 1, 5056}, {5, 2616, 2, 5056}, {6, 2617, 2,
5056}, {7, 2618, 1, 5056}, {8, 2619, 2, 5056}, {9, 2620, 1, 5056}, {4, 2621, 1, 5057}, {5,

2622, 1, 5057}, {5, 2627, 1, 5057}, {6, 2623, 1, 5057}, {6, 2633, 1, 5057}, {7, 2628, 1, 5057}, {8, 2629, 1, 5057}, {8, 2634, 1, 5057}, {9, 2635, 1, 5057}, {4, 2622, 1, 5058}, {5, 2624, 1, 5058}, {5, 2628, 1, 5058}, {6, 2625, 1, 5058}, {6, 2634, 1, 5058}, {7, 2630, 1, 5058}, {8, 2631, 1, 5058}, {8, 2636, 1, 5058}, {9, 2637, 1, 5058}, {4, 2623, 1, 5059}, {5, 2625, 1, 5059}, {5, 2629, 1, 5059}, {6, 2626, 1, 5059}, {6, 2635, 1, 5059}, {7, 2631, 1, 5059}, {8, 2632, 1, 5059}, {8, 2637, 1, 5059}, {9, 2638, 1, 5059}, {4, 2710, 1, 5060}, {5, 2711, 2, 5060}, {6, 2712, 2, 5060}, {7, 2713, 1, 5060}, {8, 2714, 2, 5060}, {9, 2715, 1, 5060}, {4, 2744, 1, 5061}, {5, 2745, 2, 5061}, {6, 2746, 2, 5061}, {7, 2747, 1, 5061}, {8, 2748, 2, 5061}, {9, 2749, 1, 5061}, {10, 833, 1, 5062}, {11, 834, 2, 5062}, {12, 835, 2, 5062}, {13, 836, 1, 5062}, {14, 837, 2, 5062}, {15, 838, 1, 5062}, {10, 839, 1, 5063}, {11, 840, 2, 5063}, {12, 841, 2, 5063}, {13, 842, 1, 5063}, {14, 843, 2, 5063}, {15, 844, 1, 5063}, {10, 845, 1, 5064}, {11, 846, 2, 5064}, {12, 847, 2, 5064}, {13, 848, 1, 5064}, {14, 849, 2, 5064}, {15, 850, 1, 5064}, {11, 833, 1, 5065}, {13, 834, 2, 5065}, {14, 835, 2, 5065}, {16, 836, 1, 5065}, {17, 837, 2, 5065}, {18, 838, 1, 5065}, {11, 839, 1, 5066}, {13, 840, 2, 5066}, {14, 841, 2, 5066}, {16, 842, 1, 5066}, {17, 843, 2, 5066}, {18, 844, 1, 5066}, {11, 845, 1, 5067}, {13, 846, 2, 5067}, {14, 847, 2, 5067}, {16, 848, 1, 5067}, {17, 849, 2, 5067}, {18, 850, 1, 5067}, {12, 833, 1, 5068}, {14, 834, 2, 5068}, {15, 835, 2, 5068}, {17, 836, 1, 5068}, {18, 837, 2, 5068}, {19, 838, 1, 5068}, {12, 839, 1, 5069}, {14, 840, 2, 5069}, {15, 841, 2, 5069}, {17, 842, 1, 5069}, {18, 843, 2, 5069}, {19, 844, 1, 5069}, {12, 845, 1, 5070}, {14, 846, 2, 5070}, {15, 847, 2, 5070}, {17, 848, 1, 5070}, {18, 849, 2, 5070}, {19, 850, 1, 5070}, {10, 993, 1, 5071}, {11, 994, 2, 5071}, {12, 995, 2, 5071}, {13, 996, 1, 5071}, {14, 997, 2, 5071}, {15, 998, 1, 5071}, {11, 993, 1, 5072}, {13, 994, 2, 5072}, {14, 995, 2, 5072}, {16, 996, 1, 5072}, {17, 997, 2, 5072}, {18, 998, 1, 5072}, {12, 993, 1, 5073}, {14, 994, 2, 5073}, {15, 995, 2, 5073}, {17, 996, 1, 5073}, {18, 997, 2, 5073}, {19, 998, 1, 5073}, {10, 999, 1, 5074}, {11, 1000, 2, 5074}, {12, 1001, 2, 5074}, {13, 1002, 1, 5074}, {14, 1003, 2, 5074}, {15, 1004, 1, 5074}, {10, 1000, 1, 5075}, {11, 1002, 2, 5075}, {12, 1003, 2, 5075}, {13, 1005, 1, 5075}, {14, 1006, 2, 5075}, {15, 1007, 1, 5075}, {10, 1001, 1, 5076}, {11, 1003, 2, 5076}, {12, 1004, 2, 5076}, {13, 1006, 1, 5076}, {14, 1007, 2, 5076}, {15, 1008, 1, 5076}, {11, 999, 1, 5077}, {13, 1000, 2, 5077}, {14, 1001, 2, 5077}, {16, 1002, 1, 5077}, {17, 1003, 2, 5077}, {18, 1004, 1, 5077}, {11, 1000, 1, 5078}, {13, 1002, 2, 5078}, {14, 1003, 2, 5078}, {16, 1005, 1, 5078}, {17, 1006, 2, 5078}, {18, 1007, 1, 5078}, {11, 1001, 1, 5079}, {13, 1003, 2, 5079}, {14, 1004, 2, 5079}, {16, 1006, 1, 5079}, {17, 1007, 2, 5079}, {18, 1008, 1, 5079}, {12, 999, 1, 5080}, {14, 1000, 2, 5080}, {15, 1001, 2, 5080}, {17, 1002, 1, 5080}, {18, 1003, 2, 5080}, {19, 1004, 1, 5080}, {12, 1000, 1, 5081}, {14, 1002, 2, 5081}, {15, 1003, 2, 5081}, {17, 1005, 1, 5081}, {18, 1006, 2, 5081}, {19, 1007, 1, 5081}, {12, 1001, 1, 5082}, {14, 1003, 2, 5082}, {15, 1004, 2, 5082}, {17, 1006, 1, 5082}, {18, 1007, 2, 5082}, {19, 1008, 1, 5082}, {10, 1073, 1, 5083}, {11, 1074, 2, 5083}, {12, 1075, 2, 5083}, {13, 1076, 1, 5083}, {14, 1077, 2, 5083}, {15, 1078, 1, 5083}, {10, 1074, 1, 5084}, {11, 1079, 2, 5084}, {12, 1080, 2, 5084}, {13, 1081, 1, 5084}, {14, 1082, 2, 5084}, {15, 1083, 1, 5084}, {10, 1075, 1, 5085}, {11, 1080, 2, 5085}, {12, 1084, 2, 5085}, {13, 1085, 1, 5085}, {14, 1086, 2, 5085}, {15, 1087, 1, 5085}, {10, 1076, 1, 5086}, {11, 1081, 2, 5086}, {12, 1085, 2, 5086}, {13, 1088, 1, 5086}, {14, 1089, 2, 5086}, {15, 1090, 1, 5086}, {10, 1077, 1, 5087}, {11, 1082, 2, 5087}, {12, 1086, 2, 5087}, {13, 1089, 1, 5087}, {14, 1091, 2, 5087}, {15, 1092, 1, 5087}, {10, 1078, 1, 5088}, {11, 1083, 2, 5088}, {12, 1087, 2, 5088}, {13, 1090, 1, 5088}, {14, 1092, 2, 5088}, {15, 1093, 1, 5088}, {11, 1073, 1, 5089}, {13, 1074, 2, 5089}, {14, 1075, 2, 5089}, {16, 1076, 1, 5089}, {17, 1077, 2, 5089}, {18, 1078, 1, 5089}, {11, 1074, 1, 5090}, {13, 1079, 2, 5090}, {14, 1080, 2, 5090}, {16, 1081, 1, 5090}, {17, 1082, 2, 5090}, {18, 1083, 1, 5090}, {11, 1075, 1, 5091}, {13, 1080, 2, 5091}, {14, 1084, 2, 5091}, {16, 1085, 1, 5091}, {17, 1086, 2, 5091}, {18, 1087, 1, 5091}, {11, 1076, 1, 5092}, {13, 1081, 2, 5092}, {14, 1085, 2, 5092}, {16, 1088, 1, 5092}, {17, 1089, 2, 5092}, {18, 1090, 1, 5092}, {11, 1077, 1, 5093}, {13, 1082, 2, 5093}, {14, 1086, 2, 5093}, {16, 1089, 1, 5093}, {17, 1091, 2, 5093}, {18, 1092, 1, 5093}, {11, 1078, 1, 5094}, {13, 1083, 2, 5094}, {14, 1087, 2, 5094}, {16, 1090, 1, 5094}, {17, 1092, 2, 5094}, {18, 1093, 1, 5094}, {12, 1073, 1, 5095}, {14, 1074, 2, 5095}, {15, 1075, 2, 5095}, {17, 1076, 1, 5095}, {18, 1077, 2, 5095}, {19, 1078, 1, 5095}, {12, 1074, 1, 5096}, {14, 1079, 2, 5096}, {15, 1080, 2, 5096}, {17, 1081, 1, 5096}, {18, 1082, 2, 5096}, {19, 1083, 1, 5096}, {12, 1075, 1, 5097}, {14, 1080, 2, 5097}, {15, 1084, 2, 5097}, {17, 1085, 1, 5097}, {18, 1086, 2, 5097}, {19, 1087, 1, 5097}, {12, 1076, 1, 5098}, {14, 1081, 2, 5098}, {15, 1085, 2, 5098}, {17, 1088, 1, 5098}, {18, 1089, 2, 5098}, {19, 1090, 1, 5098}, {12, 1077, 1, 5099}, {14, 1082, 2, 5099}, {15, 1086, 2, 5099}, {17, 1089, 1, 5099}, {18, 1091, 2, 5099}, {19, 1092, 1, 5099}, {12, 1078, 1, 5100}, {14, 1083, 2, 5100}, {15, 1087, 2, 5100}, {17, 1090, 1, 5100}, {18, 1092, 2, 5100}, {19, 1093, 1, 5100}, {10, 1376, 1, 5101}, {11, 1377, 2, 5101}, {12, 1378, 2, 5101}, {13, 1379, 1, 5101}, {14, 1380, 2, 5101}, {15, 1381, 1, 5101}, {10, 1382, 1, 5102}, {11, 1383, 2, 5102}, {12, 1384, 2, 5102}, {13, 1385, 1, 5102}, {14, 1386, 2, 5102}, {15, 1387, 1, 5102}, {10, 1388, 1, 5103}, {11, 1389, 2, 5103}, {12, 1390, 2, 5103}, {13, 1391, 1, 5103}, {14, 1392, 2, 5103}, {15, 1393, 1, 5103}, {11, 1376, 1, 5104}, {13, 1377, 2, 5104}, {14, 1378, 2, 5104}, {16, 1379, 1, 5104}, {17, 1380, 2, 5104}, {18, 1381, 1, 5104}, {11, 1382, 1, 5105}, {13, 1383, 2, 5105}, {14, 1384, 2, 5105}, {16,

1385, 1, 5105}, {17, 1386, 2, 5105}, {18, 1387, 1, 5105}, {11, 1388, 1, 5106}, {13, 1389, 2, 5106}, {14, 1390, 2, 5106}, {16, 1391, 1, 5106}, {17, 1392, 2, 5106}, {18, 1393, 1, 5106}, {12, 1376, 1, 5107}, {14, 1377, 2, 5107}, {15, 1378, 2, 5107}, {17, 1379, 1, 5107}, {18, 1380, 2, 5107}, {19, 1381, 1, 5107}, {12, 1382, 1, 5108}, {14, 1383, 2, 5108}, {15, 1384, 2, 5108}, {17, 1385, 1, 5108}, {18, 1386, 2, 5108}, {19, 1387, 1, 5108}, {12, 1388, 1, 5109}, {14, 1389, 2, 5109}, {15, 1390, 2, 5109}, {17, 1391, 1, 5109}, {18, 1392, 2, 5109}, {19, 1393, 1, 5109}, {10, 1536, 1, 5110}, {11, 1537, 2, 5110}, {12, 1538, 2, 5110}, {13, 1539, 1, 5110}, {14, 1540, 2, 5110}, {15, 1541, 1, 5110}, {11, 1536, 1, 5111}, {13, 1537, 2, 5111}, {14, 1538, 2, 5111}, {16, 1539, 1, 5111}, {17, 1540, 2, 5111}, {18, 1541, 1, 5111}, {12, 1536, 1, 5112}, {14, 1537, 2, 5112}, {15, 1538, 2, 5112}, {17, 1539, 1, 5112}, {18, 1540, 2, 5112}, {19, 1541, 1, 5112}, {10, 1542, 1, 5113}, {11, 1543, 2, 5113}, {12, 1544, 2, 5113}, {13, 1545, 1, 5113}, {14, 1546, 2, 5113}, {15, 1547, 1, 5113}, {10, 1543, 1, 5114}, {11, 1545, 2, 5114}, {12, 1546, 2, 5114}, {13, 1548, 1, 5114}, {14, 1549, 2, 5114}, {15, 1550, 1, 5114}, {10, 1544, 1, 5115}, {11, 1546, 2, 5115}, {12, 1547, 2, 5115}, {13, 1549, 1, 5115}, {14, 1550, 2, 5115}, {15, 1551, 1, 5115}, {11, 1542, 1, 5116}, {13, 1543, 2, 5116}, {14, 1544, 2, 5116}, {16, 1545, 1, 5116}, {17, 1546, 2, 5116}, {18, 1547, 1, 5116}, {11, 1543, 1, 5117}, {13, 1545, 2, 5117}, {14, 1546, 2, 5117}, {16, 1548, 1, 5117}, {17, 1549, 2, 5117}, {18, 1550, 1, 5117}, {11, 1544, 1, 5118}, {13, 1546, 2, 5118}, {14, 1547, 2, 5118}, {16, 1549, 1, 5118}, {17, 1550, 2, 5118}, {18, 1551, 1, 5118}, {12, 1542, 1, 5119}, {14, 1543, 2, 5119}, {15, 1544, 2, 5119}, {17, 1545, 1, 5119}, {18, 1546, 2, 5119}, {19, 1547, 1, 5119}, {12, 1543, 1, 5120}, {14, 1545, 2, 5120}, {15, 1546, 2, 5120}, {17, 1548, 1, 5120}, {18, 1549, 2, 5120}, {19, 1550, 1, 5120}, {12, 1544, 1, 5121}, {14, 1546, 2, 5121}, {15, 1547, 2, 5121}, {17, 1549, 1, 5121}, {18, 1550, 2, 5121}, {19, 1551, 1, 5121}, {10, 1552, 1, 5122}, {11, 1553, 2, 5122}, {12, 1554, 2, 5122}, {13, 1555, 1, 5122}, {14, 1556, 2, 5122}, {15, 1557, 1, 5122}, {10, 1553, 1, 5123}, {11, 1555, 2, 5123}, {12, 1556, 2, 5123}, {13, 1558, 1, 5123}, {14, 1559, 2, 5123}, {15, 1560, 1, 5123}, {10, 1554, 1, 5124}, {11, 1556, 2, 5124}, {12, 1557, 2, 5124}, {13, 1559, 1, 5124}, {14, 1560, 2, 5124}, {15, 1561, 1, 5124}, {10, 1555, 1, 5125}, {11, 1558, 2, 5125}, {12, 1559, 2, 5125}, {13, 1562, 1, 5125}, {14, 1563, 2, 5125}, {15, 1564, 1, 5125}, {10, 1556, 1, 5126}, {11, 1559, 2, 5126}, {12, 1560, 2, 5126}, {13, 1563, 1, 5126}, {14, 1564, 2, 5126}, {15, 1565, 1, 5126}, {10, 1557, 1, 5127}, {11, 1560, 2, 5127}, {12, 1561, 2, 5127}, {13, 1564, 1, 5127}, {14, 1565, 2, 5127}, {15, 1566, 1, 5127}, {11, 1552, 1, 5128}, {13, 1553, 2, 5128}, {14, 1554, 2, 5128}, {16, 1555, 1, 5128}, {17, 1556, 2, 5128}, {18, 1557, 1, 5128}, {11, 1553, 1, 5129}, {13, 1555, 2, 5129}, {14, 1556, 2, 5129}, {16, 1558, 1, 5129}, {17, 1559, 2, 5129}, {18, 1560, 1, 5129}, {11, 1554, 1, 5130}, {13, 1556, 2, 5130}, {14, 1557, 2, 5130}, {16, 1559, 1, 5130}, {17, 1560, 2, 5130}, {18, 1561, 1, 5130}, {11, 1555, 1, 5131}, {13, 1558, 2, 5131}, {14, 1559, 2, 5131}, {16, 1562, 1, 5131}, {17, 1563, 2, 5131}, {18, 1564, 1, 5131}, {11, 1556, 1, 5132}, {13, 1559, 2, 5132}, {14, 1560, 2, 5132}, {16, 1563, 1, 5132}, {17, 1564, 2, 5132}, {18, 1565, 1, 5132}, {11, 1557, 1, 5133}, {13, 1560, 2, 5133}, {14, 1561, 2, 5133}, {16, 1564, 1, 5133}, {17, 1565, 2, 5133}, {18, 1566, 1, 5133}, {12, 1552, 1, 5134}, {14, 1553, 2, 5134}, {15, 1554, 2, 5134}, {17, 1555, 1, 5134}, {18, 1556, 2, 5134}, {19, 1557, 1, 5134}, {12, 1553, 1, 5135}, {14, 1555, 2, 5135}, {15, 1556, 2, 5135}, {17, 1558, 1, 5135}, {18, 1559, 2, 5135}, {19, 1560, 1, 5135}, {12, 1554, 1, 5136}, {14, 1556, 2, 5136}, {15, 1557, 2, 5136}, {17, 1559, 1, 5136}, {18, 1560, 2, 5136}, {19, 1561, 1, 5136}, {12, 1555, 1, 5137}, {14, 1558, 2, 5137}, {15, 1559, 2, 5137}, {17, 1562, 1, 5137}, {18, 1563, 2, 5137}, {19, 1564, 1, 5137}, {12, 1556, 1, 5138}, {14, 1559, 2, 5138}, {15, 1560, 2, 5138}, {17, 1563, 1, 5138}, {18, 1564, 2, 5138}, {19, 1565, 1, 5138}, {12, 1557, 1, 5139}, {14, 1560, 2, 5139}, {15, 1561, 2, 5139}, {17, 1564, 1, 5139}, {18, 1565, 2, 5139}, {19, 1566, 1, 5139}, {10, 1793, 1, 5140}, {11, 1794, 2, 5140}, {12, 1795, 2, 5140}, {13, 1796, 1, 5140}, {14, 1797, 2, 5140}, {15, 1798, 1, 5140}, {10, 1794, 1, 5141}, {11, 1799, 2, 5141}, {12, 1800, 2, 5141}, {13, 1801, 1, 5141}, {14, 1802, 2, 5141}, {15, 1803, 1, 5141}, {10, 1795, 1, 5142}, {11, 1800, 2, 5142}, {12, 1804, 2, 5142}, {13, 1805, 1, 5142}, {14, 1806, 2, 5142}, {15, 1807, 1, 5142}, {10, 1796, 1, 5143}, {11, 1801, 2, 5143}, {12, 1805, 2, 5143}, {13, 1808, 1, 5143}, {14, 1809, 2, 5143}, {15, 1810, 1, 5143}, {10, 1797, 1, 5144}, {11, 1802, 2, 5144}, {12, 1806, 2, 5144}, {13, 1809, 1, 5144}, {14, 1811, 2, 5144}, {15, 1812, 1, 5144}, {10, 1798, 1, 5145}, {11, 1803, 2, 5145}, {12, 1807, 2, 5145}, {13, 1810, 1, 5145}, {14, 1812, 2, 5145}, {15, 1813, 1, 5145}, {11, 1793, 1, 5146}, {13, 1794, 2, 5146}, {14, 1795, 2, 5146}, {16, 1796, 1, 5146}, {17, 1797, 2, 5146}, {18, 1798, 1, 5146}, {11, 1794, 1, 5147}, {13, 1799, 2, 5147}, {14, 1800, 2, 5147}, {16, 1801, 1, 5147}, {17, 1802, 2, 5147}, {18, 1803, 1, 5147}, {11, 1795, 1, 5148}, {13, 1800, 2, 5148}, {14, 1804, 2, 5148}, {16, 1805, 1, 5148}, {17, 1806, 2, 5148}, {18, 1807, 1, 5148}, {11, 1796, 1, 5149}, {13, 1801, 2, 5149}, {14, 1805, 2, 5149}, {16, 1808, 1, 5149}, {17, 1809, 2, 5149}, {18, 1810, 1, 5149}, {11, 1797, 1, 5150}, {13, 1802, 2, 5150}, {14, 1806, 2, 5150}, {16, 1809, 1, 5150}, {17, 1811, 2, 5150}, {18, 1812, 1, 5150}, {11, 1798, 1, 5151}, {13, 1803, 2, 5151}, {14, 1807, 2, 5151}, {16, 1810, 1, 5151}, {17, 1812, 2, 5151}, {18, 1813, 1, 5151}, {12, 1793, 1, 5152}, {14, 1794, 2, 5152}, {15, 1795, 2, 5152}, {17, 1796, 1, 5152}, {18, 1797, 2, 5152}, {19, 1798, 1, 5152}, {12, 1794, 1, 5153}, {14, 1799, 2, 5153}, {15, 1800, 2, 5153}, {17, 1801, 1, 5153}, {18, 1802, 2, 5153}, {19, 1803, 1, 5153}, {12, 1795, 1, 5154}, {14, 1800, 2, 5154}, {15, 1804,

2, 5154}, {17, 1805, 1, 5154}, {18, 1806, 2, 5154}, {19, 1807, 1, 5154}, {12, 1796, 1, 5155}, {14, 1801, 2, 5155}, {15, 1805, 2, 5155}, {17, 1808, 1, 5155}, {18, 1809, 2, 5155}, {19, 1810, 1, 5155}, {12, 1797, 1, 5156}, {14, 1802, 2, 5156}, {15, 1806, 2, 5156}, {17, 1809, 1, 5156}, {18, 1811, 2, 5156}, {19, 1812, 1, 5156}, {12, 1798, 1, 5157}, {14, 1803, 2, 5157}, {15, 1807, 2, 5157}, {17, 1810, 1, 5157}, {18, 1812, 2, 5157}, {19, 1813, 1, 5157}, {10, 1970, 1, 5158}, {11, 1971, 2, 5158}, {12, 1972, 2, 5158}, {13, 1973, 1, 5158}, {14, 1974, 2, 5158}, {15, 1975, 1, 5158}, {10, 1976, 1, 5159}, {11, 1977, 2, 5159}, {12, 1978, 2, 5159}, {13, 1979, 1, 5159}, {14, 1980, 2, 5159}, {15, 1981, 1, 5159}, {10, 1982, 1, 5160}, {11, 1983, 2, 5160}, {12, 1984, 2, 5160}, {13, 1985, 1, 5160}, {14, 1986, 2, 5160}, {15, 1987, 1, 5160}, {11, 1970, 1, 5161}, {13, 1971, 2, 5161}, {14, 1972, 2, 5161}, {16, 1973, 1, 5161}, {17, 1974, 2, 5161}, {18, 1975, 1, 5161}, {11, 1976, 1, 5162}, {13, 1977, 2, 5162}, {14, 1978, 2, 5162}, {16, 1979, 1, 5162}, {17, 1980, 2, 5162}, {18, 1981, 1, 5162}, {11, 1982, 1, 5163}, {13, 1983, 2, 5163}, {14, 1984, 2, 5163}, {16, 1985, 1, 5163}, {17, 1986, 2, 5163}, {18, 1987, 1, 5163}, {12, 1970, 1, 5164}, {14, 1971, 2, 5164}, {15, 1972, 2, 5164}, {17, 1973, 1, 5164}, {18, 1974, 2, 5164}, {19, 1975, 1, 5164}, {12, 1976, 1, 5165}, {14, 1977, 2, 5165}, {15, 1978, 2, 5165}, {17, 1979, 1, 5165}, {18, 1980, 2, 5165}, {19, 1981, 1, 5165}, {12, 1982, 1, 5166}, {14, 1983, 2, 5166}, {15, 1984, 2, 5166}, {17, 1985, 1, 5166}, {18, 1986, 2, 5166}, {19, 1987, 1, 5166}, {10, 2067, 1, 5167}, {11, 2068, 2, 5167}, {12, 2069, 2, 5167}, {13, 2070, 1, 5167}, {14, 2071, 2, 5167}, {15, 2072, 1, 5167}, {11, 2067, 1, 5168}, {13, 2068, 2, 5168}, {14, 2069, 2, 5168}, {16, 2070, 1, 5168}, {17, 2071, 2, 5168}, {18, 2072, 1, 5168}, {12, 2067, 1, 5169}, {14, 2068, 2, 5169}, {15, 2069, 2, 5169}, {17, 2070, 1, 5169}, {18, 2071, 2, 5169}, {19, 2072, 1, 5169}, {10, 2073, 1, 5170}, {11, 2074, 2, 5170}, {12, 2075, 2, 5170}, {13, 2076, 1, 5170}, {14, 2077, 2, 5170}, {15, 2078, 1, 5170}, {10, 2074, 1, 5171}, {11, 2076, 2, 5171}, {12, 2077, 2, 5171}, {13, 2079, 1, 5171}, {14, 2080, 2, 5171}, {15, 2081, 1, 5171}, {10, 2075, 1, 5172}, {11, 2077, 2, 5172}, {12, 2078, 2, 5172}, {13, 2080, 1, 5172}, {14, 2081, 2, 5172}, {15, 2082, 1, 5172}, {11, 2073, 1, 5173}, {13, 2074, 2, 5173}, {14, 2075, 2, 5173}, {16, 2076, 1, 5173}, {17, 2077, 2, 5173}, {18, 2078, 1, 5173}, {11, 2074, 1, 5174}, {13, 2076, 2, 5174}, {14, 2077, 2, 5174}, {16, 2079, 1, 5174}, {17, 2080, 2, 5174}, {18, 2081, 1, 5174}, {11, 2075, 1, 5175}, {13, 2077, 2, 5175}, {14, 2078, 2, 5175}, {16, 2080, 1, 5175}, {17, 2081, 2, 5175}, {18, 2082, 1, 5175}, {12, 2073, 1, 5176}, {14, 2074, 2, 5176}, {15, 2075, 2, 5176}, {17, 2076, 1, 5176}, {18, 2077, 2, 5176}, {19, 2078, 1, 5176}, {12, 2074, 1, 5177}, {14, 2076, 2, 5177}, {15, 2077, 2, 5177}, {17, 2079, 1, 5177}, {18, 2080, 2, 5177}, {19, 2081, 1, 5177}, {12, 2075, 1, 5178}, {14, 2077, 2, 5178}, {15, 2078, 2, 5178}, {17, 2080, 1, 5178}, {18, 2081, 2, 5178}, {19, 2082, 1, 5178}, {10, 2174, 1, 5179}, {11, 2175, 2, 5179}, {12, 2176, 2, 5179}, {13, 2177, 1, 5179}, {14, 2178, 2, 5179}, {15, 2179, 1, 5179}, {10, 2175, 1, 5180}, {11, 2180, 2, 5180}, {12, 2181, 2, 5180}, {13, 2182, 1, 5180}, {14, 2183, 2, 5180}, {15, 2184, 1, 5180}, {10, 2176, 1, 5181}, {11, 2181, 2, 5181}, {12, 2185, 2, 5181}, {13, 2186, 1, 5181}, {14, 2187, 2, 5181}, {15, 2188, 1, 5181}, {10, 2177, 1, 5182}, {11, 2182, 2, 5182}, {12, 2186, 2, 5182}, {13, 2189, 1, 5182}, {14, 2190, 2, 5182}, {15, 2191, 1, 5182}, {10, 2178, 1, 5183}, {11, 2183, 2, 5183}, {12, 2187, 2, 5183}, {13, 2190, 1, 5183}, {14, 2192, 2, 5183}, {15, 2193, 1, 5183}, {10, 2179, 1, 5184}, {11, 2184, 2, 5184}, {12, 2188, 2, 5184}, {13, 2191, 1, 5184}, {14, 2193, 2, 5184}, {15, 2194, 1, 5184}, {11, 2174, 1, 5185}, {13, 2175, 2, 5185}, {14, 2176, 2, 5185}, {16, 2177, 1, 5185}, {17, 2178, 2, 5185}, {18, 2179, 1, 5185}, {11, 2175, 1, 5186}, {13, 2180, 2, 5186}, {14, 2181, 2, 5186}, {16, 2182, 1, 5186}, {17, 2183, 2, 5186}, {18, 2184, 1, 5186}, {11, 2176, 1, 5187}, {13, 2181, 2, 5187}, {14, 2185, 2, 5187}, {16, 2186, 1, 5187}, {17, 2187, 2, 5187}, {18, 2188, 1, 5187}, {11, 2177, 1, 5188}, {13, 2182, 2, 5188}, {14, 2186, 2, 5188}, {16, 2189, 1, 5188}, {17, 2190, 2, 5188}, {18, 2191, 1, 5188}, {11, 2178, 1, 5189}, {13, 2183, 2, 5189}, {14, 2187, 2, 5189}, {16, 2190, 1, 5189}, {17, 2192, 2, 5189}, {18, 2193, 1, 5189}, {11, 2179, 1, 5190}, {13, 2184, 2, 5190}, {14, 2188, 2, 5190}, {16, 2191, 1, 5190}, {17, 2193, 2, 5190}, {18, 2194, 1, 5190}, {12, 2174, 1, 5191}, {14, 2175, 2, 5191}, {15, 2176, 2, 5191}, {17, 2177, 1, 5191}, {18, 2178, 2, 5191}, {19, 2179, 1, 5191}, {12, 2175, 1, 5192}, {14, 2180, 2, 5192}, {15, 2181, 2, 5192}, {17, 2182, 1, 5192}, {18, 2183, 2, 5192}, {19, 2184, 1, 5192}, {12, 2176, 1, 5193}, {14, 2181, 2, 5193}, {15, 2185, 2, 5193}, {17, 2186, 1, 5193}, {18, 2187, 2, 5193}, {19, 2188, 1, 5193}, {12, 2177, 1, 5194}, {14, 2182, 2, 5194}, {15, 2186, 2, 5194}, {17, 2189, 1, 5194}, {18, 2190, 2, 5194}, {19, 2191, 1, 5194}, {12, 2178, 1, 5195}, {14, 2183, 2, 5195}, {15, 2187, 2, 5195}, {17, 2190, 1, 5195}, {18, 2192, 2, 5195}, {19, 2193, 1, 5195}, {12, 2179, 1, 5196}, {14, 2184, 2, 5196}, {15, 2188, 2, 5196}, {17, 2191, 1, 5196}, {18, 2193, 2, 5196}, {19, 2194, 1, 5196}, {10, 2351, 1, 5197}, {11, 2352, 2, 5197}, {12, 2353, 2, 5197}, {13, 2354, 1, 5197}, {14, 2355, 2, 5197}, {15, 2356, 1, 5197}, {10, 2357, 1, 5198}, {11, 2358, 2, 5198}, {12, 2359, 2, 5198}, {13, 2360, 1, 5198}, {14, 2361, 2, 5198}, {15, 2362, 1, 5198}, {10, 2363, 1, 5199}, {11, 2364, 2, 5199}, {12, 2365, 2, 5199}, {13, 2366, 1, 5199}, {14, 2367, 2, 5199}, {15, 2368, 1, 5199}, {11, 2351, 1, 5200}, {13, 2352, 2, 5200}, {14, 2353, 2, 5200}, {16, 2354, 1, 5200}, {17, 2355, 2, 5200}, {18, 2356, 1, 5200}, {11, 2357, 1, 5201}, {13, 2358, 2, 5201}, {14, 2359, 2, 5201}, {16, 2360, 1, 5201}, {17, 2361, 2, 5201}, {18, 2362, 1, 5201}, {11, 2363, 1, 5202}, {13, 2364, 2, 5202}, {14, 2365, 2, 5202}, {16, 2366, 1, 5202}, {17, 2367, 2, 5202}, {18, 2368, 1, 5202}, {12, 2351, 1, 5203}, {14, 2352, 2,

5203}, {15, 2353, 2, 5203}, {17, 2354, 1, 5203}, {18, 2355, 2, 5203}, {19, 2356, 1, 5203}, {12, 2357, 1, 5204}, {14, 2358, 2, 5204}, {15, 2359, 2, 5204}, {17, 2360, 1, 5204}, {18, 2361, 2, 5204}, {19, 2362, 1, 5204}, {12, 2363, 1, 5205}, {14, 2364, 2, 5205}, {15, 2365, 2, 5205}, {17, 2366, 1, 5205}, {18, 2367, 2, 5205}, {19, 2368, 1, 5205}, {10, 2448, 1, 5206}, {11, 2449, 2, 5206}, {12, 2450, 2, 5206}, {13, 2451, 1, 5206}, {14, 2452, 2, 5206}, {15, 2453, 1, 5206}, {11, 2448, 1, 5207}, {13, 2449, 2, 5207}, {14, 2450, 2, 5207}, {16, 2451, 1, 5207}, {17, 2452, 2, 5207}, {18, 2453, 1, 5207}, {12, 2448, 1, 5208}, {14, 2449, 2, 5208}, {15, 2450, 2, 5208}, {17, 2451, 1, 5208}, {18, 2452, 2, 5208}, {19, 2453, 1, 5208}, {10, 999, 1, 5209}, {11, 1000, 3, 5209}, {12, 1001, 3, 5209}, {13, 1002, 3, 5209}, {14, 1003, 6, 5209}, {15, 1004, 3, 5209}, {16, 1005, 1, 5209}, {17, 1006, 3, 5209}, {18, 1007, 3, 5209}, {19, 1008, 1, 5209}, {10, 1009, 1, 5210}, {11, 1010, 3, 5210}, {12, 1011, 3, 5210}, {13, 1012, 3, 5210}, {14, 1013, 6, 5210}, {15, 1014, 3, 5210}, {16, 1015, 1, 5210}, {17, 1016, 3, 5210}, {18, 1017, 3, 5210}, {19, 1018, 1, 5210}, {10, 1010, 1, 5211}, {11, 1012, 3, 5211}, {12, 1013, 3, 5211}, {13, 1015, 3, 5211}, {14, 1016, 6, 5211}, {15, 1017, 3, 5211}, {16, 1019, 1, 5211}, {17, 1020, 3, 5211}, {18, 1021, 3, 5211}, {19, 1022, 1, 5211}, {10, 1011, 1, 5212}, {11, 1013, 3, 5212}, {12, 1014, 3, 5212}, {13, 1016, 3, 5212}, {14, 1017, 6, 5212}, {15, 1018, 3, 5212}, {16, 1020, 1, 5212}, {17, 1021, 3, 5212}, {18, 1022, 3, 5212}, {19, 1023, 1, 5212}, {10, 1024, 1, 5213}, {11, 1025, 3, 5213}, {12, 1026, 3, 5213}, {13, 1027, 3, 5213}, {14, 1028, 6, 5213}, {15, 1029, 3, 5213}, {16, 1030, 1, 5213}, {17, 1031, 3, 5213}, {18, 1032, 3, 5213}, {19, 1033, 1, 5213}, {10, 1025, 1, 5214}, {11, 1027, 3, 5214}, {12, 1028, 3, 5214}, {13, 1030, 3, 5214}, {14, 1031, 6, 5214}, {15, 1032, 3, 5214}, {16, 1034, 1, 5214}, {17, 1035, 3, 5214}, {18, 1036, 3, 5214}, {19, 1037, 1, 5214}, {10, 1026, 1, 5215}, {11, 1028, 3, 5215}, {12, 1029, 3, 5215}, {13, 1031, 3, 5215}, {14, 1032, 6, 5215}, {15, 1033, 3, 5215}, {16, 1035, 1, 5215}, {17, 1036, 3, 5215}, {18, 1037, 3, 5215}, {19, 1038, 1, 5215}, {10, 1027, 1, 5216}, {11, 1030, 3, 5216}, {12, 1031, 3, 5216}, {13, 1034, 3, 5216}, {14, 1035, 6, 5216}, {15, 1036, 3, 5216}, {16, 1039, 1, 5216}, {17, 1040, 3, 5216}, {18, 1041, 3, 5216}, {19, 1042, 1, 5216}, {10, 1028, 1, 5217}, {11, 1031, 3, 5217}, {12, 1032, 3, 5217}, {13, 1035, 3, 5217}, {14, 1036, 6, 5217}, {15, 1037, 3, 5217}, {16, 1040, 1, 5217}, {17, 1041, 3, 5217}, {18, 1042, 3, 5217}, {19, 1043, 1, 5217}, {10, 1029, 1, 5218}, {11, 1032, 3, 5218}, {12, 1033, 3, 5218}, {13, 1036, 3, 5218}, {14, 1037, 6, 5218}, {15, 1038, 3, 5218}, {16, 1041, 1, 5218}, {17, 1042, 3, 5218}, {18, 1043, 3, 5218}, {19, 1044, 1, 5218}, {10, 1045, 1, 5219}, {11, 1046, 3, 5219}, {12, 1047, 3, 5219}, {13, 1048, 3, 5219}, {14, 1049, 6, 5219}, {15, 1050, 3, 5219}, {16, 1051, 1, 5219}, {17, 1052, 3, 5219}, {18, 1053, 3, 5219}, {19, 1054, 1, 5219}, {10, 1046, 1, 5220}, {11, 1048, 3, 5220}, {12, 1049, 3, 5220}, {13, 1051, 3, 5220}, {14, 1052, 6, 5220}, {15, 1053, 3, 5220}, {16, 1055, 1, 5220}, {17, 1056, 3, 5220}, {18, 1057, 3, 5220}, {19, 1058, 1, 5220}, {10, 1047, 1, 5221}, {11, 1049, 3, 5221}, {12, 1050, 3, 5221}, {13, 1052, 3, 5221}, {14, 1053, 6, 5221}, {15, 1054, 3, 5221}, {16, 1056, 1, 5221}, {17, 1057, 3, 5221}, {18, 1058, 3, 5221}, {19, 1059, 1, 5221}, {10, 1048, 1, 5222}, {11, 1051, 3, 5222}, {12, 1052, 3, 5222}, {13, 1055, 3, 5222}, {14, 1056, 6, 5222}, {15, 1057, 3, 5222}, {16, 1060, 1, 5222}, {17, 1061, 3, 5222}, {18, 1062, 3, 5222}, {19, 1063, 1, 5222}, {10, 1049, 1, 5223}, {11, 1052, 3, 5223}, {12, 1053, 3, 5223}, {13, 1056, 3, 5223}, {14, 1057, 6, 5223}, {15, 1058, 3, 5223}, {16, 1061, 1, 5223}, {17, 1062, 3, 5223}, {18, 1063, 3, 5223}, {19, 1064, 1, 5223}, {10, 1050, 1, 5224}, {11, 1053, 3, 5224}, {12, 1054, 3, 5224}, {13, 1057, 3, 5224}, {14, 1058, 6, 5224}, {15, 1059, 3, 5224}, {16, 1062, 1, 5224}, {17, 1063, 3, 5224}, {18, 1064, 3, 5224}, {19, 1065, 1, 5224}, {10, 1051, 1, 5225}, {11, 1055, 3, 5225}, {12, 1056, 3, 5225}, {13, 1060, 3, 5225}, {14, 1061, 6, 5225}, {15, 1062, 3, 5225}, {16, 1066, 1, 5225}, {17, 1067, 3, 5225}, {18, 1068, 3, 5225}, {19, 1069, 1, 5225}, {10, 1052, 1, 5226}, {11, 1056, 3, 5226}, {12, 1057, 3, 5226}, {13, 1061, 3, 5226}, {14, 1062, 6, 5226}, {15, 1063, 3, 5226}, {16, 1067, 1, 5226}, {17, 1068, 3, 5226}, {18, 1069, 3, 5226}, {19, 1070, 1, 5226}, {10, 1053, 1, 5227}, {11, 1057, 3, 5227}, {12, 1058, 3, 5227}, {13, 1062, 3, 5227}, {14, 1063, 6, 5227}, {15, 1064, 3, 5227}, {16, 1068, 1, 5227}, {17, 1069, 3, 5227}, {18, 1070, 3, 5227}, {19, 1071, 1, 5227}, {10, 1054, 1, 5228}, {11, 1058, 3, 5228}, {12, 1059, 3, 5228}, {13, 1063, 3, 5228}, {14, 1064, 6, 5228}, {15, 1065, 3, 5228}, {16, 1069, 1, 5228}, {17, 1070, 3, 5228}, {18, 1071, 3, 5228}, {19, 1072, 1, 5228}, {20, 993, 1, 5229}, {21, 994, 2, 5229}, {22, 995, 2, 5229}, {23, 996, 1, 5229}, {24, 997, 2, 5229}, {25, 998, 1, 5229}, {21, 993, 1, 5230}, {23, 994, 2, 5230}, {24, 995, 2, 5230}, {26, 996, 1, 5230}, {27, 997, 2, 5230}, {28, 998, 1, 5230}, {22, 993, 1, 5231}, {24, 994, 2, 5231}, {25, 995, 2, 5231}, {27, 996, 1, 5231}, {28, 997, 2, 5231}, {29, 998, 1, 5231}, {23, 993, 1, 5232}, {26, 994, 2, 5232}, {27, 995, 2, 5232}, {30, 996, 1, 5232}, {31, 997, 2, 5232}, {32, 998, 1, 5232}, {24, 993, 1, 5233}, {27, 994, 2, 5233}, {28, 995, 2, 5233}, {31, 996, 1, 5233}, {32, 997, 2, 5233}, {33, 998, 1, 5233}, {25, 993, 1, 5234}, {28, 994, 2, 5234}, {29, 995, 2, 5234}, {32, 996, 1, 5234}, {33, 997, 2, 5234}, {34, 998, 1, 5234}, {10, 1394, 1, 5235}, {11, 1395, 3, 5235}, {12, 1396, 3, 5235}, {13, 1397, 3, 5235}, {14, 1398, 6, 5235}, {15, 1399, 3, 5235}, {16, 1400, 1, 5235}, {17, 1401, 3, 5235}, {18, 1402, 3, 5235}, {19, 1403, 1, 5235}, {10, 1404, 1, 5236}, {11, 1405, 3, 5236}, {12, 1406, 3, 5236}, {13, 1407, 3, 5236}, {14, 1408, 6, 5236}, {15, 1409, 3, 5236}, {16, 1410, 1, 5236}, {17, 1411, 3, 5236}, {18, 1412, 3, 5236}, {19, 1413, 1, 5236}, {10, 1414, 1, 5237}, {11, 1415, 3, 5237}, {12, 1416, 3, 5237}, {13, 1417, 3, 5237}, {14, 1418, 6, 5237},

{15, 1419, 3, 5237}, {16, 1420, 1, 5237}, {17, 1421, 3, 5237}, {18, 1422, 3, 5237}, {19,
1423, 1, 5237}, {10, 1424, 1, 5238}, {11, 1425, 3, 5238}, {12, 1426, 3, 5238}, {13, 1427,
3, 5238}, {14, 1428, 6, 5238}, {15, 1429, 3, 5238}, {16, 1430, 1, 5238}, {17, 1431, 3,
5238}, {18, 1432, 3, 5238}, {19, 1433, 1, 5238}, {10, 1425, 1, 5239}, {11, 1427, 3, 5239},
{12, 1428, 3, 5239}, {13, 1430, 3, 5239}, {14, 1431, 6, 5239}, {15, 1432, 3, 5239}, {16,
1434, 1, 5239}, {17, 1435, 3, 5239}, {18, 1436, 3, 5239}, {19, 1437, 1, 5239}, {10, 1426,
1, 5240}, {11, 1428, 3, 5240}, {12, 1429, 3, 5240}, {13, 1431, 3, 5240}, {14, 1432, 6,
5240}, {15, 1433, 3, 5240}, {16, 1435, 1, 5240}, {17, 1436, 3, 5240}, {18, 1437, 3, 5240},
{19, 1438, 1, 5240}, {10, 1439, 1, 5241}, {11, 1440, 3, 5241}, {12, 1441, 3, 5241}, {13,
1442, 3, 5241}, {14, 1443, 6, 5241}, {15, 1444, 3, 5241}, {16, 1445, 1, 5241}, {17, 1446,
3, 5241}, {18, 1447, 3, 5241}, {19, 1448, 1, 5241}, {10, 1440, 1, 5242}, {11, 1442, 3,
5242}, {12, 1443, 3, 5242}, {13, 1445, 3, 5242}, {14, 1446, 6, 5242}, {15, 1447, 3, 5242},
{16, 1449, 1, 5242}, {17, 1450, 3, 5242}, {18, 1451, 3, 5242}, {19, 1452, 1, 5242}, {10,
1441, 1, 5243}, {11, 1443, 3, 5243}, {12, 1444, 3, 5243}, {13, 1446, 3, 5243}, {14, 1447,
6, 5243}, {15, 1448, 3, 5243}, {16, 1450, 1, 5243}, {17, 1451, 3, 5243}, {18, 1452, 3,
5243}, {19, 1453, 1, 5243}, {10, 1454, 1, 5244}, {11, 1455, 3, 5244}, {12, 1456, 3, 5244},
{13, 1457, 3, 5244}, {14, 1458, 6, 5244}, {15, 1459, 3, 5244}, {16, 1460, 1, 5244}, {17,
1461, 3, 5244}, {18, 1462, 3, 5244}, {19, 1463, 1, 5244}, {10, 1455, 1, 5245}, {11, 1457,
3, 5245}, {12, 1458, 3, 5245}, {13, 1460, 3, 5245}, {14, 1461, 6, 5245}, {15, 1462, 3,
5245}, {16, 1464, 1, 5245}, {17, 1465, 3, 5245}, {18, 1466, 3, 5245}, {19, 1467, 1, 5245},
{10, 1456, 1, 5246}, {11, 1458, 3, 5246}, {12, 1459, 3, 5246}, {13, 1461, 3, 5246}, {14,
1462, 6, 5246}, {15, 1463, 3, 5246}, {16, 1465, 1, 5246}, {17, 1466, 3, 5246}, {18, 1467,
3, 5246}, {19, 1468, 1, 5246}, {10, 1542, 1, 5247}, {11, 1543, 3, 5247}, {12, 1544, 3,
5247}, {13, 1545, 3, 5247}, {14, 1546, 6, 5247}, {15, 1547, 3, 5247}, {16, 1548, 1, 5247},
{17, 1549, 3, 5247}, {18, 1550, 3, 5247}, {19, 1551, 1, 5247}, {10, 1552, 1, 5248}, {11,
1553, 3, 5248}, {12, 1554, 3, 5248}, {13, 1555, 3, 5248}, {14, 1556, 6, 5248}, {15, 1557,
3, 5248}, {16, 1558, 1, 5248}, {17, 1559, 3, 5248}, {18, 1560, 3, 5248}, {19, 1561, 1,
5248}, {10, 1553, 1, 5249}, {11, 1555, 3, 5249}, {12, 1556, 3, 5249}, {13, 1558, 3, 5249},
{14, 1559, 6, 5249}, {15, 1560, 3, 5249}, {16, 1562, 1, 5249}, {17, 1563, 3, 5249}, {18,
1564, 3, 5249}, {19, 1565, 1, 5249}, {10, 1554, 1, 5250}, {11, 1556, 3, 5250}, {12, 1557,
3, 5250}, {13, 1559, 3, 5250}, {14, 1560, 6, 5250}, {15, 1561, 3, 5250}, {16, 1563, 1,
5250}, {17, 1564, 3, 5250}, {18, 1565, 3, 5250}, {19, 1566, 1, 5250}, {10, 1567, 1, 5251},
{11, 1568, 3, 5251}, {12, 1569, 3, 5251}, {13, 1570, 3, 5251}, {14, 1571, 6, 5251}, {15,
1572, 3, 5251}, {16, 1573, 1, 5251}, {17, 1574, 3, 5251}, {18, 1575, 3, 5251}, {19, 1576,
1, 5251}, {10, 1568, 1, 5252}, {11, 1570, 3, 5252}, {12, 1571, 3, 5252}, {13, 1573, 3,
5252}, {14, 1574, 6, 5252}, {15, 1575, 3, 5252}, {16, 1577, 1, 5252}, {17, 1578, 3, 5252},
{18, 1579, 3, 5252}, {19, 1580, 1, 5252}, {10, 1569, 1, 5253}, {11, 1571, 3, 5253}, {12,
1572, 3, 5253}, {13, 1574, 3, 5253}, {14, 1575, 6, 5253}, {15, 1576, 3, 5253}, {16, 1578,
1, 5253}, {17, 1579, 3, 5253}, {18, 1580, 3, 5253}, {19, 1581, 1, 5253}, {10, 1570, 1,
5254}, {11, 1573, 3, 5254}, {12, 1574, 3, 5254}, {13, 1577, 3, 5254}, {14, 1578, 6, 5254},
{15, 1579, 3, 5254}, {16, 1582, 1, 5254}, {17, 1583, 3, 5254}, {18, 1584, 3, 5254}, {19,
1585, 1, 5254}, {10, 1571, 1, 5255}, {11, 1574, 3, 5255}, {12, 1575, 3, 5255}, {13, 1578,
3, 5255}, {14, 1579, 6, 5255}, {15, 1580, 3, 5255}, {16, 1583, 1, 5255}, {17, 1584, 3,
5255}, {18, 1585, 3, 5255}, {19, 1586, 1, 5255}, {10, 1572, 1, 5256}, {11, 1575, 3, 5256},
{12, 1576, 3, 5256}, {13, 1579, 3, 5256}, {14, 1580, 6, 5256}, {15, 1581, 3, 5256}, {16,
1584, 1, 5256}, {17, 1585, 3, 5256}, {18, 1586, 3, 5256}, {19, 1587, 1, 5256}, {10, 1814,
1, 5257}, {11, 1815, 3, 5257}, {12, 1816, 3, 5257}, {13, 1817, 3, 5257}, {14, 1818, 6,
5257}, {15, 1819, 3, 5257}, {16, 1820, 1, 5257}, {17, 1821, 3, 5257}, {18, 1822, 3, 5257},
{19, 1823, 1, 5257}, {10, 1824, 1, 5258}, {11, 1825, 3, 5258}, {12, 1826, 3, 5258}, {13,
1827, 3, 5258}, {14, 1828, 6, 5258}, {15, 1829, 3, 5258}, {16, 1830, 1, 5258}, {17, 1831,
3, 5258}, {18, 1832, 3, 5258}, {19, 1833, 1, 5258}, {10, 1834, 1, 5259}, {11, 1835, 3,
5259}, {12, 1836, 3, 5259}, {13, 1837, 3, 5259}, {14, 1838, 6, 5259}, {15, 1839, 3, 5259},
{16, 1840, 1, 5259}, {17, 1841, 3, 5259}, {18, 1842, 3, 5259}, {19, 1843, 1, 5259}, {10,
1844, 1, 5260}, {11, 1845, 3, 5260}, {12, 1846, 3, 5260}, {13, 1847, 3, 5260}, {14, 1848,
6, 5260}, {15, 1849, 3, 5260}, {16, 1850, 1, 5260}, {17, 1851, 3, 5260}, {18, 1852, 3,
5260}, {19, 1853, 1, 5260}, {10, 1854, 1, 5261}, {11, 1855, 3, 5261}, {12, 1856, 3, 5261},
{13, 1857, 3, 5261}, {14, 1858, 6, 5261}, {15, 1859, 3, 5261}, {16, 1860, 1, 5261}, {17,
1861, 3, 5261}, {18, 1862, 3, 5261}, {19, 1863, 1, 5261}, {10, 1864, 1, 5262}, {11, 1865,
3, 5262}, {12, 1866, 3, 5262}, {13, 1867, 3, 5262}, {14, 1868, 6, 5262}, {15, 1869, 3,
5262}, {16, 1870, 1, 5262}, {17, 1871, 3, 5262}, {18, 1872, 3, 5262}, {19, 1873, 1, 5262},
{10, 1988, 1, 5263}, {11, 1989, 3, 5263}, {12, 1990, 3, 5263}, {13, 1991, 3, 5263}, {14,
1992, 6, 5263}, {15, 1993, 3, 5263}, {16, 1994, 1, 5263}, {17, 1995, 3, 5263}, {18, 1996,
3, 5263}, {19, 1997, 1, 5263}, {10, 1998, 1, 5264}, {11, 1999, 3, 5264}, {12, 2000, 3,
5264}, {13, 2001, 3, 5264}, {14, 2002, 6, 5264}, {15, 2003, 3, 5264}, {16, 2004, 1, 5264},
{17, 2005, 3, 5264}, {18, 2006, 3, 5264}, {19, 2007, 1, 5264}, {10, 2008, 1, 5265}, {11,
2009, 3, 5265}, {12, 2010, 3, 5265}, {13, 2011, 3, 5265}, {14, 2012, 6, 5265}, {15, 2013,
3, 5265}, {16, 2014, 1, 5265}, {17, 2015, 3, 5265}, {18, 2016, 3, 5265}, {19, 2017, 1,
5265}, {10, 2018, 1, 5266}, {11, 2019, 3, 5266}, {12, 2020, 3, 5266}, {13, 2021, 3, 5266},
{14, 2022, 6, 5266}, {15, 2023, 3, 5266}, {16, 2024, 1, 5266}, {17, 2025, 3, 5266}, {18,

2026, 3, 5266}, {19, 2027, 1, 5266}, {10, 2019, 1, 5267}, {11, 2021, 3, 5267}, {12, 2022, 3, 5267}, {13, 2024, 3, 5267}, {14, 2025, 6, 5267}, {15, 2026, 3, 5267}, {16, 2028, 1, 5267}, {17, 2029, 3, 5267}, {18, 2030, 3, 5267}, {19, 2031, 1, 5267}, {10, 2020, 1, 5268}, {11, 2022, 3, 5268}, {12, 2023, 3, 5268}, {13, 2025, 3, 5268}, {14, 2026, 6, 5268}, {15, 2027, 3, 5268}, {16, 2029, 1, 5268}, {17, 2030, 3, 5268}, {18, 2031, 3, 5268}, {19, 2032, 1, 5268}, {10, 2033, 1, 5269}, {11, 2034, 3, 5269}, {12, 2035, 3, 5269}, {13, 2036, 3, 5269}, {14, 2037, 6, 5269}, {15, 2038, 3, 5269}, {16, 2039, 1, 5269}, {17, 2040, 3, 5269}, {18, 2041, 3, 5269}, {19, 2042, 1, 5269}, {10, 2034, 1, 5270}, {11, 2036, 3, 5270}, {12, 2037, 3, 5270}, {13, 2039, 3, 5270}, {14, 2040, 6, 5270}, {15, 2041, 3, 5270}, {16, 2043, 1, 5270}, {17, 2044, 3, 5270}, {18, 2045, 3, 5270}, {19, 2046, 1, 5270}, {10, 2035, 1, 5271}, {11, 2037, 3, 5271}, {12, 2038, 3, 5271}, {13, 2040, 3, 5271}, {14, 2041, 6, 5271}, {15, 2042, 3, 5271}, {16, 2044, 1, 5271}, {17, 2045, 3, 5271}, {18, 2046, 3, 5271}, {19, 2047, 1, 5271}, {10, 2048, 1, 5272}, {11, 2049, 3, 5272}, {12, 2050, 3, 5272}, {13, 2051, 3, 5272}, {14, 2052, 6, 5272}, {15, 2053, 3, 5272}, {16, 2054, 1, 5272}, {17, 2055, 3, 5272}, {18, 2056, 3, 5272}, {19, 2057, 1, 5272}, {10, 2049, 1, 5273}, {11, 2051, 3, 5273}, {12, 2052, 3, 5273}, {13, 2054, 3, 5273}, {14, 2055, 6, 5273}, {15, 2056, 3, 5273}, {16, 2058, 1, 5273}, {17, 2059, 3, 5273}, {18, 2060, 3, 5273}, {19, 2061, 1, 5273}, {10, 2050, 1, 5274}, {11, 2052, 3, 5274}, {12, 2053, 3, 5274}, {13, 2055, 3, 5274}, {14, 2056, 6, 5274}, {15, 2057, 3, 5274}, {16, 2059, 1, 5274}, {17, 2060, 3, 5274}, {18, 2061, 3, 5274}, {19, 2062, 1, 5274}, {10, 2073, 1, 5275}, {11, 2074, 3, 5275}, {12, 2075, 3, 5275}, {13, 2076, 3, 5275}, {14, 2077, 6, 5275}, {15, 2078, 3, 5275}, {16, 2079, 1, 5275}, {17, 2080, 3, 5275}, {18, 2081, 3, 5275}, {19, 2082, 1, 5275}, {10, 2083, 1, 5276}, {11, 2084, 3, 5276}, {12, 2085, 3, 5276}, {13, 2086, 3, 5276}, {14, 2087, 6, 5276}, {15, 2088, 3, 5276}, {16, 2089, 1, 5276}, {17, 2090, 3, 5276}, {18, 2091, 3, 5276}, {19, 2092, 1, 5276}, {10, 2084, 1, 5277}, {11, 2086, 3, 5277}, {12, 2087, 3, 5277}, {13, 2089, 3, 5277}, {14, 2090, 6, 5277}, {15, 2091, 3, 5277}, {16, 2093, 1, 5277}, {17, 2094, 3, 5277}, {18, 2095, 3, 5277}, {19, 2096, 1, 5277}, {10, 2085, 1, 5278}, {11, 2087, 3, 5278}, {12, 2088, 3, 5278}, {13, 2090, 3, 5278}, {14, 2091, 6, 5278}, {15, 2092, 3, 5278}, {16, 2094, 1, 5278}, {17, 2095, 3, 5278}, {18, 2096, 3, 5278}, {19, 2097, 1, 5278}, {10, 2119, 1, 5279}, {11, 2120, 3, 5279}, {12, 2121, 3, 5279}, {13, 2122, 3, 5279}, {14, 2123, 6, 5279}, {15, 2124, 3, 5279}, {16, 2125, 1, 5279}, {17, 2126, 3, 5279}, {18, 2127, 3, 5279}, {19, 2128, 1, 5279}, {10, 2120, 1, 5280}, {11, 2129, 3, 5280}, {12, 2130, 3, 5280}, {13, 2131, 3, 5280}, {14, 2132, 6, 5280}, {15, 2133, 3, 5280}, {16, 2134, 1, 5280}, {17, 2135, 3, 5280}, {18, 2136, 3, 5280}, {19, 2137, 1, 5280}, {10, 2121, 1, 5281}, {11, 2130, 3, 5281}, {12, 2138, 3, 5281}, {13, 2139, 3, 5281}, {14, 2140, 6, 5281}, {15, 2141, 3, 5281}, {16, 2142, 1, 5281}, {17, 2143, 3, 5281}, {18, 2144, 3, 5281}, {19, 2145, 1, 5281}, {10, 2122, 1, 5282}, {11, 2131, 3, 5282}, {12, 2139, 3, 5282}, {13, 2146, 3, 5282}, {14, 2147, 6, 5282}, {15, 2148, 3, 5282}, {16, 2149, 1, 5282}, {17, 2150, 3, 5282}, {18, 2151, 3, 5282}, {19, 2152, 1, 5282}, {10, 2123, 1, 5283}, {11, 2132, 3, 5283}, {12, 2140, 3, 5283}, {13, 2147, 3, 5283}, {14, 2153, 6, 5283}, {15, 2154, 3, 5283}, {16, 2155, 1, 5283}, {17, 2156, 3, 5283}, {18, 2157, 3, 5283}, {19, 2158, 1, 5283}, {10, 2124, 1, 5284}, {11, 2133, 3, 5284}, {12, 2141, 3, 5284}, {13, 2148, 3, 5284}, {14, 2154, 6, 5284}, {15, 2159, 3, 5284}, {16, 2160, 1, 5284}, {17, 2161, 3, 5284}, {18, 2162, 3, 5284}, {19, 2163, 1, 5284}, {10, 2125, 1, 5285}, {11, 2134, 3, 5285}, {12, 2142, 3, 5285}, {13, 2149, 3, 5285}, {14, 2155, 6, 5285}, {15, 2160, 3, 5285}, {16, 2164, 1, 5285}, {17, 2165, 3, 5285}, {18, 2166, 3, 5285}, {19, 2167, 1, 5285}, {10, 2126, 1, 5286}, {11, 2135, 3, 5286}, {12, 2143, 3, 5286}, {13, 2150, 3, 5286}, {14, 2156, 6, 5286}, {15, 2161, 3, 5286}, {16, 2165, 1, 5286}, {17, 2168, 3, 5286}, {18, 2169, 3, 5286}, {19, 2170, 1, 5286}, {10, 2127, 1, 5287}, {11, 2136, 3, 5287}, {12, 2144, 3, 5287}, {13, 2151, 3, 5287}, {14, 2157, 6, 5287}, {15, 2162, 3, 5287}, {16, 2166, 1, 5287}, {17, 2169, 3, 5287}, {18, 2171, 3, 5287}, {19, 2172, 1, 5287}, {10, 2128, 1, 5288}, {11, 2137, 3, 5288}, {12, 2145, 3, 5288}, {13, 2152, 3, 5288}, {14, 2158, 6, 5288}, {15, 2163, 3, 5288}, {16, 2167, 1, 5288}, {17, 2170, 3, 5288}, {18, 2172, 3, 5288}, {19, 2173, 1, 5288}, {10, 2195, 1, 5289}, {11, 2196, 3, 5289}, {12, 2197, 3, 5289}, {13, 2198, 3, 5289}, {14, 2199, 6, 5289}, {15, 2200, 3, 5289}, {16, 2201, 1, 5289}, {17, 2202, 3, 5289}, {18, 2203, 3, 5289}, {19, 2204, 1, 5289}, {10, 2205, 1, 5290}, {11, 2206, 3, 5290}, {12, 2207, 3, 5290}, {13, 2208, 3, 5290}, {14, 2209, 6, 5290}, {15, 2210, 3, 5290}, {16, 2211, 1, 5290}, {17, 2212, 3, 5290}, {18, 2213, 3, 5290}, {19, 2214, 1, 5290}, {10, 2215, 1, 5291}, {11, 2216, 3, 5291}, {12, 2217, 3, 5291}, {13, 2218, 3, 5291}, {14, 2219, 6, 5291}, {15, 2220, 3, 5291}, {16, 2221, 1, 5291}, {17, 2222, 3, 5291}, {18, 2223, 3, 5291}, {19, 2224, 1, 5291}, {10, 2225, 1, 5292}, {11, 2226, 3, 5292}, {12, 2227, 3, 5292}, {13, 2228, 3, 5292}, {14, 2229, 6, 5292}, {15, 2230, 3, 5292}, {16, 2231, 1, 5292}, {17, 2232, 3, 5292}, {18, 2233, 3, 5292}, {19, 2234, 1, 5292}, {10, 2235, 1, 5293}, {11, 2236, 3, 5293}, {12, 2237, 3, 5293}, {13, 2238, 3, 5293}, {14, 2239, 6, 5293}, {15, 2240, 3, 5293}, {16, 2241, 1, 5293}, {17, 2242, 3, 5293}, {18, 2243, 3, 5293}, {19, 2244, 1, 5293}, {10, 2245, 1, 5294}, {11, 2246, 3, 5294}, {12, 2247, 3, 5294}, {13, 2248, 3, 5294}, {14, 2249, 6, 5294}, {15, 2250, 3, 5294}, {16, 2251, 1, 5294}, {17, 2252, 3, 5294}, {18, 2253, 3, 5294}, {19, 2254, 1, 5294}, {10, 2369, 1, 5295}, {11, 2370, 3, 5295}, {12, 2371, 3, 5295}, {13, 2372, 3, 5295}, {14, 2373, 6, 5295}, {15, 2374, 3, 5295}, {16, 2375, 1, 5295}, {17, 2376, 3, 5295}, {18, 2377, 3, 5295}, {19, 2378, 1, 5295}, {10, 2379, 1, 5296}, {11, 2380,

3, 5296}, {12, 2381, 3, 5296}, {13, 2382, 3, 5296}, {14, 2383, 6, 5296}, {15, 2384, 3, 5296}, {16, 2385, 1, 5296}, {17, 2386, 3, 5296}, {18, 2387, 3, 5296}, {19, 2388, 1, 5296}, {10, 2389, 1, 5297}, {11, 2390, 3, 5297}, {12, 2391, 3, 5297}, {13, 2392, 3, 5297}, {14, 2393, 6, 5297}, {15, 2394, 3, 5297}, {16, 2395, 1, 5297}, {17, 2396, 3, 5297}, {18, 2397, 3, 5297}, {19, 2398, 1, 5297}, {10, 2454, 1, 5298}, {11, 2455, 3, 5298}, {12, 2456, 3, 5298}, {13, 2457, 3, 5298}, {14, 2458, 6, 5298}, {15, 2459, 3, 5298}, {16, 2460, 1, 5298}, {17, 2461, 3, 5298}, {18, 2462, 3, 5298}, {19, 2463, 1, 5298}, {10, 2679, 1, 5299}, {11, 2680, 3, 5299}, {12, 2681, 3, 5299}, {13, 2682, 3, 5299}, {14, 2683, 6, 5299}, {15, 2684, 3, 5299}, {16, 2685, 1, 5299}, {17, 2686, 3, 5299}, {18, 2687, 3, 5299}, {19, 2688, 1, 5299}, {10, 806, 1, 5300}, {11, 807, 2, 5300}, {12, 808, 2, 5300}, {13, 812, 1, 5300}, {14, 813, 2, 5300}, {15, 817, 1, 5300}, {10, 807, 1, 5301}, {11, 809, 1, 5301}, {11, 812, 1, 5301}, {12, 810, 1, 5301}, {12, 813, 1, 5301}, {13, 814, 1, 5301}, {14, 815, 1, 5301}, {14, 818, 1, 5301}, {15, 819, 1, 5301}, {10, 808, 1, 5302}, {11, 810, 1, 5302}, {11, 813, 1, 5302}, {12, 811, 1, 5302}, {12, 817, 1, 5302}, {13, 815, 1, 5302}, {14, 816, 1, 5302}, {14, 819, 1, 5302}, {15, 820, 1, 5302}, {10, 812, 1, 5303}, {11, 814, 2, 5303}, {12, 815, 2, 5303}, {13, 821, 1, 5303}, {14, 822, 2, 5303}, {15, 824, 1, 5303}, {10, 813, 1, 5304}, {11, 815, 1, 5304}, {11, 818, 1, 5304}, {12, 816, 1, 5304}, {12, 819, 1, 5304}, {13, 822, 1, 5304}, {14, 823, 1, 5304}, {14, 824, 1, 5304}, {15, 825, 1, 5304}, {10, 817, 1, 5305}, {11, 819, 2, 5305}, {12, 820, 2, 5305}, {13, 824, 1, 5305}, {14, 825, 2, 5305}, {15, 826, 1, 5305}, {11, 806, 1, 5306}, {13, 807, 2, 5306}, {14, 808, 2, 5306}, {16, 812, 1, 5306}, {17, 813, 2, 5306}, {18, 817, 1, 5306}, {11, 807, 1, 5307}, {13, 809, 1, 5307}, {13, 812, 1, 5307}, {14, 810, 1, 5307}, {14, 813, 1, 5307}, {16, 814, 1, 5307}, {17, 815, 1, 5307}, {17, 818, 1, 5307}, {18, 819, 1, 5307}, {11, 808, 1, 5308}, {13, 810, 1, 5308}, {13, 813, 1, 5308}, {14, 811, 1, 5308}, {14, 817, 1, 5308}, {16, 815, 1, 5308}, {17, 816, 1, 5308}, {17, 819, 1, 5308}, {18, 820, 1, 5308}, {11, 812, 1, 5309}, {13, 814, 2, 5309}, {14, 815, 2, 5309}, {16, 821, 1, 5309}, {17, 822, 2, 5309}, {18, 824, 1, 5309}, {11, 813, 1, 5310}, {13, 815, 1, 5310}, {13, 818, 1, 5310}, {14, 816, 1, 5310}, {14, 819, 1, 5310}, {16, 822, 1, 5310}, {17, 823, 1, 5310}, {17, 824, 1, 5310}, {18, 825, 1, 5310}, {11, 817, 1, 5311}, {13, 819, 2, 5311}, {14, 820, 2, 5311}, {16, 824, 1, 5311}, {17, 825, 2, 5311}, {18, 826, 1, 5311}, {12, 806, 1, 5312}, {14, 807, 2, 5312}, {15, 808, 2, 5312}, {17, 812, 1, 5312}, {18, 813, 2, 5312}, {19, 817, 1, 5312}, {12, 807, 1, 5313}, {14, 809, 1, 5313}, {14, 812, 1, 5313}, {15, 810, 1, 5313}, {15, 813, 1, 5313}, {17, 814, 1, 5313}, {18, 815, 1, 5313}, {18, 818, 1, 5313}, {19, 819, 1, 5313}, {12, 808, 1, 5314}, {14, 810, 1, 5314}, {14, 813, 1, 5314}, {15, 811, 1, 5314}, {15, 817, 1, 5314}, {17, 815, 1, 5314}, {18, 816, 1, 5314}, {18, 819, 1, 5314}, {19, 820, 1, 5314}, {12, 812, 1, 5315}, {14, 814, 2, 5315}, {15, 815, 2, 5315}, {17, 821, 1, 5315}, {18, 822, 2, 5315}, {19, 824, 1, 5315}, {12, 813, 1, 5316}, {14, 815, 1, 5316}, {14, 818, 1, 5316}, {15, 816, 1, 5316}, {15, 819, 1, 5316}, {17, 822, 1, 5316}, {18, 823, 1, 5316}, {18, 824, 1, 5316}, {19, 825, 1, 5316}, {12, 817, 1, 5317}, {14, 819, 2, 5317}, {15, 820, 2, 5317}, {17, 824, 1, 5317}, {18, 825, 2, 5317}, {19, 826, 1, 5317}, {10, 827, 1, 5318}, {11, 828, 2, 5318}, {12, 829, 2, 5318}, {13, 830, 1, 5318}, {14, 831, 2, 5318}, {15, 832, 1, 5318}, {11, 827, 1, 5319}, {13, 828, 2, 5319}, {14, 829, 2, 5319}, {16, 830, 1, 5319}, {17, 831, 2, 5319}, {18, 832, 1, 5319}, {12, 827, 1, 5320}, {14, 828, 2, 5320}, {15, 829, 2, 5320}, {17, 830, 1, 5320}, {18, 831, 2, 5320}, {19, 832, 1, 5320}, {10, 833, 1, 5321}, {11, 834, 1, 5321}, {11, 839, 1, 5321}, {12, 835, 1, 5321}, {12, 845, 1, 5321}, {13, 840, 1, 5321}, {14, 841, 1, 5321}, {14, 846, 1, 5321}, {15, 847, 1, 5321}, {10, 834, 1, 5322}, {11, 836, 1, 5322}, {11, 840, 1, 5322}, {12, 837, 1, 5322}, {12, 846, 1, 5322}, {13, 842, 1, 5322}, {14, 843, 1, 5322}, {14, 848, 1, 5322}, {15, 849, 1, 5322}, {10, 835, 1, 5323}, {11, 837, 1, 5323}, {11, 841, 1, 5323}, {12, 838, 1, 5323}, {12, 847, 1, 5323}, {13, 843, 1, 5323}, {14, 844, 1, 5323}, {14, 849, 1, 5323}, {15, 850, 1, 5323}, {11, 833, 1, 5324}, {13, 834, 1, 5324}, {13, 839, 1, 5324}, {14, 835, 1, 5324}, {14, 845, 1, 5324}, {16, 840, 1, 5324}, {17, 841, 1, 5324}, {17, 846, 1, 5324}, {18, 847, 1, 5324}, {11, 834, 1, 5325}, {13, 836, 1, 5325}, {13, 840, 1, 5325}, {14, 837, 1, 5325}, {14, 846, 1, 5325}, {16, 842, 1, 5325}, {17, 843, 1, 5325}, {17, 848, 1, 5325}, {18, 849, 1, 5325}, {11, 835, 1, 5326}, {13, 837, 1, 5326}, {13, 841, 1, 5326}, {14, 838, 1, 5326}, {14, 847, 1, 5326}, {16, 843, 1, 5326}, {17, 844, 1, 5326}, {17, 849, 1, 5326}, {18, 850, 1, 5326}, {12, 833, 1, 5327}, {14, 834, 1, 5327}, {14, 839, 1, 5327}, {15, 835, 1, 5327}, {15, 845, 1, 5327}, {17, 840, 1, 5327}, {18, 841, 1, 5327}, {18, 846, 1, 5327}, {19, 847, 1, 5327}, {12, 834, 1, 5328}, {14, 836, 1, 5328}, {14, 840, 1, 5328}, {15, 837, 1, 5328}, {15, 846, 1, 5328}, {17, 842, 1, 5328}, {18, 843, 1, 5328}, {18, 848, 1, 5328}, {19, 849, 1, 5328}, {12, 835, 1, 5329}, {14, 837, 1, 5329}, {14, 841, 1, 5329}, {15, 838, 1, 5329}, {15, 847, 1, 5329}, {17, 843, 1, 5329}, {18, 844, 1, 5329}, {18, 849, 1, 5329}, {19, 850, 1, 5329}, {10, 851, 1, 5330}, {11, 852, 1, 5330}, {11, 861, 1, 5330}, {12, 853, 1, 5330}, {12, 871, 1, 5330}, {13, 862, 1, 5330}, {14, 863, 1, 5330}, {14, 872, 1, 5330}, {15, 873, 1, 5330}, {10, 852, 1, 5331}, {11, 854, 1, 5331}, {11, 862, 1, 5331}, {12, 855, 1, 5331}, {12, 872, 1, 5331}, {13, 864, 1, 5331}, {14, 865, 1, 5331}, {14, 874, 1, 5331}, {15, 875, 1, 5331}, {10, 853, 1, 5332}, {11, 855, 1, 5332}, {11, 863, 1, 5332}, {12, 856, 1, 5332}, {12, 873, 1, 5332}, {13, 865, 1, 5332}, {14, 866, 1, 5332}, {14, 875, 1, 5332}, {15, 876, 1, 5332}, {10, 854, 1, 5333}, {11, 857, 1, 5333}, {11, 864, 1, 5333}, {12, 858, 1, 5333}, {12, 874, 1, 5333}, {13, 867, 1, 5333}, {14, 868, 1, 5333}, {14, 877, 1, 5333}, {15, 878,

1, 5333}, {10, 855, 1, 5334}, {11, 858, 1, 5334}, {11, 865, 1, 5334}, {12, 859, 1, 5334}, {12, 875, 1, 5334}, {13, 868, 1, 5334}, {14, 869, 1, 5334}, {14, 878, 1, 5334}, {15, 879, 1, 5334}, {10, 856, 1, 5335}, {11, 859, 1, 5335}, {11, 866, 1, 5335}, {12, 860, 1, 5335}, {12, 876, 1, 5335}, {13, 869, 1, 5335}, {14, 870, 1, 5335}, {14, 879, 1, 5335}, {15, 880, 1, 5335}, {11, 851, 1, 5336}, {13, 852, 1, 5336}, {13, 861, 1, 5336}, {14, 853, 1, 5336}, {14, 871, 1, 5336}, {16, 862, 1, 5336}, {17, 863, 1, 5336}, {17, 872, 1, 5336}, {18, 873, 1, 5336}, {11, 852, 1, 5337}, {13, 854, 1, 5337}, {13, 862, 1, 5337}, {14, 855, 1, 5337}, {14, 872, 1, 5337}, {16, 864, 1, 5337}, {17, 865, 1, 5337}, {17, 874, 1, 5337}, {18, 875, 1, 5337}, {11, 853, 1, 5338}, {13, 855, 1, 5338}, {13, 863, 1, 5338}, {14, 856, 1, 5338}, {14, 873, 1, 5338}, {16, 865, 1, 5338}, {17, 866, 1, 5338}, {17, 875, 1, 5338}, {18, 876, 1, 5338}, {11, 854, 1, 5339}, {13, 857, 1, 5339}, {13, 864, 1, 5339}, {14, 858, 1, 5339}, {14, 874, 1, 5339}, {16, 867, 1, 5339}, {17, 868, 1, 5339}, {17, 877, 1, 5339}, {18, 878, 1, 5339}, {11, 855, 1, 5340}, {13, 858, 1, 5340}, {13, 865, 1, 5340}, {14, 859, 1, 5340}, {14, 875, 1, 5340}, {16, 868, 1, 5340}, {17, 869, 1, 5340}, {17, 878, 1, 5340}, {18, 879, 1, 5340}, {11, 856, 1, 5341}, {13, 859, 1, 5341}, {13, 866, 1, 5341}, {14, 860, 1, 5341}, {14, 876, 1, 5341}, {16, 869, 1, 5341}, {17, 870, 1, 5341}, {17, 879, 1, 5341}, {18, 880, 1, 5341}, {12, 851, 1, 5342}, {14, 852, 1, 5342}, {14, 861, 1, 5342}, {15, 853, 1, 5342}, {15, 871, 1, 5342}, {17, 862, 1, 5342}, {18, 863, 1, 5342}, {18, 872, 1, 5342}, {19, 873, 1, 5342}, {12, 852, 1, 5343}, {14, 854, 1, 5343}, {14, 862, 1, 5343}, {15, 855, 1, 5343}, {15, 872, 1, 5343}, {17, 864, 1, 5343}, {18, 865, 1, 5343}, {18, 874, 1, 5343}, {19, 875, 1, 5343}, {12, 853, 1, 5344}, {14, 855, 1, 5344}, {14, 863, 1, 5344}, {15, 856, 1, 5344}, {15, 873, 1, 5344}, {17, 865, 1, 5344}, {18, 866, 1, 5344}, {18, 875, 1, 5344}, {19, 876, 1, 5344}, {12, 854, 1, 5345}, {14, 857, 1, 5345}, {14, 864, 1, 5345}, {15, 858, 1, 5345}, {15, 874, 1, 5345}, {17, 867, 1, 5345}, {18, 868, 1, 5345}, {18, 877, 1, 5345}, {19, 878, 1, 5345}, {12, 855, 1, 5346}, {14, 858, 1, 5346}, {14, 865, 1, 5346}, {15, 859, 1, 5346}, {15, 875, 1, 5346}, {17, 868, 1, 5346}, {18, 869, 1, 5346}, {18, 878, 1, 5346}, {19, 879, 1, 5346}, {12, 856, 1, 5347}, {14, 859, 1, 5347}, {14, 866, 1, 5347}, {15, 860, 1, 5347}, {15, 876, 1, 5347}, {17, 869, 1, 5347}, {18, 870, 1, 5347}, {18, 879, 1, 5347}, {19, 880, 1, 5347}, {10, 1073, 1, 5348}, {11, 1074, 2, 5348}, {12, 1075, 2, 5348}, {13, 1079, 1, 5348}, {14, 1080, 2, 5348}, {15, 1084, 1, 5348}, {10, 1074, 1, 5349}, {11, 1076, 1, 5349}, {11, 1079, 1, 5349}, {12, 1077, 1, 5349}, {12, 1080, 1, 5349}, {13, 1081, 1, 5349}, {14, 1082, 1, 5349}, {14, 1085, 1, 5349}, {15, 1086, 1, 5349}, {10, 1075, 1, 5350}, {11, 1077, 1, 5350}, {11, 1080, 1, 5350}, {12, 1078, 1, 5350}, {12, 1084, 1, 5350}, {13, 1082, 1, 5350}, {14, 1083, 1, 5350}, {14, 1086, 1, 5350}, {15, 1087, 1, 5350}, {10, 1079, 1, 5351}, {11, 1081, 2, 5351}, {12, 1082, 2, 5351}, {13, 1088, 1, 5351}, {14, 1089, 2, 5351}, {15, 1091, 1, 5351}, {10, 1080, 1, 5352}, {11, 1082, 1, 5352}, {11, 1085, 1, 5352}, {12, 1083, 1, 5352}, {12, 1086, 1, 5352}, {13, 1089, 1, 5352}, {14, 1090, 1, 5352}, {14, 1091, 1, 5352}, {15, 1092, 1, 5352}, {10, 1084, 1, 5353}, {11, 1086, 2, 5353}, {12, 1087, 2, 5353}, {13, 1091, 1, 5353}, {14, 1092, 2, 5353}, {15, 1093, 1, 5353}, {11, 1073, 1, 5354}, {13, 1074, 2, 5354}, {14, 1075, 2, 5354}, {16, 1079, 1, 5354}, {17, 1080, 2, 5354}, {18, 1084, 1, 5354}, {11, 1074, 1, 5355}, {13, 1076, 1, 5355}, {13, 1079, 1, 5355}, {14, 1077, 1, 5355}, {14, 1080, 1, 5355}, {16, 1081, 1, 5355}, {17, 1082, 1, 5355}, {17, 1085, 1, 5355}, {18, 1086, 1, 5355}, {11, 1075, 1, 5356}, {13, 1077, 1, 5356}, {13, 1080, 1, 5356}, {14, 1078, 1, 5356}, {14, 1084, 1, 5356}, {16, 1082, 1, 5356}, {17, 1083, 1, 5356}, {17, 1086, 1, 5356}, {18, 1087, 1, 5356}, {11, 1079, 1, 5357}, {13, 1081, 2, 5357}, {14, 1082, 2, 5357}, {16, 1088, 1, 5357}, {17, 1089, 2, 5357}, {18, 1091, 1, 5357}, {11, 1080, 1, 5358}, {13, 1082, 1, 5358}, {13, 1085, 1, 5358}, {14, 1083, 1, 5358}, {14, 1086, 1, 5358}, {16, 1089, 1, 5358}, {17, 1090, 1, 5358}, {17, 1091, 1, 5358}, {18, 1092, 1, 5358}, {11, 1084, 1, 5359}, {13, 1086, 2, 5359}, {14, 1087, 2, 5359}, {16, 1091, 1, 5359}, {17, 1092, 2, 5359}, {18, 1093, 1, 5359}, {12, 1073, 1, 5360}, {14, 1074, 2, 5360}, {15, 1075, 2, 5360}, {17, 1079, 1, 5360}, {18, 1080, 2, 5360}, {19, 1084, 1, 5360}, {12, 1074, 1, 5361}, {14, 1076, 1, 5361}, {14, 1079, 1, 5361}, {15, 1077, 1, 5361}, {15, 1080, 1, 5361}, {17, 1081, 1, 5361}, {18, 1082, 1, 5361}, {18, 1085, 1, 5361}, {19, 1086, 1, 5361}, {12, 1075, 1, 5362}, {14, 1077, 1, 5362}, {14, 1080, 1, 5362}, {15, 1078, 1, 5362}, {15, 1084, 1, 5362}, {17, 1082, 1, 5362}, {18, 1083, 1, 5362}, {18, 1086, 1, 5362}, {19, 1087, 1, 5362}, {12, 1079, 1, 5363}, {14, 1081, 2, 5363}, {15, 1082, 2, 5363}, {17, 1088, 1, 5363}, {18, 1089, 2, 5363}, {19, 1091, 1, 5363}, {12, 1080, 1, 5364}, {14, 1082, 1, 5364}, {14, 1085, 1, 5364}, {15, 1083, 1, 5364}, {15, 1086, 1, 5364}, {17, 1089, 1, 5364}, {18, 1090, 1, 5364}, {18, 1091, 1, 5364}, {19, 1092, 1, 5364}, {12, 1084, 1, 5365}, {14, 1086, 2, 5365}, {15, 1087, 2, 5365}, {17, 1091, 1, 5365}, {18, 1092, 2, 5365}, {19, 1093, 1, 5365}, {10, 1370, 1, 5366}, {11, 1371, 2, 5366}, {12, 1372, 2, 5366}, {13, 1373, 1, 5366}, {14, 1374, 2, 5366}, {15, 1375, 1, 5366}, {11, 1370, 1, 5367}, {13, 1371, 2, 5367}, {14, 1372, 2, 5367}, {16, 1373, 1, 5367}, {17, 1374, 2, 5367}, {18, 1375, 1, 5367}, {12, 1370, 1, 5368}, {14, 1371, 2, 5368}, {15, 1372, 2, 5368}, {17, 1373, 1, 5368}, {18, 1374, 2, 5368}, {19, 1375, 1, 5368}, {10, 1376, 1, 5369}, {11, 1377, 1, 5369}, {11, 1382, 1, 5369}, {12, 1378, 1, 5369}, {12, 1388, 1, 5369}, {13, 1383, 1, 5369}, {14, 1384, 1, 5369}, {14, 1389, 1, 5369}, {15, 1390, 1, 5369}, {10, 1377, 1, 5370}, {11, 1379, 1, 5370}, {11, 1383, 1, 5370}, {12, 1380, 1, 5370}, {12, 1389, 1, 5370}, {13, 1385, 1, 5370}, {14, 1386, 1, 5370}, {14, 1391, 1, 5370}, {15, 1392, 1, 5370}, {10, 1378, 1, 5371}, {11, 1380, 1, 5371}, {11, 1384, 1,

5371}, {12, 1381, 1, 5371}, {12, 1390, 1, 5371}, {13, 1386, 1, 5371}, {14, 1387, 1, 5371},
{14, 1392, 1, 5371}, {15, 1393, 1, 5371}, {11, 1376, 1, 5372}, {13, 1377, 1, 5372}, {13,
1382, 1, 5372}, {14, 1378, 1, 5372}, {14, 1388, 1, 5372}, {16, 1383, 1, 5372}, {17, 1384,
1, 5372}, {17, 1389, 1, 5372}, {18, 1390, 1, 5372}, {11, 1377, 1, 5373}, {13, 1379, 1,
5373}, {13, 1383, 1, 5373}, {14, 1380, 1, 5373}, {14, 1389, 1, 5373}, {16, 1385, 1, 5373},
{17, 1386, 1, 5373}, {17, 1391, 1, 5373}, {18, 1392, 1, 5373}, {11, 1378, 1, 5374}, {13,
1380, 1, 5374}, {13, 1384, 1, 5374}, {14, 1381, 1, 5374}, {14, 1390, 1, 5374}, {16, 1386,
1, 5374}, {17, 1387, 1, 5374}, {17, 1392, 1, 5374}, {18, 1393, 1, 5374}, {12, 1376, 1,
5375}, {14, 1377, 1, 5375}, {14, 1382, 1, 5375}, {15, 1378, 1, 5375}, {15, 1388, 1, 5375},
{17, 1383, 1, 5375}, {18, 1384, 1, 5375}, {18, 1389, 1, 5375}, {19, 1390, 1, 5375}, {12,
1377, 1, 5376}, {14, 1379, 1, 5376}, {14, 1383, 1, 5376}, {15, 1380, 1, 5376}, {15, 1389,
1, 5376}, {17, 1385, 1, 5376}, {18, 1386, 1, 5376}, {18, 1391, 1, 5376}, {19, 1392, 1,
5376}, {12, 1378, 1, 5377}, {14, 1380, 1, 5377}, {14, 1384, 1, 5377}, {15, 1381, 1, 5377},
{15, 1390, 1, 5377}, {17, 1386, 1, 5377}, {18, 1387, 1, 5377}, {18, 1392, 1, 5377}, {19,
1393, 1, 5377}, {10, 1394, 1, 5378}, {11, 1395, 1, 5378}, {11, 1404, 1, 5378}, {12, 1396,
1, 5378}, {12, 1414, 1, 5378}, {13, 1405, 1, 5378}, {14, 1406, 1, 5378}, {14, 1415, 1,
5378}, {15, 1416, 1, 5378}, {10, 1395, 1, 5379}, {11, 1397, 1, 5379}, {11, 1405, 1, 5379},
{12, 1398, 1, 5379}, {12, 1415, 1, 5379}, {13, 1407, 1, 5379}, {14, 1408, 1, 5379}, {14,
1417, 1, 5379}, {15, 1418, 1, 5379}, {10, 1396, 1, 5380}, {11, 1398, 1, 5380}, {11, 1406,
1, 5380}, {12, 1399, 1, 5380}, {12, 1416, 1, 5380}, {13, 1408, 1, 5380}, {14, 1409, 1,
5380}, {14, 1418, 1, 5380}, {15, 1419, 1, 5380}, {10, 1397, 1, 5381}, {11, 1400, 1, 5381},
{11, 1407, 1, 5381}, {12, 1401, 1, 5381}, {12, 1417, 1, 5381}, {13, 1410, 1, 5381}, {14,
1411, 1, 5381}, {14, 1420, 1, 5381}, {15, 1421, 1, 5381}, {10, 1398, 1, 5382}, {11, 1401,
1, 5382}, {11, 1408, 1, 5382}, {12, 1402, 1, 5382}, {12, 1418, 1, 5382}, {13, 1411, 1,
5382}, {14, 1412, 1, 5382}, {14, 1421, 1, 5382}, {15, 1422, 1, 5382}, {10, 1399, 1, 5383},
{11, 1402, 1, 5383}, {11, 1409, 1, 5383}, {12, 1403, 1, 5383}, {12, 1419, 1, 5383}, {13,
1412, 1, 5383}, {14, 1413, 1, 5383}, {14, 1422, 1, 5383}, {15, 1423, 1, 5383}, {11, 1394,
1, 5384}, {13, 1395, 1, 5384}, {13, 1404, 1, 5384}, {14, 1396, 1, 5384}, {14, 1414, 1,
5384}, {16, 1405, 1, 5384}, {17, 1406, 1, 5384}, {17, 1415, 1, 5384}, {18, 1416, 1, 5384},
{11, 1395, 1, 5385}, {13, 1397, 1, 5385}, {13, 1405, 1, 5385}, {14, 1398, 1, 5385}, {14,
1415, 1, 5385}, {16, 1407, 1, 5385}, {17, 1408, 1, 5385}, {17, 1417, 1, 5385}, {18, 1418,
1, 5385}, {11, 1396, 1, 5386}, {13, 1398, 1, 5386}, {13, 1406, 1, 5386}, {14, 1399, 1,
5386}, {14, 1416, 1, 5386}, {16, 1408, 1, 5386}, {17, 1409, 1, 5386}, {17, 1418, 1, 5386},
{18, 1419, 1, 5386}, {11, 1397, 1, 5387}, {13, 1400, 1, 5387}, {13, 1407, 1, 5387}, {14,
1401, 1, 5387}, {14, 1417, 1, 5387}, {16, 1410, 1, 5387}, {17, 1411, 1, 5387}, {17, 1420,
1, 5387}, {18, 1421, 1, 5387}, {11, 1398, 1, 5388}, {13, 1401, 1, 5388}, {13, 1408, 1,
5388}, {14, 1402, 1, 5388}, {14, 1418, 1, 5388}, {16, 1411, 1, 5388}, {17, 1412, 1, 5388},
{17, 1421, 1, 5388}, {18, 1422, 1, 5388}, {11, 1399, 1, 5389}, {13, 1402, 1, 5389}, {13,
1409, 1, 5389}, {14, 1403, 1, 5389}, {14, 1419, 1, 5389}, {16, 1412, 1, 5389}, {17, 1413,
1, 5389}, {17, 1422, 1, 5389}, {18, 1423, 1, 5389}, {12, 1394, 1, 5390}, {14, 1395, 1,
5390}, {14, 1404, 1, 5390}, {15, 1396, 1, 5390}, {15, 1414, 1, 5390}, {17, 1405, 1, 5390},
{18, 1406, 1, 5390}, {18, 1415, 1, 5390}, {19, 1416, 1, 5390}, {12, 1395, 1, 5391}, {14,
1397, 1, 5391}, {14, 1405, 1, 5391}, {15, 1398, 1, 5391}, {15, 1415, 1, 5391}, {17, 1407,
1, 5391}, {18, 1408, 1, 5391}, {18, 1417, 1, 5391}, {19, 1418, 1, 5391}, {12, 1396, 1,
5392}, {14, 1398, 1, 5392}, {14, 1406, 1, 5392}, {15, 1399, 1, 5392}, {15, 1416, 1, 5392},
{17, 1408, 1, 5392}, {18, 1409, 1, 5392}, {18, 1418, 1, 5392}, {19, 1419, 1, 5392}, {12,
1397, 1, 5393}, {14, 1400, 1, 5393}, {14, 1407, 1, 5393}, {15, 1401, 1, 5393}, {15, 1417,
1, 5393}, {17, 1410, 1, 5393}, {18, 1411, 1, 5393}, {18, 1420, 1, 5393}, {19, 1421, 1,
5393}, {12, 1398, 1, 5394}, {14, 1401, 1, 5394}, {14, 1408, 1, 5394}, {15, 1402, 1, 5394},
{15, 1418, 1, 5394}, {17, 1411, 1, 5394}, {18, 1412, 1, 5394}, {18, 1421, 1, 5394}, {19,
1422, 1, 5394}, {12, 1399, 1, 5395}, {14, 1402, 1, 5395}, {14, 1409, 1, 5395}, {15, 1403,
1, 5395}, {15, 1419, 1, 5395}, {17, 1412, 1, 5395}, {18, 1413, 1, 5395}, {18, 1422, 1,
5395}, {19, 1423, 1, 5395}, {10, 1793, 1, 5396}, {11, 1794, 2, 5396}, {12, 1795, 2, 5396},
{13, 1799, 1, 5396}, {14, 1800, 2, 5396}, {15, 1804, 1, 5396}, {10, 1794, 1, 5397}, {11,
1796, 1, 5397}, {11, 1799, 1, 5397}, {12, 1797, 1, 5397}, {12, 1800, 1, 5397}, {13, 1801,
1, 5397}, {14, 1802, 1, 5397}, {14, 1805, 1, 5397}, {15, 1806, 1, 5397}, {10, 1795, 1,
5398}, {11, 1797, 1, 5398}, {11, 1800, 1, 5398}, {12, 1798, 1, 5398}, {12, 1804, 1, 5398},
{13, 1802, 1, 5398}, {14, 1803, 1, 5398}, {14, 1806, 1, 5398}, {15, 1807, 1, 5398}, {10,
1799, 1, 5399}, {11, 1801, 2, 5399}, {12, 1802, 2, 5399}, {13, 1808, 1, 5399}, {14, 1809,
2, 5399}, {15, 1811, 1, 5399}, {10, 1800, 1, 5400}, {11, 1802, 1, 5400}, {11, 1805, 1,
5400}, {12, 1803, 1, 5400}, {12, 1806, 1, 5400}, {13, 1809, 1, 5400}, {14, 1810, 1, 5400},
{14, 1811, 1, 5400}, {15, 1812, 1, 5400}, {10, 1804, 1, 5401}, {11, 1806, 2, 5401}, {12,
1807, 2, 5401}, {13, 1811, 1, 5401}, {14, 1812, 2, 5401}, {15, 1813, 1, 5401}, {11, 1793,
1, 5402}, {13, 1794, 2, 5402}, {14, 1795, 2, 5402}, {16, 1799, 1, 5402}, {17, 1800, 2,
5402}, {18, 1804, 1, 5402}, {11, 1794, 1, 5403}, {13, 1796, 1, 5403}, {13, 1799, 1, 5403},
{14, 1797, 1, 5403}, {14, 1800, 1, 5403}, {16, 1801, 1, 5403}, {17, 1802, 1, 5403}, {17,
1805, 1, 5403}, {18, 1806, 1, 5403}, {11, 1795, 1, 5404}, {13, 1797, 1, 5404}, {13, 1800,
1, 5404}, {14, 1798, 1, 5404}, {14, 1804, 1, 5404}, {16, 1802, 1, 5404}, {17, 1803, 1,
5404}, {17, 1806, 1, 5404}, {18, 1807, 1, 5404}, {11, 1799, 1, 5405}, {13, 1801, 2, 5405},

{14, 1802, 2, 5405}, {16, 1808, 1, 5405}, {17, 1809, 2, 5405}, {18, 1811, 1, 5405}, {11, 1800, 1, 5406}, {13, 1802, 1, 5406}, {13, 1805, 1, 5406}, {14, 1803, 1, 5406}, {14, 1806, 1, 5406}, {16, 1809, 1, 5406}, {17, 1810, 1, 5406}, {17, 1811, 1, 5406}, {18, 1812, 1, 5406}, {11, 1804, 1, 5407}, {13, 1806, 2, 5407}, {14, 1807, 2, 5407}, {16, 1811, 1, 5407}, {17, 1812, 2, 5407}, {18, 1813, 1, 5407}, {12, 1793, 1, 5408}, {14, 1794, 2, 5408}, {15, 1795, 2, 5408}, {17, 1799, 1, 5408}, {18, 1800, 2, 5408}, {19, 1804, 1, 5408}, {12, 1794, 1, 5409}, {14, 1796, 1, 5409}, {14, 1799, 1, 5409}, {15, 1797, 1, 5409}, {15, 1800, 1, 5409}, {17, 1801, 1, 5409}, {18, 1802, 1, 5409}, {18, 1805, 1, 5409}, {19, 1806, 1, 5409}, {12, 1795, 1, 5410}, {14, 1797, 1, 5410}, {14, 1800, 1, 5410}, {15, 1798, 1, 5410}, {15, 1804, 1, 5410}, {17, 1802, 1, 5410}, {18, 1803, 1, 5410}, {18, 1806, 1, 5410}, {19, 1807, 1, 5410}, {12, 1799, 1, 5411}, {14, 1801, 2, 5411}, {15, 1802, 2, 5411}, {17, 1808, 1, 5411}, {18, 1809, 2, 5411}, {19, 1811, 1, 5411}, {12, 1800, 1, 5412}, {14, 1802, 1, 5412}, {14, 1805, 1, 5412}, {15, 1803, 1, 5412}, {15, 1806, 1, 5412}, {17, 1809, 1, 5412}, {18, 1810, 1, 5412}, {18, 1811, 1, 5412}, {19, 1812, 1, 5412}, {12, 1804, 1, 5413}, {14, 1806, 2, 5413}, {15, 1807, 2, 5413}, {17, 1811, 1, 5413}, {18, 1812, 2, 5413}, {19, 1813, 1, 5413}, {10, 1964, 1, 5414}, {11, 1965, 2, 5414}, {12, 1966, 2, 5414}, {13, 1967, 1, 5414}, {14, 1968, 2, 5414}, {15, 1969, 1, 5414}, {11, 1964, 1, 5415}, {13, 1965, 2, 5415}, {14, 1966, 2, 5415}, {16, 1967, 1, 5415}, {17, 1968, 2, 5415}, {18, 1969, 1, 5415}, {12, 1964, 1, 5416}, {14, 1965, 2, 5416}, {15, 1966, 2, 5416}, {17, 1967, 1, 5416}, {18, 1968, 2, 5416}, {19, 1969, 1, 5416}, {10, 1970, 1, 5417}, {11, 1971, 1, 5417}, {11, 1976, 1, 5417}, {12, 1972, 1, 5417}, {12, 1982, 1, 5417}, {13, 1977, 1, 5417}, {14, 1978, 1, 5417}, {14, 1983, 1, 5417}, {15, 1984, 1, 5417}, {10, 1971, 1, 5418}, {11, 1973, 1, 5418}, {11, 1977, 1, 5418}, {12, 1974, 1, 5418}, {12, 1983, 1, 5418}, {13, 1979, 1, 5418}, {14, 1980, 1, 5418}, {14, 1985, 1, 5418}, {15, 1986, 1, 5418}, {10, 1972, 1, 5419}, {11, 1974, 1, 5419}, {11, 1978, 1, 5419}, {12, 1975, 1, 5419}, {12, 1984, 1, 5419}, {13, 1980, 1, 5419}, {14, 1981, 1, 5419}, {14, 1986, 1, 5419}, {15, 1987, 1, 5419}, {11, 1970, 1, 5420}, {13, 1971, 1, 5420}, {13, 1976, 1, 5420}, {14, 1972, 1, 5420}, {14, 1982, 1, 5420}, {16, 1977, 1, 5420}, {17, 1978, 1, 5420}, {17, 1983, 1, 5420}, {18, 1984, 1, 5420}, {11, 1971, 1, 5421}, {13, 1973, 1, 5421}, {13, 1977, 1, 5421}, {14, 1974, 1, 5421}, {14, 1983, 1, 5421}, {16, 1979, 1, 5421}, {17, 1980, 1, 5421}, {17, 1985, 1, 5421}, {18, 1986, 1, 5421}, {11, 1972, 1, 5422}, {13, 1974, 1, 5422}, {13, 1978, 1, 5422}, {14, 1975, 1, 5422}, {14, 1984, 1, 5422}, {16, 1980, 1, 5422}, {17, 1981, 1, 5422}, {17, 1986, 1, 5422}, {18, 1987, 1, 5422}, {12, 1970, 1, 5423}, {14, 1971, 1, 5423}, {14, 1976, 1, 5423}, {15, 1972, 1, 5423}, {15, 1982, 1, 5423}, {17, 1977, 1, 5423}, {18, 1978, 1, 5423}, {18, 1983, 1, 5423}, {19, 1984, 1, 5423}, {12, 1971, 1, 5424}, {14, 1973, 1, 5424}, {14, 1977, 1, 5424}, {15, 1974, 1, 5424}, {15, 1983, 1, 5424}, {17, 1979, 1, 5424}, {18, 1980, 1, 5424}, {18, 1985, 1, 5424}, {19, 1986, 1, 5424}, {12, 1972, 1, 5425}, {14, 1974, 1, 5425}, {14, 1978, 1, 5425}, {15, 1975, 1, 5425}, {15, 1984, 1, 5425}, {17, 1980, 1, 5425}, {18, 1981, 1, 5425}, {18, 1986, 1, 5425}, {19, 1987, 1, 5425}, {10, 1988, 1, 5426}, {11, 1989, 1, 5426}, {11, 1998, 1, 5426}, {12, 1990, 1, 5426}, {12, 2008, 1, 5426}, {13, 1999, 1, 5426}, {14, 2000, 1, 5426}, {14, 2009, 1, 5426}, {15, 2010, 1, 5426}, {10, 1989, 1, 5427}, {11, 1991, 1, 5427}, {11, 1999, 1, 5427}, {12, 1992, 1, 5427}, {12, 2009, 1, 5427}, {13, 2001, 1, 5427}, {14, 2002, 1, 5427}, {14, 2011, 1, 5427}, {15, 2012, 1, 5427}, {10, 1990, 1, 5428}, {11, 1992, 1, 5428}, {11, 2000, 1, 5428}, {12, 1993, 1, 5428}, {12, 2010, 1, 5428}, {13, 2002, 1, 5428}, {14, 2003, 1, 5428}, {14, 2012, 1, 5428}, {15, 2013, 1, 5428}, {10, 1991, 1, 5429}, {11, 1994, 1, 5429}, {11, 2001, 1, 5429}, {12, 1995, 1, 5429}, {12, 2011, 1, 5429}, {13, 2004, 1, 5429}, {14, 2005, 1, 5429}, {14, 2014, 1, 5429}, {15, 2015, 1, 5429}, {10, 1992, 1, 5430}, {11, 1995, 1, 5430}, {11, 2002, 1, 5430}, {12, 1996, 1, 5430}, {12, 2012, 1, 5430}, {13, 2005, 1, 5430}, {14, 2006, 1, 5430}, {14, 2015, 1, 5430}, {15, 2016, 1, 5430}, {10, 1993, 1, 5431}, {11, 1996, 1, 5431}, {11, 2003, 1, 5431}, {12, 1997, 1, 5431}, {12, 2013, 1, 5431}, {13, 2006, 1, 5431}, {14, 2007, 1, 5431}, {14, 2016, 1, 5431}, {15, 2017, 1, 5431}, {11, 1988, 1, 5432}, {13, 1989, 1, 5432}, {13, 1998, 1, 5432}, {14, 1990, 1, 5432}, {14, 2008, 1, 5432}, {16, 1999, 1, 5432}, {17, 2000, 1, 5432}, {17, 2009, 1, 5432}, {18, 2010, 1, 5432}, {11, 1989, 1, 5433}, {13, 1991, 1, 5433}, {13, 1999, 1, 5433}, {14, 1992, 1, 5433}, {14, 2009, 1, 5433}, {16, 2001, 1, 5433}, {17, 2002, 1, 5433}, {17, 2011, 1, 5433}, {18, 2012, 1, 5433}, {11, 1990, 1, 5434}, {13, 1992, 1, 5434}, {13, 2000, 1, 5434}, {14, 1993, 1, 5434}, {14, 2010, 1, 5434}, {16, 2002, 1, 5434}, {17, 2003, 1, 5434}, {17, 2012, 1, 5434}, {18, 2013, 1, 5434}, {11, 1991, 1, 5435}, {13, 1994, 1, 5435}, {13, 2001, 1, 5435}, {14, 1995, 1, 5435}, {14, 2011, 1, 5435}, {16, 2004, 1, 5435}, {17, 2005, 1, 5435}, {17, 2014, 1, 5435}, {18, 2015, 1, 5435}, {11, 1992, 1, 5436}, {13, 1995, 1, 5436}, {13, 2002, 1, 5436}, {14, 1996, 1, 5436}, {14, 2012, 1, 5436}, {16, 2005, 1, 5436}, {17, 2006, 1, 5436}, {17, 2015, 1, 5436}, {18, 2016, 1, 5436}, {11, 1993, 1, 5437}, {13, 1996, 1, 5437}, {13, 2003, 1, 5437}, {14, 1997, 1, 5437}, {14, 2013, 1, 5437}, {16, 2006, 1, 5437}, {17, 2007, 1, 5437}, {17, 2016, 1, 5437}, {18, 2017, 1, 5437}, {12, 1988, 1, 5438}, {14, 1989, 1, 5438}, {14, 1998, 1, 5438}, {15, 1990, 1, 5438}, {15, 2008, 1, 5438}, {17, 1999, 1, 5438}, {18, 2000, 1, 5438}, {18, 2009, 1, 5438}, {19, 2010, 1, 5438}, {12, 1989, 1, 5439}, {14, 1991, 1, 5439}, {14, 1999, 1, 5439}, {15, 1992, 1, 5439}, {15, 2009, 1, 5439}, {17, 2001, 1, 5439}, {18, 2002, 1, 5439}, {18, 2011, 1, 5439}, {19, 2012, 1, 5439}, {12, 1990, 1, 5440}, {14, 1992, 1, 5440}, {14, 2000, 1, 5440}, {15, 1993, 1, 5440}, {15,

2010, 1, 5440}, {17, 2002, 1, 5440}, {18, 2003, 1, 5440}, {18, 2012, 1, 5440}, {19, 2013, 1, 5440}, {12, 1991, 1, 5441}, {14, 1994, 1, 5441}, {14, 2001, 1, 5441}, {15, 1995, 1, 5441}, {15, 2011, 1, 5441}, {17, 2004, 1, 5441}, {18, 2005, 1, 5441}, {18, 2014, 1, 5441}, {19, 2015, 1, 5441}, {12, 1992, 1, 5442}, {14, 1995, 1, 5442}, {14, 2002, 1, 5442}, {15, 1996, 1, 5442}, {15, 2012, 1, 5442}, {17, 2005, 1, 5442}, {18, 2006, 1, 5442}, {18, 2015, 1, 5442}, {19, 2016, 1, 5442}, {12, 1993, 1, 5443}, {14, 1996, 1, 5443}, {14, 2003, 1, 5443}, {15, 1997, 1, 5443}, {15, 2013, 1, 5443}, {17, 2006, 1, 5443}, {18, 2007, 1, 5443}, {18, 2016, 1, 5443}, {19, 2017, 1, 5443}, {10, 2174, 1, 5444}, {11, 2175, 2, 5444}, {12, 2176, 2, 5444}, {13, 2180, 1, 5444}, {14, 2181, 2, 5444}, {15, 2185, 1, 5444}, {10, 2175, 1, 5445}, {11, 2177, 1, 5445}, {11, 2180, 1, 5445}, {12, 2178, 1, 5445}, {12, 2181, 1, 5445}, {13, 2182, 1, 5445}, {14, 2183, 1, 5445}, {14, 2186, 1, 5445}, {15, 2187, 1, 5445}, {10, 2176, 1, 5446}, {11, 2178, 1, 5446}, {11, 2181, 1, 5446}, {12, 2179, 1, 5446}, {12, 2185, 1, 5446}, {13, 2183, 1, 5446}, {14, 2184, 1, 5446}, {14, 2187, 1, 5446}, {15, 2188, 1, 5446}, {10, 2180, 1, 5447}, {11, 2182, 2, 5447}, {12, 2183, 2, 5447}, {13, 2189, 1, 5447}, {14, 2190, 2, 5447}, {15, 2192, 1, 5447}, {10, 2181, 1, 5448}, {11, 2183, 1, 5448}, {11, 2186, 1, 5448}, {12, 2184, 1, 5448}, {12, 2187, 1, 5448}, {13, 2190, 1, 5448}, {14, 2191, 1, 5448}, {14, 2192, 1, 5448}, {15, 2193, 1, 5448}, {10, 2185, 1, 5449}, {11, 2187, 2, 5449}, {12, 2188, 2, 5449}, {13, 2192, 1, 5449}, {14, 2193, 2, 5449}, {15, 2194, 1, 5449}, {11, 2174, 1, 5450}, {13, 2175, 2, 5450}, {14, 2176, 2, 5450}, {16, 2180, 1, 5450}, {17, 2181, 2, 5450}, {18, 2185, 1, 5450}, {11, 2175, 1, 5451}, {13, 2177, 1, 5451}, {13, 2180, 1, 5451}, {14, 2178, 1, 5451}, {14, 2181, 1, 5451}, {16, 2182, 1, 5451}, {17, 2183, 1, 5451}, {17, 2186, 1, 5451}, {18, 2187, 1, 5451}, {11, 2176, 1, 5452}, {13, 2178, 1, 5452}, {13, 2181, 1, 5452}, {14, 2179, 1, 5452}, {14, 2185, 1, 5452}, {16, 2183, 1, 5452}, {17, 2184, 1, 5452}, {17, 2187, 1, 5452}, {18, 2188, 1, 5452}, {11, 2180, 1, 5453}, {13, 2182, 2, 5453}, {14, 2183, 2, 5453}, {16, 2189, 1, 5453}, {17, 2190, 2, 5453}, {18, 2192, 1, 5453}, {11, 2181, 1, 5454}, {13, 2183, 1, 5454}, {13, 2186, 1, 5454}, {14, 2184, 1, 5454}, {14, 2187, 1, 5454}, {16, 2190, 1, 5454}, {17, 2191, 1, 5454}, {17, 2192, 1, 5454}, {18, 2193, 1, 5454}, {11, 2185, 1, 5455}, {13, 2187, 2, 5455}, {14, 2188, 2, 5455}, {16, 2192, 1, 5455}, {17, 2193, 2, 5455}, {18, 2194, 1, 5455}, {12, 2174, 1, 5456}, {14, 2175, 2, 5456}, {15, 2176, 2, 5456}, {17, 2180, 1, 5456}, {18, 2181, 2, 5456}, {19, 2185, 1, 5456}, {12, 2175, 1, 5457}, {14, 2177, 1, 5457}, {14, 2180, 1, 5457}, {15, 2178, 1, 5457}, {15, 2181, 1, 5457}, {17, 2182, 1, 5457}, {18, 2183, 1, 5457}, {18, 2186, 1, 5457}, {19, 2187, 1, 5457}, {12, 2176, 1, 5458}, {14, 2178, 1, 5458}, {14, 2181, 1, 5458}, {15, 2179, 1, 5458}, {15, 2185, 1, 5458}, {17, 2183, 1, 5458}, {18, 2184, 1, 5458}, {18, 2187, 1, 5458}, {19, 2188, 1, 5458}, {12, 2180, 1, 5459}, {14, 2182, 2, 5459}, {15, 2183, 2, 5459}, {17, 2189, 1, 5459}, {18, 2190, 2, 5459}, {19, 2192, 1, 5459}, {12, 2181, 1, 5460}, {14, 2183, 1, 5460}, {14, 2186, 1, 5460}, {15, 2184, 1, 5460}, {15, 2187, 1, 5460}, {17, 2190, 1, 5460}, {18, 2191, 1, 5460}, {18, 2192, 1, 5460}, {19, 2193, 1, 5460}, {12, 2185, 1, 5461}, {14, 2187, 2, 5461}, {15, 2188, 2, 5461}, {17, 2192, 1, 5461}, {18, 2193, 2, 5461}, {19, 2194, 1, 5461}, {10, 2345, 1, 5462}, {11, 2346, 2, 5462}, {12, 2347, 2, 5462}, {13, 2348, 1, 5462}, {14, 2349, 2, 5462}, {15, 2350, 1, 5462}, {11, 2345, 1, 5463}, {13, 2346, 2, 5463}, {14, 2347, 2, 5463}, {16, 2348, 1, 5463}, {17, 2349, 2, 5463}, {18, 2350, 1, 5463}, {12, 2345, 1, 5464}, {14, 2346, 2, 5464}, {15, 2347, 2, 5464}, {17, 2348, 1, 5464}, {18, 2349, 2, 5464}, {19, 2350, 1, 5464}, {10, 2351, 1, 5465}, {11, 2352, 1, 5465}, {11, 2357, 1, 5465}, {12, 2353, 1, 5465}, {12, 2363, 1, 5465}, {13, 2358, 1, 5465}, {14, 2359, 1, 5465}, {14, 2364, 1, 5465}, {15, 2365, 1, 5465}, {10, 2352, 1, 5466}, {11, 2354, 1, 5466}, {11, 2358, 1, 5466}, {12, 2355, 1, 5466}, {12, 2364, 1, 5466}, {13, 2360, 1, 5466}, {14, 2361, 1, 5466}, {14, 2366, 1, 5466}, {15, 2367, 1, 5466}, {10, 2353, 1, 5467}, {11, 2355, 1, 5467}, {11, 2359, 1, 5467}, {12, 2356, 1, 5467}, {12, 2365, 1, 5467}, {13, 2361, 1, 5467}, {14, 2362, 1, 5467}, {14, 2367, 1, 5467}, {15, 2368, 1, 5467}, {11, 2351, 1, 5468}, {13, 2352, 1, 5468}, {13, 2357, 1, 5468}, {14, 2353, 1, 5468}, {14, 2363, 1, 5468}, {16, 2358, 1, 5468}, {17, 2359, 1, 5468}, {17, 2364, 1, 5468}, {18, 2365, 1, 5468}, {11, 2352, 1, 5469}, {13, 2354, 1, 5469}, {13, 2358, 1, 5469}, {14, 2355, 1, 5469}, {14, 2364, 1, 5469}, {16, 2360, 1, 5469}, {17, 2361, 1, 5469}, {17, 2366, 1, 5469}, {18, 2367, 1, 5469}, {11, 2353, 1, 5470}, {13, 2355, 1, 5470}, {13, 2359, 1, 5470}, {14, 2356, 1, 5470}, {14, 2365, 1, 5470}, {16, 2361, 1, 5470}, {17, 2362, 1, 5470}, {17, 2367, 1, 5470}, {18, 2368, 1, 5470}, {12, 2351, 1, 5471}, {14, 2352, 1, 5471}, {14, 2357, 1, 5471}, {15, 2353, 1, 5471}, {15, 2363, 1, 5471}, {17, 2358, 1, 5471}, {18, 2359, 1, 5471}, {18, 2364, 1, 5471}, {19, 2365, 1, 5471}, {12, 2352, 1, 5472}, {14, 2354, 1, 5472}, {14, 2358, 1, 5472}, {15, 2355, 1, 5472}, {15, 2364, 1, 5472}, {17, 2360, 1, 5472}, {18, 2361, 1, 5472}, {18, 2366, 1, 5472}, {19, 2367, 1, 5472}, {12, 2353, 1, 5473}, {14, 2355, 1, 5473}, {14, 2359, 1, 5473}, {15, 2356, 1, 5473}, {15, 2365, 1, 5473}, {17, 2361, 1, 5473}, {18, 2362, 1, 5473}, {18, 2367, 1, 5473}, {19, 2368, 1, 5473}, {10, 2615, 1, 5474}, {11, 2616, 2, 5474}, {12, 2617, 2, 5474}, {13, 2618, 1, 5474}, {14, 2619, 2, 5474}, {15, 2620, 1, 5474}, {11, 2615, 1, 5475}, {13, 2616, 2, 5475}, {14, 2617, 2, 5475}, {16, 2618, 1, 5475}, {17, 2619, 2, 5475}, {18, 2620, 1, 5475}, {12, 2615, 1, 5476}, {14, 2616, 2, 5476}, {15, 2617, 2, 5476}, {17, 2618, 1, 5476}, {18, 2619, 2, 5476}, {19, 2620, 1, 5476}, {10, 833, 1, 5477}, {11, 834, 2, 5477}, {11, 839, 1, 5477}, {12, 835, 2, 5477}, {12, 845, 1, 5477}, {13, 836, 1, 5477}, {13, 840, 2, 5477}, {14, 837, 2, 5477}, {14, 841, 2, 5477}, {14, 846, 2, 5477},

{15, 838, 1, 5477}, {15, 847, 2, 5477}, {16, 842, 1, 5477}, {17, 843, 2, 5477}, {17, 848, 1, 5477}, {18, 844, 1, 5477}, {18, 849, 2, 5477}, {19, 850, 1, 5477}, {10, 851, 1, 5478}, {11, 852, 2, 5478}, {11, 861, 1, 5478}, {12, 853, 2, 5478}, {12, 871, 1, 5478}, {13, 854, 1, 5478}, {13, 862, 2, 5478}, {14, 855, 2, 5478}, {14, 863, 2, 5478}, {14, 872, 2, 5478}, {15, 856, 1, 5478}, {15, 873, 2, 5478}, {16, 864, 1, 5478}, {17, 865, 2, 5478}, {17, 874, 1, 5478}, {18, 866, 1, 5478}, {18, 875, 2, 5478}, {19, 876, 1, 5478}, {10, 852, 1, 5479}, {11, 854, 2, 5479}, {11, 862, 1, 5479}, {12, 855, 2, 5479}, {12, 872, 1, 5479}, {13, 857, 1, 5479}, {13, 864, 2, 5479}, {14, 858, 2, 5479}, {14, 865, 2, 5479}, {14, 874, 2, 5479}, {15, 859, 1, 5479}, {15, 875, 2, 5479}, {16, 867, 1, 5479}, {17, 868, 2, 5479}, {17, 877, 1, 5479}, {18, 869, 1, 5479}, {18, 878, 2, 5479}, {19, 879, 1, 5479}, {10, 853, 1, 5480}, {11, 855, 2, 5480}, {11, 863, 1, 5480}, {12, 856, 2, 5480}, {12, 873, 1, 5480}, {13, 858, 1, 5480}, {13, 865, 2, 5480}, {14, 859, 2, 5480}, {14, 866, 2, 5480}, {14, 875, 2, 5480}, {15, 860, 1, 5480}, {15, 876, 2, 5480}, {16, 868, 1, 5480}, {17, 869, 2, 5480}, {17, 878, 1, 5480}, {18, 870, 1, 5480}, {18, 879, 2, 5480}, {19, 880, 1, 5480}, {10, 881, 1, 5481}, {11, 882, 2, 5481}, {11, 896, 1, 5481}, {12, 883, 2, 5481}, {12, 911, 1, 5481}, {13, 884, 1, 5481}, {13, 897, 2, 5481}, {14, 885, 2, 5481}, {14, 898, 2, 5481}, {14, 912, 2, 5481}, {15, 886, 1, 5481}, {15, 913, 2, 5481}, {16, 899, 1, 5481}, {17, 900, 2, 5481}, {17, 914, 1, 5481}, {18, 901, 1, 5481}, {18, 915, 2, 5481}, {19, 916, 1, 5481}, {10, 882, 1, 5482}, {11, 884, 2, 5482}, {11, 897, 1, 5482}, {12, 885, 2, 5482}, {12, 912, 1, 5482}, {13, 887, 1, 5482}, {13, 899, 2, 5482}, {14, 888, 2, 5482}, {14, 900, 2, 5482}, {14, 914, 2, 5482}, {15, 889, 1, 5482}, {15, 915, 2, 5482}, {16, 902, 1, 5482}, {17, 903, 2, 5482}, {17, 917, 1, 5482}, {18, 904, 1, 5482}, {18, 918, 2, 5482}, {19, 919, 1, 5482}, {10, 883, 1, 5483}, {11, 885, 2, 5483}, {11, 898, 1, 5483}, {12, 886, 2, 5483}, {12, 913, 1, 5483}, {13, 888, 1, 5483}, {13, 900, 2, 5483}, {14, 889, 2, 5483}, {14, 901, 2, 5483}, {14, 915, 2, 5483}, {15, 890, 1, 5483}, {15, 916, 2, 5483}, {16, 903, 1, 5483}, {17, 904, 2, 5483}, {17, 918, 1, 5483}, {18, 905, 1, 5483}, {18, 919, 2, 5483}, {19, 920, 1, 5483}, {10, 884, 1, 5484}, {11, 887, 2, 5484}, {11, 899, 1, 5484}, {12, 888, 2, 5484}, {12, 914, 1, 5484}, {13, 891, 1, 5484}, {13, 902, 2, 5484}, {14, 892, 2, 5484}, {14, 903, 2, 5484}, {14, 917, 2, 5484}, {15, 893, 1, 5484}, {15, 918, 2, 5484}, {16, 906, 1, 5484}, {17, 907, 2, 5484}, {17, 921, 1, 5484}, {18, 908, 1, 5484}, {18, 922, 2, 5484}, {19, 923, 1, 5484}, {10, 885, 1, 5485}, {11, 888, 2, 5485}, {11, 900, 1, 5485}, {12, 889, 2, 5485}, {12, 915, 1, 5485}, {13, 892, 1, 5485}, {13, 903, 2, 5485}, {14, 893, 2, 5485}, {14, 904, 2, 5485}, {14, 918, 2, 5485}, {15, 894, 1, 5485}, {15, 919, 2, 5485}, {16, 907, 1, 5485}, {17, 908, 2, 5485}, {17, 922, 1, 5485}, {18, 909, 1, 5485}, {18, 923, 2, 5485}, {19, 924, 1, 5485}, {10, 886, 1, 5486}, {11, 889, 2, 5486}, {11, 901, 1, 5486}, {12, 890, 2, 5486}, {12, 916, 1, 5486}, {13, 893, 1, 5486}, {13, 904, 2, 5486}, {14, 894, 2, 5486}, {14, 905, 2, 5486}, {14, 919, 2, 5486}, {15, 895, 1, 5486}, {15, 920, 2, 5486}, {16, 908, 1, 5486}, {17, 909, 2, 5486}, {17, 923, 1, 5486}, {18, 910, 1, 5486}, {18, 924, 2, 5486}, {19, 925, 1, 5486}, {10, 926, 1, 5487}, {11, 927, 2, 5487}, {11, 947, 1, 5487}, {12, 928, 2, 5487}, {12, 968, 1, 5487}, {13, 929, 1, 5487}, {13, 948, 2, 5487}, {14, 930, 2, 5487}, {14, 949, 2, 5487}, {14, 969, 2, 5487}, {15, 931, 1, 5487}, {15, 970, 2, 5487}, {16, 950, 1, 5487}, {17, 951, 2, 5487}, {17, 971, 1, 5487}, {18, 952, 1, 5487}, {18, 972, 2, 5487}, {19, 973, 1, 5487}, {10, 927, 1, 5488}, {11, 929, 2, 5488}, {11, 948, 1, 5488}, {12, 930, 2, 5488}, {12, 969, 1, 5488}, {13, 932, 1, 5488}, {13, 950, 2, 5488}, {14, 933, 2, 5488}, {14, 951, 2, 5488}, {14, 971, 2, 5488}, {15, 934, 1, 5488}, {15, 972, 2, 5488}, {16, 953, 1, 5488}, {17, 954, 2, 5488}, {17, 974, 1, 5488}, {18, 955, 1, 5488}, {18, 975, 2, 5488}, {19, 976, 1, 5488}, {10, 928, 1, 5489}, {11, 930, 2, 5489}, {11, 949, 1, 5489}, {12, 931, 2, 5489}, {12, 970, 1, 5489}, {13, 933, 1, 5489}, {13, 951, 2, 5489}, {14, 934, 2, 5489}, {14, 952, 2, 5489}, {14, 972, 2, 5489}, {15, 935, 1, 5489}, {15, 973, 2, 5489}, {16, 954, 1, 5489}, {17, 955, 2, 5489}, {17, 975, 1, 5489}, {18, 956, 1, 5489}, {18, 976, 2, 5489}, {19, 977, 1, 5489}, {10, 929, 1, 5490}, {11, 932, 2, 5490}, {11, 950, 1, 5490}, {12, 933, 2, 5490}, {12, 971, 1, 5490}, {13, 936, 1, 5490}, {13, 953, 2, 5490}, {14, 937, 2, 5490}, {14, 954, 2, 5490}, {14, 974, 2, 5490}, {15, 938, 1, 5490}, {15, 975, 2, 5490}, {16, 957, 1, 5490}, {17, 958, 2, 5490}, {17, 978, 1, 5490}, {18, 959, 1, 5490}, {18, 979, 2, 5490}, {19, 980, 1, 5490}, {10, 930, 1, 5491}, {11, 933, 2, 5491}, {11, 951, 1, 5491}, {12, 934, 2, 5491}, {12, 972, 1, 5491}, {13, 937, 1, 5491}, {13, 954, 2, 5491}, {14, 938, 2, 5491}, {14, 955, 2, 5491}, {14, 975, 2, 5491}, {15, 939, 1, 5491}, {15, 976, 2, 5491}, {16, 958, 1, 5491}, {17, 959, 2, 5491}, {17, 979, 1, 5491}, {18, 960, 1, 5491}, {18, 980, 2, 5491}, {19, 981, 1, 5491}, {10, 931, 1, 5492}, {11, 934, 2, 5492}, {11, 952, 1, 5492}, {12, 935, 2, 5492}, {12, 973, 1, 5492}, {13, 938, 1, 5492}, {13, 955, 2, 5492}, {14, 939, 2, 5492}, {14, 956, 2, 5492}, {14, 976, 2, 5492}, {15, 940, 1, 5492}, {15, 977, 2, 5492}, {16, 959, 1, 5492}, {17, 960, 2, 5492}, {17, 980, 1, 5492}, {18, 961, 1, 5492}, {18, 981, 2, 5492}, {19, 982, 1, 5492}, {10, 932, 1, 5493}, {11, 936, 2, 5493}, {11, 953, 1, 5493}, {12, 937, 2, 5493}, {12, 974, 1, 5493}, {13, 941, 1, 5493}, {13, 957, 2, 5493}, {14, 942, 2, 5493}, {14, 958, 2, 5493}, {14, 978, 2, 5493}, {15, 943, 1, 5493}, {15, 979, 2, 5493}, {16, 962, 1, 5493}, {17, 963, 2, 5493}, {17, 983, 1, 5493}, {18, 964, 1, 5493}, {18, 984, 2, 5493}, {19, 985, 1, 5493}, {10, 933, 1, 5494}, {11, 937, 2, 5494}, {11, 954, 1, 5494}, {12, 938, 2, 5494}, {12, 975, 1, 5494}, {13, 942, 1, 5494}, {13, 958, 2, 5494}, {14, 943, 2, 5494}, {14, 959, 2, 5494}, {14, 979, 2, 5494}, {15, 944, 1, 5494}, {15, 980, 2, 5494}, {16, 963, 1, 5494}, {17, 964, 2, 5494}, {17, 984,

1, 5494}, {18, 965, 1, 5494}, {18, 985, 2, 5494}, {19, 986, 1, 5494}, {10, 934, 1, 5495}, {11, 938, 2, 5495}, {11, 955, 1, 5495}, {12, 939, 2, 5495}, {12, 976, 1, 5495}, {13, 943, 1, 5495}, {13, 959, 2, 5495}, {14, 944, 2, 5495}, {14, 960, 2, 5495}, {14, 980, 2, 5495}, {15, 945, 1, 5495}, {15, 981, 2, 5495}, {16, 964, 1, 5495}, {17, 965, 2, 5495}, {17, 985, 1, 5495}, {18, 966, 1, 5495}, {18, 986, 2, 5495}, {19, 987, 1, 5495}, {10, 935, 1, 5496}, {11, 939, 2, 5496}, {11, 956, 1, 5496}, {12, 940, 2, 5496}, {12, 977, 1, 5496}, {13, 944, 1, 5496}, {13, 960, 2, 5496}, {14, 945, 2, 5496}, {14, 961, 2, 5496}, {14, 981, 2, 5496}, {15, 946, 1, 5496}, {15, 982, 2, 5496}, {16, 965, 1, 5496}, {17, 966, 2, 5496}, {17, 986, 1, 5496}, {18, 967, 1, 5496}, {18, 987, 2, 5496}, {19, 988, 1, 5496}, {20, 827, 1, 5497}, {21, 828, 2, 5497}, {22, 829, 2, 5497}, {23, 830, 1, 5497}, {24, 831, 2, 5497}, {25, 832, 1, 5497}, {21, 827, 1, 5498}, {23, 828, 2, 5498}, {24, 829, 2, 5498}, {26, 830, 1, 5498}, {27, 831, 2, 5498}, {28, 832, 1, 5498}, {22, 827, 1, 5499}, {24, 828, 2, 5499}, {25, 829, 2, 5499}, {27, 830, 1, 5499}, {28, 831, 2, 5499}, {29, 832, 1, 5499}, {23, 827, 1, 5500}, {26, 828, 2, 5500}, {27, 829, 2, 5500}, {30, 830, 1, 5500}, {31, 831, 2, 5500}, {32, 832, 1, 5500}, {24, 827, 1, 5501}, {27, 828, 2, 5501}, {28, 829, 2, 5501}, {31, 830, 1, 5501}, {32, 831, 2, 5501}, {33, 832, 1, 5501}, {25, 827, 1, 5502}, {28, 828, 2, 5502}, {29, 829, 2, 5502}, {32, 830, 1, 5502}, {33, 831, 2, 5502}, {34, 832, 1, 5502}, {20, 833, 1, 5503}, {21, 834, 1, 5503}, {21, 839, 1, 5503}, {22, 835, 1, 5503}, {22, 845, 1, 5503}, {23, 840, 1, 5503}, {24, 841, 1, 5503}, {24, 846, 1, 5503}, {25, 847, 1, 5503}, {20, 834, 1, 5504}, {21, 836, 1, 5504}, {21, 840, 1, 5504}, {22, 837, 1, 5504}, {22, 846, 1, 5504}, {23, 842, 1, 5504}, {24, 843, 1, 5504}, {24, 848, 1, 5504}, {25, 849, 1, 5504}, {20, 835, 1, 5505}, {21, 837, 1, 5505}, {21, 841, 1, 5505}, {22, 838, 1, 5505}, {22, 847, 1, 5505}, {23, 843, 1, 5505}, {24, 844, 1, 5505}, {24, 849, 1, 5505}, {25, 850, 1, 5505}, {21, 833, 1, 5506}, {23, 834, 1, 5506}, {23, 839, 1, 5506}, {24, 835, 1, 5506}, {24, 845, 1, 5506}, {26, 840, 1, 5506}, {27, 841, 1, 5506}, {27, 846, 1, 5506}, {28, 847, 1, 5506}, {21, 834, 1, 5507}, {23, 836, 1, 5507}, {23, 840, 1, 5507}, {24, 837, 1, 5507}, {24, 846, 1, 5507}, {26, 842, 1, 5507}, {27, 843, 1, 5507}, {27, 848, 1, 5507}, {28, 849, 1, 5507}, {21, 835, 1, 5508}, {23, 837, 1, 5508}, {23, 841, 1, 5508}, {24, 838, 1, 5508}, {24, 847, 1, 5508}, {26, 843, 1, 5508}, {27, 844, 1, 5508}, {27, 849, 1, 5508}, {28, 850, 1, 5508}, {22, 833, 1, 5509}, {24, 834, 1, 5509}, {24, 839, 1, 5509}, {25, 835, 1, 5509}, {25, 845, 1, 5509}, {27, 840, 1, 5509}, {28, 841, 1, 5509}, {28, 846, 1, 5509}, {29, 847, 1, 5509}, {22, 834, 1, 5510}, {24, 836, 1, 5510}, {24, 840, 1, 5510}, {25, 837, 1, 5510}, {25, 846, 1, 5510}, {27, 842, 1, 5510}, {28, 843, 1, 5510}, {28, 848, 1, 5510}, {29, 849, 1, 5510}, {22, 835, 1, 5511}, {24, 837, 1, 5511}, {24, 841, 1, 5511}, {25, 838, 1, 5511}, {25, 847, 1, 5511}, {27, 843, 1, 5511}, {28, 844, 1, 5511}, {28, 849, 1, 5511}, {29, 850, 1, 5511}, {23, 833, 1, 5512}, {26, 834, 1, 5512}, {26, 839, 1, 5512}, {27, 835, 1, 5512}, {27, 845, 1, 5512}, {30, 840, 1, 5512}, {31, 841, 1, 5512}, {31, 846, 1, 5512}, {32, 847, 1, 5512}, {23, 834, 1, 5513}, {26, 836, 1, 5513}, {26, 840, 1, 5513}, {27, 837, 1, 5513}, {27, 846, 1, 5513}, {30, 842, 1, 5513}, {31, 843, 1, 5513}, {31, 848, 1, 5513}, {32, 849, 1, 5513}, {23, 835, 1, 5514}, {26, 837, 1, 5514}, {26, 841, 1, 5514}, {27, 838, 1, 5514}, {27, 847, 1, 5514}, {30, 843, 1, 5514}, {31, 844, 1, 5514}, {31, 849, 1, 5514}, {32, 850, 1, 5514}, {24, 833, 1, 5515}, {27, 834, 1, 5515}, {27, 839, 1, 5515}, {28, 835, 1, 5515}, {28, 845, 1, 5515}, {31, 840, 1, 5515}, {32, 841, 1, 5515}, {32, 846, 1, 5515}, {33, 847, 1, 5515}, {24, 834, 1, 5516}, {27, 836, 1, 5516}, {27, 840, 1, 5516}, {28, 837, 1, 5516}, {28, 846, 1, 5516}, {31, 842, 1, 5516}, {32, 843, 1, 5516}, {32, 848, 1, 5516}, {33, 849, 1, 5516}, {24, 835, 1, 5517}, {27, 837, 1, 5517}, {27, 841, 1, 5517}, {28, 838, 1, 5517}, {28, 847, 1, 5517}, {31, 843, 1, 5517}, {32, 844, 1, 5517}, {32, 849, 1, 5517}, {33, 850, 1, 5517}, {25, 833, 1, 5518}, {28, 834, 1, 5518}, {28, 839, 1, 5518}, {29, 835, 1, 5518}, {29, 845, 1, 5518}, {32, 840, 1, 5518}, {33, 841, 1, 5518}, {33, 846, 1, 5518}, {34, 847, 1, 5518}, {25, 834, 1, 5519}, {28, 836, 1, 5519}, {28, 840, 1, 5519}, {29, 837, 1, 5519}, {29, 846, 1, 5519}, {32, 842, 1, 5519}, {33, 843, 1, 5519}, {33, 848, 1, 5519}, {34, 849, 1, 5519}, {25, 835, 1, 5520}, {28, 837, 1, 5520}, {28, 841, 1, 5520}, {29, 838, 1, 5520}, {29, 847, 1, 5520}, {32, 843, 1, 5520}, {33, 844, 1, 5520}, {33, 849, 1, 5520}, {34, 850, 1, 5520}, {10, 1073, 1, 5521}, {11, 1074, 3, 5521}, {12, 1075, 3, 5521}, {13, 1076, 1, 5521}, {13, 1079, 2, 5521}, {14, 1077, 2, 5521}, {14, 1080, 4, 5521}, {15, 1078, 1, 5521}, {15, 1084, 2, 5521}, {16, 1081, 1, 5521}, {17, 1082, 2, 5521}, {17, 1085, 1, 5521}, {18, 1083, 1, 5521}, {18, 1086, 2, 5521}, {19, 1087, 1, 5521}, {10, 1074, 1, 5522}, {11, 1076, 1, 5522}, {11, 1079, 2, 5522}, {12, 1077, 1, 5522}, {12, 1080, 2, 5522}, {13, 1081, 3, 5522}, {14, 1082, 4, 5522}, {14, 1085, 2, 5522}, {15, 1083, 1, 5522}, {15, 1086, 2, 5522}, {16, 1088, 1, 5522}, {17, 1089, 3, 5522}, {18, 1090, 1, 5522}, {18, 1091, 2, 5522}, {19, 1092, 1, 5522}, {10, 1075, 1, 5523}, {11, 1077, 1, 5523}, {11, 1080, 2, 5523}, {12, 1078, 1, 5523}, {12, 1084, 2, 5523}, {13, 1082, 2, 5523}, {13, 1085, 1, 5523}, {14, 1083, 2, 5523}, {14, 1086, 4, 5523}, {15, 1087, 3, 5523}, {16, 1089, 1, 5523}, {17, 1090, 1, 5523}, {17, 1091, 2, 5523}, {18, 1092, 3, 5523}, {19, 1093, 1, 5523}, {10, 1094, 1, 5524}, {11, 1095, 2, 5524}, {11, 1104, 1, 5524}, {12, 1096, 2, 5524}, {12, 1114, 1, 5524}, {13, 1097, 1, 5524}, {13, 1105, 2, 5524}, {14, 1098, 2, 5524}, {14, 1106, 2, 5524}, {14, 1115, 2, 5524}, {15, 1099, 1, 5524}, {15, 1116, 2, 5524}, {16, 1107, 1, 5524}, {17, 1108, 2, 5524}, {17, 1117, 1, 5524}, {18, 1109, 1, 5524}, {18, 1118, 2, 5524}, {19, 1119, 1, 5524}, {10, 1095, 1, 5525}, {11, 1097, 2, 5525}, {11, 1105, 1, 5525}, {12, 1098, 2, 5525}, {12, 1115, 1, 5525}, {13, 1100, 1, 5525},

{13, 1107, 2, 5525}, {14, 1101, 2, 5525}, {14, 1108, 2, 5525}, {14, 1117, 2, 5525}, {15, 1102, 1, 5525}, {15, 1118, 2, 5525}, {16, 1110, 1, 5525}, {17, 1111, 2, 5525}, {17, 1120, 1, 5525}, {18, 1112, 1, 5525}, {18, 1121, 2, 5525}, {19, 1122, 1, 5525}, {10, 1096, 1, 5526}, {11, 1098, 2, 5526}, {11, 1106, 1, 5526}, {12, 1099, 2, 5526}, {12, 1116, 1, 5526}, {13, 1101, 1, 5526}, {13, 1108, 2, 5526}, {14, 1102, 2, 5526}, {14, 1109, 2, 5526}, {14, 1118, 2, 5526}, {15, 1103, 1, 5526}, {15, 1119, 2, 5526}, {16, 1111, 1, 5526}, {17, 1112, 2, 5526}, {17, 1121, 1, 5526}, {18, 1113, 1, 5526}, {18, 1122, 2, 5526}, {19, 1123, 1, 5526}, {10, 1104, 1, 5527}, {11, 1105, 2, 5527}, {11, 1124, 1, 5527}, {12, 1106, 2, 5527}, {12, 1134, 1, 5527}, {13, 1107, 1, 5527}, {13, 1125, 2, 5527}, {14, 1108, 2, 5527}, {14, 1126, 2, 5527}, {14, 1135, 2, 5527}, {15, 1109, 1, 5527}, {15, 1136, 2, 5527}, {16, 1127, 1, 5527}, {17, 1128, 2, 5527}, {17, 1137, 1, 5527}, {18, 1129, 1, 5527}, {18, 1138, 2, 5527}, {19, 1139, 1, 5527}, {10, 1105, 1, 5528}, {11, 1107, 2, 5528}, {11, 1125, 1, 5528}, {12, 1108, 2, 5528}, {12, 1135, 1, 5528}, {13, 1110, 1, 5528}, {13, 1127, 2, 5528}, {14, 1111, 2, 5528}, {14, 1128, 2, 5528}, {14, 1137, 2, 5528}, {15, 1112, 1, 5528}, {15, 1138, 2, 5528}, {16, 1130, 1, 5528}, {17, 1131, 2, 5528}, {17, 1140, 1, 5528}, {18, 1132, 1, 5528}, {18, 1141, 2, 5528}, {19, 1142, 1, 5528}, {10, 1106, 1, 5529}, {11, 1108, 2, 5529}, {11, 1126, 1, 5529}, {12, 1109, 2, 5529}, {12, 1136, 1, 5529}, {13, 1111, 1, 5529}, {13, 1128, 2, 5529}, {14, 1112, 2, 5529}, {14, 1129, 2, 5529}, {14, 1138, 2, 5529}, {15, 1113, 1, 5529}, {15, 1139, 2, 5529}, {16, 1131, 1, 5529}, {17, 1132, 2, 5529}, {17, 1141, 1, 5529}, {18, 1133, 1, 5529}, {18, 1142, 2, 5529}, {19, 1143, 1, 5529}, {10, 1114, 1, 5530}, {11, 1115, 2, 5530}, {11, 1134, 1, 5530}, {12, 1116, 2, 5530}, {12, 1144, 1, 5530}, {13, 1117, 1, 5530}, {13, 1135, 2, 5530}, {14, 1118, 2, 5530}, {14, 1136, 2, 5530}, {14, 1145, 2, 5530}, {15, 1119, 1, 5530}, {15, 1146, 2, 5530}, {16, 1137, 1, 5530}, {17, 1138, 2, 5530}, {17, 1147, 1, 5530}, {18, 1139, 1, 5530}, {18, 1148, 2, 5530}, {19, 1149, 1, 5530}, {10, 1115, 1, 5531}, {11, 1117, 2, 5531}, {11, 1135, 1, 5531}, {12, 1118, 2, 5531}, {12, 1145, 1, 5531}, {13, 1120, 1, 5531}, {13, 1137, 2, 5531}, {14, 1121, 2, 5531}, {14, 1138, 2, 5531}, {14, 1147, 2, 5531}, {15, 1122, 1, 5531}, {15, 1148, 2, 5531}, {16, 1140, 1, 5531}, {17, 1141, 2, 5531}, {17, 1150, 1, 5531}, {18, 1142, 1, 5531}, {18, 1151, 2, 5531}, {19, 1152, 1, 5531}, {10, 1116, 1, 5532}, {11, 1118, 2, 5532}, {11, 1136, 1, 5532}, {12, 1119, 2, 5532}, {12, 1146, 1, 5532}, {13, 1121, 1, 5532}, {13, 1138, 2, 5532}, {14, 1122, 2, 5532}, {14, 1139, 2, 5532}, {14, 1148, 2, 5532}, {15, 1123, 1, 5532}, {15, 1149, 2, 5532}, {16, 1141, 1, 5532}, {17, 1142, 2, 5532}, {17, 1151, 1, 5532}, {18, 1143, 1, 5532}, {18, 1152, 2, 5532}, {19, 1153, 1, 5532}, {10, 1154, 1, 5533}, {11, 1155, 2, 5533}, {11, 1169, 1, 5533}, {12, 1156, 2, 5533}, {12, 1184, 1, 5533}, {13, 1157, 1, 5533}, {13, 1170, 2, 5533}, {14, 1158, 2, 5533}, {14, 1171, 2, 5533}, {14, 1185, 2, 5533}, {15, 1159, 1, 5533}, {15, 1186, 2, 5533}, {16, 1172, 1, 5533}, {17, 1173, 2, 5533}, {17, 1187, 1, 5533}, {18, 1174, 1, 5533}, {18, 1188, 2, 5533}, {19, 1189, 1, 5533}, {10, 1155, 1, 5534}, {11, 1157, 2, 5534}, {11, 1170, 1, 5534}, {12, 1158, 2, 5534}, {12, 1185, 1, 5534}, {13, 1160, 1, 5534}, {13, 1172, 2, 5534}, {14, 1161, 2, 5534}, {14, 1173, 2, 5534}, {14, 1187, 2, 5534}, {15, 1162, 1, 5534}, {15, 1188, 2, 5534}, {16, 1175, 1, 5534}, {17, 1176, 2, 5534}, {17, 1190, 1, 5534}, {18, 1177, 1, 5534}, {18, 1191, 2, 5534}, {19, 1192, 1, 5534}, {10, 1156, 1, 5535}, {11, 1158, 2, 5535}, {11, 1171, 1, 5535}, {12, 1159, 2, 5535}, {12, 1186, 1, 5535}, {13, 1161, 1, 5535}, {13, 1173, 2, 5535}, {14, 1162, 2, 5535}, {14, 1174, 2, 5535}, {14, 1188, 2, 5535}, {15, 1163, 1, 5535}, {15, 1189, 2, 5535}, {16, 1176, 1, 5535}, {17, 1177, 2, 5535}, {17, 1191, 1, 5535}, {18, 1178, 1, 5535}, {18, 1192, 2, 5535}, {19, 1193, 1, 5535}, {10, 1157, 1, 5536}, {11, 1160, 2, 5536}, {11, 1172, 1, 5536}, {12, 1161, 2, 5536}, {12, 1187, 1, 5536}, {13, 1164, 1, 5536}, {13, 1175, 2, 5536}, {14, 1165, 2, 5536}, {14, 1176, 2, 5536}, {14, 1190, 2, 5536}, {15, 1166, 1, 5536}, {15, 1191, 2, 5536}, {16, 1179, 1, 5536}, {17, 1180, 2, 5536}, {17, 1194, 1, 5536}, {18, 1181, 1, 5536}, {18, 1195, 2, 5536}, {19, 1196, 1, 5536}, {10, 1158, 1, 5537}, {11, 1161, 2, 5537}, {11, 1173, 1, 5537}, {12, 1162, 2, 5537}, {12, 1188, 1, 5537}, {13, 1165, 1, 5537}, {13, 1176, 2, 5537}, {14, 1166, 2, 5537}, {14, 1177, 2, 5537}, {14, 1191, 2, 5537}, {15, 1167, 1, 5537}, {15, 1192, 2, 5537}, {16, 1180, 1, 5537}, {17, 1181, 2, 5537}, {17, 1195, 1, 5537}, {18, 1182, 1, 5537}, {18, 1196, 2, 5537}, {19, 1197, 1, 5537}, {10, 1159, 1, 5538}, {11, 1162, 2, 5538}, {11, 1174, 1, 5538}, {12, 1163, 2, 5538}, {12, 1189, 1, 5538}, {13, 1166, 1, 5538}, {13, 1177, 2, 5538}, {14, 1167, 2, 5538}, {14, 1178, 2, 5538}, {14, 1192, 2, 5538}, {15, 1168, 1, 5538}, {15, 1193, 2, 5538}, {16, 1181, 1, 5538}, {17, 1182, 2, 5538}, {17, 1196, 1, 5538}, {18, 1183, 1, 5538}, {18, 1197, 2, 5538}, {19, 1198, 1, 5538}, {10, 1169, 1, 5539}, {11, 1170, 2, 5539}, {11, 1199, 1, 5539}, {12, 1171, 2, 5539}, {12, 1214, 1, 5539}, {13, 1172, 1, 5539}, {13, 1200, 2, 5539}, {14, 1173, 2, 5539}, {14, 1201, 2, 5539}, {14, 1215, 2, 5539}, {15, 1174, 1, 5539}, {15, 1216, 2, 5539}, {16, 1202, 1, 5539}, {17, 1203, 2, 5539}, {17, 1217, 1, 5539}, {18, 1204, 1, 5539}, {18, 1218, 2, 5539}, {19, 1219, 1, 5539}, {10, 1170, 1, 5540}, {11, 1172, 2, 5540}, {11, 1200, 1, 5540}, {12, 1173, 2, 5540}, {12, 1215, 1, 5540}, {13, 1175, 1, 5540}, {13, 1202, 2, 5540}, {14, 1176, 2, 5540}, {14, 1203, 2, 5540}, {14, 1217, 2, 5540}, {15, 1177, 1, 5540}, {15, 1218, 2, 5540}, {16, 1205, 1, 5540}, {17, 1206, 2, 5540}, {17, 1220, 1, 5540}, {18, 1207, 1, 5540}, {18, 1221, 2, 5540}, {19, 1222, 1, 5540}, {10, 1171, 1, 5541}, {11, 1173, 2, 5541}, {11, 1201, 1, 5541}, {12, 1174, 2, 5541}, {12, 1216, 1, 5541}, {13, 1176, 1, 5541}, {13, 1203, 2, 5541}, {14, 1177, 2, 5541}, {14, 1204, 2, 5541}, {14, 1218, 2, 5541}, {15, 1178, 1, 5541}, {15,

1219, 2, 5541}, {16, 1206, 1, 5541}, {17, 1207, 2, 5541}, {17, 1221, 1, 5541}, {18, 1208, 1, 5541}, {18, 1222, 2, 5541}, {19, 1223, 1, 5541}, {10, 1172, 1, 5542}, {11, 1175, 2, 5542}, {11, 1202, 1, 5542}, {12, 1176, 2, 5542}, {12, 1217, 1, 5542}, {13, 1179, 1, 5542}, {13, 1205, 2, 5542}, {14, 1180, 2, 5542}, {14, 1206, 2, 5542}, {14, 1220, 2, 5542}, {15, 1181, 1, 5542}, {15, 1221, 2, 5542}, {16, 1209, 1, 5542}, {17, 1210, 2, 5542}, {17, 1224, 1, 5542}, {18, 1211, 1, 5542}, {18, 1225, 2, 5542}, {19, 1226, 1, 5542}, {10, 1173, 1, 5543}, {11, 1176, 2, 5543}, {11, 1203, 1, 5543}, {12, 1177, 2, 5543}, {12, 1218, 1, 5543}, {13, 1180, 1, 5543}, {13, 1206, 2, 5543}, {14, 1181, 2, 5543}, {14, 1207, 2, 5543}, {14, 1221, 2, 5543}, {15, 1182, 1, 5543}, {15, 1222, 2, 5543}, {16, 1210, 1, 5543}, {17, 1211, 2, 5543}, {17, 1225, 1, 5543}, {18, 1212, 1, 5543}, {18, 1226, 2, 5543}, {19, 1227, 1, 5543}, {10, 1174, 1, 5544}, {11, 1177, 2, 5544}, {11, 1204, 1, 5544}, {12, 1178, 2, 5544}, {12, 1219, 1, 5544}, {13, 1181, 1, 5544}, {13, 1207, 2, 5544}, {14, 1182, 2, 5544}, {14, 1208, 2, 5544}, {14, 1222, 2, 5544}, {15, 1183, 1, 5544}, {15, 1223, 2, 5544}, {16, 1211, 1, 5544}, {17, 1212, 2, 5544}, {17, 1226, 1, 5544}, {18, 1213, 1, 5544}, {18, 1227, 2, 5544}, {19, 1228, 1, 5544}, {10, 1184, 1, 5545}, {11, 1185, 2, 5545}, {11, 1214, 1, 5545}, {12, 1186, 2, 5545}, {12, 1229, 1, 5545}, {13, 1187, 1, 5545}, {13, 1215, 2, 5545}, {14, 1188, 2, 5545}, {14, 1216, 2, 5545}, {14, 1230, 2, 5545}, {15, 1189, 1, 5545}, {15, 1231, 2, 5545}, {16, 1217, 1, 5545}, {17, 1218, 2, 5545}, {17, 1232, 1, 5545}, {18, 1219, 1, 5545}, {18, 1233, 2, 5545}, {19, 1234, 1, 5545}, {10, 1185, 1, 5546}, {11, 1187, 2, 5546}, {11, 1215, 1, 5546}, {12, 1188, 2, 5546}, {12, 1230, 1, 5546}, {13, 1190, 1, 5546}, {13, 1217, 2, 5546}, {14, 1191, 2, 5546}, {14, 1218, 2, 5546}, {14, 1232, 2, 5546}, {15, 1192, 1, 5546}, {15, 1233, 2, 5546}, {16, 1220, 1, 5546}, {17, 1221, 2, 5546}, {17, 1235, 1, 5546}, {18, 1222, 1, 5546}, {18, 1236, 2, 5546}, {19, 1237, 1, 5546}, {10, 1186, 1, 5547}, {11, 1188, 2, 5547}, {11, 1216, 1, 5547}, {12, 1189, 2, 5547}, {12, 1231, 1, 5547}, {13, 1191, 1, 5547}, {13, 1218, 2, 5547}, {14, 1192, 2, 5547}, {14, 1219, 2, 5547}, {14, 1233, 2, 5547}, {15, 1193, 1, 5547}, {15, 1234, 2, 5547}, {16, 1221, 1, 5547}, {17, 1222, 2, 5547}, {17, 1236, 1, 5547}, {18, 1223, 1, 5547}, {18, 1237, 2, 5547}, {19, 1238, 1, 5547}, {10, 1187, 1, 5548}, {11, 1190, 2, 5548}, {11, 1217, 1, 5548}, {12, 1191, 2, 5548}, {12, 1232, 1, 5548}, {13, 1194, 1, 5548}, {13, 1220, 2, 5548}, {14, 1195, 2, 5548}, {14, 1221, 2, 5548}, {14, 1235, 2, 5548}, {15, 1196, 1, 5548}, {15, 1236, 2, 5548}, {16, 1224, 1, 5548}, {17, 1225, 2, 5548}, {17, 1239, 1, 5548}, {18, 1226, 1, 5548}, {18, 1240, 2, 5548}, {19, 1241, 1, 5548}, {10, 1188, 1, 5549}, {11, 1191, 2, 5549}, {11, 1218, 1, 5549}, {12, 1192, 2, 5549}, {12, 1233, 1, 5549}, {13, 1195, 1, 5549}, {13, 1221, 2, 5549}, {14, 1196, 2, 5549}, {14, 1222, 2, 5549}, {14, 1236, 2, 5549}, {15, 1197, 1, 5549}, {15, 1237, 2, 5549}, {16, 1225, 1, 5549}, {17, 1226, 2, 5549}, {17, 1240, 1, 5549}, {18, 1227, 1, 5549}, {18, 1241, 2, 5549}, {19, 1242, 1, 5549}, {10, 1189, 1, 5550}, {11, 1192, 2, 5550}, {11, 1219, 1, 5550}, {12, 1193, 2, 5550}, {12, 1234, 1, 5550}, {13, 1196, 1, 5550}, {13, 1222, 2, 5550}, {14, 1197, 2, 5550}, {14, 1223, 2, 5550}, {14, 1237, 2, 5550}, {15, 1198, 1, 5550}, {15, 1238, 2, 5550}, {16, 1226, 1, 5550}, {17, 1227, 2, 5550}, {17, 1241, 1, 5550}, {18, 1228, 1, 5550}, {18, 1242, 2, 5550}, {19, 1243, 1, 5550}, {10, 1376, 1, 5551}, {11, 1377, 2, 5551}, {11, 1382, 1, 5551}, {12, 1378, 2, 5551}, {12, 1388, 1, 5551}, {13, 1379, 1, 5551}, {13, 1383, 2, 5551}, {14, 1380, 2, 5551}, {14, 1384, 2, 5551}, {14, 1389, 2, 5551}, {15, 1381, 1, 5551}, {15, 1390, 2, 5551}, {16, 1385, 1, 5551}, {17, 1386, 2, 5551}, {17, 1391, 1, 5551}, {18, 1387, 1, 5551}, {18, 1392, 2, 5551}, {19, 1393, 1, 5551}, {10, 1394, 1, 5552}, {11, 1395, 2, 5552}, {11, 1404, 1, 5552}, {12, 1396, 2, 5552}, {12, 1414, 1, 5552}, {13, 1397, 1, 5552}, {13, 1405, 2, 5552}, {14, 1398, 2, 5552}, {14, 1406, 2, 5552}, {14, 1415, 2, 5552}, {15, 1399, 1, 5552}, {15, 1416, 2, 5552}, {16, 1407, 1, 5552}, {17, 1408, 2, 5552}, {17, 1417, 1, 5552}, {18, 1409, 1, 5552}, {18, 1418, 2, 5552}, {19, 1419, 1, 5552}, {10, 1395, 1, 5553}, {11, 1397, 2, 5553}, {11, 1405, 1, 5553}, {12, 1398, 2, 5553}, {12, 1415, 1, 5553}, {13, 1400, 1, 5553}, {13, 1407, 2, 5553}, {14, 1401, 2, 5553}, {14, 1408, 2, 5553}, {14, 1417, 2, 5553}, {15, 1402, 1, 5553}, {15, 1418, 2, 5553}, {16, 1410, 1, 5553}, {17, 1411, 2, 5553}, {17, 1420, 1, 5553}, {18, 1412, 1, 5553}, {18, 1421, 2, 5553}, {19, 1422, 1, 5553}, {10, 1396, 1, 5554}, {11, 1398, 2, 5554}, {11, 1406, 1, 5554}, {12, 1399, 2, 5554}, {12, 1416, 1, 5554}, {13, 1401, 1, 5554}, {13, 1408, 2, 5554}, {14, 1402, 2, 5554}, {14, 1409, 2, 5554}, {14, 1418, 2, 5554}, {15, 1403, 1, 5554}, {15, 1419, 2, 5554}, {16, 1411, 1, 5554}, {17, 1412, 2, 5554}, {17, 1421, 1, 5554}, {18, 1413, 1, 5554}, {18, 1422, 2, 5554}, {19, 1423, 1, 5554}, {10, 1424, 1, 5555}, {11, 1425, 2, 5555}, {11, 1439, 1, 5555}, {12, 1426, 2, 5555}, {12, 1454, 1, 5555}, {13, 1427, 1, 5555}, {13, 1440, 2, 5555}, {14, 1428, 2, 5555}, {14, 1441, 2, 5555}, {14, 1455, 2, 5555}, {15, 1429, 1, 5555}, {15, 1456, 2, 5555}, {16, 1442, 1, 5555}, {17, 1443, 2, 5555}, {17, 1457, 1, 5555}, {18, 1444, 1, 5555}, {18, 1458, 2, 5555}, {19, 1459, 1, 5555}, {10, 1425, 1, 5556}, {11, 1427, 2, 5556}, {11, 1440, 1, 5556}, {12, 1428, 2, 5556}, {12, 1455, 1, 5556}, {13, 1430, 1, 5556}, {13, 1442, 2, 5556}, {14, 1431, 2, 5556}, {14, 1443, 2, 5556}, {14, 1457, 2, 5556}, {15, 1432, 1, 5556}, {15, 1458, 2, 5556}, {16, 1445, 1, 5556}, {17, 1446, 2, 5556}, {17, 1460, 1, 5556}, {18, 1447, 1, 5556}, {18, 1461, 2, 5556}, {19, 1462, 1, 5556}, {10, 1426, 1, 5557}, {11, 1428, 2, 5557}, {11, 1441, 1, 5557}, {12, 1429, 2, 5557}, {12, 1456, 1, 5557}, {13, 1431, 1, 5557}, {13, 1443, 2, 5557}, {14, 1432, 2, 5557}, {14, 1444, 2, 5557}, {14, 1458, 2, 5557}, {15, 1433, 1, 5557}, {15, 1459, 2, 5557}, {16, 1446, 1, 5557}, {17, 1447, 2, 5557}, {17, 1461, 1, 5557}, {18, 1448, 1, 5557}, {18, 1462,

2, 5557}, {19, 1463, 1, 5557}, {10, 1427, 1, 5558}, {11, 1430, 2, 5558}, {11, 1442, 1, 5558}, {12, 1431, 2, 5558}, {12, 1457, 1, 5558}, {13, 1434, 1, 5558}, {13, 1445, 2, 5558}, {14, 1435, 2, 5558}, {14, 1446, 2, 5558}, {14, 1460, 2, 5558}, {15, 1436, 1, 5558}, {15, 1461, 2, 5558}, {16, 1449, 1, 5558}, {17, 1450, 2, 5558}, {17, 1464, 1, 5558}, {18, 1451, 1, 5558}, {18, 1465, 2, 5558}, {19, 1466, 1, 5558}, {10, 1428, 1, 5559}, {11, 1431, 2, 5559}, {11, 1443, 1, 5559}, {12, 1432, 2, 5559}, {12, 1458, 1, 5559}, {13, 1435, 1, 5559}, {13, 1446, 2, 5559}, {14, 1436, 2, 5559}, {14, 1447, 2, 5559}, {14, 1461, 2, 5559}, {15, 1437, 1, 5559}, {15, 1462, 2, 5559}, {16, 1450, 1, 5559}, {17, 1451, 2, 5559}, {17, 1465, 1, 5559}, {18, 1452, 1, 5559}, {18, 1466, 2, 5559}, {19, 1467, 1, 5559}, {10, 1429, 1, 5560}, {11, 1432, 2, 5560}, {11, 1444, 1, 5560}, {12, 1433, 2, 5560}, {12, 1459, 1, 5560}, {13, 1436, 1, 5560}, {13, 1447, 2, 5560}, {14, 1437, 2, 5560}, {14, 1448, 2, 5560}, {14, 1462, 2, 5560}, {15, 1438, 1, 5560}, {15, 1463, 2, 5560}, {16, 1451, 1, 5560}, {17, 1452, 2, 5560}, {17, 1466, 1, 5560}, {18, 1453, 1, 5560}, {18, 1467, 2, 5560}, {19, 1468, 1, 5560}, {10, 1469, 1, 5561}, {11, 1470, 2, 5561}, {11, 1490, 1, 5561}, {12, 1471, 2, 5561}, {12, 1511, 1, 5561}, {13, 1472, 1, 5561}, {13, 1491, 2, 5561}, {14, 1473, 2, 5561}, {14, 1492, 2, 5561}, {14, 1512, 2, 5561}, {15, 1474, 1, 5561}, {15, 1513, 2, 5561}, {16, 1493, 1, 5561}, {17, 1494, 2, 5561}, {17, 1514, 1, 5561}, {18, 1495, 1, 5561}, {18, 1515, 2, 5561}, {19, 1516, 1, 5561}, {10, 1470, 1, 5562}, {11, 1472, 2, 5562}, {11, 1491, 1, 5562}, {12, 1473, 2, 5562}, {12, 1512, 1, 5562}, {13, 1475, 1, 5562}, {13, 1493, 2, 5562}, {14, 1476, 2, 5562}, {14, 1494, 2, 5562}, {14, 1514, 2, 5562}, {15, 1477, 1, 5562}, {15, 1515, 2, 5562}, {16, 1496, 1, 5562}, {17, 1497, 2, 5562}, {17, 1517, 1, 5562}, {18, 1498, 1, 5562}, {18, 1518, 2, 5562}, {19, 1519, 1, 5562}, {10, 1471, 1, 5563}, {11, 1473, 2, 5563}, {11, 1492, 1, 5563}, {12, 1474, 2, 5563}, {12, 1513, 1, 5563}, {13, 1476, 1, 5563}, {13, 1494, 2, 5563}, {14, 1477, 2, 5563}, {14, 1495, 2, 5563}, {14, 1515, 2, 5563}, {15, 1478, 1, 5563}, {15, 1516, 2, 5563}, {16, 1497, 1, 5563}, {17, 1498, 2, 5563}, {17, 1518, 1, 5563}, {18, 1499, 1, 5563}, {18, 1519, 2, 5563}, {19, 1520, 1, 5563}, {10, 1472, 1, 5564}, {11, 1475, 2, 5564}, {11, 1493, 1, 5564}, {12, 1476, 2, 5564}, {12, 1514, 1, 5564}, {13, 1479, 1, 5564}, {13, 1496, 2, 5564}, {14, 1480, 2, 5564}, {14, 1497, 2, 5564}, {14, 1517, 2, 5564}, {15, 1481, 1, 5564}, {15, 1518, 2, 5564}, {16, 1500, 1, 5564}, {17, 1501, 2, 5564}, {17, 1521, 1, 5564}, {18, 1502, 1, 5564}, {18, 1522, 2, 5564}, {19, 1523, 1, 5564}, {10, 1473, 1, 5565}, {11, 1476, 2, 5565}, {11, 1494, 1, 5565}, {12, 1477, 2, 5565}, {12, 1515, 1, 5565}, {13, 1480, 1, 5565}, {13, 1497, 2, 5565}, {14, 1481, 2, 5565}, {14, 1498, 2, 5565}, {14, 1518, 2, 5565}, {15, 1482, 1, 5565}, {15, 1519, 2, 5565}, {16, 1501, 1, 5565}, {17, 1502, 2, 5565}, {17, 1522, 1, 5565}, {18, 1503, 1, 5565}, {18, 1523, 2, 5565}, {19, 1524, 1, 5565}, {10, 1474, 1, 5566}, {11, 1477, 2, 5566}, {11, 1495, 1, 5566}, {12, 1478, 2, 5566}, {12, 1516, 1, 5566}, {13, 1481, 1, 5566}, {13, 1498, 2, 5566}, {14, 1482, 2, 5566}, {14, 1499, 2, 5566}, {14, 1519, 2, 5566}, {15, 1483, 1, 5566}, {15, 1520, 2, 5566}, {16, 1502, 1, 5566}, {17, 1503, 2, 5566}, {17, 1523, 1, 5566}, {18, 1504, 1, 5566}, {18, 1524, 2, 5566}, {19, 1525, 1, 5566}, {10, 1475, 1, 5567}, {11, 1479, 2, 5567}, {11, 1496, 1, 5567}, {12, 1480, 2, 5567}, {12, 1517, 1, 5567}, {13, 1484, 1, 5567}, {13, 1500, 2, 5567}, {14, 1485, 2, 5567}, {14, 1501, 2, 5567}, {14, 1521, 2, 5567}, {15, 1486, 1, 5567}, {15, 1522, 2, 5567}, {16, 1505, 1, 5567}, {17, 1506, 2, 5567}, {17, 1526, 1, 5567}, {18, 1507, 1, 5567}, {18, 1527, 2, 5567}, {19, 1528, 1, 5567}, {10, 1476, 1, 5568}, {11, 1480, 2, 5568}, {11, 1497, 1, 5568}, {12, 1481, 2, 5568}, {12, 1518, 1, 5568}, {13, 1485, 1, 5568}, {13, 1501, 2, 5568}, {14, 1486, 2, 5568}, {14, 1502, 2, 5568}, {14, 1522, 2, 5568}, {15, 1487, 1, 5568}, {15, 1523, 2, 5568}, {16, 1506, 1, 5568}, {17, 1507, 2, 5568}, {17, 1527, 1, 5568}, {18, 1508, 1, 5568}, {18, 1528, 2, 5568}, {19, 1529, 1, 5568}, {10, 1477, 1, 5569}, {11, 1481, 2, 5569}, {11, 1498, 1, 5569}, {12, 1482, 2, 5569}, {12, 1519, 1, 5569}, {13, 1486, 1, 5569}, {13, 1502, 2, 5569}, {14, 1487, 2, 5569}, {14, 1503, 2, 5569}, {14, 1523, 2, 5569}, {15, 1488, 1, 5569}, {15, 1524, 2, 5569}, {16, 1507, 1, 5569}, {17, 1508, 2, 5569}, {17, 1528, 1, 5569}, {18, 1509, 1, 5569}, {18, 1529, 2, 5569}, {19, 1530, 1, 5569}, {10, 1478, 1, 5570}, {11, 1482, 2, 5570}, {11, 1499, 1, 5570}, {12, 1483, 2, 5570}, {12, 1520, 1, 5570}, {13, 1487, 1, 5570}, {13, 1503, 2, 5570}, {14, 1488, 2, 5570}, {14, 1504, 2, 5570}, {14, 1524, 2, 5570}, {15, 1489, 1, 5570}, {15, 1525, 2, 5570}, {16, 1508, 1, 5570}, {17, 1509, 2, 5570}, {17, 1529, 1, 5570}, {18, 1510, 1, 5570}, {18, 1530, 2, 5570}, {19, 1531, 1, 5570}, {10, 1094, 1, 5571}, {11, 1095, 1, 5571}, {11, 1104, 2, 5571}, {12, 1096, 1, 5571}, {12, 1114, 2, 5571}, {13, 1105, 2, 5571}, {13, 1124, 1, 5571}, {14, 1106, 2, 5571}, {14, 1115, 2, 5571}, {14, 1134, 2, 5571}, {15, 1116, 2, 5571}, {15, 1144, 1, 5571}, {16, 1125, 1, 5571}, {17, 1126, 1, 5571}, {17, 1135, 2, 5571}, {18, 1136, 2, 5571}, {18, 1145, 1, 5571}, {19, 1146, 1, 5571}, {10, 1095, 1, 5572}, {11, 1097, 1, 5572}, {11, 1105, 2, 5572}, {12, 1098, 1, 5572}, {12, 1115, 2, 5572}, {13, 1107, 2, 5572}, {13, 1125, 1, 5572}, {14, 1108, 2, 5572}, {14, 1117, 2, 5572}, {14, 1135, 2, 5572}, {15, 1118, 2, 5572}, {15, 1145, 1, 5572}, {16, 1127, 1, 5572}, {17, 1128, 1, 5572}, {17, 1137, 2, 5572}, {18, 1138, 2, 5572}, {18, 1147, 1, 5572}, {19, 1148, 1, 5572}, {10, 1096, 1, 5573}, {11, 1098, 1, 5573}, {11, 1106, 2, 5573}, {12, 1099, 1, 5573}, {12, 1116, 2, 5573}, {13, 1108, 2, 5573}, {13, 1126, 1, 5573}, {14, 1109, 2, 5573}, {14, 1118, 2, 5573}, {14, 1136, 2, 5573}, {15, 1119, 2, 5573}, {15, 1146, 1, 5573}, {16, 1128, 1, 5573}, {17, 1129, 1, 5573}, {17, 1138, 2, 5573}, {18, 1139, 2, 5573}, {18, 1148, 1, 5573}, {19, 1149, 1, 5573}, {10, 1097, 1, 5574}, {11, 1100, 1, 5574}, {11, 1107, 2, 5574}, {12, 1101, 1,

5574}, {12, 1117, 2, 5574}, {13, 1110, 2, 5574}, {13, 1127, 1, 5574}, {14, 1111, 2, 5574}, {14, 1120, 2, 5574}, {14, 1137, 2, 5574}, {15, 1121, 2, 5574}, {15, 1147, 1, 5574}, {16, 1130, 1, 5574}, {17, 1131, 1, 5574}, {17, 1140, 2, 5574}, {18, 1141, 2, 5574}, {18, 1150, 1, 5574}, {19, 1151, 1, 5574}, {10, 1098, 1, 5575}, {11, 1101, 1, 5575}, {11, 1108, 2, 5575}, {12, 1102, 1, 5575}, {12, 1118, 2, 5575}, {13, 1111, 2, 5575}, {13, 1128, 1, 5575}, {14, 1112, 2, 5575}, {14, 1121, 2, 5575}, {14, 1138, 2, 5575}, {15, 1122, 2, 5575}, {15, 1148, 1, 5575}, {16, 1131, 1, 5575}, {17, 1132, 1, 5575}, {17, 1141, 2, 5575}, {18, 1142, 2, 5575}, {18, 1151, 1, 5575}, {19, 1152, 1, 5575}, {10, 1099, 1, 5576}, {11, 1102, 1, 5576}, {11, 1109, 2, 5576}, {12, 1103, 1, 5576}, {12, 1119, 2, 5576}, {13, 1112, 2, 5576}, {13, 1129, 1, 5576}, {14, 1113, 2, 5576}, {14, 1122, 2, 5576}, {14, 1139, 2, 5576}, {15, 1123, 2, 5576}, {15, 1149, 1, 5576}, {16, 1132, 1, 5576}, {17, 1133, 1, 5576}, {17, 1142, 2, 5576}, {18, 1143, 2, 5576}, {18, 1152, 1, 5576}, {19, 1153, 1, 5576}, {10, 1588, 1, 5577}, {11, 1589, 3, 5577}, {12, 1590, 3, 5577}, {13, 1591, 1, 5577}, {13, 1598, 2, 5577}, {14, 1592, 2, 5577}, {14, 1599, 4, 5577}, {15, 1593, 1, 5577}, {15, 1607, 2, 5577}, {16, 1600, 1, 5577}, {17, 1601, 2, 5577}, {17, 1608, 1, 5577}, {18, 1602, 1, 5577}, {18, 1609, 2, 5577}, {19, 1610, 1, 5577}, {10, 1589, 1, 5578}, {11, 1591, 2, 5578}, {11, 1598, 1, 5578}, {12, 1592, 2, 5578}, {12, 1599, 1, 5578}, {13, 1594, 1, 5578}, {13, 1600, 2, 5578}, {14, 1595, 2, 5578}, {14, 1601, 2, 5578}, {14, 1608, 2, 5578}, {15, 1596, 1, 5578}, {15, 1609, 2, 5578}, {16, 1603, 1, 5578}, {17, 1604, 2, 5578}, {17, 1611, 1, 5578}, {18, 1605, 1, 5578}, {18, 1612, 2, 5578}, {19, 1613, 1, 5578}, {10, 1590, 1, 5579}, {11, 1592, 2, 5579}, {11, 1599, 1, 5579}, {12, 1593, 2, 5579}, {12, 1607, 1, 5579}, {13, 1595, 1, 5579}, {13, 1601, 2, 5579}, {14, 1596, 2, 5579}, {14, 1602, 2, 5579}, {14, 1609, 2, 5579}, {15, 1597, 1, 5579}, {15, 1610, 2, 5579}, {16, 1604, 1, 5579}, {17, 1605, 2, 5579}, {17, 1612, 1, 5579}, {18, 1606, 1, 5579}, {18, 1613, 2, 5579}, {19, 1614, 1, 5579}, {10, 1589, 1, 5580}, {11, 1591, 1, 5580}, {11, 1598, 2, 5580}, {12, 1592, 1, 5580}, {12, 1599, 2, 5580}, {13, 1600, 3, 5580}, {14, 1601, 4, 5580}, {14, 1608, 2, 5580}, {15, 1602, 1, 5580}, {15, 1609, 2, 5580}, {16, 1615, 1, 5580}, {17, 1616, 3, 5580}, {18, 1617, 1, 5580}, {18, 1622, 2, 5580}, {19, 1623, 1, 5580}, {10, 1598, 1, 5581}, {11, 1600, 3, 5581}, {12, 1601, 3, 5581}, {13, 1603, 1, 5581}, {13, 1615, 2, 5581}, {14, 1604, 2, 5581}, {14, 1616, 4, 5581}, {15, 1605, 1, 5581}, {15, 1622, 2, 5581}, {16, 1618, 1, 5581}, {17, 1619, 2, 5581}, {17, 1624, 1, 5581}, {18, 1620, 1, 5581}, {18, 1625, 2, 5581}, {19, 1626, 1, 5581}, {10, 1599, 1, 5582}, {11, 1601, 2, 5582}, {11, 1608, 1, 5582}, {12, 1602, 2, 5582}, {12, 1609, 1, 5582}, {13, 1604, 1, 5582}, {13, 1616, 2, 5582}, {14, 1605, 2, 5582}, {14, 1617, 2, 5582}, {14, 1622, 2, 5582}, {15, 1606, 1, 5582}, {15, 1623, 2, 5582}, {16, 1619, 1, 5582}, {17, 1620, 2, 5582}, {17, 1625, 1, 5582}, {18, 1621, 1, 5582}, {18, 1626, 2, 5582}, {19, 1627, 1, 5582}, {10, 1590, 1, 5583}, {11, 1592, 1, 5583}, {11, 1599, 2, 5583}, {12, 1593, 1, 5583}, {12, 1607, 2, 5583}, {13, 1601, 2, 5583}, {13, 1608, 1, 5583}, {14, 1602, 2, 5583}, {14, 1609, 4, 5583}, {15, 1610, 3, 5583}, {16, 1616, 1, 5583}, {17, 1617, 1, 5583}, {17, 1622, 2, 5583}, {18, 1623, 3, 5583}, {19, 1628, 1, 5583}, {10, 1599, 1, 5584}, {11, 1601, 1, 5584}, {11, 1608, 2, 5584}, {12, 1602, 1, 5584}, {12, 1609, 2, 5584}, {13, 1611, 1, 5584}, {13, 1616, 2, 5584}, {14, 1612, 2, 5584}, {14, 1617, 2, 5584}, {14, 1622, 2, 5584}, {15, 1613, 1, 5584}, {15, 1623, 2, 5584}, {16, 1624, 1, 5584}, {17, 1625, 2, 5584}, {17, 1629, 1, 5584}, {18, 1626, 1, 5584}, {18, 1630, 2, 5584}, {19, 1631, 1, 5584}, {10, 1607, 1, 5585}, {11, 1609, 3, 5585}, {12, 1610, 3, 5585}, {13, 1612, 1, 5585}, {13, 1622, 2, 5585}, {14, 1613, 2, 5585}, {14, 1623, 4, 5585}, {15, 1614, 1, 5585}, {15, 1628, 2, 5585}, {16, 1625, 1, 5585}, {17, 1626, 2, 5585}, {17, 1630, 1, 5585}, {18, 1627, 1, 5585}, {18, 1631, 2, 5585}, {19, 1632, 1, 5585}, {10, 1591, 1, 5586}, {11, 1594, 1, 5586}, {11, 1600, 2, 5586}, {12, 1595, 1, 5586}, {12, 1608, 2, 5586}, {13, 1603, 2, 5586}, {13, 1615, 1, 5586}, {14, 1604, 2, 5586}, {14, 1611, 2, 5586}, {14, 1616, 2, 5586}, {15, 1612, 2, 5586}, {15, 1617, 1, 5586}, {16, 1618, 1, 5586}, {17, 1619, 1, 5586}, {17, 1624, 2, 5586}, {18, 1625, 2, 5586}, {18, 1629, 1, 5586}, {19, 1630, 1, 5586}, {10, 1600, 1, 5587}, {11, 1603, 1, 5587}, {11, 1615, 2, 5587}, {12, 1604, 1, 5587}, {12, 1616, 2, 5587}, {13, 1618, 3, 5587}, {14, 1619, 4, 5587}, {14, 1624, 2, 5587}, {15, 1620, 1, 5587}, {15, 1625, 2, 5587}, {16, 1633, 1, 5587}, {17, 1634, 3, 5587}, {18, 1635, 1, 5587}, {18, 1637, 2, 5587}, {19, 1638, 1, 5587}, {10, 1608, 1, 5588}, {11, 1611, 1, 5588}, {11, 1616, 2, 5588}, {12, 1612, 1, 5588}, {12, 1617, 2, 5588}, {13, 1619, 1, 5588}, {13, 1624, 2, 5588}, {14, 1620, 2, 5588}, {14, 1625, 2, 5588}, {14, 1629, 2, 5588}, {15, 1621, 1, 5588}, {15, 1630, 2, 5588}, {16, 1634, 1, 5588}, {17, 1635, 2, 5588}, {17, 1637, 1, 5588}, {18, 1636, 1, 5588}, {18, 1638, 2, 5588}, {19, 1639, 1, 5588}, {10, 1592, 1, 5589}, {11, 1595, 1, 5589}, {11, 1601, 2, 5589}, {12, 1596, 1, 5589}, {12, 1609, 2, 5589}, {13, 1604, 2, 5589}, {13, 1616, 1, 5589}, {14, 1605, 2, 5589}, {14, 1612, 2, 5589}, {14, 1622, 2, 5589}, {15, 1613, 2, 5589}, {15, 1623, 1, 5589}, {16, 1619, 1, 5589}, {17, 1620, 1, 5589}, {17, 1625, 2, 5589}, {18, 1626, 2, 5589}, {18, 1630, 1, 5589}, {19, 1631, 1, 5589}, {10, 1601, 1, 5590}, {11, 1604, 1, 5590}, {11, 1616, 2, 5590}, {12, 1605, 1, 5590}, {12, 1622, 2, 5590}, {13, 1619, 2, 5590}, {13, 1624, 1, 5590}, {14, 1620, 2, 5590}, {14, 1625, 4, 5590}, {15, 1626, 3, 5590}, {16, 1634, 1, 5590}, {17, 1635, 1, 5590}, {17, 1637, 2, 5590}, {18, 1638, 3, 5590}, {19, 1640, 1, 5590}, {10, 1609, 1, 5591}, {11, 1612, 1, 5591}, {11, 1622, 2, 5591}, {12, 1613, 1, 5591}, {12, 1623, 2, 5591}, {13, 1625, 3, 5591}, {14, 1626, 4, 5591}, {14, 1630, 2, 5591}, {15, 1627, 1, 5591}, {15, 1631, 2, 5591}, {16, 1637, 1, 5591}, {17, 1638, 3, 5591},

{18, 1639, 1, 5591}, {18, 1640, 2, 5591}, {19, 1641, 1, 5591}, {10, 1593, 1, 5592}, {11, 1596, 1, 5592}, {11, 1602, 2, 5592}, {12, 1597, 1, 5592}, {12, 1610, 2, 5592}, {13, 1605, 2, 5592}, {13, 1617, 1, 5592}, {14, 1606, 2, 5592}, {14, 1613, 2, 5592}, {14, 1623, 2, 5592}, {15, 1614, 2, 5592}, {15, 1628, 1, 5592}, {16, 1620, 1, 5592}, {17, 1621, 1, 5592}, {17, 1626, 2, 5592}, {18, 1627, 2, 5592}, {18, 1631, 1, 5592}, {19, 1632, 1, 5592}, {10, 1602, 1, 5593}, {11, 1605, 1, 5593}, {11, 1617, 2, 5593}, {12, 1606, 1, 5593}, {12, 1623, 2, 5593}, {13, 1620, 2, 5593}, {13, 1629, 1, 5593}, {14, 1621, 2, 5593}, {14, 1626, 2, 5593}, {14, 1630, 2, 5593}, {15, 1627, 2, 5593}, {15, 1631, 1, 5593}, {16, 1635, 1, 5593}, {17, 1636, 1, 5593}, {17, 1638, 2, 5593}, {18, 1639, 2, 5593}, {18, 1640, 1, 5593}, {19, 1641, 1, 5593}, {10, 1610, 1, 5594}, {11, 1613, 1, 5594}, {11, 1623, 2, 5594}, {12, 1614, 1, 5594}, {12, 1628, 2, 5594}, {13, 1626, 2, 5594}, {13, 1630, 1, 5594}, {14, 1627, 2, 5594}, {14, 1631, 4, 5594}, {15, 1632, 3, 5594}, {16, 1638, 1, 5594}, {17, 1639, 1, 5594}, {17, 1640, 2, 5594}, {18, 1641, 3, 5594}, {19, 1642, 1, 5594}, {10, 1793, 1, 5595}, {11, 1794, 3, 5595}, {12, 1795, 3, 5595}, {13, 1796, 1, 5595}, {13, 1799, 2, 5595}, {14, 1797, 2, 5595}, {14, 1800, 4, 5595}, {15, 1798, 1, 5595}, {15, 1804, 2, 5595}, {16, 1801, 1, 5595}, {17, 1802, 2, 5595}, {17, 1805, 1, 5595}, {18, 1803, 1, 5595}, {18, 1806, 2, 5595}, {19, 1807, 1, 5595}, {10, 1794, 1, 5596}, {11, 1796, 1, 5596}, {11, 1799, 2, 5596}, {12, 1797, 1, 5596}, {12, 1800, 2, 5596}, {13, 1801, 3, 5596}, {14, 1802, 4, 5596}, {14, 1805, 2, 5596}, {15, 1803, 1, 5596}, {15, 1806, 2, 5596}, {16, 1808, 1, 5596}, {17, 1809, 3, 5596}, {18, 1810, 1, 5596}, {18, 1811, 2, 5596}, {19, 1812, 1, 5596}, {10, 1795, 1, 5597}, {11, 1797, 1, 5597}, {11, 1800, 2, 5597}, {12, 1798, 1, 5597}, {12, 1804, 2, 5597}, {13, 1802, 2, 5597}, {13, 1805, 1, 5597}, {14, 1803, 2, 5597}, {14, 1806, 4, 5597}, {15, 1807, 3, 5597}, {16, 1809, 1, 5597}, {17, 1810, 1, 5597}, {17, 1811, 2, 5597}, {18, 1812, 3, 5597}, {19, 1813, 1, 5597}, {10, 1814, 1, 5598}, {11, 1815, 2, 5598}, {11, 1824, 1, 5598}, {12, 1816, 2, 5598}, {12, 1834, 1, 5598}, {13, 1817, 1, 5598}, {13, 1825, 2, 5598}, {14, 1818, 2, 5598}, {14, 1826, 2, 5598}, {14, 1835, 2, 5598}, {15, 1819, 1, 5598}, {15, 1836, 2, 5598}, {16, 1827, 1, 5598}, {17, 1828, 2, 5598}, {17, 1837, 1, 5598}, {18, 1829, 1, 5598}, {18, 1838, 2, 5598}, {19, 1839, 1, 5598}, {10, 1815, 1, 5599}, {11, 1817, 2, 5599}, {11, 1825, 1, 5599}, {12, 1818, 2, 5599}, {12, 1835, 1, 5599}, {13, 1820, 1, 5599}, {13, 1827, 2, 5599}, {14, 1821, 2, 5599}, {14, 1828, 2, 5599}, {14, 1837, 2, 5599}, {15, 1822, 1, 5599}, {15, 1838, 2, 5599}, {16, 1830, 1, 5599}, {17, 1831, 2, 5599}, {17, 1840, 1, 5599}, {18, 1832, 1, 5599}, {18, 1841, 2, 5599}, {19, 1842, 1, 5599}, {10, 1816, 1, 5600}, {11, 1818, 2, 5600}, {11, 1826, 1, 5600}, {12, 1819, 2, 5600}, {12, 1836, 1, 5600}, {13, 1821, 1, 5600}, {13, 1828, 2, 5600}, {14, 1822, 2, 5600}, {14, 1829, 2, 5600}, {14, 1838, 2, 5600}, {15, 1823, 1, 5600}, {15, 1839, 2, 5600}, {16, 1831, 1, 5600}, {17, 1832, 2, 5600}, {17, 1841, 1, 5600}, {18, 1833, 1, 5600}, {18, 1842, 2, 5600}, {19, 1843, 1, 5600}, {10, 1824, 1, 5601}, {11, 1825, 2, 5601}, {11, 1844, 1, 5601}, {12, 1826, 2, 5601}, {12, 1854, 1, 5601}, {13, 1827, 1, 5601}, {13, 1845, 2, 5601}, {14, 1828, 2, 5601}, {14, 1846, 2, 5601}, {14, 1855, 2, 5601}, {15, 1829, 1, 5601}, {15, 1856, 2, 5601}, {16, 1847, 1, 5601}, {17, 1848, 2, 5601}, {17, 1857, 1, 5601}, {18, 1849, 1, 5601}, {18, 1858, 2, 5601}, {19, 1859, 1, 5601}, {10, 1825, 1, 5602}, {11, 1827, 2, 5602}, {11, 1845, 1, 5602}, {12, 1828, 2, 5602}, {12, 1855, 1, 5602}, {13, 1830, 1, 5602}, {13, 1847, 2, 5602}, {14, 1831, 2, 5602}, {14, 1848, 2, 5602}, {14, 1857, 2, 5602}, {15, 1832, 1, 5602}, {15, 1858, 2, 5602}, {16, 1850, 1, 5602}, {17, 1851, 2, 5602}, {17, 1860, 1, 5602}, {18, 1852, 1, 5602}, {18, 1861, 2, 5602}, {19, 1862, 1, 5602}, {10, 1826, 1, 5603}, {11, 1828, 2, 5603}, {11, 1846, 1, 5603}, {12, 1829, 2, 5603}, {12, 1856, 1, 5603}, {13, 1831, 1, 5603}, {13, 1848, 2, 5603}, {14, 1832, 2, 5603}, {14, 1849, 2, 5603}, {14, 1858, 2, 5603}, {15, 1833, 1, 5603}, {15, 1859, 2, 5603}, {16, 1851, 1, 5603}, {17, 1852, 2, 5603}, {17, 1861, 1, 5603}, {18, 1853, 1, 5603}, {18, 1862, 2, 5603}, {19, 1863, 1, 5603}, {10, 1834, 1, 5604}, {11, 1835, 2, 5604}, {11, 1854, 1, 5604}, {12, 1836, 2, 5604}, {12, 1864, 1, 5604}, {13, 1837, 1, 5604}, {13, 1855, 2, 5604}, {14, 1838, 2, 5604}, {14, 1856, 2, 5604}, {14, 1865, 2, 5604}, {15, 1839, 1, 5604}, {15, 1866, 2, 5604}, {16, 1857, 1, 5604}, {17, 1858, 2, 5604}, {17, 1867, 1, 5604}, {18, 1859, 1, 5604}, {18, 1868, 2, 5604}, {19, 1869, 1, 5604}, {10, 1835, 1, 5605}, {11, 1837, 2, 5605}, {11, 1855, 1, 5605}, {12, 1838, 2, 5605}, {12, 1865, 1, 5605}, {13, 1840, 1, 5605}, {13, 1857, 2, 5605}, {14, 1841, 2, 5605}, {14, 1858, 2, 5605}, {14, 1867, 2, 5605}, {15, 1842, 1, 5605}, {15, 1868, 2, 5605}, {16, 1860, 1, 5605}, {17, 1861, 2, 5605}, {17, 1870, 1, 5605}, {18, 1862, 1, 5605}, {18, 1871, 2, 5605}, {19, 1872, 1, 5605}, {10, 1836, 1, 5606}, {11, 1838, 2, 5606}, {11, 1856, 1, 5606}, {12, 1839, 2, 5606}, {12, 1866, 1, 5606}, {13, 1841, 1, 5606}, {13, 1858, 2, 5606}, {14, 1842, 2, 5606}, {14, 1859, 2, 5606}, {14, 1868, 2, 5606}, {15, 1843, 1, 5606}, {15, 1869, 2, 5606}, {16, 1861, 1, 5606}, {17, 1862, 2, 5606}, {17, 1871, 1, 5606}, {18, 1863, 1, 5606}, {18, 1872, 2, 5606}, {19, 1873, 1, 5606}, {10, 1874, 1, 5607}, {11, 1875, 2, 5607}, {11, 1889, 1, 5607}, {12, 1876, 2, 5607}, {12, 1904, 1, 5607}, {13, 1877, 1, 5607}, {13, 1890, 2, 5607}, {14, 1878, 2, 5607}, {14, 1891, 2, 5607}, {14, 1905, 2, 5607}, {15, 1879, 1, 5607}, {15, 1906, 2, 5607}, {16, 1892, 1, 5607}, {17, 1893, 2, 5607}, {17, 1907, 1, 5607}, {18, 1894, 1, 5607}, {18, 1908, 2, 5607}, {19, 1909, 1, 5607}, {10, 1875, 1, 5608}, {11, 1877, 2, 5608}, {11, 1890, 1, 5608}, {12, 1878, 2, 5608}, {12, 1905, 1, 5608}, {13, 1880, 1, 5608}, {13, 1892, 2, 5608}, {14, 1881, 2, 5608}, {14, 1893, 2, 5608}, {14, 1907, 2, 5608}, {15, 1882, 1, 5608}, {15, 1908, 2, 5608}, {16, 1895, 1, 5608}, {17, 1896, 2, 5608}, {17,

1910, 1, 5608}, {18, 1897, 1, 5608}, {18, 1911, 2, 5608}, {19, 1912, 1, 5608}, {10, 1876,
1, 5609}, {11, 1878, 2, 5609}, {11, 1891, 1, 5609}, {12, 1879, 2, 5609}, {12, 1906, 1,
5609}, {13, 1881, 1, 5609}, {13, 1893, 2, 5609}, {14, 1882, 2, 5609}, {14, 1894, 2, 5609},
{14, 1908, 2, 5609}, {15, 1883, 1, 5609}, {15, 1909, 2, 5609}, {16, 1896, 1, 5609}, {17,
1897, 2, 5609}, {17, 1911, 1, 5609}, {18, 1898, 1, 5609}, {18, 1912, 2, 5609}, {19, 1913,
1, 5609}, {10, 1877, 1, 5610}, {11, 1880, 2, 5610}, {11, 1892, 1, 5610}, {12, 1881, 2,
5610}, {12, 1907, 1, 5610}, {13, 1884, 1, 5610}, {13, 1895, 2, 5610}, {14, 1885, 2, 5610},
{14, 1896, 2, 5610}, {14, 1910, 2, 5610}, {15, 1886, 1, 5610}, {15, 1911, 2, 5610}, {16,
1899, 1, 5610}, {17, 1900, 2, 5610}, {17, 1914, 1, 5610}, {18, 1901, 1, 5610}, {18, 1915,
2, 5610}, {19, 1916, 1, 5610}, {10, 1878, 1, 5611}, {11, 1881, 2, 5611}, {11, 1893, 1,
5611}, {12, 1882, 2, 5611}, {12, 1908, 1, 5611}, {13, 1885, 1, 5611}, {13, 1896, 2, 5611},
{14, 1886, 2, 5611}, {14, 1897, 2, 5611}, {14, 1911, 2, 5611}, {15, 1887, 1, 5611}, {15,
1912, 2, 5611}, {16, 1900, 1, 5611}, {17, 1901, 2, 5611}, {17, 1915, 1, 5611}, {18, 1902,
1, 5611}, {18, 1916, 2, 5611}, {19, 1917, 1, 5611}, {10, 1879, 1, 5612}, {11, 1882, 2,
5612}, {11, 1894, 1, 5612}, {12, 1883, 2, 5612}, {12, 1909, 1, 5612}, {13, 1886, 1, 5612},
{13, 1897, 2, 5612}, {14, 1887, 2, 5612}, {14, 1898, 2, 5612}, {14, 1912, 2, 5612}, {15,
1888, 1, 5612}, {15, 1913, 2, 5612}, {16, 1901, 1, 5612}, {17, 1902, 2, 5612}, {17, 1916,
1, 5612}, {18, 1903, 1, 5612}, {18, 1917, 2, 5612}, {19, 1918, 1, 5612}, {10, 1889, 1,
5613}, {11, 1890, 2, 5613}, {11, 1919, 1, 5613}, {12, 1891, 2, 5613}, {12, 1934, 1, 5613},
{13, 1892, 1, 5613}, {13, 1920, 2, 5613}, {14, 1893, 2, 5613}, {14, 1921, 2, 5613}, {14,
1935, 2, 5613}, {15, 1894, 1, 5613}, {15, 1936, 2, 5613}, {16, 1922, 1, 5613}, {17, 1923,
2, 5613}, {17, 1937, 1, 5613}, {18, 1924, 1, 5613}, {18, 1938, 2, 5613}, {19, 1939, 1,
5613}, {10, 1890, 1, 5614}, {11, 1892, 2, 5614}, {11, 1920, 1, 5614}, {12, 1893, 2, 5614},
{12, 1935, 1, 5614}, {13, 1895, 1, 5614}, {13, 1922, 2, 5614}, {14, 1896, 2, 5614}, {14,
1923, 2, 5614}, {14, 1937, 2, 5614}, {15, 1897, 1, 5614}, {15, 1938, 2, 5614}, {16, 1925,
1, 5614}, {17, 1926, 2, 5614}, {17, 1940, 1, 5614}, {18, 1927, 1, 5614}, {18, 1941, 2,
5614}, {19, 1942, 1, 5614}, {10, 1891, 1, 5615}, {11, 1893, 2, 5615}, {11, 1921, 1, 5615},
{12, 1894, 2, 5615}, {12, 1936, 1, 5615}, {13, 1896, 1, 5615}, {13, 1923, 2, 5615}, {14,
1897, 2, 5615}, {14, 1924, 2, 5615}, {14, 1938, 2, 5615}, {15, 1898, 1, 5615}, {15, 1939,
2, 5615}, {16, 1926, 1, 5615}, {17, 1927, 2, 5615}, {17, 1941, 1, 5615}, {18, 1928, 1,
5615}, {18, 1942, 2, 5615}, {19, 1943, 1, 5615}, {10, 1892, 1, 5616}, {11, 1895, 2, 5616},
{11, 1922, 1, 5616}, {12, 1896, 2, 5616}, {12, 1937, 1, 5616}, {13, 1899, 1, 5616}, {13,
1925, 2, 5616}, {14, 1900, 2, 5616}, {14, 1926, 2, 5616}, {14, 1940, 2, 5616}, {15, 1901,
1, 5616}, {15, 1941, 2, 5616}, {16, 1929, 1, 5616}, {17, 1930, 2, 5616}, {17, 1944, 1,
5616}, {18, 1931, 1, 5616}, {18, 1945, 2, 5616}, {19, 1946, 1, 5616}, {10, 1893, 1, 5617},
{11, 1896, 2, 5617}, {11, 1923, 1, 5617}, {12, 1897, 2, 5617}, {12, 1938, 1, 5617}, {13,
1900, 1, 5617}, {13, 1926, 2, 5617}, {14, 1901, 2, 5617}, {14, 1927, 2, 5617}, {14, 1941,
2, 5617}, {15, 1902, 1, 5617}, {15, 1942, 2, 5617}, {16, 1930, 1, 5617}, {17, 1931, 2,
5617}, {17, 1945, 1, 5617}, {18, 1932, 1, 5617}, {18, 1946, 2, 5617}, {19, 1947, 1, 5617},
{10, 1894, 1, 5618}, {11, 1897, 2, 5618}, {11, 1924, 1, 5618}, {12, 1898, 2, 5618}, {12,
1939, 1, 5618}, {13, 1901, 1, 5618}, {13, 1927, 2, 5618}, {14, 1902, 2, 5618}, {14, 1928,
2, 5618}, {14, 1942, 2, 5618}, {15, 1903, 1, 5618}, {15, 1943, 2, 5618}, {16, 1931, 1,
5618}, {17, 1932, 2, 5618}, {17, 1946, 1, 5618}, {18, 1933, 1, 5618}, {18, 1947, 2, 5618},
{19, 1948, 1, 5618}, {10, 1904, 1, 5619}, {11, 1905, 2, 5619}, {11, 1934, 1, 5619}, {12,
1906, 2, 5619}, {12, 1949, 1, 5619}, {13, 1907, 1, 5619}, {13, 1935, 2, 5619}, {14, 1908,
2, 5619}, {14, 1936, 2, 5619}, {14, 1950, 2, 5619}, {15, 1909, 1, 5619}, {15, 1951, 2,
5619}, {16, 1937, 1, 5619}, {17, 1938, 2, 5619}, {17, 1952, 1, 5619}, {18, 1939, 1, 5619},
{18, 1953, 2, 5619}, {19, 1954, 1, 5619}, {10, 1905, 1, 5620}, {11, 1907, 2, 5620}, {11,
1935, 1, 5620}, {12, 1908, 2, 5620}, {12, 1950, 1, 5620}, {13, 1910, 1, 5620}, {13, 1937,
2, 5620}, {14, 1911, 2, 5620}, {14, 1938, 2, 5620}, {14, 1952, 2, 5620}, {15, 1912, 1,
5620}, {15, 1953, 2, 5620}, {16, 1940, 1, 5620}, {17, 1941, 2, 5620}, {17, 1955, 1, 5620},
{18, 1942, 1, 5620}, {18, 1956, 2, 5620}, {19, 1957, 1, 5620}, {10, 1906, 1, 5621}, {11,
1908, 2, 5621}, {11, 1936, 1, 5621}, {12, 1909, 2, 5621}, {12, 1951, 1, 5621}, {13, 1911,
1, 5621}, {13, 1938, 2, 5621}, {14, 1912, 2, 5621}, {14, 1939, 2, 5621}, {14, 1953, 2,
5621}, {15, 1913, 1, 5621}, {15, 1954, 2, 5621}, {16, 1941, 1, 5621}, {17, 1942, 2, 5621},
{17, 1956, 1, 5621}, {18, 1943, 1, 5621}, {18, 1957, 2, 5621}, {19, 1958, 1, 5621}, {10,
1907, 1, 5622}, {11, 1910, 2, 5622}, {11, 1937, 1, 5622}, {12, 1911, 2, 5622}, {12, 1952,
1, 5622}, {13, 1914, 1, 5622}, {13, 1940, 2, 5622}, {14, 1915, 2, 5622}, {14, 1941, 2,
5622}, {14, 1955, 2, 5622}, {15, 1916, 1, 5622}, {15, 1956, 2, 5622}, {16, 1944, 1, 5622},
{17, 1945, 2, 5622}, {17, 1959, 1, 5622}, {18, 1946, 1, 5622}, {18, 1960, 2, 5622}, {19,
1961, 1, 5622}, {10, 1908, 1, 5623}, {11, 1911, 2, 5623}, {11, 1938, 1, 5623}, {12, 1912,
2, 5623}, {12, 1953, 1, 5623}, {13, 1915, 1, 5623}, {13, 1941, 2, 5623}, {14, 1916, 2,
5623}, {14, 1942, 2, 5623}, {14, 1956, 2, 5623}, {15, 1917, 1, 5623}, {15, 1957, 2, 5623},
{16, 1945, 1, 5623}, {17, 1946, 2, 5623}, {17, 1960, 1, 5623}, {18, 1947, 1, 5623}, {18,
1961, 2, 5623}, {19, 1962, 1, 5623}, {10, 1909, 1, 5624}, {11, 1912, 2, 5624}, {11, 1939,
1, 5624}, {12, 1913, 2, 5624}, {12, 1954, 1, 5624}, {13, 1916, 1, 5624}, {13, 1942, 2,
5624}, {14, 1917, 2, 5624}, {14, 1943, 2, 5624}, {14, 1957, 2, 5624}, {15, 1918, 1, 5624},
{15, 1958, 2, 5624}, {16, 1946, 1, 5624}, {17, 1947, 2, 5624}, {17, 1961, 1, 5624}, {18,
1948, 1, 5624}, {18, 1962, 2, 5624}, {19, 1963, 1, 5624}, {10, 1970, 1, 5625}, {11, 1971,

2, 5625}, {11, 1976, 1, 5625}, {12, 1972, 2, 5625}, {12, 1982, 1, 5625}, {13, 1973, 1, 5625}, {13, 1977, 2, 5625}, {14, 1974, 2, 5625}, {14, 1978, 2, 5625}, {14, 1983, 2, 5625}, {15, 1975, 1, 5625}, {15, 1984, 2, 5625}, {16, 1979, 1, 5625}, {17, 1980, 2, 5625}, {17, 1985, 1, 5625}, {18, 1981, 1, 5625}, {18, 1986, 2, 5625}, {19, 1987, 1, 5625}, {10, 1988, 1, 5626}, {11, 1989, 2, 5626}, {11, 1998, 1, 5626}, {12, 1990, 2, 5626}, {12, 2008, 1, 5626}, {13, 1991, 1, 5626}, {13, 1999, 2, 5626}, {14, 1992, 2, 5626}, {14, 2000, 2, 5626}, {14, 2009, 2, 5626}, {15, 1993, 1, 5626}, {15, 2010, 2, 5626}, {16, 2001, 1, 5626}, {17, 2002, 2, 5626}, {17, 2011, 1, 5626}, {18, 2003, 1, 5626}, {18, 2012, 2, 5626}, {19, 2013, 1, 5626}, {10, 1989, 1, 5627}, {11, 1991, 2, 5627}, {11, 1999, 1, 5627}, {12, 1992, 2, 5627}, {12, 2009, 1, 5627}, {13, 1994, 1, 5627}, {13, 2001, 2, 5627}, {14, 1995, 2, 5627}, {14, 2002, 2, 5627}, {14, 2011, 2, 5627}, {15, 1996, 1, 5627}, {15, 2012, 2, 5627}, {16, 2004, 1, 5627}, {17, 2005, 2, 5627}, {17, 2014, 1, 5627}, {18, 2006, 1, 5627}, {18, 2015, 2, 5627}, {19, 2016, 1, 5627}, {10, 1990, 1, 5628}, {11, 1992, 2, 5628}, {11, 2000, 1, 5628}, {12, 1993, 2, 5628}, {12, 2010, 1, 5628}, {13, 1995, 1, 5628}, {13, 2002, 2, 5628}, {14, 1996, 2, 5628}, {14, 2003, 2, 5628}, {14, 2012, 2, 5628}, {15, 1997, 1, 5628}, {15, 2013, 2, 5628}, {16, 2005, 1, 5628}, {17, 2006, 2, 5628}, {17, 2015, 1, 5628}, {18, 2007, 1, 5628}, {18, 2016, 2, 5628}, {19, 2017, 1, 5628}, {10, 2018, 1, 5629}, {11, 2019, 2, 5629}, {11, 2033, 1, 5629}, {12, 2020, 2, 5629}, {12, 2048, 1, 5629}, {13, 2021, 1, 5629}, {13, 2034, 2, 5629}, {14, 2022, 2, 5629}, {14, 2035, 2, 5629}, {14, 2049, 2, 5629}, {15, 2023, 1, 5629}, {15, 2050, 2, 5629}, {16, 2036, 1, 5629}, {17, 2037, 2, 5629}, {17, 2051, 1, 5629}, {18, 2038, 1, 5629}, {18, 2052, 2, 5629}, {19, 2053, 1, 5629}, {10, 2019, 1, 5630}, {11, 2021, 2, 5630}, {11, 2034, 1, 5630}, {12, 2022, 2, 5630}, {12, 2049, 1, 5630}, {13, 2024, 1, 5630}, {13, 2036, 2, 5630}, {14, 2025, 2, 5630}, {14, 2037, 2, 5630}, {14, 2051, 2, 5630}, {15, 2026, 1, 5630}, {15, 2052, 2, 5630}, {16, 2039, 1, 5630}, {17, 2040, 2, 5630}, {17, 2054, 1, 5630}, {18, 2041, 1, 5630}, {18, 2055, 2, 5630}, {19, 2056, 1, 5630}, {10, 2020, 1, 5631}, {11, 2022, 2, 5631}, {11, 2035, 1, 5631}, {12, 2023, 2, 5631}, {12, 2050, 1, 5631}, {13, 2025, 1, 5631}, {13, 2037, 2, 5631}, {14, 2026, 2, 5631}, {14, 2038, 2, 5631}, {14, 2052, 2, 5631}, {15, 2027, 1, 5631}, {15, 2053, 2, 5631}, {16, 2040, 1, 5631}, {17, 2041, 2, 5631}, {17, 2055, 1, 5631}, {18, 2042, 1, 5631}, {18, 2056, 2, 5631}, {19, 2057, 1, 5631}, {10, 2021, 1, 5632}, {11, 2024, 2, 5632}, {11, 2036, 1, 5632}, {12, 2025, 2, 5632}, {12, 2051, 1, 5632}, {13, 2028, 1, 5632}, {13, 2039, 2, 5632}, {14, 2029, 2, 5632}, {14, 2040, 2, 5632}, {14, 2054, 2, 5632}, {15, 2030, 1, 5632}, {15, 2055, 2, 5632}, {16, 2043, 1, 5632}, {17, 2044, 2, 5632}, {17, 2058, 1, 5632}, {18, 2045, 1, 5632}, {18, 2059, 2, 5632}, {19, 2060, 1, 5632}, {10, 2022, 1, 5633}, {11, 2025, 2, 5633}, {11, 2037, 1, 5633}, {12, 2026, 2, 5633}, {12, 2052, 1, 5633}, {13, 2029, 1, 5633}, {13, 2040, 2, 5633}, {14, 2030, 2, 5633}, {14, 2041, 2, 5633}, {14, 2055, 2, 5633}, {15, 2031, 1, 5633}, {15, 2056, 2, 5633}, {16, 2044, 1, 5633}, {17, 2045, 2, 5633}, {17, 2059, 1, 5633}, {18, 2046, 1, 5633}, {18, 2060, 2, 5633}, {19, 2061, 1, 5633}, {10, 2023, 1, 5634}, {11, 2026, 2, 5634}, {11, 2038, 1, 5634}, {12, 2027, 2, 5634}, {12, 2053, 1, 5634}, {13, 2030, 1, 5634}, {13, 2041, 2, 5634}, {14, 2031, 2, 5634}, {14, 2042, 2, 5634}, {14, 2056, 2, 5634}, {15, 2032, 1, 5634}, {15, 2057, 2, 5634}, {16, 2045, 1, 5634}, {17, 2046, 2, 5634}, {17, 2060, 1, 5634}, {18, 2047, 1, 5634}, {18, 2061, 2, 5634}, {19, 2062, 1, 5634}, {10, 1154, 1, 5635}, {11, 1155, 1, 5635}, {11, 1169, 2, 5635}, {12, 1156, 1, 5635}, {12, 1184, 2, 5635}, {13, 1170, 2, 5635}, {13, 1199, 1, 5635}, {14, 1171, 2, 5635}, {14, 1185, 2, 5635}, {14, 1214, 2, 5635}, {15, 1186, 2, 5635}, {15, 1229, 1, 5635}, {16, 1200, 1, 5635}, {17, 1201, 1, 5635}, {17, 1215, 2, 5635}, {18, 1216, 2, 5635}, {18, 1230, 1, 5635}, {19, 1231, 1, 5635}, {10, 1155, 1, 5636}, {11, 1157, 1, 5636}, {11, 1170, 2, 5636}, {12, 1158, 1, 5636}, {12, 1185, 2, 5636}, {13, 1172, 2, 5636}, {13, 1200, 1, 5636}, {14, 1173, 2, 5636}, {14, 1187, 2, 5636}, {14, 1215, 2, 5636}, {15, 1188, 2, 5636}, {15, 1230, 1, 5636}, {16, 1202, 1, 5636}, {17, 1203, 1, 5636}, {17, 1217, 2, 5636}, {18, 1218, 2, 5636}, {18, 1232, 1, 5636}, {19, 1233, 1, 5636}, {10, 1156, 1, 5637}, {11, 1158, 1, 5637}, {11, 1171, 2, 5637}, {12, 1159, 1, 5637}, {12, 1186, 2, 5637}, {13, 1173, 2, 5637}, {13, 1201, 1, 5637}, {14, 1174, 2, 5637}, {14, 1188, 2, 5637}, {14, 1216, 2, 5637}, {15, 1189, 2, 5637}, {15, 1231, 1, 5637}, {16, 1203, 1, 5637}, {17, 1204, 1, 5637}, {17, 1218, 2, 5637}, {18, 1219, 2, 5637}, {18, 1233, 1, 5637}, {19, 1234, 1, 5637}, {10, 1157, 1, 5638}, {11, 1160, 1, 5638}, {11, 1172, 2, 5638}, {12, 1161, 1, 5638}, {12, 1187, 2, 5638}, {13, 1175, 2, 5638}, {13, 1202, 1, 5638}, {14, 1176, 2, 5638}, {14, 1190, 2, 5638}, {14, 1217, 2, 5638}, {15, 1191, 2, 5638}, {15, 1232, 1, 5638}, {16, 1205, 1, 5638}, {17, 1206, 1, 5638}, {17, 1220, 2, 5638}, {18, 1221, 2, 5638}, {18, 1235, 1, 5638}, {19, 1236, 1, 5638}, {10, 1158, 1, 5639}, {11, 1161, 1, 5639}, {11, 1173, 2, 5639}, {12, 1162, 1, 5639}, {12, 1188, 2, 5639}, {13, 1176, 2, 5639}, {13, 1203, 1, 5639}, {14, 1177, 2, 5639}, {14, 1191, 2, 5639}, {14, 1218, 2, 5639}, {15, 1192, 2, 5639}, {15, 1233, 1, 5639}, {16, 1206, 1, 5639}, {17, 1207, 1, 5639}, {17, 1221, 2, 5639}, {18, 1222, 2, 5639}, {18, 1236, 1, 5639}, {19, 1237, 1, 5639}, {10, 1159, 1, 5640}, {11, 1162, 1, 5640}, {11, 1174, 2, 5640}, {12, 1163, 1, 5640}, {12, 1189, 2, 5640}, {13, 1177, 2, 5640}, {13, 1204, 1, 5640}, {14, 1178, 2, 5640}, {14, 1192, 2, 5640}, {14, 1219, 2, 5640}, {15, 1193, 2, 5640}, {15, 1234, 1, 5640}, {16, 1207, 1, 5640}, {17, 1208, 1, 5640}, {17, 1222, 2, 5640}, {18, 1223, 2, 5640}, {18, 1237, 1, 5640}, {19, 1238, 1, 5640}, {10, 1160, 1, 5641}, {11, 1164, 1, 5641}, {11, 1175, 2, 5641}, {12, 1165, 1, 5641}, {12, 1190, 2, 5641}, {13, 1179, 2, 5641}, {13, 1205, 1,

5641}, {14, 1180, 2, 5641}, {14, 1194, 2, 5641}, {14, 1220, 2, 5641}, {15, 1195, 2, 5641}, {15, 1235, 1, 5641}, {16, 1209, 1, 5641}, {17, 1210, 1, 5641}, {17, 1224, 2, 5641}, {18, 1225, 2, 5641}, {18, 1239, 1, 5641}, {19, 1240, 1, 5641}, {10, 1161, 1, 5642}, {11, 1165, 1, 5642}, {11, 1176, 2, 5642}, {12, 1166, 1, 5642}, {12, 1191, 2, 5642}, {13, 1180, 2, 5642}, {13, 1206, 1, 5642}, {14, 1181, 2, 5642}, {14, 1195, 2, 5642}, {14, 1221, 2, 5642}, {15, 1196, 2, 5642}, {15, 1236, 1, 5642}, {16, 1210, 1, 5642}, {17, 1211, 1, 5642}, {17, 1225, 2, 5642}, {18, 1226, 2, 5642}, {18, 1240, 1, 5642}, {19, 1241, 1, 5642}, {10, 1162, 1, 5643}, {11, 1166, 1, 5643}, {11, 1177, 2, 5643}, {12, 1167, 1, 5643}, {12, 1192, 2, 5643}, {13, 1181, 2, 5643}, {13, 1207, 1, 5643}, {14, 1182, 2, 5643}, {14, 1196, 2, 5643}, {14, 1222, 2, 5643}, {15, 1197, 2, 5643}, {15, 1237, 1, 5643}, {16, 1211, 1, 5643}, {17, 1212, 1, 5643}, {17, 1226, 2, 5643}, {18, 1227, 2, 5643}, {18, 1241, 1, 5643}, {19, 1242, 1, 5643}, {10, 1163, 1, 5644}, {11, 1167, 1, 5644}, {11, 1178, 2, 5644}, {12, 1168, 1, 5644}, {12, 1193, 2, 5644}, {13, 1182, 2, 5644}, {13, 1208, 1, 5644}, {14, 1183, 2, 5644}, {14, 1197, 2, 5644}, {14, 1223, 2, 5644}, {15, 1198, 2, 5644}, {15, 1238, 1, 5644}, {16, 1212, 1, 5644}, {17, 1213, 1, 5644}, {17, 1227, 2, 5644}, {18, 1228, 2, 5644}, {18, 1242, 1, 5644}, {19, 1243, 1, 5644}, {10, 1814, 1, 5645}, {11, 1815, 1, 5645}, {11, 1824, 2, 5645}, {12, 1816, 1, 5645}, {12, 1834, 2, 5645}, {13, 1825, 2, 5645}, {13, 1844, 1, 5645}, {14, 1826, 2, 5645}, {14, 1835, 2, 5645}, {14, 1854, 2, 5645}, {15, 1836, 2, 5645}, {15, 1864, 1, 5645}, {16, 1845, 1, 5645}, {17, 1846, 1, 5645}, {17, 1855, 2, 5645}, {18, 1856, 2, 5645}, {18, 1865, 1, 5645}, {19, 1866, 1, 5645}, {10, 1815, 1, 5646}, {11, 1817, 1, 5646}, {11, 1825, 2, 5646}, {12, 1818, 1, 5646}, {12, 1835, 2, 5646}, {13, 1827, 2, 5646}, {13, 1845, 1, 5646}, {14, 1828, 2, 5646}, {14, 1837, 2, 5646}, {14, 1855, 2, 5646}, {15, 1838, 2, 5646}, {15, 1865, 1, 5646}, {16, 1847, 1, 5646}, {17, 1848, 1, 5646}, {17, 1857, 2, 5646}, {18, 1858, 2, 5646}, {18, 1867, 1, 5646}, {19, 1868, 1, 5646}, {10, 1816, 1, 5647}, {11, 1818, 1, 5647}, {11, 1826, 2, 5647}, {12, 1819, 1, 5647}, {12, 1836, 2, 5647}, {13, 1828, 2, 5647}, {13, 1846, 1, 5647}, {14, 1829, 2, 5647}, {14, 1838, 2, 5647}, {14, 1856, 2, 5647}, {15, 1839, 2, 5647}, {15, 1866, 1, 5647}, {16, 1848, 1, 5647}, {17, 1849, 1, 5647}, {17, 1858, 2, 5647}, {18, 1859, 2, 5647}, {18, 1868, 1, 5647}, {19, 1869, 1, 5647}, {10, 1817, 1, 5648}, {11, 1820, 1, 5648}, {11, 1827, 2, 5648}, {12, 1821, 1, 5648}, {12, 1837, 2, 5648}, {13, 1830, 2, 5648}, {13, 1847, 1, 5648}, {14, 1831, 2, 5648}, {14, 1840, 2, 5648}, {14, 1857, 2, 5648}, {15, 1841, 2, 5648}, {15, 1867, 1, 5648}, {16, 1850, 1, 5648}, {17, 1851, 1, 5648}, {17, 1860, 2, 5648}, {18, 1861, 2, 5648}, {18, 1870, 1, 5648}, {19, 1871, 1, 5648}, {10, 1818, 1, 5649}, {11, 1821, 1, 5649}, {11, 1828, 2, 5649}, {12, 1822, 1, 5649}, {12, 1838, 2, 5649}, {13, 1831, 2, 5649}, {13, 1848, 1, 5649}, {14, 1832, 2, 5649}, {14, 1841, 2, 5649}, {14, 1858, 2, 5649}, {15, 1842, 2, 5649}, {15, 1868, 1, 5649}, {16, 1851, 1, 5649}, {17, 1852, 1, 5649}, {17, 1861, 2, 5649}, {18, 1862, 2, 5649}, {18, 1871, 1, 5649}, {19, 1872, 1, 5649}, {10, 1819, 1, 5650}, {11, 1822, 1, 5650}, {11, 1829, 2, 5650}, {12, 1823, 1, 5650}, {12, 1839, 2, 5650}, {13, 1832, 2, 5650}, {13, 1849, 1, 5650}, {14, 1833, 2, 5650}, {14, 1842, 2, 5650}, {14, 1859, 2, 5650}, {15, 1843, 2, 5650}, {15, 1869, 1, 5650}, {16, 1852, 1, 5650}, {17, 1853, 1, 5650}, {17, 1862, 2, 5650}, {18, 1863, 2, 5650}, {18, 1872, 1, 5650}, {19, 1873, 1, 5650}, {10, 2119, 1, 5651}, {11, 2120, 3, 5651}, {12, 2121, 3, 5651}, {13, 2122, 1, 5651}, {13, 2129, 2, 5651}, {14, 2123, 2, 5651}, {14, 2130, 4, 5651}, {15, 2124, 1, 5651}, {15, 2138, 2, 5651}, {16, 2131, 1, 5651}, {17, 2132, 2, 5651}, {17, 2139, 1, 5651}, {18, 2133, 1, 5651}, {18, 2140, 2, 5651}, {19, 2141, 1, 5651}, {10, 2120, 1, 5652}, {11, 2122, 2, 5652}, {11, 2129, 1, 5652}, {12, 2123, 2, 5652}, {12, 2130, 1, 5652}, {13, 2125, 1, 5652}, {13, 2131, 2, 5652}, {14, 2126, 2, 5652}, {14, 2132, 2, 5652}, {14, 2139, 2, 5652}, {15, 2127, 1, 5652}, {15, 2140, 2, 5652}, {16, 2134, 1, 5652}, {17, 2135, 2, 5652}, {17, 2142, 1, 5652}, {18, 2136, 1, 5652}, {18, 2143, 2, 5652}, {19, 2144, 1, 5652}, {10, 2121, 1, 5653}, {11, 2123, 2, 5653}, {11, 2130, 1, 5653}, {12, 2124, 2, 5653}, {12, 2138, 1, 5653}, {13, 2126, 1, 5653}, {13, 2132, 2, 5653}, {14, 2127, 2, 5653}, {14, 2133, 2, 5653}, {14, 2140, 2, 5653}, {15, 2128, 1, 5653}, {15, 2141, 2, 5653}, {16, 2135, 1, 5653}, {17, 2136, 2, 5653}, {17, 2143, 1, 5653}, {18, 2137, 1, 5653}, {18, 2144, 2, 5653}, {19, 2145, 1, 5653}, {10, 2120, 1, 5654}, {11, 2122, 1, 5654}, {11, 2129, 2, 5654}, {12, 2123, 1, 5654}, {12, 2130, 2, 5654}, {13, 2131, 3, 5654}, {14, 2132, 4, 5654}, {14, 2139, 2, 5654}, {15, 2133, 1, 5654}, {15, 2140, 2, 5654}, {16, 2146, 1, 5654}, {17, 2147, 3, 5654}, {18, 2148, 1, 5654}, {18, 2153, 2, 5654}, {19, 2154, 1, 5654}, {10, 2129, 1, 5655}, {11, 2131, 3, 5655}, {12, 2132, 3, 5655}, {13, 2134, 1, 5655}, {13, 2146, 2, 5655}, {14, 2135, 2, 5655}, {14, 2147, 4, 5655}, {15, 2136, 1, 5655}, {15, 2153, 2, 5655}, {16, 2149, 1, 5655}, {17, 2150, 2, 5655}, {17, 2155, 1, 5655}, {18, 2151, 1, 5655}, {18, 2156, 2, 5655}, {19, 2157, 1, 5655}, {10, 2130, 1, 5656}, {11, 2132, 2, 5656}, {11, 2139, 1, 5656}, {12, 2133, 2, 5656}, {12, 2140, 1, 5656}, {13, 2135, 1, 5656}, {13, 2147, 2, 5656}, {14, 2136, 2, 5656}, {14, 2148, 2, 5656}, {14, 2153, 2, 5656}, {15, 2137, 1, 5656}, {15, 2154, 2, 5656}, {16, 2150, 1, 5656}, {17, 2151, 2, 5656}, {17, 2156, 1, 5656}, {18, 2152, 1, 5656}, {18, 2157, 2, 5656}, {19, 2158, 1, 5656}, {10, 2121, 1, 5657}, {11, 2123, 1, 5657}, {11, 2130, 2, 5657}, {12, 2124, 1, 5657}, {12, 2138, 2, 5657}, {13, 2132, 2, 5657}, {13, 2139, 1, 5657}, {14, 2133, 2, 5657}, {14, 2140, 4, 5657}, {15, 2141, 3, 5657}, {16, 2147, 1, 5657}, {17, 2148, 1, 5657}, {17, 2153, 2, 5657}, {18, 2154, 3, 5657}, {19, 2159, 1, 5657}, {10, 2130, 1, 5658}, {11, 2132, 1, 5658}, {11, 2139, 2, 5658}, {12, 2133, 1, 5658}, {12, 2140, 2, 5658}, {13, 2142, 1, 5658},

{13, 2147, 2, 5658}, {14, 2143, 2, 5658}, {14, 2148, 2, 5658}, {14, 2153, 2, 5658}, {15, 2144, 1, 5658}, {15, 2154, 2, 5658}, {16, 2155, 1, 5658}, {17, 2156, 2, 5658}, {17, 2160, 1, 5658}, {18, 2157, 1, 5658}, {18, 2161, 2, 5658}, {19, 2162, 1, 5658}, {10, 2138, 1, 5659}, {11, 2140, 3, 5659}, {12, 2141, 3, 5659}, {13, 2143, 1, 5659}, {13, 2153, 2, 5659}, {14, 2144, 2, 5659}, {14, 2154, 4, 5659}, {15, 2145, 1, 5659}, {15, 2159, 2, 5659}, {16, 2156, 1, 5659}, {17, 2157, 2, 5659}, {17, 2161, 1, 5659}, {18, 2158, 1, 5659}, {18, 2162, 2, 5659}, {19, 2163, 1, 5659}, {10, 2122, 1, 5660}, {11, 2125, 1, 5660}, {11, 2131, 2, 5660}, {12, 2126, 1, 5660}, {12, 2139, 2, 5660}, {13, 2134, 2, 5660}, {13, 2146, 1, 5660}, {14, 2135, 2, 5660}, {14, 2142, 2, 5660}, {14, 2147, 2, 5660}, {15, 2143, 2, 5660}, {15, 2148, 1, 5660}, {16, 2149, 1, 5660}, {17, 2150, 1, 5660}, {17, 2155, 2, 5660}, {18, 2156, 2, 5660}, {18, 2160, 1, 5660}, {19, 2161, 1, 5660}, {10, 2131, 1, 5661}, {11, 2134, 1, 5661}, {11, 2146, 2, 5661}, {12, 2135, 1, 5661}, {12, 2147, 2, 5661}, {13, 2149, 3, 5661}, {14, 2150, 4, 5661}, {14, 2155, 2, 5661}, {15, 2151, 1, 5661}, {15, 2156, 2, 5661}, {16, 2164, 1, 5661}, {17, 2165, 3, 5661}, {18, 2166, 1, 5661}, {18, 2168, 2, 5661}, {19, 2169, 1, 5661}, {10, 2139, 1, 5662}, {11, 2142, 1, 5662}, {11, 2147, 2, 5662}, {12, 2143, 1, 5662}, {12, 2148, 2, 5662}, {13, 2150, 1, 5662}, {13, 2155, 2, 5662}, {14, 2151, 2, 5662}, {14, 2156, 2, 5662}, {14, 2160, 2, 5662}, {15, 2152, 1, 5662}, {15, 2161, 2, 5662}, {16, 2165, 1, 5662}, {17, 2166, 2, 5662}, {17, 2168, 1, 5662}, {18, 2167, 1, 5662}, {18, 2169, 2, 5662}, {19, 2170, 1, 5662}, {10, 2123, 1, 5663}, {11, 2126, 1, 5663}, {11, 2132, 2, 5663}, {12, 2127, 1, 5663}, {12, 2140, 2, 5663}, {13, 2135, 2, 5663}, {13, 2147, 1, 5663}, {14, 2136, 2, 5663}, {14, 2143, 2, 5663}, {14, 2153, 2, 5663}, {15, 2144, 2, 5663}, {15, 2154, 1, 5663}, {16, 2150, 1, 5663}, {17, 2151, 1, 5663}, {17, 2156, 2, 5663}, {18, 2157, 2, 5663}, {18, 2161, 1, 5663}, {19, 2162, 1, 5663}, {10, 2132, 1, 5664}, {11, 2135, 1, 5664}, {11, 2147, 2, 5664}, {12, 2136, 1, 5664}, {12, 2153, 2, 5664}, {13, 2150, 2, 5664}, {13, 2155, 1, 5664}, {14, 2151, 2, 5664}, {14, 2156, 4, 5664}, {15, 2157, 3, 5664}, {16, 2165, 1, 5664}, {17, 2166, 1, 5664}, {17, 2168, 2, 5664}, {18, 2169, 3, 5664}, {19, 2171, 1, 5664}, {10, 2140, 1, 5665}, {11, 2143, 1, 5665}, {11, 2153, 2, 5665}, {12, 2144, 1, 5665}, {12, 2154, 2, 5665}, {13, 2156, 3, 5665}, {14, 2157, 4, 5665}, {14, 2161, 2, 5665}, {15, 2158, 1, 5665}, {15, 2162, 2, 5665}, {16, 2168, 1, 5665}, {17, 2169, 3, 5665}, {18, 2170, 1, 5665}, {18, 2171, 2, 5665}, {19, 2172, 1, 5665}, {10, 2124, 1, 5666}, {11, 2127, 1, 5666}, {11, 2133, 2, 5666}, {12, 2128, 1, 5666}, {12, 2141, 2, 5666}, {13, 2136, 2, 5666}, {13, 2148, 1, 5666}, {14, 2137, 2, 5666}, {14, 2144, 2, 5666}, {14, 2154, 2, 5666}, {15, 2145, 2, 5666}, {15, 2159, 1, 5666}, {16, 2151, 1, 5666}, {17, 2152, 1, 5666}, {17, 2157, 2, 5666}, {18, 2158, 2, 5666}, {18, 2162, 1, 5666}, {19, 2163, 1, 5666}, {10, 2133, 1, 5667}, {11, 2136, 1, 5667}, {11, 2148, 2, 5667}, {12, 2137, 1, 5667}, {12, 2154, 2, 5667}, {13, 2151, 2, 5667}, {13, 2160, 1, 5667}, {14, 2152, 2, 5667}, {14, 2157, 2, 5667}, {14, 2161, 2, 5667}, {15, 2158, 2, 5667}, {15, 2162, 1, 5667}, {16, 2166, 1, 5667}, {17, 2167, 1, 5667}, {17, 2169, 2, 5667}, {18, 2170, 2, 5667}, {18, 2171, 1, 5667}, {19, 2172, 1, 5667}, {10, 2141, 1, 5668}, {11, 2144, 1, 5668}, {11, 2154, 2, 5668}, {12, 2145, 1, 5668}, {12, 2159, 2, 5668}, {13, 2157, 2, 5668}, {13, 2161, 1, 5668}, {14, 2158, 2, 5668}, {14, 2162, 4, 5668}, {15, 2163, 3, 5668}, {16, 2169, 1, 5668}, {17, 2170, 1, 5668}, {17, 2171, 2, 5668}, {18, 2172, 3, 5668}, {19, 2173, 1, 5668}, {10, 2174, 1, 5669}, {11, 2175, 3, 5669}, {12, 2176, 3, 5669}, {13, 2177, 1, 5669}, {13, 2180, 2, 5669}, {14, 2178, 2, 5669}, {14, 2181, 4, 5669}, {15, 2179, 1, 5669}, {15, 2185, 2, 5669}, {16, 2182, 1, 5669}, {17, 2183, 2, 5669}, {17, 2186, 1, 5669}, {18, 2184, 1, 5669}, {18, 2187, 2, 5669}, {19, 2188, 1, 5669}, {10, 2175, 1, 5670}, {11, 2177, 1, 5670}, {11, 2180, 2, 5670}, {12, 2178, 1, 5670}, {12, 2181, 2, 5670}, {13, 2182, 3, 5670}, {14, 2183, 4, 5670}, {14, 2186, 2, 5670}, {15, 2184, 1, 5670}, {15, 2187, 2, 5670}, {16, 2189, 1, 5670}, {17, 2190, 3, 5670}, {18, 2191, 1, 5670}, {18, 2192, 2, 5670}, {19, 2193, 1, 5670}, {10, 2176, 1, 5671}, {11, 2178, 1, 5671}, {11, 2181, 2, 5671}, {12, 2179, 1, 5671}, {12, 2185, 2, 5671}, {13, 2183, 2, 5671}, {13, 2186, 1, 5671}, {14, 2184, 2, 5671}, {14, 2187, 4, 5671}, {15, 2188, 3, 5671}, {16, 2190, 1, 5671}, {17, 2191, 1, 5671}, {17, 2192, 2, 5671}, {18, 2193, 3, 5671}, {19, 2194, 1, 5671}, {10, 2195, 1, 5672}, {11, 2196, 2, 5672}, {11, 2205, 1, 5672}, {12, 2197, 2, 5672}, {12, 2215, 1, 5672}, {13, 2198, 1, 5672}, {13, 2206, 2, 5672}, {14, 2199, 2, 5672}, {14, 2207, 2, 5672}, {14, 2216, 2, 5672}, {15, 2200, 1, 5672}, {15, 2217, 2, 5672}, {16, 2208, 1, 5672}, {17, 2209, 2, 5672}, {17, 2218, 1, 5672}, {18, 2210, 1, 5672}, {18, 2219, 2, 5672}, {19, 2220, 1, 5672}, {10, 2196, 1, 5673}, {11, 2198, 2, 5673}, {11, 2206, 1, 5673}, {12, 2199, 2, 5673}, {12, 2216, 1, 5673}, {13, 2201, 1, 5673}, {13, 2208, 2, 5673}, {14, 2202, 2, 5673}, {14, 2209, 2, 5673}, {14, 2218, 2, 5673}, {15, 2203, 1, 5673}, {15, 2219, 2, 5673}, {16, 2211, 1, 5673}, {17, 2212, 2, 5673}, {17, 2221, 1, 5673}, {18, 2213, 1, 5673}, {18, 2222, 2, 5673}, {19, 2223, 1, 5673}, {10, 2197, 1, 5674}, {11, 2199, 2, 5674}, {11, 2207, 1, 5674}, {12, 2200, 2, 5674}, {12, 2217, 1, 5674}, {13, 2202, 1, 5674}, {13, 2209, 2, 5674}, {14, 2203, 2, 5674}, {14, 2210, 2, 5674}, {14, 2219, 2, 5674}, {15, 2204, 1, 5674}, {15, 2220, 2, 5674}, {16, 2212, 1, 5674}, {17, 2213, 2, 5674}, {17, 2222, 1, 5674}, {18, 2214, 1, 5674}, {18, 2223, 2, 5674}, {19, 2224, 1, 5674}, {10, 2205, 1, 5675}, {11, 2206, 2, 5675}, {11, 2225, 1, 5675}, {12, 2207, 2, 5675}, {12, 2235, 1, 5675}, {13, 2208, 1, 5675}, {13, 2226, 2, 5675}, {14, 2209, 2, 5675}, {14, 2227, 2, 5675}, {14, 2236, 2, 5675}, {15, 2210, 1, 5675}, {15, 2237, 2, 5675}, {16, 2228, 1, 5675}, {17, 2229, 2, 5675}, {17, 2238, 1, 5675}, {18, 2230, 1, 5675}, {18, 2239, 2, 5675}, {19,

2240, 1, 5675}, {10, 2206, 1, 5676}, {11, 2208, 2, 5676}, {11, 2226, 1, 5676}, {12, 2209,
2, 5676}, {12, 2236, 1, 5676}, {13, 2211, 1, 5676}, {13, 2228, 2, 5676}, {14, 2212, 2,
5676}, {14, 2229, 2, 5676}, {14, 2238, 2, 5676}, {15, 2213, 1, 5676}, {15, 2239, 2, 5676},
{16, 2231, 1, 5676}, {17, 2232, 2, 5676}, {17, 2241, 1, 5676}, {18, 2233, 1, 5676}, {18,
2242, 2, 5676}, {19, 2243, 1, 5676}, {10, 2207, 1, 5677}, {11, 2209, 2, 5677}, {11, 2227,
1, 5677}, {12, 2210, 2, 5677}, {12, 2237, 1, 5677}, {13, 2212, 1, 5677}, {13, 2229, 2,
5677}, {14, 2213, 2, 5677}, {14, 2230, 2, 5677}, {14, 2239, 2, 5677}, {15, 2214, 1, 5677},
{15, 2240, 2, 5677}, {16, 2232, 1, 5677}, {17, 2233, 2, 5677}, {17, 2242, 1, 5677}, {18,
2234, 1, 5677}, {18, 2243, 2, 5677}, {19, 2244, 1, 5677}, {10, 2215, 1, 5678}, {11, 2216,
2, 5678}, {11, 2235, 1, 5678}, {12, 2217, 2, 5678}, {12, 2245, 1, 5678}, {13, 2218, 1,
5678}, {13, 2236, 2, 5678}, {14, 2219, 2, 5678}, {14, 2237, 2, 5678}, {14, 2246, 2, 5678},
{15, 2220, 1, 5678}, {15, 2247, 2, 5678}, {16, 2238, 1, 5678}, {17, 2239, 2, 5678}, {17,
2248, 1, 5678}, {18, 2240, 1, 5678}, {18, 2249, 2, 5678}, {19, 2250, 1, 5678}, {10, 2216,
1, 5679}, {11, 2218, 2, 5679}, {11, 2236, 1, 5679}, {12, 2219, 2, 5679}, {12, 2246, 1,
5679}, {13, 2221, 1, 5679}, {13, 2238, 2, 5679}, {14, 2222, 2, 5679}, {14, 2239, 2, 5679},
{14, 2248, 2, 5679}, {15, 2223, 1, 5679}, {15, 2249, 2, 5679}, {16, 2241, 1, 5679}, {17,
2242, 2, 5679}, {17, 2251, 1, 5679}, {18, 2243, 1, 5679}, {18, 2252, 2, 5679}, {19, 2253,
1, 5679}, {10, 2217, 1, 5680}, {11, 2219, 2, 5680}, {11, 2237, 1, 5680}, {12, 2220, 2,
5680}, {12, 2247, 1, 5680}, {13, 2222, 1, 5680}, {13, 2239, 2, 5680}, {14, 2223, 2, 5680},
{14, 2240, 2, 5680}, {14, 2249, 2, 5680}, {15, 2224, 1, 5680}, {15, 2250, 2, 5680}, {16,
2242, 1, 5680}, {17, 2243, 2, 5680}, {17, 2252, 1, 5680}, {18, 2244, 1, 5680}, {18, 2253,
2, 5680}, {19, 2254, 1, 5680}, {10, 2351, 1, 5681}, {11, 2352, 2, 5681}, {11, 2357, 1,
5681}, {12, 2353, 2, 5681}, {12, 2363, 1, 5681}, {13, 2354, 1, 5681}, {13, 2358, 2, 5681},
{14, 2355, 2, 5681}, {14, 2359, 2, 5681}, {14, 2364, 2, 5681}, {15, 2356, 1, 5681}, {15,
2365, 2, 5681}, {16, 2360, 1, 5681}, {17, 2361, 2, 5681}, {17, 2366, 1, 5681}, {18, 2362,
1, 5681}, {18, 2367, 2, 5681}, {19, 2368, 1, 5681}, {10, 2369, 1, 5682}, {11, 2370, 2,
5682}, {11, 2379, 1, 5682}, {12, 2371, 2, 5682}, {12, 2389, 1, 5682}, {13, 2372, 1, 5682},
{13, 2380, 2, 5682}, {14, 2373, 2, 5682}, {14, 2381, 2, 5682}, {14, 2390, 2, 5682}, {15,
2374, 1, 5682}, {15, 2391, 2, 5682}, {16, 2382, 1, 5682}, {17, 2383, 2, 5682}, {17, 2392,
1, 5682}, {18, 2384, 1, 5682}, {18, 2393, 2, 5682}, {19, 2394, 1, 5682}, {10, 2370, 1,
5683}, {11, 2372, 2, 5683}, {11, 2380, 1, 5683}, {12, 2373, 2, 5683}, {12, 2390, 1, 5683},
{13, 2375, 1, 5683}, {13, 2382, 2, 5683}, {14, 2376, 2, 5683}, {14, 2383, 2, 5683}, {14,
2392, 2, 5683}, {15, 2377, 1, 5683}, {15, 2393, 2, 5683}, {16, 2385, 1, 5683}, {17, 2386,
2, 5683}, {17, 2395, 1, 5683}, {18, 2387, 1, 5683}, {18, 2396, 2, 5683}, {19, 2397, 1,
5683}, {10, 2371, 1, 5684}, {11, 2373, 2, 5684}, {11, 2381, 1, 5684}, {12, 2374, 2, 5684},
{12, 2391, 1, 5684}, {13, 2376, 1, 5684}, {13, 2383, 2, 5684}, {14, 2377, 2, 5684}, {14,
2384, 2, 5684}, {14, 2393, 2, 5684}, {15, 2378, 1, 5684}, {15, 2394, 2, 5684}, {16, 2386,
1, 5684}, {17, 2387, 2, 5684}, {17, 2396, 1, 5684}, {18, 2388, 1, 5684}, {18, 2397, 2,
5684}, {19, 2398, 1, 5684}, {10, 1874, 1, 5685}, {11, 1875, 1, 5685}, {11, 1889, 2, 5685},
{12, 1876, 1, 5685}, {12, 1904, 2, 5685}, {13, 1890, 2, 5685}, {13, 1919, 1, 5685}, {14,
1891, 2, 5685}, {14, 1905, 2, 5685}, {14, 1934, 2, 5685}, {15, 1906, 2, 5685}, {15, 1949,
1, 5685}, {16, 1920, 1, 5685}, {17, 1921, 1, 5685}, {17, 1935, 2, 5685}, {18, 1936, 2,
5685}, {18, 1950, 1, 5685}, {19, 1951, 1, 5685}, {10, 1875, 1, 5686}, {11, 1877, 1, 5686},
{11, 1890, 2, 5686}, {12, 1878, 1, 5686}, {12, 1905, 2, 5686}, {13, 1892, 2, 5686}, {13,
1920, 1, 5686}, {14, 1893, 2, 5686}, {14, 1907, 2, 5686}, {14, 1935, 2, 5686}, {15, 1908,
2, 5686}, {15, 1950, 1, 5686}, {16, 1922, 1, 5686}, {17, 1923, 1, 5686}, {17, 1937, 2,
5686}, {18, 1938, 2, 5686}, {18, 1952, 1, 5686}, {19, 1953, 1, 5686}, {10, 1876, 1, 5687},
{11, 1878, 1, 5687}, {11, 1891, 2, 5687}, {12, 1879, 1, 5687}, {12, 1906, 2, 5687}, {13,
1893, 2, 5687}, {13, 1921, 1, 5687}, {14, 1894, 2, 5687}, {14, 1908, 2, 5687}, {14, 1936,
2, 5687}, {15, 1909, 2, 5687}, {15, 1951, 1, 5687}, {16, 1923, 1, 5687}, {17, 1924, 1,
5687}, {17, 1938, 2, 5687}, {18, 1939, 2, 5687}, {18, 1953, 1, 5687}, {19, 1954, 1, 5687},
{10, 1877, 1, 5688}, {11, 1880, 1, 5688}, {11, 1892, 2, 5688}, {12, 1881, 1, 5688}, {12,
1907, 2, 5688}, {13, 1895, 2, 5688}, {13, 1922, 1, 5688}, {14, 1896, 2, 5688}, {14, 1910,
2, 5688}, {14, 1937, 2, 5688}, {15, 1911, 2, 5688}, {15, 1952, 1, 5688}, {16, 1925, 1,
5688}, {17, 1926, 1, 5688}, {17, 1940, 2, 5688}, {18, 1941, 2, 5688}, {18, 1955, 1, 5688},
{19, 1956, 1, 5688}, {10, 1878, 1, 5689}, {11, 1881, 1, 5689}, {11, 1893, 2, 5689}, {12,
1882, 1, 5689}, {12, 1908, 2, 5689}, {13, 1896, 2, 5689}, {13, 1923, 1, 5689}, {14, 1897,
2, 5689}, {14, 1911, 2, 5689}, {14, 1938, 2, 5689}, {15, 1912, 2, 5689}, {15, 1953, 1,
5689}, {16, 1926, 1, 5689}, {17, 1927, 1, 5689}, {17, 1941, 2, 5689}, {18, 1942, 2, 5689},
{18, 1956, 1, 5689}, {19, 1957, 1, 5689}, {10, 1879, 1, 5690}, {11, 1882, 1, 5690}, {11,
1894, 2, 5690}, {12, 1883, 1, 5690}, {12, 1909, 2, 5690}, {13, 1897, 2, 5690}, {13, 1924,
1, 5690}, {14, 1898, 2, 5690}, {14, 1912, 2, 5690}, {14, 1939, 2, 5690}, {15, 1913, 2,
5690}, {15, 1954, 1, 5690}, {16, 1927, 1, 5690}, {17, 1928, 1, 5690}, {17, 1942, 2, 5690},
{18, 1943, 2, 5690}, {18, 1957, 1, 5690}, {19, 1958, 1, 5690}, {10, 1880, 1, 5691}, {11,
1884, 1, 5691}, {11, 1895, 2, 5691}, {12, 1885, 1, 5691}, {12, 1910, 2, 5691}, {13, 1899,
2, 5691}, {13, 1925, 1, 5691}, {14, 1900, 2, 5691}, {14, 1914, 2, 5691}, {14, 1940, 2,
5691}, {15, 1915, 2, 5691}, {15, 1955, 1, 5691}, {16, 1929, 1, 5691}, {17, 1930, 1, 5691},
{17, 1944, 2, 5691}, {18, 1945, 2, 5691}, {18, 1959, 1, 5691}, {19, 1960, 1, 5691}, {10,
1881, 1, 5692}, {11, 1885, 1, 5692}, {11, 1896, 2, 5692}, {12, 1886, 1, 5692}, {12, 1911,

2, 5692}, {13, 1900, 2, 5692}, {13, 1926, 1, 5692}, {14, 1901, 2, 5692}, {14, 1915, 2,
5692}, {14, 1941, 2, 5692}, {15, 1916, 2, 5692}, {15, 1956, 1, 5692}, {16, 1930, 1, 5692},
{17, 1931, 1, 5692}, {17, 1945, 2, 5692}, {18, 1946, 2, 5692}, {18, 1960, 1, 5692}, {19,
1961, 1, 5692}, {10, 1882, 1, 5693}, {11, 1886, 1, 5693}, {11, 1897, 2, 5693}, {12, 1887,
1, 5693}, {12, 1912, 2, 5693}, {13, 1901, 2, 5693}, {13, 1927, 1, 5693}, {14, 1902, 2,
5693}, {14, 1916, 2, 5693}, {14, 1942, 2, 5693}, {15, 1917, 2, 5693}, {15, 1957, 1, 5693},
{16, 1931, 1, 5693}, {17, 1932, 1, 5693}, {17, 1946, 2, 5693}, {18, 1947, 2, 5693}, {18,
1961, 1, 5693}, {19, 1962, 1, 5693}, {10, 1883, 1, 5694}, {11, 1887, 1, 5694}, {11, 1898,
2, 5694}, {12, 1888, 1, 5694}, {12, 1913, 2, 5694}, {13, 1902, 2, 5694}, {13, 1928, 1,
5694}, {14, 1903, 2, 5694}, {14, 1917, 2, 5694}, {14, 1943, 2, 5694}, {15, 1918, 2, 5694},
{15, 1958, 1, 5694}, {16, 1932, 1, 5694}, {17, 1933, 1, 5694}, {17, 1947, 2, 5694}, {18,
1948, 2, 5694}, {18, 1962, 1, 5694}, {19, 1963, 1, 5694}, {10, 2195, 1, 5695}, {11, 2196,
1, 5695}, {11, 2205, 2, 5695}, {12, 2197, 1, 5695}, {12, 2215, 2, 5695}, {13, 2206, 2,
5695}, {13, 2225, 1, 5695}, {14, 2207, 2, 5695}, {14, 2216, 2, 5695}, {14, 2235, 2, 5695},
{15, 2217, 2, 5695}, {15, 2245, 1, 5695}, {16, 2226, 1, 5695}, {17, 2227, 1, 5695}, {17,
2236, 2, 5695}, {18, 2237, 2, 5695}, {18, 2246, 1, 5695}, {19, 2247, 1, 5695}, {10, 2196,
1, 5696}, {11, 2198, 1, 5696}, {11, 2206, 2, 5696}, {12, 2199, 1, 5696}, {12, 2216, 2,
5696}, {13, 2208, 2, 5696}, {13, 2226, 1, 5696}, {14, 2209, 2, 5696}, {14, 2218, 2, 5696},
{14, 2236, 2, 5696}, {15, 2219, 2, 5696}, {15, 2246, 1, 5696}, {16, 2228, 1, 5696}, {17,
2229, 1, 5696}, {17, 2238, 2, 5696}, {18, 2239, 2, 5696}, {18, 2248, 1, 5696}, {19, 2249,
1, 5696}, {10, 2197, 1, 5697}, {11, 2199, 1, 5697}, {11, 2207, 2, 5697}, {12, 2200, 1,
5697}, {12, 2217, 2, 5697}, {13, 2209, 2, 5697}, {13, 2227, 1, 5697}, {14, 2210, 2, 5697},
{14, 2219, 2, 5697}, {14, 2237, 2, 5697}, {15, 2220, 2, 5697}, {15, 2247, 1, 5697}, {16,
2229, 1, 5697}, {17, 2230, 1, 5697}, {17, 2239, 2, 5697}, {18, 2240, 2, 5697}, {18, 2249,
1, 5697}, {19, 2250, 1, 5697}, {10, 2198, 1, 5698}, {11, 2201, 1, 5698}, {11, 2208, 2,
5698}, {12, 2202, 1, 5698}, {12, 2218, 2, 5698}, {13, 2211, 2, 5698}, {13, 2228, 1, 5698},
{14, 2212, 2, 5698}, {14, 2221, 2, 5698}, {14, 2238, 2, 5698}, {15, 2222, 2, 5698}, {15,
2248, 1, 5698}, {16, 2231, 1, 5698}, {17, 2232, 1, 5698}, {17, 2241, 2, 5698}, {18, 2242,
2, 5698}, {18, 2251, 1, 5698}, {19, 2252, 1, 5698}, {10, 2199, 1, 5699}, {11, 2202, 1,
5699}, {11, 2209, 2, 5699}, {12, 2203, 1, 5699}, {12, 2219, 2, 5699}, {13, 2212, 2, 5699},
{13, 2229, 1, 5699}, {14, 2213, 2, 5699}, {14, 2222, 2, 5699}, {14, 2239, 2, 5699}, {15,
2223, 2, 5699}, {15, 2249, 1, 5699}, {16, 2232, 1, 5699}, {17, 2233, 1, 5699}, {17, 2242,
2, 5699}, {18, 2243, 2, 5699}, {18, 2252, 1, 5699}, {19, 2253, 1, 5699}, {10, 2200, 1,
5700}, {11, 2203, 1, 5700}, {11, 2210, 2, 5700}, {12, 2204, 1, 5700}, {12, 2220, 2, 5700},
{13, 2213, 2, 5700}, {13, 2230, 1, 5700}, {14, 2214, 2, 5700}, {14, 2223, 2, 5700}, {14,
2240, 2, 5700}, {15, 2224, 2, 5700}, {15, 2250, 1, 5700}, {16, 2233, 1, 5700}, {17, 2234,
1, 5700}, {17, 2243, 2, 5700}, {18, 2244, 2, 5700}, {18, 2253, 1, 5700}, {19, 2254, 1,
5700}, {10, 2534, 1, 5701}, {11, 2535, 3, 5701}, {12, 2536, 3, 5701}, {13, 2537, 1, 5701},
{13, 2540, 2, 5701}, {14, 2538, 2, 5701}, {14, 2541, 4, 5701}, {15, 2539, 1, 5701}, {15,
2545, 2, 5701}, {16, 2542, 1, 5701}, {17, 2543, 2, 5701}, {17, 2546, 1, 5701}, {18, 2544,
1, 5701}, {18, 2547, 2, 5701}, {19, 2548, 1, 5701}, {10, 2535, 1, 5702}, {11, 2537, 1,
5702}, {11, 2540, 2, 5702}, {12, 2538, 1, 5702}, {12, 2541, 2, 5702}, {13, 2542, 3, 5702},
{14, 2543, 4, 5702}, {14, 2546, 2, 5702}, {15, 2544, 1, 5702}, {15, 2547, 2, 5702}, {16,
2549, 1, 5702}, {17, 2550, 3, 5702}, {18, 2551, 1, 5702}, {18, 2552, 2, 5702}, {19, 2553,
1, 5702}, {10, 2536, 1, 5703}, {11, 2538, 1, 5703}, {11, 2541, 2, 5703}, {12, 2539, 1,
5703}, {12, 2545, 2, 5703}, {13, 2543, 2, 5703}, {13, 2546, 1, 5703}, {14, 2544, 2, 5703},
{14, 2547, 4, 5703}, {15, 2548, 3, 5703}, {16, 2550, 1, 5703}, {17, 2551, 1, 5703}, {17,
2552, 2, 5703}, {18, 2553, 3, 5703}, {19, 2554, 1, 5703}, {10, 2621, 1, 5704}, {11, 2622,
2, 5704}, {11, 2627, 1, 5704}, {12, 2623, 2, 5704}, {12, 2633, 1, 5704}, {13, 2624, 1,
5704}, {13, 2628, 2, 5704}, {14, 2625, 2, 5704}, {14, 2629, 2, 5704}, {14, 2634, 2, 5704},
{15, 2626, 1, 5704}, {15, 2635, 2, 5704}, {16, 2630, 1, 5704}, {17, 2631, 2, 5704}, {17,
2636, 1, 5704}, {18, 2632, 1, 5704}, {18, 2637, 2, 5704}, {19, 2638, 1, 5704}, {10, 2716,
1, 5705}, {11, 2717, 2, 5705}, {11, 2722, 1, 5705}, {12, 2718, 2, 5705}, {12, 2728, 1,
5705}, {13, 2719, 1, 5705}, {13, 2723, 2, 5705}, {14, 2720, 2, 5705}, {14, 2724, 2, 5705},
{14, 2729, 2, 5705}, {15, 2721, 1, 5705}, {15, 2730, 2, 5705}, {16, 2725, 1, 5705}, {17,
2726, 2, 5705}, {17, 2731, 1, 5705}, {18, 2727, 1, 5705}, {18, 2732, 2, 5705}, {19, 2733,
1, 5705}, {20, 1073, 1, 5706}, {21, 1074, 2, 5706}, {22, 1075, 2, 5706}, {23, 1076, 1,
5706}, {24, 1077, 2, 5706}, {25, 1078, 1, 5706}, {20, 1074, 1, 5707}, {21, 1079, 2, 5707},
{22, 1080, 2, 5707}, {23, 1081, 1, 5707}, {24, 1082, 2, 5707}, {25, 1083, 1, 5707}, {20,
1075, 1, 5708}, {21, 1080, 2, 5708}, {22, 1084, 2, 5708}, {23, 1085, 1, 5708}, {24, 1086,
2, 5708}, {25, 1087, 1, 5708}, {20, 1076, 1, 5709}, {21, 1081, 2, 5709}, {22, 1085, 2,
5709}, {23, 1088, 1, 5709}, {24, 1089, 2, 5709}, {25, 1090, 1, 5709}, {20, 1077, 1, 5710},
{21, 1082, 2, 5710}, {22, 1086, 2, 5710}, {23, 1089, 1, 5710}, {24, 1091, 2, 5710}, {25,
1092, 1, 5710}, {20, 1078, 1, 5711}, {21, 1083, 2, 5711}, {22, 1087, 2, 5711}, {23, 1090,
1, 5711}, {24, 1092, 2, 5711}, {25, 1093, 1, 5711}, {21, 1073, 1, 5712}, {23, 1074, 2,
5712}, {24, 1075, 2, 5712}, {26, 1076, 1, 5712}, {27, 1077, 2, 5712}, {28, 1078, 1, 5712},
{21, 1074, 1, 5713}, {23, 1079, 2, 5713}, {24, 1080, 2, 5713}, {26, 1081, 1, 5713}, {27,
1082, 2, 5713}, {28, 1083, 1, 5713}, {21, 1075, 1, 5714}, {23, 1080, 2, 5714}, {24, 1084,
2, 5714}, {26, 1085, 1, 5714}, {27, 1086, 2, 5714}, {28, 1087, 1, 5714}, {21, 1076, 1,

5715}, {23, 1081, 2, 5715}, {24, 1085, 2, 5715}, {26, 1088, 1, 5715}, {27, 1089, 2, 5715}, {28, 1090, 1, 5715}, {21, 1077, 1, 5716}, {23, 1082, 2, 5716}, {24, 1086, 2, 5716}, {26, 1089, 1, 5716}, {27, 1091, 2, 5716}, {28, 1092, 1, 5716}, {21, 1078, 1, 5717}, {23, 1083, 2, 5717}, {24, 1087, 2, 5717}, {26, 1090, 1, 5717}, {27, 1092, 2, 5717}, {28, 1093, 1, 5717}, {22, 1073, 1, 5718}, {24, 1074, 2, 5718}, {25, 1075, 2, 5718}, {27, 1076, 1, 5718}, {28, 1077, 2, 5718}, {29, 1078, 1, 5718}, {22, 1074, 1, 5719}, {24, 1079, 2, 5719}, {25, 1080, 2, 5719}, {27, 1081, 1, 5719}, {28, 1082, 2, 5719}, {29, 1083, 1, 5719}, {22, 1075, 1, 5720}, {24, 1080, 2, 5720}, {25, 1084, 2, 5720}, {27, 1085, 1, 5720}, {28, 1086, 2, 5720}, {29, 1087, 1, 5720}, {22, 1076, 1, 5721}, {24, 1081, 2, 5721}, {25, 1085, 2, 5721}, {27, 1088, 1, 5721}, {28, 1089, 2, 5721}, {29, 1090, 1, 5721}, {22, 1077, 1, 5722}, {24, 1082, 2, 5722}, {25, 1086, 2, 5722}, {27, 1089, 1, 5722}, {28, 1091, 2, 5722}, {29, 1092, 1, 5722}, {22, 1078, 1, 5723}, {24, 1083, 2, 5723}, {25, 1087, 2, 5723}, {27, 1090, 1, 5723}, {28, 1092, 2, 5723}, {29, 1093, 1, 5723}, {23, 1073, 1, 5724}, {26, 1074, 2, 5724}, {27, 1075, 2, 5724}, {30, 1076, 1, 5724}, {31, 1077, 2, 5724}, {32, 1078, 1, 5724}, {23, 1074, 1, 5725}, {26, 1079, 2, 5725}, {27, 1080, 2, 5725}, {30, 1081, 1, 5725}, {31, 1082, 2, 5725}, {32, 1083, 1, 5725}, {23, 1075, 1, 5726}, {26, 1080, 2, 5726}, {27, 1084, 2, 5726}, {30, 1085, 1, 5726}, {31, 1086, 2, 5726}, {32, 1087, 1, 5726}, {23, 1076, 1, 5727}, {26, 1081, 2, 5727}, {27, 1085, 2, 5727}, {30, 1088, 1, 5727}, {31, 1089, 2, 5727}, {32, 1090, 1, 5727}, {23, 1077, 1, 5728}, {26, 1082, 2, 5728}, {27, 1086, 2, 5728}, {30, 1089, 1, 5728}, {31, 1091, 2, 5728}, {32, 1092, 1, 5728}, {23, 1078, 1, 5729}, {26, 1083, 2, 5729}, {27, 1087, 2, 5729}, {30, 1090, 1, 5729}, {31, 1092, 2, 5729}, {32, 1093, 1, 5729}, {24, 1073, 1, 5730}, {27, 1074, 2, 5730}, {28, 1075, 2, 5730}, {31, 1076, 1, 5730}, {32, 1077, 2, 5730}, {33, 1078, 1, 5730}, {24, 1074, 1, 5731}, {27, 1079, 2, 5731}, {28, 1080, 2, 5731}, {31, 1081, 1, 5731}, {32, 1082, 2, 5731}, {33, 1083, 1, 5731}, {24, 1075, 1, 5732}, {27, 1080, 2, 5732}, {28, 1084, 2, 5732}, {31, 1085, 1, 5732}, {32, 1086, 2, 5732}, {33, 1087, 1, 5732}, {24, 1076, 1, 5733}, {27, 1081, 2, 5733}, {28, 1085, 2, 5733}, {31, 1088, 1, 5733}, {32, 1089, 2, 5733}, {33, 1090, 1, 5733}, {24, 1077, 1, 5734}, {27, 1082, 2, 5734}, {28, 1086, 2, 5734}, {31, 1089, 1, 5734}, {32, 1091, 2, 5734}, {33, 1092, 1, 5734}, {24, 1078, 1, 5735}, {27, 1083, 2, 5735}, {28, 1087, 2, 5735}, {31, 1090, 1, 5735}, {32, 1092, 2, 5735}, {33, 1093, 1, 5735}, {25, 1073, 1, 5736}, {28, 1074, 2, 5736}, {29, 1075, 2, 5736}, {32, 1076, 1, 5736}, {33, 1077, 2, 5736}, {34, 1078, 1, 5736}, {25, 1074, 1, 5737}, {28, 1079, 2, 5737}, {29, 1080, 2, 5737}, {32, 1081, 1, 5737}, {33, 1082, 2, 5737}, {34, 1083, 1, 5737}, {25, 1075, 1, 5738}, {28, 1080, 2, 5738}, {29, 1084, 2, 5738}, {32, 1085, 1, 5738}, {33, 1086, 2, 5738}, {34, 1087, 1, 5738}, {25, 1076, 1, 5739}, {28, 1081, 2, 5739}, {29, 1085, 2, 5739}, {32, 1088, 1, 5739}, {33, 1089, 2, 5739}, {34, 1090, 1, 5739}, {25, 1077, 1, 5740}, {28, 1082, 2, 5740}, {29, 1086, 2, 5740}, {32, 1089, 1, 5740}, {33, 1091, 2, 5740}, {34, 1092, 1, 5740}, {25, 1078, 1, 5741}, {28, 1083, 2, 5741}, {29, 1087, 2, 5741}, {32, 1090, 1, 5741}, {33, 1092, 2, 5741}, {34, 1093, 1, 5741}, {20, 1376, 1, 5742}, {21, 1377, 2, 5742}, {22, 1378, 2, 5742}, {23, 1379, 1, 5742}, {24, 1380, 2, 5742}, {25, 1381, 1, 5742}, {20, 1382, 1, 5743}, {21, 1383, 2, 5743}, {22, 1384, 2, 5743}, {23, 1385, 1, 5743}, {24, 1386, 2, 5743}, {25, 1387, 1, 5743}, {20, 1388, 1, 5744}, {21, 1389, 2, 5744}, {22, 1390, 2, 5744}, {23, 1391, 1, 5744}, {24, 1392, 2, 5744}, {25, 1393, 1, 5744}, {21, 1376, 1, 5745}, {23, 1377, 2, 5745}, {24, 1378, 2, 5745}, {26, 1379, 1, 5745}, {27, 1380, 2, 5745}, {28, 1381, 1, 5745}, {21, 1382, 1, 5746}, {23, 1383, 2, 5746}, {24, 1384, 2, 5746}, {26, 1385, 1, 5746}, {27, 1386, 2, 5746}, {28, 1387, 1, 5746}, {21, 1388, 1, 5747}, {23, 1389, 2, 5747}, {24, 1390, 2, 5747}, {26, 1391, 1, 5747}, {27, 1392, 2, 5747}, {28, 1393, 1, 5747}, {22, 1376, 1, 5748}, {24, 1377, 2, 5748}, {25, 1378, 2, 5748}, {27, 1379, 1, 5748}, {28, 1380, 2, 5748}, {29, 1381, 1, 5748}, {22, 1382, 1, 5749}, {24, 1383, 2, 5749}, {25, 1384, 2, 5749}, {27, 1385, 1, 5749}, {28, 1386, 2, 5749}, {29, 1387, 1, 5749}, {22, 1388, 1, 5750}, {24, 1389, 2, 5750}, {25, 1390, 2, 5750}, {27, 1391, 1, 5750}, {28, 1392, 2, 5750}, {29, 1393, 1, 5750}, {23, 1376, 1, 5751}, {26, 1377, 2, 5751}, {27, 1378, 2, 5751}, {30, 1379, 1, 5751}, {31, 1380, 2, 5751}, {32, 1381, 1, 5751}, {23, 1382, 1, 5752}, {26, 1383, 2, 5752}, {27, 1384, 2, 5752}, {30, 1385, 1, 5752}, {31, 1386, 2, 5752}, {32, 1387, 1, 5752}, {23, 1388, 1, 5753}, {26, 1389, 2, 5753}, {27, 1390, 2, 5753}, {30, 1391, 1, 5753}, {31, 1392, 2, 5753}, {32, 1393, 1, 5753}, {24, 1376, 1, 5754}, {27, 1377, 2, 5754}, {28, 1378, 2, 5754}, {31, 1379, 1, 5754}, {32, 1380, 2, 5754}, {33, 1381, 1, 5754}, {24, 1382, 1, 5755}, {27, 1383, 2, 5755}, {28, 1384, 2, 5755}, {31, 1385, 1, 5755}, {32, 1386, 2, 5755}, {33, 1387, 1, 5755}, {24, 1388, 1, 5756}, {27, 1389, 2, 5756}, {28, 1390, 2, 5756}, {31, 1391, 1, 5756}, {32, 1392, 2, 5756}, {33, 1393, 1, 5756}, {25, 1376, 1, 5757}, {28, 1377, 2, 5757}, {29, 1378, 2, 5757}, {32, 1379, 1, 5757}, {33, 1380, 2, 5757}, {34, 1381, 1, 5757}, {25, 1382, 1, 5758}, {28, 1383, 2, 5758}, {29, 1384, 2, 5758}, {32, 1385, 1, 5758}, {33, 1386, 2, 5758}, {34, 1387, 1, 5758}, {25, 1388, 1, 5759}, {28, 1389, 2, 5759}, {29, 1390, 2, 5759}, {32, 1391, 1, 5759}, {33, 1392, 2, 5759}, {34, 1393, 1, 5759}, {20, 1536, 1, 5760}, {21, 1537, 2, 5760}, {22, 1538, 2, 5760}, {23, 1539, 1, 5760}, {24, 1540, 2, 5760}, {25, 1541, 1, 5760}, {21, 1536, 1, 5761}, {23, 1537, 2, 5761}, {24, 1538, 2, 5761}, {26, 1539, 1, 5761}, {27, 1540, 2, 5761}, {28, 1541, 1, 5761}, {22, 1536, 1, 5762}, {24, 1537, 2, 5762}, {25, 1538, 2, 5762}, {27, 1539, 1, 5762}, {28, 1540, 2, 5762}, {29, 1541, 1, 5762}, {23, 1536, 1, 5763}, {26, 1537, 2, 5763}, {27, 1538, 2, 5763}, {30, 1539, 1, 5763}, {31, 1540, 2, 5763}, {32, 1541, 1, 5763},

{24, 1536, 1, 5764}, {27, 1537, 2, 5764}, {28, 1538, 2, 5764}, {31, 1539, 1, 5764}, {32, 1540, 2, 5764}, {33, 1541, 1, 5764}, {25, 1536, 1, 5765}, {28, 1537, 2, 5765}, {29, 1538, 2, 5765}, {32, 1539, 1, 5765}, {33, 1540, 2, 5765}, {34, 1541, 1, 5765}, {20, 1542, 1, 5766}, {21, 1543, 2, 5766}, {22, 1544, 2, 5766}, {23, 1545, 1, 5766}, {24, 1546, 2, 5766}, {25, 1547, 1, 5766}, {20, 1543, 1, 5767}, {21, 1545, 2, 5767}, {22, 1546, 2, 5767}, {23, 1548, 1, 5767}, {24, 1549, 2, 5767}, {25, 1550, 1, 5767}, {20, 1544, 1, 5768}, {21, 1546, 2, 5768}, {22, 1547, 2, 5768}, {23, 1549, 1, 5768}, {24, 1550, 2, 5768}, {25, 1551, 1, 5768}, {21, 1542, 1, 5769}, {23, 1543, 2, 5769}, {24, 1544, 2, 5769}, {26, 1545, 1, 5769}, {27, 1546, 2, 5769}, {28, 1547, 1, 5769}, {21, 1543, 1, 5770}, {23, 1545, 2, 5770}, {24, 1546, 2, 5770}, {26, 1548, 1, 5770}, {27, 1549, 2, 5770}, {28, 1550, 1, 5770}, {21, 1544, 1, 5771}, {23, 1546, 2, 5771}, {24, 1547, 2, 5771}, {26, 1549, 1, 5771}, {27, 1550, 2, 5771}, {28, 1551, 1, 5771}, {22, 1542, 1, 5772}, {24, 1543, 2, 5772}, {25, 1544, 2, 5772}, {27, 1545, 1, 5772}, {28, 1546, 2, 5772}, {29, 1547, 1, 5772}, {22, 1543, 1, 5773}, {24, 1545, 2, 5773}, {25, 1546, 2, 5773}, {27, 1548, 1, 5773}, {28, 1549, 2, 5773}, {29, 1550, 1, 5773}, {22, 1544, 1, 5774}, {24, 1546, 2, 5774}, {25, 1547, 2, 5774}, {27, 1549, 1, 5774}, {28, 1550, 2, 5774}, {29, 1551, 1, 5774}, {23, 1542, 1, 5775}, {26, 1543, 2, 5775}, {27, 1544, 2, 5775}, {30, 1545, 1, 5775}, {31, 1546, 2, 5775}, {32, 1547, 1, 5775}, {23, 1543, 1, 5776}, {26, 1545, 2, 5776}, {27, 1546, 2, 5776}, {30, 1548, 1, 5776}, {31, 1549, 2, 5776}, {32, 1550, 1, 5776}, {23, 1544, 1, 5777}, {26, 1546, 2, 5777}, {27, 1547, 2, 5777}, {30, 1549, 1, 5777}, {31, 1550, 2, 5777}, {32, 1551, 1, 5777}, {24, 1542, 1, 5778}, {27, 1543, 2, 5778}, {28, 1544, 2, 5778}, {31, 1545, 1, 5778}, {32, 1546, 2, 5778}, {33, 1547, 1, 5778}, {24, 1543, 1, 5779}, {27, 1545, 2, 5779}, {28, 1546, 2, 5779}, {31, 1548, 1, 5779}, {32, 1549, 2, 5779}, {33, 1550, 1, 5779}, {24, 1544, 1, 5780}, {27, 1546, 2, 5780}, {28, 1547, 2, 5780}, {31, 1549, 1, 5780}, {32, 1550, 2, 5780}, {33, 1551, 1, 5780}, {25, 1542, 1, 5781}, {28, 1543, 2, 5781}, {29, 1544, 2, 5781}, {32, 1545, 1, 5781}, {33, 1546, 2, 5781}, {34, 1547, 1, 5781}, {25, 1543, 1, 5782}, {28, 1545, 2, 5782}, {29, 1546, 2, 5782}, {32, 1548, 1, 5782}, {33, 1549, 2, 5782}, {34, 1550, 1, 5782}, {25, 1544, 1, 5783}, {28, 1546, 2, 5783}, {29, 1547, 2, 5783}, {32, 1549, 1, 5783}, {33, 1550, 2, 5783}, {34, 1551, 1, 5783}, {20, 1793, 1, 5784}, {21, 1794, 2, 5784}, {22, 1795, 2, 5784}, {23, 1796, 1, 5784}, {24, 1797, 2, 5784}, {25, 1798, 1, 5784}, {20, 1794, 1, 5785}, {21, 1799, 2, 5785}, {22, 1800, 2, 5785}, {23, 1801, 1, 5785}, {24, 1802, 2, 5785}, {25, 1803, 1, 5785}, {20, 1795, 1, 5786}, {21, 1800, 2, 5786}, {22, 1804, 2, 5786}, {23, 1805, 1, 5786}, {24, 1806, 2, 5786}, {25, 1807, 1, 5786}, {20, 1796, 1, 5787}, {21, 1801, 2, 5787}, {22, 1805, 2, 5787}, {23, 1808, 1, 5787}, {24, 1809, 2, 5787}, {25, 1810, 1, 5787}, {20, 1797, 1, 5788}, {21, 1802, 2, 5788}, {22, 1806, 2, 5788}, {23, 1809, 1, 5788}, {24, 1811, 2, 5788}, {25, 1812, 1, 5788}, {20, 1798, 1, 5789}, {21, 1803, 2, 5789}, {22, 1807, 2, 5789}, {23, 1810, 1, 5789}, {24, 1812, 2, 5789}, {25, 1813, 1, 5789}, {21, 1793, 1, 5790}, {23, 1794, 2, 5790}, {24, 1795, 2, 5790}, {26, 1796, 1, 5790}, {27, 1797, 2, 5790}, {28, 1798, 1, 5790}, {21, 1794, 1, 5791}, {23, 1799, 2, 5791}, {24, 1800, 2, 5791}, {26, 1801, 1, 5791}, {27, 1802, 2, 5791}, {28, 1803, 1, 5791}, {21, 1795, 1, 5792}, {23, 1800, 2, 5792}, {24, 1804, 2, 5792}, {26, 1805, 1, 5792}, {27, 1806, 2, 5792}, {28, 1807, 1, 5792}, {21, 1796, 1, 5793}, {23, 1801, 2, 5793}, {24, 1805, 2, 5793}, {26, 1808, 1, 5793}, {27, 1809, 2, 5793}, {28, 1810, 1, 5793}, {21, 1797, 1, 5794}, {23, 1802, 2, 5794}, {24, 1806, 2, 5794}, {26, 1809, 1, 5794}, {27, 1811, 2, 5794}, {28, 1812, 1, 5794}, {21, 1798, 1, 5795}, {23, 1803, 2, 5795}, {24, 1807, 2, 5795}, {26, 1810, 1, 5795}, {27, 1812, 2, 5795}, {28, 1813, 1, 5795}, {22, 1793, 1, 5796}, {24, 1794, 2, 5796}, {25, 1795, 2, 5796}, {27, 1796, 1, 5796}, {28, 1797, 2, 5796}, {29, 1798, 1, 5796}, {22, 1794, 1, 5797}, {24, 1799, 2, 5797}, {25, 1800, 2, 5797}, {27, 1801, 1, 5797}, {28, 1802, 2, 5797}, {29, 1803, 1, 5797}, {22, 1795, 1, 5798}, {24, 1800, 2, 5798}, {25, 1804, 2, 5798}, {27, 1805, 1, 5798}, {28, 1806, 2, 5798}, {29, 1807, 1, 5798}, {22, 1796, 1, 5799}, {24, 1801, 2, 5799}, {25, 1805, 2, 5799}, {27, 1808, 1, 5799}, {28, 1809, 2, 5799}, {29, 1810, 1, 5799}, {22, 1797, 1, 5800}, {24, 1802, 2, 5800}, {25, 1806, 2, 5800}, {27, 1809, 1, 5800}, {28, 1811, 2, 5800}, {29, 1812, 1, 5800}, {22, 1798, 1, 5801}, {24, 1803, 2, 5801}, {25, 1807, 2, 5801}, {27, 1810, 1, 5801}, {28, 1812, 2, 5801}, {29, 1813, 1, 5801}, {23, 1793, 1, 5802}, {26, 1794, 2, 5802}, {27, 1795, 2, 5802}, {30, 1796, 1, 5802}, {31, 1797, 2, 5802}, {32, 1798, 1, 5802}, {23, 1794, 1, 5803}, {26, 1799, 2, 5803}, {27, 1800, 2, 5803}, {30, 1801, 1, 5803}, {31, 1802, 2, 5803}, {32, 1803, 1, 5803}, {23, 1795, 1, 5804}, {26, 1800, 2, 5804}, {27, 1804, 2, 5804}, {30, 1805, 1, 5804}, {31, 1806, 2, 5804}, {32, 1807, 1, 5804}, {23, 1796, 1, 5805}, {26, 1801, 2, 5805}, {27, 1805, 2, 5805}, {30, 1808, 1, 5805}, {31, 1809, 2, 5805}, {32, 1810, 1, 5805}, {23, 1797, 1, 5806}, {26, 1802, 2, 5806}, {27, 1806, 2, 5806}, {30, 1809, 1, 5806}, {31, 1811, 2, 5806}, {32, 1812, 1, 5806}, {23, 1798, 1, 5807}, {26, 1803, 2, 5807}, {27, 1807, 2, 5807}, {30, 1810, 1, 5807}, {31, 1812, 2, 5807}, {32, 1813, 1, 5807}, {24, 1793, 1, 5808}, {27, 1794, 2, 5808}, {28, 1795, 2, 5808}, {31, 1796, 1, 5808}, {32, 1797, 2, 5808}, {33, 1798, 1, 5808}, {24, 1794, 1, 5809}, {27, 1799, 2, 5809}, {28, 1800, 2, 5809}, {31, 1801, 1, 5809}, {32, 1802, 2, 5809}, {33, 1803, 1, 5809}, {24, 1795, 1, 5810}, {27, 1800, 2, 5810}, {28, 1804, 2, 5810}, {31, 1805, 1, 5810}, {32, 1806, 2, 5810}, {33, 1807, 1, 5810}, {24, 1796, 1, 5811}, {27, 1801, 2, 5811}, {28, 1805, 2, 5811}, {31, 1808, 1, 5811}, {32, 1809, 2, 5811}, {33, 1810, 1, 5811}, {24, 1797, 1, 5812}, {27, 1802, 2, 5812}, {28, 1806, 2, 5812}, {31, 1809, 1, 5812}, {32, 1811, 2, 5812}, {33,

1812, 1, 5812}, {24, 1798, 1, 5813}, {27, 1803, 2, 5813}, {28, 1807, 2, 5813}, {31, 1810, 1, 5813}, {32, 1812, 2, 5813}, {33, 1813, 1, 5813}, {25, 1793, 1, 5814}, {28, 1794, 2, 5814}, {29, 1795, 2, 5814}, {32, 1796, 1, 5814}, {33, 1797, 2, 5814}, {34, 1798, 1, 5814}, {25, 1794, 1, 5815}, {28, 1799, 2, 5815}, {29, 1800, 2, 5815}, {32, 1801, 1, 5815}, {33, 1802, 2, 5815}, {34, 1803, 1, 5815}, {25, 1795, 1, 5816}, {28, 1800, 2, 5816}, {29, 1804, 2, 5816}, {32, 1805, 1, 5816}, {33, 1806, 2, 5816}, {34, 1807, 1, 5816}, {25, 1796, 1, 5817}, {28, 1801, 2, 5817}, {29, 1805, 2, 5817}, {32, 1808, 1, 5817}, {33, 1809, 2, 5817}, {34, 1810, 1, 5817}, {25, 1797, 1, 5818}, {28, 1802, 2, 5818}, {29, 1806, 2, 5818}, {32, 1809, 1, 5818}, {33, 1811, 2, 5818}, {34, 1812, 1, 5818}, {25, 1798, 1, 5819}, {28, 1803, 2, 5819}, {29, 1807, 2, 5819}, {32, 1810, 1, 5819}, {33, 1812, 2, 5819}, {34, 1813, 1, 5819}, {20, 1970, 1, 5820}, {21, 1971, 2, 5820}, {22, 1972, 2, 5820}, {23, 1973, 1, 5820}, {24, 1974, 2, 5820}, {25, 1975, 1, 5820}, {20, 1976, 1, 5821}, {21, 1977, 2, 5821}, {22, 1978, 2, 5821}, {23, 1979, 1, 5821}, {24, 1980, 2, 5821}, {25, 1981, 1, 5821}, {20, 1982, 1, 5822}, {21, 1983, 2, 5822}, {22, 1984, 2, 5822}, {23, 1985, 1, 5822}, {24, 1986, 2, 5822}, {25, 1987, 1, 5822}, {21, 1970, 1, 5823}, {23, 1971, 2, 5823}, {24, 1972, 2, 5823}, {26, 1973, 1, 5823}, {27, 1974, 2, 5823}, {28, 1975, 1, 5823}, {21, 1976, 1, 5824}, {23, 1977, 2, 5824}, {24, 1978, 2, 5824}, {26, 1979, 1, 5824}, {27, 1980, 2, 5824}, {28, 1981, 1, 5824}, {21, 1982, 1, 5825}, {23, 1983, 2, 5825}, {24, 1984, 2, 5825}, {26, 1985, 1, 5825}, {27, 1986, 2, 5825}, {28, 1987, 1, 5825}, {22, 1970, 1, 5826}, {24, 1971, 2, 5826}, {25, 1972, 2, 5826}, {27, 1973, 1, 5826}, {28, 1974, 2, 5826}, {29, 1975, 1, 5826}, {22, 1976, 1, 5827}, {24, 1977, 2, 5827}, {25, 1978, 2, 5827}, {27, 1979, 1, 5827}, {28, 1980, 2, 5827}, {29, 1981, 1, 5827}, {22, 1982, 1, 5828}, {24, 1983, 2, 5828}, {25, 1984, 2, 5828}, {27, 1985, 1, 5828}, {28, 1986, 2, 5828}, {29, 1987, 1, 5828}, {23, 1970, 1, 5829}, {26, 1971, 2, 5829}, {27, 1972, 2, 5829}, {30, 1973, 1, 5829}, {31, 1974, 2, 5829}, {32, 1975, 1, 5829}, {23, 1976, 1, 5830}, {26, 1977, 2, 5830}, {27, 1978, 2, 5830}, {30, 1979, 1, 5830}, {31, 1980, 2, 5830}, {32, 1981, 1, 5830}, {23, 1982, 1, 5831}, {26, 1983, 2, 5831}, {27, 1984, 2, 5831}, {30, 1985, 1, 5831}, {31, 1986, 2, 5831}, {32, 1987, 1, 5831}, {24, 1970, 1, 5832}, {27, 1971, 2, 5832}, {28, 1972, 2, 5832}, {31, 1973, 1, 5832}, {32, 1974, 2, 5832}, {33, 1975, 1, 5832}, {24, 1976, 1, 5833}, {27, 1977, 2, 5833}, {28, 1978, 2, 5833}, {31, 1979, 1, 5833}, {32, 1980, 2, 5833}, {33, 1981, 1, 5833}, {24, 1982, 1, 5834}, {27, 1983, 2, 5834}, {28, 1984, 2, 5834}, {31, 1985, 1, 5834}, {32, 1986, 2, 5834}, {33, 1987, 1, 5834}, {25, 1970, 1, 5835}, {28, 1971, 2, 5835}, {29, 1972, 2, 5835}, {32, 1973, 1, 5835}, {33, 1974, 2, 5835}, {34, 1975, 1, 5835}, {25, 1976, 1, 5836}, {28, 1977, 2, 5836}, {29, 1978, 2, 5836}, {32, 1979, 1, 5836}, {33, 1980, 2, 5836}, {34, 1981, 1, 5836}, {25, 1982, 1, 5837}, {28, 1983, 2, 5837}, {29, 1984, 2, 5837}, {32, 1985, 1, 5837}, {33, 1986, 2, 5837}, {34, 1987, 1, 5837}, {20, 2067, 1, 5838}, {21, 2068, 2, 5838}, {22, 2069, 2, 5838}, {23, 2070, 1, 5838}, {24, 2071, 2, 5838}, {25, 2072, 1, 5838}, {21, 2067, 1, 5839}, {23, 2068, 2, 5839}, {24, 2069, 2, 5839}, {26, 2070, 1, 5839}, {27, 2071, 2, 5839}, {28, 2072, 1, 5839}, {22, 2067, 1, 5840}, {24, 2068, 2, 5840}, {25, 2069, 2, 5840}, {27, 2070, 1, 5840}, {28, 2071, 2, 5840}, {29, 2072, 1, 5840}, {23, 2067, 1, 5841}, {26, 2068, 2, 5841}, {27, 2069, 2, 5841}, {30, 2070, 1, 5841}, {31, 2071, 2, 5841}, {32, 2072, 1, 5841}, {24, 2067, 1, 5842}, {27, 2068, 2, 5842}, {28, 2069, 2, 5842}, {31, 2070, 1, 5842}, {32, 2071, 2, 5842}, {33, 2072, 1, 5842}, {25, 2067, 1, 5843}, {28, 2068, 2, 5843}, {29, 2069, 2, 5843}, {32, 2070, 1, 5843}, {33, 2071, 2, 5843}, {34, 2072, 1, 5843}, {20, 1394, 1, 5844}, {21, 1395, 3, 5844}, {22, 1396, 3, 5844}, {23, 1397, 3, 5844}, {24, 1398, 6, 5844}, {25, 1399, 3, 5844}, {26, 1400, 1, 5844}, {27, 1401, 3, 5844}, {28, 1402, 3, 5844}, {29, 1403, 1, 5844}, {20, 1404, 1, 5845}, {21, 1405, 3, 5845}, {22, 1406, 3, 5845}, {23, 1407, 3, 5845}, {24, 1408, 6, 5845}, {25, 1409, 3, 5845}, {26, 1410, 1, 5845}, {27, 1411, 3, 5845}, {28, 1412, 3, 5845}, {29, 1413, 1, 5845}, {20, 1414, 1, 5846}, {21, 1415, 3, 5846}, {22, 1416, 3, 5846}, {23, 1417, 3, 5846}, {24, 1418, 6, 5846}, {25, 1419, 3, 5846}, {26, 1420, 1, 5846}, {27, 1421, 3, 5846}, {28, 1422, 3, 5846}, {29, 1423, 1, 5846}, {21, 1394, 1, 5847}, {23, 1395, 3, 5847}, {24, 1396, 3, 5847}, {26, 1397, 3, 5847}, {27, 1398, 6, 5847}, {28, 1399, 3, 5847}, {30, 1400, 1, 5847}, {31, 1401, 3, 5847}, {32, 1402, 3, 5847}, {33, 1403, 1, 5847}, {21, 1404, 1, 5848}, {23, 1405, 3, 5848}, {24, 1406, 3, 5848}, {26, 1407, 3, 5848}, {27, 1408, 6, 5848}, {28, 1409, 3, 5848}, {30, 1410, 1, 5848}, {31, 1411, 3, 5848}, {32, 1412, 3, 5848}, {33, 1413, 1, 5848}, {21, 1414, 1, 5849}, {23, 1415, 3, 5849}, {24, 1416, 3, 5849}, {26, 1417, 3, 5849}, {27, 1418, 6, 5849}, {28, 1419, 3, 5849}, {30, 1420, 1, 5849}, {31, 1421, 3, 5849}, {32, 1422, 3, 5849}, {33, 1423, 1, 5849}, {22, 1394, 1, 5850}, {24, 1395, 3, 5850}, {25, 1396, 3, 5850}, {27, 1397, 3, 5850}, {28, 1398, 6, 5850}, {29, 1399, 3, 5850}, {31, 1400, 1, 5850}, {32, 1401, 3, 5850}, {33, 1402, 3, 5850}, {34, 1403, 1, 5850}, {22, 1404, 1, 5851}, {24, 1405, 3, 5851}, {25, 1406, 3, 5851}, {27, 1407, 3, 5851}, {28, 1408, 6, 5851}, {29, 1409, 3, 5851}, {31, 1410, 1, 5851}, {32, 1411, 3, 5851}, {33, 1412, 3, 5851}, {34, 1413, 1, 5851}, {22, 1414, 1, 5852}, {24, 1415, 3, 5852}, {25, 1416, 3, 5852}, {27, 1417, 3, 5852}, {28, 1418, 6, 5852}, {29, 1419, 3, 5852}, {31, 1420, 1, 5852}, {32, 1421, 3, 5852}, {33, 1422, 3, 5852}, {34, 1423, 1, 5852}, {20, 1424, 1, 5853}, {21, 1425, 3, 5853}, {22, 1426, 3, 5853}, {23, 1427, 3, 5853}, {24, 1428, 6, 5853}, {25, 1429, 3, 5853}, {26, 1430, 1, 5853}, {27, 1431, 3, 5853}, {28, 1432, 3, 5853}, {29, 1433, 1, 5853}, {20, 1425, 1, 5854}, {21, 1427, 3, 5854}, {22, 1428, 3, 5854}, {23, 1430, 3, 5854}, {24, 1431, 6, 5854}, {25, 1432, 3, 5854}, {26, 1434,

1, 5854}, {27, 1435, 3, 5854}, {28, 1436, 3, 5854}, {29, 1437, 1, 5854}, {20, 1426, 1,
5855}, {21, 1428, 3, 5855}, {22, 1429, 3, 5855}, {23, 1431, 3, 5855}, {24, 1432, 6, 5855},
{25, 1433, 3, 5855}, {26, 1435, 1, 5855}, {27, 1436, 3, 5855}, {28, 1437, 3, 5855}, {29,
1438, 1, 5855}, {20, 1439, 1, 5856}, {21, 1440, 3, 5856}, {22, 1441, 3, 5856}, {23, 1442,
3, 5856}, {24, 1443, 6, 5856}, {25, 1444, 3, 5856}, {26, 1445, 1, 5856}, {27, 1446, 3,
5856}, {28, 1447, 3, 5856}, {29, 1448, 1, 5856}, {20, 1440, 1, 5857}, {21, 1442, 3, 5857},
{22, 1443, 3, 5857}, {23, 1445, 3, 5857}, {24, 1446, 6, 5857}, {25, 1447, 3, 5857}, {26,
1449, 1, 5857}, {27, 1450, 3, 5857}, {28, 1451, 3, 5857}, {29, 1452, 1, 5857}, {20, 1441,
1, 5858}, {21, 1443, 3, 5858}, {22, 1444, 3, 5858}, {23, 1446, 3, 5858}, {24, 1447, 6,
5858}, {25, 1448, 3, 5858}, {26, 1450, 1, 5858}, {27, 1451, 3, 5858}, {28, 1452, 3, 5858},
{29, 1453, 1, 5858}, {20, 1454, 1, 5859}, {21, 1455, 3, 5859}, {22, 1456, 3, 5859}, {23,
1457, 3, 5859}, {24, 1458, 6, 5859}, {25, 1459, 3, 5859}, {26, 1460, 1, 5859}, {27, 1461,
3, 5859}, {28, 1462, 3, 5859}, {29, 1463, 1, 5859}, {20, 1455, 1, 5860}, {21, 1457, 3,
5860}, {22, 1458, 3, 5860}, {23, 1460, 3, 5860}, {24, 1461, 6, 5860}, {25, 1462, 3, 5860},
{26, 1464, 1, 5860}, {27, 1465, 3, 5860}, {28, 1466, 3, 5860}, {29, 1467, 1, 5860}, {20,
1456, 1, 5861}, {21, 1458, 3, 5861}, {22, 1459, 3, 5861}, {23, 1461, 3, 5861}, {24, 1462,
6, 5861}, {25, 1463, 3, 5861}, {26, 1465, 1, 5861}, {27, 1466, 3, 5861}, {28, 1467, 3,
5861}, {29, 1468, 1, 5861}, {21, 1424, 1, 5862}, {23, 1425, 3, 5862}, {24, 1426, 3, 5862},
{26, 1427, 3, 5862}, {27, 1428, 6, 5862}, {28, 1429, 3, 5862}, {30, 1430, 1, 5862}, {31,
1431, 3, 5862}, {32, 1432, 3, 5862}, {33, 1433, 1, 5862}, {21, 1425, 1, 5863}, {23, 1427,
3, 5863}, {24, 1428, 3, 5863}, {26, 1430, 3, 5863}, {27, 1431, 6, 5863}, {28, 1432, 3,
5863}, {30, 1434, 1, 5863}, {31, 1435, 3, 5863}, {32, 1436, 3, 5863}, {33, 1437, 1, 5863},
{21, 1426, 1, 5864}, {23, 1428, 3, 5864}, {24, 1429, 3, 5864}, {26, 1431, 3, 5864}, {27,
1432, 6, 5864}, {28, 1433, 3, 5864}, {30, 1435, 1, 5864}, {31, 1436, 3, 5864}, {32, 1437,
3, 5864}, {33, 1438, 1, 5864}, {21, 1439, 1, 5865}, {23, 1440, 3, 5865}, {24, 1441, 3,
5865}, {26, 1442, 3, 5865}, {27, 1443, 6, 5865}, {28, 1444, 3, 5865}, {30, 1445, 1, 5865},
{31, 1446, 3, 5865}, {32, 1447, 3, 5865}, {33, 1448, 1, 5865}, {21, 1440, 1, 5866}, {23,
1442, 3, 5866}, {24, 1443, 3, 5866}, {26, 1445, 3, 5866}, {27, 1446, 6, 5866}, {28, 1447,
3, 5866}, {30, 1449, 1, 5866}, {31, 1450, 3, 5866}, {32, 1451, 3, 5866}, {33, 1452, 1,
5866}, {21, 1441, 1, 5867}, {23, 1443, 3, 5867}, {24, 1444, 3, 5867}, {26, 1446, 3, 5867},
{27, 1447, 6, 5867}, {28, 1448, 3, 5867}, {30, 1450, 1, 5867}, {31, 1451, 3, 5867}, {32,
1452, 3, 5867}, {33, 1453, 1, 5867}, {21, 1454, 1, 5868}, {23, 1455, 3, 5868}, {24, 1456,
3, 5868}, {26, 1457, 3, 5868}, {27, 1458, 6, 5868}, {28, 1459, 3, 5868}, {30, 1460, 1,
5868}, {31, 1461, 3, 5868}, {32, 1462, 3, 5868}, {33, 1463, 1, 5868}, {21, 1455, 1, 5869},
{23, 1457, 3, 5869}, {24, 1458, 3, 5869}, {26, 1460, 3, 5869}, {27, 1461, 6, 5869}, {28,
1462, 3, 5869}, {30, 1464, 1, 5869}, {31, 1465, 3, 5869}, {32, 1466, 3, 5869}, {33, 1467,
1, 5869}, {21, 1456, 1, 5870}, {23, 1458, 3, 5870}, {24, 1459, 3, 5870}, {26, 1461, 3,
5870}, {27, 1462, 6, 5870}, {28, 1463, 3, 5870}, {30, 1465, 1, 5870}, {31, 1466, 3, 5870},
{32, 1467, 3, 5870}, {33, 1468, 1, 5870}, {22, 1424, 1, 5871}, {24, 1425, 3, 5871}, {25,
1426, 3, 5871}, {27, 1427, 3, 5871}, {28, 1428, 6, 5871}, {29, 1429, 3, 5871}, {31, 1430,
1, 5871}, {32, 1431, 3, 5871}, {33, 1432, 3, 5871}, {34, 1433, 1, 5871}, {22, 1425, 1,
5872}, {24, 1427, 3, 5872}, {25, 1428, 3, 5872}, {27, 1430, 3, 5872}, {28, 1431, 6, 5872},
{29, 1432, 3, 5872}, {31, 1434, 1, 5872}, {32, 1435, 3, 5872}, {33, 1436, 3, 5872}, {34,
1437, 1, 5872}, {22, 1426, 1, 5873}, {24, 1428, 3, 5873}, {25, 1429, 3, 5873}, {27, 1431,
3, 5873}, {28, 1432, 6, 5873}, {29, 1433, 3, 5873}, {31, 1435, 1, 5873}, {32, 1436, 3,
5873}, {33, 1437, 3, 5873}, {34, 1438, 1, 5873}, {22, 1439, 1, 5874}, {24, 1440, 3, 5874},
{25, 1441, 3, 5874}, {27, 1442, 3, 5874}, {28, 1443, 6, 5874}, {29, 1444, 3, 5874}, {31,
1445, 1, 5874}, {32, 1446, 3, 5874}, {33, 1447, 3, 5874}, {34, 1448, 1, 5874}, {22, 1440,
1, 5875}, {24, 1442, 3, 5875}, {25, 1443, 3, 5875}, {27, 1445, 3, 5875}, {28, 1446, 6,
5875}, {29, 1447, 3, 5875}, {31, 1449, 1, 5875}, {32, 1450, 3, 5875}, {33, 1451, 3, 5875},
{34, 1452, 1, 5875}, {22, 1441, 1, 5876}, {24, 1443, 3, 5876}, {25, 1444, 3, 5876}, {27,
1446, 3, 5876}, {28, 1447, 6, 5876}, {29, 1448, 3, 5876}, {31, 1450, 1, 5876}, {32, 1451,
3, 5876}, {33, 1452, 3, 5876}, {34, 1453, 1, 5876}, {22, 1454, 1, 5877}, {24, 1455, 3,
5877}, {25, 1456, 3, 5877}, {27, 1457, 3, 5877}, {28, 1458, 6, 5877}, {29, 1459, 3, 5877},
{31, 1460, 1, 5877}, {32, 1461, 3, 5877}, {33, 1462, 3, 5877}, {34, 1463, 1, 5877}, {22,
1455, 1, 5878}, {24, 1457, 3, 5878}, {25, 1458, 3, 5878}, {27, 1460, 3, 5878}, {28, 1461,
6, 5878}, {29, 1462, 3, 5878}, {31, 1464, 1, 5878}, {32, 1465, 3, 5878}, {33, 1466, 3,
5878}, {34, 1467, 1, 5878}, {22, 1456, 1, 5879}, {24, 1458, 3, 5879}, {25, 1459, 3, 5879},
{27, 1461, 3, 5879}, {28, 1462, 6, 5879}, {29, 1463, 3, 5879}, {31, 1465, 1, 5879}, {32,
1466, 3, 5879}, {33, 1467, 3, 5879}, {34, 1468, 1, 5879}, {20, 1542, 1, 5880}, {21, 1543,
3, 5880}, {22, 1544, 3, 5880}, {23, 1545, 3, 5880}, {24, 1546, 6, 5880}, {25, 1547, 3,
5880}, {26, 1548, 1, 5880}, {27, 1549, 3, 5880}, {28, 1550, 3, 5880}, {29, 1551, 1, 5880},
{21, 1542, 1, 5881}, {23, 1543, 3, 5881}, {24, 1544, 3, 5881}, {26, 1545, 3, 5881}, {27,
1546, 6, 5881}, {28, 1547, 3, 5881}, {30, 1548, 1, 5881}, {31, 1549, 3, 5881}, {32, 1550,
3, 5881}, {33, 1551, 1, 5881}, {22, 1542, 1, 5882}, {24, 1543, 3, 5882}, {25, 1544, 3,
5882}, {27, 1545, 3, 5882}, {28, 1546, 6, 5882}, {29, 1547, 3, 5882}, {31, 1548, 1, 5882},
{32, 1549, 3, 5882}, {33, 1550, 3, 5882}, {34, 1551, 1, 5882}, {20, 1552, 1, 5883}, {21,
1553, 3, 5883}, {22, 1554, 3, 5883}, {23, 1555, 3, 5883}, {24, 1556, 6, 5883}, {25, 1557,
3, 5883}, {26, 1558, 1, 5883}, {27, 1559, 3, 5883}, {28, 1560, 3, 5883}, {29, 1561, 1,

5883}, {20, 1553, 1, 5884}, {21, 1555, 3, 5884}, {22, 1556, 3, 5884}, {23, 1558, 3, 5884}, {24, 1559, 6, 5884}, {25, 1560, 3, 5884}, {26, 1562, 1, 5884}, {27, 1563, 3, 5884}, {28, 1564, 3, 5884}, {29, 1565, 1, 5884}, {20, 1554, 1, 5885}, {21, 1556, 3, 5885}, {22, 1557, 3, 5885}, {23, 1559, 3, 5885}, {24, 1560, 6, 5885}, {25, 1561, 3, 5885}, {26, 1563, 1, 5885}, {27, 1564, 3, 5885}, {28, 1565, 3, 5885}, {29, 1566, 1, 5885}, {21, 1552, 1, 5886}, {23, 1553, 3, 5886}, {24, 1554, 3, 5886}, {26, 1555, 3, 5886}, {27, 1556, 6, 5886}, {28, 1557, 3, 5886}, {30, 1558, 1, 5886}, {31, 1559, 3, 5886}, {32, 1560, 3, 5886}, {33, 1561, 1, 5886}, {21, 1553, 1, 5887}, {23, 1555, 3, 5887}, {24, 1556, 3, 5887}, {26, 1558, 3, 5887}, {27, 1559, 6, 5887}, {28, 1560, 3, 5887}, {30, 1562, 1, 5887}, {31, 1563, 3, 5887}, {32, 1564, 3, 5887}, {33, 1565, 1, 5887}, {21, 1554, 1, 5888}, {23, 1556, 3, 5888}, {24, 1557, 3, 5888}, {26, 1559, 3, 5888}, {27, 1560, 6, 5888}, {28, 1561, 3, 5888}, {30, 1563, 1, 5888}, {31, 1564, 3, 5888}, {32, 1565, 3, 5888}, {33, 1566, 1, 5888}, {22, 1552, 1, 5889}, {24, 1553, 3, 5889}, {25, 1554, 3, 5889}, {27, 1555, 3, 5889}, {28, 1556, 6, 5889}, {29, 1557, 3, 5889}, {31, 1558, 1, 5889}, {32, 1559, 3, 5889}, {33, 1560, 3, 5889}, {34, 1561, 1, 5889}, {22, 1553, 1, 5890}, {24, 1555, 3, 5890}, {25, 1556, 3, 5890}, {27, 1558, 3, 5890}, {28, 1559, 6, 5890}, {29, 1560, 3, 5890}, {31, 1562, 1, 5890}, {32, 1563, 3, 5890}, {33, 1564, 3, 5890}, {34, 1565, 1, 5890}, {22, 1554, 1, 5891}, {24, 1556, 3, 5891}, {25, 1557, 3, 5891}, {27, 1559, 3, 5891}, {28, 1560, 6, 5891}, {29, 1561, 3, 5891}, {31, 1563, 1, 5891}, {32, 1564, 3, 5891}, {33, 1565, 3, 5891}, {34, 1566, 1, 5891}, {20, 1814, 1, 5892}, {21, 1815, 3, 5892}, {22, 1816, 3, 5892}, {23, 1817, 3, 5892}, {24, 1818, 6, 5892}, {25, 1819, 3, 5892}, {26, 1820, 1, 5892}, {27, 1821, 3, 5892}, {28, 1822, 3, 5892}, {29, 1823, 1, 5892}, {20, 1824, 1, 5893}, {21, 1825, 3, 5893}, {22, 1826, 3, 5893}, {23, 1827, 3, 5893}, {24, 1828, 6, 5893}, {25, 1829, 3, 5893}, {26, 1830, 1, 5893}, {27, 1831, 3, 5893}, {28, 1832, 3, 5893}, {29, 1833, 1, 5893}, {20, 1834, 1, 5894}, {21, 1835, 3, 5894}, {22, 1836, 3, 5894}, {23, 1837, 3, 5894}, {24, 1838, 6, 5894}, {25, 1839, 3, 5894}, {26, 1840, 1, 5894}, {27, 1841, 3, 5894}, {28, 1842, 3, 5894}, {29, 1843, 1, 5894}, {20, 1844, 1, 5895}, {21, 1845, 3, 5895}, {22, 1846, 3, 5895}, {23, 1847, 3, 5895}, {24, 1848, 6, 5895}, {25, 1849, 3, 5895}, {26, 1850, 1, 5895}, {27, 1851, 3, 5895}, {28, 1852, 3, 5895}, {29, 1853, 1, 5895}, {20, 1854, 1, 5896}, {21, 1855, 3, 5896}, {22, 1856, 3, 5896}, {23, 1857, 3, 5896}, {24, 1858, 6, 5896}, {25, 1859, 3, 5896}, {26, 1860, 1, 5896}, {27, 1861, 3, 5896}, {28, 1862, 3, 5896}, {29, 1863, 1, 5896}, {20, 1864, 1, 5897}, {21, 1865, 3, 5897}, {22, 1866, 3, 5897}, {23, 1867, 3, 5897}, {24, 1868, 6, 5897}, {25, 1869, 3, 5897}, {26, 1870, 1, 5897}, {27, 1871, 3, 5897}, {28, 1872, 3, 5897}, {29, 1873, 1, 5897}, {21, 1814, 1, 5898}, {23, 1815, 3, 5898}, {24, 1816, 3, 5898}, {26, 1817, 3, 5898}, {27, 1818, 6, 5898}, {28, 1819, 3, 5898}, {30, 1820, 1, 5898}, {31, 1821, 3, 5898}, {32, 1822, 3, 5898}, {33, 1823, 1, 5898}, {21, 1824, 1, 5899}, {23, 1825, 3, 5899}, {24, 1826, 3, 5899}, {26, 1827, 3, 5899}, {27, 1828, 6, 5899}, {28, 1829, 3, 5899}, {30, 1830, 1, 5899}, {31, 1831, 3, 5899}, {32, 1832, 3, 5899}, {33, 1833, 1, 5899}, {21, 1834, 1, 5900}, {23, 1835, 3, 5900}, {24, 1836, 3, 5900}, {26, 1837, 3, 5900}, {27, 1838, 6, 5900}, {28, 1839, 3, 5900}, {30, 1840, 1, 5900}, {31, 1841, 3, 5900}, {32, 1842, 3, 5900}, {33, 1843, 1, 5900}, {21, 1844, 1, 5901}, {23, 1845, 3, 5901}, {24, 1846, 3, 5901}, {26, 1847, 3, 5901}, {27, 1848, 6, 5901}, {28, 1849, 3, 5901}, {30, 1850, 1, 5901}, {31, 1851, 3, 5901}, {32, 1852, 3, 5901}, {33, 1853, 1, 5901}, {21, 1854, 1, 5902}, {23, 1855, 3, 5902}, {24, 1856, 3, 5902}, {26, 1857, 3, 5902}, {27, 1858, 6, 5902}, {28, 1859, 3, 5902}, {30, 1860, 1, 5902}, {31, 1861, 3, 5902}, {32, 1862, 3, 5902}, {33, 1863, 1, 5902}, {21, 1864, 1, 5903}, {23, 1865, 3, 5903}, {24, 1866, 3, 5903}, {26, 1867, 3, 5903}, {27, 1868, 6, 5903}, {28, 1869, 3, 5903}, {30, 1870, 1, 5903}, {31, 1871, 3, 5903}, {32, 1872, 3, 5903}, {33, 1873, 1, 5903}, {22, 1814, 1, 5904}, {24, 1815, 3, 5904}, {25, 1816, 3, 5904}, {27, 1817, 3, 5904}, {28, 1818, 6, 5904}, {29, 1819, 3, 5904}, {31, 1820, 1, 5904}, {32, 1821, 3, 5904}, {33, 1822, 3, 5904}, {34, 1823, 1, 5904}, {22, 1824, 1, 5905}, {24, 1825, 3, 5905}, {25, 1826, 3, 5905}, {27, 1827, 3, 5905}, {28, 1828, 6, 5905}, {29, 1829, 3, 5905}, {31, 1830, 1, 5905}, {32, 1831, 3, 5905}, {33, 1832, 3, 5905}, {34, 1833, 1, 5905}, {22, 1834, 1, 5906}, {24, 1835, 3, 5906}, {25, 1836, 3, 5906}, {27, 1837, 3, 5906}, {28, 1838, 6, 5906}, {29, 1839, 3, 5906}, {31, 1840, 1, 5906}, {32, 1841, 3, 5906}, {33, 1842, 3, 5906}, {34, 1843, 1, 5906}, {22, 1844, 1, 5907}, {24, 1845, 3, 5907}, {25, 1846, 3, 5907}, {27, 1847, 3, 5907}, {28, 1848, 6, 5907}, {29, 1849, 3, 5907}, {31, 1850, 1, 5907}, {32, 1851, 3, 5907}, {33, 1852, 3, 5907}, {34, 1853, 1, 5907}, {22, 1854, 1, 5908}, {24, 1855, 3, 5908}, {25, 1856, 3, 5908}, {27, 1857, 3, 5908}, {28, 1858, 6, 5908}, {29, 1859, 3, 5908}, {31, 1860, 1, 5908}, {32, 1861, 3, 5908}, {33, 1862, 3, 5908}, {34, 1863, 1, 5908}, {22, 1864, 1, 5909}, {24, 1865, 3, 5909}, {25, 1866, 3, 5909}, {27, 1867, 3, 5909}, {28, 1868, 6, 5909}, {29, 1869, 3, 5909}, {31, 1870, 1, 5909}, {32, 1871, 3, 5909}, {33, 1872, 3, 5909}, {34, 1873, 1, 5909}, {20, 1988, 1, 5910}, {21, 1989, 3, 5910}, {22, 1990, 3, 5910}, {23, 1991, 3, 5910}, {24, 1992, 6, 5910}, {25, 1993, 3, 5910}, {26, 1994, 1, 5910}, {27, 1995, 3, 5910}, {28, 1996, 3, 5910}, {29, 1997, 1, 5910}, {20, 1998, 1, 5911}, {21, 1999, 3, 5911}, {22, 2000, 3, 5911}, {23, 2001, 3, 5911}, {24, 2002, 6, 5911}, {25, 2003, 3, 5911}, {26, 2004, 1, 5911}, {27, 2005, 3, 5911}, {28, 2006, 3, 5911}, {29, 2007, 1, 5911}, {20, 2008, 1, 5912}, {21, 2009, 3, 5912}, {22, 2010, 3, 5912}, {23, 2011, 3, 5912}, {24, 2012, 6, 5912}, {25, 2013, 3, 5912}, {26, 2014, 1, 5912}, {27, 2015, 3, 5912}, {28, 2016, 3, 5912}, {29, 2017, 1, 5912}, {21, 1988, 1, 5913}, {23, 1989, 3, 5913}, {24, 1990, 3, 5913},

{26, 1991, 3, 5913}, {27, 1992, 6, 5913}, {28, 1993, 3, 5913}, {30, 1994, 1, 5913}, {31, 1995, 3, 5913}, {32, 1996, 3, 5913}, {33, 1997, 1, 5913}, {21, 1998, 1, 5914}, {23, 1999, 3, 5914}, {24, 2000, 3, 5914}, {26, 2001, 3, 5914}, {27, 2002, 6, 5914}, {28, 2003, 3, 5914}, {30, 2004, 1, 5914}, {31, 2005, 3, 5914}, {32, 2006, 3, 5914}, {33, 2007, 1, 5914}, {21, 2008, 1, 5915}, {23, 2009, 3, 5915}, {24, 2010, 3, 5915}, {26, 2011, 3, 5915}, {27, 2012, 6, 5915}, {28, 2013, 3, 5915}, {30, 2014, 1, 5915}, {31, 2015, 3, 5915}, {32, 2016, 3, 5915}, {33, 2017, 1, 5915}, {22, 1988, 1, 5916}, {24, 1989, 3, 5916}, {25, 1990, 3, 5916}, {27, 1991, 3, 5916}, {28, 1992, 6, 5916}, {29, 1993, 3, 5916}, {31, 1994, 1, 5916}, {32, 1995, 3, 5916}, {33, 1996, 3, 5916}, {34, 1997, 1, 5916}, {22, 1998, 1, 5917}, {24, 1999, 3, 5917}, {25, 2000, 3, 5917}, {27, 2001, 3, 5917}, {28, 2002, 6, 5917}, {29, 2003, 3, 5917}, {31, 2004, 1, 5917}, {32, 2005, 3, 5917}, {33, 2006, 3, 5917}, {34, 2007, 1, 5917}, {22, 2008, 1, 5918}, {24, 2009, 3, 5918}, {25, 2010, 3, 5918}, {27, 2011, 3, 5918}, {28, 2012, 6, 5918}, {29, 2013, 3, 5918}, {31, 2014, 1, 5918}, {32, 2015, 3, 5918}, {33, 2016, 3, 5918}, {34, 2017, 1, 5918}, {20, 2073, 1, 5919}, {21, 2074, 3, 5919}, {22, 2075, 3, 5919}, {23, 2076, 3, 5919}, {24, 2077, 6, 5919}, {25, 2078, 3, 5919}, {26, 2079, 1, 5919}, {27, 2080, 3, 5919}, {28, 2081, 3, 5919}, {29, 2082, 1, 5919}, {21, 2073, 1, 5920}, {23, 2074, 3, 5920}, {24, 2075, 3, 5920}, {26, 2076, 3, 5920}, {27, 2077, 6, 5920}, {28, 2078, 3, 5920}, {30, 2079, 1, 5920}, {31, 2080, 3, 5920}, {32, 2081, 3, 5920}, {33, 2082, 1, 5920}, {22, 2073, 1, 5921}, {24, 2074, 3, 5921}, {25, 2075, 3, 5921}, {27, 2076, 3, 5921}, {28, 2077, 6, 5921}, {29, 2078, 3, 5921}, {31, 2079, 1, 5921}, {32, 2080, 3, 5921}, {33, 2081, 3, 5921}, {34, 2082, 1, 5921}, {20, 1552, 1, 5922}, {21, 1553, 4, 5922}, {22, 1554, 4, 5922}, {23, 1555, 6, 5922}, {24, 1556, 12, 5922}, {25, 1557, 6, 5922}, {26, 1558, 4, 5922}, {27, 1559, 12, 5922}, {28, 1560, 12, 5922}, {29, 1561, 4, 5922}, {30, 1562, 1, 5922}, {31, 1563, 4, 5922}, {32, 1564, 6, 5922}, {33, 1565, 4, 5922}, {34, 1566, 1, 5922}, {20, 1567, 1, 5923}, {21, 1568, 4, 5923}, {22, 1569, 4, 5923}, {23, 1570, 6, 5923}, {24, 1571, 12, 5923}, {25, 1572, 6, 5923}, {26, 1573, 4, 5923}, {27, 1574, 12, 5923}, {28, 1575, 12, 5923}, {29, 1576, 4, 5923}, {30, 1577, 1, 5923}, {31, 1578, 4, 5923}, {32, 1579, 6, 5923}, {33, 1580, 4, 5923}, {34, 1581, 1, 5923}, {20, 1568, 1, 5924}, {21, 1570, 4, 5924}, {22, 1571, 4, 5924}, {23, 1573, 6, 5924}, {24, 1574, 12, 5924}, {25, 1575, 6, 5924}, {26, 1577, 4, 5924}, {27, 1578, 12, 5924}, {28, 1579, 12, 5924}, {29, 1580, 4, 5924}, {30, 1582, 1, 5924}, {31, 1583, 4, 5924}, {32, 1584, 6, 5924}, {33, 1585, 4, 5924}, {34, 1586, 1, 5924}, {20, 1569, 1, 5925}, {21, 1571, 4, 5925}, {22, 1572, 4, 5925}, {23, 1574, 6, 5925}, {24, 1575, 12, 5925}, {25, 1576, 6, 5925}, {26, 1578, 4, 5925}, {27, 1579, 12, 5925}, {28, 1580, 12, 5925}, {29, 1581, 4, 5925}, {30, 1583, 1, 5925}, {31, 1584, 4, 5925}, {32, 1585, 6, 5925}, {33, 1586, 4, 5925}, {34, 1587, 1, 5925}, {35, 1542, 1, 5926}, {36, 1543, 3, 5926}, {37, 1544, 3, 5926}, {38, 1545, 3, 5926}, {39, 1546, 6, 5926}, {40, 1547, 3, 5926}, {41, 1548, 1, 5926}, {42, 1549, 3, 5926}, {43, 1550, 3, 5926}, {44, 1551, 1, 5926}, {36, 1542, 1, 5927}, {38, 1543, 3, 5927}, {39, 1544, 3, 5927}, {41, 1545, 3, 5927}, {42, 1546, 6, 5927}, {43, 1547, 3, 5927}, {45, 1548, 1, 5927}, {46, 1549, 3, 5927}, {47, 1550, 3, 5927}, {48, 1551, 1, 5927}, {37, 1542, 1, 5928}, {39, 1543, 3, 5928}, {40, 1544, 3, 5928}, {42, 1545, 3, 5928}, {43, 1546, 6, 5928}, {44, 1547, 3, 5928}, {46, 1548, 1, 5928}, {47, 1549, 3, 5928}, {48, 1550, 3, 5928}, {49, 1551, 1, 5928}, {38, 1542, 1, 5929}, {41, 1543, 3, 5929}, {42, 1544, 3, 5929}, {45, 1545, 3, 5929}, {46, 1546, 6, 5929}, {47, 1547, 3, 5929}, {50, 1548, 1, 5929}, {51, 1549, 3, 5929}, {52, 1550, 3, 5929}, {53, 1551, 1, 5929}, {39, 1542, 1, 5930}, {42, 1543, 3, 5930}, {43, 1544, 3, 5930}, {46, 1545, 3, 5930}, {47, 1546, 6, 5930}, {48, 1547, 3, 5930}, {51, 1548, 1, 5930}, {52, 1549, 3, 5930}, {53, 1550, 3, 5930}, {54, 1551, 1, 5930}, {40, 1542, 1, 5931}, {43, 1543, 3, 5931}, {44, 1544, 3, 5931}, {47, 1545, 3, 5931}, {48, 1546, 6, 5931}, {49, 1547, 3, 5931}, {52, 1548, 1, 5931}, {53, 1549, 3, 5931}, {54, 1550, 3, 5931}, {55, 1551, 1, 5931}, {20, 2018, 1, 5932}, {21, 2019, 4, 5932}, {22, 2020, 4, 5932}, {23, 2021, 6, 5932}, {24, 2022, 12, 5932}, {25, 2023, 6, 5932}, {26, 2024, 4, 5932}, {27, 2025, 12, 5932}, {28, 2026, 12, 5932}, {29, 2027, 4, 5932}, {30, 2028, 1, 5932}, {31, 2029, 4, 5932}, {32, 2030, 6, 5932}, {33, 2031, 4, 5932}, {34, 2032, 1, 5932}, {20, 2033, 1, 5933}, {21, 2034, 4, 5933}, {22, 2035, 4, 5933}, {23, 2036, 6, 5933}, {24, 2037, 12, 5933}, {25, 2038, 6, 5933}, {26, 2039, 4, 5933}, {27, 2040, 12, 5933}, {28, 2041, 12, 5933}, {29, 2042, 4, 5933}, {30, 2043, 1, 5933}, {31, 2044, 4, 5933}, {32, 2045, 6, 5933}, {33, 2046, 4, 5933}, {34, 2047, 1, 5933}, {20, 2048, 1, 5934}, {21, 2049, 4, 5934}, {22, 2050, 4, 5934}, {23, 2051, 6, 5934}, {24, 2052, 12, 5934}, {25, 2053, 6, 5934}, {26, 2054, 4, 5934}, {27, 2055, 12, 5934}, {28, 2056, 12, 5934}, {29, 2057, 4, 5934}, {30, 2058, 1, 5934}, {31, 2059, 4, 5934}, {32, 2060, 6, 5934}, {33, 2061, 4, 5934}, {34, 2062, 1, 5934}, {20, 2083, 1, 5935}, {21, 2084, 4, 5935}, {22, 2085, 4, 5935}, {23, 2086, 6, 5935}, {24, 2087, 12, 5935}, {25, 2088, 6, 5935}, {26, 2089, 4, 5935}, {27, 2090, 12, 5935}, {28, 2091, 12, 5935}, {29, 2092, 4, 5935}, {30, 2093, 1, 5935}, {31, 2094, 4, 5935}, {32, 2095, 6, 5935}, {33, 2096, 4, 5935}, {34, 2097, 1, 5935}, {20, 2464, 1, 5936}, {21, 2465, 4, 5936}, {22, 2466, 4, 5936}, {23, 2467, 6, 5936}, {24, 2468, 12, 5936}, {25, 2469, 6, 5936}, {26, 2470, 4, 5936}, {27, 2471, 12, 5936}, {28, 2472, 12, 5936}, {29, 2473, 4, 5936}, {30, 2474, 1, 5936}, {31, 2475, 4, 5936}, {32, 2476, 6, 5936}, {33, 2477, 4, 5936}, {34, 2478, 1, 5936}, {20, 1073, 1, 5937}, {21, 1074, 2, 5937}, {22, 1075, 2, 5937}, {23, 1079, 1, 5937}, {24, 1080, 2, 5937}, {25, 1084, 1, 5937}, {20, 1074, 1, 5938}, {21, 1076, 1, 5938}, {21, 1079, 1, 5938}, {22, 1077,

1, 5938}, {22, 1080, 1, 5938}, {23, 1081, 1, 5938}, {24, 1082, 1, 5938}, {24, 1085, 1,
5938}, {25, 1086, 1, 5938}, {20, 1075, 1, 5939}, {21, 1077, 1, 5939}, {21, 1080, 1, 5939},
{22, 1078, 1, 5939}, {22, 1084, 1, 5939}, {23, 1082, 1, 5939}, {24, 1083, 1, 5939}, {24,
1086, 1, 5939}, {25, 1087, 1, 5939}, {20, 1079, 1, 5940}, {21, 1081, 2, 5940}, {22, 1082,
2, 5940}, {23, 1088, 1, 5940}, {24, 1089, 2, 5940}, {25, 1091, 1, 5940}, {20, 1080, 1,
5941}, {21, 1082, 1, 5941}, {21, 1085, 1, 5941}, {22, 1083, 1, 5941}, {22, 1086, 1, 5941},
{23, 1089, 1, 5941}, {24, 1090, 1, 5941}, {24, 1091, 1, 5941}, {25, 1092, 1, 5941}, {20,
1084, 1, 5942}, {21, 1086, 2, 5942}, {22, 1087, 2, 5942}, {23, 1091, 1, 5942}, {24, 1092,
2, 5942}, {25, 1093, 1, 5942}, {21, 1073, 1, 5943}, {23, 1074, 2, 5943}, {24, 1075, 2,
5943}, {26, 1079, 1, 5943}, {27, 1080, 2, 5943}, {28, 1084, 1, 5943}, {21, 1074, 1, 5944},
{23, 1076, 1, 5944}, {23, 1079, 1, 5944}, {24, 1077, 1, 5944}, {24, 1080, 1, 5944}, {26,
1081, 1, 5944}, {27, 1082, 1, 5944}, {27, 1085, 1, 5944}, {28, 1086, 1, 5944}, {21, 1075,
1, 5945}, {23, 1077, 1, 5945}, {23, 1080, 1, 5945}, {24, 1078, 1, 5945}, {24, 1084, 1,
5945}, {26, 1082, 1, 5945}, {27, 1083, 1, 5945}, {27, 1086, 1, 5945}, {28, 1087, 1, 5945},
{21, 1079, 1, 5946}, {23, 1081, 2, 5946}, {24, 1082, 2, 5946}, {26, 1088, 1, 5946}, {27,
1089, 2, 5946}, {28, 1091, 1, 5946}, {21, 1080, 1, 5947}, {23, 1082, 1, 5947}, {23, 1085,
1, 5947}, {24, 1083, 1, 5947}, {24, 1086, 1, 5947}, {26, 1089, 1, 5947}, {27, 1090, 1,
5947}, {27, 1091, 1, 5947}, {28, 1092, 1, 5947}, {21, 1084, 1, 5948}, {23, 1086, 2, 5948},
{24, 1087, 2, 5948}, {26, 1091, 1, 5948}, {27, 1092, 2, 5948}, {28, 1093, 1, 5948}, {22,
1073, 1, 5949}, {24, 1074, 2, 5949}, {25, 1075, 2, 5949}, {27, 1079, 1, 5949}, {28, 1080,
2, 5949}, {29, 1084, 1, 5949}, {22, 1074, 1, 5950}, {24, 1076, 1, 5950}, {24, 1079, 1,
5950}, {25, 1077, 1, 5950}, {25, 1080, 1, 5950}, {27, 1081, 1, 5950}, {28, 1082, 1, 5950},
{28, 1085, 1, 5950}, {29, 1086, 1, 5950}, {22, 1075, 1, 5951}, {24, 1077, 1, 5951}, {24,
1080, 1, 5951}, {25, 1078, 1, 5951}, {25, 1084, 1, 5951}, {27, 1082, 1, 5951}, {28, 1083,
1, 5951}, {28, 1086, 1, 5951}, {29, 1087, 1, 5951}, {22, 1079, 1, 5952}, {24, 1081, 2,
5952}, {25, 1082, 2, 5952}, {27, 1088, 1, 5952}, {28, 1089, 2, 5952}, {29, 1091, 1, 5952},
{22, 1080, 1, 5953}, {24, 1082, 1, 5953}, {24, 1085, 1, 5953}, {25, 1083, 1, 5953}, {25,
1086, 1, 5953}, {27, 1089, 1, 5953}, {28, 1090, 1, 5953}, {28, 1091, 1, 5953}, {29, 1092,
1, 5953}, {22, 1084, 1, 5954}, {24, 1086, 2, 5954}, {25, 1087, 2, 5954}, {27, 1091, 1,
5954}, {28, 1092, 2, 5954}, {29, 1093, 1, 5954}, {23, 1073, 1, 5955}, {26, 1074, 2, 5955},
{27, 1075, 2, 5955}, {30, 1079, 1, 5955}, {31, 1080, 2, 5955}, {32, 1084, 1, 5955}, {23,
1074, 1, 5956}, {26, 1076, 1, 5956}, {26, 1079, 1, 5956}, {27, 1077, 1, 5956}, {27, 1080,
1, 5956}, {30, 1081, 1, 5956}, {31, 1082, 1, 5956}, {31, 1085, 1, 5956}, {32, 1086, 1,
5956}, {23, 1075, 1, 5957}, {26, 1077, 1, 5957}, {26, 1080, 1, 5957}, {27, 1078, 1, 5957},
{27, 1084, 1, 5957}, {30, 1082, 1, 5957}, {31, 1083, 1, 5957}, {31, 1086, 1, 5957}, {32,
1087, 1, 5957}, {23, 1079, 1, 5958}, {26, 1081, 2, 5958}, {27, 1082, 2, 5958}, {30, 1088,
1, 5958}, {31, 1089, 2, 5958}, {32, 1091, 1, 5958}, {23, 1080, 1, 5959}, {26, 1082, 1,
5959}, {26, 1085, 1, 5959}, {27, 1083, 1, 5959}, {27, 1086, 1, 5959}, {30, 1089, 1, 5959},
{31, 1090, 1, 5959}, {31, 1091, 1, 5959}, {32, 1092, 1, 5959}, {23, 1084, 1, 5960}, {26,
1086, 2, 5960}, {27, 1087, 2, 5960}, {30, 1091, 1, 5960}, {31, 1092, 2, 5960}, {32, 1093,
1, 5960}, {24, 1073, 1, 5961}, {27, 1074, 2, 5961}, {28, 1075, 2, 5961}, {31, 1079, 1,
5961}, {32, 1080, 2, 5961}, {33, 1084, 1, 5961}, {24, 1074, 1, 5962}, {27, 1076, 1, 5962},
{27, 1079, 1, 5962}, {28, 1077, 1, 5962}, {28, 1080, 1, 5962}, {31, 1081, 1, 5962}, {32,
1082, 1, 5962}, {32, 1085, 1, 5962}, {33, 1086, 1, 5962}, {24, 1075, 1, 5963}, {27, 1077,
1, 5963}, {27, 1080, 1, 5963}, {28, 1078, 1, 5963}, {28, 1084, 1, 5963}, {31, 1082, 1,
5963}, {32, 1083, 1, 5963}, {32, 1086, 1, 5963}, {33, 1087, 1, 5963}, {24, 1079, 1, 5964},
{27, 1081, 2, 5964}, {28, 1082, 2, 5964}, {31, 1088, 1, 5964}, {32, 1089, 2, 5964}, {33,
1091, 1, 5964}, {24, 1080, 1, 5965}, {27, 1082, 1, 5965}, {27, 1085, 1, 5965}, {28, 1083,
1, 5965}, {28, 1086, 1, 5965}, {31, 1089, 1, 5965}, {32, 1090, 1, 5965}, {32, 1091, 1,
5965}, {33, 1092, 1, 5965}, {24, 1084, 1, 5966}, {27, 1086, 2, 5966}, {28, 1087, 2, 5966},
{31, 1091, 1, 5966}, {32, 1092, 2, 5966}, {33, 1093, 1, 5966}, {25, 1073, 1, 5967}, {28,
1074, 2, 5967}, {29, 1075, 2, 5967}, {32, 1079, 1, 5967}, {33, 1080, 2, 5967}, {34, 1084,
1, 5967}, {25, 1074, 1, 5968}, {28, 1076, 1, 5968}, {28, 1079, 1, 5968}, {29, 1077, 1,
5968}, {29, 1080, 1, 5968}, {32, 1081, 1, 5968}, {33, 1082, 1, 5968}, {33, 1085, 1, 5968},
{34, 1086, 1, 5968}, {25, 1075, 1, 5969}, {28, 1077, 1, 5969}, {28, 1080, 1, 5969}, {29,
1078, 1, 5969}, {29, 1084, 1, 5969}, {32, 1082, 1, 5969}, {33, 1083, 1, 5969}, {33, 1086,
1, 5969}, {34, 1087, 1, 5969}, {25, 1079, 1, 5970}, {28, 1081, 2, 5970}, {29, 1082, 2,
5970}, {32, 1088, 1, 5970}, {33, 1089, 2, 5970}, {34, 1091, 1, 5970}, {25, 1080, 1, 5971},
{28, 1082, 1, 5971}, {28, 1085, 1, 5971}, {29, 1083, 1, 5971}, {29, 1086, 1, 5971}, {32,
1089, 1, 5971}, {33, 1090, 1, 5971}, {33, 1091, 1, 5971}, {34, 1092, 1, 5971}, {25, 1084,
1, 5972}, {28, 1086, 2, 5972}, {29, 1087, 2, 5972}, {32, 1091, 1, 5972}, {33, 1092, 2,
5972}, {34, 1093, 1, 5972}, {20, 1370, 1, 5973}, {21, 1371, 2, 5973}, {22, 1372, 2, 5973},
{23, 1373, 1, 5973}, {24, 1374, 2, 5973}, {25, 1375, 1, 5973}, {21, 1370, 1, 5974}, {23,
1371, 2, 5974}, {24, 1372, 2, 5974}, {26, 1373, 1, 5974}, {27, 1374, 2, 5974}, {28, 1375,
1, 5974}, {22, 1370, 1, 5975}, {24, 1371, 2, 5975}, {25, 1372, 2, 5975}, {27, 1373, 1,
5975}, {28, 1374, 2, 5975}, {29, 1375, 1, 5975}, {23, 1370, 1, 5976}, {26, 1371, 2, 5976},
{27, 1372, 2, 5976}, {30, 1373, 1, 5976}, {31, 1374, 2, 5976}, {32, 1375, 1, 5976}, {24,
1370, 1, 5977}, {27, 1371, 2, 5977}, {28, 1372, 2, 5977}, {31, 1373, 1, 5977}, {32, 1374,
2, 5977}, {33, 1375, 1, 5977}, {25, 1370, 1, 5978}, {28, 1371, 2, 5978}, {29, 1372, 2,

5978}, {32, 1373, 1, 5978}, {33, 1374, 2, 5978}, {34, 1375, 1, 5978}, {20, 1376, 1, 5979}, {21, 1377, 1, 5979}, {21, 1382, 1, 5979}, {22, 1378, 1, 5979}, {22, 1388, 1, 5979}, {23, 1383, 1, 5979}, {24, 1384, 1, 5979}, {24, 1389, 1, 5979}, {25, 1390, 1, 5979}, {20, 1377, 1, 5980}, {21, 1379, 1, 5980}, {21, 1383, 1, 5980}, {22, 1380, 1, 5980}, {22, 1389, 1, 5980}, {23, 1385, 1, 5980}, {24, 1386, 1, 5980}, {24, 1391, 1, 5980}, {25, 1392, 1, 5980}, {20, 1378, 1, 5981}, {21, 1380, 1, 5981}, {21, 1384, 1, 5981}, {22, 1381, 1, 5981}, {22, 1390, 1, 5981}, {23, 1386, 1, 5981}, {24, 1387, 1, 5981}, {24, 1392, 1, 5981}, {25, 1393, 1, 5981}, {21, 1376, 1, 5982}, {23, 1377, 1, 5982}, {23, 1382, 1, 5982}, {24, 1378, 1, 5982}, {24, 1388, 1, 5982}, {26, 1383, 1, 5982}, {27, 1384, 1, 5982}, {27, 1389, 1, 5982}, {28, 1390, 1, 5982}, {21, 1377, 1, 5983}, {23, 1379, 1, 5983}, {23, 1383, 1, 5983}, {24, 1380, 1, 5983}, {24, 1389, 1, 5983}, {26, 1385, 1, 5983}, {27, 1386, 1, 5983}, {27, 1391, 1, 5983}, {28, 1392, 1, 5983}, {21, 1378, 1, 5984}, {23, 1380, 1, 5984}, {23, 1384, 1, 5984}, {24, 1381, 1, 5984}, {24, 1390, 1, 5984}, {26, 1386, 1, 5984}, {27, 1387, 1, 5984}, {27, 1392, 1, 5984}, {28, 1393, 1, 5984}, {22, 1376, 1, 5985}, {24, 1377, 1, 5985}, {24, 1382, 1, 5985}, {25, 1378, 1, 5985}, {25, 1388, 1, 5985}, {27, 1383, 1, 5985}, {28, 1384, 1, 5985}, {28, 1389, 1, 5985}, {29, 1390, 1, 5985}, {22, 1377, 1, 5986}, {24, 1379, 1, 5986}, {24, 1383, 1, 5986}, {25, 1380, 1, 5986}, {25, 1389, 1, 5986}, {27, 1385, 1, 5986}, {28, 1386, 1, 5986}, {28, 1391, 1, 5986}, {29, 1392, 1, 5986}, {22, 1378, 1, 5987}, {24, 1380, 1, 5987}, {24, 1384, 1, 5987}, {25, 1381, 1, 5987}, {25, 1390, 1, 5987}, {27, 1386, 1, 5987}, {28, 1387, 1, 5987}, {28, 1392, 1, 5987}, {29, 1393, 1, 5987}, {23, 1376, 1, 5988}, {26, 1377, 1, 5988}, {26, 1382, 1, 5988}, {27, 1378, 1, 5988}, {27, 1388, 1, 5988}, {30, 1383, 1, 5988}, {31, 1384, 1, 5988}, {31, 1389, 1, 5988}, {32, 1390, 1, 5988}, {23, 1377, 1, 5989}, {26, 1379, 1, 5989}, {26, 1383, 1, 5989}, {27, 1380, 1, 5989}, {27, 1389, 1, 5989}, {30, 1385, 1, 5989}, {31, 1386, 1, 5989}, {31, 1391, 1, 5989}, {32, 1392, 1, 5989}, {23, 1378, 1, 5990}, {26, 1380, 1, 5990}, {26, 1384, 1, 5990}, {27, 1381, 1, 5990}, {27, 1390, 1, 5990}, {30, 1386, 1, 5990}, {31, 1387, 1, 5990}, {31, 1392, 1, 5990}, {32, 1393, 1, 5990}, {24, 1376, 1, 5991}, {27, 1377, 1, 5991}, {27, 1382, 1, 5991}, {28, 1378, 1, 5991}, {28, 1388, 1, 5991}, {31, 1383, 1, 5991}, {32, 1384, 1, 5991}, {32, 1389, 1, 5991}, {33, 1390, 1, 5991}, {24, 1377, 1, 5992}, {27, 1379, 1, 5992}, {27, 1383, 1, 5992}, {28, 1380, 1, 5992}, {28, 1389, 1, 5992}, {31, 1385, 1, 5992}, {32, 1386, 1, 5992}, {32, 1391, 1, 5992}, {33, 1392, 1, 5992}, {24, 1378, 1, 5993}, {27, 1380, 1, 5993}, {27, 1384, 1, 5993}, {28, 1381, 1, 5993}, {28, 1390, 1, 5993}, {31, 1386, 1, 5993}, {32, 1387, 1, 5993}, {32, 1392, 1, 5993}, {33, 1393, 1, 5993}, {25, 1376, 1, 5994}, {28, 1377, 1, 5994}, {28, 1382, 1, 5994}, {29, 1378, 1, 5994}, {29, 1388, 1, 5994}, {32, 1383, 1, 5994}, {33, 1384, 1, 5994}, {33, 1389, 1, 5994}, {34, 1390, 1, 5994}, {25, 1377, 1, 5995}, {28, 1379, 1, 5995}, {28, 1383, 1, 5995}, {29, 1380, 1, 5995}, {29, 1389, 1, 5995}, {32, 1385, 1, 5995}, {33, 1386, 1, 5995}, {33, 1391, 1, 5995}, {34, 1392, 1, 5995}, {25, 1378, 1, 5996}, {28, 1380, 1, 5996}, {28, 1384, 1, 5996}, {29, 1381, 1, 5996}, {29, 1390, 1, 5996}, {32, 1386, 1, 5996}, {33, 1387, 1, 5996}, {33, 1392, 1, 5996}, {34, 1393, 1, 5996}, {20, 1793, 1, 5997}, {21, 1794, 2, 5997}, {22, 1795, 2, 5997}, {23, 1799, 1, 5997}, {24, 1800, 2, 5997}, {25, 1804, 1, 5997}, {20, 1794, 1, 5998}, {21, 1796, 1, 5998}, {21, 1799, 1, 5998}, {22, 1797, 1, 5998}, {22, 1800, 1, 5998}, {23, 1801, 1, 5998}, {24, 1802, 1, 5998}, {24, 1805, 1, 5998}, {25, 1806, 1, 5998}, {20, 1795, 1, 5999}, {21, 1797, 1, 5999}, {21, 1800, 1, 5999}, {22, 1798, 1, 5999}, {22, 1804, 1, 5999}, {23, 1802, 1, 5999}, {24, 1803, 1, 5999}, {24, 1806, 1, 5999}, {25, 1807, 1, 5999}, {20, 1799, 1, 6000}, {21, 1801, 2, 6000}, {22, 1802, 2, 6000}, {23, 1808, 1, 6000}, {24, 1809, 2, 6000}, {25, 1811, 1, 6000}, {20, 1800, 1, 6001}, {21, 1802, 1, 6001}, {21, 1805, 1, 6001}, {22, 1803, 1, 6001}, {22, 1806, 1, 6001}, {23, 1809, 1, 6001}, {24, 1810, 1, 6001}, {24, 1811, 1, 6001}, {25, 1812, 1, 6001}, {20, 1804, 1, 6002}, {21, 1806, 2, 6002}, {22, 1807, 2, 6002}, {23, 1811, 1, 6002}, {24, 1812, 2, 6002}, {25, 1813, 1, 6002}, {21, 1793, 1, 6003}, {23, 1794, 2, 6003}, {24, 1795, 2, 6003}, {26, 1799, 1, 6003}, {27, 1800, 2, 6003}, {28, 1804, 1, 6003}, {21, 1794, 1, 6004}, {23, 1796, 1, 6004}, {23, 1799, 1, 6004}, {24, 1797, 1, 6004}, {24, 1800, 1, 6004}, {26, 1801, 1, 6004}, {27, 1802, 1, 6004}, {27, 1805, 1, 6004}, {28, 1806, 1, 6004}, {21, 1795, 1, 6005}, {23, 1797, 1, 6005}, {23, 1800, 1, 6005}, {24, 1798, 1, 6005}, {24, 1804, 1, 6005}, {26, 1802, 1, 6005}, {27, 1803, 1, 6005}, {27, 1806, 1, 6005}, {28, 1807, 1, 6005}, {21, 1799, 1, 6006}, {23, 1801, 2, 6006}, {24, 1802, 2, 6006}, {26, 1808, 1, 6006}, {27, 1809, 2, 6006}, {28, 1811, 1, 6006}, {21, 1800, 1, 6007}, {23, 1802, 1, 6007}, {23, 1805, 1, 6007}, {24, 1803, 1, 6007}, {24, 1806, 1, 6007}, {26, 1809, 1, 6007}, {27, 1810, 1, 6007}, {27, 1811, 1, 6007}, {28, 1812, 1, 6007}, {21, 1804, 1, 6008}, {23, 1806, 2, 6008}, {24, 1807, 2, 6008}, {26, 1811, 1, 6008}, {27, 1812, 2, 6008}, {28, 1813, 1, 6008}, {22, 1793, 1, 6009}, {24, 1794, 2, 6009}, {25, 1795, 2, 6009}, {27, 1799, 1, 6009}, {28, 1800, 2, 6009}, {29, 1804, 1, 6009}, {22, 1794, 1, 6010}, {24, 1796, 1, 6010}, {24, 1799, 1, 6010}, {25, 1797, 1, 6010}, {25, 1800, 1, 6010}, {27, 1801, 1, 6010}, {28, 1802, 1, 6010}, {28, 1805, 1, 6010}, {29, 1806, 1, 6010}, {22, 1795, 1, 6011}, {24, 1797, 1, 6011}, {24, 1800, 1, 6011}, {25, 1798, 1, 6011}, {25, 1804, 1, 6011}, {27, 1802, 1, 6011}, {28, 1803, 1, 6011}, {28, 1806, 1, 6011}, {29, 1807, 1, 6011}, {22, 1799, 1, 6012}, {24, 1801, 2, 6012}, {25, 1802, 2, 6012}, {27, 1808, 1, 6012}, {28, 1809, 2, 6012}, {29, 1811, 1, 6012}, {22, 1800, 1, 6013}, {24, 1802, 1, 6013}, {24, 1805, 1, 6013}, {25, 1803, 1, 6013}, {25, 1806, 1, 6013}, {27, 1809, 1, 6013}, {28, 1810, 1, 6013}, {28, 1811, 1, 6013},

{29, 1812, 1, 6013}, {22, 1804, 1, 6014}, {24, 1806, 2, 6014}, {25, 1807, 2, 6014}, {27, 1811, 1, 6014}, {28, 1812, 2, 6014}, {29, 1813, 1, 6014}, {23, 1793, 1, 6015}, {26, 1794, 2, 6015}, {27, 1795, 2, 6015}, {30, 1799, 1, 6015}, {31, 1800, 2, 6015}, {32, 1804, 1, 6015}, {23, 1794, 1, 6016}, {26, 1796, 1, 6016}, {26, 1799, 1, 6016}, {27, 1797, 1, 6016}, {27, 1800, 1, 6016}, {30, 1801, 1, 6016}, {31, 1802, 1, 6016}, {31, 1805, 1, 6016}, {32, 1806, 1, 6016}, {23, 1795, 1, 6017}, {26, 1797, 1, 6017}, {26, 1800, 1, 6017}, {27, 1798, 1, 6017}, {27, 1804, 1, 6017}, {30, 1802, 1, 6017}, {31, 1803, 1, 6017}, {31, 1806, 1, 6017}, {32, 1807, 1, 6017}, {23, 1799, 1, 6018}, {26, 1801, 2, 6018}, {27, 1802, 2, 6018}, {30, 1808, 1, 6018}, {31, 1809, 2, 6018}, {32, 1811, 1, 6018}, {23, 1800, 1, 6019}, {26, 1802, 1, 6019}, {26, 1805, 1, 6019}, {27, 1803, 1, 6019}, {27, 1806, 1, 6019}, {30, 1809, 1, 6019}, {31, 1810, 1, 6019}, {31, 1811, 1, 6019}, {32, 1812, 1, 6019}, {23, 1804, 1, 6020}, {26, 1806, 2, 6020}, {27, 1807, 2, 6020}, {30, 1811, 1, 6020}, {31, 1812, 2, 6020}, {32, 1813, 1, 6020}, {24, 1793, 1, 6021}, {27, 1794, 2, 6021}, {28, 1795, 2, 6021}, {31, 1799, 1, 6021}, {32, 1800, 2, 6021}, {33, 1804, 1, 6021}, {24, 1794, 1, 6022}, {27, 1796, 1, 6022}, {27, 1799, 1, 6022}, {28, 1797, 1, 6022}, {28, 1800, 1, 6022}, {31, 1801, 1, 6022}, {32, 1802, 1, 6022}, {32, 1805, 1, 6022}, {33, 1806, 1, 6022}, {24, 1795, 1, 6023}, {27, 1797, 1, 6023}, {27, 1800, 1, 6023}, {28, 1798, 1, 6023}, {28, 1804, 1, 6023}, {31, 1802, 1, 6023}, {32, 1803, 1, 6023}, {32, 1806, 1, 6023}, {33, 1807, 1, 6023}, {24, 1799, 1, 6024}, {27, 1801, 2, 6024}, {28, 1802, 2, 6024}, {31, 1808, 1, 6024}, {32, 1809, 2, 6024}, {33, 1811, 1, 6024}, {24, 1800, 1, 6025}, {27, 1802, 1, 6025}, {27, 1805, 1, 6025}, {28, 1803, 1, 6025}, {28, 1806, 1, 6025}, {31, 1809, 1, 6025}, {32, 1810, 1, 6025}, {32, 1811, 1, 6025}, {33, 1812, 1, 6025}, {24, 1804, 1, 6026}, {27, 1806, 2, 6026}, {28, 1807, 2, 6026}, {31, 1811, 1, 6026}, {32, 1812, 2, 6026}, {33, 1813, 1, 6026}, {25, 1793, 1, 6027}, {28, 1794, 2, 6027}, {29, 1795, 2, 6027}, {32, 1799, 1, 6027}, {33, 1800, 2, 6027}, {34, 1804, 1, 6027}, {25, 1794, 1, 6028}, {28, 1796, 1, 6028}, {28, 1799, 1, 6028}, {29, 1797, 1, 6028}, {29, 1800, 1, 6028}, {32, 1801, 1, 6028}, {33, 1802, 1, 6028}, {33, 1805, 1, 6028}, {34, 1806, 1, 6028}, {25, 1795, 1, 6029}, {28, 1797, 1, 6029}, {28, 1800, 1, 6029}, {29, 1798, 1, 6029}, {29, 1804, 1, 6029}, {32, 1802, 1, 6029}, {33, 1803, 1, 6029}, {33, 1806, 1, 6029}, {34, 1807, 1, 6029}, {25, 1799, 1, 6030}, {28, 1801, 2, 6030}, {29, 1802, 2, 6030}, {32, 1808, 1, 6030}, {33, 1809, 2, 6030}, {34, 1811, 1, 6030}, {25, 1800, 1, 6031}, {28, 1802, 1, 6031}, {28, 1805, 1, 6031}, {29, 1803, 1, 6031}, {29, 1806, 1, 6031}, {32, 1809, 1, 6031}, {33, 1810, 1, 6031}, {33, 1811, 1, 6031}, {34, 1812, 1, 6031}, {25, 1804, 1, 6032}, {28, 1806, 2, 6032}, {29, 1807, 2, 6032}, {32, 1811, 1, 6032}, {33, 1812, 2, 6032}, {34, 1813, 1, 6032}, {20, 1964, 1, 6033}, {21, 1965, 2, 6033}, {22, 1966, 2, 6033}, {23, 1967, 1, 6033}, {24, 1968, 2, 6033}, {25, 1969, 1, 6033}, {21, 1964, 1, 6034}, {23, 1965, 2, 6034}, {24, 1966, 2, 6034}, {26, 1967, 1, 6034}, {27, 1968, 2, 6034}, {28, 1969, 1, 6034}, {22, 1964, 1, 6035}, {24, 1965, 2, 6035}, {25, 1966, 2, 6035}, {27, 1967, 1, 6035}, {28, 1968, 2, 6035}, {29, 1969, 1, 6035}, {23, 1964, 1, 6036}, {26, 1965, 2, 6036}, {27, 1966, 2, 6036}, {30, 1967, 1, 6036}, {31, 1968, 2, 6036}, {32, 1969, 1, 6036}, {24, 1964, 1, 6037}, {27, 1965, 2, 6037}, {28, 1966, 2, 6037}, {31, 1967, 1, 6037}, {32, 1968, 2, 6037}, {33, 1969, 1, 6037}, {25, 1964, 1, 6038}, {28, 1965, 2, 6038}, {29, 1966, 2, 6038}, {32, 1967, 1, 6038}, {33, 1968, 2, 6038}, {34, 1969, 1, 6038}, {20, 1970, 1, 6039}, {21, 1971, 1, 6039}, {21, 1976, 1, 6039}, {22, 1972, 1, 6039}, {22, 1982, 1, 6039}, {23, 1977, 1, 6039}, {24, 1978, 1, 6039}, {24, 1983, 1, 6039}, {25, 1984, 1, 6039}, {20, 1971, 1, 6040}, {21, 1973, 1, 6040}, {21, 1977, 1, 6040}, {22, 1974, 1, 6040}, {22, 1983, 1, 6040}, {23, 1979, 1, 6040}, {24, 1980, 1, 6040}, {24, 1985, 1, 6040}, {25, 1986, 1, 6040}, {20, 1972, 1, 6041}, {21, 1974, 1, 6041}, {21, 1978, 1, 6041}, {22, 1975, 1, 6041}, {22, 1984, 1, 6041}, {23, 1980, 1, 6041}, {24, 1981, 1, 6041}, {24, 1986, 1, 6041}, {25, 1987, 1, 6041}, {21, 1970, 1, 6042}, {23, 1971, 1, 6042}, {23, 1976, 1, 6042}, {24, 1972, 1, 6042}, {24, 1982, 1, 6042}, {26, 1977, 1, 6042}, {27, 1978, 1, 6042}, {27, 1983, 1, 6042}, {28, 1984, 1, 6042}, {21, 1971, 1, 6043}, {23, 1973, 1, 6043}, {23, 1977, 1, 6043}, {24, 1974, 1, 6043}, {24, 1983, 1, 6043}, {26, 1979, 1, 6043}, {27, 1980, 1, 6043}, {27, 1985, 1, 6043}, {28, 1986, 1, 6043}, {21, 1972, 1, 6044}, {23, 1974, 1, 6044}, {23, 1978, 1, 6044}, {24, 1975, 1, 6044}, {24, 1984, 1, 6044}, {26, 1980, 1, 6044}, {27, 1981, 1, 6044}, {27, 1986, 1, 6044}, {28, 1987, 1, 6044}, {22, 1970, 1, 6045}, {24, 1971, 1, 6045}, {24, 1976, 1, 6045}, {25, 1972, 1, 6045}, {25, 1982, 1, 6045}, {27, 1977, 1, 6045}, {28, 1978, 1, 6045}, {28, 1983, 1, 6045}, {29, 1984, 1, 6045}, {22, 1971, 1, 6046}, {24, 1973, 1, 6046}, {24, 1977, 1, 6046}, {25, 1974, 1, 6046}, {25, 1983, 1, 6046}, {27, 1979, 1, 6046}, {28, 1980, 1, 6046}, {28, 1985, 1, 6046}, {29, 1986, 1, 6046}, {22, 1972, 1, 6047}, {24, 1974, 1, 6047}, {24, 1978, 1, 6047}, {25, 1975, 1, 6047}, {25, 1984, 1, 6047}, {27, 1980, 1, 6047}, {28, 1981, 1, 6047}, {28, 1986, 1, 6047}, {29, 1987, 1, 6047}, {23, 1970, 1, 6048}, {26, 1971, 1, 6048}, {26, 1976, 1, 6048}, {27, 1972, 1, 6048}, {27, 1982, 1, 6048}, {30, 1977, 1, 6048}, {31, 1978, 1, 6048}, {31, 1983, 1, 6048}, {32, 1984, 1, 6048}, {23, 1971, 1, 6049}, {26, 1973, 1, 6049}, {26, 1977, 1, 6049}, {27, 1974, 1, 6049}, {27, 1983, 1, 6049}, {30, 1979, 1, 6049}, {31, 1980, 1, 6049}, {31, 1985, 1, 6049}, {32, 1986, 1, 6049}, {23, 1972, 1, 6050}, {26, 1974, 1, 6050}, {26, 1978, 1, 6050}, {27, 1975, 1, 6050}, {27, 1984, 1, 6050}, {30, 1980, 1, 6050}, {31, 1981, 1, 6050}, {31, 1986, 1, 6050}, {32, 1987, 1, 6050}, {24, 1970, 1, 6051}, {27, 1971, 1, 6051}, {27, 1976, 1, 6051}, {28, 1972, 1, 6051}, {28, 1982, 1, 6051}, {31, 1977, 1, 6051}, {32, 1978, 1, 6051}, {32,

1983, 1, 6051}, {33, 1984, 1, 6051}, {24, 1971, 1, 6052}, {27, 1973, 1, 6052}, {27, 1977, 1, 6052}, {28, 1974, 1, 6052}, {28, 1983, 1, 6052}, {31, 1979, 1, 6052}, {32, 1980, 1, 6052}, {32, 1985, 1, 6052}, {33, 1986, 1, 6052}, {24, 1972, 1, 6053}, {27, 1974, 1, 6053}, {27, 1978, 1, 6053}, {28, 1975, 1, 6053}, {28, 1984, 1, 6053}, {31, 1980, 1, 6053}, {32, 1981, 1, 6053}, {32, 1986, 1, 6053}, {33, 1987, 1, 6053}, {25, 1970, 1, 6054}, {28, 1971, 1, 6054}, {28, 1976, 1, 6054}, {29, 1972, 1, 6054}, {29, 1982, 1, 6054}, {32, 1977, 1, 6054}, {33, 1978, 1, 6054}, {33, 1983, 1, 6054}, {34, 1984, 1, 6054}, {25, 1971, 1, 6055}, {28, 1973, 1, 6055}, {28, 1977, 1, 6055}, {29, 1974, 1, 6055}, {29, 1983, 1, 6055}, {32, 1979, 1, 6055}, {33, 1980, 1, 6055}, {33, 1985, 1, 6055}, {34, 1986, 1, 6055}, {25, 1972, 1, 6056}, {28, 1974, 1, 6056}, {28, 1978, 1, 6056}, {29, 1975, 1, 6056}, {29, 1984, 1, 6056}, {32, 1980, 1, 6056}, {33, 1981, 1, 6056}, {33, 1986, 1, 6056}, {34, 1987, 1, 6056}, {20, 2345, 1, 6057}, {21, 2346, 2, 6057}, {22, 2347, 2, 6057}, {23, 2348, 1, 6057}, {24, 2349, 2, 6057}, {25, 2350, 1, 6057}, {21, 2345, 1, 6058}, {23, 2346, 2, 6058}, {24, 2347, 2, 6058}, {26, 2348, 1, 6058}, {27, 2349, 2, 6058}, {28, 2350, 1, 6058}, {22, 2345, 1, 6059}, {24, 2346, 2, 6059}, {25, 2347, 2, 6059}, {27, 2348, 1, 6059}, {28, 2349, 2, 6059}, {29, 2350, 1, 6059}, {23, 2345, 1, 6060}, {26, 2346, 2, 6060}, {27, 2347, 2, 6060}, {30, 2348, 1, 6060}, {31, 2349, 2, 6060}, {32, 2350, 1, 6060}, {24, 2345, 1, 6061}, {27, 2346, 2, 6061}, {28, 2347, 2, 6061}, {31, 2348, 1, 6061}, {32, 2349, 2, 6061}, {33, 2350, 1, 6061}, {25, 2345, 1, 6062}, {28, 2346, 2, 6062}, {29, 2347, 2, 6062}, {32, 2348, 1, 6062}, {33, 2349, 2, 6062}, {34, 2350, 1, 6062}, {20, 1073, 1, 6063}, {21, 1074, 3, 6063}, {22, 1075, 3, 6063}, {23, 1076, 1, 6063}, {23, 1079, 2, 6063}, {24, 1077, 2, 6063}, {24, 1080, 4, 6063}, {25, 1078, 1, 6063}, {25, 1084, 2, 6063}, {26, 1081, 1, 6063}, {27, 1082, 2, 6063}, {27, 1085, 1, 6063}, {28, 1083, 1, 6063}, {28, 1086, 2, 6063}, {29, 1087, 1, 6063}, {20, 1074, 1, 6064}, {21, 1076, 1, 6064}, {21, 1079, 2, 6064}, {22, 1077, 1, 6064}, {22, 1080, 2, 6064}, {23, 1081, 3, 6064}, {24, 1082, 4, 6064}, {24, 1085, 2, 6064}, {25, 1083, 1, 6064}, {25, 1086, 2, 6064}, {26, 1088, 1, 6064}, {27, 1089, 3, 6064}, {28, 1090, 1, 6064}, {28, 1091, 2, 6064}, {29, 1092, 1, 6064}, {20, 1075, 1, 6065}, {21, 1077, 1, 6065}, {21, 1080, 2, 6065}, {22, 1078, 1, 6065}, {22, 1084, 2, 6065}, {23, 1082, 2, 6065}, {23, 1085, 1, 6065}, {24, 1083, 2, 6065}, {24, 1086, 4, 6065}, {25, 1087, 3, 6065}, {26, 1089, 1, 6065}, {27, 1090, 1, 6065}, {27, 1091, 2, 6065}, {28, 1092, 3, 6065}, {29, 1093, 1, 6065}, {21, 1073, 1, 6066}, {23, 1074, 3, 6066}, {24, 1075, 3, 6066}, {26, 1076, 1, 6066}, {26, 1079, 2, 6066}, {27, 1077, 2, 6066}, {27, 1080, 4, 6066}, {28, 1078, 1, 6066}, {28, 1084, 2, 6066}, {30, 1081, 1, 6066}, {31, 1082, 2, 6066}, {31, 1085, 1, 6066}, {32, 1083, 1, 6066}, {32, 1086, 2, 6066}, {33, 1087, 1, 6066}, {21, 1074, 1, 6067}, {23, 1076, 1, 6067}, {23, 1079, 2, 6067}, {24, 1077, 1, 6067}, {24, 1080, 2, 6067}, {26, 1081, 3, 6067}, {27, 1082, 4, 6067}, {27, 1085, 2, 6067}, {28, 1083, 1, 6067}, {28, 1086, 2, 6067}, {30, 1088, 1, 6067}, {31, 1089, 3, 6067}, {32, 1090, 1, 6067}, {32, 1091, 2, 6067}, {33, 1092, 1, 6067}, {21, 1075, 1, 6068}, {23, 1077, 1, 6068}, {23, 1080, 2, 6068}, {24, 1078, 1, 6068}, {24, 1084, 2, 6068}, {26, 1082, 2, 6068}, {26, 1085, 1, 6068}, {27, 1083, 2, 6068}, {27, 1086, 4, 6068}, {28, 1087, 3, 6068}, {30, 1089, 1, 6068}, {31, 1090, 1, 6068}, {31, 1091, 2, 6068}, {32, 1092, 3, 6068}, {33, 1093, 1, 6068}, {22, 1073, 1, 6069}, {24, 1074, 3, 6069}, {25, 1075, 3, 6069}, {27, 1076, 1, 6069}, {27, 1079, 2, 6069}, {28, 1077, 2, 6069}, {28, 1080, 4, 6069}, {29, 1078, 1, 6069}, {29, 1084, 2, 6069}, {31, 1081, 1, 6069}, {32, 1082, 2, 6069}, {32, 1085, 1, 6069}, {33, 1083, 1, 6069}, {33, 1086, 2, 6069}, {34, 1087, 1, 6069}, {22, 1074, 1, 6070}, {24, 1076, 1, 6070}, {24, 1079, 2, 6070}, {25, 1077, 1, 6070}, {25, 1080, 2, 6070}, {27, 1081, 3, 6070}, {28, 1082, 4, 6070}, {28, 1085, 2, 6070}, {29, 1083, 1, 6070}, {29, 1086, 2, 6070}, {31, 1088, 1, 6070}, {32, 1089, 3, 6070}, {33, 1090, 1, 6070}, {33, 1091, 2, 6070}, {34, 1092, 1, 6070}, {22, 1075, 1, 6071}, {24, 1077, 1, 6071}, {24, 1080, 2, 6071}, {25, 1078, 1, 6071}, {25, 1084, 2, 6071}, {27, 1082, 2, 6071}, {27, 1085, 1, 6071}, {28, 1083, 2, 6071}, {28, 1086, 4, 6071}, {29, 1087, 3, 6071}, {31, 1089, 1, 6071}, {32, 1090, 1, 6071}, {32, 1091, 2, 6071}, {33, 1092, 3, 6071}, {34, 1093, 1, 6071}, {20, 1094, 1, 6072}, {21, 1095, 2, 6072}, {21, 1104, 1, 6072}, {22, 1096, 2, 6072}, {22, 1114, 1, 6072}, {23, 1097, 1, 6072}, {23, 1105, 2, 6072}, {24, 1098, 2, 6072}, {24, 1106, 2, 6072}, {24, 1115, 2, 6072}, {25, 1099, 1, 6072}, {25, 1116, 2, 6072}, {26, 1107, 1, 6072}, {27, 1108, 2, 6072}, {27, 1117, 1, 6072}, {28, 1109, 1, 6072}, {28, 1118, 2, 6072}, {29, 1119, 1, 6072}, {20, 1095, 1, 6073}, {21, 1097, 2, 6073}, {21, 1105, 1, 6073}, {22, 1098, 2, 6073}, {22, 1115, 1, 6073}, {23, 1100, 1, 6073}, {23, 1107, 2, 6073}, {24, 1101, 2, 6073}, {24, 1108, 2, 6073}, {24, 1117, 2, 6073}, {25, 1102, 1, 6073}, {25, 1118, 2, 6073}, {26, 1110, 1, 6073}, {27, 1111, 2, 6073}, {27, 1120, 1, 6073}, {28, 1112, 1, 6073}, {28, 1121, 2, 6073}, {29, 1122, 1, 6073}, {20, 1096, 1, 6074}, {21, 1098, 2, 6074}, {21, 1106, 1, 6074}, {22, 1099, 2, 6074}, {22, 1116, 1, 6074}, {23, 1101, 1, 6074}, {23, 1108, 2, 6074}, {24, 1102, 2, 6074}, {24, 1109, 2, 6074}, {24, 1118, 2, 6074}, {25, 1103, 1, 6074}, {25, 1119, 2, 6074}, {26, 1111, 1, 6074}, {27, 1112, 2, 6074}, {27, 1121, 1, 6074}, {28, 1113, 1, 6074}, {28, 1122, 2, 6074}, {29, 1123, 1, 6074}, {20, 1104, 1, 6075}, {21, 1105, 2, 6075}, {21, 1124, 1, 6075}, {22, 1106, 2, 6075}, {22, 1134, 1, 6075}, {23, 1107, 1, 6075}, {23, 1125, 2, 6075}, {24, 1108, 2, 6075}, {24, 1126, 2, 6075}, {24, 1135, 2, 6075}, {25, 1109, 1, 6075}, {25, 1136, 2, 6075}, {26, 1127, 1, 6075}, {27, 1128, 2, 6075}, {27, 1137, 1, 6075}, {28, 1129, 1, 6075}, {28, 1138, 2, 6075}, {29, 1139, 1, 6075}, {20, 1105, 1, 6076}, {21, 1107, 2, 6076}, {21, 1125, 1, 6076}, {22, 1108,

2, 6076}, {22, 1135, 1, 6076}, {23, 1110, 1, 6076}, {23, 1127, 2, 6076}, {24, 1111, 2, 6076}, {24, 1128, 2, 6076}, {24, 1137, 2, 6076}, {25, 1112, 1, 6076}, {25, 1138, 2, 6076}, {26, 1130, 1, 6076}, {27, 1131, 2, 6076}, {27, 1140, 1, 6076}, {28, 1132, 1, 6076}, {28, 1141, 2, 6076}, {29, 1142, 1, 6076}, {20, 1106, 1, 6077}, {21, 1108, 2, 6077}, {21, 1126, 1, 6077}, {22, 1109, 2, 6077}, {22, 1136, 1, 6077}, {23, 1111, 1, 6077}, {23, 1128, 2, 6077}, {24, 1112, 2, 6077}, {24, 1129, 2, 6077}, {24, 1138, 2, 6077}, {25, 1113, 1, 6077}, {25, 1139, 2, 6077}, {26, 1131, 1, 6077}, {27, 1132, 2, 6077}, {27, 1141, 1, 6077}, {28, 1133, 1, 6077}, {28, 1142, 2, 6077}, {29, 1143, 1, 6077}, {20, 1114, 1, 6078}, {21, 1115, 2, 6078}, {21, 1134, 1, 6078}, {22, 1116, 2, 6078}, {22, 1144, 1, 6078}, {23, 1117, 1, 6078}, {23, 1135, 2, 6078}, {24, 1118, 2, 6078}, {24, 1136, 2, 6078}, {24, 1145, 2, 6078}, {25, 1119, 1, 6078}, {25, 1146, 2, 6078}, {26, 1137, 1, 6078}, {27, 1138, 2, 6078}, {27, 1147, 1, 6078}, {28, 1139, 1, 6078}, {28, 1148, 2, 6078}, {29, 1149, 1, 6078}, {20, 1115, 1, 6079}, {21, 1117, 2, 6079}, {21, 1135, 1, 6079}, {22, 1118, 2, 6079}, {22, 1145, 1, 6079}, {23, 1120, 1, 6079}, {23, 1137, 2, 6079}, {24, 1121, 2, 6079}, {24, 1138, 2, 6079}, {24, 1147, 2, 6079}, {25, 1122, 1, 6079}, {25, 1148, 2, 6079}, {26, 1140, 1, 6079}, {27, 1141, 2, 6079}, {27, 1150, 1, 6079}, {28, 1142, 1, 6079}, {28, 1151, 2, 6079}, {29, 1152, 1, 6079}, {20, 1116, 1, 6080}, {21, 1118, 2, 6080}, {21, 1136, 1, 6080}, {22, 1119, 2, 6080}, {22, 1146, 1, 6080}, {23, 1121, 1, 6080}, {23, 1138, 2, 6080}, {24, 1122, 2, 6080}, {24, 1139, 2, 6080}, {24, 1148, 2, 6080}, {25, 1123, 1, 6080}, {25, 1149, 2, 6080}, {26, 1141, 1, 6080}, {27, 1142, 2, 6080}, {27, 1151, 1, 6080}, {28, 1143, 1, 6080}, {28, 1152, 2, 6080}, {29, 1153, 1, 6080}, {21, 1094, 1, 6081}, {23, 1095, 2, 6081}, {23, 1104, 1, 6081}, {24, 1096, 2, 6081}, {24, 1114, 1, 6081}, {26, 1097, 1, 6081}, {26, 1105, 2, 6081}, {27, 1098, 2, 6081}, {27, 1106, 2, 6081}, {27, 1115, 2, 6081}, {28, 1099, 1, 6081}, {28, 1116, 2, 6081}, {30, 1107, 1, 6081}, {31, 1108, 2, 6081}, {31, 1117, 1, 6081}, {32, 1109, 1, 6081}, {32, 1118, 2, 6081}, {33, 1119, 1, 6081}, {21, 1095, 1, 6082}, {23, 1097, 2, 6082}, {23, 1105, 1, 6082}, {24, 1098, 2, 6082}, {24, 1115, 1, 6082}, {26, 1100, 1, 6082}, {26, 1107, 2, 6082}, {27, 1101, 2, 6082}, {27, 1108, 2, 6082}, {27, 1117, 2, 6082}, {28, 1102, 1, 6082}, {28, 1118, 2, 6082}, {30, 1110, 1, 6082}, {31, 1111, 2, 6082}, {31, 1120, 1, 6082}, {32, 1112, 1, 6082}, {32, 1121, 2, 6082}, {33, 1122, 1, 6082}, {21, 1096, 1, 6083}, {23, 1098, 2, 6083}, {23, 1106, 1, 6083}, {24, 1099, 2, 6083}, {24, 1116, 1, 6083}, {26, 1101, 1, 6083}, {26, 1108, 2, 6083}, {27, 1102, 2, 6083}, {27, 1109, 2, 6083}, {27, 1118, 2, 6083}, {28, 1103, 1, 6083}, {28, 1119, 2, 6083}, {30, 1111, 1, 6083}, {31, 1112, 2, 6083}, {31, 1121, 1, 6083}, {32, 1113, 1, 6083}, {32, 1122, 2, 6083}, {33, 1123, 1, 6083}, {21, 1104, 1, 6084}, {23, 1105, 2, 6084}, {23, 1124, 1, 6084}, {24, 1106, 2, 6084}, {24, 1134, 1, 6084}, {26, 1107, 1, 6084}, {26, 1125, 2, 6084}, {27, 1108, 2, 6084}, {27, 1126, 2, 6084}, {27, 1135, 2, 6084}, {28, 1109, 1, 6084}, {28, 1136, 2, 6084}, {30, 1127, 1, 6084}, {31, 1128, 2, 6084}, {31, 1137, 1, 6084}, {32, 1129, 1, 6084}, {32, 1138, 2, 6084}, {33, 1139, 1, 6084}, {21, 1105, 1, 6085}, {23, 1107, 2, 6085}, {23, 1125, 1, 6085}, {24, 1108, 2, 6085}, {24, 1135, 1, 6085}, {26, 1110, 1, 6085}, {26, 1127, 2, 6085}, {27, 1111, 2, 6085}, {27, 1128, 2, 6085}, {27, 1137, 2, 6085}, {28, 1112, 1, 6085}, {28, 1138, 2, 6085}, {30, 1130, 1, 6085}, {31, 1131, 2, 6085}, {31, 1140, 1, 6085}, {32, 1132, 1, 6085}, {32, 1141, 2, 6085}, {33, 1142, 1, 6085}, {21, 1106, 1, 6086}, {23, 1108, 2, 6086}, {23, 1126, 1, 6086}, {24, 1109, 2, 6086}, {24, 1136, 1, 6086}, {26, 1111, 1, 6086}, {26, 1128, 2, 6086}, {27, 1112, 2, 6086}, {27, 1129, 2, 6086}, {27, 1138, 2, 6086}, {28, 1113, 1, 6086}, {28, 1139, 2, 6086}, {30, 1131, 1, 6086}, {31, 1132, 2, 6086}, {31, 1141, 1, 6086}, {32, 1133, 1, 6086}, {32, 1142, 2, 6086}, {33, 1143, 1, 6086}, {21, 1114, 1, 6087}, {23, 1115, 2, 6087}, {23, 1134, 1, 6087}, {24, 1116, 2, 6087}, {24, 1144, 1, 6087}, {26, 1117, 1, 6087}, {26, 1135, 2, 6087}, {27, 1118, 2, 6087}, {27, 1136, 2, 6087}, {27, 1145, 2, 6087}, {28, 1119, 1, 6087}, {28, 1146, 2, 6087}, {30, 1137, 1, 6087}, {31, 1138, 2, 6087}, {31, 1147, 1, 6087}, {32, 1139, 1, 6087}, {32, 1148, 2, 6087}, {33, 1149, 1, 6087}, {21, 1115, 1, 6088}, {23, 1117, 2, 6088}, {23, 1135, 1, 6088}, {24, 1118, 2, 6088}, {24, 1145, 1, 6088}, {26, 1120, 1, 6088}, {26, 1137, 2, 6088}, {27, 1121, 2, 6088}, {27, 1138, 2, 6088}, {27, 1147, 2, 6088}, {28, 1122, 1, 6088}, {28, 1148, 2, 6088}, {30, 1140, 1, 6088}, {31, 1141, 2, 6088}, {31, 1150, 1, 6088}, {32, 1142, 1, 6088}, {32, 1151, 2, 6088}, {33, 1152, 1, 6088}, {21, 1116, 1, 6089}, {23, 1118, 2, 6089}, {23, 1136, 1, 6089}, {24, 1119, 2, 6089}, {24, 1146, 1, 6089}, {26, 1121, 1, 6089}, {26, 1138, 2, 6089}, {27, 1122, 2, 6089}, {27, 1139, 2, 6089}, {27, 1148, 2, 6089}, {28, 1123, 1, 6089}, {28, 1149, 2, 6089}, {30, 1141, 1, 6089}, {31, 1142, 2, 6089}, {31, 1151, 1, 6089}, {32, 1143, 1, 6089}, {32, 1152, 2, 6089}, {33, 1153, 1, 6089}, {22, 1094, 1, 6090}, {24, 1095, 2, 6090}, {24, 1104, 1, 6090}, {25, 1096, 2, 6090}, {25, 1114, 1, 6090}, {27, 1097, 1, 6090}, {27, 1105, 2, 6090}, {28, 1098, 2, 6090}, {28, 1106, 2, 6090}, {28, 1115, 2, 6090}, {29, 1099, 1, 6090}, {29, 1116, 2, 6090}, {31, 1107, 1, 6090}, {32, 1108, 2, 6090}, {32, 1117, 1, 6090}, {33, 1109, 1, 6090}, {33, 1118, 2, 6090}, {34, 1119, 1, 6090}, {22, 1095, 1, 6091}, {24, 1097, 2, 6091}, {24, 1105, 1, 6091}, {25, 1098, 2, 6091}, {25, 1115, 1, 6091}, {27, 1100, 1, 6091}, {27, 1107, 2, 6091}, {28, 1101, 2, 6091}, {28, 1108, 2, 6091}, {28, 1117, 2, 6091}, {29, 1102, 1, 6091}, {29, 1118, 2, 6091}, {31, 1110, 1, 6091}, {32, 1111, 2, 6091}, {32, 1120, 1, 6091}, {33, 1112, 1, 6091}, {33, 1121, 2, 6091}, {34, 1122, 1, 6091}, {22, 1096, 1, 6092}, {24, 1098, 2, 6092}, {24, 1106, 1, 6092}, {25, 1099, 2, 6092}, {25, 1116, 1, 6092}, {27, 1101, 1, 6092}, {27, 1108, 2, 6092}, {28, 1102, 2, 6092}, {28, 1109, 2,

6092}, {28, 1118, 2, 6092}, {29, 1103, 1, 6092}, {29, 1119, 2, 6092}, {31, 1111, 1, 6092},
{32, 1112, 2, 6092}, {32, 1121, 1, 6092}, {33, 1113, 1, 6092}, {33, 1122, 2, 6092}, {34,
1123, 1, 6092}, {22, 1104, 1, 6093}, {24, 1105, 2, 6093}, {24, 1124, 1, 6093}, {25, 1106,
2, 6093}, {25, 1134, 1, 6093}, {27, 1107, 1, 6093}, {27, 1125, 2, 6093}, {28, 1108, 2,
6093}, {28, 1126, 2, 6093}, {28, 1135, 2, 6093}, {29, 1109, 1, 6093}, {29, 1136, 2, 6093},
{31, 1127, 1, 6093}, {32, 1128, 2, 6093}, {32, 1137, 1, 6093}, {33, 1129, 1, 6093}, {33,
1138, 2, 6093}, {34, 1139, 1, 6093}, {22, 1105, 1, 6094}, {24, 1107, 2, 6094}, {24, 1125,
1, 6094}, {25, 1108, 2, 6094}, {25, 1135, 1, 6094}, {27, 1110, 1, 6094}, {27, 1127, 2,
6094}, {28, 1111, 2, 6094}, {28, 1128, 2, 6094}, {28, 1137, 2, 6094}, {29, 1112, 1, 6094},
{29, 1138, 2, 6094}, {31, 1130, 1, 6094}, {32, 1131, 2, 6094}, {32, 1140, 1, 6094}, {33,
1132, 1, 6094}, {33, 1141, 2, 6094}, {34, 1142, 1, 6094}, {22, 1106, 1, 6095}, {24, 1108,
2, 6095}, {24, 1126, 1, 6095}, {25, 1109, 2, 6095}, {25, 1136, 1, 6095}, {27, 1111, 1,
6095}, {27, 1128, 2, 6095}, {28, 1112, 2, 6095}, {28, 1129, 2, 6095}, {28, 1138, 2, 6095},
{29, 1113, 1, 6095}, {29, 1139, 2, 6095}, {31, 1131, 1, 6095}, {32, 1132, 2, 6095}, {32,
1141, 1, 6095}, {33, 1133, 1, 6095}, {33, 1142, 2, 6095}, {34, 1143, 1, 6095}, {22, 1114,
1, 6096}, {24, 1115, 2, 6096}, {24, 1134, 1, 6096}, {25, 1116, 2, 6096}, {25, 1144, 1,
6096}, {27, 1117, 1, 6096}, {27, 1135, 2, 6096}, {28, 1118, 2, 6096}, {28, 1136, 2, 6096},
{28, 1145, 2, 6096}, {29, 1119, 1, 6096}, {29, 1146, 2, 6096}, {31, 1137, 1, 6096}, {32,
1138, 2, 6096}, {32, 1147, 1, 6096}, {33, 1139, 1, 6096}, {33, 1148, 2, 6096}, {34, 1149,
1, 6096}, {22, 1115, 1, 6097}, {24, 1117, 2, 6097}, {24, 1135, 1, 6097}, {25, 1118, 2,
6097}, {25, 1145, 1, 6097}, {27, 1120, 1, 6097}, {27, 1137, 2, 6097}, {28, 1121, 2, 6097},
{28, 1138, 2, 6097}, {28, 1147, 2, 6097}, {29, 1122, 1, 6097}, {29, 1148, 2, 6097}, {31,
1140, 1, 6097}, {32, 1141, 2, 6097}, {32, 1150, 1, 6097}, {33, 1142, 1, 6097}, {33, 1151,
2, 6097}, {34, 1152, 1, 6097}, {22, 1116, 1, 6098}, {24, 1118, 2, 6098}, {24, 1136, 1,
6098}, {25, 1119, 2, 6098}, {25, 1146, 1, 6098}, {27, 1121, 1, 6098}, {27, 1138, 2, 6098},
{28, 1122, 2, 6098}, {28, 1139, 2, 6098}, {28, 1148, 2, 6098}, {29, 1123, 1, 6098}, {29,
1149, 2, 6098}, {31, 1141, 1, 6098}, {32, 1142, 2, 6098}, {32, 1151, 1, 6098}, {33, 1143,
1, 6098}, {33, 1152, 2, 6098}, {34, 1153, 1, 6098}, {20, 1154, 1, 6099}, {21, 1155, 2,
6099}, {21, 1169, 1, 6099}, {22, 1156, 2, 6099}, {22, 1184, 1, 6099}, {23, 1157, 1, 6099},
{23, 1170, 2, 6099}, {24, 1158, 2, 6099}, {24, 1171, 2, 6099}, {24, 1185, 2, 6099}, {25,
1159, 1, 6099}, {25, 1186, 2, 6099}, {26, 1172, 1, 6099}, {27, 1173, 2, 6099}, {27, 1187,
1, 6099}, {28, 1174, 1, 6099}, {28, 1188, 2, 6099}, {29, 1189, 1, 6099}, {20, 1155, 1,
6100}, {21, 1157, 2, 6100}, {21, 1170, 1, 6100}, {22, 1158, 2, 6100}, {22, 1185, 1, 6100},
{23, 1160, 1, 6100}, {23, 1172, 2, 6100}, {24, 1161, 2, 6100}, {24, 1173, 2, 6100}, {24,
1187, 2, 6100}, {25, 1162, 1, 6100}, {25, 1188, 2, 6100}, {26, 1175, 1, 6100}, {27, 1176,
2, 6100}, {27, 1190, 1, 6100}, {28, 1177, 1, 6100}, {28, 1191, 2, 6100}, {29, 1192, 1,
6100}, {20, 1156, 1, 6101}, {21, 1158, 2, 6101}, {21, 1171, 1, 6101}, {22, 1159, 2, 6101},
{22, 1186, 1, 6101}, {23, 1161, 1, 6101}, {23, 1173, 2, 6101}, {24, 1162, 2, 6101}, {24,
1174, 2, 6101}, {24, 1188, 2, 6101}, {25, 1163, 1, 6101}, {25, 1189, 2, 6101}, {26, 1176,
1, 6101}, {27, 1177, 2, 6101}, {27, 1191, 1, 6101}, {28, 1178, 1, 6101}, {28, 1192, 2,
6101}, {29, 1193, 1, 6101}, {20, 1157, 1, 6102}, {21, 1160, 2, 6102}, {21, 1172, 1, 6102},
{22, 1161, 2, 6102}, {22, 1187, 1, 6102}, {23, 1164, 1, 6102}, {23, 1175, 2, 6102}, {24,
1165, 2, 6102}, {24, 1176, 2, 6102}, {24, 1190, 2, 6102}, {25, 1166, 1, 6102}, {25, 1191,
2, 6102}, {26, 1179, 1, 6102}, {27, 1180, 2, 6102}, {27, 1194, 1, 6102}, {28, 1181, 1,
6102}, {28, 1195, 2, 6102}, {29, 1196, 1, 6102}, {20, 1158, 1, 6103}, {21, 1161, 2, 6103},
{21, 1173, 1, 6103}, {22, 1162, 2, 6103}, {22, 1188, 1, 6103}, {23, 1165, 1, 6103}, {23,
1176, 2, 6103}, {24, 1166, 2, 6103}, {24, 1177, 2, 6103}, {24, 1191, 2, 6103}, {25, 1167,
1, 6103}, {25, 1192, 2, 6103}, {26, 1180, 1, 6103}, {27, 1181, 2, 6103}, {27, 1195, 1,
6103}, {28, 1182, 1, 6103}, {28, 1196, 2, 6103}, {29, 1197, 1, 6103}, {20, 1159, 1, 6104},
{21, 1162, 2, 6104}, {21, 1174, 1, 6104}, {22, 1163, 2, 6104}, {22, 1189, 1, 6104}, {23,
1166, 1, 6104}, {23, 1177, 2, 6104}, {24, 1167, 2, 6104}, {24, 1178, 2, 6104}, {24, 1192,
2, 6104}, {25, 1168, 1, 6104}, {25, 1193, 2, 6104}, {26, 1181, 1, 6104}, {27, 1182, 2,
6104}, {27, 1196, 1, 6104}, {28, 1183, 1, 6104}, {28, 1197, 2, 6104}, {29, 1198, 1, 6104},
{20, 1169, 1, 6105}, {21, 1170, 2, 6105}, {21, 1199, 1, 6105}, {22, 1171, 2, 6105}, {22,
1214, 1, 6105}, {23, 1172, 1, 6105}, {23, 1200, 2, 6105}, {24, 1173, 2, 6105}, {24, 1201,
2, 6105}, {24, 1215, 2, 6105}, {25, 1174, 1, 6105}, {25, 1216, 2, 6105}, {26, 1202, 1,
6105}, {27, 1203, 2, 6105}, {27, 1217, 1, 6105}, {28, 1204, 1, 6105}, {28, 1218, 2, 6105},
{29, 1219, 1, 6105}, {20, 1170, 1, 6106}, {21, 1172, 2, 6106}, {21, 1200, 1, 6106}, {22,
1173, 2, 6106}, {22, 1215, 1, 6106}, {23, 1175, 1, 6106}, {23, 1202, 2, 6106}, {24, 1176,
2, 6106}, {24, 1203, 2, 6106}, {24, 1217, 2, 6106}, {25, 1177, 1, 6106}, {25, 1218, 2,
6106}, {26, 1205, 1, 6106}, {27, 1206, 2, 6106}, {27, 1220, 1, 6106}, {28, 1207, 1, 6106},
{28, 1221, 2, 6106}, {29, 1222, 1, 6106}, {20, 1171, 1, 6107}, {21, 1173, 2, 6107}, {21,
1201, 1, 6107}, {22, 1174, 2, 6107}, {22, 1216, 1, 6107}, {23, 1176, 1, 6107}, {23, 1203,
2, 6107}, {24, 1177, 2, 6107}, {24, 1204, 2, 6107}, {24, 1218, 2, 6107}, {25, 1178, 1,
6107}, {25, 1219, 2, 6107}, {26, 1206, 1, 6107}, {27, 1207, 2, 6107}, {27, 1221, 1, 6107},
{28, 1208, 1, 6107}, {28, 1222, 2, 6107}, {29, 1223, 1, 6107}, {20, 1172, 1, 6108}, {21,
1175, 2, 6108}, {21, 1202, 1, 6108}, {22, 1176, 2, 6108}, {22, 1217, 1, 6108}, {23, 1179,
1, 6108}, {23, 1205, 2, 6108}, {24, 1180, 2, 6108}, {24, 1206, 2, 6108}, {24, 1220, 2,
6108}, {25, 1181, 1, 6108}, {25, 1221, 2, 6108}, {26, 1209, 1, 6108}, {27, 1210, 2, 6108},

{27, 1224, 1, 6108}, {28, 1211, 1, 6108}, {28, 1225, 2, 6108}, {29, 1226, 1, 6108}, {20, 1173, 1, 6109}, {21, 1176, 2, 6109}, {21, 1203, 1, 6109}, {22, 1177, 2, 6109}, {22, 1218, 1, 6109}, {23, 1180, 1, 6109}, {23, 1206, 2, 6109}, {24, 1181, 2, 6109}, {24, 1207, 2, 6109}, {24, 1221, 2, 6109}, {25, 1182, 1, 6109}, {25, 1222, 2, 6109}, {26, 1210, 1, 6109}, {27, 1211, 2, 6109}, {27, 1225, 1, 6109}, {28, 1212, 1, 6109}, {28, 1226, 2, 6109}, {29, 1227, 1, 6109}, {20, 1174, 1, 6110}, {21, 1177, 2, 6110}, {21, 1204, 1, 6110}, {22, 1178, 2, 6110}, {22, 1219, 1, 6110}, {23, 1181, 1, 6110}, {23, 1207, 2, 6110}, {24, 1182, 2, 6110}, {24, 1208, 2, 6110}, {24, 1222, 2, 6110}, {25, 1183, 1, 6110}, {25, 1223, 2, 6110}, {26, 1211, 1, 6110}, {27, 1212, 2, 6110}, {27, 1226, 1, 6110}, {28, 1213, 1, 6110}, {28, 1227, 2, 6110}, {29, 1228, 1, 6110}, {20, 1184, 1, 6111}, {21, 1185, 2, 6111}, {21, 1214, 1, 6111}, {22, 1186, 2, 6111}, {22, 1229, 1, 6111}, {23, 1187, 1, 6111}, {23, 1215, 2, 6111}, {24, 1188, 2, 6111}, {24, 1216, 2, 6111}, {24, 1230, 2, 6111}, {25, 1189, 1, 6111}, {25, 1231, 2, 6111}, {26, 1217, 1, 6111}, {27, 1218, 2, 6111}, {27, 1232, 1, 6111}, {28, 1219, 1, 6111}, {28, 1233, 2, 6111}, {29, 1234, 1, 6111}, {20, 1185, 1, 6112}, {21, 1187, 2, 6112}, {21, 1215, 1, 6112}, {22, 1188, 2, 6112}, {22, 1230, 1, 6112}, {23, 1190, 1, 6112}, {23, 1217, 2, 6112}, {24, 1191, 2, 6112}, {24, 1218, 2, 6112}, {24, 1232, 2, 6112}, {25, 1192, 1, 6112}, {25, 1233, 2, 6112}, {26, 1220, 1, 6112}, {27, 1221, 2, 6112}, {27, 1235, 1, 6112}, {28, 1222, 1, 6112}, {28, 1236, 2, 6112}, {29, 1237, 1, 6112}, {20, 1186, 1, 6113}, {21, 1188, 2, 6113}, {21, 1216, 1, 6113}, {22, 1189, 2, 6113}, {22, 1231, 1, 6113}, {23, 1191, 1, 6113}, {23, 1218, 2, 6113}, {24, 1192, 2, 6113}, {24, 1219, 2, 6113}, {24, 1233, 2, 6113}, {25, 1193, 1, 6113}, {25, 1234, 2, 6113}, {26, 1221, 1, 6113}, {27, 1222, 2, 6113}, {27, 1236, 1, 6113}, {28, 1223, 1, 6113}, {28, 1237, 2, 6113}, {29, 1238, 1, 6113}, {20, 1187, 1, 6114}, {21, 1190, 2, 6114}, {21, 1217, 1, 6114}, {22, 1191, 2, 6114}, {22, 1232, 1, 6114}, {23, 1194, 1, 6114}, {23, 1220, 2, 6114}, {24, 1195, 2, 6114}, {24, 1221, 2, 6114}, {24, 1235, 2, 6114}, {25, 1196, 1, 6114}, {25, 1236, 2, 6114}, {26, 1224, 1, 6114}, {27, 1225, 2, 6114}, {27, 1239, 1, 6114}, {28, 1226, 1, 6114}, {28, 1240, 2, 6114}, {29, 1241, 1, 6114}, {20, 1188, 1, 6115}, {21, 1191, 2, 6115}, {21, 1218, 1, 6115}, {22, 1192, 2, 6115}, {22, 1233, 1, 6115}, {23, 1195, 1, 6115}, {23, 1221, 2, 6115}, {24, 1196, 2, 6115}, {24, 1222, 2, 6115}, {24, 1236, 2, 6115}, {25, 1197, 1, 6115}, {25, 1237, 2, 6115}, {26, 1225, 1, 6115}, {27, 1226, 2, 6115}, {27, 1240, 1, 6115}, {28, 1227, 1, 6115}, {28, 1241, 2, 6115}, {29, 1242, 1, 6115}, {20, 1189, 1, 6116}, {21, 1192, 2, 6116}, {21, 1219, 1, 6116}, {22, 1193, 2, 6116}, {22, 1234, 1, 6116}, {23, 1196, 1, 6116}, {23, 1222, 2, 6116}, {24, 1197, 2, 6116}, {24, 1223, 2, 6116}, {24, 1237, 2, 6116}, {25, 1198, 1, 6116}, {25, 1238, 2, 6116}, {26, 1226, 1, 6116}, {27, 1227, 2, 6116}, {27, 1241, 1, 6116}, {28, 1228, 1, 6116}, {28, 1242, 2, 6116}, {29, 1243, 1, 6116}, {21, 1154, 1, 6117}, {23, 1155, 2, 6117}, {23, 1169, 1, 6117}, {24, 1156, 2, 6117}, {24, 1184, 1, 6117}, {26, 1157, 1, 6117}, {26, 1170, 2, 6117}, {27, 1158, 2, 6117}, {27, 1171, 2, 6117}, {27, 1185, 2, 6117}, {28, 1159, 1, 6117}, {28, 1186, 2, 6117}, {30, 1172, 1, 6117}, {31, 1173, 2, 6117}, {31, 1187, 1, 6117}, {32, 1174, 1, 6117}, {32, 1188, 2, 6117}, {33, 1189, 1, 6117}, {21, 1155, 1, 6118}, {23, 1157, 2, 6118}, {23, 1170, 1, 6118}, {24, 1158, 2, 6118}, {24, 1185, 1, 6118}, {26, 1160, 1, 6118}, {26, 1172, 2, 6118}, {27, 1161, 2, 6118}, {27, 1173, 2, 6118}, {27, 1187, 2, 6118}, {28, 1162, 1, 6118}, {28, 1188, 2, 6118}, {30, 1175, 1, 6118}, {31, 1176, 2, 6118}, {31, 1190, 1, 6118}, {32, 1177, 1, 6118}, {32, 1191, 2, 6118}, {33, 1192, 1, 6118}, {21, 1156, 1, 6119}, {23, 1158, 2, 6119}, {23, 1171, 1, 6119}, {24, 1159, 2, 6119}, {24, 1186, 1, 6119}, {26, 1161, 1, 6119}, {26, 1173, 2, 6119}, {27, 1162, 2, 6119}, {27, 1174, 2, 6119}, {27, 1188, 2, 6119}, {28, 1163, 1, 6119}, {28, 1189, 2, 6119}, {30, 1176, 1, 6119}, {31, 1177, 2, 6119}, {31, 1191, 1, 6119}, {32, 1178, 1, 6119}, {32, 1192, 2, 6119}, {33, 1193, 1, 6119}, {21, 1157, 1, 6120}, {23, 1160, 2, 6120}, {23, 1172, 1, 6120}, {24, 1161, 2, 6120}, {24, 1187, 1, 6120}, {26, 1164, 1, 6120}, {26, 1175, 2, 6120}, {27, 1165, 2, 6120}, {27, 1176, 2, 6120}, {27, 1190, 2, 6120}, {28, 1166, 1, 6120}, {28, 1191, 2, 6120}, {30, 1179, 1, 6120}, {31, 1180, 2, 6120}, {31, 1194, 1, 6120}, {32, 1181, 1, 6120}, {32, 1195, 2, 6120}, {33, 1196, 1, 6120}, {21, 1158, 1, 6121}, {23, 1161, 2, 6121}, {23, 1173, 1, 6121}, {24, 1162, 2, 6121}, {24, 1188, 1, 6121}, {26, 1165, 1, 6121}, {26, 1176, 2, 6121}, {27, 1166, 2, 6121}, {27, 1177, 2, 6121}, {27, 1191, 2, 6121}, {28, 1167, 1, 6121}, {28, 1192, 2, 6121}, {30, 1180, 1, 6121}, {31, 1181, 2, 6121}, {31, 1195, 1, 6121}, {32, 1182, 1, 6121}, {32, 1196, 2, 6121}, {33, 1197, 1, 6121}, {21, 1159, 1, 6122}, {23, 1162, 2, 6122}, {23, 1174, 1, 6122}, {24, 1163, 2, 6122}, {24, 1189, 1, 6122}, {26, 1166, 1, 6122}, {26, 1177, 2, 6122}, {27, 1167, 2, 6122}, {27, 1178, 2, 6122}, {27, 1192, 2, 6122}, {28, 1168, 1, 6122}, {28, 1193, 2, 6122}, {30, 1181, 1, 6122}, {31, 1182, 2, 6122}, {31, 1196, 1, 6122}, {32, 1183, 1, 6122}, {32, 1197, 2, 6122}, {33, 1198, 1, 6122}, {21, 1169, 1, 6123}, {23, 1170, 2, 6123}, {23, 1199, 1, 6123}, {24, 1171, 2, 6123}, {24, 1214, 1, 6123}, {26, 1172, 1, 6123}, {26, 1200, 2, 6123}, {27, 1173, 2, 6123}, {27, 1201, 2, 6123}, {27, 1215, 2, 6123}, {28, 1174, 1, 6123}, {28, 1216, 2, 6123}, {30, 1202, 1, 6123}, {31, 1203, 2, 6123}, {31, 1217, 1, 6123}, {32, 1204, 1, 6123}, {32, 1218, 2, 6123}, {33, 1219, 1, 6123}, {21, 1170, 1, 6124}, {23, 1172, 2, 6124}, {23, 1200, 1, 6124}, {24, 1173, 2, 6124}, {24, 1215, 1, 6124}, {26, 1175, 1, 6124}, {26, 1202, 2, 6124}, {27, 1176, 2, 6124}, {27, 1203, 2, 6124}, {27, 1217, 2, 6124}, {28, 1177, 1, 6124}, {28, 1218, 2, 6124}, {30, 1205, 1, 6124}, {31, 1206, 2, 6124}, {31, 1220, 1, 6124}, {32, 1207, 1, 6124}, {32, 1221, 2, 6124}, {33, 1222, 1, 6124}, {21, 1171, 1, 6125}, {23,

1173, 2, 6125}, {23, 1201, 1, 6125}, {24, 1174, 2, 6125}, {24, 1216, 1, 6125}, {26, 1176, 1, 6125}, {26, 1203, 2, 6125}, {27, 1177, 2, 6125}, {27, 1204, 2, 6125}, {27, 1218, 2, 6125}, {28, 1178, 1, 6125}, {28, 1219, 2, 6125}, {30, 1206, 1, 6125}, {31, 1207, 2, 6125}, {31, 1221, 1, 6125}, {32, 1208, 1, 6125}, {32, 1222, 2, 6125}, {33, 1223, 1, 6125}, {21, 1172, 1, 6126}, {23, 1175, 2, 6126}, {23, 1202, 1, 6126}, {24, 1176, 2, 6126}, {24, 1217, 1, 6126}, {26, 1179, 1, 6126}, {26, 1205, 2, 6126}, {27, 1180, 2, 6126}, {27, 1206, 2, 6126}, {27, 1220, 2, 6126}, {28, 1181, 1, 6126}, {28, 1221, 2, 6126}, {30, 1209, 1, 6126}, {31, 1210, 2, 6126}, {31, 1224, 1, 6126}, {32, 1211, 1, 6126}, {32, 1225, 2, 6126}, {33, 1226, 1, 6126}, {21, 1173, 1, 6127}, {23, 1176, 2, 6127}, {23, 1203, 1, 6127}, {24, 1177, 2, 6127}, {24, 1218, 1, 6127}, {26, 1180, 1, 6127}, {26, 1206, 2, 6127}, {27, 1181, 2, 6127}, {27, 1207, 2, 6127}, {27, 1221, 2, 6127}, {28, 1182, 1, 6127}, {28, 1222, 2, 6127}, {30, 1210, 1, 6127}, {31, 1211, 2, 6127}, {31, 1225, 1, 6127}, {32, 1212, 1, 6127}, {32, 1226, 2, 6127}, {33, 1227, 1, 6127}, {21, 1174, 1, 6128}, {23, 1177, 2, 6128}, {23, 1204, 1, 6128}, {24, 1178, 2, 6128}, {24, 1219, 1, 6128}, {26, 1181, 1, 6128}, {26, 1207, 2, 6128}, {27, 1182, 2, 6128}, {27, 1208, 2, 6128}, {27, 1222, 2, 6128}, {28, 1183, 1, 6128}, {28, 1223, 2, 6128}, {30, 1211, 1, 6128}, {31, 1212, 2, 6128}, {31, 1226, 1, 6128}, {32, 1213, 1, 6128}, {32, 1227, 2, 6128}, {33, 1228, 1, 6128}, {21, 1184, 1, 6129}, {23, 1185, 2, 6129}, {23, 1214, 1, 6129}, {24, 1186, 2, 6129}, {24, 1229, 1, 6129}, {26, 1187, 1, 6129}, {26, 1215, 2, 6129}, {27, 1188, 2, 6129}, {27, 1216, 2, 6129}, {27, 1230, 2, 6129}, {28, 1189, 1, 6129}, {28, 1231, 2, 6129}, {30, 1217, 1, 6129}, {31, 1218, 2, 6129}, {31, 1232, 1, 6129}, {32, 1219, 1, 6129}, {32, 1233, 2, 6129}, {33, 1234, 1, 6129}, {21, 1185, 1, 6130}, {23, 1187, 2, 6130}, {23, 1215, 1, 6130}, {24, 1188, 2, 6130}, {24, 1230, 1, 6130}, {26, 1190, 1, 6130}, {26, 1217, 2, 6130}, {27, 1191, 2, 6130}, {27, 1218, 2, 6130}, {27, 1232, 2, 6130}, {28, 1192, 1, 6130}, {28, 1233, 2, 6130}, {30, 1220, 1, 6130}, {31, 1221, 2, 6130}, {31, 1235, 1, 6130}, {32, 1222, 1, 6130}, {32, 1236, 2, 6130}, {33, 1237, 1, 6130}, {21, 1186, 1, 6131}, {23, 1188, 2, 6131}, {23, 1216, 1, 6131}, {24, 1189, 2, 6131}, {24, 1231, 1, 6131}, {26, 1191, 1, 6131}, {26, 1218, 2, 6131}, {27, 1192, 2, 6131}, {27, 1219, 2, 6131}, {27, 1233, 2, 6131}, {28, 1193, 1, 6131}, {28, 1234, 2, 6131}, {30, 1221, 1, 6131}, {31, 1222, 2, 6131}, {31, 1236, 1, 6131}, {32, 1223, 1, 6131}, {32, 1237, 2, 6131}, {33, 1238, 1, 6131}, {21, 1187, 1, 6132}, {23, 1190, 2, 6132}, {23, 1217, 1, 6132}, {24, 1191, 2, 6132}, {24, 1232, 1, 6132}, {26, 1194, 1, 6132}, {26, 1220, 2, 6132}, {27, 1195, 2, 6132}, {27, 1221, 2, 6132}, {27, 1235, 2, 6132}, {28, 1196, 1, 6132}, {28, 1236, 2, 6132}, {30, 1224, 1, 6132}, {31, 1225, 2, 6132}, {31, 1239, 1, 6132}, {32, 1226, 1, 6132}, {32, 1240, 2, 6132}, {33, 1241, 1, 6132}, {21, 1188, 1, 6133}, {23, 1191, 2, 6133}, {23, 1218, 1, 6133}, {24, 1192, 2, 6133}, {24, 1233, 1, 6133}, {26, 1195, 1, 6133}, {26, 1221, 2, 6133}, {27, 1196, 2, 6133}, {27, 1222, 2, 6133}, {27, 1236, 2, 6133}, {28, 1197, 1, 6133}, {28, 1237, 2, 6133}, {30, 1225, 1, 6133}, {31, 1226, 2, 6133}, {31, 1240, 1, 6133}, {32, 1227, 1, 6133}, {32, 1241, 2, 6133}, {33, 1242, 1, 6133}, {21, 1189, 1, 6134}, {23, 1192, 2, 6134}, {23, 1219, 1, 6134}, {24, 1193, 2, 6134}, {24, 1234, 1, 6134}, {26, 1196, 1, 6134}, {26, 1222, 2, 6134}, {27, 1197, 2, 6134}, {27, 1223, 2, 6134}, {27, 1237, 2, 6134}, {28, 1198, 1, 6134}, {28, 1238, 2, 6134}, {30, 1226, 1, 6134}, {31, 1227, 2, 6134}, {31, 1241, 1, 6134}, {32, 1228, 1, 6134}, {32, 1242, 2, 6134}, {33, 1243, 1, 6134}, {22, 1154, 1, 6135}, {24, 1155, 2, 6135}, {24, 1169, 1, 6135}, {25, 1156, 2, 6135}, {25, 1184, 1, 6135}, {27, 1157, 1, 6135}, {27, 1170, 2, 6135}, {28, 1158, 2, 6135}, {28, 1171, 2, 6135}, {28, 1185, 2, 6135}, {29, 1159, 1, 6135}, {29, 1186, 2, 6135}, {31, 1172, 1, 6135}, {32, 1173, 2, 6135}, {32, 1187, 1, 6135}, {33, 1174, 1, 6135}, {33, 1188, 2, 6135}, {34, 1189, 1, 6135}, {22, 1155, 1, 6136}, {24, 1157, 2, 6136}, {24, 1170, 1, 6136}, {25, 1158, 2, 6136}, {25, 1185, 1, 6136}, {27, 1160, 1, 6136}, {27, 1172, 2, 6136}, {28, 1161, 2, 6136}, {28, 1173, 2, 6136}, {28, 1187, 2, 6136}, {29, 1162, 1, 6136}, {29, 1188, 2, 6136}, {31, 1175, 1, 6136}, {32, 1176, 2, 6136}, {32, 1190, 1, 6136}, {33, 1177, 1, 6136}, {33, 1191, 2, 6136}, {34, 1192, 1, 6136}, {22, 1156, 1, 6137}, {24, 1158, 2, 6137}, {24, 1171, 1, 6137}, {25, 1159, 2, 6137}, {25, 1186, 1, 6137}, {27, 1161, 1, 6137}, {27, 1173, 2, 6137}, {28, 1162, 2, 6137}, {28, 1174, 2, 6137}, {28, 1188, 2, 6137}, {29, 1163, 1, 6137}, {29, 1189, 2, 6137}, {31, 1176, 1, 6137}, {32, 1177, 2, 6137}, {32, 1191, 1, 6137}, {33, 1178, 1, 6137}, {33, 1192, 2, 6137}, {34, 1193, 1, 6137}, {22, 1157, 1, 6138}, {24, 1160, 2, 6138}, {24, 1172, 1, 6138}, {25, 1161, 2, 6138}, {25, 1187, 1, 6138}, {27, 1164, 1, 6138}, {27, 1175, 2, 6138}, {28, 1165, 2, 6138}, {28, 1176, 2, 6138}, {28, 1190, 2, 6138}, {29, 1166, 1, 6138}, {29, 1191, 2, 6138}, {31, 1179, 1, 6138}, {32, 1180, 2, 6138}, {32, 1194, 1, 6138}, {33, 1181, 1, 6138}, {33, 1195, 2, 6138}, {34, 1196, 1, 6138}, {22, 1158, 1, 6139}, {24, 1161, 2, 6139}, {24, 1173, 1, 6139}, {25, 1162, 2, 6139}, {25, 1188, 1, 6139}, {27, 1165, 1, 6139}, {27, 1176, 2, 6139}, {28, 1166, 2, 6139}, {28, 1177, 2, 6139}, {28, 1191, 2, 6139}, {29, 1167, 1, 6139}, {29, 1192, 2, 6139}, {31, 1180, 1, 6139}, {32, 1181, 2, 6139}, {32, 1195, 1, 6139}, {33, 1182, 1, 6139}, {33, 1196, 2, 6139}, {34, 1197, 1, 6139}, {22, 1159, 1, 6140}, {24, 1162, 2, 6140}, {24, 1174, 1, 6140}, {25, 1163, 2, 6140}, {25, 1189, 1, 6140}, {27, 1166, 1, 6140}, {27, 1177, 2, 6140}, {28, 1167, 2, 6140}, {28, 1178, 2, 6140}, {28, 1192, 2, 6140}, {29, 1168, 1, 6140}, {29, 1193, 2, 6140}, {31, 1181, 1, 6140}, {32, 1182, 2, 6140}, {32, 1196, 1, 6140}, {33, 1183, 1, 6140}, {33, 1197, 2, 6140}, {34, 1198, 1, 6140}, {22, 1169, 1, 6141}, {24, 1170, 2, 6141}, {24, 1199, 1, 6141}, {25, 1171, 2, 6141}, {25, 1214, 1, 6141}, {27, 1172, 1, 6141}, {27, 1200,

2, 6141}, {28, 1173, 2, 6141}, {28, 1201, 2, 6141}, {28, 1215, 2, 6141}, {29, 1174, 1, 6141}, {29, 1216, 2, 6141}, {31, 1202, 1, 6141}, {32, 1203, 2, 6141}, {32, 1217, 1, 6141}, {33, 1204, 1, 6141}, {33, 1218, 2, 6141}, {34, 1219, 1, 6141}, {22, 1170, 1, 6142}, {24, 1172, 2, 6142}, {24, 1200, 1, 6142}, {25, 1173, 2, 6142}, {25, 1215, 1, 6142}, {27, 1175, 1, 6142}, {27, 1202, 2, 6142}, {28, 1176, 2, 6142}, {28, 1203, 2, 6142}, {28, 1217, 2, 6142}, {29, 1177, 1, 6142}, {29, 1218, 2, 6142}, {31, 1205, 1, 6142}, {32, 1206, 2, 6142}, {32, 1220, 1, 6142}, {33, 1207, 1, 6142}, {33, 1221, 2, 6142}, {34, 1222, 1, 6142}, {22, 1171, 1, 6143}, {24, 1173, 2, 6143}, {24, 1201, 1, 6143}, {25, 1174, 2, 6143}, {25, 1216, 1, 6143}, {27, 1176, 1, 6143}, {27, 1203, 2, 6143}, {28, 1177, 2, 6143}, {28, 1204, 2, 6143}, {28, 1218, 2, 6143}, {29, 1178, 1, 6143}, {29, 1219, 2, 6143}, {31, 1206, 1, 6143}, {32, 1207, 2, 6143}, {32, 1221, 1, 6143}, {33, 1208, 1, 6143}, {33, 1222, 2, 6143}, {34, 1223, 1, 6143}, {22, 1172, 1, 6144}, {24, 1175, 2, 6144}, {24, 1202, 1, 6144}, {25, 1176, 2, 6144}, {25, 1217, 1, 6144}, {27, 1179, 1, 6144}, {27, 1205, 2, 6144}, {28, 1180, 2, 6144}, {28, 1206, 2, 6144}, {28, 1220, 2, 6144}, {29, 1181, 1, 6144}, {29, 1221, 2, 6144}, {31, 1209, 1, 6144}, {32, 1210, 2, 6144}, {32, 1224, 1, 6144}, {33, 1211, 1, 6144}, {33, 1225, 2, 6144}, {34, 1226, 1, 6144}, {22, 1173, 1, 6145}, {24, 1176, 2, 6145}, {24, 1203, 1, 6145}, {25, 1177, 2, 6145}, {25, 1218, 1, 6145}, {27, 1180, 1, 6145}, {27, 1206, 2, 6145}, {28, 1181, 2, 6145}, {28, 1207, 2, 6145}, {28, 1221, 2, 6145}, {29, 1182, 1, 6145}, {29, 1222, 2, 6145}, {31, 1210, 1, 6145}, {32, 1211, 2, 6145}, {32, 1225, 1, 6145}, {33, 1212, 1, 6145}, {33, 1226, 2, 6145}, {34, 1227, 1, 6145}, {22, 1174, 1, 6146}, {24, 1177, 2, 6146}, {24, 1204, 1, 6146}, {25, 1178, 2, 6146}, {25, 1219, 1, 6146}, {27, 1181, 1, 6146}, {27, 1207, 2, 6146}, {28, 1182, 2, 6146}, {28, 1208, 2, 6146}, {28, 1222, 2, 6146}, {29, 1183, 1, 6146}, {29, 1223, 2, 6146}, {31, 1211, 1, 6146}, {32, 1212, 2, 6146}, {32, 1226, 1, 6146}, {33, 1213, 1, 6146}, {33, 1227, 2, 6146}, {34, 1228, 1, 6146}, {22, 1184, 1, 6147}, {24, 1185, 2, 6147}, {24, 1214, 1, 6147}, {25, 1186, 2, 6147}, {25, 1229, 1, 6147}, {27, 1187, 1, 6147}, {27, 1215, 2, 6147}, {28, 1188, 2, 6147}, {28, 1216, 2, 6147}, {28, 1230, 2, 6147}, {29, 1189, 1, 6147}, {29, 1231, 2, 6147}, {31, 1217, 1, 6147}, {32, 1218, 2, 6147}, {32, 1232, 1, 6147}, {33, 1219, 1, 6147}, {33, 1233, 2, 6147}, {34, 1234, 1, 6147}, {22, 1185, 1, 6148}, {24, 1187, 2, 6148}, {24, 1215, 1, 6148}, {25, 1188, 2, 6148}, {25, 1230, 1, 6148}, {27, 1190, 1, 6148}, {27, 1217, 2, 6148}, {28, 1191, 2, 6148}, {28, 1218, 2, 6148}, {28, 1232, 2, 6148}, {29, 1192, 1, 6148}, {29, 1233, 2, 6148}, {31, 1220, 1, 6148}, {32, 1221, 2, 6148}, {32, 1235, 1, 6148}, {33, 1222, 1, 6148}, {33, 1236, 2, 6148}, {34, 1237, 1, 6148}, {22, 1186, 1, 6149}, {24, 1188, 2, 6149}, {24, 1216, 1, 6149}, {25, 1189, 2, 6149}, {25, 1231, 1, 6149}, {27, 1191, 1, 6149}, {27, 1218, 2, 6149}, {28, 1192, 2, 6149}, {28, 1219, 2, 6149}, {28, 1233, 2, 6149}, {29, 1193, 1, 6149}, {29, 1234, 2, 6149}, {31, 1221, 1, 6149}, {32, 1222, 2, 6149}, {32, 1236, 1, 6149}, {33, 1223, 1, 6149}, {33, 1237, 2, 6149}, {34, 1238, 1, 6149}, {22, 1187, 1, 6150}, {24, 1190, 2, 6150}, {24, 1217, 1, 6150}, {25, 1191, 2, 6150}, {25, 1232, 1, 6150}, {27, 1194, 1, 6150}, {27, 1220, 2, 6150}, {28, 1195, 2, 6150}, {28, 1221, 2, 6150}, {28, 1235, 2, 6150}, {29, 1196, 1, 6150}, {29, 1236, 2, 6150}, {31, 1224, 1, 6150}, {32, 1225, 2, 6150}, {32, 1239, 1, 6150}, {33, 1226, 1, 6150}, {33, 1240, 2, 6150}, {34, 1241, 1, 6150}, {22, 1188, 1, 6151}, {24, 1191, 2, 6151}, {24, 1218, 1, 6151}, {25, 1192, 2, 6151}, {25, 1233, 1, 6151}, {27, 1195, 1, 6151}, {27, 1221, 2, 6151}, {28, 1196, 2, 6151}, {28, 1222, 2, 6151}, {28, 1236, 2, 6151}, {29, 1197, 1, 6151}, {29, 1237, 2, 6151}, {31, 1225, 1, 6151}, {32, 1226, 2, 6151}, {32, 1240, 1, 6151}, {33, 1227, 1, 6151}, {33, 1241, 2, 6151}, {34, 1242, 1, 6151}, {22, 1189, 1, 6152}, {24, 1192, 2, 6152}, {24, 1219, 1, 6152}, {25, 1193, 2, 6152}, {25, 1234, 1, 6152}, {27, 1196, 1, 6152}, {27, 1222, 2, 6152}, {28, 1197, 2, 6152}, {28, 1223, 2, 6152}, {28, 1237, 2, 6152}, {29, 1198, 1, 6152}, {29, 1238, 2, 6152}, {31, 1226, 1, 6152}, {32, 1227, 2, 6152}, {32, 1241, 1, 6152}, {33, 1228, 1, 6152}, {33, 1242, 2, 6152}, {34, 1243, 1, 6152}, {20, 1376, 1, 6153}, {21, 1377, 2, 6153}, {21, 1382, 1, 6153}, {22, 1378, 2, 6153}, {22, 1388, 1, 6153}, {23, 1379, 1, 6153}, {23, 1383, 2, 6153}, {24, 1380, 2, 6153}, {24, 1384, 2, 6153}, {24, 1389, 2, 6153}, {25, 1381, 1, 6153}, {25, 1390, 2, 6153}, {26, 1385, 1, 6153}, {27, 1386, 2, 6153}, {27, 1391, 1, 6153}, {28, 1387, 1, 6153}, {28, 1392, 2, 6153}, {29, 1393, 1, 6153}, {21, 1376, 1, 6154}, {23, 1377, 2, 6154}, {23, 1382, 1, 6154}, {24, 1378, 2, 6154}, {24, 1388, 1, 6154}, {26, 1379, 1, 6154}, {26, 1383, 2, 6154}, {27, 1380, 2, 6154}, {27, 1384, 2, 6154}, {27, 1389, 2, 6154}, {28, 1381, 1, 6154}, {28, 1390, 2, 6154}, {30, 1385, 1, 6154}, {31, 1386, 2, 6154}, {31, 1391, 1, 6154}, {32, 1387, 1, 6154}, {32, 1392, 2, 6154}, {33, 1393, 1, 6154}, {22, 1376, 1, 6155}, {24, 1377, 2, 6155}, {24, 1382, 1, 6155}, {25, 1378, 2, 6155}, {25, 1388, 1, 6155}, {27, 1379, 1, 6155}, {27, 1383, 2, 6155}, {28, 1380, 2, 6155}, {28, 1384, 2, 6155}, {28, 1389, 2, 6155}, {29, 1381, 1, 6155}, {29, 1390, 2, 6155}, {31, 1385, 1, 6155}, {32, 1386, 2, 6155}, {32, 1391, 1, 6155}, {33, 1387, 1, 6155}, {33, 1392, 2, 6155}, {34, 1393, 1, 6155}, {20, 1394, 1, 6156}, {21, 1395, 2, 6156}, {21, 1404, 1, 6156}, {22, 1396, 2, 6156}, {22, 1414, 1, 6156}, {23, 1397, 1, 6156}, {23, 1405, 2, 6156}, {24, 1398, 2, 6156}, {24, 1406, 2, 6156}, {24, 1415, 2, 6156}, {25, 1399, 1, 6156}, {25, 1416, 2, 6156}, {26, 1407, 1, 6156}, {27, 1408, 2, 6156}, {27, 1417, 1, 6156}, {28, 1409, 1, 6156}, {28, 1418, 2, 6156}, {29, 1419, 1, 6156}, {20, 1395, 1, 6157}, {21, 1397, 2, 6157}, {21, 1405, 1, 6157}, {22, 1398, 2, 6157}, {22, 1415, 1, 6157}, {23, 1400, 1, 6157}, {23, 1407, 2, 6157}, {24, 1401, 2, 6157}, {24, 1408, 2, 6157}, {24, 1417, 2, 6157}, {25, 1402, 1, 6157}, {25, 1418, 2,

6157}, {26, 1410, 1, 6157}, {27, 1411, 2, 6157}, {27, 1420, 1, 6157}, {28, 1412, 1, 6157}, {28, 1421, 2, 6157}, {29, 1422, 1, 6157}, {20, 1396, 1, 6158}, {21, 1398, 2, 6158}, {21, 1406, 1, 6158}, {22, 1399, 2, 6158}, {22, 1416, 1, 6158}, {23, 1401, 1, 6158}, {23, 1408, 2, 6158}, {24, 1402, 2, 6158}, {24, 1409, 2, 6158}, {24, 1418, 2, 6158}, {25, 1403, 1, 6158}, {25, 1419, 2, 6158}, {26, 1411, 1, 6158}, {27, 1412, 2, 6158}, {27, 1421, 1, 6158}, {28, 1413, 1, 6158}, {28, 1422, 2, 6158}, {29, 1423, 1, 6158}, {21, 1394, 1, 6159}, {23, 1395, 2, 6159}, {23, 1404, 1, 6159}, {24, 1396, 2, 6159}, {24, 1414, 1, 6159}, {26, 1397, 1, 6159}, {26, 1405, 2, 6159}, {27, 1398, 2, 6159}, {27, 1406, 2, 6159}, {27, 1415, 2, 6159}, {28, 1399, 1, 6159}, {28, 1416, 2, 6159}, {30, 1407, 1, 6159}, {31, 1408, 2, 6159}, {31, 1417, 1, 6159}, {32, 1409, 1, 6159}, {32, 1418, 2, 6159}, {33, 1419, 1, 6159}, {21, 1395, 1, 6160}, {23, 1397, 2, 6160}, {23, 1405, 1, 6160}, {24, 1398, 2, 6160}, {24, 1415, 1, 6160}, {26, 1400, 1, 6160}, {26, 1407, 2, 6160}, {27, 1401, 2, 6160}, {27, 1408, 2, 6160}, {27, 1417, 2, 6160}, {28, 1402, 1, 6160}, {28, 1418, 2, 6160}, {30, 1410, 1, 6160}, {31, 1411, 2, 6160}, {31, 1420, 1, 6160}, {32, 1412, 1, 6160}, {32, 1421, 2, 6160}, {33, 1422, 1, 6160}, {21, 1396, 1, 6161}, {23, 1398, 2, 6161}, {23, 1406, 1, 6161}, {24, 1399, 2, 6161}, {24, 1416, 1, 6161}, {26, 1401, 1, 6161}, {26, 1408, 2, 6161}, {27, 1402, 2, 6161}, {27, 1409, 2, 6161}, {27, 1418, 2, 6161}, {28, 1403, 1, 6161}, {28, 1419, 2, 6161}, {30, 1411, 1, 6161}, {31, 1412, 2, 6161}, {31, 1421, 1, 6161}, {32, 1413, 1, 6161}, {32, 1422, 2, 6161}, {33, 1423, 1, 6161}, {22, 1394, 1, 6162}, {24, 1395, 2, 6162}, {24, 1404, 1, 6162}, {25, 1396, 2, 6162}, {25, 1414, 1, 6162}, {27, 1397, 1, 6162}, {27, 1405, 2, 6162}, {28, 1398, 2, 6162}, {28, 1406, 2, 6162}, {28, 1415, 2, 6162}, {29, 1399, 1, 6162}, {29, 1416, 2, 6162}, {31, 1407, 1, 6162}, {32, 1408, 2, 6162}, {32, 1417, 1, 6162}, {33, 1409, 1, 6162}, {33, 1418, 2, 6162}, {34, 1419, 1, 6162}, {22, 1395, 1, 6163}, {24, 1397, 2, 6163}, {24, 1405, 1, 6163}, {25, 1398, 2, 6163}, {25, 1415, 1, 6163}, {27, 1400, 1, 6163}, {27, 1407, 2, 6163}, {28, 1401, 2, 6163}, {28, 1408, 2, 6163}, {28, 1417, 2, 6163}, {29, 1402, 1, 6163}, {29, 1418, 2, 6163}, {31, 1410, 1, 6163}, {32, 1411, 2, 6163}, {32, 1420, 1, 6163}, {33, 1412, 1, 6163}, {33, 1421, 2, 6163}, {34, 1422, 1, 6163}, {22, 1396, 1, 6164}, {24, 1398, 2, 6164}, {24, 1406, 1, 6164}, {25, 1399, 2, 6164}, {25, 1416, 1, 6164}, {27, 1401, 1, 6164}, {27, 1408, 2, 6164}, {28, 1402, 2, 6164}, {28, 1409, 2, 6164}, {28, 1418, 2, 6164}, {29, 1403, 1, 6164}, {29, 1419, 2, 6164}, {31, 1411, 1, 6164}, {32, 1412, 2, 6164}, {32, 1421, 1, 6164}, {33, 1413, 1, 6164}, {33, 1422, 2, 6164}, {34, 1423, 1, 6164}, {20, 1424, 1, 6165}, {21, 1425, 2, 6165}, {21, 1439, 1, 6165}, {22, 1426, 2, 6165}, {22, 1454, 1, 6165}, {23, 1427, 1, 6165}, {23, 1440, 2, 6165}, {24, 1428, 2, 6165}, {24, 1441, 2, 6165}, {24, 1455, 2, 6165}, {25, 1429, 1, 6165}, {25, 1456, 2, 6165}, {26, 1442, 1, 6165}, {27, 1443, 2, 6165}, {27, 1457, 1, 6165}, {28, 1444, 1, 6165}, {28, 1458, 2, 6165}, {29, 1459, 1, 6165}, {20, 1425, 1, 6166}, {21, 1427, 2, 6166}, {21, 1440, 1, 6166}, {22, 1428, 2, 6166}, {22, 1455, 1, 6166}, {23, 1430, 1, 6166}, {23, 1442, 2, 6166}, {24, 1431, 2, 6166}, {24, 1443, 2, 6166}, {24, 1457, 2, 6166}, {25, 1432, 1, 6166}, {25, 1458, 2, 6166}, {26, 1445, 1, 6166}, {27, 1446, 2, 6166}, {27, 1460, 1, 6166}, {28, 1447, 1, 6166}, {28, 1461, 2, 6166}, {29, 1462, 1, 6166}, {20, 1426, 1, 6167}, {21, 1428, 2, 6167}, {21, 1441, 1, 6167}, {22, 1429, 2, 6167}, {22, 1456, 1, 6167}, {23, 1431, 1, 6167}, {23, 1443, 2, 6167}, {24, 1432, 2, 6167}, {24, 1444, 2, 6167}, {24, 1458, 2, 6167}, {25, 1433, 1, 6167}, {25, 1459, 2, 6167}, {26, 1446, 1, 6167}, {27, 1447, 2, 6167}, {27, 1461, 1, 6167}, {28, 1448, 1, 6167}, {28, 1462, 2, 6167}, {29, 1463, 1, 6167}, {20, 1427, 1, 6168}, {21, 1430, 2, 6168}, {21, 1442, 1, 6168}, {22, 1431, 2, 6168}, {22, 1457, 1, 6168}, {23, 1434, 1, 6168}, {23, 1445, 2, 6168}, {24, 1435, 2, 6168}, {24, 1446, 2, 6168}, {24, 1460, 2, 6168}, {25, 1436, 1, 6168}, {25, 1461, 2, 6168}, {26, 1449, 1, 6168}, {27, 1450, 2, 6168}, {27, 1464, 1, 6168}, {28, 1451, 1, 6168}, {28, 1465, 2, 6168}, {29, 1466, 1, 6168}, {20, 1428, 1, 6169}, {21, 1431, 2, 6169}, {21, 1443, 1, 6169}, {22, 1432, 2, 6169}, {22, 1458, 1, 6169}, {23, 1435, 1, 6169}, {23, 1446, 2, 6169}, {24, 1436, 2, 6169}, {24, 1447, 2, 6169}, {24, 1461, 2, 6169}, {25, 1437, 1, 6169}, {25, 1462, 2, 6169}, {26, 1450, 1, 6169}, {27, 1451, 2, 6169}, {27, 1465, 1, 6169}, {28, 1452, 1, 6169}, {28, 1466, 2, 6169}, {29, 1467, 1, 6169}, {20, 1429, 1, 6170}, {21, 1432, 2, 6170}, {21, 1444, 1, 6170}, {22, 1433, 2, 6170}, {22, 1459, 1, 6170}, {23, 1436, 1, 6170}, {23, 1447, 2, 6170}, {24, 1437, 2, 6170}, {24, 1448, 2, 6170}, {24, 1462, 2, 6170}, {25, 1438, 1, 6170}, {25, 1463, 2, 6170}, {26, 1451, 1, 6170}, {27, 1452, 2, 6170}, {27, 1466, 1, 6170}, {28, 1453, 1, 6170}, {28, 1467, 2, 6170}, {29, 1468, 1, 6170}, {21, 1424, 1, 6171}, {23, 1425, 2, 6171}, {23, 1439, 1, 6171}, {24, 1426, 2, 6171}, {24, 1454, 1, 6171}, {26, 1427, 1, 6171}, {26, 1440, 2, 6171}, {27, 1428, 2, 6171}, {27, 1441, 2, 6171}, {27, 1455, 2, 6171}, {28, 1429, 1, 6171}, {28, 1456, 2, 6171}, {30, 1442, 1, 6171}, {31, 1443, 2, 6171}, {31, 1457, 1, 6171}, {32, 1444, 1, 6171}, {32, 1458, 2, 6171}, {33, 1459, 1, 6171}, {21, 1425, 1, 6172}, {23, 1427, 2, 6172}, {23, 1440, 1, 6172}, {24, 1428, 2, 6172}, {24, 1455, 1, 6172}, {26, 1430, 1, 6172}, {26, 1442, 2, 6172}, {27, 1431, 2, 6172}, {27, 1443, 2, 6172}, {27, 1457, 2, 6172}, {28, 1432, 1, 6172}, {28, 1458, 2, 6172}, {30, 1445, 1, 6172}, {31, 1446, 2, 6172}, {31, 1460, 1, 6172}, {32, 1447, 1, 6172}, {32, 1461, 2, 6172}, {33, 1462, 1, 6172}, {21, 1426, 1, 6173}, {23, 1428, 2, 6173}, {23, 1441, 1, 6173}, {24, 1429, 2, 6173}, {24, 1456, 1, 6173}, {26, 1431, 1, 6173}, {26, 1443, 2, 6173}, {27, 1432, 2, 6173}, {27, 1444, 2, 6173}, {27, 1458, 2, 6173}, {28, 1433, 1, 6173}, {28, 1459, 2, 6173}, {30, 1446, 1, 6173}, {31, 1447, 2, 6173}, {31, 1461, 1, 6173}, {32, 1448, 1, 6173}, {32, 1462, 2, 6173},

{33, 1463, 1, 6173}, {21, 1427, 1, 6174}, {23, 1430, 2, 6174}, {23, 1442, 1, 6174}, {24, 1431, 2, 6174}, {24, 1457, 1, 6174}, {26, 1434, 1, 6174}, {26, 1445, 2, 6174}, {27, 1435, 2, 6174}, {27, 1446, 2, 6174}, {27, 1460, 2, 6174}, {28, 1436, 1, 6174}, {28, 1461, 2, 6174}, {30, 1449, 1, 6174}, {31, 1450, 2, 6174}, {31, 1464, 1, 6174}, {32, 1451, 1, 6174}, {32, 1465, 2, 6174}, {33, 1466, 1, 6174}, {21, 1428, 1, 6175}, {23, 1431, 2, 6175}, {23, 1443, 1, 6175}, {24, 1432, 2, 6175}, {24, 1458, 1, 6175}, {26, 1435, 1, 6175}, {26, 1446, 2, 6175}, {27, 1436, 2, 6175}, {27, 1447, 2, 6175}, {27, 1461, 2, 6175}, {28, 1437, 1, 6175}, {28, 1462, 2, 6175}, {30, 1450, 1, 6175}, {31, 1451, 2, 6175}, {31, 1465, 1, 6175}, {32, 1452, 1, 6175}, {32, 1466, 2, 6175}, {33, 1467, 1, 6175}, {21, 1429, 1, 6176}, {23, 1432, 2, 6176}, {23, 1444, 1, 6176}, {24, 1433, 2, 6176}, {24, 1459, 1, 6176}, {26, 1436, 1, 6176}, {26, 1447, 2, 6176}, {27, 1437, 2, 6176}, {27, 1448, 2, 6176}, {27, 1462, 2, 6176}, {28, 1438, 1, 6176}, {28, 1463, 2, 6176}, {30, 1451, 1, 6176}, {31, 1452, 2, 6176}, {31, 1466, 1, 6176}, {32, 1453, 1, 6176}, {32, 1467, 2, 6176}, {33, 1468, 1, 6176}, {22, 1424, 1, 6177}, {24, 1425, 2, 6177}, {24, 1439, 1, 6177}, {25, 1426, 2, 6177}, {25, 1454, 1, 6177}, {27, 1427, 1, 6177}, {27, 1440, 2, 6177}, {28, 1428, 2, 6177}, {28, 1441, 2, 6177}, {28, 1455, 2, 6177}, {29, 1429, 1, 6177}, {29, 1456, 2, 6177}, {31, 1442, 1, 6177}, {32, 1443, 2, 6177}, {32, 1457, 1, 6177}, {33, 1444, 1, 6177}, {33, 1458, 2, 6177}, {34, 1459, 1, 6177}, {22, 1425, 1, 6178}, {24, 1427, 2, 6178}, {24, 1440, 1, 6178}, {25, 1428, 2, 6178}, {25, 1455, 1, 6178}, {27, 1430, 1, 6178}, {27, 1442, 2, 6178}, {28, 1431, 2, 6178}, {28, 1443, 2, 6178}, {28, 1457, 2, 6178}, {29, 1432, 1, 6178}, {29, 1458, 2, 6178}, {31, 1445, 1, 6178}, {32, 1446, 2, 6178}, {32, 1460, 1, 6178}, {33, 1447, 1, 6178}, {33, 1461, 2, 6178}, {34, 1462, 1, 6178}, {22, 1426, 1, 6179}, {24, 1428, 2, 6179}, {24, 1441, 1, 6179}, {25, 1429, 2, 6179}, {25, 1456, 1, 6179}, {27, 1431, 1, 6179}, {27, 1443, 2, 6179}, {28, 1432, 2, 6179}, {28, 1444, 2, 6179}, {28, 1458, 2, 6179}, {29, 1433, 1, 6179}, {29, 1459, 2, 6179}, {31, 1446, 1, 6179}, {32, 1447, 2, 6179}, {32, 1461, 1, 6179}, {33, 1448, 1, 6179}, {33, 1462, 2, 6179}, {34, 1463, 1, 6179}, {22, 1427, 1, 6180}, {24, 1430, 2, 6180}, {24, 1442, 1, 6180}, {25, 1431, 2, 6180}, {25, 1457, 1, 6180}, {27, 1434, 1, 6180}, {27, 1445, 2, 6180}, {28, 1435, 2, 6180}, {28, 1446, 2, 6180}, {28, 1460, 2, 6180}, {29, 1436, 1, 6180}, {29, 1461, 2, 6180}, {31, 1449, 1, 6180}, {32, 1450, 2, 6180}, {32, 1464, 1, 6180}, {33, 1451, 1, 6180}, {33, 1465, 2, 6180}, {34, 1466, 1, 6180}, {22, 1428, 1, 6181}, {24, 1431, 2, 6181}, {24, 1443, 1, 6181}, {25, 1432, 2, 6181}, {25, 1458, 1, 6181}, {27, 1435, 1, 6181}, {27, 1446, 2, 6181}, {28, 1436, 2, 6181}, {28, 1447, 2, 6181}, {28, 1461, 2, 6181}, {29, 1437, 1, 6181}, {29, 1462, 2, 6181}, {31, 1450, 1, 6181}, {32, 1451, 2, 6181}, {32, 1465, 1, 6181}, {33, 1452, 1, 6181}, {33, 1466, 2, 6181}, {34, 1467, 1, 6181}, {22, 1429, 1, 6182}, {24, 1432, 2, 6182}, {24, 1444, 1, 6182}, {25, 1433, 2, 6182}, {25, 1459, 1, 6182}, {27, 1436, 1, 6182}, {27, 1447, 2, 6182}, {28, 1437, 2, 6182}, {28, 1448, 2, 6182}, {28, 1462, 2, 6182}, {29, 1438, 1, 6182}, {29, 1463, 2, 6182}, {31, 1451, 1, 6182}, {32, 1452, 2, 6182}, {32, 1466, 1, 6182}, {33, 1453, 1, 6182}, {33, 1467, 2, 6182}, {34, 1468, 1, 6182}, {20, 1094, 1, 6183}, {21, 1095, 1, 6183}, {21, 1104, 2, 6183}, {22, 1096, 1, 6183}, {22, 1114, 2, 6183}, {23, 1105, 2, 6183}, {23, 1124, 1, 6183}, {24, 1106, 2, 6183}, {24, 1115, 2, 6183}, {24, 1134, 2, 6183}, {25, 1116, 2, 6183}, {25, 1144, 1, 6183}, {26, 1125, 1, 6183}, {27, 1126, 1, 6183}, {27, 1135, 2, 6183}, {28, 1136, 2, 6183}, {28, 1145, 1, 6183}, {29, 1146, 1, 6183}, {20, 1095, 1, 6184}, {21, 1097, 1, 6184}, {21, 1105, 2, 6184}, {22, 1098, 1, 6184}, {22, 1115, 2, 6184}, {23, 1107, 2, 6184}, {23, 1125, 1, 6184}, {24, 1108, 2, 6184}, {24, 1117, 2, 6184}, {24, 1135, 2, 6184}, {25, 1118, 2, 6184}, {25, 1145, 1, 6184}, {26, 1127, 1, 6184}, {27, 1128, 1, 6184}, {27, 1137, 2, 6184}, {28, 1138, 2, 6184}, {28, 1147, 1, 6184}, {29, 1148, 1, 6184}, {20, 1096, 1, 6185}, {21, 1098, 1, 6185}, {21, 1106, 2, 6185}, {22, 1099, 1, 6185}, {22, 1116, 2, 6185}, {23, 1108, 2, 6185}, {23, 1126, 1, 6185}, {24, 1109, 2, 6185}, {24, 1118, 2, 6185}, {24, 1136, 2, 6185}, {25, 1119, 2, 6185}, {25, 1146, 1, 6185}, {26, 1128, 1, 6185}, {27, 1129, 1, 6185}, {27, 1138, 2, 6185}, {28, 1139, 2, 6185}, {28, 1148, 1, 6185}, {29, 1149, 1, 6185}, {20, 1097, 1, 6186}, {21, 1100, 1, 6186}, {21, 1107, 2, 6186}, {22, 1101, 1, 6186}, {22, 1117, 2, 6186}, {23, 1110, 2, 6186}, {23, 1127, 1, 6186}, {24, 1111, 2, 6186}, {24, 1120, 2, 6186}, {24, 1137, 2, 6186}, {25, 1121, 2, 6186}, {25, 1147, 1, 6186}, {26, 1130, 1, 6186}, {27, 1131, 1, 6186}, {27, 1140, 2, 6186}, {28, 1141, 2, 6186}, {28, 1150, 1, 6186}, {29, 1151, 1, 6186}, {20, 1098, 1, 6187}, {21, 1101, 1, 6187}, {21, 1108, 2, 6187}, {22, 1102, 1, 6187}, {22, 1118, 2, 6187}, {23, 1111, 2, 6187}, {23, 1128, 1, 6187}, {24, 1112, 2, 6187}, {24, 1121, 2, 6187}, {24, 1138, 2, 6187}, {25, 1122, 2, 6187}, {25, 1148, 1, 6187}, {26, 1131, 1, 6187}, {27, 1132, 1, 6187}, {27, 1141, 2, 6187}, {28, 1142, 2, 6187}, {28, 1151, 1, 6187}, {29, 1152, 1, 6187}, {20, 1099, 1, 6188}, {21, 1102, 1, 6188}, {21, 1109, 2, 6188}, {22, 1103, 1, 6188}, {22, 1119, 2, 6188}, {23, 1112, 2, 6188}, {23, 1129, 1, 6188}, {24, 1113, 2, 6188}, {24, 1122, 2, 6188}, {24, 1139, 2, 6188}, {25, 1123, 2, 6188}, {25, 1149, 1, 6188}, {26, 1132, 1, 6188}, {27, 1133, 1, 6188}, {27, 1142, 2, 6188}, {28, 1143, 2, 6188}, {28, 1152, 1, 6188}, {29, 1153, 1, 6188}, {21, 1094, 1, 6189}, {23, 1095, 1, 6189}, {23, 1104, 2, 6189}, {24, 1096, 1, 6189}, {24, 1114, 2, 6189}, {26, 1105, 2, 6189}, {26, 1124, 1, 6189}, {27, 1106, 2, 6189}, {27, 1115, 2, 6189}, {27, 1134, 2, 6189}, {28, 1116, 2, 6189}, {28, 1144, 1, 6189}, {30, 1125, 1, 6189}, {31, 1126, 1, 6189}, {31, 1135, 2, 6189}, {32, 1136, 2, 6189}, {32, 1145, 1, 6189}, {33, 1146, 1, 6189}, {21, 1095, 1, 6190}, {23, 1097, 1, 6190}, {23, 1105, 2, 6190}, {24, 1098, 1, 6190}, {24,

1115, 2, 6190}, {26, 1107, 2, 6190}, {26, 1125, 1, 6190}, {27, 1108, 2, 6190}, {27, 1117,
2, 6190}, {27, 1135, 2, 6190}, {28, 1118, 2, 6190}, {28, 1145, 1, 6190}, {30, 1127, 1,
6190}, {31, 1128, 1, 6190}, {31, 1137, 2, 6190}, {32, 1138, 2, 6190}, {32, 1147, 1, 6190},
{33, 1148, 1, 6190}, {21, 1096, 1, 6191}, {23, 1098, 1, 6191}, {23, 1106, 2, 6191}, {24,
1099, 1, 6191}, {24, 1116, 2, 6191}, {26, 1108, 2, 6191}, {26, 1126, 1, 6191}, {27, 1109,
2, 6191}, {27, 1118, 2, 6191}, {27, 1136, 2, 6191}, {28, 1119, 2, 6191}, {28, 1146, 1,
6191}, {30, 1128, 1, 6191}, {31, 1129, 1, 6191}, {31, 1138, 2, 6191}, {32, 1139, 2, 6191},
{32, 1148, 1, 6191}, {33, 1149, 1, 6191}, {21, 1097, 1, 6192}, {23, 1100, 1, 6192}, {23,
1107, 2, 6192}, {24, 1101, 1, 6192}, {24, 1117, 2, 6192}, {26, 1110, 2, 6192}, {26, 1127,
1, 6192}, {27, 1111, 2, 6192}, {27, 1120, 2, 6192}, {27, 1137, 2, 6192}, {28, 1121, 2,
6192}, {28, 1147, 1, 6192}, {30, 1130, 1, 6192}, {31, 1131, 1, 6192}, {31, 1140, 2, 6192},
{32, 1141, 2, 6192}, {32, 1150, 1, 6192}, {33, 1151, 1, 6192}, {21, 1098, 1, 6193}, {23,
1101, 1, 6193}, {23, 1108, 2, 6193}, {24, 1102, 1, 6193}, {24, 1118, 2, 6193}, {26, 1111,
2, 6193}, {26, 1128, 1, 6193}, {27, 1112, 2, 6193}, {27, 1121, 2, 6193}, {27, 1138, 2,
6193}, {28, 1122, 2, 6193}, {28, 1148, 1, 6193}, {30, 1131, 1, 6193}, {31, 1132, 1, 6193},
{31, 1141, 2, 6193}, {32, 1142, 2, 6193}, {32, 1151, 1, 6193}, {33, 1152, 1, 6193}, {21,
1099, 1, 6194}, {23, 1102, 1, 6194}, {23, 1109, 2, 6194}, {24, 1103, 1, 6194}, {24, 1119,
2, 6194}, {26, 1112, 2, 6194}, {26, 1129, 1, 6194}, {27, 1113, 2, 6194}, {27, 1122, 2,
6194}, {27, 1139, 2, 6194}, {28, 1123, 2, 6194}, {28, 1149, 1, 6194}, {30, 1132, 1, 6194},
{31, 1133, 1, 6194}, {31, 1142, 2, 6194}, {32, 1143, 2, 6194}, {32, 1152, 1, 6194}, {33,
1153, 1, 6194}, {22, 1094, 1, 6195}, {24, 1095, 1, 6195}, {24, 1104, 2, 6195}, {25, 1096,
1, 6195}, {25, 1114, 2, 6195}, {27, 1105, 2, 6195}, {27, 1124, 1, 6195}, {28, 1106, 2,
6195}, {28, 1115, 2, 6195}, {28, 1134, 2, 6195}, {29, 1116, 2, 6195}, {29, 1144, 1, 6195},
{31, 1125, 1, 6195}, {32, 1126, 1, 6195}, {32, 1135, 2, 6195}, {33, 1136, 2, 6195}, {33,
1145, 1, 6195}, {34, 1146, 1, 6195}, {22, 1095, 1, 6196}, {24, 1097, 1, 6196}, {24, 1105,
2, 6196}, {25, 1098, 1, 6196}, {25, 1115, 2, 6196}, {27, 1107, 2, 6196}, {27, 1125, 1,
6196}, {28, 1108, 2, 6196}, {28, 1117, 2, 6196}, {28, 1135, 2, 6196}, {29, 1118, 2, 6196},
{29, 1145, 1, 6196}, {31, 1127, 1, 6196}, {32, 1128, 1, 6196}, {32, 1137, 2, 6196}, {33,
1138, 2, 6196}, {33, 1147, 1, 6196}, {34, 1148, 1, 6196}, {22, 1096, 1, 6197}, {24, 1098,
1, 6197}, {24, 1106, 2, 6197}, {25, 1099, 1, 6197}, {25, 1116, 2, 6197}, {27, 1108, 2,
6197}, {27, 1126, 1, 6197}, {28, 1109, 2, 6197}, {28, 1118, 2, 6197}, {28, 1136, 2, 6197},
{29, 1119, 2, 6197}, {29, 1146, 1, 6197}, {31, 1128, 1, 6197}, {32, 1129, 1, 6197}, {32,
1138, 2, 6197}, {33, 1139, 2, 6197}, {33, 1148, 1, 6197}, {34, 1149, 1, 6197}, {22, 1097,
1, 6198}, {24, 1100, 1, 6198}, {24, 1107, 2, 6198}, {25, 1101, 1, 6198}, {25, 1117, 2,
6198}, {27, 1110, 2, 6198}, {27, 1127, 1, 6198}, {28, 1111, 2, 6198}, {28, 1120, 2, 6198},
{28, 1137, 2, 6198}, {29, 1121, 2, 6198}, {29, 1147, 1, 6198}, {31, 1130, 1, 6198}, {32,
1131, 1, 6198}, {32, 1140, 2, 6198}, {33, 1141, 2, 6198}, {33, 1150, 1, 6198}, {34, 1151,
1, 6198}, {22, 1098, 1, 6199}, {24, 1101, 1, 6199}, {24, 1108, 2, 6199}, {25, 1102, 1,
6199}, {25, 1118, 2, 6199}, {27, 1111, 2, 6199}, {27, 1128, 1, 6199}, {28, 1112, 2, 6199},
{28, 1121, 2, 6199}, {28, 1138, 2, 6199}, {29, 1122, 2, 6199}, {29, 1148, 1, 6199}, {31,
1131, 1, 6199}, {32, 1132, 1, 6199}, {32, 1141, 2, 6199}, {33, 1142, 2, 6199}, {33, 1151,
1, 6199}, {34, 1152, 1, 6199}, {22, 1099, 1, 6200}, {24, 1102, 1, 6200}, {24, 1109, 2,
6200}, {25, 1103, 1, 6200}, {25, 1119, 2, 6200}, {27, 1112, 2, 6200}, {27, 1129, 1, 6200},
{28, 1113, 2, 6200}, {28, 1122, 2, 6200}, {28, 1139, 2, 6200}, {29, 1123, 2, 6200}, {29,
1149, 1, 6200}, {31, 1132, 1, 6200}, {32, 1133, 1, 6200}, {32, 1142, 2, 6200}, {33, 1143,
2, 6200}, {33, 1152, 1, 6200}, {34, 1153, 1, 6200}, {20, 1588, 1, 6201}, {21, 1589, 3,
6201}, {22, 1590, 3, 6201}, {23, 1591, 1, 6201}, {23, 1598, 2, 6201}, {24, 1592, 2, 6201},
{24, 1599, 4, 6201}, {25, 1593, 1, 6201}, {25, 1607, 2, 6201}, {26, 1600, 1, 6201}, {27,
1601, 2, 6201}, {27, 1608, 1, 6201}, {28, 1602, 1, 6201}, {28, 1609, 2, 6201}, {29, 1610,
1, 6201}, {20, 1589, 1, 6202}, {21, 1591, 2, 6202}, {21, 1598, 1, 6202}, {22, 1592, 2,
6202}, {22, 1599, 1, 6202}, {23, 1594, 1, 6202}, {23, 1600, 2, 6202}, {24, 1595, 2, 6202},
{24, 1601, 2, 6202}, {24, 1608, 2, 6202}, {25, 1596, 1, 6202}, {25, 1609, 2, 6202}, {26,
1603, 1, 6202}, {27, 1604, 2, 6202}, {27, 1611, 1, 6202}, {28, 1605, 1, 6202}, {28, 1612,
2, 6202}, {29, 1613, 1, 6202}, {20, 1590, 1, 6203}, {21, 1592, 2, 6203}, {21, 1599, 1,
6203}, {22, 1593, 2, 6203}, {22, 1607, 1, 6203}, {23, 1595, 1, 6203}, {23, 1601, 2, 6203},
{24, 1596, 2, 6203}, {24, 1602, 2, 6203}, {24, 1609, 2, 6203}, {25, 1597, 1, 6203}, {25,
1610, 2, 6203}, {26, 1604, 1, 6203}, {27, 1605, 2, 6203}, {27, 1612, 1, 6203}, {28, 1606,
1, 6203}, {28, 1613, 2, 6203}, {29, 1614, 1, 6203}, {20, 1589, 1, 6204}, {21, 1591, 1,
6204}, {21, 1598, 2, 6204}, {22, 1592, 1, 6204}, {22, 1599, 2, 6204}, {23, 1600, 3, 6204},
{24, 1601, 4, 6204}, {24, 1608, 2, 6204}, {25, 1602, 1, 6204}, {25, 1609, 2, 6204}, {26,
1615, 1, 6204}, {27, 1616, 3, 6204}, {28, 1617, 1, 6204}, {28, 1622, 2, 6204}, {29, 1623,
1, 6204}, {20, 1598, 1, 6205}, {21, 1600, 3, 6205}, {22, 1601, 3, 6205}, {23, 1603, 1,
6205}, {23, 1615, 2, 6205}, {24, 1604, 2, 6205}, {24, 1616, 4, 6205}, {25, 1605, 1, 6205},
{25, 1622, 2, 6205}, {26, 1618, 1, 6205}, {27, 1619, 2, 6205}, {27, 1624, 1, 6205}, {28,
1620, 1, 6205}, {28, 1625, 2, 6205}, {29, 1626, 1, 6205}, {20, 1599, 1, 6206}, {21, 1601,
2, 6206}, {21, 1608, 1, 6206}, {22, 1602, 2, 6206}, {22, 1609, 1, 6206}, {23, 1604, 1,
6206}, {23, 1616, 2, 6206}, {24, 1605, 2, 6206}, {24, 1617, 2, 6206}, {24, 1622, 2, 6206},
{25, 1606, 1, 6206}, {25, 1623, 2, 6206}, {26, 1619, 1, 6206}, {27, 1620, 2, 6206}, {27,
1625, 1, 6206}, {28, 1621, 1, 6206}, {28, 1626, 2, 6206}, {29, 1627, 1, 6206}, {20, 1590,

1, 6207}, {21, 1592, 1, 6207}, {21, 1599, 2, 6207}, {22, 1593, 1, 6207}, {22, 1607, 2, 6207}, {23, 1601, 2, 6207}, {23, 1608, 1, 6207}, {24, 1602, 2, 6207}, {24, 1609, 4, 6207}, {25, 1610, 3, 6207}, {26, 1616, 1, 6207}, {27, 1617, 1, 6207}, {27, 1622, 2, 6207}, {28, 1623, 3, 6207}, {29, 1628, 1, 6207}, {20, 1599, 1, 6208}, {21, 1601, 1, 6208}, {21, 1608, 2, 6208}, {22, 1602, 1, 6208}, {22, 1609, 2, 6208}, {23, 1611, 1, 6208}, {23, 1616, 2, 6208}, {24, 1612, 2, 6208}, {24, 1617, 2, 6208}, {24, 1622, 2, 6208}, {25, 1613, 1, 6208}, {25, 1623, 2, 6208}, {26, 1624, 1, 6208}, {27, 1625, 2, 6208}, {27, 1629, 1, 6208}, {28, 1626, 1, 6208}, {28, 1630, 2, 6208}, {29, 1631, 1, 6208}, {20, 1607, 1, 6209}, {21, 1609, 3, 6209}, {22, 1610, 3, 6209}, {23, 1612, 1, 6209}, {23, 1622, 2, 6209}, {24, 1613, 2, 6209}, {24, 1623, 4, 6209}, {25, 1614, 1, 6209}, {25, 1628, 2, 6209}, {26, 1625, 1, 6209}, {27, 1626, 2, 6209}, {27, 1630, 1, 6209}, {28, 1627, 1, 6209}, {28, 1631, 2, 6209}, {29, 1632, 1, 6209}, {20, 1591, 1, 6210}, {21, 1594, 1, 6210}, {21, 1600, 2, 6210}, {22, 1595, 1, 6210}, {22, 1608, 2, 6210}, {23, 1603, 2, 6210}, {23, 1615, 1, 6210}, {24, 1604, 2, 6210}, {24, 1611, 2, 6210}, {24, 1616, 2, 6210}, {25, 1612, 2, 6210}, {25, 1617, 1, 6210}, {26, 1618, 1, 6210}, {27, 1619, 1, 6210}, {27, 1624, 2, 6210}, {28, 1625, 2, 6210}, {28, 1629, 1, 6210}, {29, 1630, 1, 6210}, {20, 1600, 1, 6211}, {21, 1603, 1, 6211}, {21, 1615, 2, 6211}, {22, 1604, 1, 6211}, {22, 1616, 2, 6211}, {23, 1618, 3, 6211}, {24, 1619, 4, 6211}, {24, 1624, 2, 6211}, {25, 1620, 1, 6211}, {25, 1625, 2, 6211}, {26, 1633, 1, 6211}, {27, 1634, 3, 6211}, {28, 1635, 1, 6211}, {28, 1637, 2, 6211}, {29, 1638, 1, 6211}, {20, 1608, 1, 6212}, {21, 1611, 1, 6212}, {21, 1616, 2, 6212}, {22, 1612, 1, 6212}, {22, 1617, 2, 6212}, {23, 1619, 1, 6212}, {23, 1624, 2, 6212}, {24, 1620, 2, 6212}, {24, 1625, 2, 6212}, {24, 1629, 2, 6212}, {25, 1621, 1, 6212}, {25, 1630, 2, 6212}, {26, 1634, 1, 6212}, {27, 1635, 2, 6212}, {27, 1637, 1, 6212}, {28, 1636, 1, 6212}, {28, 1638, 2, 6212}, {29, 1639, 1, 6212}, {20, 1592, 1, 6213}, {21, 1595, 1, 6213}, {21, 1601, 2, 6213}, {22, 1596, 1, 6213}, {22, 1609, 2, 6213}, {23, 1604, 2, 6213}, {23, 1616, 1, 6213}, {24, 1605, 2, 6213}, {24, 1612, 2, 6213}, {24, 1622, 2, 6213}, {25, 1613, 2, 6213}, {25, 1623, 1, 6213}, {26, 1619, 1, 6213}, {27, 1620, 1, 6213}, {27, 1625, 2, 6213}, {28, 1626, 2, 6213}, {28, 1630, 1, 6213}, {29, 1631, 1, 6213}, {20, 1601, 1, 6214}, {21, 1604, 1, 6214}, {21, 1616, 2, 6214}, {22, 1605, 1, 6214}, {22, 1622, 2, 6214}, {23, 1619, 2, 6214}, {23, 1624, 1, 6214}, {24, 1620, 2, 6214}, {24, 1625, 4, 6214}, {25, 1626, 3, 6214}, {26, 1634, 1, 6214}, {27, 1635, 1, 6214}, {27, 1637, 2, 6214}, {28, 1638, 3, 6214}, {29, 1640, 1, 6214}, {20, 1609, 1, 6215}, {21, 1612, 1, 6215}, {21, 1622, 2, 6215}, {22, 1613, 1, 6215}, {22, 1623, 2, 6215}, {23, 1625, 3, 6215}, {24, 1626, 4, 6215}, {24, 1630, 2, 6215}, {25, 1627, 1, 6215}, {25, 1631, 2, 6215}, {26, 1637, 1, 6215}, {27, 1638, 3, 6215}, {28, 1639, 1, 6215}, {28, 1640, 2, 6215}, {29, 1641, 1, 6215}, {20, 1593, 1, 6216}, {21, 1596, 1, 6216}, {21, 1602, 2, 6216}, {22, 1597, 1, 6216}, {22, 1610, 2, 6216}, {23, 1605, 2, 6216}, {23, 1617, 1, 6216}, {24, 1606, 2, 6216}, {24, 1613, 2, 6216}, {24, 1623, 2, 6216}, {25, 1614, 2, 6216}, {25, 1628, 1, 6216}, {26, 1620, 1, 6216}, {27, 1621, 1, 6216}, {27, 1626, 2, 6216}, {28, 1627, 2, 6216}, {28, 1631, 1, 6216}, {29, 1632, 1, 6216}, {20, 1602, 1, 6217}, {21, 1605, 1, 6217}, {21, 1617, 2, 6217}, {22, 1606, 1, 6217}, {22, 1623, 2, 6217}, {23, 1620, 2, 6217}, {23, 1629, 1, 6217}, {24, 1621, 2, 6217}, {24, 1626, 2, 6217}, {24, 1630, 2, 6217}, {25, 1627, 2, 6217}, {25, 1631, 1, 6217}, {26, 1635, 1, 6217}, {27, 1636, 1, 6217}, {27, 1638, 2, 6217}, {28, 1639, 2, 6217}, {28, 1640, 1, 6217}, {29, 1641, 1, 6217}, {20, 1610, 1, 6218}, {21, 1613, 1, 6218}, {21, 1623, 2, 6218}, {22, 1614, 1, 6218}, {22, 1628, 2, 6218}, {23, 1626, 2, 6218}, {23, 1630, 1, 6218}, {24, 1627, 2, 6218}, {24, 1631, 4, 6218}, {25, 1632, 3, 6218}, {26, 1638, 1, 6218}, {27, 1639, 1, 6218}, {27, 1640, 2, 6218}, {28, 1641, 3, 6218}, {29, 1642, 1, 6218}, {21, 1588, 1, 6219}, {23, 1589, 3, 6219}, {24, 1590, 3, 6219}, {26, 1591, 1, 6219}, {26, 1598, 2, 6219}, {27, 1592, 2, 6219}, {27, 1599, 4, 6219}, {28, 1593, 1, 6219}, {28, 1607, 2, 6219}, {30, 1600, 1, 6219}, {31, 1601, 2, 6219}, {31, 1608, 1, 6219}, {32, 1602, 1, 6219}, {32, 1609, 2, 6219}, {33, 1610, 1, 6219}, {21, 1589, 1, 6220}, {23, 1591, 2, 6220}, {23, 1598, 1, 6220}, {24, 1592, 2, 6220}, {24, 1599, 1, 6220}, {26, 1594, 1, 6220}, {26, 1600, 2, 6220}, {27, 1595, 2, 6220}, {27, 1601, 2, 6220}, {27, 1608, 2, 6220}, {28, 1596, 1, 6220}, {28, 1609, 2, 6220}, {30, 1603, 1, 6220}, {31, 1604, 2, 6220}, {31, 1611, 1, 6220}, {32, 1605, 1, 6220}, {32, 1612, 2, 6220}, {33, 1613, 1, 6220}, {21, 1590, 1, 6221}, {23, 1592, 2, 6221}, {23, 1599, 1, 6221}, {24, 1593, 2, 6221}, {24, 1607, 1, 6221}, {26, 1595, 1, 6221}, {26, 1601, 2, 6221}, {27, 1596, 2, 6221}, {27, 1602, 2, 6221}, {27, 1609, 2, 6221}, {28, 1597, 1, 6221}, {28, 1610, 2, 6221}, {30, 1604, 1, 6221}, {31, 1605, 2, 6221}, {31, 1612, 1, 6221}, {32, 1606, 1, 6221}, {32, 1613, 2, 6221}, {33, 1614, 1, 6221}, {21, 1589, 1, 6222}, {23, 1591, 1, 6222}, {23, 1598, 2, 6222}, {24, 1592, 1, 6222}, {24, 1599, 2, 6222}, {26, 1600, 3, 6222}, {27, 1601, 4, 6222}, {27, 1608, 2, 6222}, {28, 1602, 1, 6222}, {28, 1609, 2, 6222}, {30, 1615, 1, 6222}, {31, 1616, 3, 6222}, {32, 1617, 1, 6222}, {32, 1622, 2, 6222}, {33, 1623, 1, 6222}, {21, 1598, 1, 6223}, {23, 1600, 3, 6223}, {24, 1601, 3, 6223}, {26, 1603, 1, 6223}, {26, 1615, 2, 6223}, {27, 1604, 2, 6223}, {27, 1616, 4, 6223}, {28, 1605, 1, 6223}, {28, 1622, 2, 6223}, {30, 1618, 1, 6223}, {31, 1619, 2, 6223}, {31, 1624, 1, 6223}, {32, 1620, 1, 6223}, {32, 1625, 2, 6223}, {33, 1626, 1, 6223}, {21, 1599, 1, 6224}, {23, 1601, 2, 6224}, {23, 1608, 1, 6224}, {24, 1602, 2, 6224}, {24, 1609, 1, 6224}, {26, 1604, 1, 6224}, {26, 1616, 2, 6224}, {27, 1605, 2, 6224}, {27, 1617, 2, 6224}, {27, 1622, 2, 6224}, {28, 1606, 1, 6224}, {28, 1623, 2, 6224}, {30, 1619, 1, 6224}, {31, 1620, 2, 6224}, {31, 1625, 1,

6224}, {32, 1621, 1, 6224}, {32, 1626, 2, 6224}, {33, 1627, 1, 6224}, {21, 1590, 1, 6225}, {23, 1592, 1, 6225}, {23, 1599, 2, 6225}, {24, 1593, 1, 6225}, {24, 1607, 2, 6225}, {26, 1601, 2, 6225}, {26, 1608, 1, 6225}, {27, 1602, 2, 6225}, {27, 1609, 4, 6225}, {28, 1610, 3, 6225}, {30, 1616, 1, 6225}, {31, 1617, 1, 6225}, {31, 1622, 2, 6225}, {32, 1623, 3, 6225}, {33, 1628, 1, 6225}, {21, 1599, 1, 6226}, {23, 1601, 1, 6226}, {23, 1608, 2, 6226}, {24, 1602, 1, 6226}, {24, 1609, 2, 6226}, {26, 1611, 1, 6226}, {26, 1616, 2, 6226}, {27, 1612, 2, 6226}, {27, 1617, 2, 6226}, {27, 1622, 2, 6226}, {28, 1613, 1, 6226}, {28, 1623, 2, 6226}, {30, 1624, 1, 6226}, {31, 1625, 2, 6226}, {31, 1629, 1, 6226}, {32, 1626, 1, 6226}, {32, 1630, 2, 6226}, {33, 1631, 1, 6226}, {21, 1607, 1, 6227}, {23, 1609, 3, 6227}, {24, 1610, 3, 6227}, {26, 1612, 1, 6227}, {26, 1622, 2, 6227}, {27, 1613, 2, 6227}, {27, 1623, 4, 6227}, {28, 1614, 1, 6227}, {28, 1628, 2, 6227}, {30, 1625, 1, 6227}, {31, 1626, 2, 6227}, {31, 1630, 1, 6227}, {32, 1627, 1, 6227}, {32, 1631, 2, 6227}, {33, 1632, 1, 6227}, {21, 1591, 1, 6228}, {23, 1594, 1, 6228}, {23, 1600, 2, 6228}, {24, 1595, 1, 6228}, {24, 1608, 2, 6228}, {26, 1603, 2, 6228}, {26, 1615, 1, 6228}, {27, 1604, 2, 6228}, {27, 1611, 2, 6228}, {27, 1616, 2, 6228}, {28, 1612, 2, 6228}, {28, 1617, 1, 6228}, {30, 1618, 1, 6228}, {31, 1619, 1, 6228}, {31, 1624, 2, 6228}, {32, 1625, 2, 6228}, {32, 1629, 1, 6228}, {33, 1630, 1, 6228}, {21, 1600, 1, 6229}, {23, 1603, 1, 6229}, {23, 1615, 2, 6229}, {24, 1604, 1, 6229}, {24, 1616, 2, 6229}, {26, 1618, 3, 6229}, {27, 1619, 4, 6229}, {27, 1624, 2, 6229}, {28, 1620, 1, 6229}, {28, 1625, 2, 6229}, {30, 1633, 1, 6229}, {31, 1634, 3, 6229}, {32, 1635, 1, 6229}, {32, 1637, 2, 6229}, {33, 1638, 1, 6229}, {21, 1608, 1, 6230}, {23, 1611, 1, 6230}, {23, 1616, 2, 6230}, {24, 1612, 1, 6230}, {24, 1617, 2, 6230}, {26, 1619, 1, 6230}, {26, 1624, 2, 6230}, {27, 1620, 2, 6230}, {27, 1625, 2, 6230}, {27, 1629, 2, 6230}, {28, 1621, 1, 6230}, {28, 1630, 2, 6230}, {30, 1634, 1, 6230}, {31, 1635, 2, 6230}, {31, 1637, 1, 6230}, {32, 1636, 1, 6230}, {32, 1638, 2, 6230}, {33, 1639, 1, 6230}, {21, 1592, 1, 6231}, {23, 1595, 1, 6231}, {23, 1601, 2, 6231}, {24, 1596, 1, 6231}, {24, 1609, 2, 6231}, {26, 1604, 2, 6231}, {26, 1616, 1, 6231}, {27, 1605, 2, 6231}, {27, 1612, 2, 6231}, {27, 1622, 2, 6231}, {28, 1613, 2, 6231}, {28, 1623, 1, 6231}, {30, 1619, 1, 6231}, {31, 1620, 1, 6231}, {31, 1625, 2, 6231}, {32, 1626, 2, 6231}, {32, 1630, 1, 6231}, {33, 1631, 1, 6231}, {21, 1601, 1, 6232}, {23, 1604, 1, 6232}, {23, 1616, 2, 6232}, {24, 1605, 1, 6232}, {24, 1622, 2, 6232}, {26, 1619, 2, 6232}, {26, 1624, 1, 6232}, {27, 1620, 2, 6232}, {27, 1625, 4, 6232}, {28, 1626, 3, 6232}, {30, 1634, 1, 6232}, {31, 1635, 1, 6232}, {31, 1637, 2, 6232}, {32, 1638, 3, 6232}, {33, 1640, 1, 6232}, {21, 1609, 1, 6233}, {23, 1612, 1, 6233}, {23, 1622, 2, 6233}, {24, 1613, 1, 6233}, {24, 1623, 2, 6233}, {26, 1625, 3, 6233}, {27, 1626, 4, 6233}, {27, 1630, 2, 6233}, {28, 1627, 1, 6233}, {28, 1631, 2, 6233}, {30, 1637, 1, 6233}, {31, 1638, 3, 6233}, {32, 1639, 1, 6233}, {32, 1640, 2, 6233}, {33, 1641, 1, 6233}, {21, 1593, 1, 6234}, {23, 1596, 1, 6234}, {23, 1602, 2, 6234}, {24, 1597, 1, 6234}, {24, 1610, 2, 6234}, {26, 1605, 2, 6234}, {26, 1617, 1, 6234}, {27, 1606, 2, 6234}, {27, 1613, 2, 6234}, {27, 1623, 2, 6234}, {28, 1614, 2, 6234}, {28, 1628, 1, 6234}, {30, 1620, 1, 6234}, {31, 1621, 1, 6234}, {31, 1626, 2, 6234}, {32, 1627, 2, 6234}, {32, 1631, 1, 6234}, {33, 1632, 1, 6234}, {21, 1602, 1, 6235}, {23, 1605, 1, 6235}, {23, 1617, 2, 6235}, {24, 1606, 1, 6235}, {24, 1623, 2, 6235}, {26, 1620, 2, 6235}, {26, 1629, 1, 6235}, {27, 1621, 2, 6235}, {27, 1626, 2, 6235}, {27, 1630, 2, 6235}, {28, 1627, 2, 6235}, {28, 1631, 1, 6235}, {30, 1635, 1, 6235}, {31, 1636, 1, 6235}, {31, 1638, 2, 6235}, {32, 1639, 2, 6235}, {32, 1640, 1, 6235}, {33, 1641, 1, 6235}, {21, 1610, 1, 6236}, {23, 1613, 1, 6236}, {23, 1623, 2, 6236}, {24, 1614, 1, 6236}, {24, 1628, 2, 6236}, {26, 1626, 2, 6236}, {26, 1630, 1, 6236}, {27, 1627, 2, 6236}, {27, 1631, 4, 6236}, {28, 1632, 3, 6236}, {30, 1638, 1, 6236}, {31, 1639, 1, 6236}, {31, 1640, 2, 6236}, {32, 1641, 3, 6236}, {33, 1642, 1, 6236}, {22, 1588, 1, 6237}, {24, 1589, 3, 6237}, {25, 1590, 3, 6237}, {27, 1591, 1, 6237}, {27, 1598, 2, 6237}, {28, 1592, 2, 6237}, {28, 1599, 4, 6237}, {29, 1593, 1, 6237}, {29, 1607, 2, 6237}, {31, 1600, 1, 6237}, {32, 1601, 2, 6237}, {32, 1608, 1, 6237}, {33, 1602, 1, 6237}, {33, 1609, 2, 6237}, {34, 1610, 1, 6237}, {22, 1589, 1, 6238}, {24, 1591, 2, 6238}, {24, 1598, 1, 6238}, {25, 1592, 2, 6238}, {25, 1599, 1, 6238}, {27, 1594, 1, 6238}, {27, 1600, 2, 6238}, {28, 1595, 2, 6238}, {28, 1601, 2, 6238}, {28, 1608, 2, 6238}, {29, 1596, 1, 6238}, {29, 1609, 2, 6238}, {31, 1603, 1, 6238}, {32, 1604, 2, 6238}, {32, 1611, 1, 6238}, {33, 1605, 1, 6238}, {33, 1612, 2, 6238}, {34, 1613, 1, 6238}, {22, 1590, 1, 6239}, {24, 1592, 2, 6239}, {24, 1599, 1, 6239}, {25, 1593, 2, 6239}, {25, 1607, 1, 6239}, {27, 1595, 1, 6239}, {27, 1601, 2, 6239}, {28, 1596, 2, 6239}, {28, 1602, 2, 6239}, {28, 1609, 2, 6239}, {29, 1597, 1, 6239}, {29, 1610, 2, 6239}, {31, 1604, 1, 6239}, {32, 1605, 2, 6239}, {32, 1612, 1, 6239}, {33, 1606, 1, 6239}, {33, 1613, 2, 6239}, {34, 1614, 1, 6239}, {22, 1589, 1, 6240}, {24, 1591, 1, 6240}, {24, 1598, 2, 6240}, {25, 1592, 1, 6240}, {25, 1599, 2, 6240}, {27, 1600, 3, 6240}, {28, 1601, 4, 6240}, {28, 1608, 2, 6240}, {29, 1602, 1, 6240}, {29, 1609, 2, 6240}, {31, 1615, 1, 6240}, {32, 1616, 3, 6240}, {33, 1617, 1, 6240}, {33, 1622, 2, 6240}, {34, 1623, 1, 6240}, {22, 1598, 1, 6241}, {24, 1600, 3, 6241}, {25, 1601, 3, 6241}, {27, 1603, 1, 6241}, {27, 1615, 2, 6241}, {28, 1604, 2, 6241}, {28, 1616, 4, 6241}, {29, 1605, 1, 6241}, {29, 1622, 2, 6241}, {31, 1618, 1, 6241}, {32, 1619, 2, 6241}, {32, 1624, 1, 6241}, {33, 1620, 1, 6241}, {33, 1625, 2, 6241}, {34, 1626, 1, 6241}, {22, 1599, 1, 6242}, {24, 1601, 2, 6242}, {24, 1608, 1, 6242}, {25, 1602, 2, 6242}, {25, 1609, 1, 6242}, {27, 1604, 1, 6242}, {27, 1616, 2, 6242}, {28, 1605, 2, 6242}, {28, 1617, 2, 6242}, {28, 1622, 2, 6242}, {29, 1606, 1, 6242},

{29, 1623, 2, 6242}, {31, 1619, 1, 6242}, {32, 1620, 2, 6242}, {32, 1625, 1, 6242}, {33,
1621, 1, 6242}, {33, 1626, 2, 6242}, {34, 1627, 1, 6242}, {22, 1590, 1, 6243}, {24, 1592,
1, 6243}, {24, 1599, 2, 6243}, {25, 1593, 1, 6243}, {25, 1607, 2, 6243}, {27, 1601, 2,
6243}, {27, 1608, 1, 6243}, {28, 1602, 2, 6243}, {28, 1609, 4, 6243}, {29, 1610, 3, 6243},
{31, 1616, 1, 6243}, {32, 1617, 1, 6243}, {32, 1622, 2, 6243}, {33, 1623, 3, 6243}, {34,
1628, 1, 6243}, {22, 1599, 1, 6244}, {24, 1601, 1, 6244}, {24, 1608, 2, 6244}, {25, 1602,
1, 6244}, {25, 1609, 2, 6244}, {27, 1611, 1, 6244}, {27, 1616, 2, 6244}, {28, 1612, 2,
6244}, {28, 1617, 2, 6244}, {28, 1622, 2, 6244}, {29, 1613, 1, 6244}, {29, 1623, 2, 6244},
{31, 1624, 1, 6244}, {32, 1625, 2, 6244}, {32, 1629, 1, 6244}, {33, 1626, 1, 6244}, {33,
1630, 2, 6244}, {34, 1631, 1, 6244}, {22, 1607, 1, 6245}, {24, 1609, 3, 6245}, {25, 1610,
3, 6245}, {27, 1612, 1, 6245}, {27, 1622, 2, 6245}, {28, 1613, 2, 6245}, {28, 1623, 4,
6245}, {29, 1614, 1, 6245}, {29, 1628, 2, 6245}, {31, 1625, 1, 6245}, {32, 1626, 2, 6245},
{32, 1630, 1, 6245}, {33, 1627, 1, 6245}, {33, 1631, 2, 6245}, {34, 1632, 1, 6245}, {22,
1591, 1, 6246}, {24, 1594, 1, 6246}, {24, 1600, 2, 6246}, {25, 1595, 1, 6246}, {25, 1608,
2, 6246}, {27, 1603, 2, 6246}, {27, 1615, 1, 6246}, {28, 1604, 2, 6246}, {28, 1611, 2,
6246}, {28, 1616, 2, 6246}, {29, 1612, 2, 6246}, {29, 1617, 1, 6246}, {31, 1618, 1, 6246},
{32, 1619, 1, 6246}, {32, 1624, 2, 6246}, {33, 1625, 2, 6246}, {33, 1629, 1, 6246}, {34,
1630, 1, 6246}, {22, 1600, 1, 6247}, {24, 1603, 1, 6247}, {24, 1615, 2, 6247}, {25, 1604,
1, 6247}, {25, 1616, 2, 6247}, {27, 1618, 3, 6247}, {28, 1619, 4, 6247}, {28, 1624, 2,
6247}, {29, 1620, 1, 6247}, {29, 1625, 2, 6247}, {31, 1633, 1, 6247}, {32, 1634, 3, 6247},
{33, 1635, 1, 6247}, {33, 1637, 2, 6247}, {34, 1638, 1, 6247}, {22, 1608, 1, 6248}, {24,
1611, 1, 6248}, {24, 1616, 2, 6248}, {25, 1612, 1, 6248}, {25, 1617, 2, 6248}, {27, 1619,
1, 6248}, {27, 1624, 2, 6248}, {28, 1620, 2, 6248}, {28, 1625, 2, 6248}, {28, 1629, 2,
6248}, {29, 1621, 1, 6248}, {29, 1630, 2, 6248}, {31, 1634, 1, 6248}, {32, 1635, 2, 6248},
{32, 1637, 1, 6248}, {33, 1636, 1, 6248}, {33, 1638, 2, 6248}, {34, 1639, 1, 6248}, {22,
1592, 1, 6249}, {24, 1595, 1, 6249}, {24, 1601, 2, 6249}, {25, 1596, 1, 6249}, {25, 1609,
2, 6249}, {27, 1604, 2, 6249}, {27, 1616, 1, 6249}, {28, 1605, 2, 6249}, {28, 1612, 2,
6249}, {28, 1622, 2, 6249}, {29, 1613, 2, 6249}, {29, 1623, 1, 6249}, {31, 1619, 1, 6249},
{32, 1620, 1, 6249}, {32, 1625, 2, 6249}, {33, 1626, 2, 6249}, {33, 1630, 1, 6249}, {34,
1631, 1, 6249}, {22, 1601, 1, 6250}, {24, 1604, 1, 6250}, {24, 1616, 2, 6250}, {25, 1605,
1, 6250}, {25, 1622, 2, 6250}, {27, 1619, 2, 6250}, {27, 1624, 1, 6250}, {28, 1620, 2,
6250}, {28, 1625, 4, 6250}, {29, 1626, 3, 6250}, {31, 1634, 1, 6250}, {32, 1635, 1, 6250},
{32, 1637, 2, 6250}, {33, 1638, 3, 6250}, {34, 1640, 1, 6250}, {22, 1609, 1, 6251}, {24,
1612, 1, 6251}, {24, 1622, 2, 6251}, {25, 1613, 1, 6251}, {25, 1623, 2, 6251}, {27, 1625,
3, 6251}, {28, 1626, 4, 6251}, {28, 1630, 2, 6251}, {29, 1627, 1, 6251}, {29, 1631, 2,
6251}, {31, 1637, 1, 6251}, {32, 1638, 3, 6251}, {33, 1639, 1, 6251}, {33, 1640, 2, 6251},
{34, 1641, 1, 6251}, {22, 1593, 1, 6252}, {24, 1596, 1, 6252}, {24, 1602, 2, 6252}, {25,
1597, 1, 6252}, {25, 1610, 2, 6252}, {27, 1605, 2, 6252}, {27, 1617, 1, 6252}, {28, 1606,
2, 6252}, {28, 1613, 2, 6252}, {28, 1623, 2, 6252}, {29, 1614, 2, 6252}, {29, 1628, 1,
6252}, {31, 1620, 1, 6252}, {32, 1621, 1, 6252}, {32, 1626, 2, 6252}, {33, 1627, 2, 6252},
{33, 1631, 1, 6252}, {34, 1632, 1, 6252}, {22, 1602, 1, 6253}, {24, 1605, 1, 6253}, {24,
1617, 2, 6253}, {25, 1606, 1, 6253}, {25, 1623, 2, 6253}, {27, 1620, 2, 6253}, {27, 1629,
1, 6253}, {28, 1621, 2, 6253}, {28, 1626, 2, 6253}, {28, 1630, 2, 6253}, {29, 1627, 2,
6253}, {29, 1631, 1, 6253}, {31, 1635, 1, 6253}, {32, 1636, 1, 6253}, {32, 1638, 2, 6253},
{33, 1639, 2, 6253}, {33, 1640, 1, 6253}, {34, 1641, 1, 6253}, {22, 1610, 1, 6254}, {24,
1613, 1, 6254}, {24, 1623, 2, 6254}, {25, 1614, 1, 6254}, {25, 1628, 2, 6254}, {27, 1626,
2, 6254}, {27, 1630, 1, 6254}, {28, 1627, 2, 6254}, {28, 1631, 4, 6254}, {29, 1632, 3,
6254}, {31, 1638, 1, 6254}, {32, 1639, 1, 6254}, {32, 1640, 2, 6254}, {33, 1641, 3, 6254},
{34, 1642, 1, 6254}, {20, 1793, 1, 6255}, {21, 1794, 3, 6255}, {22, 1795, 3, 6255}, {23,
1796, 1, 6255}, {23, 1799, 2, 6255}, {24, 1797, 2, 6255}, {24, 1800, 4, 6255}, {25, 1798,
1, 6255}, {25, 1804, 2, 6255}, {26, 1801, 1, 6255}, {27, 1802, 2, 6255}, {27, 1805, 1,
6255}, {28, 1803, 1, 6255}, {28, 1806, 2, 6255}, {29, 1807, 1, 6255}, {20, 1794, 1, 6256},
{21, 1796, 1, 6256}, {21, 1799, 2, 6256}, {22, 1797, 1, 6256}, {22, 1800, 2, 6256}, {23,
1801, 3, 6256}, {24, 1802, 4, 6256}, {24, 1805, 2, 6256}, {25, 1803, 1, 6256}, {25, 1806,
2, 6256}, {26, 1808, 1, 6256}, {27, 1809, 3, 6256}, {28, 1810, 1, 6256}, {28, 1811, 2,
6256}, {29, 1812, 1, 6256}, {20, 1795, 1, 6257}, {21, 1797, 1, 6257}, {21, 1800, 2, 6257},
{22, 1798, 1, 6257}, {22, 1804, 2, 6257}, {23, 1802, 2, 6257}, {23, 1805, 1, 6257}, {24,
1803, 2, 6257}, {24, 1806, 4, 6257}, {25, 1807, 3, 6257}, {26, 1809, 1, 6257}, {27, 1810,
1, 6257}, {27, 1811, 2, 6257}, {28, 1812, 3, 6257}, {29, 1813, 1, 6257}, {21, 1793, 1,
6258}, {23, 1794, 3, 6258}, {24, 1795, 3, 6258}, {26, 1796, 1, 6258}, {26, 1799, 2, 6258},
{27, 1797, 2, 6258}, {27, 1800, 4, 6258}, {28, 1798, 1, 6258}, {28, 1804, 2, 6258}, {30,
1801, 1, 6258}, {31, 1802, 2, 6258}, {31, 1805, 1, 6258}, {32, 1803, 1, 6258}, {32, 1806,
2, 6258}, {33, 1807, 1, 6258}, {21, 1794, 1, 6259}, {23, 1796, 1, 6259}, {23, 1799, 2,
6259}, {24, 1797, 1, 6259}, {24, 1800, 2, 6259}, {26, 1801, 3, 6259}, {27, 1802, 4, 6259},
{27, 1805, 2, 6259}, {28, 1803, 1, 6259}, {28, 1806, 2, 6259}, {30, 1808, 1, 6259}, {31,
1809, 3, 6259}, {32, 1810, 1, 6259}, {32, 1811, 2, 6259}, {33, 1812, 1, 6259}, {21, 1795,
1, 6260}, {23, 1797, 1, 6260}, {23, 1800, 2, 6260}, {24, 1798, 1, 6260}, {24, 1804, 2,
6260}, {26, 1802, 2, 6260}, {26, 1805, 1, 6260}, {27, 1803, 2, 6260}, {27, 1806, 4, 6260},
{28, 1807, 3, 6260}, {30, 1809, 1, 6260}, {31, 1810, 1, 6260}, {31, 1811, 2, 6260}, {32,

1812, 3, 6260}, {33, 1813, 1, 6260}, {22, 1793, 1, 6261}, {24, 1794, 3, 6261}, {25, 1795, 3, 6261}, {27, 1796, 1, 6261}, {27, 1799, 2, 6261}, {28, 1797, 2, 6261}, {28, 1800, 4, 6261}, {29, 1798, 1, 6261}, {29, 1804, 2, 6261}, {31, 1801, 1, 6261}, {32, 1802, 2, 6261}, {32, 1805, 1, 6261}, {33, 1803, 1, 6261}, {33, 1806, 2, 6261}, {34, 1807, 1, 6261}, {22, 1794, 1, 6262}, {24, 1796, 1, 6262}, {24, 1799, 2, 6262}, {25, 1797, 1, 6262}, {25, 1800, 2, 6262}, {27, 1801, 3, 6262}, {28, 1802, 4, 6262}, {28, 1805, 2, 6262}, {29, 1803, 1, 6262}, {29, 1806, 2, 6262}, {31, 1808, 1, 6262}, {32, 1809, 3, 6262}, {33, 1810, 1, 6262}, {33, 1811, 2, 6262}, {34, 1812, 1, 6262}, {22, 1795, 1, 6263}, {24, 1797, 1, 6263}, {24, 1800, 2, 6263}, {25, 1798, 1, 6263}, {25, 1804, 2, 6263}, {27, 1802, 2, 6263}, {27, 1805, 1, 6263}, {28, 1803, 2, 6263}, {28, 1806, 4, 6263}, {29, 1807, 3, 6263}, {31, 1809, 1, 6263}, {32, 1810, 1, 6263}, {32, 1811, 2, 6263}, {33, 1812, 3, 6263}, {34, 1813, 1, 6263}, {20, 1814, 1, 6264}, {21, 1815, 2, 6264}, {21, 1824, 1, 6264}, {22, 1816, 2, 6264}, {22, 1834, 1, 6264}, {23, 1817, 1, 6264}, {23, 1825, 2, 6264}, {24, 1818, 2, 6264}, {24, 1826, 2, 6264}, {24, 1835, 2, 6264}, {25, 1819, 1, 6264}, {25, 1836, 2, 6264}, {26, 1827, 1, 6264}, {27, 1828, 2, 6264}, {27, 1837, 1, 6264}, {28, 1829, 1, 6264}, {28, 1838, 2, 6264}, {29, 1839, 1, 6264}, {20, 1815, 1, 6265}, {21, 1817, 2, 6265}, {21, 1825, 1, 6265}, {22, 1818, 2, 6265}, {22, 1835, 1, 6265}, {23, 1820, 1, 6265}, {23, 1827, 2, 6265}, {24, 1821, 2, 6265}, {24, 1828, 2, 6265}, {24, 1837, 2, 6265}, {25, 1822, 1, 6265}, {25, 1838, 2, 6265}, {26, 1830, 1, 6265}, {27, 1831, 2, 6265}, {27, 1840, 1, 6265}, {28, 1832, 1, 6265}, {28, 1841, 2, 6265}, {29, 1842, 1, 6265}, {20, 1816, 1, 6266}, {21, 1818, 2, 6266}, {21, 1826, 1, 6266}, {22, 1819, 2, 6266}, {22, 1836, 1, 6266}, {23, 1821, 1, 6266}, {23, 1828, 2, 6266}, {24, 1822, 2, 6266}, {24, 1829, 2, 6266}, {24, 1838, 2, 6266}, {25, 1823, 1, 6266}, {25, 1839, 2, 6266}, {26, 1831, 1, 6266}, {27, 1832, 2, 6266}, {27, 1841, 1, 6266}, {28, 1833, 1, 6266}, {28, 1842, 2, 6266}, {29, 1843, 1, 6266}, {20, 1824, 1, 6267}, {21, 1825, 2, 6267}, {21, 1844, 1, 6267}, {22, 1826, 2, 6267}, {22, 1854, 1, 6267}, {23, 1827, 1, 6267}, {23, 1845, 2, 6267}, {24, 1828, 2, 6267}, {24, 1846, 2, 6267}, {24, 1855, 2, 6267}, {25, 1829, 1, 6267}, {25, 1856, 2, 6267}, {26, 1847, 1, 6267}, {27, 1848, 2, 6267}, {27, 1857, 1, 6267}, {28, 1849, 1, 6267}, {28, 1858, 2, 6267}, {29, 1859, 1, 6267}, {20, 1825, 1, 6268}, {21, 1827, 2, 6268}, {21, 1845, 1, 6268}, {22, 1828, 2, 6268}, {22, 1855, 1, 6268}, {23, 1830, 1, 6268}, {23, 1847, 2, 6268}, {24, 1831, 2, 6268}, {24, 1848, 2, 6268}, {24, 1857, 2, 6268}, {25, 1832, 1, 6268}, {25, 1858, 2, 6268}, {26, 1850, 1, 6268}, {27, 1851, 2, 6268}, {27, 1860, 1, 6268}, {28, 1852, 1, 6268}, {28, 1861, 2, 6268}, {29, 1862, 1, 6268}, {20, 1826, 1, 6269}, {21, 1828, 2, 6269}, {21, 1846, 1, 6269}, {22, 1829, 2, 6269}, {22, 1856, 1, 6269}, {23, 1831, 1, 6269}, {23, 1848, 2, 6269}, {24, 1832, 2, 6269}, {24, 1849, 2, 6269}, {24, 1858, 2, 6269}, {25, 1833, 1, 6269}, {25, 1859, 2, 6269}, {26, 1851, 1, 6269}, {27, 1852, 2, 6269}, {27, 1861, 1, 6269}, {28, 1853, 1, 6269}, {28, 1862, 2, 6269}, {29, 1863, 1, 6269}, {20, 1834, 1, 6270}, {21, 1835, 2, 6270}, {21, 1854, 1, 6270}, {22, 1836, 2, 6270}, {22, 1864, 1, 6270}, {23, 1837, 1, 6270}, {23, 1855, 2, 6270}, {24, 1838, 2, 6270}, {24, 1856, 2, 6270}, {24, 1865, 2, 6270}, {25, 1839, 1, 6270}, {25, 1866, 2, 6270}, {26, 1857, 1, 6270}, {27, 1858, 2, 6270}, {27, 1867, 1, 6270}, {28, 1859, 1, 6270}, {28, 1868, 2, 6270}, {29, 1869, 1, 6270}, {20, 1835, 1, 6271}, {21, 1837, 2, 6271}, {21, 1855, 1, 6271}, {22, 1838, 2, 6271}, {22, 1865, 1, 6271}, {23, 1840, 1, 6271}, {23, 1857, 2, 6271}, {24, 1841, 2, 6271}, {24, 1858, 2, 6271}, {24, 1867, 2, 6271}, {25, 1842, 1, 6271}, {25, 1868, 2, 6271}, {26, 1860, 1, 6271}, {27, 1861, 2, 6271}, {27, 1870, 1, 6271}, {28, 1862, 1, 6271}, {28, 1871, 2, 6271}, {29, 1872, 1, 6271}, {20, 1836, 1, 6272}, {21, 1838, 2, 6272}, {21, 1856, 1, 6272}, {22, 1839, 2, 6272}, {22, 1866, 1, 6272}, {23, 1841, 1, 6272}, {23, 1858, 2, 6272}, {24, 1842, 2, 6272}, {24, 1859, 2, 6272}, {24, 1868, 2, 6272}, {25, 1843, 1, 6272}, {25, 1869, 2, 6272}, {26, 1861, 1, 6272}, {27, 1862, 2, 6272}, {27, 1871, 1, 6272}, {28, 1863, 1, 6272}, {28, 1872, 2, 6272}, {29, 1873, 1, 6272}, {21, 1814, 1, 6273}, {23, 1815, 2, 6273}, {23, 1824, 1, 6273}, {24, 1816, 2, 6273}, {24, 1834, 1, 6273}, {26, 1817, 1, 6273}, {26, 1825, 2, 6273}, {27, 1818, 2, 6273}, {27, 1826, 2, 6273}, {27, 1835, 2, 6273}, {28, 1819, 1, 6273}, {28, 1836, 2, 6273}, {30, 1827, 1, 6273}, {31, 1828, 2, 6273}, {31, 1837, 1, 6273}, {32, 1829, 1, 6273}, {32, 1838, 2, 6273}, {33, 1839, 1, 6273}, {21, 1815, 1, 6274}, {23, 1817, 2, 6274}, {23, 1825, 1, 6274}, {24, 1818, 2, 6274}, {24, 1835, 1, 6274}, {26, 1820, 1, 6274}, {26, 1827, 2, 6274}, {27, 1821, 2, 6274}, {27, 1828, 2, 6274}, {27, 1837, 2, 6274}, {28, 1822, 1, 6274}, {28, 1838, 2, 6274}, {30, 1830, 1, 6274}, {31, 1831, 2, 6274}, {31, 1840, 1, 6274}, {32, 1832, 1, 6274}, {32, 1841, 2, 6274}, {33, 1842, 1, 6274}, {21, 1816, 1, 6275}, {23, 1818, 2, 6275}, {23, 1826, 1, 6275}, {24, 1819, 2, 6275}, {24, 1836, 1, 6275}, {26, 1821, 1, 6275}, {26, 1828, 2, 6275}, {27, 1822, 2, 6275}, {27, 1829, 2, 6275}, {27, 1838, 2, 6275}, {28, 1823, 1, 6275}, {28, 1839, 2, 6275}, {30, 1831, 1, 6275}, {31, 1832, 2, 6275}, {31, 1841, 1, 6275}, {32, 1833, 1, 6275}, {32, 1842, 2, 6275}, {33, 1843, 1, 6275}, {21, 1824, 1, 6276}, {23, 1825, 2, 6276}, {23, 1844, 1, 6276}, {24, 1826, 2, 6276}, {24, 1854, 1, 6276}, {26, 1827, 1, 6276}, {26, 1845, 2, 6276}, {27, 1828, 2, 6276}, {27, 1846, 2, 6276}, {27, 1855, 2, 6276}, {28, 1829, 1, 6276}, {28, 1856, 2, 6276}, {30, 1847, 1, 6276}, {31, 1848, 2, 6276}, {31, 1857, 1, 6276}, {32, 1849, 1, 6276}, {32, 1858, 2, 6276}, {33, 1859, 1, 6276}, {21, 1825, 1, 6277}, {23, 1827, 2, 6277}, {23, 1845, 1, 6277}, {24, 1828, 2, 6277}, {24, 1855, 1, 6277}, {26, 1830, 1, 6277}, {26, 1847, 2, 6277}, {27, 1831, 2, 6277}, {27, 1848, 2, 6277}, {27, 1857, 2, 6277}, {28, 1832, 1, 6277}, {28, 1858, 2, 6277}, {30, 1850,

1, 6277}, {31, 1851, 2, 6277}, {31, 1860, 1, 6277}, {32, 1852, 1, 6277}, {32, 1861, 2,
6277}, {33, 1862, 1, 6277}, {21, 1826, 1, 6278}, {23, 1828, 2, 6278}, {23, 1846, 1, 6278},
{24, 1829, 2, 6278}, {24, 1856, 1, 6278}, {26, 1831, 1, 6278}, {26, 1848, 2, 6278}, {27,
1832, 2, 6278}, {27, 1849, 2, 6278}, {27, 1858, 2, 6278}, {28, 1833, 1, 6278}, {28, 1859,
2, 6278}, {30, 1851, 1, 6278}, {31, 1852, 2, 6278}, {31, 1861, 1, 6278}, {32, 1853, 1,
6278}, {32, 1862, 2, 6278}, {33, 1863, 1, 6278}, {21, 1834, 1, 6279}, {23, 1835, 2, 6279},
{23, 1854, 1, 6279}, {24, 1836, 2, 6279}, {24, 1864, 1, 6279}, {26, 1837, 1, 6279}, {26,
1855, 2, 6279}, {27, 1838, 2, 6279}, {27, 1856, 2, 6279}, {27, 1865, 2, 6279}, {28, 1839,
1, 6279}, {28, 1866, 2, 6279}, {30, 1857, 1, 6279}, {31, 1858, 2, 6279}, {31, 1867, 1,
6279}, {32, 1859, 1, 6279}, {32, 1868, 2, 6279}, {33, 1869, 1, 6279}, {21, 1835, 1, 6280},
{23, 1837, 2, 6280}, {23, 1855, 1, 6280}, {24, 1838, 2, 6280}, {24, 1865, 1, 6280}, {26,
1840, 1, 6280}, {26, 1857, 2, 6280}, {27, 1841, 2, 6280}, {27, 1858, 2, 6280}, {27, 1867,
2, 6280}, {28, 1842, 1, 6280}, {28, 1868, 2, 6280}, {30, 1860, 1, 6280}, {31, 1861, 2,
6280}, {31, 1870, 1, 6280}, {32, 1862, 1, 6280}, {32, 1871, 2, 6280}, {33, 1872, 1, 6280},
{21, 1836, 1, 6281}, {23, 1838, 2, 6281}, {23, 1856, 1, 6281}, {24, 1839, 2, 6281}, {24,
1866, 1, 6281}, {26, 1841, 1, 6281}, {26, 1858, 2, 6281}, {27, 1842, 2, 6281}, {27, 1859,
2, 6281}, {27, 1868, 2, 6281}, {28, 1843, 1, 6281}, {28, 1869, 2, 6281}, {30, 1861, 1,
6281}, {31, 1862, 2, 6281}, {31, 1871, 1, 6281}, {32, 1863, 1, 6281}, {32, 1872, 2, 6281},
{33, 1873, 1, 6281}, {22, 1814, 1, 6282}, {24, 1815, 2, 6282}, {24, 1824, 1, 6282}, {25,
1816, 2, 6282}, {25, 1834, 1, 6282}, {27, 1817, 1, 6282}, {27, 1825, 2, 6282}, {28, 1818,
2, 6282}, {28, 1826, 2, 6282}, {28, 1835, 2, 6282}, {29, 1819, 1, 6282}, {29, 1836, 2,
6282}, {31, 1827, 1, 6282}, {32, 1828, 2, 6282}, {32, 1837, 1, 6282}, {33, 1829, 1, 6282},
{33, 1838, 2, 6282}, {34, 1839, 1, 6282}, {22, 1815, 1, 6283}, {24, 1817, 2, 6283}, {24,
1825, 1, 6283}, {25, 1818, 2, 6283}, {25, 1835, 1, 6283}, {27, 1820, 1, 6283}, {27, 1827,
2, 6283}, {28, 1821, 2, 6283}, {28, 1828, 2, 6283}, {28, 1837, 2, 6283}, {29, 1822, 1,
6283}, {29, 1838, 2, 6283}, {31, 1830, 1, 6283}, {32, 1831, 2, 6283}, {32, 1840, 1, 6283},
{33, 1832, 1, 6283}, {33, 1841, 2, 6283}, {34, 1842, 1, 6283}, {22, 1816, 1, 6284}, {24,
1818, 2, 6284}, {24, 1826, 1, 6284}, {25, 1819, 2, 6284}, {25, 1836, 1, 6284}, {27, 1821,
1, 6284}, {27, 1828, 2, 6284}, {28, 1822, 2, 6284}, {28, 1829, 2, 6284}, {28, 1838, 2,
6284}, {29, 1823, 1, 6284}, {29, 1839, 2, 6284}, {31, 1831, 1, 6284}, {32, 1832, 2, 6284},
{32, 1841, 1, 6284}, {33, 1833, 1, 6284}, {33, 1842, 2, 6284}, {34, 1843, 1, 6284}, {22,
1824, 1, 6285}, {24, 1825, 2, 6285}, {24, 1844, 1, 6285}, {25, 1826, 2, 6285}, {25, 1854,
1, 6285}, {27, 1827, 1, 6285}, {27, 1845, 2, 6285}, {28, 1828, 2, 6285}, {28, 1846, 2,
6285}, {28, 1855, 2, 6285}, {29, 1829, 1, 6285}, {29, 1856, 2, 6285}, {31, 1847, 1, 6285},
{32, 1848, 2, 6285}, {32, 1857, 1, 6285}, {33, 1849, 1, 6285}, {33, 1858, 2, 6285}, {34,
1859, 1, 6285}, {22, 1825, 1, 6286}, {24, 1827, 2, 6286}, {24, 1845, 1, 6286}, {25, 1828,
2, 6286}, {25, 1855, 1, 6286}, {27, 1830, 1, 6286}, {27, 1847, 2, 6286}, {28, 1831, 2,
6286}, {28, 1848, 2, 6286}, {28, 1857, 2, 6286}, {29, 1832, 1, 6286}, {29, 1858, 2, 6286},
{31, 1850, 1, 6286}, {32, 1851, 2, 6286}, {32, 1860, 1, 6286}, {33, 1852, 1, 6286}, {33,
1861, 2, 6286}, {34, 1862, 1, 6286}, {22, 1826, 1, 6287}, {24, 1828, 2, 6287}, {24, 1846,
1, 6287}, {25, 1829, 2, 6287}, {25, 1856, 1, 6287}, {27, 1831, 1, 6287}, {27, 1848, 2,
6287}, {28, 1832, 2, 6287}, {28, 1849, 2, 6287}, {28, 1858, 2, 6287}, {29, 1833, 1, 6287},
{29, 1859, 2, 6287}, {31, 1851, 1, 6287}, {32, 1852, 2, 6287}, {32, 1861, 1, 6287}, {33,
1853, 1, 6287}, {33, 1862, 2, 6287}, {34, 1863, 1, 6287}, {22, 1834, 1, 6288}, {24, 1835,
2, 6288}, {24, 1854, 1, 6288}, {25, 1836, 2, 6288}, {25, 1864, 1, 6288}, {27, 1837, 1,
6288}, {27, 1855, 2, 6288}, {28, 1838, 2, 6288}, {28, 1856, 2, 6288}, {28, 1865, 2, 6288},
{29, 1839, 1, 6288}, {29, 1866, 2, 6288}, {31, 1857, 1, 6288}, {32, 1858, 2, 6288}, {32,
1867, 1, 6288}, {33, 1859, 1, 6288}, {33, 1868, 2, 6288}, {34, 1869, 1, 6288}, {22, 1835,
1, 6289}, {24, 1837, 2, 6289}, {24, 1855, 1, 6289}, {25, 1838, 2, 6289}, {25, 1865, 1,
6289}, {27, 1840, 1, 6289}, {27, 1857, 2, 6289}, {28, 1841, 2, 6289}, {28, 1858, 2, 6289},
{28, 1867, 2, 6289}, {29, 1842, 1, 6289}, {29, 1868, 2, 6289}, {31, 1860, 1, 6289}, {32,
1861, 2, 6289}, {32, 1870, 1, 6289}, {33, 1862, 1, 6289}, {33, 1871, 2, 6289}, {34, 1872,
1, 6289}, {22, 1836, 1, 6290}, {24, 1838, 2, 6290}, {24, 1856, 1, 6290}, {25, 1839, 2,
6290}, {25, 1866, 1, 6290}, {27, 1841, 1, 6290}, {27, 1858, 2, 6290}, {28, 1842, 2, 6290},
{28, 1859, 2, 6290}, {28, 1868, 2, 6290}, {29, 1843, 1, 6290}, {29, 1869, 2, 6290}, {31,
1861, 1, 6290}, {32, 1862, 2, 6290}, {32, 1871, 1, 6290}, {33, 1863, 1, 6290}, {33, 1872,
2, 6290}, {34, 1873, 1, 6290}, {20, 1970, 1, 6291}, {21, 1971, 2, 6291}, {21, 1976, 1,
6291}, {22, 1972, 2, 6291}, {22, 1982, 1, 6291}, {23, 1973, 1, 6291}, {23, 1977, 2, 6291},
{24, 1974, 2, 6291}, {24, 1978, 2, 6291}, {24, 1983, 2, 6291}, {25, 1975, 1, 6291}, {25,
1984, 2, 6291}, {26, 1979, 1, 6291}, {27, 1980, 2, 6291}, {27, 1985, 1, 6291}, {28, 1981,
1, 6291}, {28, 1986, 2, 6291}, {29, 1987, 1, 6291}, {21, 1970, 1, 6292}, {23, 1971, 2,
6292}, {23, 1976, 1, 6292}, {24, 1972, 2, 6292}, {24, 1982, 1, 6292}, {26, 1973, 1, 6292},
{26, 1977, 2, 6292}, {27, 1974, 2, 6292}, {27, 1978, 2, 6292}, {27, 1983, 2, 6292}, {28,
1975, 1, 6292}, {28, 1984, 2, 6292}, {30, 1979, 1, 6292}, {31, 1980, 2, 6292}, {31, 1985,
1, 6292}, {32, 1981, 1, 6292}, {32, 1986, 2, 6292}, {33, 1987, 1, 6292}, {22, 1970, 1,
6293}, {24, 1971, 2, 6293}, {24, 1976, 1, 6293}, {25, 1972, 2, 6293}, {25, 1982, 1, 6293},
{27, 1973, 1, 6293}, {27, 1977, 2, 6293}, {28, 1974, 2, 6293}, {28, 1978, 2, 6293}, {28,
1983, 2, 6293}, {29, 1975, 1, 6293}, {29, 1984, 2, 6293}, {31, 1979, 1, 6293}, {32, 1980,
2, 6293}, {32, 1985, 1, 6293}, {33, 1981, 1, 6293}, {33, 1986, 2, 6293}, {34, 1987, 1,

6293}, {20, 1988, 1, 6294}, {21, 1989, 2, 6294}, {21, 1998, 1, 6294}, {22, 1990, 2, 6294}, {22, 2008, 1, 6294}, {23, 1991, 1, 6294}, {23, 1999, 2, 6294}, {24, 1992, 2, 6294}, {24, 2000, 2, 6294}, {24, 2009, 2, 6294}, {25, 1993, 1, 6294}, {25, 2010, 2, 6294}, {26, 2001, 1, 6294}, {27, 2002, 2, 6294}, {27, 2011, 1, 6294}, {28, 2003, 1, 6294}, {28, 2012, 2, 6294}, {29, 2013, 1, 6294}, {20, 1989, 1, 6295}, {21, 1991, 2, 6295}, {21, 1999, 1, 6295}, {22, 1992, 2, 6295}, {22, 2009, 1, 6295}, {23, 1994, 1, 6295}, {23, 2001, 2, 6295}, {24, 1995, 2, 6295}, {24, 2002, 2, 6295}, {24, 2011, 2, 6295}, {25, 1996, 1, 6295}, {25, 2012, 2, 6295}, {26, 2004, 1, 6295}, {27, 2005, 2, 6295}, {27, 2014, 1, 6295}, {28, 2006, 1, 6295}, {28, 2015, 2, 6295}, {29, 2016, 1, 6295}, {20, 1990, 1, 6296}, {21, 1992, 2, 6296}, {21, 2000, 1, 6296}, {22, 1993, 2, 6296}, {22, 2010, 1, 6296}, {23, 1995, 1, 6296}, {23, 2002, 2, 6296}, {24, 1996, 2, 6296}, {24, 2003, 2, 6296}, {24, 2012, 2, 6296}, {25, 1997, 1, 6296}, {25, 2013, 2, 6296}, {26, 2005, 1, 6296}, {27, 2006, 2, 6296}, {27, 2015, 1, 6296}, {28, 2007, 1, 6296}, {28, 2016, 2, 6296}, {29, 2017, 1, 6296}, {21, 1988, 1, 6297}, {23, 1989, 2, 6297}, {23, 1998, 1, 6297}, {24, 1990, 2, 6297}, {24, 2008, 1, 6297}, {26, 1991, 1, 6297}, {26, 1999, 2, 6297}, {27, 1992, 2, 6297}, {27, 2000, 2, 6297}, {27, 2009, 2, 6297}, {28, 1993, 1, 6297}, {28, 2010, 2, 6297}, {30, 2001, 1, 6297}, {31, 2002, 2, 6297}, {31, 2011, 1, 6297}, {32, 2003, 1, 6297}, {32, 2012, 2, 6297}, {33, 2013, 1, 6297}, {21, 1989, 1, 6298}, {23, 1991, 2, 6298}, {23, 1999, 1, 6298}, {24, 1992, 2, 6298}, {24, 2009, 1, 6298}, {26, 1994, 1, 6298}, {26, 2001, 2, 6298}, {27, 1995, 2, 6298}, {27, 2002, 2, 6298}, {27, 2011, 2, 6298}, {28, 1996, 1, 6298}, {28, 2012, 2, 6298}, {30, 2004, 1, 6298}, {31, 2005, 2, 6298}, {31, 2014, 1, 6298}, {32, 2006, 1, 6298}, {32, 2015, 2, 6298}, {33, 2016, 1, 6298}, {21, 1990, 1, 6299}, {23, 1992, 2, 6299}, {23, 2000, 1, 6299}, {24, 1993, 2, 6299}, {24, 2010, 1, 6299}, {26, 1995, 1, 6299}, {26, 2002, 2, 6299}, {27, 1996, 2, 6299}, {27, 2003, 2, 6299}, {27, 2012, 2, 6299}, {28, 1997, 1, 6299}, {28, 2013, 2, 6299}, {30, 2005, 1, 6299}, {31, 2006, 2, 6299}, {31, 2015, 1, 6299}, {32, 2007, 1, 6299}, {32, 2016, 2, 6299}, {33, 2017, 1, 6299}, {22, 1988, 1, 6300}, {24, 1989, 2, 6300}, {24, 1998, 1, 6300}, {25, 1990, 2, 6300}, {25, 2008, 1, 6300}, {27, 1991, 1, 6300}, {27, 1999, 2, 6300}, {28, 1992, 2, 6300}, {28, 2000, 2, 6300}, {28, 2009, 2, 6300}, {29, 1993, 1, 6300}, {29, 2010, 2, 6300}, {31, 2001, 1, 6300}, {32, 2002, 2, 6300}, {32, 2011, 1, 6300}, {33, 2003, 1, 6300}, {33, 2012, 2, 6300}, {34, 2013, 1, 6300}, {22, 1989, 1, 6301}, {24, 1991, 2, 6301}, {24, 1999, 1, 6301}, {25, 1992, 2, 6301}, {25, 2009, 1, 6301}, {27, 1994, 1, 6301}, {27, 2001, 2, 6301}, {28, 1995, 2, 6301}, {28, 2002, 2, 6301}, {28, 2011, 2, 6301}, {29, 1996, 1, 6301}, {29, 2012, 2, 6301}, {31, 2004, 1, 6301}, {32, 2005, 2, 6301}, {32, 2014, 1, 6301}, {33, 2006, 1, 6301}, {33, 2015, 2, 6301}, {34, 2016, 1, 6301}, {22, 1990, 1, 6302}, {24, 1992, 2, 6302}, {24, 2000, 1, 6302}, {25, 1993, 2, 6302}, {25, 2010, 1, 6302}, {27, 1995, 1, 6302}, {27, 2002, 2, 6302}, {28, 1996, 2, 6302}, {28, 2003, 2, 6302}, {28, 2012, 2, 6302}, {29, 1997, 1, 6302}, {29, 2013, 2, 6302}, {31, 2005, 1, 6302}, {32, 2006, 2, 6302}, {32, 2015, 1, 6302}, {33, 2007, 1, 6302}, {33, 2016, 2, 6302}, {34, 2017, 1, 6302}, {20, 1154, 1, 6303}, {21, 1155, 1, 6303}, {21, 1169, 2, 6303}, {22, 1156, 1, 6303}, {22, 1184, 2, 6303}, {23, 1170, 2, 6303}, {23, 1199, 1, 6303}, {24, 1171, 2, 6303}, {24, 1185, 2, 6303}, {24, 1214, 2, 6303}, {25, 1186, 2, 6303}, {25, 1229, 1, 6303}, {26, 1200, 1, 6303}, {27, 1201, 1, 6303}, {27, 1215, 2, 6303}, {28, 1216, 2, 6303}, {28, 1230, 1, 6303}, {29, 1231, 1, 6303}, {20, 1155, 1, 6304}, {21, 1157, 1, 6304}, {21, 1170, 2, 6304}, {22, 1158, 1, 6304}, {22, 1185, 2, 6304}, {23, 1172, 2, 6304}, {23, 1200, 1, 6304}, {24, 1173, 2, 6304}, {24, 1187, 2, 6304}, {24, 1215, 2, 6304}, {25, 1188, 2, 6304}, {25, 1230, 1, 6304}, {26, 1202, 1, 6304}, {27, 1203, 1, 6304}, {27, 1217, 2, 6304}, {28, 1218, 2, 6304}, {28, 1232, 1, 6304}, {29, 1233, 1, 6304}, {20, 1156, 1, 6305}, {21, 1158, 1, 6305}, {21, 1171, 2, 6305}, {22, 1159, 1, 6305}, {22, 1186, 2, 6305}, {23, 1173, 2, 6305}, {23, 1201, 1, 6305}, {24, 1174, 2, 6305}, {24, 1188, 2, 6305}, {24, 1216, 2, 6305}, {25, 1189, 2, 6305}, {25, 1231, 1, 6305}, {26, 1203, 1, 6305}, {27, 1204, 1, 6305}, {27, 1218, 2, 6305}, {28, 1219, 2, 6305}, {28, 1233, 1, 6305}, {29, 1234, 1, 6305}, {20, 1157, 1, 6306}, {21, 1160, 1, 6306}, {21, 1172, 2, 6306}, {22, 1161, 1, 6306}, {22, 1187, 2, 6306}, {23, 1175, 2, 6306}, {23, 1202, 1, 6306}, {24, 1176, 2, 6306}, {24, 1190, 2, 6306}, {24, 1217, 2, 6306}, {25, 1191, 2, 6306}, {25, 1232, 1, 6306}, {26, 1205, 1, 6306}, {27, 1206, 1, 6306}, {27, 1220, 2, 6306}, {28, 1221, 2, 6306}, {28, 1235, 1, 6306}, {29, 1236, 1, 6306}, {20, 1158, 1, 6307}, {21, 1161, 1, 6307}, {21, 1173, 2, 6307}, {22, 1162, 1, 6307}, {22, 1188, 2, 6307}, {23, 1176, 2, 6307}, {23, 1203, 1, 6307}, {24, 1177, 2, 6307}, {24, 1191, 2, 6307}, {24, 1218, 2, 6307}, {25, 1192, 2, 6307}, {25, 1233, 1, 6307}, {26, 1206, 1, 6307}, {27, 1207, 1, 6307}, {27, 1221, 2, 6307}, {28, 1222, 2, 6307}, {28, 1236, 1, 6307}, {29, 1237, 1, 6307}, {20, 1159, 1, 6308}, {21, 1162, 1, 6308}, {21, 1174, 2, 6308}, {22, 1163, 1, 6308}, {22, 1189, 2, 6308}, {23, 1177, 2, 6308}, {23, 1204, 1, 6308}, {24, 1178, 2, 6308}, {24, 1192, 2, 6308}, {24, 1219, 2, 6308}, {25, 1193, 2, 6308}, {25, 1234, 1, 6308}, {26, 1207, 1, 6308}, {27, 1208, 1, 6308}, {27, 1222, 2, 6308}, {28, 1223, 2, 6308}, {28, 1237, 1, 6308}, {29, 1238, 1, 6308}, {20, 1160, 1, 6309}, {21, 1164, 1, 6309}, {21, 1175, 2, 6309}, {22, 1165, 1, 6309}, {22, 1190, 2, 6309}, {23, 1179, 2, 6309}, {23, 1205, 1, 6309}, {24, 1180, 2, 6309}, {24, 1194, 2, 6309}, {24, 1220, 2, 6309}, {25, 1195, 2, 6309}, {25, 1235, 1, 6309}, {26, 1209, 1, 6309}, {27, 1210, 1, 6309}, {27, 1224, 2, 6309}, {28, 1225, 2, 6309}, {28, 1239, 1, 6309}, {29, 1240, 1, 6309}, {20, 1161, 1, 6310}, {21, 1165, 1, 6310}, {21, 1176, 2, 6310}, {22, 1166, 1, 6310}, {22, 1191, 2, 6310},

{23, 1180, 2, 6310}, {23, 1206, 1, 6310}, {24, 1181, 2, 6310}, {24, 1195, 2, 6310}, {24, 1221, 2, 6310}, {25, 1196, 2, 6310}, {25, 1236, 1, 6310}, {26, 1210, 1, 6310}, {27, 1211, 1, 6310}, {27, 1225, 2, 6310}, {28, 1226, 2, 6310}, {28, 1240, 1, 6310}, {29, 1241, 1, 6310}, {20, 1162, 1, 6311}, {21, 1166, 1, 6311}, {21, 1177, 2, 6311}, {22, 1167, 1, 6311}, {22, 1192, 2, 6311}, {23, 1181, 2, 6311}, {23, 1207, 1, 6311}, {24, 1182, 2, 6311}, {24, 1196, 2, 6311}, {24, 1222, 2, 6311}, {25, 1197, 2, 6311}, {25, 1237, 1, 6311}, {26, 1211, 1, 6311}, {27, 1212, 1, 6311}, {27, 1226, 2, 6311}, {28, 1227, 2, 6311}, {28, 1241, 1, 6311}, {29, 1242, 1, 6311}, {20, 1163, 1, 6312}, {21, 1167, 1, 6312}, {21, 1178, 2, 6312}, {22, 1168, 1, 6312}, {22, 1193, 2, 6312}, {23, 1182, 2, 6312}, {23, 1208, 1, 6312}, {24, 1183, 2, 6312}, {24, 1197, 2, 6312}, {24, 1223, 2, 6312}, {25, 1198, 2, 6312}, {25, 1238, 1, 6312}, {26, 1212, 1, 6312}, {27, 1213, 1, 6312}, {27, 1227, 2, 6312}, {28, 1228, 2, 6312}, {28, 1242, 1, 6312}, {29, 1243, 1, 6312}, {21, 1154, 1, 6313}, {23, 1155, 1, 6313}, {23, 1169, 2, 6313}, {24, 1156, 1, 6313}, {24, 1184, 2, 6313}, {26, 1170, 2, 6313}, {26, 1199, 1, 6313}, {27, 1171, 2, 6313}, {27, 1185, 2, 6313}, {27, 1214, 2, 6313}, {28, 1186, 2, 6313}, {28, 1229, 1, 6313}, {30, 1200, 1, 6313}, {31, 1201, 1, 6313}, {31, 1215, 2, 6313}, {32, 1216, 2, 6313}, {32, 1230, 1, 6313}, {33, 1231, 1, 6313}, {21, 1155, 1, 6314}, {23, 1157, 1, 6314}, {23, 1170, 2, 6314}, {24, 1158, 1, 6314}, {24, 1185, 2, 6314}, {26, 1172, 2, 6314}, {26, 1200, 1, 6314}, {27, 1173, 2, 6314}, {27, 1187, 2, 6314}, {27, 1215, 2, 6314}, {28, 1188, 2, 6314}, {28, 1230, 1, 6314}, {30, 1202, 1, 6314}, {31, 1203, 1, 6314}, {31, 1217, 2, 6314}, {32, 1218, 2, 6314}, {32, 1232, 1, 6314}, {33, 1233, 1, 6314}, {21, 1156, 1, 6315}, {23, 1158, 1, 6315}, {23, 1171, 2, 6315}, {24, 1159, 1, 6315}, {24, 1186, 2, 6315}, {26, 1173, 2, 6315}, {26, 1201, 1, 6315}, {27, 1174, 2, 6315}, {27, 1188, 2, 6315}, {27, 1216, 2, 6315}, {28, 1189, 2, 6315}, {28, 1231, 1, 6315}, {30, 1203, 1, 6315}, {31, 1204, 1, 6315}, {31, 1218, 2, 6315}, {32, 1219, 2, 6315}, {32, 1233, 1, 6315}, {33, 1234, 1, 6315}, {21, 1157, 1, 6316}, {23, 1160, 1, 6316}, {23, 1172, 2, 6316}, {24, 1161, 1, 6316}, {24, 1187, 2, 6316}, {26, 1175, 2, 6316}, {26, 1202, 1, 6316}, {27, 1176, 2, 6316}, {27, 1190, 2, 6316}, {27, 1217, 2, 6316}, {28, 1191, 2, 6316}, {28, 1232, 1, 6316}, {30, 1205, 1, 6316}, {31, 1206, 1, 6316}, {31, 1220, 2, 6316}, {32, 1221, 2, 6316}, {32, 1235, 1, 6316}, {33, 1236, 1, 6316}, {21, 1158, 1, 6317}, {23, 1161, 1, 6317}, {23, 1173, 2, 6317}, {24, 1162, 1, 6317}, {24, 1188, 2, 6317}, {26, 1176, 2, 6317}, {26, 1203, 1, 6317}, {27, 1177, 2, 6317}, {27, 1191, 2, 6317}, {27, 1218, 2, 6317}, {28, 1192, 2, 6317}, {28, 1233, 1, 6317}, {30, 1206, 1, 6317}, {31, 1207, 1, 6317}, {31, 1221, 2, 6317}, {32, 1222, 2, 6317}, {32, 1236, 1, 6317}, {33, 1237, 1, 6317}, {21, 1159, 1, 6318}, {23, 1162, 1, 6318}, {23, 1174, 2, 6318}, {24, 1163, 1, 6318}, {24, 1189, 2, 6318}, {26, 1177, 2, 6318}, {26, 1204, 1, 6318}, {27, 1178, 2, 6318}, {27, 1192, 2, 6318}, {27, 1219, 2, 6318}, {28, 1193, 2, 6318}, {28, 1234, 1, 6318}, {30, 1207, 1, 6318}, {31, 1208, 1, 6318}, {31, 1222, 2, 6318}, {32, 1223, 2, 6318}, {32, 1237, 1, 6318}, {33, 1238, 1, 6318}, {21, 1160, 1, 6319}, {23, 1164, 1, 6319}, {23, 1175, 2, 6319}, {24, 1165, 1, 6319}, {24, 1190, 2, 6319}, {26, 1179, 2, 6319}, {26, 1205, 1, 6319}, {27, 1180, 2, 6319}, {27, 1194, 2, 6319}, {27, 1220, 2, 6319}, {28, 1195, 2, 6319}, {28, 1235, 1, 6319}, {30, 1209, 1, 6319}, {31, 1210, 1, 6319}, {31, 1224, 2, 6319}, {32, 1225, 2, 6319}, {32, 1239, 1, 6319}, {33, 1240, 1, 6319}, {21, 1161, 1, 6320}, {23, 1165, 1, 6320}, {23, 1176, 2, 6320}, {24, 1166, 1, 6320}, {24, 1191, 2, 6320}, {26, 1180, 2, 6320}, {26, 1206, 1, 6320}, {27, 1181, 2, 6320}, {27, 1195, 2, 6320}, {27, 1221, 2, 6320}, {28, 1196, 2, 6320}, {28, 1236, 1, 6320}, {30, 1210, 1, 6320}, {31, 1211, 1, 6320}, {31, 1225, 2, 6320}, {32, 1226, 2, 6320}, {32, 1240, 1, 6320}, {33, 1241, 1, 6320}, {21, 1162, 1, 6321}, {23, 1166, 1, 6321}, {23, 1177, 2, 6321}, {24, 1167, 1, 6321}, {24, 1192, 2, 6321}, {26, 1181, 2, 6321}, {26, 1207, 1, 6321}, {27, 1182, 2, 6321}, {27, 1196, 2, 6321}, {27, 1222, 2, 6321}, {28, 1197, 2, 6321}, {28, 1237, 1, 6321}, {30, 1211, 1, 6321}, {31, 1212, 1, 6321}, {31, 1226, 2, 6321}, {32, 1227, 2, 6321}, {32, 1241, 1, 6321}, {33, 1242, 1, 6321}, {21, 1163, 1, 6322}, {23, 1167, 1, 6322}, {23, 1178, 2, 6322}, {24, 1168, 1, 6322}, {24, 1193, 2, 6322}, {26, 1182, 2, 6322}, {26, 1208, 1, 6322}, {27, 1183, 2, 6322}, {27, 1197, 2, 6322}, {27, 1223, 2, 6322}, {28, 1198, 2, 6322}, {28, 1238, 1, 6322}, {30, 1212, 1, 6322}, {31, 1213, 1, 6322}, {31, 1227, 2, 6322}, {32, 1228, 2, 6322}, {32, 1242, 1, 6322}, {33, 1243, 1, 6322}, {22, 1154, 1, 6323}, {24, 1155, 1, 6323}, {24, 1169, 2, 6323}, {25, 1156, 1, 6323}, {25, 1184, 2, 6323}, {27, 1170, 2, 6323}, {27, 1199, 1, 6323}, {28, 1171, 2, 6323}, {28, 1185, 2, 6323}, {28, 1214, 2, 6323}, {29, 1186, 2, 6323}, {29, 1229, 1, 6323}, {31, 1200, 1, 6323}, {32, 1201, 1, 6323}, {32, 1215, 2, 6323}, {33, 1216, 2, 6323}, {33, 1230, 1, 6323}, {34, 1231, 1, 6323}, {22, 1155, 1, 6324}, {24, 1157, 1, 6324}, {24, 1170, 2, 6324}, {25, 1158, 1, 6324}, {25, 1185, 2, 6324}, {27, 1172, 2, 6324}, {27, 1200, 1, 6324}, {28, 1173, 2, 6324}, {28, 1187, 2, 6324}, {28, 1215, 2, 6324}, {29, 1188, 2, 6324}, {29, 1230, 1, 6324}, {31, 1202, 1, 6324}, {32, 1203, 1, 6324}, {32, 1217, 2, 6324}, {33, 1218, 2, 6324}, {33, 1232, 1, 6324}, {34, 1233, 1, 6324}, {22, 1156, 1, 6325}, {24, 1158, 1, 6325}, {24, 1171, 2, 6325}, {25, 1159, 1, 6325}, {25, 1186, 2, 6325}, {27, 1173, 2, 6325}, {27, 1201, 1, 6325}, {28, 1174, 2, 6325}, {28, 1188, 2, 6325}, {28, 1216, 2, 6325}, {29, 1189, 2, 6325}, {29, 1231, 1, 6325}, {31, 1203, 1, 6325}, {32, 1204, 1, 6325}, {32, 1218, 2, 6325}, {33, 1219, 2, 6325}, {33, 1233, 1, 6325}, {34, 1234, 1, 6325}, {22, 1157, 1, 6326}, {24, 1160, 1, 6326}, {24, 1172, 2, 6326}, {25, 1161, 1, 6326}, {25, 1187, 2, 6326}, {27, 1175, 2, 6326}, {27, 1202, 1, 6326}, {28, 1176, 2, 6326}, {28, 1190, 2, 6326}, {28, 1217, 2, 6326}, {29,

1191, 2, 6326}, {29, 1232, 1, 6326}, {31, 1205, 1, 6326}, {32, 1206, 1, 6326}, {32, 1220, 2, 6326}, {33, 1221, 2, 6326}, {33, 1235, 1, 6326}, {34, 1236, 1, 6326}, {22, 1158, 1, 6327}, {24, 1161, 1, 6327}, {24, 1173, 2, 6327}, {25, 1162, 1, 6327}, {25, 1188, 2, 6327}, {27, 1176, 2, 6327}, {27, 1203, 1, 6327}, {28, 1177, 2, 6327}, {28, 1191, 2, 6327}, {28, 1218, 2, 6327}, {29, 1192, 2, 6327}, {29, 1233, 1, 6327}, {31, 1206, 1, 6327}, {32, 1207, 1, 6327}, {32, 1221, 2, 6327}, {33, 1222, 2, 6327}, {33, 1236, 1, 6327}, {34, 1237, 1, 6327}, {22, 1159, 1, 6328}, {24, 1162, 1, 6328}, {24, 1174, 2, 6328}, {25, 1163, 1, 6328}, {25, 1189, 2, 6328}, {27, 1177, 2, 6328}, {27, 1204, 1, 6328}, {28, 1178, 2, 6328}, {28, 1192, 2, 6328}, {28, 1219, 2, 6328}, {29, 1193, 2, 6328}, {29, 1234, 1, 6328}, {31, 1207, 1, 6328}, {32, 1208, 1, 6328}, {32, 1222, 2, 6328}, {33, 1223, 2, 6328}, {33, 1237, 1, 6328}, {34, 1238, 1, 6328}, {22, 1160, 1, 6329}, {24, 1164, 1, 6329}, {24, 1175, 2, 6329}, {25, 1165, 1, 6329}, {25, 1190, 2, 6329}, {27, 1179, 2, 6329}, {27, 1205, 1, 6329}, {28, 1180, 2, 6329}, {28, 1194, 2, 6329}, {28, 1220, 2, 6329}, {29, 1195, 2, 6329}, {29, 1235, 1, 6329}, {31, 1209, 1, 6329}, {32, 1210, 1, 6329}, {32, 1224, 2, 6329}, {33, 1225, 2, 6329}, {33, 1239, 1, 6329}, {34, 1240, 1, 6329}, {22, 1161, 1, 6330}, {24, 1165, 1, 6330}, {24, 1176, 2, 6330}, {25, 1166, 1, 6330}, {25, 1191, 2, 6330}, {27, 1180, 2, 6330}, {27, 1206, 1, 6330}, {28, 1181, 2, 6330}, {28, 1195, 2, 6330}, {28, 1221, 2, 6330}, {29, 1196, 2, 6330}, {29, 1236, 1, 6330}, {31, 1210, 1, 6330}, {32, 1211, 1, 6330}, {32, 1225, 2, 6330}, {33, 1226, 2, 6330}, {33, 1240, 1, 6330}, {34, 1241, 1, 6330}, {22, 1162, 1, 6331}, {24, 1166, 1, 6331}, {24, 1177, 2, 6331}, {25, 1167, 1, 6331}, {25, 1192, 2, 6331}, {27, 1181, 2, 6331}, {27, 1207, 1, 6331}, {28, 1182, 2, 6331}, {28, 1196, 2, 6331}, {28, 1222, 2, 6331}, {29, 1197, 2, 6331}, {29, 1237, 1, 6331}, {31, 1211, 1, 6331}, {32, 1212, 1, 6331}, {32, 1226, 2, 6331}, {33, 1227, 2, 6331}, {33, 1241, 1, 6331}, {34, 1242, 1, 6331}, {22, 1163, 1, 6332}, {24, 1167, 1, 6332}, {24, 1178, 2, 6332}, {25, 1168, 1, 6332}, {25, 1193, 2, 6332}, {27, 1182, 2, 6332}, {27, 1208, 1, 6332}, {28, 1183, 2, 6332}, {28, 1197, 2, 6332}, {28, 1223, 2, 6332}, {29, 1198, 2, 6332}, {29, 1238, 1, 6332}, {31, 1212, 1, 6332}, {32, 1213, 1, 6332}, {32, 1227, 2, 6332}, {33, 1228, 2, 6332}, {33, 1242, 1, 6332}, {34, 1243, 1, 6332}, {20, 1814, 1, 6333}, {21, 1815, 1, 6333}, {21, 1824, 2, 6333}, {22, 1816, 1, 6333}, {22, 1834, 2, 6333}, {23, 1825, 2, 6333}, {23, 1844, 1, 6333}, {24, 1826, 2, 6333}, {24, 1835, 2, 6333}, {24, 1854, 2, 6333}, {25, 1836, 2, 6333}, {25, 1864, 1, 6333}, {26, 1845, 1, 6333}, {27, 1846, 1, 6333}, {27, 1855, 2, 6333}, {28, 1856, 2, 6333}, {28, 1865, 1, 6333}, {29, 1866, 1, 6333}, {20, 1815, 1, 6334}, {21, 1817, 1, 6334}, {21, 1825, 2, 6334}, {22, 1818, 1, 6334}, {22, 1835, 2, 6334}, {23, 1827, 2, 6334}, {23, 1845, 1, 6334}, {24, 1828, 2, 6334}, {24, 1837, 2, 6334}, {24, 1855, 2, 6334}, {25, 1838, 2, 6334}, {25, 1865, 1, 6334}, {26, 1847, 1, 6334}, {27, 1848, 1, 6334}, {27, 1857, 2, 6334}, {28, 1858, 2, 6334}, {28, 1867, 1, 6334}, {29, 1868, 1, 6334}, {20, 1816, 1, 6335}, {21, 1818, 1, 6335}, {21, 1826, 2, 6335}, {22, 1819, 1, 6335}, {22, 1836, 2, 6335}, {23, 1828, 2, 6335}, {23, 1846, 1, 6335}, {24, 1829, 2, 6335}, {24, 1838, 2, 6335}, {24, 1856, 2, 6335}, {25, 1839, 2, 6335}, {25, 1866, 1, 6335}, {26, 1848, 1, 6335}, {27, 1849, 1, 6335}, {27, 1858, 2, 6335}, {28, 1859, 2, 6335}, {28, 1868, 1, 6335}, {29, 1869, 1, 6335}, {20, 1817, 1, 6336}, {21, 1820, 1, 6336}, {21, 1827, 2, 6336}, {22, 1821, 1, 6336}, {22, 1837, 2, 6336}, {23, 1830, 2, 6336}, {23, 1847, 1, 6336}, {24, 1831, 2, 6336}, {24, 1840, 2, 6336}, {24, 1857, 2, 6336}, {25, 1841, 2, 6336}, {25, 1867, 1, 6336}, {26, 1850, 1, 6336}, {27, 1851, 1, 6336}, {27, 1860, 2, 6336}, {28, 1861, 2, 6336}, {28, 1870, 1, 6336}, {29, 1871, 1, 6336}, {20, 1818, 1, 6337}, {21, 1821, 1, 6337}, {21, 1828, 2, 6337}, {22, 1822, 1, 6337}, {22, 1838, 2, 6337}, {23, 1831, 2, 6337}, {23, 1848, 1, 6337}, {24, 1832, 2, 6337}, {24, 1841, 2, 6337}, {24, 1858, 2, 6337}, {25, 1842, 2, 6337}, {25, 1868, 1, 6337}, {26, 1851, 1, 6337}, {27, 1852, 1, 6337}, {27, 1861, 2, 6337}, {28, 1862, 2, 6337}, {28, 1871, 1, 6337}, {29, 1872, 1, 6337}, {20, 1819, 1, 6338}, {21, 1822, 1, 6338}, {21, 1829, 2, 6338}, {22, 1823, 1, 6338}, {22, 1839, 2, 6338}, {23, 1832, 2, 6338}, {23, 1849, 1, 6338}, {24, 1833, 2, 6338}, {24, 1842, 2, 6338}, {24, 1859, 2, 6338}, {25, 1843, 2, 6338}, {25, 1869, 1, 6338}, {26, 1852, 1, 6338}, {27, 1853, 1, 6338}, {27, 1862, 2, 6338}, {28, 1863, 2, 6338}, {28, 1872, 1, 6338}, {29, 1873, 1, 6338}, {21, 1814, 1, 6339}, {23, 1815, 1, 6339}, {23, 1824, 2, 6339}, {24, 1816, 1, 6339}, {24, 1834, 2, 6339}, {26, 1825, 2, 6339}, {26, 1844, 1, 6339}, {27, 1826, 2, 6339}, {27, 1835, 2, 6339}, {27, 1854, 2, 6339}, {28, 1836, 2, 6339}, {28, 1864, 1, 6339}, {30, 1845, 1, 6339}, {31, 1846, 1, 6339}, {31, 1855, 2, 6339}, {32, 1856, 2, 6339}, {32, 1865, 1, 6339}, {33, 1866, 1, 6339}, {21, 1815, 1, 6340}, {23, 1817, 1, 6340}, {23, 1825, 2, 6340}, {24, 1818, 1, 6340}, {24, 1835, 2, 6340}, {26, 1827, 2, 6340}, {26, 1845, 1, 6340}, {27, 1828, 2, 6340}, {27, 1837, 2, 6340}, {27, 1855, 2, 6340}, {28, 1838, 2, 6340}, {28, 1865, 1, 6340}, {30, 1847, 1, 6340}, {31, 1848, 1, 6340}, {31, 1857, 2, 6340}, {32, 1858, 2, 6340}, {32, 1867, 1, 6340}, {33, 1868, 1, 6340}, {21, 1816, 1, 6341}, {23, 1818, 1, 6341}, {23, 1826, 2, 6341}, {24, 1819, 1, 6341}, {24, 1836, 2, 6341}, {26, 1828, 2, 6341}, {26, 1846, 1, 6341}, {27, 1829, 2, 6341}, {27, 1838, 2, 6341}, {27, 1856, 2, 6341}, {28, 1839, 2, 6341}, {28, 1866, 1, 6341}, {30, 1848, 1, 6341}, {31, 1849, 1, 6341}, {31, 1858, 2, 6341}, {32, 1859, 2, 6341}, {32, 1868, 1, 6341}, {33, 1869, 1, 6341}, {21, 1817, 1, 6342}, {23, 1820, 1, 6342}, {23, 1827, 2, 6342}, {24, 1821, 1, 6342}, {24, 1837, 2, 6342}, {26, 1830, 2, 6342}, {26, 1847, 1, 6342}, {27, 1831, 2, 6342}, {27, 1840, 2, 6342}, {27, 1857, 2, 6342}, {28, 1841, 2, 6342}, {28, 1867, 1, 6342}, {30, 1850, 1, 6342}, {31, 1851, 1, 6342}, {31, 1860, 2, 6342}, {32, 1861,

2, 6342}, {32, 1870, 1, 6342}, {33, 1871, 1, 6342}, {21, 1818, 1, 6343}, {23, 1821, 1, 6343}, {23, 1828, 2, 6343}, {24, 1822, 1, 6343}, {24, 1838, 2, 6343}, {26, 1831, 2, 6343}, {26, 1848, 1, 6343}, {27, 1832, 2, 6343}, {27, 1841, 2, 6343}, {27, 1858, 2, 6343}, {28, 1842, 2, 6343}, {28, 1868, 1, 6343}, {30, 1851, 1, 6343}, {31, 1852, 1, 6343}, {31, 1861, 2, 6343}, {32, 1862, 2, 6343}, {32, 1871, 1, 6343}, {33, 1872, 1, 6343}, {21, 1819, 1, 6344}, {23, 1822, 1, 6344}, {23, 1829, 2, 6344}, {24, 1823, 1, 6344}, {24, 1839, 2, 6344}, {26, 1832, 2, 6344}, {26, 1849, 1, 6344}, {27, 1833, 2, 6344}, {27, 1842, 2, 6344}, {27, 1859, 2, 6344}, {28, 1843, 2, 6344}, {28, 1869, 1, 6344}, {30, 1852, 1, 6344}, {31, 1853, 1, 6344}, {31, 1862, 2, 6344}, {32, 1863, 2, 6344}, {32, 1872, 1, 6344}, {33, 1873, 1, 6344}, {22, 1814, 1, 6345}, {24, 1815, 1, 6345}, {24, 1824, 2, 6345}, {25, 1816, 1, 6345}, {25, 1834, 2, 6345}, {27, 1825, 2, 6345}, {27, 1844, 1, 6345}, {28, 1826, 2, 6345}, {28, 1835, 2, 6345}, {28, 1854, 2, 6345}, {29, 1836, 2, 6345}, {29, 1864, 1, 6345}, {31, 1845, 1, 6345}, {32, 1846, 1, 6345}, {32, 1855, 2, 6345}, {33, 1856, 2, 6345}, {33, 1865, 1, 6345}, {34, 1866, 1, 6345}, {22, 1815, 1, 6346}, {24, 1817, 1, 6346}, {24, 1825, 2, 6346}, {25, 1818, 1, 6346}, {25, 1835, 2, 6346}, {27, 1827, 2, 6346}, {27, 1845, 1, 6346}, {28, 1828, 2, 6346}, {28, 1837, 2, 6346}, {28, 1855, 2, 6346}, {29, 1838, 2, 6346}, {29, 1865, 1, 6346}, {31, 1847, 1, 6346}, {32, 1848, 1, 6346}, {32, 1857, 2, 6346}, {33, 1858, 2, 6346}, {33, 1867, 1, 6346}, {34, 1868, 1, 6346}, {22, 1816, 1, 6347}, {24, 1818, 1, 6347}, {24, 1826, 2, 6347}, {25, 1819, 1, 6347}, {25, 1836, 2, 6347}, {27, 1828, 2, 6347}, {27, 1846, 1, 6347}, {28, 1829, 2, 6347}, {28, 1838, 2, 6347}, {28, 1856, 2, 6347}, {29, 1839, 2, 6347}, {29, 1866, 1, 6347}, {31, 1848, 1, 6347}, {32, 1849, 1, 6347}, {32, 1858, 2, 6347}, {33, 1859, 2, 6347}, {33, 1868, 1, 6347}, {34, 1869, 1, 6347}, {22, 1817, 1, 6348}, {24, 1820, 1, 6348}, {24, 1827, 2, 6348}, {25, 1821, 1, 6348}, {25, 1837, 2, 6348}, {27, 1830, 2, 6348}, {27, 1847, 1, 6348}, {28, 1831, 2, 6348}, {28, 1840, 2, 6348}, {28, 1857, 2, 6348}, {29, 1841, 2, 6348}, {29, 1867, 1, 6348}, {31, 1850, 1, 6348}, {32, 1851, 1, 6348}, {32, 1860, 2, 6348}, {33, 1861, 2, 6348}, {33, 1870, 1, 6348}, {34, 1871, 1, 6348}, {22, 1818, 1, 6349}, {24, 1821, 1, 6349}, {24, 1828, 2, 6349}, {25, 1822, 1, 6349}, {25, 1838, 2, 6349}, {27, 1831, 2, 6349}, {27, 1848, 1, 6349}, {28, 1832, 2, 6349}, {28, 1841, 2, 6349}, {28, 1858, 2, 6349}, {29, 1842, 2, 6349}, {29, 1868, 1, 6349}, {31, 1851, 1, 6349}, {32, 1852, 1, 6349}, {32, 1861, 2, 6349}, {33, 1862, 2, 6349}, {33, 1871, 1, 6349}, {34, 1872, 1, 6349}, {22, 1819, 1, 6350}, {24, 1822, 1, 6350}, {24, 1829, 2, 6350}, {25, 1823, 1, 6350}, {25, 1839, 2, 6350}, {27, 1832, 2, 6350}, {27, 1849, 1, 6350}, {28, 1833, 2, 6350}, {28, 1842, 2, 6350}, {28, 1859, 2, 6350}, {29, 1843, 2, 6350}, {29, 1869, 1, 6350}, {31, 1852, 1, 6350}, {32, 1853, 1, 6350}, {32, 1862, 2, 6350}, {33, 1863, 2, 6350}, {33, 1872, 1, 6350}, {34, 1873, 1, 6350}, {20, 2174, 1, 6351}, {21, 2175, 3, 6351}, {22, 2176, 3, 6351}, {23, 2177, 1, 6351}, {23, 2180, 2, 6351}, {24, 2178, 2, 6351}, {24, 2181, 4, 6351}, {25, 2179, 1, 6351}, {25, 2185, 2, 6351}, {26, 2182, 1, 6351}, {27, 2183, 2, 6351}, {27, 2186, 1, 6351}, {28, 2184, 1, 6351}, {28, 2187, 2, 6351}, {29, 2188, 1, 6351}, {20, 2175, 1, 6352}, {21, 2177, 1, 6352}, {21, 2180, 2, 6352}, {22, 2178, 1, 6352}, {22, 2181, 2, 6352}, {23, 2182, 3, 6352}, {24, 2183, 4, 6352}, {24, 2186, 2, 6352}, {25, 2184, 1, 6352}, {25, 2187, 2, 6352}, {26, 2189, 1, 6352}, {27, 2190, 3, 6352}, {28, 2191, 1, 6352}, {28, 2192, 2, 6352}, {29, 2193, 1, 6352}, {20, 2176, 1, 6353}, {21, 2178, 1, 6353}, {21, 2181, 2, 6353}, {22, 2179, 1, 6353}, {22, 2185, 2, 6353}, {23, 2183, 2, 6353}, {23, 2186, 1, 6353}, {24, 2184, 2, 6353}, {24, 2187, 4, 6353}, {25, 2188, 3, 6353}, {26, 2190, 1, 6353}, {27, 2191, 1, 6353}, {27, 2192, 2, 6353}, {28, 2193, 3, 6353}, {29, 2194, 1, 6353}, {21, 2174, 1, 6354}, {23, 2175, 3, 6354}, {24, 2176, 3, 6354}, {26, 2177, 1, 6354}, {26, 2180, 2, 6354}, {27, 2178, 2, 6354}, {27, 2181, 4, 6354}, {28, 2179, 1, 6354}, {28, 2185, 2, 6354}, {30, 2182, 1, 6354}, {31, 2183, 2, 6354}, {31, 2186, 1, 6354}, {32, 2184, 1, 6354}, {32, 2187, 2, 6354}, {33, 2188, 1, 6354}, {21, 2175, 1, 6355}, {23, 2177, 1, 6355}, {23, 2180, 2, 6355}, {24, 2178, 1, 6355}, {24, 2181, 2, 6355}, {26, 2182, 3, 6355}, {27, 2183, 4, 6355}, {27, 2186, 2, 6355}, {28, 2184, 1, 6355}, {28, 2187, 2, 6355}, {30, 2189, 1, 6355}, {31, 2190, 3, 6355}, {32, 2191, 1, 6355}, {32, 2192, 2, 6355}, {33, 2193, 1, 6355}, {21, 2176, 1, 6356}, {23, 2178, 1, 6356}, {23, 2181, 2, 6356}, {24, 2179, 1, 6356}, {24, 2185, 2, 6356}, {26, 2183, 2, 6356}, {26, 2186, 1, 6356}, {27, 2184, 2, 6356}, {27, 2187, 4, 6356}, {28, 2188, 3, 6356}, {30, 2190, 1, 6356}, {31, 2191, 1, 6356}, {31, 2192, 2, 6356}, {32, 2193, 3, 6356}, {33, 2194, 1, 6356}, {22, 2174, 1, 6357}, {24, 2175, 3, 6357}, {25, 2176, 3, 6357}, {27, 2177, 1, 6357}, {27, 2180, 2, 6357}, {28, 2178, 2, 6357}, {28, 2181, 4, 6357}, {29, 2179, 1, 6357}, {29, 2185, 2, 6357}, {31, 2182, 1, 6357}, {32, 2183, 2, 6357}, {32, 2186, 1, 6357}, {33, 2184, 1, 6357}, {33, 2187, 2, 6357}, {34, 2188, 1, 6357}, {22, 2175, 1, 6358}, {24, 2177, 1, 6358}, {24, 2180, 2, 6358}, {25, 2178, 1, 6358}, {25, 2181, 2, 6358}, {27, 2182, 3, 6358}, {28, 2183, 4, 6358}, {28, 2186, 2, 6358}, {29, 2184, 1, 6358}, {29, 2187, 2, 6358}, {31, 2189, 1, 6358}, {32, 2190, 3, 6358}, {33, 2191, 1, 6358}, {33, 2192, 2, 6358}, {34, 2193, 1, 6358}, {22, 2176, 1, 6359}, {24, 2178, 1, 6359}, {24, 2181, 2, 6359}, {25, 2179, 1, 6359}, {25, 2185, 2, 6359}, {27, 2183, 2, 6359}, {27, 2186, 1, 6359}, {28, 2184, 2, 6359}, {28, 2187, 4, 6359}, {29, 2188, 3, 6359}, {31, 2190, 1, 6359}, {32, 2191, 1, 6359}, {32, 2192, 2, 6359}, {33, 2193, 3, 6359}, {34, 2194, 1, 6359}, {20, 2351, 1, 6360}, {21, 2352, 2, 6360}, {21, 2357, 1, 6360}, {22, 2353, 2, 6360}, {22, 2363, 1, 6360}, {23, 2354, 1, 6360}, {23, 2358, 2, 6360}, {24, 2355, 2, 6360}, {24, 2359, 2, 6360}, {24, 2364, 2, 6360}, {25, 2356, 1, 6360}, {25, 2365, 2,

6360}, {26, 2360, 1, 6360}, {27, 2361, 2, 6360}, {27, 2366, 1, 6360}, {28, 2362, 1, 6360}, {28, 2367, 2, 6360}, {29, 2368, 1, 6360}, {21, 2351, 1, 6361}, {23, 2352, 2, 6361}, {23, 2357, 1, 6361}, {24, 2353, 2, 6361}, {24, 2363, 1, 6361}, {26, 2354, 1, 6361}, {26, 2358, 2, 6361}, {27, 2355, 2, 6361}, {27, 2359, 2, 6361}, {27, 2364, 2, 6361}, {28, 2356, 1, 6361}, {28, 2365, 2, 6361}, {30, 2360, 1, 6361}, {31, 2361, 2, 6361}, {31, 2366, 1, 6361}, {32, 2362, 1, 6361}, {32, 2367, 2, 6361}, {33, 2368, 1, 6361}, {22, 2351, 1, 6362}, {24, 2352, 2, 6362}, {24, 2357, 1, 6362}, {25, 2353, 2, 6362}, {25, 2363, 1, 6362}, {27, 2354, 1, 6362}, {27, 2358, 2, 6362}, {28, 2355, 2, 6362}, {28, 2359, 2, 6362}, {28, 2364, 2, 6362}, {29, 2356, 1, 6362}, {29, 2365, 2, 6362}, {31, 2360, 1, 6362}, {32, 2361, 2, 6362}, {32, 2366, 1, 6362}, {33, 2362, 1, 6362}, {33, 2367, 2, 6362}, {34, 2368, 1, 6362}, {20, 1394, 1, 6363}, {21, 1395, 3, 6363}, {21, 1404, 1, 6363}, {22, 1396, 3, 6363}, {22, 1414, 1, 6363}, {23, 1397, 3, 6363}, {23, 1405, 3, 6363}, {24, 1398, 6, 6363}, {24, 1406, 3, 6363}, {24, 1415, 3, 6363}, {25, 1399, 3, 6363}, {25, 1416, 3, 6363}, {26, 1400, 1, 6363}, {26, 1407, 3, 6363}, {27, 1401, 3, 6363}, {27, 1408, 6, 6363}, {27, 1417, 3, 6363}, {28, 1402, 3, 6363}, {28, 1409, 3, 6363}, {28, 1418, 6, 6363}, {29, 1403, 1, 6363}, {29, 1419, 3, 6363}, {30, 1410, 1, 6363}, {31, 1411, 3, 6363}, {31, 1420, 1, 6363}, {32, 1412, 3, 6363}, {32, 1421, 3, 6363}, {33, 1413, 1, 6363}, {33, 1422, 3, 6363}, {34, 1423, 1, 6363}, {20, 1424, 1, 6364}, {21, 1425, 3, 6364}, {21, 1439, 1, 6364}, {22, 1426, 3, 6364}, {22, 1454, 1, 6364}, {23, 1427, 3, 6364}, {23, 1440, 3, 6364}, {24, 1428, 6, 6364}, {24, 1441, 3, 6364}, {24, 1455, 3, 6364}, {25, 1429, 3, 6364}, {25, 1456, 3, 6364}, {26, 1430, 1, 6364}, {26, 1442, 3, 6364}, {27, 1431, 3, 6364}, {27, 1443, 6, 6364}, {27, 1457, 3, 6364}, {28, 1432, 3, 6364}, {28, 1444, 3, 6364}, {28, 1458, 6, 6364}, {29, 1433, 1, 6364}, {29, 1459, 3, 6364}, {30, 1445, 1, 6364}, {31, 1446, 3, 6364}, {31, 1460, 1, 6364}, {32, 1447, 3, 6364}, {32, 1461, 3, 6364}, {33, 1448, 1, 6364}, {33, 1462, 3, 6364}, {34, 1463, 1, 6364}, {20, 1425, 1, 6365}, {21, 1427, 3, 6365}, {21, 1440, 1, 6365}, {22, 1428, 3, 6365}, {22, 1455, 1, 6365}, {23, 1430, 3, 6365}, {23, 1442, 3, 6365}, {24, 1431, 6, 6365}, {24, 1443, 3, 6365}, {24, 1457, 3, 6365}, {25, 1432, 3, 6365}, {25, 1458, 3, 6365}, {26, 1434, 1, 6365}, {26, 1445, 3, 6365}, {27, 1435, 3, 6365}, {27, 1446, 6, 6365}, {27, 1460, 3, 6365}, {28, 1436, 3, 6365}, {28, 1447, 3, 6365}, {28, 1461, 6, 6365}, {29, 1437, 1, 6365}, {29, 1462, 3, 6365}, {30, 1449, 1, 6365}, {31, 1450, 3, 6365}, {31, 1464, 1, 6365}, {32, 1451, 3, 6365}, {32, 1465, 3, 6365}, {33, 1452, 1, 6365}, {33, 1466, 3, 6365}, {34, 1467, 1, 6365}, {20, 1426, 1, 6366}, {21, 1428, 3, 6366}, {21, 1441, 1, 6366}, {22, 1429, 3, 6366}, {22, 1456, 1, 6366}, {23, 1431, 3, 6366}, {23, 1443, 3, 6366}, {24, 1432, 6, 6366}, {24, 1444, 3, 6366}, {24, 1458, 3, 6366}, {25, 1433, 3, 6366}, {25, 1459, 3, 6366}, {26, 1435, 1, 6366}, {26, 1446, 3, 6366}, {27, 1436, 3, 6366}, {27, 1447, 6, 6366}, {27, 1461, 3, 6366}, {28, 1437, 3, 6366}, {28, 1448, 3, 6366}, {28, 1462, 6, 6366}, {29, 1438, 1, 6366}, {29, 1463, 3, 6366}, {30, 1450, 1, 6366}, {31, 1451, 3, 6366}, {31, 1465, 1, 6366}, {32, 1452, 3, 6366}, {32, 1466, 3, 6366}, {33, 1453, 1, 6366}, {33, 1467, 3, 6366}, {34, 1468, 1, 6366}, {20, 1469, 1, 6367}, {21, 1470, 3, 6367}, {21, 1490, 1, 6367}, {22, 1471, 3, 6367}, {22, 1511, 1, 6367}, {23, 1472, 3, 6367}, {23, 1491, 3, 6367}, {24, 1473, 6, 6367}, {24, 1492, 3, 6367}, {24, 1512, 3, 6367}, {25, 1474, 3, 6367}, {25, 1513, 3, 6367}, {26, 1475, 1, 6367}, {26, 1493, 3, 6367}, {27, 1476, 3, 6367}, {27, 1494, 6, 6367}, {27, 1514, 3, 6367}, {28, 1477, 3, 6367}, {28, 1495, 3, 6367}, {28, 1515, 6, 6367}, {29, 1478, 1, 6367}, {29, 1516, 3, 6367}, {30, 1496, 1, 6367}, {31, 1497, 3, 6367}, {31, 1517, 1, 6367}, {32, 1498, 3, 6367}, {32, 1518, 3, 6367}, {33, 1499, 1, 6367}, {33, 1519, 3, 6367}, {34, 1520, 1, 6367}, {20, 1470, 1, 6368}, {21, 1472, 3, 6368}, {21, 1491, 1, 6368}, {22, 1473, 3, 6368}, {22, 1512, 1, 6368}, {23, 1475, 3, 6368}, {23, 1493, 3, 6368}, {24, 1476, 6, 6368}, {24, 1494, 3, 6368}, {24, 1514, 3, 6368}, {25, 1477, 3, 6368}, {25, 1515, 3, 6368}, {26, 1479, 1, 6368}, {26, 1496, 3, 6368}, {27, 1480, 3, 6368}, {27, 1497, 6, 6368}, {27, 1517, 3, 6368}, {28, 1481, 3, 6368}, {28, 1498, 3, 6368}, {28, 1518, 6, 6368}, {29, 1482, 1, 6368}, {29, 1519, 3, 6368}, {30, 1500, 1, 6368}, {31, 1501, 3, 6368}, {31, 1521, 1, 6368}, {32, 1502, 3, 6368}, {32, 1522, 3, 6368}, {33, 1503, 1, 6368}, {33, 1523, 3, 6368}, {34, 1524, 1, 6368}, {20, 1471, 1, 6369}, {21, 1473, 3, 6369}, {21, 1492, 1, 6369}, {22, 1474, 3, 6369}, {22, 1513, 1, 6369}, {23, 1476, 3, 6369}, {23, 1494, 3, 6369}, {24, 1477, 6, 6369}, {24, 1495, 3, 6369}, {24, 1515, 3, 6369}, {25, 1478, 3, 6369}, {25, 1516, 3, 6369}, {26, 1480, 1, 6369}, {26, 1497, 3, 6369}, {27, 1481, 3, 6369}, {27, 1498, 6, 6369}, {27, 1518, 3, 6369}, {28, 1482, 3, 6369}, {28, 1499, 3, 6369}, {28, 1519, 6, 6369}, {29, 1483, 1, 6369}, {29, 1520, 3, 6369}, {30, 1501, 1, 6369}, {31, 1502, 3, 6369}, {31, 1522, 1, 6369}, {32, 1503, 3, 6369}, {32, 1523, 3, 6369}, {33, 1504, 1, 6369}, {33, 1524, 3, 6369}, {34, 1525, 1, 6369}, {20, 1472, 1, 6370}, {21, 1475, 3, 6370}, {21, 1493, 1, 6370}, {22, 1476, 3, 6370}, {22, 1514, 1, 6370}, {23, 1479, 3, 6370}, {23, 1496, 3, 6370}, {24, 1480, 6, 6370}, {24, 1497, 3, 6370}, {24, 1517, 3, 6370}, {25, 1481, 3, 6370}, {25, 1518, 3, 6370}, {26, 1484, 1, 6370}, {26, 1500, 3, 6370}, {27, 1485, 3, 6370}, {27, 1501, 6, 6370}, {27, 1521, 3, 6370}, {28, 1486, 3, 6370}, {28, 1502, 3, 6370}, {28, 1522, 6, 6370}, {29, 1487, 1, 6370}, {29, 1523, 3, 6370}, {30, 1505, 1, 6370}, {31, 1506, 3, 6370}, {31, 1526, 1, 6370}, {32, 1507, 3, 6370}, {32, 1527, 3, 6370}, {33, 1508, 1, 6370}, {33, 1528, 3, 6370}, {34, 1529, 1, 6370}, {20, 1473, 1, 6371}, {21, 1476, 3, 6371}, {21, 1494, 1, 6371}, {22, 1477, 3, 6371}, {22, 1515, 1, 6371}, {23, 1480, 3, 6371}, {23, 1497, 3, 6371}, {24, 1481, 6, 6371}, {24, 1498, 3, 6371}, {24, 1518, 3, 6371}, {25, 1482, 3, 6371},

{25, 1519, 3, 6371}, {26, 1485, 1, 6371}, {26, 1501, 3, 6371}, {27, 1486, 3, 6371}, {27, 1502, 6, 6371}, {27, 1522, 3, 6371}, {28, 1487, 3, 6371}, {28, 1503, 3, 6371}, {28, 1523, 6, 6371}, {29, 1488, 1, 6371}, {29, 1524, 3, 6371}, {30, 1506, 1, 6371}, {31, 1507, 3, 6371}, {31, 1527, 1, 6371}, {32, 1508, 3, 6371}, {32, 1528, 3, 6371}, {33, 1509, 1, 6371}, {33, 1529, 3, 6371}, {34, 1530, 1, 6371}, {20, 1474, 1, 6372}, {21, 1477, 3, 6372}, {21, 1495, 1, 6372}, {22, 1478, 3, 6372}, {22, 1516, 1, 6372}, {23, 1481, 3, 6372}, {23, 1498, 3, 6372}, {24, 1482, 6, 6372}, {24, 1499, 3, 6372}, {24, 1519, 3, 6372}, {25, 1483, 3, 6372}, {25, 1520, 3, 6372}, {26, 1486, 1, 6372}, {26, 1502, 3, 6372}, {27, 1487, 3, 6372}, {27, 1503, 6, 6372}, {27, 1523, 3, 6372}, {28, 1488, 3, 6372}, {28, 1504, 3, 6372}, {28, 1524, 6, 6372}, {29, 1489, 1, 6372}, {29, 1525, 3, 6372}, {30, 1507, 1, 6372}, {31, 1508, 3, 6372}, {31, 1528, 1, 6372}, {32, 1509, 3, 6372}, {32, 1529, 3, 6372}, {33, 1510, 1, 6372}, {33, 1530, 3, 6372}, {34, 1531, 1, 6372}, {35, 1376, 1, 6373}, {36, 1377, 2, 6373}, {36, 1382, 1, 6373}, {37, 1378, 2, 6373}, {37, 1388, 1, 6373}, {38, 1379, 1, 6373}, {38, 1383, 2, 6373}, {39, 1380, 2, 6373}, {39, 1384, 2, 6373}, {39, 1389, 2, 6373}, {40, 1381, 1, 6373}, {40, 1390, 2, 6373}, {41, 1385, 1, 6373}, {42, 1386, 2, 6373}, {42, 1391, 1, 6373}, {43, 1387, 1, 6373}, {43, 1392, 2, 6373}, {44, 1393, 1, 6373}, {36, 1376, 1, 6374}, {38, 1377, 2, 6374}, {38, 1382, 1, 6374}, {39, 1378, 2, 6374}, {39, 1388, 1, 6374}, {41, 1379, 1, 6374}, {41, 1383, 2, 6374}, {42, 1380, 2, 6374}, {42, 1384, 2, 6374}, {42, 1389, 2, 6374}, {43, 1381, 1, 6374}, {43, 1390, 2, 6374}, {45, 1385, 1, 6374}, {46, 1386, 2, 6374}, {46, 1391, 1, 6374}, {47, 1387, 1, 6374}, {47, 1392, 2, 6374}, {48, 1393, 1, 6374}, {37, 1376, 1, 6375}, {39, 1377, 2, 6375}, {39, 1382, 1, 6375}, {40, 1378, 2, 6375}, {40, 1388, 1, 6375}, {42, 1379, 1, 6375}, {42, 1383, 2, 6375}, {43, 1380, 2, 6375}, {43, 1384, 2, 6375}, {43, 1389, 2, 6375}, {44, 1381, 1, 6375}, {44, 1390, 2, 6375}, {46, 1385, 1, 6375}, {47, 1386, 2, 6375}, {47, 1391, 1, 6375}, {48, 1387, 1, 6375}, {48, 1392, 2, 6375}, {49, 1393, 1, 6375}, {38, 1376, 1, 6376}, {41, 1377, 2, 6376}, {41, 1382, 1, 6376}, {42, 1378, 2, 6376}, {42, 1388, 1, 6376}, {45, 1379, 1, 6376}, {45, 1383, 2, 6376}, {46, 1380, 2, 6376}, {46, 1384, 2, 6376}, {46, 1389, 2, 6376}, {47, 1381, 1, 6376}, {47, 1390, 2, 6376}, {50, 1385, 1, 6376}, {51, 1386, 2, 6376}, {51, 1391, 1, 6376}, {52, 1387, 1, 6376}, {52, 1392, 2, 6376}, {53, 1393, 1, 6376}, {39, 1376, 1, 6377}, {42, 1377, 2, 6377}, {42, 1382, 1, 6377}, {43, 1378, 2, 6377}, {43, 1388, 1, 6377}, {46, 1379, 1, 6377}, {46, 1383, 2, 6377}, {47, 1380, 2, 6377}, {47, 1384, 2, 6377}, {47, 1389, 2, 6377}, {48, 1381, 1, 6377}, {48, 1390, 2, 6377}, {51, 1385, 1, 6377}, {52, 1386, 2, 6377}, {52, 1391, 1, 6377}, {53, 1387, 1, 6377}, {53, 1392, 2, 6377}, {54, 1393, 1, 6377}, {40, 1376, 1, 6378}, {43, 1377, 2, 6378}, {43, 1382, 1, 6378}, {44, 1378, 2, 6378}, {44, 1388, 1, 6378}, {47, 1379, 1, 6378}, {47, 1383, 2, 6378}, {48, 1380, 2, 6378}, {48, 1384, 2, 6378}, {48, 1389, 2, 6378}, {49, 1381, 1, 6378}, {49, 1390, 2, 6378}, {52, 1385, 1, 6378}, {53, 1386, 2, 6378}, {53, 1391, 1, 6378}, {54, 1387, 1, 6378}, {54, 1392, 2, 6378}, {55, 1393, 1, 6378}, {35, 1394, 1, 6379}, {36, 1395, 2, 6379}, {36, 1404, 1, 6379}, {37, 1396, 2, 6379}, {37, 1414, 1, 6379}, {38, 1397, 1, 6379}, {38, 1405, 2, 6379}, {39, 1398, 2, 6379}, {39, 1406, 2, 6379}, {39, 1415, 2, 6379}, {40, 1399, 1, 6379}, {40, 1416, 2, 6379}, {41, 1407, 1, 6379}, {42, 1408, 2, 6379}, {42, 1417, 1, 6379}, {43, 1409, 1, 6379}, {43, 1418, 2, 6379}, {44, 1419, 1, 6379}, {35, 1395, 1, 6380}, {36, 1397, 2, 6380}, {36, 1405, 1, 6380}, {37, 1398, 2, 6380}, {37, 1415, 1, 6380}, {38, 1400, 1, 6380}, {38, 1407, 2, 6380}, {39, 1401, 2, 6380}, {39, 1408, 2, 6380}, {39, 1417, 2, 6380}, {40, 1402, 1, 6380}, {40, 1418, 2, 6380}, {41, 1410, 1, 6380}, {42, 1411, 2, 6380}, {42, 1420, 1, 6380}, {43, 1412, 1, 6380}, {43, 1421, 2, 6380}, {44, 1422, 1, 6380}, {35, 1396, 1, 6381}, {36, 1398, 2, 6381}, {36, 1406, 1, 6381}, {37, 1399, 2, 6381}, {37, 1416, 1, 6381}, {38, 1401, 1, 6381}, {38, 1408, 2, 6381}, {39, 1402, 2, 6381}, {39, 1409, 2, 6381}, {39, 1418, 2, 6381}, {40, 1403, 1, 6381}, {40, 1419, 2, 6381}, {41, 1411, 1, 6381}, {42, 1412, 2, 6381}, {42, 1421, 1, 6381}, {43, 1413, 1, 6381}, {43, 1422, 2, 6381}, {44, 1423, 1, 6381}, {36, 1394, 1, 6382}, {38, 1395, 2, 6382}, {38, 1404, 1, 6382}, {39, 1396, 2, 6382}, {39, 1414, 1, 6382}, {41, 1397, 1, 6382}, {41, 1405, 2, 6382}, {42, 1398, 2, 6382}, {42, 1406, 2, 6382}, {42, 1415, 2, 6382}, {43, 1399, 1, 6382}, {43, 1416, 2, 6382}, {45, 1407, 1, 6382}, {46, 1408, 2, 6382}, {46, 1417, 1, 6382}, {47, 1409, 1, 6382}, {47, 1418, 2, 6382}, {48, 1419, 1, 6382}, {36, 1395, 1, 6383}, {38, 1397, 2, 6383}, {38, 1405, 1, 6383}, {39, 1398, 2, 6383}, {39, 1415, 1, 6383}, {41, 1400, 1, 6383}, {41, 1407, 2, 6383}, {42, 1401, 2, 6383}, {42, 1408, 2, 6383}, {42, 1417, 2, 6383}, {43, 1402, 1, 6383}, {43, 1418, 2, 6383}, {45, 1410, 1, 6383}, {46, 1411, 2, 6383}, {46, 1420, 1, 6383}, {47, 1412, 1, 6383}, {47, 1421, 2, 6383}, {48, 1422, 1, 6383}, {36, 1396, 1, 6384}, {38, 1398, 2, 6384}, {38, 1406, 1, 6384}, {39, 1399, 2, 6384}, {39, 1416, 1, 6384}, {41, 1401, 1, 6384}, {41, 1408, 2, 6384}, {42, 1402, 2, 6384}, {42, 1409, 2, 6384}, {42, 1418, 2, 6384}, {43, 1403, 1, 6384}, {43, 1419, 2, 6384}, {45, 1411, 1, 6384}, {46, 1412, 2, 6384}, {46, 1421, 1, 6384}, {47, 1413, 1, 6384}, {47, 1422, 2, 6384}, {48, 1423, 1, 6384}, {37, 1394, 1, 6385}, {39, 1395, 2, 6385}, {39, 1404, 1, 6385}, {40, 1396, 2, 6385}, {40, 1414, 1, 6385}, {42, 1397, 1, 6385}, {42, 1405, 2, 6385}, {43, 1398, 2, 6385}, {43, 1406, 2, 6385}, {43, 1415, 2, 6385}, {44, 1399, 1, 6385}, {44, 1416, 2, 6385}, {46, 1407, 1, 6385}, {47, 1408, 2, 6385}, {47, 1417, 1, 6385}, {48, 1409, 1, 6385}, {48, 1418, 2, 6385}, {49, 1419, 1, 6385}, {37, 1395, 1, 6386}, {39, 1397, 2, 6386}, {39, 1405, 1, 6386}, {40, 1398, 2, 6386}, {40, 1415, 1, 6386}, {42, 1400, 1, 6386}, {42, 1407, 2, 6386}, {43, 1401, 2, 6386}, {43, 1408, 2, 6386}, {43, 1417, 2, 6386}, {44,

1402, 1, 6386}, {44, 1418, 2, 6386}, {46, 1410, 1, 6386}, {47, 1411, 2, 6386}, {47, 1420, 1, 6386}, {48, 1412, 1, 6386}, {48, 1421, 2, 6386}, {49, 1422, 1, 6386}, {37, 1396, 1, 6387}, {39, 1398, 2, 6387}, {39, 1406, 1, 6387}, {40, 1399, 2, 6387}, {40, 1416, 1, 6387}, {42, 1401, 1, 6387}, {42, 1408, 2, 6387}, {43, 1402, 2, 6387}, {43, 1409, 2, 6387}, {43, 1418, 2, 6387}, {44, 1403, 1, 6387}, {44, 1419, 2, 6387}, {46, 1411, 1, 6387}, {47, 1412, 2, 6387}, {47, 1421, 1, 6387}, {48, 1413, 1, 6387}, {48, 1422, 2, 6387}, {49, 1423, 1, 6387}, {38, 1394, 1, 6388}, {41, 1395, 2, 6388}, {41, 1404, 1, 6388}, {42, 1396, 2, 6388}, {42, 1414, 1, 6388}, {45, 1397, 1, 6388}, {45, 1405, 2, 6388}, {46, 1398, 2, 6388}, {46, 1406, 2, 6388}, {46, 1415, 2, 6388}, {47, 1399, 1, 6388}, {47, 1416, 2, 6388}, {50, 1407, 1, 6388}, {51, 1408, 2, 6388}, {51, 1417, 1, 6388}, {52, 1409, 1, 6388}, {52, 1418, 2, 6388}, {53, 1419, 1, 6388}, {38, 1395, 1, 6389}, {41, 1397, 2, 6389}, {41, 1405, 1, 6389}, {42, 1398, 2, 6389}, {42, 1415, 1, 6389}, {45, 1400, 1, 6389}, {45, 1407, 2, 6389}, {46, 1401, 2, 6389}, {46, 1408, 2, 6389}, {46, 1417, 2, 6389}, {47, 1402, 1, 6389}, {47, 1418, 2, 6389}, {50, 1410, 1, 6389}, {51, 1411, 2, 6389}, {51, 1420, 1, 6389}, {52, 1412, 1, 6389}, {52, 1421, 2, 6389}, {53, 1422, 1, 6389}, {38, 1396, 1, 6390}, {41, 1398, 2, 6390}, {41, 1406, 1, 6390}, {42, 1399, 2, 6390}, {42, 1416, 1, 6390}, {45, 1401, 1, 6390}, {45, 1408, 2, 6390}, {46, 1402, 2, 6390}, {46, 1409, 2, 6390}, {46, 1418, 2, 6390}, {47, 1403, 1, 6390}, {47, 1419, 2, 6390}, {50, 1411, 1, 6390}, {51, 1412, 2, 6390}, {51, 1421, 1, 6390}, {52, 1413, 1, 6390}, {52, 1422, 2, 6390}, {53, 1423, 1, 6390}, {39, 1394, 1, 6391}, {42, 1395, 2, 6391}, {42, 1404, 1, 6391}, {43, 1396, 2, 6391}, {43, 1414, 1, 6391}, {46, 1397, 1, 6391}, {46, 1405, 2, 6391}, {47, 1398, 2, 6391}, {47, 1406, 2, 6391}, {47, 1415, 2, 6391}, {48, 1399, 1, 6391}, {48, 1416, 2, 6391}, {51, 1407, 1, 6391}, {52, 1408, 2, 6391}, {52, 1417, 1, 6391}, {53, 1409, 1, 6391}, {53, 1418, 2, 6391}, {54, 1419, 1, 6391}, {39, 1395, 1, 6392}, {42, 1397, 2, 6392}, {42, 1405, 1, 6392}, {43, 1398, 2, 6392}, {43, 1415, 1, 6392}, {46, 1400, 1, 6392}, {46, 1407, 2, 6392}, {47, 1401, 2, 6392}, {47, 1408, 2, 6392}, {47, 1417, 2, 6392}, {48, 1402, 1, 6392}, {48, 1418, 2, 6392}, {51, 1410, 1, 6392}, {52, 1411, 2, 6392}, {52, 1420, 1, 6392}, {53, 1412, 1, 6392}, {53, 1421, 2, 6392}, {54, 1422, 1, 6392}, {39, 1396, 1, 6393}, {42, 1398, 2, 6393}, {42, 1406, 1, 6393}, {43, 1399, 2, 6393}, {43, 1416, 1, 6393}, {46, 1401, 1, 6393}, {46, 1408, 2, 6393}, {47, 1402, 2, 6393}, {47, 1409, 2, 6393}, {47, 1418, 2, 6393}, {48, 1403, 1, 6393}, {48, 1419, 2, 6393}, {51, 1411, 1, 6393}, {52, 1412, 2, 6393}, {52, 1421, 1, 6393}, {53, 1413, 1, 6393}, {53, 1422, 2, 6393}, {54, 1423, 1, 6393}, {40, 1394, 1, 6394}, {43, 1395, 2, 6394}, {43, 1404, 1, 6394}, {44, 1396, 2, 6394}, {44, 1414, 1, 6394}, {47, 1397, 1, 6394}, {47, 1405, 2, 6394}, {48, 1398, 2, 6394}, {48, 1406, 2, 6394}, {48, 1415, 2, 6394}, {49, 1399, 1, 6394}, {49, 1416, 2, 6394}, {52, 1407, 1, 6394}, {53, 1408, 2, 6394}, {53, 1417, 1, 6394}, {54, 1409, 1, 6394}, {54, 1418, 2, 6394}, {55, 1419, 1, 6394}, {40, 1395, 1, 6395}, {43, 1397, 2, 6395}, {43, 1405, 1, 6395}, {44, 1398, 2, 6395}, {44, 1415, 1, 6395}, {47, 1400, 1, 6395}, {47, 1407, 2, 6395}, {48, 1401, 2, 6395}, {48, 1408, 2, 6395}, {48, 1417, 2, 6395}, {49, 1402, 1, 6395}, {49, 1418, 2, 6395}, {52, 1410, 1, 6395}, {53, 1411, 2, 6395}, {53, 1420, 1, 6395}, {54, 1412, 1, 6395}, {54, 1421, 2, 6395}, {55, 1422, 1, 6395}, {40, 1396, 1, 6396}, {43, 1398, 2, 6396}, {43, 1406, 1, 6396}, {44, 1399, 2, 6396}, {44, 1416, 1, 6396}, {47, 1401, 1, 6396}, {47, 1408, 2, 6396}, {48, 1402, 2, 6396}, {48, 1409, 2, 6396}, {48, 1418, 2, 6396}, {49, 1403, 1, 6396}, {49, 1419, 2, 6396}, {52, 1411, 1, 6396}, {53, 1412, 2, 6396}, {53, 1421, 1, 6396}, {54, 1413, 1, 6396}, {54, 1422, 2, 6396}, {55, 1423, 1, 6396}, {20, 1814, 1, 6397}, {21, 1815, 3, 6397}, {21, 1824, 1, 6397}, {22, 1816, 3, 6397}, {22, 1834, 1, 6397}, {23, 1817, 3, 6397}, {23, 1825, 3, 6397}, {24, 1818, 6, 6397}, {24, 1826, 3, 6397}, {24, 1835, 3, 6397}, {25, 1819, 3, 6397}, {25, 1836, 3, 6397}, {26, 1820, 1, 6397}, {26, 1827, 3, 6397}, {27, 1821, 3, 6397}, {27, 1828, 6, 6397}, {27, 1837, 3, 6397}, {28, 1822, 3, 6397}, {28, 1829, 3, 6397}, {28, 1838, 6, 6397}, {29, 1823, 1, 6397}, {29, 1839, 3, 6397}, {30, 1830, 1, 6397}, {31, 1831, 3, 6397}, {31, 1840, 1, 6397}, {32, 1832, 3, 6397}, {32, 1841, 3, 6397}, {33, 1833, 1, 6397}, {33, 1842, 3, 6397}, {34, 1843, 1, 6397}, {20, 1824, 1, 6398}, {21, 1825, 3, 6398}, {21, 1844, 1, 6398}, {22, 1826, 3, 6398}, {22, 1854, 1, 6398}, {23, 1827, 3, 6398}, {23, 1845, 3, 6398}, {24, 1828, 6, 6398}, {24, 1846, 3, 6398}, {24, 1855, 3, 6398}, {25, 1829, 3, 6398}, {25, 1856, 3, 6398}, {26, 1830, 1, 6398}, {26, 1847, 3, 6398}, {27, 1831, 3, 6398}, {27, 1848, 6, 6398}, {27, 1857, 3, 6398}, {28, 1832, 3, 6398}, {28, 1849, 3, 6398}, {28, 1858, 6, 6398}, {29, 1833, 1, 6398}, {29, 1859, 3, 6398}, {30, 1850, 1, 6398}, {31, 1851, 3, 6398}, {31, 1860, 1, 6398}, {32, 1852, 3, 6398}, {32, 1861, 3, 6398}, {33, 1853, 1, 6398}, {33, 1862, 3, 6398}, {34, 1863, 1, 6398}, {20, 1834, 1, 6399}, {21, 1835, 3, 6399}, {21, 1854, 1, 6399}, {22, 1836, 3, 6399}, {22, 1864, 1, 6399}, {23, 1837, 3, 6399}, {23, 1855, 3, 6399}, {24, 1838, 6, 6399}, {24, 1856, 3, 6399}, {24, 1865, 3, 6399}, {25, 1839, 3, 6399}, {25, 1866, 3, 6399}, {26, 1840, 1, 6399}, {26, 1857, 3, 6399}, {27, 1841, 3, 6399}, {27, 1858, 6, 6399}, {27, 1867, 3, 6399}, {28, 1842, 3, 6399}, {28, 1859, 3, 6399}, {28, 1868, 6, 6399}, {29, 1843, 1, 6399}, {29, 1869, 3, 6399}, {30, 1860, 1, 6399}, {31, 1861, 3, 6399}, {31, 1870, 1, 6399}, {32, 1862, 3, 6399}, {32, 1871, 3, 6399}, {33, 1863, 1, 6399}, {33, 1872, 3, 6399}, {34, 1873, 1, 6399}, {20, 1874, 1, 6400}, {21, 1875, 3, 6400}, {21, 1889, 1, 6400}, {22, 1876, 3, 6400}, {22, 1904, 1, 6400}, {23, 1877, 3, 6400}, {23, 1890, 3, 6400}, {24, 1878, 6, 6400}, {24, 1891, 3, 6400}, {24, 1905, 3, 6400}, {25, 1879, 3, 6400}, {25, 1906, 3, 6400}, {26, 1880, 1, 6400}, {26, 1892, 3, 6400}, {27, 1881, 3, 6400}, {27, 1893,

6, 6400}, {27, 1907, 3, 6400}, {28, 1882, 3, 6400}, {28, 1894, 3, 6400}, {28, 1908, 6, 6400}, {29, 1883, 1, 6400}, {29, 1909, 3, 6400}, {30, 1895, 1, 6400}, {31, 1896, 3, 6400}, {31, 1910, 1, 6400}, {32, 1897, 3, 6400}, {32, 1911, 3, 6400}, {33, 1898, 1, 6400}, {33, 1912, 3, 6400}, {34, 1913, 1, 6400}, {20, 1875, 1, 6401}, {21, 1877, 3, 6401}, {21, 1890, 1, 6401}, {22, 1878, 3, 6401}, {22, 1905, 1, 6401}, {23, 1880, 3, 6401}, {23, 1892, 3, 6401}, {24, 1881, 6, 6401}, {24, 1893, 3, 6401}, {24, 1907, 3, 6401}, {25, 1882, 3, 6401}, {25, 1908, 3, 6401}, {26, 1884, 1, 6401}, {26, 1895, 3, 6401}, {27, 1885, 3, 6401}, {27, 1896, 6, 6401}, {27, 1910, 3, 6401}, {28, 1886, 3, 6401}, {28, 1897, 3, 6401}, {28, 1911, 6, 6401}, {29, 1887, 1, 6401}, {29, 1912, 3, 6401}, {30, 1899, 1, 6401}, {31, 1900, 3, 6401}, {31, 1914, 1, 6401}, {32, 1901, 3, 6401}, {32, 1915, 3, 6401}, {33, 1902, 1, 6401}, {33, 1916, 3, 6401}, {34, 1917, 1, 6401}, {20, 1876, 1, 6402}, {21, 1878, 3, 6402}, {21, 1891, 1, 6402}, {22, 1879, 3, 6402}, {22, 1906, 1, 6402}, {23, 1881, 3, 6402}, {23, 1893, 3, 6402}, {24, 1882, 6, 6402}, {24, 1894, 3, 6402}, {24, 1908, 3, 6402}, {25, 1883, 3, 6402}, {25, 1909, 3, 6402}, {26, 1885, 1, 6402}, {26, 1896, 3, 6402}, {27, 1886, 3, 6402}, {27, 1897, 6, 6402}, {27, 1911, 3, 6402}, {28, 1887, 3, 6402}, {28, 1898, 3, 6402}, {28, 1912, 6, 6402}, {29, 1888, 1, 6402}, {29, 1913, 3, 6402}, {30, 1900, 1, 6402}, {31, 1901, 3, 6402}, {31, 1915, 1, 6402}, {32, 1902, 3, 6402}, {32, 1916, 3, 6402}, {33, 1903, 1, 6402}, {33, 1917, 3, 6402}, {34, 1918, 1, 6402}, {20, 1889, 1, 6403}, {21, 1890, 3, 6403}, {21, 1919, 1, 6403}, {22, 1891, 3, 6403}, {22, 1934, 1, 6403}, {23, 1892, 3, 6403}, {23, 1920, 3, 6403}, {24, 1893, 6, 6403}, {24, 1921, 3, 6403}, {24, 1935, 3, 6403}, {25, 1894, 3, 6403}, {25, 1936, 3, 6403}, {26, 1895, 1, 6403}, {26, 1922, 3, 6403}, {27, 1896, 3, 6403}, {27, 1923, 6, 6403}, {27, 1937, 3, 6403}, {28, 1897, 3, 6403}, {28, 1924, 3, 6403}, {28, 1938, 6, 6403}, {29, 1898, 1, 6403}, {29, 1939, 3, 6403}, {30, 1925, 1, 6403}, {31, 1926, 3, 6403}, {31, 1940, 1, 6403}, {32, 1927, 3, 6403}, {32, 1941, 3, 6403}, {33, 1928, 1, 6403}, {33, 1942, 3, 6403}, {34, 1943, 1, 6403}, {20, 1890, 1, 6404}, {21, 1892, 3, 6404}, {21, 1920, 1, 6404}, {22, 1893, 3, 6404}, {22, 1935, 1, 6404}, {23, 1895, 3, 6404}, {23, 1922, 3, 6404}, {24, 1896, 6, 6404}, {24, 1923, 3, 6404}, {24, 1937, 3, 6404}, {25, 1897, 3, 6404}, {25, 1938, 3, 6404}, {26, 1899, 1, 6404}, {26, 1925, 3, 6404}, {27, 1900, 3, 6404}, {27, 1926, 6, 6404}, {27, 1940, 3, 6404}, {28, 1901, 3, 6404}, {28, 1927, 3, 6404}, {28, 1941, 6, 6404}, {29, 1902, 1, 6404}, {29, 1942, 3, 6404}, {30, 1929, 1, 6404}, {31, 1930, 3, 6404}, {31, 1944, 1, 6404}, {32, 1931, 3, 6404}, {32, 1945, 3, 6404}, {33, 1932, 1, 6404}, {33, 1946, 3, 6404}, {34, 1947, 1, 6404}, {20, 1891, 1, 6405}, {21, 1893, 3, 6405}, {21, 1921, 1, 6405}, {22, 1894, 3, 6405}, {22, 1936, 1, 6405}, {23, 1896, 3, 6405}, {23, 1923, 3, 6405}, {24, 1897, 6, 6405}, {24, 1924, 3, 6405}, {24, 1938, 3, 6405}, {25, 1898, 3, 6405}, {25, 1939, 3, 6405}, {26, 1900, 1, 6405}, {26, 1926, 3, 6405}, {27, 1901, 3, 6405}, {27, 1927, 6, 6405}, {27, 1941, 3, 6405}, {28, 1902, 3, 6405}, {28, 1928, 3, 6405}, {28, 1942, 6, 6405}, {29, 1903, 1, 6405}, {29, 1943, 3, 6405}, {30, 1930, 1, 6405}, {31, 1931, 3, 6405}, {31, 1945, 1, 6405}, {32, 1932, 3, 6405}, {32, 1946, 3, 6405}, {33, 1933, 1, 6405}, {33, 1947, 3, 6405}, {34, 1948, 1, 6405}, {20, 1904, 1, 6406}, {21, 1905, 3, 6406}, {21, 1934, 1, 6406}, {22, 1906, 3, 6406}, {22, 1949, 1, 6406}, {23, 1907, 3, 6406}, {23, 1935, 3, 6406}, {24, 1908, 6, 6406}, {24, 1936, 3, 6406}, {24, 1950, 3, 6406}, {25, 1909, 3, 6406}, {25, 1951, 3, 6406}, {26, 1910, 1, 6406}, {26, 1937, 3, 6406}, {27, 1911, 3, 6406}, {27, 1938, 6, 6406}, {27, 1952, 3, 6406}, {28, 1912, 3, 6406}, {28, 1939, 3, 6406}, {28, 1953, 6, 6406}, {29, 1913, 1, 6406}, {29, 1954, 3, 6406}, {30, 1940, 1, 6406}, {31, 1941, 3, 6406}, {31, 1955, 1, 6406}, {32, 1942, 3, 6406}, {32, 1956, 3, 6406}, {33, 1943, 1, 6406}, {33, 1957, 3, 6406}, {34, 1958, 1, 6406}, {20, 1905, 1, 6407}, {21, 1907, 3, 6407}, {21, 1935, 1, 6407}, {22, 1908, 3, 6407}, {22, 1950, 1, 6407}, {23, 1910, 3, 6407}, {23, 1937, 3, 6407}, {24, 1911, 6, 6407}, {24, 1938, 3, 6407}, {24, 1952, 3, 6407}, {25, 1912, 3, 6407}, {25, 1953, 3, 6407}, {26, 1914, 1, 6407}, {26, 1940, 3, 6407}, {27, 1915, 3, 6407}, {27, 1941, 6, 6407}, {27, 1955, 3, 6407}, {28, 1916, 3, 6407}, {28, 1942, 3, 6407}, {28, 1956, 6, 6407}, {29, 1917, 1, 6407}, {29, 1957, 3, 6407}, {30, 1944, 1, 6407}, {31, 1945, 3, 6407}, {31, 1959, 1, 6407}, {32, 1946, 3, 6407}, {32, 1960, 3, 6407}, {33, 1947, 1, 6407}, {33, 1961, 3, 6407}, {34, 1962, 1, 6407}, {20, 1906, 1, 6408}, {21, 1908, 3, 6408}, {21, 1936, 1, 6408}, {22, 1909, 3, 6408}, {22, 1951, 1, 6408}, {23, 1911, 3, 6408}, {23, 1938, 3, 6408}, {24, 1912, 6, 6408}, {24, 1939, 3, 6408}, {24, 1953, 3, 6408}, {25, 1913, 3, 6408}, {25, 1954, 3, 6408}, {26, 1915, 1, 6408}, {26, 1941, 3, 6408}, {27, 1916, 3, 6408}, {27, 1942, 6, 6408}, {27, 1956, 3, 6408}, {28, 1917, 3, 6408}, {28, 1943, 3, 6408}, {28, 1957, 6, 6408}, {29, 1918, 1, 6408}, {29, 1958, 3, 6408}, {30, 1945, 1, 6408}, {31, 1946, 3, 6408}, {31, 1960, 1, 6408}, {32, 1947, 3, 6408}, {32, 1961, 3, 6408}, {33, 1948, 1, 6408}, {33, 1962, 3, 6408}, {34, 1963, 1, 6408}, {20, 1988, 1, 6409}, {21, 1989, 3, 6409}, {21, 1998, 1, 6409}, {22, 1990, 3, 6409}, {22, 2008, 1, 6409}, {23, 1991, 3, 6409}, {23, 1999, 3, 6409}, {24, 1992, 6, 6409}, {24, 2000, 3, 6409}, {24, 2009, 3, 6409}, {25, 1993, 3, 6409}, {25, 2010, 3, 6409}, {26, 1994, 1, 6409}, {26, 2001, 3, 6409}, {27, 1995, 3, 6409}, {27, 2002, 6, 6409}, {27, 2011, 3, 6409}, {28, 1996, 3, 6409}, {28, 2003, 3, 6409}, {28, 2012, 6, 6409}, {29, 1997, 1, 6409}, {29, 2013, 3, 6409}, {30, 2004, 1, 6409}, {31, 2005, 3, 6409}, {31, 2014, 1, 6409}, {32, 2006, 3, 6409}, {32, 2015, 3, 6409}, {33, 2007, 1, 6409}, {33, 2016, 3, 6409}, {34, 2017, 1, 6409}, {20, 2018, 1, 6410}, {21, 2019, 3, 6410}, {21, 2033, 1, 6410}, {22, 2020, 3, 6410}, {22, 2048, 1, 6410}, {23, 2021, 3, 6410}, {23, 2034, 3, 6410}, {24, 2022, 6, 6410}, {24, 2035, 3,

6410}, {24, 2049, 3, 6410}, {25, 2023, 3, 6410}, {25, 2050, 3, 6410}, {26, 2024, 1, 6410}, {26, 2036, 3, 6410}, {27, 2025, 3, 6410}, {27, 2037, 6, 6410}, {27, 2051, 3, 6410}, {28, 2026, 3, 6410}, {28, 2038, 3, 6410}, {28, 2052, 6, 6410}, {29, 2027, 1, 6410}, {29, 2053, 3, 6410}, {30, 2039, 1, 6410}, {31, 2040, 3, 6410}, {31, 2054, 1, 6410}, {32, 2041, 3, 6410}, {32, 2055, 3, 6410}, {33, 2042, 1, 6410}, {33, 2056, 3, 6410}, {34, 2057, 1, 6410}, {20, 2019, 1, 6411}, {21, 2021, 3, 6411}, {21, 2034, 1, 6411}, {22, 2022, 3, 6411}, {22, 2049, 1, 6411}, {23, 2024, 3, 6411}, {23, 2036, 3, 6411}, {24, 2025, 6, 6411}, {24, 2037, 3, 6411}, {24, 2051, 3, 6411}, {25, 2026, 3, 6411}, {25, 2052, 3, 6411}, {26, 2028, 1, 6411}, {26, 2039, 3, 6411}, {27, 2029, 3, 6411}, {27, 2040, 6, 6411}, {27, 2054, 3, 6411}, {28, 2030, 3, 6411}, {28, 2041, 3, 6411}, {28, 2055, 6, 6411}, {29, 2031, 1, 6411}, {29, 2056, 3, 6411}, {30, 2043, 1, 6411}, {31, 2044, 3, 6411}, {31, 2058, 1, 6411}, {32, 2045, 3, 6411}, {32, 2059, 3, 6411}, {33, 2046, 1, 6411}, {33, 2060, 3, 6411}, {34, 2061, 1, 6411}, {20, 2020, 1, 6412}, {21, 2022, 3, 6412}, {21, 2035, 1, 6412}, {22, 2023, 3, 6412}, {22, 2050, 1, 6412}, {23, 2025, 3, 6412}, {23, 2037, 3, 6412}, {24, 2026, 6, 6412}, {24, 2038, 3, 6412}, {24, 2052, 3, 6412}, {25, 2027, 3, 6412}, {25, 2053, 3, 6412}, {26, 2029, 1, 6412}, {26, 2040, 3, 6412}, {27, 2030, 3, 6412}, {27, 2041, 6, 6412}, {27, 2055, 3, 6412}, {28, 2031, 3, 6412}, {28, 2042, 3, 6412}, {28, 2056, 6, 6412}, {29, 2032, 1, 6412}, {29, 2057, 3, 6412}, {30, 2044, 1, 6412}, {31, 2045, 3, 6412}, {31, 2059, 1, 6412}, {32, 2046, 3, 6412}, {32, 2060, 3, 6412}, {33, 2047, 1, 6412}, {33, 2061, 3, 6412}, {34, 2062, 1, 6412}, {56, 1376, 1, 6413}, {57, 1377, 2, 6413}, {57, 1382, 1, 6413}, {58, 1378, 2, 6413}, {58, 1388, 1, 6413}, {59, 1379, 1, 6413}, {59, 1383, 2, 6413}, {60, 1380, 2, 6413}, {60, 1384, 2, 6413}, {60, 1389, 2, 6413}, {61, 1381, 1, 6413}, {61, 1390, 2, 6413}, {62, 1385, 1, 6413}, {63, 1386, 2, 6413}, {63, 1391, 1, 6413}, {64, 1387, 1, 6413}, {64, 1392, 2, 6413}, {65, 1393, 1, 6413}, {57, 1376, 1, 6414}, {59, 1377, 2, 6414}, {59, 1382, 1, 6414}, {60, 1378, 2, 6414}, {60, 1388, 1, 6414}, {62, 1379, 1, 6414}, {62, 1383, 2, 6414}, {63, 1380, 2, 6414}, {63, 1384, 2, 6414}, {63, 1389, 2, 6414}, {64, 1381, 1, 6414}, {64, 1390, 2, 6414}, {66, 1385, 1, 6414}, {67, 1386, 2, 6414}, {67, 1391, 1, 6414}, {68, 1387, 1, 6414}, {68, 1392, 2, 6414}, {69, 1393, 1, 6414}, {58, 1376, 1, 6415}, {60, 1377, 2, 6415}, {60, 1382, 1, 6415}, {61, 1378, 2, 6415}, {61, 1388, 1, 6415}, {63, 1379, 1, 6415}, {63, 1383, 2, 6415}, {64, 1380, 2, 6415}, {64, 1384, 2, 6415}, {64, 1389, 2, 6415}, {65, 1381, 1, 6415}, {65, 1390, 2, 6415}, {67, 1385, 1, 6415}, {68, 1386, 2, 6415}, {68, 1391, 1, 6415}, {69, 1387, 1, 6415}, {69, 1392, 2, 6415}, {70, 1393, 1, 6415}, {59, 1376, 1, 6416}, {62, 1377, 2, 6416}, {62, 1382, 1, 6416}, {63, 1378, 2, 6416}, {63, 1388, 1, 6416}, {66, 1379, 1, 6416}, {66, 1383, 2, 6416}, {67, 1380, 2, 6416}, {67, 1384, 2, 6416}, {67, 1389, 2, 6416}, {68, 1381, 1, 6416}, {68, 1390, 2, 6416}, {71, 1385, 1, 6416}, {72, 1386, 2, 6416}, {72, 1391, 1, 6416}, {73, 1387, 1, 6416}, {73, 1392, 2, 6416}, {74, 1393, 1, 6416}, {60, 1376, 1, 6417}, {63, 1377, 2, 6417}, {63, 1382, 1, 6417}, {64, 1378, 2, 6417}, {64, 1388, 1, 6417}, {67, 1379, 1, 6417}, {67, 1383, 2, 6417}, {68, 1380, 2, 6417}, {68, 1384, 2, 6417}, {68, 1389, 2, 6417}, {69, 1381, 1, 6417}, {69, 1390, 2, 6417}, {72, 1385, 1, 6417}, {73, 1386, 2, 6417}, {73, 1391, 1, 6417}, {74, 1387, 1, 6417}, {74, 1392, 2, 6417}, {75, 1393, 1, 6417}, {61, 1376, 1, 6418}, {64, 1377, 2, 6418}, {64, 1382, 1, 6418}, {65, 1378, 2, 6418}, {65, 1388, 1, 6418}, {68, 1379, 1, 6418}, {68, 1383, 2, 6418}, {69, 1380, 2, 6418}, {69, 1384, 2, 6418}, {69, 1389, 2, 6418}, {70, 1381, 1, 6418}, {70, 1390, 2, 6418}, {73, 1385, 1, 6418}, {74, 1386, 2, 6418}, {74, 1391, 1, 6418}, {75, 1387, 1, 6418}, {75, 1392, 2, 6418}, {76, 1393, 1, 6418}, {62, 1376, 1, 6419}, {66, 1377, 2, 6419}, {66, 1382, 1, 6419}, {67, 1378, 2, 6419}, {67, 1388, 1, 6419}, {71, 1379, 1, 6419}, {71, 1383, 2, 6419}, {72, 1380, 2, 6419}, {72, 1384, 2, 6419}, {72, 1389, 2, 6419}, {73, 1381, 1, 6419}, {73, 1390, 2, 6419}, {77, 1385, 1, 6419}, {78, 1386, 2, 6419}, {78, 1391, 1, 6419}, {79, 1387, 1, 6419}, {79, 1392, 2, 6419}, {80, 1393, 1, 6419}, {63, 1376, 1, 6420}, {67, 1377, 2, 6420}, {67, 1382, 1, 6420}, {68, 1378, 2, 6420}, {68, 1388, 1, 6420}, {72, 1379, 1, 6420}, {72, 1383, 2, 6420}, {73, 1380, 2, 6420}, {73, 1384, 2, 6420}, {73, 1389, 2, 6420}, {74, 1381, 1, 6420}, {74, 1390, 2, 6420}, {78, 1385, 1, 6420}, {79, 1386, 2, 6420}, {79, 1391, 1, 6420}, {80, 1387, 1, 6420}, {80, 1392, 2, 6420}, {81, 1393, 1, 6420}, {64, 1376, 1, 6421}, {68, 1377, 2, 6421}, {68, 1382, 1, 6421}, {69, 1378, 2, 6421}, {69, 1388, 1, 6421}, {73, 1379, 1, 6421}, {73, 1383, 2, 6421}, {74, 1380, 2, 6421}, {74, 1384, 2, 6421}, {74, 1389, 2, 6421}, {75, 1381, 1, 6421}, {75, 1390, 2, 6421}, {79, 1385, 1, 6421}, {80, 1386, 2, 6421}, {80, 1391, 1, 6421}, {81, 1387, 1, 6421}, {81, 1392, 2, 6421}, {82, 1393, 1, 6421}, {65, 1376, 1, 6422}, {69, 1377, 2, 6422}, {69, 1382, 1, 6422}, {70, 1378, 2, 6422}, {70, 1388, 1, 6422}, {74, 1379, 1, 6422}, {74, 1383, 2, 6422}, {75, 1380, 2, 6422}, {75, 1384, 2, 6422}, {75, 1389, 2, 6422}, {76, 1381, 1, 6422}, {76, 1390, 2, 6422}, {80, 1385, 1, 6422}, {81, 1386, 2, 6422}, {81, 1391, 1, 6422}, {82, 1387, 1, 6422}, {82, 1392, 2, 6422}, {83, 1393, 1, 6422}, {20, 2119, 1, 6423}, {21, 2120, 4, 6423}, {22, 2121, 4, 6423}, {23, 2122, 3, 6423}, {23, 2129, 3, 6423}, {24, 2123, 6, 6423}, {24, 2130, 6, 6423}, {25, 2124, 3, 6423}, {25, 2138, 3, 6423}, {26, 2125, 1, 6423}, {26, 2131, 3, 6423}, {27, 2126, 3, 6423}, {27, 2132, 6, 6423}, {27, 2139, 3, 6423}, {28, 2127, 3, 6423}, {28, 2133, 3, 6423}, {28, 2140, 6, 6423}, {29, 2128, 1, 6423}, {29, 2141, 3, 6423}, {30, 2134, 1, 6423}, {31, 2135, 3, 6423}, {31, 2142, 1, 6423}, {32, 2136, 3, 6423}, {32, 2143, 3, 6423}, {33, 2137, 1, 6423}, {33, 2144, 3, 6423}, {34, 2145, 1, 6423}, {20, 2120, 1, 6424}, {21, 2122, 1, 6424}, {21, 2129, 3, 6424}, {22, 2123, 1, 6424}, {22, 2130, 3, 6424},

{23, 2131, 6, 6424}, {24, 2132, 9, 6424}, {24, 2139, 3, 6424}, {25, 2133, 3, 6424}, {25, 2140, 3, 6424}, {26, 2134, 1, 6424}, {26, 2146, 3, 6424}, {27, 2135, 3, 6424}, {27, 2147, 9, 6424}, {28, 2136, 3, 6424}, {28, 2148, 3, 6424}, {28, 2153, 6, 6424}, {29, 2137, 1, 6424}, {29, 2154, 3, 6424}, {30, 2149, 1, 6424}, {31, 2150, 3, 6424}, {31, 2155, 1, 6424}, {32, 2151, 3, 6424}, {32, 2156, 3, 6424}, {33, 2152, 1, 6424}, {33, 2157, 3, 6424}, {34, 2158, 1, 6424}, {20, 2121, 1, 6425}, {21, 2123, 1, 6425}, {21, 2130, 3, 6425}, {22, 2124, 1, 6425}, {22, 2138, 3, 6425}, {23, 2132, 3, 6425}, {23, 2139, 3, 6425}, {24, 2133, 3, 6425}, {24, 2140, 9, 6425}, {25, 2141, 6, 6425}, {26, 2142, 1, 6425}, {26, 2147, 3, 6425}, {27, 2143, 3, 6425}, {27, 2148, 3, 6425}, {27, 2153, 6, 6425}, {28, 2144, 3, 6425}, {28, 2154, 9, 6425}, {29, 2145, 1, 6425}, {29, 2159, 3, 6425}, {30, 2155, 1, 6425}, {31, 2156, 3, 6425}, {31, 2160, 1, 6425}, {32, 2157, 3, 6425}, {32, 2161, 3, 6425}, {33, 2158, 1, 6425}, {33, 2162, 3, 6425}, {34, 2163, 1, 6425}, {20, 2122, 1, 6426}, {21, 2125, 1, 6426}, {21, 2131, 3, 6426}, {22, 2126, 1, 6426}, {22, 2139, 3, 6426}, {23, 2134, 3, 6426}, {23, 2146, 3, 6426}, {24, 2135, 3, 6426}, {24, 2142, 3, 6426}, {24, 2147, 6, 6426}, {25, 2143, 3, 6426}, {25, 2148, 3, 6426}, {26, 2149, 4, 6426}, {27, 2150, 6, 6426}, {27, 2155, 6, 6426}, {28, 2151, 3, 6426}, {28, 2156, 6, 6426}, {28, 2160, 3, 6426}, {29, 2152, 1, 6426}, {29, 2161, 3, 6426}, {30, 2164, 1, 6426}, {31, 2165, 4, 6426}, {32, 2166, 3, 6426}, {32, 2168, 3, 6426}, {33, 2167, 1, 6426}, {33, 2169, 3, 6426}, {34, 2170, 1, 6426}, {20, 2123, 1, 6427}, {21, 2126, 1, 6427}, {21, 2132, 3, 6427}, {22, 2127, 1, 6427}, {22, 2140, 3, 6427}, {23, 2135, 3, 6427}, {23, 2147, 3, 6427}, {24, 2136, 3, 6427}, {24, 2143, 3, 6427}, {24, 2153, 6, 6427}, {25, 2144, 3, 6427}, {25, 2154, 3, 6427}, {26, 2150, 3, 6427}, {26, 2155, 1, 6427}, {27, 2151, 3, 6427}, {27, 2156, 9, 6427}, {28, 2157, 9, 6427}, {28, 2161, 3, 6427}, {29, 2158, 1, 6427}, {29, 2162, 3, 6427}, {30, 2165, 1, 6427}, {31, 2166, 1, 6427}, {31, 2168, 3, 6427}, {32, 2169, 6, 6427}, {33, 2170, 1, 6427}, {33, 2171, 3, 6427}, {34, 2172, 1, 6427}, {20, 2124, 1, 6428}, {21, 2127, 1, 6428}, {21, 2133, 3, 6428}, {22, 2128, 1, 6428}, {22, 2141, 3, 6428}, {23, 2136, 3, 6428}, {23, 2148, 3, 6428}, {24, 2137, 3, 6428}, {24, 2144, 3, 6428}, {24, 2154, 6, 6428}, {25, 2145, 3, 6428}, {25, 2159, 3, 6428}, {26, 2151, 3, 6428}, {26, 2160, 1, 6428}, {27, 2152, 3, 6428}, {27, 2157, 6, 6428}, {27, 2161, 3, 6428}, {28, 2158, 6, 6428}, {28, 2162, 6, 6428}, {29, 2163, 4, 6428}, {30, 2166, 1, 6428}, {31, 2167, 1, 6428}, {31, 2169, 3, 6428}, {32, 2170, 3, 6428}, {32, 2171, 3, 6428}, {33, 2172, 4, 6428}, {34, 2173, 1, 6428}, {20, 2195, 1, 6429}, {21, 2196, 3, 6429}, {21, 2205, 1, 6429}, {22, 2197, 3, 6429}, {22, 2215, 1, 6429}, {23, 2198, 3, 6429}, {23, 2206, 3, 6429}, {24, 2199, 6, 6429}, {24, 2207, 3, 6429}, {24, 2216, 3, 6429}, {25, 2200, 3, 6429}, {25, 2217, 3, 6429}, {26, 2201, 1, 6429}, {26, 2208, 3, 6429}, {27, 2202, 3, 6429}, {27, 2209, 6, 6429}, {27, 2218, 3, 6429}, {28, 2203, 3, 6429}, {28, 2210, 3, 6429}, {28, 2219, 6, 6429}, {29, 2204, 1, 6429}, {29, 2220, 3, 6429}, {30, 2211, 1, 6429}, {31, 2212, 3, 6429}, {31, 2221, 1, 6429}, {32, 2213, 3, 6429}, {32, 2222, 3, 6429}, {33, 2214, 1, 6429}, {33, 2223, 3, 6429}, {34, 2224, 1, 6429}, {20, 2205, 1, 6430}, {21, 2206, 3, 6430}, {21, 2225, 1, 6430}, {22, 2207, 3, 6430}, {22, 2235, 1, 6430}, {23, 2208, 3, 6430}, {23, 2226, 3, 6430}, {24, 2209, 6, 6430}, {24, 2227, 3, 6430}, {24, 2236, 3, 6430}, {25, 2210, 3, 6430}, {25, 2237, 3, 6430}, {26, 2211, 1, 6430}, {26, 2228, 3, 6430}, {27, 2212, 3, 6430}, {27, 2229, 6, 6430}, {27, 2238, 3, 6430}, {28, 2213, 3, 6430}, {28, 2230, 3, 6430}, {28, 2239, 6, 6430}, {29, 2214, 1, 6430}, {29, 2240, 3, 6430}, {30, 2231, 1, 6430}, {31, 2232, 3, 6430}, {31, 2241, 1, 6430}, {32, 2233, 3, 6430}, {32, 2242, 3, 6430}, {33, 2234, 1, 6430}, {33, 2243, 3, 6430}, {34, 2244, 1, 6430}, {20, 2215, 1, 6431}, {21, 2216, 3, 6431}, {21, 2235, 1, 6431}, {22, 2217, 3, 6431}, {22, 2245, 1, 6431}, {23, 2218, 3, 6431}, {23, 2236, 3, 6431}, {24, 2219, 6, 6431}, {24, 2237, 3, 6431}, {24, 2246, 3, 6431}, {25, 2220, 3, 6431}, {25, 2247, 3, 6431}, {26, 2221, 1, 6431}, {26, 2238, 3, 6431}, {27, 2222, 3, 6431}, {27, 2239, 6, 6431}, {27, 2248, 3, 6431}, {28, 2223, 3, 6431}, {28, 2240, 3, 6431}, {28, 2249, 6, 6431}, {29, 2224, 1, 6431}, {29, 2250, 3, 6431}, {30, 2241, 1, 6431}, {31, 2242, 3, 6431}, {31, 2251, 1, 6431}, {32, 2243, 3, 6431}, {32, 2252, 3, 6431}, {33, 2244, 1, 6431}, {33, 2253, 3, 6431}, {34, 2254, 1, 6431}, {20, 2369, 1, 6432}, {21, 2370, 3, 6432}, {21, 2379, 1, 6432}, {22, 2371, 3, 6432}, {22, 2389, 1, 6432}, {23, 2372, 3, 6432}, {23, 2380, 3, 6432}, {24, 2373, 6, 6432}, {24, 2381, 3, 6432}, {24, 2390, 3, 6432}, {25, 2374, 3, 6432}, {25, 2391, 3, 6432}, {26, 2375, 1, 6432}, {26, 2382, 3, 6432}, {27, 2376, 3, 6432}, {27, 2383, 6, 6432}, {27, 2392, 3, 6432}, {28, 2377, 3, 6432}, {28, 2384, 3, 6432}, {28, 2393, 6, 6432}, {29, 2378, 1, 6432}, {29, 2394, 3, 6432}, {30, 2385, 1, 6432}, {31, 2386, 3, 6432}, {31, 2395, 1, 6432}, {32, 2387, 3, 6432}, {32, 2396, 3, 6432}, {33, 2388, 1, 6432}, {33, 2397, 3, 6432}, {34, 2398, 1, 6432}, {20, 2639, 1, 6433}, {21, 2640, 3, 6433}, {21, 2649, 1, 6433}, {22, 2641, 3, 6433}, {22, 2659, 1, 6433}, {23, 2642, 3, 6433}, {23, 2650, 3, 6433}, {24, 2643, 6, 6433}, {24, 2651, 3, 6433}, {24, 2660, 3, 6433}, {25, 2644, 3, 6433}, {25, 2661, 3, 6433}, {26, 2645, 1, 6433}, {26, 2652, 3, 6433}, {27, 2646, 3, 6433}, {27, 2653, 6, 6433}, {27, 2662, 3, 6433}, {28, 2647, 3, 6433}, {28, 2654, 3, 6433}, {28, 2663, 6, 6433}, {29, 2648, 1, 6433}, {29, 2664, 3, 6433}, {30, 2655, 1, 6433}, {31, 2656, 3, 6433}, {31, 2665, 1, 6433}, {32, 2657, 3, 6433}, {32, 2666, 3, 6433}, {33, 2658, 1, 6433}, {33, 2667, 3, 6433}, {34, 2668, 1, 6433}, {20, 1073, 1, 6434}, {21, 1074, 4, 6434}, {22, 1075, 4, 6434}, {23, 1076, 2, 6434}, {23, 1079, 4, 6434}, {24, 1077, 4, 6434}, {24, 1080, 8, 6434}, {25, 1078, 2, 6434}, {25, 1084, 4, 6434}, {26, 1081, 4, 6434}, {27, 1082, 8, 6434}, {27, 1085, 4, 6434}, {28, 1083, 4, 6434}, {28,

1086, 8, 6434}, {29, 1087, 4, 6434}, {30, 1088, 1, 6434}, {31, 1089, 4, 6434}, {32, 1090, 2, 6434}, {32, 1091, 4, 6434}, {33, 1092, 4, 6434}, {34, 1093, 1, 6434}, {20, 1094, 1, 6435}, {21, 1095, 2, 6435}, {21, 1104, 2, 6435}, {22, 1096, 2, 6435}, {22, 1114, 2, 6435}, {23, 1097, 1, 6435}, {23, 1105, 4, 6435}, {23, 1124, 1, 6435}, {24, 1098, 2, 6435}, {24, 1106, 4, 6435}, {24, 1115, 4, 6435}, {24, 1134, 2, 6435}, {25, 1099, 1, 6435}, {25, 1116, 4, 6435}, {25, 1144, 1, 6435}, {26, 1107, 2, 6435}, {26, 1125, 2, 6435}, {27, 1108, 4, 6435}, {27, 1117, 2, 6435}, {27, 1126, 2, 6435}, {27, 1135, 4, 6435}, {28, 1109, 2, 6435}, {28, 1118, 4, 6435}, {28, 1136, 4, 6435}, {28, 1145, 2, 6435}, {29, 1119, 2, 6435}, {29, 1146, 2, 6435}, {30, 1127, 1, 6435}, {31, 1128, 2, 6435}, {31, 1137, 2, 6435}, {32, 1129, 1, 6435}, {32, 1138, 4, 6435}, {32, 1147, 1, 6435}, {33, 1139, 2, 6435}, {33, 1148, 2, 6435}, {34, 1149, 1, 6435}, {20, 1095, 1, 6436}, {21, 1097, 2, 6436}, {21, 1105, 2, 6436}, {22, 1098, 2, 6436}, {22, 1115, 2, 6436}, {23, 1100, 1, 6436}, {23, 1107, 4, 6436}, {23, 1125, 1, 6436}, {24, 1101, 2, 6436}, {24, 1108, 4, 6436}, {24, 1117, 4, 6436}, {24, 1135, 2, 6436}, {25, 1102, 1, 6436}, {25, 1118, 4, 6436}, {25, 1145, 1, 6436}, {26, 1110, 2, 6436}, {26, 1127, 2, 6436}, {27, 1111, 4, 6436}, {27, 1120, 2, 6436}, {27, 1128, 2, 6436}, {27, 1137, 4, 6436}, {28, 1112, 2, 6436}, {28, 1121, 4, 6436}, {28, 1138, 4, 6436}, {28, 1147, 2, 6436}, {29, 1122, 2, 6436}, {29, 1148, 2, 6436}, {30, 1130, 1, 6436}, {31, 1131, 2, 6436}, {31, 1140, 2, 6436}, {32, 1132, 1, 6436}, {32, 1141, 4, 6436}, {32, 1150, 1, 6436}, {33, 1142, 2, 6436}, {33, 1151, 2, 6436}, {34, 1152, 1, 6436}, {20, 1096, 1, 6437}, {21, 1098, 2, 6437}, {21, 1106, 2, 6437}, {22, 1099, 2, 6437}, {22, 1116, 2, 6437}, {23, 1101, 1, 6437}, {23, 1108, 4, 6437}, {23, 1126, 1, 6437}, {24, 1102, 2, 6437}, {24, 1109, 4, 6437}, {24, 1118, 4, 6437}, {24, 1136, 2, 6437}, {25, 1103, 1, 6437}, {25, 1119, 4, 6437}, {25, 1146, 1, 6437}, {26, 1111, 2, 6437}, {26, 1128, 2, 6437}, {27, 1112, 4, 6437}, {27, 1121, 2, 6437}, {27, 1129, 2, 6437}, {27, 1138, 4, 6437}, {28, 1113, 2, 6437}, {28, 1122, 4, 6437}, {28, 1139, 4, 6437}, {28, 1148, 2, 6437}, {29, 1123, 2, 6437}, {29, 1149, 2, 6437}, {30, 1131, 1, 6437}, {31, 1132, 2, 6437}, {31, 1141, 2, 6437}, {32, 1133, 1, 6437}, {32, 1142, 4, 6437}, {32, 1151, 1, 6437}, {33, 1143, 2, 6437}, {33, 1152, 2, 6437}, {34, 1153, 1, 6437}, {20, 1154, 1, 6438}, {21, 1155, 2, 6438}, {21, 1169, 2, 6438}, {22, 1156, 2, 6438}, {22, 1184, 2, 6438}, {23, 1157, 1, 6438}, {23, 1170, 4, 6438}, {23, 1199, 1, 6438}, {24, 1158, 2, 6438}, {24, 1171, 4, 6438}, {24, 1185, 4, 6438}, {24, 1214, 2, 6438}, {25, 1159, 1, 6438}, {25, 1186, 4, 6438}, {25, 1229, 1, 6438}, {26, 1172, 2, 6438}, {26, 1200, 2, 6438}, {27, 1173, 4, 6438}, {27, 1187, 2, 6438}, {27, 1201, 2, 6438}, {27, 1215, 4, 6438}, {28, 1174, 2, 6438}, {28, 1188, 4, 6438}, {28, 1216, 4, 6438}, {28, 1230, 2, 6438}, {29, 1189, 2, 6438}, {29, 1231, 2, 6438}, {30, 1202, 1, 6438}, {31, 1203, 2, 6438}, {31, 1217, 2, 6438}, {32, 1204, 1, 6438}, {32, 1218, 4, 6438}, {32, 1232, 1, 6438}, {33, 1219, 2, 6438}, {33, 1233, 2, 6438}, {34, 1234, 1, 6438}, {20, 1155, 1, 6439}, {21, 1157, 2, 6439}, {21, 1170, 2, 6439}, {22, 1158, 2, 6439}, {22, 1185, 2, 6439}, {23, 1160, 1, 6439}, {23, 1172, 4, 6439}, {23, 1200, 1, 6439}, {24, 1161, 2, 6439}, {24, 1173, 4, 6439}, {24, 1187, 4, 6439}, {24, 1215, 2, 6439}, {25, 1162, 1, 6439}, {25, 1188, 4, 6439}, {25, 1230, 1, 6439}, {26, 1175, 2, 6439}, {26, 1202, 2, 6439}, {27, 1176, 4, 6439}, {27, 1190, 2, 6439}, {27, 1203, 2, 6439}, {27, 1217, 4, 6439}, {28, 1177, 2, 6439}, {28, 1191, 4, 6439}, {28, 1218, 4, 6439}, {28, 1232, 2, 6439}, {29, 1192, 2, 6439}, {29, 1233, 2, 6439}, {30, 1205, 1, 6439}, {31, 1206, 2, 6439}, {31, 1220, 2, 6439}, {32, 1207, 1, 6439}, {32, 1221, 4, 6439}, {32, 1235, 1, 6439}, {33, 1222, 2, 6439}, {33, 1236, 2, 6439}, {34, 1237, 1, 6439}, {20, 1156, 1, 6440}, {21, 1158, 2, 6440}, {21, 1171, 2, 6440}, {22, 1159, 2, 6440}, {22, 1186, 2, 6440}, {23, 1161, 1, 6440}, {23, 1173, 4, 6440}, {23, 1201, 1, 6440}, {24, 1162, 2, 6440}, {24, 1174, 4, 6440}, {24, 1188, 4, 6440}, {24, 1216, 2, 6440}, {25, 1163, 1, 6440}, {25, 1189, 4, 6440}, {25, 1231, 1, 6440}, {26, 1176, 2, 6440}, {26, 1203, 2, 6440}, {27, 1177, 4, 6440}, {27, 1191, 2, 6440}, {27, 1204, 2, 6440}, {27, 1218, 4, 6440}, {28, 1178, 2, 6440}, {28, 1192, 4, 6440}, {28, 1219, 4, 6440}, {28, 1233, 2, 6440}, {29, 1193, 2, 6440}, {29, 1234, 2, 6440}, {30, 1206, 1, 6440}, {31, 1207, 2, 6440}, {31, 1221, 2, 6440}, {32, 1208, 1, 6440}, {32, 1222, 4, 6440}, {32, 1236, 1, 6440}, {33, 1223, 2, 6440}, {33, 1237, 2, 6440}, {34, 1238, 1, 6440}, {20, 1157, 1, 6441}, {21, 1160, 2, 6441}, {21, 1172, 2, 6441}, {22, 1161, 2, 6441}, {22, 1187, 2, 6441}, {23, 1164, 1, 6441}, {23, 1175, 4, 6441}, {23, 1202, 1, 6441}, {24, 1165, 2, 6441}, {24, 1176, 4, 6441}, {24, 1190, 4, 6441}, {24, 1217, 2, 6441}, {25, 1166, 1, 6441}, {25, 1191, 4, 6441}, {25, 1232, 1, 6441}, {26, 1179, 2, 6441}, {26, 1205, 2, 6441}, {27, 1180, 4, 6441}, {27, 1194, 2, 6441}, {27, 1206, 2, 6441}, {27, 1220, 4, 6441}, {28, 1181, 2, 6441}, {28, 1195, 4, 6441}, {28, 1221, 4, 6441}, {28, 1235, 2, 6441}, {29, 1196, 2, 6441}, {29, 1236, 2, 6441}, {30, 1209, 1, 6441}, {31, 1210, 2, 6441}, {31, 1224, 2, 6441}, {32, 1211, 1, 6441}, {32, 1225, 4, 6441}, {32, 1239, 1, 6441}, {33, 1226, 2, 6441}, {33, 1240, 2, 6441}, {34, 1241, 1, 6441}, {20, 1158, 1, 6442}, {21, 1161, 2, 6442}, {21, 1173, 2, 6442}, {22, 1162, 2, 6442}, {22, 1188, 2, 6442}, {23, 1165, 1, 6442}, {23, 1176, 4, 6442}, {23, 1203, 1, 6442}, {24, 1166, 2, 6442}, {24, 1177, 4, 6442}, {24, 1191, 4, 6442}, {24, 1218, 2, 6442}, {25, 1167, 1, 6442}, {25, 1192, 4, 6442}, {25, 1233, 1, 6442}, {26, 1180, 2, 6442}, {26, 1206, 2, 6442}, {27, 1181, 4, 6442}, {27, 1195, 2, 6442}, {27, 1207, 2, 6442}, {27, 1221, 4, 6442}, {28, 1182, 2, 6442}, {28, 1196, 4, 6442}, {28, 1222, 4, 6442}, {28, 1236, 2, 6442}, {29, 1197, 2, 6442}, {29, 1237, 2, 6442}, {30, 1210, 1, 6442}, {31, 1211, 2, 6442}, {31, 1225, 2, 6442}, {32, 1212, 1, 6442}, {32, 1226, 4, 6442}, {32, 1240, 1, 6442}, {33, 1227,

2, 6442}, {33, 1241, 2, 6442}, {34, 1242, 1, 6442}, {20, 1159, 1, 6443}, {21, 1162, 2, 6443}, {21, 1174, 2, 6443}, {22, 1163, 2, 6443}, {22, 1189, 2, 6443}, {23, 1166, 1, 6443}, {23, 1177, 4, 6443}, {23, 1204, 1, 6443}, {24, 1167, 2, 6443}, {24, 1178, 4, 6443}, {24, 1192, 4, 6443}, {24, 1219, 2, 6443}, {25, 1168, 1, 6443}, {25, 1193, 4, 6443}, {25, 1234, 1, 6443}, {26, 1181, 2, 6443}, {26, 1207, 2, 6443}, {27, 1182, 4, 6443}, {27, 1196, 2, 6443}, {27, 1208, 2, 6443}, {27, 1222, 4, 6443}, {28, 1183, 2, 6443}, {28, 1197, 4, 6443}, {28, 1223, 4, 6443}, {28, 1237, 2, 6443}, {29, 1198, 2, 6443}, {29, 1238, 2, 6443}, {30, 1211, 1, 6443}, {31, 1212, 2, 6443}, {31, 1226, 2, 6443}, {32, 1213, 1, 6443}, {32, 1227, 4, 6443}, {32, 1241, 1, 6443}, {33, 1228, 2, 6443}, {33, 1242, 2, 6443}, {34, 1243, 1, 6443}, {20, 1244, 1, 6444}, {21, 1245, 2, 6444}, {21, 1265, 2, 6444}, {22, 1246, 2, 6444}, {22, 1286, 2, 6444}, {23, 1247, 1, 6444}, {23, 1266, 4, 6444}, {23, 1307, 1, 6444}, {24, 1248, 2, 6444}, {24, 1267, 4, 6444}, {24, 1287, 4, 6444}, {24, 1328, 2, 6444}, {25, 1249, 1, 6444}, {25, 1288, 4, 6444}, {25, 1349, 1, 6444}, {26, 1268, 2, 6444}, {26, 1308, 2, 6444}, {27, 1269, 4, 6444}, {27, 1289, 2, 6444}, {27, 1309, 2, 6444}, {27, 1329, 4, 6444}, {28, 1270, 2, 6444}, {28, 1290, 4, 6444}, {28, 1330, 4, 6444}, {28, 1350, 2, 6444}, {29, 1291, 2, 6444}, {29, 1351, 2, 6444}, {30, 1310, 1, 6444}, {31, 1311, 2, 6444}, {31, 1331, 2, 6444}, {32, 1312, 1, 6444}, {32, 1332, 4, 6444}, {32, 1352, 1, 6444}, {33, 1333, 2, 6444}, {33, 1353, 2, 6444}, {34, 1354, 1, 6444}, {20, 1245, 1, 6445}, {21, 1247, 2, 6445}, {21, 1266, 2, 6445}, {22, 1248, 2, 6445}, {22, 1287, 2, 6445}, {23, 1250, 1, 6445}, {23, 1268, 4, 6445}, {23, 1308, 1, 6445}, {24, 1251, 2, 6445}, {24, 1269, 4, 6445}, {24, 1289, 4, 6445}, {24, 1329, 2, 6445}, {25, 1252, 1, 6445}, {25, 1290, 4, 6445}, {25, 1350, 1, 6445}, {26, 1271, 2, 6445}, {26, 1310, 2, 6445}, {27, 1272, 4, 6445}, {27, 1292, 2, 6445}, {27, 1311, 2, 6445}, {27, 1331, 4, 6445}, {28, 1273, 2, 6445}, {28, 1293, 4, 6445}, {28, 1332, 4, 6445}, {28, 1352, 2, 6445}, {29, 1294, 2, 6445}, {29, 1353, 2, 6445}, {30, 1313, 1, 6445}, {31, 1314, 2, 6445}, {31, 1334, 2, 6445}, {32, 1315, 1, 6445}, {32, 1335, 4, 6445}, {32, 1355, 1, 6445}, {33, 1336, 2, 6445}, {33, 1356, 2, 6445}, {34, 1357, 1, 6445}, {20, 1246, 1, 6446}, {21, 1248, 2, 6446}, {21, 1267, 2, 6446}, {22, 1249, 2, 6446}, {22, 1288, 2, 6446}, {23, 1251, 1, 6446}, {23, 1269, 4, 6446}, {23, 1309, 1, 6446}, {24, 1252, 2, 6446}, {24, 1270, 4, 6446}, {24, 1290, 4, 6446}, {24, 1330, 2, 6446}, {25, 1253, 1, 6446}, {25, 1291, 4, 6446}, {25, 1351, 1, 6446}, {26, 1272, 2, 6446}, {26, 1311, 2, 6446}, {27, 1273, 4, 6446}, {27, 1293, 2, 6446}, {27, 1312, 2, 6446}, {27, 1332, 4, 6446}, {28, 1274, 2, 6446}, {28, 1294, 4, 6446}, {28, 1333, 4, 6446}, {28, 1353, 2, 6446}, {29, 1295, 2, 6446}, {29, 1354, 2, 6446}, {30, 1314, 1, 6446}, {31, 1315, 2, 6446}, {31, 1335, 2, 6446}, {32, 1316, 1, 6446}, {32, 1336, 4, 6446}, {32, 1356, 1, 6446}, {33, 1337, 2, 6446}, {33, 1357, 2, 6446}, {34, 1358, 1, 6446}, {20, 1247, 1, 6447}, {21, 1250, 2, 6447}, {21, 1268, 2, 6447}, {22, 1251, 2, 6447}, {22, 1289, 2, 6447}, {23, 1254, 1, 6447}, {23, 1271, 4, 6447}, {23, 1310, 1, 6447}, {24, 1255, 2, 6447}, {24, 1272, 4, 6447}, {24, 1292, 4, 6447}, {24, 1331, 2, 6447}, {25, 1256, 1, 6447}, {25, 1293, 4, 6447}, {25, 1352, 1, 6447}, {26, 1275, 2, 6447}, {26, 1313, 2, 6447}, {27, 1276, 4, 6447}, {27, 1296, 2, 6447}, {27, 1314, 2, 6447}, {27, 1334, 4, 6447}, {28, 1277, 2, 6447}, {28, 1297, 4, 6447}, {28, 1335, 4, 6447}, {28, 1355, 2, 6447}, {29, 1298, 2, 6447}, {29, 1356, 2, 6447}, {30, 1317, 1, 6447}, {31, 1318, 2, 6447}, {31, 1338, 2, 6447}, {32, 1319, 1, 6447}, {32, 1339, 4, 6447}, {32, 1359, 1, 6447}, {33, 1340, 2, 6447}, {33, 1360, 2, 6447}, {34, 1361, 1, 6447}, {20, 1248, 1, 6448}, {21, 1251, 2, 6448}, {21, 1269, 2, 6448}, {22, 1252, 2, 6448}, {22, 1290, 2, 6448}, {23, 1255, 1, 6448}, {23, 1272, 4, 6448}, {23, 1311, 1, 6448}, {24, 1256, 2, 6448}, {24, 1273, 4, 6448}, {24, 1293, 4, 6448}, {24, 1332, 2, 6448}, {25, 1257, 1, 6448}, {25, 1294, 4, 6448}, {25, 1353, 1, 6448}, {26, 1276, 2, 6448}, {26, 1314, 2, 6448}, {27, 1277, 4, 6448}, {27, 1297, 2, 6448}, {27, 1315, 2, 6448}, {27, 1335, 4, 6448}, {28, 1278, 2, 6448}, {28, 1298, 4, 6448}, {28, 1336, 4, 6448}, {28, 1356, 2, 6448}, {29, 1299, 2, 6448}, {29, 1357, 2, 6448}, {30, 1318, 1, 6448}, {31, 1319, 2, 6448}, {31, 1339, 2, 6448}, {32, 1320, 1, 6448}, {32, 1340, 4, 6448}, {32, 1360, 1, 6448}, {33, 1341, 2, 6448}, {33, 1361, 2, 6448}, {34, 1362, 1, 6448}, {20, 1249, 1, 6449}, {21, 1252, 2, 6449}, {21, 1270, 2, 6449}, {22, 1253, 2, 6449}, {22, 1291, 2, 6449}, {23, 1256, 1, 6449}, {23, 1273, 4, 6449}, {23, 1312, 1, 6449}, {24, 1257, 2, 6449}, {24, 1274, 4, 6449}, {24, 1294, 4, 6449}, {24, 1333, 2, 6449}, {25, 1258, 1, 6449}, {25, 1295, 4, 6449}, {25, 1354, 1, 6449}, {26, 1277, 2, 6449}, {26, 1315, 2, 6449}, {27, 1278, 4, 6449}, {27, 1298, 2, 6449}, {27, 1316, 2, 6449}, {27, 1336, 4, 6449}, {28, 1279, 2, 6449}, {28, 1299, 4, 6449}, {28, 1337, 4, 6449}, {28, 1357, 2, 6449}, {29, 1300, 2, 6449}, {29, 1358, 2, 6449}, {30, 1319, 1, 6449}, {31, 1320, 2, 6449}, {31, 1340, 2, 6449}, {32, 1321, 1, 6449}, {32, 1341, 4, 6449}, {32, 1361, 1, 6449}, {33, 1342, 2, 6449}, {33, 1362, 2, 6449}, {34, 1363, 1, 6449}, {20, 1250, 1, 6450}, {21, 1254, 2, 6450}, {21, 1271, 2, 6450}, {22, 1255, 2, 6450}, {22, 1292, 2, 6450}, {23, 1259, 1, 6450}, {23, 1275, 4, 6450}, {23, 1313, 1, 6450}, {24, 1260, 2, 6450}, {24, 1276, 4, 6450}, {24, 1296, 4, 6450}, {24, 1334, 2, 6450}, {25, 1261, 1, 6450}, {25, 1297, 4, 6450}, {25, 1355, 1, 6450}, {26, 1280, 2, 6450}, {26, 1317, 2, 6450}, {27, 1281, 4, 6450}, {27, 1301, 2, 6450}, {27, 1318, 2, 6450}, {27, 1338, 4, 6450}, {28, 1282, 2, 6450}, {28, 1302, 4, 6450}, {28, 1339, 4, 6450}, {28, 1359, 2, 6450}, {29, 1303, 2, 6450}, {29, 1360, 2, 6450}, {30, 1322, 1, 6450}, {31, 1323, 2, 6450}, {31, 1343, 2, 6450}, {32, 1324, 1, 6450}, {32, 1344, 4, 6450}, {32, 1364, 1, 6450}, {33, 1345, 2, 6450}, {33, 1365, 2, 6450}, {34, 1366, 1, 6450}, {20, 1251, 1, 6451}, {21, 1255, 2, 6451}, {21, 1272, 2,

6451}, {22, 1256, 2, 6451}, {22, 1293, 2, 6451}, {23, 1260, 1, 6451}, {23, 1276, 4, 6451},
{23, 1314, 1, 6451}, {24, 1261, 2, 6451}, {24, 1277, 4, 6451}, {24, 1297, 4, 6451}, {24,
1335, 2, 6451}, {25, 1262, 1, 6451}, {25, 1298, 4, 6451}, {25, 1356, 1, 6451}, {26, 1281,
2, 6451}, {26, 1318, 2, 6451}, {27, 1282, 4, 6451}, {27, 1302, 2, 6451}, {27, 1319, 2,
6451}, {27, 1339, 4, 6451}, {28, 1283, 2, 6451}, {28, 1303, 4, 6451}, {28, 1340, 4, 6451},
{28, 1360, 2, 6451}, {29, 1304, 2, 6451}, {29, 1361, 2, 6451}, {30, 1323, 1, 6451}, {31,
1324, 2, 6451}, {31, 1344, 2, 6451}, {32, 1325, 1, 6451}, {32, 1345, 4, 6451}, {32, 1365,
1, 6451}, {33, 1346, 2, 6451}, {33, 1366, 2, 6451}, {34, 1367, 1, 6451}, {20, 1252, 1,
6452}, {21, 1256, 2, 6452}, {21, 1273, 2, 6452}, {22, 1257, 2, 6452}, {22, 1294, 2, 6452},
{23, 1261, 1, 6452}, {23, 1277, 4, 6452}, {23, 1315, 1, 6452}, {24, 1262, 2, 6452}, {24,
1278, 4, 6452}, {24, 1298, 4, 6452}, {24, 1336, 2, 6452}, {25, 1263, 1, 6452}, {25, 1299,
4, 6452}, {25, 1357, 1, 6452}, {26, 1282, 2, 6452}, {26, 1319, 2, 6452}, {27, 1283, 4,
6452}, {27, 1303, 2, 6452}, {27, 1320, 2, 6452}, {27, 1340, 4, 6452}, {28, 1284, 2, 6452},
{28, 1304, 4, 6452}, {28, 1341, 4, 6452}, {28, 1361, 2, 6452}, {29, 1305, 2, 6452}, {29,
1362, 2, 6452}, {30, 1324, 1, 6452}, {31, 1325, 2, 6452}, {31, 1345, 2, 6452}, {32, 1326,
1, 6452}, {32, 1346, 4, 6452}, {32, 1366, 1, 6452}, {33, 1347, 2, 6452}, {33, 1367, 2,
6452}, {34, 1368, 1, 6452}, {20, 1253, 1, 6453}, {21, 1257, 2, 6453}, {21, 1274, 2, 6453},
{22, 1258, 2, 6453}, {22, 1295, 2, 6453}, {23, 1262, 1, 6453}, {23, 1278, 4, 6453}, {23,
1316, 1, 6453}, {24, 1263, 2, 6453}, {24, 1279, 4, 6453}, {24, 1299, 4, 6453}, {24, 1337,
2, 6453}, {25, 1264, 1, 6453}, {25, 1300, 4, 6453}, {25, 1358, 1, 6453}, {26, 1283, 2,
6453}, {26, 1320, 2, 6453}, {27, 1284, 4, 6453}, {27, 1304, 2, 6453}, {27, 1321, 2, 6453},
{27, 1341, 4, 6453}, {28, 1285, 2, 6453}, {28, 1305, 4, 6453}, {28, 1342, 4, 6453}, {28,
1362, 2, 6453}, {29, 1306, 2, 6453}, {29, 1363, 2, 6453}, {30, 1325, 1, 6453}, {31, 1326,
2, 6453}, {31, 1346, 2, 6453}, {32, 1327, 1, 6453}, {32, 1347, 4, 6453}, {32, 1367, 1,
6453}, {33, 1348, 2, 6453}, {33, 1368, 2, 6453}, {34, 1369, 1, 6453}, {35, 1094, 1, 6454},
{36, 1095, 1, 6454}, {36, 1104, 2, 6454}, {37, 1096, 1, 6454}, {37, 1114, 2, 6454}, {38,
1105, 2, 6454}, {38, 1124, 1, 6454}, {39, 1106, 2, 6454}, {39, 1115, 2, 6454}, {39, 1134,
2, 6454}, {40, 1116, 2, 6454}, {40, 1144, 1, 6454}, {41, 1125, 1, 6454}, {42, 1126, 1,
6454}, {42, 1135, 2, 6454}, {43, 1136, 2, 6454}, {43, 1145, 1, 6454}, {44, 1146, 1, 6454},
{35, 1095, 1, 6455}, {36, 1097, 1, 6455}, {36, 1105, 2, 6455}, {37, 1098, 1, 6455}, {37,
1115, 2, 6455}, {38, 1107, 2, 6455}, {38, 1125, 1, 6455}, {39, 1108, 2, 6455}, {39, 1117,
2, 6455}, {39, 1135, 2, 6455}, {40, 1118, 2, 6455}, {40, 1145, 1, 6455}, {41, 1127, 1,
6455}, {42, 1128, 1, 6455}, {42, 1137, 2, 6455}, {43, 1138, 2, 6455}, {43, 1147, 1, 6455},
{44, 1148, 1, 6455}, {35, 1096, 1, 6456}, {36, 1098, 1, 6456}, {36, 1106, 2, 6456}, {37,
1099, 1, 6456}, {37, 1116, 2, 6456}, {38, 1108, 2, 6456}, {38, 1126, 1, 6456}, {39, 1109,
2, 6456}, {39, 1118, 2, 6456}, {39, 1136, 2, 6456}, {40, 1119, 2, 6456}, {40, 1146, 1,
6456}, {41, 1128, 1, 6456}, {42, 1129, 1, 6456}, {42, 1138, 2, 6456}, {43, 1139, 2, 6456},
{43, 1148, 1, 6456}, {44, 1149, 1, 6456}, {35, 1097, 1, 6457}, {36, 1100, 1, 6457}, {36,
1107, 2, 6457}, {37, 1101, 1, 6457}, {37, 1117, 2, 6457}, {38, 1110, 2, 6457}, {38, 1127,
1, 6457}, {39, 1111, 2, 6457}, {39, 1120, 2, 6457}, {39, 1137, 2, 6457}, {40, 1121, 2,
6457}, {40, 1147, 1, 6457}, {41, 1130, 1, 6457}, {42, 1131, 1, 6457}, {42, 1140, 2, 6457},
{43, 1141, 2, 6457}, {43, 1150, 1, 6457}, {44, 1151, 1, 6457}, {35, 1098, 1, 6458}, {36,
1101, 1, 6458}, {36, 1108, 2, 6458}, {37, 1102, 1, 6458}, {37, 1118, 2, 6458}, {38, 1111,
2, 6458}, {38, 1128, 1, 6458}, {39, 1112, 2, 6458}, {39, 1121, 2, 6458}, {39, 1138, 2,
6458}, {40, 1122, 2, 6458}, {40, 1148, 1, 6458}, {41, 1131, 1, 6458}, {42, 1132, 1, 6458},
{42, 1141, 2, 6458}, {43, 1142, 2, 6458}, {43, 1151, 1, 6458}, {44, 1152, 1, 6458}, {35,
1099, 1, 6459}, {36, 1102, 1, 6459}, {36, 1109, 2, 6459}, {37, 1103, 1, 6459}, {37, 1119,
2, 6459}, {38, 1112, 2, 6459}, {38, 1129, 1, 6459}, {39, 1113, 2, 6459}, {39, 1122, 2,
6459}, {39, 1139, 2, 6459}, {40, 1123, 2, 6459}, {40, 1149, 1, 6459}, {41, 1132, 1, 6459},
{42, 1133, 1, 6459}, {42, 1142, 2, 6459}, {43, 1143, 2, 6459}, {43, 1152, 1, 6459}, {44,
1153, 1, 6459}, {36, 1094, 1, 6460}, {38, 1095, 1, 6460}, {38, 1104, 2, 6460}, {39, 1096,
1, 6460}, {39, 1114, 2, 6460}, {41, 1105, 2, 6460}, {41, 1124, 1, 6460}, {42, 1106, 2,
6460}, {42, 1115, 2, 6460}, {42, 1134, 2, 6460}, {43, 1116, 2, 6460}, {43, 1144, 1, 6460},
{45, 1125, 1, 6460}, {46, 1126, 1, 6460}, {46, 1135, 2, 6460}, {47, 1136, 2, 6460}, {47,
1145, 1, 6460}, {48, 1146, 1, 6460}, {36, 1095, 1, 6461}, {38, 1097, 1, 6461}, {38, 1105,
2, 6461}, {39, 1098, 1, 6461}, {39, 1115, 2, 6461}, {41, 1107, 2, 6461}, {41, 1125, 1,
6461}, {42, 1108, 2, 6461}, {42, 1117, 2, 6461}, {42, 1135, 2, 6461}, {43, 1118, 2, 6461},
{43, 1145, 1, 6461}, {45, 1127, 1, 6461}, {46, 1128, 1, 6461}, {46, 1137, 2, 6461}, {47,
1138, 2, 6461}, {47, 1147, 1, 6461}, {48, 1148, 1, 6461}, {36, 1096, 1, 6462}, {38, 1098,
1, 6462}, {38, 1106, 2, 6462}, {39, 1099, 1, 6462}, {39, 1116, 2, 6462}, {41, 1108, 2,
6462}, {41, 1126, 1, 6462}, {42, 1109, 2, 6462}, {42, 1118, 2, 6462}, {42, 1136, 2, 6462},
{43, 1119, 2, 6462}, {43, 1146, 1, 6462}, {45, 1128, 1, 6462}, {46, 1129, 1, 6462}, {46,
1138, 2, 6462}, {47, 1139, 2, 6462}, {47, 1148, 1, 6462}, {48, 1149, 1, 6462}, {36, 1097,
1, 6463}, {38, 1100, 1, 6463}, {38, 1107, 2, 6463}, {39, 1101, 1, 6463}, {39, 1117, 2,
6463}, {41, 1110, 2, 6463}, {41, 1127, 1, 6463}, {42, 1111, 2, 6463}, {42, 1120, 2, 6463},
{42, 1137, 2, 6463}, {43, 1121, 2, 6463}, {43, 1147, 1, 6463}, {45, 1130, 1, 6463}, {46,
1131, 1, 6463}, {46, 1140, 2, 6463}, {47, 1141, 2, 6463}, {47, 1150, 1, 6463}, {48, 1151,
1, 6463}, {36, 1098, 1, 6464}, {38, 1101, 1, 6464}, {38, 1108, 2, 6464}, {39, 1102, 1,
6464}, {39, 1118, 2, 6464}, {41, 1111, 2, 6464}, {41, 1128, 1, 6464}, {42, 1112, 2, 6464},

{42, 1121, 2, 6464}, {42, 1138, 2, 6464}, {43, 1122, 2, 6464}, {43, 1148, 1, 6464}, {45, 1131, 1, 6464}, {46, 1132, 1, 6464}, {46, 1141, 2, 6464}, {47, 1142, 2, 6464}, {47, 1151, 1, 6464}, {48, 1152, 1, 6464}, {36, 1099, 1, 6465}, {38, 1102, 1, 6465}, {38, 1109, 2, 6465}, {39, 1103, 1, 6465}, {39, 1119, 2, 6465}, {41, 1112, 2, 6465}, {41, 1129, 1, 6465}, {42, 1113, 2, 6465}, {42, 1122, 2, 6465}, {42, 1139, 2, 6465}, {43, 1123, 2, 6465}, {43, 1149, 1, 6465}, {45, 1132, 1, 6465}, {46, 1133, 1, 6465}, {46, 1142, 2, 6465}, {47, 1143, 2, 6465}, {47, 1152, 1, 6465}, {48, 1153, 1, 6465}, {37, 1094, 1, 6466}, {39, 1095, 1, 6466}, {39, 1104, 2, 6466}, {40, 1096, 1, 6466}, {40, 1114, 2, 6466}, {42, 1105, 2, 6466}, {42, 1124, 1, 6466}, {43, 1106, 2, 6466}, {43, 1115, 2, 6466}, {43, 1134, 2, 6466}, {44, 1116, 2, 6466}, {44, 1144, 1, 6466}, {46, 1125, 1, 6466}, {47, 1126, 1, 6466}, {47, 1135, 2, 6466}, {48, 1136, 2, 6466}, {48, 1145, 1, 6466}, {49, 1146, 1, 6466}, {37, 1095, 1, 6467}, {39, 1097, 1, 6467}, {39, 1105, 2, 6467}, {40, 1098, 1, 6467}, {40, 1115, 2, 6467}, {42, 1107, 2, 6467}, {42, 1125, 1, 6467}, {43, 1108, 2, 6467}, {43, 1117, 2, 6467}, {43, 1135, 2, 6467}, {44, 1118, 2, 6467}, {44, 1145, 1, 6467}, {46, 1127, 1, 6467}, {47, 1128, 1, 6467}, {47, 1137, 2, 6467}, {48, 1138, 2, 6467}, {48, 1147, 1, 6467}, {49, 1148, 1, 6467}, {37, 1096, 1, 6468}, {39, 1098, 1, 6468}, {39, 1106, 2, 6468}, {40, 1099, 1, 6468}, {40, 1116, 2, 6468}, {42, 1108, 2, 6468}, {42, 1126, 1, 6468}, {43, 1109, 2, 6468}, {43, 1118, 2, 6468}, {43, 1136, 2, 6468}, {44, 1119, 2, 6468}, {44, 1146, 1, 6468}, {46, 1128, 1, 6468}, {47, 1129, 1, 6468}, {47, 1138, 2, 6468}, {48, 1139, 2, 6468}, {48, 1148, 1, 6468}, {49, 1149, 1, 6468}, {37, 1097, 1, 6469}, {39, 1100, 1, 6469}, {39, 1107, 2, 6469}, {40, 1101, 1, 6469}, {40, 1117, 2, 6469}, {42, 1110, 2, 6469}, {42, 1127, 1, 6469}, {43, 1111, 2, 6469}, {43, 1120, 2, 6469}, {43, 1137, 2, 6469}, {44, 1121, 2, 6469}, {44, 1147, 1, 6469}, {46, 1130, 1, 6469}, {47, 1131, 1, 6469}, {47, 1140, 2, 6469}, {48, 1141, 2, 6469}, {48, 1150, 1, 6469}, {49, 1151, 1, 6469}, {37, 1098, 1, 6470}, {39, 1101, 1, 6470}, {39, 1108, 2, 6470}, {40, 1102, 1, 6470}, {40, 1118, 2, 6470}, {42, 1111, 2, 6470}, {42, 1128, 1, 6470}, {43, 1112, 2, 6470}, {43, 1121, 2, 6470}, {43, 1138, 2, 6470}, {44, 1122, 2, 6470}, {44, 1148, 1, 6470}, {46, 1131, 1, 6470}, {47, 1132, 1, 6470}, {47, 1141, 2, 6470}, {48, 1142, 2, 6470}, {48, 1151, 1, 6470}, {49, 1152, 1, 6470}, {37, 1099, 1, 6471}, {39, 1102, 1, 6471}, {39, 1109, 2, 6471}, {40, 1103, 1, 6471}, {40, 1119, 2, 6471}, {42, 1112, 2, 6471}, {42, 1129, 1, 6471}, {43, 1113, 2, 6471}, {43, 1122, 2, 6471}, {43, 1139, 2, 6471}, {44, 1123, 2, 6471}, {44, 1149, 1, 6471}, {46, 1132, 1, 6471}, {47, 1133, 1, 6471}, {47, 1142, 2, 6471}, {48, 1143, 2, 6471}, {48, 1152, 1, 6471}, {49, 1153, 1, 6471}, {38, 1094, 1, 6472}, {41, 1095, 1, 6472}, {41, 1104, 2, 6472}, {42, 1096, 1, 6472}, {42, 1114, 2, 6472}, {45, 1105, 2, 6472}, {45, 1124, 1, 6472}, {46, 1106, 2, 6472}, {46, 1115, 2, 6472}, {46, 1134, 2, 6472}, {47, 1116, 2, 6472}, {47, 1144, 1, 6472}, {50, 1125, 1, 6472}, {51, 1126, 1, 6472}, {51, 1135, 2, 6472}, {52, 1136, 2, 6472}, {52, 1145, 1, 6472}, {53, 1146, 1, 6472}, {38, 1095, 1, 6473}, {41, 1097, 1, 6473}, {41, 1105, 2, 6473}, {42, 1098, 1, 6473}, {42, 1115, 2, 6473}, {45, 1107, 2, 6473}, {45, 1125, 1, 6473}, {46, 1108, 2, 6473}, {46, 1117, 2, 6473}, {46, 1135, 2, 6473}, {47, 1118, 2, 6473}, {47, 1145, 1, 6473}, {50, 1127, 1, 6473}, {51, 1128, 1, 6473}, {51, 1137, 2, 6473}, {52, 1138, 2, 6473}, {52, 1147, 1, 6473}, {53, 1148, 1, 6473}, {38, 1096, 1, 6474}, {41, 1098, 1, 6474}, {41, 1106, 2, 6474}, {42, 1099, 1, 6474}, {42, 1116, 2, 6474}, {45, 1108, 2, 6474}, {45, 1126, 1, 6474}, {46, 1109, 2, 6474}, {46, 1118, 2, 6474}, {46, 1136, 2, 6474}, {47, 1119, 2, 6474}, {47, 1146, 1, 6474}, {50, 1128, 1, 6474}, {51, 1129, 1, 6474}, {51, 1138, 2, 6474}, {52, 1139, 2, 6474}, {52, 1148, 1, 6474}, {53, 1149, 1, 6474}, {38, 1097, 1, 6475}, {41, 1100, 1, 6475}, {41, 1107, 2, 6475}, {42, 1101, 1, 6475}, {42, 1117, 2, 6475}, {45, 1110, 2, 6475}, {45, 1127, 1, 6475}, {46, 1111, 2, 6475}, {46, 1120, 2, 6475}, {46, 1137, 2, 6475}, {47, 1121, 2, 6475}, {47, 1147, 1, 6475}, {50, 1130, 1, 6475}, {51, 1131, 1, 6475}, {51, 1140, 2, 6475}, {52, 1141, 2, 6475}, {52, 1150, 1, 6475}, {53, 1151, 1, 6475}, {38, 1098, 1, 6476}, {41, 1101, 1, 6476}, {41, 1108, 2, 6476}, {42, 1102, 1, 6476}, {42, 1118, 2, 6476}, {45, 1111, 2, 6476}, {45, 1128, 1, 6476}, {46, 1112, 2, 6476}, {46, 1121, 2, 6476}, {46, 1138, 2, 6476}, {47, 1122, 2, 6476}, {47, 1148, 1, 6476}, {50, 1131, 1, 6476}, {51, 1132, 1, 6476}, {51, 1141, 2, 6476}, {52, 1142, 2, 6476}, {52, 1151, 1, 6476}, {53, 1152, 1, 6476}, {38, 1099, 1, 6477}, {41, 1102, 1, 6477}, {41, 1109, 2, 6477}, {42, 1103, 1, 6477}, {42, 1119, 2, 6477}, {45, 1112, 2, 6477}, {45, 1129, 1, 6477}, {46, 1113, 2, 6477}, {46, 1122, 2, 6477}, {46, 1139, 2, 6477}, {47, 1123, 2, 6477}, {47, 1149, 1, 6477}, {50, 1132, 1, 6477}, {51, 1133, 1, 6477}, {51, 1142, 2, 6477}, {52, 1143, 2, 6477}, {52, 1152, 1, 6477}, {53, 1153, 1, 6477}, {39, 1094, 1, 6478}, {42, 1095, 1, 6478}, {42, 1104, 2, 6478}, {43, 1096, 1, 6478}, {43, 1114, 2, 6478}, {46, 1105, 2, 6478}, {46, 1124, 1, 6478}, {47, 1106, 2, 6478}, {47, 1115, 2, 6478}, {47, 1134, 2, 6478}, {48, 1116, 2, 6478}, {48, 1144, 1, 6478}, {51, 1125, 1, 6478}, {52, 1126, 1, 6478}, {52, 1135, 2, 6478}, {53, 1136, 2, 6478}, {53, 1145, 1, 6478}, {54, 1146, 1, 6478}, {39, 1095, 1, 6479}, {42, 1097, 1, 6479}, {42, 1105, 2, 6479}, {43, 1098, 1, 6479}, {43, 1115, 2, 6479}, {46, 1107, 2, 6479}, {46, 1125, 1, 6479}, {47, 1108, 2, 6479}, {47, 1117, 2, 6479}, {47, 1135, 2, 6479}, {48, 1118, 2, 6479}, {48, 1145, 1, 6479}, {51, 1127, 1, 6479}, {52, 1128, 1, 6479}, {52, 1137, 2, 6479}, {53, 1138, 2, 6479}, {53, 1147, 1, 6479}, {54, 1148, 1, 6479}, {39, 1096, 1, 6480}, {42, 1098, 1, 6480}, {42, 1106, 2, 6480}, {43, 1099, 1, 6480}, {43, 1116, 2, 6480}, {46, 1108, 2, 6480}, {46, 1126, 1, 6480}, {47, 1109, 2, 6480}, {47, 1118, 2, 6480}, {47, 1136, 2, 6480}, {48, 1119, 2, 6480}, {48, 1146, 1, 6480}, {51, 1128, 1, 6480}, {52,

1129, 1, 6480}, {52, 1138, 2, 6480}, {53, 1139, 2, 6480}, {53, 1148, 1, 6480}, {54, 1149,
1, 6480}, {39, 1097, 1, 6481}, {42, 1100, 1, 6481}, {42, 1107, 2, 6481}, {43, 1101, 1,
6481}, {43, 1117, 2, 6481}, {46, 1110, 2, 6481}, {46, 1127, 1, 6481}, {47, 1111, 2, 6481},
{47, 1120, 2, 6481}, {47, 1137, 2, 6481}, {48, 1121, 2, 6481}, {48, 1147, 1, 6481}, {51,
1130, 1, 6481}, {52, 1131, 1, 6481}, {52, 1140, 2, 6481}, {53, 1141, 2, 6481}, {53, 1150,
1, 6481}, {54, 1151, 1, 6481}, {39, 1098, 1, 6482}, {42, 1101, 1, 6482}, {42, 1108, 2,
6482}, {43, 1102, 1, 6482}, {43, 1118, 2, 6482}, {46, 1111, 2, 6482}, {46, 1128, 1, 6482},
{47, 1112, 2, 6482}, {47, 1121, 2, 6482}, {47, 1138, 2, 6482}, {48, 1122, 2, 6482}, {48,
1148, 1, 6482}, {51, 1131, 1, 6482}, {52, 1132, 1, 6482}, {52, 1141, 2, 6482}, {53, 1142,
2, 6482}, {53, 1151, 1, 6482}, {54, 1152, 1, 6482}, {39, 1099, 1, 6483}, {42, 1102, 1,
6483}, {42, 1109, 2, 6483}, {43, 1103, 1, 6483}, {43, 1119, 2, 6483}, {46, 1112, 2, 6483},
{46, 1129, 1, 6483}, {47, 1113, 2, 6483}, {47, 1122, 2, 6483}, {47, 1139, 2, 6483}, {48,
1123, 2, 6483}, {48, 1149, 1, 6483}, {51, 1132, 1, 6483}, {52, 1133, 1, 6483}, {52, 1142,
2, 6483}, {53, 1143, 2, 6483}, {53, 1152, 1, 6483}, {54, 1153, 1, 6483}, {40, 1094, 1,
6484}, {43, 1095, 1, 6484}, {43, 1104, 2, 6484}, {44, 1096, 1, 6484}, {44, 1114, 2, 6484},
{47, 1105, 2, 6484}, {47, 1124, 1, 6484}, {48, 1106, 2, 6484}, {48, 1115, 2, 6484}, {48,
1134, 2, 6484}, {49, 1116, 2, 6484}, {49, 1144, 1, 6484}, {52, 1125, 1, 6484}, {53, 1126,
1, 6484}, {53, 1135, 2, 6484}, {54, 1136, 2, 6484}, {54, 1145, 1, 6484}, {55, 1146, 1,
6484}, {40, 1095, 1, 6485}, {43, 1097, 1, 6485}, {43, 1105, 2, 6485}, {44, 1098, 1, 6485},
{44, 1115, 2, 6485}, {47, 1107, 2, 6485}, {47, 1125, 1, 6485}, {48, 1108, 2, 6485}, {48,
1117, 2, 6485}, {48, 1135, 2, 6485}, {49, 1118, 2, 6485}, {49, 1145, 1, 6485}, {52, 1127,
1, 6485}, {53, 1128, 1, 6485}, {53, 1137, 2, 6485}, {54, 1138, 2, 6485}, {54, 1147, 1,
6485}, {55, 1148, 1, 6485}, {40, 1096, 1, 6486}, {43, 1098, 1, 6486}, {43, 1106, 2, 6486},
{44, 1099, 1, 6486}, {44, 1116, 2, 6486}, {47, 1108, 2, 6486}, {47, 1126, 1, 6486}, {48,
1109, 2, 6486}, {48, 1118, 2, 6486}, {48, 1136, 2, 6486}, {49, 1119, 2, 6486}, {49, 1146,
1, 6486}, {52, 1128, 1, 6486}, {53, 1129, 1, 6486}, {53, 1138, 2, 6486}, {54, 1139, 2,
6486}, {54, 1148, 1, 6486}, {55, 1149, 1, 6486}, {40, 1097, 1, 6487}, {43, 1100, 1, 6487},
{43, 1107, 2, 6487}, {44, 1101, 1, 6487}, {44, 1117, 2, 6487}, {47, 1110, 2, 6487}, {47,
1127, 1, 6487}, {48, 1111, 2, 6487}, {48, 1120, 2, 6487}, {48, 1137, 2, 6487}, {49, 1121,
2, 6487}, {49, 1147, 1, 6487}, {52, 1130, 1, 6487}, {53, 1131, 1, 6487}, {53, 1140, 2,
6487}, {54, 1141, 2, 6487}, {54, 1150, 1, 6487}, {55, 1151, 1, 6487}, {40, 1098, 1, 6488},
{43, 1101, 1, 6488}, {43, 1108, 2, 6488}, {44, 1102, 1, 6488}, {44, 1118, 2, 6488}, {47,
1111, 2, 6488}, {47, 1128, 1, 6488}, {48, 1112, 2, 6488}, {48, 1121, 2, 6488}, {48, 1138,
2, 6488}, {49, 1122, 2, 6488}, {49, 1148, 1, 6488}, {52, 1131, 1, 6488}, {53, 1132, 1,
6488}, {53, 1141, 2, 6488}, {54, 1142, 2, 6488}, {54, 1151, 1, 6488}, {55, 1152, 1, 6488},
{40, 1099, 1, 6489}, {43, 1102, 1, 6489}, {43, 1109, 2, 6489}, {44, 1103, 1, 6489}, {44,
1119, 2, 6489}, {47, 1112, 2, 6489}, {47, 1129, 1, 6489}, {48, 1113, 2, 6489}, {48, 1122,
2, 6489}, {48, 1139, 2, 6489}, {49, 1123, 2, 6489}, {49, 1149, 1, 6489}, {52, 1132, 1,
6489}, {53, 1133, 1, 6489}, {53, 1142, 2, 6489}, {54, 1143, 2, 6489}, {54, 1152, 1, 6489},
{55, 1153, 1, 6489}, {20, 1588, 1, 6490}, {21, 1589, 4, 6490}, {22, 1590, 4, 6490}, {23,
1591, 2, 6490}, {23, 1598, 4, 6490}, {24, 1592, 4, 6490}, {24, 1599, 8, 6490}, {25, 1593,
2, 6490}, {25, 1607, 4, 6490}, {26, 1600, 4, 6490}, {27, 1601, 8, 6490}, {27, 1608, 4,
6490}, {28, 1602, 4, 6490}, {28, 1609, 8, 6490}, {29, 1610, 4, 6490}, {30, 1615, 1, 6490},
{31, 1616, 4, 6490}, {32, 1617, 2, 6490}, {32, 1622, 4, 6490}, {33, 1623, 4, 6490}, {34,
1628, 1, 6490}, {20, 1589, 1, 6491}, {21, 1591, 2, 6491}, {21, 1598, 2, 6491}, {22, 1592,
2, 6491}, {22, 1599, 2, 6491}, {23, 1594, 1, 6491}, {23, 1600, 5, 6491}, {24, 1595, 2,
6491}, {24, 1601, 6, 6491}, {24, 1608, 4, 6491}, {25, 1596, 1, 6491}, {25, 1602, 1, 6491},
{25, 1609, 4, 6491}, {26, 1603, 2, 6491}, {26, 1615, 2, 6491}, {27, 1604, 4, 6491}, {27,
1611, 2, 6491}, {27, 1616, 6, 6491}, {28, 1605, 2, 6491}, {28, 1612, 4, 6491}, {28, 1617,
2, 6491}, {28, 1622, 4, 6491}, {29, 1613, 2, 6491}, {29, 1623, 2, 6491}, {30, 1618, 1,
6491}, {31, 1619, 2, 6491}, {31, 1624, 2, 6491}, {32, 1620, 1, 6491}, {32, 1625, 4, 6491},
{32, 1629, 1, 6491}, {33, 1626, 2, 6491}, {33, 1630, 2, 6491}, {34, 1631, 1, 6491}, {20,
1590, 1, 6492}, {21, 1592, 2, 6492}, {21, 1599, 2, 6492}, {22, 1593, 2, 6492}, {22, 1607,
2, 6492}, {23, 1595, 1, 6492}, {23, 1601, 4, 6492}, {23, 1608, 1, 6492}, {24, 1596, 2,
6492}, {24, 1602, 4, 6492}, {24, 1609, 6, 6492}, {25, 1597, 1, 6492}, {25, 1610, 5, 6492},
{26, 1604, 2, 6492}, {26, 1616, 2, 6492}, {27, 1605, 4, 6492}, {27, 1612, 2, 6492}, {27,
1617, 2, 6492}, {27, 1622, 4, 6492}, {28, 1606, 2, 6492}, {28, 1613, 4, 6492}, {28, 1623,
6, 6492}, {29, 1614, 2, 6492}, {29, 1628, 2, 6492}, {30, 1619, 1, 6492}, {31, 1620, 2,
6492}, {31, 1625, 2, 6492}, {32, 1621, 1, 6492}, {32, 1626, 4, 6492}, {32, 1630, 1, 6492},
{33, 1627, 2, 6492}, {33, 1631, 2, 6492}, {34, 1632, 1, 6492}, {20, 1598, 1, 6493}, {21,
1600, 4, 6493}, {22, 1601, 4, 6493}, {23, 1603, 2, 6493}, {23, 1615, 4, 6493}, {24, 1604,
4, 6493}, {24, 1616, 8, 6493}, {25, 1605, 2, 6493}, {25, 1622, 4, 6493}, {26, 1618, 4,
6493}, {27, 1619, 8, 6493}, {27, 1624, 4, 6493}, {28, 1620, 4, 6493}, {28, 1625, 8, 6493},
{29, 1626, 4, 6493}, {30, 1633, 1, 6493}, {31, 1634, 4, 6493}, {32, 1635, 2, 6493}, {32,
1637, 4, 6493}, {33, 1638, 4, 6493}, {34, 1640, 1, 6493}, {20, 1599, 1, 6494}, {21, 1601,
2, 6494}, {21, 1608, 2, 6494}, {22, 1602, 2, 6494}, {22, 1609, 2, 6494}, {23, 1604, 1,
6494}, {23, 1611, 1, 6494}, {23, 1616, 4, 6494}, {24, 1605, 2, 6494}, {24, 1612, 2, 6494},
{24, 1617, 4, 6494}, {24, 1622, 4, 6494}, {25, 1606, 1, 6494}, {25, 1613, 1, 6494}, {25,
1623, 4, 6494}, {26, 1619, 2, 6494}, {26, 1624, 2, 6494}, {27, 1620, 4, 6494}, {27, 1625,

6, 6494}, {27, 1629, 2, 6494}, {28, 1621, 2, 6494}, {28, 1626, 6, 6494}, {28, 1630, 4, 6494}, {29, 1627, 2, 6494}, {29, 1631, 2, 6494}, {30, 1634, 1, 6494}, {31, 1635, 2, 6494}, {31, 1637, 2, 6494}, {32, 1636, 1, 6494}, {32, 1638, 5, 6494}, {33, 1639, 2, 6494}, {33, 1640, 2, 6494}, {34, 1641, 1, 6494}, {20, 1607, 1, 6495}, {21, 1609, 4, 6495}, {22, 1610, 4, 6495}, {23, 1612, 2, 6495}, {23, 1622, 4, 6495}, {24, 1613, 4, 6495}, {24, 1623, 8, 6495}, {25, 1614, 2, 6495}, {25, 1628, 4, 6495}, {26, 1625, 4, 6495}, {27, 1626, 8, 6495}, {27, 1630, 4, 6495}, {28, 1627, 4, 6495}, {28, 1631, 8, 6495}, {29, 1632, 4, 6495}, {30, 1637, 1, 6495}, {31, 1638, 4, 6495}, {32, 1639, 2, 6495}, {32, 1640, 4, 6495}, {33, 1641, 4, 6495}, {34, 1642, 1, 6495}, {20, 1643, 1, 6496}, {21, 1644, 2, 6496}, {21, 1658, 2, 6496}, {22, 1645, 2, 6496}, {22, 1673, 2, 6496}, {23, 1646, 1, 6496}, {23, 1659, 4, 6496}, {23, 1688, 1, 6496}, {24, 1647, 2, 6496}, {24, 1660, 4, 6496}, {24, 1674, 4, 6496}, {24, 1703, 2, 6496}, {25, 1648, 1, 6496}, {25, 1675, 4, 6496}, {25, 1718, 1, 6496}, {26, 1661, 2, 6496}, {26, 1689, 2, 6496}, {27, 1662, 4, 6496}, {27, 1676, 2, 6496}, {27, 1690, 2, 6496}, {27, 1704, 4, 6496}, {28, 1663, 2, 6496}, {28, 1677, 4, 6496}, {28, 1705, 4, 6496}, {28, 1719, 2, 6496}, {29, 1678, 2, 6496}, {29, 1720, 2, 6496}, {30, 1691, 1, 6496}, {31, 1692, 2, 6496}, {31, 1706, 2, 6496}, {32, 1693, 1, 6496}, {32, 1707, 4, 6496}, {32, 1721, 1, 6496}, {33, 1708, 2, 6496}, {33, 1722, 2, 6496}, {34, 1723, 1, 6496}, {20, 1644, 1, 6497}, {21, 1646, 2, 6497}, {21, 1659, 2, 6497}, {22, 1647, 2, 6497}, {22, 1674, 2, 6497}, {23, 1649, 1, 6497}, {23, 1661, 4, 6497}, {23, 1689, 1, 6497}, {24, 1650, 2, 6497}, {24, 1662, 4, 6497}, {24, 1676, 4, 6497}, {24, 1704, 2, 6497}, {25, 1651, 1, 6497}, {25, 1677, 4, 6497}, {25, 1719, 1, 6497}, {26, 1664, 2, 6497}, {26, 1691, 2, 6497}, {27, 1665, 4, 6497}, {27, 1679, 2, 6497}, {27, 1692, 2, 6497}, {27, 1706, 4, 6497}, {28, 1666, 2, 6497}, {28, 1680, 4, 6497}, {28, 1707, 4, 6497}, {28, 1721, 2, 6497}, {29, 1681, 2, 6497}, {29, 1722, 2, 6497}, {30, 1694, 1, 6497}, {31, 1695, 2, 6497}, {31, 1709, 2, 6497}, {32, 1696, 1, 6497}, {32, 1710, 4, 6497}, {32, 1724, 1, 6497}, {33, 1711, 2, 6497}, {33, 1725, 2, 6497}, {34, 1726, 1, 6497}, {20, 1645, 1, 6498}, {21, 1647, 2, 6498}, {21, 1660, 2, 6498}, {22, 1648, 2, 6498}, {22, 1675, 2, 6498}, {23, 1650, 1, 6498}, {23, 1662, 4, 6498}, {23, 1690, 1, 6498}, {24, 1651, 2, 6498}, {24, 1663, 4, 6498}, {24, 1677, 4, 6498}, {24, 1705, 2, 6498}, {25, 1652, 1, 6498}, {25, 1678, 4, 6498}, {25, 1720, 1, 6498}, {26, 1665, 2, 6498}, {26, 1692, 2, 6498}, {27, 1666, 4, 6498}, {27, 1680, 2, 6498}, {27, 1693, 2, 6498}, {27, 1707, 4, 6498}, {28, 1667, 2, 6498}, {28, 1681, 4, 6498}, {28, 1708, 4, 6498}, {28, 1722, 2, 6498}, {29, 1682, 2, 6498}, {29, 1723, 2, 6498}, {30, 1695, 1, 6498}, {31, 1696, 2, 6498}, {31, 1710, 2, 6498}, {32, 1697, 1, 6498}, {32, 1711, 4, 6498}, {32, 1725, 1, 6498}, {33, 1712, 2, 6498}, {33, 1726, 2, 6498}, {34, 1727, 1, 6498}, {20, 1646, 1, 6499}, {21, 1649, 2, 6499}, {21, 1661, 2, 6499}, {22, 1650, 2, 6499}, {22, 1676, 2, 6499}, {23, 1653, 1, 6499}, {23, 1664, 4, 6499}, {23, 1691, 1, 6499}, {24, 1654, 2, 6499}, {24, 1665, 4, 6499}, {24, 1679, 4, 6499}, {24, 1706, 2, 6499}, {25, 1655, 1, 6499}, {25, 1680, 4, 6499}, {25, 1721, 1, 6499}, {26, 1668, 2, 6499}, {26, 1694, 2, 6499}, {27, 1669, 4, 6499}, {27, 1683, 2, 6499}, {27, 1695, 2, 6499}, {27, 1709, 4, 6499}, {28, 1670, 2, 6499}, {28, 1684, 4, 6499}, {28, 1710, 4, 6499}, {28, 1724, 2, 6499}, {29, 1685, 2, 6499}, {29, 1725, 2, 6499}, {30, 1698, 1, 6499}, {31, 1699, 2, 6499}, {31, 1713, 2, 6499}, {32, 1700, 1, 6499}, {32, 1714, 4, 6499}, {32, 1728, 1, 6499}, {33, 1715, 2, 6499}, {33, 1729, 2, 6499}, {34, 1730, 1, 6499}, {20, 1647, 1, 6500}, {21, 1650, 2, 6500}, {21, 1662, 2, 6500}, {22, 1651, 2, 6500}, {22, 1677, 2, 6500}, {23, 1654, 1, 6500}, {23, 1665, 4, 6500}, {23, 1692, 1, 6500}, {24, 1655, 2, 6500}, {24, 1666, 4, 6500}, {24, 1680, 4, 6500}, {24, 1707, 2, 6500}, {25, 1656, 1, 6500}, {25, 1681, 4, 6500}, {25, 1722, 1, 6500}, {26, 1669, 2, 6500}, {26, 1695, 2, 6500}, {27, 1670, 4, 6500}, {27, 1684, 2, 6500}, {27, 1696, 2, 6500}, {27, 1710, 4, 6500}, {28, 1671, 2, 6500}, {28, 1685, 4, 6500}, {28, 1711, 4, 6500}, {28, 1725, 2, 6500}, {29, 1686, 2, 6500}, {29, 1726, 2, 6500}, {30, 1699, 1, 6500}, {31, 1700, 2, 6500}, {31, 1714, 2, 6500}, {32, 1701, 1, 6500}, {32, 1715, 4, 6500}, {32, 1729, 1, 6500}, {33, 1716, 2, 6500}, {33, 1730, 2, 6500}, {34, 1731, 1, 6500}, {20, 1648, 1, 6501}, {21, 1651, 2, 6501}, {21, 1663, 2, 6501}, {22, 1652, 2, 6501}, {22, 1678, 2, 6501}, {23, 1655, 1, 6501}, {23, 1666, 4, 6501}, {23, 1693, 1, 6501}, {24, 1656, 2, 6501}, {24, 1667, 4, 6501}, {24, 1681, 4, 6501}, {24, 1708, 2, 6501}, {25, 1657, 1, 6501}, {25, 1682, 4, 6501}, {25, 1723, 1, 6501}, {26, 1670, 2, 6501}, {26, 1696, 2, 6501}, {27, 1671, 4, 6501}, {27, 1685, 2, 6501}, {27, 1697, 2, 6501}, {27, 1711, 4, 6501}, {28, 1672, 2, 6501}, {28, 1686, 4, 6501}, {28, 1712, 4, 6501}, {28, 1726, 2, 6501}, {29, 1687, 2, 6501}, {29, 1727, 2, 6501}, {30, 1700, 1, 6501}, {31, 1701, 2, 6501}, {31, 1715, 2, 6501}, {32, 1702, 1, 6501}, {32, 1716, 4, 6501}, {32, 1730, 1, 6501}, {33, 1717, 2, 6501}, {33, 1731, 2, 6501}, {34, 1732, 1, 6501}, {20, 1658, 1, 6502}, {21, 1659, 2, 6502}, {21, 1688, 2, 6502}, {22, 1660, 2, 6502}, {22, 1703, 2, 6502}, {23, 1661, 1, 6502}, {23, 1689, 4, 6502}, {23, 1733, 1, 6502}, {24, 1662, 2, 6502}, {24, 1690, 4, 6502}, {24, 1704, 4, 6502}, {24, 1748, 2, 6502}, {25, 1663, 1, 6502}, {25, 1705, 4, 6502}, {25, 1763, 1, 6502}, {26, 1691, 2, 6502}, {26, 1734, 2, 6502}, {27, 1692, 4, 6502}, {27, 1706, 2, 6502}, {27, 1735, 2, 6502}, {27, 1749, 4, 6502}, {28, 1693, 2, 6502}, {28, 1707, 4, 6502}, {28, 1750, 4, 6502}, {28, 1764, 2, 6502}, {29, 1708, 2, 6502}, {29, 1765, 2, 6502}, {30, 1736, 1, 6502}, {31, 1737, 2, 6502}, {31, 1751, 2, 6502}, {32, 1738, 1, 6502}, {32, 1752, 4, 6502}, {32, 1766, 1, 6502}, {33, 1753, 2, 6502}, {33, 1767, 2, 6502}, {34, 1768, 1, 6502}, {20, 1659, 1, 6503}, {21, 1661, 2, 6503}, {21, 1689, 2, 6503}, {22, 1662, 2, 6503}, {22, 1704, 2, 6503}, {23, 1664, 1,

6503}, {23, 1691, 4, 6503}, {23, 1734, 1, 6503}, {24, 1665, 2, 6503}, {24, 1692, 4, 6503}, {24, 1706, 4, 6503}, {24, 1749, 2, 6503}, {25, 1666, 1, 6503}, {25, 1707, 4, 6503}, {25, 1764, 1, 6503}, {26, 1694, 2, 6503}, {26, 1736, 2, 6503}, {27, 1695, 4, 6503}, {27, 1709, 2, 6503}, {27, 1737, 2, 6503}, {27, 1751, 4, 6503}, {28, 1696, 2, 6503}, {28, 1710, 4, 6503}, {28, 1752, 4, 6503}, {28, 1766, 2, 6503}, {29, 1711, 2, 6503}, {29, 1767, 2, 6503}, {30, 1739, 1, 6503}, {31, 1740, 2, 6503}, {31, 1754, 2, 6503}, {32, 1741, 1, 6503}, {32, 1755, 4, 6503}, {32, 1769, 1, 6503}, {33, 1756, 2, 6503}, {33, 1770, 2, 6503}, {34, 1771, 1, 6503}, {20, 1660, 1, 6504}, {21, 1662, 2, 6504}, {21, 1690, 2, 6504}, {22, 1663, 2, 6504}, {22, 1705, 2, 6504}, {23, 1665, 1, 6504}, {23, 1692, 4, 6504}, {23, 1735, 1, 6504}, {24, 1666, 2, 6504}, {24, 1693, 4, 6504}, {24, 1707, 4, 6504}, {24, 1750, 2, 6504}, {25, 1667, 1, 6504}, {25, 1708, 4, 6504}, {25, 1765, 1, 6504}, {26, 1695, 2, 6504}, {26, 1737, 2, 6504}, {27, 1696, 4, 6504}, {27, 1710, 2, 6504}, {27, 1738, 2, 6504}, {27, 1752, 4, 6504}, {28, 1697, 2, 6504}, {28, 1711, 4, 6504}, {28, 1753, 4, 6504}, {28, 1767, 2, 6504}, {29, 1712, 2, 6504}, {29, 1768, 2, 6504}, {30, 1740, 1, 6504}, {31, 1741, 2, 6504}, {31, 1755, 2, 6504}, {32, 1742, 1, 6504}, {32, 1756, 4, 6504}, {32, 1770, 1, 6504}, {33, 1757, 2, 6504}, {33, 1771, 2, 6504}, {34, 1772, 1, 6504}, {20, 1661, 1, 6505}, {21, 1664, 2, 6505}, {21, 1691, 2, 6505}, {22, 1665, 2, 6505}, {22, 1706, 2, 6505}, {23, 1668, 1, 6505}, {23, 1694, 4, 6505}, {23, 1736, 1, 6505}, {24, 1669, 2, 6505}, {24, 1695, 4, 6505}, {24, 1709, 4, 6505}, {24, 1751, 2, 6505}, {25, 1670, 1, 6505}, {25, 1710, 4, 6505}, {25, 1766, 1, 6505}, {26, 1698, 2, 6505}, {26, 1739, 2, 6505}, {27, 1699, 4, 6505}, {27, 1713, 2, 6505}, {27, 1740, 2, 6505}, {27, 1754, 4, 6505}, {28, 1700, 2, 6505}, {28, 1714, 4, 6505}, {28, 1755, 4, 6505}, {28, 1769, 2, 6505}, {29, 1715, 2, 6505}, {29, 1770, 2, 6505}, {30, 1743, 1, 6505}, {31, 1744, 2, 6505}, {31, 1758, 2, 6505}, {32, 1745, 1, 6505}, {32, 1759, 4, 6505}, {32, 1773, 1, 6505}, {33, 1760, 2, 6505}, {33, 1774, 2, 6505}, {34, 1775, 1, 6505}, {20, 1662, 1, 6506}, {21, 1665, 2, 6506}, {21, 1692, 2, 6506}, {22, 1666, 2, 6506}, {22, 1707, 2, 6506}, {23, 1669, 1, 6506}, {23, 1695, 4, 6506}, {23, 1737, 1, 6506}, {24, 1670, 2, 6506}, {24, 1696, 4, 6506}, {24, 1710, 4, 6506}, {24, 1752, 2, 6506}, {25, 1671, 1, 6506}, {25, 1711, 4, 6506}, {25, 1767, 1, 6506}, {26, 1699, 2, 6506}, {26, 1740, 2, 6506}, {27, 1700, 4, 6506}, {27, 1714, 2, 6506}, {27, 1741, 2, 6506}, {27, 1755, 4, 6506}, {28, 1701, 2, 6506}, {28, 1715, 4, 6506}, {28, 1756, 4, 6506}, {28, 1770, 2, 6506}, {29, 1716, 2, 6506}, {29, 1771, 2, 6506}, {30, 1744, 1, 6506}, {31, 1745, 2, 6506}, {31, 1759, 2, 6506}, {32, 1746, 1, 6506}, {32, 1760, 4, 6506}, {32, 1774, 1, 6506}, {33, 1761, 2, 6506}, {33, 1775, 2, 6506}, {34, 1776, 1, 6506}, {20, 1663, 1, 6507}, {21, 1666, 2, 6507}, {21, 1693, 2, 6507}, {22, 1667, 2, 6507}, {22, 1708, 2, 6507}, {23, 1670, 1, 6507}, {23, 1696, 4, 6507}, {23, 1738, 1, 6507}, {24, 1671, 2, 6507}, {24, 1697, 4, 6507}, {24, 1711, 4, 6507}, {24, 1753, 2, 6507}, {25, 1672, 1, 6507}, {25, 1712, 4, 6507}, {25, 1768, 1, 6507}, {26, 1700, 2, 6507}, {26, 1741, 2, 6507}, {27, 1701, 4, 6507}, {27, 1715, 2, 6507}, {27, 1742, 2, 6507}, {27, 1756, 4, 6507}, {28, 1702, 2, 6507}, {28, 1716, 4, 6507}, {28, 1757, 4, 6507}, {28, 1771, 2, 6507}, {29, 1717, 2, 6507}, {29, 1772, 2, 6507}, {30, 1745, 1, 6507}, {31, 1746, 2, 6507}, {31, 1760, 2, 6507}, {32, 1747, 1, 6507}, {32, 1761, 4, 6507}, {32, 1775, 1, 6507}, {33, 1762, 2, 6507}, {33, 1776, 2, 6507}, {34, 1777, 1, 6507}, {20, 1673, 1, 6508}, {21, 1674, 2, 6508}, {21, 1703, 2, 6508}, {22, 1675, 2, 6508}, {22, 1718, 2, 6508}, {23, 1676, 1, 6508}, {23, 1704, 4, 6508}, {23, 1748, 1, 6508}, {24, 1677, 2, 6508}, {24, 1705, 4, 6508}, {24, 1719, 4, 6508}, {24, 1763, 2, 6508}, {25, 1678, 1, 6508}, {25, 1720, 4, 6508}, {25, 1778, 1, 6508}, {26, 1706, 2, 6508}, {26, 1749, 2, 6508}, {27, 1707, 4, 6508}, {27, 1721, 2, 6508}, {27, 1750, 2, 6508}, {27, 1764, 4, 6508}, {28, 1708, 2, 6508}, {28, 1722, 4, 6508}, {28, 1765, 4, 6508}, {28, 1779, 2, 6508}, {29, 1723, 2, 6508}, {29, 1780, 2, 6508}, {30, 1751, 1, 6508}, {31, 1752, 2, 6508}, {31, 1766, 2, 6508}, {32, 1753, 1, 6508}, {32, 1767, 4, 6508}, {32, 1781, 1, 6508}, {33, 1768, 2, 6508}, {33, 1782, 2, 6508}, {34, 1783, 1, 6508}, {20, 1674, 1, 6509}, {21, 1676, 2, 6509}, {21, 1704, 2, 6509}, {22, 1677, 2, 6509}, {22, 1719, 2, 6509}, {23, 1679, 1, 6509}, {23, 1706, 4, 6509}, {23, 1749, 1, 6509}, {24, 1680, 2, 6509}, {24, 1707, 4, 6509}, {24, 1721, 4, 6509}, {24, 1764, 2, 6509}, {25, 1681, 1, 6509}, {25, 1722, 4, 6509}, {25, 1779, 1, 6509}, {26, 1709, 2, 6509}, {26, 1751, 2, 6509}, {27, 1710, 4, 6509}, {27, 1724, 2, 6509}, {27, 1752, 2, 6509}, {27, 1766, 4, 6509}, {28, 1711, 2, 6509}, {28, 1725, 4, 6509}, {28, 1767, 4, 6509}, {28, 1781, 2, 6509}, {29, 1726, 2, 6509}, {29, 1782, 2, 6509}, {30, 1754, 1, 6509}, {31, 1755, 2, 6509}, {31, 1769, 2, 6509}, {32, 1756, 1, 6509}, {32, 1770, 4, 6509}, {32, 1784, 1, 6509}, {33, 1771, 2, 6509}, {33, 1785, 2, 6509}, {34, 1786, 1, 6509}, {20, 1675, 1, 6510}, {21, 1677, 2, 6510}, {21, 1705, 2, 6510}, {22, 1678, 2, 6510}, {22, 1720, 2, 6510}, {23, 1680, 1, 6510}, {23, 1707, 4, 6510}, {23, 1750, 1, 6510}, {24, 1681, 2, 6510}, {24, 1708, 4, 6510}, {24, 1722, 4, 6510}, {24, 1765, 2, 6510}, {25, 1682, 1, 6510}, {25, 1723, 4, 6510}, {25, 1780, 1, 6510}, {26, 1710, 2, 6510}, {26, 1752, 2, 6510}, {27, 1711, 4, 6510}, {27, 1725, 2, 6510}, {27, 1753, 2, 6510}, {27, 1767, 4, 6510}, {28, 1712, 2, 6510}, {28, 1726, 4, 6510}, {28, 1768, 4, 6510}, {28, 1782, 2, 6510}, {29, 1727, 2, 6510}, {29, 1783, 2, 6510}, {30, 1755, 1, 6510}, {31, 1756, 2, 6510}, {31, 1770, 2, 6510}, {32, 1757, 1, 6510}, {32, 1771, 4, 6510}, {32, 1785, 1, 6510}, {33, 1772, 2, 6510}, {33, 1786, 2, 6510}, {34, 1787, 1, 6510}, {20, 1676, 1, 6511}, {21, 1679, 2, 6511}, {21, 1706, 2, 6511}, {22, 1680, 2, 6511}, {22, 1721, 2, 6511}, {23, 1683, 1, 6511}, {23, 1709, 4, 6511}, {23, 1751, 1, 6511}, {24, 1684, 2, 6511}, {24, 1710, 4, 6511}, {24, 1724, 4, 6511},

{24, 1766, 2, 6511}, {25, 1685, 1, 6511}, {25, 1725, 4, 6511}, {25, 1781, 1, 6511}, {26, 1713, 2, 6511}, {26, 1754, 2, 6511}, {27, 1714, 4, 6511}, {27, 1728, 2, 6511}, {27, 1755, 2, 6511}, {27, 1769, 4, 6511}, {28, 1715, 2, 6511}, {28, 1729, 4, 6511}, {28, 1770, 4, 6511}, {28, 1784, 2, 6511}, {29, 1730, 2, 6511}, {29, 1785, 2, 6511}, {30, 1758, 1, 6511}, {31, 1759, 2, 6511}, {31, 1773, 2, 6511}, {32, 1760, 1, 6511}, {32, 1774, 4, 6511}, {32, 1788, 1, 6511}, {33, 1775, 2, 6511}, {33, 1789, 2, 6511}, {34, 1790, 1, 6511}, {20, 1677, 1, 6512}, {21, 1680, 2, 6512}, {21, 1707, 2, 6512}, {22, 1681, 2, 6512}, {22, 1722, 2, 6512}, {23, 1684, 1, 6512}, {23, 1710, 4, 6512}, {23, 1752, 1, 6512}, {24, 1685, 2, 6512}, {24, 1711, 4, 6512}, {24, 1725, 4, 6512}, {24, 1767, 2, 6512}, {25, 1686, 1, 6512}, {25, 1726, 4, 6512}, {25, 1782, 1, 6512}, {26, 1714, 2, 6512}, {26, 1755, 2, 6512}, {27, 1715, 4, 6512}, {27, 1729, 2, 6512}, {27, 1756, 2, 6512}, {27, 1770, 4, 6512}, {28, 1716, 2, 6512}, {28, 1730, 4, 6512}, {28, 1771, 4, 6512}, {28, 1785, 2, 6512}, {29, 1731, 2, 6512}, {29, 1786, 2, 6512}, {30, 1759, 1, 6512}, {31, 1760, 2, 6512}, {31, 1774, 2, 6512}, {32, 1761, 1, 6512}, {32, 1775, 4, 6512}, {32, 1789, 1, 6512}, {33, 1776, 2, 6512}, {33, 1790, 2, 6512}, {34, 1791, 1, 6512}, {20, 1678, 1, 6513}, {21, 1681, 2, 6513}, {21, 1708, 2, 6513}, {22, 1682, 2, 6513}, {22, 1723, 2, 6513}, {23, 1685, 1, 6513}, {23, 1711, 4, 6513}, {23, 1753, 1, 6513}, {24, 1686, 2, 6513}, {24, 1712, 4, 6513}, {24, 1726, 4, 6513}, {24, 1768, 2, 6513}, {25, 1687, 1, 6513}, {25, 1727, 4, 6513}, {25, 1783, 1, 6513}, {26, 1715, 2, 6513}, {26, 1756, 2, 6513}, {27, 1716, 4, 6513}, {27, 1730, 2, 6513}, {27, 1757, 2, 6513}, {27, 1771, 4, 6513}, {28, 1717, 2, 6513}, {28, 1731, 4, 6513}, {28, 1772, 4, 6513}, {28, 1786, 2, 6513}, {29, 1732, 2, 6513}, {29, 1787, 2, 6513}, {30, 1760, 1, 6513}, {31, 1761, 2, 6513}, {31, 1775, 2, 6513}, {32, 1762, 1, 6513}, {32, 1776, 4, 6513}, {32, 1790, 1, 6513}, {33, 1777, 2, 6513}, {33, 1791, 2, 6513}, {34, 1792, 1, 6513}, {20, 1793, 1, 6514}, {21, 1794, 4, 6514}, {22, 1795, 4, 6514}, {23, 1796, 2, 6514}, {23, 1799, 4, 6514}, {24, 1797, 4, 6514}, {24, 1800, 8, 6514}, {25, 1798, 2, 6514}, {25, 1804, 4, 6514}, {26, 1801, 4, 6514}, {27, 1802, 8, 6514}, {27, 1805, 4, 6514}, {28, 1803, 4, 6514}, {28, 1806, 8, 6514}, {29, 1807, 4, 6514}, {30, 1808, 1, 6514}, {31, 1809, 4, 6514}, {32, 1810, 2, 6514}, {32, 1811, 4, 6514}, {33, 1812, 4, 6514}, {34, 1813, 1, 6514}, {20, 1814, 1, 6515}, {21, 1815, 2, 6515}, {21, 1824, 2, 6515}, {22, 1816, 2, 6515}, {22, 1834, 2, 6515}, {23, 1817, 1, 6515}, {23, 1825, 4, 6515}, {23, 1844, 1, 6515}, {24, 1818, 2, 6515}, {24, 1826, 4, 6515}, {24, 1835, 4, 6515}, {24, 1854, 2, 6515}, {25, 1819, 1, 6515}, {25, 1836, 4, 6515}, {25, 1864, 1, 6515}, {26, 1827, 2, 6515}, {26, 1845, 2, 6515}, {27, 1828, 4, 6515}, {27, 1837, 2, 6515}, {27, 1846, 2, 6515}, {27, 1855, 4, 6515}, {28, 1829, 2, 6515}, {28, 1838, 4, 6515}, {28, 1856, 4, 6515}, {28, 1865, 2, 6515}, {29, 1839, 2, 6515}, {29, 1866, 2, 6515}, {30, 1847, 1, 6515}, {31, 1848, 2, 6515}, {31, 1857, 2, 6515}, {32, 1849, 1, 6515}, {32, 1858, 4, 6515}, {32, 1867, 1, 6515}, {33, 1859, 2, 6515}, {33, 1868, 2, 6515}, {34, 1869, 1, 6515}, {20, 1815, 1, 6516}, {21, 1817, 2, 6516}, {21, 1825, 2, 6516}, {22, 1818, 2, 6516}, {22, 1835, 2, 6516}, {23, 1820, 1, 6516}, {23, 1827, 4, 6516}, {23, 1845, 1, 6516}, {24, 1821, 2, 6516}, {24, 1828, 4, 6516}, {24, 1837, 4, 6516}, {24, 1855, 2, 6516}, {25, 1822, 1, 6516}, {25, 1838, 4, 6516}, {25, 1865, 1, 6516}, {26, 1830, 2, 6516}, {26, 1847, 2, 6516}, {27, 1831, 4, 6516}, {27, 1840, 2, 6516}, {27, 1848, 2, 6516}, {27, 1857, 4, 6516}, {28, 1832, 2, 6516}, {28, 1841, 4, 6516}, {28, 1858, 4, 6516}, {28, 1867, 2, 6516}, {29, 1842, 2, 6516}, {29, 1868, 2, 6516}, {30, 1850, 1, 6516}, {31, 1851, 2, 6516}, {31, 1860, 2, 6516}, {32, 1852, 1, 6516}, {32, 1861, 4, 6516}, {32, 1870, 1, 6516}, {33, 1862, 2, 6516}, {33, 1871, 2, 6516}, {34, 1872, 1, 6516}, {20, 1816, 1, 6517}, {21, 1818, 2, 6517}, {21, 1826, 2, 6517}, {22, 1819, 2, 6517}, {22, 1836, 2, 6517}, {23, 1821, 1, 6517}, {23, 1828, 4, 6517}, {23, 1846, 1, 6517}, {24, 1822, 2, 6517}, {24, 1829, 4, 6517}, {24, 1838, 4, 6517}, {24, 1856, 2, 6517}, {25, 1823, 1, 6517}, {25, 1839, 4, 6517}, {25, 1866, 1, 6517}, {26, 1831, 2, 6517}, {26, 1848, 2, 6517}, {27, 1832, 4, 6517}, {27, 1841, 2, 6517}, {27, 1849, 2, 6517}, {27, 1858, 4, 6517}, {28, 1833, 2, 6517}, {28, 1842, 4, 6517}, {28, 1859, 4, 6517}, {28, 1868, 2, 6517}, {29, 1843, 2, 6517}, {29, 1869, 2, 6517}, {30, 1851, 1, 6517}, {31, 1852, 2, 6517}, {31, 1861, 2, 6517}, {32, 1853, 1, 6517}, {32, 1862, 4, 6517}, {32, 1871, 1, 6517}, {33, 1863, 2, 6517}, {33, 1872, 2, 6517}, {34, 1873, 1, 6517}, {20, 1874, 1, 6518}, {21, 1875, 2, 6518}, {21, 1889, 2, 6518}, {22, 1876, 2, 6518}, {22, 1904, 2, 6518}, {23, 1877, 1, 6518}, {23, 1890, 4, 6518}, {23, 1919, 1, 6518}, {24, 1878, 2, 6518}, {24, 1891, 4, 6518}, {24, 1905, 4, 6518}, {24, 1934, 2, 6518}, {25, 1879, 1, 6518}, {25, 1906, 4, 6518}, {25, 1949, 1, 6518}, {26, 1892, 2, 6518}, {26, 1920, 2, 6518}, {27, 1893, 4, 6518}, {27, 1907, 2, 6518}, {27, 1921, 2, 6518}, {27, 1935, 4, 6518}, {28, 1894, 2, 6518}, {28, 1908, 4, 6518}, {28, 1936, 4, 6518}, {28, 1950, 2, 6518}, {29, 1909, 2, 6518}, {29, 1951, 2, 6518}, {30, 1922, 1, 6518}, {31, 1923, 2, 6518}, {31, 1937, 2, 6518}, {32, 1924, 1, 6518}, {32, 1938, 4, 6518}, {32, 1952, 1, 6518}, {33, 1939, 2, 6518}, {33, 1953, 2, 6518}, {34, 1954, 1, 6518}, {20, 1875, 1, 6519}, {21, 1877, 2, 6519}, {21, 1890, 2, 6519}, {22, 1878, 2, 6519}, {22, 1905, 2, 6519}, {23, 1880, 1, 6519}, {23, 1892, 4, 6519}, {23, 1920, 1, 6519}, {24, 1881, 2, 6519}, {24, 1893, 4, 6519}, {24, 1907, 4, 6519}, {24, 1935, 2, 6519}, {25, 1882, 1, 6519}, {25, 1908, 4, 6519}, {25, 1950, 1, 6519}, {26, 1895, 2, 6519}, {26, 1922, 2, 6519}, {27, 1896, 4, 6519}, {27, 1910, 2, 6519}, {27, 1923, 2, 6519}, {27, 1937, 4, 6519}, {28, 1897, 2, 6519}, {28, 1911, 4, 6519}, {28, 1938, 4, 6519}, {28, 1952, 2, 6519}, {29, 1912, 2, 6519}, {29, 1953, 2, 6519}, {30, 1925, 1, 6519}, {31, 1926, 2, 6519}, {31, 1940, 2, 6519}, {32, 1927, 1, 6519}, {32,

1941, 4, 6519}, {32, 1955, 1, 6519}, {33, 1942, 2, 6519}, {33, 1956, 2, 6519}, {34, 1957, 1, 6519}, {20, 1876, 1, 6520}, {21, 1878, 2, 6520}, {21, 1891, 2, 6520}, {22, 1879, 2, 6520}, {22, 1906, 2, 6520}, {23, 1881, 1, 6520}, {23, 1893, 4, 6520}, {23, 1921, 1, 6520}, {24, 1882, 2, 6520}, {24, 1894, 4, 6520}, {24, 1908, 4, 6520}, {24, 1936, 2, 6520}, {25, 1883, 1, 6520}, {25, 1909, 4, 6520}, {25, 1951, 1, 6520}, {26, 1896, 2, 6520}, {26, 1923, 2, 6520}, {27, 1897, 4, 6520}, {27, 1911, 2, 6520}, {27, 1924, 2, 6520}, {27, 1938, 4, 6520}, {28, 1898, 2, 6520}, {28, 1912, 4, 6520}, {28, 1939, 4, 6520}, {28, 1953, 2, 6520}, {29, 1913, 2, 6520}, {29, 1954, 2, 6520}, {30, 1926, 1, 6520}, {31, 1927, 2, 6520}, {31, 1941, 2, 6520}, {32, 1928, 1, 6520}, {32, 1942, 4, 6520}, {32, 1956, 1, 6520}, {33, 1943, 2, 6520}, {33, 1957, 2, 6520}, {34, 1958, 1, 6520}, {20, 1877, 1, 6521}, {21, 1880, 2, 6521}, {21, 1892, 2, 6521}, {22, 1881, 2, 6521}, {22, 1907, 2, 6521}, {23, 1884, 1, 6521}, {23, 1895, 4, 6521}, {23, 1922, 1, 6521}, {24, 1885, 2, 6521}, {24, 1896, 4, 6521}, {24, 1910, 4, 6521}, {24, 1937, 2, 6521}, {25, 1886, 1, 6521}, {25, 1911, 4, 6521}, {25, 1952, 1, 6521}, {26, 1899, 2, 6521}, {26, 1925, 2, 6521}, {27, 1900, 4, 6521}, {27, 1914, 2, 6521}, {27, 1926, 2, 6521}, {27, 1940, 4, 6521}, {28, 1901, 2, 6521}, {28, 1915, 4, 6521}, {28, 1941, 4, 6521}, {28, 1955, 2, 6521}, {29, 1916, 2, 6521}, {29, 1956, 2, 6521}, {30, 1929, 1, 6521}, {31, 1930, 2, 6521}, {31, 1944, 2, 6521}, {32, 1931, 1, 6521}, {32, 1945, 4, 6521}, {32, 1959, 1, 6521}, {33, 1946, 2, 6521}, {33, 1960, 2, 6521}, {34, 1961, 1, 6521}, {20, 1878, 1, 6522}, {21, 1881, 2, 6522}, {21, 1893, 2, 6522}, {22, 1882, 2, 6522}, {22, 1908, 2, 6522}, {23, 1885, 1, 6522}, {23, 1896, 4, 6522}, {23, 1923, 1, 6522}, {24, 1886, 2, 6522}, {24, 1897, 4, 6522}, {24, 1911, 4, 6522}, {24, 1938, 2, 6522}, {25, 1887, 1, 6522}, {25, 1912, 4, 6522}, {25, 1953, 1, 6522}, {26, 1900, 2, 6522}, {26, 1926, 2, 6522}, {27, 1901, 4, 6522}, {27, 1915, 2, 6522}, {27, 1927, 2, 6522}, {27, 1941, 4, 6522}, {28, 1902, 2, 6522}, {28, 1916, 4, 6522}, {28, 1942, 4, 6522}, {28, 1956, 2, 6522}, {29, 1917, 2, 6522}, {29, 1957, 2, 6522}, {30, 1930, 1, 6522}, {31, 1931, 2, 6522}, {31, 1945, 2, 6522}, {32, 1932, 1, 6522}, {32, 1946, 4, 6522}, {32, 1960, 1, 6522}, {33, 1947, 2, 6522}, {33, 1961, 2, 6522}, {34, 1962, 1, 6522}, {20, 1879, 1, 6523}, {21, 1882, 2, 6523}, {21, 1894, 2, 6523}, {22, 1883, 2, 6523}, {22, 1909, 2, 6523}, {23, 1886, 1, 6523}, {23, 1897, 4, 6523}, {23, 1924, 1, 6523}, {24, 1887, 2, 6523}, {24, 1898, 4, 6523}, {24, 1912, 4, 6523}, {24, 1939, 2, 6523}, {25, 1888, 1, 6523}, {25, 1913, 4, 6523}, {25, 1954, 1, 6523}, {26, 1901, 2, 6523}, {26, 1927, 2, 6523}, {27, 1902, 4, 6523}, {27, 1916, 2, 6523}, {27, 1928, 2, 6523}, {27, 1942, 4, 6523}, {28, 1903, 2, 6523}, {28, 1917, 4, 6523}, {28, 1943, 4, 6523}, {28, 1957, 2, 6523}, {29, 1918, 2, 6523}, {29, 1958, 2, 6523}, {30, 1931, 1, 6523}, {31, 1932, 2, 6523}, {31, 1946, 2, 6523}, {32, 1933, 1, 6523}, {32, 1947, 4, 6523}, {32, 1961, 1, 6523}, {33, 1948, 2, 6523}, {33, 1962, 2, 6523}, {34, 1963, 1, 6523}, {20, 2119, 1, 6524}, {21, 2120, 4, 6524}, {22, 2121, 4, 6524}, {23, 2122, 2, 6524}, {23, 2129, 4, 6524}, {24, 2123, 4, 6524}, {24, 2130, 8, 6524}, {25, 2124, 2, 6524}, {25, 2138, 4, 6524}, {26, 2131, 4, 6524}, {27, 2132, 8, 6524}, {27, 2139, 4, 6524}, {28, 2133, 4, 6524}, {28, 2140, 8, 6524}, {29, 2141, 4, 6524}, {30, 2146, 1, 6524}, {31, 2147, 4, 6524}, {32, 2148, 2, 6524}, {32, 2153, 4, 6524}, {33, 2154, 4, 6524}, {34, 2159, 1, 6524}, {20, 2120, 1, 6525}, {21, 2122, 2, 6525}, {21, 2129, 2, 6525}, {22, 2123, 2, 6525}, {22, 2130, 2, 6525}, {23, 2125, 1, 6525}, {23, 2131, 5, 6525}, {24, 2126, 2, 6525}, {24, 2132, 6, 6525}, {24, 2139, 4, 6525}, {25, 2127, 1, 6525}, {25, 2133, 1, 6525}, {25, 2140, 4, 6525}, {26, 2134, 2, 6525}, {26, 2146, 2, 6525}, {27, 2135, 4, 6525}, {27, 2142, 2, 6525}, {27, 2147, 6, 6525}, {28, 2136, 2, 6525}, {28, 2143, 4, 6525}, {28, 2148, 2, 6525}, {28, 2153, 4, 6525}, {29, 2144, 2, 6525}, {29, 2154, 2, 6525}, {30, 2149, 1, 6525}, {31, 2150, 2, 6525}, {31, 2155, 2, 6525}, {32, 2151, 1, 6525}, {32, 2156, 4, 6525}, {32, 2160, 1, 6525}, {33, 2157, 2, 6525}, {33, 2161, 2, 6525}, {34, 2162, 1, 6525}, {20, 2121, 1, 6526}, {21, 2123, 2, 6526}, {21, 2130, 2, 6526}, {22, 2124, 2, 6526}, {22, 2138, 2, 6526}, {23, 2126, 1, 6526}, {23, 2132, 4, 6526}, {23, 2139, 1, 6526}, {24, 2127, 2, 6526}, {24, 2133, 4, 6526}, {24, 2140, 6, 6526}, {25, 2128, 1, 6526}, {25, 2141, 5, 6526}, {26, 2135, 2, 6526}, {26, 2147, 2, 6526}, {27, 2136, 4, 6526}, {27, 2143, 2, 6526}, {27, 2148, 2, 6526}, {27, 2153, 4, 6526}, {28, 2137, 2, 6526}, {28, 2144, 4, 6526}, {28, 2154, 6, 6526}, {29, 2145, 2, 6526}, {29, 2159, 2, 6526}, {30, 2150, 1, 6526}, {31, 2151, 2, 6526}, {31, 2156, 2, 6526}, {32, 2152, 1, 6526}, {32, 2157, 4, 6526}, {32, 2161, 1, 6526}, {33, 2158, 2, 6526}, {33, 2162, 2, 6526}, {34, 2163, 1, 6526}, {20, 2129, 1, 6527}, {21, 2131, 4, 6527}, {22, 2132, 4, 6527}, {23, 2134, 2, 6527}, {23, 2146, 4, 6527}, {24, 2135, 4, 6527}, {24, 2147, 8, 6527}, {25, 2136, 2, 6527}, {25, 2153, 4, 6527}, {26, 2149, 4, 6527}, {27, 2150, 8, 6527}, {27, 2155, 4, 6527}, {28, 2151, 4, 6527}, {28, 2156, 8, 6527}, {29, 2157, 4, 6527}, {30, 2164, 1, 6527}, {31, 2165, 4, 6527}, {32, 2166, 2, 6527}, {32, 2168, 4, 6527}, {33, 2169, 4, 6527}, {34, 2171, 1, 6527}, {20, 2130, 1, 6528}, {21, 2132, 2, 6528}, {21, 2139, 2, 6528}, {22, 2133, 2, 6528}, {22, 2140, 2, 6528}, {23, 2135, 1, 6528}, {23, 2142, 1, 6528}, {23, 2147, 4, 6528}, {24, 2136, 2, 6528}, {24, 2143, 2, 6528}, {24, 2148, 4, 6528}, {24, 2153, 4, 6528}, {25, 2137, 1, 6528}, {25, 2144, 1, 6528}, {25, 2154, 4, 6528}, {26, 2150, 2, 6528}, {26, 2155, 2, 6528}, {27, 2151, 4, 6528}, {27, 2156, 6, 6528}, {27, 2160, 2, 6528}, {28, 2152, 2, 6528}, {28, 2157, 6, 6528}, {28, 2161, 4, 6528}, {29, 2158, 2, 6528}, {29, 2162, 2, 6528}, {30, 2165, 1, 6528}, {31, 2166, 2, 6528}, {31, 2168, 2, 6528}, {32, 2167, 1, 6528}, {32, 2169, 5, 6528}, {33, 2170, 2, 6528}, {33, 2171, 2, 6528}, {34, 2172, 1, 6528}, {20, 2138, 1, 6529}, {21, 2140, 4, 6529}, {22, 2141, 4, 6529}, {23, 2143,

2, 6529}, {23, 2153, 4, 6529}, {24, 2144, 4, 6529}, {24, 2154, 8, 6529}, {25, 2145, 2, 6529}, {25, 2159, 4, 6529}, {26, 2156, 4, 6529}, {27, 2157, 8, 6529}, {27, 2161, 4, 6529}, {28, 2158, 4, 6529}, {28, 2162, 8, 6529}, {29, 2163, 4, 6529}, {30, 2168, 1, 6529}, {31, 2169, 4, 6529}, {32, 2170, 2, 6529}, {32, 2171, 4, 6529}, {33, 2172, 4, 6529}, {34, 2173, 1, 6529}, {20, 2174, 1, 6530}, {21, 2175, 4, 6530}, {22, 2176, 4, 6530}, {23, 2177, 2, 6530}, {23, 2180, 4, 6530}, {24, 2178, 4, 6530}, {24, 2181, 8, 6530}, {25, 2179, 2, 6530}, {25, 2185, 4, 6530}, {26, 2182, 4, 6530}, {27, 2183, 8, 6530}, {27, 2186, 4, 6530}, {28, 2184, 4, 6530}, {28, 2187, 8, 6530}, {29, 2188, 4, 6530}, {30, 2189, 1, 6530}, {31, 2190, 4, 6530}, {32, 2191, 2, 6530}, {32, 2192, 4, 6530}, {33, 2193, 4, 6530}, {34, 2194, 1, 6530}, {20, 2195, 1, 6531}, {21, 2196, 2, 6531}, {21, 2205, 2, 6531}, {22, 2197, 2, 6531}, {22, 2215, 2, 6531}, {23, 2198, 1, 6531}, {23, 2206, 4, 6531}, {23, 2225, 1, 6531}, {24, 2199, 2, 6531}, {24, 2207, 4, 6531}, {24, 2216, 4, 6531}, {24, 2235, 2, 6531}, {25, 2200, 1, 6531}, {25, 2217, 4, 6531}, {25, 2245, 1, 6531}, {26, 2208, 2, 6531}, {26, 2226, 2, 6531}, {27, 2209, 4, 6531}, {27, 2218, 2, 6531}, {27, 2227, 2, 6531}, {27, 2236, 4, 6531}, {28, 2210, 2, 6531}, {28, 2219, 4, 6531}, {28, 2237, 4, 6531}, {28, 2246, 2, 6531}, {29, 2220, 2, 6531}, {29, 2247, 2, 6531}, {30, 2228, 1, 6531}, {31, 2229, 2, 6531}, {31, 2238, 2, 6531}, {32, 2230, 1, 6531}, {32, 2239, 4, 6531}, {32, 2248, 1, 6531}, {33, 2240, 2, 6531}, {33, 2249, 2, 6531}, {34, 2250, 1, 6531}, {20, 2196, 1, 6532}, {21, 2198, 2, 6532}, {21, 2206, 2, 6532}, {22, 2199, 2, 6532}, {22, 2216, 2, 6532}, {23, 2201, 1, 6532}, {23, 2208, 4, 6532}, {23, 2226, 1, 6532}, {24, 2202, 2, 6532}, {24, 2209, 4, 6532}, {24, 2218, 4, 6532}, {24, 2236, 2, 6532}, {25, 2203, 1, 6532}, {25, 2219, 4, 6532}, {25, 2246, 1, 6532}, {26, 2211, 2, 6532}, {26, 2228, 2, 6532}, {27, 2212, 4, 6532}, {27, 2221, 2, 6532}, {27, 2229, 2, 6532}, {27, 2238, 4, 6532}, {28, 2213, 2, 6532}, {28, 2222, 4, 6532}, {28, 2239, 4, 6532}, {28, 2248, 2, 6532}, {29, 2223, 2, 6532}, {29, 2249, 2, 6532}, {30, 2231, 1, 6532}, {31, 2232, 2, 6532}, {31, 2241, 2, 6532}, {32, 2233, 1, 6532}, {32, 2242, 4, 6532}, {32, 2251, 1, 6532}, {33, 2243, 2, 6532}, {33, 2252, 2, 6532}, {34, 2253, 1, 6532}, {20, 2197, 1, 6533}, {21, 2199, 2, 6533}, {21, 2207, 2, 6533}, {22, 2200, 2, 6533}, {22, 2217, 2, 6533}, {23, 2202, 1, 6533}, {23, 2209, 4, 6533}, {23, 2227, 1, 6533}, {24, 2203, 2, 6533}, {24, 2210, 4, 6533}, {24, 2219, 4, 6533}, {24, 2237, 2, 6533}, {25, 2204, 1, 6533}, {25, 2220, 4, 6533}, {25, 2247, 1, 6533}, {26, 2212, 2, 6533}, {26, 2229, 2, 6533}, {27, 2213, 4, 6533}, {27, 2222, 2, 6533}, {27, 2230, 2, 6533}, {27, 2239, 4, 6533}, {28, 2214, 2, 6533}, {28, 2223, 4, 6533}, {28, 2240, 4, 6533}, {28, 2249, 2, 6533}, {29, 2224, 2, 6533}, {29, 2250, 2, 6533}, {30, 2232, 1, 6533}, {31, 2233, 2, 6533}, {31, 2242, 2, 6533}, {32, 2234, 1, 6533}, {32, 2243, 4, 6533}, {32, 2252, 1, 6533}, {33, 2244, 2, 6533}, {33, 2253, 2, 6533}, {34, 2254, 1, 6533}, {20, 2534, 1, 6534}, {21, 2535, 4, 6534}, {22, 2536, 4, 6534}, {23, 2537, 2, 6534}, {23, 2540, 4, 6534}, {24, 2538, 4, 6534}, {24, 2541, 8, 6534}, {25, 2539, 2, 6534}, {25, 2545, 4, 6534}, {26, 2542, 4, 6534}, {27, 2543, 8, 6534}, {27, 2546, 4, 6534}, {28, 2544, 4, 6534}, {28, 2547, 8, 6534}, {29, 2548, 4, 6534}, {30, 2549, 1, 6534}, {31, 2550, 4, 6534}, {32, 2551, 2, 6534}, {32, 2552, 4, 6534}, {33, 2553, 4, 6534}, {34, 2554, 1, 6534}, {20, 2689, 1, 6535}, {21, 2690, 4, 6535}, {22, 2691, 4, 6535}, {23, 2692, 2, 6535}, {23, 2695, 4, 6535}, {24, 2693, 4, 6535}, {24, 2696, 8, 6535}, {25, 2694, 2, 6535}, {25, 2700, 4, 6535}, {26, 2697, 4, 6535}, {27, 2698, 8, 6535}, {27, 2701, 4, 6535}, {28, 2699, 4, 6535}, {28, 2702, 8, 6535}, {29, 2703, 4, 6535}, {30, 2704, 1, 6535}, {31, 2705, 4, 6535}, {32, 2706, 2, 6535}, {32, 2707, 4, 6535}, {33, 2708, 4, 6535}, {34, 2709, 1, 6535}, {35, 2098, 1, 6536}, {36, 2099, 5, 6536}, {37, 2100, 5, 6536}, {38, 2101, 10, 6536}, {39, 2102, 20, 6536}, {40, 2103, 10, 6536}, {41, 2104, 10, 6536}, {42, 2105, 30, 6536}, {43, 2106, 30, 6536}, {44, 2107, 10, 6536}, {45, 2108, 5, 6536}, {46, 2109, 20, 6536}, {47, 2110, 30, 6536}, {48, 2111, 20, 6536}, {49, 2112, 5, 6536}, {50, 2113, 1, 6536}, {51, 2114, 5, 6536}, {52, 2115, 10, 6536}, {53, 2116, 10, 6536}, {54, 2117, 5, 6536}, {55, 2118, 1, 6536}, {35, 1793, 1, 6537}, {36, 1794, 3, 6537}, {37, 1795, 3, 6537}, {38, 1796, 1, 6537}, {38, 1799, 2, 6537}, {39, 1797, 2, 6537}, {39, 1800, 4, 6537}, {40, 1798, 1, 6537}, {40, 1804, 2, 6537}, {41, 1801, 1, 6537}, {42, 1802, 2, 6537}, {42, 1805, 1, 6537}, {43, 1803, 1, 6537}, {43, 1806, 2, 6537}, {44, 1807, 1, 6537}, {35, 1794, 1, 6538}, {36, 1796, 1, 6538}, {36, 1799, 2, 6538}, {37, 1797, 1, 6538}, {37, 1800, 2, 6538}, {38, 1801, 3, 6538}, {39, 1802, 4, 6538}, {39, 1805, 2, 6538}, {40, 1803, 1, 6538}, {40, 1806, 2, 6538}, {41, 1808, 1, 6538}, {42, 1809, 3, 6538}, {43, 1810, 1, 6538}, {43, 1811, 2, 6538}, {44, 1812, 1, 6538}, {35, 1795, 1, 6539}, {36, 1797, 1, 6539}, {36, 1800, 2, 6539}, {37, 1798, 1, 6539}, {37, 1804, 2, 6539}, {38, 1802, 2, 6539}, {38, 1805, 1, 6539}, {39, 1803, 2, 6539}, {39, 1806, 4, 6539}, {40, 1807, 3, 6539}, {41, 1809, 1, 6539}, {42, 1810, 1, 6539}, {42, 1811, 2, 6539}, {43, 1812, 3, 6539}, {44, 1813, 1, 6539}, {36, 1793, 1, 6540}, {38, 1794, 3, 6540}, {39, 1795, 3, 6540}, {41, 1796, 1, 6540}, {41, 1799, 2, 6540}, {42, 1797, 2, 6540}, {42, 1800, 4, 6540}, {43, 1798, 1, 6540}, {43, 1804, 2, 6540}, {45, 1801, 1, 6540}, {46, 1802, 2, 6540}, {46, 1805, 1, 6540}, {47, 1803, 1, 6540}, {47, 1806, 2, 6540}, {48, 1807, 1, 6540}, {36, 1794, 1, 6541}, {38, 1796, 1, 6541}, {38, 1799, 2, 6541}, {39, 1797, 1, 6541}, {39, 1800, 2, 6541}, {41, 1801, 3, 6541}, {42, 1802, 4, 6541}, {42, 1805, 2, 6541}, {43, 1803, 1, 6541}, {43, 1806, 2, 6541}, {45, 1808, 1, 6541}, {46, 1809, 3, 6541}, {47, 1810, 1, 6541}, {47, 1811, 2, 6541}, {48, 1812, 1, 6541}, {36, 1795, 1, 6542}, {38, 1797, 1, 6542}, {38, 1800, 2, 6542}, {39, 1798, 1, 6542}, {39, 1804, 2, 6542}, {41, 1802, 2, 6542}, {41, 1805, 1, 6542}, {42, 1803, 2,

6542}, {42, 1806, 4, 6542}, {43, 1807, 3, 6542}, {45, 1809, 1, 6542}, {46, 1810, 1, 6542}, {46, 1811, 2, 6542}, {47, 1812, 3, 6542}, {48, 1813, 1, 6542}, {37, 1793, 1, 6543}, {39, 1794, 3, 6543}, {40, 1795, 3, 6543}, {42, 1796, 1, 6543}, {42, 1799, 2, 6543}, {43, 1797, 2, 6543}, {43, 1800, 4, 6543}, {44, 1798, 1, 6543}, {44, 1804, 2, 6543}, {46, 1801, 1, 6543}, {47, 1802, 2, 6543}, {47, 1805, 1, 6543}, {48, 1803, 1, 6543}, {48, 1806, 2, 6543}, {49, 1807, 1, 6543}, {37, 1794, 1, 6544}, {39, 1796, 1, 6544}, {39, 1799, 2, 6544}, {40, 1797, 1, 6544}, {40, 1800, 2, 6544}, {42, 1801, 3, 6544}, {43, 1802, 4, 6544}, {43, 1805, 2, 6544}, {44, 1803, 1, 6544}, {44, 1806, 2, 6544}, {46, 1808, 1, 6544}, {47, 1809, 3, 6544}, {48, 1810, 1, 6544}, {48, 1811, 2, 6544}, {49, 1812, 1, 6544}, {37, 1795, 1, 6545}, {39, 1797, 1, 6545}, {39, 1800, 2, 6545}, {40, 1798, 1, 6545}, {40, 1804, 2, 6545}, {42, 1802, 2, 6545}, {42, 1805, 1, 6545}, {43, 1803, 2, 6545}, {43, 1806, 4, 6545}, {44, 1807, 3, 6545}, {46, 1809, 1, 6545}, {47, 1810, 1, 6545}, {47, 1811, 2, 6545}, {48, 1812, 3, 6545}, {49, 1813, 1, 6545}, {38, 1793, 1, 6546}, {41, 1794, 3, 6546}, {42, 1795, 3, 6546}, {45, 1796, 1, 6546}, {45, 1799, 2, 6546}, {46, 1797, 2, 6546}, {46, 1800, 4, 6546}, {47, 1798, 1, 6546}, {47, 1804, 2, 6546}, {50, 1801, 1, 6546}, {51, 1802, 2, 6546}, {51, 1805, 1, 6546}, {52, 1803, 1, 6546}, {52, 1806, 2, 6546}, {53, 1807, 1, 6546}, {38, 1794, 1, 6547}, {41, 1796, 1, 6547}, {41, 1799, 2, 6547}, {42, 1797, 1, 6547}, {42, 1800, 2, 6547}, {45, 1801, 3, 6547}, {46, 1802, 4, 6547}, {46, 1805, 2, 6547}, {47, 1803, 1, 6547}, {47, 1806, 2, 6547}, {50, 1808, 1, 6547}, {51, 1809, 3, 6547}, {52, 1810, 1, 6547}, {52, 1811, 2, 6547}, {53, 1812, 1, 6547}, {38, 1795, 1, 6548}, {41, 1797, 1, 6548}, {41, 1800, 2, 6548}, {42, 1798, 1, 6548}, {42, 1804, 2, 6548}, {45, 1802, 2, 6548}, {45, 1805, 1, 6548}, {46, 1803, 2, 6548}, {46, 1806, 4, 6548}, {47, 1807, 3, 6548}, {50, 1809, 1, 6548}, {51, 1810, 1, 6548}, {51, 1811, 2, 6548}, {52, 1812, 3, 6548}, {53, 1813, 1, 6548}, {39, 1793, 1, 6549}, {42, 1794, 3, 6549}, {43, 1795, 3, 6549}, {46, 1796, 1, 6549}, {46, 1799, 2, 6549}, {47, 1797, 2, 6549}, {47, 1800, 4, 6549}, {48, 1798, 1, 6549}, {48, 1804, 2, 6549}, {51, 1801, 1, 6549}, {52, 1802, 2, 6549}, {52, 1805, 1, 6549}, {53, 1803, 1, 6549}, {53, 1806, 2, 6549}, {54, 1807, 1, 6549}, {39, 1794, 1, 6550}, {42, 1796, 1, 6550}, {42, 1799, 2, 6550}, {43, 1797, 1, 6550}, {43, 1800, 2, 6550}, {46, 1801, 3, 6550}, {47, 1802, 4, 6550}, {47, 1805, 2, 6550}, {48, 1803, 1, 6550}, {48, 1806, 2, 6550}, {51, 1808, 1, 6550}, {52, 1809, 3, 6550}, {53, 1810, 1, 6550}, {53, 1811, 2, 6550}, {54, 1812, 1, 6550}, {39, 1795, 1, 6551}, {42, 1797, 1, 6551}, {42, 1800, 2, 6551}, {43, 1798, 1, 6551}, {43, 1804, 2, 6551}, {46, 1802, 2, 6551}, {46, 1805, 1, 6551}, {47, 1803, 2, 6551}, {47, 1806, 4, 6551}, {48, 1807, 3, 6551}, {51, 1809, 1, 6551}, {52, 1810, 1, 6551}, {52, 1811, 2, 6551}, {53, 1812, 3, 6551}, {54, 1813, 1, 6551}, {40, 1793, 1, 6552}, {43, 1794, 3, 6552}, {44, 1795, 3, 6552}, {47, 1796, 1, 6552}, {47, 1799, 2, 6552}, {48, 1797, 2, 6552}, {48, 1800, 4, 6552}, {49, 1798, 1, 6552}, {49, 1804, 2, 6552}, {52, 1801, 1, 6552}, {53, 1802, 2, 6552}, {53, 1805, 1, 6552}, {54, 1803, 1, 6552}, {54, 1806, 2, 6552}, {55, 1807, 1, 6552}, {40, 1794, 1, 6553}, {43, 1796, 1, 6553}, {43, 1799, 2, 6553}, {44, 1797, 1, 6553}, {44, 1800, 2, 6553}, {47, 1801, 3, 6553}, {48, 1802, 4, 6553}, {48, 1805, 2, 6553}, {49, 1803, 1, 6553}, {49, 1806, 2, 6553}, {52, 1808, 1, 6553}, {53, 1809, 3, 6553}, {54, 1810, 1, 6553}, {54, 1811, 2, 6553}, {55, 1812, 1, 6553}, {40, 1795, 1, 6554}, {43, 1797, 1, 6554}, {43, 1800, 2, 6554}, {44, 1798, 1, 6554}, {44, 1804, 2, 6554}, {47, 1802, 2, 6554}, {47, 1805, 1, 6554}, {48, 1803, 2, 6554}, {48, 1806, 4, 6554}, {49, 1807, 3, 6554}, {52, 1809, 1, 6554}, {53, 1810, 1, 6554}, {53, 1811, 2, 6554}, {54, 1812, 3, 6554}, {55, 1813, 1, 6554}, {35, 1970, 1, 6555}, {36, 1971, 2, 6555}, {36, 1976, 1, 6555}, {37, 1972, 2, 6555}, {37, 1982, 1, 6555}, {38, 1973, 1, 6555}, {38, 1977, 2, 6555}, {39, 1974, 2, 6555}, {39, 1978, 2, 6555}, {39, 1983, 2, 6555}, {40, 1975, 1, 6555}, {40, 1984, 2, 6555}, {41, 1979, 1, 6555}, {42, 1980, 2, 6555}, {42, 1985, 1, 6555}, {43, 1981, 1, 6555}, {43, 1986, 2, 6555}, {44, 1987, 1, 6555}, {36, 1970, 1, 6556}, {38, 1971, 2, 6556}, {38, 1976, 1, 6556}, {39, 1972, 2, 6556}, {39, 1982, 1, 6556}, {41, 1973, 1, 6556}, {41, 1977, 2, 6556}, {42, 1974, 2, 6556}, {42, 1978, 2, 6556}, {42, 1983, 2, 6556}, {43, 1975, 1, 6556}, {43, 1984, 2, 6556}, {45, 1979, 1, 6556}, {46, 1980, 2, 6556}, {46, 1985, 1, 6556}, {47, 1981, 1, 6556}, {47, 1986, 2, 6556}, {48, 1987, 1, 6556}, {37, 1970, 1, 6557}, {39, 1971, 2, 6557}, {39, 1976, 1, 6557}, {40, 1972, 2, 6557}, {40, 1982, 1, 6557}, {42, 1973, 1, 6557}, {42, 1977, 2, 6557}, {43, 1974, 2, 6557}, {43, 1978, 2, 6557}, {43, 1983, 2, 6557}, {44, 1975, 1, 6557}, {44, 1984, 2, 6557}, {46, 1979, 1, 6557}, {47, 1980, 2, 6557}, {47, 1985, 1, 6557}, {48, 1981, 1, 6557}, {48, 1986, 2, 6557}, {49, 1987, 1, 6557}, {38, 1970, 1, 6558}, {41, 1971, 2, 6558}, {41, 1976, 1, 6558}, {42, 1972, 2, 6558}, {42, 1982, 1, 6558}, {45, 1973, 1, 6558}, {45, 1977, 2, 6558}, {46, 1974, 2, 6558}, {46, 1978, 2, 6558}, {46, 1983, 2, 6558}, {47, 1975, 1, 6558}, {47, 1984, 2, 6558}, {50, 1979, 1, 6558}, {51, 1980, 2, 6558}, {51, 1985, 1, 6558}, {52, 1981, 1, 6558}, {52, 1986, 2, 6558}, {53, 1987, 1, 6558}, {39, 1970, 1, 6559}, {42, 1971, 2, 6559}, {42, 1976, 1, 6559}, {43, 1972, 2, 6559}, {43, 1982, 1, 6559}, {46, 1973, 1, 6559}, {46, 1977, 2, 6559}, {47, 1974, 2, 6559}, {47, 1978, 2, 6559}, {47, 1983, 2, 6559}, {48, 1975, 1, 6559}, {48, 1984, 2, 6559}, {51, 1979, 1, 6559}, {52, 1980, 2, 6559}, {52, 1985, 1, 6559}, {53, 1981, 1, 6559}, {53, 1986, 2, 6559}, {54, 1987, 1, 6559}, {40, 1970, 1, 6560}, {43, 1971, 2, 6560}, {43, 1976, 1, 6560}, {44, 1972, 2, 6560}, {44, 1982, 1, 6560}, {47, 1973, 1, 6560}, {47, 1977, 2, 6560}, {48, 1974, 2, 6560}, {48, 1978, 2, 6560}, {48, 1983, 2, 6560}, {49, 1975, 1, 6560}, {49, 1984, 2, 6560}, {52, 1979, 1, 6560}, {53, 1980, 2, 6560}, {53, 1985, 1, 6560}, {54, 1981, 1, 6560},

{54, 1986, 2, 6560}, {55, 1987, 1, 6560}, {35, 1814, 1, 6561}, {36, 1815, 3, 6561}, {36, 1824, 1, 6561}, {37, 1816, 3, 6561}, {37, 1834, 1, 6561}, {38, 1817, 3, 6561}, {38, 1825, 3, 6561}, {39, 1818, 6, 6561}, {39, 1826, 3, 6561}, {39, 1835, 3, 6561}, {40, 1819, 3, 6561}, {40, 1836, 3, 6561}, {41, 1820, 1, 6561}, {41, 1827, 3, 6561}, {42, 1821, 3, 6561}, {42, 1828, 6, 6561}, {42, 1837, 3, 6561}, {43, 1822, 3, 6561}, {43, 1829, 3, 6561}, {43, 1838, 6, 6561}, {44, 1823, 1, 6561}, {44, 1839, 3, 6561}, {45, 1830, 1, 6561}, {46, 1831, 3, 6561}, {46, 1840, 1, 6561}, {47, 1832, 3, 6561}, {47, 1841, 3, 6561}, {48, 1833, 1, 6561}, {48, 1842, 3, 6561}, {49, 1843, 1, 6561}, {35, 1824, 1, 6562}, {36, 1825, 3, 6562}, {36, 1844, 1, 6562}, {37, 1826, 3, 6562}, {37, 1854, 1, 6562}, {38, 1827, 3, 6562}, {38, 1845, 3, 6562}, {39, 1828, 6, 6562}, {39, 1846, 3, 6562}, {39, 1855, 3, 6562}, {40, 1829, 3, 6562}, {40, 1856, 3, 6562}, {41, 1830, 1, 6562}, {41, 1847, 3, 6562}, {42, 1831, 3, 6562}, {42, 1848, 6, 6562}, {42, 1857, 3, 6562}, {43, 1832, 3, 6562}, {43, 1849, 3, 6562}, {43, 1858, 6, 6562}, {44, 1833, 1, 6562}, {44, 1859, 3, 6562}, {45, 1850, 1, 6562}, {46, 1851, 3, 6562}, {46, 1860, 1, 6562}, {47, 1852, 3, 6562}, {47, 1861, 3, 6562}, {48, 1853, 1, 6562}, {48, 1862, 3, 6562}, {49, 1863, 1, 6562}, {35, 1834, 1, 6563}, {36, 1835, 3, 6563}, {36, 1854, 1, 6563}, {37, 1836, 3, 6563}, {37, 1864, 1, 6563}, {38, 1837, 3, 6563}, {38, 1855, 3, 6563}, {39, 1838, 6, 6563}, {39, 1856, 3, 6563}, {39, 1865, 3, 6563}, {40, 1839, 3, 6563}, {40, 1866, 3, 6563}, {41, 1840, 1, 6563}, {41, 1857, 3, 6563}, {42, 1841, 3, 6563}, {42, 1858, 6, 6563}, {42, 1867, 3, 6563}, {43, 1842, 3, 6563}, {43, 1859, 3, 6563}, {43, 1868, 6, 6563}, {44, 1843, 1, 6563}, {44, 1869, 3, 6563}, {45, 1860, 1, 6563}, {46, 1861, 3, 6563}, {46, 1870, 1, 6563}, {47, 1862, 3, 6563}, {47, 1871, 3, 6563}, {48, 1863, 1, 6563}, {48, 1872, 3, 6563}, {49, 1873, 1, 6563}, {36, 1814, 1, 6564}, {38, 1815, 3, 6564}, {38, 1824, 1, 6564}, {39, 1816, 3, 6564}, {39, 1834, 1, 6564}, {41, 1817, 3, 6564}, {41, 1825, 3, 6564}, {42, 1818, 6, 6564}, {42, 1826, 3, 6564}, {42, 1835, 3, 6564}, {43, 1819, 3, 6564}, {43, 1836, 3, 6564}, {45, 1820, 1, 6564}, {45, 1827, 3, 6564}, {46, 1821, 3, 6564}, {46, 1828, 6, 6564}, {46, 1837, 3, 6564}, {47, 1822, 3, 6564}, {47, 1829, 3, 6564}, {47, 1838, 6, 6564}, {48, 1823, 1, 6564}, {48, 1839, 3, 6564}, {50, 1830, 1, 6564}, {51, 1831, 3, 6564}, {51, 1840, 1, 6564}, {52, 1832, 3, 6564}, {52, 1841, 3, 6564}, {53, 1833, 1, 6564}, {53, 1842, 3, 6564}, {54, 1843, 1, 6564}, {36, 1824, 1, 6565}, {38, 1825, 3, 6565}, {38, 1844, 1, 6565}, {39, 1826, 3, 6565}, {39, 1854, 1, 6565}, {41, 1827, 3, 6565}, {41, 1845, 3, 6565}, {42, 1828, 6, 6565}, {42, 1846, 3, 6565}, {42, 1855, 3, 6565}, {43, 1829, 3, 6565}, {43, 1856, 3, 6565}, {45, 1830, 1, 6565}, {45, 1847, 3, 6565}, {46, 1831, 3, 6565}, {46, 1848, 6, 6565}, {46, 1857, 3, 6565}, {47, 1832, 3, 6565}, {47, 1849, 3, 6565}, {47, 1858, 6, 6565}, {48, 1833, 1, 6565}, {48, 1859, 3, 6565}, {50, 1850, 1, 6565}, {51, 1851, 3, 6565}, {51, 1860, 1, 6565}, {52, 1852, 3, 6565}, {52, 1861, 3, 6565}, {53, 1853, 1, 6565}, {53, 1862, 3, 6565}, {54, 1863, 1, 6565}, {36, 1834, 1, 6566}, {38, 1835, 3, 6566}, {38, 1854, 1, 6566}, {39, 1836, 3, 6566}, {39, 1864, 1, 6566}, {41, 1837, 3, 6566}, {41, 1855, 3, 6566}, {42, 1838, 6, 6566}, {42, 1856, 3, 6566}, {42, 1865, 3, 6566}, {43, 1839, 3, 6566}, {43, 1866, 3, 6566}, {45, 1840, 1, 6566}, {45, 1857, 3, 6566}, {46, 1841, 3, 6566}, {46, 1858, 6, 6566}, {46, 1867, 3, 6566}, {47, 1842, 3, 6566}, {47, 1859, 3, 6566}, {47, 1868, 6, 6566}, {48, 1843, 1, 6566}, {48, 1869, 3, 6566}, {50, 1860, 1, 6566}, {51, 1861, 3, 6566}, {51, 1870, 1, 6566}, {52, 1862, 3, 6566}, {52, 1871, 3, 6566}, {53, 1863, 1, 6566}, {53, 1872, 3, 6566}, {54, 1873, 1, 6566}, {37, 1814, 1, 6567}, {39, 1815, 3, 6567}, {39, 1824, 1, 6567}, {40, 1816, 3, 6567}, {40, 1834, 1, 6567}, {42, 1817, 3, 6567}, {42, 1825, 3, 6567}, {43, 1818, 6, 6567}, {43, 1826, 3, 6567}, {43, 1835, 3, 6567}, {44, 1819, 3, 6567}, {44, 1836, 3, 6567}, {46, 1820, 1, 6567}, {46, 1827, 3, 6567}, {47, 1821, 3, 6567}, {47, 1828, 6, 6567}, {47, 1837, 3, 6567}, {48, 1822, 3, 6567}, {48, 1829, 3, 6567}, {48, 1838, 6, 6567}, {49, 1823, 1, 6567}, {49, 1839, 3, 6567}, {51, 1830, 1, 6567}, {52, 1831, 3, 6567}, {52, 1840, 1, 6567}, {53, 1832, 3, 6567}, {53, 1841, 3, 6567}, {54, 1833, 1, 6567}, {54, 1842, 3, 6567}, {55, 1843, 1, 6567}, {37, 1824, 1, 6568}, {39, 1825, 3, 6568}, {39, 1844, 1, 6568}, {40, 1826, 3, 6568}, {40, 1854, 1, 6568}, {42, 1827, 3, 6568}, {42, 1845, 3, 6568}, {43, 1828, 6, 6568}, {43, 1846, 3, 6568}, {43, 1855, 3, 6568}, {44, 1829, 3, 6568}, {44, 1856, 3, 6568}, {46, 1830, 1, 6568}, {46, 1847, 3, 6568}, {47, 1831, 3, 6568}, {47, 1848, 6, 6568}, {47, 1857, 3, 6568}, {48, 1832, 3, 6568}, {48, 1849, 3, 6568}, {48, 1858, 6, 6568}, {49, 1833, 1, 6568}, {49, 1859, 3, 6568}, {51, 1850, 1, 6568}, {52, 1851, 3, 6568}, {52, 1860, 1, 6568}, {53, 1852, 3, 6568}, {53, 1861, 3, 6568}, {54, 1853, 1, 6568}, {54, 1862, 3, 6568}, {55, 1863, 1, 6568}, {37, 1834, 1, 6569}, {39, 1835, 3, 6569}, {39, 1854, 1, 6569}, {40, 1836, 3, 6569}, {40, 1864, 1, 6569}, {42, 1837, 3, 6569}, {42, 1855, 3, 6569}, {43, 1838, 6, 6569}, {43, 1856, 3, 6569}, {43, 1865, 3, 6569}, {44, 1839, 3, 6569}, {44, 1866, 3, 6569}, {46, 1840, 1, 6569}, {46, 1857, 3, 6569}, {47, 1841, 3, 6569}, {47, 1858, 6, 6569}, {47, 1867, 3, 6569}, {48, 1842, 3, 6569}, {48, 1859, 3, 6569}, {48, 1868, 6, 6569}, {49, 1843, 1, 6569}, {49, 1869, 3, 6569}, {51, 1860, 1, 6569}, {52, 1861, 3, 6569}, {52, 1870, 1, 6569}, {53, 1862, 3, 6569}, {53, 1871, 3, 6569}, {54, 1863, 1, 6569}, {54, 1872, 3, 6569}, {55, 1873, 1, 6569}, {35, 1988, 1, 6570}, {36, 1989, 3, 6570}, {36, 1998, 1, 6570}, {37, 1990, 3, 6570}, {37, 2008, 1, 6570}, {38, 1991, 3, 6570}, {38, 1999, 3, 6570}, {39, 1992, 6, 6570}, {39, 2000, 3, 6570}, {39, 2009, 3, 6570}, {40, 1993, 3, 6570}, {40, 2010, 3, 6570}, {41, 1994, 1, 6570}, {41, 2001, 3, 6570}, {42, 1995, 3, 6570}, {42, 2002, 6, 6570}, {42, 2011, 3, 6570}, {43, 1996, 3, 6570}, {43, 2003, 3, 6570}, {43, 2012, 6, 6570}, {44, 1997, 1, 6570}, {44,

2013, 3, 6570}, {45, 2004, 1, 6570}, {46, 2005, 3, 6570}, {46, 2014, 1, 6570}, {47, 2006, 3, 6570}, {47, 2015, 3, 6570}, {48, 2007, 1, 6570}, {48, 2016, 3, 6570}, {49, 2017, 1, 6570}, {36, 1988, 1, 6571}, {38, 1989, 3, 6571}, {38, 1998, 1, 6571}, {39, 1990, 3, 6571}, {39, 2008, 1, 6571}, {41, 1991, 3, 6571}, {41, 1999, 3, 6571}, {42, 1992, 6, 6571}, {42, 2000, 3, 6571}, {42, 2009, 3, 6571}, {43, 1993, 3, 6571}, {43, 2010, 3, 6571}, {45, 1994, 1, 6571}, {45, 2001, 3, 6571}, {46, 1995, 3, 6571}, {46, 2002, 6, 6571}, {46, 2011, 3, 6571}, {47, 1996, 3, 6571}, {47, 2003, 3, 6571}, {47, 2012, 6, 6571}, {48, 1997, 1, 6571}, {48, 2013, 3, 6571}, {50, 2004, 1, 6571}, {51, 2005, 3, 6571}, {51, 2014, 1, 6571}, {52, 2006, 3, 6571}, {52, 2015, 3, 6571}, {53, 2007, 1, 6571}, {53, 2016, 3, 6571}, {54, 2017, 1, 6571}, {37, 1988, 1, 6572}, {39, 1989, 3, 6572}, {39, 1998, 1, 6572}, {40, 1990, 3, 6572}, {40, 2008, 1, 6572}, {42, 1991, 3, 6572}, {42, 1999, 3, 6572}, {43, 1992, 6, 6572}, {43, 2000, 3, 6572}, {43, 2009, 3, 6572}, {44, 1993, 3, 6572}, {44, 2010, 3, 6572}, {46, 1994, 1, 6572}, {46, 2001, 3, 6572}, {47, 1995, 3, 6572}, {47, 2002, 6, 6572}, {47, 2011, 3, 6572}, {48, 1996, 3, 6572}, {48, 2003, 3, 6572}, {48, 2012, 6, 6572}, {49, 1997, 1, 6572}, {49, 2013, 3, 6572}, {51, 2004, 1, 6572}, {52, 2005, 3, 6572}, {52, 2014, 1, 6572}, {53, 2006, 3, 6572}, {53, 2015, 3, 6572}, {54, 2007, 1, 6572}, {54, 2016, 3, 6572}, {55, 2017, 1, 6572}, {35, 2018, 1, 6573}, {36, 2019, 4, 6573}, {36, 2033, 1, 6573}, {37, 2020, 4, 6573}, {37, 2048, 1, 6573}, {38, 2021, 6, 6573}, {38, 2034, 4, 6573}, {39, 2022, 12, 6573}, {39, 2035, 4, 6573}, {39, 2049, 4, 6573}, {40, 2023, 6, 6573}, {40, 2050, 4, 6573}, {41, 2024, 4, 6573}, {41, 2036, 6, 6573}, {42, 2025, 12, 6573}, {42, 2037, 12, 6573}, {42, 2051, 6, 6573}, {43, 2026, 12, 6573}, {43, 2038, 6, 6573}, {43, 2052, 12, 6573}, {44, 2027, 4, 6573}, {44, 2053, 6, 6573}, {45, 2028, 1, 6573}, {45, 2039, 4, 6573}, {46, 2029, 4, 6573}, {46, 2040, 12, 6573}, {46, 2054, 4, 6573}, {47, 2030, 6, 6573}, {47, 2041, 12, 6573}, {47, 2055, 12, 6573}, {48, 2031, 4, 6573}, {48, 2042, 4, 6573}, {48, 2056, 12, 6573}, {49, 2032, 1, 6573}, {49, 2057, 4, 6573}, {50, 2043, 1, 6573}, {51, 2044, 4, 6573}, {51, 2058, 1, 6573}, {52, 2045, 6, 6573}, {52, 2059, 4, 6573}, {53, 2046, 4, 6573}, {53, 2060, 6, 6573}, {54, 2047, 1, 6573}, {54, 2061, 4, 6573}, {55, 2062, 1, 6573}, {35, 2399, 1, 6574}, {36, 2400, 4, 6574}, {36, 2414, 1, 6574}, {37, 2401, 4, 6574}, {37, 2429, 1, 6574}, {38, 2402, 6, 6574}, {38, 2415, 4, 6574}, {39, 2403, 12, 6574}, {39, 2416, 4, 6574}, {39, 2430, 4, 6574}, {40, 2404, 6, 6574}, {40, 2431, 4, 6574}, {41, 2405, 4, 6574}, {41, 2417, 6, 6574}, {42, 2406, 12, 6574}, {42, 2418, 12, 6574}, {42, 2432, 6, 6574}, {43, 2407, 12, 6574}, {43, 2419, 6, 6574}, {43, 2433, 12, 6574}, {44, 2408, 4, 6574}, {44, 2434, 6, 6574}, {45, 2409, 1, 6574}, {45, 2420, 4, 6574}, {46, 2410, 4, 6574}, {46, 2421, 12, 6574}, {46, 2435, 4, 6574}, {47, 2411, 6, 6574}, {47, 2422, 12, 6574}, {47, 2436, 12, 6574}, {48, 2412, 4, 6574}, {48, 2423, 4, 6574}, {48, 2437, 12, 6574}, {49, 2413, 1, 6574}, {49, 2438, 4, 6574}, {50, 2424, 1, 6574}, {51, 2425, 4, 6574}, {51, 2439, 1, 6574}, {52, 2426, 6, 6574}, {52, 2440, 4, 6574}, {53, 2427, 4, 6574}, {53, 2441, 6, 6574}, {54, 2428, 1, 6574}, {54, 2442, 4, 6574}, {55, 2443, 1, 6574}, {35, 1588, 1, 6575}, {36, 1589, 4, 6575}, {37, 1590, 4, 6575}, {38, 1591, 2, 6575}, {38, 1598, 4, 6575}, {39, 1592, 4, 6575}, {39, 1599, 8, 6575}, {40, 1593, 2, 6575}, {40, 1607, 4, 6575}, {41, 1600, 4, 6575}, {42, 1601, 8, 6575}, {42, 1608, 4, 6575}, {43, 1602, 4, 6575}, {43, 1609, 8, 6575}, {44, 1610, 4, 6575}, {45, 1615, 1, 6575}, {46, 1616, 4, 6575}, {47, 1617, 2, 6575}, {47, 1622, 4, 6575}, {48, 1623, 4, 6575}, {49, 1628, 1, 6575}, {35, 1589, 1, 6576}, {36, 1591, 2, 6576}, {36, 1598, 2, 6576}, {37, 1592, 2, 6576}, {37, 1599, 2, 6576}, {38, 1594, 1, 6576}, {38, 1600, 5, 6576}, {39, 1595, 2, 6576}, {39, 1601, 6, 6576}, {39, 1608, 4, 6576}, {40, 1596, 1, 6576}, {40, 1602, 1, 6576}, {40, 1609, 4, 6576}, {41, 1603, 2, 6576}, {41, 1615, 2, 6576}, {42, 1604, 4, 6576}, {42, 1611, 2, 6576}, {42, 1616, 6, 6576}, {43, 1605, 2, 6576}, {43, 1612, 4, 6576}, {43, 1617, 2, 6576}, {43, 1622, 4, 6576}, {44, 1613, 2, 6576}, {44, 1623, 2, 6576}, {45, 1618, 1, 6576}, {46, 1619, 2, 6576}, {46, 1624, 2, 6576}, {47, 1620, 1, 6576}, {47, 1625, 4, 6576}, {47, 1629, 1, 6576}, {48, 1626, 2, 6576}, {48, 1630, 2, 6576}, {49, 1631, 1, 6576}, {35, 1590, 1, 6577}, {36, 1592, 2, 6577}, {36, 1599, 2, 6577}, {37, 1593, 2, 6577}, {37, 1607, 2, 6577}, {38, 1595, 1, 6577}, {38, 1601, 4, 6577}, {38, 1608, 1, 6577}, {39, 1596, 2, 6577}, {39, 1602, 4, 6577}, {39, 1609, 6, 6577}, {40, 1597, 1, 6577}, {40, 1610, 5, 6577}, {41, 1604, 2, 6577}, {41, 1616, 2, 6577}, {42, 1605, 4, 6577}, {42, 1612, 2, 6577}, {42, 1617, 2, 6577}, {42, 1622, 4, 6577}, {43, 1606, 2, 6577}, {43, 1613, 4, 6577}, {43, 1623, 6, 6577}, {44, 1614, 2, 6577}, {44, 1628, 2, 6577}, {45, 1619, 1, 6577}, {46, 1620, 2, 6577}, {46, 1625, 2, 6577}, {47, 1621, 1, 6577}, {47, 1626, 4, 6577}, {47, 1630, 1, 6577}, {48, 1627, 2, 6577}, {48, 1631, 2, 6577}, {49, 1632, 1, 6577}, {35, 1598, 1, 6578}, {36, 1600, 4, 6578}, {37, 1601, 4, 6578}, {38, 1603, 2, 6578}, {38, 1615, 4, 6578}, {39, 1604, 4, 6578}, {39, 1616, 8, 6578}, {40, 1605, 2, 6578}, {40, 1622, 4, 6578}, {41, 1618, 4, 6578}, {42, 1619, 8, 6578}, {42, 1624, 4, 6578}, {43, 1620, 4, 6578}, {43, 1625, 8, 6578}, {44, 1626, 4, 6578}, {45, 1633, 1, 6578}, {46, 1634, 4, 6578}, {47, 1635, 2, 6578}, {47, 1637, 4, 6578}, {48, 1638, 4, 6578}, {49, 1640, 1, 6578}, {35, 1599, 1, 6579}, {36, 1601, 2, 6579}, {36, 1608, 2, 6579}, {37, 1602, 2, 6579}, {37, 1609, 2, 6579}, {38, 1604, 1, 6579}, {38, 1611, 1, 6579}, {38, 1616, 4, 6579}, {39, 1605, 2, 6579}, {39, 1612, 2, 6579}, {39, 1617, 4, 6579}, {39, 1622, 4, 6579}, {40, 1606, 1, 6579}, {40, 1613, 1, 6579}, {40, 1623, 4, 6579}, {41, 1619, 2, 6579}, {41, 1624, 2, 6579}, {42, 1620, 4, 6579}, {42, 1625, 6, 6579}, {42, 1629, 2, 6579}, {43, 1621, 2, 6579}, {43, 1626, 6, 6579}, {43, 1630, 4, 6579}, {44, 1627, 2, 6579}, {44, 1631, 2, 6579}, {45,

1634, 1, 6579}, {46, 1635, 2, 6579}, {46, 1637, 2, 6579}, {47, 1636, 1, 6579}, {47, 1638, 5, 6579}, {48, 1639, 2, 6579}, {48, 1640, 2, 6579}, {49, 1641, 1, 6579}, {35, 1607, 1, 6580}, {36, 1609, 4, 6580}, {37, 1610, 4, 6580}, {38, 1612, 2, 6580}, {38, 1622, 4, 6580}, {39, 1613, 4, 6580}, {39, 1623, 8, 6580}, {40, 1614, 2, 6580}, {40, 1628, 4, 6580}, {41, 1625, 4, 6580}, {42, 1626, 8, 6580}, {42, 1630, 4, 6580}, {43, 1627, 4, 6580}, {43, 1631, 8, 6580}, {44, 1632, 4, 6580}, {45, 1637, 1, 6580}, {46, 1638, 4, 6580}, {47, 1639, 2, 6580}, {47, 1640, 4, 6580}, {48, 1641, 4, 6580}, {49, 1642, 1, 6580}, {36, 1588, 1, 6581}, {38, 1589, 4, 6581}, {39, 1590, 4, 6581}, {41, 1591, 2, 6581}, {41, 1598, 4, 6581}, {42, 1592, 4, 6581}, {42, 1599, 8, 6581}, {43, 1593, 2, 6581}, {43, 1607, 4, 6581}, {45, 1600, 4, 6581}, {46, 1601, 8, 6581}, {46, 1608, 4, 6581}, {47, 1602, 4, 6581}, {47, 1609, 8, 6581}, {48, 1610, 4, 6581}, {50, 1615, 1, 6581}, {51, 1616, 4, 6581}, {52, 1617, 2, 6581}, {52, 1622, 4, 6581}, {53, 1623, 4, 6581}, {54, 1628, 1, 6581}, {36, 1589, 1, 6582}, {38, 1591, 2, 6582}, {38, 1598, 2, 6582}, {39, 1592, 2, 6582}, {39, 1599, 2, 6582}, {41, 1594, 1, 6582}, {41, 1600, 5, 6582}, {42, 1595, 2, 6582}, {42, 1601, 6, 6582}, {42, 1608, 4, 6582}, {43, 1596, 1, 6582}, {43, 1602, 1, 6582}, {43, 1609, 4, 6582}, {45, 1603, 2, 6582}, {45, 1615, 2, 6582}, {46, 1604, 4, 6582}, {46, 1611, 2, 6582}, {46, 1616, 6, 6582}, {47, 1605, 2, 6582}, {47, 1612, 4, 6582}, {47, 1617, 2, 6582}, {47, 1622, 4, 6582}, {48, 1613, 2, 6582}, {48, 1623, 2, 6582}, {50, 1618, 1, 6582}, {51, 1619, 2, 6582}, {51, 1624, 2, 6582}, {52, 1620, 1, 6582}, {52, 1625, 4, 6582}, {52, 1629, 1, 6582}, {53, 1626, 2, 6582}, {53, 1630, 2, 6582}, {54, 1631, 1, 6582}, {36, 1590, 1, 6583}, {38, 1592, 2, 6583}, {38, 1599, 2, 6583}, {39, 1593, 2, 6583}, {39, 1607, 2, 6583}, {41, 1595, 1, 6583}, {41, 1601, 4, 6583}, {41, 1608, 1, 6583}, {42, 1596, 2, 6583}, {42, 1602, 4, 6583}, {42, 1609, 6, 6583}, {43, 1597, 1, 6583}, {43, 1610, 5, 6583}, {45, 1604, 2, 6583}, {45, 1616, 2, 6583}, {46, 1605, 4, 6583}, {46, 1612, 2, 6583}, {46, 1617, 2, 6583}, {46, 1622, 4, 6583}, {47, 1606, 2, 6583}, {47, 1613, 4, 6583}, {47, 1623, 6, 6583}, {48, 1614, 2, 6583}, {48, 1628, 2, 6583}, {50, 1619, 1, 6583}, {51, 1620, 2, 6583}, {51, 1625, 2, 6583}, {52, 1621, 1, 6583}, {52, 1626, 4, 6583}, {52, 1630, 1, 6583}, {53, 1627, 2, 6583}, {53, 1631, 2, 6583}, {54, 1632, 1, 6583}, {36, 1598, 1, 6584}, {38, 1600, 4, 6584}, {39, 1601, 4, 6584}, {41, 1603, 2, 6584}, {41, 1615, 4, 6584}, {42, 1604, 4, 6584}, {42, 1616, 8, 6584}, {43, 1605, 2, 6584}, {43, 1622, 4, 6584}, {45, 1618, 4, 6584}, {46, 1619, 8, 6584}, {46, 1624, 4, 6584}, {47, 1620, 4, 6584}, {47, 1625, 8, 6584}, {48, 1626, 4, 6584}, {50, 1633, 1, 6584}, {51, 1634, 4, 6584}, {52, 1635, 2, 6584}, {52, 1637, 4, 6584}, {53, 1638, 4, 6584}, {54, 1640, 1, 6584}, {36, 1599, 1, 6585}, {38, 1601, 2, 6585}, {38, 1608, 2, 6585}, {39, 1602, 2, 6585}, {39, 1609, 2, 6585}, {41, 1604, 1, 6585}, {41, 1611, 1, 6585}, {41, 1616, 4, 6585}, {42, 1605, 2, 6585}, {42, 1612, 2, 6585}, {42, 1617, 4, 6585}, {42, 1622, 4, 6585}, {43, 1606, 1, 6585}, {43, 1613, 1, 6585}, {43, 1623, 4, 6585}, {45, 1619, 2, 6585}, {45, 1624, 2, 6585}, {46, 1620, 4, 6585}, {46, 1625, 6, 6585}, {46, 1629, 2, 6585}, {47, 1621, 2, 6585}, {47, 1626, 6, 6585}, {47, 1630, 4, 6585}, {48, 1627, 2, 6585}, {48, 1631, 2, 6585}, {50, 1634, 1, 6585}, {51, 1635, 2, 6585}, {51, 1637, 2, 6585}, {52, 1636, 1, 6585}, {52, 1638, 5, 6585}, {53, 1639, 2, 6585}, {53, 1640, 2, 6585}, {54, 1641, 1, 6585}, {36, 1607, 1, 6586}, {38, 1609, 4, 6586}, {39, 1610, 4, 6586}, {41, 1612, 2, 6586}, {41, 1622, 4, 6586}, {42, 1613, 4, 6586}, {42, 1623, 8, 6586}, {43, 1614, 2, 6586}, {43, 1628, 4, 6586}, {45, 1625, 4, 6586}, {46, 1626, 8, 6586}, {46, 1630, 4, 6586}, {47, 1627, 4, 6586}, {47, 1631, 8, 6586}, {48, 1632, 4, 6586}, {50, 1637, 1, 6586}, {51, 1638, 4, 6586}, {52, 1639, 2, 6586}, {52, 1640, 4, 6586}, {53, 1641, 4, 6586}, {54, 1642, 1, 6586}, {37, 1588, 1, 6587}, {39, 1589, 4, 6587}, {40, 1590, 4, 6587}, {42, 1591, 2, 6587}, {42, 1598, 4, 6587}, {43, 1592, 4, 6587}, {43, 1599, 8, 6587}, {44, 1593, 2, 6587}, {44, 1607, 4, 6587}, {46, 1600, 4, 6587}, {47, 1601, 8, 6587}, {47, 1608, 4, 6587}, {48, 1602, 4, 6587}, {48, 1609, 8, 6587}, {49, 1610, 4, 6587}, {51, 1615, 1, 6587}, {52, 1616, 4, 6587}, {53, 1617, 2, 6587}, {53, 1622, 4, 6587}, {54, 1623, 4, 6587}, {55, 1628, 1, 6587}, {37, 1589, 1, 6588}, {39, 1591, 2, 6588}, {39, 1598, 2, 6588}, {40, 1592, 2, 6588}, {40, 1599, 2, 6588}, {42, 1594, 1, 6588}, {42, 1600, 5, 6588}, {43, 1595, 2, 6588}, {43, 1601, 6, 6588}, {43, 1608, 4, 6588}, {44, 1596, 1, 6588}, {44, 1602, 1, 6588}, {44, 1609, 4, 6588}, {46, 1603, 2, 6588}, {46, 1615, 2, 6588}, {47, 1604, 4, 6588}, {47, 1611, 2, 6588}, {47, 1616, 6, 6588}, {48, 1605, 2, 6588}, {48, 1612, 4, 6588}, {48, 1617, 2, 6588}, {48, 1622, 4, 6588}, {49, 1613, 2, 6588}, {49, 1623, 2, 6588}, {51, 1618, 1, 6588}, {52, 1619, 2, 6588}, {52, 1624, 2, 6588}, {53, 1620, 1, 6588}, {53, 1625, 4, 6588}, {53, 1629, 1, 6588}, {54, 1626, 2, 6588}, {54, 1630, 2, 6588}, {55, 1631, 1, 6588}, {37, 1590, 1, 6589}, {39, 1592, 2, 6589}, {39, 1599, 2, 6589}, {40, 1593, 2, 6589}, {40, 1607, 2, 6589}, {42, 1595, 1, 6589}, {42, 1601, 4, 6589}, {42, 1608, 1, 6589}, {43, 1596, 2, 6589}, {43, 1602, 4, 6589}, {43, 1609, 6, 6589}, {44, 1597, 1, 6589}, {44, 1610, 5, 6589}, {46, 1604, 2, 6589}, {46, 1616, 2, 6589}, {47, 1605, 4, 6589}, {47, 1612, 2, 6589}, {47, 1617, 2, 6589}, {47, 1622, 4, 6589}, {48, 1606, 2, 6589}, {48, 1613, 4, 6589}, {48, 1623, 6, 6589}, {49, 1614, 2, 6589}, {49, 1628, 2, 6589}, {51, 1619, 1, 6589}, {52, 1620, 2, 6589}, {52, 1625, 2, 6589}, {53, 1621, 1, 6589}, {53, 1626, 4, 6589}, {53, 1630, 1, 6589}, {54, 1627, 2, 6589}, {54, 1631, 2, 6589}, {55, 1632, 1, 6589}, {37, 1598, 1, 6590}, {39, 1600, 4, 6590}, {40, 1601, 4, 6590}, {42, 1603, 2, 6590}, {42, 1615, 4, 6590}, {43, 1604, 4, 6590}, {43, 1616, 8, 6590}, {44, 1605, 2, 6590}, {44, 1622, 4, 6590}, {46, 1618, 4, 6590}, {47, 1619, 8, 6590}, {47, 1624, 4, 6590}, {48, 1620, 4, 6590}, {48, 1625, 8, 6590}, {49, 1626, 4, 6590}, {51, 1633,

1, 6590}, {52, 1634, 4, 6590}, {53, 1635, 2, 6590}, {53, 1637, 4, 6590}, {54, 1638, 4, 6590}, {55, 1640, 1, 6590}, {37, 1599, 1, 6591}, {39, 1601, 2, 6591}, {39, 1608, 2, 6591}, {40, 1602, 2, 6591}, {40, 1609, 2, 6591}, {42, 1604, 1, 6591}, {42, 1611, 1, 6591}, {42, 1616, 4, 6591}, {43, 1605, 2, 6591}, {43, 1612, 2, 6591}, {43, 1617, 4, 6591}, {43, 1622, 4, 6591}, {44, 1606, 1, 6591}, {44, 1613, 1, 6591}, {44, 1623, 4, 6591}, {46, 1619, 2, 6591}, {46, 1624, 2, 6591}, {47, 1620, 4, 6591}, {47, 1625, 6, 6591}, {47, 1629, 2, 6591}, {48, 1621, 2, 6591}, {48, 1626, 6, 6591}, {48, 1630, 4, 6591}, {49, 1627, 2, 6591}, {49, 1631, 2, 6591}, {51, 1634, 1, 6591}, {52, 1635, 2, 6591}, {52, 1637, 2, 6591}, {53, 1636, 1, 6591}, {53, 1638, 5, 6591}, {54, 1639, 2, 6591}, {54, 1640, 2, 6591}, {55, 1641, 1, 6591}, {37, 1607, 1, 6592}, {39, 1609, 4, 6592}, {40, 1610, 4, 6592}, {42, 1612, 2, 6592}, {42, 1622, 4, 6592}, {43, 1613, 4, 6592}, {43, 1623, 8, 6592}, {44, 1614, 2, 6592}, {44, 1628, 4, 6592}, {46, 1625, 4, 6592}, {47, 1626, 8, 6592}, {47, 1630, 4, 6592}, {48, 1627, 4, 6592}, {48, 1631, 8, 6592}, {49, 1632, 4, 6592}, {51, 1637, 1, 6592}, {52, 1638, 4, 6592}, {53, 1639, 2, 6592}, {53, 1640, 4, 6592}, {54, 1641, 4, 6592}, {55, 1642, 1, 6592}, {35, 1793, 1, 6593}, {36, 1794, 4, 6593}, {37, 1795, 4, 6593}, {38, 1796, 2, 6593}, {38, 1799, 4, 6593}, {39, 1797, 4, 6593}, {39, 1800, 8, 6593}, {40, 1798, 2, 6593}, {40, 1804, 4, 6593}, {41, 1801, 4, 6593}, {42, 1802, 8, 6593}, {42, 1805, 4, 6593}, {43, 1803, 4, 6593}, {43, 1806, 8, 6593}, {44, 1807, 4, 6593}, {45, 1808, 1, 6593}, {46, 1809, 4, 6593}, {47, 1810, 2, 6593}, {47, 1811, 4, 6593}, {48, 1812, 4, 6593}, {49, 1813, 1, 6593}, {36, 1793, 1, 6594}, {38, 1794, 4, 6594}, {39, 1795, 4, 6594}, {41, 1796, 2, 6594}, {41, 1799, 4, 6594}, {42, 1797, 4, 6594}, {42, 1800, 8, 6594}, {43, 1798, 2, 6594}, {43, 1804, 4, 6594}, {45, 1801, 4, 6594}, {46, 1802, 8, 6594}, {46, 1805, 4, 6594}, {47, 1803, 4, 6594}, {47, 1806, 8, 6594}, {48, 1807, 4, 6594}, {50, 1808, 1, 6594}, {51, 1809, 4, 6594}, {52, 1810, 2, 6594}, {52, 1811, 4, 6594}, {53, 1812, 4, 6594}, {54, 1813, 1, 6594}, {37, 1793, 1, 6595}, {39, 1794, 4, 6595}, {40, 1795, 4, 6595}, {42, 1796, 2, 6595}, {42, 1799, 4, 6595}, {43, 1797, 4, 6595}, {43, 1800, 8, 6595}, {44, 1798, 2, 6595}, {44, 1804, 4, 6595}, {46, 1801, 4, 6595}, {47, 1802, 8, 6595}, {47, 1805, 4, 6595}, {48, 1803, 4, 6595}, {48, 1806, 8, 6595}, {49, 1807, 4, 6595}, {51, 1808, 1, 6595}, {52, 1809, 4, 6595}, {53, 1810, 2, 6595}, {53, 1811, 4, 6595}, {54, 1812, 4, 6595}, {55, 1813, 1, 6595}, {35, 1814, 1, 6596}, {36, 1815, 2, 6596}, {36, 1824, 2, 6596}, {37, 1816, 2, 6596}, {37, 1834, 2, 6596}, {38, 1817, 1, 6596}, {38, 1825, 4, 6596}, {38, 1844, 1, 6596}, {39, 1818, 2, 6596}, {39, 1826, 4, 6596}, {39, 1835, 4, 6596}, {39, 1854, 2, 6596}, {40, 1819, 1, 6596}, {40, 1836, 4, 6596}, {40, 1864, 1, 6596}, {41, 1827, 2, 6596}, {41, 1845, 2, 6596}, {42, 1828, 4, 6596}, {42, 1837, 2, 6596}, {42, 1846, 2, 6596}, {42, 1855, 4, 6596}, {43, 1829, 2, 6596}, {43, 1838, 4, 6596}, {43, 1856, 4, 6596}, {43, 1865, 2, 6596}, {44, 1839, 2, 6596}, {44, 1866, 2, 6596}, {45, 1847, 1, 6596}, {46, 1848, 2, 6596}, {46, 1857, 2, 6596}, {47, 1849, 1, 6596}, {47, 1858, 4, 6596}, {47, 1867, 1, 6596}, {48, 1859, 2, 6596}, {48, 1868, 2, 6596}, {49, 1869, 1, 6596}, {35, 1815, 1, 6597}, {36, 1817, 2, 6597}, {36, 1825, 2, 6597}, {37, 1818, 2, 6597}, {37, 1835, 2, 6597}, {38, 1820, 1, 6597}, {38, 1827, 4, 6597}, {38, 1845, 1, 6597}, {39, 1821, 2, 6597}, {39, 1828, 4, 6597}, {39, 1837, 4, 6597}, {39, 1855, 2, 6597}, {40, 1822, 1, 6597}, {40, 1838, 4, 6597}, {40, 1865, 1, 6597}, {41, 1830, 2, 6597}, {41, 1847, 2, 6597}, {42, 1831, 4, 6597}, {42, 1840, 2, 6597}, {42, 1848, 2, 6597}, {42, 1857, 4, 6597}, {43, 1832, 2, 6597}, {43, 1841, 4, 6597}, {43, 1858, 4, 6597}, {43, 1867, 2, 6597}, {44, 1842, 2, 6597}, {44, 1868, 2, 6597}, {45, 1850, 1, 6597}, {46, 1851, 2, 6597}, {46, 1860, 2, 6597}, {47, 1852, 1, 6597}, {47, 1861, 4, 6597}, {47, 1870, 1, 6597}, {48, 1862, 2, 6597}, {48, 1871, 2, 6597}, {49, 1872, 1, 6597}, {35, 1816, 1, 6598}, {36, 1818, 2, 6598}, {36, 1826, 2, 6598}, {37, 1819, 2, 6598}, {37, 1836, 2, 6598}, {38, 1821, 1, 6598}, {38, 1828, 4, 6598}, {38, 1846, 1, 6598}, {39, 1822, 2, 6598}, {39, 1829, 4, 6598}, {39, 1838, 4, 6598}, {39, 1856, 2, 6598}, {40, 1823, 1, 6598}, {40, 1839, 4, 6598}, {40, 1866, 1, 6598}, {41, 1831, 2, 6598}, {41, 1848, 2, 6598}, {42, 1832, 4, 6598}, {42, 1841, 2, 6598}, {42, 1849, 2, 6598}, {42, 1858, 4, 6598}, {43, 1833, 2, 6598}, {43, 1842, 4, 6598}, {43, 1859, 4, 6598}, {43, 1868, 2, 6598}, {44, 1843, 2, 6598}, {44, 1869, 2, 6598}, {45, 1851, 1, 6598}, {46, 1852, 2, 6598}, {46, 1861, 2, 6598}, {47, 1853, 1, 6598}, {47, 1862, 4, 6598}, {47, 1871, 1, 6598}, {48, 1863, 2, 6598}, {48, 1872, 2, 6598}, {49, 1873, 1, 6598}, {36, 1814, 1, 6599}, {38, 1815, 2, 6599}, {38, 1824, 2, 6599}, {39, 1816, 2, 6599}, {39, 1834, 2, 6599}, {41, 1817, 1, 6599}, {41, 1825, 4, 6599}, {41, 1844, 1, 6599}, {42, 1818, 2, 6599}, {42, 1826, 4, 6599}, {42, 1835, 4, 6599}, {42, 1854, 2, 6599}, {43, 1819, 1, 6599}, {43, 1836, 4, 6599}, {43, 1864, 1, 6599}, {45, 1827, 2, 6599}, {45, 1845, 2, 6599}, {46, 1828, 4, 6599}, {46, 1837, 2, 6599}, {46, 1846, 2, 6599}, {46, 1855, 4, 6599}, {47, 1829, 2, 6599}, {47, 1838, 4, 6599}, {47, 1856, 4, 6599}, {47, 1865, 2, 6599}, {48, 1839, 2, 6599}, {48, 1866, 2, 6599}, {50, 1847, 1, 6599}, {51, 1848, 2, 6599}, {51, 1857, 2, 6599}, {52, 1849, 1, 6599}, {52, 1858, 4, 6599}, {52, 1867, 1, 6599}, {53, 1859, 2, 6599}, {53, 1868, 2, 6599}, {54, 1869, 1, 6599}, {36, 1815, 1, 6600}, {38, 1817, 2, 6600}, {38, 1825, 2, 6600}, {39, 1818, 2, 6600}, {39, 1835, 2, 6600}, {41, 1820, 1, 6600}, {41, 1827, 4, 6600}, {41, 1845, 1, 6600}, {42, 1821, 2, 6600}, {42, 1828, 4, 6600}, {42, 1837, 4, 6600}, {42, 1855, 2, 6600}, {43, 1822, 1, 6600}, {43, 1838, 4, 6600}, {43, 1865, 1, 6600}, {45, 1830, 2, 6600}, {45, 1847, 2, 6600}, {46, 1831, 4, 6600}, {46, 1840, 2, 6600}, {46, 1848, 2, 6600}, {46, 1857, 4, 6600}, {47, 1832, 2, 6600}, {47, 1841, 4, 6600}, {47, 1858, 4, 6600}, {47, 1867, 2, 6600}, {48, 1842, 2, 6600}, {48, 1868, 2,

6600}, {50, 1850, 1, 6600}, {51, 1851, 2, 6600}, {51, 1860, 2, 6600}, {52, 1852, 1, 6600},
{52, 1861, 4, 6600}, {52, 1870, 1, 6600}, {53, 1862, 2, 6600}, {53, 1871, 2, 6600}, {54,
1872, 1, 6600}, {36, 1816, 1, 6601}, {38, 1818, 2, 6601}, {38, 1826, 2, 6601}, {39, 1819,
2, 6601}, {39, 1836, 2, 6601}, {41, 1821, 1, 6601}, {41, 1828, 4, 6601}, {41, 1846, 1,
6601}, {42, 1822, 2, 6601}, {42, 1829, 4, 6601}, {42, 1838, 4, 6601}, {42, 1856, 2, 6601},
{43, 1823, 1, 6601}, {43, 1839, 4, 6601}, {43, 1866, 1, 6601}, {45, 1831, 2, 6601}, {45,
1848, 2, 6601}, {46, 1832, 4, 6601}, {46, 1841, 2, 6601}, {46, 1849, 2, 6601}, {46, 1858,
4, 6601}, {47, 1833, 2, 6601}, {47, 1842, 4, 6601}, {47, 1859, 4, 6601}, {47, 1868, 2,
6601}, {48, 1843, 2, 6601}, {48, 1869, 2, 6601}, {50, 1851, 1, 6601}, {51, 1852, 2, 6601},
{51, 1861, 2, 6601}, {52, 1853, 1, 6601}, {52, 1862, 4, 6601}, {52, 1871, 1, 6601}, {53,
1863, 2, 6601}, {53, 1872, 2, 6601}, {54, 1873, 1, 6601}, {37, 1814, 1, 6602}, {39, 1815,
2, 6602}, {39, 1824, 2, 6602}, {40, 1816, 2, 6602}, {40, 1834, 2, 6602}, {42, 1817, 1,
6602}, {42, 1825, 4, 6602}, {42, 1844, 1, 6602}, {43, 1818, 2, 6602}, {43, 1826, 4, 6602},
{43, 1835, 4, 6602}, {43, 1854, 2, 6602}, {44, 1819, 1, 6602}, {44, 1836, 4, 6602}, {44,
1864, 1, 6602}, {46, 1827, 2, 6602}, {46, 1845, 2, 6602}, {47, 1828, 4, 6602}, {47, 1837,
2, 6602}, {47, 1846, 2, 6602}, {47, 1855, 4, 6602}, {48, 1829, 2, 6602}, {48, 1838, 4,
6602}, {48, 1856, 4, 6602}, {48, 1865, 2, 6602}, {49, 1839, 2, 6602}, {49, 1866, 2, 6602},
{51, 1847, 1, 6602}, {52, 1848, 2, 6602}, {52, 1857, 2, 6602}, {53, 1849, 1, 6602}, {53,
1858, 4, 6602}, {53, 1867, 1, 6602}, {54, 1859, 2, 6602}, {54, 1868, 2, 6602}, {55, 1869,
1, 6602}, {37, 1815, 1, 6603}, {39, 1817, 2, 6603}, {39, 1825, 2, 6603}, {40, 1818, 2,
6603}, {40, 1835, 2, 6603}, {42, 1820, 1, 6603}, {42, 1827, 4, 6603}, {42, 1845, 1, 6603},
{43, 1821, 2, 6603}, {43, 1828, 4, 6603}, {43, 1837, 4, 6603}, {43, 1855, 2, 6603}, {44,
1822, 1, 6603}, {44, 1838, 4, 6603}, {44, 1865, 1, 6603}, {46, 1830, 2, 6603}, {46, 1847,
2, 6603}, {47, 1831, 4, 6603}, {47, 1840, 2, 6603}, {47, 1848, 2, 6603}, {47, 1857, 4,
6603}, {48, 1832, 2, 6603}, {48, 1841, 4, 6603}, {48, 1858, 4, 6603}, {48, 1867, 2, 6603},
{49, 1842, 2, 6603}, {49, 1868, 2, 6603}, {51, 1850, 1, 6603}, {52, 1851, 2, 6603}, {52,
1860, 2, 6603}, {53, 1852, 1, 6603}, {53, 1861, 4, 6603}, {53, 1870, 1, 6603}, {54, 1862,
2, 6603}, {54, 1871, 2, 6603}, {55, 1872, 1, 6603}, {37, 1816, 1, 6604}, {39, 1818, 2,
6604}, {39, 1826, 2, 6604}, {40, 1819, 2, 6604}, {40, 1836, 2, 6604}, {42, 1821, 1, 6604},
{42, 1828, 4, 6604}, {42, 1846, 1, 6604}, {43, 1822, 2, 6604}, {43, 1829, 4, 6604}, {43,
1838, 4, 6604}, {43, 1856, 2, 6604}, {44, 1823, 1, 6604}, {44, 1839, 4, 6604}, {44, 1866,
1, 6604}, {46, 1831, 2, 6604}, {46, 1848, 2, 6604}, {47, 1832, 4, 6604}, {47, 1841, 2,
6604}, {47, 1849, 2, 6604}, {47, 1858, 4, 6604}, {48, 1833, 2, 6604}, {48, 1842, 4, 6604},
{48, 1859, 4, 6604}, {48, 1868, 2, 6604}, {49, 1843, 2, 6604}, {49, 1869, 2, 6604}, {51,
1851, 1, 6604}, {52, 1852, 2, 6604}, {52, 1861, 2, 6604}, {53, 1853, 1, 6604}, {53, 1862,
4, 6604}, {53, 1871, 1, 6604}, {54, 1863, 2, 6604}, {54, 1872, 2, 6604}, {55, 1873, 1,
6604}, {35, 2174, 1, 6605}, {36, 2175, 4, 6605}, {37, 2176, 4, 6605}, {38, 2177, 2, 6605},
{38, 2180, 4, 6605}, {39, 2178, 4, 6605}, {39, 2181, 8, 6605}, {40, 2179, 2, 6605}, {40,
2185, 4, 6605}, {41, 2182, 4, 6605}, {42, 2183, 8, 6605}, {42, 2186, 4, 6605}, {43, 2184,
4, 6605}, {43, 2187, 8, 6605}, {44, 2188, 4, 6605}, {45, 2189, 1, 6605}, {46, 2190, 4,
6605}, {47, 2191, 2, 6605}, {47, 2192, 4, 6605}, {48, 2193, 4, 6605}, {49, 2194, 1, 6605},
{36, 2174, 1, 6606}, {38, 2175, 4, 6606}, {39, 2176, 4, 6606}, {41, 2177, 2, 6606}, {41,
2180, 4, 6606}, {42, 2178, 4, 6606}, {42, 2181, 8, 6606}, {43, 2179, 2, 6606}, {43, 2185,
4, 6606}, {45, 2182, 4, 6606}, {46, 2183, 8, 6606}, {46, 2186, 4, 6606}, {47, 2184, 4,
6606}, {47, 2187, 8, 6606}, {48, 2188, 4, 6606}, {50, 2189, 1, 6606}, {51, 2190, 4, 6606},
{52, 2191, 2, 6606}, {52, 2192, 4, 6606}, {53, 2193, 4, 6606}, {54, 2194, 1, 6606}, {37,
2174, 1, 6607}, {39, 2175, 4, 6607}, {40, 2176, 4, 6607}, {42, 2177, 2, 6607}, {42, 2180,
4, 6607}, {43, 2178, 4, 6607}, {43, 2181, 8, 6607}, {44, 2179, 2, 6607}, {44, 2185, 4,
6607}, {46, 2182, 4, 6607}, {47, 2183, 8, 6607}, {47, 2186, 4, 6607}, {48, 2184, 4, 6607},
{48, 2187, 8, 6607}, {49, 2188, 4, 6607}, {51, 2189, 1, 6607}, {52, 2190, 4, 6607}, {53,
2191, 2, 6607}, {53, 2192, 4, 6607}, {54, 2193, 4, 6607}, {55, 2194, 1, 6607}, {35, 1814,
1, 6608}, {36, 1815, 3, 6608}, {36, 1824, 2, 6608}, {37, 1816, 3, 6608}, {37, 1834, 2,
6608}, {38, 1817, 3, 6608}, {38, 1825, 6, 6608}, {38, 1844, 1, 6608}, {39, 1818, 6, 6608},
{39, 1826, 6, 6608}, {39, 1835, 6, 6608}, {39, 1854, 2, 6608}, {40, 1819, 3, 6608}, {40,
1836, 6, 6608}, {40, 1864, 1, 6608}, {41, 1820, 1, 6608}, {41, 1827, 6, 6608}, {41, 1845,
3, 6608}, {42, 1821, 3, 6608}, {42, 1828, 12, 6608}, {42, 1837, 6, 6608}, {42, 1846, 3,
6608}, {42, 1855, 6, 6608}, {43, 1822, 3, 6608}, {43, 1829, 6, 6608}, {43, 1838, 12,
6608}, {43, 1856, 6, 6608}, {43, 1865, 3, 6608}, {44, 1823, 1, 6608}, {44, 1839, 6, 6608},
{44, 1866, 3, 6608}, {45, 1830, 2, 6608}, {45, 1847, 3, 6608}, {46, 1831, 6, 6608}, {46,
1840, 2, 6608}, {46, 1848, 6, 6608}, {46, 1857, 6, 6608}, {47, 1832, 6, 6608}, {47, 1841,
6, 6608}, {47, 1849, 3, 6608}, {47, 1858, 12, 6608}, {47, 1867, 3, 6608}, {48, 1833, 2,
6608}, {48, 1842, 6, 6608}, {48, 1859, 6, 6608}, {48, 1868, 6, 6608}, {49, 1843, 2, 6608},
{49, 1869, 3, 6608}, {50, 1850, 1, 6608}, {51, 1851, 3, 6608}, {51, 1860, 2, 6608}, {52,
1852, 3, 6608}, {52, 1861, 6, 6608}, {52, 1870, 1, 6608}, {53, 1853, 1, 6608}, {53, 1862,
6, 6608}, {53, 1871, 3, 6608}, {54, 1863, 2, 6608}, {54, 1872, 3, 6608}, {55, 1873, 1,
6608}, {35, 1874, 1, 6609}, {36, 1875, 3, 6609}, {36, 1889, 2, 6609}, {37, 1876, 3, 6609},
{37, 1904, 2, 6609}, {38, 1877, 3, 6609}, {38, 1890, 6, 6609}, {38, 1919, 1, 6609}, {39,
1878, 6, 6609}, {39, 1891, 6, 6609}, {39, 1905, 6, 6609}, {39, 1934, 2, 6609}, {40, 1879,
3, 6609}, {40, 1906, 6, 6609}, {40, 1949, 1, 6609}, {41, 1880, 1, 6609}, {41, 1892, 6,

6609}, {41, 1920, 3, 6609}, {42, 1881, 3, 6609}, {42, 1893, 12, 6609}, {42, 1907, 6, 6609}, {42, 1921, 3, 6609}, {42, 1935, 6, 6609}, {43, 1882, 3, 6609}, {43, 1894, 6, 6609}, {43, 1908, 12, 6609}, {43, 1936, 6, 6609}, {43, 1950, 3, 6609}, {44, 1883, 1, 6609}, {44, 1909, 6, 6609}, {44, 1951, 3, 6609}, {45, 1895, 2, 6609}, {45, 1922, 3, 6609}, {46, 1896, 6, 6609}, {46, 1910, 2, 6609}, {46, 1923, 6, 6609}, {46, 1937, 6, 6609}, {47, 1897, 6, 6609}, {47, 1911, 6, 6609}, {47, 1924, 3, 6609}, {47, 1938, 12, 6609}, {47, 1952, 3, 6609}, {48, 1898, 2, 6609}, {48, 1912, 6, 6609}, {48, 1939, 6, 6609}, {48, 1953, 6, 6609}, {49, 1913, 2, 6609}, {49, 1954, 3, 6609}, {50, 1925, 1, 6609}, {51, 1926, 3, 6609}, {51, 1940, 2, 6609}, {52, 1927, 3, 6609}, {52, 1941, 6, 6609}, {52, 1955, 1, 6609}, {53, 1928, 1, 6609}, {53, 1942, 6, 6609}, {53, 1956, 3, 6609}, {54, 1943, 2, 6609}, {54, 1957, 3, 6609}, {55, 1958, 1, 6609}, {35, 1875, 1, 6610}, {36, 1877, 3, 6610}, {36, 1890, 2, 6610}, {37, 1878, 3, 6610}, {37, 1905, 2, 6610}, {38, 1880, 3, 6610}, {38, 1892, 6, 6610}, {38, 1920, 1, 6610}, {39, 1881, 6, 6610}, {39, 1893, 6, 6610}, {39, 1907, 6, 6610}, {39, 1935, 2, 6610}, {40, 1882, 3, 6610}, {40, 1908, 6, 6610}, {40, 1950, 1, 6610}, {41, 1884, 1, 6610}, {41, 1895, 6, 6610}, {41, 1922, 3, 6610}, {42, 1885, 3, 6610}, {42, 1896, 12, 6610}, {42, 1910, 6, 6610}, {42, 1923, 3, 6610}, {42, 1937, 6, 6610}, {43, 1886, 3, 6610}, {43, 1897, 6, 6610}, {43, 1911, 12, 6610}, {43, 1938, 6, 6610}, {43, 1952, 3, 6610}, {44, 1887, 1, 6610}, {44, 1912, 6, 6610}, {44, 1953, 3, 6610}, {45, 1899, 2, 6610}, {45, 1925, 3, 6610}, {46, 1900, 6, 6610}, {46, 1914, 2, 6610}, {46, 1926, 6, 6610}, {46, 1940, 6, 6610}, {47, 1901, 6, 6610}, {47, 1915, 6, 6610}, {47, 1927, 3, 6610}, {47, 1941, 12, 6610}, {47, 1955, 3, 6610}, {48, 1902, 2, 6610}, {48, 1916, 6, 6610}, {48, 1942, 6, 6610}, {48, 1956, 6, 6610}, {49, 1917, 2, 6610}, {49, 1957, 3, 6610}, {50, 1929, 1, 6610}, {51, 1930, 3, 6610}, {51, 1944, 2, 6610}, {52, 1931, 3, 6610}, {52, 1945, 6, 6610}, {52, 1959, 1, 6610}, {53, 1932, 1, 6610}, {53, 1946, 6, 6610}, {53, 1960, 3, 6610}, {54, 1947, 2, 6610}, {54, 1961, 3, 6610}, {55, 1962, 1, 6610}, {35, 1876, 1, 6611}, {36, 1878, 3, 6611}, {36, 1891, 2, 6611}, {37, 1879, 3, 6611}, {37, 1906, 2, 6611}, {38, 1881, 3, 6611}, {38, 1893, 6, 6611}, {38, 1921, 1, 6611}, {39, 1882, 6, 6611}, {39, 1894, 6, 6611}, {39, 1908, 6, 6611}, {39, 1936, 2, 6611}, {40, 1883, 3, 6611}, {40, 1909, 6, 6611}, {40, 1951, 1, 6611}, {41, 1885, 1, 6611}, {41, 1896, 6, 6611}, {41, 1923, 3, 6611}, {42, 1886, 3, 6611}, {42, 1897, 12, 6611}, {42, 1911, 6, 6611}, {42, 1924, 3, 6611}, {42, 1938, 6, 6611}, {43, 1887, 3, 6611}, {43, 1898, 6, 6611}, {43, 1912, 12, 6611}, {43, 1939, 6, 6611}, {43, 1953, 3, 6611}, {44, 1888, 1, 6611}, {44, 1913, 6, 6611}, {44, 1954, 3, 6611}, {45, 1900, 2, 6611}, {45, 1926, 3, 6611}, {46, 1901, 6, 6611}, {46, 1915, 2, 6611}, {46, 1927, 6, 6611}, {46, 1941, 6, 6611}, {47, 1902, 6, 6611}, {47, 1916, 6, 6611}, {47, 1928, 3, 6611}, {47, 1942, 12, 6611}, {47, 1956, 3, 6611}, {48, 1903, 2, 6611}, {48, 1917, 6, 6611}, {48, 1943, 6, 6611}, {48, 1957, 6, 6611}, {49, 1918, 2, 6611}, {49, 1958, 3, 6611}, {50, 1930, 1, 6611}, {51, 1931, 3, 6611}, {51, 1945, 2, 6611}, {52, 1932, 3, 6611}, {52, 1946, 6, 6611}, {52, 1960, 1, 6611}, {53, 1933, 1, 6611}, {53, 1947, 6, 6611}, {53, 1961, 3, 6611}, {54, 1948, 2, 6611}, {54, 1962, 3, 6611}, {55, 1963, 1, 6611}, {56, 1793, 1, 6612}, {57, 1794, 4, 6612}, {58, 1795, 4, 6612}, {59, 1796, 2, 6612}, {59, 1799, 4, 6612}, {60, 1797, 4, 6612}, {60, 1800, 8, 6612}, {61, 1798, 2, 6612}, {61, 1804, 4, 6612}, {62, 1801, 4, 6612}, {63, 1802, 8, 6612}, {63, 1805, 4, 6612}, {64, 1803, 4, 6612}, {64, 1806, 8, 6612}, {65, 1807, 4, 6612}, {66, 1808, 1, 6612}, {67, 1809, 4, 6612}, {68, 1810, 2, 6612}, {68, 1811, 4, 6612}, {69, 1812, 4, 6612}, {70, 1813, 1, 6612}, {57, 1793, 1, 6613}, {59, 1794, 4, 6613}, {60, 1795, 4, 6613}, {62, 1796, 2, 6613}, {62, 1799, 4, 6613}, {63, 1797, 4, 6613}, {63, 1800, 8, 6613}, {64, 1798, 2, 6613}, {64, 1804, 4, 6613}, {66, 1801, 4, 6613}, {67, 1802, 8, 6613}, {67, 1805, 4, 6613}, {68, 1803, 4, 6613}, {68, 1806, 8, 6613}, {69, 1807, 4, 6613}, {71, 1808, 1, 6613}, {72, 1809, 4, 6613}, {73, 1810, 2, 6613}, {73, 1811, 4, 6613}, {74, 1812, 4, 6613}, {75, 1813, 1, 6613}, {58, 1793, 1, 6614}, {60, 1794, 4, 6614}, {61, 1795, 4, 6614}, {63, 1796, 2, 6614}, {63, 1799, 4, 6614}, {64, 1797, 4, 6614}, {64, 1800, 8, 6614}, {65, 1798, 2, 6614}, {65, 1804, 4, 6614}, {67, 1801, 4, 6614}, {68, 1802, 8, 6614}, {68, 1805, 4, 6614}, {69, 1803, 4, 6614}, {69, 1806, 8, 6614}, {70, 1807, 4, 6614}, {72, 1808, 1, 6614}, {73, 1809, 4, 6614}, {74, 1810, 2, 6614}, {74, 1811, 4, 6614}, {75, 1812, 4, 6614}, {76, 1813, 1, 6614}, {59, 1793, 1, 6615}, {62, 1794, 4, 6615}, {63, 1795, 4, 6615}, {66, 1796, 2, 6615}, {66, 1799, 4, 6615}, {67, 1797, 4, 6615}, {67, 1800, 8, 6615}, {68, 1798, 2, 6615}, {68, 1804, 4, 6615}, {71, 1801, 4, 6615}, {72, 1802, 8, 6615}, {72, 1805, 4, 6615}, {73, 1803, 4, 6615}, {73, 1806, 8, 6615}, {74, 1807, 4, 6615}, {77, 1808, 1, 6615}, {78, 1809, 4, 6615}, {79, 1810, 2, 6615}, {79, 1811, 4, 6615}, {80, 1812, 4, 6615}, {81, 1813, 1, 6615}, {60, 1793, 1, 6616}, {63, 1794, 4, 6616}, {64, 1795, 4, 6616}, {67, 1796, 2, 6616}, {67, 1799, 4, 6616}, {68, 1797, 4, 6616}, {68, 1800, 8, 6616}, {69, 1798, 2, 6616}, {69, 1804, 4, 6616}, {72, 1801, 4, 6616}, {73, 1802, 8, 6616}, {73, 1805, 4, 6616}, {74, 1803, 4, 6616}, {74, 1806, 8, 6616}, {75, 1807, 4, 6616}, {78, 1808, 1, 6616}, {79, 1809, 4, 6616}, {80, 1810, 2, 6616}, {80, 1811, 4, 6616}, {81, 1812, 4, 6616}, {82, 1813, 1, 6616}, {61, 1793, 1, 6617}, {64, 1794, 4, 6617}, {65, 1795, 4, 6617}, {68, 1796, 2, 6617}, {68, 1799, 4, 6617}, {69, 1797, 4, 6617}, {69, 1800, 8, 6617}, {70, 1798, 2, 6617}, {70, 1804, 4, 6617}, {73, 1801, 4, 6617}, {74, 1802, 8, 6617}, {74, 1805, 4, 6617}, {75, 1803, 4, 6617}, {75, 1806, 8, 6617}, {76, 1807, 4, 6617}, {79, 1808, 1, 6617}, {80, 1809, 4, 6617}, {81, 1810, 2, 6617}, {81, 1811, 4, 6617}, {82, 1812, 4, 6617}, {83, 1813, 1, 6617}, {35, 2119, 1, 6618}, {36, 2120, 5, 6618}, {37, 2121, 5, 6618},

{38, 2122, 4, 6618}, {38, 2129, 6, 6618}, {39, 2123, 8, 6618}, {39, 2130, 12, 6618}, {40, 2124, 4, 6618}, {40, 2138, 6, 6618}, {41, 2125, 1, 6618}, {41, 2131, 9, 6618}, {42, 2126, 3, 6618}, {42, 2132, 18, 6618}, {42, 2139, 9, 6618}, {43, 2127, 3, 6618}, {43, 2133, 9, 6618}, {43, 2140, 18, 6618}, {44, 2128, 1, 6618}, {44, 2141, 9, 6618}, {45, 2134, 2, 6618}, {45, 2146, 3, 6618}, {46, 2135, 6, 6618}, {46, 2142, 2, 6618}, {46, 2147, 12, 6618}, {47, 2136, 6, 6618}, {47, 2143, 6, 6618}, {47, 2148, 6, 6618}, {47, 2153, 12, 6618}, {48, 2137, 2, 6618}, {48, 2144, 6, 6618}, {48, 2154, 12, 6618}, {49, 2145, 2, 6618}, {49, 2159, 3, 6618}, {50, 2149, 1, 6618}, {51, 2150, 3, 6618}, {51, 2155, 2, 6618}, {52, 2151, 3, 6618}, {52, 2156, 6, 6618}, {52, 2160, 1, 6618}, {53, 2152, 1, 6618}, {53, 2157, 6, 6618}, {53, 2161, 3, 6618}, {54, 2158, 2, 6618}, {54, 2162, 3, 6618}, {55, 2163, 1, 6618}, {35, 2120, 1, 6619}, {36, 2122, 2, 6619}, {36, 2129, 3, 6619}, {37, 2123, 2, 6619}, {37, 2130, 3, 6619}, {38, 2125, 1, 6619}, {38, 2131, 9, 6619}, {39, 2126, 2, 6619}, {39, 2132, 12, 6619}, {39, 2139, 6, 6619}, {40, 2127, 1, 6619}, {40, 2133, 3, 6619}, {40, 2140, 6, 6619}, {41, 2134, 4, 6619}, {41, 2146, 6, 6619}, {42, 2135, 9, 6619}, {42, 2142, 3, 6619}, {42, 2147, 18, 6619}, {43, 2136, 6, 6619}, {43, 2143, 6, 6619}, {43, 2148, 6, 6619}, {43, 2153, 12, 6619}, {44, 2137, 1, 6619}, {44, 2144, 3, 6619}, {44, 2154, 6, 6619}, {45, 2149, 5, 6619}, {46, 2150, 12, 6619}, {46, 2155, 8, 6619}, {47, 2151, 9, 6619}, {47, 2156, 18, 6619}, {47, 2160, 3, 6619}, {48, 2152, 2, 6619}, {48, 2157, 12, 6619}, {48, 2161, 6, 6619}, {49, 2158, 2, 6619}, {49, 2162, 3, 6619}, {50, 2164, 1, 6619}, {51, 2165, 5, 6619}, {52, 2166, 4, 6619}, {52, 2168, 6, 6619}, {53, 2167, 1, 6619}, {53, 2169, 9, 6619}, {54, 2170, 2, 6619}, {54, 2171, 3, 6619}, {55, 2172, 1, 6619}, {35, 2121, 1, 6620}, {36, 2123, 2, 6620}, {36, 2130, 3, 6620}, {37, 2124, 2, 6620}, {37, 2138, 3, 6620}, {38, 2126, 1, 6620}, {38, 2132, 6, 6620}, {38, 2139, 3, 6620}, {39, 2127, 2, 6620}, {39, 2133, 6, 6620}, {39, 2140, 12, 6620}, {40, 2128, 1, 6620}, {40, 2141, 9, 6620}, {41, 2135, 3, 6620}, {41, 2142, 1, 6620}, {41, 2147, 6, 6620}, {42, 2136, 6, 6620}, {42, 2143, 6, 6620}, {42, 2148, 6, 6620}, {42, 2153, 12, 6620}, {43, 2137, 3, 6620}, {43, 2144, 9, 6620}, {43, 2154, 18, 6620}, {44, 2145, 4, 6620}, {44, 2159, 6, 6620}, {45, 2150, 3, 6620}, {45, 2155, 2, 6620}, {46, 2151, 6, 6620}, {46, 2156, 12, 6620}, {46, 2160, 2, 6620}, {47, 2152, 3, 6620}, {47, 2157, 18, 6620}, {47, 2161, 9, 6620}, {48, 2158, 8, 6620}, {48, 2162, 12, 6620}, {49, 2163, 5, 6620}, {50, 2165, 1, 6620}, {51, 2166, 2, 6620}, {51, 2168, 3, 6620}, {52, 2167, 1, 6620}, {52, 2169, 9, 6620}, {53, 2170, 4, 6620}, {53, 2171, 6, 6620}, {54, 2172, 5, 6620}, {55, 2173, 1, 6620}, {35, 2195, 1, 6621}, {36, 2196, 3, 6621}, {36, 2205, 2, 6621}, {37, 2197, 3, 6621}, {37, 2215, 2, 6621}, {38, 2198, 3, 6621}, {38, 2206, 6, 6621}, {38, 2225, 1, 6621}, {39, 2199, 6, 6621}, {39, 2207, 6, 6621}, {39, 2216, 6, 6621}, {39, 2235, 2, 6621}, {40, 2200, 3, 6621}, {40, 2217, 6, 6621}, {40, 2245, 1, 6621}, {41, 2201, 1, 6621}, {41, 2208, 6, 6621}, {41, 2226, 3, 6621}, {42, 2202, 3, 6621}, {42, 2209, 12, 6621}, {42, 2218, 6, 6621}, {42, 2227, 3, 6621}, {42, 2236, 6, 6621}, {43, 2203, 3, 6621}, {43, 2210, 6, 6621}, {43, 2219, 12, 6621}, {43, 2237, 6, 6621}, {43, 2246, 3, 6621}, {44, 2204, 1, 6621}, {44, 2220, 6, 6621}, {44, 2247, 3, 6621}, {45, 2211, 2, 6621}, {45, 2228, 3, 6621}, {46, 2212, 6, 6621}, {46, 2221, 2, 6621}, {46, 2229, 6, 6621}, {46, 2238, 6, 6621}, {47, 2213, 6, 6621}, {47, 2222, 6, 6621}, {47, 2230, 3, 6621}, {47, 2239, 12, 6621}, {47, 2248, 3, 6621}, {48, 2214, 2, 6621}, {48, 2223, 6, 6621}, {48, 2240, 6, 6621}, {48, 2249, 6, 6621}, {49, 2224, 2, 6621}, {49, 2250, 3, 6621}, {50, 2231, 1, 6621}, {51, 2232, 3, 6621}, {51, 2241, 2, 6621}, {52, 2233, 3, 6621}, {52, 2242, 6, 6621}, {52, 2251, 1, 6621}, {53, 2234, 1, 6621}, {53, 2243, 6, 6621}, {53, 2252, 3, 6621}, {54, 2244, 2, 6621}, {54, 2253, 3, 6621}, {55, 2254, 1, 6621}, {35, 2555, 1, 6622}, {36, 2556, 3, 6622}, {36, 2565, 2, 6622}, {37, 2557, 3, 6622}, {37, 2575, 2, 6622}, {38, 2558, 3, 6622}, {38, 2566, 6, 6622}, {38, 2585, 1, 6622}, {39, 2559, 6, 6622}, {39, 2567, 6, 6622}, {39, 2576, 6, 6622}, {39, 2595, 2, 6622}, {40, 2560, 3, 6622}, {40, 2577, 6, 6622}, {40, 2605, 1, 6622}, {41, 2561, 1, 6622}, {41, 2568, 6, 6622}, {41, 2586, 3, 6622}, {42, 2562, 3, 6622}, {42, 2569, 12, 6622}, {42, 2578, 6, 6622}, {42, 2587, 3, 6622}, {42, 2596, 6, 6622}, {43, 2563, 3, 6622}, {43, 2570, 6, 6622}, {43, 2579, 12, 6622}, {43, 2597, 6, 6622}, {43, 2606, 3, 6622}, {44, 2564, 1, 6622}, {44, 2580, 6, 6622}, {44, 2607, 3, 6622}, {45, 2571, 2, 6622}, {45, 2588, 3, 6622}, {46, 2572, 6, 6622}, {46, 2581, 2, 6622}, {46, 2589, 6, 6622}, {46, 2598, 6, 6622}, {47, 2573, 6, 6622}, {47, 2582, 6, 6622}, {47, 2590, 3, 6622}, {47, 2599, 12, 6622}, {47, 2608, 3, 6622}, {48, 2574, 2, 6622}, {48, 2583, 6, 6622}, {48, 2600, 6, 6622}, {48, 2609, 6, 6622}, {49, 2584, 2, 6622}, {49, 2610, 3, 6622}, {50, 2591, 1, 6622}, {51, 2592, 3, 6622}, {51, 2601, 2, 6622}, {52, 2593, 3, 6622}, {52, 2602, 6, 6622}, {52, 2611, 1, 6622}, {53, 2594, 1, 6622}, {53, 2603, 6, 6622}, {53, 2612, 3, 6622}, {54, 2604, 2, 6622}, {54, 2613, 3, 6622}, {55, 2614, 1, 6622}, {56, 2255, 1, 6623}, {57, 2256, 4, 6623}, {57, 2270, 2, 6623}, {58, 2257, 4, 6623}, {58, 2285, 2, 6623}, {59, 2258, 6, 6623}, {59, 2271, 8, 6623}, {59, 2300, 1, 6623}, {60, 2259, 12, 6623}, {60, 2272, 8, 6623}, {60, 2286, 8, 6623}, {60, 2315, 2, 6623}, {61, 2260, 6, 6623}, {61, 2287, 8, 6623}, {61, 2330, 1, 6623}, {62, 2261, 4, 6623}, {62, 2273, 12, 6623}, {62, 2301, 4, 6623}, {63, 2262, 12, 6623}, {63, 2274, 24, 6623}, {63, 2288, 12, 6623}, {63, 2302, 4, 6623}, {63, 2316, 8, 6623}, {64, 2263, 12, 6623}, {64, 2275, 12, 6623}, {64, 2289, 24, 6623}, {64, 2317, 8, 6623}, {64, 2331, 4, 6623}, {65, 2264, 4, 6623}, {65, 2290, 12, 6623}, {65, 2332, 4, 6623}, {66, 2265, 1, 6623}, {66, 2276, 8, 6623}, {66, 2303, 6, 6623}, {67, 2266, 4, 6623}, {67, 2277, 24, 6623}, {67, 2291, 8,

6623}, {67, 2304, 12, 6623}, {67, 2318, 12, 6623}, {68, 2267, 6, 6623}, {68, 2278, 24, 6623}, {68, 2292, 24, 6623}, {68, 2305, 6, 6623}, {68, 2319, 24, 6623}, {68, 2333, 6, 6623}, {69, 2268, 4, 6623}, {69, 2279, 8, 6623}, {69, 2293, 24, 6623}, {69, 2320, 12, 6623}, {69, 2334, 12, 6623}, {70, 2269, 1, 6623}, {70, 2294, 8, 6623}, {70, 2335, 6, 6623}, {71, 2280, 2, 6623}, {71, 2306, 4, 6623}, {72, 2281, 8, 6623}, {72, 2295, 2, 6623}, {72, 2307, 12, 6623}, {72, 2321, 8, 6623}, {73, 2282, 12, 6623}, {73, 2296, 8, 6623}, {73, 2308, 12, 6623}, {73, 2322, 24, 6623}, {73, 2336, 4, 6623}, {74, 2283, 8, 6623}, {74, 2297, 12, 6623}, {74, 2309, 4, 6623}, {74, 2323, 24, 6623}, {74, 2337, 12, 6623}, {75, 2284, 2, 6623}, {75, 2298, 8, 6623}, {75, 2324, 8, 6623}, {75, 2338, 12, 6623}, {76, 2299, 2, 6623}, {76, 2339, 4, 6623}, {77, 2310, 1, 6623}, {78, 2311, 4, 6623}, {78, 2325, 2, 6623}, {79, 2312, 6, 6623}, {79, 2326, 8, 6623}, {79, 2340, 1, 6623}, {80, 2313, 4, 6623}, {80, 2327, 12, 6623}, {80, 2341, 4, 6623}, {81, 2314, 1, 6623}, {81, 2328, 8, 6623}, {81, 2342, 6, 6623}, {82, 2329, 2, 6623}, {82, 2343, 4, 6623}, {83, 2344, 1, 6623}, {56, 2119, 1, 6624}, {57, 2120, 6, 6624}, {58, 2121, 6, 6624}, {59, 2122, 6, 6624}, {59, 2129, 9, 6624}, {60, 2123, 12, 6624}, {60, 2130, 18, 6624}, {61, 2124, 6, 6624}, {61, 2138, 9, 6624}, {62, 2125, 2, 6624}, {62, 2131, 18, 6624}, {63, 2126, 6, 6624}, {63, 2132, 36, 6624}, {63, 2139, 18, 6624}, {64, 2127, 6, 6624}, {64, 2133, 18, 6624}, {64, 2140, 36, 6624}, {65, 2128, 2, 6624}, {65, 2141, 18, 6624}, {66, 2134, 6, 6624}, {66, 2146, 9, 6624}, {67, 2135, 18, 6624}, {67, 2142, 6, 6624}, {67, 2147, 36, 6624}, {68, 2136, 18, 6624}, {68, 2143, 18, 6624}, {68, 2148, 18, 6624}, {68, 2153, 36, 6624}, {69, 2137, 6, 6624}, {69, 2144, 18, 6624}, {69, 2154, 36, 6624}, {70, 2145, 6, 6624}, {70, 2159, 9, 6624}, {71, 2149, 6, 6624}, {72, 2150, 18, 6624}, {72, 2155, 12, 6624}, {73, 2151, 18, 6624}, {73, 2156, 36, 6624}, {73, 2160, 6, 6624}, {74, 2152, 6, 6624}, {74, 2157, 36, 6624}, {74, 2161, 18, 6624}, {75, 2158, 12, 6624}, {75, 2162, 18, 6624}, {76, 2163, 6, 6624}, {77, 2164, 1, 6624}, {78, 2165, 6, 6624}, {79, 2166, 6, 6624}, {79, 2168, 9, 6624}, {80, 2167, 2, 6624}, {80, 2169, 18, 6624}, {81, 2170, 6, 6624}, {81, 2171, 9, 6624}, {82, 2172, 6, 6624}, {83, 2173, 1, 6624}, {56, 2479, 1, 6625}, {57, 2480, 6, 6625}, {58, 2481, 6, 6625}, {59, 2482, 6, 6625}, {59, 2489, 9, 6625}, {60, 2483, 12, 6625}, {60, 2490, 18, 6625}, {61, 2484, 6, 6625}, {61, 2498, 9, 6625}, {62, 2485, 2, 6625}, {62, 2491, 18, 6625}, {63, 2486, 6, 6625}, {63, 2492, 36, 6625}, {63, 2499, 18, 6625}, {64, 2487, 6, 6625}, {64, 2493, 18, 6625}, {64, 2500, 36, 6625}, {65, 2488, 2, 6625}, {65, 2501, 18, 6625}, {66, 2494, 6, 6625}, {66, 2506, 9, 6625}, {67, 2495, 18, 6625}, {67, 2502, 6, 6625}, {67, 2507, 36, 6625}, {68, 2496, 18, 6625}, {68, 2503, 18, 6625}, {68, 2508, 18, 6625}, {68, 2513, 36, 6625}, {69, 2497, 6, 6625}, {69, 2504, 18, 6625}, {69, 2514, 36, 6625}, {70, 2505, 6, 6625}, {70, 2519, 9, 6625}, {71, 2509, 6, 6625}, {72, 2510, 18, 6625}, {72, 2515, 12, 6625}, {73, 2511, 18, 6625}, {73, 2516, 36, 6625}, {73, 2520, 6, 6625}, {74, 2512, 6, 6625}, {74, 2517, 36, 6625}, {74, 2521, 18, 6625}, {75, 2518, 12, 6625}, {75, 2522, 18, 6625}, {76, 2523, 6, 6625}, {77, 2524, 1, 6625}, {78, 2525, 6, 6625}, {79, 2526, 6, 6625}, {79, 2528, 9, 6625}, {80, 2527, 2, 6625}, {80, 2529, 18, 6625}, {81, 2530, 6, 6625}, {81, 2531, 9, 6625}, {82, 2532, 6, 6625}, {83, 2533, 1, 6625}, {0, 2755, 1, 6626}, {0, 2756, 1, 6627}, {0, 2826, 1, 6628}, {0, 3026, 1, 6629}, {0, 3241, 1, 6630}, {0, 3556, 1, 6631}, {0, 3826, 1, 6632}, {0, 3927, 1, 6633}, {0, 3965, 1, 6634}, {1, 2757, 1, 6635}, {2, 2758, 1, 6635}, {3, 2759, 1, 6635}, {1, 2760, 1, 6636}, {2, 2761, 1, 6636}, {3, 2762, 1, 6636}, {1, 2761, 1, 6637}, {2, 2763, 1, 6637}, {3, 2764, 1, 6637}, {1, 2762, 1, 6638}, {2, 2764, 1, 6638}, {3, 2765, 1, 6638}, {1, 2827, 1, 6639}, {2, 2828, 1, 6639}, {3, 2829, 1, 6639}, {1, 2830, 1, 6640}, {2, 2831, 1, 6640}, {3, 2832, 1, 6640}, {1, 2831, 1, 6641}, {2, 2833, 1, 6641}, {3, 2834, 1, 6641}, {1, 2832, 1, 6642}, {2, 2834, 1, 6642}, {3, 2835, 1, 6642}, {1, 3027, 1, 6643}, {2, 3028, 1, 6643}, {3, 3029, 1, 6643}, {1, 3030, 1, 6644}, {2, 3031, 1, 6644}, {3, 3032, 1, 6644}, {1, 3031, 1, 6645}, {2, 3033, 1, 6645}, {3, 3034, 1, 6645}, {1, 3032, 1, 6646}, {2, 3034, 1, 6646}, {3, 3035, 1, 6646}, {1, 3242, 1, 6647}, {2, 3243, 1, 6647}, {3, 3244, 1, 6647}, {1, 3245, 1, 6648}, {2, 3246, 1, 6648}, {3, 3247, 1, 6648}, {1, 3246, 1, 6649}, {2, 3248, 1, 6649}, {3, 3249, 1, 6649}, {1, 3247, 1, 6650}, {2, 3249, 1, 6650}, {3, 3250, 1, 6650}, {1, 3557, 1, 6651}, {2, 3558, 1, 6651}, {3, 3559, 1, 6651}, {1, 3560, 1, 6652}, {2, 3561, 1, 6652}, {3, 3562, 1, 6652}, {1, 3561, 1, 6653}, {2, 3563, 1, 6653}, {3, 3564, 1, 6653}, {1, 3562, 1, 6654}, {2, 3564, 1, 6654}, {3, 3565, 1, 6654}, {1, 3827, 1, 6655}, {2, 3828, 1, 6655}, {3, 3829, 1, 6655}, {1, 3928, 1, 6656}, {2, 3929, 1, 6656}, {3, 3930, 1, 6656}, {286, 707, 1, 6657}, {1, 2766, 1, 6658}, {2, 2767, 1, 6658}, {3, 2768, 1, 6658}, {1, 2769, 1, 6659}, {2, 2772, 1, 6659}, {3, 2775, 1, 6659}, {1, 2770, 1, 6660}, {2, 2773, 1, 6660}, {3, 2776, 1, 6660}, {1, 2771, 1, 6661}, {2, 2774, 1, 6661}, {3, 2777, 1, 6661}, {1, 2861, 1, 6662}, {2, 2862, 1, 6662}, {3, 2863, 1, 6662}, {1, 2862, 1, 6663}, {2, 2864, 1, 6663}, {3, 2865, 1, 6663}, {1, 2863, 1, 6664}, {2, 2865, 1, 6664}, {3, 2866, 1, 6664}, {1, 2921, 1, 6665}, {2, 2922, 1, 6665}, {3, 2923, 1, 6665}, {1, 2924, 1, 6666}, {2, 2927, 1, 6666}, {3, 2930, 1, 6666}, {1, 2925, 1, 6667}, {2, 2928, 1, 6667}, {3, 2931, 1, 6667}, {1, 2926, 1, 6668}, {2, 2929, 1, 6668}, {3, 2932, 1, 6668}, {1, 3061, 1, 6669}, {2, 3062, 1, 6669}, {3, 3063, 1, 6669}, {1, 3062, 1, 6670}, {2, 3064, 1, 6670}, {3, 3065, 1, 6670}, {1, 3063, 1, 6671}, {2, 3065, 1, 6671}, {3, 3066, 1, 6671}, {1, 3181, 1, 6672}, {2, 3182, 1, 6672}, {3, 3183, 1, 6672}, {1, 3184, 1, 6673}, {2, 3187, 1, 6673}, {3, 3190, 1, 6673}, {1, 3185, 1, 6674}, {2, 3188, 1, 6674}, {3, 3191, 1, 6674}, {1, 3186, 1, 6675}, {2, 3189, 1, 6675}, {3, 3192, 1, 6675}, {1, 3376,

1, 6676}, {2, 3377, 1, 6676}, {3, 3378, 1, 6676}, {1, 3377, 1, 6677}, {2, 3379, 1, 6677},
{3, 3380, 1, 6677}, {1, 3378, 1, 6678}, {2, 3380, 1, 6678}, {3, 3381, 1, 6678}, {1, 3496,
1, 6679}, {2, 3497, 1, 6679}, {3, 3498, 1, 6679}, {1, 3499, 1, 6680}, {2, 3502, 1, 6680},
{3, 3505, 1, 6680}, {1, 3500, 1, 6681}, {2, 3503, 1, 6681}, {3, 3506, 1, 6681}, {1, 3501,
1, 6682}, {2, 3504, 1, 6682}, {3, 3507, 1, 6682}, {1, 3736, 1, 6683}, {2, 3737, 1, 6683},
{3, 3738, 1, 6683}, {1, 3737, 1, 6684}, {2, 3739, 1, 6684}, {3, 3740, 1, 6684}, {1, 3738,
1, 6685}, {2, 3740, 1, 6685}, {3, 3741, 1, 6685}, {1, 3796, 1, 6686}, {2, 3797, 1, 6686},
{3, 3798, 1, 6686}, {1, 3799, 1, 6687}, {2, 3802, 1, 6687}, {3, 3805, 1, 6687}, {1, 3800,
1, 6688}, {2, 3803, 1, 6688}, {3, 3806, 1, 6688}, {1, 3801, 1, 6689}, {2, 3804, 1, 6689},
{3, 3807, 1, 6689}, {1, 3891, 1, 6690}, {2, 3892, 1, 6690}, {3, 3893, 1, 6690}, {1, 3892,
1, 6691}, {2, 3894, 1, 6691}, {3, 3895, 1, 6691}, {1, 3893, 1, 6692}, {2, 3895, 1, 6692},
{3, 3896, 1, 6692}, {1, 3915, 1, 6693}, {2, 3916, 1, 6693}, {3, 3917, 1, 6693}, {1, 3962,
1, 6694}, {2, 3963, 1, 6694}, {3, 3964, 1, 6694}, {4, 2827, 1, 6695}, {5, 2828, 1, 6695},
{6, 2829, 1, 6695}, {5, 2827, 1, 6696}, {7, 2828, 1, 6696}, {8, 2829, 1, 6696}, {6, 2827,
1, 6697}, {8, 2828, 1, 6697}, {9, 2829, 1, 6697}, {4, 3027, 1, 6698}, {5, 3028, 1, 6698},
{6, 3029, 1, 6698}, {5, 3027, 1, 6699}, {7, 3028, 1, 6699}, {8, 3029, 1, 6699}, {6, 3027,
1, 6700}, {8, 3028, 1, 6700}, {9, 3029, 1, 6700}, {4, 3030, 1, 6701}, {5, 3031, 1, 6701},
{6, 3032, 1, 6701}, {4, 3031, 1, 6702}, {5, 3033, 1, 6702}, {6, 3034, 1, 6702}, {4, 3032,
1, 6703}, {5, 3034, 1, 6703}, {6, 3035, 1, 6703}, {5, 3030, 1, 6704}, {7, 3031, 1, 6704},
{8, 3032, 1, 6704}, {5, 3031, 1, 6705}, {7, 3033, 1, 6705}, {8, 3034, 1, 6705}, {5, 3032,
1, 6706}, {7, 3034, 1, 6706}, {8, 3035, 1, 6706}, {6, 3030, 1, 6707}, {8, 3031, 1, 6707},
{9, 3032, 1, 6707}, {6, 3031, 1, 6708}, {8, 3033, 1, 6708}, {9, 3034, 1, 6708}, {6, 3032,
1, 6709}, {8, 3034, 1, 6709}, {9, 3035, 1, 6709}, {4, 3242, 1, 6710}, {5, 3243, 1, 6710},
{6, 3244, 1, 6710}, {5, 3242, 1, 6711}, {7, 3243, 1, 6711}, {8, 3244, 1, 6711}, {6, 3242,
1, 6712}, {8, 3243, 1, 6712}, {9, 3244, 1, 6712}, {4, 3245, 1, 6713}, {5, 3246, 1, 6713},
{6, 3247, 1, 6713}, {4, 3246, 1, 6714}, {5, 3248, 1, 6714}, {6, 3249, 1, 6714}, {4, 3247,
1, 6715}, {5, 3249, 1, 6715}, {6, 3250, 1, 6715}, {5, 3245, 1, 6716}, {7, 3246, 1, 6716},
{8, 3247, 1, 6716}, {5, 3246, 1, 6717}, {7, 3248, 1, 6717}, {8, 3249, 1, 6717}, {5, 3247,
1, 6718}, {7, 3249, 1, 6718}, {8, 3250, 1, 6718}, {6, 3245, 1, 6719}, {8, 3246, 1, 6719},
{9, 3247, 1, 6719}, {6, 3246, 1, 6720}, {8, 3248, 1, 6720}, {9, 3249, 1, 6720}, {6, 3247,
1, 6721}, {8, 3249, 1, 6721}, {9, 3250, 1, 6721}, {4, 3557, 1, 6722}, {5, 3558, 1, 6722},
{6, 3559, 1, 6722}, {5, 3557, 1, 6723}, {7, 3558, 1, 6723}, {8, 3559, 1, 6723}, {6, 3557,
1, 6724}, {8, 3558, 1, 6724}, {9, 3559, 1, 6724}, {4, 2830, 1, 6725}, {5, 2831, 2, 6725},
{6, 2832, 2, 6725}, {7, 2833, 1, 6725}, {8, 2834, 2, 6725}, {9, 2835, 1, 6725}, {4, 2836,
1, 6726}, {5, 2837, 2, 6726}, {6, 2838, 2, 6726}, {7, 2839, 1, 6726}, {8, 2840, 2, 6726},
{9, 2841, 1, 6726}, {4, 2837, 1, 6727}, {5, 2839, 2, 6727}, {6, 2840, 2, 6727}, {7, 2842,
1, 6727}, {8, 2843, 2, 6727}, {9, 2844, 1, 6727}, {4, 2838, 1, 6728}, {5, 2840, 2, 6728},
{6, 2841, 2, 6728}, {7, 2843, 1, 6728}, {8, 2844, 2, 6728}, {9, 2845, 1, 6728}, {4, 2846,
1, 6729}, {5, 2847, 2, 6729}, {6, 2848, 2, 6729}, {7, 2849, 1, 6729}, {8, 2850, 2, 6729},
{9, 2851, 1, 6729}, {4, 2847, 1, 6730}, {5, 2849, 2, 6730}, {6, 2850, 2, 6730}, {7, 2852,
1, 6730}, {8, 2853, 2, 6730}, {9, 2854, 1, 6730}, {4, 2848, 1, 6731}, {5, 2850, 2, 6731},
{6, 2851, 2, 6731}, {7, 2853, 1, 6731}, {8, 2854, 2, 6731}, {9, 2855, 1, 6731}, {4, 2849,
1, 6732}, {5, 2852, 2, 6732}, {6, 2853, 2, 6732}, {7, 2856, 1, 6732}, {8, 2857, 2, 6732},
{9, 2858, 1, 6732}, {4, 2850, 1, 6733}, {5, 2853, 2, 6733}, {6, 2854, 2, 6733}, {7, 2857,
1, 6733}, {8, 2858, 2, 6733}, {9, 2859, 1, 6733}, {4, 2851, 1, 6734}, {5, 2854, 2, 6734},
{6, 2855, 2, 6734}, {7, 2858, 1, 6734}, {8, 2859, 2, 6734}, {9, 2860, 1, 6734}, {4, 2933,
1, 6735}, {5, 2934, 2, 6735}, {6, 2935, 2, 6735}, {7, 2936, 1, 6735}, {8, 2937, 2, 6735},
{9, 2938, 1, 6735}, {4, 2939, 1, 6736}, {5, 2940, 2, 6736}, {6, 2941, 2, 6736}, {7, 2942,
1, 6736}, {8, 2943, 2, 6736}, {9, 2944, 1, 6736}, {4, 2945, 1, 6737}, {5, 2946, 2, 6737},
{6, 2947, 2, 6737}, {7, 2948, 1, 6737}, {8, 2949, 2, 6737}, {9, 2950, 1, 6737}, {4, 3030,
1, 6738}, {5, 3031, 2, 6738}, {6, 3032, 2, 6738}, {7, 3033, 1, 6738}, {8, 3034, 2, 6738},
{9, 3035, 1, 6738}, {4, 3036, 1, 6739}, {5, 3037, 2, 6739}, {6, 3038, 2, 6739}, {7, 3039,
1, 6739}, {8, 3040, 2, 6739}, {9, 3041, 1, 6739}, {4, 3037, 1, 6740}, {5, 3039, 2, 6740},
{6, 3040, 2, 6740}, {7, 3042, 1, 6740}, {8, 3043, 2, 6740}, {9, 3044, 1, 6740}, {4, 3038,
1, 6741}, {5, 3040, 2, 6741}, {6, 3041, 2, 6741}, {7, 3043, 1, 6741}, {8, 3044, 2, 6741},
{9, 3045, 1, 6741}, {4, 3046, 1, 6742}, {5, 3047, 2, 6742}, {6, 3048, 2, 6742}, {7, 3049,
1, 6742}, {8, 3050, 2, 6742}, {9, 3051, 1, 6742}, {4, 3047, 1, 6743}, {5, 3049, 2, 6743},
{6, 3050, 2, 6743}, {7, 3052, 1, 6743}, {8, 3053, 2, 6743}, {9, 3054, 1, 6743}, {4, 3048,
1, 6744}, {5, 3050, 2, 6744}, {6, 3051, 2, 6744}, {7, 3053, 1, 6744}, {8, 3054, 2, 6744},
{9, 3055, 1, 6744}, {4, 3049, 1, 6745}, {5, 3052, 2, 6745}, {6, 3053, 2, 6745}, {7, 3056,
1, 6745}, {8, 3057, 2, 6745}, {9, 3058, 1, 6745}, {4, 3050, 1, 6746}, {5, 3053, 2, 6746},
{6, 3054, 2, 6746}, {7, 3057, 1, 6746}, {8, 3058, 2, 6746}, {9, 3059, 1, 6746}, {4, 3051,
1, 6747}, {5, 3054, 2, 6747}, {6, 3055, 2, 6747}, {7, 3058, 1, 6747}, {8, 3059, 2, 6747},
{9, 3060, 1, 6747}, {4, 3193, 1, 6748}, {5, 3194, 2, 6748}, {6, 3195, 2, 6748}, {7, 3196,
1, 6748}, {8, 3197, 2, 6748}, {9, 3198, 1, 6748}, {4, 3199, 1, 6749}, {5, 3200, 2, 6749},
{6, 3201, 2, 6749}, {7, 3202, 1, 6749}, {8, 3203, 2, 6749}, {9, 3204, 1, 6749}, {4, 3205,
1, 6750}, {5, 3206, 2, 6750}, {6, 3207, 2, 6750}, {7, 3208, 1, 6750}, {8, 3209, 2, 6750},
{9, 3210, 1, 6750}, {4, 3245, 1, 6751}, {5, 3246, 2, 6751}, {6, 3247, 2, 6751}, {7, 3248,
1, 6751}, {8, 3249, 2, 6751}, {9, 3250, 1, 6751}, {4, 3251, 1, 6752}, {5, 3252, 2, 6752},

{6, 3253, 2, 6752}, {7, 3254, 1, 6752}, {8, 3255, 2, 6752}, {9, 3256, 1, 6752}, {4, 3252, 1, 6753}, {5, 3254, 2, 6753}, {6, 3255, 2, 6753}, {7, 3257, 1, 6753}, {8, 3258, 2, 6753}, {9, 3259, 1, 6753}, {4, 3253, 1, 6754}, {5, 3255, 2, 6754}, {6, 3256, 2, 6754}, {7, 3258, 1, 6754}, {8, 3259, 2, 6754}, {9, 3260, 1, 6754}, {4, 3508, 1, 6755}, {5, 3509, 2, 6755}, {6, 3510, 2, 6755}, {7, 3511, 1, 6755}, {8, 3512, 2, 6755}, {9, 3513, 1, 6755}, {4, 3514, 1, 6756}, {5, 3515, 2, 6756}, {6, 3516, 2, 6756}, {7, 3517, 1, 6756}, {8, 3518, 2, 6756}, {9, 3519, 1, 6756}, {4, 3520, 1, 6757}, {5, 3521, 2, 6757}, {6, 3522, 2, 6757}, {7, 3523, 1, 6757}, {8, 3524, 2, 6757}, {9, 3525, 1, 6757}, {4, 3560, 1, 6758}, {5, 3561, 2, 6758}, {6, 3562, 2, 6758}, {7, 3563, 1, 6758}, {8, 3564, 2, 6758}, {9, 3565, 1, 6758}, {4, 3830, 1, 6759}, {5, 3831, 2, 6759}, {6, 3832, 2, 6759}, {7, 3833, 1, 6759}, {8, 3834, 2, 6759}, {9, 3835, 1, 6759}, {4, 2766, 1, 6760}, {5, 2767, 1, 6760}, {6, 2768, 1, 6760}, {5, 2766, 1, 6761}, {7, 2767, 1, 6761}, {8, 2768, 1, 6761}, {6, 2766, 1, 6762}, {8, 2767, 1, 6762}, {9, 2768, 1, 6762}, {4, 2769, 1, 6763}, {5, 2772, 1, 6763}, {6, 2775, 1, 6763}, {4, 2770, 1, 6764}, {5, 2773, 1, 6764}, {6, 2776, 1, 6764}, {4, 2771, 1, 6765}, {5, 2774, 1, 6765}, {6, 2777, 1, 6765}, {5, 2769, 1, 6766}, {7, 2772, 1, 6766}, {8, 2775, 1, 6766}, {5, 2770, 1, 6767}, {7, 2773, 1, 6767}, {8, 2776, 1, 6767}, {5, 2771, 1, 6768}, {7, 2774, 1, 6768}, {8, 2777, 1, 6768}, {6, 2769, 1, 6769}, {8, 2772, 1, 6769}, {9, 2775, 1, 6769}, {6, 2770, 1, 6770}, {8, 2773, 1, 6770}, {9, 2776, 1, 6770}, {6, 2771, 1, 6771}, {8, 2774, 1, 6771}, {9, 2777, 1, 6771}, {4, 2921, 1, 6772}, {5, 2922, 1, 6772}, {6, 2923, 1, 6772}, {5, 2921, 1, 6773}, {7, 2922, 1, 6773}, {8, 2923, 1, 6773}, {6, 2921, 1, 6774}, {8, 2922, 1, 6774}, {9, 2923, 1, 6774}, {4, 2924, 1, 6775}, {5, 2927, 1, 6775}, {6, 2930, 1, 6775}, {4, 2925, 1, 6776}, {5, 2928, 1, 6776}, {6, 2931, 1, 6776}, {4, 2926, 1, 6777}, {5, 2929, 1, 6777}, {6, 2932, 1, 6777}, {5, 2924, 1, 6778}, {7, 2927, 1, 6778}, {8, 2930, 1, 6778}, {5, 2925, 1, 6779}, {7, 2928, 1, 6779}, {8, 2931, 1, 6779}, {5, 2926, 1, 6780}, {7, 2929, 1, 6780}, {8, 2932, 1, 6780}, {6, 2924, 1, 6781}, {8, 2927, 1, 6781}, {9, 2930, 1, 6781}, {6, 2925, 1, 6782}, {8, 2928, 1, 6782}, {9, 2931, 1, 6782}, {6, 2926, 1, 6783}, {8, 2929, 1, 6783}, {9, 2932, 1, 6783}, {4, 3061, 1, 6784}, {5, 3062, 1, 6784}, {6, 3063, 1, 6784}, {4, 3062, 1, 6785}, {5, 3064, 1, 6785}, {6, 3065, 1, 6785}, {4, 3063, 1, 6786}, {5, 3065, 1, 6786}, {6, 3066, 1, 6786}, {5, 3061, 1, 6787}, {7, 3062, 1, 6787}, {8, 3063, 1, 6787}, {5, 3062, 1, 6788}, {7, 3064, 1, 6788}, {8, 3065, 1, 6788}, {5, 3063, 1, 6789}, {7, 3065, 1, 6789}, {8, 3066, 1, 6789}, {6, 3061, 1, 6790}, {8, 3062, 1, 6790}, {9, 3063, 1, 6790}, {6, 3062, 1, 6791}, {8, 3064, 1, 6791}, {9, 3065, 1, 6791}, {6, 3063, 1, 6792}, {8, 3065, 1, 6792}, {9, 3066, 1, 6792}, {4, 3181, 1, 6793}, {5, 3182, 1, 6793}, {6, 3183, 1, 6793}, {5, 3181, 1, 6794}, {7, 3182, 1, 6794}, {8, 3183, 1, 6794}, {6, 3181, 1, 6795}, {8, 3182, 1, 6795}, {9, 3183, 1, 6795}, {4, 3184, 1, 6796}, {5, 3187, 1, 6796}, {6, 3190, 1, 6796}, {4, 3185, 1, 6797}, {5, 3188, 1, 6797}, {6, 3191, 1, 6797}, {4, 3186, 1, 6798}, {5, 3189, 1, 6798}, {6, 3192, 1, 6798}, {5, 3184, 1, 6799}, {7, 3187, 1, 6799}, {8, 3190, 1, 6799}, {5, 3185, 1, 6800}, {7, 3188, 1, 6800}, {8, 3191, 1, 6800}, {5, 3186, 1, 6801}, {7, 3189, 1, 6801}, {8, 3192, 1, 6801}, {6, 3184, 1, 6802}, {8, 3187, 1, 6802}, {9, 3190, 1, 6802}, {6, 3185, 1, 6803}, {8, 3188, 1, 6803}, {9, 3191, 1, 6803}, {6, 3186, 1, 6804}, {8, 3189, 1, 6804}, {9, 3192, 1, 6804}, {4, 3376, 1, 6805}, {5, 3377, 1, 6805}, {6, 3378, 1, 6805}, {4, 3377, 1, 6806}, {5, 3379, 1, 6806}, {6, 3380, 1, 6806}, {4, 3378, 1, 6807}, {5, 3380, 1, 6807}, {6, 3381, 1, 6807}, {5, 3376, 1, 6808}, {7, 3377, 1, 6808}, {8, 3378, 1, 6808}, {5, 3377, 1, 6809}, {7, 3379, 1, 6809}, {8, 3380, 1, 6809}, {5, 3378, 1, 6810}, {7, 3380, 1, 6810}, {8, 3381, 1, 6810}, {6, 3376, 1, 6811}, {8, 3377, 1, 6811}, {9, 3378, 1, 6811}, {6, 3377, 1, 6812}, {8, 3379, 1, 6812}, {9, 3380, 1, 6812}, {6, 3378, 1, 6813}, {8, 3380, 1, 6813}, {9, 3381, 1, 6813}, {4, 3496, 1, 6814}, {5, 3497, 1, 6814}, {6, 3498, 1, 6814}, {5, 3496, 1, 6815}, {7, 3497, 1, 6815}, {8, 3498, 1, 6815}, {6, 3496, 1, 6816}, {8, 3497, 1, 6816}, {9, 3498, 1, 6816}, {4, 3499, 1, 6817}, {5, 3502, 1, 6817}, {6, 3505, 1, 6817}, {4, 3500, 1, 6818}, {5, 3503, 1, 6818}, {6, 3506, 1, 6818}, {4, 3501, 1, 6819}, {5, 3504, 1, 6819}, {6, 3507, 1, 6819}, {5, 3499, 1, 6820}, {7, 3502, 1, 6820}, {8, 3505, 1, 6820}, {5, 3500, 1, 6821}, {7, 3503, 1, 6821}, {8, 3506, 1, 6821}, {5, 3501, 1, 6822}, {7, 3504, 1, 6822}, {8, 3507, 1, 6822}, {6, 3499, 1, 6823}, {8, 3502, 1, 6823}, {9, 3505, 1, 6823}, {6, 3500, 1, 6824}, {8, 3503, 1, 6824}, {9, 3506, 1, 6824}, {6, 3501, 1, 6825}, {8, 3504, 1, 6825}, {9, 3507, 1, 6825}, {4, 3736, 1, 6826}, {5, 3737, 1, 6826}, {6, 3738, 1, 6826}, {4, 3737, 1, 6827}, {5, 3739, 1, 6827}, {6, 3740, 1, 6827}, {4, 3738, 1, 6828}, {5, 3740, 1, 6828}, {6, 3741, 1, 6828}, {5, 3736, 1, 6829}, {7, 3737, 1, 6829}, {8, 3738, 1, 6829}, {5, 3737, 1, 6830}, {7, 3739, 1, 6830}, {8, 3740, 1, 6830}, {5, 3738, 1, 6831}, {7, 3740, 1, 6831}, {8, 3741, 1, 6831}, {6, 3736, 1, 6832}, {8, 3737, 1, 6832}, {9, 3738, 1, 6832}, {6, 3737, 1, 6833}, {8, 3739, 1, 6833}, {9, 3740, 1, 6833}, {6, 3738, 1, 6834}, {8, 3740, 1, 6834}, {9, 3741, 1, 6834}, {4, 3796, 1, 6835}, {5, 3797, 1, 6835}, {6, 3798, 1, 6835}, {5, 3796, 1, 6836}, {7, 3797, 1, 6836}, {8, 3798, 1, 6836}, {6, 3796, 1, 6837}, {8, 3797, 1, 6837}, {9, 3798, 1, 6837}, {4, 2769, 1, 6838}, {5, 2770, 1, 6838}, {5, 2772, 1, 6838}, {6, 2771, 1, 6838}, {6, 2775, 1, 6838}, {7, 2773, 1, 6838}, {8, 2774, 1, 6838}, {8, 2776, 1, 6838}, {9, 2777, 1, 6838}, {4, 2778, 1, 6839}, {5, 2779, 1, 6839}, {5, 2784, 1, 6839}, {6, 2780, 1, 6839}, {6, 2790, 1, 6839}, {7, 2785, 1, 6839}, {8, 2786, 1, 6839}, {8, 2791, 1, 6839}, {9, 2792, 1, 6839}, {4, 2779, 1, 6840}, {5, 2781, 1, 6840}, {5, 2785, 1, 6840}, {6, 2782, 1, 6840}, {6, 2791, 1, 6840}, {7, 2787, 1, 6840}, {8, 2788, 1, 6840}, {8, 2793, 1, 6840}, {9, 2794, 1, 6840}, {4, 2780, 1, 6841}, {5, 2782, 1, 6841}, {5, 2786, 1, 6841}, {6, 2783,

1, 6841}, {6, 2792, 1, 6841}, {7, 2788, 1, 6841}, {8, 2789, 1, 6841}, {8, 2794, 1, 6841},
{9, 2795, 1, 6841}, {4, 2796, 1, 6842}, {5, 2797, 1, 6842}, {5, 2806, 1, 6842}, {6, 2798,
1, 6842}, {6, 2816, 1, 6842}, {7, 2807, 1, 6842}, {8, 2808, 1, 6842}, {8, 2817, 1, 6842},
{9, 2818, 1, 6842}, {4, 2797, 1, 6843}, {5, 2799, 1, 6843}, {5, 2807, 1, 6843}, {6, 2800,
1, 6843}, {6, 2817, 1, 6843}, {7, 2809, 1, 6843}, {8, 2810, 1, 6843}, {8, 2819, 1, 6843},
{9, 2820, 1, 6843}, {4, 2798, 1, 6844}, {5, 2800, 1, 6844}, {5, 2808, 1, 6844}, {6, 2801,
1, 6844}, {6, 2818, 1, 6844}, {7, 2810, 1, 6844}, {8, 2811, 1, 6844}, {8, 2820, 1, 6844},
{9, 2821, 1, 6844}, {4, 2799, 1, 6845}, {5, 2802, 1, 6845}, {5, 2809, 1, 6845}, {6, 2803,
1, 6845}, {6, 2819, 1, 6845}, {7, 2812, 1, 6845}, {8, 2813, 1, 6845}, {8, 2822, 1, 6845},
{9, 2823, 1, 6845}, {4, 2800, 1, 6846}, {5, 2803, 1, 6846}, {5, 2810, 1, 6846}, {6, 2804,
1, 6846}, {6, 2820, 1, 6846}, {7, 2813, 1, 6846}, {8, 2814, 1, 6846}, {8, 2823, 1, 6846},
{9, 2824, 1, 6846}, {4, 2801, 1, 6847}, {5, 2804, 1, 6847}, {5, 2811, 1, 6847}, {6, 2805,
1, 6847}, {6, 2821, 1, 6847}, {7, 2814, 1, 6847}, {8, 2815, 1, 6847}, {8, 2824, 1, 6847},
{9, 2825, 1, 6847}, {4, 2867, 1, 6848}, {5, 2868, 1, 6848}, {5, 2870, 1, 6848}, {6, 2869,
1, 6848}, {6, 2873, 1, 6848}, {7, 2871, 1, 6848}, {8, 2872, 1, 6848}, {8, 2874, 1, 6848},
{9, 2875, 1, 6848}, {4, 2870, 1, 6849}, {5, 2871, 1, 6849}, {5, 2876, 1, 6849}, {6, 2872,
1, 6849}, {6, 2879, 1, 6849}, {7, 2877, 1, 6849}, {8, 2878, 1, 6849}, {8, 2880, 1, 6849},
{9, 2881, 1, 6849}, {4, 2873, 1, 6850}, {5, 2874, 1, 6850}, {5, 2879, 1, 6850}, {6, 2875,
1, 6850}, {6, 2882, 1, 6850}, {7, 2880, 1, 6850}, {8, 2881, 1, 6850}, {8, 2883, 1, 6850},
{9, 2884, 1, 6850}, {4, 2885, 1, 6851}, {5, 2886, 1, 6851}, {5, 2891, 1, 6851}, {6, 2887,
1, 6851}, {6, 2897, 1, 6851}, {7, 2892, 1, 6851}, {8, 2893, 1, 6851}, {8, 2898, 1, 6851},
{9, 2899, 1, 6851}, {4, 2886, 1, 6852}, {5, 2888, 1, 6852}, {5, 2892, 1, 6852}, {6, 2889,
1, 6852}, {6, 2898, 1, 6852}, {7, 2894, 1, 6852}, {8, 2895, 1, 6852}, {8, 2900, 1, 6852},
{9, 2901, 1, 6852}, {4, 2887, 1, 6853}, {5, 2889, 1, 6853}, {5, 2893, 1, 6853}, {6, 2890,
1, 6853}, {6, 2899, 1, 6853}, {7, 2895, 1, 6853}, {8, 2896, 1, 6853}, {8, 2901, 1, 6853},
{9, 2902, 1, 6853}, {4, 2891, 1, 6854}, {5, 2892, 1, 6854}, {5, 2903, 1, 6854}, {6, 2893,
1, 6854}, {6, 2909, 1, 6854}, {7, 2904, 1, 6854}, {8, 2905, 1, 6854}, {8, 2910, 1, 6854},
{9, 2911, 1, 6854}, {4, 2892, 1, 6855}, {5, 2894, 1, 6855}, {5, 2904, 1, 6855}, {6, 2895,
1, 6855}, {6, 2910, 1, 6855}, {7, 2906, 1, 6855}, {8, 2907, 1, 6855}, {8, 2912, 1, 6855},
{9, 2913, 1, 6855}, {4, 2893, 1, 6856}, {5, 2895, 1, 6856}, {5, 2905, 1, 6856}, {6, 2896,
1, 6856}, {6, 2911, 1, 6856}, {7, 2907, 1, 6856}, {8, 2908, 1, 6856}, {8, 2913, 1, 6856},
{9, 2914, 1, 6856}, {4, 2897, 1, 6857}, {5, 2898, 1, 6857}, {5, 2909, 1, 6857}, {6, 2899,
1, 6857}, {6, 2915, 1, 6857}, {7, 2910, 1, 6857}, {8, 2911, 1, 6857}, {8, 2916, 1, 6857},
{9, 2917, 1, 6857}, {4, 2898, 1, 6858}, {5, 2900, 1, 6858}, {5, 2910, 1, 6858}, {6, 2901,
1, 6858}, {6, 2916, 1, 6858}, {7, 2912, 1, 6858}, {8, 2913, 1, 6858}, {8, 2918, 1, 6858},
{9, 2919, 1, 6858}, {4, 2899, 1, 6859}, {5, 2901, 1, 6859}, {5, 2911, 1, 6859}, {6, 2902,
1, 6859}, {6, 2917, 1, 6859}, {7, 2913, 1, 6859}, {8, 2914, 1, 6859}, {8, 2919, 1, 6859},
{9, 2920, 1, 6859}, {4, 2924, 1, 6860}, {5, 2925, 1, 6860}, {5, 2927, 1, 6860}, {6, 2926,
1, 6860}, {6, 2930, 1, 6860}, {7, 2928, 1, 6860}, {8, 2929, 1, 6860}, {8, 2931, 1, 6860},
{9, 2932, 1, 6860}, {4, 2933, 1, 6861}, {5, 2934, 1, 6861}, {5, 2939, 1, 6861}, {6, 2935,
1, 6861}, {6, 2945, 1, 6861}, {7, 2940, 1, 6861}, {8, 2941, 1, 6861}, {8, 2946, 1, 6861},
{9, 2947, 1, 6861}, {4, 2934, 1, 6862}, {5, 2936, 1, 6862}, {5, 2940, 1, 6862}, {6, 2937,
1, 6862}, {6, 2946, 1, 6862}, {7, 2942, 1, 6862}, {8, 2943, 1, 6862}, {8, 2948, 1, 6862},
{9, 2949, 1, 6862}, {4, 2935, 1, 6863}, {5, 2937, 1, 6863}, {5, 2941, 1, 6863}, {6, 2938,
1, 6863}, {6, 2947, 1, 6863}, {7, 2943, 1, 6863}, {8, 2944, 1, 6863}, {8, 2949, 1, 6863},
{9, 2950, 1, 6863}, {4, 2951, 1, 6864}, {5, 2952, 1, 6864}, {5, 2961, 1, 6864}, {6, 2953,
1, 6864}, {6, 2971, 1, 6864}, {7, 2962, 1, 6864}, {8, 2963, 1, 6864}, {8, 2972, 1, 6864},
{9, 2973, 1, 6864}, {4, 2952, 1, 6865}, {5, 2954, 1, 6865}, {5, 2962, 1, 6865}, {6, 2955,
1, 6865}, {6, 2972, 1, 6865}, {7, 2964, 1, 6865}, {8, 2965, 1, 6865}, {8, 2974, 1, 6865},
{9, 2975, 1, 6865}, {4, 2953, 1, 6866}, {5, 2955, 1, 6866}, {5, 2963, 1, 6866}, {6, 2956,
1, 6866}, {6, 2973, 1, 6866}, {7, 2965, 1, 6866}, {8, 2966, 1, 6866}, {8, 2975, 1, 6866},
{9, 2976, 1, 6866}, {4, 2954, 1, 6867}, {5, 2957, 1, 6867}, {5, 2964, 1, 6867}, {6, 2958,
1, 6867}, {6, 2974, 1, 6867}, {7, 2967, 1, 6867}, {8, 2968, 1, 6867}, {8, 2977, 1, 6867},
{9, 2978, 1, 6867}, {4, 2955, 1, 6868}, {5, 2958, 1, 6868}, {5, 2965, 1, 6868}, {6, 2959,
1, 6868}, {6, 2975, 1, 6868}, {7, 2968, 1, 6868}, {8, 2969, 1, 6868}, {8, 2978, 1, 6868},
{9, 2979, 1, 6868}, {4, 2956, 1, 6869}, {5, 2959, 1, 6869}, {5, 2966, 1, 6869}, {6, 2960,
1, 6869}, {6, 2976, 1, 6869}, {7, 2969, 1, 6869}, {8, 2970, 1, 6869}, {8, 2979, 1, 6869},
{9, 2980, 1, 6869}, {4, 3067, 1, 6870}, {5, 3068, 1, 6870}, {5, 3070, 1, 6870}, {6, 3069,
1, 6870}, {6, 3073, 1, 6870}, {7, 3071, 1, 6870}, {8, 3072, 1, 6870}, {8, 3074, 1, 6870},
{9, 3075, 1, 6870}, {4, 3070, 1, 6871}, {5, 3071, 1, 6871}, {5, 3076, 1, 6871}, {6, 3072,
1, 6871}, {6, 3079, 1, 6871}, {7, 3077, 1, 6871}, {8, 3078, 1, 6871}, {8, 3080, 1, 6871},
{9, 3081, 1, 6871}, {4, 3073, 1, 6872}, {5, 3074, 1, 6872}, {5, 3079, 1, 6872}, {6, 3075,
1, 6872}, {6, 3082, 1, 6872}, {7, 3080, 1, 6872}, {8, 3081, 1, 6872}, {8, 3083, 1, 6872},
{9, 3084, 1, 6872}, {4, 3085, 1, 6873}, {5, 3086, 1, 6873}, {5, 3091, 1, 6873}, {6, 3087,
1, 6873}, {6, 3097, 1, 6873}, {7, 3092, 1, 6873}, {8, 3093, 1, 6873}, {8, 3098, 1, 6873},
{9, 3099, 1, 6873}, {4, 3086, 1, 6874}, {5, 3088, 1, 6874}, {5, 3092, 1, 6874}, {6, 3089,
1, 6874}, {6, 3098, 1, 6874}, {7, 3094, 1, 6874}, {8, 3095, 1, 6874}, {8, 3100, 1, 6874},
{9, 3101, 1, 6874}, {4, 3087, 1, 6875}, {5, 3089, 1, 6875}, {5, 3093, 1, 6875}, {6, 3090,
1, 6875}, {6, 3099, 1, 6875}, {7, 3095, 1, 6875}, {8, 3096, 1, 6875}, {8, 3101, 1, 6875},

{9, 3102, 1, 6875}, {4, 3091, 1, 6876}, {5, 3092, 1, 6876}, {5, 3103, 1, 6876}, {6, 3093, 1, 6876}, {6, 3109, 1, 6876}, {7, 3104, 1, 6876}, {8, 3105, 1, 6876}, {8, 3110, 1, 6876}, {9, 3111, 1, 6876}, {4, 3092, 1, 6877}, {5, 3094, 1, 6877}, {5, 3104, 1, 6877}, {6, 3095, 1, 6877}, {6, 3110, 1, 6877}, {7, 3106, 1, 6877}, {8, 3107, 1, 6877}, {8, 3112, 1, 6877}, {9, 3113, 1, 6877}, {4, 3093, 1, 6878}, {5, 3095, 1, 6878}, {5, 3105, 1, 6878}, {6, 3096, 1, 6878}, {6, 3111, 1, 6878}, {7, 3107, 1, 6878}, {8, 3108, 1, 6878}, {8, 3113, 1, 6878}, {9, 3114, 1, 6878}, {4, 3097, 1, 6879}, {5, 3098, 1, 6879}, {5, 3109, 1, 6879}, {6, 3099, 1, 6879}, {6, 3115, 1, 6879}, {7, 3110, 1, 6879}, {8, 3111, 1, 6879}, {8, 3116, 1, 6879}, {9, 3117, 1, 6879}, {4, 3098, 1, 6880}, {5, 3100, 1, 6880}, {5, 3110, 1, 6880}, {6, 3101, 1, 6880}, {6, 3116, 1, 6880}, {7, 3112, 1, 6880}, {8, 3113, 1, 6880}, {8, 3118, 1, 6880}, {9, 3119, 1, 6880}, {4, 3099, 1, 6881}, {5, 3101, 1, 6881}, {5, 3111, 1, 6881}, {6, 3102, 1, 6881}, {6, 3117, 1, 6881}, {7, 3113, 1, 6881}, {8, 3114, 1, 6881}, {8, 3119, 1, 6881}, {9, 3120, 1, 6881}, {4, 3184, 1, 6882}, {5, 3185, 1, 6882}, {5, 3187, 1, 6882}, {6, 3186, 1, 6882}, {6, 3190, 1, 6882}, {7, 3188, 1, 6882}, {8, 3189, 1, 6882}, {8, 3191, 1, 6882}, {9, 3192, 1, 6882}, {4, 3193, 1, 6883}, {5, 3194, 1, 6883}, {5, 3199, 1, 6883}, {6, 3195, 1, 6883}, {6, 3205, 1, 6883}, {7, 3200, 1, 6883}, {8, 3201, 1, 6883}, {8, 3206, 1, 6883}, {9, 3207, 1, 6883}, {4, 3194, 1, 6884}, {5, 3196, 1, 6884}, {5, 3200, 1, 6884}, {6, 3197, 1, 6884}, {6, 3206, 1, 6884}, {7, 3202, 1, 6884}, {8, 3203, 1, 6884}, {8, 3208, 1, 6884}, {9, 3209, 1, 6884}, {4, 3195, 1, 6885}, {5, 3197, 1, 6885}, {5, 3201, 1, 6885}, {6, 3198, 1, 6885}, {6, 3207, 1, 6885}, {7, 3203, 1, 6885}, {8, 3204, 1, 6885}, {8, 3209, 1, 6885}, {9, 3210, 1, 6885}, {4, 3211, 1, 6886}, {5, 3212, 1, 6886}, {5, 3221, 1, 6886}, {6, 3213, 1, 6886}, {6, 3231, 1, 6886}, {7, 3222, 1, 6886}, {8, 3223, 1, 6886}, {8, 3232, 1, 6886}, {9, 3233, 1, 6886}, {4, 3212, 1, 6887}, {5, 3214, 1, 6887}, {5, 3222, 1, 6887}, {6, 3215, 1, 6887}, {6, 3232, 1, 6887}, {7, 3224, 1, 6887}, {8, 3225, 1, 6887}, {8, 3234, 1, 6887}, {9, 3235, 1, 6887}, {4, 3213, 1, 6888}, {5, 3215, 1, 6888}, {5, 3223, 1, 6888}, {6, 3216, 1, 6888}, {6, 3233, 1, 6888}, {7, 3225, 1, 6888}, {8, 3226, 1, 6888}, {8, 3235, 1, 6888}, {9, 3236, 1, 6888}, {4, 3214, 1, 6889}, {5, 3217, 1, 6889}, {5, 3224, 1, 6889}, {6, 3218, 1, 6889}, {6, 3234, 1, 6889}, {7, 3227, 1, 6889}, {8, 3228, 1, 6889}, {8, 3237, 1, 6889}, {9, 3238, 1, 6889}, {4, 3215, 1, 6890}, {5, 3218, 1, 6890}, {5, 3225, 1, 6890}, {6, 3219, 1, 6890}, {6, 3235, 1, 6890}, {7, 3228, 1, 6890}, {8, 3229, 1, 6890}, {8, 3238, 1, 6890}, {9, 3239, 1, 6890}, {4, 3216, 1, 6891}, {5, 3219, 1, 6891}, {5, 3226, 1, 6891}, {6, 3220, 1, 6891}, {6, 3236, 1, 6891}, {7, 3229, 1, 6891}, {8, 3230, 1, 6891}, {8, 3239, 1, 6891}, {9, 3240, 1, 6891}, {4, 3382, 1, 6892}, {5, 3383, 1, 6892}, {5, 3385, 1, 6892}, {6, 3384, 1, 6892}, {6, 3388, 1, 6892}, {7, 3386, 1, 6892}, {8, 3387, 1, 6892}, {8, 3389, 1, 6892}, {9, 3390, 1, 6892}, {4, 3385, 1, 6893}, {5, 3386, 1, 6893}, {5, 3391, 1, 6893}, {6, 3387, 1, 6893}, {6, 3394, 1, 6893}, {7, 3392, 1, 6893}, {8, 3393, 1, 6893}, {8, 3395, 1, 6893}, {9, 3396, 1, 6893}, {4, 3388, 1, 6894}, {5, 3389, 1, 6894}, {5, 3394, 1, 6894}, {6, 3390, 1, 6894}, {6, 3397, 1, 6894}, {7, 3395, 1, 6894}, {8, 3396, 1, 6894}, {8, 3398, 1, 6894}, {9, 3399, 1, 6894}, {4, 3400, 1, 6895}, {5, 3401, 1, 6895}, {5, 3406, 1, 6895}, {6, 3402, 1, 6895}, {6, 3412, 1, 6895}, {7, 3407, 1, 6895}, {8, 3408, 1, 6895}, {8, 3413, 1, 6895}, {9, 3414, 1, 6895}, {4, 3401, 1, 6896}, {5, 3403, 1, 6896}, {5, 3407, 1, 6896}, {6, 3404, 1, 6896}, {6, 3413, 1, 6896}, {7, 3409, 1, 6896}, {8, 3410, 1, 6896}, {8, 3415, 1, 6896}, {9, 3416, 1, 6896}, {4, 3402, 1, 6897}, {5, 3404, 1, 6897}, {5, 3408, 1, 6897}, {6, 3405, 1, 6897}, {6, 3414, 1, 6897}, {7, 3410, 1, 6897}, {8, 3411, 1, 6897}, {8, 3416, 1, 6897}, {9, 3417, 1, 6897}, {4, 3406, 1, 6898}, {5, 3407, 1, 6898}, {5, 3418, 1, 6898}, {6, 3408, 1, 6898}, {6, 3424, 1, 6898}, {7, 3419, 1, 6898}, {8, 3420, 1, 6898}, {8, 3425, 1, 6898}, {9, 3426, 1, 6898}, {4, 3407, 1, 6899}, {5, 3409, 1, 6899}, {5, 3419, 1, 6899}, {6, 3410, 1, 6899}, {6, 3425, 1, 6899}, {7, 3421, 1, 6899}, {8, 3422, 1, 6899}, {8, 3427, 1, 6899}, {9, 3428, 1, 6899}, {4, 3408, 1, 6900}, {5, 3410, 1, 6900}, {5, 3420, 1, 6900}, {6, 3411, 1, 6900}, {6, 3426, 1, 6900}, {7, 3422, 1, 6900}, {8, 3423, 1, 6900}, {8, 3428, 1, 6900}, {9, 3429, 1, 6900}, {4, 3412, 1, 6901}, {5, 3413, 1, 6901}, {5, 3424, 1, 6901}, {6, 3414, 1, 6901}, {6, 3430, 1, 6901}, {7, 3425, 1, 6901}, {8, 3426, 1, 6901}, {8, 3431, 1, 6901}, {9, 3432, 1, 6901}, {4, 3413, 1, 6902}, {5, 3415, 1, 6902}, {5, 3425, 1, 6902}, {6, 3416, 1, 6902}, {6, 3431, 1, 6902}, {7, 3427, 1, 6902}, {8, 3428, 1, 6902}, {8, 3433, 1, 6902}, {9, 3434, 1, 6902}, {4, 3414, 1, 6903}, {5, 3416, 1, 6903}, {5, 3426, 1, 6903}, {6, 3417, 1, 6903}, {6, 3432, 1, 6903}, {7, 3428, 1, 6903}, {8, 3429, 1, 6903}, {8, 3434, 1, 6903}, {9, 3435, 1, 6903}, {4, 3499, 1, 6904}, {5, 3500, 1, 6904}, {5, 3502, 1, 6904}, {6, 3501, 1, 6904}, {6, 3505, 1, 6904}, {7, 3503, 1, 6904}, {8, 3504, 1, 6904}, {8, 3506, 1, 6904}, {9, 3507, 1, 6904}, {4, 3508, 1, 6905}, {5, 3509, 1, 6905}, {5, 3514, 1, 6905}, {6, 3510, 1, 6905}, {6, 3520, 1, 6905}, {7, 3515, 1, 6905}, {8, 3516, 1, 6905}, {8, 3521, 1, 6905}, {9, 3522, 1, 6905}, {4, 3509, 1, 6906}, {5, 3511, 1, 6906}, {5, 3515, 1, 6906}, {6, 3512, 1, 6906}, {6, 3521, 1, 6906}, {7, 3517, 1, 6906}, {8, 3518, 1, 6906}, {8, 3523, 1, 6906}, {9, 3524, 1, 6906}, {4, 3510, 1, 6907}, {5, 3512, 1, 6907}, {5, 3516, 1, 6907}, {6, 3513, 1, 6907}, {6, 3522, 1, 6907}, {7, 3518, 1, 6907}, {8, 3519, 1, 6907}, {8, 3524, 1, 6907}, {9, 3525, 1, 6907}, {4, 3742, 1, 6908}, {5, 3743, 1, 6908}, {5, 3745, 1, 6908}, {6, 3744, 1, 6908}, {6, 3748, 1, 6908}, {7, 3746, 1, 6908}, {8, 3747, 1, 6908}, {8, 3749, 1, 6908}, {9, 3750, 1, 6908}, {4, 3745, 1, 6909}, {5, 3746, 1, 6909}, {5, 3751, 1, 6909}, {6, 3747, 1, 6909}, {6, 3754, 1, 6909}, {7, 3752, 1, 6909}, {8, 3753, 1, 6909}, {8, 3755, 1, 6909}, {9, 3756, 1, 6909}, {4, 3748, 1, 6910}, {5, 3749, 1, 6910}, {5, 3754, 1, 6910}, {6, 3750,

1, 6910}, {6, 3757, 1, 6910}, {7, 3755, 1, 6910}, {8, 3756, 1, 6910}, {8, 3758, 1, 6910},
{9, 3759, 1, 6910}, {4, 3799, 1, 6911}, {5, 3800, 1, 6911}, {5, 3802, 1, 6911}, {6, 3801,
1, 6911}, {6, 3805, 1, 6911}, {7, 3803, 1, 6911}, {8, 3804, 1, 6911}, {8, 3806, 1, 6911},
{9, 3807, 1, 6911}, {4, 3918, 1, 6912}, {5, 3919, 1, 6912}, {5, 3921, 1, 6912}, {6, 3920,
1, 6912}, {6, 3924, 1, 6912}, {7, 3922, 1, 6912}, {8, 3923, 1, 6912}, {8, 3925, 1, 6912},
{9, 3926, 1, 6912}, {286, 771, 1, 6913}, {287, 772, 1, 6914}, {288, 773, 1, 6914}, {289,
774, 1, 6914}, {290, 772, 1, 6915}, {291, 773, 1, 6915}, {292, 774, 1, 6915}, {291, 772,
1, 6916}, {293, 773, 1, 6916}, {294, 774, 1, 6916}, {292, 772, 1, 6917}, {294, 773, 1,
6917}, {295, 774, 1, 6917}, {287, 775, 1, 6918}, {288, 776, 1, 6918}, {289, 777, 1, 6918},
{287, 776, 1, 6919}, {288, 778, 1, 6919}, {289, 779, 1, 6919}, {287, 777, 1, 6920}, {288,
779, 1, 6920}, {289, 780, 1, 6920}, {290, 775, 1, 6921}, {291, 776, 1, 6921}, {292, 777,
1, 6921}, {291, 775, 1, 6922}, {293, 776, 1, 6922}, {294, 777, 1, 6922}, {292, 775, 1,
6923}, {294, 776, 1, 6923}, {295, 777, 1, 6923}, {290, 776, 1, 6924}, {291, 778, 1, 6924},
{292, 779, 1, 6924}, {291, 776, 1, 6925}, {293, 778, 1, 6925}, {294, 779, 1, 6925}, {292,
776, 1, 6926}, {294, 778, 1, 6926}, {295, 779, 1, 6926}, {290, 777, 1, 6927}, {291, 779,
1, 6927}, {292, 780, 1, 6927}, {291, 777, 1, 6928}, {293, 779, 1, 6928}, {294, 780, 1,
6928}, {292, 777, 1, 6929}, {294, 779, 1, 6929}, {295, 780, 1, 6929}, {4, 3061, 1, 6930},
{5, 3062, 2, 6930}, {6, 3063, 2, 6930}, {7, 3064, 1, 6930}, {8, 3065, 2, 6930}, {9, 3066,
1, 6930}, {4, 3067, 1, 6931}, {5, 3070, 2, 6931}, {6, 3073, 2, 6931}, {7, 3076, 1, 6931},
{8, 3079, 2, 6931}, {9, 3082, 1, 6931}, {4, 3068, 1, 6932}, {5, 3071, 2, 6932}, {6, 3074,
2, 6932}, {7, 3077, 1, 6932}, {8, 3080, 2, 6932}, {9, 3083, 1, 6932}, {4, 3069, 1, 6933},
{5, 3072, 2, 6933}, {6, 3075, 2, 6933}, {7, 3078, 1, 6933}, {8, 3081, 2, 6933}, {9, 3084,
1, 6933}, {4, 3085, 1, 6934}, {5, 3091, 2, 6934}, {6, 3097, 2, 6934}, {7, 3103, 1, 6934},
{8, 3109, 2, 6934}, {9, 3115, 1, 6934}, {4, 3086, 1, 6935}, {5, 3092, 2, 6935}, {6, 3098,
2, 6935}, {7, 3104, 1, 6935}, {8, 3110, 2, 6935}, {9, 3116, 1, 6935}, {4, 3087, 1, 6936},
{5, 3093, 2, 6936}, {6, 3099, 2, 6936}, {7, 3105, 1, 6936}, {8, 3111, 2, 6936}, {9, 3117,
1, 6936}, {4, 3088, 1, 6937}, {5, 3094, 2, 6937}, {6, 3100, 2, 6937}, {7, 3106, 1, 6937},
{8, 3112, 2, 6937}, {9, 3118, 1, 6937}, {4, 3089, 1, 6938}, {5, 3095, 2, 6938}, {6, 3101,
2, 6938}, {7, 3107, 1, 6938}, {8, 3113, 2, 6938}, {9, 3119, 1, 6938}, {4, 3090, 1, 6939},
{5, 3096, 2, 6939}, {6, 3102, 2, 6939}, {7, 3108, 1, 6939}, {8, 3114, 2, 6939}, {9, 3120,
1, 6939}, {4, 3276, 1, 6940}, {5, 3277, 2, 6940}, {6, 3278, 2, 6940}, {7, 3279, 1, 6940},
{8, 3280, 2, 6940}, {9, 3281, 1, 6940}, {4, 3277, 1, 6941}, {5, 3279, 2, 6941}, {6, 3280,
2, 6941}, {7, 3282, 1, 6941}, {8, 3283, 2, 6941}, {9, 3284, 1, 6941}, {4, 3278, 1, 6942},
{5, 3280, 2, 6942}, {6, 3281, 2, 6942}, {7, 3283, 1, 6942}, {8, 3284, 2, 6942}, {9, 3285,
1, 6942}, {4, 3286, 1, 6943}, {5, 3289, 2, 6943}, {6, 3292, 2, 6943}, {7, 3295, 1, 6943},
{8, 3298, 2, 6943}, {9, 3301, 1, 6943}, {4, 3287, 1, 6944}, {5, 3290, 2, 6944}, {6, 3293,
2, 6944}, {7, 3296, 1, 6944}, {8, 3299, 2, 6944}, {9, 3302, 1, 6944}, {4, 3288, 1, 6945},
{5, 3291, 2, 6945}, {6, 3294, 2, 6945}, {7, 3297, 1, 6945}, {8, 3300, 2, 6945}, {9, 3303,
1, 6945}, {4, 3289, 1, 6946}, {5, 3295, 2, 6946}, {6, 3298, 2, 6946}, {7, 3304, 1, 6946},
{8, 3307, 2, 6946}, {9, 3310, 1, 6946}, {4, 3290, 1, 6947}, {5, 3296, 2, 6947}, {6, 3299,
2, 6947}, {7, 3305, 1, 6947}, {8, 3308, 2, 6947}, {9, 3311, 1, 6947}, {4, 3291, 1, 6948},
{5, 3297, 2, 6948}, {6, 3300, 2, 6948}, {7, 3306, 1, 6948}, {8, 3309, 2, 6948}, {9, 3312,
1, 6948}, {4, 3292, 1, 6949}, {5, 3298, 2, 6949}, {6, 3301, 2, 6949}, {7, 3307, 1, 6949},
{8, 3310, 2, 6949}, {9, 3313, 1, 6949}, {4, 3293, 1, 6950}, {5, 3299, 2, 6950}, {6, 3302,
2, 6950}, {7, 3308, 1, 6950}, {8, 3311, 2, 6950}, {9, 3314, 1, 6950}, {4, 3294, 1, 6951},
{5, 3300, 2, 6951}, {6, 3303, 2, 6951}, {7, 3309, 1, 6951}, {8, 3312, 2, 6951}, {9, 3315,
1, 6951}, {4, 3376, 1, 6952}, {5, 3377, 2, 6952}, {6, 3378, 2, 6952}, {7, 3379, 1, 6952},
{8, 3380, 2, 6952}, {9, 3381, 1, 6952}, {4, 3382, 1, 6953}, {5, 3385, 2, 6953}, {6, 3388,
2, 6953}, {7, 3391, 1, 6953}, {8, 3394, 2, 6953}, {9, 3397, 1, 6953}, {4, 3383, 1, 6954},
{5, 3386, 2, 6954}, {6, 3389, 2, 6954}, {7, 3392, 1, 6954}, {8, 3395, 2, 6954}, {9, 3398,
1, 6954}, {4, 3384, 1, 6955}, {5, 3387, 2, 6955}, {6, 3390, 2, 6955}, {7, 3393, 1, 6955},
{8, 3396, 2, 6955}, {9, 3399, 1, 6955}, {4, 3400, 1, 6956}, {5, 3406, 2, 6956}, {6, 3412,
2, 6956}, {7, 3418, 1, 6956}, {8, 3424, 2, 6956}, {9, 3430, 1, 6956}, {4, 3401, 1, 6957},
{5, 3407, 2, 6957}, {6, 3413, 2, 6957}, {7, 3419, 1, 6957}, {8, 3425, 2, 6957}, {9, 3431,
1, 6957}, {4, 3402, 1, 6958}, {5, 3408, 2, 6958}, {6, 3414, 2, 6958}, {7, 3420, 1, 6958},
{8, 3426, 2, 6958}, {9, 3432, 1, 6958}, {4, 3403, 1, 6959}, {5, 3409, 2, 6959}, {6, 3415,
2, 6959}, {7, 3421, 1, 6959}, {8, 3427, 2, 6959}, {9, 3433, 1, 6959}, {4, 3404, 1, 6960},
{5, 3410, 2, 6960}, {6, 3416, 2, 6960}, {7, 3422, 1, 6960}, {8, 3428, 2, 6960}, {9, 3434,
1, 6960}, {4, 3405, 1, 6961}, {5, 3411, 2, 6961}, {6, 3417, 2, 6961}, {7, 3423, 1, 6961},
{8, 3429, 2, 6961}, {9, 3435, 1, 6961}, {4, 3576, 1, 6962}, {5, 3577, 2, 6962}, {6, 3578,
2, 6962}, {7, 3579, 1, 6962}, {8, 3580, 2, 6962}, {9, 3581, 1, 6962}, {4, 3577, 1, 6963},
{5, 3579, 2, 6963}, {6, 3580, 2, 6963}, {7, 3582, 1, 6963}, {8, 3583, 2, 6963}, {9, 3584,
1, 6963}, {4, 3578, 1, 6964}, {5, 3580, 2, 6964}, {6, 3581, 2, 6964}, {7, 3583, 1, 6964},
{8, 3584, 2, 6964}, {9, 3585, 1, 6964}, {4, 3579, 1, 6965}, {5, 3582, 2, 6965}, {6, 3583,
2, 6965}, {7, 3586, 1, 6965}, {8, 3587, 2, 6965}, {9, 3588, 1, 6965}, {4, 3580, 1, 6966},
{5, 3583, 2, 6966}, {6, 3584, 2, 6966}, {7, 3587, 1, 6966}, {8, 3588, 2, 6966}, {9, 3589,
1, 6966}, {4, 3581, 1, 6967}, {5, 3584, 2, 6967}, {6, 3585, 2, 6967}, {7, 3588, 1, 6967},
{8, 3589, 2, 6967}, {9, 3590, 1, 6967}, {4, 3636, 1, 6968}, {5, 3637, 2, 6968}, {6, 3638,
2, 6968}, {7, 3639, 1, 6968}, {8, 3640, 2, 6968}, {9, 3641, 1, 6968}, {4, 3637, 1, 6969},

{5, 3639, 2, 6969}, {6, 3640, 2, 6969}, {7, 3642, 1, 6969}, {8, 3643, 2, 6969}, {9, 3644, 1, 6969}, {4, 3638, 1, 6970}, {5, 3640, 2, 6970}, {6, 3641, 2, 6970}, {7, 3643, 1, 6970}, {8, 3644, 2, 6970}, {9, 3645, 1, 6970}, {4, 3646, 1, 6971}, {5, 3649, 2, 6971}, {6, 3652, 2, 6971}, {7, 3655, 1, 6971}, {8, 3658, 2, 6971}, {9, 3661, 1, 6971}, {4, 3647, 1, 6972}, {5, 3650, 2, 6972}, {6, 3653, 2, 6972}, {7, 3656, 1, 6972}, {8, 3659, 2, 6972}, {9, 3662, 1, 6972}, {4, 3648, 1, 6973}, {5, 3651, 2, 6973}, {6, 3654, 2, 6973}, {7, 3657, 1, 6973}, {8, 3660, 2, 6973}, {9, 3663, 1, 6973}, {4, 3649, 1, 6974}, {5, 3655, 2, 6974}, {6, 3658, 2, 6974}, {7, 3664, 1, 6974}, {8, 3667, 2, 6974}, {9, 3670, 1, 6974}, {4, 3650, 1, 6975}, {5, 3656, 2, 6975}, {6, 3659, 2, 6975}, {7, 3665, 1, 6975}, {8, 3668, 2, 6975}, {9, 3671, 1, 6975}, {4, 3651, 1, 6976}, {5, 3657, 2, 6976}, {6, 3660, 2, 6976}, {7, 3666, 1, 6976}, {8, 3669, 2, 6976}, {9, 3672, 1, 6976}, {4, 3652, 1, 6977}, {5, 3658, 2, 6977}, {6, 3661, 2, 6977}, {7, 3667, 1, 6977}, {8, 3670, 2, 6977}, {9, 3673, 1, 6977}, {4, 3653, 1, 6978}, {5, 3659, 2, 6978}, {6, 3662, 2, 6978}, {7, 3668, 1, 6978}, {8, 3671, 2, 6978}, {9, 3674, 1, 6978}, {4, 3654, 1, 6979}, {5, 3660, 2, 6979}, {6, 3663, 2, 6979}, {7, 3669, 1, 6979}, {8, 3672, 2, 6979}, {9, 3675, 1, 6979}, {4, 3736, 1, 6980}, {5, 3737, 2, 6980}, {6, 3738, 2, 6980}, {7, 3739, 1, 6980}, {8, 3740, 2, 6980}, {9, 3741, 1, 6980}, {4, 3742, 1, 6981}, {5, 3745, 2, 6981}, {6, 3748, 2, 6981}, {7, 3751, 1, 6981}, {8, 3754, 2, 6981}, {9, 3757, 1, 6981}, {4, 3743, 1, 6982}, {5, 3746, 2, 6982}, {6, 3749, 2, 6982}, {7, 3752, 1, 6982}, {8, 3755, 2, 6982}, {9, 3758, 1, 6982}, {4, 3744, 1, 6983}, {5, 3747, 2, 6983}, {6, 3750, 2, 6983}, {7, 3753, 1, 6983}, {8, 3756, 2, 6983}, {9, 3759, 1, 6983}, {4, 3836, 1, 6984}, {5, 3837, 2, 6984}, {6, 3838, 2, 6984}, {7, 3839, 1, 6984}, {8, 3840, 2, 6984}, {9, 3841, 1, 6984}, {4, 3837, 1, 6985}, {5, 3839, 2, 6985}, {6, 3840, 2, 6985}, {7, 3842, 1, 6985}, {8, 3843, 2, 6985}, {9, 3844, 1, 6985}, {4, 3838, 1, 6986}, {5, 3840, 2, 6986}, {6, 3841, 2, 6986}, {7, 3843, 1, 6986}, {8, 3844, 2, 6986}, {9, 3845, 1, 6986}, {4, 3839, 1, 6987}, {5, 3842, 2, 6987}, {6, 3843, 2, 6987}, {7, 3846, 1, 6987}, {8, 3847, 2, 6987}, {9, 3848, 1, 6987}, {4, 3840, 1, 6988}, {5, 3843, 2, 6988}, {6, 3844, 2, 6988}, {7, 3847, 1, 6988}, {8, 3848, 2, 6988}, {9, 3849, 1, 6988}, {4, 3841, 1, 6989}, {5, 3844, 2, 6989}, {6, 3845, 2, 6989}, {7, 3848, 1, 6989}, {8, 3849, 2, 6989}, {9, 3850, 1, 6989}, {4, 3851, 1, 6990}, {5, 3852, 2, 6990}, {6, 3853, 2, 6990}, {7, 3854, 1, 6990}, {8, 3855, 2, 6990}, {9, 3856, 1, 6990}, {4, 3852, 1, 6991}, {5, 3854, 2, 6991}, {6, 3855, 2, 6991}, {7, 3857, 1, 6991}, {8, 3858, 2, 6991}, {9, 3859, 1, 6991}, {4, 3853, 1, 6992}, {5, 3855, 2, 6992}, {6, 3856, 2, 6992}, {7, 3858, 1, 6992}, {8, 3859, 2, 6992}, {9, 3860, 1, 6992}, {4, 3891, 1, 6993}, {5, 3892, 2, 6993}, {6, 3893, 2, 6993}, {7, 3894, 1, 6993}, {8, 3895, 2, 6993}, {9, 3896, 1, 6993}, {4, 3956, 1, 6994}, {5, 3957, 2, 6994}, {6, 3958, 2, 6994}, {7, 3959, 1, 6994}, {8, 3960, 2, 6994}, {9, 3961, 1, 6994}, {10, 2933, 1, 6995}, {11, 2934, 2, 6995}, {12, 2935, 2, 6995}, {13, 2936, 1, 6995}, {14, 2937, 2, 6995}, {15, 2938, 1, 6995}, {10, 2939, 1, 6996}, {11, 2940, 2, 6996}, {12, 2941, 2, 6996}, {13, 2942, 1, 6996}, {14, 2943, 2, 6996}, {15, 2944, 1, 6996}, {10, 2945, 1, 6997}, {11, 2946, 2, 6997}, {12, 2947, 2, 6997}, {13, 2948, 1, 6997}, {14, 2949, 2, 6997}, {15, 2950, 1, 6997}, {11, 2933, 1, 6998}, {13, 2934, 2, 6998}, {14, 2935, 2, 6998}, {16, 2936, 1, 6998}, {17, 2937, 2, 6998}, {18, 2938, 1, 6998}, {11, 2939, 1, 6999}, {13, 2940, 2, 6999}, {14, 2941, 2, 6999}, {16, 2942, 1, 6999}, {17, 2943, 2, 6999}, {18, 2944, 1, 6999}, {11, 2945, 1, 7000}, {13, 2946, 2, 7000}, {14, 2947, 2, 7000}, {16, 2948, 1, 7000}, {17, 2949, 2, 7000}, {18, 2950, 1, 7000}, {12, 2933, 1, 7001}, {14, 2934, 2, 7001}, {15, 2935, 2, 7001}, {17, 2936, 1, 7001}, {18, 2937, 2, 7001}, {19, 2938, 1, 7001}, {12, 2939, 1, 7002}, {14, 2940, 2, 7002}, {15, 2941, 2, 7002}, {17, 2942, 1, 7002}, {18, 2943, 2, 7002}, {19, 2944, 1, 7002}, {12, 2945, 1, 7003}, {14, 2946, 2, 7003}, {15, 2947, 2, 7003}, {17, 2948, 1, 7003}, {18, 2949, 2, 7003}, {19, 2950, 1, 7003}, {10, 3030, 1, 7004}, {11, 3031, 2, 7004}, {12, 3032, 2, 7004}, {13, 3033, 1, 7004}, {14, 3034, 2, 7004}, {15, 3035, 1, 7004}, {11, 3030, 1, 7005}, {13, 3031, 2, 7005}, {14, 3032, 2, 7005}, {16, 3033, 1, 7005}, {17, 3034, 2, 7005}, {18, 3035, 1, 7005}, {12, 3030, 1, 7006}, {14, 3031, 2, 7006}, {15, 3032, 2, 7006}, {17, 3033, 1, 7006}, {18, 3034, 2, 7006}, {19, 3035, 1, 7006}, {10, 3036, 1, 7007}, {11, 3037, 2, 7007}, {12, 3038, 2, 7007}, {13, 3039, 1, 7007}, {14, 3040, 2, 7007}, {15, 3041, 1, 7007}, {10, 3037, 1, 7008}, {11, 3039, 2, 7008}, {12, 3040, 2, 7008}, {13, 3042, 1, 7008}, {14, 3043, 2, 7008}, {15, 3044, 1, 7008}, {10, 3038, 1, 7009}, {11, 3040, 2, 7009}, {12, 3041, 2, 7009}, {13, 3043, 1, 7009}, {14, 3044, 2, 7009}, {15, 3045, 1, 7009}, {11, 3036, 1, 7010}, {13, 3037, 2, 7010}, {14, 3038, 2, 7010}, {16, 3039, 1, 7010}, {17, 3040, 2, 7010}, {18, 3041, 1, 7010}, {11, 3037, 1, 7011}, {13, 3039, 2, 7011}, {14, 3040, 2, 7011}, {16, 3042, 1, 7011}, {17, 3043, 2, 7011}, {18, 3044, 1, 7011}, {11, 3038, 1, 7012}, {13, 3040, 2, 7012}, {14, 3041, 2, 7012}, {16, 3043, 1, 7012}, {17, 3044, 2, 7012}, {18, 3045, 1, 7012}, {12, 3036, 1, 7013}, {14, 3037, 2, 7013}, {15, 3038, 2, 7013}, {17, 3039, 1, 7013}, {18, 3040, 2, 7013}, {19, 3041, 1, 7013}, {12, 3037, 1, 7014}, {14, 3039, 2, 7014}, {15, 3040, 2, 7014}, {17, 3042, 1, 7014}, {18, 3043, 2, 7014}, {19, 3044, 1, 7014}, {12, 3038, 1, 7015}, {14, 3040, 2, 7015}, {15, 3041, 2, 7015}, {17, 3043, 1, 7015}, {18, 3044, 2, 7015}, {19, 3045, 1, 7015}, {10, 3193, 1, 7016}, {11, 3194, 2, 7016}, {12, 3195, 2, 7016}, {13, 3196, 1, 7016}, {14, 3197, 2, 7016}, {15, 3198, 1, 7016}, {10, 3199, 1, 7017}, {11, 3200, 2, 7017}, {12, 3201, 2, 7017}, {13, 3202, 1, 7017}, {14, 3203, 2, 7017}, {15, 3204, 1, 7017}, {10, 3205, 1, 7018}, {11, 3206, 2, 7018}, {12, 3207, 2, 7018}, {13, 3208, 1, 7018}, {14, 3209, 2, 7018}, {15, 3210, 1, 7018}, {11, 3193, 1, 7019}, {13, 3194, 2, 7019}, {14, 3195, 2,

7019}, {16, 3196, 1, 7019}, {17, 3197, 2, 7019}, {18, 3198, 1, 7019}, {11, 3199, 1, 7020}, {13, 3200, 2, 7020}, {14, 3201, 2, 7020}, {16, 3202, 1, 7020}, {17, 3203, 2, 7020}, {18, 3204, 1, 7020}, {11, 3205, 1, 7021}, {13, 3206, 2, 7021}, {14, 3207, 2, 7021}, {16, 3208, 1, 7021}, {17, 3209, 2, 7021}, {18, 3210, 1, 7021}, {12, 3193, 1, 7022}, {14, 3194, 2, 7022}, {15, 3195, 2, 7022}, {17, 3196, 1, 7022}, {18, 3197, 2, 7022}, {19, 3198, 1, 7022}, {12, 3199, 1, 7023}, {14, 3200, 2, 7023}, {15, 3201, 2, 7023}, {17, 3202, 1, 7023}, {18, 3203, 2, 7023}, {19, 3204, 1, 7023}, {12, 3205, 1, 7024}, {14, 3206, 2, 7024}, {15, 3207, 2, 7024}, {17, 3208, 1, 7024}, {18, 3209, 2, 7024}, {19, 3210, 1, 7024}, {10, 3245, 1, 7025}, {11, 3246, 2, 7025}, {12, 3247, 2, 7025}, {13, 3248, 1, 7025}, {14, 3249, 2, 7025}, {15, 3250, 1, 7025}, {11, 3245, 1, 7026}, {13, 3246, 2, 7026}, {14, 3247, 2, 7026}, {16, 3248, 1, 7026}, {17, 3249, 2, 7026}, {18, 3250, 1, 7026}, {12, 3245, 1, 7027}, {14, 3246, 2, 7027}, {15, 3247, 2, 7027}, {17, 3248, 1, 7027}, {18, 3249, 2, 7027}, {19, 3250, 1, 7027}, {10, 3036, 1, 7028}, {11, 3037, 3, 7028}, {12, 3038, 3, 7028}, {13, 3039, 3, 7028}, {14, 3040, 6, 7028}, {15, 3041, 3, 7028}, {16, 3042, 1, 7028}, {17, 3043, 3, 7028}, {18, 3044, 3, 7028}, {19, 3045, 1, 7028}, {10, 3046, 1, 7029}, {11, 3047, 3, 7029}, {12, 3048, 3, 7029}, {13, 3049, 3, 7029}, {14, 3050, 6, 7029}, {15, 3051, 3, 7029}, {16, 3052, 1, 7029}, {17, 3053, 3, 7029}, {18, 3054, 3, 7029}, {19, 3055, 1, 7029}, {10, 3047, 1, 7030}, {11, 3049, 3, 7030}, {12, 3050, 3, 7030}, {13, 3052, 3, 7030}, {14, 3053, 6, 7030}, {15, 3054, 3, 7030}, {16, 3056, 1, 7030}, {17, 3057, 3, 7030}, {18, 3058, 3, 7030}, {19, 3059, 1, 7030}, {10, 3048, 1, 7031}, {11, 3050, 3, 7031}, {12, 3051, 3, 7031}, {13, 3053, 3, 7031}, {14, 3054, 6, 7031}, {15, 3055, 3, 7031}, {16, 3057, 1, 7031}, {17, 3058, 3, 7031}, {18, 3059, 3, 7031}, {19, 3060, 1, 7031}, {20, 3030, 1, 7032}, {21, 3031, 2, 7032}, {22, 3032, 2, 7032}, {23, 3033, 1, 7032}, {24, 3034, 2, 7032}, {25, 3035, 1, 7032}, {21, 3030, 1, 7033}, {23, 3031, 2, 7033}, {24, 3032, 2, 7033}, {26, 3033, 1, 7033}, {27, 3034, 2, 7033}, {28, 3035, 1, 7033}, {22, 3030, 1, 7034}, {24, 3031, 2, 7034}, {25, 3032, 2, 7034}, {27, 3033, 1, 7034}, {28, 3034, 2, 7034}, {29, 3035, 1, 7034}, {23, 3030, 1, 7035}, {26, 3031, 2, 7035}, {27, 3032, 2, 7035}, {30, 3033, 1, 7035}, {31, 3034, 2, 7035}, {32, 3035, 1, 7035}, {24, 3030, 1, 7036}, {27, 3031, 2, 7036}, {28, 3032, 2, 7036}, {31, 3033, 1, 7036}, {32, 3034, 2, 7036}, {33, 3035, 1, 7036}, {25, 3030, 1, 7037}, {28, 3031, 2, 7037}, {29, 3032, 2, 7037}, {32, 3033, 1, 7037}, {33, 3034, 2, 7037}, {34, 3035, 1, 7037}, {10, 3211, 1, 7038}, {11, 3212, 3, 7038}, {12, 3213, 3, 7038}, {13, 3214, 3, 7038}, {14, 3215, 6, 7038}, {15, 3216, 3, 7038}, {16, 3217, 1, 7038}, {17, 3218, 3, 7038}, {18, 3219, 3, 7038}, {19, 3220, 1, 7038}, {10, 3221, 1, 7039}, {11, 3222, 3, 7039}, {12, 3223, 3, 7039}, {13, 3224, 3, 7039}, {14, 3225, 6, 7039}, {15, 3226, 3, 7039}, {16, 3227, 1, 7039}, {17, 3228, 3, 7039}, {18, 3229, 3, 7039}, {19, 3230, 1, 7039}, {10, 3231, 1, 7040}, {11, 3232, 3, 7040}, {12, 3233, 3, 7040}, {13, 3234, 3, 7040}, {14, 3235, 6, 7040}, {15, 3236, 3, 7040}, {16, 3237, 1, 7040}, {17, 3238, 3, 7040}, {18, 3239, 3, 7040}, {19, 3240, 1, 7040}, {10, 3251, 1, 7041}, {11, 3252, 3, 7041}, {12, 3253, 3, 7041}, {13, 3254, 3, 7041}, {14, 3255, 6, 7041}, {15, 3256, 3, 7041}, {16, 3257, 1, 7041}, {17, 3258, 3, 7041}, {18, 3259, 3, 7041}, {19, 3260, 1, 7041}, {10, 3566, 1, 7042}, {11, 3567, 3, 7042}, {12, 3568, 3, 7042}, {13, 3569, 3, 7042}, {14, 3570, 6, 7042}, {15, 3571, 3, 7042}, {16, 3572, 1, 7042}, {17, 3573, 3, 7042}, {18, 3574, 3, 7042}, {19, 3575, 1, 7042}, {10, 2867, 1, 7043}, {11, 2868, 1, 7043}, {11, 2870, 1, 7043}, {12, 2869, 1, 7043}, {12, 2873, 1, 7043}, {13, 2871, 1, 7043}, {14, 2872, 1, 7043}, {14, 2874, 1, 7043}, {15, 2875, 1, 7043}, {10, 2870, 1, 7044}, {11, 2871, 1, 7044}, {11, 2876, 1, 7044}, {12, 2872, 1, 7044}, {12, 2879, 1, 7044}, {13, 2877, 1, 7044}, {14, 2878, 1, 7044}, {14, 2880, 1, 7044}, {15, 2881, 1, 7044}, {10, 2873, 1, 7045}, {11, 2874, 1, 7045}, {11, 2879, 1, 7045}, {12, 2875, 1, 7045}, {12, 2882, 1, 7045}, {13, 2880, 1, 7045}, {14, 2881, 1, 7045}, {14, 2883, 1, 7045}, {15, 2884, 1, 7045}, {11, 2867, 1, 7046}, {13, 2868, 1, 7046}, {13, 2870, 1, 7046}, {14, 2869, 1, 7046}, {14, 2873, 1, 7046}, {16, 2871, 1, 7046}, {17, 2872, 1, 7046}, {17, 2874, 1, 7046}, {18, 2875, 1, 7046}, {11, 2870, 1, 7047}, {13, 2871, 1, 7047}, {13, 2876, 1, 7047}, {14, 2872, 1, 7047}, {14, 2879, 1, 7047}, {16, 2877, 1, 7047}, {17, 2878, 1, 7047}, {17, 2880, 1, 7047}, {18, 2881, 1, 7047}, {11, 2873, 1, 7048}, {13, 2874, 1, 7048}, {13, 2879, 1, 7048}, {14, 2875, 1, 7048}, {14, 2882, 1, 7048}, {16, 2880, 1, 7048}, {17, 2881, 1, 7048}, {17, 2883, 1, 7048}, {18, 2884, 1, 7048}, {12, 2867, 1, 7049}, {14, 2868, 1, 7049}, {14, 2870, 1, 7049}, {15, 2869, 1, 7049}, {15, 2873, 1, 7049}, {17, 2871, 1, 7049}, {18, 2872, 1, 7049}, {18, 2874, 1, 7049}, {19, 2875, 1, 7049}, {12, 2870, 1, 7050}, {14, 2871, 1, 7050}, {14, 2876, 1, 7050}, {15, 2872, 1, 7050}, {15, 2879, 1, 7050}, {17, 2877, 1, 7050}, {18, 2878, 1, 7050}, {18, 2880, 1, 7050}, {19, 2881, 1, 7050}, {12, 2873, 1, 7051}, {14, 2874, 1, 7051}, {14, 2879, 1, 7051}, {15, 2875, 1, 7051}, {15, 2882, 1, 7051}, {17, 2880, 1, 7051}, {18, 2881, 1, 7051}, {18, 2883, 1, 7051}, {19, 2884, 1, 7051}, {10, 2885, 1, 7052}, {11, 2886, 1, 7052}, {11, 2891, 1, 7052}, {12, 2887, 1, 7052}, {12, 2897, 1, 7052}, {13, 2892, 1, 7052}, {14, 2893, 1, 7052}, {14, 2898, 1, 7052}, {15, 2899, 1, 7052}, {10, 2886, 1, 7053}, {11, 2888, 1, 7053}, {11, 2892, 1, 7053}, {12, 2889, 1, 7053}, {12, 2898, 1, 7053}, {13, 2894, 1, 7053}, {14, 2895, 1, 7053}, {14, 2900, 1, 7053}, {15, 2901, 1, 7053}, {10, 2887, 1, 7054}, {11, 2889, 1, 7054}, {11, 2893, 1, 7054}, {12, 2890, 1, 7054}, {12, 2899, 1, 7054}, {13, 2895, 1, 7054}, {14, 2896, 1, 7054}, {14, 2901, 1, 7054}, {15, 2902, 1, 7054}, {10, 2891, 1, 7055}, {11, 2892, 1, 7055}, {11, 2903, 1, 7055}, {12, 2893, 1, 7055}, {12, 2909, 1, 7055}, {13, 2904, 1, 7055}, {14, 2905, 1, 7055}, {14, 2910, 1, 7055},

{15, 2911, 1, 7055}, {10, 2892, 1, 7056}, {11, 2894, 1, 7056}, {11, 2904, 1, 7056}, {12, 2895, 1, 7056}, {12, 2910, 1, 7056}, {13, 2906, 1, 7056}, {14, 2907, 1, 7056}, {14, 2912, 1, 7056}, {15, 2913, 1, 7056}, {10, 2893, 1, 7057}, {11, 2895, 1, 7057}, {11, 2905, 1, 7057}, {12, 2896, 1, 7057}, {12, 2911, 1, 7057}, {13, 2907, 1, 7057}, {14, 2908, 1, 7057}, {14, 2913, 1, 7057}, {15, 2914, 1, 7057}, {10, 2897, 1, 7058}, {11, 2898, 1, 7058}, {11, 2909, 1, 7058}, {12, 2899, 1, 7058}, {12, 2915, 1, 7058}, {13, 2910, 1, 7058}, {14, 2911, 1, 7058}, {14, 2916, 1, 7058}, {15, 2917, 1, 7058}, {10, 2898, 1, 7059}, {11, 2900, 1, 7059}, {11, 2910, 1, 7059}, {12, 2901, 1, 7059}, {12, 2916, 1, 7059}, {13, 2912, 1, 7059}, {14, 2913, 1, 7059}, {14, 2918, 1, 7059}, {15, 2919, 1, 7059}, {10, 2899, 1, 7060}, {11, 2901, 1, 7060}, {11, 2911, 1, 7060}, {12, 2902, 1, 7060}, {12, 2917, 1, 7060}, {13, 2913, 1, 7060}, {14, 2914, 1, 7060}, {14, 2919, 1, 7060}, {15, 2920, 1, 7060}, {11, 2885, 1, 7061}, {13, 2886, 1, 7061}, {13, 2891, 1, 7061}, {14, 2887, 1, 7061}, {14, 2897, 1, 7061}, {16, 2892, 1, 7061}, {17, 2893, 1, 7061}, {17, 2898, 1, 7061}, {18, 2899, 1, 7061}, {11, 2886, 1, 7062}, {13, 2888, 1, 7062}, {13, 2892, 1, 7062}, {14, 2889, 1, 7062}, {14, 2898, 1, 7062}, {16, 2894, 1, 7062}, {17, 2895, 1, 7062}, {17, 2900, 1, 7062}, {18, 2901, 1, 7062}, {11, 2887, 1, 7063}, {13, 2889, 1, 7063}, {13, 2893, 1, 7063}, {14, 2890, 1, 7063}, {14, 2899, 1, 7063}, {16, 2895, 1, 7063}, {17, 2896, 1, 7063}, {17, 2901, 1, 7063}, {18, 2902, 1, 7063}, {11, 2891, 1, 7064}, {13, 2892, 1, 7064}, {13, 2903, 1, 7064}, {14, 2893, 1, 7064}, {14, 2909, 1, 7064}, {16, 2904, 1, 7064}, {17, 2905, 1, 7064}, {17, 2910, 1, 7064}, {18, 2911, 1, 7064}, {11, 2892, 1, 7065}, {13, 2894, 1, 7065}, {13, 2904, 1, 7065}, {14, 2895, 1, 7065}, {14, 2910, 1, 7065}, {16, 2906, 1, 7065}, {17, 2907, 1, 7065}, {17, 2912, 1, 7065}, {18, 2913, 1, 7065}, {11, 2893, 1, 7066}, {13, 2895, 1, 7066}, {13, 2905, 1, 7066}, {14, 2896, 1, 7066}, {14, 2911, 1, 7066}, {16, 2907, 1, 7066}, {17, 2908, 1, 7066}, {17, 2913, 1, 7066}, {18, 2914, 1, 7066}, {11, 2897, 1, 7067}, {13, 2898, 1, 7067}, {13, 2909, 1, 7067}, {14, 2899, 1, 7067}, {14, 2915, 1, 7067}, {16, 2910, 1, 7067}, {17, 2911, 1, 7067}, {17, 2916, 1, 7067}, {18, 2917, 1, 7067}, {11, 2898, 1, 7068}, {13, 2900, 1, 7068}, {13, 2910, 1, 7068}, {14, 2901, 1, 7068}, {14, 2916, 1, 7068}, {16, 2912, 1, 7068}, {17, 2913, 1, 7068}, {17, 2918, 1, 7068}, {18, 2919, 1, 7068}, {11, 2899, 1, 7069}, {13, 2901, 1, 7069}, {13, 2911, 1, 7069}, {14, 2902, 1, 7069}, {14, 2917, 1, 7069}, {16, 2913, 1, 7069}, {17, 2914, 1, 7069}, {17, 2919, 1, 7069}, {18, 2920, 1, 7069}, {12, 2885, 1, 7070}, {14, 2886, 1, 7070}, {14, 2891, 1, 7070}, {15, 2887, 1, 7070}, {15, 2897, 1, 7070}, {17, 2892, 1, 7070}, {18, 2893, 1, 7070}, {18, 2898, 1, 7070}, {19, 2899, 1, 7070}, {12, 2886, 1, 7071}, {14, 2888, 1, 7071}, {14, 2892, 1, 7071}, {15, 2889, 1, 7071}, {15, 2898, 1, 7071}, {17, 2894, 1, 7071}, {18, 2895, 1, 7071}, {18, 2900, 1, 7071}, {19, 2901, 1, 7071}, {12, 2887, 1, 7072}, {14, 2889, 1, 7072}, {14, 2893, 1, 7072}, {15, 2890, 1, 7072}, {15, 2899, 1, 7072}, {17, 2895, 1, 7072}, {18, 2896, 1, 7072}, {18, 2901, 1, 7072}, {19, 2902, 1, 7072}, {12, 2891, 1, 7073}, {14, 2892, 1, 7073}, {14, 2903, 1, 7073}, {15, 2893, 1, 7073}, {15, 2909, 1, 7073}, {17, 2904, 1, 7073}, {18, 2905, 1, 7073}, {18, 2910, 1, 7073}, {19, 2911, 1, 7073}, {12, 2892, 1, 7074}, {14, 2894, 1, 7074}, {14, 2904, 1, 7074}, {15, 2895, 1, 7074}, {15, 2910, 1, 7074}, {17, 2906, 1, 7074}, {18, 2907, 1, 7074}, {18, 2912, 1, 7074}, {19, 2913, 1, 7074}, {12, 2893, 1, 7075}, {14, 2895, 1, 7075}, {14, 2905, 1, 7075}, {15, 2896, 1, 7075}, {15, 2911, 1, 7075}, {17, 2907, 1, 7075}, {18, 2908, 1, 7075}, {18, 2913, 1, 7075}, {19, 2914, 1, 7075}, {12, 2897, 1, 7076}, {14, 2898, 1, 7076}, {14, 2909, 1, 7076}, {15, 2899, 1, 7076}, {15, 2915, 1, 7076}, {17, 2910, 1, 7076}, {18, 2911, 1, 7076}, {18, 2916, 1, 7076}, {19, 2917, 1, 7076}, {12, 2898, 1, 7077}, {14, 2900, 1, 7077}, {14, 2910, 1, 7077}, {15, 2901, 1, 7077}, {15, 2916, 1, 7077}, {17, 2912, 1, 7077}, {18, 2913, 1, 7077}, {18, 2918, 1, 7077}, {19, 2919, 1, 7077}, {12, 2899, 1, 7078}, {14, 2901, 1, 7078}, {14, 2911, 1, 7078}, {15, 2902, 1, 7078}, {15, 2917, 1, 7078}, {17, 2913, 1, 7078}, {18, 2914, 1, 7078}, {18, 2919, 1, 7078}, {19, 2920, 1, 7078}, {10, 2924, 1, 7079}, {11, 2925, 1, 7079}, {11, 2927, 1, 7079}, {12, 2926, 1, 7079}, {12, 2930, 1, 7079}, {13, 2928, 1, 7079}, {14, 2929, 1, 7079}, {14, 2931, 1, 7079}, {15, 2932, 1, 7079}, {11, 2924, 1, 7080}, {13, 2925, 1, 7080}, {13, 2927, 1, 7080}, {14, 2926, 1, 7080}, {14, 2930, 1, 7080}, {16, 2928, 1, 7080}, {17, 2929, 1, 7080}, {17, 2931, 1, 7080}, {18, 2932, 1, 7080}, {12, 2924, 1, 7081}, {14, 2925, 1, 7081}, {14, 2927, 1, 7081}, {15, 2926, 1, 7081}, {15, 2930, 1, 7081}, {17, 2928, 1, 7081}, {18, 2929, 1, 7081}, {18, 2931, 1, 7081}, {19, 2932, 1, 7081}, {10, 2933, 1, 7082}, {11, 2934, 1, 7082}, {11, 2939, 1, 7082}, {12, 2935, 1, 7082}, {12, 2945, 1, 7082}, {13, 2940, 1, 7082}, {14, 2941, 1, 7082}, {14, 2946, 1, 7082}, {15, 2947, 1, 7082}, {10, 2934, 1, 7083}, {11, 2936, 1, 7083}, {11, 2940, 1, 7083}, {12, 2937, 1, 7083}, {12, 2946, 1, 7083}, {13, 2942, 1, 7083}, {14, 2943, 1, 7083}, {14, 2948, 1, 7083}, {15, 2949, 1, 7083}, {10, 2935, 1, 7084}, {11, 2937, 1, 7084}, {11, 2941, 1, 7084}, {12, 2938, 1, 7084}, {12, 2947, 1, 7084}, {13, 2943, 1, 7084}, {14, 2944, 1, 7084}, {14, 2949, 1, 7084}, {15, 2950, 1, 7084}, {11, 2933, 1, 7085}, {13, 2934, 1, 7085}, {13, 2939, 1, 7085}, {14, 2935, 1, 7085}, {14, 2945, 1, 7085}, {16, 2940, 1, 7085}, {17, 2941, 1, 7085}, {17, 2946, 1, 7085}, {18, 2947, 1, 7085}, {11, 2934, 1, 7086}, {13, 2936, 1, 7086}, {13, 2940, 1, 7086}, {14, 2937, 1, 7086}, {14, 2946, 1, 7086}, {16, 2942, 1, 7086}, {17, 2943, 1, 7086}, {17, 2948, 1, 7086}, {18, 2949, 1, 7086}, {11, 2935, 1, 7087}, {13, 2937, 1, 7087}, {13, 2941, 1, 7087}, {14, 2938, 1, 7087}, {14, 2947, 1, 7087}, {16, 2943, 1, 7087}, {17, 2944, 1, 7087}, {17, 2949, 1, 7087}, {18, 2950, 1, 7087}, {12, 2933, 1, 7088}, {14, 2934, 1, 7088}, {14, 2939, 1, 7088}, {15, 2935, 1, 7088}, {15,

2945, 1, 7088}, {17, 2940, 1, 7088}, {18, 2941, 1, 7088}, {18, 2946, 1, 7088}, {19, 2947, 1, 7088}, {12, 2934, 1, 7089}, {14, 2936, 1, 7089}, {14, 2940, 1, 7089}, {15, 2937, 1, 7089}, {15, 2946, 1, 7089}, {17, 2942, 1, 7089}, {18, 2943, 1, 7089}, {18, 2948, 1, 7089}, {19, 2949, 1, 7089}, {12, 2935, 1, 7090}, {14, 2937, 1, 7090}, {14, 2941, 1, 7090}, {15, 2938, 1, 7090}, {15, 2947, 1, 7090}, {17, 2943, 1, 7090}, {18, 2944, 1, 7090}, {18, 2949, 1, 7090}, {19, 2950, 1, 7090}, {10, 2951, 1, 7091}, {11, 2952, 1, 7091}, {11, 2961, 1, 7091}, {12, 2953, 1, 7091}, {12, 2971, 1, 7091}, {13, 2962, 1, 7091}, {14, 2963, 1, 7091}, {14, 2972, 1, 7091}, {15, 2973, 1, 7091}, {10, 2952, 1, 7092}, {11, 2954, 1, 7092}, {11, 2962, 1, 7092}, {12, 2955, 1, 7092}, {12, 2972, 1, 7092}, {13, 2964, 1, 7092}, {14, 2965, 1, 7092}, {14, 2974, 1, 7092}, {15, 2975, 1, 7092}, {10, 2953, 1, 7093}, {11, 2955, 1, 7093}, {11, 2963, 1, 7093}, {12, 2956, 1, 7093}, {12, 2973, 1, 7093}, {13, 2965, 1, 7093}, {14, 2966, 1, 7093}, {14, 2975, 1, 7093}, {15, 2976, 1, 7093}, {10, 2954, 1, 7094}, {11, 2957, 1, 7094}, {11, 2964, 1, 7094}, {12, 2958, 1, 7094}, {12, 2974, 1, 7094}, {13, 2967, 1, 7094}, {14, 2968, 1, 7094}, {14, 2977, 1, 7094}, {15, 2978, 1, 7094}, {10, 2955, 1, 7095}, {11, 2958, 1, 7095}, {11, 2965, 1, 7095}, {12, 2959, 1, 7095}, {12, 2975, 1, 7095}, {13, 2968, 1, 7095}, {14, 2969, 1, 7095}, {14, 2978, 1, 7095}, {15, 2979, 1, 7095}, {10, 2956, 1, 7096}, {11, 2959, 1, 7096}, {11, 2966, 1, 7096}, {12, 2960, 1, 7096}, {12, 2976, 1, 7096}, {13, 2969, 1, 7096}, {14, 2970, 1, 7096}, {14, 2979, 1, 7096}, {15, 2980, 1, 7096}, {11, 2951, 1, 7097}, {13, 2952, 1, 7097}, {13, 2961, 1, 7097}, {14, 2953, 1, 7097}, {14, 2971, 1, 7097}, {16, 2962, 1, 7097}, {17, 2963, 1, 7097}, {17, 2972, 1, 7097}, {18, 2973, 1, 7097}, {11, 2952, 1, 7098}, {13, 2954, 1, 7098}, {13, 2962, 1, 7098}, {14, 2955, 1, 7098}, {14, 2972, 1, 7098}, {16, 2964, 1, 7098}, {17, 2965, 1, 7098}, {17, 2974, 1, 7098}, {18, 2975, 1, 7098}, {11, 2953, 1, 7099}, {13, 2955, 1, 7099}, {13, 2963, 1, 7099}, {14, 2956, 1, 7099}, {14, 2973, 1, 7099}, {16, 2965, 1, 7099}, {17, 2966, 1, 7099}, {17, 2975, 1, 7099}, {18, 2976, 1, 7099}, {11, 2954, 1, 7100}, {13, 2957, 1, 7100}, {13, 2964, 1, 7100}, {14, 2958, 1, 7100}, {14, 2974, 1, 7100}, {16, 2967, 1, 7100}, {17, 2968, 1, 7100}, {17, 2977, 1, 7100}, {18, 2978, 1, 7100}, {11, 2955, 1, 7101}, {13, 2958, 1, 7101}, {13, 2965, 1, 7101}, {14, 2959, 1, 7101}, {14, 2975, 1, 7101}, {16, 2968, 1, 7101}, {17, 2969, 1, 7101}, {17, 2978, 1, 7101}, {18, 2979, 1, 7101}, {11, 2956, 1, 7102}, {13, 2959, 1, 7102}, {13, 2966, 1, 7102}, {14, 2960, 1, 7102}, {14, 2976, 1, 7102}, {16, 2969, 1, 7102}, {17, 2970, 1, 7102}, {17, 2979, 1, 7102}, {18, 2980, 1, 7102}, {12, 2951, 1, 7103}, {14, 2952, 1, 7103}, {14, 2961, 1, 7103}, {15, 2953, 1, 7103}, {15, 2971, 1, 7103}, {17, 2962, 1, 7103}, {18, 2963, 1, 7103}, {18, 2972, 1, 7103}, {19, 2973, 1, 7103}, {12, 2952, 1, 7104}, {14, 2954, 1, 7104}, {14, 2962, 1, 7104}, {15, 2955, 1, 7104}, {15, 2972, 1, 7104}, {17, 2964, 1, 7104}, {18, 2965, 1, 7104}, {18, 2974, 1, 7104}, {19, 2975, 1, 7104}, {12, 2953, 1, 7105}, {14, 2955, 1, 7105}, {14, 2963, 1, 7105}, {15, 2956, 1, 7105}, {15, 2973, 1, 7105}, {17, 2965, 1, 7105}, {18, 2966, 1, 7105}, {18, 2975, 1, 7105}, {19, 2976, 1, 7105}, {12, 2954, 1, 7106}, {14, 2957, 1, 7106}, {14, 2964, 1, 7106}, {15, 2958, 1, 7106}, {15, 2974, 1, 7106}, {17, 2967, 1, 7106}, {18, 2968, 1, 7106}, {18, 2977, 1, 7106}, {19, 2978, 1, 7106}, {12, 2955, 1, 7107}, {14, 2958, 1, 7107}, {14, 2965, 1, 7107}, {15, 2959, 1, 7107}, {15, 2975, 1, 7107}, {17, 2968, 1, 7107}, {18, 2969, 1, 7107}, {18, 2978, 1, 7107}, {19, 2979, 1, 7107}, {12, 2956, 1, 7108}, {14, 2959, 1, 7108}, {14, 2966, 1, 7108}, {15, 2960, 1, 7108}, {15, 2976, 1, 7108}, {17, 2969, 1, 7108}, {18, 2970, 1, 7108}, {18, 2979, 1, 7108}, {19, 2980, 1, 7108}, {10, 3067, 1, 7109}, {11, 3068, 1, 7109}, {11, 3070, 1, 7109}, {12, 3069, 1, 7109}, {12, 3073, 1, 7109}, {13, 3071, 1, 7109}, {14, 3072, 1, 7109}, {14, 3074, 1, 7109}, {15, 3075, 1, 7109}, {10, 3070, 1, 7110}, {11, 3071, 1, 7110}, {11, 3076, 1, 7110}, {12, 3072, 1, 7110}, {12, 3079, 1, 7110}, {13, 3077, 1, 7110}, {14, 3078, 1, 7110}, {14, 3080, 1, 7110}, {15, 3081, 1, 7110}, {10, 3073, 1, 7111}, {11, 3074, 1, 7111}, {11, 3079, 1, 7111}, {12, 3075, 1, 7111}, {12, 3082, 1, 7111}, {13, 3080, 1, 7111}, {14, 3081, 1, 7111}, {14, 3083, 1, 7111}, {15, 3084, 1, 7111}, {11, 3067, 1, 7112}, {13, 3068, 1, 7112}, {13, 3070, 1, 7112}, {14, 3069, 1, 7112}, {14, 3073, 1, 7112}, {16, 3071, 1, 7112}, {17, 3072, 1, 7112}, {17, 3074, 1, 7112}, {18, 3075, 1, 7112}, {11, 3070, 1, 7113}, {13, 3071, 1, 7113}, {13, 3076, 1, 7113}, {14, 3072, 1, 7113}, {14, 3079, 1, 7113}, {16, 3077, 1, 7113}, {17, 3078, 1, 7113}, {17, 3080, 1, 7113}, {18, 3081, 1, 7113}, {11, 3073, 1, 7114}, {13, 3074, 1, 7114}, {13, 3079, 1, 7114}, {14, 3075, 1, 7114}, {14, 3082, 1, 7114}, {16, 3080, 1, 7114}, {17, 3081, 1, 7114}, {17, 3083, 1, 7114}, {18, 3084, 1, 7114}, {12, 3067, 1, 7115}, {14, 3068, 1, 7115}, {14, 3070, 1, 7115}, {15, 3069, 1, 7115}, {15, 3073, 1, 7115}, {17, 3071, 1, 7115}, {18, 3072, 1, 7115}, {18, 3074, 1, 7115}, {19, 3075, 1, 7115}, {12, 3070, 1, 7116}, {14, 3071, 1, 7116}, {14, 3076, 1, 7116}, {15, 3072, 1, 7116}, {15, 3079, 1, 7116}, {17, 3077, 1, 7116}, {18, 3078, 1, 7116}, {18, 3080, 1, 7116}, {19, 3081, 1, 7116}, {12, 3073, 1, 7117}, {14, 3074, 1, 7117}, {14, 3079, 1, 7117}, {15, 3075, 1, 7117}, {15, 3082, 1, 7117}, {17, 3080, 1, 7117}, {18, 3081, 1, 7117}, {18, 3083, 1, 7117}, {19, 3084, 1, 7117}, {10, 3085, 1, 7118}, {11, 3086, 1, 7118}, {11, 3091, 1, 7118}, {12, 3087, 1, 7118}, {12, 3097, 1, 7118}, {13, 3092, 1, 7118}, {14, 3093, 1, 7118}, {14, 3098, 1, 7118}, {15, 3099, 1, 7118}, {10, 3086, 1, 7119}, {11, 3088, 1, 7119}, {11, 3092, 1, 7119}, {12, 3089, 1, 7119}, {12, 3098, 1, 7119}, {13, 3094, 1, 7119}, {14, 3095, 1, 7119}, {14, 3100, 1, 7119}, {15, 3101, 1, 7119}, {10, 3087, 1, 7120}, {11, 3089, 1, 7120}, {11, 3093, 1, 7120}, {12, 3090, 1, 7120}, {12, 3099, 1, 7120}, {13, 3095, 1, 7120}, {14, 3096, 1, 7120}, {14, 3101, 1, 7120}, {15, 3102, 1, 7120}, {10, 3091,

1, 7121}, {11, 3092, 1, 7121}, {11, 3103, 1, 7121}, {12, 3093, 1, 7121}, {12, 3109, 1, 7121}, {13, 3104, 1, 7121}, {14, 3105, 1, 7121}, {14, 3110, 1, 7121}, {15, 3111, 1, 7121}, {10, 3092, 1, 7122}, {11, 3094, 1, 7122}, {11, 3104, 1, 7122}, {12, 3095, 1, 7122}, {12, 3110, 1, 7122}, {13, 3106, 1, 7122}, {14, 3107, 1, 7122}, {14, 3112, 1, 7122}, {15, 3113, 1, 7122}, {10, 3093, 1, 7123}, {11, 3095, 1, 7123}, {11, 3105, 1, 7123}, {12, 3096, 1, 7123}, {12, 3111, 1, 7123}, {13, 3107, 1, 7123}, {14, 3108, 1, 7123}, {14, 3113, 1, 7123}, {15, 3114, 1, 7123}, {10, 3097, 1, 7124}, {11, 3098, 1, 7124}, {11, 3109, 1, 7124}, {12, 3099, 1, 7124}, {12, 3115, 1, 7124}, {13, 3110, 1, 7124}, {14, 3111, 1, 7124}, {14, 3116, 1, 7124}, {15, 3117, 1, 7124}, {10, 3098, 1, 7125}, {11, 3100, 1, 7125}, {11, 3110, 1, 7125}, {12, 3101, 1, 7125}, {12, 3116, 1, 7125}, {13, 3112, 1, 7125}, {14, 3113, 1, 7125}, {14, 3118, 1, 7125}, {15, 3119, 1, 7125}, {10, 3099, 1, 7126}, {11, 3101, 1, 7126}, {11, 3111, 1, 7126}, {12, 3102, 1, 7126}, {12, 3117, 1, 7126}, {13, 3113, 1, 7126}, {14, 3114, 1, 7126}, {14, 3119, 1, 7126}, {15, 3120, 1, 7126}, {11, 3085, 1, 7127}, {13, 3086, 1, 7127}, {13, 3091, 1, 7127}, {14, 3087, 1, 7127}, {14, 3097, 1, 7127}, {16, 3092, 1, 7127}, {17, 3093, 1, 7127}, {17, 3098, 1, 7127}, {18, 3099, 1, 7127}, {11, 3086, 1, 7128}, {13, 3088, 1, 7128}, {13, 3092, 1, 7128}, {14, 3089, 1, 7128}, {14, 3098, 1, 7128}, {16, 3094, 1, 7128}, {17, 3095, 1, 7128}, {17, 3100, 1, 7128}, {18, 3101, 1, 7128}, {11, 3087, 1, 7129}, {13, 3089, 1, 7129}, {13, 3093, 1, 7129}, {14, 3090, 1, 7129}, {14, 3099, 1, 7129}, {16, 3095, 1, 7129}, {17, 3096, 1, 7129}, {17, 3101, 1, 7129}, {18, 3102, 1, 7129}, {11, 3091, 1, 7130}, {13, 3092, 1, 7130}, {13, 3103, 1, 7130}, {14, 3093, 1, 7130}, {14, 3109, 1, 7130}, {16, 3104, 1, 7130}, {17, 3105, 1, 7130}, {17, 3110, 1, 7130}, {18, 3111, 1, 7130}, {11, 3092, 1, 7131}, {13, 3094, 1, 7131}, {13, 3104, 1, 7131}, {14, 3095, 1, 7131}, {14, 3110, 1, 7131}, {16, 3106, 1, 7131}, {17, 3107, 1, 7131}, {17, 3112, 1, 7131}, {18, 3113, 1, 7131}, {11, 3093, 1, 7132}, {13, 3095, 1, 7132}, {13, 3105, 1, 7132}, {14, 3096, 1, 7132}, {14, 3111, 1, 7132}, {16, 3107, 1, 7132}, {17, 3108, 1, 7132}, {17, 3113, 1, 7132}, {18, 3114, 1, 7132}, {11, 3097, 1, 7133}, {13, 3098, 1, 7133}, {13, 3109, 1, 7133}, {14, 3099, 1, 7133}, {14, 3115, 1, 7133}, {16, 3110, 1, 7133}, {17, 3111, 1, 7133}, {17, 3116, 1, 7133}, {18, 3117, 1, 7133}, {11, 3098, 1, 7134}, {13, 3100, 1, 7134}, {13, 3110, 1, 7134}, {14, 3101, 1, 7134}, {14, 3116, 1, 7134}, {16, 3112, 1, 7134}, {17, 3113, 1, 7134}, {17, 3118, 1, 7134}, {18, 3119, 1, 7134}, {11, 3099, 1, 7135}, {13, 3101, 1, 7135}, {13, 3111, 1, 7135}, {14, 3102, 1, 7135}, {14, 3117, 1, 7135}, {16, 3113, 1, 7135}, {17, 3114, 1, 7135}, {17, 3119, 1, 7135}, {18, 3120, 1, 7135}, {12, 3085, 1, 7136}, {14, 3086, 1, 7136}, {14, 3091, 1, 7136}, {15, 3087, 1, 7136}, {15, 3097, 1, 7136}, {17, 3092, 1, 7136}, {18, 3093, 1, 7136}, {18, 3098, 1, 7136}, {19, 3099, 1, 7136}, {12, 3086, 1, 7137}, {14, 3088, 1, 7137}, {14, 3092, 1, 7137}, {15, 3089, 1, 7137}, {15, 3098, 1, 7137}, {17, 3094, 1, 7137}, {18, 3095, 1, 7137}, {18, 3100, 1, 7137}, {19, 3101, 1, 7137}, {12, 3087, 1, 7138}, {14, 3089, 1, 7138}, {14, 3093, 1, 7138}, {15, 3090, 1, 7138}, {15, 3099, 1, 7138}, {17, 3095, 1, 7138}, {18, 3096, 1, 7138}, {18, 3101, 1, 7138}, {19, 3102, 1, 7138}, {12, 3091, 1, 7139}, {14, 3092, 1, 7139}, {14, 3103, 1, 7139}, {15, 3093, 1, 7139}, {15, 3109, 1, 7139}, {17, 3104, 1, 7139}, {18, 3105, 1, 7139}, {18, 3110, 1, 7139}, {19, 3111, 1, 7139}, {12, 3092, 1, 7140}, {14, 3094, 1, 7140}, {14, 3104, 1, 7140}, {15, 3095, 1, 7140}, {15, 3110, 1, 7140}, {17, 3106, 1, 7140}, {18, 3107, 1, 7140}, {18, 3112, 1, 7140}, {19, 3113, 1, 7140}, {12, 3093, 1, 7141}, {14, 3095, 1, 7141}, {14, 3105, 1, 7141}, {15, 3096, 1, 7141}, {15, 3111, 1, 7141}, {17, 3107, 1, 7141}, {18, 3108, 1, 7141}, {18, 3113, 1, 7141}, {19, 3114, 1, 7141}, {12, 3097, 1, 7142}, {14, 3098, 1, 7142}, {14, 3109, 1, 7142}, {15, 3099, 1, 7142}, {15, 3115, 1, 7142}, {17, 3110, 1, 7142}, {18, 3111, 1, 7142}, {18, 3116, 1, 7142}, {19, 3117, 1, 7142}, {12, 3098, 1, 7143}, {14, 3100, 1, 7143}, {14, 3110, 1, 7143}, {15, 3101, 1, 7143}, {15, 3116, 1, 7143}, {17, 3112, 1, 7143}, {18, 3113, 1, 7143}, {18, 3118, 1, 7143}, {19, 3119, 1, 7143}, {12, 3099, 1, 7144}, {14, 3101, 1, 7144}, {14, 3111, 1, 7144}, {15, 3102, 1, 7144}, {15, 3117, 1, 7144}, {17, 3113, 1, 7144}, {18, 3114, 1, 7144}, {18, 3119, 1, 7144}, {19, 3120, 1, 7144}, {10, 3184, 1, 7145}, {11, 3185, 1, 7145}, {11, 3187, 1, 7145}, {12, 3186, 1, 7145}, {12, 3190, 1, 7145}, {13, 3188, 1, 7145}, {14, 3189, 1, 7145}, {14, 3191, 1, 7145}, {15, 3192, 1, 7145}, {11, 3184, 1, 7146}, {13, 3185, 1, 7146}, {13, 3187, 1, 7146}, {14, 3186, 1, 7146}, {14, 3190, 1, 7146}, {16, 3188, 1, 7146}, {17, 3189, 1, 7146}, {17, 3191, 1, 7146}, {18, 3192, 1, 7146}, {12, 3184, 1, 7147}, {14, 3185, 1, 7147}, {14, 3187, 1, 7147}, {15, 3186, 1, 7147}, {15, 3190, 1, 7147}, {17, 3188, 1, 7147}, {18, 3189, 1, 7147}, {18, 3191, 1, 7147}, {19, 3192, 1, 7147}, {10, 3193, 1, 7148}, {11, 3194, 1, 7148}, {11, 3199, 1, 7148}, {12, 3195, 1, 7148}, {12, 3205, 1, 7148}, {13, 3200, 1, 7148}, {14, 3201, 1, 7148}, {14, 3206, 1, 7148}, {15, 3207, 1, 7148}, {10, 3194, 1, 7149}, {11, 3196, 1, 7149}, {11, 3200, 1, 7149}, {12, 3197, 1, 7149}, {12, 3206, 1, 7149}, {13, 3202, 1, 7149}, {14, 3203, 1, 7149}, {14, 3208, 1, 7149}, {15, 3209, 1, 7149}, {10, 3195, 1, 7150}, {11, 3197, 1, 7150}, {11, 3201, 1, 7150}, {12, 3198, 1, 7150}, {12, 3207, 1, 7150}, {13, 3203, 1, 7150}, {14, 3204, 1, 7150}, {14, 3209, 1, 7150}, {15, 3210, 1, 7150}, {11, 3193, 1, 7151}, {13, 3194, 1, 7151}, {13, 3199, 1, 7151}, {14, 3195, 1, 7151}, {14, 3205, 1, 7151}, {16, 3200, 1, 7151}, {17, 3201, 1, 7151}, {17, 3206, 1, 7151}, {18, 3207, 1, 7151}, {11, 3194, 1, 7152}, {13, 3196, 1, 7152}, {13, 3200, 1, 7152}, {14, 3197, 1, 7152}, {14, 3206, 1, 7152}, {16, 3202, 1, 7152}, {17, 3203, 1, 7152}, {17, 3208, 1, 7152}, {18, 3209, 1, 7152}, {11, 3195, 1, 7153}, {13, 3197, 1, 7153}, {13, 3201, 1, 7153}, {14, 3198, 1, 7153}, {14, 3207, 1, 7153}, {16, 3203, 1,

7153}, {17, 3204, 1, 7153}, {17, 3209, 1, 7153}, {18, 3210, 1, 7153}, {12, 3193, 1, 7154}, {14, 3194, 1, 7154}, {14, 3199, 1, 7154}, {15, 3195, 1, 7154}, {15, 3205, 1, 7154}, {17, 3200, 1, 7154}, {18, 3201, 1, 7154}, {18, 3206, 1, 7154}, {19, 3207, 1, 7154}, {12, 3194, 1, 7155}, {14, 3196, 1, 7155}, {14, 3200, 1, 7155}, {15, 3197, 1, 7155}, {15, 3206, 1, 7155}, {17, 3202, 1, 7155}, {18, 3203, 1, 7155}, {18, 3208, 1, 7155}, {19, 3209, 1, 7155}, {12, 3195, 1, 7156}, {14, 3197, 1, 7156}, {14, 3201, 1, 7156}, {15, 3198, 1, 7156}, {15, 3207, 1, 7156}, {17, 3203, 1, 7156}, {18, 3204, 1, 7156}, {18, 3209, 1, 7156}, {19, 3210, 1, 7156}, {10, 3382, 1, 7157}, {11, 3383, 1, 7157}, {11, 3385, 1, 7157}, {12, 3384, 1, 7157}, {12, 3388, 1, 7157}, {13, 3386, 1, 7157}, {14, 3387, 1, 7157}, {14, 3389, 1, 7157}, {15, 3390, 1, 7157}, {10, 3385, 1, 7158}, {11, 3386, 1, 7158}, {11, 3391, 1, 7158}, {12, 3387, 1, 7158}, {12, 3394, 1, 7158}, {13, 3392, 1, 7158}, {14, 3393, 1, 7158}, {14, 3395, 1, 7158}, {15, 3396, 1, 7158}, {10, 3388, 1, 7159}, {11, 3389, 1, 7159}, {11, 3394, 1, 7159}, {12, 3390, 1, 7159}, {12, 3397, 1, 7159}, {13, 3395, 1, 7159}, {14, 3396, 1, 7159}, {14, 3398, 1, 7159}, {15, 3399, 1, 7159}, {11, 3382, 1, 7160}, {13, 3383, 1, 7160}, {13, 3385, 1, 7160}, {14, 3384, 1, 7160}, {14, 3388, 1, 7160}, {16, 3386, 1, 7160}, {17, 3387, 1, 7160}, {17, 3389, 1, 7160}, {18, 3390, 1, 7160}, {11, 3385, 1, 7161}, {13, 3386, 1, 7161}, {13, 3391, 1, 7161}, {14, 3387, 1, 7161}, {14, 3394, 1, 7161}, {16, 3392, 1, 7161}, {17, 3393, 1, 7161}, {17, 3395, 1, 7161}, {18, 3396, 1, 7161}, {11, 3388, 1, 7162}, {13, 3389, 1, 7162}, {13, 3394, 1, 7162}, {14, 3390, 1, 7162}, {14, 3397, 1, 7162}, {16, 3395, 1, 7162}, {17, 3396, 1, 7162}, {17, 3398, 1, 7162}, {18, 3399, 1, 7162}, {12, 3382, 1, 7163}, {14, 3383, 1, 7163}, {14, 3385, 1, 7163}, {15, 3384, 1, 7163}, {15, 3388, 1, 7163}, {17, 3386, 1, 7163}, {18, 3387, 1, 7163}, {18, 3389, 1, 7163}, {19, 3390, 1, 7163}, {12, 3385, 1, 7164}, {14, 3386, 1, 7164}, {14, 3391, 1, 7164}, {15, 3387, 1, 7164}, {15, 3394, 1, 7164}, {17, 3392, 1, 7164}, {18, 3393, 1, 7164}, {18, 3395, 1, 7164}, {19, 3396, 1, 7164}, {12, 3388, 1, 7165}, {14, 3389, 1, 7165}, {14, 3394, 1, 7165}, {15, 3390, 1, 7165}, {15, 3397, 1, 7165}, {17, 3395, 1, 7165}, {18, 3396, 1, 7165}, {18, 3398, 1, 7165}, {19, 3399, 1, 7165}, {10, 3499, 1, 7166}, {11, 3500, 1, 7166}, {11, 3502, 1, 7166}, {12, 3501, 1, 7166}, {12, 3505, 1, 7166}, {13, 3503, 1, 7166}, {14, 3504, 1, 7166}, {14, 3506, 1, 7166}, {15, 3507, 1, 7166}, {11, 3499, 1, 7167}, {13, 3500, 1, 7167}, {13, 3502, 1, 7167}, {14, 3501, 1, 7167}, {14, 3505, 1, 7167}, {16, 3503, 1, 7167}, {17, 3504, 1, 7167}, {17, 3506, 1, 7167}, {18, 3507, 1, 7167}, {12, 3499, 1, 7168}, {14, 3500, 1, 7168}, {14, 3502, 1, 7168}, {15, 3501, 1, 7168}, {15, 3505, 1, 7168}, {17, 3503, 1, 7168}, {18, 3504, 1, 7168}, {18, 3506, 1, 7168}, {19, 3507, 1, 7168}, {10, 2933, 1, 7169}, {11, 2934, 2, 7169}, {11, 2939, 1, 7169}, {12, 2935, 2, 7169}, {12, 2945, 1, 7169}, {13, 2936, 1, 7169}, {13, 2940, 2, 7169}, {14, 2937, 2, 7169}, {14, 2941, 2, 7169}, {14, 2946, 2, 7169}, {15, 2938, 1, 7169}, {15, 2947, 2, 7169}, {16, 2942, 1, 7169}, {17, 2943, 2, 7169}, {17, 2948, 1, 7169}, {18, 2944, 1, 7169}, {18, 2949, 2, 7169}, {19, 2950, 1, 7169}, {10, 2951, 1, 7170}, {11, 2952, 2, 7170}, {11, 2961, 1, 7170}, {12, 2953, 2, 7170}, {12, 2971, 1, 7170}, {13, 2954, 1, 7170}, {13, 2962, 2, 7170}, {14, 2955, 2, 7170}, {14, 2963, 2, 7170}, {14, 2972, 2, 7170}, {15, 2956, 1, 7170}, {15, 2973, 2, 7170}, {16, 2964, 1, 7170}, {17, 2965, 2, 7170}, {17, 2974, 1, 7170}, {18, 2966, 1, 7170}, {18, 2975, 2, 7170}, {19, 2976, 1, 7170}, {10, 2952, 1, 7171}, {11, 2954, 2, 7171}, {11, 2962, 1, 7171}, {12, 2955, 2, 7171}, {12, 2972, 1, 7171}, {13, 2957, 1, 7171}, {13, 2964, 2, 7171}, {14, 2958, 2, 7171}, {14, 2965, 2, 7171}, {14, 2974, 2, 7171}, {15, 2959, 1, 7171}, {15, 2975, 2, 7171}, {16, 2967, 1, 7171}, {17, 2968, 2, 7171}, {17, 2977, 1, 7171}, {18, 2969, 1, 7171}, {18, 2978, 2, 7171}, {19, 2979, 1, 7171}, {10, 2953, 1, 7172}, {11, 2955, 2, 7172}, {11, 2963, 1, 7172}, {12, 2956, 2, 7172}, {12, 2973, 1, 7172}, {13, 2958, 1, 7172}, {13, 2965, 2, 7172}, {14, 2959, 2, 7172}, {14, 2966, 2, 7172}, {14, 2975, 2, 7172}, {15, 2960, 1, 7172}, {15, 2976, 2, 7172}, {16, 2968, 1, 7172}, {17, 2969, 2, 7172}, {17, 2978, 1, 7172}, {18, 2970, 1, 7172}, {18, 2979, 2, 7172}, {19, 2980, 1, 7172}, {10, 2981, 1, 7173}, {11, 2982, 2, 7173}, {11, 2996, 1, 7173}, {12, 2983, 2, 7173}, {12, 3011, 1, 7173}, {13, 2984, 1, 7173}, {13, 2997, 2, 7173}, {14, 2985, 2, 7173}, {14, 2998, 2, 7173}, {14, 3012, 2, 7173}, {15, 2986, 1, 7173}, {15, 3013, 2, 7173}, {16, 2999, 1, 7173}, {17, 3000, 2, 7173}, {17, 3014, 1, 7173}, {18, 3001, 1, 7173}, {18, 3015, 2, 7173}, {19, 3016, 1, 7173}, {10, 2982, 1, 7174}, {11, 2984, 2, 7174}, {11, 2997, 1, 7174}, {12, 2985, 2, 7174}, {12, 3012, 1, 7174}, {13, 2987, 1, 7174}, {13, 2999, 2, 7174}, {14, 2988, 2, 7174}, {14, 3000, 2, 7174}, {14, 3014, 2, 7174}, {15, 2989, 1, 7174}, {15, 3015, 2, 7174}, {16, 3002, 1, 7174}, {17, 3003, 2, 7174}, {17, 3017, 1, 7174}, {18, 3004, 1, 7174}, {18, 3018, 2, 7174}, {19, 3019, 1, 7174}, {10, 2983, 1, 7175}, {11, 2985, 2, 7175}, {11, 2998, 1, 7175}, {12, 2986, 2, 7175}, {12, 3013, 1, 7175}, {13, 2988, 1, 7175}, {13, 3000, 2, 7175}, {14, 2989, 2, 7175}, {14, 3001, 2, 7175}, {14, 3015, 2, 7175}, {15, 2990, 1, 7175}, {15, 3016, 2, 7175}, {16, 3003, 1, 7175}, {17, 3004, 2, 7175}, {17, 3018, 1, 7175}, {18, 3005, 1, 7175}, {18, 3019, 2, 7175}, {19, 3020, 1, 7175}, {10, 2984, 1, 7176}, {11, 2987, 2, 7176}, {11, 2999, 1, 7176}, {12, 2988, 2, 7176}, {12, 3014, 1, 7176}, {13, 2991, 1, 7176}, {13, 3002, 2, 7176}, {14, 2992, 2, 7176}, {14, 3003, 2, 7176}, {14, 3017, 2, 7176}, {15, 2993, 1, 7176}, {15, 3018, 2, 7176}, {16, 3006, 1, 7176}, {17, 3007, 2, 7176}, {17, 3021, 1, 7176}, {18, 3008, 1, 7176}, {18, 3022, 2, 7176}, {19, 3023, 1, 7176}, {10, 2985, 1, 7177}, {11, 2988, 2, 7177}, {11, 3000, 1, 7177}, {12, 2989, 2, 7177}, {12, 3015, 1, 7177}, {13, 2992, 1, 7177}, {13, 3003, 2, 7177}, {14, 2993, 2, 7177}, {14, 3004, 2, 7177}, {14, 3018, 2, 7177}, {15, 2994, 1, 7177},

{15, 3019, 2, 7177}, {16, 3007, 1, 7177}, {17, 3008, 2, 7177}, {17, 3022, 1, 7177}, {18, 3009, 1, 7177}, {18, 3023, 2, 7177}, {19, 3024, 1, 7177}, {10, 2986, 1, 7178}, {11, 2989, 2, 7178}, {11, 3001, 1, 7178}, {12, 2990, 2, 7178}, {12, 3016, 1, 7178}, {13, 2993, 1, 7178}, {13, 3004, 2, 7178}, {14, 2994, 2, 7178}, {14, 3005, 2, 7178}, {14, 3019, 2, 7178}, {15, 2995, 1, 7178}, {15, 3020, 2, 7178}, {16, 3008, 1, 7178}, {17, 3009, 2, 7178}, {17, 3023, 1, 7178}, {18, 3010, 1, 7178}, {18, 3024, 2, 7178}, {19, 3025, 1, 7178}, {20, 2924, 1, 7179}, {21, 2925, 1, 7179}, {21, 2927, 1, 7179}, {22, 2926, 1, 7179}, {22, 2930, 1, 7179}, {23, 2928, 1, 7179}, {24, 2929, 1, 7179}, {24, 2931, 1, 7179}, {25, 2932, 1, 7179}, {21, 2924, 1, 7180}, {23, 2925, 1, 7180}, {23, 2927, 1, 7180}, {24, 2926, 1, 7180}, {24, 2930, 1, 7180}, {26, 2928, 1, 7180}, {27, 2929, 1, 7180}, {27, 2931, 1, 7180}, {28, 2932, 1, 7180}, {22, 2924, 1, 7181}, {24, 2925, 1, 7181}, {24, 2927, 1, 7181}, {25, 2926, 1, 7181}, {25, 2930, 1, 7181}, {27, 2928, 1, 7181}, {28, 2929, 1, 7181}, {28, 2931, 1, 7181}, {29, 2932, 1, 7181}, {23, 2924, 1, 7182}, {26, 2925, 1, 7182}, {26, 2927, 1, 7182}, {27, 2926, 1, 7182}, {27, 2930, 1, 7182}, {30, 2928, 1, 7182}, {31, 2929, 1, 7182}, {31, 2931, 1, 7182}, {32, 2932, 1, 7182}, {24, 2924, 1, 7183}, {27, 2925, 1, 7183}, {27, 2927, 1, 7183}, {28, 2926, 1, 7183}, {28, 2930, 1, 7183}, {31, 2928, 1, 7183}, {32, 2929, 1, 7183}, {32, 2931, 1, 7183}, {33, 2932, 1, 7183}, {25, 2924, 1, 7184}, {28, 2925, 1, 7184}, {28, 2927, 1, 7184}, {29, 2926, 1, 7184}, {29, 2930, 1, 7184}, {32, 2928, 1, 7184}, {33, 2929, 1, 7184}, {33, 2931, 1, 7184}, {34, 2932, 1, 7184}, {10, 3085, 1, 7185}, {11, 3086, 2, 7185}, {11, 3091, 1, 7185}, {12, 3087, 2, 7185}, {12, 3097, 1, 7185}, {13, 3088, 1, 7185}, {13, 3092, 2, 7185}, {14, 3089, 2, 7185}, {14, 3093, 2, 7185}, {14, 3098, 2, 7185}, {15, 3090, 1, 7185}, {15, 3099, 2, 7185}, {16, 3094, 1, 7185}, {17, 3095, 2, 7185}, {17, 3100, 1, 7185}, {18, 3096, 1, 7185}, {18, 3101, 2, 7185}, {19, 3102, 1, 7185}, {10, 3091, 1, 7186}, {11, 3092, 2, 7186}, {11, 3103, 1, 7186}, {12, 3093, 2, 7186}, {12, 3109, 1, 7186}, {13, 3094, 1, 7186}, {13, 3104, 2, 7186}, {14, 3095, 2, 7186}, {14, 3105, 2, 7186}, {14, 3110, 2, 7186}, {15, 3096, 1, 7186}, {15, 3111, 2, 7186}, {16, 3106, 1, 7186}, {17, 3107, 2, 7186}, {17, 3112, 1, 7186}, {18, 3108, 1, 7186}, {18, 3113, 2, 7186}, {19, 3114, 1, 7186}, {10, 3097, 1, 7187}, {11, 3098, 2, 7187}, {11, 3109, 1, 7187}, {12, 3099, 2, 7187}, {12, 3115, 1, 7187}, {13, 3100, 1, 7187}, {13, 3110, 2, 7187}, {14, 3101, 2, 7187}, {14, 3111, 2, 7187}, {14, 3116, 2, 7187}, {15, 3102, 1, 7187}, {15, 3117, 2, 7187}, {16, 3112, 1, 7187}, {17, 3113, 2, 7187}, {17, 3118, 1, 7187}, {18, 3114, 1, 7187}, {18, 3119, 2, 7187}, {19, 3120, 1, 7187}, {10, 3121, 1, 7188}, {11, 3122, 2, 7188}, {11, 3131, 1, 7188}, {12, 3123, 2, 7188}, {12, 3141, 1, 7188}, {13, 3124, 1, 7188}, {13, 3132, 2, 7188}, {14, 3125, 2, 7188}, {14, 3133, 2, 7188}, {14, 3142, 2, 7188}, {15, 3126, 1, 7188}, {15, 3143, 2, 7188}, {16, 3134, 1, 7188}, {17, 3135, 2, 7188}, {17, 3144, 1, 7188}, {18, 3136, 1, 7188}, {18, 3145, 2, 7188}, {19, 3146, 1, 7188}, {10, 3122, 1, 7189}, {11, 3124, 2, 7189}, {11, 3132, 1, 7189}, {12, 3125, 2, 7189}, {12, 3142, 1, 7189}, {13, 3127, 1, 7189}, {13, 3134, 2, 7189}, {14, 3128, 2, 7189}, {14, 3135, 2, 7189}, {14, 3144, 2, 7189}, {15, 3129, 1, 7189}, {15, 3145, 2, 7189}, {16, 3137, 1, 7189}, {17, 3138, 2, 7189}, {17, 3147, 1, 7189}, {18, 3139, 1, 7189}, {18, 3148, 2, 7189}, {19, 3149, 1, 7189}, {10, 3123, 1, 7190}, {11, 3125, 2, 7190}, {11, 3133, 1, 7190}, {12, 3126, 2, 7190}, {12, 3143, 1, 7190}, {13, 3128, 1, 7190}, {13, 3135, 2, 7190}, {14, 3129, 2, 7190}, {14, 3136, 2, 7190}, {14, 3145, 2, 7190}, {15, 3130, 1, 7190}, {15, 3146, 2, 7190}, {16, 3138, 1, 7190}, {17, 3139, 2, 7190}, {17, 3148, 1, 7190}, {18, 3140, 1, 7190}, {18, 3149, 2, 7190}, {19, 3150, 1, 7190}, {10, 3131, 1, 7191}, {11, 3132, 2, 7191}, {11, 3151, 1, 7191}, {12, 3133, 2, 7191}, {12, 3161, 1, 7191}, {13, 3134, 1, 7191}, {13, 3152, 2, 7191}, {14, 3135, 2, 7191}, {14, 3153, 2, 7191}, {14, 3162, 2, 7191}, {15, 3136, 1, 7191}, {15, 3163, 2, 7191}, {16, 3154, 1, 7191}, {17, 3155, 2, 7191}, {17, 3164, 1, 7191}, {18, 3156, 1, 7191}, {18, 3165, 2, 7191}, {19, 3166, 1, 7191}, {10, 3132, 1, 7192}, {11, 3134, 2, 7192}, {11, 3152, 1, 7192}, {12, 3135, 2, 7192}, {12, 3162, 1, 7192}, {13, 3137, 1, 7192}, {13, 3154, 2, 7192}, {14, 3138, 2, 7192}, {14, 3155, 2, 7192}, {14, 3164, 2, 7192}, {15, 3139, 1, 7192}, {15, 3165, 2, 7192}, {16, 3157, 1, 7192}, {17, 3158, 2, 7192}, {17, 3167, 1, 7192}, {18, 3159, 1, 7192}, {18, 3168, 2, 7192}, {19, 3169, 1, 7192}, {10, 3133, 1, 7193}, {11, 3135, 2, 7193}, {11, 3153, 1, 7193}, {12, 3136, 2, 7193}, {12, 3163, 1, 7193}, {13, 3138, 1, 7193}, {13, 3155, 2, 7193}, {14, 3139, 2, 7193}, {14, 3156, 2, 7193}, {14, 3165, 2, 7193}, {15, 3140, 1, 7193}, {15, 3166, 2, 7193}, {16, 3158, 1, 7193}, {17, 3159, 2, 7193}, {17, 3168, 1, 7193}, {18, 3160, 1, 7193}, {18, 3169, 2, 7193}, {19, 3170, 1, 7193}, {10, 3141, 1, 7194}, {11, 3142, 2, 7194}, {11, 3161, 1, 7194}, {12, 3143, 2, 7194}, {12, 3171, 1, 7194}, {13, 3144, 1, 7194}, {13, 3162, 2, 7194}, {14, 3145, 2, 7194}, {14, 3163, 2, 7194}, {14, 3172, 2, 7194}, {15, 3146, 1, 7194}, {15, 3173, 2, 7194}, {16, 3164, 1, 7194}, {17, 3165, 2, 7194}, {17, 3174, 1, 7194}, {18, 3166, 1, 7194}, {18, 3175, 2, 7194}, {19, 3176, 1, 7194}, {10, 3142, 1, 7195}, {11, 3144, 2, 7195}, {11, 3162, 1, 7195}, {12, 3145, 2, 7195}, {12, 3172, 1, 7195}, {13, 3147, 1, 7195}, {13, 3164, 2, 7195}, {14, 3148, 2, 7195}, {14, 3165, 2, 7195}, {14, 3174, 2, 7195}, {15, 3149, 1, 7195}, {15, 3175, 2, 7195}, {16, 3167, 1, 7195}, {17, 3168, 2, 7195}, {17, 3177, 1, 7195}, {18, 3169, 1, 7195}, {18, 3178, 2, 7195}, {19, 3179, 1, 7195}, {10, 3143, 1, 7196}, {11, 3145, 2, 7196}, {11, 3163, 1, 7196}, {12, 3146, 2, 7196}, {12, 3173, 1, 7196}, {13, 3148, 1, 7196}, {13, 3165, 2, 7196}, {14, 3149, 2, 7196}, {14, 3166, 2, 7196}, {14, 3175, 2, 7196}, {15, 3150, 1, 7196}, {15, 3176, 2, 7196}, {16, 3168, 1, 7196}, {17, 3169, 2, 7196}, {17, 3178, 1, 7196}, {18, 3170, 1, 7196}, {18,

3179, 2, 7196}, {19, 3180, 1, 7196}, {10, 3193, 1, 7197}, {11, 3194, 2, 7197}, {11, 3199, 1, 7197}, {12, 3195, 2, 7197}, {12, 3205, 1, 7197}, {13, 3196, 1, 7197}, {13, 3200, 2, 7197}, {14, 3197, 2, 7197}, {14, 3201, 2, 7197}, {14, 3206, 2, 7197}, {15, 3198, 1, 7197}, {15, 3207, 2, 7197}, {16, 3202, 1, 7197}, {17, 3203, 2, 7197}, {17, 3208, 1, 7197}, {18, 3204, 1, 7197}, {18, 3209, 2, 7197}, {19, 3210, 1, 7197}, {10, 3211, 1, 7198}, {11, 3212, 2, 7198}, {11, 3221, 1, 7198}, {12, 3213, 2, 7198}, {12, 3231, 1, 7198}, {13, 3214, 1, 7198}, {13, 3222, 2, 7198}, {14, 3215, 2, 7198}, {14, 3223, 2, 7198}, {14, 3232, 2, 7198}, {15, 3216, 1, 7198}, {15, 3233, 2, 7198}, {16, 3224, 1, 7198}, {17, 3225, 2, 7198}, {17, 3234, 1, 7198}, {18, 3226, 1, 7198}, {18, 3235, 2, 7198}, {19, 3236, 1, 7198}, {10, 3212, 1, 7199}, {11, 3214, 2, 7199}, {11, 3222, 1, 7199}, {12, 3215, 2, 7199}, {12, 3232, 1, 7199}, {13, 3217, 1, 7199}, {13, 3224, 2, 7199}, {14, 3218, 2, 7199}, {14, 3225, 2, 7199}, {14, 3234, 2, 7199}, {15, 3219, 1, 7199}, {15, 3235, 2, 7199}, {16, 3227, 1, 7199}, {17, 3228, 2, 7199}, {17, 3237, 1, 7199}, {18, 3229, 1, 7199}, {18, 3238, 2, 7199}, {19, 3239, 1, 7199}, {10, 3213, 1, 7200}, {11, 3215, 2, 7200}, {11, 3223, 1, 7200}, {12, 3216, 2, 7200}, {12, 3233, 1, 7200}, {13, 3218, 1, 7200}, {13, 3225, 2, 7200}, {14, 3219, 2, 7200}, {14, 3226, 2, 7200}, {14, 3235, 2, 7200}, {15, 3220, 1, 7200}, {15, 3236, 2, 7200}, {16, 3228, 1, 7200}, {17, 3229, 2, 7200}, {17, 3238, 1, 7200}, {18, 3230, 1, 7200}, {18, 3239, 2, 7200}, {19, 3240, 1, 7200}, {10, 3316, 1, 7201}, {11, 3317, 2, 7201}, {11, 3322, 1, 7201}, {12, 3318, 2, 7201}, {12, 3328, 1, 7201}, {13, 3319, 1, 7201}, {13, 3323, 2, 7201}, {14, 3320, 2, 7201}, {14, 3324, 2, 7201}, {14, 3329, 2, 7201}, {15, 3321, 1, 7201}, {15, 3330, 2, 7201}, {16, 3325, 1, 7201}, {17, 3326, 2, 7201}, {17, 3331, 1, 7201}, {18, 3327, 1, 7201}, {18, 3332, 2, 7201}, {19, 3333, 1, 7201}, {10, 3322, 1, 7202}, {11, 3323, 2, 7202}, {11, 3334, 1, 7202}, {12, 3324, 2, 7202}, {12, 3340, 1, 7202}, {13, 3325, 1, 7202}, {13, 3335, 2, 7202}, {14, 3326, 2, 7202}, {14, 3336, 2, 7202}, {14, 3341, 2, 7202}, {15, 3327, 1, 7202}, {15, 3342, 2, 7202}, {16, 3337, 1, 7202}, {17, 3338, 2, 7202}, {17, 3343, 1, 7202}, {18, 3339, 1, 7202}, {18, 3344, 2, 7202}, {19, 3345, 1, 7202}, {10, 3328, 1, 7203}, {11, 3329, 2, 7203}, {11, 3340, 1, 7203}, {12, 3330, 2, 7203}, {12, 3346, 1, 7203}, {13, 3331, 1, 7203}, {13, 3341, 2, 7203}, {14, 3332, 2, 7203}, {14, 3342, 2, 7203}, {14, 3347, 2, 7203}, {15, 3333, 1, 7203}, {15, 3348, 2, 7203}, {16, 3343, 1, 7203}, {17, 3344, 2, 7203}, {17, 3349, 1, 7203}, {18, 3345, 1, 7203}, {18, 3350, 2, 7203}, {19, 3351, 1, 7203}, {10, 3334, 1, 7204}, {11, 3335, 2, 7204}, {11, 3352, 1, 7204}, {12, 3336, 2, 7204}, {12, 3358, 1, 7204}, {13, 3337, 1, 7204}, {13, 3353, 2, 7204}, {14, 3338, 2, 7204}, {14, 3354, 2, 7204}, {14, 3359, 2, 7204}, {15, 3339, 1, 7204}, {15, 3360, 2, 7204}, {16, 3355, 1, 7204}, {17, 3356, 2, 7204}, {17, 3361, 1, 7204}, {18, 3357, 1, 7204}, {18, 3362, 2, 7204}, {19, 3363, 1, 7204}, {10, 3340, 1, 7205}, {11, 3341, 2, 7205}, {11, 3358, 1, 7205}, {12, 3342, 2, 7205}, {12, 3364, 1, 7205}, {13, 3343, 1, 7205}, {13, 3359, 2, 7205}, {14, 3344, 2, 7205}, {14, 3360, 2, 7205}, {14, 3365, 2, 7205}, {15, 3345, 1, 7205}, {15, 3366, 2, 7205}, {16, 3361, 1, 7205}, {17, 3362, 2, 7205}, {17, 3367, 1, 7205}, {18, 3363, 1, 7205}, {18, 3368, 2, 7205}, {19, 3369, 1, 7205}, {10, 3346, 1, 7206}, {11, 3347, 2, 7206}, {11, 3364, 1, 7206}, {12, 3348, 2, 7206}, {12, 3370, 1, 7206}, {13, 3349, 1, 7206}, {13, 3365, 2, 7206}, {14, 3350, 2, 7206}, {14, 3366, 2, 7206}, {14, 3371, 2, 7206}, {15, 3351, 1, 7206}, {15, 3372, 2, 7206}, {16, 3367, 1, 7206}, {17, 3368, 2, 7206}, {17, 3373, 1, 7206}, {18, 3369, 1, 7206}, {18, 3374, 2, 7206}, {19, 3375, 1, 7206}, {10, 3400, 1, 7207}, {11, 3401, 2, 7207}, {11, 3406, 1, 7207}, {12, 3402, 2, 7207}, {12, 3412, 1, 7207}, {13, 3403, 1, 7207}, {13, 3407, 2, 7207}, {14, 3404, 2, 7207}, {14, 3408, 2, 7207}, {14, 3413, 2, 7207}, {15, 3405, 1, 7207}, {15, 3414, 2, 7207}, {16, 3409, 1, 7207}, {17, 3410, 2, 7207}, {17, 3415, 1, 7207}, {18, 3411, 1, 7207}, {18, 3416, 2, 7207}, {19, 3417, 1, 7207}, {10, 3406, 1, 7208}, {11, 3407, 2, 7208}, {11, 3418, 1, 7208}, {12, 3408, 2, 7208}, {12, 3424, 1, 7208}, {13, 3409, 1, 7208}, {13, 3419, 2, 7208}, {14, 3410, 2, 7208}, {14, 3420, 2, 7208}, {14, 3425, 2, 7208}, {15, 3411, 1, 7208}, {15, 3426, 2, 7208}, {16, 3421, 1, 7208}, {17, 3422, 2, 7208}, {17, 3427, 1, 7208}, {18, 3423, 1, 7208}, {18, 3428, 2, 7208}, {19, 3429, 1, 7208}, {10, 3412, 1, 7209}, {11, 3413, 2, 7209}, {11, 3424, 1, 7209}, {12, 3414, 2, 7209}, {12, 3430, 1, 7209}, {13, 3415, 1, 7209}, {13, 3425, 2, 7209}, {14, 3416, 2, 7209}, {14, 3426, 2, 7209}, {14, 3431, 2, 7209}, {15, 3417, 1, 7209}, {15, 3432, 2, 7209}, {16, 3427, 1, 7209}, {17, 3428, 2, 7209}, {17, 3433, 1, 7209}, {18, 3429, 1, 7209}, {18, 3434, 2, 7209}, {19, 3435, 1, 7209}, {10, 3508, 1, 7210}, {11, 3509, 2, 7210}, {11, 3514, 1, 7210}, {12, 3510, 2, 7210}, {12, 3520, 1, 7210}, {13, 3511, 1, 7210}, {13, 3515, 2, 7210}, {14, 3512, 2, 7210}, {14, 3516, 2, 7210}, {14, 3521, 2, 7210}, {15, 3513, 1, 7210}, {15, 3522, 2, 7210}, {16, 3517, 1, 7210}, {17, 3518, 2, 7210}, {17, 3523, 1, 7210}, {18, 3519, 1, 7210}, {18, 3524, 2, 7210}, {19, 3525, 1, 7210}, {10, 3808, 1, 7211}, {11, 3809, 2, 7211}, {11, 3814, 1, 7211}, {12, 3810, 2, 7211}, {12, 3820, 1, 7211}, {13, 3811, 1, 7211}, {13, 3815, 2, 7211}, {14, 3812, 2, 7211}, {14, 3816, 2, 7211}, {14, 3821, 2, 7211}, {15, 3813, 1, 7211}, {15, 3822, 2, 7211}, {16, 3817, 1, 7211}, {17, 3818, 2, 7211}, {17, 3823, 1, 7211}, {18, 3819, 1, 7211}, {18, 3824, 2, 7211}, {19, 3825, 1, 7211}, {287, 827, 1, 7212}, {288, 828, 1, 7212}, {289, 829, 1, 7212}, {287, 828, 1, 7213}, {288, 830, 1, 7213}, {289, 831, 1, 7213}, {287, 829, 1, 7214}, {288, 831, 1, 7214}, {289, 832, 1, 7214}, {290, 827, 1, 7215}, {291, 828, 1, 7215}, {292, 829, 1, 7215}, {291, 827, 1, 7216}, {293, 828, 1, 7216}, {294, 829, 1, 7216}, {292, 827, 1, 7217}, {294, 828, 1, 7217}, {295, 829, 1, 7217}, {290, 828, 1, 7218}, {291, 830, 1, 7218}, {292, 831, 1, 7218}, {291, 828,

1, 7219}, {293, 830, 1, 7219}, {294, 831, 1, 7219}, {292, 828, 1, 7220}, {294, 830, 1, 7220}, {295, 831, 1, 7220}, {290, 829, 1, 7221}, {291, 831, 1, 7221}, {292, 832, 1, 7221}, {291, 829, 1, 7222}, {293, 831, 1, 7222}, {294, 832, 1, 7222}, {292, 829, 1, 7223}, {294, 831, 1, 7223}, {295, 832, 1, 7223}, {10, 3061, 1, 7224}, {11, 3062, 2, 7224}, {12, 3063, 2, 7224}, {13, 3064, 1, 7224}, {14, 3065, 2, 7224}, {15, 3066, 1, 7224}, {11, 3061, 1, 7225}, {13, 3062, 2, 7225}, {14, 3063, 2, 7225}, {16, 3064, 1, 7225}, {17, 3065, 2, 7225}, {18, 3066, 1, 7225}, {12, 3061, 1, 7226}, {14, 3062, 2, 7226}, {15, 3063, 2, 7226}, {17, 3064, 1, 7226}, {18, 3065, 2, 7226}, {19, 3066, 1, 7226}, {10, 3067, 1, 7227}, {11, 3070, 2, 7227}, {12, 3073, 2, 7227}, {13, 3076, 1, 7227}, {14, 3079, 2, 7227}, {15, 3082, 1, 7227}, {10, 3068, 1, 7228}, {11, 3071, 2, 7228}, {12, 3074, 2, 7228}, {13, 3077, 1, 7228}, {14, 3080, 2, 7228}, {15, 3083, 1, 7228}, {10, 3069, 1, 7229}, {11, 3072, 2, 7229}, {12, 3075, 2, 7229}, {13, 3078, 1, 7229}, {14, 3081, 2, 7229}, {15, 3084, 1, 7229}, {11, 3067, 1, 7230}, {13, 3070, 2, 7230}, {14, 3073, 2, 7230}, {16, 3076, 1, 7230}, {17, 3079, 2, 7230}, {18, 3082, 1, 7230}, {11, 3068, 1, 7231}, {13, 3071, 2, 7231}, {14, 3074, 2, 7231}, {16, 3077, 1, 7231}, {17, 3080, 2, 7231}, {18, 3083, 1, 7231}, {11, 3069, 1, 7232}, {13, 3072, 2, 7232}, {14, 3075, 2, 7232}, {16, 3078, 1, 7232}, {17, 3081, 2, 7232}, {18, 3084, 1, 7232}, {12, 3067, 1, 7233}, {14, 3070, 2, 7233}, {15, 3073, 2, 7233}, {17, 3076, 1, 7233}, {18, 3079, 2, 7233}, {19, 3082, 1, 7233}, {12, 3068, 1, 7234}, {14, 3071, 2, 7234}, {15, 3074, 2, 7234}, {17, 3077, 1, 7234}, {18, 3080, 2, 7234}, {19, 3083, 1, 7234}, {12, 3069, 1, 7235}, {14, 3072, 2, 7235}, {15, 3075, 2, 7235}, {17, 3078, 1, 7235}, {18, 3081, 2, 7235}, {19, 3084, 1, 7235}, {10, 3085, 1, 7236}, {11, 3091, 2, 7236}, {12, 3097, 2, 7236}, {13, 3103, 1, 7236}, {14, 3109, 2, 7236}, {15, 3115, 1, 7236}, {10, 3086, 1, 7237}, {11, 3092, 2, 7237}, {12, 3098, 2, 7237}, {13, 3104, 1, 7237}, {14, 3110, 2, 7237}, {15, 3116, 1, 7237}, {10, 3087, 1, 7238}, {11, 3093, 2, 7238}, {12, 3099, 2, 7238}, {13, 3105, 1, 7238}, {14, 3111, 2, 7238}, {15, 3117, 1, 7238}, {10, 3088, 1, 7239}, {11, 3094, 2, 7239}, {12, 3100, 2, 7239}, {13, 3106, 1, 7239}, {14, 3112, 2, 7239}, {15, 3118, 1, 7239}, {10, 3089, 1, 7240}, {11, 3095, 2, 7240}, {12, 3101, 2, 7240}, {13, 3107, 1, 7240}, {14, 3113, 2, 7240}, {15, 3119, 1, 7240}, {10, 3090, 1, 7241}, {11, 3096, 2, 7241}, {12, 3102, 2, 7241}, {13, 3108, 1, 7241}, {14, 3114, 2, 7241}, {15, 3120, 1, 7241}, {11, 3085, 1, 7242}, {13, 3091, 2, 7242}, {14, 3097, 2, 7242}, {16, 3103, 1, 7242}, {17, 3109, 2, 7242}, {18, 3115, 1, 7242}, {11, 3086, 1, 7243}, {13, 3092, 2, 7243}, {14, 3098, 2, 7243}, {16, 3104, 1, 7243}, {17, 3110, 2, 7243}, {18, 3116, 1, 7243}, {11, 3087, 1, 7244}, {13, 3093, 2, 7244}, {14, 3099, 2, 7244}, {16, 3105, 1, 7244}, {17, 3111, 2, 7244}, {18, 3117, 1, 7244}, {11, 3088, 1, 7245}, {13, 3094, 2, 7245}, {14, 3100, 2, 7245}, {16, 3106, 1, 7245}, {17, 3112, 2, 7245}, {18, 3118, 1, 7245}, {11, 3089, 1, 7246}, {13, 3095, 2, 7246}, {14, 3101, 2, 7246}, {16, 3107, 1, 7246}, {17, 3113, 2, 7246}, {18, 3119, 1, 7246}, {11, 3090, 1, 7247}, {13, 3096, 2, 7247}, {14, 3102, 2, 7247}, {16, 3108, 1, 7247}, {17, 3114, 2, 7247}, {18, 3120, 1, 7247}, {12, 3085, 1, 7248}, {14, 3091, 2, 7248}, {15, 3097, 2, 7248}, {17, 3103, 1, 7248}, {18, 3109, 2, 7248}, {19, 3115, 1, 7248}, {12, 3086, 1, 7249}, {14, 3092, 2, 7249}, {15, 3098, 2, 7249}, {17, 3104, 1, 7249}, {18, 3110, 2, 7249}, {19, 3116, 1, 7249}, {12, 3087, 1, 7250}, {14, 3093, 2, 7250}, {15, 3099, 2, 7250}, {17, 3105, 1, 7250}, {18, 3111, 2, 7250}, {19, 3117, 1, 7250}, {12, 3088, 1, 7251}, {14, 3094, 2, 7251}, {15, 3100, 2, 7251}, {17, 3106, 1, 7251}, {18, 3112, 2, 7251}, {19, 3118, 1, 7251}, {12, 3089, 1, 7252}, {14, 3095, 2, 7252}, {15, 3101, 2, 7252}, {17, 3107, 1, 7252}, {18, 3113, 2, 7252}, {19, 3119, 1, 7252}, {12, 3090, 1, 7253}, {14, 3096, 2, 7253}, {15, 3102, 2, 7253}, {17, 3108, 1, 7253}, {18, 3114, 2, 7253}, {19, 3120, 1, 7253}, {10, 3276, 1, 7254}, {11, 3277, 2, 7254}, {12, 3278, 2, 7254}, {13, 3279, 1, 7254}, {14, 3280, 2, 7254}, {15, 3281, 1, 7254}, {10, 3277, 1, 7255}, {11, 3279, 2, 7255}, {12, 3280, 2, 7255}, {13, 3282, 1, 7255}, {14, 3283, 2, 7255}, {15, 3284, 1, 7255}, {10, 3278, 1, 7256}, {11, 3280, 2, 7256}, {12, 3281, 2, 7256}, {13, 3283, 1, 7256}, {14, 3284, 2, 7256}, {15, 3285, 1, 7256}, {11, 3276, 1, 7257}, {13, 3277, 2, 7257}, {14, 3278, 2, 7257}, {16, 3279, 1, 7257}, {17, 3280, 2, 7257}, {18, 3281, 1, 7257}, {11, 3277, 1, 7258}, {13, 3279, 2, 7258}, {14, 3280, 2, 7258}, {16, 3282, 1, 7258}, {17, 3283, 2, 7258}, {18, 3284, 1, 7258}, {11, 3278, 1, 7259}, {13, 3280, 2, 7259}, {14, 3281, 2, 7259}, {16, 3283, 1, 7259}, {17, 3284, 2, 7259}, {18, 3285, 1, 7259}, {12, 3276, 1, 7260}, {14, 3277, 2, 7260}, {15, 3278, 2, 7260}, {17, 3279, 1, 7260}, {18, 3280, 2, 7260}, {19, 3281, 1, 7260}, {12, 3277, 1, 7261}, {14, 3279, 2, 7261}, {15, 3280, 2, 7261}, {17, 3282, 1, 7261}, {18, 3283, 2, 7261}, {19, 3284, 1, 7261}, {12, 3278, 1, 7262}, {14, 3280, 2, 7262}, {15, 3281, 2, 7262}, {17, 3283, 1, 7262}, {18, 3284, 2, 7262}, {19, 3285, 1, 7262}, {10, 3286, 1, 7263}, {11, 3289, 2, 7263}, {12, 3292, 2, 7263}, {13, 3295, 1, 7263}, {14, 3298, 2, 7263}, {15, 3301, 1, 7263}, {10, 3287, 1, 7264}, {11, 3290, 2, 7264}, {12, 3293, 2, 7264}, {13, 3296, 1, 7264}, {14, 3299, 2, 7264}, {15, 3302, 1, 7264}, {10, 3288, 1, 7265}, {11, 3291, 2, 7265}, {12, 3294, 2, 7265}, {13, 3297, 1, 7265}, {14, 3300, 2, 7265}, {15, 3303, 1, 7265}, {10, 3289, 1, 7266}, {11, 3295, 2, 7266}, {12, 3298, 2, 7266}, {13, 3304, 1, 7266}, {14, 3307, 2, 7266}, {15, 3310, 1, 7266}, {10, 3290, 1, 7267}, {11, 3296, 2, 7267}, {12, 3299, 2, 7267}, {13, 3305, 1, 7267}, {14, 3308, 2, 7267}, {15, 3311, 1, 7267}, {10, 3291, 1, 7268}, {11, 3297, 2, 7268}, {12, 3300, 2, 7268}, {13, 3306, 1, 7268}, {14, 3309, 2, 7268}, {15, 3312, 1, 7268}, {10, 3292, 1, 7269}, {11, 3298, 2, 7269}, {12, 3301, 2, 7269}, {13, 3307, 1, 7269}, {14, 3310, 2, 7269}, {15, 3313, 1, 7269}, {10, 3293, 1, 7270}, {11, 3299, 2, 7270}, {12, 3302, 2,

7270}, {13, 3308, 1, 7270}, {14, 3311, 2, 7270}, {15, 3314, 1, 7270}, {10, 3294, 1, 7271}, {11, 3300, 2, 7271}, {12, 3303, 2, 7271}, {13, 3309, 1, 7271}, {14, 3312, 2, 7271}, {15, 3315, 1, 7271}, {11, 3286, 1, 7272}, {13, 3289, 2, 7272}, {14, 3292, 2, 7272}, {16, 3295, 1, 7272}, {17, 3298, 2, 7272}, {18, 3301, 1, 7272}, {11, 3287, 1, 7273}, {13, 3290, 2, 7273}, {14, 3293, 2, 7273}, {16, 3296, 1, 7273}, {17, 3299, 2, 7273}, {18, 3302, 1, 7273}, {11, 3288, 1, 7274}, {13, 3291, 2, 7274}, {14, 3294, 2, 7274}, {16, 3297, 1, 7274}, {17, 3300, 2, 7274}, {18, 3303, 1, 7274}, {11, 3289, 1, 7275}, {13, 3295, 2, 7275}, {14, 3298, 2, 7275}, {16, 3304, 1, 7275}, {17, 3307, 2, 7275}, {18, 3310, 1, 7275}, {11, 3290, 1, 7276}, {13, 3296, 2, 7276}, {14, 3299, 2, 7276}, {16, 3305, 1, 7276}, {17, 3308, 2, 7276}, {18, 3311, 1, 7276}, {11, 3291, 1, 7277}, {13, 3297, 2, 7277}, {14, 3300, 2, 7277}, {16, 3306, 1, 7277}, {17, 3309, 2, 7277}, {18, 3312, 1, 7277}, {11, 3292, 1, 7278}, {13, 3298, 2, 7278}, {14, 3301, 2, 7278}, {16, 3307, 1, 7278}, {17, 3310, 2, 7278}, {18, 3313, 1, 7278}, {11, 3293, 1, 7279}, {13, 3299, 2, 7279}, {14, 3302, 2, 7279}, {16, 3308, 1, 7279}, {17, 3311, 2, 7279}, {18, 3314, 1, 7279}, {11, 3294, 1, 7280}, {13, 3300, 2, 7280}, {14, 3303, 2, 7280}, {16, 3309, 1, 7280}, {17, 3312, 2, 7280}, {18, 3315, 1, 7280}, {12, 3286, 1, 7281}, {14, 3289, 2, 7281}, {15, 3292, 2, 7281}, {17, 3295, 1, 7281}, {18, 3298, 2, 7281}, {19, 3301, 1, 7281}, {12, 3287, 1, 7282}, {14, 3290, 2, 7282}, {15, 3293, 2, 7282}, {17, 3296, 1, 7282}, {18, 3299, 2, 7282}, {19, 3302, 1, 7282}, {12, 3288, 1, 7283}, {14, 3291, 2, 7283}, {15, 3294, 2, 7283}, {17, 3297, 1, 7283}, {18, 3300, 2, 7283}, {19, 3303, 1, 7283}, {12, 3289, 1, 7284}, {14, 3295, 2, 7284}, {15, 3298, 2, 7284}, {17, 3304, 1, 7284}, {18, 3307, 2, 7284}, {19, 3310, 1, 7284}, {12, 3290, 1, 7285}, {14, 3296, 2, 7285}, {15, 3299, 2, 7285}, {17, 3305, 1, 7285}, {18, 3308, 2, 7285}, {19, 3311, 1, 7285}, {12, 3291, 1, 7286}, {14, 3297, 2, 7286}, {15, 3300, 2, 7286}, {17, 3306, 1, 7286}, {18, 3309, 2, 7286}, {19, 3312, 1, 7286}, {12, 3292, 1, 7287}, {14, 3298, 2, 7287}, {15, 3301, 2, 7287}, {17, 3307, 1, 7287}, {18, 3310, 2, 7287}, {19, 3313, 1, 7287}, {12, 3293, 1, 7288}, {14, 3299, 2, 7288}, {15, 3302, 2, 7288}, {17, 3308, 1, 7288}, {18, 3311, 2, 7288}, {19, 3314, 1, 7288}, {12, 3294, 1, 7289}, {14, 3300, 2, 7289}, {15, 3303, 2, 7289}, {17, 3309, 1, 7289}, {18, 3312, 2, 7289}, {19, 3315, 1, 7289}, {10, 3376, 1, 7290}, {11, 3377, 2, 7290}, {12, 3378, 2, 7290}, {13, 3379, 1, 7290}, {14, 3380, 2, 7290}, {15, 3381, 1, 7290}, {11, 3376, 1, 7291}, {13, 3377, 2, 7291}, {14, 3378, 2, 7291}, {16, 3379, 1, 7291}, {17, 3380, 2, 7291}, {18, 3381, 1, 7291}, {12, 3376, 1, 7292}, {14, 3377, 2, 7292}, {15, 3378, 2, 7292}, {17, 3379, 1, 7292}, {18, 3380, 2, 7292}, {19, 3381, 1, 7292}, {10, 3382, 1, 7293}, {11, 3385, 2, 7293}, {12, 3388, 2, 7293}, {13, 3391, 1, 7293}, {14, 3394, 2, 7293}, {15, 3397, 1, 7293}, {10, 3383, 1, 7294}, {11, 3386, 2, 7294}, {12, 3389, 2, 7294}, {13, 3392, 1, 7294}, {14, 3395, 2, 7294}, {15, 3398, 1, 7294}, {10, 3384, 1, 7295}, {11, 3387, 2, 7295}, {12, 3390, 2, 7295}, {13, 3393, 1, 7295}, {14, 3396, 2, 7295}, {15, 3399, 1, 7295}, {11, 3382, 1, 7296}, {13, 3385, 2, 7296}, {14, 3388, 2, 7296}, {16, 3391, 1, 7296}, {17, 3394, 2, 7296}, {18, 3397, 1, 7296}, {11, 3383, 1, 7297}, {13, 3386, 2, 7297}, {14, 3389, 2, 7297}, {16, 3392, 1, 7297}, {17, 3395, 2, 7297}, {18, 3398, 1, 7297}, {11, 3384, 1, 7298}, {13, 3387, 2, 7298}, {14, 3390, 2, 7298}, {16, 3393, 1, 7298}, {17, 3396, 2, 7298}, {18, 3399, 1, 7298}, {12, 3382, 1, 7299}, {14, 3385, 2, 7299}, {15, 3388, 2, 7299}, {17, 3391, 1, 7299}, {18, 3394, 2, 7299}, {19, 3397, 1, 7299}, {12, 3383, 1, 7300}, {14, 3386, 2, 7300}, {15, 3389, 2, 7300}, {17, 3392, 1, 7300}, {18, 3395, 2, 7300}, {19, 3398, 1, 7300}, {12, 3384, 1, 7301}, {14, 3387, 2, 7301}, {15, 3390, 2, 7301}, {17, 3393, 1, 7301}, {18, 3396, 2, 7301}, {19, 3399, 1, 7301}, {10, 3636, 1, 7302}, {11, 3637, 2, 7302}, {12, 3638, 2, 7302}, {13, 3639, 1, 7302}, {14, 3640, 2, 7302}, {15, 3641, 1, 7302}, {10, 3637, 1, 7303}, {11, 3639, 2, 7303}, {12, 3640, 2, 7303}, {13, 3642, 1, 7303}, {14, 3643, 2, 7303}, {15, 3644, 1, 7303}, {10, 3638, 1, 7304}, {11, 3640, 2, 7304}, {12, 3641, 2, 7304}, {13, 3643, 1, 7304}, {14, 3644, 2, 7304}, {15, 3645, 1, 7304}, {11, 3636, 1, 7305}, {13, 3637, 2, 7305}, {14, 3638, 2, 7305}, {16, 3639, 1, 7305}, {17, 3640, 2, 7305}, {18, 3641, 1, 7305}, {11, 3637, 1, 7306}, {13, 3639, 2, 7306}, {14, 3640, 2, 7306}, {16, 3642, 1, 7306}, {17, 3643, 2, 7306}, {18, 3644, 1, 7306}, {11, 3638, 1, 7307}, {13, 3640, 2, 7307}, {14, 3641, 2, 7307}, {16, 3643, 1, 7307}, {17, 3644, 2, 7307}, {18, 3645, 1, 7307}, {12, 3636, 1, 7308}, {14, 3637, 2, 7308}, {15, 3638, 2, 7308}, {17, 3639, 1, 7308}, {18, 3640, 2, 7308}, {19, 3641, 1, 7308}, {12, 3637, 1, 7309}, {14, 3639, 2, 7309}, {15, 3640, 2, 7309}, {17, 3642, 1, 7309}, {18, 3643, 2, 7309}, {19, 3644, 1, 7309}, {12, 3638, 1, 7310}, {14, 3640, 2, 7310}, {15, 3641, 2, 7310}, {17, 3643, 1, 7310}, {18, 3644, 2, 7310}, {19, 3645, 1, 7310}, {10, 3736, 1, 7311}, {11, 3737, 2, 7311}, {12, 3738, 2, 7311}, {13, 3739, 1, 7311}, {14, 3740, 2, 7311}, {15, 3741, 1, 7311}, {11, 3736, 1, 7312}, {13, 3737, 2, 7312}, {14, 3738, 2, 7312}, {16, 3739, 1, 7312}, {17, 3740, 2, 7312}, {18, 3741, 1, 7312}, {12, 3736, 1, 7313}, {14, 3737, 2, 7313}, {15, 3738, 2, 7313}, {17, 3739, 1, 7313}, {18, 3740, 2, 7313}, {19, 3741, 1, 7313}, {290, 827, 1, 7314}, {291, 828, 2, 7314}, {292, 829, 2, 7314}, {293, 830, 1, 7314}, {294, 831, 2, 7314}, {295, 832, 1, 7314}, {296, 827, 1, 7315}, {297, 828, 2, 7315}, {298, 829, 2, 7315}, {299, 830, 1, 7315}, {300, 831, 2, 7315}, {301, 832, 1, 7315}, {297, 827, 1, 7316}, {299, 828, 2, 7316}, {300, 829, 2, 7316}, {302, 830, 1, 7316}, {303, 831, 2, 7316}, {304, 832, 1, 7316}, {298, 827, 1, 7317}, {300, 828, 2, 7317}, {301, 829, 2, 7317}, {303, 830, 1, 7317}, {304, 831, 2, 7317}, {305, 832, 1, 7317}, {306, 827, 1, 7318}, {307, 828, 2, 7318}, {308, 829, 2, 7318}, {309, 830, 1, 7318}, {310, 831, 2, 7318}, {311, 832, 1, 7318}, {307, 827, 1, 7319}, {309, 828, 2, 7319},

{310, 829, 2, 7319}, {312, 830, 1, 7319}, {313, 831, 2, 7319}, {314, 832, 1, 7319}, {308, 827, 1, 7320}, {310, 828, 2, 7320}, {311, 829, 2, 7320}, {313, 830, 1, 7320}, {314, 831, 2, 7320}, {315, 832, 1, 7320}, {309, 827, 1, 7321}, {312, 828, 2, 7321}, {313, 829, 2, 7321}, {316, 830, 1, 7321}, {317, 831, 2, 7321}, {318, 832, 1, 7321}, {310, 827, 1, 7322}, {313, 828, 2, 7322}, {314, 829, 2, 7322}, {317, 830, 1, 7322}, {318, 831, 2, 7322}, {319, 832, 1, 7322}, {311, 827, 1, 7323}, {314, 828, 2, 7323}, {315, 829, 2, 7323}, {318, 830, 1, 7323}, {319, 831, 2, 7323}, {320, 832, 1, 7323}, {290, 833, 1, 7324}, {291, 834, 1, 7324}, {291, 839, 1, 7324}, {292, 835, 1, 7324}, {292, 845, 1, 7324}, {293, 840, 1, 7324}, {294, 841, 1, 7324}, {294, 846, 1, 7324}, {295, 847, 1, 7324}, {290, 834, 1, 7325}, {291, 836, 1, 7325}, {291, 840, 1, 7325}, {292, 837, 1, 7325}, {292, 846, 1, 7325}, {293, 842, 1, 7325}, {294, 843, 1, 7325}, {294, 848, 1, 7325}, {295, 849, 1, 7325}, {290, 835, 1, 7326}, {291, 837, 1, 7326}, {291, 841, 1, 7326}, {292, 838, 1, 7326}, {292, 847, 1, 7326}, {293, 843, 1, 7326}, {294, 844, 1, 7326}, {294, 849, 1, 7326}, {295, 850, 1, 7326}, {296, 833, 1, 7327}, {297, 834, 1, 7327}, {297, 839, 1, 7327}, {298, 835, 1, 7327}, {298, 845, 1, 7327}, {299, 840, 1, 7327}, {300, 841, 1, 7327}, {300, 846, 1, 7327}, {301, 847, 1, 7327}, {297, 833, 1, 7328}, {299, 834, 1, 7328}, {299, 839, 1, 7328}, {300, 835, 1, 7328}, {300, 845, 1, 7328}, {302, 840, 1, 7328}, {303, 841, 1, 7328}, {303, 846, 1, 7328}, {304, 847, 1, 7328}, {298, 833, 1, 7329}, {300, 834, 1, 7329}, {300, 839, 1, 7329}, {301, 835, 1, 7329}, {301, 845, 1, 7329}, {303, 840, 1, 7329}, {304, 841, 1, 7329}, {304, 846, 1, 7329}, {305, 847, 1, 7329}, {296, 834, 1, 7330}, {297, 836, 1, 7330}, {297, 840, 1, 7330}, {298, 837, 1, 7330}, {298, 846, 1, 7330}, {299, 842, 1, 7330}, {300, 843, 1, 7330}, {300, 848, 1, 7330}, {301, 849, 1, 7330}, {297, 834, 1, 7331}, {299, 836, 1, 7331}, {299, 840, 1, 7331}, {300, 837, 1, 7331}, {300, 846, 1, 7331}, {302, 842, 1, 7331}, {303, 843, 1, 7331}, {303, 848, 1, 7331}, {304, 849, 1, 7331}, {298, 834, 1, 7332}, {300, 836, 1, 7332}, {300, 840, 1, 7332}, {301, 837, 1, 7332}, {301, 846, 1, 7332}, {303, 842, 1, 7332}, {304, 843, 1, 7332}, {304, 848, 1, 7332}, {305, 849, 1, 7332}, {296, 835, 1, 7333}, {297, 837, 1, 7333}, {297, 841, 1, 7333}, {298, 838, 1, 7333}, {298, 847, 1, 7333}, {299, 843, 1, 7333}, {300, 844, 1, 7333}, {300, 849, 1, 7333}, {301, 850, 1, 7333}, {297, 835, 1, 7334}, {299, 837, 1, 7334}, {299, 841, 1, 7334}, {300, 838, 1, 7334}, {300, 847, 1, 7334}, {302, 843, 1, 7334}, {303, 844, 1, 7334}, {303, 849, 1, 7334}, {304, 850, 1, 7334}, {298, 835, 1, 7335}, {300, 837, 1, 7335}, {300, 841, 1, 7335}, {301, 838, 1, 7335}, {301, 847, 1, 7335}, {303, 843, 1, 7335}, {304, 844, 1, 7335}, {304, 849, 1, 7335}, {305, 850, 1, 7335}, {306, 833, 1, 7336}, {307, 834, 1, 7336}, {307, 839, 1, 7336}, {308, 835, 1, 7336}, {308, 845, 1, 7336}, {309, 840, 1, 7336}, {310, 841, 1, 7336}, {310, 846, 1, 7336}, {311, 847, 1, 7336}, {307, 833, 1, 7337}, {309, 834, 1, 7337}, {309, 839, 1, 7337}, {310, 835, 1, 7337}, {310, 845, 1, 7337}, {312, 840, 1, 7337}, {313, 841, 1, 7337}, {313, 846, 1, 7337}, {314, 847, 1, 7337}, {308, 833, 1, 7338}, {310, 834, 1, 7338}, {310, 839, 1, 7338}, {311, 835, 1, 7338}, {311, 845, 1, 7338}, {313, 840, 1, 7338}, {314, 841, 1, 7338}, {314, 846, 1, 7338}, {315, 847, 1, 7338}, {309, 833, 1, 7339}, {312, 834, 1, 7339}, {312, 839, 1, 7339}, {313, 835, 1, 7339}, {313, 845, 1, 7339}, {316, 840, 1, 7339}, {317, 841, 1, 7339}, {317, 846, 1, 7339}, {318, 847, 1, 7339}, {310, 833, 1, 7340}, {313, 834, 1, 7340}, {313, 839, 1, 7340}, {314, 835, 1, 7340}, {314, 845, 1, 7340}, {317, 840, 1, 7340}, {318, 841, 1, 7340}, {318, 846, 1, 7340}, {319, 847, 1, 7340}, {311, 833, 1, 7341}, {314, 834, 1, 7341}, {314, 839, 1, 7341}, {315, 835, 1, 7341}, {315, 845, 1, 7341}, {318, 840, 1, 7341}, {319, 841, 1, 7341}, {319, 846, 1, 7341}, {320, 847, 1, 7341}, {306, 834, 1, 7342}, {307, 836, 1, 7342}, {307, 840, 1, 7342}, {308, 837, 1, 7342}, {308, 846, 1, 7342}, {309, 842, 1, 7342}, {310, 843, 1, 7342}, {310, 848, 1, 7342}, {311, 849, 1, 7342}, {307, 834, 1, 7343}, {309, 836, 1, 7343}, {309, 840, 1, 7343}, {310, 837, 1, 7343}, {310, 846, 1, 7343}, {312, 842, 1, 7343}, {313, 843, 1, 7343}, {313, 848, 1, 7343}, {314, 849, 1, 7343}, {308, 834, 1, 7344}, {310, 836, 1, 7344}, {310, 840, 1, 7344}, {311, 837, 1, 7344}, {311, 846, 1, 7344}, {313, 842, 1, 7344}, {314, 843, 1, 7344}, {314, 848, 1, 7344}, {315, 849, 1, 7344}, {309, 834, 1, 7345}, {312, 836, 1, 7345}, {312, 840, 1, 7345}, {313, 837, 1, 7345}, {313, 846, 1, 7345}, {316, 842, 1, 7345}, {317, 843, 1, 7345}, {317, 848, 1, 7345}, {318, 849, 1, 7345}, {310, 834, 1, 7346}, {313, 836, 1, 7346}, {313, 840, 1, 7346}, {314, 837, 1, 7346}, {314, 846, 1, 7346}, {317, 842, 1, 7346}, {318, 843, 1, 7346}, {318, 848, 1, 7346}, {319, 849, 1, 7346}, {311, 834, 1, 7347}, {314, 836, 1, 7347}, {314, 840, 1, 7347}, {315, 837, 1, 7347}, {315, 846, 1, 7347}, {318, 842, 1, 7347}, {319, 843, 1, 7347}, {319, 848, 1, 7347}, {320, 849, 1, 7347}, {306, 835, 1, 7348}, {307, 837, 1, 7348}, {307, 841, 1, 7348}, {308, 838, 1, 7348}, {308, 847, 1, 7348}, {309, 843, 1, 7348}, {310, 844, 1, 7348}, {310, 849, 1, 7348}, {311, 850, 1, 7348}, {307, 835, 1, 7349}, {309, 837, 1, 7349}, {309, 841, 1, 7349}, {310, 838, 1, 7349}, {310, 847, 1, 7349}, {312, 843, 1, 7349}, {313, 844, 1, 7349}, {313, 849, 1, 7349}, {314, 850, 1, 7349}, {308, 835, 1, 7350}, {310, 837, 1, 7350}, {310, 841, 1, 7350}, {311, 838, 1, 7350}, {311, 847, 1, 7350}, {313, 843, 1, 7350}, {314, 844, 1, 7350}, {314, 849, 1, 7350}, {315, 850, 1, 7350}, {309, 835, 1, 7351}, {312, 837, 1, 7351}, {312, 841, 1, 7351}, {313, 838, 1, 7351}, {313, 847, 1, 7351}, {316, 843, 1, 7351}, {317, 844, 1, 7351}, {317, 849, 1, 7351}, {318, 850, 1, 7351}, {310, 835, 1, 7352}, {313, 837, 1, 7352}, {313, 841, 1, 7352}, {314, 838, 1, 7352}, {314, 847, 1, 7352}, {317, 843, 1, 7352}, {318, 844, 1, 7352}, {318, 849, 1, 7352}, {319, 850, 1, 7352}, {311, 835, 1, 7353}, {314, 837, 1, 7353}, {314, 841, 1, 7353}, {315, 838, 1, 7353}, {315,

847, 1, 7353}, {318, 843, 1, 7353}, {319, 844, 1, 7353}, {319, 849, 1, 7353}, {320, 850, 1, 7353}, {10, 3067, 1, 7354}, {11, 3068, 1, 7354}, {11, 3070, 2, 7354}, {12, 3069, 1, 7354}, {12, 3073, 2, 7354}, {13, 3071, 2, 7354}, {13, 3076, 1, 7354}, {14, 3072, 2, 7354}, {14, 3074, 2, 7354}, {14, 3079, 2, 7354}, {15, 3075, 2, 7354}, {15, 3082, 1, 7354}, {16, 3077, 1, 7354}, {17, 3078, 1, 7354}, {17, 3080, 2, 7354}, {18, 3081, 2, 7354}, {18, 3083, 1, 7354}, {19, 3084, 1, 7354}, {10, 3085, 1, 7355}, {11, 3086, 1, 7355}, {11, 3091, 2, 7355}, {12, 3087, 1, 7355}, {12, 3097, 2, 7355}, {13, 3092, 2, 7355}, {13, 3103, 1, 7355}, {14, 3093, 2, 7355}, {14, 3098, 2, 7355}, {14, 3109, 2, 7355}, {15, 3099, 2, 7355}, {15, 3115, 1, 7355}, {16, 3104, 1, 7355}, {17, 3105, 1, 7355}, {17, 3110, 2, 7355}, {18, 3111, 2, 7355}, {18, 3116, 1, 7355}, {19, 3117, 1, 7355}, {10, 3086, 1, 7356}, {11, 3088, 1, 7356}, {11, 3092, 2, 7356}, {12, 3089, 1, 7356}, {12, 3098, 2, 7356}, {13, 3094, 2, 7356}, {13, 3104, 1, 7356}, {14, 3095, 2, 7356}, {14, 3100, 2, 7356}, {14, 3110, 2, 7356}, {15, 3101, 2, 7356}, {15, 3116, 1, 7356}, {16, 3106, 1, 7356}, {17, 3107, 1, 7356}, {17, 3112, 2, 7356}, {18, 3113, 2, 7356}, {18, 3118, 1, 7356}, {19, 3119, 1, 7356}, {10, 3087, 1, 7357}, {11, 3089, 1, 7357}, {11, 3093, 2, 7357}, {12, 3090, 1, 7357}, {12, 3099, 2, 7357}, {13, 3095, 2, 7357}, {13, 3105, 1, 7357}, {14, 3096, 2, 7357}, {14, 3101, 2, 7357}, {14, 3111, 2, 7357}, {15, 3102, 2, 7357}, {15, 3117, 1, 7357}, {16, 3107, 1, 7357}, {17, 3108, 1, 7357}, {17, 3113, 2, 7357}, {18, 3114, 2, 7357}, {18, 3119, 1, 7357}, {19, 3120, 1, 7357}, {10, 3121, 1, 7358}, {11, 3122, 1, 7358}, {11, 3131, 2, 7358}, {12, 3123, 1, 7358}, {12, 3141, 2, 7358}, {13, 3132, 2, 7358}, {13, 3151, 1, 7358}, {14, 3133, 2, 7358}, {14, 3142, 2, 7358}, {14, 3161, 2, 7358}, {15, 3143, 2, 7358}, {15, 3171, 1, 7358}, {16, 3152, 1, 7358}, {17, 3153, 1, 7358}, {17, 3162, 2, 7358}, {18, 3163, 2, 7358}, {18, 3172, 1, 7358}, {19, 3173, 1, 7358}, {10, 3122, 1, 7359}, {11, 3124, 1, 7359}, {11, 3132, 2, 7359}, {12, 3125, 1, 7359}, {12, 3142, 2, 7359}, {13, 3134, 2, 7359}, {13, 3152, 1, 7359}, {14, 3135, 2, 7359}, {14, 3144, 2, 7359}, {14, 3162, 2, 7359}, {15, 3145, 2, 7359}, {15, 3172, 1, 7359}, {16, 3154, 1, 7359}, {17, 3155, 1, 7359}, {17, 3164, 2, 7359}, {18, 3165, 2, 7359}, {18, 3174, 1, 7359}, {19, 3175, 1, 7359}, {10, 3123, 1, 7360}, {11, 3125, 1, 7360}, {11, 3133, 2, 7360}, {12, 3126, 1, 7360}, {12, 3143, 2, 7360}, {13, 3135, 2, 7360}, {13, 3153, 1, 7360}, {14, 3136, 2, 7360}, {14, 3145, 2, 7360}, {14, 3163, 2, 7360}, {15, 3146, 2, 7360}, {15, 3173, 1, 7360}, {16, 3155, 1, 7360}, {17, 3156, 1, 7360}, {17, 3165, 2, 7360}, {18, 3166, 2, 7360}, {18, 3175, 1, 7360}, {19, 3176, 1, 7360}, {10, 3124, 1, 7361}, {11, 3127, 1, 7361}, {11, 3134, 2, 7361}, {12, 3128, 1, 7361}, {12, 3144, 2, 7361}, {13, 3137, 2, 7361}, {13, 3154, 1, 7361}, {14, 3138, 2, 7361}, {14, 3147, 2, 7361}, {14, 3164, 2, 7361}, {15, 3148, 2, 7361}, {15, 3174, 1, 7361}, {16, 3157, 1, 7361}, {17, 3158, 1, 7361}, {17, 3167, 2, 7361}, {18, 3168, 2, 7361}, {18, 3177, 1, 7361}, {19, 3178, 1, 7361}, {10, 3125, 1, 7362}, {11, 3128, 1, 7362}, {11, 3135, 2, 7362}, {12, 3129, 1, 7362}, {12, 3145, 2, 7362}, {13, 3138, 2, 7362}, {13, 3155, 1, 7362}, {14, 3139, 2, 7362}, {14, 3148, 2, 7362}, {14, 3165, 2, 7362}, {15, 3149, 2, 7362}, {15, 3175, 1, 7362}, {16, 3158, 1, 7362}, {17, 3159, 1, 7362}, {17, 3168, 2, 7362}, {18, 3169, 2, 7362}, {18, 3178, 1, 7362}, {19, 3179, 1, 7362}, {10, 3126, 1, 7363}, {11, 3129, 1, 7363}, {11, 3136, 2, 7363}, {12, 3130, 1, 7363}, {12, 3146, 2, 7363}, {13, 3139, 2, 7363}, {13, 3156, 1, 7363}, {14, 3140, 2, 7363}, {14, 3149, 2, 7363}, {14, 3166, 2, 7363}, {15, 3150, 2, 7363}, {15, 3176, 1, 7363}, {16, 3159, 1, 7363}, {17, 3160, 1, 7363}, {17, 3169, 2, 7363}, {18, 3170, 2, 7363}, {18, 3179, 1, 7363}, {19, 3180, 1, 7363}, {290, 851, 1, 7364}, {291, 852, 1, 7364}, {291, 861, 1, 7364}, {292, 853, 1, 7364}, {292, 871, 1, 7364}, {293, 862, 1, 7364}, {294, 863, 1, 7364}, {294, 872, 1, 7364}, {295, 873, 1, 7364}, {290, 852, 1, 7365}, {291, 854, 1, 7365}, {291, 862, 1, 7365}, {292, 855, 1, 7365}, {292, 872, 1, 7365}, {293, 864, 1, 7365}, {294, 865, 1, 7365}, {294, 874, 1, 7365}, {295, 875, 1, 7365}, {290, 853, 1, 7366}, {291, 855, 1, 7366}, {291, 863, 1, 7366}, {292, 856, 1, 7366}, {292, 873, 1, 7366}, {293, 865, 1, 7366}, {294, 866, 1, 7366}, {294, 875, 1, 7366}, {295, 876, 1, 7366}, {290, 854, 1, 7367}, {291, 857, 1, 7367}, {291, 864, 1, 7367}, {292, 858, 1, 7367}, {292, 874, 1, 7367}, {293, 867, 1, 7367}, {294, 868, 1, 7367}, {294, 877, 1, 7367}, {295, 878, 1, 7367}, {290, 855, 1, 7368}, {291, 858, 1, 7368}, {291, 865, 1, 7368}, {292, 859, 1, 7368}, {292, 875, 1, 7368}, {293, 868, 1, 7368}, {294, 869, 1, 7368}, {294, 878, 1, 7368}, {295, 879, 1, 7368}, {290, 856, 1, 7369}, {291, 859, 1, 7369}, {291, 866, 1, 7369}, {292, 860, 1, 7369}, {292, 876, 1, 7369}, {293, 869, 1, 7369}, {294, 870, 1, 7369}, {294, 879, 1, 7369}, {295, 880, 1, 7369}, {296, 851, 1, 7370}, {297, 852, 1, 7370}, {297, 861, 1, 7370}, {298, 853, 1, 7370}, {298, 871, 1, 7370}, {299, 862, 1, 7370}, {300, 863, 1, 7370}, {300, 872, 1, 7370}, {301, 873, 1, 7370}, {297, 851, 1, 7371}, {299, 852, 1, 7371}, {299, 861, 1, 7371}, {300, 853, 1, 7371}, {300, 871, 1, 7371}, {302, 862, 1, 7371}, {303, 863, 1, 7371}, {303, 872, 1, 7371}, {304, 873, 1, 7371}, {298, 851, 1, 7372}, {300, 852, 1, 7372}, {300, 861, 1, 7372}, {301, 853, 1, 7372}, {301, 871, 1, 7372}, {303, 862, 1, 7372}, {304, 863, 1, 7372}, {304, 872, 1, 7372}, {305, 873, 1, 7372}, {296, 852, 1, 7373}, {297, 854, 1, 7373}, {297, 862, 1, 7373}, {298, 855, 1, 7373}, {298, 872, 1, 7373}, {299, 864, 1, 7373}, {300, 865, 1, 7373}, {300, 874, 1, 7373}, {301, 875, 1, 7373}, {297, 852, 1, 7374}, {299, 854, 1, 7374}, {299, 862, 1, 7374}, {300, 855, 1, 7374}, {300, 872, 1, 7374}, {302, 864, 1, 7374}, {303, 865, 1, 7374}, {303, 874, 1, 7374}, {304, 875, 1, 7374}, {298, 852, 1, 7375}, {300, 854, 1, 7375}, {300, 862, 1, 7375}, {301, 855, 1, 7375}, {301, 872, 1, 7375}, {303, 864, 1, 7375}, {304, 865, 1, 7375}, {304, 874, 1, 7375}, {305, 875, 1, 7375}, {296, 853,

1, 7376}, {297, 855, 1, 7376}, {297, 863, 1, 7376}, {298, 856, 1, 7376}, {298, 873, 1,
7376}, {299, 865, 1, 7376}, {300, 866, 1, 7376}, {300, 875, 1, 7376}, {301, 876, 1, 7376},
{297, 853, 1, 7377}, {299, 855, 1, 7377}, {299, 863, 1, 7377}, {300, 856, 1, 7377}, {300,
873, 1, 7377}, {302, 865, 1, 7377}, {303, 866, 1, 7377}, {303, 875, 1, 7377}, {304, 876,
1, 7377}, {298, 853, 1, 7378}, {300, 855, 1, 7378}, {300, 863, 1, 7378}, {301, 856, 1,
7378}, {301, 873, 1, 7378}, {303, 865, 1, 7378}, {304, 866, 1, 7378}, {304, 875, 1, 7378},
{305, 876, 1, 7378}, {296, 854, 1, 7379}, {297, 857, 1, 7379}, {297, 864, 1, 7379}, {298,
858, 1, 7379}, {298, 874, 1, 7379}, {299, 867, 1, 7379}, {300, 868, 1, 7379}, {300, 877,
1, 7379}, {301, 878, 1, 7379}, {297, 854, 1, 7380}, {299, 857, 1, 7380}, {299, 864, 1,
7380}, {300, 858, 1, 7380}, {300, 874, 1, 7380}, {302, 867, 1, 7380}, {303, 868, 1, 7380},
{303, 877, 1, 7380}, {304, 878, 1, 7380}, {298, 854, 1, 7381}, {300, 857, 1, 7381}, {300,
864, 1, 7381}, {301, 858, 1, 7381}, {301, 874, 1, 7381}, {303, 867, 1, 7381}, {304, 868,
1, 7381}, {304, 877, 1, 7381}, {305, 878, 1, 7381}, {296, 855, 1, 7382}, {297, 858, 1,
7382}, {297, 865, 1, 7382}, {298, 859, 1, 7382}, {298, 875, 1, 7382}, {299, 868, 1, 7382},
{300, 869, 1, 7382}, {300, 878, 1, 7382}, {301, 879, 1, 7382}, {297, 855, 1, 7383}, {299,
858, 1, 7383}, {299, 865, 1, 7383}, {300, 859, 1, 7383}, {300, 875, 1, 7383}, {302, 868,
1, 7383}, {303, 869, 1, 7383}, {303, 878, 1, 7383}, {304, 879, 1, 7383}, {298, 855, 1,
7384}, {300, 858, 1, 7384}, {300, 865, 1, 7384}, {301, 859, 1, 7384}, {301, 875, 1, 7384},
{303, 868, 1, 7384}, {304, 869, 1, 7384}, {304, 878, 1, 7384}, {305, 879, 1, 7384}, {296,
856, 1, 7385}, {297, 859, 1, 7385}, {297, 866, 1, 7385}, {298, 860, 1, 7385}, {298, 876,
1, 7385}, {299, 869, 1, 7385}, {300, 870, 1, 7385}, {300, 879, 1, 7385}, {301, 880, 1,
7385}, {297, 856, 1, 7386}, {299, 859, 1, 7386}, {299, 866, 1, 7386}, {300, 860, 1, 7386},
{300, 876, 1, 7386}, {302, 869, 1, 7386}, {303, 870, 1, 7386}, {303, 879, 1, 7386}, {304,
880, 1, 7386}, {298, 856, 1, 7387}, {300, 859, 1, 7387}, {300, 866, 1, 7387}, {301, 860,
1, 7387}, {301, 876, 1, 7387}, {303, 869, 1, 7387}, {304, 870, 1, 7387}, {304, 879, 1,
7387}, {305, 880, 1, 7387}, {10, 3286, 1, 7388}, {11, 3287, 1, 7388}, {11, 3289, 2, 7388},
{12, 3288, 1, 7388}, {12, 3292, 2, 7388}, {13, 3290, 2, 7388}, {13, 3295, 1, 7388}, {14,
3291, 2, 7388}, {14, 3293, 2, 7388}, {14, 3298, 2, 7388}, {15, 3294, 2, 7388}, {15, 3301,
1, 7388}, {16, 3296, 1, 7388}, {17, 3297, 1, 7388}, {17, 3299, 2, 7388}, {18, 3300, 2,
7388}, {18, 3302, 1, 7388}, {19, 3303, 1, 7388}, {10, 3289, 1, 7389}, {11, 3290, 1, 7389},
{11, 3295, 2, 7389}, {12, 3291, 1, 7389}, {12, 3298, 2, 7389}, {13, 3296, 2, 7389}, {13,
3304, 1, 7389}, {14, 3297, 2, 7389}, {14, 3299, 2, 7389}, {14, 3307, 2, 7389}, {15, 3300,
2, 7389}, {15, 3310, 1, 7389}, {16, 3305, 1, 7389}, {17, 3306, 1, 7389}, {17, 3308, 2,
7389}, {18, 3309, 2, 7389}, {18, 3311, 1, 7389}, {19, 3312, 1, 7389}, {10, 3292, 1, 7390},
{11, 3293, 1, 7390}, {11, 3298, 2, 7390}, {12, 3294, 1, 7390}, {12, 3301, 2, 7390}, {13,
3299, 2, 7390}, {13, 3307, 1, 7390}, {14, 3300, 2, 7390}, {14, 3302, 2, 7390}, {14, 3310,
2, 7390}, {15, 3303, 2, 7390}, {15, 3313, 1, 7390}, {16, 3308, 1, 7390}, {17, 3309, 1,
7390}, {17, 3311, 2, 7390}, {18, 3312, 2, 7390}, {18, 3314, 1, 7390}, {19, 3315, 1, 7390},
{10, 3316, 1, 7391}, {11, 3317, 1, 7391}, {11, 3322, 2, 7391}, {12, 3318, 1, 7391}, {12,
3328, 2, 7391}, {13, 3323, 2, 7391}, {13, 3334, 1, 7391}, {14, 3324, 2, 7391}, {14, 3329,
2, 7391}, {14, 3340, 2, 7391}, {15, 3330, 2, 7391}, {15, 3346, 1, 7391}, {16, 3335, 1,
7391}, {17, 3336, 1, 7391}, {17, 3341, 2, 7391}, {18, 3342, 2, 7391}, {18, 3347, 1, 7391},
{19, 3348, 1, 7391}, {10, 3317, 1, 7392}, {11, 3319, 1, 7392}, {11, 3323, 2, 7392}, {12,
3320, 1, 7392}, {12, 3329, 2, 7392}, {13, 3325, 2, 7392}, {13, 3335, 1, 7392}, {14, 3326,
2, 7392}, {14, 3331, 2, 7392}, {14, 3341, 2, 7392}, {15, 3332, 2, 7392}, {15, 3347, 1,
7392}, {16, 3337, 1, 7392}, {17, 3338, 1, 7392}, {17, 3343, 2, 7392}, {18, 3344, 2, 7392},
{18, 3349, 1, 7392}, {19, 3350, 1, 7392}, {10, 3318, 1, 7393}, {11, 3320, 1, 7393}, {11,
3324, 2, 7393}, {12, 3321, 1, 7393}, {12, 3330, 2, 7393}, {13, 3326, 2, 7393}, {13, 3336,
1, 7393}, {14, 3327, 2, 7393}, {14, 3332, 2, 7393}, {14, 3342, 2, 7393}, {15, 3333, 2,
7393}, {15, 3348, 1, 7393}, {16, 3338, 1, 7393}, {17, 3339, 1, 7393}, {17, 3344, 2, 7393},
{18, 3345, 2, 7393}, {18, 3350, 1, 7393}, {19, 3351, 1, 7393}, {10, 3322, 1, 7394}, {11,
3323, 1, 7394}, {11, 3334, 2, 7394}, {12, 3324, 1, 7394}, {12, 3340, 2, 7394}, {13, 3335,
2, 7394}, {13, 3352, 1, 7394}, {14, 3336, 2, 7394}, {14, 3341, 2, 7394}, {14, 3358, 2,
7394}, {15, 3342, 2, 7394}, {15, 3364, 1, 7394}, {16, 3353, 1, 7394}, {17, 3354, 1, 7394},
{17, 3359, 2, 7394}, {18, 3360, 2, 7394}, {18, 3365, 1, 7394}, {19, 3366, 1, 7394}, {10,
3323, 1, 7395}, {11, 3325, 1, 7395}, {11, 3335, 2, 7395}, {12, 3326, 1, 7395}, {12, 3341,
2, 7395}, {13, 3337, 2, 7395}, {13, 3353, 1, 7395}, {14, 3338, 2, 7395}, {14, 3343, 2,
7395}, {14, 3359, 2, 7395}, {15, 3344, 2, 7395}, {15, 3365, 1, 7395}, {16, 3355, 1, 7395},
{17, 3356, 1, 7395}, {17, 3361, 2, 7395}, {18, 3362, 2, 7395}, {18, 3367, 1, 7395}, {19,
3368, 1, 7395}, {10, 3324, 1, 7396}, {11, 3326, 1, 7396}, {11, 3336, 2, 7396}, {12, 3327,
1, 7396}, {12, 3342, 2, 7396}, {13, 3338, 2, 7396}, {13, 3354, 1, 7396}, {14, 3339, 2,
7396}, {14, 3344, 2, 7396}, {14, 3360, 2, 7396}, {15, 3345, 2, 7396}, {15, 3366, 1, 7396},
{16, 3356, 1, 7396}, {17, 3357, 1, 7396}, {17, 3362, 2, 7396}, {18, 3363, 2, 7396}, {18,
3368, 1, 7396}, {19, 3369, 1, 7396}, {10, 3328, 1, 7397}, {11, 3329, 1, 7397}, {11, 3340,
2, 7397}, {12, 3330, 1, 7397}, {12, 3346, 2, 7397}, {13, 3341, 2, 7397}, {13, 3358, 1,
7397}, {14, 3342, 2, 7397}, {14, 3347, 2, 7397}, {14, 3364, 2, 7397}, {15, 3348, 2, 7397},
{15, 3370, 1, 7397}, {16, 3359, 1, 7397}, {17, 3360, 1, 7397}, {17, 3365, 2, 7397}, {18,
3366, 2, 7397}, {18, 3371, 1, 7397}, {19, 3372, 1, 7397}, {10, 3329, 1, 7398}, {11, 3331,
1, 7398}, {11, 3341, 2, 7398}, {12, 3332, 1, 7398}, {12, 3347, 2, 7398}, {13, 3343, 2,

7398}, {13, 3359, 1, 7398}, {14, 3344, 2, 7398}, {14, 3349, 2, 7398}, {14, 3365, 2, 7398}, {15, 3350, 2, 7398}, {15, 3371, 1, 7398}, {16, 3361, 1, 7398}, {17, 3362, 1, 7398}, {17, 3367, 2, 7398}, {18, 3368, 2, 7398}, {18, 3373, 1, 7398}, {19, 3374, 1, 7398}, {10, 3330, 1, 7399}, {11, 3332, 1, 7399}, {11, 3342, 2, 7399}, {12, 3333, 1, 7399}, {12, 3348, 2, 7399}, {13, 3344, 2, 7399}, {13, 3360, 1, 7399}, {14, 3345, 2, 7399}, {14, 3350, 2, 7399}, {14, 3366, 2, 7399}, {15, 3351, 2, 7399}, {15, 3372, 1, 7399}, {16, 3362, 1, 7399}, {17, 3363, 1, 7399}, {17, 3368, 2, 7399}, {18, 3369, 2, 7399}, {18, 3374, 1, 7399}, {19, 3375, 1, 7399}, {10, 3382, 1, 7400}, {11, 3383, 1, 7400}, {11, 3385, 2, 7400}, {12, 3384, 1, 7400}, {12, 3388, 2, 7400}, {13, 3386, 2, 7400}, {13, 3391, 1, 7400}, {14, 3387, 2, 7400}, {14, 3389, 2, 7400}, {14, 3394, 2, 7400}, {15, 3390, 2, 7400}, {15, 3397, 1, 7400}, {16, 3392, 1, 7400}, {17, 3393, 1, 7400}, {17, 3395, 2, 7400}, {18, 3396, 2, 7400}, {18, 3398, 1, 7400}, {19, 3399, 1, 7400}, {10, 3400, 1, 7401}, {11, 3401, 1, 7401}, {11, 3406, 2, 7401}, {12, 3402, 1, 7401}, {12, 3412, 2, 7401}, {13, 3407, 2, 7401}, {13, 3418, 1, 7401}, {14, 3408, 2, 7401}, {14, 3413, 2, 7401}, {14, 3424, 2, 7401}, {15, 3414, 2, 7401}, {15, 3430, 1, 7401}, {16, 3419, 1, 7401}, {17, 3420, 1, 7401}, {17, 3425, 2, 7401}, {18, 3426, 2, 7401}, {18, 3431, 1, 7401}, {19, 3432, 1, 7401}, {10, 3401, 1, 7402}, {11, 3403, 1, 7402}, {11, 3407, 2, 7402}, {12, 3404, 1, 7402}, {12, 3413, 2, 7402}, {13, 3409, 2, 7402}, {13, 3419, 1, 7402}, {14, 3410, 2, 7402}, {14, 3415, 2, 7402}, {14, 3425, 2, 7402}, {15, 3416, 2, 7402}, {15, 3431, 1, 7402}, {16, 3421, 1, 7402}, {17, 3422, 1, 7402}, {17, 3427, 2, 7402}, {18, 3428, 2, 7402}, {18, 3433, 1, 7402}, {19, 3434, 1, 7402}, {10, 3402, 1, 7403}, {11, 3404, 1, 7403}, {11, 3408, 2, 7403}, {12, 3405, 1, 7403}, {12, 3414, 2, 7403}, {13, 3410, 2, 7403}, {13, 3420, 1, 7403}, {14, 3411, 2, 7403}, {14, 3416, 2, 7403}, {14, 3426, 2, 7403}, {15, 3417, 2, 7403}, {15, 3432, 1, 7403}, {16, 3422, 1, 7403}, {17, 3423, 1, 7403}, {17, 3428, 2, 7403}, {18, 3429, 2, 7403}, {18, 3434, 1, 7403}, {19, 3435, 1, 7403}, {10, 3591, 1, 7404}, {11, 3592, 1, 7404}, {11, 3594, 2, 7404}, {12, 3593, 1, 7404}, {12, 3597, 2, 7404}, {13, 3595, 2, 7404}, {13, 3600, 1, 7404}, {14, 3596, 2, 7404}, {14, 3598, 2, 7404}, {14, 3603, 2, 7404}, {15, 3599, 2, 7404}, {15, 3606, 1, 7404}, {16, 3601, 1, 7404}, {17, 3602, 1, 7404}, {17, 3604, 2, 7404}, {18, 3605, 2, 7404}, {18, 3607, 1, 7404}, {19, 3608, 1, 7404}, {10, 3594, 1, 7405}, {11, 3595, 1, 7405}, {11, 3600, 2, 7405}, {12, 3596, 1, 7405}, {12, 3603, 2, 7405}, {13, 3601, 2, 7405}, {13, 3609, 1, 7405}, {14, 3602, 2, 7405}, {14, 3604, 2, 7405}, {14, 3612, 2, 7405}, {15, 3605, 2, 7405}, {15, 3615, 1, 7405}, {16, 3610, 1, 7405}, {17, 3611, 1, 7405}, {17, 3613, 2, 7405}, {18, 3614, 2, 7405}, {18, 3616, 1, 7405}, {19, 3617, 1, 7405}, {10, 3597, 1, 7406}, {11, 3598, 1, 7406}, {11, 3603, 2, 7406}, {12, 3599, 1, 7406}, {12, 3606, 2, 7406}, {13, 3604, 2, 7406}, {13, 3612, 1, 7406}, {14, 3605, 2, 7406}, {14, 3607, 2, 7406}, {14, 3615, 2, 7406}, {15, 3608, 2, 7406}, {15, 3618, 1, 7406}, {16, 3613, 1, 7406}, {17, 3614, 1, 7406}, {17, 3616, 2, 7406}, {18, 3617, 2, 7406}, {18, 3619, 1, 7406}, {19, 3620, 1, 7406}, {10, 3600, 1, 7407}, {11, 3601, 1, 7407}, {11, 3609, 2, 7407}, {12, 3602, 1, 7407}, {12, 3612, 2, 7407}, {13, 3610, 2, 7407}, {13, 3621, 1, 7407}, {14, 3611, 2, 7407}, {14, 3613, 2, 7407}, {14, 3624, 2, 7407}, {15, 3614, 2, 7407}, {15, 3627, 1, 7407}, {16, 3622, 1, 7407}, {17, 3623, 1, 7407}, {17, 3625, 2, 7407}, {18, 3626, 2, 7407}, {18, 3628, 1, 7407}, {19, 3629, 1, 7407}, {10, 3603, 1, 7408}, {11, 3604, 1, 7408}, {11, 3612, 2, 7408}, {12, 3605, 1, 7408}, {12, 3615, 2, 7408}, {13, 3613, 2, 7408}, {13, 3624, 1, 7408}, {14, 3614, 2, 7408}, {14, 3616, 2, 7408}, {14, 3627, 2, 7408}, {15, 3617, 2, 7408}, {15, 3630, 1, 7408}, {16, 3625, 1, 7408}, {17, 3626, 1, 7408}, {17, 3628, 2, 7408}, {18, 3629, 2, 7408}, {18, 3631, 1, 7408}, {19, 3632, 1, 7408}, {10, 3606, 1, 7409}, {11, 3607, 1, 7409}, {11, 3615, 2, 7409}, {12, 3608, 1, 7409}, {12, 3618, 2, 7409}, {13, 3616, 2, 7409}, {13, 3627, 1, 7409}, {14, 3617, 2, 7409}, {14, 3619, 2, 7409}, {14, 3630, 2, 7409}, {15, 3620, 2, 7409}, {15, 3633, 1, 7409}, {16, 3628, 1, 7409}, {17, 3629, 1, 7409}, {17, 3631, 2, 7409}, {18, 3632, 2, 7409}, {18, 3634, 1, 7409}, {19, 3635, 1, 7409}, {10, 3646, 1, 7410}, {11, 3647, 1, 7410}, {11, 3649, 2, 7410}, {12, 3648, 1, 7410}, {12, 3652, 2, 7410}, {13, 3650, 2, 7410}, {13, 3655, 1, 7410}, {14, 3651, 2, 7410}, {14, 3653, 2, 7410}, {14, 3658, 2, 7410}, {15, 3654, 2, 7410}, {15, 3661, 1, 7410}, {16, 3656, 1, 7410}, {17, 3657, 1, 7410}, {17, 3659, 2, 7410}, {18, 3660, 2, 7410}, {18, 3662, 1, 7410}, {19, 3663, 1, 7410}, {10, 3649, 1, 7411}, {11, 3650, 1, 7411}, {11, 3655, 2, 7411}, {12, 3651, 1, 7411}, {12, 3658, 2, 7411}, {13, 3656, 2, 7411}, {13, 3664, 1, 7411}, {14, 3657, 2, 7411}, {14, 3659, 2, 7411}, {14, 3667, 2, 7411}, {15, 3660, 2, 7411}, {15, 3670, 1, 7411}, {16, 3665, 1, 7411}, {17, 3666, 1, 7411}, {17, 3668, 2, 7411}, {18, 3669, 2, 7411}, {18, 3671, 1, 7411}, {19, 3672, 1, 7411}, {10, 3652, 1, 7412}, {11, 3653, 1, 7412}, {11, 3658, 2, 7412}, {12, 3654, 1, 7412}, {12, 3661, 2, 7412}, {13, 3659, 2, 7412}, {13, 3667, 1, 7412}, {14, 3660, 2, 7412}, {14, 3662, 2, 7412}, {14, 3670, 2, 7412}, {15, 3663, 2, 7412}, {15, 3673, 1, 7412}, {16, 3668, 1, 7412}, {17, 3669, 1, 7412}, {17, 3671, 2, 7412}, {18, 3672, 2, 7412}, {18, 3674, 1, 7412}, {19, 3675, 1, 7412}, {10, 3742, 1, 7413}, {11, 3743, 1, 7413}, {11, 3745, 2, 7413}, {12, 3744, 1, 7413}, {12, 3748, 2, 7413}, {13, 3746, 2, 7413}, {13, 3751, 1, 7413}, {14, 3747, 2, 7413}, {14, 3749, 2, 7413}, {14, 3754, 2, 7413}, {15, 3750, 2, 7413}, {15, 3757, 1, 7413}, {16, 3752, 1, 7413}, {17, 3753, 1, 7413}, {17, 3755, 2, 7413}, {18, 3756, 2, 7413}, {18, 3758, 1, 7413}, {19, 3759, 1, 7413}, {10, 3897, 1, 7414}, {11, 3898, 1, 7414}, {11, 3900, 2, 7414}, {12, 3899, 1, 7414}, {12, 3903, 2, 7414}, {13, 3901, 2, 7414}, {13, 3906, 1, 7414}, {14, 3902, 2, 7414}, {14, 3904, 2, 7414}, {14, 3909, 2, 7414}, {15, 3905, 2, 7414},

{15, 3912, 1, 7414}, {16, 3907, 1, 7414}, {17, 3908, 1, 7414}, {17, 3910, 2, 7414}, {18, 3911, 2, 7414}, {18, 3913, 1, 7414}, {19, 3914, 1, 7414}, {286, 989, 1, 7415}, {287, 990, 1, 7416}, {288, 991, 1, 7416}, {289, 992, 1, 7416}, {290, 990, 1, 7417}, {291, 991, 1, 7417}, {292, 992, 1, 7417}, {291, 990, 1, 7418}, {293, 991, 1, 7418}, {294, 992, 1, 7418}, {292, 990, 1, 7419}, {294, 991, 1, 7419}, {295, 992, 1, 7419}, {287, 993, 1, 7420}, {288, 994, 1, 7420}, {289, 995, 1, 7420}, {287, 994, 1, 7421}, {288, 996, 1, 7421}, {289, 997, 1, 7421}, {287, 995, 1, 7422}, {288, 997, 1, 7422}, {289, 998, 1, 7422}, {290, 993, 1, 7423}, {291, 994, 1, 7423}, {292, 995, 1, 7423}, {291, 993, 1, 7424}, {293, 994, 1, 7424}, {294, 995, 1, 7424}, {292, 993, 1, 7425}, {294, 994, 1, 7425}, {295, 995, 1, 7425}, {290, 994, 1, 7426}, {291, 996, 1, 7426}, {292, 997, 1, 7426}, {291, 994, 1, 7427}, {293, 996, 1, 7427}, {294, 997, 1, 7427}, {292, 994, 1, 7428}, {294, 996, 1, 7428}, {295, 997, 1, 7428}, {290, 995, 1, 7429}, {291, 997, 1, 7429}, {292, 998, 1, 7429}, {291, 995, 1, 7430}, {293, 997, 1, 7430}, {294, 998, 1, 7430}, {292, 995, 1, 7431}, {294, 997, 1, 7431}, {295, 998, 1, 7431}, {290, 993, 1, 7432}, {291, 994, 2, 7432}, {292, 995, 2, 7432}, {293, 996, 1, 7432}, {294, 997, 2, 7432}, {295, 998, 1, 7432}, {296, 993, 1, 7433}, {297, 994, 2, 7433}, {298, 995, 2, 7433}, {299, 996, 1, 7433}, {300, 997, 2, 7433}, {301, 998, 1, 7433}, {297, 993, 1, 7434}, {299, 994, 2, 7434}, {300, 995, 2, 7434}, {302, 996, 1, 7434}, {303, 997, 2, 7434}, {304, 998, 1, 7434}, {298, 993, 1, 7435}, {300, 994, 2, 7435}, {301, 995, 2, 7435}, {303, 996, 1, 7435}, {304, 997, 2, 7435}, {305, 998, 1, 7435}, {306, 993, 1, 7436}, {307, 994, 2, 7436}, {308, 995, 2, 7436}, {309, 996, 1, 7436}, {310, 997, 2, 7436}, {311, 998, 1, 7436}, {307, 993, 1, 7437}, {309, 994, 2, 7437}, {310, 995, 2, 7437}, {312, 996, 1, 7437}, {313, 997, 2, 7437}, {314, 998, 1, 7437}, {308, 993, 1, 7438}, {310, 994, 2, 7438}, {311, 995, 2, 7438}, {313, 996, 1, 7438}, {314, 997, 2, 7438}, {315, 998, 1, 7438}, {309, 993, 1, 7439}, {312, 994, 2, 7439}, {313, 995, 2, 7439}, {316, 996, 1, 7439}, {317, 997, 2, 7439}, {318, 998, 1, 7439}, {310, 993, 1, 7440}, {313, 994, 2, 7440}, {314, 995, 2, 7440}, {317, 996, 1, 7440}, {318, 997, 2, 7440}, {319, 998, 1, 7440}, {311, 993, 1, 7441}, {314, 994, 2, 7441}, {315, 995, 2, 7441}, {318, 996, 1, 7441}, {319, 997, 2, 7441}, {320, 998, 1, 7441}, {290, 999, 1, 7442}, {291, 1000, 2, 7442}, {292, 1001, 2, 7442}, {293, 1002, 1, 7442}, {294, 1003, 2, 7442}, {295, 1004, 1, 7442}, {290, 1000, 1, 7443}, {291, 1002, 2, 7443}, {292, 1003, 2, 7443}, {293, 1005, 1, 7443}, {294, 1006, 2, 7443}, {295, 1007, 1, 7443}, {290, 1001, 1, 7444}, {291, 1003, 2, 7444}, {292, 1004, 2, 7444}, {293, 1006, 1, 7444}, {294, 1007, 2, 7444}, {295, 1008, 1, 7444}, {296, 999, 1, 7445}, {297, 1000, 2, 7445}, {298, 1001, 2, 7445}, {299, 1002, 1, 7445}, {300, 1003, 2, 7445}, {301, 1004, 1, 7445}, {297, 999, 1, 7446}, {299, 1000, 2, 7446}, {300, 1001, 2, 7446}, {302, 1002, 1, 7446}, {303, 1003, 2, 7446}, {304, 1004, 1, 7446}, {298, 999, 1, 7447}, {300, 1000, 2, 7447}, {301, 1001, 2, 7447}, {303, 1002, 1, 7447}, {304, 1003, 2, 7447}, {305, 1004, 1, 7447}, {296, 1000, 1, 7448}, {297, 1002, 2, 7448}, {298, 1003, 2, 7448}, {299, 1005, 1, 7448}, {300, 1006, 2, 7448}, {301, 1007, 1, 7448}, {297, 1000, 1, 7449}, {299, 1002, 2, 7449}, {300, 1003, 2, 7449}, {302, 1005, 1, 7449}, {303, 1006, 2, 7449}, {304, 1007, 1, 7449}, {298, 1000, 1, 7450}, {300, 1002, 2, 7450}, {301, 1003, 2, 7450}, {303, 1005, 1, 7450}, {304, 1006, 2, 7450}, {305, 1007, 1, 7450}, {296, 1001, 1, 7451}, {297, 1003, 2, 7451}, {298, 1004, 2, 7451}, {299, 1006, 1, 7451}, {300, 1007, 2, 7451}, {301, 1008, 1, 7451}, {297, 1001, 1, 7452}, {299, 1003, 2, 7452}, {300, 1004, 2, 7452}, {302, 1006, 1, 7452}, {303, 1007, 2, 7452}, {304, 1008, 1, 7452}, {298, 1001, 1, 7453}, {300, 1003, 2, 7453}, {301, 1004, 2, 7453}, {303, 1006, 1, 7453}, {304, 1007, 2, 7453}, {305, 1008, 1, 7453}, {10, 3636, 1, 7454}, {11, 3637, 3, 7454}, {12, 3638, 3, 7454}, {13, 3639, 3, 7454}, {14, 3640, 6, 7454}, {15, 3641, 3, 7454}, {16, 3642, 1, 7454}, {17, 3643, 3, 7454}, {18, 3644, 3, 7454}, {19, 3645, 1, 7454}, {10, 3646, 1, 7455}, {11, 3649, 3, 7455}, {12, 3652, 3, 7455}, {13, 3655, 3, 7455}, {14, 3658, 6, 7455}, {15, 3661, 3, 7455}, {16, 3664, 1, 7455}, {17, 3667, 3, 7455}, {18, 3670, 3, 7455}, {19, 3673, 1, 7455}, {10, 3647, 1, 7456}, {11, 3650, 3, 7456}, {12, 3653, 3, 7456}, {13, 3656, 3, 7456}, {14, 3659, 6, 7456}, {15, 3662, 3, 7456}, {16, 3665, 1, 7456}, {17, 3668, 3, 7456}, {18, 3671, 3, 7456}, {19, 3674, 1, 7456}, {10, 3648, 1, 7457}, {11, 3651, 3, 7457}, {12, 3654, 3, 7457}, {13, 3657, 3, 7457}, {14, 3660, 6, 7457}, {15, 3663, 3, 7457}, {16, 3666, 1, 7457}, {17, 3669, 3, 7457}, {18, 3672, 3, 7457}, {19, 3675, 1, 7457}, {290, 1009, 1, 7458}, {291, 1010, 2, 7458}, {292, 1011, 2, 7458}, {293, 1012, 1, 7458}, {294, 1013, 2, 7458}, {295, 1014, 1, 7458}, {290, 1010, 1, 7459}, {291, 1012, 2, 7459}, {292, 1013, 2, 7459}, {293, 1015, 1, 7459}, {294, 1016, 2, 7459}, {295, 1017, 1, 7459}, {290, 1011, 1, 7460}, {291, 1013, 2, 7460}, {292, 1014, 2, 7460}, {293, 1016, 1, 7460}, {294, 1017, 2, 7460}, {295, 1018, 1, 7460}, {290, 1012, 1, 7461}, {291, 1015, 2, 7461}, {292, 1016, 2, 7461}, {293, 1019, 1, 7461}, {294, 1020, 2, 7461}, {295, 1021, 1, 7461}, {290, 1013, 1, 7462}, {291, 1016, 2, 7462}, {292, 1017, 2, 7462}, {293, 1020, 1, 7462}, {294, 1021, 2, 7462}, {295, 1022, 1, 7462}, {290, 1014, 1, 7463}, {291, 1017, 2, 7463}, {292, 1018, 2, 7463}, {293, 1021, 1, 7463}, {294, 1022, 2, 7463}, {295, 1023, 1, 7463}, {10, 3836, 1, 7464}, {11, 3837, 3, 7464}, {12, 3838, 3, 7464}, {13, 3839, 3, 7464}, {14, 3840, 6, 7464}, {15, 3841, 3, 7464}, {16, 3842, 1, 7464}, {17, 3843, 3, 7464}, {18, 3844, 3, 7464}, {19, 3845, 1, 7464}, {10, 3837, 1, 7465}, {11, 3839, 3, 7465}, {12, 3840, 3, 7465}, {13, 3842, 3, 7465}, {14, 3843, 6, 7465}, {15, 3844, 3, 7465}, {16, 3846, 1, 7465}, {17, 3847, 3, 7465}, {18, 3848, 3, 7465}, {19, 3849, 1, 7465}, {10, 3838, 1, 7466}, {11, 3840, 3, 7466},

{12, 3841, 3, 7466}, {13, 3843, 3, 7466}, {14, 3844, 6, 7466}, {15, 3845, 3, 7466}, {16, 3847, 1, 7466}, {17, 3848, 3, 7466}, {18, 3849, 3, 7466}, {19, 3850, 1, 7466}, {10, 3851, 1, 7467}, {11, 3852, 3, 7467}, {12, 3853, 3, 7467}, {13, 3854, 3, 7467}, {14, 3855, 6, 7467}, {15, 3856, 3, 7467}, {16, 3857, 1, 7467}, {17, 3858, 3, 7467}, {18, 3859, 3, 7467}, {19, 3860, 1, 7467}, {10, 3946, 1, 7468}, {11, 3947, 3, 7468}, {12, 3948, 3, 7468}, {13, 3949, 3, 7468}, {14, 3950, 6, 7468}, {15, 3951, 3, 7468}, {16, 3952, 1, 7468}, {17, 3953, 3, 7468}, {18, 3954, 3, 7468}, {19, 3955, 1, 7468}, {20, 3261, 1, 7469}, {21, 3262, 4, 7469}, {22, 3263, 4, 7469}, {23, 3264, 6, 7469}, {24, 3265, 12, 7469}, {25, 3266, 6, 7469}, {26, 3267, 4, 7469}, {27, 3268, 12, 7469}, {28, 3269, 12, 7469}, {29, 3270, 4, 7469}, {30, 3271, 1, 7469}, {31, 3272, 4, 7469}, {32, 3273, 6, 7469}, {33, 3274, 4, 7469}, {34, 3275, 1, 7469}, {20, 3067, 1, 7470}, {21, 3068, 1, 7470}, {21, 3070, 1, 7470}, {22, 3069, 1, 7470}, {22, 3073, 1, 7470}, {23, 3071, 1, 7470}, {24, 3072, 1, 7470}, {24, 3074, 1, 7470}, {25, 3075, 1, 7470}, {20, 3070, 1, 7471}, {21, 3071, 1, 7471}, {21, 3076, 1, 7471}, {22, 3072, 1, 7471}, {22, 3079, 1, 7471}, {23, 3077, 1, 7471}, {24, 3078, 1, 7471}, {24, 3080, 1, 7471}, {25, 3081, 1, 7471}, {20, 3073, 1, 7472}, {21, 3074, 1, 7472}, {21, 3079, 1, 7472}, {22, 3075, 1, 7472}, {22, 3082, 1, 7472}, {23, 3080, 1, 7472}, {24, 3081, 1, 7472}, {24, 3083, 1, 7472}, {25, 3084, 1, 7472}, {21, 3067, 1, 7473}, {23, 3068, 1, 7473}, {23, 3070, 1, 7473}, {24, 3069, 1, 7473}, {24, 3073, 1, 7473}, {26, 3071, 1, 7473}, {27, 3072, 1, 7473}, {27, 3074, 1, 7473}, {28, 3075, 1, 7473}, {21, 3070, 1, 7474}, {23, 3071, 1, 7474}, {23, 3076, 1, 7474}, {24, 3072, 1, 7474}, {24, 3079, 1, 7474}, {26, 3077, 1, 7474}, {27, 3078, 1, 7474}, {27, 3080, 1, 7474}, {28, 3081, 1, 7474}, {21, 3073, 1, 7475}, {23, 3074, 1, 7475}, {23, 3079, 1, 7475}, {24, 3075, 1, 7475}, {24, 3082, 1, 7475}, {26, 3080, 1, 7475}, {27, 3081, 1, 7475}, {27, 3083, 1, 7475}, {28, 3084, 1, 7475}, {22, 3067, 1, 7476}, {24, 3068, 1, 7476}, {24, 3070, 1, 7476}, {25, 3069, 1, 7476}, {25, 3073, 1, 7476}, {27, 3071, 1, 7476}, {28, 3072, 1, 7476}, {28, 3074, 1, 7476}, {29, 3075, 1, 7476}, {22, 3070, 1, 7477}, {24, 3071, 1, 7477}, {24, 3076, 1, 7477}, {25, 3072, 1, 7477}, {25, 3079, 1, 7477}, {27, 3077, 1, 7477}, {28, 3078, 1, 7477}, {28, 3080, 1, 7477}, {29, 3081, 1, 7477}, {22, 3073, 1, 7478}, {24, 3074, 1, 7478}, {24, 3079, 1, 7478}, {25, 3075, 1, 7478}, {25, 3082, 1, 7478}, {27, 3080, 1, 7478}, {28, 3081, 1, 7478}, {28, 3083, 1, 7478}, {29, 3084, 1, 7478}, {23, 3067, 1, 7479}, {26, 3068, 1, 7479}, {26, 3070, 1, 7479}, {27, 3069, 1, 7479}, {27, 3073, 1, 7479}, {30, 3071, 1, 7479}, {31, 3072, 1, 7479}, {31, 3074, 1, 7479}, {32, 3075, 1, 7479}, {23, 3070, 1, 7480}, {26, 3071, 1, 7480}, {26, 3076, 1, 7480}, {27, 3072, 1, 7480}, {27, 3079, 1, 7480}, {30, 3077, 1, 7480}, {31, 3078, 1, 7480}, {31, 3080, 1, 7480}, {32, 3081, 1, 7480}, {23, 3073, 1, 7481}, {26, 3074, 1, 7481}, {26, 3079, 1, 7481}, {27, 3075, 1, 7481}, {27, 3082, 1, 7481}, {30, 3080, 1, 7481}, {31, 3081, 1, 7481}, {31, 3083, 1, 7481}, {32, 3084, 1, 7481}, {24, 3067, 1, 7482}, {27, 3068, 1, 7482}, {27, 3070, 1, 7482}, {28, 3069, 1, 7482}, {28, 3073, 1, 7482}, {31, 3071, 1, 7482}, {32, 3072, 1, 7482}, {32, 3074, 1, 7482}, {33, 3075, 1, 7482}, {24, 3070, 1, 7483}, {27, 3071, 1, 7483}, {27, 3076, 1, 7483}, {28, 3072, 1, 7483}, {28, 3079, 1, 7483}, {31, 3077, 1, 7483}, {32, 3078, 1, 7483}, {32, 3080, 1, 7483}, {33, 3081, 1, 7483}, {24, 3073, 1, 7484}, {27, 3074, 1, 7484}, {27, 3079, 1, 7484}, {28, 3075, 1, 7484}, {28, 3082, 1, 7484}, {31, 3080, 1, 7484}, {32, 3081, 1, 7484}, {32, 3083, 1, 7484}, {33, 3084, 1, 7484}, {25, 3067, 1, 7485}, {28, 3068, 1, 7485}, {28, 3070, 1, 7485}, {29, 3069, 1, 7485}, {29, 3073, 1, 7485}, {32, 3071, 1, 7485}, {33, 3072, 1, 7485}, {33, 3074, 1, 7485}, {34, 3075, 1, 7485}, {25, 3070, 1, 7486}, {28, 3071, 1, 7486}, {28, 3076, 1, 7486}, {29, 3072, 1, 7486}, {29, 3079, 1, 7486}, {32, 3077, 1, 7486}, {33, 3078, 1, 7486}, {33, 3080, 1, 7486}, {34, 3081, 1, 7486}, {25, 3073, 1, 7487}, {28, 3074, 1, 7487}, {28, 3079, 1, 7487}, {29, 3075, 1, 7487}, {29, 3082, 1, 7487}, {32, 3080, 1, 7487}, {33, 3081, 1, 7487}, {33, 3083, 1, 7487}, {34, 3084, 1, 7487}, {20, 3184, 1, 7488}, {21, 3185, 1, 7488}, {21, 3187, 1, 7488}, {22, 3186, 1, 7488}, {22, 3190, 1, 7488}, {23, 3188, 1, 7488}, {24, 3189, 1, 7488}, {24, 3191, 1, 7488}, {25, 3192, 1, 7488}, {21, 3184, 1, 7489}, {23, 3185, 1, 7489}, {23, 3187, 1, 7489}, {24, 3186, 1, 7489}, {24, 3190, 1, 7489}, {26, 3188, 1, 7489}, {27, 3189, 1, 7489}, {27, 3191, 1, 7489}, {28, 3192, 1, 7489}, {22, 3184, 1, 7490}, {24, 3185, 1, 7490}, {24, 3187, 1, 7490}, {25, 3186, 1, 7490}, {25, 3190, 1, 7490}, {27, 3188, 1, 7490}, {28, 3189, 1, 7490}, {28, 3191, 1, 7490}, {29, 3192, 1, 7490}, {23, 3184, 1, 7491}, {26, 3185, 1, 7491}, {26, 3187, 1, 7491}, {27, 3186, 1, 7491}, {27, 3190, 1, 7491}, {30, 3188, 1, 7491}, {31, 3189, 1, 7491}, {31, 3191, 1, 7491}, {32, 3192, 1, 7491}, {24, 3184, 1, 7492}, {27, 3185, 1, 7492}, {27, 3187, 1, 7492}, {28, 3186, 1, 7492}, {28, 3190, 1, 7492}, {31, 3188, 1, 7492}, {32, 3189, 1, 7492}, {32, 3191, 1, 7492}, {33, 3192, 1, 7492}, {25, 3184, 1, 7493}, {28, 3185, 1, 7493}, {28, 3187, 1, 7493}, {29, 3186, 1, 7493}, {29, 3190, 1, 7493}, {32, 3188, 1, 7493}, {33, 3189, 1, 7493}, {33, 3191, 1, 7493}, {34, 3192, 1, 7493}, {20, 3085, 1, 7494}, {21, 3086, 2, 7494}, {21, 3091, 1, 7494}, {22, 3087, 2, 7494}, {22, 3097, 1, 7494}, {23, 3088, 1, 7494}, {23, 3092, 2, 7494}, {24, 3089, 2, 7494}, {24, 3093, 2, 7494}, {24, 3098, 2, 7494}, {25, 3090, 1, 7494}, {25, 3099, 2, 7494}, {26, 3094, 1, 7494}, {27, 3095, 2, 7494}, {27, 3100, 1, 7494}, {28, 3096, 1, 7494}, {28, 3101, 2, 7494}, {29, 3102, 1, 7494}, {20, 3091, 1, 7495}, {21, 3092, 2, 7495}, {21, 3103, 1, 7495}, {22, 3093, 2, 7495}, {22, 3109, 1, 7495}, {23, 3094, 1, 7495}, {23, 3104, 2, 7495}, {24, 3095, 2, 7495}, {24, 3105, 2, 7495}, {24, 3110, 2, 7495}, {25, 3096, 1, 7495}, {25, 3111, 2, 7495}, {26, 3106, 1, 7495}, {27, 3107, 2, 7495}, {27, 3112, 1, 7495}, {28, 3108, 1,

7495}, {28, 3113, 2, 7495}, {29, 3114, 1, 7495}, {20, 3097, 1, 7496}, {21, 3098, 2, 7496}, {21, 3109, 1, 7496}, {22, 3099, 2, 7496}, {22, 3115, 1, 7496}, {23, 3100, 1, 7496}, {23, 3110, 2, 7496}, {24, 3101, 2, 7496}, {24, 3111, 2, 7496}, {24, 3116, 2, 7496}, {25, 3102, 1, 7496}, {25, 3117, 2, 7496}, {26, 3112, 1, 7496}, {27, 3113, 2, 7496}, {27, 3118, 1, 7496}, {28, 3114, 1, 7496}, {28, 3119, 2, 7496}, {29, 3120, 1, 7496}, {21, 3085, 1, 7497}, {23, 3086, 2, 7497}, {23, 3091, 1, 7497}, {24, 3087, 2, 7497}, {24, 3097, 1, 7497}, {26, 3088, 1, 7497}, {26, 3092, 2, 7497}, {27, 3089, 2, 7497}, {27, 3093, 2, 7497}, {27, 3098, 2, 7497}, {28, 3090, 1, 7497}, {28, 3099, 2, 7497}, {30, 3094, 1, 7497}, {31, 3095, 2, 7497}, {31, 3100, 1, 7497}, {32, 3096, 1, 7497}, {32, 3101, 2, 7497}, {33, 3102, 1, 7497}, {21, 3091, 1, 7498}, {23, 3092, 2, 7498}, {23, 3103, 1, 7498}, {24, 3093, 2, 7498}, {24, 3109, 1, 7498}, {26, 3094, 1, 7498}, {26, 3104, 2, 7498}, {27, 3095, 2, 7498}, {27, 3105, 2, 7498}, {27, 3110, 2, 7498}, {28, 3096, 1, 7498}, {28, 3111, 2, 7498}, {30, 3106, 1, 7498}, {31, 3107, 2, 7498}, {31, 3112, 1, 7498}, {32, 3108, 1, 7498}, {32, 3113, 2, 7498}, {33, 3114, 1, 7498}, {21, 3097, 1, 7499}, {23, 3098, 2, 7499}, {23, 3109, 1, 7499}, {24, 3099, 2, 7499}, {24, 3115, 1, 7499}, {26, 3100, 1, 7499}, {26, 3110, 2, 7499}, {27, 3101, 2, 7499}, {27, 3111, 2, 7499}, {27, 3116, 2, 7499}, {28, 3102, 1, 7499}, {28, 3117, 2, 7499}, {30, 3112, 1, 7499}, {31, 3113, 2, 7499}, {31, 3118, 1, 7499}, {32, 3114, 1, 7499}, {32, 3119, 2, 7499}, {33, 3120, 1, 7499}, {22, 3085, 1, 7500}, {24, 3086, 2, 7500}, {24, 3091, 1, 7500}, {25, 3087, 2, 7500}, {25, 3097, 1, 7500}, {27, 3088, 1, 7500}, {27, 3092, 2, 7500}, {28, 3089, 2, 7500}, {28, 3093, 2, 7500}, {28, 3098, 2, 7500}, {29, 3090, 1, 7500}, {29, 3099, 2, 7500}, {31, 3094, 1, 7500}, {32, 3095, 2, 7500}, {32, 3100, 1, 7500}, {33, 3096, 1, 7500}, {33, 3101, 2, 7500}, {34, 3102, 1, 7500}, {22, 3091, 1, 7501}, {24, 3092, 2, 7501}, {24, 3103, 1, 7501}, {25, 3093, 2, 7501}, {25, 3109, 1, 7501}, {27, 3094, 1, 7501}, {27, 3104, 2, 7501}, {28, 3095, 2, 7501}, {28, 3105, 2, 7501}, {28, 3110, 2, 7501}, {29, 3096, 1, 7501}, {29, 3111, 2, 7501}, {31, 3106, 1, 7501}, {32, 3107, 2, 7501}, {32, 3112, 1, 7501}, {33, 3108, 1, 7501}, {33, 3113, 2, 7501}, {34, 3114, 1, 7501}, {22, 3097, 1, 7502}, {24, 3098, 2, 7502}, {24, 3109, 1, 7502}, {25, 3099, 2, 7502}, {25, 3115, 1, 7502}, {27, 3100, 1, 7502}, {27, 3110, 2, 7502}, {28, 3101, 2, 7502}, {28, 3111, 2, 7502}, {28, 3116, 2, 7502}, {29, 3102, 1, 7502}, {29, 3117, 2, 7502}, {31, 3112, 1, 7502}, {32, 3113, 2, 7502}, {32, 3118, 1, 7502}, {33, 3114, 1, 7502}, {33, 3119, 2, 7502}, {34, 3120, 1, 7502}, {20, 3193, 1, 7503}, {21, 3194, 2, 7503}, {21, 3199, 1, 7503}, {22, 3195, 2, 7503}, {22, 3205, 1, 7503}, {23, 3196, 1, 7503}, {23, 3200, 2, 7503}, {24, 3197, 2, 7503}, {24, 3201, 2, 7503}, {24, 3206, 2, 7503}, {25, 3198, 1, 7503}, {25, 3207, 2, 7503}, {26, 3202, 1, 7503}, {27, 3203, 2, 7503}, {27, 3208, 1, 7503}, {28, 3204, 1, 7503}, {28, 3209, 2, 7503}, {29, 3210, 1, 7503}, {21, 3193, 1, 7504}, {23, 3194, 2, 7504}, {23, 3199, 1, 7504}, {24, 3195, 2, 7504}, {24, 3205, 1, 7504}, {26, 3196, 1, 7504}, {26, 3200, 2, 7504}, {27, 3197, 2, 7504}, {27, 3201, 2, 7504}, {27, 3206, 2, 7504}, {28, 3198, 1, 7504}, {28, 3207, 2, 7504}, {30, 3202, 1, 7504}, {31, 3203, 2, 7504}, {31, 3208, 1, 7504}, {32, 3204, 1, 7504}, {32, 3209, 2, 7504}, {33, 3210, 1, 7504}, {22, 3193, 1, 7505}, {24, 3194, 2, 7505}, {24, 3199, 1, 7505}, {25, 3195, 2, 7505}, {25, 3205, 1, 7505}, {27, 3196, 1, 7505}, {27, 3200, 2, 7505}, {28, 3197, 2, 7505}, {28, 3201, 2, 7505}, {28, 3206, 2, 7505}, {29, 3198, 1, 7505}, {29, 3207, 2, 7505}, {31, 3202, 1, 7505}, {32, 3203, 2, 7505}, {32, 3208, 1, 7505}, {33, 3204, 1, 7505}, {33, 3209, 2, 7505}, {34, 3210, 1, 7505}, {20, 3211, 1, 7506}, {21, 3212, 3, 7506}, {21, 3221, 1, 7506}, {22, 3213, 3, 7506}, {22, 3231, 1, 7506}, {23, 3214, 3, 7506}, {23, 3222, 3, 7506}, {24, 3215, 6, 7506}, {24, 3223, 3, 7506}, {24, 3232, 3, 7506}, {25, 3216, 3, 7506}, {25, 3233, 3, 7506}, {26, 3217, 1, 7506}, {26, 3224, 3, 7506}, {27, 3218, 3, 7506}, {27, 3225, 6, 7506}, {27, 3234, 3, 7506}, {28, 3219, 3, 7506}, {28, 3226, 3, 7506}, {28, 3235, 6, 7506}, {29, 3220, 1, 7506}, {29, 3236, 3, 7506}, {30, 3227, 1, 7506}, {31, 3228, 3, 7506}, {31, 3237, 1, 7506}, {32, 3229, 3, 7506}, {32, 3238, 3, 7506}, {33, 3230, 1, 7506}, {33, 3239, 3, 7506}, {34, 3240, 1, 7506}, {20, 3526, 1, 7507}, {21, 3527, 3, 7507}, {21, 3536, 1, 7507}, {22, 3528, 3, 7507}, {22, 3546, 1, 7507}, {23, 3529, 3, 7507}, {23, 3537, 3, 7507}, {24, 3530, 6, 7507}, {24, 3538, 3, 7507}, {24, 3547, 3, 7507}, {25, 3531, 3, 7507}, {25, 3548, 3, 7507}, {26, 3532, 1, 7507}, {26, 3539, 3, 7507}, {27, 3533, 3, 7507}, {27, 3540, 6, 7507}, {27, 3549, 3, 7507}, {28, 3534, 3, 7507}, {28, 3541, 3, 7507}, {28, 3550, 6, 7507}, {29, 3535, 1, 7507}, {29, 3551, 3, 7507}, {30, 3542, 1, 7507}, {31, 3543, 3, 7507}, {31, 3552, 1, 7507}, {32, 3544, 3, 7507}, {32, 3553, 3, 7507}, {33, 3545, 1, 7507}, {33, 3554, 3, 7507}, {34, 3555, 1, 7507}, {20, 3061, 1, 7508}, {21, 3062, 2, 7508}, {22, 3063, 2, 7508}, {23, 3064, 1, 7508}, {24, 3065, 2, 7508}, {25, 3066, 1, 7508}, {21, 3061, 1, 7509}, {23, 3062, 2, 7509}, {24, 3063, 2, 7509}, {26, 3064, 1, 7509}, {27, 3065, 2, 7509}, {28, 3066, 1, 7509}, {22, 3061, 1, 7510}, {24, 3062, 2, 7510}, {25, 3063, 2, 7510}, {27, 3064, 1, 7510}, {28, 3065, 2, 7510}, {29, 3066, 1, 7510}, {23, 3061, 1, 7511}, {26, 3062, 2, 7511}, {27, 3063, 2, 7511}, {30, 3064, 1, 7511}, {31, 3065, 2, 7511}, {32, 3066, 1, 7511}, {24, 3061, 1, 7512}, {27, 3062, 2, 7512}, {28, 3063, 2, 7512}, {31, 3064, 1, 7512}, {32, 3065, 2, 7512}, {33, 3066, 1, 7512}, {25, 3061, 1, 7513}, {28, 3062, 2, 7513}, {29, 3063, 2, 7513}, {32, 3064, 1, 7513}, {33, 3065, 2, 7513}, {34, 3066, 1, 7513}, {20, 3067, 1, 7514}, {21, 3070, 2, 7514}, {22, 3073, 2, 7514}, {23, 3076, 1, 7514}, {24, 3079, 2, 7514}, {25, 3082, 1, 7514}, {20, 3068, 1, 7515}, {21, 3071, 2, 7515}, {22, 3074, 2, 7515}, {23, 3077, 1, 7515}, {24, 3080, 2, 7515}, {25, 3083, 1, 7515}, {20, 3069, 1, 7516}, {21, 3072, 2, 7516}, {22, 3075, 2, 7516},

{23, 3078, 1, 7516}, {24, 3081, 2, 7516}, {25, 3084, 1, 7516}, {21, 3067, 1, 7517}, {23, 3070, 2, 7517}, {24, 3073, 2, 7517}, {26, 3076, 1, 7517}, {27, 3079, 2, 7517}, {28, 3082, 1, 7517}, {21, 3068, 1, 7518}, {23, 3071, 2, 7518}, {24, 3074, 2, 7518}, {26, 3077, 1, 7518}, {27, 3080, 2, 7518}, {28, 3083, 1, 7518}, {21, 3069, 1, 7519}, {23, 3072, 2, 7519}, {24, 3075, 2, 7519}, {26, 3078, 1, 7519}, {27, 3081, 2, 7519}, {28, 3084, 1, 7519}, {22, 3067, 1, 7520}, {24, 3070, 2, 7520}, {25, 3073, 2, 7520}, {27, 3076, 1, 7520}, {28, 3079, 2, 7520}, {29, 3082, 1, 7520}, {22, 3068, 1, 7521}, {24, 3071, 2, 7521}, {25, 3074, 2, 7521}, {27, 3077, 1, 7521}, {28, 3080, 2, 7521}, {29, 3083, 1, 7521}, {22, 3069, 1, 7522}, {24, 3072, 2, 7522}, {25, 3075, 2, 7522}, {27, 3078, 1, 7522}, {28, 3081, 2, 7522}, {29, 3084, 1, 7522}, {23, 3067, 1, 7523}, {26, 3070, 2, 7523}, {27, 3073, 2, 7523}, {30, 3076, 1, 7523}, {31, 3079, 2, 7523}, {32, 3082, 1, 7523}, {23, 3068, 1, 7524}, {26, 3071, 2, 7524}, {27, 3074, 2, 7524}, {30, 3077, 1, 7524}, {31, 3080, 2, 7524}, {32, 3083, 1, 7524}, {23, 3069, 1, 7525}, {26, 3072, 2, 7525}, {27, 3075, 2, 7525}, {30, 3078, 1, 7525}, {31, 3081, 2, 7525}, {32, 3084, 1, 7525}, {24, 3067, 1, 7526}, {27, 3070, 2, 7526}, {28, 3073, 2, 7526}, {31, 3076, 1, 7526}, {32, 3079, 2, 7526}, {33, 3082, 1, 7526}, {24, 3068, 1, 7527}, {27, 3071, 2, 7527}, {28, 3074, 2, 7527}, {31, 3077, 1, 7527}, {32, 3080, 2, 7527}, {33, 3083, 1, 7527}, {24, 3069, 1, 7528}, {27, 3072, 2, 7528}, {28, 3075, 2, 7528}, {31, 3078, 1, 7528}, {32, 3081, 2, 7528}, {33, 3084, 1, 7528}, {25, 3067, 1, 7529}, {28, 3070, 2, 7529}, {29, 3073, 2, 7529}, {32, 3076, 1, 7529}, {33, 3079, 2, 7529}, {34, 3082, 1, 7529}, {25, 3068, 1, 7530}, {28, 3071, 2, 7530}, {29, 3074, 2, 7530}, {32, 3077, 1, 7530}, {33, 3080, 2, 7530}, {34, 3083, 1, 7530}, {25, 3069, 1, 7531}, {28, 3072, 2, 7531}, {29, 3075, 2, 7531}, {32, 3078, 1, 7531}, {33, 3081, 2, 7531}, {34, 3084, 1, 7531}, {20, 3376, 1, 7532}, {21, 3377, 2, 7532}, {22, 3378, 2, 7532}, {23, 3379, 1, 7532}, {24, 3380, 2, 7532}, {25, 3381, 1, 7532}, {21, 3376, 1, 7533}, {23, 3377, 2, 7533}, {24, 3378, 2, 7533}, {26, 3379, 1, 7533}, {27, 3380, 2, 7533}, {28, 3381, 1, 7533}, {22, 3376, 1, 7534}, {24, 3377, 2, 7534}, {25, 3378, 2, 7534}, {27, 3379, 1, 7534}, {28, 3380, 2, 7534}, {29, 3381, 1, 7534}, {23, 3376, 1, 7535}, {26, 3377, 2, 7535}, {27, 3378, 2, 7535}, {30, 3379, 1, 7535}, {31, 3380, 2, 7535}, {32, 3381, 1, 7535}, {24, 3376, 1, 7536}, {27, 3377, 2, 7536}, {28, 3378, 2, 7536}, {31, 3379, 1, 7536}, {32, 3380, 2, 7536}, {33, 3381, 1, 7536}, {25, 3376, 1, 7537}, {28, 3377, 2, 7537}, {29, 3378, 2, 7537}, {32, 3379, 1, 7537}, {33, 3380, 2, 7537}, {34, 3381, 1, 7537}, {290, 1073, 1, 7538}, {291, 1074, 2, 7538}, {292, 1075, 2, 7538}, {293, 1079, 1, 7538}, {294, 1080, 2, 7538}, {295, 1084, 1, 7538}, {290, 1074, 1, 7539}, {291, 1076, 1, 7539}, {291, 1079, 1, 7539}, {292, 1077, 1, 7539}, {292, 1080, 1, 7539}, {293, 1081, 1, 7539}, {294, 1082, 1, 7539}, {294, 1085, 1, 7539}, {295, 1086, 1, 7539}, {290, 1075, 1, 7540}, {291, 1077, 1, 7540}, {291, 1080, 1, 7540}, {292, 1078, 1, 7540}, {292, 1084, 1, 7540}, {293, 1082, 1, 7540}, {294, 1083, 1, 7540}, {294, 1086, 1, 7540}, {295, 1087, 1, 7540}, {290, 1079, 1, 7541}, {291, 1081, 2, 7541}, {292, 1082, 2, 7541}, {293, 1088, 1, 7541}, {294, 1089, 2, 7541}, {295, 1091, 1, 7541}, {290, 1080, 1, 7542}, {291, 1082, 1, 7542}, {291, 1085, 1, 7542}, {292, 1083, 1, 7542}, {292, 1086, 1, 7542}, {293, 1089, 1, 7542}, {294, 1090, 1, 7542}, {294, 1091, 1, 7542}, {295, 1092, 1, 7542}, {290, 1084, 1, 7543}, {291, 1086, 2, 7543}, {292, 1087, 2, 7543}, {293, 1091, 1, 7543}, {294, 1092, 2, 7543}, {295, 1093, 1, 7543}, {296, 1073, 1, 7544}, {297, 1074, 2, 7544}, {298, 1075, 2, 7544}, {299, 1079, 1, 7544}, {300, 1080, 2, 7544}, {301, 1084, 1, 7544}, {297, 1073, 1, 7545}, {299, 1074, 2, 7545}, {300, 1075, 2, 7545}, {302, 1079, 1, 7545}, {303, 1080, 2, 7545}, {304, 1084, 1, 7545}, {298, 1073, 1, 7546}, {300, 1074, 2, 7546}, {301, 1075, 2, 7546}, {303, 1079, 1, 7546}, {304, 1080, 2, 7546}, {305, 1084, 1, 7546}, {296, 1074, 1, 7547}, {297, 1076, 1, 7547}, {297, 1079, 1, 7547}, {298, 1077, 1, 7547}, {298, 1080, 1, 7547}, {299, 1081, 1, 7547}, {300, 1082, 1, 7547}, {300, 1085, 1, 7547}, {301, 1086, 1, 7547}, {297, 1074, 1, 7548}, {299, 1076, 1, 7548}, {299, 1079, 1, 7548}, {300, 1077, 1, 7548}, {300, 1080, 1, 7548}, {302, 1081, 1, 7548}, {303, 1082, 1, 7548}, {303, 1085, 1, 7548}, {304, 1086, 1, 7548}, {298, 1074, 1, 7549}, {300, 1076, 1, 7549}, {300, 1079, 1, 7549}, {301, 1077, 1, 7549}, {301, 1080, 1, 7549}, {303, 1081, 1, 7549}, {304, 1082, 1, 7549}, {304, 1085, 1, 7549}, {305, 1086, 1, 7549}, {296, 1075, 1, 7550}, {297, 1077, 1, 7550}, {297, 1080, 1, 7550}, {298, 1078, 1, 7550}, {298, 1084, 1, 7550}, {299, 1082, 1, 7550}, {300, 1083, 1, 7550}, {300, 1086, 1, 7550}, {301, 1087, 1, 7550}, {297, 1075, 1, 7551}, {299, 1077, 1, 7551}, {299, 1080, 1, 7551}, {300, 1078, 1, 7551}, {300, 1084, 1, 7551}, {302, 1082, 1, 7551}, {303, 1083, 1, 7551}, {303, 1086, 1, 7551}, {304, 1087, 1, 7551}, {298, 1075, 1, 7552}, {300, 1077, 1, 7552}, {300, 1080, 1, 7552}, {301, 1078, 1, 7552}, {301, 1084, 1, 7552}, {303, 1082, 1, 7552}, {304, 1083, 1, 7552}, {304, 1086, 1, 7552}, {305, 1087, 1, 7552}, {296, 1079, 1, 7553}, {297, 1081, 2, 7553}, {298, 1082, 2, 7553}, {299, 1088, 1, 7553}, {300, 1089, 2, 7553}, {301, 1091, 1, 7553}, {297, 1079, 1, 7554}, {299, 1081, 2, 7554}, {300, 1082, 2, 7554}, {302, 1088, 1, 7554}, {303, 1089, 2, 7554}, {304, 1091, 1, 7554}, {298, 1079, 1, 7555}, {300, 1081, 2, 7555}, {301, 1082, 2, 7555}, {303, 1088, 1, 7555}, {304, 1089, 2, 7555}, {305, 1091, 1, 7555}, {296, 1080, 1, 7556}, {297, 1082, 1, 7556}, {297, 1085, 1, 7556}, {298, 1083, 1, 7556}, {298, 1086, 1, 7556}, {299, 1089, 1, 7556}, {300, 1090, 1, 7556}, {300, 1091, 1, 7556}, {301, 1092, 1, 7556}, {297, 1080, 1, 7557}, {299, 1082, 1, 7557}, {299, 1085, 1, 7557}, {300, 1083, 1, 7557}, {300, 1086, 1, 7557}, {302, 1089, 1, 7557}, {303, 1090, 1, 7557}, {303, 1091, 1, 7557}, {304, 1092, 1, 7557}, {298, 1080, 1, 7558}, {300, 1082, 1,

7558}, {300, 1085, 1, 7558}, {301, 1083, 1, 7558}, {301, 1086, 1, 7558}, {303, 1089, 1,
7558}, {304, 1090, 1, 7558}, {304, 1091, 1, 7558}, {305, 1092, 1, 7558}, {296, 1084, 1,
7559}, {297, 1086, 2, 7559}, {298, 1087, 2, 7559}, {299, 1091, 1, 7559}, {300, 1092, 2,
7559}, {301, 1093, 1, 7559}, {297, 1084, 1, 7560}, {299, 1086, 2, 7560}, {300, 1087, 2,
7560}, {302, 1091, 1, 7560}, {303, 1092, 2, 7560}, {304, 1093, 1, 7560}, {298, 1084, 1,
7561}, {300, 1086, 2, 7561}, {301, 1087, 2, 7561}, {303, 1091, 1, 7561}, {304, 1092, 2,
7561}, {305, 1093, 1, 7561}, {20, 3067, 1, 7562}, {21, 3068, 1, 7562}, {21, 3070, 2,
7562}, {22, 3069, 1, 7562}, {22, 3073, 2, 7562}, {23, 3071, 2, 7562}, {23, 3076, 1, 7562},
{24, 3072, 2, 7562}, {24, 3074, 2, 7562}, {24, 3079, 2, 7562}, {25, 3075, 2, 7562}, {25,
3082, 1, 7562}, {26, 3077, 1, 7562}, {27, 3078, 1, 7562}, {27, 3080, 2, 7562}, {28, 3081,
2, 7562}, {28, 3083, 1, 7562}, {29, 3084, 1, 7562}, {21, 3067, 1, 7563}, {23, 3068, 1,
7563}, {23, 3070, 2, 7563}, {24, 3069, 1, 7563}, {24, 3073, 2, 7563}, {26, 3071, 2, 7563},
{26, 3076, 1, 7563}, {27, 3072, 2, 7563}, {27, 3074, 2, 7563}, {27, 3079, 2, 7563}, {28,
3075, 2, 7563}, {28, 3082, 1, 7563}, {30, 3077, 1, 7563}, {31, 3078, 1, 7563}, {31, 3080,
2, 7563}, {32, 3081, 2, 7563}, {32, 3083, 1, 7563}, {33, 3084, 1, 7563}, {22, 3067, 1,
7564}, {24, 3068, 1, 7564}, {24, 3070, 2, 7564}, {25, 3069, 1, 7564}, {25, 3073, 2, 7564},
{27, 3071, 2, 7564}, {27, 3076, 1, 7564}, {28, 3072, 2, 7564}, {28, 3074, 2, 7564}, {28,
3079, 2, 7564}, {29, 3075, 2, 7564}, {29, 3082, 1, 7564}, {31, 3077, 1, 7564}, {32, 3078,
1, 7564}, {32, 3080, 2, 7564}, {33, 3081, 2, 7564}, {33, 3083, 1, 7564}, {34, 3084, 1,
7564}, {20, 3085, 1, 7565}, {21, 3086, 1, 7565}, {21, 3091, 2, 7565}, {22, 3087, 1, 7565},
{22, 3097, 2, 7565}, {23, 3092, 2, 7565}, {23, 3103, 1, 7565}, {24, 3093, 2, 7565}, {24,
3098, 2, 7565}, {24, 3109, 2, 7565}, {25, 3099, 2, 7565}, {25, 3115, 1, 7565}, {26, 3104,
1, 7565}, {27, 3105, 1, 7565}, {27, 3110, 2, 7565}, {28, 3111, 2, 7565}, {28, 3116, 1,
7565}, {29, 3117, 1, 7565}, {20, 3086, 1, 7566}, {21, 3088, 1, 7566}, {21, 3092, 2, 7566},
{22, 3089, 1, 7566}, {22, 3098, 2, 7566}, {23, 3094, 2, 7566}, {23, 3104, 1, 7566}, {24,
3095, 2, 7566}, {24, 3100, 2, 7566}, {24, 3110, 2, 7566}, {25, 3101, 2, 7566}, {25, 3116,
1, 7566}, {26, 3106, 1, 7566}, {27, 3107, 1, 7566}, {27, 3112, 2, 7566}, {28, 3113, 2,
7566}, {28, 3118, 1, 7566}, {29, 3119, 1, 7566}, {20, 3087, 1, 7567}, {21, 3089, 1, 7567},
{21, 3093, 2, 7567}, {22, 3090, 1, 7567}, {22, 3099, 2, 7567}, {23, 3095, 2, 7567}, {23,
3105, 1, 7567}, {24, 3096, 2, 7567}, {24, 3101, 2, 7567}, {24, 3111, 2, 7567}, {25, 3102,
2, 7567}, {25, 3117, 1, 7567}, {26, 3107, 1, 7567}, {27, 3108, 1, 7567}, {27, 3113, 2,
7567}, {28, 3114, 2, 7567}, {28, 3119, 1, 7567}, {29, 3120, 1, 7567}, {21, 3085, 1, 7568},
{23, 3086, 1, 7568}, {23, 3091, 2, 7568}, {24, 3087, 1, 7568}, {24, 3097, 2, 7568}, {26,
3092, 2, 7568}, {26, 3103, 1, 7568}, {27, 3093, 2, 7568}, {27, 3098, 2, 7568}, {27, 3109,
2, 7568}, {28, 3099, 2, 7568}, {28, 3115, 1, 7568}, {30, 3104, 1, 7568}, {31, 3105, 1,
7568}, {31, 3110, 2, 7568}, {32, 3111, 2, 7568}, {32, 3116, 1, 7568}, {33, 3117, 1, 7568},
{21, 3086, 1, 7569}, {23, 3088, 1, 7569}, {23, 3092, 2, 7569}, {24, 3089, 1, 7569}, {24,
3098, 2, 7569}, {26, 3094, 2, 7569}, {26, 3104, 1, 7569}, {27, 3095, 2, 7569}, {27, 3100,
2, 7569}, {27, 3110, 2, 7569}, {28, 3101, 2, 7569}, {28, 3116, 1, 7569}, {30, 3106, 1,
7569}, {31, 3107, 1, 7569}, {31, 3112, 2, 7569}, {32, 3113, 2, 7569}, {32, 3118, 1, 7569},
{33, 3119, 1, 7569}, {21, 3087, 1, 7570}, {23, 3089, 1, 7570}, {23, 3093, 2, 7570}, {24,
3090, 1, 7570}, {24, 3099, 2, 7570}, {26, 3095, 2, 7570}, {26, 3105, 1, 7570}, {27, 3096,
2, 7570}, {27, 3101, 2, 7570}, {27, 3111, 2, 7570}, {28, 3102, 2, 7570}, {28, 3117, 1,
7570}, {30, 3107, 1, 7570}, {31, 3108, 1, 7570}, {31, 3113, 2, 7570}, {32, 3114, 2, 7570},
{32, 3119, 1, 7570}, {33, 3120, 1, 7570}, {22, 3085, 1, 7571}, {24, 3086, 1, 7571}, {24,
3091, 2, 7571}, {25, 3087, 1, 7571}, {25, 3097, 2, 7571}, {27, 3092, 2, 7571}, {27, 3103,
1, 7571}, {28, 3093, 2, 7571}, {28, 3098, 2, 7571}, {28, 3109, 2, 7571}, {29, 3099, 2,
7571}, {29, 3115, 1, 7571}, {31, 3104, 1, 7571}, {32, 3105, 1, 7571}, {32, 3110, 2, 7571},
{33, 3111, 2, 7571}, {33, 3116, 1, 7571}, {34, 3117, 1, 7571}, {22, 3086, 1, 7572}, {24,
3088, 1, 7572}, {24, 3092, 2, 7572}, {25, 3089, 1, 7572}, {25, 3098, 2, 7572}, {27, 3094,
2, 7572}, {27, 3104, 1, 7572}, {28, 3095, 2, 7572}, {28, 3100, 2, 7572}, {28, 3110, 2,
7572}, {29, 3101, 2, 7572}, {29, 3116, 1, 7572}, {31, 3106, 1, 7572}, {32, 3107, 1, 7572},
{32, 3112, 2, 7572}, {33, 3113, 2, 7572}, {33, 3118, 1, 7572}, {34, 3119, 1, 7572}, {22,
3087, 1, 7573}, {24, 3089, 1, 7573}, {24, 3093, 2, 7573}, {25, 3090, 1, 7573}, {25, 3099,
2, 7573}, {27, 3095, 2, 7573}, {27, 3105, 1, 7573}, {28, 3096, 2, 7573}, {28, 3101, 2,
7573}, {28, 3111, 2, 7573}, {29, 3102, 2, 7573}, {29, 3117, 1, 7573}, {31, 3107, 1, 7573},
{32, 3108, 1, 7573}, {32, 3113, 2, 7573}, {33, 3114, 2, 7573}, {33, 3119, 1, 7573}, {34,
3120, 1, 7573}, {20, 3286, 1, 7574}, {21, 3287, 1, 7574}, {21, 3289, 2, 7574}, {22, 3288,
1, 7574}, {22, 3292, 2, 7574}, {23, 3290, 2, 7574}, {23, 3295, 1, 7574}, {24, 3291, 2,
7574}, {24, 3293, 2, 7574}, {24, 3298, 2, 7574}, {25, 3294, 2, 7574}, {25, 3301, 1, 7574},
{26, 3296, 1, 7574}, {27, 3297, 1, 7574}, {27, 3299, 2, 7574}, {28, 3300, 2, 7574}, {28,
3302, 1, 7574}, {29, 3303, 1, 7574}, {20, 3289, 1, 7575}, {21, 3290, 1, 7575}, {21, 3295,
2, 7575}, {22, 3291, 1, 7575}, {22, 3298, 2, 7575}, {23, 3296, 2, 7575}, {23, 3304, 1,
7575}, {24, 3297, 2, 7575}, {24, 3299, 2, 7575}, {24, 3307, 2, 7575}, {25, 3300, 2, 7575},
{25, 3310, 1, 7575}, {26, 3305, 1, 7575}, {27, 3306, 1, 7575}, {27, 3308, 2, 7575}, {28,
3309, 2, 7575}, {28, 3311, 1, 7575}, {29, 3312, 1, 7575}, {20, 3292, 1, 7576}, {21, 3293,
1, 7576}, {21, 3298, 2, 7576}, {22, 3294, 1, 7576}, {22, 3301, 2, 7576}, {23, 3299, 2,
7576}, {23, 3307, 1, 7576}, {24, 3300, 2, 7576}, {24, 3302, 2, 7576}, {24, 3310, 2, 7576},
{25, 3303, 2, 7576}, {25, 3313, 1, 7576}, {26, 3308, 1, 7576}, {27, 3309, 1, 7576}, {27,

3311, 2, 7576}, {28, 3312, 2, 7576}, {28, 3314, 1, 7576}, {29, 3315, 1, 7576}, {21, 3286, 1, 7577}, {23, 3287, 1, 7577}, {23, 3289, 2, 7577}, {24, 3288, 1, 7577}, {24, 3292, 2, 7577}, {26, 3290, 2, 7577}, {26, 3295, 1, 7577}, {27, 3291, 2, 7577}, {27, 3293, 2, 7577}, {27, 3298, 2, 7577}, {28, 3294, 2, 7577}, {28, 3301, 1, 7577}, {30, 3296, 1, 7577}, {31, 3297, 1, 7577}, {31, 3299, 2, 7577}, {32, 3300, 2, 7577}, {32, 3302, 1, 7577}, {33, 3303, 1, 7577}, {21, 3289, 1, 7578}, {23, 3290, 1, 7578}, {23, 3295, 2, 7578}, {24, 3291, 1, 7578}, {24, 3298, 2, 7578}, {26, 3296, 2, 7578}, {26, 3304, 1, 7578}, {27, 3297, 2, 7578}, {27, 3299, 2, 7578}, {27, 3307, 2, 7578}, {28, 3300, 2, 7578}, {28, 3310, 1, 7578}, {30, 3305, 1, 7578}, {31, 3306, 1, 7578}, {31, 3308, 2, 7578}, {32, 3309, 2, 7578}, {32, 3311, 1, 7578}, {33, 3312, 1, 7578}, {21, 3292, 1, 7579}, {23, 3293, 1, 7579}, {23, 3298, 2, 7579}, {24, 3294, 1, 7579}, {24, 3301, 2, 7579}, {26, 3299, 2, 7579}, {26, 3307, 1, 7579}, {27, 3300, 2, 7579}, {27, 3302, 2, 7579}, {27, 3310, 2, 7579}, {28, 3303, 2, 7579}, {28, 3313, 1, 7579}, {30, 3308, 1, 7579}, {31, 3309, 1, 7579}, {31, 3311, 2, 7579}, {32, 3312, 2, 7579}, {32, 3314, 1, 7579}, {33, 3315, 1, 7579}, {22, 3286, 1, 7580}, {24, 3287, 1, 7580}, {24, 3289, 2, 7580}, {25, 3288, 1, 7580}, {25, 3292, 2, 7580}, {27, 3290, 2, 7580}, {27, 3295, 1, 7580}, {28, 3291, 2, 7580}, {28, 3293, 2, 7580}, {28, 3298, 2, 7580}, {29, 3294, 2, 7580}, {29, 3301, 1, 7580}, {31, 3296, 1, 7580}, {32, 3297, 1, 7580}, {32, 3299, 2, 7580}, {33, 3300, 2, 7580}, {33, 3302, 1, 7580}, {34, 3303, 1, 7580}, {22, 3289, 1, 7581}, {24, 3290, 1, 7581}, {24, 3295, 2, 7581}, {25, 3291, 1, 7581}, {25, 3298, 2, 7581}, {27, 3296, 2, 7581}, {27, 3304, 1, 7581}, {28, 3297, 2, 7581}, {28, 3299, 2, 7581}, {28, 3307, 2, 7581}, {29, 3300, 2, 7581}, {29, 3310, 1, 7581}, {31, 3305, 1, 7581}, {32, 3306, 1, 7581}, {32, 3308, 2, 7581}, {33, 3309, 2, 7581}, {33, 3311, 1, 7581}, {34, 3312, 1, 7581}, {22, 3292, 1, 7582}, {24, 3293, 1, 7582}, {24, 3298, 2, 7582}, {25, 3294, 1, 7582}, {25, 3301, 2, 7582}, {27, 3299, 2, 7582}, {27, 3307, 1, 7582}, {28, 3300, 2, 7582}, {28, 3302, 2, 7582}, {28, 3310, 2, 7582}, {29, 3303, 2, 7582}, {29, 3313, 1, 7582}, {31, 3308, 1, 7582}, {32, 3309, 1, 7582}, {32, 3311, 2, 7582}, {33, 3312, 2, 7582}, {33, 3314, 1, 7582}, {34, 3315, 1, 7582}, {20, 3382, 1, 7583}, {21, 3383, 1, 7583}, {21, 3385, 2, 7583}, {22, 3384, 1, 7583}, {22, 3388, 2, 7583}, {23, 3386, 2, 7583}, {23, 3391, 1, 7583}, {24, 3387, 2, 7583}, {24, 3389, 2, 7583}, {24, 3394, 2, 7583}, {25, 3390, 2, 7583}, {25, 3397, 1, 7583}, {26, 3392, 1, 7583}, {27, 3393, 1, 7583}, {27, 3395, 2, 7583}, {28, 3396, 2, 7583}, {28, 3398, 1, 7583}, {29, 3399, 1, 7583}, {21, 3382, 1, 7584}, {23, 3383, 1, 7584}, {23, 3385, 2, 7584}, {24, 3384, 1, 7584}, {24, 3388, 2, 7584}, {26, 3386, 2, 7584}, {26, 3391, 1, 7584}, {27, 3387, 2, 7584}, {27, 3389, 2, 7584}, {27, 3394, 2, 7584}, {28, 3390, 2, 7584}, {28, 3397, 1, 7584}, {30, 3392, 1, 7584}, {31, 3393, 1, 7584}, {31, 3395, 2, 7584}, {32, 3396, 2, 7584}, {32, 3398, 1, 7584}, {33, 3399, 1, 7584}, {22, 3382, 1, 7585}, {24, 3383, 1, 7585}, {24, 3385, 2, 7585}, {25, 3384, 1, 7585}, {25, 3388, 2, 7585}, {27, 3386, 2, 7585}, {27, 3391, 1, 7585}, {28, 3387, 2, 7585}, {28, 3389, 2, 7585}, {28, 3394, 2, 7585}, {29, 3390, 2, 7585}, {29, 3397, 1, 7585}, {31, 3392, 1, 7585}, {32, 3393, 1, 7585}, {32, 3395, 2, 7585}, {33, 3396, 2, 7585}, {33, 3398, 1, 7585}, {34, 3399, 1, 7585}, {20, 3085, 1, 7586}, {21, 3086, 2, 7586}, {21, 3091, 2, 7586}, {22, 3087, 2, 7586}, {22, 3097, 2, 7586}, {23, 3088, 1, 7586}, {23, 3092, 4, 7586}, {23, 3103, 1, 7586}, {24, 3089, 2, 7586}, {24, 3093, 4, 7586}, {24, 3098, 4, 7586}, {24, 3109, 2, 7586}, {25, 3090, 1, 7586}, {25, 3099, 4, 7586}, {25, 3115, 1, 7586}, {26, 3094, 2, 7586}, {26, 3104, 2, 7586}, {27, 3095, 4, 7586}, {27, 3100, 2, 7586}, {27, 3105, 2, 7586}, {27, 3110, 4, 7586}, {28, 3096, 2, 7586}, {28, 3101, 4, 7586}, {28, 3111, 4, 7586}, {28, 3116, 2, 7586}, {29, 3102, 2, 7586}, {29, 3117, 2, 7586}, {30, 3106, 1, 7586}, {31, 3107, 2, 7586}, {31, 3112, 2, 7586}, {32, 3108, 1, 7586}, {32, 3113, 4, 7586}, {32, 3118, 1, 7586}, {33, 3114, 2, 7586}, {33, 3119, 2, 7586}, {34, 3120, 1, 7586}, {20, 3121, 1, 7587}, {21, 3122, 2, 7587}, {21, 3131, 2, 7587}, {22, 3123, 2, 7587}, {22, 3141, 2, 7587}, {23, 3124, 1, 7587}, {23, 3132, 4, 7587}, {23, 3151, 1, 7587}, {24, 3125, 2, 7587}, {24, 3133, 4, 7587}, {24, 3142, 4, 7587}, {24, 3161, 2, 7587}, {25, 3126, 1, 7587}, {25, 3143, 4, 7587}, {25, 3171, 1, 7587}, {26, 3134, 2, 7587}, {26, 3152, 2, 7587}, {27, 3135, 4, 7587}, {27, 3144, 2, 7587}, {27, 3153, 2, 7587}, {27, 3162, 4, 7587}, {28, 3136, 2, 7587}, {28, 3145, 4, 7587}, {28, 3163, 4, 7587}, {28, 3172, 2, 7587}, {29, 3146, 2, 7587}, {29, 3173, 2, 7587}, {30, 3154, 1, 7587}, {31, 3155, 2, 7587}, {31, 3164, 2, 7587}, {32, 3156, 1, 7587}, {32, 3165, 4, 7587}, {32, 3174, 1, 7587}, {33, 3166, 2, 7587}, {33, 3175, 2, 7587}, {34, 3176, 1, 7587}, {20, 3122, 1, 7588}, {21, 3124, 2, 7588}, {21, 3132, 2, 7588}, {22, 3125, 2, 7588}, {22, 3142, 2, 7588}, {23, 3127, 1, 7588}, {23, 3134, 4, 7588}, {23, 3152, 1, 7588}, {24, 3128, 2, 7588}, {24, 3135, 4, 7588}, {24, 3144, 4, 7588}, {24, 3162, 2, 7588}, {25, 3129, 1, 7588}, {25, 3145, 4, 7588}, {25, 3172, 1, 7588}, {26, 3137, 2, 7588}, {26, 3154, 2, 7588}, {27, 3138, 4, 7588}, {27, 3147, 2, 7588}, {27, 3155, 2, 7588}, {27, 3164, 4, 7588}, {28, 3139, 2, 7588}, {28, 3148, 4, 7588}, {28, 3165, 4, 7588}, {28, 3174, 2, 7588}, {29, 3149, 2, 7588}, {29, 3175, 2, 7588}, {30, 3157, 1, 7588}, {31, 3158, 2, 7588}, {31, 3167, 2, 7588}, {32, 3159, 1, 7588}, {32, 3168, 4, 7588}, {32, 3177, 1, 7588}, {33, 3169, 2, 7588}, {33, 3178, 2, 7588}, {34, 3179, 1, 7588}, {20, 3123, 1, 7589}, {21, 3125, 2, 7589}, {21, 3133, 2, 7589}, {22, 3126, 2, 7589}, {22, 3143, 2, 7589}, {23, 3128, 1, 7589}, {23, 3135, 4, 7589}, {23, 3153, 1, 7589}, {24, 3129, 2, 7589}, {24, 3136, 4, 7589}, {24, 3145, 4, 7589}, {24, 3163, 2, 7589}, {25, 3130, 1, 7589}, {25, 3146, 4, 7589}, {25, 3173, 1, 7589}, {26, 3138, 2, 7589}, {26, 3155, 2, 7589}, {27, 3139, 4, 7589}, {27, 3148, 2, 7589}, {27, 3156,

2, 7589}, {27, 3165, 4, 7589}, {28, 3140, 2, 7589}, {28, 3149, 4, 7589}, {28, 3166, 4, 7589}, {28, 3175, 2, 7589}, {29, 3150, 2, 7589}, {29, 3176, 2, 7589}, {30, 3158, 1, 7589}, {31, 3159, 2, 7589}, {31, 3168, 2, 7589}, {32, 3160, 1, 7589}, {32, 3169, 4, 7589}, {32, 3178, 1, 7589}, {33, 3170, 2, 7589}, {33, 3179, 2, 7589}, {34, 3180, 1, 7589}, {35, 3067, 1, 7590}, {36, 3068, 1, 7590}, {36, 3070, 2, 7590}, {37, 3069, 1, 7590}, {37, 3073, 2, 7590}, {38, 3071, 2, 7590}, {38, 3076, 1, 7590}, {39, 3072, 2, 7590}, {39, 3074, 2, 7590}, {39, 3079, 2, 7590}, {40, 3075, 2, 7590}, {40, 3082, 1, 7590}, {41, 3077, 1, 7590}, {42, 3078, 1, 7590}, {42, 3080, 2, 7590}, {43, 3081, 2, 7590}, {43, 3083, 1, 7590}, {44, 3084, 1, 7590}, {36, 3067, 1, 7591}, {38, 3068, 1, 7591}, {38, 3070, 2, 7591}, {39, 3069, 1, 7591}, {39, 3073, 2, 7591}, {41, 3071, 2, 7591}, {41, 3076, 1, 7591}, {42, 3072, 2, 7591}, {42, 3074, 2, 7591}, {42, 3079, 2, 7591}, {43, 3075, 2, 7591}, {43, 3082, 1, 7591}, {45, 3077, 1, 7591}, {46, 3078, 1, 7591}, {46, 3080, 2, 7591}, {47, 3081, 2, 7591}, {47, 3083, 1, 7591}, {48, 3084, 1, 7591}, {37, 3067, 1, 7592}, {39, 3068, 1, 7592}, {39, 3070, 2, 7592}, {40, 3069, 1, 7592}, {40, 3073, 2, 7592}, {42, 3071, 2, 7592}, {42, 3076, 1, 7592}, {43, 3072, 2, 7592}, {43, 3074, 2, 7592}, {43, 3079, 2, 7592}, {44, 3075, 2, 7592}, {44, 3082, 1, 7592}, {46, 3077, 1, 7592}, {47, 3078, 1, 7592}, {47, 3080, 2, 7592}, {48, 3081, 2, 7592}, {48, 3083, 1, 7592}, {49, 3084, 1, 7592}, {38, 3067, 1, 7593}, {41, 3068, 1, 7593}, {41, 3070, 2, 7593}, {42, 3069, 1, 7593}, {42, 3073, 2, 7593}, {45, 3071, 2, 7593}, {45, 3076, 1, 7593}, {46, 3072, 2, 7593}, {46, 3074, 2, 7593}, {46, 3079, 2, 7593}, {47, 3075, 2, 7593}, {47, 3082, 1, 7593}, {50, 3077, 1, 7593}, {51, 3078, 1, 7593}, {51, 3080, 2, 7593}, {52, 3081, 2, 7593}, {52, 3083, 1, 7593}, {53, 3084, 1, 7593}, {39, 3067, 1, 7594}, {42, 3068, 1, 7594}, {42, 3070, 2, 7594}, {43, 3069, 1, 7594}, {43, 3073, 2, 7594}, {46, 3071, 2, 7594}, {46, 3076, 1, 7594}, {47, 3072, 2, 7594}, {47, 3074, 2, 7594}, {47, 3079, 2, 7594}, {48, 3075, 2, 7594}, {48, 3082, 1, 7594}, {51, 3077, 1, 7594}, {52, 3078, 1, 7594}, {52, 3080, 2, 7594}, {53, 3081, 2, 7594}, {53, 3083, 1, 7594}, {54, 3084, 1, 7594}, {40, 3067, 1, 7595}, {43, 3068, 1, 7595}, {43, 3070, 2, 7595}, {44, 3069, 1, 7595}, {44, 3073, 2, 7595}, {47, 3071, 2, 7595}, {47, 3076, 1, 7595}, {48, 3072, 2, 7595}, {48, 3074, 2, 7595}, {48, 3079, 2, 7595}, {49, 3075, 2, 7595}, {49, 3082, 1, 7595}, {52, 3077, 1, 7595}, {53, 3078, 1, 7595}, {53, 3080, 2, 7595}, {54, 3081, 2, 7595}, {54, 3083, 1, 7595}, {55, 3084, 1, 7595}, {20, 3316, 1, 7596}, {21, 3317, 2, 7596}, {21, 3322, 2, 7596}, {22, 3318, 2, 7596}, {22, 3328, 2, 7596}, {23, 3319, 1, 7596}, {23, 3323, 4, 7596}, {23, 3334, 1, 7596}, {24, 3320, 2, 7596}, {24, 3324, 4, 7596}, {24, 3329, 4, 7596}, {24, 3340, 2, 7596}, {25, 3321, 1, 7596}, {25, 3330, 4, 7596}, {25, 3346, 1, 7596}, {26, 3325, 2, 7596}, {26, 3335, 2, 7596}, {27, 3326, 4, 7596}, {27, 3331, 2, 7596}, {27, 3336, 2, 7596}, {27, 3341, 4, 7596}, {28, 3327, 2, 7596}, {28, 3332, 4, 7596}, {28, 3342, 4, 7596}, {28, 3347, 2, 7596}, {29, 3333, 2, 7596}, {29, 3348, 2, 7596}, {30, 3337, 1, 7596}, {31, 3338, 2, 7596}, {31, 3343, 2, 7596}, {32, 3339, 1, 7596}, {32, 3344, 4, 7596}, {32, 3349, 1, 7596}, {33, 3345, 2, 7596}, {33, 3350, 2, 7596}, {34, 3351, 1, 7596}, {20, 3322, 1, 7597}, {21, 3323, 2, 7597}, {21, 3334, 2, 7597}, {22, 3324, 2, 7597}, {22, 3340, 2, 7597}, {23, 3325, 1, 7597}, {23, 3335, 4, 7597}, {23, 3352, 1, 7597}, {24, 3326, 2, 7597}, {24, 3336, 4, 7597}, {24, 3341, 4, 7597}, {24, 3358, 2, 7597}, {25, 3327, 1, 7597}, {25, 3342, 4, 7597}, {25, 3364, 1, 7597}, {26, 3337, 2, 7597}, {26, 3353, 2, 7597}, {27, 3338, 4, 7597}, {27, 3343, 2, 7597}, {27, 3354, 2, 7597}, {27, 3359, 4, 7597}, {28, 3339, 2, 7597}, {28, 3344, 4, 7597}, {28, 3360, 4, 7597}, {28, 3365, 2, 7597}, {29, 3345, 2, 7597}, {29, 3366, 2, 7597}, {30, 3355, 1, 7597}, {31, 3356, 2, 7597}, {31, 3361, 2, 7597}, {32, 3357, 1, 7597}, {32, 3362, 4, 7597}, {32, 3367, 1, 7597}, {33, 3363, 2, 7597}, {33, 3368, 2, 7597}, {34, 3369, 1, 7597}, {20, 3328, 1, 7598}, {21, 3329, 2, 7598}, {21, 3340, 2, 7598}, {22, 3330, 2, 7598}, {22, 3346, 2, 7598}, {23, 3331, 1, 7598}, {23, 3341, 4, 7598}, {23, 3358, 1, 7598}, {24, 3332, 2, 7598}, {24, 3342, 4, 7598}, {24, 3347, 4, 7598}, {24, 3364, 2, 7598}, {25, 3333, 1, 7598}, {25, 3348, 4, 7598}, {25, 3370, 1, 7598}, {26, 3343, 2, 7598}, {26, 3359, 2, 7598}, {27, 3344, 4, 7598}, {27, 3349, 2, 7598}, {27, 3360, 2, 7598}, {27, 3365, 4, 7598}, {28, 3345, 2, 7598}, {28, 3350, 4, 7598}, {28, 3366, 4, 7598}, {28, 3371, 2, 7598}, {29, 3351, 2, 7598}, {29, 3372, 2, 7598}, {30, 3361, 1, 7598}, {31, 3362, 2, 7598}, {31, 3367, 2, 7598}, {32, 3363, 1, 7598}, {32, 3368, 4, 7598}, {32, 3373, 1, 7598}, {33, 3369, 2, 7598}, {33, 3374, 2, 7598}, {34, 3375, 1, 7598}, {20, 3400, 1, 7599}, {21, 3401, 2, 7599}, {21, 3406, 2, 7599}, {22, 3402, 2, 7599}, {22, 3412, 2, 7599}, {23, 3403, 1, 7599}, {23, 3407, 4, 7599}, {23, 3418, 1, 7599}, {24, 3404, 2, 7599}, {24, 3408, 4, 7599}, {24, 3413, 4, 7599}, {24, 3424, 2, 7599}, {25, 3405, 1, 7599}, {25, 3414, 4, 7599}, {25, 3430, 1, 7599}, {26, 3409, 2, 7599}, {26, 3419, 2, 7599}, {27, 3410, 4, 7599}, {27, 3415, 2, 7599}, {27, 3420, 2, 7599}, {27, 3425, 4, 7599}, {28, 3411, 2, 7599}, {28, 3416, 4, 7599}, {28, 3426, 4, 7599}, {28, 3431, 2, 7599}, {29, 3417, 2, 7599}, {29, 3432, 2, 7599}, {30, 3421, 1, 7599}, {31, 3422, 2, 7599}, {31, 3427, 2, 7599}, {32, 3423, 1, 7599}, {32, 3428, 4, 7599}, {32, 3433, 1, 7599}, {33, 3429, 2, 7599}, {33, 3434, 2, 7599}, {34, 3435, 1, 7599}, {20, 3760, 1, 7600}, {21, 3761, 2, 7600}, {21, 3766, 2, 7600}, {22, 3762, 2, 7600}, {22, 3772, 2, 7600}, {23, 3763, 1, 7600}, {23, 3767, 4, 7600}, {23, 3778, 1, 7600}, {24, 3764, 2, 7600}, {24, 3768, 4, 7600}, {24, 3773, 4, 7600}, {24, 3784, 2, 7600}, {25, 3765, 1, 7600}, {25, 3774, 4, 7600}, {25, 3790, 1, 7600}, {26, 3769, 2, 7600}, {26, 3779, 2, 7600}, {27, 3770, 4, 7600}, {27, 3775, 2, 7600}, {27, 3780, 2, 7600}, {27, 3785, 4, 7600}, {28, 3771, 2, 7600}, {28, 3776, 4, 7600}, {28, 3786, 4, 7600}, {28, 3791, 2,

7600}, {29, 3777, 2, 7600}, {29, 3792, 2, 7600}, {30, 3781, 1, 7600}, {31, 3782, 2, 7600},
{31, 3787, 2, 7600}, {32, 3783, 1, 7600}, {32, 3788, 4, 7600}, {32, 3793, 1, 7600}, {33,
3789, 2, 7600}, {33, 3794, 2, 7600}, {34, 3795, 1, 7600}, {287, 1370, 1, 7601}, {288,
1371, 1, 7601}, {289, 1372, 1, 7601}, {287, 1371, 1, 7602}, {288, 1373, 1, 7602}, {289,
1374, 1, 7602}, {287, 1372, 1, 7603}, {288, 1374, 1, 7603}, {289, 1375, 1, 7603}, {290,
1370, 1, 7604}, {291, 1371, 1, 7604}, {292, 1372, 1, 7604}, {291, 1370, 1, 7605}, {293,
1371, 1, 7605}, {294, 1372, 1, 7605}, {292, 1370, 1, 7606}, {294, 1371, 1, 7606}, {295,
1372, 1, 7606}, {290, 1371, 1, 7607}, {291, 1373, 1, 7607}, {292, 1374, 1, 7607}, {291,
1371, 1, 7608}, {293, 1373, 1, 7608}, {294, 1374, 1, 7608}, {292, 1371, 1, 7609}, {294,
1373, 1, 7609}, {295, 1374, 1, 7609}, {290, 1372, 1, 7610}, {291, 1374, 1, 7610}, {292,
1375, 1, 7610}, {291, 1372, 1, 7611}, {293, 1374, 1, 7611}, {294, 1375, 1, 7611}, {292,
1372, 1, 7612}, {294, 1374, 1, 7612}, {295, 1375, 1, 7612}, {290, 1376, 1, 7613}, {291,
1377, 2, 7613}, {292, 1378, 2, 7613}, {293, 1379, 1, 7613}, {294, 1380, 2, 7613}, {295,
1381, 1, 7613}, {290, 1382, 1, 7614}, {291, 1383, 2, 7614}, {292, 1384, 2, 7614}, {293,
1385, 1, 7614}, {294, 1386, 2, 7614}, {295, 1387, 1, 7614}, {290, 1388, 1, 7615}, {291,
1389, 2, 7615}, {292, 1390, 2, 7615}, {293, 1391, 1, 7615}, {294, 1392, 2, 7615}, {295,
1393, 1, 7615}, {296, 1376, 1, 7616}, {297, 1377, 2, 7616}, {298, 1378, 2, 7616}, {299,
1379, 1, 7616}, {300, 1380, 2, 7616}, {301, 1381, 1, 7616}, {297, 1376, 1, 7617}, {299,
1377, 2, 7617}, {300, 1378, 2, 7617}, {302, 1379, 1, 7617}, {303, 1380, 2, 7617}, {304,
1381, 1, 7617}, {298, 1376, 1, 7618}, {300, 1377, 2, 7618}, {301, 1378, 2, 7618}, {303,
1379, 1, 7618}, {304, 1380, 2, 7618}, {305, 1381, 1, 7618}, {296, 1382, 1, 7619}, {297,
1383, 2, 7619}, {298, 1384, 2, 7619}, {299, 1385, 1, 7619}, {300, 1386, 2, 7619}, {301,
1387, 1, 7619}, {297, 1382, 1, 7620}, {299, 1383, 2, 7620}, {300, 1384, 2, 7620}, {302,
1385, 1, 7620}, {303, 1386, 2, 7620}, {304, 1387, 1, 7620}, {298, 1382, 1, 7621}, {300,
1383, 2, 7621}, {301, 1384, 2, 7621}, {303, 1385, 1, 7621}, {304, 1386, 2, 7621}, {305,
1387, 1, 7621}, {296, 1388, 1, 7622}, {297, 1389, 2, 7622}, {298, 1390, 2, 7622}, {299,
1391, 1, 7622}, {300, 1392, 2, 7622}, {301, 1393, 1, 7622}, {297, 1388, 1, 7623}, {299,
1389, 2, 7623}, {300, 1390, 2, 7623}, {302, 1391, 1, 7623}, {303, 1392, 2, 7623}, {304,
1393, 1, 7623}, {298, 1388, 1, 7624}, {300, 1389, 2, 7624}, {301, 1390, 2, 7624}, {303,
1391, 1, 7624}, {304, 1392, 2, 7624}, {305, 1393, 1, 7624}, {290, 1394, 1, 7625}, {291,
1395, 2, 7625}, {292, 1396, 2, 7625}, {293, 1397, 1, 7625}, {294, 1398, 2, 7625}, {295,
1399, 1, 7625}, {290, 1395, 1, 7626}, {291, 1397, 2, 7626}, {292, 1398, 2, 7626}, {293,
1400, 1, 7626}, {294, 1401, 2, 7626}, {295, 1402, 1, 7626}, {290, 1396, 1, 7627}, {291,
1398, 2, 7627}, {292, 1399, 2, 7627}, {293, 1401, 1, 7627}, {294, 1402, 2, 7627}, {295,
1403, 1, 7627}, {290, 1404, 1, 7628}, {291, 1405, 2, 7628}, {292, 1406, 2, 7628}, {293,
1407, 1, 7628}, {294, 1408, 2, 7628}, {295, 1409, 1, 7628}, {290, 1405, 1, 7629}, {291,
1407, 2, 7629}, {292, 1408, 2, 7629}, {293, 1410, 1, 7629}, {294, 1411, 2, 7629}, {295,
1412, 1, 7629}, {290, 1406, 1, 7630}, {291, 1408, 2, 7630}, {292, 1409, 2, 7630}, {293,
1411, 1, 7630}, {294, 1412, 2, 7630}, {295, 1413, 1, 7630}, {290, 1414, 1, 7631}, {291,
1415, 2, 7631}, {292, 1416, 2, 7631}, {293, 1417, 1, 7631}, {294, 1418, 2, 7631}, {295,
1419, 1, 7631}, {290, 1415, 1, 7632}, {291, 1417, 2, 7632}, {292, 1418, 2, 7632}, {293,
1420, 1, 7632}, {294, 1421, 2, 7632}, {295, 1422, 1, 7632}, {290, 1416, 1, 7633}, {291,
1418, 2, 7633}, {292, 1419, 2, 7633}, {293, 1421, 1, 7633}, {294, 1422, 2, 7633}, {295,
1423, 1, 7633}, {20, 3636, 1, 7634}, {21, 3637, 3, 7634}, {22, 3638, 3, 7634}, {23, 3639,
3, 7634}, {24, 3640, 6, 7634}, {25, 3641, 3, 7634}, {26, 3642, 1, 7634}, {27, 3643, 3,
7634}, {28, 3644, 3, 7634}, {29, 3645, 1, 7634}, {21, 3636, 1, 7635}, {23, 3637, 3, 7635},
{24, 3638, 3, 7635}, {26, 3639, 3, 7635}, {27, 3640, 6, 7635}, {28, 3641, 3, 7635}, {30,
3642, 1, 7635}, {31, 3643, 3, 7635}, {32, 3644, 3, 7635}, {33, 3645, 1, 7635}, {22, 3636,
1, 7636}, {24, 3637, 3, 7636}, {25, 3638, 3, 7636}, {27, 3639, 3, 7636}, {28, 3640, 6,
7636}, {29, 3641, 3, 7636}, {31, 3642, 1, 7636}, {32, 3643, 3, 7636}, {33, 3644, 3, 7636},
{34, 3645, 1, 7636}, {290, 1370, 1, 7637}, {291, 1371, 2, 7637}, {292, 1372, 2, 7637},
{293, 1373, 1, 7637}, {294, 1374, 2, 7637}, {295, 1375, 1, 7637}, {296, 1370, 1, 7638},
{297, 1371, 2, 7638}, {298, 1372, 2, 7638}, {299, 1373, 1, 7638}, {300, 1374, 2, 7638},
{301, 1375, 1, 7638}, {297, 1370, 1, 7639}, {299, 1371, 2, 7639}, {300, 1372, 2, 7639},
{302, 1373, 1, 7639}, {303, 1374, 2, 7639}, {304, 1375, 1, 7639}, {298, 1370, 1, 7640},
{300, 1371, 2, 7640}, {301, 1372, 2, 7640}, {303, 1373, 1, 7640}, {304, 1374, 2, 7640},
{305, 1375, 1, 7640}, {306, 1370, 1, 7641}, {307, 1371, 2, 7641}, {308, 1372, 2, 7641},
{309, 1373, 1, 7641}, {310, 1374, 2, 7641}, {311, 1375, 1, 7641}, {307, 1370, 1, 7642},
{309, 1371, 2, 7642}, {310, 1372, 2, 7642}, {312, 1373, 1, 7642}, {313, 1374, 2, 7642},
{314, 1375, 1, 7642}, {308, 1370, 1, 7643}, {310, 1371, 2, 7643}, {311, 1372, 2, 7643},
{313, 1373, 1, 7643}, {314, 1374, 2, 7643}, {315, 1375, 1, 7643}, {309, 1370, 1, 7644},
{312, 1371, 2, 7644}, {313, 1372, 2, 7644}, {316, 1373, 1, 7644}, {317, 1374, 2, 7644},
{318, 1375, 1, 7644}, {310, 1370, 1, 7645}, {313, 1371, 2, 7645}, {314, 1372, 2, 7645},
{317, 1373, 1, 7645}, {318, 1374, 2, 7645}, {319, 1375, 1, 7645}, {311, 1370, 1, 7646},
{314, 1371, 2, 7646}, {315, 1372, 2, 7646}, {318, 1373, 1, 7646}, {319, 1374, 2, 7646},
{320, 1375, 1, 7646}, {290, 1376, 1, 7647}, {291, 1377, 1, 7647}, {291, 1382, 1, 7647},
{292, 1378, 1, 7647}, {292, 1388, 1, 7647}, {293, 1383, 1, 7647}, {294, 1384, 1, 7647},
{294, 1389, 1, 7647}, {295, 1390, 1, 7647}, {290, 1377, 1, 7648}, {291, 1379, 1, 7648},
{291, 1383, 1, 7648}, {292, 1380, 1, 7648}, {292, 1389, 1, 7648}, {293, 1385, 1, 7648},

{294, 1386, 1, 7648}, {294, 1391, 1, 7648}, {295, 1392, 1, 7648}, {290, 1378, 1, 7649},
{291, 1380, 1, 7649}, {291, 1384, 1, 7649}, {292, 1381, 1, 7649}, {292, 1390, 1, 7649},
{293, 1386, 1, 7649}, {294, 1387, 1, 7649}, {294, 1392, 1, 7649}, {295, 1393, 1, 7649},
{296, 1376, 1, 7650}, {297, 1377, 1, 7650}, {297, 1382, 1, 7650}, {298, 1378, 1, 7650},
{298, 1388, 1, 7650}, {299, 1383, 1, 7650}, {300, 1384, 1, 7650}, {300, 1389, 1, 7650},
{301, 1390, 1, 7650}, {297, 1376, 1, 7651}, {299, 1377, 1, 7651}, {299, 1382, 1, 7651},
{300, 1378, 1, 7651}, {300, 1388, 1, 7651}, {302, 1383, 1, 7651}, {303, 1384, 1, 7651},
{303, 1389, 1, 7651}, {304, 1390, 1, 7651}, {298, 1376, 1, 7652}, {300, 1377, 1, 7652},
{300, 1382, 1, 7652}, {301, 1378, 1, 7652}, {301, 1388, 1, 7652}, {303, 1383, 1, 7652},
{304, 1384, 1, 7652}, {304, 1389, 1, 7652}, {305, 1390, 1, 7652}, {296, 1377, 1, 7653},
{297, 1379, 1, 7653}, {297, 1383, 1, 7653}, {298, 1380, 1, 7653}, {298, 1389, 1, 7653},
{299, 1385, 1, 7653}, {300, 1386, 1, 7653}, {300, 1391, 1, 7653}, {301, 1392, 1, 7653},
{297, 1377, 1, 7654}, {299, 1379, 1, 7654}, {299, 1383, 1, 7654}, {300, 1380, 1, 7654},
{300, 1389, 1, 7654}, {302, 1385, 1, 7654}, {303, 1386, 1, 7654}, {303, 1391, 1, 7654},
{304, 1392, 1, 7654}, {298, 1377, 1, 7655}, {300, 1379, 1, 7655}, {300, 1383, 1, 7655},
{301, 1380, 1, 7655}, {301, 1389, 1, 7655}, {303, 1385, 1, 7655}, {304, 1386, 1, 7655},
{304, 1391, 1, 7655}, {305, 1392, 1, 7655}, {296, 1378, 1, 7656}, {297, 1380, 1, 7656},
{297, 1384, 1, 7656}, {298, 1381, 1, 7656}, {298, 1390, 1, 7656}, {299, 1386, 1, 7656},
{300, 1387, 1, 7656}, {300, 1392, 1, 7656}, {301, 1393, 1, 7656}, {297, 1378, 1, 7657},
{299, 1380, 1, 7657}, {299, 1384, 1, 7657}, {300, 1381, 1, 7657}, {300, 1390, 1, 7657},
{302, 1386, 1, 7657}, {303, 1387, 1, 7657}, {303, 1392, 1, 7657}, {304, 1393, 1, 7657},
{298, 1378, 1, 7658}, {300, 1380, 1, 7658}, {300, 1384, 1, 7658}, {301, 1381, 1, 7658},
{301, 1390, 1, 7658}, {303, 1386, 1, 7658}, {304, 1387, 1, 7658}, {304, 1392, 1, 7658},
{305, 1393, 1, 7658}, {296, 1376, 1, 7659}, {297, 1377, 2, 7659}, {297, 1382, 1, 7659},
{298, 1378, 2, 7659}, {298, 1388, 1, 7659}, {299, 1379, 1, 7659}, {299, 1383, 2, 7659},
{300, 1380, 2, 7659}, {300, 1384, 2, 7659}, {300, 1389, 2, 7659}, {301, 1381, 1, 7659},
{301, 1390, 2, 7659}, {302, 1385, 1, 7659}, {303, 1386, 2, 7659}, {303, 1391, 1, 7659},
{304, 1387, 1, 7659}, {304, 1392, 2, 7659}, {305, 1393, 1, 7659}, {306, 1376, 1, 7660},
{307, 1377, 2, 7660}, {307, 1382, 1, 7660}, {308, 1378, 2, 7660}, {308, 1388, 1, 7660},
{309, 1379, 1, 7660}, {309, 1383, 2, 7660}, {310, 1380, 2, 7660}, {310, 1384, 2, 7660},
{310, 1389, 2, 7660}, {311, 1381, 1, 7660}, {311, 1390, 2, 7660}, {312, 1385, 1, 7660},
{313, 1386, 2, 7660}, {313, 1391, 1, 7660}, {314, 1387, 1, 7660}, {314, 1392, 2, 7660},
{315, 1393, 1, 7660}, {307, 1376, 1, 7661}, {309, 1377, 2, 7661}, {309, 1382, 1, 7661},
{310, 1378, 2, 7661}, {310, 1388, 1, 7661}, {312, 1379, 1, 7661}, {312, 1383, 2, 7661},
{313, 1380, 2, 7661}, {313, 1384, 2, 7661}, {313, 1389, 2, 7661}, {314, 1381, 1, 7661},
{314, 1390, 2, 7661}, {316, 1385, 1, 7661}, {317, 1386, 2, 7661}, {317, 1391, 1, 7661},
{318, 1387, 1, 7661}, {318, 1392, 2, 7661}, {319, 1393, 1, 7661}, {308, 1376, 1, 7662},
{310, 1377, 2, 7662}, {310, 1382, 1, 7662}, {311, 1378, 2, 7662}, {311, 1388, 1, 7662},
{313, 1379, 1, 7662}, {313, 1383, 2, 7662}, {314, 1380, 2, 7662}, {314, 1384, 2, 7662},
{314, 1389, 2, 7662}, {315, 1381, 1, 7662}, {315, 1390, 2, 7662}, {317, 1385, 1, 7662},
{318, 1386, 2, 7662}, {318, 1391, 1, 7662}, {319, 1387, 1, 7662}, {319, 1392, 2, 7662},
{320, 1393, 1, 7662}, {290, 1394, 1, 7663}, {291, 1395, 1, 7663}, {291, 1404, 1, 7663},
{292, 1396, 1, 7663}, {292, 1414, 1, 7663}, {293, 1405, 1, 7663}, {294, 1406, 1, 7663},
{294, 1415, 1, 7663}, {295, 1416, 1, 7663}, {290, 1395, 1, 7664}, {291, 1397, 1, 7664},
{291, 1405, 1, 7664}, {292, 1398, 1, 7664}, {292, 1415, 1, 7664}, {293, 1407, 1, 7664},
{294, 1408, 1, 7664}, {294, 1417, 1, 7664}, {295, 1418, 1, 7664}, {290, 1396, 1, 7665},
{291, 1398, 1, 7665}, {291, 1406, 1, 7665}, {292, 1399, 1, 7665}, {292, 1416, 1, 7665},
{293, 1408, 1, 7665}, {294, 1409, 1, 7665}, {294, 1418, 1, 7665}, {295, 1419, 1, 7665},
{290, 1397, 1, 7666}, {291, 1400, 1, 7666}, {291, 1407, 1, 7666}, {292, 1401, 1, 7666},
{292, 1417, 1, 7666}, {293, 1410, 1, 7666}, {294, 1411, 1, 7666}, {294, 1420, 1, 7666},
{295, 1421, 1, 7666}, {290, 1398, 1, 7667}, {291, 1401, 1, 7667}, {291, 1408, 1, 7667},
{292, 1402, 1, 7667}, {292, 1418, 1, 7667}, {293, 1411, 1, 7667}, {294, 1412, 1, 7667},
{294, 1421, 1, 7667}, {295, 1422, 1, 7667}, {290, 1399, 1, 7668}, {291, 1402, 1, 7668},
{291, 1409, 1, 7668}, {292, 1403, 1, 7668}, {292, 1419, 1, 7668}, {293, 1412, 1, 7668},
{294, 1413, 1, 7668}, {294, 1422, 1, 7668}, {295, 1423, 1, 7668}, {296, 1394, 1, 7669},
{297, 1395, 2, 7669}, {297, 1404, 1, 7669}, {298, 1396, 2, 7669}, {298, 1414, 1, 7669},
{299, 1397, 1, 7669}, {299, 1405, 2, 7669}, {300, 1398, 2, 7669}, {300, 1406, 2, 7669},
{300, 1415, 2, 7669}, {301, 1399, 1, 7669}, {301, 1416, 2, 7669}, {302, 1407, 1, 7669},
{303, 1408, 2, 7669}, {303, 1417, 1, 7669}, {304, 1409, 1, 7669}, {304, 1418, 2, 7669},
{305, 1419, 1, 7669}, {296, 1395, 1, 7670}, {297, 1397, 2, 7670}, {297, 1405, 1, 7670},
{298, 1398, 2, 7670}, {298, 1415, 1, 7670}, {299, 1400, 1, 7670}, {299, 1407, 2, 7670},
{300, 1401, 2, 7670}, {300, 1408, 2, 7670}, {300, 1417, 2, 7670}, {301, 1402, 1, 7670},
{301, 1418, 2, 7670}, {302, 1410, 1, 7670}, {303, 1411, 2, 7670}, {303, 1420, 1, 7670},
{304, 1412, 1, 7670}, {304, 1421, 2, 7670}, {305, 1422, 1, 7670}, {296, 1396, 1, 7671},
{297, 1398, 2, 7671}, {297, 1406, 1, 7671}, {298, 1399, 2, 7671}, {298, 1416, 1, 7671},
{299, 1401, 1, 7671}, {299, 1408, 2, 7671}, {300, 1402, 2, 7671}, {300, 1409, 2, 7671},
{300, 1418, 2, 7671}, {301, 1403, 1, 7671}, {301, 1419, 2, 7671}, {302, 1411, 1, 7671},
{303, 1412, 2, 7671}, {303, 1421, 1, 7671}, {304, 1413, 1, 7671}, {304, 1422, 2, 7671},
{305, 1423, 1, 7671}, {20, 3646, 1, 7672}, {21, 3647, 1, 7672}, {21, 3649, 3, 7672}, {22,

3648, 1, 7672}, {22, 3652, 3, 7672}, {23, 3650, 3, 7672}, {23, 3655, 3, 7672}, {24, 3651,
3, 7672}, {24, 3653, 3, 7672}, {24, 3658, 6, 7672}, {25, 3654, 3, 7672}, {25, 3661, 3,
7672}, {26, 3656, 3, 7672}, {26, 3664, 1, 7672}, {27, 3657, 3, 7672}, {27, 3659, 6, 7672},
{27, 3667, 3, 7672}, {28, 3660, 6, 7672}, {28, 3662, 3, 7672}, {28, 3670, 3, 7672}, {29,
3663, 3, 7672}, {29, 3673, 1, 7672}, {30, 3665, 1, 7672}, {31, 3666, 1, 7672}, {31, 3668,
3, 7672}, {32, 3669, 3, 7672}, {32, 3671, 3, 7672}, {33, 3672, 3, 7672}, {33, 3674, 1,
7672}, {34, 3675, 1, 7672}, {20, 3861, 1, 7673}, {21, 3862, 1, 7673}, {21, 3864, 3, 7673},
{22, 3863, 1, 7673}, {22, 3867, 3, 7673}, {23, 3865, 3, 7673}, {23, 3870, 3, 7673}, {24,
3866, 3, 7673}, {24, 3868, 3, 7673}, {24, 3873, 6, 7673}, {25, 3869, 3, 7673}, {25, 3876,
3, 7673}, {26, 3871, 3, 7673}, {26, 3879, 1, 7673}, {27, 3872, 3, 7673}, {27, 3874, 6,
7673}, {27, 3882, 3, 7673}, {28, 3875, 6, 7673}, {28, 3877, 3, 7673}, {28, 3885, 3, 7673},
{29, 3878, 3, 7673}, {29, 3888, 1, 7673}, {30, 3880, 1, 7673}, {31, 3881, 1, 7673}, {31,
3883, 3, 7673}, {32, 3884, 3, 7673}, {32, 3886, 3, 7673}, {33, 3887, 3, 7673}, {33, 3889,
1, 7673}, {34, 3890, 1, 7673}, {286, 1532, 1, 7674}, {287, 1533, 1, 7675}, {288, 1534, 1,
7675}, {289, 1535, 1, 7675}, {290, 1533, 1, 7676}, {291, 1534, 1, 7676}, {292, 1535, 1,
7676}, {291, 1533, 1, 7677}, {293, 1534, 1, 7677}, {294, 1535, 1, 7677}, {292, 1533, 1,
7678}, {294, 1534, 1, 7678}, {295, 1535, 1, 7678}, {287, 1536, 1, 7679}, {288, 1537, 1,
7679}, {289, 1538, 1, 7679}, {287, 1537, 1, 7680}, {288, 1539, 1, 7680}, {289, 1540, 1,
7680}, {287, 1538, 1, 7681}, {288, 1540, 1, 7681}, {289, 1541, 1, 7681}, {290, 1536, 1,
7682}, {291, 1537, 1, 7682}, {292, 1538, 1, 7682}, {291, 1536, 1, 7683}, {293, 1537, 1,
7683}, {294, 1538, 1, 7683}, {292, 1536, 1, 7684}, {294, 1537, 1, 7684}, {295, 1538, 1,
7684}, {290, 1537, 1, 7685}, {291, 1539, 1, 7685}, {292, 1540, 1, 7685}, {291, 1537, 1,
7686}, {293, 1539, 1, 7686}, {294, 1540, 1, 7686}, {292, 1537, 1, 7687}, {294, 1539, 1,
7687}, {295, 1540, 1, 7687}, {290, 1538, 1, 7688}, {291, 1540, 1, 7688}, {292, 1541, 1,
7688}, {291, 1538, 1, 7689}, {293, 1540, 1, 7689}, {294, 1541, 1, 7689}, {292, 1538, 1,
7690}, {294, 1540, 1, 7690}, {295, 1541, 1, 7690}, {290, 1536, 1, 7691}, {291, 1537, 2,
7691}, {292, 1538, 2, 7691}, {293, 1539, 1, 7691}, {294, 1540, 2, 7691}, {295, 1541, 1,
7691}, {296, 1536, 1, 7692}, {297, 1537, 2, 7692}, {298, 1538, 2, 7692}, {299, 1539, 1,
7692}, {300, 1540, 2, 7692}, {301, 1541, 1, 7692}, {297, 1536, 1, 7693}, {299, 1537, 2,
7693}, {300, 1538, 2, 7693}, {302, 1539, 1, 7693}, {303, 1540, 2, 7693}, {304, 1541, 1,
7693}, {298, 1536, 1, 7694}, {300, 1537, 2, 7694}, {301, 1538, 2, 7694}, {303, 1539, 1,
7694}, {304, 1540, 2, 7694}, {305, 1541, 1, 7694}, {290, 1542, 1, 7695}, {291, 1543, 2,
7695}, {292, 1544, 2, 7695}, {293, 1545, 1, 7695}, {294, 1546, 2, 7695}, {295, 1547, 1,
7695}, {290, 1543, 1, 7696}, {291, 1545, 2, 7696}, {292, 1546, 2, 7696}, {293, 1548, 1,
7696}, {294, 1549, 2, 7696}, {295, 1550, 1, 7696}, {290, 1544, 1, 7697}, {291, 1546, 2,
7697}, {292, 1547, 2, 7697}, {293, 1549, 1, 7697}, {294, 1550, 2, 7697}, {295, 1551, 1,
7697}, {296, 1542, 1, 7698}, {297, 1543, 3, 7698}, {298, 1544, 3, 7698}, {299, 1545, 3,
7698}, {300, 1546, 6, 7698}, {301, 1547, 3, 7698}, {302, 1548, 1, 7698}, {303, 1549, 3,
7698}, {304, 1550, 3, 7698}, {305, 1551, 1, 7698}, {20, 3931, 1, 7699}, {21, 3932, 4,
7699}, {22, 3933, 4, 7699}, {23, 3934, 6, 7699}, {24, 3935, 12, 7699}, {25, 3936, 6,
7699}, {26, 3937, 4, 7699}, {27, 3938, 12, 7699}, {28, 3939, 12, 7699}, {29, 3940, 4,
7699}, {30, 3941, 1, 7699}, {31, 3942, 4, 7699}, {32, 3943, 6, 7699}, {33, 3944, 4, 7699},
{34, 3945, 1, 7699}, {35, 3436, 1, 7700}, {36, 3437, 3, 7700}, {36, 3446, 2, 7700}, {37,
3438, 3, 7700}, {37, 3456, 2, 7700}, {38, 3439, 3, 7700}, {38, 3447, 6, 7700}, {38, 3466,
1, 7700}, {39, 3440, 6, 7700}, {39, 3448, 6, 7700}, {39, 3457, 6, 7700}, {39, 3476, 2,
7700}, {40, 3441, 3, 7700}, {40, 3458, 6, 7700}, {40, 3486, 1, 7700}, {41, 3442, 1, 7700},
{41, 3449, 6, 7700}, {41, 3467, 3, 7700}, {42, 3443, 3, 7700}, {42, 3450, 12, 7700}, {42,
3459, 6, 7700}, {42, 3468, 3, 7700}, {42, 3477, 6, 7700}, {43, 3444, 3, 7700}, {43, 3451,
6, 7700}, {43, 3460, 12, 7700}, {43, 3478, 6, 7700}, {43, 3487, 3, 7700}, {44, 3445, 1,
7700}, {44, 3461, 6, 7700}, {44, 3488, 3, 7700}, {45, 3452, 2, 7700}, {45, 3469, 3, 7700},
{46, 3453, 6, 7700}, {46, 3462, 2, 7700}, {46, 3470, 6, 7700}, {46, 3479, 6, 7700}, {47,
3454, 6, 7700}, {47, 3463, 6, 7700}, {47, 3471, 3, 7700}, {47, 3480, 12, 7700}, {47, 3489,
3, 7700}, {48, 3455, 2, 7700}, {48, 3464, 6, 7700}, {48, 3481, 6, 7700}, {48, 3490, 6,
7700}, {49, 3465, 2, 7700}, {49, 3491, 3, 7700}, {50, 3472, 1, 7700}, {51, 3473, 3, 7700},
{51, 3482, 2, 7700}, {52, 3474, 3, 7700}, {52, 3483, 6, 7700}, {52, 3492, 1, 7700}, {53,
3475, 1, 7700}, {53, 3484, 6, 7700}, {53, 3493, 3, 7700}, {54, 3485, 2, 7700}, {54, 3494,
3, 7700}, {55, 3495, 1, 7700}, {290, 1793, 1, 7701}, {291, 1794, 2, 7701}, {292, 1795, 2,
7701}, {293, 1796, 1, 7701}, {294, 1797, 2, 7701}, {295, 1798, 1, 7701}, {290, 1794, 1,
7702}, {291, 1799, 2, 7702}, {292, 1800, 2, 7702}, {293, 1801, 1, 7702}, {294, 1802, 2,
7702}, {295, 1803, 1, 7702}, {290, 1795, 1, 7703}, {291, 1800, 2, 7703}, {292, 1804, 2,
7703}, {293, 1805, 1, 7703}, {294, 1806, 2, 7703}, {295, 1807, 1, 7703}, {290, 1796, 1,
7704}, {291, 1801, 2, 7704}, {292, 1805, 2, 7704}, {293, 1808, 1, 7704}, {294, 1809, 2,
7704}, {295, 1810, 1, 7704}, {290, 1797, 1, 7705}, {291, 1802, 2, 7705}, {292, 1806, 2,
7705}, {293, 1809, 1, 7705}, {294, 1811, 2, 7705}, {295, 1812, 1, 7705}, {290, 1798, 1,
7706}, {291, 1803, 2, 7706}, {292, 1807, 2, 7706}, {293, 1810, 1, 7706}, {294, 1812, 2,
7706}, {295, 1813, 1, 7706}, {290, 1793, 1, 7707}, {291, 1794, 2, 7707}, {292, 1795, 2,
7707}, {293, 1799, 1, 7707}, {294, 1800, 2, 7707}, {295, 1804, 1, 7707}, {290, 1794, 1,
7708}, {291, 1796, 1, 7708}, {291, 1799, 1, 7708}, {292, 1797, 1, 7708}, {292, 1800, 1,
7708}, {293, 1801, 1, 7708}, {294, 1802, 1, 7708}, {294, 1805, 1, 7708}, {295, 1806, 1,

7708}, {290, 1795, 1, 7709}, {291, 1797, 1, 7709}, {291, 1800, 1, 7709}, {292, 1798, 1, 7709}, {292, 1804, 1, 7709}, {293, 1802, 1, 7709}, {294, 1803, 1, 7709}, {294, 1806, 1, 7709}, {295, 1807, 1, 7709}, {290, 1799, 1, 7710}, {291, 1801, 2, 7710}, {292, 1802, 2, 7710}, {293, 1808, 1, 7710}, {294, 1809, 2, 7710}, {295, 1811, 1, 7710}, {290, 1800, 1, 7711}, {291, 1802, 1, 7711}, {291, 1805, 1, 7711}, {292, 1803, 1, 7711}, {292, 1806, 1, 7711}, {293, 1809, 1, 7711}, {294, 1810, 1, 7711}, {294, 1811, 1, 7711}, {295, 1812, 1, 7711}, {290, 1804, 1, 7712}, {291, 1806, 2, 7712}, {292, 1807, 2, 7712}, {293, 1811, 1, 7712}, {294, 1812, 2, 7712}, {295, 1813, 1, 7712}, {296, 1793, 1, 7713}, {297, 1794, 3, 7713}, {298, 1795, 3, 7713}, {299, 1796, 1, 7713}, {299, 1799, 2, 7713}, {300, 1797, 2, 7713}, {300, 1800, 4, 7713}, {301, 1798, 1, 7713}, {301, 1804, 2, 7713}, {302, 1801, 1, 7713}, {303, 1802, 2, 7713}, {303, 1805, 1, 7713}, {304, 1803, 1, 7713}, {304, 1806, 2, 7713}, {305, 1807, 1, 7713}, {296, 1794, 1, 7714}, {297, 1796, 1, 7714}, {297, 1799, 2, 7714}, {298, 1797, 1, 7714}, {298, 1800, 2, 7714}, {299, 1801, 3, 7714}, {300, 1802, 4, 7714}, {300, 1805, 2, 7714}, {301, 1803, 1, 7714}, {301, 1806, 2, 7714}, {302, 1808, 1, 7714}, {303, 1809, 3, 7714}, {304, 1810, 1, 7714}, {304, 1811, 2, 7714}, {305, 1812, 1, 7714}, {296, 1795, 1, 7715}, {297, 1797, 1, 7715}, {297, 1800, 2, 7715}, {298, 1798, 1, 7715}, {298, 1804, 2, 7715}, {299, 1802, 2, 7715}, {299, 1805, 1, 7715}, {300, 1803, 2, 7715}, {300, 1806, 4, 7715}, {301, 1807, 3, 7715}, {302, 1809, 1, 7715}, {303, 1810, 1, 7715}, {303, 1811, 2, 7715}, {304, 1812, 3, 7715}, {305, 1813, 1, 7715}, {306, 1793, 1, 7716}, {307, 1794, 4, 7716}, {308, 1795, 4, 7716}, {309, 1796, 2, 7716}, {309, 1799, 4, 7716}, {310, 1797, 4, 7716}, {310, 1800, 8, 7716}, {311, 1798, 2, 7716}, {311, 1804, 4, 7716}, {312, 1801, 4, 7716}, {313, 1802, 8, 7716}, {313, 1805, 4, 7716}, {314, 1803, 4, 7716}, {314, 1806, 8, 7716}, {315, 1807, 4, 7716}, {316, 1808, 1, 7716}, {317, 1809, 4, 7716}, {318, 1810, 2, 7716}, {318, 1811, 4, 7716}, {319, 1812, 4, 7716}, {320, 1813, 1, 7716}, {35, 3676, 1, 7717}, {36, 3677, 2, 7717}, {36, 3682, 3, 7717}, {37, 3678, 2, 7717}, {37, 3688, 3, 7717}, {38, 3679, 1, 7717}, {38, 3683, 6, 7717}, {38, 3694, 3, 7717}, {39, 3680, 2, 7717}, {39, 3684, 6, 7717}, {39, 3689, 6, 7717}, {39, 3700, 6, 7717}, {40, 3681, 1, 7717}, {40, 3690, 6, 7717}, {40, 3706, 3, 7717}, {41, 3685, 3, 7717}, {41, 3695, 6, 7717}, {41, 3712, 1, 7717}, {42, 3686, 6, 7717}, {42, 3691, 3, 7717}, {42, 3696, 6, 7717}, {42, 3701, 12, 7717}, {42, 3718, 3, 7717}, {43, 3687, 3, 7717}, {43, 3692, 6, 7717}, {43, 3702, 12, 7717}, {43, 3707, 6, 7717}, {43, 3724, 3, 7717}, {44, 3693, 3, 7717}, {44, 3708, 6, 7717}, {44, 3730, 1, 7717}, {45, 3697, 3, 7717}, {45, 3713, 2, 7717}, {46, 3698, 6, 7717}, {46, 3703, 6, 7717}, {46, 3714, 2, 7717}, {46, 3719, 6, 7717}, {47, 3699, 3, 7717}, {47, 3704, 12, 7717}, {47, 3709, 3, 7717}, {47, 3720, 6, 7717}, {47, 3725, 6, 7717}, {48, 3705, 6, 7717}, {48, 3710, 6, 7717}, {48, 3726, 6, 7717}, {48, 3731, 2, 7717}, {49, 3711, 3, 7717}, {49, 3732, 2, 7717}, {50, 3715, 1, 7717}, {51, 3716, 2, 7717}, {51, 3721, 3, 7717}, {52, 3717, 1, 7717}, {52, 3722, 6, 7717}, {52, 3727, 3, 7717}, {53, 3723, 3, 7717}, {53, 3728, 6, 7717}, {53, 3733, 1, 7717}, {54, 3729, 3, 7717}, {54, 3734, 2, 7717}, {55, 3735, 1, 7717}, {287, 1964, 1, 7718}, {288, 1965, 1, 7718}, {289, 1966, 1, 7718}, {287, 1965, 1, 7719}, {288, 1967, 1, 7719}, {289, 1968, 1, 7719}, {287, 1966, 1, 7720}, {288, 1968, 1, 7720}, {289, 1969, 1, 7720}, {290, 1964, 1, 7721}, {291, 1965, 1, 7721}, {292, 1966, 1, 7721}, {291, 1964, 1, 7722}, {293, 1965, 1, 7722}, {294, 1966, 1, 7722}, {292, 1964, 1, 7723}, {294, 1965, 1, 7723}, {295, 1966, 1, 7723}, {290, 1965, 1, 7724}, {291, 1967, 1, 7724}, {292, 1968, 1, 7724}, {291, 1965, 1, 7725}, {293, 1967, 1, 7725}, {294, 1968, 1, 7725}, {292, 1965, 1, 7726}, {294, 1967, 1, 7726}, {295, 1968, 1, 7726}, {290, 1966, 1, 7727}, {291, 1968, 1, 7727}, {292, 1969, 1, 7727}, {291, 1966, 1, 7728}, {293, 1968, 1, 7728}, {294, 1969, 1, 7728}, {292, 1966, 1, 7729}, {294, 1968, 1, 7729}, {295, 1969, 1, 7729}, {290, 1970, 1, 7730}, {291, 1971, 2, 7730}, {292, 1972, 2, 7730}, {293, 1973, 1, 7730}, {294, 1974, 2, 7730}, {295, 1975, 1, 7730}, {290, 1976, 1, 7731}, {291, 1977, 2, 7731}, {292, 1978, 2, 7731}, {293, 1979, 1, 7731}, {294, 1980, 2, 7731}, {295, 1981, 1, 7731}, {290, 1982, 1, 7732}, {291, 1983, 2, 7732}, {292, 1984, 2, 7732}, {293, 1985, 1, 7732}, {294, 1986, 2, 7732}, {295, 1987, 1, 7732}, {290, 1964, 1, 7733}, {291, 1965, 2, 7733}, {292, 1966, 2, 7733}, {293, 1967, 1, 7733}, {294, 1968, 2, 7733}, {295, 1969, 1, 7733}, {296, 1964, 1, 7734}, {297, 1965, 2, 7734}, {298, 1966, 2, 7734}, {299, 1967, 1, 7734}, {300, 1968, 2, 7734}, {301, 1969, 1, 7734}, {297, 1964, 1, 7735}, {299, 1965, 2, 7735}, {300, 1966, 2, 7735}, {302, 1967, 1, 7735}, {303, 1968, 2, 7735}, {304, 1969, 1, 7735}, {298, 1964, 1, 7736}, {300, 1965, 2, 7736}, {301, 1966, 2, 7736}, {303, 1967, 1, 7736}, {304, 1968, 2, 7736}, {305, 1969, 1, 7736}, {290, 1970, 1, 7737}, {291, 1971, 1, 7737}, {291, 1976, 1, 7737}, {292, 1972, 1, 7737}, {292, 1982, 1, 7737}, {293, 1977, 1, 7737}, {294, 1978, 1, 7737}, {294, 1983, 1, 7737}, {295, 1984, 1, 7737}, {290, 1971, 1, 7738}, {291, 1973, 1, 7738}, {291, 1977, 1, 7738}, {292, 1974, 1, 7738}, {292, 1983, 1, 7738}, {293, 1979, 1, 7738}, {294, 1980, 1, 7738}, {294, 1985, 1, 7738}, {295, 1986, 1, 7738}, {290, 1972, 1, 7739}, {291, 1974, 1, 7739}, {291, 1978, 1, 7739}, {292, 1975, 1, 7739}, {292, 1984, 1, 7739}, {293, 1980, 1, 7739}, {294, 1981, 1, 7739}, {294, 1986, 1, 7739}, {295, 1987, 1, 7739}, {296, 1970, 1, 7740}, {297, 1971, 2, 7740}, {297, 1976, 1, 7740}, {298, 1972, 2, 7740}, {298, 1982, 1, 7740}, {299, 1973, 1, 7740}, {299, 1977, 2, 7740}, {300, 1974, 2, 7740}, {300, 1978, 2, 7740}, {300, 1983, 2, 7740}, {301, 1975, 1, 7740}, {301, 1984, 2, 7740}, {302, 1979, 1, 7740}, {303, 1980, 2, 7740}, {303, 1985, 1, 7740}, {304, 1981, 1, 7740}, {304, 1986, 2, 7740}, {305, 1987, 1, 7740}, {306,

1988, 1, 7741}, {307, 1989, 3, 7741}, {307, 1998, 1, 7741}, {308, 1990, 3, 7741}, {308, 2008, 1, 7741}, {309, 1991, 3, 7741}, {309, 1999, 3, 7741}, {310, 1992, 6, 7741}, {310, 2000, 3, 7741}, {310, 2009, 3, 7741}, {311, 1993, 3, 7741}, {311, 2010, 3, 7741}, {312, 1994, 1, 7741}, {312, 2001, 3, 7741}, {313, 1995, 3, 7741}, {313, 2002, 6, 7741}, {313, 2011, 3, 7741}, {314, 1996, 3, 7741}, {314, 2003, 3, 7741}, {314, 2012, 6, 7741}, {315, 1997, 1, 7741}, {315, 2013, 3, 7741}, {316, 2004, 1, 7741}, {317, 2005, 3, 7741}, {317, 2014, 1, 7741}, {318, 2006, 3, 7741}, {318, 2015, 3, 7741}, {319, 2007, 1, 7741}, {319, 2016, 3, 7741}, {320, 2017, 1, 7741}, {286, 2063, 1, 7742}, {287, 2064, 1, 7743}, {288, 2065, 1, 7743}, {289, 2066, 1, 7743}, {290, 2064, 1, 7744}, {291, 2065, 1, 7744}, {292, 2066, 1, 7744}, {291, 2064, 1, 7745}, {293, 2065, 1, 7745}, {294, 2066, 1, 7745}, {292, 2064, 1, 7746}, {294, 2065, 1, 7746}, {295, 2066, 1, 7746}, {287, 2067, 1, 7747}, {288, 2068, 1, 7747}, {289, 2069, 1, 7747}, {287, 2068, 1, 7748}, {288, 2070, 1, 7748}, {289, 2071, 1, 7748}, {287, 2069, 1, 7749}, {288, 2071, 1, 7749}, {289, 2072, 1, 7749}, {290, 2067, 1, 7750}, {291, 2068, 2, 7750}, {292, 2069, 2, 7750}, {293, 2070, 1, 7750}, {294, 2071, 2, 7750}, {295, 2072, 1, 7750}, {296, 2073, 1, 7751}, {297, 2074, 3, 7751}, {298, 2075, 3, 7751}, {299, 2076, 3, 7751}, {300, 2077, 6, 7751}, {301, 2078, 3, 7751}, {302, 2079, 1, 7751}, {303, 2080, 3, 7751}, {304, 2081, 3, 7751}, {305, 2082, 1, 7751}, {306, 2174, 1, 7752}, {307, 2175, 4, 7752}, {308, 2176, 4, 7752}, {309, 2177, 2, 7752}, {309, 2180, 4, 7752}, {310, 2178, 4, 7752}, {310, 2181, 8, 7752}, {311, 2179, 2, 7752}, {311, 2185, 4, 7752}, {312, 2182, 4, 7752}, {313, 2183, 8, 7752}, {313, 2186, 4, 7752}, {314, 2184, 4, 7752}, {314, 2187, 8, 7752}, {315, 2188, 4, 7752}, {316, 2189, 1, 7752}, {317, 2190, 4, 7752}, {318, 2191, 2, 7752}, {318, 2192, 4, 7752}, {319, 2193, 4, 7752}, {320, 2194, 1, 7752}, {287, 2345, 1, 7753}, {288, 2346, 1, 7753}, {289, 2347, 1, 7753}, {287, 2346, 1, 7754}, {288, 2348, 1, 7754}, {289, 2349, 1, 7754}, {287, 2347, 1, 7755}, {288, 2349, 1, 7755}, {289, 2350, 1, 7755}, {290, 2345, 1, 7756}, {291, 2346, 2, 7756}, {292, 2347, 2, 7756}, {293, 2348, 1, 7756}, {294, 2349, 2, 7756}, {295, 2350, 1, 7756}, {296, 2351, 1, 7757}, {297, 2352, 2, 7757}, {297, 2357, 1, 7757}, {298, 2353, 2, 7757}, {298, 2363, 1, 7757}, {299, 2354, 1, 7757}, {299, 2358, 2, 7757}, {300, 2355, 2, 7757}, {300, 2359, 2, 7757}, {300, 2364, 2, 7757}, {301, 2356, 1, 7757}, {301, 2365, 2, 7757}, {302, 2360, 1, 7757}, {303, 2361, 2, 7757}, {303, 2366, 1, 7757}, {304, 2362, 1, 7757}, {304, 2367, 2, 7757}, {305, 2368, 1, 7757}, {286, 2444, 1, 7758}, {287, 2445, 1, 7759}, {288, 2446, 1, 7759}, {289, 2447, 1, 7759}, {290, 2448, 1, 7760}, {291, 2449, 2, 7760}, {292, 2450, 2, 7760}, {293, 2451, 1, 7760}, {294, 2452, 2, 7760}, {295, 2453, 1, 7760}, {290, 2615, 1, 7761}, {291, 2616, 2, 7761}, {292, 2617, 2, 7761}, {293, 2618, 1, 7761}, {294, 2619, 2, 7761}, {295, 2620, 1, 7761}, {286, 2669, 1, 7762}, {287, 2670, 1, 7763}, {288, 2671, 1, 7763}, {289, 2672, 1, 7763}, {286, 2734, 1, 7764}, {0, 3967, 1, 7765}, {0, 3968, 1, 7766}, {0, 4038, 1, 7767}, {0, 4148, 1, 7768}, {0, 4258, 1, 7769}, {0, 4314, 1, 7770}, {0, 4337, 1, 7771}, {1, 3969, 1, 7772}, {2, 3970, 1, 7772}, {3, 3971, 1, 7772}, {1, 3972, 1, 7773}, {2, 3973, 1, 7773}, {3, 3974, 1, 7773}, {1, 3973, 1, 7774}, {2, 3975, 1, 7774}, {3, 3976, 1, 7774}, {1, 3974, 1, 7775}, {2, 3976, 1, 7775}, {3, 3977, 1, 7775}, {1, 4039, 1, 7776}, {2, 4040, 1, 7776}, {3, 4041, 1, 7776}, {1, 4042, 1, 7777}, {2, 4043, 1, 7777}, {3, 4044, 1, 7777}, {1, 4043, 1, 7778}, {2, 4045, 1, 7778}, {3, 4046, 1, 7778}, {1, 4044, 1, 7779}, {2, 4046, 1, 7779}, {3, 4047, 1, 7779}, {1, 4149, 1, 7780}, {2, 4150, 1, 7780}, {3, 4151, 1, 7780}, {1, 4152, 1, 7781}, {2, 4153, 1, 7781}, {3, 4154, 1, 7781}, {1, 4153, 1, 7782}, {2, 4155, 1, 7782}, {3, 4156, 1, 7782}, {1, 4154, 1, 7783}, {2, 4156, 1, 7783}, {3, 4157, 1, 7783}, {1, 4259, 1, 7784}, {2, 4260, 1, 7784}, {3, 4261, 1, 7784}, {1, 4315, 1, 7785}, {2, 4316, 1, 7785}, {3, 4317, 1, 7785}, {506, 707, 1, 7786}, {1, 3978, 1, 7787}, {2, 3979, 1, 7787}, {3, 3980, 1, 7787}, {1, 3981, 1, 7788}, {2, 3984, 1, 7788}, {3, 3987, 1, 7788}, {1, 3982, 1, 7789}, {2, 3985, 1, 7789}, {3, 3988, 1, 7789}, {1, 3983, 1, 7790}, {2, 3986, 1, 7790}, {3, 3989, 1, 7790}, {1, 4058, 1, 7791}, {2, 4059, 1, 7791}, {3, 4060, 1, 7791}, {1, 4059, 1, 7792}, {2, 4061, 1, 7792}, {3, 4062, 1, 7792}, {1, 4060, 1, 7793}, {2, 4062, 1, 7793}, {3, 4063, 1, 7793}, {1, 4118, 1, 7794}, {2, 4119, 1, 7794}, {3, 4120, 1, 7794}, {1, 4121, 1, 7795}, {2, 4124, 1, 7795}, {3, 4127, 1, 7795}, {1, 4122, 1, 7796}, {2, 4125, 1, 7796}, {3, 4128, 1, 7796}, {1, 4123, 1, 7797}, {2, 4126, 1, 7797}, {3, 4129, 1, 7797}, {1, 4168, 1, 7798}, {2, 4169, 1, 7798}, {3, 4170, 1, 7798}, {1, 4169, 1, 7799}, {2, 4171, 1, 7799}, {3, 4172, 1, 7799}, {1, 4170, 1, 7800}, {2, 4172, 1, 7800}, {3, 4173, 1, 7800}, {1, 4228, 1, 7801}, {2, 4229, 1, 7801}, {3, 4230, 1, 7801}, {1, 4231, 1, 7802}, {2, 4234, 1, 7802}, {3, 4237, 1, 7802}, {1, 4232, 1, 7803}, {2, 4235, 1, 7803}, {3, 4238, 1, 7803}, {1, 4233, 1, 7804}, {2, 4236, 1, 7804}, {3, 4239, 1, 7804}, {1, 4278, 1, 7805}, {2, 4279, 1, 7805}, {3, 4280, 1, 7805}, {1, 4279, 1, 7806}, {2, 4281, 1, 7806}, {3, 4282, 1, 7806}, {1, 4280, 1, 7807}, {2, 4282, 1, 7807}, {3, 4283, 1, 7807}, {1, 4302, 1, 7808}, {2, 4303, 1, 7808}, {3, 4304, 1, 7808}, {1, 4334, 1, 7809}, {2, 4335, 1, 7809}, {3, 4336, 1, 7809}, {4, 4039, 1, 7810}, {5, 4040, 1, 7810}, {6, 4041, 1, 7810}, {5, 4039, 1, 7811}, {7, 4040, 1, 7811}, {8, 4041, 1, 7811}, {6, 4039, 1, 7812}, {8, 4040, 1, 7812}, {9, 4041, 1, 7812}, {4, 4149, 1, 7813}, {5, 4150, 1, 7813}, {6, 4151, 1, 7813}, {5, 4149, 1, 7814}, {7, 4150, 1, 7814}, {8, 4151, 1, 7814}, {6, 4149, 1, 7815}, {8, 4150, 1, 7815}, {9, 4151, 1, 7815}, {4, 4042, 1, 7816}, {5, 4043, 2, 7816}, {6, 4044, 2, 7816}, {7, 4045, 1, 7816}, {8, 4046, 2, 7816}, {9, 4047, 1, 7816}, {4, 4048, 1, 7817}, {5, 4049, 2, 7817}, {6, 4050, 2, 7817}, {7, 4051, 1, 7817}, {8, 4052, 2, 7817}, {9, 4053, 1, 7817}, {4, 4049, 1, 7818},

{5, 4051, 2, 7818}, {6, 4052, 2, 7818}, {7, 4054, 1, 7818}, {8, 4055, 2, 7818}, {9, 4056, 1, 7818}, {4, 4050, 1, 7819}, {5, 4052, 2, 7819}, {6, 4053, 2, 7819}, {7, 4055, 1, 7819}, {8, 4056, 2, 7819}, {9, 4057, 1, 7819}, {4, 4130, 1, 7820}, {5, 4131, 2, 7820}, {6, 4132, 2, 7820}, {7, 4133, 1, 7820}, {8, 4134, 2, 7820}, {9, 4135, 1, 7820}, {4, 4136, 1, 7821}, {5, 4137, 2, 7821}, {6, 4138, 2, 7821}, {7, 4139, 1, 7821}, {8, 4140, 2, 7821}, {9, 4141, 1, 7821}, {4, 4142, 1, 7822}, {5, 4143, 2, 7822}, {6, 4144, 2, 7822}, {7, 4145, 1, 7822}, {8, 4146, 2, 7822}, {9, 4147, 1, 7822}, {4, 4152, 1, 7823}, {5, 4153, 2, 7823}, {6, 4154, 2, 7823}, {7, 4155, 1, 7823}, {8, 4156, 2, 7823}, {9, 4157, 1, 7823}, {4, 4262, 1, 7824}, {5, 4263, 2, 7824}, {6, 4264, 2, 7824}, {7, 4265, 1, 7824}, {8, 4266, 2, 7824}, {9, 4267, 1, 7824}, {4, 3978, 1, 7825}, {5, 3979, 1, 7825}, {6, 3980, 1, 7825}, {5, 3978, 1, 7826}, {7, 3979, 1, 7826}, {8, 3980, 1, 7826}, {6, 3978, 1, 7827}, {8, 3979, 1, 7827}, {9, 3980, 1, 7827}, {4, 3981, 1, 7828}, {5, 3984, 1, 7828}, {6, 3987, 1, 7828}, {4, 3982, 1, 7829}, {5, 3985, 1, 7829}, {6, 3988, 1, 7829}, {4, 3983, 1, 7830}, {5, 3986, 1, 7830}, {6, 3989, 1, 7830}, {5, 3981, 1, 7831}, {7, 3984, 1, 7831}, {8, 3987, 1, 7831}, {5, 3982, 1, 7832}, {7, 3985, 1, 7832}, {8, 3988, 1, 7832}, {5, 3983, 1, 7833}, {7, 3986, 1, 7833}, {8, 3989, 1, 7833}, {6, 3981, 1, 7834}, {8, 3984, 1, 7834}, {9, 3987, 1, 7834}, {6, 3982, 1, 7835}, {8, 3985, 1, 7835}, {9, 3988, 1, 7835}, {6, 3983, 1, 7836}, {8, 3986, 1, 7836}, {9, 3989, 1, 7836}, {4, 4118, 1, 7837}, {5, 4119, 1, 7837}, {6, 4120, 1, 7837}, {5, 4118, 1, 7838}, {7, 4119, 1, 7838}, {8, 4120, 1, 7838}, {6, 4118, 1, 7839}, {8, 4119, 1, 7839}, {9, 4120, 1, 7839}, {4, 4121, 1, 7840}, {5, 4124, 1, 7840}, {6, 4127, 1, 7840}, {4, 4122, 1, 7841}, {5, 4125, 1, 7841}, {6, 4128, 1, 7841}, {4, 4123, 1, 7842}, {5, 4126, 1, 7842}, {6, 4129, 1, 7842}, {5, 4121, 1, 7843}, {7, 4124, 1, 7843}, {8, 4127, 1, 7843}, {5, 4122, 1, 7844}, {7, 4125, 1, 7844}, {8, 4128, 1, 7844}, {5, 4123, 1, 7845}, {7, 4126, 1, 7845}, {8, 4129, 1, 7845}, {6, 4121, 1, 7846}, {8, 4124, 1, 7846}, {9, 4127, 1, 7846}, {6, 4122, 1, 7847}, {8, 4125, 1, 7847}, {9, 4128, 1, 7847}, {6, 4123, 1, 7848}, {8, 4126, 1, 7848}, {9, 4129, 1, 7848}, {4, 4168, 1, 7849}, {5, 4169, 1, 7849}, {6, 4170, 1, 7849}, {4, 4169, 1, 7850}, {5, 4171, 1, 7850}, {6, 4172, 1, 7850}, {4, 4170, 1, 7851}, {5, 4172, 1, 7851}, {6, 4173, 1, 7851}, {5, 4168, 1, 7852}, {7, 4169, 1, 7852}, {8, 4170, 1, 7852}, {5, 4169, 1, 7853}, {7, 4171, 1, 7853}, {8, 4172, 1, 7853}, {5, 4170, 1, 7854}, {7, 4172, 1, 7854}, {8, 4173, 1, 7854}, {6, 4168, 1, 7855}, {8, 4169, 1, 7855}, {9, 4170, 1, 7855}, {6, 4169, 1, 7856}, {8, 4171, 1, 7856}, {9, 4172, 1, 7856}, {6, 4170, 1, 7857}, {8, 4172, 1, 7857}, {9, 4173, 1, 7857}, {4, 4228, 1, 7858}, {5, 4229, 1, 7858}, {6, 4230, 1, 7858}, {5, 4228, 1, 7859}, {7, 4229, 1, 7859}, {8, 4230, 1, 7859}, {6, 4228, 1, 7860}, {8, 4229, 1, 7860}, {9, 4230, 1, 7860}, {4, 3981, 1, 7861}, {5, 3982, 1, 7861}, {5, 3984, 1, 7861}, {6, 3983, 1, 7861}, {6, 3987, 1, 7861}, {7, 3985, 1, 7861}, {8, 3986, 1, 7861}, {8, 3988, 1, 7861}, {9, 3989, 1, 7861}, {4, 3990, 1, 7862}, {5, 3991, 1, 7862}, {5, 3996, 1, 7862}, {6, 3992, 1, 7862}, {6, 4002, 1, 7862}, {7, 3997, 1, 7862}, {8, 3998, 1, 7862}, {8, 4003, 1, 7862}, {9, 4004, 1, 7862}, {4, 3991, 1, 7863}, {5, 3993, 1, 7863}, {5, 3997, 1, 7863}, {6, 3994, 1, 7863}, {6, 4003, 1, 7863}, {7, 3999, 1, 7863}, {8, 4000, 1, 7863}, {8, 4005, 1, 7863}, {9, 4006, 1, 7863}, {4, 3992, 1, 7864}, {5, 3994, 1, 7864}, {5, 3998, 1, 7864}, {6, 3995, 1, 7864}, {6, 4004, 1, 7864}, {7, 4000, 1, 7864}, {8, 4001, 1, 7864}, {8, 4006, 1, 7864}, {9, 4007, 1, 7864}, {4, 4008, 1, 7865}, {5, 4009, 1, 7865}, {5, 4018, 1, 7865}, {6, 4010, 1, 7865}, {6, 4028, 1, 7865}, {7, 4019, 1, 7865}, {8, 4020, 1, 7865}, {8, 4029, 1, 7865}, {9, 4030, 1, 7865}, {4, 4009, 1, 7866}, {5, 4011, 1, 7866}, {5, 4019, 1, 7866}, {6, 4012, 1, 7866}, {6, 4029, 1, 7866}, {7, 4021, 1, 7866}, {8, 4022, 1, 7866}, {8, 4031, 1, 7866}, {9, 4032, 1, 7866}, {4, 4010, 1, 7867}, {5, 4012, 1, 7867}, {5, 4020, 1, 7867}, {6, 4013, 1, 7867}, {6, 4030, 1, 7867}, {7, 4022, 1, 7867}, {8, 4023, 1, 7867}, {8, 4032, 1, 7867}, {9, 4033, 1, 7867}, {4, 4011, 1, 7868}, {5, 4014, 1, 7868}, {5, 4021, 1, 7868}, {6, 4015, 1, 7868}, {6, 4031, 1, 7868}, {7, 4024, 1, 7868}, {8, 4025, 1, 7868}, {8, 4034, 1, 7868}, {9, 4035, 1, 7868}, {4, 4012, 1, 7869}, {5, 4015, 1, 7869}, {5, 4022, 1, 7869}, {6, 4016, 1, 7869}, {6, 4032, 1, 7869}, {7, 4025, 1, 7869}, {8, 4026, 1, 7869}, {8, 4035, 1, 7869}, {9, 4036, 1, 7869}, {4, 4013, 1, 7870}, {5, 4016, 1, 7870}, {5, 4023, 1, 7870}, {6, 4017, 1, 7870}, {6, 4033, 1, 7870}, {7, 4026, 1, 7870}, {8, 4027, 1, 7870}, {8, 4036, 1, 7870}, {9, 4037, 1, 7870}, {4, 4064, 1, 7871}, {5, 4065, 1, 7871}, {5, 4067, 1, 7871}, {6, 4066, 1, 7871}, {6, 4070, 1, 7871}, {7, 4068, 1, 7871}, {8, 4069, 1, 7871}, {8, 4071, 1, 7871}, {9, 4072, 1, 7871}, {4, 4067, 1, 7872}, {5, 4068, 1, 7872}, {5, 4073, 1, 7872}, {6, 4069, 1, 7872}, {6, 4076, 1, 7872}, {7, 4074, 1, 7872}, {8, 4075, 1, 7872}, {8, 4077, 1, 7872}, {9, 4078, 1, 7872}, {4, 4070, 1, 7873}, {5, 4071, 1, 7873}, {5, 4076, 1, 7873}, {6, 4072, 1, 7873}, {6, 4079, 1, 7873}, {7, 4077, 1, 7873}, {8, 4078, 1, 7873}, {8, 4080, 1, 7873}, {9, 4081, 1, 7873}, {4, 4082, 1, 7874}, {5, 4083, 1, 7874}, {5, 4088, 1, 7874}, {6, 4084, 1, 7874}, {6, 4094, 1, 7874}, {7, 4089, 1, 7874}, {8, 4090, 1, 7874}, {8, 4095, 1, 7874}, {9, 4096, 1, 7874}, {4, 4083, 1, 7875}, {5, 4085, 1, 7875}, {5, 4089, 1, 7875}, {6, 4086, 1, 7875}, {6, 4095, 1, 7875}, {7, 4091, 1, 7875}, {8, 4092, 1, 7875}, {8, 4097, 1, 7875}, {9, 4098, 1, 7875}, {4, 4084, 1, 7876}, {5, 4086, 1, 7876}, {5, 4090, 1, 7876}, {6, 4087, 1, 7876}, {6, 4096, 1, 7876}, {7, 4092, 1, 7876}, {8, 4093, 1, 7876}, {8, 4098, 1, 7876}, {9, 4099, 1, 7876}, {4, 4088, 1, 7877}, {5, 4089, 1, 7877}, {5, 4100, 1, 7877}, {6, 4090, 1, 7877}, {6, 4106, 1, 7877}, {7, 4101, 1, 7877}, {8, 4102, 1, 7877}, {8, 4107, 1, 7877}, {9, 4108, 1, 7877}, {4, 4089, 1, 7878}, {5, 4091, 1, 7878}, {5, 4101, 1, 7878}, {6, 4092, 1, 7878}, {6, 4107, 1, 7878}, {7, 4103, 1, 7878}, {8, 4104, 1, 7878}, {8, 4109, 1, 7878}, {9, 4110,

1, 7878}, {4, 4090, 1, 7879}, {5, 4092, 1, 7879}, {5, 4102, 1, 7879}, {6, 4093, 1, 7879}, {6, 4108, 1, 7879}, {7, 4104, 1, 7879}, {8, 4105, 1, 7879}, {8, 4110, 1, 7879}, {9, 4111, 1, 7879}, {4, 4094, 1, 7880}, {5, 4095, 1, 7880}, {5, 4106, 1, 7880}, {6, 4096, 1, 7880}, {6, 4112, 1, 7880}, {7, 4107, 1, 7880}, {8, 4108, 1, 7880}, {8, 4113, 1, 7880}, {9, 4114, 1, 7880}, {4, 4095, 1, 7881}, {5, 4097, 1, 7881}, {5, 4107, 1, 7881}, {6, 4098, 1, 7881}, {6, 4113, 1, 7881}, {7, 4109, 1, 7881}, {8, 4110, 1, 7881}, {8, 4115, 1, 7881}, {9, 4116, 1, 7881}, {4, 4096, 1, 7882}, {5, 4098, 1, 7882}, {5, 4108, 1, 7882}, {6, 4099, 1, 7882}, {6, 4114, 1, 7882}, {7, 4110, 1, 7882}, {8, 4111, 1, 7882}, {8, 4116, 1, 7882}, {9, 4117, 1, 7882}, {4, 4121, 1, 7883}, {5, 4122, 1, 7883}, {5, 4124, 1, 7883}, {6, 4123, 1, 7883}, {6, 4127, 1, 7883}, {7, 4125, 1, 7883}, {8, 4126, 1, 7883}, {8, 4128, 1, 7883}, {9, 4129, 1, 7883}, {4, 4130, 1, 7884}, {5, 4131, 1, 7884}, {5, 4136, 1, 7884}, {6, 4132, 1, 7884}, {6, 4142, 1, 7884}, {7, 4137, 1, 7884}, {8, 4138, 1, 7884}, {8, 4143, 1, 7884}, {9, 4144, 1, 7884}, {4, 4131, 1, 7885}, {5, 4133, 1, 7885}, {5, 4137, 1, 7885}, {6, 4134, 1, 7885}, {6, 4143, 1, 7885}, {7, 4139, 1, 7885}, {8, 4140, 1, 7885}, {8, 4145, 1, 7885}, {9, 4146, 1, 7885}, {4, 4132, 1, 7886}, {5, 4134, 1, 7886}, {5, 4138, 1, 7886}, {6, 4135, 1, 7886}, {6, 4144, 1, 7886}, {7, 4140, 1, 7886}, {8, 4141, 1, 7886}, {8, 4146, 1, 7886}, {9, 4147, 1, 7886}, {4, 4174, 1, 7887}, {5, 4175, 1, 7887}, {5, 4177, 1, 7887}, {6, 4176, 1, 7887}, {6, 4180, 1, 7887}, {7, 4178, 1, 7887}, {8, 4179, 1, 7887}, {8, 4181, 1, 7887}, {9, 4182, 1, 7887}, {4, 4177, 1, 7888}, {5, 4178, 1, 7888}, {5, 4183, 1, 7888}, {6, 4179, 1, 7888}, {6, 4186, 1, 7888}, {7, 4184, 1, 7888}, {8, 4185, 1, 7888}, {8, 4187, 1, 7888}, {9, 4188, 1, 7888}, {4, 4180, 1, 7889}, {5, 4181, 1, 7889}, {5, 4186, 1, 7889}, {6, 4182, 1, 7889}, {6, 4189, 1, 7889}, {7, 4187, 1, 7889}, {8, 4188, 1, 7889}, {8, 4190, 1, 7889}, {9, 4191, 1, 7889}, {4, 4231, 1, 7890}, {5, 4232, 1, 7890}, {5, 4234, 1, 7890}, {6, 4233, 1, 7890}, {6, 4237, 1, 7890}, {7, 4235, 1, 7890}, {8, 4236, 1, 7890}, {8, 4238, 1, 7890}, {9, 4239, 1, 7890}, {4, 4305, 1, 7891}, {5, 4306, 1, 7891}, {5, 4308, 1, 7891}, {6, 4307, 1, 7891}, {6, 4311, 1, 7891}, {7, 4309, 1, 7891}, {8, 4310, 1, 7891}, {8, 4312, 1, 7891}, {9, 4313, 1, 7891}, {506, 771, 1, 7892}, {507, 772, 1, 7893}, {508, 773, 1, 7893}, {509, 774, 1, 7893}, {510, 772, 1, 7894}, {511, 773, 1, 7894}, {512, 774, 1, 7894}, {511, 772, 1, 7895}, {513, 773, 1, 7895}, {514, 774, 1, 7895}, {512, 772, 1, 7896}, {514, 773, 1, 7896}, {515, 774, 1, 7896}, {507, 775, 1, 7897}, {508, 776, 1, 7897}, {509, 777, 1, 7897}, {507, 776, 1, 7898}, {508, 778, 1, 7898}, {509, 779, 1, 7898}, {507, 777, 1, 7899}, {508, 779, 1, 7899}, {509, 780, 1, 7899}, {510, 775, 1, 7900}, {511, 776, 1, 7900}, {512, 777, 1, 7900}, {511, 775, 1, 7901}, {513, 776, 1, 7901}, {514, 777, 1, 7901}, {512, 775, 1, 7902}, {514, 776, 1, 7902}, {515, 777, 1, 7902}, {510, 776, 1, 7903}, {511, 778, 1, 7903}, {512, 779, 1, 7903}, {511, 776, 1, 7904}, {513, 778, 1, 7904}, {514, 779, 1, 7904}, {512, 776, 1, 7905}, {514, 778, 1, 7905}, {515, 779, 1, 7905}, {510, 777, 1, 7906}, {511, 779, 1, 7906}, {512, 780, 1, 7906}, {511, 777, 1, 7907}, {513, 779, 1, 7907}, {514, 780, 1, 7907}, {512, 777, 1, 7908}, {514, 779, 1, 7908}, {515, 780, 1, 7908}, {4, 4168, 1, 7909}, {5, 4169, 2, 7909}, {6, 4170, 2, 7909}, {7, 4171, 1, 7909}, {8, 4172, 2, 7909}, {9, 4173, 1, 7909}, {4, 4174, 1, 7910}, {5, 4177, 2, 7910}, {6, 4180, 2, 7910}, {7, 4183, 1, 7910}, {8, 4186, 2, 7910}, {9, 4189, 1, 7910}, {4, 4175, 1, 7911}, {5, 4178, 2, 7911}, {6, 4181, 2, 7911}, {7, 4184, 1, 7911}, {8, 4187, 2, 7911}, {9, 4190, 1, 7911}, {4, 4176, 1, 7912}, {5, 4179, 2, 7912}, {6, 4182, 2, 7912}, {7, 4185, 1, 7912}, {8, 4188, 2, 7912}, {9, 4191, 1, 7912}, {4, 4268, 1, 7913}, {5, 4269, 2, 7913}, {6, 4270, 2, 7913}, {7, 4271, 1, 7913}, {8, 4272, 2, 7913}, {9, 4273, 1, 7913}, {4, 4269, 1, 7914}, {5, 4271, 2, 7914}, {6, 4272, 2, 7914}, {7, 4274, 1, 7914}, {8, 4275, 2, 7914}, {9, 4276, 1, 7914}, {4, 4270, 1, 7915}, {5, 4272, 2, 7915}, {6, 4273, 2, 7915}, {7, 4275, 1, 7915}, {8, 4276, 2, 7915}, {9, 4277, 1, 7915}, {4, 4278, 1, 7916}, {5, 4279, 2, 7916}, {6, 4280, 2, 7916}, {7, 4281, 1, 7916}, {8, 4282, 2, 7916}, {9, 4283, 1, 7916}, {4, 4328, 1, 7917}, {5, 4329, 2, 7917}, {6, 4330, 2, 7917}, {7, 4331, 1, 7917}, {8, 4332, 2, 7917}, {9, 4333, 1, 7917}, {10, 4158, 1, 7918}, {11, 4159, 3, 7918}, {12, 4160, 3, 7918}, {13, 4161, 3, 7918}, {14, 4162, 6, 7918}, {15, 4163, 3, 7918}, {16, 4164, 1, 7918}, {17, 4165, 3, 7918}, {18, 4166, 3, 7918}, {19, 4167, 1, 7918}, {10, 4064, 1, 7919}, {11, 4065, 1, 7919}, {11, 4067, 1, 7919}, {12, 4066, 1, 7919}, {12, 4070, 1, 7919}, {13, 4068, 1, 7919}, {14, 4069, 1, 7919}, {14, 4071, 1, 7919}, {15, 4072, 1, 7919}, {10, 4067, 1, 7920}, {11, 4068, 1, 7920}, {11, 4073, 1, 7920}, {12, 4069, 1, 7920}, {12, 4076, 1, 7920}, {13, 4074, 1, 7920}, {14, 4075, 1, 7920}, {14, 4077, 1, 7920}, {15, 4078, 1, 7920}, {10, 4070, 1, 7921}, {11, 4071, 1, 7921}, {11, 4076, 1, 7921}, {12, 4072, 1, 7921}, {12, 4079, 1, 7921}, {13, 4077, 1, 7921}, {14, 4078, 1, 7921}, {14, 4080, 1, 7921}, {15, 4081, 1, 7921}, {11, 4064, 1, 7922}, {13, 4065, 1, 7922}, {13, 4067, 1, 7922}, {14, 4066, 1, 7922}, {14, 4070, 1, 7922}, {16, 4068, 1, 7922}, {17, 4069, 1, 7922}, {17, 4071, 1, 7922}, {18, 4072, 1, 7922}, {11, 4067, 1, 7923}, {13, 4068, 1, 7923}, {13, 4073, 1, 7923}, {14, 4069, 1, 7923}, {14, 4076, 1, 7923}, {16, 4074, 1, 7923}, {17, 4075, 1, 7923}, {17, 4077, 1, 7923}, {18, 4078, 1, 7923}, {11, 4070, 1, 7924}, {13, 4071, 1, 7924}, {13, 4076, 1, 7924}, {14, 4072, 1, 7924}, {14, 4079, 1, 7924}, {16, 4077, 1, 7924}, {17, 4078, 1, 7924}, {17, 4080, 1, 7924}, {18, 4081, 1, 7924}, {12, 4064, 1, 7925}, {14, 4065, 1, 7925}, {14, 4067, 1, 7925}, {15, 4066, 1, 7925}, {15, 4070, 1, 7925}, {17, 4068, 1, 7925}, {18, 4069, 1, 7925}, {18, 4071, 1, 7925}, {19, 4072, 1, 7925}, {12, 4067, 1, 7926}, {14, 4068, 1, 7926}, {14, 4073, 1, 7926}, {15, 4069, 1, 7926}, {15, 4076, 1, 7926}, {17, 4074, 1, 7926}, {18, 4075, 1, 7926}, {18, 4077, 1, 7926}, {19, 4078, 1, 7926}, {12, 4070,

1, 7927}, {14, 4071, 1, 7927}, {14, 4076, 1, 7927}, {15, 4072, 1, 7927}, {15, 4079, 1, 7927}, {17, 4077, 1, 7927}, {18, 4078, 1, 7927}, {18, 4080, 1, 7927}, {19, 4081, 1, 7927}, {10, 4121, 1, 7928}, {11, 4122, 1, 7928}, {11, 4124, 1, 7928}, {12, 4123, 1, 7928}, {12, 4127, 1, 7928}, {13, 4125, 1, 7928}, {14, 4126, 1, 7928}, {14, 4128, 1, 7928}, {15, 4129, 1, 7928}, {11, 4121, 1, 7929}, {13, 4122, 1, 7929}, {13, 4124, 1, 7929}, {14, 4123, 1, 7929}, {14, 4127, 1, 7929}, {16, 4125, 1, 7929}, {17, 4126, 1, 7929}, {17, 4128, 1, 7929}, {18, 4129, 1, 7929}, {12, 4121, 1, 7930}, {14, 4122, 1, 7930}, {14, 4124, 1, 7930}, {15, 4123, 1, 7930}, {15, 4127, 1, 7930}, {17, 4125, 1, 7930}, {18, 4126, 1, 7930}, {18, 4128, 1, 7930}, {19, 4129, 1, 7930}, {10, 4130, 1, 7931}, {11, 4131, 2, 7931}, {11, 4136, 1, 7931}, {12, 4132, 2, 7931}, {12, 4142, 1, 7931}, {13, 4133, 1, 7931}, {13, 4137, 2, 7931}, {14, 4134, 2, 7931}, {14, 4138, 2, 7931}, {14, 4143, 2, 7931}, {15, 4135, 1, 7931}, {15, 4144, 2, 7931}, {16, 4139, 1, 7931}, {17, 4140, 2, 7931}, {17, 4145, 1, 7931}, {18, 4141, 1, 7931}, {18, 4146, 2, 7931}, {19, 4147, 1, 7931}, {10, 4240, 1, 7932}, {11, 4241, 2, 7932}, {11, 4246, 1, 7932}, {12, 4242, 2, 7932}, {12, 4252, 1, 7932}, {13, 4243, 1, 7932}, {13, 4247, 2, 7932}, {14, 4244, 2, 7932}, {14, 4248, 2, 7932}, {14, 4253, 2, 7932}, {15, 4245, 1, 7932}, {15, 4254, 2, 7932}, {16, 4249, 1, 7932}, {17, 4250, 2, 7932}, {17, 4255, 1, 7932}, {18, 4251, 1, 7932}, {18, 4256, 2, 7932}, {19, 4257, 1, 7932}, {507, 827, 1, 7933}, {508, 828, 1, 7933}, {509, 829, 1, 7933}, {507, 828, 1, 7934}, {508, 830, 1, 7934}, {509, 831, 1, 7934}, {507, 829, 1, 7935}, {508, 831, 1, 7935}, {509, 832, 1, 7935}, {510, 827, 1, 7936}, {511, 828, 1, 7936}, {512, 829, 1, 7936}, {511, 827, 1, 7937}, {513, 828, 1, 7937}, {514, 829, 1, 7937}, {512, 827, 1, 7938}, {514, 828, 1, 7938}, {515, 829, 1, 7938}, {510, 828, 1, 7939}, {511, 830, 1, 7939}, {512, 831, 1, 7939}, {511, 828, 1, 7940}, {513, 830, 1, 7940}, {514, 831, 1, 7940}, {512, 828, 1, 7941}, {514, 830, 1, 7941}, {515, 831, 1, 7941}, {510, 829, 1, 7942}, {511, 831, 1, 7942}, {512, 832, 1, 7942}, {511, 829, 1, 7943}, {513, 831, 1, 7943}, {514, 832, 1, 7943}, {512, 829, 1, 7944}, {514, 831, 1, 7944}, {515, 832, 1, 7944}, {10, 4168, 1, 7945}, {11, 4169, 2, 7945}, {12, 4170, 2, 7945}, {13, 4171, 1, 7945}, {14, 4172, 2, 7945}, {15, 4173, 1, 7945}, {11, 4168, 1, 7946}, {13, 4169, 2, 7946}, {14, 4170, 2, 7946}, {16, 4171, 1, 7946}, {17, 4172, 2, 7946}, {18, 4173, 1, 7946}, {12, 4168, 1, 7947}, {14, 4169, 2, 7947}, {15, 4170, 2, 7947}, {17, 4171, 1, 7947}, {18, 4172, 2, 7947}, {19, 4173, 1, 7947}, {510, 827, 1, 7948}, {511, 828, 2, 7948}, {512, 829, 2, 7948}, {513, 830, 1, 7948}, {514, 831, 2, 7948}, {515, 832, 1, 7948}, {516, 827, 1, 7949}, {517, 828, 2, 7949}, {518, 829, 2, 7949}, {519, 830, 1, 7949}, {520, 831, 2, 7949}, {521, 832, 1, 7949}, {517, 827, 1, 7950}, {519, 828, 2, 7950}, {520, 829, 2, 7950}, {522, 830, 1, 7950}, {523, 831, 2, 7950}, {524, 832, 1, 7950}, {518, 827, 1, 7951}, {520, 828, 2, 7951}, {521, 829, 2, 7951}, {523, 830, 1, 7951}, {524, 831, 2, 7951}, {525, 832, 1, 7951}, {510, 833, 1, 7952}, {511, 834, 1, 7952}, {511, 839, 1, 7952}, {512, 835, 1, 7952}, {512, 845, 1, 7952}, {513, 840, 1, 7952}, {514, 841, 1, 7952}, {514, 846, 1, 7952}, {515, 847, 1, 7952}, {510, 834, 1, 7953}, {511, 836, 1, 7953}, {511, 840, 1, 7953}, {512, 837, 1, 7953}, {512, 846, 1, 7953}, {513, 842, 1, 7953}, {514, 843, 1, 7953}, {514, 848, 1, 7953}, {515, 849, 1, 7953}, {510, 835, 1, 7954}, {511, 837, 1, 7954}, {511, 841, 1, 7954}, {512, 838, 1, 7954}, {512, 847, 1, 7954}, {513, 843, 1, 7954}, {514, 844, 1, 7954}, {514, 849, 1, 7954}, {515, 850, 1, 7954}, {10, 4174, 1, 7955}, {11, 4175, 1, 7955}, {11, 4177, 2, 7955}, {12, 4176, 1, 7955}, {12, 4180, 2, 7955}, {13, 4178, 2, 7955}, {13, 4183, 1, 7955}, {14, 4179, 2, 7955}, {14, 4181, 2, 7955}, {14, 4186, 2, 7955}, {15, 4182, 2, 7955}, {15, 4189, 1, 7955}, {16, 4184, 1, 7955}, {17, 4185, 1, 7955}, {17, 4187, 2, 7955}, {18, 4188, 2, 7955}, {18, 4190, 1, 7955}, {19, 4191, 1, 7955}, {10, 4284, 1, 7956}, {11, 4285, 1, 7956}, {11, 4287, 2, 7956}, {12, 4286, 1, 7956}, {12, 4290, 2, 7956}, {13, 4288, 2, 7956}, {13, 4293, 1, 7956}, {14, 4289, 2, 7956}, {14, 4291, 2, 7956}, {14, 4296, 2, 7956}, {15, 4292, 2, 7956}, {15, 4299, 1, 7956}, {16, 4294, 1, 7956}, {17, 4295, 1, 7956}, {17, 4297, 2, 7956}, {18, 4298, 2, 7956}, {18, 4300, 1, 7956}, {19, 4301, 1, 7956}, {506, 989, 1, 7957}, {507, 990, 1, 7958}, {508, 991, 1, 7958}, {509, 992, 1, 7958}, {510, 990, 1, 7959}, {511, 991, 1, 7959}, {512, 992, 1, 7959}, {511, 990, 1, 7960}, {513, 991, 1, 7960}, {514, 992, 1, 7960}, {512, 990, 1, 7961}, {514, 991, 1, 7961}, {515, 992, 1, 7961}, {507, 993, 1, 7962}, {508, 994, 1, 7962}, {509, 995, 1, 7962}, {507, 994, 1, 7963}, {508, 996, 1, 7963}, {509, 997, 1, 7963}, {507, 995, 1, 7964}, {508, 997, 1, 7964}, {509, 998, 1, 7964}, {510, 993, 1, 7965}, {511, 994, 2, 7965}, {512, 995, 2, 7965}, {513, 996, 1, 7965}, {514, 997, 2, 7965}, {515, 998, 1, 7965}, {10, 4318, 1, 7966}, {11, 4319, 3, 7966}, {12, 4320, 3, 7966}, {13, 4321, 3, 7966}, {14, 4322, 6, 7966}, {15, 4323, 3, 7966}, {16, 4324, 1, 7966}, {17, 4325, 3, 7966}, {18, 4326, 3, 7966}, {19, 4327, 1, 7966}, {20, 4192, 1, 7967}, {21, 4193, 2, 7967}, {21, 4198, 2, 7967}, {22, 4194, 2, 7967}, {22, 4204, 2, 7967}, {23, 4195, 1, 7967}, {23, 4199, 4, 7967}, {23, 4210, 1, 7967}, {24, 4196, 2, 7967}, {24, 4200, 4, 7967}, {24, 4205, 4, 7967}, {24, 4216, 2, 7967}, {25, 4197, 1, 7967}, {25, 4206, 4, 7967}, {25, 4222, 1, 7967}, {26, 4201, 2, 7967}, {26, 4211, 2, 7967}, {27, 4202, 4, 7967}, {27, 4207, 2, 7967}, {27, 4212, 2, 7967}, {27, 4217, 4, 7967}, {28, 4203, 2, 7967}, {28, 4208, 4, 7967}, {28, 4218, 4, 7967}, {28, 4223, 2, 7967}, {29, 4209, 2, 7967}, {29, 4224, 2, 7967}, {30, 4213, 1, 7967}, {31, 4214, 2, 7967}, {31, 4219, 2, 7967}, {32, 4215, 1, 7967}, {32, 4220, 4, 7967}, {32, 4225, 1, 7967}, {33, 4221, 2, 7967}, {33, 4226, 2, 7967}, {34, 4227, 1, 7967}, {507, 1370, 1, 7968}, {508, 1371, 1, 7968}, {509, 1372, 1, 7968}, {507, 1371, 1, 7969}, {508, 1373, 1, 7969}, {509, 1374, 1, 7969}, {507,

1372, 1, 7970}, {508, 1374, 1, 7970}, {509, 1375, 1, 7970}, {510, 1370, 1, 7971}, {511,
1371, 2, 7971}, {512, 1372, 2, 7971}, {513, 1373, 1, 7971}, {514, 1374, 2, 7971}, {515,
1375, 1, 7971}, {516, 1376, 1, 7972}, {517, 1377, 2, 7972}, {517, 1382, 1, 7972}, {518,
1378, 2, 7972}, {518, 1388, 1, 7972}, {519, 1379, 1, 7972}, {519, 1383, 2, 7972}, {520,
1380, 2, 7972}, {520, 1384, 2, 7972}, {520, 1389, 2, 7972}, {521, 1381, 1, 7972}, {521,
1390, 2, 7972}, {522, 1385, 1, 7972}, {523, 1386, 2, 7972}, {523, 1391, 1, 7972}, {524,
1387, 1, 7972}, {524, 1392, 2, 7972}, {525, 1393, 1, 7972}, {506, 1532, 1, 7973}, {507,
1533, 1, 7974}, {508, 1534, 1, 7974}, {509, 1535, 1, 7974}, {510, 1536, 1, 7975}, {511,
1537, 2, 7975}, {512, 1538, 2, 7975}, {513, 1539, 1, 7975}, {514, 1540, 2, 7975}, {515,
1541, 1, 7975}, {510, 1964, 1, 7976}, {511, 1965, 2, 7976}, {512, 1966, 2, 7976}, {513,
1967, 1, 7976}, {514, 1968, 2, 7976}, {515, 1969, 1, 7976}, {506, 2063, 1, 7977}, {507,
2064, 1, 7978}, {508, 2065, 1, 7978}, {509, 2066, 1, 7978}, {506, 2444, 1, 7979}, {0,
4339, 1, 7980}, {0, 4340, 1, 7981}, {0, 4362, 1, 7982}, {0, 4390, 1, 7983}, {0, 4403, 1,
7984}, {1, 4341, 1, 7985}, {2, 4342, 1, 7985}, {3, 4343, 1, 7985}, {1, 4344, 1, 7986}, {2,
4345, 1, 7986}, {3, 4346, 1, 7986}, {1, 4345, 1, 7987}, {2, 4347, 1, 7987}, {3, 4348, 1,
7987}, {1, 4346, 1, 7988}, {2, 4348, 1, 7988}, {3, 4349, 1, 7988}, {1, 4363, 1, 7989}, {2,
4364, 1, 7989}, {3, 4365, 1, 7989}, {1, 4391, 1, 7990}, {2, 4392, 1, 7990}, {3, 4393, 1,
7990}, {626, 707, 1, 7991}, {1, 4350, 1, 7992}, {2, 4351, 1, 7992}, {3, 4352, 1, 7992},
{1, 4353, 1, 7993}, {2, 4356, 1, 7993}, {3, 4359, 1, 7993}, {1, 4354, 1, 7994}, {2, 4357,
1, 7994}, {3, 4360, 1, 7994}, {1, 4355, 1, 7995}, {2, 4358, 1, 7995}, {3, 4361, 1, 7995},
{1, 4372, 1, 7996}, {2, 4373, 1, 7996}, {3, 4374, 1, 7996}, {1, 4373, 1, 7997}, {2, 4375,
1, 7997}, {3, 4376, 1, 7997}, {1, 4374, 1, 7998}, {2, 4376, 1, 7998}, {3, 4377, 1, 7998},
{1, 4378, 1, 7999}, {2, 4379, 1, 7999}, {3, 4380, 1, 7999}, {1, 4400, 1, 8000}, {2, 4401,
1, 8000}, {3, 4402, 1, 8000}, {4, 4366, 1, 8001}, {5, 4367, 2, 8001}, {6, 4368, 2, 8001},
{7, 4369, 1, 8001}, {8, 4370, 2, 8001}, {9, 4371, 1, 8001}, {4, 4350, 1, 8002}, {5, 4351,
1, 8002}, {6, 4352, 1, 8002}, {5, 4350, 1, 8003}, {7, 4351, 1, 8003}, {8, 4352, 1, 8003},
{6, 4350, 1, 8004}, {8, 4351, 1, 8004}, {9, 4352, 1, 8004}, {4, 4353, 1, 8005}, {5, 4354,
1, 8005}, {5, 4356, 1, 8005}, {6, 4355, 1, 8005}, {6, 4359, 1, 8005}, {7, 4357, 1, 8005},
{8, 4358, 1, 8005}, {8, 4360, 1, 8005}, {9, 4361, 1, 8005}, {4, 4381, 1, 8006}, {5, 4382,
1, 8006}, {5, 4384, 1, 8006}, {6, 4383, 1, 8006}, {6, 4387, 1, 8006}, {7, 4385, 1, 8006},
{8, 4386, 1, 8006}, {8, 4388, 1, 8006}, {9, 4389, 1, 8006}, {626, 771, 1, 8007}, {627,
772, 1, 8008}, {628, 773, 1, 8008}, {629, 774, 1, 8008}, {4, 4394, 1, 8009}, {5, 4395, 2,
8009}, {6, 4396, 2, 8009}, {7, 4397, 1, 8009}, {8, 4398, 2, 8009}, {9, 4399, 1, 8009},
{630, 827, 1, 8010}, {631, 828, 2, 8010}, {632, 829, 2, 8010}, {633, 830, 1, 8010}, {634,
831, 2, 8010}, {635, 832, 1, 8010}, {626, 989, 1, 8011}, {627, 990, 1, 8012}, {628, 991,
1, 8012}, {629, 992, 1, 8012}, {626, 1532, 1, 8013}, {0, 4405, 1, 8014}, {0, 4406, 1,
8015}, {0, 4413, 1, 8016}, {1, 4407, 1, 8017}, {2, 4408, 1, 8017}, {3, 4409, 1, 8017},
{682, 707, 1, 8018}, {1, 4410, 1, 8019}, {2, 4411, 1, 8019}, {3, 4412, 1, 8019}, {682,
771, 1, 8020}, {0, 4415, 1, 8021}, {0, 4417, 1, 8022}, {0, 4418, 1, 8023}, {0, 4482, 1,
8024}, {0, 4547, 1, 8025}, {0, 4612, 1, 8026}, {0, 4628, 1, 8027}, {0, 4632, 1, 8028},
{286, 2756, 1, 8029}, {1, 4419, 1, 8030}, {2, 4420, 1, 8030}, {3, 4421, 1, 8030}, {1,
4422, 1, 8031}, {2, 4423, 1, 8031}, {3, 4424, 1, 8031}, {1, 4423, 1, 8032}, {2, 4425, 1,
8032}, {3, 4426, 1, 8032}, {1, 4424, 1, 8033}, {2, 4426, 1, 8033}, {3, 4427, 1, 8033}, {1,
4428, 1, 8034}, {2, 4429, 1, 8034}, {3, 4430, 1, 8034}, {1, 4429, 1, 8035}, {2, 4431, 1,
8035}, {3, 4432, 1, 8035}, {1, 4430, 1, 8036}, {2, 4432, 1, 8036}, {3, 4433, 1, 8036}, {1,
4483, 1, 8037}, {2, 4484, 1, 8037}, {3, 4485, 1, 8037}, {1, 4486, 1, 8038}, {2, 4487, 1,
8038}, {3, 4488, 1, 8038}, {1, 4487, 1, 8039}, {2, 4489, 1, 8039}, {3, 4490, 1, 8039}, {1,
4488, 1, 8040}, {2, 4490, 1, 8040}, {3, 4491, 1, 8040}, {1, 4523, 1, 8041}, {2, 4524, 1,
8041}, {3, 4525, 1, 8041}, {1, 4524, 1, 8042}, {2, 4526, 1, 8042}, {3, 4527, 1, 8042}, {1,
4525, 1, 8043}, {2, 4527, 1, 8043}, {3, 4528, 1, 8043}, {1, 4548, 1, 8044}, {2, 4549, 1,
8044}, {3, 4550, 1, 8044}, {1, 4551, 1, 8045}, {2, 4552, 1, 8045}, {3, 4553, 1, 8045}, {1,
4552, 1, 8046}, {2, 4554, 1, 8046}, {3, 4555, 1, 8046}, {1, 4553, 1, 8047}, {2, 4555, 1,
8047}, {3, 4556, 1, 8047}, {1, 4588, 1, 8048}, {2, 4589, 1, 8048}, {3, 4590, 1, 8048}, {1,
4589, 1, 8049}, {2, 4591, 1, 8049}, {3, 4592, 1, 8049}, {1, 4590, 1, 8050}, {2, 4592, 1,
8050}, {3, 4593, 1, 8050}, {1, 4613, 1, 8051}, {2, 4614, 1, 8051}, {3, 4615, 1, 8051}, {1,
4629, 1, 8052}, {2, 4630, 1, 8052}, {3, 4631, 1, 8052}, {4, 4419, 1, 8053}, {5, 4420, 1,
8053}, {6, 4421, 1, 8053}, {5, 4419, 1, 8054}, {7, 4420, 1, 8054}, {8, 4421, 1, 8054}, {6,
4419, 1, 8055}, {8, 4420, 1, 8055}, {9, 4421, 1, 8055}, {4, 4428, 1, 8056}, {5, 4429, 1,
8056}, {6, 4430, 1, 8056}, {4, 4429, 1, 8057}, {5, 4431, 1, 8057}, {6, 4432, 1, 8057}, {4,
4430, 1, 8058}, {5, 4432, 1, 8058}, {6, 4433, 1, 8058}, {5, 4428, 1, 8059}, {7, 4429, 1,
8059}, {8, 4430, 1, 8059}, {5, 4429, 1, 8060}, {7, 4431, 1, 8060}, {8, 4432, 1, 8060}, {5,
4430, 1, 8061}, {7, 4432, 1, 8061}, {8, 4433, 1, 8061}, {6, 4428, 1, 8062}, {8, 4429, 1,
8062}, {9, 4430, 1, 8062}, {6, 4429, 1, 8063}, {8, 4431, 1, 8063}, {9, 4432, 1, 8063}, {6,
4430, 1, 8064}, {8, 4432, 1, 8064}, {9, 4433, 1, 8064}, {4, 4483, 1, 8065}, {5, 4484, 1,
8065}, {6, 4485, 1, 8065}, {5, 4483, 1, 8066}, {7, 4484, 1, 8066}, {8, 4485, 1, 8066}, {6,
4483, 1, 8067}, {8, 4484, 1, 8067}, {9, 4485, 1, 8067}, {4, 4486, 1, 8068}, {5, 4487, 1,
8068}, {6, 4488, 1, 8068}, {4, 4487, 1, 8069}, {5, 4489, 1, 8069}, {6, 4490, 1, 8069}, {4,
4488, 1, 8070}, {5, 4490, 1, 8070}, {6, 4491, 1, 8070}, {5, 4486, 1, 8071}, {7, 4487, 1,
8071}, {8, 4488, 1, 8071}, {5, 4487, 1, 8072}, {7, 4489, 1, 8072}, {8, 4490, 1, 8072}, {5,

4488, 1, 8073}, {7, 4490, 1, 8073}, {8, 4491, 1, 8073}, {6, 4486, 1, 8074}, {8, 4487, 1, 8074}, {9, 4488, 1, 8074}, {6, 4487, 1, 8075}, {8, 4489, 1, 8075}, {9, 4490, 1, 8075}, {6, 4488, 1, 8076}, {8, 4490, 1, 8076}, {9, 4491, 1, 8076}, {4, 4523, 1, 8077}, {5, 4524, 1, 8077}, {6, 4525, 1, 8077}, {4, 4524, 1, 8078}, {5, 4526, 1, 8078}, {6, 4527, 1, 8078}, {4, 4525, 1, 8079}, {5, 4527, 1, 8079}, {6, 4528, 1, 8079}, {5, 4523, 1, 8080}, {7, 4524, 1, 8080}, {8, 4525, 1, 8080}, {5, 4524, 1, 8081}, {7, 4526, 1, 8081}, {8, 4527, 1, 8081}, {5, 4525, 1, 8082}, {7, 4527, 1, 8082}, {8, 4528, 1, 8082}, {6, 4523, 1, 8083}, {8, 4524, 1, 8083}, {9, 4525, 1, 8083}, {6, 4524, 1, 8084}, {8, 4526, 1, 8084}, {9, 4527, 1, 8084}, {6, 4525, 1, 8085}, {8, 4527, 1, 8085}, {9, 4528, 1, 8085}, {4, 4548, 1, 8086}, {5, 4549, 1, 8086}, {6, 4550, 1, 8086}, {5, 4548, 1, 8087}, {7, 4549, 1, 8087}, {8, 4550, 1, 8087}, {6, 4548, 1, 8088}, {8, 4549, 1, 8088}, {9, 4550, 1, 8088}, {286, 2826, 1, 8089}, {287, 2827, 1, 8090}, {288, 2828, 1, 8090}, {289, 2829, 1, 8090}, {287, 2830, 1, 8091}, {288, 2831, 1, 8091}, {289, 2832, 1, 8091}, {287, 2831, 1, 8092}, {288, 2833, 1, 8092}, {289, 2834, 1, 8092}, {287, 2832, 1, 8093}, {288, 2834, 1, 8093}, {289, 2835, 1, 8093}, {290, 2827, 1, 8094}, {291, 2828, 1, 8094}, {292, 2829, 1, 8094}, {291, 2827, 1, 8095}, {293, 2828, 1, 8095}, {294, 2829, 1, 8095}, {292, 2827, 1, 8096}, {294, 2828, 1, 8096}, {295, 2829, 1, 8096}, {4, 4486, 1, 8097}, {5, 4487, 2, 8097}, {6, 4488, 2, 8097}, {7, 4489, 1, 8097}, {8, 4490, 2, 8097}, {9, 4491, 1, 8097}, {4, 4492, 1, 8098}, {5, 4493, 2, 8098}, {6, 4494, 2, 8098}, {7, 4495, 1, 8098}, {8, 4496, 2, 8098}, {9, 4497, 1, 8098}, {4, 4493, 1, 8099}, {5, 4495, 2, 8099}, {6, 4496, 2, 8099}, {7, 4498, 1, 8099}, {8, 4499, 2, 8099}, {9, 4500, 1, 8099}, {4, 4494, 1, 8100}, {5, 4496, 2, 8100}, {6, 4497, 2, 8100}, {7, 4499, 1, 8100}, {8, 4500, 2, 8100}, {9, 4501, 1, 8100}, {4, 4529, 1, 8101}, {5, 4530, 2, 8101}, {6, 4531, 2, 8101}, {7, 4532, 1, 8101}, {8, 4533, 2, 8101}, {9, 4534, 1, 8101}, {4, 4535, 1, 8102}, {5, 4536, 2, 8102}, {6, 4537, 2, 8102}, {7, 4538, 1, 8102}, {8, 4539, 2, 8102}, {9, 4540, 1, 8102}, {4, 4541, 1, 8103}, {5, 4542, 2, 8103}, {6, 4543, 2, 8103}, {7, 4544, 1, 8103}, {8, 4545, 2, 8103}, {9, 4546, 1, 8103}, {4, 4551, 1, 8104}, {5, 4552, 2, 8104}, {6, 4553, 2, 8104}, {7, 4554, 1, 8104}, {8, 4555, 2, 8104}, {9, 4556, 1, 8104}, {4, 4616, 1, 8105}, {5, 4617, 2, 8105}, {6, 4618, 2, 8105}, {7, 4619, 1, 8105}, {8, 4620, 2, 8105}, {9, 4621, 1, 8105}, {287, 2766, 1, 8106}, {288, 2767, 1, 8106}, {289, 2768, 1, 8106}, {287, 2769, 1, 8107}, {288, 2772, 1, 8107}, {289, 2775, 1, 8107}, {287, 2770, 1, 8108}, {288, 2773, 1, 8108}, {289, 2776, 1, 8108}, {287, 2771, 1, 8109}, {288, 2774, 1, 8109}, {289, 2777, 1, 8109}, {290, 2769, 1, 8110}, {291, 2772, 1, 8110}, {292, 2775, 1, 8110}, {290, 2770, 1, 8111}, {291, 2773, 1, 8111}, {292, 2776, 1, 8111}, {290, 2771, 1, 8112}, {291, 2774, 1, 8112}, {292, 2777, 1, 8112}, {291, 2769, 1, 8113}, {293, 2772, 1, 8113}, {294, 2775, 1, 8113}, {291, 2770, 1, 8114}, {293, 2773, 1, 8114}, {294, 2776, 1, 8114}, {291, 2771, 1, 8115}, {293, 2774, 1, 8115}, {294, 2777, 1, 8115}, {292, 2769, 1, 8116}, {294, 2772, 1, 8116}, {295, 2775, 1, 8116}, {292, 2770, 1, 8117}, {294, 2773, 1, 8117}, {295, 2776, 1, 8117}, {292, 2771, 1, 8118}, {294, 2774, 1, 8118}, {295, 2777, 1, 8118}, {4, 4428, 1, 8119}, {5, 4429, 2, 8119}, {6, 4430, 2, 8119}, {7, 4431, 1, 8119}, {8, 4432, 2, 8119}, {9, 4433, 1, 8119}, {4, 4434, 1, 8120}, {5, 4435, 1, 8120}, {5, 4440, 1, 8120}, {6, 4436, 1, 8120}, {6, 4446, 1, 8120}, {7, 4441, 1, 8120}, {8, 4442, 1, 8120}, {8, 4447, 1, 8120}, {9, 4448, 1, 8120}, {4, 4435, 1, 8121}, {5, 4437, 1, 8121}, {5, 4441, 1, 8121}, {6, 4438, 1, 8121}, {6, 4447, 1, 8121}, {7, 4443, 1, 8121}, {8, 4444, 1, 8121}, {8, 4449, 1, 8121}, {9, 4450, 1, 8121}, {4, 4436, 1, 8122}, {5, 4438, 1, 8122}, {5, 4442, 1, 8122}, {6, 4439, 1, 8122}, {6, 4448, 1, 8122}, {7, 4444, 1, 8122}, {8, 4445, 1, 8122}, {8, 4450, 1, 8122}, {9, 4451, 1, 8122}, {4, 4452, 1, 8123}, {5, 4453, 1, 8123}, {5, 4462, 1, 8123}, {6, 4454, 1, 8123}, {6, 4472, 1, 8123}, {7, 4463, 1, 8123}, {8, 4464, 1, 8123}, {8, 4473, 1, 8123}, {9, 4474, 1, 8123}, {4, 4453, 1, 8124}, {5, 4455, 1, 8124}, {5, 4463, 1, 8124}, {6, 4456, 1, 8124}, {6, 4473, 1, 8124}, {7, 4465, 1, 8124}, {8, 4466, 1, 8124}, {8, 4475, 1, 8124}, {9, 4476, 1, 8124}, {4, 4454, 1, 8125}, {5, 4456, 1, 8125}, {5, 4464, 1, 8125}, {6, 4457, 1, 8125}, {6, 4474, 1, 8125}, {7, 4466, 1, 8125}, {8, 4467, 1, 8125}, {8, 4476, 1, 8125}, {9, 4477, 1, 8125}, {4, 4455, 1, 8126}, {5, 4458, 1, 8126}, {5, 4465, 1, 8126}, {6, 4459, 1, 8126}, {6, 4475, 1, 8126}, {7, 4468, 1, 8126}, {8, 4469, 1, 8126}, {8, 4478, 1, 8126}, {9, 4479, 1, 8126}, {4, 4456, 1, 8127}, {5, 4459, 1, 8127}, {5, 4466, 1, 8127}, {6, 4460, 1, 8127}, {6, 4476, 1, 8127}, {7, 4469, 1, 8127}, {8, 4470, 1, 8127}, {8, 4479, 1, 8127}, {9, 4480, 1, 8127}, {4, 4457, 1, 8128}, {5, 4460, 1, 8128}, {5, 4467, 1, 8128}, {6, 4461, 1, 8128}, {6, 4477, 1, 8128}, {7, 4470, 1, 8128}, {8, 4471, 1, 8128}, {8, 4480, 1, 8128}, {9, 4481, 1, 8128}, {4, 4502, 1, 8129}, {5, 4503, 2, 8129}, {6, 4504, 2, 8129}, {7, 4508, 1, 8129}, {8, 4509, 2, 8129}, {9, 4513, 1, 8129}, {4, 4503, 1, 8130}, {5, 4505, 1, 8130}, {5, 4508, 1, 8130}, {6, 4506, 1, 8130}, {6, 4509, 1, 8130}, {7, 4510, 1, 8130}, {8, 4511, 1, 8130}, {8, 4514, 1, 8130}, {9, 4515, 1, 8130}, {4, 4504, 1, 8131}, {5, 4506, 1, 8131}, {5, 4509, 1, 8131}, {6, 4507, 1, 8131}, {6, 4513, 1, 8131}, {7, 4511, 1, 8131}, {8, 4512, 1, 8131}, {8, 4515, 1, 8131}, {9, 4516, 1, 8131}, {4, 4508, 1, 8132}, {5, 4510, 2, 8132}, {6, 4511, 2, 8132}, {7, 4517, 1, 8132}, {8, 4518, 2, 8132}, {9, 4520, 1, 8132}, {4, 4509, 1, 8133}, {5, 4511, 1, 8133}, {5, 4514, 1, 8133}, {6, 4512, 1, 8133}, {6, 4515, 1, 8133}, {7, 4518, 1, 8133}, {8, 4519, 1, 8133}, {8, 4520, 1, 8133}, {9, 4521, 1, 8133}, {4, 4513, 1, 8134}, {5, 4515, 2, 8134}, {6, 4516, 2, 8134}, {7, 4520, 1, 8134}, {8, 4521, 2, 8134}, {9, 4522, 1, 8134}, {4, 4523, 1, 8135}, {5, 4524, 2, 8135}, {6, 4525, 2, 8135}, {7, 4526, 1, 8135}, {8, 4527, 2, 8135}, {9, 4528, 1, 8135}, {4, 4529, 1, 8136}, {5, 4530, 1, 8136}, {5,

4535, 1, 8136}, {6, 4531, 1, 8136}, {6, 4541, 1, 8136}, {7, 4536, 1, 8136}, {8, 4537, 1,
8136}, {8, 4542, 1, 8136}, {9, 4543, 1, 8136}, {4, 4530, 1, 8137}, {5, 4532, 1, 8137}, {5,
4536, 1, 8137}, {6, 4533, 1, 8137}, {6, 4542, 1, 8137}, {7, 4538, 1, 8137}, {8, 4539, 1,
8137}, {8, 4544, 1, 8137}, {9, 4545, 1, 8137}, {4, 4531, 1, 8138}, {5, 4533, 1, 8138}, {5,
4537, 1, 8138}, {6, 4534, 1, 8138}, {6, 4543, 1, 8138}, {7, 4539, 1, 8138}, {8, 4540, 1,
8138}, {8, 4545, 1, 8138}, {9, 4546, 1, 8138}, {4, 4588, 1, 8139}, {5, 4589, 2, 8139}, {6,
4590, 2, 8139}, {7, 4591, 1, 8139}, {8, 4592, 2, 8139}, {9, 4593, 1, 8139}, {4, 4622, 1,
8140}, {5, 4623, 2, 8140}, {6, 4624, 2, 8140}, {7, 4625, 1, 8140}, {8, 4626, 2, 8140}, {9,
4627, 1, 8140}, {287, 2924, 1, 8141}, {288, 2925, 1, 8141}, {289, 2926, 1, 8141}, {287,
2927, 1, 8142}, {288, 2928, 1, 8142}, {289, 2929, 1, 8142}, {287, 2930, 1, 8143}, {288,
2931, 1, 8143}, {289, 2932, 1, 8143}, {290, 2924, 1, 8144}, {291, 2925, 1, 8144}, {292,
2926, 1, 8144}, {291, 2924, 1, 8145}, {293, 2925, 1, 8145}, {294, 2926, 1, 8145}, {292,
2924, 1, 8146}, {294, 2925, 1, 8146}, {295, 2926, 1, 8146}, {290, 2927, 1, 8147}, {291,
2928, 1, 8147}, {292, 2929, 1, 8147}, {291, 2927, 1, 8148}, {293, 2928, 1, 8148}, {294,
2929, 1, 8148}, {292, 2927, 1, 8149}, {294, 2928, 1, 8149}, {295, 2929, 1, 8149}, {290,
2930, 1, 8150}, {291, 2931, 1, 8150}, {292, 2932, 1, 8150}, {291, 2930, 1, 8151}, {293,
2931, 1, 8151}, {294, 2932, 1, 8151}, {292, 2930, 1, 8152}, {294, 2931, 1, 8152}, {295,
2932, 1, 8152}, {10, 4486, 1, 8153}, {11, 4487, 2, 8153}, {12, 4488, 2, 8153}, {13, 4489,
1, 8153}, {14, 4490, 2, 8153}, {15, 4491, 1, 8153}, {11, 4486, 1, 8154}, {13, 4487, 2,
8154}, {14, 4488, 2, 8154}, {16, 4489, 1, 8154}, {17, 4490, 2, 8154}, {18, 4491, 1, 8154},
{12, 4486, 1, 8155}, {14, 4487, 2, 8155}, {15, 4488, 2, 8155}, {17, 4489, 1, 8155}, {18,
4490, 2, 8155}, {19, 4491, 1, 8155}, {286, 3026, 1, 8156}, {287, 3027, 1, 8157}, {288,
3028, 1, 8157}, {289, 3029, 1, 8157}, {287, 3030, 1, 8158}, {288, 3031, 1, 8158}, {289,
3032, 1, 8158}, {287, 3031, 1, 8159}, {288, 3033, 1, 8159}, {289, 3034, 1, 8159}, {287,
3032, 1, 8160}, {288, 3034, 1, 8160}, {289, 3035, 1, 8160}, {290, 3027, 1, 8161}, {291,
3028, 1, 8161}, {292, 3029, 1, 8161}, {291, 3027, 1, 8162}, {293, 3028, 1, 8162}, {294,
3029, 1, 8162}, {292, 3027, 1, 8163}, {294, 3028, 1, 8163}, {295, 3029, 1, 8163}, {290,
3030, 1, 8164}, {291, 3031, 2, 8164}, {292, 3032, 2, 8164}, {293, 3033, 1, 8164}, {294,
3034, 2, 8164}, {295, 3035, 1, 8164}, {10, 4557, 1, 8165}, {11, 4558, 3, 8165}, {12, 4559,
3, 8165}, {13, 4560, 3, 8165}, {14, 4561, 6, 8165}, {15, 4562, 3, 8165}, {16, 4563, 1,
8165}, {17, 4564, 3, 8165}, {18, 4565, 3, 8165}, {19, 4566, 1, 8165}, {287, 2861, 1,
8166}, {288, 2862, 1, 8166}, {289, 2863, 1, 8166}, {287, 2862, 1, 8167}, {288, 2864, 1,
8167}, {289, 2865, 1, 8167}, {287, 2863, 1, 8168}, {288, 2865, 1, 8168}, {289, 2866, 1,
8168}, {10, 4428, 1, 8169}, {11, 4429, 2, 8169}, {12, 4430, 2, 8169}, {13, 4431, 1, 8169},
{14, 4432, 2, 8169}, {15, 4433, 1, 8169}, {11, 4428, 1, 8170}, {13, 4429, 2, 8170}, {14,
4430, 2, 8170}, {16, 4431, 1, 8170}, {17, 4432, 2, 8170}, {18, 4433, 1, 8170}, {12, 4428,
1, 8171}, {14, 4429, 2, 8171}, {15, 4430, 2, 8171}, {17, 4431, 1, 8171}, {18, 4432, 2,
8171}, {19, 4433, 1, 8171}, {10, 4434, 1, 8172}, {11, 4435, 1, 8172}, {11, 4440, 1, 8172},
{12, 4436, 1, 8172}, {12, 4446, 1, 8172}, {13, 4441, 1, 8172}, {14, 4442, 1, 8172}, {14,
4447, 1, 8172}, {15, 4448, 1, 8172}, {10, 4435, 1, 8173}, {11, 4437, 1, 8173}, {11, 4441,
1, 8173}, {12, 4438, 1, 8173}, {12, 4447, 1, 8173}, {13, 4443, 1, 8173}, {14, 4444, 1,
8173}, {14, 4449, 1, 8173}, {15, 4450, 1, 8173}, {10, 4436, 1, 8174}, {11, 4438, 1, 8174},
{11, 4442, 1, 8174}, {12, 4439, 1, 8174}, {12, 4448, 1, 8174}, {13, 4444, 1, 8174}, {14,
4445, 1, 8174}, {14, 4450, 1, 8174}, {15, 4451, 1, 8174}, {11, 4434, 1, 8175}, {13, 4435,
1, 8175}, {13, 4440, 1, 8175}, {14, 4436, 1, 8175}, {14, 4446, 1, 8175}, {16, 4441, 1,
8175}, {17, 4442, 1, 8175}, {17, 4447, 1, 8175}, {18, 4448, 1, 8175}, {11, 4435, 1, 8176},
{13, 4437, 1, 8176}, {13, 4441, 1, 8176}, {14, 4438, 1, 8176}, {14, 4447, 1, 8176}, {16,
4443, 1, 8176}, {17, 4444, 1, 8176}, {17, 4449, 1, 8176}, {18, 4450, 1, 8176}, {11, 4436,
1, 8177}, {13, 4438, 1, 8177}, {13, 4442, 1, 8177}, {14, 4439, 1, 8177}, {14, 4448, 1,
8177}, {16, 4444, 1, 8177}, {17, 4445, 1, 8177}, {17, 4450, 1, 8177}, {18, 4451, 1, 8177},
{12, 4434, 1, 8178}, {14, 4435, 1, 8178}, {14, 4440, 1, 8178}, {15, 4436, 1, 8178}, {15,
4446, 1, 8178}, {17, 4441, 1, 8178}, {18, 4442, 1, 8178}, {18, 4447, 1, 8178}, {19, 4448,
1, 8178}, {12, 4435, 1, 8179}, {14, 4437, 1, 8179}, {14, 4441, 1, 8179}, {15, 4438, 1,
8179}, {15, 4447, 1, 8179}, {17, 4443, 1, 8179}, {18, 4444, 1, 8179}, {18, 4449, 1, 8179},
{19, 4450, 1, 8179}, {12, 4436, 1, 8180}, {14, 4438, 1, 8180}, {14, 4442, 1, 8180}, {15,
4439, 1, 8180}, {15, 4448, 1, 8180}, {17, 4444, 1, 8180}, {18, 4445, 1, 8180}, {18, 4450,
1, 8180}, {19, 4451, 1, 8180}, {10, 4523, 1, 8181}, {11, 4524, 2, 8181}, {12, 4525, 2,
8181}, {13, 4526, 1, 8181}, {14, 4527, 2, 8181}, {15, 4528, 1, 8181}, {11, 4523, 1, 8182},
{13, 4524, 2, 8182}, {14, 4525, 2, 8182}, {16, 4526, 1, 8182}, {17, 4527, 2, 8182}, {18,
4528, 1, 8182}, {12, 4523, 1, 8183}, {14, 4524, 2, 8183}, {15, 4525, 2, 8183}, {17, 4526,
1, 8183}, {18, 4527, 2, 8183}, {19, 4528, 1, 8183}, {287, 2921, 1, 8184}, {288, 2922, 1,
8184}, {289, 2923, 1, 8184}, {287, 2924, 1, 8185}, {288, 2927, 1, 8185}, {289, 2930, 1,
8185}, {287, 2925, 1, 8186}, {288, 2928, 1, 8186}, {289, 2931, 1, 8186}, {287, 2926, 1,
8187}, {288, 2929, 1, 8187}, {289, 2932, 1, 8187}, {290, 2921, 1, 8188}, {291, 2922, 1,
8188}, {292, 2923, 1, 8188}, {291, 2921, 1, 8189}, {293, 2922, 1, 8189}, {294, 2923, 1,
8189}, {292, 2921, 1, 8190}, {294, 2922, 1, 8190}, {295, 2923, 1, 8190}, {290, 2924, 1,
8191}, {291, 2927, 1, 8191}, {292, 2930, 1, 8191}, {290, 2925, 1, 8192}, {291, 2928, 1,
8192}, {292, 2931, 1, 8192}, {290, 2926, 1, 8193}, {291, 2929, 1, 8193}, {292, 2932, 1,
8193}, {291, 2924, 1, 8194}, {293, 2927, 1, 8194}, {294, 2930, 1, 8194}, {291, 2925, 1,

8195}, {293, 2928, 1, 8195}, {294, 2931, 1, 8195}, {291, 2926, 1, 8196}, {293, 2929, 1,
8196}, {294, 2932, 1, 8196}, {292, 2924, 1, 8197}, {294, 2927, 1, 8197}, {295, 2930, 1,
8197}, {292, 2925, 1, 8198}, {294, 2928, 1, 8198}, {295, 2931, 1, 8198}, {292, 2926, 1,
8199}, {294, 2929, 1, 8199}, {295, 2932, 1, 8199}, {290, 2924, 1, 8200}, {291, 2925, 1,
8200}, {291, 2927, 1, 8200}, {292, 2926, 1, 8200}, {292, 2930, 1, 8200}, {293, 2928, 1,
8200}, {294, 2929, 1, 8200}, {294, 2931, 1, 8200}, {295, 2932, 1, 8200}, {290, 2933, 1,
8201}, {291, 2934, 1, 8201}, {291, 2939, 1, 8201}, {292, 2935, 1, 8201}, {292, 2945, 1,
8201}, {293, 2940, 1, 8201}, {294, 2941, 1, 8201}, {294, 2946, 1, 8201}, {295, 2947, 1,
8201}, {290, 2934, 1, 8202}, {291, 2936, 1, 8202}, {291, 2940, 1, 8202}, {292, 2937, 1,
8202}, {292, 2946, 1, 8202}, {293, 2942, 1, 8202}, {294, 2943, 1, 8202}, {294, 2948, 1,
8202}, {295, 2949, 1, 8202}, {290, 2935, 1, 8203}, {291, 2937, 1, 8203}, {291, 2941, 1,
8203}, {292, 2938, 1, 8203}, {292, 2947, 1, 8203}, {293, 2943, 1, 8203}, {294, 2944, 1,
8203}, {294, 2949, 1, 8203}, {295, 2950, 1, 8203}, {296, 2924, 1, 8204}, {297, 2925, 1,
8204}, {297, 2927, 1, 8204}, {298, 2926, 1, 8204}, {298, 2930, 1, 8204}, {299, 2928, 1,
8204}, {300, 2929, 1, 8204}, {300, 2931, 1, 8204}, {301, 2932, 1, 8204}, {297, 2924, 1,
8205}, {299, 2925, 1, 8205}, {299, 2927, 1, 8205}, {300, 2926, 1, 8205}, {300, 2930, 1,
8205}, {302, 2928, 1, 8205}, {303, 2929, 1, 8205}, {303, 2931, 1, 8205}, {304, 2932, 1,
8205}, {298, 2924, 1, 8206}, {300, 2925, 1, 8206}, {300, 2927, 1, 8206}, {301, 2926, 1,
8206}, {301, 2930, 1, 8206}, {303, 2928, 1, 8206}, {304, 2929, 1, 8206}, {304, 2931, 1,
8206}, {305, 2932, 1, 8206}, {10, 4529, 1, 8207}, {11, 4530, 2, 8207}, {11, 4535, 1,
8207}, {12, 4531, 2, 8207}, {12, 4541, 1, 8207}, {13, 4532, 1, 8207}, {13, 4536, 2, 8207},
{14, 4533, 2, 8207}, {14, 4537, 2, 8207}, {14, 4542, 2, 8207}, {15, 4534, 1, 8207}, {15,
4543, 2, 8207}, {16, 4538, 1, 8207}, {17, 4539, 2, 8207}, {17, 4544, 1, 8207}, {18, 4540,
1, 8207}, {18, 4545, 2, 8207}, {19, 4546, 1, 8207}, {10, 4594, 1, 8208}, {11, 4595, 2,
8208}, {11, 4600, 1, 8208}, {12, 4596, 2, 8208}, {12, 4606, 1, 8208}, {13, 4597, 1, 8208},
{13, 4601, 2, 8208}, {14, 4598, 2, 8208}, {14, 4602, 2, 8208}, {14, 4607, 2, 8208}, {15,
4599, 1, 8208}, {15, 4608, 2, 8208}, {16, 4603, 1, 8208}, {17, 4604, 2, 8208}, {17, 4609,
1, 8208}, {18, 4605, 1, 8208}, {18, 4610, 2, 8208}, {19, 4611, 1, 8208}, {287, 3184, 1,
8209}, {288, 3185, 1, 8209}, {289, 3186, 1, 8209}, {287, 3187, 1, 8210}, {288, 3188, 1,
8210}, {289, 3189, 1, 8210}, {287, 3190, 1, 8211}, {288, 3191, 1, 8211}, {289, 3192, 1,
8211}, {286, 3241, 1, 8212}, {287, 3242, 1, 8213}, {288, 3243, 1, 8213}, {289, 3244, 1,
8213}, {290, 3245, 1, 8214}, {291, 3246, 2, 8214}, {292, 3247, 2, 8214}, {293, 3248, 1,
8214}, {294, 3249, 2, 8214}, {295, 3250, 1, 8214}, {20, 4428, 1, 8215}, {21, 4429, 2,
8215}, {22, 4430, 2, 8215}, {23, 4431, 1, 8215}, {24, 4432, 2, 8215}, {25, 4433, 1, 8215},
{21, 4428, 1, 8216}, {23, 4429, 2, 8216}, {24, 4430, 2, 8216}, {26, 4431, 1, 8216}, {27,
4432, 2, 8216}, {28, 4433, 1, 8216}, {22, 4428, 1, 8217}, {24, 4429, 2, 8217}, {25, 4430,
2, 8217}, {27, 4431, 1, 8217}, {28, 4432, 2, 8217}, {29, 4433, 1, 8217}, {23, 4428, 1,
8218}, {26, 4429, 2, 8218}, {27, 4430, 2, 8218}, {30, 4431, 1, 8218}, {31, 4432, 2, 8218},
{32, 4433, 1, 8218}, {24, 4428, 1, 8219}, {27, 4429, 2, 8219}, {28, 4430, 2, 8219}, {31,
4431, 1, 8219}, {32, 4432, 2, 8219}, {33, 4433, 1, 8219}, {25, 4428, 1, 8220}, {28, 4429,
2, 8220}, {29, 4430, 2, 8220}, {32, 4431, 1, 8220}, {33, 4432, 2, 8220}, {34, 4433, 1,
8220}, {287, 3061, 1, 8221}, {288, 3062, 1, 8221}, {289, 3063, 1, 8221}, {287, 3062, 1,
8222}, {288, 3064, 1, 8222}, {289, 3065, 1, 8222}, {287, 3063, 1, 8223}, {288, 3065, 1,
8223}, {289, 3066, 1, 8223}, {290, 3061, 1, 8224}, {291, 3062, 1, 8224}, {292, 3063, 1,
8224}, {291, 3061, 1, 8225}, {293, 3062, 1, 8225}, {294, 3063, 1, 8225}, {292, 3061, 1,
8226}, {294, 3062, 1, 8226}, {295, 3063, 1, 8226}, {290, 3062, 1, 8227}, {291, 3064, 1,
8227}, {292, 3065, 1, 8227}, {291, 3062, 1, 8228}, {293, 3064, 1, 8228}, {294, 3065, 1,
8228}, {292, 3062, 1, 8229}, {294, 3064, 1, 8229}, {295, 3065, 1, 8229}, {290, 3063, 1,
8230}, {291, 3065, 1, 8230}, {292, 3066, 1, 8230}, {291, 3063, 1, 8231}, {293, 3065, 1,
8231}, {294, 3066, 1, 8231}, {292, 3063, 1, 8232}, {294, 3065, 1, 8232}, {295, 3066, 1,
8232}, {290, 3067, 1, 8233}, {291, 3068, 1, 8233}, {291, 3070, 1, 8233}, {292, 3069, 1,
8233}, {292, 3073, 1, 8233}, {293, 3071, 1, 8233}, {294, 3072, 1, 8233}, {294, 3074, 1,
8233}, {295, 3075, 1, 8233}, {290, 3070, 1, 8234}, {291, 3071, 1, 8234}, {291, 3076, 1,
8234}, {292, 3072, 1, 8234}, {292, 3079, 1, 8234}, {293, 3077, 1, 8234}, {294, 3078, 1,
8234}, {294, 3080, 1, 8234}, {295, 3081, 1, 8234}, {290, 3073, 1, 8235}, {291, 3074, 1,
8235}, {291, 3079, 1, 8235}, {292, 3075, 1, 8235}, {292, 3082, 1, 8235}, {293, 3080, 1,
8235}, {294, 3081, 1, 8235}, {294, 3083, 1, 8235}, {295, 3084, 1, 8235}, {287, 3181, 1,
8236}, {288, 3182, 1, 8236}, {289, 3183, 1, 8236}, {287, 3184, 1, 8237}, {288, 3187, 1,
8237}, {289, 3190, 1, 8237}, {287, 3185, 1, 8238}, {288, 3188, 1, 8238}, {289, 3191, 1,
8238}, {287, 3186, 1, 8239}, {288, 3189, 1, 8239}, {289, 3192, 1, 8239}, {290, 3181, 1,
8240}, {291, 3182, 1, 8240}, {292, 3183, 1, 8240}, {291, 3181, 1, 8241}, {293, 3182, 1,
8241}, {294, 3183, 1, 8241}, {292, 3181, 1, 8242}, {294, 3182, 1, 8242}, {295, 3183, 1,
8242}, {290, 3184, 1, 8243}, {291, 3185, 1, 8243}, {291, 3187, 1, 8243}, {292, 3186, 1,
8243}, {292, 3190, 1, 8243}, {293, 3188, 1, 8243}, {294, 3189, 1, 8243}, {294, 3191, 1,
8243}, {295, 3192, 1, 8243}, {296, 3193, 1, 8244}, {297, 3194, 2, 8244}, {297, 3199, 1,
8244}, {298, 3195, 2, 8244}, {298, 3205, 1, 8244}, {299, 3196, 1, 8244}, {299, 3200, 2,
8244}, {300, 3197, 2, 8244}, {300, 3201, 2, 8244}, {300, 3206, 2, 8244}, {301, 3198, 1,
8244}, {301, 3207, 2, 8244}, {302, 3202, 1, 8244}, {303, 3203, 2, 8244}, {303, 3208, 1,
8244}, {304, 3204, 1, 8244}, {304, 3209, 2, 8244}, {305, 3210, 1, 8244}, {290, 3061, 1,

8245}, {291, 3062, 2, 8245}, {292, 3063, 2, 8245}, {293, 3064, 1, 8245}, {294, 3065, 2, 8245}, {295, 3066, 1, 8245}, {290, 3067, 1, 8246}, {291, 3070, 2, 8246}, {292, 3073, 2, 8246}, {293, 3076, 1, 8246}, {294, 3079, 2, 8246}, {295, 3082, 1, 8246}, {290, 3068, 1, 8247}, {291, 3071, 2, 8247}, {292, 3074, 2, 8247}, {293, 3077, 1, 8247}, {294, 3080, 2, 8247}, {295, 3083, 1, 8247}, {290, 3069, 1, 8248}, {291, 3072, 2, 8248}, {292, 3075, 2, 8248}, {293, 3078, 1, 8248}, {294, 3081, 2, 8248}, {295, 3084, 1, 8248}, {296, 3067, 1, 8249}, {297, 3068, 1, 8249}, {297, 3070, 2, 8249}, {298, 3069, 1, 8249}, {298, 3073, 2, 8249}, {299, 3071, 2, 8249}, {299, 3076, 1, 8249}, {300, 3072, 2, 8249}, {300, 3074, 2, 8249}, {300, 3079, 2, 8249}, {301, 3075, 2, 8249}, {301, 3082, 1, 8249}, {302, 3077, 1, 8249}, {303, 3078, 1, 8249}, {303, 3080, 2, 8249}, {304, 3081, 2, 8249}, {304, 3083, 1, 8249}, {305, 3084, 1, 8249}, {20, 4567, 1, 8250}, {21, 4568, 4, 8250}, {22, 4569, 4, 8250}, {23, 4570, 2, 8250}, {23, 4573, 4, 8250}, {24, 4571, 4, 8250}, {24, 4574, 8, 8250}, {25, 4572, 2, 8250}, {25, 4578, 4, 8250}, {26, 4575, 4, 8250}, {27, 4576, 8, 8250}, {27, 4579, 4, 8250}, {28, 4577, 4, 8250}, {28, 4580, 8, 8250}, {29, 4581, 4, 8250}, {30, 4582, 1, 8250}, {31, 4583, 4, 8250}, {32, 4584, 2, 8250}, {32, 4585, 4, 8250}, {33, 4586, 4, 8250}, {34, 4587, 1, 8250}, {286, 3556, 1, 8251}, {287, 3557, 1, 8252}, {288, 3558, 1, 8252}, {289, 3559, 1, 8252}, {287, 3376, 1, 8253}, {288, 3377, 1, 8253}, {289, 3378, 1, 8253}, {287, 3377, 1, 8254}, {288, 3379, 1, 8254}, {289, 3380, 1, 8254}, {287, 3378, 1, 8255}, {288, 3380, 1, 8255}, {289, 3381, 1, 8255}, {287, 3496, 1, 8256}, {288, 3497, 1, 8256}, {289, 3498, 1, 8256}, {290, 3499, 1, 8257}, {291, 3500, 1, 8257}, {291, 3502, 1, 8257}, {292, 3501, 1, 8257}, {292, 3505, 1, 8257}, {293, 3503, 1, 8257}, {294, 3504, 1, 8257}, {294, 3506, 1, 8257}, {295, 3507, 1, 8257}, {290, 3276, 1, 8258}, {291, 3277, 2, 8258}, {292, 3278, 2, 8258}, {293, 3279, 1, 8258}, {294, 3280, 2, 8258}, {295, 3281, 1, 8258}, {290, 3277, 1, 8259}, {291, 3279, 2, 8259}, {292, 3280, 2, 8259}, {293, 3282, 1, 8259}, {294, 3283, 2, 8259}, {295, 3284, 1, 8259}, {290, 3278, 1, 8260}, {291, 3280, 2, 8260}, {292, 3281, 2, 8260}, {293, 3283, 1, 8260}, {294, 3284, 2, 8260}, {295, 3285, 1, 8260}, {290, 3376, 1, 8261}, {291, 3377, 2, 8261}, {292, 3378, 2, 8261}, {293, 3379, 1, 8261}, {294, 3380, 2, 8261}, {295, 3381, 1, 8261}, {296, 3382, 1, 8262}, {297, 3383, 1, 8262}, {297, 3385, 2, 8262}, {298, 3384, 1, 8262}, {298, 3388, 2, 8262}, {299, 3386, 2, 8262}, {299, 3391, 1, 8262}, {300, 3387, 2, 8262}, {300, 3389, 2, 8262}, {300, 3394, 2, 8262}, {301, 3390, 2, 8262}, {301, 3397, 1, 8262}, {302, 3392, 1, 8262}, {303, 3393, 1, 8262}, {303, 3395, 2, 8262}, {304, 3396, 2, 8262}, {304, 3398, 1, 8262}, {305, 3399, 1, 8262}, {286, 3826, 1, 8263}, {287, 3796, 1, 8264}, {288, 3797, 1, 8264}, {289, 3798, 1, 8264}, {290, 3736, 1, 8265}, {291, 3737, 2, 8265}, {292, 3738, 2, 8265}, {293, 3739, 1, 8265}, {294, 3740, 2, 8265}, {295, 3741, 1, 8265}, {296, 3636, 1, 8266}, {297, 3637, 3, 8266}, {298, 3638, 3, 8266}, {299, 3639, 3, 8266}, {300, 3640, 6, 8266}, {301, 3641, 3, 8266}, {302, 3642, 1, 8266}, {303, 3643, 3, 8266}, {304, 3644, 3, 8266}, {305, 3645, 1, 8266}, {0, 4634, 1, 8267}, {0, 4635, 1, 8268}, {0, 4657, 1, 8269}, {0, 4685, 1, 8270}, {0, 4698, 1, 8271}, {286, 3968, 1, 8272}, {1, 4636, 1, 8273}, {2, 4637, 1, 8273}, {3, 4638, 1, 8273}, {1, 4639, 1, 8274}, {2, 4640, 1, 8274}, {3, 4641, 1, 8274}, {1, 4640, 1, 8275}, {2, 4642, 1, 8275}, {3, 4643, 1, 8275}, {1, 4641, 1, 8276}, {2, 4643, 1, 8276}, {3, 4644, 1, 8276}, {1, 4658, 1, 8277}, {2, 4659, 1, 8277}, {3, 4660, 1, 8277}, {1, 4686, 1, 8278}, {2, 4687, 1, 8278}, {3, 4688, 1, 8278}, {506, 2756, 1, 8279}, {1, 4645, 1, 8280}, {2, 4646, 1, 8280}, {3, 4647, 1, 8280}, {1, 4648, 1, 8281}, {2, 4651, 1, 8281}, {3, 4654, 1, 8281}, {1, 4649, 1, 8282}, {2, 4652, 1, 8282}, {3, 4655, 1, 8282}, {1, 4650, 1, 8283}, {2, 4653, 1, 8283}, {3, 4656, 1, 8283}, {1, 4667, 1, 8284}, {2, 4668, 1, 8284}, {3, 4669, 1, 8284}, {1, 4668, 1, 8285}, {2, 4670, 1, 8285}, {3, 4671, 1, 8285}, {1, 4669, 1, 8286}, {2, 4671, 1, 8286}, {3, 4672, 1, 8286}, {1, 4673, 1, 8287}, {2, 4674, 1, 8287}, {3, 4675, 1, 8287}, {1, 4695, 1, 8288}, {2, 4696, 1, 8288}, {3, 4697, 1, 8288}, {4, 4636, 1, 8289}, {5, 4637, 1, 8289}, {6, 4638, 1, 8289}, {5, 4636, 1, 8290}, {7, 4637, 1, 8290}, {8, 4638, 1, 8290}, {6, 4636, 1, 8291}, {8, 4637, 1, 8291}, {9, 4638, 1, 8291}, {286, 4038, 1, 8292}, {287, 4039, 1, 8293}, {288, 4040, 1, 8293}, {289, 4041, 1, 8293}, {4, 4661, 1, 8294}, {5, 4662, 2, 8294}, {6, 4663, 2, 8294}, {7, 4664, 1, 8294}, {8, 4665, 2, 8294}, {9, 4666, 1, 8294}, {4, 4645, 1, 8295}, {5, 4646, 1, 8295}, {6, 4647, 1, 8295}, {5, 4645, 1, 8296}, {7, 4646, 1, 8296}, {8, 4647, 1, 8296}, {6, 4645, 1, 8297}, {8, 4646, 1, 8297}, {9, 4647, 1, 8297}, {287, 3978, 1, 8298}, {288, 3979, 1, 8298}, {289, 3980, 1, 8298}, {287, 3981, 1, 8299}, {288, 3984, 1, 8299}, {289, 3987, 1, 8299}, {287, 3982, 1, 8300}, {288, 3985, 1, 8300}, {289, 3988, 1, 8300}, {287, 3983, 1, 8301}, {288, 3986, 1, 8301}, {289, 3989, 1, 8301}, {290, 3978, 1, 8302}, {291, 3979, 1, 8302}, {292, 3980, 1, 8302}, {291, 3978, 1, 8303}, {293, 3979, 1, 8303}, {294, 3980, 1, 8303}, {292, 3978, 1, 8304}, {294, 3979, 1, 8304}, {295, 3980, 1, 8304}, {4, 4648, 1, 8305}, {5, 4649, 1, 8305}, {5, 4651, 1, 8305}, {6, 4650, 1, 8305}, {6, 4654, 1, 8305}, {7, 4652, 1, 8305}, {8, 4653, 1, 8305}, {8, 4655, 1, 8305}, {9, 4656, 1, 8305}, {4, 4676, 1, 8306}, {5, 4677, 1, 8306}, {5, 4679, 1, 8306}, {6, 4678, 1, 8306}, {6, 4682, 1, 8306}, {7, 4680, 1, 8306}, {8, 4681, 1, 8306}, {8, 4683, 1, 8306}, {9, 4684, 1, 8306}, {506, 2826, 1, 8307}, {507, 2827, 1, 8308}, {508, 2828, 1, 8308}, {509, 2829, 1, 8308}, {4, 4689, 1, 8309}, {5, 4690, 2, 8309}, {6, 4691, 2, 8309}, {7, 4692, 1, 8309}, {8, 4693, 2, 8309}, {9, 4694, 1, 8309}, {286, 4148, 1, 8310}, {287, 4149, 1, 8311}, {288, 4150, 1, 8311}, {289, 4151, 1, 8311}, {287, 4058, 1, 8312}, {288, 4059, 1, 8312}, {289, 4060, 1, 8312}, {287, 4059, 1, 8313}, {288, 4061, 1, 8313}, {289,

4062, 1, 8313}, {287, 4060, 1, 8314}, {288, 4062, 1, 8314}, {289, 4063, 1, 8314}, {287,
4118, 1, 8315}, {288, 4119, 1, 8315}, {289, 4120, 1, 8315}, {290, 4121, 1, 8316}, {291,
4122, 1, 8316}, {291, 4124, 1, 8316}, {292, 4123, 1, 8316}, {292, 4127, 1, 8316}, {293,
4125, 1, 8316}, {294, 4126, 1, 8316}, {294, 4128, 1, 8316}, {295, 4129, 1, 8316}, {507,
2921, 1, 8317}, {508, 2922, 1, 8317}, {509, 2923, 1, 8317}, {510, 2924, 1, 8318}, {511,
2925, 1, 8318}, {511, 2927, 1, 8318}, {512, 2926, 1, 8318}, {512, 2930, 1, 8318}, {513,
2928, 1, 8318}, {514, 2929, 1, 8318}, {514, 2931, 1, 8318}, {515, 2932, 1, 8318}, {506,
3026, 1, 8319}, {507, 3027, 1, 8320}, {508, 3028, 1, 8320}, {509, 3029, 1, 8320}, {286,
4258, 1, 8321}, {287, 4228, 1, 8322}, {288, 4229, 1, 8322}, {289, 4230, 1, 8322}, {290,
4168, 1, 8323}, {291, 4169, 2, 8323}, {292, 4170, 2, 8323}, {293, 4171, 1, 8323}, {294,
4172, 2, 8323}, {295, 4173, 1, 8323}, {507, 3181, 1, 8324}, {508, 3182, 1, 8324}, {509,
3183, 1, 8324}, {506, 3241, 1, 8325}, {0, 4700, 1, 8326}, {0, 4701, 1, 8327}, {0, 4708, 1,
8328}, {286, 4340, 1, 8329}, {1, 4702, 1, 8330}, {2, 4703, 1, 8330}, {3, 4704, 1, 8330},
{626, 2756, 1, 8331}, {1, 4705, 1, 8332}, {2, 4706, 1, 8332}, {3, 4707, 1, 8332}, {286,
4362, 1, 8333}, {287, 4350, 1, 8334}, {288, 4351, 1, 8334}, {289, 4352, 1, 8334}, {626,
2826, 1, 8335}, {0, 4710, 1, 8336}, {0, 4712, 1, 8337}, {0, 4713, 1, 8338}, {0, 4717, 1,
8339}, {506, 3968, 1, 8340}, {1, 4714, 1, 8341}, {2, 4715, 1, 8341}, {3, 4716, 1, 8341},
{506, 4038, 1, 8342}, {507, 3978, 1, 8343}, {508, 3979, 1, 8343}, {509, 3980, 1, 8343},
{0, 4719, 1, 8344}, {286, 4417, 1, 8345}, {286, 4418, 1, 8346}, {286, 4482, 1, 8347},
{286, 4547, 1, 8348}, {286, 4612, 1, 8349}, {287, 4419, 1, 8350}, {288, 4420, 1, 8350},
{289, 4421, 1, 8350}, {287, 4422, 1, 8351}, {288, 4423, 1, 8351}, {289, 4424, 1, 8351},
{287, 4423, 1, 8352}, {288, 4425, 1, 8352}, {289, 4426, 1, 8352}, {287, 4424, 1, 8353},
{288, 4426, 1, 8353}, {289, 4427, 1, 8353}, {287, 4428, 1, 8354}, {288, 4429, 1, 8354},
{289, 4430, 1, 8354}, {287, 4429, 1, 8355}, {288, 4431, 1, 8355}, {289, 4432, 1, 8355},
{287, 4430, 1, 8356}, {288, 4432, 1, 8356}, {289, 4433, 1, 8356}, {287, 4483, 1, 8357},
{288, 4484, 1, 8357}, {289, 4485, 1, 8357}, {287, 4548, 1, 8358}, {288, 4549, 1, 8358},
{289, 4550, 1, 8358}, {290, 4486, 1, 8359}, {291, 4487, 2, 8359}, {292, 4488, 2, 8359},
{293, 4489, 1, 8359}, {294, 4490, 2, 8359}, {295, 4491, 1, 8359}, {290, 4428, 1, 8360},
{291, 4429, 2, 8360}, {292, 4430, 2, 8360}, {293, 4431, 1, 8360}, {294, 4432, 2, 8360},
{295, 4433, 1, 8360}, {290, 4523, 1, 8361}, {291, 4524, 2, 8361}, {292, 4525, 2, 8361},
{293, 4526, 1, 8361}, {294, 4527, 2, 8361}, {295, 4528, 1, 8361}, {286, 4634, 1, 8362},
{286, 4635, 1, 8363}, {286, 4657, 1, 8364}, {287, 4636, 1, 8365}, {288, 4637, 1, 8365},
{289, 4638, 1, 8365}, {506, 4418, 1, 8366}, {287, 4645, 1, 8367}, {288, 4646, 1, 8367},
{289, 4647, 1, 8367}, {506, 4482, 1, 8368}, {286, 4700, 1, 8369}, {286, 4712, 1, 8370},
{707, 707, 1, 8371}, {707, 771, 1, 8372}, {707, 989, 1, 8373}, {707, 1532, 1, 8374}, {707,
2063, 1, 8375}, {707, 2444, 1, 8376}, {707, 2669, 1, 8377}, {707, 2734, 1, 8378}, {771,
771, 1, 8379}, {772, 772, 1, 8380}, {773, 773, 1, 8380}, {774, 774, 1, 8380}, {771, 989,
1, 8381}, {772, 990, 1, 8382}, {773, 991, 1, 8382}, {774, 992, 1, 8382}, {771, 1532, 1,
8383}, {772, 1533, 1, 8384}, {773, 1534, 1, 8384}, {774, 1535, 1, 8384}, {771, 2063, 1,
8385}, {772, 2064, 1, 8386}, {773, 2065, 1, 8386}, {774, 2066, 1, 8386}, {771, 2444, 1,
8387}, {772, 2445, 1, 8388}, {773, 2446, 1, 8388}, {774, 2447, 1, 8388}, {771, 2669, 1,
8389}, {827, 993, 1, 8390}, {828, 994, 2, 8390}, {829, 995, 2, 8390}, {830, 996, 1, 8390},
{831, 997, 2, 8390}, {832, 998, 1, 8390}, {989, 989, 1, 8391}, {990, 990, 1, 8392}, {991,
991, 1, 8392}, {992, 992, 1, 8392}, {993, 993, 1, 8393}, {994, 994, 2, 8393}, {995, 995,
2, 8393}, {996, 996, 1, 8393}, {997, 997, 2, 8393}, {998, 998, 1, 8393}, {993, 1370, 1,
8394}, {994, 1371, 2, 8394}, {995, 1372, 2, 8394}, {996, 1373, 1, 8394}, {997, 1374, 2,
8394}, {998, 1375, 1, 8394}, {989, 1532, 1, 8395}, {990, 1533, 1, 8396}, {991, 1534, 1,
8396}, {992, 1535, 1, 8396}, {993, 1536, 1, 8397}, {994, 1537, 2, 8397}, {995, 1538, 2,
8397}, {996, 1539, 1, 8397}, {997, 1540, 2, 8397}, {998, 1541, 1, 8397}, {993, 1964, 1,
8398}, {994, 1965, 2, 8398}, {995, 1966, 2, 8398}, {996, 1967, 1, 8398}, {997, 1968, 2,
8398}, {998, 1969, 1, 8398}, {989, 2063, 1, 8399}, {990, 2064, 1, 8400}, {991, 2065, 1,
8400}, {992, 2066, 1, 8400}, {989, 2444, 1, 8401}, {827, 827, 1, 8402}, {828, 828, 2,
8402}, {829, 829, 2, 8402}, {830, 830, 1, 8402}, {831, 831, 2, 8402}, {832, 832, 1, 8402},
{827, 1370, 1, 8403}, {828, 1371, 2, 8403}, {829, 1372, 2, 8403}, {830, 1373, 1, 8403},
{831, 1374, 2, 8403}, {832, 1375, 1, 8403}, {827, 1536, 1, 8404}, {828, 1537, 2, 8404},
{829, 1538, 2, 8404}, {830, 1539, 1, 8404}, {831, 1540, 2, 8404}, {832, 1541, 1, 8404},
{827, 1964, 1, 8405}, {828, 1965, 2, 8405}, {829, 1966, 2, 8405}, {830, 1967, 1, 8405},
{831, 1968, 2, 8405}, {832, 1969, 1, 8405}, {827, 2067, 1, 8406}, {828, 2068, 2, 8406},
{829, 2069, 2, 8406}, {830, 2070, 1, 8406}, {831, 2071, 2, 8406}, {832, 2072, 1, 8406},
{827, 2345, 1, 8407}, {828, 2346, 2, 8407}, {829, 2347, 2, 8407}, {830, 2348, 1, 8407},
{831, 2349, 2, 8407}, {832, 2350, 1, 8407}, {1370, 1536, 1, 8408}, {1371, 1537, 2, 8408},
{1372, 1538, 2, 8408}, {1373, 1539, 1, 8408}, {1374, 1540, 2, 8408}, {1375, 1541, 1,
8408}, {1532, 1532, 1, 8409}, {1533, 1533, 1, 8410}, {1534, 1534, 1, 8410}, {1535, 1535,
1, 8410}, {1532, 2063, 1, 8411}, {1370, 1370, 1, 8412}, {1371, 1371, 2, 8412}, {1372,
1372, 2, 8412}, {1373, 1373, 1, 8412}, {1374, 1374, 2, 8412}, {1375, 1375, 1, 8412},
{1376, 1376, 1, 8413}, {1377, 1377, 1, 8413}, {1377, 1382, 2, 8413}, {1378, 1378, 1,
8413}, {1378, 1388, 2, 8413}, {1379, 1383, 2, 8413}, {1380, 1384, 2, 8413}, {1380, 1389,
2, 8413}, {1381, 1390, 2, 8413}, {1383, 1383, 1, 8413}, {1384, 1389, 2, 8413}, {1385,
1385, 1, 8413}, {1386, 1386, 1, 8413}, {1386, 1391, 2, 8413}, {1387, 1392, 2, 8413},

{1390, 1390, 1, 8413}, {1392, 1392, 1, 8413}, {1393, 1393, 1, 8413}, {1376, 1376, 1, 8414}, {1377, 1377, 2, 8414}, {1378, 1378, 2, 8414}, {1379, 1379, 1, 8414}, {1380, 1380, 2, 8414}, {1381, 1381, 1, 8414}, {1382, 1382, 1, 8414}, {1383, 1383, 2, 8414}, {1384, 1384, 2, 8414}, {1385, 1385, 1, 8414}, {1386, 1386, 2, 8414}, {1387, 1387, 1, 8414}, {1388, 1388, 1, 8414}, {1389, 1389, 2, 8414}, {1390, 1390, 2, 8414}, {1391, 1391, 1, 8414}, {1392, 1392, 2, 8414}, {1393, 1393, 1, 8414}, {1370, 1964, 1, 8415}, {1371, 1965, 2, 8415}, {1372, 1966, 2, 8415}, {1373, 1967, 1, 8415}, {1374, 1968, 2, 8415}, {1375, 1969, 1, 8415}, {707, 2756, 1, 8416}, {771, 2756, 1, 8417}, {989, 2756, 1, 8418}, {1532, 2756, 1, 8419}, {2063, 2756, 1, 8420}, {2444, 2756, 1, 8421}, {707, 2826, 1, 8422}, {707, 3026, 1, 8423}, {707, 3241, 1, 8424}, {707, 3556, 1, 8425}, {707, 3826, 1, 8426}, {771, 2826, 1, 8427}, {772, 2827, 1, 8428}, {773, 2828, 1, 8428}, {774, 2829, 1, 8428}, {989, 2826, 1, 8429}, {990, 2827, 1, 8430}, {991, 2828, 1, 8430}, {992, 2829, 1, 8430}, {1532, 2826, 1, 8431}, {1533, 2827, 1, 8432}, {1534, 2828, 1, 8432}, {1535, 2829, 1, 8432}, {2063, 2826, 1, 8433}, {771, 3026, 1, 8434}, {772, 3027, 1, 8435}, {773, 3028, 1, 8435}, {774, 3029, 1, 8435}, {772, 2921, 1, 8436}, {773, 2922, 1, 8436}, {774, 2923, 1, 8436}, {771, 3241, 1, 8437}, {772, 3242, 1, 8438}, {773, 3243, 1, 8438}, {774, 3244, 1, 8438}, {772, 3181, 1, 8439}, {773, 3182, 1, 8439}, {774, 3183, 1, 8439}, {771, 3556, 1, 8440}, {772, 3496, 1, 8441}, {773, 3497, 1, 8441}, {774, 3498, 1, 8441}, {827, 3030, 1, 8442}, {828, 3031, 2, 8442}, {829, 3032, 2, 8442}, {830, 3033, 1, 8442}, {831, 3034, 2, 8442}, {832, 3035, 1, 8442}, {989, 3026, 1, 8443}, {990, 3027, 1, 8444}, {991, 3028, 1, 8444}, {992, 3029, 1, 8444}, {1532, 3026, 1, 8445}, {827, 2924, 1, 8446}, {828, 2925, 1, 8446}, {828, 2927, 1, 8446}, {829, 2926, 1, 8446}, {829, 2930, 1, 8446}, {830, 2928, 1, 8446}, {831, 2929, 1, 8446}, {831, 2931, 1, 8446}, {832, 2932, 1, 8446}, {990, 2921, 1, 8447}, {991, 2922, 1, 8447}, {992, 2923, 1, 8447}, {993, 2924, 1, 8448}, {994, 2925, 1, 8448}, {994, 2927, 1, 8448}, {995, 2926, 1, 8448}, {995, 2930, 1, 8448}, {996, 2928, 1, 8448}, {997, 2929, 1, 8448}, {997, 2931, 1, 8448}, {998, 2932, 1, 8448}, {1370, 2924, 1, 8449}, {1371, 2925, 1, 8449}, {1371, 2927, 1, 8449}, {1372, 2926, 1, 8449}, {1372, 2930, 1, 8449}, {1373, 2928, 1, 8449}, {1374, 2929, 1, 8449}, {1374, 2931, 1, 8449}, {1375, 2932, 1, 8449}, {1533, 2921, 1, 8450}, {1534, 2922, 1, 8450}, {1535, 2923, 1, 8450}, {827, 3184, 1, 8451}, {828, 3185, 1, 8451}, {828, 3187, 1, 8451}, {829, 3186, 1, 8451}, {829, 3190, 1, 8451}, {830, 3188, 1, 8451}, {831, 3189, 1, 8451}, {831, 3191, 1, 8451}, {832, 3192, 1, 8451}, {989, 3241, 1, 8452}, {990, 3181, 1, 8453}, {991, 3182, 1, 8453}, {992, 3183, 1, 8453}, {827, 3376, 1, 8454}, {828, 3377, 2, 8454}, {829, 3378, 2, 8454}, {830, 3379, 1, 8454}, {831, 3380, 2, 8454}, {832, 3381, 1, 8454}, {707, 3968, 1, 8455}, {771, 3968, 1, 8456}, {989, 3968, 1, 8457}, {1532, 3968, 1, 8458}, {707, 4038, 1, 8459}, {707, 4148, 1, 8460}, {707, 4258, 1, 8461}, {771, 4038, 1, 8462}, {772, 4039, 1, 8463}, {773, 4040, 1, 8463}, {774, 4041, 1, 8463}, {989, 4038, 1, 8464}, {771, 4148, 1, 8465}, {772, 4118, 1, 8466}, {773, 4119, 1, 8466}, {774, 4120, 1, 8466}, {707, 4340, 1, 8467}, {771, 4340, 1, 8468}, {707, 4362, 1, 8469}, {2756, 2756, 1, 8470}, {2756, 2826, 1, 8471}, {2756, 3026, 1, 8472}, {2756, 3241, 1, 8473}, {707, 4418, 1, 8474}, {707, 4482, 1, 8475}, {707, 4547, 1, 8476}, {707, 4612, 1, 8477}, {2826, 2826, 1, 8478}, {2827, 2827, 1, 8479}, {2828, 2828, 1, 8479}, {2829, 2829, 1, 8479}, {2827, 2921, 1, 8480}, {2828, 2922, 1, 8480}, {2829, 2923, 1, 8480}, {2826, 3026, 1, 8481}, {771, 4418, 1, 8482}, {771, 4482, 1, 8483}, {771, 4547, 1, 8484}, {772, 4483, 1, 8485}, {773, 4484, 1, 8485}, {774, 4485, 1, 8485}, {2921, 2921, 1, 8486}, {2922, 2922, 1, 8486}, {2923, 2923, 1, 8486}, {989, 4418, 1, 8487}, {989, 4482, 1, 8488}, {1532, 4418, 1, 8489}, {2756, 3968, 1, 8490}, {2826, 3968, 1, 8491}, {2756, 4038, 1, 8492}, {707, 4635, 1, 8493}, {707, 4657, 1, 8494}, {771, 4635, 1, 8495}, {2756, 4418, 1, 8496}, {2756, 4482, 1, 8497}, {2826, 4418, 1, 8498}, {0, 4721, 1, 8499}, {0, 4722, 1, 8500}, {0, 4723, 1, 8501}, {0, 4724, 1, 8502}, {0, 4725, 1, 8503}, {0, 4726, 1, 8504}, {0, 4727, 1, 8505}, {0, 4728, 1, 8506}, {0, 4729, 1, 8507}, {0, 4730, 1, 8508}, {0, 4732, 1, 8509}, {0, 4739, 1, 8510}, {0, 4746, 1, 8511}, {0, 4753, 1, 8512}, {0, 4760, 1, 8513}, {0, 4767, 1, 8514}, {0, 4774, 1, 8515}, {0, 4781, 1, 8516}, {0, 4891, 1, 8517}, {0, 4904, 1, 8518}, {0, 4923, 1, 8519}, {0, 4942, 1, 8520}, {0, 4961, 1, 8521}, {0, 4974, 1, 8522}, {0, 4976, 1, 8523}, {0, 4992, 1, 8524}, {0, 5008, 1, 8525}, {0, 5024, 1, 8526}, {0, 5040, 1, 8527}, {0, 5056, 1, 8528}, {0, 5060, 1, 8529}, {0, 5209, 1, 8530}, {0, 5247, 1, 8531}, {0, 5275, 1, 8532}, {0, 5298, 1, 8533}, {0, 5477, 1, 8534}, {0, 5551, 1, 8535}, {0, 5625, 1, 8536}, {0, 5681, 1, 8537}, {0, 5704, 1, 8538}, {0, 5922, 1, 8539}, {0, 5935, 1, 8540}, {0, 6363, 1, 8541}, {0, 6409, 1, 8542}, {0, 6432, 1, 8543}, {0, 6434, 1, 8544}, {0, 6514, 1, 8545}, {0, 6530, 1, 8546}, {0, 6534, 1, 8547}, {0, 6573, 1, 8548}, {0, 6608, 1, 8549}, {0, 6621, 1, 8550}, {0, 6624, 1, 8551}, {1, 4733, 1, 8552}, {2, 4734, 1, 8552}, {3, 4735, 1, 8552}, {1, 4740, 1, 8553}, {2, 4741, 1, 8553}, {3, 4742, 1, 8553}, {1, 4747, 1, 8554}, {2, 4748, 1, 8554}, {3, 4749, 1, 8554}, {1, 4754, 1, 8555}, {2, 4755, 1, 8555}, {3, 4756, 1, 8555}, {1, 4761, 1, 8556}, {2, 4762, 1, 8556}, {3, 4763, 1, 8556}, {1, 4768, 1, 8557}, {2, 4769, 1, 8557}, {3, 4770, 1, 8557}, {1, 4775, 1, 8558}, {2, 4776, 1, 8558}, {3, 4777, 1, 8558}, {1, 4736, 1, 8559}, {2, 4737, 1, 8559}, {3, 4738, 1, 8559}, {1, 4743, 1, 8560}, {2, 4744, 1, 8560}, {3, 4745, 1, 8560}, {1, 4750, 1, 8561}, {2, 4751, 1, 8561}, {3, 4752, 1, 8561}, {1, 4757, 1, 8562}, {2, 4758, 1, 8562}, {3, 4759, 1, 8562}, {1, 4764, 1, 8563}, {2, 4765, 1, 8563}, {3, 4766, 1, 8563}, {1, 4771, 1, 8564}, {2, 4772, 1, 8564}, {3, 4773, 1, 8564}, {1, 4778, 1, 8565}, {2, 4779, 1, 8565}, {3, 4780, 1, 8565}, {1, 4892, 1,

8566}, {2, 4893, 1, 8566}, {3, 4894, 1, 8566}, {1, 4905, 1, 8567}, {2, 4906, 1, 8567}, {3, 4907, 1, 8567}, {1, 4924, 1, 8568}, {2, 4925, 1, 8568}, {3, 4926, 1, 8568}, {1, 4943, 1, 8569}, {2, 4944, 1, 8569}, {3, 4945, 1, 8569}, {1, 4962, 1, 8570}, {2, 4963, 1, 8570}, {3, 4964, 1, 8570}, {1, 4977, 1, 8571}, {2, 4978, 1, 8571}, {3, 4979, 1, 8571}, {1, 4993, 1, 8572}, {2, 4994, 1, 8572}, {3, 4995, 1, 8572}, {1, 5009, 1, 8573}, {2, 5010, 1, 8573}, {3, 5011, 1, 8573}, {1, 5025, 1, 8574}, {2, 5026, 1, 8574}, {3, 5027, 1, 8574}, {1, 5041, 1, 8575}, {2, 5042, 1, 8575}, {3, 5043, 1, 8575}, {1, 5057, 1, 8576}, {2, 5058, 1, 8576}, {3, 5059, 1, 8576}, {1, 4792, 1, 8577}, {2, 4793, 1, 8577}, {3, 4794, 1, 8577}, {1, 4901, 1, 8578}, {2, 4902, 1, 8578}, {3, 4903, 1, 8578}, {1, 4920, 1, 8579}, {2, 4921, 1, 8579}, {3, 4922, 1, 8579}, {1, 4939, 1, 8580}, {2, 4940, 1, 8580}, {3, 4941, 1, 8580}, {1, 4958, 1, 8581}, {2, 4959, 1, 8581}, {3, 4960, 1, 8581}, {1, 4971, 1, 8582}, {2, 4972, 1, 8582}, {3, 4973, 1, 8582}, {1, 5210, 1, 8583}, {2, 5211, 1, 8583}, {3, 5212, 1, 8583}, {1, 5248, 1, 8584}, {2, 5249, 1, 8584}, {3, 5250, 1, 8584}, {1, 5276, 1, 8585}, {2, 5277, 1, 8585}, {3, 5278, 1, 8585}, {1, 5478, 1, 8586}, {2, 5479, 1, 8586}, {3, 5480, 1, 8586}, {1, 5552, 1, 8587}, {2, 5553, 1, 8587}, {3, 5554, 1, 8587}, {1, 5626, 1, 8588}, {2, 5627, 1, 8588}, {3, 5628, 1, 8588}, {1, 5682, 1, 8589}, {2, 5683, 1, 8589}, {3, 5684, 1, 8589}, {1, 5318, 1, 8590}, {2, 5319, 1, 8590}, {3, 5320, 1, 8590}, {1, 5521, 1, 8591}, {2, 5522, 1, 8591}, {3, 5523, 1, 8591}, {1, 5595, 1, 8592}, {2, 5596, 1, 8592}, {3, 5597, 1, 8592}, {1, 5669, 1, 8593}, {2, 5670, 1, 8593}, {3, 5671, 1, 8593}, {1, 5701, 1, 8594}, {2, 5702, 1, 8594}, {3, 5703, 1, 8594}, {1, 4804, 1, 8595}, {2, 4805, 1, 8595}, {3, 4806, 1, 8595}, {1, 5071, 1, 8596}, {2, 5072, 1, 8596}, {3, 5073, 1, 8596}, {1, 5235, 1, 8597}, {2, 5236, 1, 8597}, {3, 5237, 1, 8597}, {1, 5263, 1, 8598}, {2, 5264, 1, 8598}, {3, 5265, 1, 8598}, {1, 5295, 1, 8599}, {2, 5296, 1, 8599}, {3, 5297, 1, 8599}, {1, 5923, 1, 8600}, {2, 5924, 1, 8600}, {3, 5925, 1, 8600}, {1, 6364, 1, 8601}, {2, 6365, 1, 8601}, {3, 6366, 1, 8601}, {1, 6410, 1, 8602}, {2, 6411, 1, 8602}, {3, 6412, 1, 8602}, {1, 6435, 1, 8603}, {2, 6436, 1, 8603}, {3, 6437, 1, 8603}, {1, 6515, 1, 8604}, {2, 6516, 1, 8604}, {3, 6517, 1, 8604}, {1, 6531, 1, 8605}, {2, 6532, 1, 8605}, {3, 6533, 1, 8605}, {1, 5366, 1, 8606}, {2, 5367, 1, 8606}, {3, 5368, 1, 8606}, {1, 6153, 1, 8607}, {2, 6154, 1, 8607}, {3, 6155, 1, 8607}, {1, 6397, 1, 8608}, {2, 6398, 1, 8608}, {3, 6399, 1, 8608}, {1, 6429, 1, 8609}, {2, 6430, 1, 8609}, {3, 6431, 1, 8609}, {1, 4825, 1, 8610}, {2, 4826, 1, 8610}, {3, 4827, 1, 8610}, {1, 5110, 1, 8611}, {2, 5111, 1, 8611}, {3, 5112, 1, 8611}, {1, 5880, 1, 8612}, {2, 5881, 1, 8612}, {3, 5882, 1, 8612}, {1, 5932, 1, 8613}, {2, 5933, 1, 8613}, {3, 5934, 1, 8613}, {1, 6609, 1, 8614}, {2, 6610, 1, 8614}, {3, 6611, 1, 8614}, {1, 6593, 1, 8615}, {2, 6594, 1, 8615}, {3, 6595, 1, 8615}, {1, 6618, 1, 8616}, {2, 6619, 1, 8616}, {3, 6620, 1, 8616}, {1, 5414, 1, 8617}, {2, 5415, 1, 8617}, {3, 5416, 1, 8617}, {1, 6291, 1, 8618}, {2, 6292, 1, 8618}, {3, 6293, 1, 8618}, {1, 6570, 1, 8619}, {2, 6571, 1, 8619}, {3, 6572, 1, 8619}, {1, 4846, 1, 8620}, {2, 4847, 1, 8620}, {3, 4848, 1, 8620}, {1, 5167, 1, 8621}, {2, 5168, 1, 8621}, {3, 5169, 1, 8621}, {1, 5919, 1, 8622}, {2, 5920, 1, 8622}, {3, 5921, 1, 8622}, {1, 6605, 1, 8623}, {2, 6606, 1, 8623}, {3, 6607, 1, 8623}, {1, 5462, 1, 8624}, {2, 5463, 1, 8624}, {3, 5464, 1, 8624}, {1, 6360, 1, 8625}, {2, 6361, 1, 8625}, {3, 6362, 1, 8625}, {1, 4867, 1, 8626}, {2, 4868, 1, 8626}, {3, 4869, 1, 8626}, {1, 5206, 1, 8627}, {2, 5207, 1, 8627}, {3, 5208, 1, 8627}, {1, 5474, 1, 8628}, {2, 5475, 1, 8628}, {3, 5476, 1, 8628}, {1, 4888, 1, 8629}, {2, 4889, 1, 8629}, {3, 4890, 1, 8629}, {4, 4895, 1, 8630}, {5, 4896, 2, 8630}, {6, 4897, 2, 8630}, {7, 4898, 1, 8630}, {8, 4899, 2, 8630}, {9, 4900, 1, 8630}, {4, 4908, 1, 8631}, {5, 4909, 2, 8631}, {6, 4910, 2, 8631}, {7, 4911, 1, 8631}, {8, 4912, 2, 8631}, {9, 4913, 1, 8631}, {4, 4927, 1, 8632}, {5, 4928, 2, 8632}, {6, 4929, 2, 8632}, {7, 4930, 1, 8632}, {8, 4931, 2, 8632}, {9, 4932, 1, 8632}, {4, 4946, 1, 8633}, {5, 4947, 2, 8633}, {6, 4948, 2, 8633}, {7, 4949, 1, 8633}, {8, 4950, 2, 8633}, {9, 4951, 1, 8633}, {4, 4980, 1, 8634}, {5, 4981, 2, 8634}, {6, 4982, 2, 8634}, {7, 4983, 1, 8634}, {8, 4984, 2, 8634}, {9, 4985, 1, 8634}, {4, 4996, 1, 8635}, {5, 4997, 2, 8635}, {6, 4998, 2, 8635}, {7, 4999, 1, 8635}, {8, 5000, 2, 8635}, {9, 5001, 1, 8635}, {4, 5012, 1, 8636}, {5, 5013, 2, 8636}, {6, 5014, 2, 8636}, {7, 5015, 1, 8636}, {8, 5016, 2, 8636}, {9, 5017, 1, 8636}, {4, 5028, 1, 8637}, {5, 5029, 2, 8637}, {6, 5030, 2, 8637}, {7, 5031, 1, 8637}, {8, 5032, 2, 8637}, {9, 5033, 1, 8637}, {4, 5044, 1, 8638}, {5, 5045, 2, 8638}, {6, 5046, 2, 8638}, {7, 5047, 1, 8638}, {8, 5048, 2, 8638}, {9, 5049, 1, 8638}, {4, 4914, 1, 8639}, {5, 4915, 2, 8639}, {6, 4916, 2, 8639}, {7, 4917, 1, 8639}, {8, 4918, 2, 8639}, {9, 4919, 1, 8639}, {4, 4933, 1, 8640}, {5, 4934, 2, 8640}, {6, 4935, 2, 8640}, {7, 4936, 1, 8640}, {8, 4937, 2, 8640}, {9, 4938, 1, 8640}, {4, 4952, 1, 8641}, {5, 4953, 2, 8641}, {6, 4954, 2, 8641}, {7, 4955, 1, 8641}, {8, 4956, 2, 8641}, {9, 4957, 1, 8641}, {4, 4965, 1, 8642}, {5, 4966, 2, 8642}, {6, 4967, 2, 8642}, {7, 4968, 1, 8642}, {8, 4969, 2, 8642}, {9, 4970, 1, 8642}, {4, 5213, 1, 8643}, {5, 5214, 2, 8643}, {6, 5215, 2, 8643}, {7, 5216, 1, 8643}, {8, 5217, 2, 8643}, {9, 5218, 1, 8643}, {4, 5251, 1, 8644}, {5, 5252, 2, 8644}, {6, 5253, 2, 8644}, {7, 5254, 1, 8644}, {8, 5255, 2, 8644}, {9, 5256, 1, 8644}, {4, 5481, 1, 8645}, {5, 5482, 2, 8645}, {6, 5483, 2, 8645}, {7, 5484, 1, 8645}, {8, 5485, 2, 8645}, {9, 5486, 1, 8645}, {4, 5555, 1, 8646}, {5, 5556, 2, 8646}, {6, 5557, 2, 8646}, {7, 5558, 1, 8646}, {8, 5559, 2, 8646}, {9, 5560, 1, 8646}, {4, 5629, 1, 8647}, {5, 5630, 2, 8647}, {6, 5631, 2, 8647}, {7, 5632, 1, 8647}, {8, 5633, 2, 8647}, {9, 5634, 1, 8647}, {4, 5497, 1, 8648}, {5, 5498, 2, 8648}, {6, 5499, 2, 8648}, {7, 5500, 1, 8648}, {8, 5501, 2, 8648}, {9, 5502, 1, 8648}, {4, 5571, 1, 8649}, {5, 5572, 2, 8649}, {6, 5573, 2, 8649}, {7, 5574, 1, 8649}, {8, 5575, 2, 8649}, {9,

5576, 1, 8649}, {4, 5645, 1, 8650}, {5, 5646, 2, 8650}, {6, 5647, 2, 8650}, {7, 5648, 1, 8650}, {8, 5649, 2, 8650}, {9, 5650, 1, 8650}, {4, 5695, 1, 8651}, {5, 5696, 2, 8651}, {6, 5697, 2, 8651}, {7, 5698, 1, 8651}, {8, 5699, 2, 8651}, {9, 5700, 1, 8651}, {4, 5229, 1, 8652}, {5, 5230, 2, 8652}, {6, 5231, 2, 8652}, {7, 5232, 1, 8652}, {8, 5233, 2, 8652}, {9, 5234, 1, 8652}, {4, 5257, 1, 8653}, {5, 5258, 2, 8653}, {6, 5259, 2, 8653}, {7, 5260, 1, 8653}, {8, 5261, 2, 8653}, {9, 5262, 1, 8653}, {4, 5289, 1, 8654}, {5, 5290, 2, 8654}, {6, 5291, 2, 8654}, {7, 5292, 1, 8654}, {8, 5293, 2, 8654}, {9, 5294, 1, 8654}, {4, 6367, 1, 8655}, {5, 6368, 2, 8655}, {6, 6369, 2, 8655}, {7, 6370, 1, 8655}, {8, 6371, 2, 8655}, {9, 6372, 1, 8655}, {4, 6438, 1, 8656}, {5, 6439, 2, 8656}, {6, 6440, 2, 8656}, {7, 6441, 1, 8656}, {8, 6442, 2, 8656}, {9, 6443, 1, 8656}, {4, 6518, 1, 8657}, {5, 6519, 2, 8657}, {6, 6520, 2, 8657}, {7, 6521, 1, 8657}, {8, 6522, 2, 8657}, {9, 6523, 1, 8657}, {4, 5973, 1, 8658}, {5, 5974, 2, 8658}, {6, 5975, 2, 8658}, {7, 5976, 1, 8658}, {8, 5977, 2, 8658}, {9, 5978, 1, 8658}, {4, 6373, 1, 8659}, {5, 6374, 2, 8659}, {6, 6375, 2, 8659}, {7, 6376, 1, 8659}, {8, 6377, 2, 8659}, {9, 6378, 1, 8659}, {4, 6423, 1, 8660}, {5, 6424, 2, 8660}, {6, 6425, 2, 8660}, {7, 6426, 1, 8660}, {8, 6427, 2, 8660}, {9, 6428, 1, 8660}, {4, 5760, 1, 8661}, {5, 5761, 2, 8661}, {6, 5762, 2, 8661}, {7, 5763, 1, 8661}, {8, 5764, 2, 8661}, {9, 5765, 1, 8661}, {4, 5926, 1, 8662}, {5, 5927, 2, 8662}, {6, 5928, 2, 8662}, {7, 5929, 1, 8662}, {8, 5930, 2, 8662}, {9, 5931, 1, 8662}, {4, 6612, 1, 8663}, {5, 6613, 2, 8663}, {6, 6614, 2, 8663}, {7, 6615, 1, 8663}, {8, 6616, 2, 8663}, {9, 6617, 1, 8663}, {4, 6033, 1, 8664}, {5, 6034, 2, 8664}, {6, 6035, 2, 8664}, {7, 6036, 1, 8664}, {8, 6037, 2, 8664}, {9, 6038, 1, 8664}, {4, 6555, 1, 8665}, {5, 6556, 2, 8665}, {6, 6557, 2, 8665}, {7, 6558, 1, 8665}, {8, 6559, 2, 8665}, {9, 6560, 1, 8665}, {4, 5838, 1, 8666}, {5, 5839, 2, 8666}, {6, 5840, 2, 8666}, {7, 5841, 1, 8666}, {8, 5842, 2, 8666}, {9, 5843, 1, 8666}, {4, 6057, 1, 8667}, {5, 6058, 2, 8667}, {6, 6059, 2, 8667}, {7, 6060, 1, 8667}, {8, 6061, 2, 8667}, {9, 6062, 1, 8667}, {4, 4783, 1, 8668}, {5, 4784, 1, 8668}, {5, 4786, 1, 8668}, {6, 4785, 1, 8668}, {6, 4789, 1, 8668}, {7, 4787, 1, 8668}, {8, 4788, 1, 8668}, {8, 4790, 1, 8668}, {9, 4791, 1, 8668}, {4, 4986, 1, 8669}, {5, 4987, 2, 8669}, {6, 4988, 2, 8669}, {7, 4989, 1, 8669}, {8, 4990, 2, 8669}, {9, 4991, 1, 8669}, {4, 5002, 1, 8670}, {5, 5003, 2, 8670}, {6, 5004, 2, 8670}, {7, 5005, 1, 8670}, {8, 5006, 2, 8670}, {9, 5007, 1, 8670}, {4, 5018, 1, 8671}, {5, 5019, 2, 8671}, {6, 5020, 2, 8671}, {7, 5021, 1, 8671}, {8, 5022, 2, 8671}, {9, 5023, 1, 8671}, {4, 5034, 1, 8672}, {5, 5035, 2, 8672}, {6, 5036, 2, 8672}, {7, 5037, 1, 8672}, {8, 5038, 2, 8672}, {9, 5039, 1, 8672}, {4, 5050, 1, 8673}, {5, 5051, 2, 8673}, {6, 5052, 2, 8673}, {7, 5053, 1, 8673}, {8, 5054, 2, 8673}, {9, 5055, 1, 8673}, {4, 4807, 1, 8674}, {5, 4808, 1, 8674}, {5, 4810, 1, 8674}, {6, 4809, 1, 8674}, {6, 4813, 1, 8674}, {7, 4811, 1, 8674}, {8, 4812, 1, 8674}, {8, 4814, 1, 8674}, {9, 4815, 1, 8674}, {4, 5074, 1, 8675}, {5, 5075, 1, 8675}, {5, 5077, 1, 8675}, {6, 5076, 1, 8675}, {6, 5080, 1, 8675}, {7, 5078, 1, 8675}, {8, 5079, 1, 8675}, {8, 5081, 1, 8675}, {9, 5082, 1, 8675}, {4, 5238, 1, 8676}, {5, 5239, 1, 8676}, {5, 5241, 1, 8676}, {6, 5240, 1, 8676}, {6, 5244, 1, 8676}, {7, 5242, 1, 8676}, {8, 5243, 1, 8676}, {8, 5245, 1, 8676}, {9, 5246, 1, 8676}, {4, 5266, 1, 8677}, {5, 5267, 1, 8677}, {5, 5269, 1, 8677}, {6, 5268, 1, 8677}, {6, 5272, 1, 8677}, {7, 5270, 1, 8677}, {8, 5271, 1, 8677}, {8, 5273, 1, 8677}, {9, 5274, 1, 8677}, {4, 4795, 1, 8678}, {5, 4796, 1, 8678}, {5, 4798, 1, 8678}, {6, 4797, 1, 8678}, {6, 4801, 1, 8678}, {7, 4799, 1, 8678}, {8, 4800, 1, 8678}, {8, 4802, 1, 8678}, {9, 4803, 1, 8678}, {4, 5321, 1, 8679}, {5, 5322, 1, 8679}, {5, 5324, 1, 8679}, {6, 5323, 1, 8679}, {6, 5327, 1, 8679}, {7, 5325, 1, 8679}, {8, 5326, 1, 8679}, {8, 5328, 1, 8679}, {9, 5329, 1, 8679}, {4, 5524, 1, 8680}, {5, 5525, 1, 8680}, {5, 5527, 1, 8680}, {6, 5526, 1, 8680}, {6, 5530, 1, 8680}, {7, 5528, 1, 8680}, {8, 5529, 1, 8680}, {8, 5531, 1, 8680}, {9, 5532, 1, 8680}, {4, 5598, 1, 8681}, {5, 5599, 1, 8681}, {5, 5601, 1, 8681}, {6, 5600, 1, 8681}, {6, 5604, 1, 8681}, {7, 5602, 1, 8681}, {8, 5603, 1, 8681}, {8, 5605, 1, 8681}, {9, 5606, 1, 8681}, {4, 5672, 1, 8682}, {5, 5673, 1, 8682}, {5, 5675, 1, 8682}, {6, 5674, 1, 8682}, {6, 5678, 1, 8682}, {7, 5676, 1, 8682}, {8, 5677, 1, 8682}, {8, 5679, 1, 8682}, {9, 5680, 1, 8682}, {4, 4828, 1, 8683}, {5, 4829, 1, 8683}, {5, 4831, 1, 8683}, {6, 4830, 1, 8683}, {6, 4834, 1, 8683}, {7, 4832, 1, 8683}, {8, 4833, 1, 8683}, {8, 4835, 1, 8683}, {9, 4836, 1, 8683}, {4, 5113, 1, 8684}, {5, 5114, 1, 8684}, {5, 5116, 1, 8684}, {6, 5115, 1, 8684}, {6, 5119, 1, 8684}, {7, 5117, 1, 8684}, {8, 5118, 1, 8684}, {8, 5120, 1, 8684}, {9, 5121, 1, 8684}, {4, 5883, 1, 8685}, {5, 5884, 1, 8685}, {5, 5886, 1, 8685}, {6, 5885, 1, 8685}, {6, 5889, 1, 8685}, {7, 5887, 1, 8685}, {8, 5888, 1, 8685}, {8, 5890, 1, 8685}, {9, 5891, 1, 8685}, {4, 4816, 1, 8686}, {5, 4817, 1, 8686}, {5, 4819, 1, 8686}, {6, 4818, 1, 8686}, {6, 4822, 1, 8686}, {7, 4820, 1, 8686}, {8, 4821, 1, 8686}, {8, 4823, 1, 8686}, {9, 4824, 1, 8686}, {4, 5369, 1, 8687}, {5, 5370, 1, 8687}, {5, 5372, 1, 8687}, {6, 5371, 1, 8687}, {6, 5375, 1, 8687}, {7, 5373, 1, 8687}, {8, 5374, 1, 8687}, {8, 5376, 1, 8687}, {9, 5377, 1, 8687}, {4, 6156, 1, 8688}, {5, 6157, 1, 8688}, {5, 6159, 1, 8688}, {6, 6158, 1, 8688}, {6, 6162, 1, 8688}, {7, 6160, 1, 8688}, {8, 6161, 1, 8688}, {8, 6163, 1, 8688}, {9, 6164, 1, 8688}, {4, 6400, 1, 8689}, {5, 6401, 1, 8689}, {5, 6403, 1, 8689}, {6, 6402, 1, 8689}, {6, 6406, 1, 8689}, {7, 6404, 1, 8689}, {8, 6405, 1, 8689}, {8, 6407, 1, 8689}, {9, 6408, 1, 8689}, {4, 5062, 1, 8690}, {5, 5063, 1, 8690}, {5, 5065, 1, 8690}, {6, 5064, 1, 8690}, {6, 5068, 1, 8690}, {7, 5066, 1, 8690}, {8, 5067, 1, 8690}, {8, 5069, 1, 8690}, {9, 5070, 1, 8690}, {4, 6063, 1, 8691}, {5, 6064, 1, 8691}, {5, 6066, 1, 8691}, {6, 6065, 1, 8691}, {6, 6069, 1, 8691}, {7, 6067, 1, 8691}, {8, 6068, 1, 8691}, {8, 6070, 1, 8691}, {9, 6071, 1, 8691}, {4, 6490, 1,

8692}, {5, 6491, 2, 8692}, {6, 6492, 2, 8692}, {7, 6493, 1, 8692}, {8, 6494, 2, 8692}, {9, 6495, 1, 8692}, {4, 6524, 1, 8693}, {5, 6525, 2, 8693}, {6, 6526, 2, 8693}, {7, 6527, 1, 8693}, {8, 6528, 2, 8693}, {9, 6529, 1, 8693}, {4, 4849, 1, 8694}, {5, 4850, 1, 8694}, {5, 4852, 1, 8694}, {6, 4851, 1, 8694}, {6, 4855, 1, 8694}, {7, 4853, 1, 8694}, {8, 4854, 1, 8694}, {8, 4856, 1, 8694}, {9, 4857, 1, 8694}, {4, 5170, 1, 8695}, {5, 5171, 1, 8695}, {5, 5173, 1, 8695}, {6, 5172, 1, 8695}, {6, 5176, 1, 8695}, {7, 5174, 1, 8695}, {8, 5175, 1, 8695}, {8, 5177, 1, 8695}, {9, 5178, 1, 8695}, {4, 4837, 1, 8696}, {5, 4838, 1, 8696}, {5, 4840, 1, 8696}, {6, 4839, 1, 8696}, {6, 4843, 1, 8696}, {7, 4841, 1, 8696}, {8, 4842, 1, 8696}, {8, 4844, 1, 8696}, {9, 4845, 1, 8696}, {4, 5417, 1, 8697}, {5, 5418, 1, 8697}, {5, 5420, 1, 8697}, {6, 5419, 1, 8697}, {6, 5423, 1, 8697}, {7, 5421, 1, 8697}, {8, 5422, 1, 8697}, {8, 5424, 1, 8697}, {9, 5425, 1, 8697}, {4, 6294, 1, 8698}, {5, 6295, 1, 8698}, {5, 6297, 1, 8698}, {6, 6296, 1, 8698}, {6, 6300, 1, 8698}, {7, 6298, 1, 8698}, {8, 6299, 1, 8698}, {8, 6301, 1, 8698}, {9, 6302, 1, 8698}, {4, 5101, 1, 8699}, {5, 5102, 1, 8699}, {5, 5104, 1, 8699}, {6, 5103, 1, 8699}, {6, 5107, 1, 8699}, {7, 5105, 1, 8699}, {8, 5106, 1, 8699}, {8, 5108, 1, 8699}, {9, 5109, 1, 8699}, {4, 6255, 1, 8700}, {5, 6256, 1, 8700}, {5, 6258, 1, 8700}, {6, 6257, 1, 8700}, {6, 6261, 1, 8700}, {7, 6259, 1, 8700}, {8, 6260, 1, 8700}, {8, 6262, 1, 8700}, {9, 6263, 1, 8700}, {4, 6596, 1, 8701}, {5, 6597, 1, 8701}, {5, 6599, 1, 8701}, {6, 6598, 1, 8701}, {6, 6602, 1, 8701}, {7, 6600, 1, 8701}, {8, 6601, 1, 8701}, {8, 6603, 1, 8701}, {9, 6604, 1, 8701}, {4, 4870, 1, 8702}, {5, 4871, 1, 8702}, {5, 4873, 1, 8702}, {6, 4872, 1, 8702}, {6, 4876, 1, 8702}, {7, 4874, 1, 8702}, {8, 4875, 1, 8702}, {8, 4877, 1, 8702}, {9, 4878, 1, 8702}, {4, 4858, 1, 8703}, {5, 4859, 1, 8703}, {5, 4861, 1, 8703}, {6, 4860, 1, 8703}, {6, 4864, 1, 8703}, {7, 4862, 1, 8703}, {8, 4863, 1, 8703}, {8, 4865, 1, 8703}, {9, 4866, 1, 8703}, {4, 5465, 1, 8704}, {5, 5466, 1, 8704}, {5, 5468, 1, 8704}, {6, 5467, 1, 8704}, {6, 5471, 1, 8704}, {7, 5469, 1, 8704}, {8, 5470, 1, 8704}, {8, 5472, 1, 8704}, {9, 5473, 1, 8704}, {4, 5158, 1, 8705}, {5, 5159, 1, 8705}, {5, 5161, 1, 8705}, {6, 5160, 1, 8705}, {6, 5164, 1, 8705}, {7, 5162, 1, 8705}, {8, 5163, 1, 8705}, {8, 5165, 1, 8705}, {9, 5166, 1, 8705}, {4, 6351, 1, 8706}, {5, 6352, 1, 8706}, {5, 6354, 1, 8706}, {6, 6353, 1, 8706}, {6, 6357, 1, 8706}, {7, 6355, 1, 8706}, {8, 6356, 1, 8706}, {8, 6358, 1, 8706}, {9, 6359, 1, 8706}, {4, 5844, 1, 8707}, {5, 5845, 1, 8707}, {5, 5847, 1, 8707}, {6, 5846, 1, 8707}, {6, 5850, 1, 8707}, {7, 5848, 1, 8707}, {8, 5849, 1, 8707}, {8, 5851, 1, 8707}, {9, 5852, 1, 8707}, {4, 6561, 1, 8708}, {5, 6562, 1, 8708}, {5, 6564, 1, 8708}, {6, 6563, 1, 8708}, {6, 6567, 1, 8708}, {7, 6565, 1, 8708}, {8, 6566, 1, 8708}, {8, 6568, 1, 8708}, {9, 6569, 1, 8708}, {4, 4879, 1, 8709}, {5, 4880, 1, 8709}, {5, 4882, 1, 8709}, {6, 4881, 1, 8709}, {6, 4885, 1, 8709}, {7, 4883, 1, 8709}, {8, 4884, 1, 8709}, {8, 4886, 1, 8709}, {9, 4887, 1, 8709}, {4, 5197, 1, 8710}, {5, 5198, 1, 8710}, {5, 5200, 1, 8710}, {6, 5199, 1, 8710}, {6, 5203, 1, 8710}, {7, 5201, 1, 8710}, {8, 5202, 1, 8710}, {8, 5204, 1, 8710}, {9, 5205, 1, 8710}, {4, 5910, 1, 8711}, {5, 5911, 1, 8711}, {5, 5913, 1, 8711}, {6, 5912, 1, 8711}, {6, 5916, 1, 8711}, {7, 5914, 1, 8711}, {8, 5915, 1, 8711}, {8, 5917, 1, 8711}, {9, 5918, 1, 8711}, {10, 5219, 1, 8712}, {11, 5220, 3, 8712}, {12, 5221, 3, 8712}, {13, 5222, 3, 8712}, {14, 5223, 6, 8712}, {15, 5224, 3, 8712}, {16, 5225, 1, 8712}, {17, 5226, 3, 8712}, {18, 5227, 3, 8712}, {19, 5228, 1, 8712}, {10, 5487, 1, 8713}, {11, 5488, 3, 8713}, {12, 5489, 3, 8713}, {13, 5490, 3, 8713}, {14, 5491, 6, 8713}, {15, 5492, 3, 8713}, {16, 5493, 1, 8713}, {17, 5494, 3, 8713}, {18, 5495, 3, 8713}, {19, 5496, 1, 8713}, {10, 5561, 1, 8714}, {11, 5562, 3, 8714}, {12, 5563, 3, 8714}, {13, 5564, 3, 8714}, {14, 5565, 6, 8714}, {15, 5566, 3, 8714}, {16, 5567, 1, 8714}, {17, 5568, 3, 8714}, {18, 5569, 3, 8714}, {19, 5570, 1, 8714}, {10, 5635, 1, 8715}, {11, 5636, 3, 8715}, {12, 5637, 3, 8715}, {13, 5638, 3, 8715}, {14, 5639, 6, 8715}, {15, 5640, 3, 8715}, {16, 5641, 1, 8715}, {17, 5642, 3, 8715}, {18, 5643, 3, 8715}, {19, 5644, 1, 8715}, {10, 5685, 1, 8716}, {11, 5686, 3, 8716}, {12, 5687, 3, 8716}, {13, 5688, 3, 8716}, {14, 5689, 6, 8716}, {15, 5690, 3, 8716}, {16, 5691, 1, 8716}, {17, 5692, 3, 8716}, {18, 5693, 3, 8716}, {19, 5694, 1, 8716}, {10, 5279, 1, 8717}, {11, 5280, 3, 8717}, {12, 5281, 3, 8717}, {13, 5282, 3, 8717}, {14, 5283, 6, 8717}, {15, 5284, 3, 8717}, {16, 5285, 1, 8717}, {17, 5286, 3, 8717}, {18, 5287, 3, 8717}, {19, 5288, 1, 8717}, {10, 6444, 1, 8718}, {11, 6445, 3, 8718}, {12, 6446, 3, 8718}, {13, 6447, 3, 8718}, {14, 6448, 6, 8718}, {15, 6449, 3, 8718}, {16, 6450, 1, 8718}, {17, 6451, 3, 8718}, {18, 6452, 3, 8718}, {19, 6453, 1, 8718}, {10, 6413, 1, 8719}, {11, 6414, 3, 8719}, {12, 6415, 3, 8719}, {13, 6416, 3, 8719}, {14, 6417, 6, 8719}, {15, 6418, 3, 8719}, {16, 6419, 1, 8719}, {17, 6420, 3, 8719}, {18, 6421, 3, 8719}, {19, 6422, 1, 8719}, {10, 5330, 1, 8720}, {11, 5331, 2, 8720}, {11, 5336, 1, 8720}, {12, 5332, 2, 8720}, {12, 5342, 1, 8720}, {13, 5333, 1, 8720}, {13, 5337, 2, 8720}, {14, 5334, 2, 8720}, {14, 5338, 2, 8720}, {14, 5343, 2, 8720}, {15, 5335, 1, 8720}, {15, 5344, 2, 8720}, {16, 5339, 1, 8720}, {17, 5340, 2, 8720}, {17, 5345, 1, 8720}, {18, 5341, 1, 8720}, {18, 5346, 2, 8720}, {19, 5347, 1, 8720}, {10, 5533, 1, 8721}, {11, 5534, 2, 8721}, {11, 5539, 1, 8721}, {12, 5535, 2, 8721}, {12, 5545, 1, 8721}, {13, 5536, 1, 8721}, {13, 5540, 2, 8721}, {14, 5537, 2, 8721}, {14, 5541, 2, 8721}, {14, 5546, 2, 8721}, {15, 5538, 1, 8721}, {15, 5547, 2, 8721}, {16, 5542, 1, 8721}, {17, 5543, 2, 8721}, {17, 5548, 1, 8721}, {18, 5544, 1, 8721}, {18, 5549, 2, 8721}, {19, 5550, 1, 8721}, {10, 5607, 1, 8722}, {11, 5608, 2, 8722}, {11, 5613, 1, 8722}, {12, 5609, 2, 8722}, {12, 5619, 1, 8722}, {13, 5610, 1, 8722}, {13, 5614, 2, 8722}, {14, 5611, 2, 8722}, {14, 5615, 2, 8722}, {14, 5620, 2, 8722}, {15, 5612, 1, 8722}, {15, 5621, 2, 8722}, {16, 5616, 1, 8722}, {17, 5617,

2, 8722}, {17, 5622, 1, 8722}, {18, 5618, 1, 8722}, {18, 5623, 2, 8722}, {19, 5624, 1, 8722}, {10, 5503, 1, 8723}, {11, 5504, 1, 8723}, {11, 5506, 2, 8723}, {12, 5505, 1, 8723}, {12, 5509, 2, 8723}, {13, 5507, 2, 8723}, {13, 5512, 1, 8723}, {14, 5508, 2, 8723}, {14, 5510, 2, 8723}, {14, 5515, 2, 8723}, {15, 5511, 2, 8723}, {15, 5518, 1, 8723}, {16, 5513, 1, 8723}, {17, 5514, 1, 8723}, {17, 5516, 2, 8723}, {18, 5517, 2, 8723}, {18, 5519, 1, 8723}, {19, 5520, 1, 8723}, {10, 5577, 1, 8724}, {11, 5578, 1, 8724}, {11, 5580, 2, 8724}, {12, 5579, 1, 8724}, {12, 5583, 2, 8724}, {13, 5581, 2, 8724}, {13, 5586, 1, 8724}, {14, 5582, 2, 8724}, {14, 5584, 2, 8724}, {14, 5589, 2, 8724}, {15, 5585, 2, 8724}, {15, 5592, 1, 8724}, {16, 5587, 1, 8724}, {17, 5588, 1, 8724}, {17, 5590, 2, 8724}, {18, 5591, 2, 8724}, {18, 5593, 1, 8724}, {19, 5594, 1, 8724}, {10, 5651, 1, 8725}, {11, 5652, 1, 8725}, {11, 5654, 2, 8725}, {12, 5653, 1, 8725}, {12, 5657, 2, 8725}, {13, 5655, 2, 8725}, {13, 5660, 1, 8725}, {14, 5656, 2, 8725}, {14, 5658, 2, 8725}, {14, 5663, 2, 8725}, {15, 5659, 2, 8725}, {15, 5666, 1, 8725}, {16, 5661, 1, 8725}, {17, 5662, 1, 8725}, {17, 5664, 2, 8725}, {18, 5665, 2, 8725}, {18, 5667, 1, 8725}, {19, 5668, 1, 8725}, {10, 5122, 1, 8726}, {11, 5123, 2, 8726}, {11, 5128, 1, 8726}, {12, 5124, 2, 8726}, {12, 5134, 1, 8726}, {13, 5125, 1, 8726}, {13, 5129, 2, 8726}, {14, 5126, 2, 8726}, {14, 5130, 2, 8726}, {14, 5135, 2, 8726}, {15, 5127, 1, 8726}, {15, 5136, 2, 8726}, {16, 5131, 1, 8726}, {17, 5132, 2, 8726}, {17, 5137, 1, 8726}, {18, 5133, 1, 8726}, {18, 5138, 2, 8726}, {19, 5139, 1, 8726}, {10, 5378, 1, 8727}, {11, 5379, 2, 8727}, {11, 5384, 1, 8727}, {12, 5380, 2, 8727}, {12, 5390, 1, 8727}, {13, 5381, 1, 8727}, {13, 5385, 2, 8727}, {14, 5382, 2, 8727}, {14, 5386, 2, 8727}, {14, 5391, 2, 8727}, {15, 5383, 1, 8727}, {15, 5392, 2, 8727}, {16, 5387, 1, 8727}, {17, 5388, 2, 8727}, {17, 5393, 1, 8727}, {18, 5389, 1, 8727}, {18, 5394, 2, 8727}, {19, 5395, 1, 8727}, {10, 6165, 1, 8728}, {11, 6166, 2, 8728}, {11, 6171, 1, 8728}, {12, 6167, 2, 8728}, {12, 6177, 1, 8728}, {13, 6168, 1, 8728}, {13, 6172, 2, 8728}, {14, 6169, 2, 8728}, {14, 6173, 2, 8728}, {14, 6178, 2, 8728}, {15, 6170, 1, 8728}, {15, 6179, 2, 8728}, {16, 6174, 1, 8728}, {17, 6175, 2, 8728}, {17, 6180, 1, 8728}, {18, 6176, 1, 8728}, {18, 6181, 2, 8728}, {19, 6182, 1, 8728}, {10, 5083, 1, 8729}, {11, 5084, 2, 8729}, {11, 5089, 1, 8729}, {12, 5085, 2, 8729}, {12, 5095, 1, 8729}, {13, 5086, 1, 8729}, {13, 5090, 2, 8729}, {14, 5087, 2, 8729}, {14, 5091, 2, 8729}, {14, 5096, 2, 8729}, {15, 5088, 1, 8729}, {15, 5097, 2, 8729}, {16, 5092, 1, 8729}, {17, 5093, 2, 8729}, {17, 5098, 1, 8729}, {18, 5094, 1, 8729}, {18, 5099, 2, 8729}, {19, 5100, 1, 8729}, {10, 6183, 1, 8730}, {11, 6184, 2, 8730}, {11, 6189, 1, 8730}, {12, 6185, 2, 8730}, {12, 6195, 1, 8730}, {13, 6186, 1, 8730}, {13, 6190, 2, 8730}, {14, 6187, 2, 8730}, {14, 6191, 2, 8730}, {14, 6196, 2, 8730}, {15, 6188, 1, 8730}, {15, 6197, 2, 8730}, {16, 6192, 1, 8730}, {17, 6193, 2, 8730}, {17, 6198, 1, 8730}, {18, 6194, 1, 8730}, {18, 6199, 2, 8730}, {19, 6200, 1, 8730}, {10, 6496, 1, 8731}, {11, 6497, 2, 8731}, {11, 6502, 1, 8731}, {12, 6498, 2, 8731}, {12, 6508, 1, 8731}, {13, 6499, 1, 8731}, {13, 6503, 2, 8731}, {14, 6500, 2, 8731}, {14, 6504, 2, 8731}, {14, 6509, 2, 8731}, {15, 6501, 1, 8731}, {15, 6510, 2, 8731}, {16, 6505, 1, 8731}, {17, 6506, 2, 8731}, {17, 6511, 1, 8731}, {18, 6507, 1, 8731}, {18, 6512, 2, 8731}, {19, 6513, 1, 8731}, {10, 5979, 1, 8732}, {11, 5980, 1, 8732}, {11, 5982, 2, 8732}, {12, 5981, 1, 8732}, {12, 5985, 2, 8732}, {13, 5983, 2, 8732}, {13, 5988, 1, 8732}, {14, 5984, 2, 8732}, {14, 5986, 2, 8732}, {14, 5991, 2, 8732}, {15, 5987, 2, 8732}, {15, 5994, 1, 8732}, {16, 5989, 1, 8732}, {17, 5990, 1, 8732}, {17, 5992, 2, 8732}, {18, 5993, 2, 8732}, {18, 5995, 1, 8732}, {19, 5996, 1, 8732}, {10, 6379, 1, 8733}, {11, 6380, 1, 8733}, {11, 6382, 2, 8733}, {12, 6381, 1, 8733}, {12, 6385, 2, 8733}, {13, 6383, 2, 8733}, {13, 6388, 1, 8733}, {14, 6384, 2, 8733}, {14, 6386, 2, 8733}, {14, 6391, 2, 8733}, {15, 6387, 2, 8733}, {15, 6394, 1, 8733}, {16, 6389, 1, 8733}, {17, 6390, 1, 8733}, {17, 6392, 2, 8733}, {18, 6393, 2, 8733}, {18, 6395, 1, 8733}, {19, 6396, 1, 8733}, {10, 5766, 1, 8734}, {11, 5767, 1, 8734}, {11, 5769, 2, 8734}, {12, 5768, 1, 8734}, {12, 5772, 2, 8734}, {13, 5770, 2, 8734}, {13, 5775, 1, 8734}, {14, 5771, 2, 8734}, {14, 5773, 2, 8734}, {14, 5778, 2, 8734}, {15, 5774, 2, 8734}, {15, 5781, 1, 8734}, {16, 5776, 1, 8734}, {17, 5777, 1, 8734}, {17, 5779, 2, 8734}, {18, 5780, 2, 8734}, {18, 5782, 1, 8734}, {19, 5783, 1, 8734}, {10, 5426, 1, 8735}, {11, 5427, 2, 8735}, {11, 5432, 1, 8735}, {12, 5428, 2, 8735}, {12, 5438, 1, 8735}, {13, 5429, 1, 8735}, {13, 5433, 2, 8735}, {14, 5430, 2, 8735}, {14, 5434, 2, 8735}, {14, 5439, 2, 8735}, {15, 5431, 1, 8735}, {15, 5440, 2, 8735}, {16, 5435, 1, 8735}, {17, 5436, 2, 8735}, {17, 5441, 1, 8735}, {18, 5437, 1, 8735}, {18, 5442, 2, 8735}, {19, 5443, 1, 8735}, {10, 5140, 1, 8736}, {11, 5141, 2, 8736}, {11, 5146, 1, 8736}, {12, 5142, 2, 8736}, {12, 5152, 1, 8736}, {13, 5143, 1, 8736}, {13, 5147, 2, 8736}, {14, 5144, 2, 8736}, {14, 5148, 2, 8736}, {14, 5153, 2, 8736}, {15, 5145, 1, 8736}, {15, 5154, 2, 8736}, {16, 5149, 1, 8736}, {17, 5150, 2, 8736}, {17, 5155, 1, 8736}, {18, 5151, 1, 8736}, {18, 5156, 2, 8736}, {19, 5157, 1, 8736}, {10, 6333, 1, 8737}, {11, 6334, 2, 8737}, {11, 6339, 1, 8737}, {12, 6335, 2, 8737}, {12, 6345, 1, 8737}, {13, 6336, 1, 8737}, {13, 6340, 2, 8737}, {14, 6337, 2, 8737}, {14, 6341, 2, 8737}, {14, 6346, 2, 8737}, {15, 6338, 1, 8737}, {15, 6347, 2, 8737}, {16, 6342, 1, 8737}, {17, 6343, 2, 8737}, {17, 6348, 1, 8737}, {18, 6344, 1, 8737}, {18, 6349, 2, 8737}, {19, 6350, 1, 8737}, {10, 5742, 1, 8738}, {11, 5743, 1, 8738}, {11, 5745, 2, 8738}, {12, 5744, 1, 8738}, {12, 5748, 2, 8738}, {13, 5746, 2, 8738}, {13, 5751, 1, 8738}, {14, 5747, 2, 8738}, {14, 5749, 2, 8738}, {14, 5754, 2, 8738}, {15, 5750, 2, 8738}, {15, 5757, 1, 8738}, {16, 5752, 1, 8738}, {17, 5753, 1, 8738}, {17, 5755, 2, 8738}, {18, 5756, 2, 8738}, {18, 5758, 1, 8738}, {19, 5759, 1, 8738}, {10, 6537, 1,

8739}, {11, 6538, 1, 8739}, {11, 6540, 2, 8739}, {12, 6539, 1, 8739}, {12, 6543, 2, 8739},
{13, 6541, 2, 8739}, {13, 6546, 1, 8739}, {14, 6542, 2, 8739}, {14, 6544, 2, 8739}, {14,
6549, 2, 8739}, {15, 6545, 2, 8739}, {15, 6552, 1, 8739}, {16, 6547, 1, 8739}, {17, 6548,
1, 8739}, {17, 6550, 2, 8739}, {18, 6551, 2, 8739}, {18, 6553, 1, 8739}, {19, 6554, 1,
8739}, {10, 6039, 1, 8740}, {11, 6040, 1, 8740}, {11, 6042, 2, 8740}, {12, 6041, 1, 8740},
{12, 6045, 2, 8740}, {13, 6043, 2, 8740}, {13, 6048, 1, 8740}, {14, 6044, 2, 8740}, {14,
6046, 2, 8740}, {14, 6051, 2, 8740}, {15, 6047, 2, 8740}, {15, 6054, 1, 8740}, {16, 6049,
1, 8740}, {17, 6050, 1, 8740}, {17, 6052, 2, 8740}, {18, 6053, 2, 8740}, {18, 6055, 1,
8740}, {19, 6056, 1, 8740}, {10, 5179, 1, 8741}, {11, 5180, 2, 8741}, {11, 5185, 1, 8741},
{12, 5181, 2, 8741}, {12, 5191, 1, 8741}, {13, 5182, 1, 8741}, {13, 5186, 2, 8741}, {14,
5183, 2, 8741}, {14, 5187, 2, 8741}, {14, 5192, 2, 8741}, {15, 5184, 1, 8741}, {15, 5193,
2, 8741}, {16, 5188, 1, 8741}, {17, 5189, 2, 8741}, {17, 5194, 1, 8741}, {18, 5190, 1,
8741}, {18, 5195, 2, 8741}, {19, 5196, 1, 8741}, {10, 5892, 1, 8742}, {11, 5893, 2, 8742},
{11, 5898, 1, 8742}, {12, 5894, 2, 8742}, {12, 5904, 1, 8742}, {13, 5895, 1, 8742}, {13,
5899, 2, 8742}, {14, 5896, 2, 8742}, {14, 5900, 2, 8742}, {14, 5905, 2, 8742}, {15, 5897,
1, 8742}, {15, 5906, 2, 8742}, {16, 5901, 1, 8742}, {17, 5902, 2, 8742}, {17, 5907, 1,
8742}, {18, 5903, 1, 8742}, {18, 5908, 2, 8742}, {19, 5909, 1, 8742}, {10, 5820, 1, 8743},
{11, 5821, 1, 8743}, {11, 5823, 2, 8743}, {12, 5822, 1, 8743}, {12, 5826, 2, 8743}, {13,
5824, 2, 8743}, {13, 5829, 1, 8743}, {14, 5825, 2, 8743}, {14, 5827, 2, 8743}, {14, 5832,
2, 8743}, {15, 5828, 2, 8743}, {15, 5835, 1, 8743}, {16, 5830, 1, 8743}, {17, 5831, 1,
8743}, {17, 5833, 2, 8743}, {18, 5834, 2, 8743}, {18, 5836, 1, 8743}, {19, 5837, 1, 8743},
{10, 5300, 1, 8744}, {11, 5301, 2, 8744}, {11, 5306, 1, 8744}, {12, 5302, 2, 8744}, {12,
5312, 1, 8744}, {13, 5303, 1, 8744}, {13, 5307, 2, 8744}, {14, 5304, 2, 8744}, {14, 5308,
2, 8744}, {14, 5313, 2, 8744}, {15, 5305, 1, 8744}, {15, 5314, 2, 8744}, {16, 5309, 1,
8744}, {17, 5310, 2, 8744}, {17, 5315, 1, 8744}, {18, 5311, 1, 8744}, {18, 5316, 2, 8744},
{19, 5317, 1, 8744}, {10, 5348, 1, 8745}, {11, 5349, 2, 8745}, {11, 5354, 1, 8745}, {12,
5350, 2, 8745}, {12, 5360, 1, 8745}, {13, 5351, 1, 8745}, {13, 5355, 2, 8745}, {14, 5352,
2, 8745}, {14, 5356, 2, 8745}, {14, 5361, 2, 8745}, {15, 5353, 1, 8745}, {15, 5362, 2,
8745}, {16, 5357, 1, 8745}, {17, 5358, 2, 8745}, {17, 5363, 1, 8745}, {18, 5359, 1, 8745},
{18, 5364, 2, 8745}, {19, 5365, 1, 8745}, {10, 5396, 1, 8746}, {11, 5397, 2, 8746}, {11,
5402, 1, 8746}, {12, 5398, 2, 8746}, {12, 5408, 1, 8746}, {13, 5399, 1, 8746}, {13, 5403,
2, 8746}, {14, 5400, 2, 8746}, {14, 5404, 2, 8746}, {14, 5409, 2, 8746}, {15, 5401, 1,
8746}, {15, 5410, 2, 8746}, {16, 5405, 1, 8746}, {17, 5406, 2, 8746}, {17, 5411, 1, 8746},
{18, 5407, 1, 8746}, {18, 5412, 2, 8746}, {19, 5413, 1, 8746}, {10, 5444, 1, 8747}, {11,
5445, 2, 8747}, {11, 5450, 1, 8747}, {12, 5446, 2, 8747}, {12, 5456, 1, 8747}, {13, 5447,
1, 8747}, {13, 5451, 2, 8747}, {14, 5448, 2, 8747}, {14, 5452, 2, 8747}, {14, 5457, 2,
8747}, {15, 5449, 1, 8747}, {15, 5458, 2, 8747}, {16, 5453, 1, 8747}, {17, 5454, 2, 8747},
{17, 5459, 1, 8747}, {18, 5455, 1, 8747}, {18, 5460, 2, 8747}, {19, 5461, 1, 8747}, {10,
5853, 1, 8748}, {11, 5854, 1, 8748}, {11, 5856, 1, 8748}, {11, 5862, 1, 8748}, {12, 5855,
1, 8748}, {12, 5859, 1, 8748}, {12, 5871, 1, 8748}, {13, 5857, 1, 8748}, {13, 5863, 1,
8748}, {13, 5865, 1, 8748}, {14, 5858, 1, 8748}, {14, 5860, 1, 8748}, {14, 5864, 1, 8748},
{14, 5868, 1, 8748}, {14, 5872, 1, 8748}, {14, 5874, 1, 8748}, {15, 5861, 1, 8748}, {15,
5873, 1, 8748}, {15, 5877, 1, 8748}, {16, 5866, 1, 8748}, {17, 5867, 1, 8748}, {17, 5869,
1, 8748}, {17, 5875, 1, 8748}, {18, 5870, 1, 8748}, {18, 5876, 1, 8748}, {18, 5878, 1,
8748}, {19, 5879, 1, 8748}, {10, 6072, 1, 8749}, {11, 6073, 1, 8749}, {11, 6075, 1, 8749},
{11, 6081, 1, 8749}, {12, 6074, 1, 8749}, {12, 6078, 1, 8749}, {12, 6090, 1, 8749}, {13,
6076, 1, 8749}, {13, 6082, 1, 8749}, {13, 6084, 1, 8749}, {14, 6077, 1, 8749}, {14, 6079,
1, 8749}, {14, 6083, 1, 8749}, {14, 6087, 1, 8749}, {14, 6091, 1, 8749}, {14, 6093, 1,
8749}, {15, 6080, 1, 8749}, {15, 6092, 1, 8749}, {15, 6096, 1, 8749}, {16, 6085, 1, 8749},
{17, 6086, 1, 8749}, {17, 6088, 1, 8749}, {17, 6094, 1, 8749}, {18, 6089, 1, 8749}, {18,
6095, 1, 8749}, {18, 6097, 1, 8749}, {19, 6098, 1, 8749}, {10, 6264, 1, 8750}, {11, 6265,
1, 8750}, {11, 6267, 1, 8750}, {11, 6273, 1, 8750}, {12, 6266, 1, 8750}, {12, 6270, 1,
8750}, {12, 6282, 1, 8750}, {13, 6268, 1, 8750}, {13, 6274, 1, 8750}, {13, 6276, 1, 8750},
{14, 6269, 1, 8750}, {14, 6271, 1, 8750}, {14, 6275, 1, 8750}, {14, 6279, 1, 8750}, {14,
6283, 1, 8750}, {14, 6285, 1, 8750}, {15, 6272, 1, 8750}, {15, 6284, 1, 8750}, {15, 6288,
1, 8750}, {16, 6277, 1, 8750}, {17, 6278, 1, 8750}, {17, 6280, 1, 8750}, {17, 6286, 1,
8750}, {18, 6281, 1, 8750}, {18, 6287, 1, 8750}, {18, 6289, 1, 8750}, {19, 6290, 1, 8750},
{10, 6575, 1, 8751}, {11, 6576, 2, 8751}, {11, 6581, 1, 8751}, {12, 6577, 2, 8751}, {12,
6587, 1, 8751}, {13, 6578, 1, 8751}, {13, 6582, 2, 8751}, {14, 6579, 2, 8751}, {14, 6583,
2, 8751}, {14, 6588, 2, 8751}, {15, 6580, 1, 8751}, {15, 6589, 2, 8751}, {16, 6584, 1,
8751}, {17, 6585, 2, 8751}, {17, 6590, 1, 8751}, {18, 6586, 1, 8751}, {18, 6591, 2, 8751},
{19, 6592, 1, 8751}, {20, 6303, 1, 8752}, {21, 6304, 3, 8752}, {21, 6313, 1, 8752}, {22,
6305, 3, 8752}, {22, 6323, 1, 8752}, {23, 6306, 3, 8752}, {23, 6314, 3, 8752}, {24, 6307,
6, 8752}, {24, 6315, 3, 8752}, {24, 6324, 3, 8752}, {25, 6308, 3, 8752}, {25, 6325, 3,
8752}, {26, 6309, 1, 8752}, {26, 6316, 3, 8752}, {27, 6310, 3, 8752}, {27, 6317, 6, 8752},
{27, 6326, 3, 8752}, {28, 6311, 3, 8752}, {28, 6318, 3, 8752}, {28, 6327, 6, 8752}, {29,
6312, 1, 8752}, {29, 6328, 3, 8752}, {30, 6319, 1, 8752}, {31, 6320, 3, 8752}, {31, 6329,
1, 8752}, {32, 6321, 3, 8752}, {32, 6330, 3, 8752}, {33, 6322, 1, 8752}, {33, 6331, 3,
8752}, {34, 6332, 1, 8752}, {20, 5706, 1, 8753}, {21, 5707, 2, 8753}, {21, 5712, 2, 8753},

{22, 5708, 2, 8753}, {22, 5718, 2, 8753}, {23, 5709, 1, 8753}, {23, 5713, 4, 8753}, {23, 5724, 1, 8753}, {24, 5710, 2, 8753}, {24, 5714, 4, 8753}, {24, 5719, 4, 8753}, {24, 5730, 2, 8753}, {25, 5711, 1, 8753}, {25, 5720, 4, 8753}, {25, 5736, 1, 8753}, {26, 5715, 2, 8753}, {26, 5725, 2, 8753}, {27, 5716, 4, 8753}, {27, 5721, 2, 8753}, {27, 5726, 2, 8753}, {27, 5731, 4, 8753}, {28, 5717, 2, 8753}, {28, 5722, 4, 8753}, {28, 5732, 4, 8753}, {28, 5737, 2, 8753}, {29, 5723, 2, 8753}, {29, 5738, 2, 8753}, {30, 5727, 1, 8753}, {31, 5728, 2, 8753}, {31, 5733, 2, 8753}, {32, 5729, 1, 8753}, {32, 5734, 4, 8753}, {32, 5739, 1, 8753}, {33, 5735, 2, 8753}, {33, 5740, 2, 8753}, {34, 5741, 1, 8753}, {20, 6454, 1, 8754}, {21, 6455, 2, 8754}, {21, 6460, 2, 8754}, {22, 6456, 2, 8754}, {22, 6466, 2, 8754}, {23, 6457, 1, 8754}, {23, 6461, 4, 8754}, {23, 6472, 1, 8754}, {24, 6458, 2, 8754}, {24, 6462, 4, 8754}, {24, 6467, 4, 8754}, {24, 6478, 2, 8754}, {25, 6459, 1, 8754}, {25, 6468, 4, 8754}, {25, 6484, 1, 8754}, {26, 6463, 2, 8754}, {26, 6473, 2, 8754}, {27, 6464, 4, 8754}, {27, 6469, 2, 8754}, {27, 6474, 2, 8754}, {27, 6479, 4, 8754}, {28, 6465, 2, 8754}, {28, 6470, 4, 8754}, {28, 6480, 4, 8754}, {28, 6485, 2, 8754}, {29, 6471, 2, 8754}, {29, 6486, 2, 8754}, {30, 6475, 1, 8754}, {31, 6476, 2, 8754}, {31, 6481, 2, 8754}, {32, 6477, 1, 8754}, {32, 6482, 4, 8754}, {32, 6487, 1, 8754}, {33, 6483, 2, 8754}, {33, 6488, 2, 8754}, {34, 6489, 1, 8754}, {20, 5784, 1, 8755}, {21, 5785, 2, 8755}, {21, 5790, 2, 8755}, {22, 5786, 2, 8755}, {22, 5796, 2, 8755}, {23, 5787, 1, 8755}, {23, 5791, 4, 8755}, {23, 5802, 1, 8755}, {24, 5788, 2, 8755}, {24, 5792, 4, 8755}, {24, 5797, 4, 8755}, {24, 5808, 2, 8755}, {25, 5789, 1, 8755}, {25, 5798, 4, 8755}, {25, 5814, 1, 8755}, {26, 5793, 2, 8755}, {26, 5803, 2, 8755}, {27, 5794, 4, 8755}, {27, 5799, 2, 8755}, {27, 5804, 2, 8755}, {27, 5809, 4, 8755}, {28, 5795, 2, 8755}, {28, 5800, 4, 8755}, {28, 5810, 4, 8755}, {28, 5815, 2, 8755}, {29, 5801, 2, 8755}, {29, 5816, 2, 8755}, {30, 5805, 1, 8755}, {31, 5806, 2, 8755}, {31, 5811, 2, 8755}, {32, 5807, 1, 8755}, {32, 5812, 4, 8755}, {32, 5817, 1, 8755}, {33, 5813, 2, 8755}, {33, 5818, 2, 8755}, {34, 5819, 1, 8755}, {20, 6099, 1, 8756}, {21, 6100, 2, 8756}, {21, 6105, 1, 8756}, {21, 6117, 1, 8756}, {22, 6101, 2, 8756}, {22, 6111, 1, 8756}, {22, 6135, 1, 8756}, {23, 6102, 1, 8756}, {23, 6106, 2, 8756}, {23, 6118, 2, 8756}, {23, 6123, 1, 8756}, {24, 6103, 2, 8756}, {24, 6107, 2, 8756}, {24, 6112, 2, 8756}, {24, 6119, 2, 8756}, {24, 6129, 1, 8756}, {24, 6136, 2, 8756}, {24, 6141, 1, 8756}, {25, 6104, 1, 8756}, {25, 6113, 2, 8756}, {25, 6137, 2, 8756}, {25, 6147, 1, 8756}, {26, 6108, 1, 8756}, {26, 6120, 1, 8756}, {26, 6124, 2, 8756}, {27, 6109, 2, 8756}, {27, 6114, 1, 8756}, {27, 6121, 2, 8756}, {27, 6125, 2, 8756}, {27, 6130, 2, 8756}, {27, 6138, 1, 8756}, {27, 6142, 2, 8756}, {28, 6110, 1, 8756}, {28, 6115, 2, 8756}, {28, 6122, 1, 8756}, {28, 6131, 2, 8756}, {28, 6139, 2, 8756}, {28, 6143, 2, 8756}, {28, 6148, 2, 8756}, {29, 6116, 1, 8756}, {29, 6140, 1, 8756}, {29, 6149, 2, 8756}, {30, 6126, 1, 8756}, {31, 6127, 2, 8756}, {31, 6132, 1, 8756}, {31, 6144, 1, 8756}, {32, 6128, 1, 8756}, {32, 6133, 2, 8756}, {32, 6145, 2, 8756}, {32, 6150, 1, 8756}, {33, 6134, 1, 8756}, {33, 6146, 1, 8756}, {33, 6151, 2, 8756}, {34, 6152, 1, 8756}, {20, 5937, 1, 8757}, {21, 5938, 2, 8757}, {21, 5943, 2, 8757}, {22, 5939, 2, 8757}, {22, 5949, 2, 8757}, {23, 5940, 1, 8757}, {23, 5944, 4, 8757}, {23, 5955, 1, 8757}, {24, 5941, 2, 8757}, {24, 5945, 4, 8757}, {24, 5950, 4, 8757}, {24, 5961, 2, 8757}, {25, 5942, 1, 8757}, {25, 5951, 4, 8757}, {25, 5967, 1, 8757}, {26, 5946, 2, 8757}, {26, 5956, 2, 8757}, {27, 5947, 4, 8757}, {27, 5952, 2, 8757}, {27, 5957, 2, 8757}, {27, 5962, 4, 8757}, {28, 5948, 2, 8757}, {28, 5953, 4, 8757}, {28, 5963, 4, 8757}, {28, 5968, 2, 8757}, {29, 5954, 2, 8757}, {29, 5969, 2, 8757}, {30, 5958, 1, 8757}, {31, 5959, 2, 8757}, {31, 5964, 2, 8757}, {32, 5960, 1, 8757}, {32, 5965, 4, 8757}, {32, 5970, 1, 8757}, {33, 5966, 2, 8757}, {33, 5971, 2, 8757}, {34, 5972, 1, 8757}, {20, 6201, 1, 8758}, {21, 6202, 1, 8758}, {21, 6204, 2, 8758}, {21, 6219, 1, 8758}, {22, 6203, 1, 8758}, {22, 6207, 2, 8758}, {22, 6237, 1, 8758}, {23, 6205, 2, 8758}, {23, 6210, 1, 8758}, {23, 6220, 1, 8758}, {23, 6222, 2, 8758}, {24, 6206, 2, 8758}, {24, 6208, 2, 8758}, {24, 6213, 2, 8758}, {24, 6221, 1, 8758}, {24, 6225, 2, 8758}, {24, 6238, 1, 8758}, {24, 6240, 2, 8758}, {25, 6209, 2, 8758}, {25, 6216, 1, 8758}, {25, 6239, 1, 8758}, {25, 6243, 2, 8758}, {26, 6211, 1, 8758}, {26, 6223, 2, 8758}, {26, 6228, 1, 8758}, {27, 6212, 1, 8758}, {27, 6214, 2, 8758}, {27, 6224, 2, 8758}, {27, 6226, 2, 8758}, {27, 6231, 2, 8758}, {27, 6241, 2, 8758}, {27, 6246, 1, 8758}, {28, 6215, 2, 8758}, {28, 6217, 1, 8758}, {28, 6227, 2, 8758}, {28, 6234, 1, 8758}, {28, 6242, 2, 8758}, {28, 6244, 2, 8758}, {28, 6249, 2, 8758}, {29, 6218, 1, 8758}, {29, 6245, 2, 8758}, {29, 6252, 1, 8758}, {30, 6229, 1, 8758}, {31, 6230, 1, 8758}, {31, 6232, 2, 8758}, {31, 6247, 1, 8758}, {32, 6233, 2, 8758}, {32, 6235, 1, 8758}, {32, 6248, 1, 8758}, {32, 6250, 2, 8758}, {33, 6236, 1, 8758}, {33, 6251, 2, 8758}, {33, 6253, 1, 8758}, {34, 6254, 1, 8758}, {20, 5997, 1, 8759}, {21, 5998, 2, 8759}, {21, 6003, 2, 8759}, {22, 5999, 2, 8759}, {22, 6009, 2, 8759}, {23, 6000, 1, 8759}, {23, 6004, 4, 8759}, {23, 6015, 1, 8759}, {24, 6001, 2, 8759}, {24, 6005, 4, 8759}, {24, 6010, 4, 8759}, {24, 6021, 2, 8759}, {25, 6002, 1, 8759}, {25, 6011, 4, 8759}, {25, 6027, 1, 8759}, {26, 6006, 2, 8759}, {26, 6016, 2, 8759}, {27, 6007, 4, 8759}, {27, 6012, 2, 8759}, {27, 6017, 2, 8759}, {27, 6022, 4, 8759}, {28, 6008, 2, 8759}, {28, 6013, 4, 8759}, {28, 6023, 4, 8759}, {28, 6028, 2, 8759}, {29, 6014, 2, 8759}, {29, 6029, 2, 8759}, {30, 6018, 1, 8759}, {31, 6019, 2, 8759}, {31, 6024, 2, 8759}, {32, 6020, 1, 8759}, {32, 6025, 4, 8759}, {32, 6030, 1, 8759}, {33, 6026, 2, 8759}, {33, 6031, 2, 8759}, {34, 6032, 1, 8759}, {0, 6626, 1, 8760}, {0, 6627, 1, 8761}, {0, 6628, 1, 8762}, {0, 6629, 1, 8763}, {0, 6630, 1, 8764}, {0, 6631, 1, 8765}, {0, 6632, 1, 8766}, {0, 6633, 1, 8767}, {0, 6635, 1,

8768}, {0, 6639, 1, 8769}, {0, 6643, 1, 8770}, {0, 6647, 1, 8771}, {0, 6651, 1, 8772}, {0,
6655, 1, 8773}, {0, 6657, 1, 8774}, {0, 6658, 1, 8775}, {0, 6665, 1, 8776}, {0, 6672, 1,
8777}, {0, 6679, 1, 8778}, {0, 6686, 1, 8779}, {0, 6693, 1, 8780}, {0, 6725, 1, 8781}, {0,
6738, 1, 8782}, {0, 6751, 1, 8783}, {0, 6758, 1, 8784}, {0, 6838, 1, 8785}, {0, 6860, 1,
8786}, {0, 6882, 1, 8787}, {0, 6904, 1, 8788}, {0, 6911, 1, 8789}, {0, 6913, 1, 8790}, {0,
6914, 1, 8791}, {0, 6930, 1, 8792}, {0, 6952, 1, 8793}, {0, 6980, 1, 8794}, {0, 6993, 1,
8795}, {0, 7028, 1, 8796}, {0, 7041, 1, 8797}, {0, 7169, 1, 8798}, {0, 7197, 1, 8799}, {0,
7210, 1, 8800}, {0, 7314, 1, 8801}, {0, 7354, 1, 8802}, {0, 7400, 1, 8803}, {0, 7413, 1,
8804}, {0, 7415, 1, 8805}, {0, 7416, 1, 8806}, {0, 7432, 1, 8807}, {0, 7454, 1, 8808}, {0,
7467, 1, 8809}, {0, 7506, 1, 8810}, {0, 7586, 1, 8811}, {0, 7599, 1, 8812}, {0, 7637, 1,
8813}, {0, 7659, 1, 8814}, {0, 7672, 1, 8815}, {0, 7674, 1, 8816}, {0, 7675, 1, 8817}, {0,
7691, 1, 8818}, {0, 7698, 1, 8819}, {0, 7716, 1, 8820}, {0, 7733, 1, 8821}, {0, 7740, 1,
8822}, {0, 7742, 1, 8823}, {0, 7743, 1, 8824}, {0, 7750, 1, 8825}, {0, 7756, 1, 8826}, {0,
7758, 1, 8827}, {0, 7759, 1, 8828}, {0, 7762, 1, 8829}, {1, 6636, 1, 8830}, {2, 6637, 1,
8830}, {3, 6638, 1, 8830}, {1, 6640, 1, 8831}, {2, 6641, 1, 8831}, {3, 6642, 1, 8831}, {1,
6644, 1, 8832}, {2, 6645, 1, 8832}, {3, 6646, 1, 8832}, {1, 6648, 1, 8833}, {2, 6649, 1,
8833}, {3, 6650, 1, 8833}, {1, 6652, 1, 8834}, {2, 6653, 1, 8834}, {3, 6654, 1, 8834}, {1,
6659, 1, 8835}, {2, 6660, 1, 8835}, {3, 6661, 1, 8835}, {1, 6666, 1, 8836}, {2, 6667, 1,
8836}, {3, 6668, 1, 8836}, {1, 6673, 1, 8837}, {2, 6674, 1, 8837}, {3, 6675, 1, 8837}, {1,
6680, 1, 8838}, {2, 6681, 1, 8838}, {3, 6682, 1, 8838}, {1, 6687, 1, 8839}, {2, 6688, 1,
8839}, {3, 6689, 1, 8839}, {1, 6726, 1, 8840}, {2, 6727, 1, 8840}, {3, 6728, 1, 8840}, {1,
6739, 1, 8841}, {2, 6740, 1, 8841}, {3, 6741, 1, 8841}, {1, 6752, 1, 8842}, {2, 6753, 1,
8842}, {3, 6754, 1, 8842}, {1, 6839, 1, 8843}, {2, 6840, 1, 8843}, {3, 6841, 1, 8843}, {1,
6861, 1, 8844}, {2, 6862, 1, 8844}, {3, 6863, 1, 8844}, {1, 6883, 1, 8845}, {2, 6884, 1,
8845}, {3, 6885, 1, 8845}, {1, 6905, 1, 8846}, {2, 6906, 1, 8846}, {3, 6907, 1, 8846}, {1,
6915, 1, 8847}, {2, 6916, 1, 8847}, {3, 6917, 1, 8847}, {1, 6931, 1, 8848}, {2, 6932, 1,
8848}, {3, 6933, 1, 8848}, {1, 6953, 1, 8849}, {2, 6954, 1, 8849}, {3, 6955, 1, 8849}, {1,
6981, 1, 8850}, {2, 6982, 1, 8850}, {3, 6983, 1, 8850}, {1, 7029, 1, 8851}, {2, 7030, 1,
8851}, {3, 7031, 1, 8851}, {1, 7170, 1, 8852}, {2, 7171, 1, 8852}, {3, 7172, 1, 8852}, {1,
7198, 1, 8853}, {2, 7199, 1, 8853}, {3, 7200, 1, 8853}, {1, 7315, 1, 8854}, {2, 7316, 1,
8854}, {3, 7317, 1, 8854}, {1, 7355, 1, 8855}, {2, 7356, 1, 8855}, {3, 7357, 1, 8855}, {1,
7401, 1, 8856}, {2, 7402, 1, 8856}, {3, 7403, 1, 8856}, {1, 7417, 1, 8857}, {2, 7418, 1,
8857}, {3, 7419, 1, 8857}, {1, 7433, 1, 8858}, {2, 7434, 1, 8858}, {3, 7435, 1, 8858}, {1,
7455, 1, 8859}, {2, 7456, 1, 8859}, {3, 7457, 1, 8859}, {1, 7587, 1, 8860}, {2, 7588, 1,
8860}, {3, 7589, 1, 8860}, {1, 7638, 1, 8861}, {2, 7639, 1, 8861}, {3, 7640, 1, 8861}, {1,
7660, 1, 8862}, {2, 7661, 1, 8862}, {3, 7662, 1, 8862}, {1, 7676, 1, 8863}, {2, 7677, 1,
8863}, {3, 7678, 1, 8863}, {1, 7692, 1, 8864}, {2, 7693, 1, 8864}, {3, 7694, 1, 8864}, {1,
7734, 1, 8865}, {2, 7735, 1, 8865}, {3, 7736, 1, 8865}, {1, 7744, 1, 8866}, {2, 7745, 1,
8866}, {3, 7746, 1, 8866}, {286, 4732, 1, 8867}, {1, 6662, 1, 8868}, {2, 6663, 1, 8868},
{3, 6664, 1, 8868}, {1, 6669, 1, 8869}, {2, 6670, 1, 8869}, {3, 6671, 1, 8869}, {1, 6676,
1, 8870}, {2, 6677, 1, 8870}, {3, 6678, 1, 8870}, {1, 6683, 1, 8871}, {2, 6684, 1, 8871},
{3, 6685, 1, 8871}, {1, 6690, 1, 8872}, {2, 6691, 1, 8872}, {3, 6692, 1, 8872}, {1, 6695,
1, 8873}, {2, 6696, 1, 8873}, {3, 6697, 1, 8873}, {1, 6735, 1, 8874}, {2, 6736, 1, 8874},
{3, 6737, 1, 8874}, {1, 6748, 1, 8875}, {2, 6749, 1, 8875}, {3, 6750, 1, 8875}, {1, 6755,
1, 8876}, {2, 6756, 1, 8876}, {3, 6757, 1, 8876}, {1, 6760, 1, 8877}, {2, 6761, 1, 8877},
{3, 6762, 1, 8877}, {1, 6848, 1, 8878}, {2, 6849, 1, 8878}, {3, 6850, 1, 8878}, {1, 6870,
1, 8879}, {2, 6871, 1, 8879}, {3, 6872, 1, 8879}, {1, 6892, 1, 8880}, {2, 6893, 1, 8880},
{3, 6894, 1, 8880}, {1, 6908, 1, 8881}, {2, 6909, 1, 8881}, {3, 6910, 1, 8881}, {286,
4739, 1, 8882}, {1, 6918, 1, 8883}, {2, 6919, 1, 8883}, {3, 6920, 1, 8883}, {1, 6940, 1,
8884}, {2, 6941, 1, 8884}, {3, 6942, 1, 8884}, {1, 6968, 1, 8885}, {2, 6969, 1, 8885}, {3,
6970, 1, 8885}, {1, 6990, 1, 8886}, {2, 6991, 1, 8886}, {3, 6992, 1, 8886}, {1, 6698, 1,
8887}, {2, 6699, 1, 8887}, {3, 6700, 1, 8887}, {1, 7004, 1, 8888}, {2, 7005, 1, 8888}, {3,
7006, 1, 8888}, {1, 7038, 1, 8889}, {2, 7039, 1, 8889}, {3, 7040, 1, 8889}, {1, 6772, 1,
8890}, {2, 6773, 1, 8890}, {3, 6774, 1, 8890}, {1, 7079, 1, 8891}, {2, 7080, 1, 8891}, {3,
7081, 1, 8891}, {1, 7185, 1, 8892}, {2, 7186, 1, 8892}, {3, 7187, 1, 8892}, {1, 7207, 1,
8893}, {2, 7208, 1, 8893}, {3, 7209, 1, 8893}, {1, 7212, 1, 8894}, {2, 7213, 1, 8894}, {3,
7214, 1, 8894}, {1, 7324, 1, 8895}, {2, 7325, 1, 8895}, {3, 7326, 1, 8895}, {1, 7388, 1,
8896}, {2, 7389, 1, 8896}, {3, 7390, 1, 8896}, {1, 7410, 1, 8897}, {2, 7411, 1, 8897}, {3,
7412, 1, 8897}, {286, 4746, 1, 8898}, {1, 7420, 1, 8899}, {2, 7421, 1, 8899}, {3, 7422, 1,
8899}, {1, 7442, 1, 8900}, {2, 7443, 1, 8900}, {3, 7444, 1, 8900}, {1, 7464, 1, 8901}, {2,
7465, 1, 8901}, {3, 7466, 1, 8901}, {1, 6710, 1, 8902}, {2, 6711, 1, 8902}, {3, 6712, 1,
8902}, {1, 7025, 1, 8903}, {2, 7026, 1, 8903}, {3, 7027, 1, 8903}, {1, 6793, 1, 8904}, {2,
6794, 1, 8904}, {3, 6795, 1, 8904}, {1, 7145, 1, 8905}, {2, 7146, 1, 8905}, {3, 7147, 1,
8905}, {1, 7503, 1, 8906}, {2, 7504, 1, 8906}, {3, 7505, 1, 8906}, {1, 7224, 1, 8907}, {2,
7225, 1, 8907}, {3, 7226, 1, 8907}, {1, 7562, 1, 8908}, {2, 7563, 1, 8908}, {3, 7564, 1,
8908}, {1, 7596, 1, 8909}, {2, 7597, 1, 8909}, {3, 7598, 1, 8909}, {1, 7601, 1, 8910}, {2,
7602, 1, 8910}, {3, 7603, 1, 8910}, {1, 7647, 1, 8911}, {2, 7648, 1, 8911}, {3, 7649, 1,
8911}, {1, 7669, 1, 8912}, {2, 7670, 1, 8912}, {3, 7671, 1, 8912}, {286, 4753, 1, 8913},
{1, 7679, 1, 8914}, {2, 7680, 1, 8914}, {3, 7681, 1, 8914}, {1, 7695, 1, 8915}, {2, 7696,

1, 8915}, {3, 7697, 1, 8915}, {1, 6722, 1, 8916}, {2, 6723, 1, 8916}, {3, 6724, 1, 8916}, {1, 6814, 1, 8917}, {2, 6815, 1, 8917}, {3, 6816, 1, 8917}, {1, 7166, 1, 8918}, {2, 7167, 1, 8918}, {3, 7168, 1, 8918}, {1, 7290, 1, 8919}, {2, 7291, 1, 8919}, {3, 7292, 1, 8919}, {1, 7583, 1, 8920}, {2, 7584, 1, 8920}, {3, 7585, 1, 8920}, {1, 7613, 1, 8921}, {2, 7614, 1, 8921}, {3, 7615, 1, 8921}, {1, 7713, 1, 8922}, {2, 7714, 1, 8922}, {3, 7715, 1, 8922}, {1, 7718, 1, 8923}, {2, 7719, 1, 8923}, {3, 7720, 1, 8923}, {1, 7737, 1, 8924}, {2, 7738, 1, 8924}, {3, 7739, 1, 8924}, {286, 4760, 1, 8925}, {1, 7747, 1, 8926}, {2, 7748, 1, 8926}, {3, 7749, 1, 8926}, {1, 6835, 1, 8927}, {2, 6836, 1, 8927}, {3, 6837, 1, 8927}, {1, 7311, 1, 8928}, {2, 7312, 1, 8928}, {3, 7313, 1, 8928}, {1, 7634, 1, 8929}, {2, 7635, 1, 8929}, {3, 7636, 1, 8929}, {1, 7730, 1, 8930}, {2, 7731, 1, 8930}, {3, 7732, 1, 8930}, {1, 7753, 1, 8931}, {2, 7754, 1, 8931}, {3, 7755, 1, 8931}, {286, 4767, 1, 8932}, {4, 6729, 1, 8933}, {5, 6730, 2, 8933}, {6, 6731, 2, 8933}, {7, 6732, 1, 8933}, {8, 6733, 2, 8933}, {9, 6734, 1, 8933}, {4, 6742, 1, 8934}, {5, 6743, 2, 8934}, {6, 6744, 2, 8934}, {7, 6745, 1, 8934}, {8, 6746, 2, 8934}, {9, 6747, 1, 8934}, {4, 6842, 1, 8935}, {5, 6843, 2, 8935}, {6, 6844, 2, 8935}, {7, 6845, 1, 8935}, {8, 6846, 2, 8935}, {9, 6847, 1, 8935}, {4, 6864, 1, 8936}, {5, 6865, 2, 8936}, {6, 6866, 2, 8936}, {7, 6867, 1, 8936}, {8, 6868, 2, 8936}, {9, 6869, 1, 8936}, {4, 6886, 1, 8937}, {5, 6887, 2, 8937}, {6, 6888, 2, 8937}, {7, 6889, 1, 8937}, {8, 6890, 2, 8937}, {9, 6891, 1, 8937}, {4, 6934, 1, 8938}, {5, 6935, 2, 8938}, {6, 6936, 2, 8938}, {7, 6937, 1, 8938}, {8, 6938, 2, 8938}, {9, 6939, 1, 8938}, {4, 6956, 1, 8939}, {5, 6957, 2, 8939}, {6, 6958, 2, 8939}, {7, 6959, 1, 8939}, {8, 6960, 2, 8939}, {9, 6961, 1, 8939}, {4, 7173, 1, 8940}, {5, 7174, 2, 8940}, {6, 7175, 2, 8940}, {7, 7176, 1, 8940}, {8, 7177, 2, 8940}, {9, 7178, 1, 8940}, {4, 7318, 1, 8941}, {5, 7319, 2, 8941}, {6, 7320, 2, 8941}, {7, 7321, 1, 8941}, {8, 7322, 2, 8941}, {9, 7323, 1, 8941}, {4, 7358, 1, 8942}, {5, 7359, 2, 8942}, {6, 7360, 2, 8942}, {7, 7361, 1, 8942}, {8, 7362, 2, 8942}, {9, 7363, 1, 8942}, {4, 7436, 1, 8943}, {5, 7437, 2, 8943}, {6, 7438, 2, 8943}, {7, 7439, 1, 8943}, {8, 7440, 2, 8943}, {9, 7441, 1, 8943}, {4, 7641, 1, 8944}, {5, 7642, 2, 8944}, {6, 7643, 2, 8944}, {7, 7644, 1, 8944}, {8, 7645, 2, 8944}, {9, 7646, 1, 8944}, {4, 6763, 1, 8945}, {5, 6764, 1, 8945}, {5, 6766, 1, 8945}, {6, 6765, 1, 8945}, {6, 6769, 1, 8945}, {7, 6767, 1, 8945}, {8, 6768, 1, 8945}, {8, 6770, 1, 8945}, {9, 6771, 1, 8945}, {4, 6851, 1, 8946}, {5, 6852, 1, 8946}, {5, 6854, 1, 8946}, {6, 6853, 1, 8946}, {6, 6857, 1, 8946}, {7, 6855, 1, 8946}, {8, 6856, 1, 8946}, {8, 6858, 1, 8946}, {9, 6859, 1, 8946}, {4, 6873, 1, 8947}, {5, 6874, 1, 8947}, {5, 6876, 1, 8947}, {6, 6875, 1, 8947}, {6, 6879, 1, 8947}, {7, 6877, 1, 8947}, {8, 6878, 1, 8947}, {8, 6880, 1, 8947}, {9, 6881, 1, 8947}, {4, 6895, 1, 8948}, {5, 6896, 1, 8948}, {5, 6898, 1, 8948}, {6, 6897, 1, 8948}, {6, 6901, 1, 8948}, {7, 6899, 1, 8948}, {8, 6900, 1, 8948}, {8, 6902, 1, 8948}, {9, 6903, 1, 8948}, {287, 4792, 1, 8949}, {288, 4793, 1, 8949}, {289, 4794, 1, 8949}, {4, 6921, 1, 8950}, {5, 6922, 1, 8950}, {5, 6924, 1, 8950}, {6, 6923, 1, 8950}, {6, 6927, 1, 8950}, {7, 6925, 1, 8950}, {8, 6926, 1, 8950}, {8, 6928, 1, 8950}, {9, 6929, 1, 8950}, {4, 6943, 1, 8951}, {5, 6944, 1, 8951}, {5, 6946, 1, 8951}, {6, 6945, 1, 8951}, {6, 6949, 1, 8951}, {7, 6947, 1, 8951}, {8, 6948, 1, 8951}, {8, 6950, 1, 8951}, {9, 6951, 1, 8951}, {4, 6971, 1, 8952}, {5, 6972, 1, 8952}, {5, 6974, 1, 8952}, {6, 6973, 1, 8952}, {6, 6977, 1, 8952}, {7, 6975, 1, 8952}, {8, 6976, 1, 8952}, {8, 6978, 1, 8952}, {9, 6979, 1, 8952}, {4, 6701, 1, 8953}, {5, 6702, 1, 8953}, {5, 6704, 1, 8953}, {6, 6703, 1, 8953}, {6, 6707, 1, 8953}, {7, 6705, 1, 8953}, {8, 6706, 1, 8953}, {8, 6708, 1, 8953}, {9, 6709, 1, 8953}, {4, 7007, 1, 8954}, {5, 7008, 1, 8954}, {5, 7010, 1, 8954}, {6, 7009, 1, 8954}, {6, 7013, 1, 8954}, {7, 7011, 1, 8954}, {8, 7012, 1, 8954}, {8, 7014, 1, 8954}, {9, 7015, 1, 8954}, {4, 6775, 1, 8955}, {5, 6776, 1, 8955}, {5, 6778, 1, 8955}, {6, 6777, 1, 8955}, {6, 6781, 1, 8955}, {7, 6779, 1, 8955}, {8, 6780, 1, 8955}, {8, 6782, 1, 8955}, {9, 6783, 1, 8955}, {4, 7082, 1, 8956}, {5, 7083, 1, 8956}, {5, 7085, 1, 8956}, {6, 7084, 1, 8956}, {6, 7088, 1, 8956}, {7, 7086, 1, 8956}, {8, 7087, 1, 8956}, {8, 7089, 1, 8956}, {9, 7090, 1, 8956}, {4, 7188, 1, 8957}, {5, 7189, 1, 8957}, {5, 7191, 1, 8957}, {6, 7190, 1, 8957}, {6, 7194, 1, 8957}, {7, 7192, 1, 8957}, {8, 7193, 1, 8957}, {8, 7195, 1, 8957}, {9, 7196, 1, 8957}, {4, 7215, 1, 8958}, {5, 7216, 1, 8958}, {5, 7218, 1, 8958}, {6, 7217, 1, 8958}, {6, 7221, 1, 8958}, {7, 7219, 1, 8958}, {8, 7220, 1, 8958}, {8, 7222, 1, 8958}, {9, 7223, 1, 8958}, {4, 7327, 1, 8959}, {5, 7328, 1, 8959}, {5, 7330, 1, 8959}, {6, 7329, 1, 8959}, {6, 7333, 1, 8959}, {7, 7331, 1, 8959}, {8, 7332, 1, 8959}, {8, 7334, 1, 8959}, {9, 7335, 1, 8959}, {4, 7391, 1, 8960}, {5, 7392, 1, 8960}, {5, 7394, 1, 8960}, {6, 7393, 1, 8960}, {6, 7397, 1, 8960}, {7, 7395, 1, 8960}, {8, 7396, 1, 8960}, {8, 7398, 1, 8960}, {9, 7399, 1, 8960}, {287, 4804, 1, 8961}, {288, 4805, 1, 8961}, {289, 4806, 1, 8961}, {4, 7423, 1, 8962}, {5, 7424, 1, 8962}, {5, 7426, 1, 8962}, {6, 7425, 1, 8962}, {6, 7429, 1, 8962}, {7, 7427, 1, 8962}, {8, 7428, 1, 8962}, {8, 7430, 1, 8962}, {9, 7431, 1, 8962}, {4, 7445, 1, 8963}, {5, 7446, 1, 8963}, {5, 7448, 1, 8963}, {6, 7447, 1, 8963}, {6, 7451, 1, 8963}, {7, 7449, 1, 8963}, {8, 7450, 1, 8963}, {8, 7452, 1, 8963}, {9, 7453, 1, 8963}, {4, 6713, 1, 8964}, {5, 6714, 1, 8964}, {5, 6716, 1, 8964}, {6, 6715, 1, 8964}, {6, 6719, 1, 8964}, {7, 6717, 1, 8964}, {8, 6718, 1, 8964}, {8, 6720, 1, 8964}, {9, 6721, 1, 8964}, {4, 6796, 1, 8965}, {5, 6797, 1, 8965}, {5, 6799, 1, 8965}, {6, 6798, 1, 8965}, {6, 6802, 1, 8965}, {7, 6800, 1, 8965}, {8, 6801, 1, 8965}, {8, 6803, 1, 8965}, {9, 6804, 1, 8965}, {4, 7148, 1, 8966}, {5, 7149, 1, 8966}, {5, 7151, 1, 8966}, {6, 7150, 1, 8966}, {6, 7154, 1, 8966}, {7, 7152, 1, 8966}, {8, 7153, 1, 8966}, {8, 7155, 1, 8966}, {9, 7156, 1, 8966}, {4, 7227, 1, 8967}, {5, 7228, 1, 8967}, {5, 7230, 1, 8967},

{6, 7229, 1, 8967}, {6, 7233, 1, 8967}, {7, 7231, 1, 8967}, {8, 7232, 1, 8967}, {8, 7234, 1, 8967}, {9, 7235, 1, 8967}, {4, 7565, 1, 8968}, {5, 7566, 1, 8968}, {5, 7568, 1, 8968}, {6, 7567, 1, 8968}, {6, 7571, 1, 8968}, {7, 7569, 1, 8968}, {8, 7570, 1, 8968}, {8, 7572, 1, 8968}, {9, 7573, 1, 8968}, {4, 7604, 1, 8969}, {5, 7605, 1, 8969}, {5, 7607, 1, 8969}, {6, 7606, 1, 8969}, {6, 7610, 1, 8969}, {7, 7608, 1, 8969}, {8, 7609, 1, 8969}, {8, 7611, 1, 8969}, {9, 7612, 1, 8969}, {4, 7650, 1, 8970}, {5, 7651, 1, 8970}, {5, 7653, 1, 8970}, {6, 7652, 1, 8970}, {6, 7656, 1, 8970}, {7, 7654, 1, 8970}, {8, 7655, 1, 8970}, {8, 7657, 1, 8970}, {9, 7658, 1, 8970}, {287, 4825, 1, 8971}, {288, 4826, 1, 8971}, {289, 4827, 1, 8971}, {4, 7682, 1, 8972}, {5, 7683, 1, 8972}, {5, 7685, 1, 8972}, {6, 7684, 1, 8972}, {6, 7688, 1, 8972}, {7, 7686, 1, 8972}, {8, 7687, 1, 8972}, {8, 7689, 1, 8972}, {9, 7690, 1, 8972}, {4, 6817, 1, 8973}, {5, 6818, 1, 8973}, {5, 6820, 1, 8973}, {6, 6819, 1, 8973}, {6, 6823, 1, 8973}, {7, 6821, 1, 8973}, {8, 6822, 1, 8973}, {8, 6824, 1, 8973}, {9, 6825, 1, 8973}, {4, 7293, 1, 8974}, {5, 7294, 1, 8974}, {5, 7296, 1, 8974}, {6, 7295, 1, 8974}, {6, 7299, 1, 8974}, {7, 7297, 1, 8974}, {8, 7298, 1, 8974}, {8, 7300, 1, 8974}, {9, 7301, 1, 8974}, {4, 7616, 1, 8975}, {5, 7617, 1, 8975}, {5, 7619, 1, 8975}, {6, 7618, 1, 8975}, {6, 7622, 1, 8975}, {7, 7620, 1, 8975}, {8, 7621, 1, 8975}, {8, 7623, 1, 8975}, {9, 7624, 1, 8975}, {4, 7721, 1, 8976}, {5, 7722, 1, 8976}, {5, 7724, 1, 8976}, {6, 7723, 1, 8976}, {6, 7727, 1, 8976}, {7, 7725, 1, 8976}, {8, 7726, 1, 8976}, {8, 7728, 1, 8976}, {9, 7729, 1, 8976}, {287, 4846, 1, 8977}, {288, 4847, 1, 8977}, {289, 4848, 1, 8977}, {286, 4891, 1, 8978}, {287, 4892, 1, 8979}, {288, 4893, 1, 8979}, {289, 4894, 1, 8979}, {4, 6962, 1, 8980}, {5, 6963, 2, 8980}, {6, 6964, 2, 8980}, {7, 6965, 1, 8980}, {8, 6966, 2, 8980}, {9, 6967, 1, 8980}, {4, 6984, 1, 8981}, {5, 6985, 2, 8981}, {6, 6986, 2, 8981}, {7, 6987, 1, 8981}, {8, 6988, 2, 8981}, {9, 6989, 1, 8981}, {4, 7032, 1, 8982}, {5, 7033, 2, 8982}, {6, 7034, 2, 8982}, {7, 7035, 1, 8982}, {8, 7036, 2, 8982}, {9, 7037, 1, 8982}, {4, 7179, 1, 8983}, {5, 7180, 2, 8983}, {6, 7181, 2, 8983}, {7, 7182, 1, 8983}, {8, 7183, 2, 8983}, {9, 7184, 1, 8983}, {4, 7201, 1, 8984}, {5, 7202, 2, 8984}, {6, 7203, 2, 8984}, {7, 7204, 1, 8984}, {8, 7205, 2, 8984}, {9, 7206, 1, 8984}, {287, 4901, 1, 8985}, {288, 4902, 1, 8985}, {289, 4903, 1, 8985}, {4, 7364, 1, 8986}, {5, 7365, 2, 8986}, {6, 7366, 2, 8986}, {7, 7367, 1, 8986}, {8, 7368, 2, 8986}, {9, 7369, 1, 8986}, {4, 7404, 1, 8987}, {5, 7405, 2, 8987}, {6, 7406, 2, 8987}, {7, 7407, 1, 8987}, {8, 7408, 2, 8987}, {9, 7409, 1, 8987}, {286, 4904, 1, 8988}, {287, 4905, 1, 8989}, {288, 4906, 1, 8989}, {289, 4907, 1, 8989}, {4, 7458, 1, 8990}, {5, 7459, 2, 8990}, {6, 7460, 2, 8990}, {7, 7461, 1, 8990}, {8, 7462, 2, 8990}, {9, 7463, 1, 8990}, {4, 7488, 1, 8991}, {5, 7489, 2, 8991}, {6, 7490, 2, 8991}, {7, 7491, 1, 8991}, {8, 7492, 2, 8991}, {9, 7493, 1, 8991}, {4, 7508, 1, 8992}, {5, 7509, 2, 8992}, {6, 7510, 2, 8992}, {7, 7511, 1, 8992}, {8, 7512, 2, 8992}, {9, 7513, 1, 8992}, {4, 7590, 1, 8993}, {5, 7591, 2, 8993}, {6, 7592, 2, 8993}, {7, 7593, 1, 8993}, {8, 7594, 2, 8993}, {9, 7595, 1, 8993}, {287, 4920, 1, 8994}, {288, 4921, 1, 8994}, {289, 4922, 1, 8994}, {4, 7663, 1, 8995}, {5, 7664, 2, 8995}, {6, 7665, 2, 8995}, {7, 7666, 1, 8995}, {8, 7667, 2, 8995}, {9, 7668, 1, 8995}, {286, 4923, 1, 8996}, {287, 4924, 1, 8997}, {288, 4925, 1, 8997}, {289, 4926, 1, 8997}, {4, 7532, 1, 8998}, {5, 7533, 2, 8998}, {6, 7534, 2, 8998}, {7, 7535, 1, 8998}, {8, 7536, 2, 8998}, {9, 7537, 1, 8998}, {4, 7701, 1, 8999}, {5, 7702, 2, 8999}, {6, 7703, 2, 8999}, {7, 7704, 1, 8999}, {8, 7705, 2, 8999}, {9, 7706, 1, 8999}, {287, 4939, 1, 9000}, {288, 4940, 1, 9000}, {289, 4941, 1, 9000}, {286, 4942, 1, 9001}, {286, 4976, 1, 9002}, {287, 4977, 1, 9003}, {288, 4978, 1, 9003}, {289, 4979, 1, 9003}, {4, 6784, 1, 9004}, {5, 6785, 1, 9004}, {5, 6787, 1, 9004}, {6, 6786, 1, 9004}, {6, 6790, 1, 9004}, {7, 6788, 1, 9004}, {8, 6789, 1, 9004}, {8, 6791, 1, 9004}, {9, 6792, 1, 9004}, {4, 6805, 1, 9005}, {5, 6806, 1, 9005}, {5, 6808, 1, 9005}, {6, 6807, 1, 9005}, {6, 6811, 1, 9005}, {7, 6809, 1, 9005}, {8, 6810, 1, 9005}, {8, 6812, 1, 9005}, {9, 6813, 1, 9005}, {4, 6826, 1, 9006}, {5, 6827, 1, 9006}, {5, 6829, 1, 9006}, {6, 6828, 1, 9006}, {6, 6832, 1, 9006}, {7, 6830, 1, 9006}, {8, 6831, 1, 9006}, {8, 6833, 1, 9006}, {9, 6834, 1, 9006}, {4, 6995, 1, 9007}, {5, 6996, 1, 9007}, {5, 6998, 1, 9007}, {6, 6997, 1, 9007}, {6, 7001, 1, 9007}, {7, 6999, 1, 9007}, {8, 7000, 1, 9007}, {8, 7002, 1, 9007}, {9, 7003, 1, 9007}, {4, 7016, 1, 9008}, {5, 7017, 1, 9008}, {5, 7019, 1, 9008}, {6, 7018, 1, 9008}, {6, 7022, 1, 9008}, {7, 7020, 1, 9008}, {8, 7021, 1, 9008}, {8, 7023, 1, 9008}, {9, 7024, 1, 9008}, {4, 7043, 1, 9009}, {5, 7044, 1, 9009}, {5, 7046, 1, 9009}, {6, 7045, 1, 9009}, {6, 7049, 1, 9009}, {7, 7047, 1, 9009}, {8, 7048, 1, 9009}, {8, 7050, 1, 9009}, {9, 7051, 1, 9009}, {4, 7109, 1, 9010}, {5, 7110, 1, 9010}, {5, 7112, 1, 9010}, {6, 7111, 1, 9010}, {6, 7115, 1, 9010}, {7, 7113, 1, 9010}, {8, 7114, 1, 9010}, {8, 7116, 1, 9010}, {9, 7117, 1, 9010}, {4, 7157, 1, 9011}, {5, 7158, 1, 9011}, {5, 7160, 1, 9011}, {6, 7159, 1, 9011}, {6, 7163, 1, 9011}, {7, 7161, 1, 9011}, {8, 7162, 1, 9011}, {8, 7164, 1, 9011}, {9, 7165, 1, 9011}, {286, 4992, 1, 9012}, {287, 4993, 1, 9013}, {288, 4994, 1, 9013}, {289, 4995, 1, 9013}, {4, 7254, 1, 9014}, {5, 7255, 1, 9014}, {5, 7257, 1, 9014}, {6, 7256, 1, 9014}, {6, 7260, 1, 9014}, {7, 7258, 1, 9014}, {8, 7259, 1, 9014}, {8, 7261, 1, 9014}, {9, 7262, 1, 9014}, {4, 7302, 1, 9015}, {5, 7303, 1, 9015}, {5, 7305, 1, 9015}, {6, 7304, 1, 9015}, {6, 7308, 1, 9015}, {7, 7306, 1, 9015}, {8, 7307, 1, 9015}, {8, 7309, 1, 9015}, {9, 7310, 1, 9015}, {4, 7494, 1, 9016}, {5, 7495, 1, 9016}, {5, 7497, 1, 9016}, {6, 7496, 1, 9016}, {6, 7500, 1, 9016}, {7, 7498, 1, 9016}, {8, 7499, 1, 9016}, {8, 7501, 1, 9016}, {9, 7502, 1, 9016}, {4, 7538, 1, 9017}, {5, 7539, 2, 9017}, {6, 7540, 2, 9017}, {7, 7541, 1, 9017}, {8, 7542, 2, 9017}, {9, 7543, 1, 9017}, {4, 7574, 1, 9018}, {5, 7575, 1, 9018}, {5, 7577, 1,

9018}, {6, 7576, 1, 9018}, {6, 7580, 1, 9018}, {7, 7578, 1, 9018}, {8, 7579, 1, 9018}, {8,
7581, 1, 9018}, {9, 7582, 1, 9018}, {286, 5008, 1, 9019}, {287, 5009, 1, 9020}, {288,
5010, 1, 9020}, {289, 5011, 1, 9020}, {4, 7625, 1, 9021}, {5, 7626, 1, 9021}, {5, 7628, 1,
9021}, {6, 7627, 1, 9021}, {6, 7631, 1, 9021}, {7, 7629, 1, 9021}, {8, 7630, 1, 9021}, {8,
7632, 1, 9021}, {9, 7633, 1, 9021}, {4, 7707, 1, 9022}, {5, 7708, 2, 9022}, {6, 7709, 2,
9022}, {7, 7710, 1, 9022}, {8, 7711, 2, 9022}, {9, 7712, 1, 9022}, {286, 5024, 1, 9023},
{287, 5025, 1, 9024}, {288, 5026, 1, 9024}, {289, 5027, 1, 9024}, {286, 5040, 1, 9025},
{10, 7091, 1, 9026}, {11, 7092, 2, 9026}, {11, 7097, 1, 9026}, {12, 7093, 2, 9026}, {12,
7103, 1, 9026}, {13, 7094, 1, 9026}, {13, 7098, 2, 9026}, {14, 7095, 2, 9026}, {14, 7099,
2, 9026}, {14, 7104, 2, 9026}, {15, 7096, 1, 9026}, {15, 7105, 2, 9026}, {16, 7100, 1,
9026}, {17, 7101, 2, 9026}, {17, 7106, 1, 9026}, {18, 7102, 1, 9026}, {18, 7107, 2, 9026},
{19, 7108, 1, 9026}, {10, 7336, 1, 9027}, {11, 7337, 2, 9027}, {11, 7342, 1, 9027}, {12,
7338, 2, 9027}, {12, 7348, 1, 9027}, {13, 7339, 1, 9027}, {13, 7343, 2, 9027}, {14, 7340,
2, 9027}, {14, 7344, 2, 9027}, {14, 7349, 2, 9027}, {15, 7341, 1, 9027}, {15, 7350, 2,
9027}, {16, 7345, 1, 9027}, {17, 7346, 2, 9027}, {17, 7351, 1, 9027}, {18, 7347, 1, 9027},
{18, 7352, 2, 9027}, {19, 7353, 1, 9027}, {10, 7236, 1, 9028}, {11, 7237, 2, 9028}, {11,
7242, 1, 9028}, {12, 7238, 2, 9028}, {12, 7248, 1, 9028}, {13, 7239, 1, 9028}, {13, 7243,
2, 9028}, {14, 7240, 2, 9028}, {14, 7244, 2, 9028}, {14, 7249, 2, 9028}, {15, 7241, 1,
9028}, {15, 7250, 2, 9028}, {16, 7245, 1, 9028}, {17, 7246, 2, 9028}, {17, 7251, 1, 9028},
{18, 7247, 1, 9028}, {18, 7252, 2, 9028}, {19, 7253, 1, 9028}, {290, 5062, 1, 9029}, {291,
5063, 1, 9029}, {291, 5065, 1, 9029}, {292, 5064, 1, 9029}, {292, 5068, 1, 9029}, {293,
5066, 1, 9029}, {294, 5067, 1, 9029}, {294, 5069, 1, 9029}, {295, 5070, 1, 9029}, {10,
7370, 1, 9030}, {11, 7371, 1, 9030}, {11, 7373, 2, 9030}, {12, 7372, 1, 9030}, {12, 7376,
2, 9030}, {13, 7374, 2, 9030}, {13, 7379, 1, 9030}, {14, 7375, 2, 9030}, {14, 7377, 2,
9030}, {14, 7382, 2, 9030}, {15, 7378, 2, 9030}, {15, 7385, 1, 9030}, {16, 7380, 1, 9030},
{17, 7381, 1, 9030}, {17, 7383, 2, 9030}, {18, 7384, 2, 9030}, {18, 7386, 1, 9030}, {19,
7387, 1, 9030}, {287, 5071, 1, 9031}, {288, 5072, 1, 9031}, {289, 5073, 1, 9031}, {290,
5074, 1, 9032}, {291, 5075, 1, 9032}, {291, 5077, 1, 9032}, {292, 5076, 1, 9032}, {292,
5080, 1, 9032}, {293, 5078, 1, 9032}, {294, 5079, 1, 9032}, {294, 5081, 1, 9032}, {295,
5082, 1, 9032}, {10, 7514, 1, 9033}, {11, 7515, 1, 9033}, {11, 7517, 2, 9033}, {12, 7516,
1, 9033}, {12, 7520, 2, 9033}, {13, 7518, 2, 9033}, {13, 7523, 1, 9033}, {14, 7519, 2,
9033}, {14, 7521, 2, 9033}, {14, 7526, 2, 9033}, {15, 7522, 2, 9033}, {15, 7529, 1, 9033},
{16, 7524, 1, 9033}, {17, 7525, 1, 9033}, {17, 7527, 2, 9033}, {18, 7528, 2, 9033}, {18,
7530, 1, 9033}, {19, 7531, 1, 9033}, {290, 5101, 1, 9034}, {291, 5102, 1, 9034}, {291,
5104, 1, 9034}, {292, 5103, 1, 9034}, {292, 5107, 1, 9034}, {293, 5105, 1, 9034}, {294,
5106, 1, 9034}, {294, 5108, 1, 9034}, {295, 5109, 1, 9034}, {287, 5110, 1, 9035}, {288,
5111, 1, 9035}, {289, 5112, 1, 9035}, {286, 5209, 1, 9036}, {287, 5210, 1, 9037}, {288,
5211, 1, 9037}, {289, 5212, 1, 9037}, {290, 5229, 1, 9038}, {291, 5230, 2, 9038}, {292,
5231, 2, 9038}, {293, 5232, 1, 9038}, {294, 5233, 2, 9038}, {295, 5234, 1, 9038}, {287,
5235, 1, 9039}, {288, 5236, 1, 9039}, {289, 5237, 1, 9039}, {286, 5247, 1, 9040}, {10,
7052, 1, 9041}, {11, 7053, 1, 9041}, {11, 7055, 1, 9041}, {11, 7061, 1, 9041}, {12, 7054,
1, 9041}, {12, 7058, 1, 9041}, {12, 7070, 1, 9041}, {13, 7056, 1, 9041}, {13, 7062, 1,
9041}, {13, 7064, 1, 9041}, {14, 7057, 1, 9041}, {14, 7059, 1, 9041}, {14, 7063, 1, 9041},
{14, 7067, 1, 9041}, {14, 7071, 1, 9041}, {14, 7073, 1, 9041}, {15, 7060, 1, 9041}, {15,
7072, 1, 9041}, {15, 7076, 1, 9041}, {16, 7065, 1, 9041}, {17, 7066, 1, 9041}, {17, 7068,
1, 9041}, {17, 7074, 1, 9041}, {18, 7069, 1, 9041}, {18, 7075, 1, 9041}, {18, 7077, 1,
9041}, {19, 7078, 1, 9041}, {10, 7118, 1, 9042}, {11, 7119, 1, 9042}, {11, 7121, 1, 9042},
{11, 7127, 1, 9042}, {12, 7120, 1, 9042}, {12, 7124, 1, 9042}, {12, 7136, 1, 9042}, {13,
7122, 1, 9042}, {13, 7128, 1, 9042}, {13, 7130, 1, 9042}, {14, 7123, 1, 9042}, {14, 7125,
1, 9042}, {14, 7129, 1, 9042}, {14, 7133, 1, 9042}, {14, 7137, 1, 9042}, {14, 7139, 1,
9042}, {15, 7126, 1, 9042}, {15, 7138, 1, 9042}, {15, 7142, 1, 9042}, {16, 7131, 1, 9042},
{17, 7132, 1, 9042}, {17, 7134, 1, 9042}, {17, 7140, 1, 9042}, {18, 7135, 1, 9042}, {18,
7141, 1, 9042}, {18, 7143, 1, 9042}, {19, 7144, 1, 9042}, {287, 5318, 1, 9043}, {288,
5319, 1, 9043}, {289, 5320, 1, 9043}, {290, 5321, 1, 9044}, {291, 5322, 1, 9044}, {291,
5324, 1, 9044}, {292, 5323, 1, 9044}, {292, 5327, 1, 9044}, {293, 5325, 1, 9044}, {294,
5326, 1, 9044}, {294, 5328, 1, 9044}, {295, 5329, 1, 9044}, {10, 7263, 1, 9045}, {11,
7264, 1, 9045}, {11, 7266, 1, 9045}, {11, 7272, 1, 9045}, {12, 7265, 1, 9045}, {12, 7269,
1, 9045}, {12, 7281, 1, 9045}, {13, 7267, 1, 9045}, {13, 7273, 1, 9045}, {13, 7275, 1,
9045}, {14, 7268, 1, 9045}, {14, 7270, 1, 9045}, {14, 7274, 1, 9045}, {14, 7278, 1, 9045},
{14, 7282, 1, 9045}, {14, 7284, 1, 9045}, {15, 7271, 1, 9045}, {15, 7283, 1, 9045}, {15,
7287, 1, 9045}, {16, 7276, 1, 9045}, {17, 7277, 1, 9045}, {17, 7279, 1, 9045}, {17, 7285,
1, 9045}, {18, 7280, 1, 9045}, {18, 7286, 1, 9045}, {18, 7288, 1, 9045}, {19, 7289, 1,
9045}, {10, 7544, 1, 9046}, {11, 7545, 1, 9046}, {11, 7547, 2, 9046}, {12, 7546, 1, 9046},
{12, 7550, 2, 9046}, {13, 7548, 2, 9046}, {13, 7553, 1, 9046}, {14, 7549, 2, 9046}, {14,
7551, 2, 9046}, {14, 7556, 2, 9046}, {15, 7552, 2, 9046}, {15, 7559, 1, 9046}, {16, 7554,
1, 9046}, {17, 7555, 1, 9046}, {17, 7557, 2, 9046}, {18, 7558, 2, 9046}, {18, 7560, 1,
9046}, {19, 7561, 1, 9046}, {287, 5366, 1, 9047}, {288, 5367, 1, 9047}, {289, 5368, 1,
9047}, {290, 5369, 1, 9048}, {291, 5370, 1, 9048}, {291, 5372, 1, 9048}, {292, 5371, 1,
9048}, {292, 5375, 1, 9048}, {293, 5373, 1, 9048}, {294, 5374, 1, 9048}, {294, 5376, 1,

9048}, {295, 5377, 1, 9048}, {287, 5414, 1, 9049}, {288, 5415, 1, 9049}, {289, 5416, 1, 9049}, {286, 5477, 1, 9050}, {287, 5478, 1, 9051}, {288, 5479, 1, 9051}, {289, 5480, 1, 9051}, {290, 5481, 1, 9052}, {291, 5482, 2, 9052}, {292, 5483, 2, 9052}, {293, 5484, 1, 9052}, {294, 5485, 2, 9052}, {295, 5486, 1, 9052}, {290, 5497, 1, 9053}, {291, 5498, 2, 9053}, {292, 5499, 2, 9053}, {293, 5500, 1, 9053}, {294, 5501, 2, 9053}, {295, 5502, 1, 9053}, {10, 7470, 1, 9054}, {11, 7471, 1, 9054}, {11, 7473, 2, 9054}, {12, 7472, 1, 9054}, {12, 7476, 2, 9054}, {13, 7474, 2, 9054}, {13, 7479, 1, 9054}, {14, 7475, 2, 9054}, {14, 7477, 2, 9054}, {14, 7482, 2, 9054}, {15, 7478, 2, 9054}, {15, 7485, 1, 9054}, {16, 7480, 1, 9054}, {17, 7481, 1, 9054}, {17, 7483, 2, 9054}, {18, 7484, 2, 9054}, {18, 7486, 1, 9054}, {19, 7487, 1, 9054}, {287, 5521, 1, 9055}, {288, 5522, 1, 9055}, {289, 5523, 1, 9055}, {290, 5524, 1, 9056}, {291, 5525, 1, 9056}, {291, 5527, 1, 9056}, {292, 5526, 1, 9056}, {292, 5530, 1, 9056}, {293, 5528, 1, 9056}, {294, 5529, 1, 9056}, {294, 5531, 1, 9056}, {295, 5532, 1, 9056}, {286, 5551, 1, 9057}, {287, 5552, 1, 9058}, {288, 5553, 1, 9058}, {289, 5554, 1, 9058}, {290, 5571, 1, 9059}, {291, 5572, 2, 9059}, {292, 5573, 2, 9059}, {293, 5574, 1, 9059}, {294, 5575, 2, 9059}, {295, 5576, 1, 9059}, {287, 5595, 1, 9060}, {288, 5596, 1, 9060}, {289, 5597, 1, 9060}, {286, 5625, 1, 9061}, {290, 5973, 1, 9062}, {291, 5974, 2, 9062}, {292, 5975, 2, 9062}, {293, 5976, 1, 9062}, {294, 5977, 2, 9062}, {295, 5978, 1, 9062}, {290, 6063, 1, 9063}, {291, 6064, 1, 9063}, {291, 6066, 1, 9063}, {292, 6065, 1, 9063}, {292, 6069, 1, 9063}, {293, 6067, 1, 9063}, {294, 6068, 1, 9063}, {294, 6070, 1, 9063}, {295, 6071, 1, 9063}, {287, 6153, 1, 9064}, {288, 6154, 1, 9064}, {289, 6155, 1, 9064}, {286, 6363, 1, 9065}, {286, 6434, 1, 9066}, {287, 6435, 1, 9067}, {288, 6436, 1, 9067}, {289, 6437, 1, 9067}, {286, 6514, 1, 9068}, {0, 7765, 1, 9069}, {0, 7766, 1, 9070}, {0, 7767, 1, 9071}, {0, 7768, 1, 9072}, {0, 7769, 1, 9073}, {0, 7770, 1, 9074}, {0, 7772, 1, 9075}, {0, 7776, 1, 9076}, {0, 7780, 1, 9077}, {0, 7784, 1, 9078}, {0, 7786, 1, 9079}, {0, 7787, 1, 9080}, {0, 7794, 1, 9081}, {0, 7801, 1, 9082}, {0, 7808, 1, 9083}, {0, 7816, 1, 9084}, {0, 7823, 1, 9085}, {0, 7861, 1, 9086}, {0, 7883, 1, 9087}, {0, 7890, 1, 9088}, {0, 7892, 1, 9089}, {0, 7893, 1, 9090}, {0, 7909, 1, 9091}, {0, 7916, 1, 9092}, {0, 7931, 1, 9093}, {0, 7948, 1, 9094}, {0, 7955, 1, 9095}, {0, 7957, 1, 9096}, {0, 7958, 1, 9097}, {0, 7965, 1, 9098}, {0, 7971, 1, 9099}, {0, 7973, 1, 9100}, {0, 7974, 1, 9101}, {0, 7977, 1, 9102}, {1, 7773, 1, 9103}, {2, 7774, 1, 9103}, {3, 7775, 1, 9103}, {1, 7777, 1, 9104}, {2, 7778, 1, 9104}, {3, 7779, 1, 9104}, {1, 7781, 1, 9105}, {2, 7782, 1, 9105}, {3, 7783, 1, 9105}, {1, 7788, 1, 9106}, {2, 7789, 1, 9106}, {3, 7790, 1, 9106}, {1, 7795, 1, 9107}, {2, 7796, 1, 9107}, {3, 7797, 1, 9107}, {1, 7802, 1, 9108}, {2, 7803, 1, 9108}, {3, 7804, 1, 9108}, {1, 7817, 1, 9109}, {2, 7818, 1, 9109}, {3, 7819, 1, 9109}, {1, 7862, 1, 9110}, {2, 7863, 1, 9110}, {3, 7864, 1, 9110}, {1, 7884, 1, 9111}, {2, 7885, 1, 9111}, {3, 7886, 1, 9111}, {1, 7894, 1, 9112}, {2, 7895, 1, 9112}, {3, 7896, 1, 9112}, {1, 7910, 1, 9113}, {2, 7911, 1, 9113}, {3, 7912, 1, 9113}, {1, 7949, 1, 9114}, {2, 7950, 1, 9114}, {3, 7951, 1, 9114}, {1, 7959, 1, 9115}, {2, 7960, 1, 9115}, {3, 7961, 1, 9115}, {506, 4732, 1, 9116}, {1, 7791, 1, 9117}, {2, 7792, 1, 9117}, {3, 7793, 1, 9117}, {1, 7798, 1, 9118}, {2, 7799, 1, 9118}, {3, 7800, 1, 9118}, {1, 7805, 1, 9119}, {2, 7806, 1, 9119}, {3, 7807, 1, 9119}, {1, 7810, 1, 9120}, {2, 7811, 1, 9120}, {3, 7812, 1, 9120}, {1, 7820, 1, 9121}, {2, 7821, 1, 9121}, {3, 7822, 1, 9121}, {1, 7825, 1, 9122}, {2, 7826, 1, 9122}, {3, 7827, 1, 9122}, {1, 7871, 1, 9123}, {2, 7872, 1, 9123}, {3, 7873, 1, 9123}, {1, 7887, 1, 9124}, {2, 7888, 1, 9124}, {3, 7889, 1, 9124}, {506, 4739, 1, 9125}, {1, 7897, 1, 9126}, {2, 7898, 1, 9126}, {3, 7899, 1, 9126}, {1, 7913, 1, 9127}, {2, 7914, 1, 9127}, {3, 7915, 1, 9127}, {1, 7813, 1, 9128}, {2, 7814, 1, 9128}, {3, 7815, 1, 9128}, {1, 7837, 1, 9129}, {2, 7838, 1, 9129}, {3, 7839, 1, 9129}, {1, 7928, 1, 9130}, {2, 7929, 1, 9130}, {3, 7930, 1, 9130}, {1, 7933, 1, 9131}, {2, 7934, 1, 9131}, {3, 7935, 1, 9131}, {1, 7952, 1, 9132}, {2, 7953, 1, 9132}, {3, 7954, 1, 9132}, {506, 4746, 1, 9133}, {1, 7962, 1, 9134}, {2, 7963, 1, 9134}, {3, 7964, 1, 9134}, {1, 7858, 1, 9135}, {2, 7859, 1, 9135}, {3, 7860, 1, 9135}, {1, 7945, 1, 9136}, {2, 7946, 1, 9136}, {3, 7947, 1, 9136}, {1, 7968, 1, 9137}, {2, 7969, 1, 9137}, {3, 7970, 1, 9137}, {506, 4753, 1, 9138}, {4, 7865, 1, 9139}, {5, 7866, 2, 9139}, {6, 7867, 2, 9139}, {7, 7868, 1, 9139}, {8, 7869, 2, 9139}, {9, 7870, 1, 9139}, {4, 7828, 1, 9140}, {5, 7829, 1, 9140}, {5, 7831, 1, 9140}, {6, 7830, 1, 9140}, {6, 7834, 1, 9140}, {7, 7832, 1, 9140}, {8, 7833, 1, 9140}, {8, 7835, 1, 9140}, {9, 7836, 1, 9140}, {4, 7874, 1, 9141}, {5, 7875, 1, 9141}, {5, 7877, 1, 9141}, {6, 7876, 1, 9141}, {6, 7880, 1, 9141}, {7, 7878, 1, 9141}, {8, 7879, 1, 9141}, {8, 7881, 1, 9141}, {9, 7882, 1, 9141}, {507, 4792, 1, 9142}, {508, 4793, 1, 9142}, {509, 4794, 1, 9142}, {4, 7900, 1, 9143}, {5, 7901, 1, 9143}, {5, 7903, 1, 9143}, {6, 7902, 1, 9143}, {6, 7906, 1, 9143}, {7, 7904, 1, 9143}, {8, 7905, 1, 9143}, {8, 7907, 1, 9143}, {9, 7908, 1, 9143}, {4, 7840, 1, 9144}, {5, 7841, 1, 9144}, {5, 7843, 1, 9144}, {6, 7842, 1, 9144}, {6, 7846, 1, 9144}, {7, 7844, 1, 9144}, {8, 7845, 1, 9144}, {8, 7847, 1, 9144}, {9, 7848, 1, 9144}, {4, 7936, 1, 9145}, {5, 7937, 1, 9145}, {5, 7939, 1, 9145}, {6, 7938, 1, 9145}, {6, 7942, 1, 9145}, {7, 7940, 1, 9145}, {8, 7941, 1, 9145}, {8, 7943, 1, 9145}, {9, 7944, 1, 9145}, {507, 4804, 1, 9146}, {508, 4805, 1, 9146}, {509, 4806, 1, 9146}, {506, 4891, 1, 9147}, {507, 4892, 1, 9148}, {508, 4893, 1, 9148}, {509, 4894, 1, 9148}, {507, 4901, 1, 9149}, {508, 4902, 1, 9149}, {509, 4903, 1, 9149}, {506, 4904, 1, 9150}, {506, 4976, 1, 9151}, {507, 4977, 1, 9152}, {508, 4978, 1, 9152}, {509, 4979, 1, 9152}, {4, 7849, 1, 9153}, {5, 7850, 1, 9153}, {5, 7852, 1, 9153}, {6, 7851, 1, 9153}, {6, 7855, 1, 9153}, {7, 7853, 1, 9153}, {8, 7854,

1, 9153}, {8, 7856, 1, 9153}, {9, 7857, 1, 9153}, {4, 7919, 1, 9154}, {5, 7920, 1, 9154}, {5, 7922, 1, 9154}, {6, 7921, 1, 9154}, {6, 7925, 1, 9154}, {7, 7923, 1, 9154}, {8, 7924, 1, 9154}, {8, 7926, 1, 9154}, {9, 7927, 1, 9154}, {506, 4992, 1, 9155}, {507, 4993, 1, 9156}, {508, 4994, 1, 9156}, {509, 4995, 1, 9156}, {506, 5008, 1, 9157}, {507, 5318, 1, 9158}, {508, 5319, 1, 9158}, {509, 5320, 1, 9158}, {506, 5477, 1, 9159}, {0, 7980, 1, 9160}, {0, 7981, 1, 9161}, {0, 7982, 1, 9162}, {0, 7983, 1, 9163}, {0, 7985, 1, 9164}, {0, 7989, 1, 9165}, {0, 7991, 1, 9166}, {0, 7992, 1, 9167}, {0, 7999, 1, 9168}, {0, 8005, 1, 9169}, {0, 8007, 1, 9170}, {0, 8008, 1, 9171}, {0, 8011, 1, 9172}, {1, 7986, 1, 9173}, {2, 7987, 1, 9173}, {3, 7988, 1, 9173}, {1, 7993, 1, 9174}, {2, 7994, 1, 9174}, {3, 7995, 1, 9174}, {626, 4732, 1, 9175}, {1, 7996, 1, 9176}, {2, 7997, 1, 9176}, {3, 7998, 1, 9176}, {1, 8002, 1, 9177}, {2, 8003, 1, 9177}, {3, 8004, 1, 9177}, {626, 4739, 1, 9178}, {626, 4976, 1, 9179}, {0, 8014, 1, 9180}, {0, 8015, 1, 9181}, {0, 8018, 1, 9182}, {0, 8022, 1, 9183}, {0, 8023, 1, 9184}, {0, 8024, 1, 9185}, {0, 8025, 1, 9186}, {0, 8026, 1, 9187}, {0, 8027, 1, 9188}, {0, 8029, 1, 9189}, {0, 8030, 1, 9190}, {0, 8037, 1, 9191}, {0, 8044, 1, 9192}, {0, 8051, 1, 9193}, {0, 8089, 1, 9194}, {0, 8090, 1, 9195}, {0, 8097, 1, 9196}, {0, 8104, 1, 9197}, {0, 8106, 1, 9198}, {0, 8119, 1, 9199}, {0, 8135, 1, 9200}, {0, 8139, 1, 9201}, {0, 8156, 1, 9202}, {0, 8157, 1, 9203}, {0, 8164, 1, 9204}, {0, 8184, 1, 9205}, {0, 8200, 1, 9206}, {0, 8207, 1, 9207}, {0, 8212, 1, 9208}, {0, 8213, 1, 9209}, {0, 8236, 1, 9210}, {0, 8243, 1, 9211}, {0, 8245, 1, 9212}, {0, 8249, 1, 9213}, {0, 8251, 1, 9214}, {0, 8256, 1, 9215}, {0, 8261, 1, 9216}, {286, 6635, 1, 9217}, {1, 8031, 1, 9218}, {2, 8032, 1, 9218}, {3, 8033, 1, 9218}, {1, 8038, 1, 9219}, {2, 8039, 1, 9219}, {3, 8040, 1, 9219}, {1, 8045, 1, 9220}, {2, 8046, 1, 9220}, {3, 8047, 1, 9220}, {1, 8034, 1, 9221}, {2, 8035, 1, 9221}, {3, 8036, 1, 9221}, {1, 8041, 1, 9222}, {2, 8042, 1, 9222}, {3, 8043, 1, 9222}, {1, 8048, 1, 9223}, {2, 8049, 1, 9223}, {3, 8050, 1, 9223}, {286, 6639, 1, 9224}, {1, 8091, 1, 9225}, {2, 8092, 1, 9225}, {3, 8093, 1, 9225}, {1, 8098, 1, 9226}, {2, 8099, 1, 9226}, {3, 8100, 1, 9226}, {1, 8107, 1, 9227}, {2, 8108, 1, 9227}, {3, 8109, 1, 9227}, {1, 8120, 1, 9228}, {2, 8121, 1, 9228}, {3, 8122, 1, 9228}, {1, 8136, 1, 9229}, {2, 8137, 1, 9229}, {3, 8138, 1, 9229}, {1, 8094, 1, 9230}, {2, 8095, 1, 9230}, {3, 8096, 1, 9230}, {1, 8101, 1, 9231}, {2, 8102, 1, 9231}, {3, 8103, 1, 9231}, {286, 6643, 1, 9232}, {1, 8158, 1, 9233}, {2, 8159, 1, 9233}, {3, 8160, 1, 9233}, {1, 8185, 1, 9234}, {2, 8186, 1, 9234}, {3, 8187, 1, 9234}, {1, 8201, 1, 9235}, {2, 8202, 1, 9235}, {3, 8203, 1, 9235}, {1, 8188, 1, 9236}, {2, 8189, 1, 9236}, {3, 8190, 1, 9236}, {1, 8204, 1, 9237}, {2, 8205, 1, 9237}, {3, 8206, 1, 9237}, {1, 8161, 1, 9238}, {2, 8162, 1, 9238}, {3, 8163, 1, 9238}, {286, 6647, 1, 9239}, {1, 8237, 1, 9240}, {2, 8238, 1, 9240}, {3, 8239, 1, 9240}, {1, 8246, 1, 9241}, {2, 8247, 1, 9241}, {3, 8248, 1, 9241}, {1, 8240, 1, 9242}, {2, 8241, 1, 9242}, {3, 8242, 1, 9242}, {286, 6657, 1, 9243}, {286, 6658, 1, 9244}, {1, 8053, 1, 9245}, {2, 8054, 1, 9245}, {3, 8055, 1, 9245}, {1, 8065, 1, 9246}, {2, 8066, 1, 9246}, {3, 8067, 1, 9246}, {1, 8086, 1, 9247}, {2, 8087, 1, 9247}, {3, 8088, 1, 9247}, {286, 6665, 1, 9248}, {1, 8141, 1, 9249}, {2, 8142, 1, 9249}, {3, 8143, 1, 9249}, {1, 8153, 1, 9250}, {2, 8154, 1, 9250}, {3, 8155, 1, 9250}, {1, 8166, 1, 9251}, {2, 8167, 1, 9251}, {3, 8168, 1, 9251}, {1, 8169, 1, 9252}, {2, 8170, 1, 9252}, {3, 8171, 1, 9252}, {1, 8181, 1, 9253}, {2, 8182, 1, 9253}, {3, 8183, 1, 9253}, {286, 6672, 1, 9254}, {1, 8209, 1, 9255}, {2, 8210, 1, 9255}, {3, 8211, 1, 9255}, {1, 8221, 1, 9256}, {2, 8222, 1, 9256}, {3, 8223, 1, 9256}, {1, 8233, 1, 9257}, {2, 8234, 1, 9257}, {3, 8235, 1, 9257}, {286, 6679, 1, 9258}, {1, 8253, 1, 9259}, {2, 8254, 1, 9259}, {3, 8255, 1, 9259}, {1, 8258, 1, 9260}, {2, 8259, 1, 9260}, {3, 8260, 1, 9260}, {286, 6725, 1, 9261}, {287, 6726, 1, 9262}, {288, 6727, 1, 9262}, {289, 6728, 1, 9262}, {287, 6695, 1, 9263}, {288, 6696, 1, 9263}, {289, 6697, 1, 9263}, {4, 8123, 1, 9264}, {5, 8124, 2, 9264}, {6, 8125, 2, 9264}, {7, 8126, 1, 9264}, {8, 8127, 2, 9264}, {9, 8128, 1, 9264}, {286, 6738, 1, 9265}, {287, 6735, 1, 9266}, {288, 6736, 1, 9266}, {289, 6737, 1, 9266}, {4, 8110, 1, 9267}, {5, 8111, 1, 9267}, {5, 8113, 1, 9267}, {6, 8112, 1, 9267}, {6, 8116, 1, 9267}, {7, 8114, 1, 9267}, {8, 8115, 1, 9267}, {8, 8117, 1, 9267}, {9, 8118, 1, 9267}, {4, 8129, 1, 9268}, {5, 8130, 2, 9268}, {6, 8131, 2, 9268}, {7, 8132, 1, 9268}, {8, 8133, 2, 9268}, {9, 8134, 1, 9268}, {287, 6698, 1, 9269}, {288, 6699, 1, 9269}, {289, 6700, 1, 9269}, {4, 8191, 1, 9270}, {5, 8192, 1, 9270}, {5, 8194, 1, 9270}, {6, 8193, 1, 9270}, {6, 8197, 1, 9270}, {7, 8195, 1, 9270}, {8, 8196, 1, 9270}, {8, 8198, 1, 9270}, {9, 8199, 1, 9270}, {286, 6838, 1, 9271}, {287, 6839, 1, 9272}, {288, 6840, 1, 9272}, {289, 6841, 1, 9272}, {4, 8068, 1, 9273}, {5, 8069, 1, 9273}, {5, 8071, 1, 9273}, {6, 8070, 1, 9273}, {6, 8074, 1, 9273}, {7, 8072, 1, 9273}, {8, 8073, 1, 9273}, {8, 8075, 1, 9273}, {9, 8076, 1, 9273}, {287, 6760, 1, 9274}, {288, 6761, 1, 9274}, {289, 6762, 1, 9274}, {4, 8056, 1, 9275}, {5, 8057, 1, 9275}, {5, 8059, 1, 9275}, {6, 8058, 1, 9275}, {6, 8062, 1, 9275}, {7, 8060, 1, 9275}, {8, 8061, 1, 9275}, {8, 8063, 1, 9275}, {9, 8064, 1, 9275}, {4, 8077, 1, 9276}, {5, 8078, 1, 9276}, {5, 8080, 1, 9276}, {6, 8079, 1, 9276}, {6, 8083, 1, 9276}, {7, 8081, 1, 9276}, {8, 8082, 1, 9276}, {8, 8084, 1, 9276}, {9, 8085, 1, 9276}, {286, 6860, 1, 9277}, {287, 6861, 1, 9278}, {288, 6862, 1, 9278}, {289, 6863, 1, 9278}, {287, 6848, 1, 9279}, {288, 6849, 1, 9279}, {289, 6850, 1, 9279}, {4, 8172, 1, 9280}, {5, 8173, 1, 9280}, {5, 8175, 1, 9280}, {6, 8174, 1, 9280}, {6, 8178, 1, 9280}, {7, 8176, 1, 9280}, {8, 8177, 1, 9280}, {8, 8179, 1, 9280}, {9, 8180, 1, 9280}, {287, 6772, 1, 9281}, {288, 6773, 1, 9281}, {289, 6774, 1, 9281}, {4, 8144, 1, 9282}, {5, 8145, 1, 9282}, {5, 8147, 1, 9282}, {6, 8146, 1, 9282}, {6, 8150, 1, 9282}, {7, 8148, 1, 9282}, {8, 8149, 1,

9282}, {8, 8151, 1, 9282}, {9, 8152, 1, 9282}, {286, 6882, 1, 9283}, {287, 6870, 1, 9284},
{288, 6871, 1, 9284}, {289, 6872, 1, 9284}, {4, 8224, 1, 9285}, {5, 8225, 1, 9285}, {5,
8227, 1, 9285}, {6, 8226, 1, 9285}, {6, 8230, 1, 9285}, {7, 8228, 1, 9285}, {8, 8229, 1,
9285}, {8, 8231, 1, 9285}, {9, 8232, 1, 9285}, {287, 6793, 1, 9286}, {288, 6794, 1, 9286},
{289, 6795, 1, 9286}, {286, 6913, 1, 9287}, {286, 6914, 1, 9288}, {287, 6915, 1, 9289},
{288, 6916, 1, 9289}, {289, 6917, 1, 9289}, {286, 6930, 1, 9290}, {287, 6931, 1, 9291},
{288, 6932, 1, 9291}, {289, 6933, 1, 9291}, {287, 6918, 1, 9292}, {288, 6919, 1, 9292},
{289, 6920, 1, 9292}, {4, 8215, 1, 9293}, {5, 8216, 2, 9293}, {6, 8217, 2, 9293}, {7,
8218, 1, 9293}, {8, 8219, 2, 9293}, {9, 8220, 1, 9293}, {286, 6952, 1, 9294}, {287, 6940,
1, 9295}, {288, 6941, 1, 9295}, {289, 6942, 1, 9295}, {286, 7169, 1, 9296}, {287, 7079, 1,
9297}, {288, 7080, 1, 9297}, {289, 7081, 1, 9297}, {290, 7043, 1, 9298}, {291, 7044, 1,
9298}, {291, 7046, 1, 9298}, {292, 7045, 1, 9298}, {292, 7049, 1, 9298}, {293, 7047, 1,
9298}, {294, 7048, 1, 9298}, {294, 7050, 1, 9298}, {295, 7051, 1, 9298}, {286, 7314, 1,
9299}, {287, 7315, 1, 9300}, {288, 7316, 1, 9300}, {289, 7317, 1, 9300}, {287, 7212, 1,
9301}, {288, 7213, 1, 9301}, {289, 7214, 1, 9301}, {290, 7215, 1, 9302}, {291, 7216, 1,
9302}, {291, 7218, 1, 9302}, {292, 7217, 1, 9302}, {292, 7221, 1, 9302}, {293, 7219, 1,
9302}, {294, 7220, 1, 9302}, {294, 7222, 1, 9302}, {295, 7223, 1, 9302}, {286, 7354, 1,
9303}, {287, 7324, 1, 9304}, {288, 7325, 1, 9304}, {289, 7326, 1, 9304}, {287, 7224, 1,
9305}, {288, 7225, 1, 9305}, {289, 7226, 1, 9305}, {286, 7415, 1, 9306}, {286, 7416, 1,
9307}, {287, 7417, 1, 9308}, {288, 7418, 1, 9308}, {289, 7419, 1, 9308}, {286, 7432, 1,
9309}, {287, 7420, 1, 9310}, {288, 7421, 1, 9310}, {289, 7422, 1, 9310}, {286, 7637, 1,
9311}, {287, 7601, 1, 9312}, {288, 7602, 1, 9312}, {289, 7603, 1, 9312}, {286, 7674, 1,
9313}, {286, 7675, 1, 9314}, {286, 7742, 1, 9315}, {0, 8267, 1, 9316}, {0, 8268, 1, 9317},
{0, 8269, 1, 9318}, {0, 8270, 1, 9319}, {0, 8272, 1, 9320}, {0, 8273, 1, 9321}, {0, 8277,
1, 9322}, {0, 8279, 1, 9323}, {0, 8280, 1, 9324}, {0, 8287, 1, 9325}, {0, 8292, 1, 9326},
{0, 8293, 1, 9327}, {0, 8298, 1, 9328}, {0, 8305, 1, 9329}, {0, 8307, 1, 9330}, {0, 8308,
1, 9331}, {0, 8310, 1, 9332}, {0, 8315, 1, 9333}, {0, 8317, 1, 9334}, {0, 8319, 1, 9335},
{286, 7772, 1, 9336}, {1, 8274, 1, 9337}, {2, 8275, 1, 9337}, {3, 8276, 1, 9337}, {1,
8281, 1, 9338}, {2, 8282, 1, 9338}, {3, 8283, 1, 9338}, {286, 7776, 1, 9339}, {1, 8299, 1,
9340}, {2, 8300, 1, 9340}, {3, 8301, 1, 9340}, {506, 6635, 1, 9341}, {1, 8284, 1, 9342},
{2, 8285, 1, 9342}, {3, 8286, 1, 9342}, {1, 8302, 1, 9343}, {2, 8303, 1, 9343}, {3, 8304,
1, 9343}, {506, 6639, 1, 9344}, {286, 7786, 1, 9345}, {286, 7787, 1, 9346}, {1, 8289, 1,
9347}, {2, 8290, 1, 9347}, {3, 8291, 1, 9347}, {506, 6658, 1, 9348}, {1, 8295, 1, 9349},
{2, 8296, 1, 9349}, {3, 8297, 1, 9349}, {286, 7794, 1, 9350}, {1, 8312, 1, 9351}, {2,
8313, 1, 9351}, {3, 8314, 1, 9351}, {506, 6665, 1, 9352}, {286, 7861, 1, 9353}, {287,
7825, 1, 9354}, {288, 7826, 1, 9354}, {289, 7827, 1, 9354}, {506, 6838, 1, 9355}, {286,
7892, 1, 9356}, {286, 7893, 1, 9357}, {506, 6914, 1, 9358}, {286, 7957, 1, 9359}, {0,
8326, 1, 9360}, {0, 8327, 1, 9361}, {0, 8329, 1, 9362}, {0, 8331, 1, 9363}, {286, 7991, 1,
9364}, {0, 8337, 1, 9365}, {0, 8338, 1, 9366}, {0, 8340, 1, 9367}, {506, 7786, 1, 9368},
{0, 8345, 1, 9369}, {0, 8346, 1, 9370}, {0, 8347, 1, 9371}, {0, 8348, 1, 9372}, {0, 8350,
1, 9373}, {0, 8357, 1, 9374}, {0, 8360, 1, 9375}, {286, 8029, 1, 9376}, {286, 8030, 1,
9377}, {286, 8037, 1, 9378}, {1, 8351, 1, 9379}, {2, 8352, 1, 9379}, {3, 8353, 1, 9379},
{1, 8354, 1, 9380}, {2, 8355, 1, 9380}, {3, 8356, 1, 9380}, {286, 8089, 1, 9381}, {286,
8090, 1, 9382}, {286, 8106, 1, 9383}, {286, 8119, 1, 9384}, {287, 8107, 1, 9385}, {288,
8108, 1, 9385}, {289, 8109, 1, 9385}, {286, 8156, 1, 9386}, {286, 8184, 1, 9387}, {287,
8166, 1, 9388}, {288, 8167, 1, 9388}, {289, 8168, 1, 9388}, {0, 8362, 1, 9389}, {0, 8363,
1, 9390}, {0, 8366, 1, 9391}, {286, 8272, 1, 9392}, {286, 8279, 1, 9393}, {286, 8345, 1,
9394}, {286, 8346, 1, 9395}}
alpha_scalar_moments = 1464
alpha_moment_mapping = {0, 286, 506, 626, 682, 702, 706, 707, 771, 989, 1532, 2063, 2444,
2669, 2734, 2750, 2754, 2755, 2756, 2826, 3026, 3241, 3556, 3826, 3927, 3965, 3966, 3967,
3968, 4038, 4148, 4258, 4314, 4337, 4338, 4339, 4340, 4362, 4390, 4403, 4404, 4405, 4406,
4413, 4414, 4415, 4416, 4417, 4418, 4482, 4547, 4612, 4628, 4632, 4633, 4634, 4635, 4657,
4685, 4698, 4699, 4700, 4701, 4708, 4709, 4710, 4711, 4712, 4713, 4717, 4718, 4719, 4720,
4721, 4722, 4723, 4724, 4725, 4726, 4727, 4728, 4729, 4730, 4731, 4732, 4739, 4746, 4753,
4760, 4767, 4774, 4781, 4782, 4891, 4904, 4923, 4942, 4961, 4974, 4975, 4976, 4992, 5008,
5024, 5040, 5056, 5060, 5061, 5209, 5247, 5275, 5298, 5299, 5477, 5551, 5625, 5681, 5704,
5705, 5922, 5935, 5936, 6363, 6409, 6432, 6433, 6434, 6514, 6530, 6534, 6535, 6536, 6573,
6574, 6608, 6621, 6622, 6623, 6624, 6625, 6626, 6627, 6628, 6629, 6630, 6631, 6632, 6633,
6634, 6635, 6639, 6643, 6647, 6651, 6655, 6656, 6657, 6658, 6665, 6672, 6679, 6686, 6693,
6694, 6725, 6738, 6751, 6758, 6759, 6838, 6860, 6882, 6904, 6911, 6912, 6913, 6914, 6930,
6952, 6980, 6993, 6994, 7028, 7041, 7042, 7169, 7197, 7210, 7211, 7314, 7354, 7400, 7413,
7414, 7415, 7416, 7432, 7454, 7467, 7468, 7469, 7506, 7507, 7586, 7599, 7600, 7637, 7659,
7672, 7673, 7674, 7675, 7691, 7698, 7699, 7700, 7716, 7717, 7733, 7740, 7741, 7742, 7743,
7750, 7751, 7752, 7756, 7757, 7758, 7759, 7760, 7761, 7762, 7763, 7764, 7765, 7766, 7767,
7768, 7769, 7770, 7771, 7772, 7776, 7780, 7784, 7785, 7786, 7787, 7794, 7801, 7808, 7809,
7816, 7823, 7824, 7861, 7883, 7890, 7891, 7892, 7893, 7909, 7916, 7917, 7918, 7931, 7932,
7948, 7955, 7956, 7957, 7958, 7965, 7966, 7967, 7971, 7972, 7973, 7974, 7975, 7976, 7977,
7978, 7979, 7980, 7981, 7982, 7983, 7984, 7985, 7989, 7990, 7991, 7992, 7999, 8000, 8001,

8005, 8006, 8007, 8008, 8009, 8010, 8011, 8012, 8013, 8014, 8015, 8016, 8017, 8018, 8019,
8020, 8021, 8022, 8023, 8024, 8025, 8026, 8027, 8028, 8029, 8030, 8037, 8044, 8051, 8052,
8089, 8090, 8097, 8104, 8105, 8106, 8119, 8135, 8139, 8140, 8156, 8157, 8164, 8165, 8184,
8200, 8207, 8208, 8212, 8213, 8214, 8236, 8243, 8244, 8245, 8249, 8250, 8251, 8252, 8256,
8257, 8261, 8262, 8263, 8264, 8265, 8266, 8267, 8268, 8269, 8270, 8271, 8272, 8273, 8277,
8278, 8279, 8280, 8287, 8288, 8292, 8293, 8294, 8298, 8305, 8306, 8307, 8308, 8309, 8310,
8311, 8315, 8316, 8317, 8318, 8319, 8320, 8321, 8322, 8323, 8324, 8325, 8326, 8327, 8328,
8329, 8330, 8331, 8332, 8333, 8334, 8335, 8336, 8337, 8338, 8339, 8340, 8341, 8342, 8343,
8344, 8345, 8346, 8347, 8348, 8349, 8350, 8357, 8358, 8359, 8360, 8361, 8362, 8363, 8364,
8365, 8366, 8367, 8368, 8369, 8370, 8499, 8500, 8501, 8502, 8503, 8504, 8505, 8506, 8507,
8508, 8509, 8510, 8511, 8512, 8513, 8514, 8515, 8516, 8517, 8518, 8519, 8520, 8521, 8522,
8523, 8524, 8525, 8526, 8527, 8528, 8529, 8530, 8531, 8532, 8533, 8534, 8535, 8536, 8537,
8538, 8539, 8540, 8541, 8542, 8543, 8544, 8545, 8546, 8547, 8548, 8549, 8550, 8551, 8552,
8553, 8554, 8555, 8556, 8557, 8558, 8371, 8559, 8560, 8561, 8562, 8563, 8564, 8565, 8566,
8567, 8568, 8569, 8570, 8571, 8572, 8573, 8574, 8575, 8576, 8372, 8577, 8578, 8579, 8580,
8581, 8582, 8583, 8584, 8585, 8586, 8587, 8588, 8589, 8590, 8591, 8592, 8593, 8594, 8373,
8595, 8596, 8597, 8598, 8599, 8600, 8601, 8602, 8603, 8604, 8605, 8606, 8607, 8608, 8609,
8374, 8610, 8611, 8612, 8613, 8614, 8615, 8616, 8617, 8618, 8619, 8375, 8620, 8621, 8622,
8623, 8624, 8625, 8376, 8626, 8627, 8628, 8377, 8629, 8378, 8630, 8631, 8632, 8633, 8634,
8635, 8636, 8637, 8638, 8379, 8380, 8639, 8640, 8641, 8642, 8643, 8644, 8645, 8646, 8647,
8648, 8649, 8650, 8651, 8381, 8382, 8652, 8653, 8654, 8655, 8656, 8657, 8658, 8659, 8660,
8383, 8384, 8661, 8662, 8663, 8664, 8665, 8385, 8386, 8666, 8667, 8387, 8388, 8389, 8668,
8669, 8670, 8671, 8672, 8673, 8674, 8675, 8676, 8677, 8678, 8679, 8680, 8681, 8682, 8683,
8684, 8685, 8686, 8687, 8688, 8689, 8690, 8691, 8692, 8693, 8694, 8695, 8696, 8697, 8698,
8699, 8700, 8701, 8702, 8703, 8704, 8705, 8706, 8707, 8708, 8709, 8710, 8711, 8712, 8713,
8714, 8390, 8715, 8716, 8391, 8392, 8393, 8717, 8718, 8394, 8719, 8395, 8396, 8397, 8398,
8399, 8400, 8401, 8720, 8721, 8722, 8402, 8723, 8724, 8725, 8726, 8727, 8728, 8729, 8730,
8731, 8403, 8732, 8733, 8404, 8734, 8735, 8736, 8737, 8738, 8739, 8405, 8740, 8406, 8741,
8742, 8743, 8407, 8744, 8745, 8746, 8747, 8748, 8749, 8750, 8751, 8408, 8409, 8410, 8411,
8752, 8412, 8413, 8414, 8415, 8753, 8754, 8755, 8756, 8757, 8758, 8759, 8760, 8761, 8762,
8763, 8764, 8765, 8766, 8767, 8768, 8769, 8770, 8771, 8772, 8773, 8774, 8775, 8776, 8777,
8778, 8779, 8780, 8781, 8782, 8783, 8784, 8785, 8786, 8787, 8788, 8789, 8790, 8791, 8792,
8793, 8794, 8795, 8796, 8797, 8798, 8799, 8800, 8801, 8802, 8803, 8804, 8805, 8806, 8807,
8808, 8809, 8810, 8811, 8812, 8813, 8814, 8815, 8816, 8817, 8818, 8819, 8820, 8821, 8822,
8823, 8824, 8825, 8826, 8827, 8828, 8829, 8830, 8831, 8832, 8833, 8834, 8416, 8835, 8836,
8837, 8838, 8839, 8840, 8841, 8842, 8843, 8844, 8845, 8846, 8417, 8847, 8848, 8849, 8850,
8851, 8852, 8853, 8854, 8855, 8856, 8418, 8857, 8858, 8859, 8860, 8861, 8862, 8419, 8863,
8864, 8865, 8420, 8866, 8421, 8867, 8868, 8869, 8870, 8871, 8872, 8422, 8873, 8874, 8875,
8876, 8877, 8878, 8879, 8880, 8881, 8882, 8883, 8884, 8885, 8886, 8423, 8887, 8888, 8889,
8890, 8891, 8892, 8893, 8894, 8895, 8896, 8897, 8898, 8899, 8900, 8901, 8424, 8902, 8903,
8904, 8905, 8906, 8907, 8908, 8909, 8910, 8911, 8912, 8913, 8914, 8915, 8425, 8916, 8917,
8918, 8919, 8920, 8921, 8922, 8923, 8924, 8925, 8926, 8426, 8927, 8928, 8929, 8930, 8931,
8932, 8933, 8934, 8935, 8936, 8937, 8427, 8428, 8938, 8939, 8940, 8941, 8942, 8429, 8430,
8943, 8944, 8431, 8432, 8433, 8945, 8946, 8947, 8948, 8949, 8950, 8951, 8952, 8953, 8954,
8955, 8956, 8957, 8958, 8959, 8960, 8961, 8962, 8963, 8964, 8965, 8966, 8967, 8968, 8969,
8970, 8971, 8972, 8973, 8974, 8975, 8976, 8977, 8978, 8979, 8980, 8981, 8434, 8435, 8982,
8436, 8983, 8984, 8985, 8986, 8987, 8988, 8989, 8990, 8437, 8438, 8439, 8991, 8992, 8993,
8994, 8995, 8996, 8997, 8440, 8441, 8998, 8999, 9000, 9001, 9002, 9003, 9004, 9005, 9006,
9007, 9008, 9009, 9010, 9011, 9012, 9013, 9014, 9015, 9016, 9017, 9018, 9019, 9020, 9021,
9022, 9023, 9024, 9025, 8442, 8443, 8444, 8445, 9026, 8446, 9027, 8447, 8448, 9028, 8449,
8450, 9029, 9030, 9031, 9032, 8451, 9033, 9034, 9035, 9036, 9037, 8452, 8453, 9038, 9039,
9040, 9041, 9042, 9043, 9044, 9045, 9046, 9047, 9048, 9049, 9050, 9051, 9052, 9053, 9054,
9055, 9056, 9057, 9058, 8454, 9059, 9060, 9061, 9062, 9063, 9064, 9065, 9066, 9067, 9068,
9069, 9070, 9071, 9072, 9073, 9074, 9075, 9076, 9077, 9078, 9079, 9080, 9081, 9082, 9083,
9084, 9085, 9086, 9087, 9088, 9089, 9090, 9091, 9092, 9093, 9094, 9095, 9096, 9097, 9098,
9099, 9100, 9101, 9102, 9103, 9104, 9105, 8455, 9106, 9107, 9108, 9109, 9110, 9111, 8456,
9112, 9113, 9114, 8457, 9115, 8458, 9116, 9117, 9118, 9119, 8459, 9120, 9121, 9122, 9123,
9124, 9125, 9126, 9127, 8460, 9128, 9129, 9130, 9131, 9132, 9133, 9134, 8461, 9135, 9136,
9137, 9138, 9139, 8462, 8463, 8464, 9140, 9141, 9142, 9143, 9144, 9145, 9146, 9147, 9148,
8465, 8466, 9149, 9150, 9151, 9152, 9153, 9154, 9155, 9156, 9157, 9158, 9159, 9160, 9161,
9162, 9163, 9164, 9165, 9166, 9167, 9168, 9169, 9170, 9171, 9172, 9173, 8467, 9174, 8468,
9175, 9176, 8469, 9177, 9178, 9179, 9180, 9181, 9182, 9183, 9184, 9185, 9186, 9187, 9188,
9189, 9190, 9191, 9192, 9193, 9194, 9195, 9196, 9197, 9198, 9199, 9200, 9201, 9202, 9203,
9204, 9205, 9206, 9207, 9208, 9209, 9210, 9211, 9212, 9213, 9214, 9215, 9216, 9217, 9218,
9219, 9220, 8470, 9221, 9222, 9223, 9224, 9225, 9226, 9227, 9228, 9229, 8471, 9230, 9231,
9232, 9233, 9234, 9235, 9236, 9237, 8472, 9238, 9239, 9240, 9241, 9242, 8473, 9243, 8474,
8475, 8476, 8477, 9244, 9245, 9246, 9247, 9248, 9249, 9250, 9251, 9252, 9253, 9254, 9255,
9256, 9257, 9258, 9259, 9260, 9261, 9262, 9263, 9264, 8478, 8479, 9265, 9266, 8480, 8481,
9267, 9268, 9269, 9270, 9271, 9272, 9273, 9274, 9275, 9276, 9277, 9278, 9279, 9280, 9281,

9282, 9283, 9284, 9285, 9286, 9287, 8482, 8483, 8484, 9288, 9289, 8485, 9290, 9291, 9292,
9293, 9294, 9295, 9296, 9297, 8486, 9298, 9299, 9300, 9301, 9302, 9303, 9304, 9305, 9306,
8487, 8488, 9307, 9308, 9309, 9310, 9311, 9312, 9313, 8489, 9314, 9315, 9316, 9317, 9318,
9319, 9320, 9321, 9322, 9323, 9324, 9325, 9326, 9327, 9328, 9329, 9330, 9331, 9332, 9333,
9334, 9335, 9336, 9337, 8490, 9338, 9339, 9340, 8491, 9341, 9342, 8492, 9343, 9344, 9345,
8493, 8494, 9346, 9347, 9348, 9349, 9350, 9351, 9352, 9353, 9354, 9355, 9356, 8495, 9357,
9358, 9359, 9360, 9361, 9362, 9363, 9364, 9365, 9366, 9367, 9368, 9369, 9370, 9371, 9372,
9373, 9374, 9375, 9376, 9377, 9378, 9379, 8496, 9380, 8497, 9381, 9382, 8498, 9383, 9384,
9385, 9386, 9387, 9388, 9389, 9390, 9391, 9392, 9393, 9394, 9395}
species_coeffs = {3.915032908698106e+00}
moment_coeffs = {1.617342212820079e+01, -1.037426849501096e+01, -9.340744616767168e+00,
-1.913959339892077e+01, 2.329330990550649e+01, 3.284874959497165e+00,
1.476012991728794e+01, -3.044553817245940e+00, 1.821792372979555e+01,
5.728442798026283e+00, 3.653042931666175e+00, 1.061538403412536e+01,
6.878919785639890e+00, -4.241856624898755e+00, -4.960426804346412e+01,
-2.618465075145229e+01, 1.171516295946780e+01, -2.658082900948406e+01,
-1.877549781576717e+00, -1.052830371602957e+01, -9.344942882212516e+00,
4.020016878432737e+01, 4.005345920550086e+01, -1.369388709222656e+01,
-2.589870804486234e+01, -1.109512373979146e+01, 2.923658060511005e-02,
-1.654510870897005e+01, -4.678865044241322e+00, 6.271625671753224e-01,
-1.747885165603633e+01, -1.555145415644546e+01, 1.339995072433954e+01,
1.555848359533125e+01, 2.570598940786228e+00, -2.520733826909517e+01,
4.567150780255679e+00, -2.390348359862040e-01, -3.702407860872189e+00,
-7.091211671677886e+00, -4.279546571883155e+00, 3.611931371492166e+01,
-5.802818268356170e+00, -3.649540317619305e-01, 1.674439474289122e+00,
1.046522586376038e+00, 5.741381098638668e-01, 5.561363990947624e-01,
-8.984183167228923e-01, -6.155306551897319e+00, -2.777687844575613e+00,
8.731166201244688e+00, 4.024405294692413e+00, -4.534659347016688e+00,
-2.804001894431914e-01, -7.312522504752346e-01, -4.272554992524025e+00,
-2.039245667227564e+00, 2.725593946080140e+00, -1.432269771162905e+00,
-1.369691752019126e+00, 4.223308814875394e+00, 4.173097772050347e+00,
-2.399402585936282e+00, -1.832809870242438e+00, -2.858916049037211e+00,
6.744418346595092e-02, 4.634170466474368e-01, -2.937412655910405e+00,
-2.845152197257153e-03, 4.231518044095393e-01, 2.270776122127371e+00,
-2.557309650739475e-01, 5.584089307695304e+00, 1.220999236946027e+01,
2.337158977794837e+00, -3.569277188348761e+01, 1.068750536798207e+01,
3.304438522624949e+01, 8.846193955453552e+00, 2.393254520734910e+00,
-3.605634360524378e+01, -2.430088578292839e+01, 8.547151528248550e+00,
8.309377160000941e-01, -6.550824657664865e+01, -4.859332313516994e+01,
8.842649137626921e+01, 6.414407288618263e+01, -6.090340294614332e+01,
-3.416426163270079e+01, 6.226649371966024e+00, 2.013832702095725e+00,
1.785630709338938e+01, 1.369448107670814e+02, 3.450535473212186e+01,
-3.936369355339303e+01, -4.308382202729152e+01, -1.540353126801226e+01,
-6.040530372594701e+00, -6.776976067924084e-01, 3.937218107998269e+01,
-9.247017502517649e-01, -7.278207142825553e+01, 1.251453497026252e+01,
3.981615494952927e+01, 1.164617987502169e+01, -8.384714012086515e-02,
1.806364917016697e+01, -8.186965486869335e+01, 1.775853827507006e+01,
8.982006500436592e+00, -1.410265104014885e+01, 1.579749775409860e+02,
-9.738216670987090e+01, -2.812032909859379e+02, 1.266998457068932e+02,
4.046624895189184e+01, -1.720476743351325e+00, -2.617092771626668e+01,
-1.747688542037267e+00, 9.314399777175104e-01, -6.251453986201839e+01,
1.302286308439137e+02, 1.682157169903127e+02, 1.653486048253447e+01,
1.739341325386525e+01, -1.463527747840816e+02, -9.520588336097072e+01,
1.995986215280764e+00, -1.879095346010349e+01, 1.160804071989997e+01,
2.031139508527814e+01, -1.174335457109310e+01, 1.012575438309891e+02,
1.290228973874616e+01, -1.073547229212357e+01, -6.018861307099292e+01,
4.927308576570545e+01, -6.547084350247836e+01, -1.629835083228520e+01,
8.409203223916797e-01, -6.882695935706880e+00, -5.150470848342749e+00,
2.023848917826769e+01, 1.124286537430394e+01, -1.245791132010356e+01,
-1.008427782137823e+01, -4.161041051238636e+00, -2.336858748728977e+00,
-3.987565784853488e+01, -1.915055697945328e+01, 3.889698123356722e+01,
3.045521158850028e+01, -4.701154559795896e+00, 4.252324683872585e-01,
-1.022036127883987e+01, -1.376903960173271e+01, 4.435447252862376e+00,
3.468301893761850e+01, 1.422404347397883e+01, -2.689332476546035e+01,
-1.470583122761003e+01, 4.630636013847769e+00, -2.379082499687484e+01,
2.259576452114597e+01, 4.523848793488091e+01, 3.667451777193573e+00,
1.057980775589741e+00, -3.785061752947226e+00, -4.822434441059677e+00,
2.966299328170838e+01, 3.470436809847386e+01, -1.950530071549504e+01,
-3.607198317707828e+00, -1.322372486070067e+00, -6.009688752542916e+00,

2.732296689271502e+01, 2.133508325417375e+01, -6.997834657178712e+00,
-2.063863313142417e+01, -2.940590822362017e+00, 1.800117071785315e+01,
-2.085607251385329e+00, -8.269780225001379e-01, 1.669100883948129e+01,
-1.923924927273645e+01, -4.974431524109838e+01, -2.907457539469503e+00,
-1.115387657367852e+00, 2.478242166293716e+01, 1.233432177253743e+01,
4.146634713332813e-01, -1.641327155965732e+01, 1.235432296447988e+01,
-9.688434777741790e+00, -1.150192885390637e+01, -4.318403980079073e+00,
-2.487136711512486e+00, -1.730065447083076e-01, -3.573582564130212e+00,
-2.917330968382681e+01, -5.719960625689217e+00, -7.510972255126983e+00,
-6.233434920341831e+01, -1.201298010321030e+01, -1.118168907469605e+01,
2.256203957854497e+01, 1.424826590621289e+01, -3.385078587229178e+00,
3.357776425727277e+00, -9.888173079891272e+00, -2.846684675950289e+01,
1.085914580841836e+01, 4.636307611511317e+00, -7.118952398724372e+00,
1.239175960851707e+01, -8.269930630676262e+00, -2.232737769001391e-01,
-1.323022387672156e+01, 1.505095907253106e+01, -6.818561195710116e+00,
2.595858948832871e+01, 6.565976522509839e-01, 3.341636519325457e+00,
3.087444218483640e+00, 2.852689985702546e+01, 2.592012671211088e+00,
3.869943230352074e+00, 4.707815613346396e+00, -3.488894758276785e+00,
3.634834477444795e+00, 3.522126173904823e+00, -2.934399893820341e+00,
-1.041246112982490e+00, -8.111905238249800e+00, 7.719734528986464e+00,
2.340575881389912e+00, -1.416330689487449e+01, -9.041445179278774e+00,
4.874220731001572e+00, 2.613392200672166e+00, 4.673711288195728e+00,
-1.288863356140119e+01, -8.991330498208599e+00, -5.415898784505970e+00,
1.373816050243897e+00, -7.735047905860195e+00, -2.084685752449373e+00,
6.873772796147979e+00, 1.842373122217587e+00, 1.429081216333454e+00,
6.627725517627905e+00, -7.671510901136849e+00, 1.402818358517889e+00,
3.817976138224525e+00, 2.630667017795952e+00, 2.110276160531518e+00,
2.647588459526427e+00, 5.465689022159001e+00, -5.693971285979927e+00,
-1.825316719934692e+01, -1.547755922181842e+00, 7.711597655006675e+00,
6.803747102318469e+00, 9.263547256754611e-01, 1.081507982820911e+01,
1.303653589703735e+01, -2.188049582565746e+01, -1.215143812057379e+01,
9.286119114967832e+00, -1.344998026248448e+00, -9.144254055859179e+00,
2.407038172259275e+00, 4.746511180809287e+00, 9.851878932369010e+00,
-1.174211150232559e+01, 5.478050900389984e+00, 3.954424683457936e+00,
3.013612691890029e+00, 2.296791009437612e+00, -6.177516479190589e-01,
4.570493571477042e+00, 3.039285121426013e+00, 1.422398822002652e+00,
-1.105516746279311e+01, 3.518955598687171e+00, -3.360170608072824e+00,
2.194426006617885e+00, 8.519688264161347e-01, 1.087262276571893e+00,
-7.274397644083558e+00, 7.733077631154679e-01, -6.725191692654987e+00,
8.958714480668533e+00, 6.320642966261600e+00, 8.334147449394992e-01,
9.866721714692517e-01, 4.759838878165610e+00, -1.830570853057016e+00,
-4.686269421368965e+00, -3.646580196894379e+00, -4.281430816597432e+00,
-7.536600823183471e+00, 7.035909315511732e-01, -2.633829754125288e+00,
1.228836411604427e+00, 1.984986628348552e+01, -2.251233945111358e+00,
-1.719183132222470e+00, -1.657900337936720e+00, 5.880017189004514e+00,
-1.457853150977316e+00, -1.256321736879855e-01, 4.195967333337582e-03,
6.124593395989102e-01, -6.984300191421057e+00, -1.109668043019734e+01,
3.495690900208206e+00, 1.060885104938509e+01, 3.311088125253086e+00,
-1.940418495623120e+00, -4.925671576901262e+00, -1.499311729400677e+01,
1.463661090681758e+01, 8.079191840423764e+00, -6.004494198656432e+00,
2.504118135039964e+00, -7.756854445319441e+00, 1.227392364487501e+01,
6.155641258945186e+00, -6.910188018996058e+00, 4.737275924694198e-01,
-2.396011813417099e+00, 6.986807799948414e+00, -5.669374286870211e+00,
-7.623500847246568e+00, 1.191697794926230e+00, 7.304323910339295e-01,
1.637751221098621e+01, -9.579155542068678e+00, -1.851849133996422e+00,
5.908817613087968e+00, 1.032722165383757e+01, -2.590203109565736e+01,
-2.080225011820318e+00, 2.157264450929077e+00, 3.008776607514720e+00,
-7.286881211904412e-01, 5.274504060885437e+00, -5.602749105891163e+00,
3.319833074139494e+00, 1.632633688840626e+00, -1.029669671916826e+01,
-3.100108046191381e+00, 8.815952034295175e-01, 1.735944779154169e-02,
-4.939753665870401e+00, 5.096750961516906e+00, 6.462525436987436e-01,
2.804307106679559e+00, 1.297998206859548e+00, 1.670243586079952e+00,
1.321263339862172e+00, -6.220898546809265e-01, -2.226331447414422e+00,
-1.805391654887515e+00, -4.917161887561923e+00, -7.305791589177550e-01,
1.683927631533520e-01, -5.047165637899712e-01, -5.441965913867522e+00,
2.070555443891329e+00, -6.391072533115633e-03, -7.480221034649558e-01,
-2.259590173819782e+00, 2.612017661950841e+00, 5.866331584031756e-01,
1.249120177364725e+00, 1.621818005762777e+00, 8.674977500132826e-01,
-3.103866513519283e-01, -1.975227085528169e-01, 1.954560828974151e+00,

-3.416493561303184e+00, -4.668927481236940e-01, 1.429661155178328e+00,
1.404486433965740e+00, 6.774118886905653e-01, 1.801131777340584e+00,
1.054815094321778e+00, -1.085841009568420e+00, 1.128084273870551e+00,
-1.678812031101402e+00, -3.277959116216191e-01, 1.659260196309653e+00,
1.357548961818402e-01, 3.547377538332003e-01, -8.081617672093244e-01,
-6.932951413719541e-01, 8.640232252951202e-01, 4.280924970279125e+00,
-2.932074713343036e-01, 1.249200655399517e+00, -2.642185470296572e-01,
1.494150927030945e+00, 4.206837333030459e-01, 5.667362540003368e-01,
4.197723773867288e-01, 6.135229202060592e-02, -8.691587899531050e-01,
-9.812647168772586e-01, -1.625177565502679e+00, -9.734054428085742e-02,
-5.709330607669428e-01, 3.648383848672846e-01, 7.664035727138936e-01,
-2.383739602459009e-01, 8.737339376691761e-01, 4.009045925070656e-01,
-1.368389096600391e+00, -1.733740304151982e+00, 6.235797697474056e-01,
4.662141314901233e-03, 4.834367479909704e-01, -1.218819846607660e+00,
6.276967783614846e-01, 1.773399040564946e-01, 3.696425278044408e-01,
-2.774027301558615e-02, -1.351990451388281e-01, -2.604588897625060e-01,
1.422742700069136e-01, 1.017922538555747e-01, -8.207847062931234e-01,
8.602522344090492e-02, 3.730721649018063e-02, 1.436344742813152e-01,
9.347340143164061e-02, -3.462078051904970e-01, -1.024811553204571e+01,
5.638813822584511e+00, 1.183926995664295e+01, 1.015182147296814e+01,
8.735539019061237e+00, -7.822384870769046e+00, -8.743823505199472e-01,
-3.188561362362046e+00, -6.992280066674315e+00, -2.217802954109407e+01,
2.718939051208268e+01, 4.657707469454130e+01, 6.065878091491275e+01,
2.503535829964751e+01, -2.637436067399041e+01, -3.135909180765447e+01,
1.112969719827929e+00, -8.935560015802967e+00, -2.454496912531776e+01,
3.026020598096366e+01, 1.836609419952585e+01, -1.983400059215917e+01,
-1.335979405063810e+00, 2.929657115860052e+00, -2.703649213887266e+01,
-4.241010771562542e+01, -1.980571432609244e+01, 4.355825521953390e+01,
1.509571285120959e+01, -3.299851405974513e+00, -3.437759487187229e+01,
-3.790309898028369e+00, 9.312579628671724e+00, 3.185706421594062e+00,
-1.743108459774491e+01, -3.805706830399140e+01, -1.255933796182372e+01,
1.992552102834744e+01, 1.272137304367918e+01, -9.320746493431619e+00,
-8.266633142042688e+00, -4.851994758220964e+01, -3.317518417810599e+01,
1.500264594426433e+01, 2.611596491303845e+01, 4.864333748233944e+01,
9.524416227946105e+00, -2.786543537903118e+00, 1.650592035275873e+01,
-3.716672311035797e+01, 2.054445157862554e+01, -2.491643225571205e+00,
1.883796390267155e+01, -9.753519199332695e+00, -4.672686378719406e+01,
5.633318037466336e+01, 3.722444233584967e+01, -3.563794514691374e+01,
-1.238599140104709e+01, -2.607633731586860e+01, 6.091284956927956e+00,
1.281254511799398e+02, -2.245771575247701e+01, -4.089343974306389e+01,
2.219606442410647e+01, -1.128930733576249e+01, 6.970423115854637e+00,
-1.748329100367508e+01, -3.723295750902224e+01, 2.201067297146905e+01,
-2.791301968035434e+01, -7.662500608450754e+00, -1.035740628782533e+01,
-9.240069942908500e+00, -1.356645487793050e+02, 1.079138011828502e+02,
4.974882902237505e+01, 3.372919128206588e+01, 1.087639442964775e+00,
8.274597128771570e+01, 4.811780732950779e+01, -5.261523749301533e+01,
-1.796236548042718e+01, -2.525107562316754e+01, 4.729459511853181e+00,
-7.805035882096418e+01, -1.897277732248290e+01, 8.192127532355899e+00,
1.209951493841148e+02, -5.076455015042506e+01, 7.990952560538322e+00,
5.434315003649374e+01, -2.487882473608753e+02, 5.493346795000003e+00,
-4.333129816799030e+01, -1.308466986918875e+02, 2.660184020767075e+01,
6.119685995844453e+01, -8.406881405539641e+00, -3.446168867461613e+01,
6.093451027050014e+01, 4.340135770631792e+01, -6.585066580385237e+00,
7.959005905514711e+00, -6.852381803510822e+01, 2.419433209616861e+01,
1.713603897657721e+02, -1.472152766597500e+02, 1.551366445304601e+01,
-6.796705229597160e+01, 3.905163160569398e+01, -6.607729747963097e+01,
4.121812418279854e+01, 2.509989740643199e+01, -2.563036460991619e+01,
-3.144157247065454e+01, -3.018805352288470e+01, 2.195400466774009e+01,
1.969383559780861e+01, 1.970884480370078e+01, 1.316094041787011e+02,
-8.911615199992816e+00, -7.730376971464116e+01, 4.606192114670210e+01,
-1.011797395958560e+00, 1.063078089932108e+01, -7.360939803061331e+01,
-1.126049413937627e+01, -1.601157467771960e+01, 1.835959222417017e+01,
4.363534276528795e+01, -1.420435300310979e+01, -2.110174875868117e+01,
1.299859088023124e+01, 5.864770146219516e+00, 7.595326064960403e-01,
-6.657843932562834e+00, -8.818790476140906e-01, -2.652175041064964e+01,
-6.152669614126704e+01, -1.327878603034169e+01, -6.043883785369840e+00,
3.806135614159832e+01, 6.481180133120782e+01, 1.059340162874280e+02,
1.147894359265103e+02, -1.369872893551839e+01, -4.083258946755532e+00,
-3.928300461536296e+01, -2.015607363206266e+01, -1.391803358905325e+01,

2.726995723252221e+00, -1.472437540265706e+01, -3.205943347676577e+00,
-2.075634641953476e-01, 2.374423136078682e+01, 5.355900919276061e+01,
2.194592452565412e+01, 5.163529604990114e+00, 1.458991948649980e+02,
1.262522199813169e+01, -4.589169129315160e+01, -1.211892040584380e+01,
-1.025914310501382e+02, 3.756129600776175e+01, 7.891841939963412e+01,
1.478714493652806e+01, 1.112621953733771e+01, 5.331631416291089e-01,
-3.644224431629148e+01, -1.207009387094470e+01, -3.997676643632760e+01,
4.297205310082047e+00, 5.466383907513541e+00, 1.385848265884413e+00,
1.077582118292015e+01, 4.003613244612090e+01, -3.274497969579296e+01,
-1.734929659884485e+01, 3.328544804791238e+00, 4.264727478608492e+00,
1.716505306757698e+01, -6.660985204236905e+00, 2.730862497356647e+00,
4.233508391343714e-01, -1.442271310165563e+01, 3.694724312708692e+00,
-9.180348824175447e+00, 1.378112953637810e+01, -3.461940946448257e+01,
-5.612715497910094e+01, 1.514695939494104e+00, -1.010811843822156e+01,
3.561351104713522e+01, 2.310877304083911e+01, 7.746293991362275e+01,
-2.718369733443830e+01, 2.686651210582238e+01, 1.092163528199097e+01,
-1.057932498383262e+02, -3.006933462478879e+01, -9.918101063618427e+01,
-5.903702208706743e+01, -6.136460111034366e+01, 6.294574760154309e+00,
3.596501938455127e+01, 3.268100913243492e+01, 1.909352608925185e+01,
-1.377087858567214e+01, -5.004022532504940e+01, 2.356817275706232e+01,
1.730027436672440e+01, -3.885570489984474e+01, -3.005724849673762e+01,
-4.047500159150280e+00, -2.302365375884385e+01, -6.715399487361991e+01,
1.300595794897641e+01, 2.770526911744749e+01, 5.404573782640106e+01,
6.230342454463427e+01, 2.080345759154039e+01, -2.111781042026798e+01,
3.755292502633806e+01, 7.164703213224880e-01, -6.188007423796821e+00,
-2.625930897898110e+01, -1.844842426082741e+01, 1.239978681661718e+01,
5.250598504395279e+00, -3.436777430193297e+01, 1.410071882753421e-01,
1.224875208869517e+02, 1.513428469976698e+01, -9.701599871942085e+00,
7.111296825631085e+01, -8.268260159700942e+01, -3.822376146730964e+01,
-1.550238808277784e+01, 7.753690864824358e+01, -1.410346870011553e+01,
-4.597842908796719e+01, 1.144799963188182e+00, -2.500300779815388e+01,
-1.766114577561436e+01, 4.737818748693300e+01, -1.779236799817563e+01,
2.605573979317312e+01, 3.637523747809526e+00, 7.951497792307203e+00,
1.632182201344652e+01, -2.214541915899294e+02, 1.156650250470394e+02,
-4.616988305207087e+01, 5.483019810232925e+01, 2.189222987187786e+01,
1.982716249045394e+02, -3.341951491358525e+01, -8.115199288918571e+00,
-4.185113657317612e+01, 1.059158533461846e+02, 1.346127220198322e+01,
-6.329014354878732e+01, -1.011406425430181e+02, 3.749427118816160e+01,
6.829432380495581e+01, 8.486377724099069e+01, -9.052477880437033e+00,
-6.013872619900621e+01, -1.093261393585776e+01, -7.982208825147258e+00,
-2.824244303728878e+01, 2.480400676860175e+01, -1.104287899535621e+02,
2.084640479800649e+01, -7.151716373365862e+01, 6.964565505011943e+00,
3.250761477516157e+01, 4.630889282474494e+01, -4.315796817769571e+01,
-4.975382404369508e+01, 1.223296429525892e+02, 2.408727289938183e+01,
6.998535830479565e+00, 3.376537565259142e+01, 3.539446034336853e+01,
-8.477459872892189e+01, 3.033790292371399e+01, -3.077453832275031e+01,
1.216034574262759e+01, -3.003625282985814e+00, 7.591754428779782e+00,
-8.671937155344427e+00, -1.113919498808217e+01, 7.421538920668956e+00,
9.745687670224269e+00, -2.428320407746477e+01, 5.108640809506199e+01,
-2.630197317789319e+00, -3.310129744583882e+01, -2.433470460578139e+01,
1.258989352213262e+01, -1.767124378616606e+01, 4.115293128698588e+01,
-5.136888506149191e+01, -1.925860370855680e+00, 2.585354989433073e+00,
-5.557525593807890e+00, -6.883781259596151e-01, 6.294218236541800e+00,
-6.772623066411688e-01, -5.458352885687065e+00, -6.617516414132404e-01,
-9.839078608581968e-01, -1.494063503682812e+01, 3.147771284370950e-01,
8.969518629970341e-01, 1.471095841199402e+01, -3.611558516854035e+00,
-7.037115591560354e+00, -8.402928088926748e+00, -9.377366706671918e+00,
1.451162429629361e+01, 2.154290119766148e+01, 1.895105191027989e+00,
-8.539907443790279e+00, -9.561831121652666e-01, 4.483916166176298e+00,
1.161686356383509e+01, 7.181485249753096e-01, 4.186388150278799e+00,
7.492663750086243e+00, 4.362869904674713e+00, 1.819728472128351e+01,
-4.952397459903670e-01, -2.475459870245114e+00, -2.363537550954485e+01,
-4.657241801112169e+00, 1.268112731177343e+01, 1.609115101771576e+00,
-6.715344280609558e+00, 7.548202518027924e+00, 4.062834766528037e-01,
-5.720277254849139e+00, 3.240187751353532e+01, -2.307434599977796e+01,
-4.455131862610844e+00, -1.011560570124908e+01, 6.385926857701949e+00,
-5.325377272426623e+00, 5.483323660622081e+00, -1.087394225984023e+00,
1.084227695194306e+00, 2.920343045223092e+00, -1.298207416164547e+00,
-1.469858501946725e+01, 1.384642820468709e+01, -4.998150511900364e+01,

-3.608280090692876e+00, 1.713251010678056e+01, -9.365800838574561e+00,
9.651758156973793e-01, 3.333016986695485e+00, -1.092834518877138e+00,
2.033371082186328e+00, -2.299130766359816e+00, -2.192930983936970e+00,
-1.388475022585083e+01, -1.606264131342992e+00, 9.611431280321627e+00,
6.356037218719782e+00, 7.773537329610099e+00, -5.511948145960379e-01,
3.785646815981825e+00, 2.343489536024640e+00, -6.698204762946992e+00,
2.844255340959322e+00, 2.026022255120366e+01, 1.602242741091165e+01,
1.395010812573773e+00, -2.512040813689751e+01, 7.140619953138836e+00,
4.825916219320501e+01, -1.674635834370771e+01, -1.584022119390043e+01,
-1.755831508707101e+01, -2.097194229323482e+00, 2.769040529752838e+00,
2.314070218346117e+00, 2.847622021060690e+01, -4.061878196662757e+01,
-3.562549523359799e+00, -1.317314489559685e+00, -8.650360801345224e+00,
1.800815629295148e+00, -1.130898745600987e+01, 2.906346979155720e+00,
7.303594782923382e+00, 1.125635600490730e+00, -3.122202723828575e+01,
-8.000111753896762e+00, -3.489055629716661e+01, 1.805647655550347e+01,
1.101332949738037e+01, 2.874812488541418e+01, -1.324648799628345e+01,
-1.087149280333998e+00, 3.443516316860208e+00, 2.989427901609864e+00,
-5.883064729392911e+00, 1.846505088898892e+00, 2.517966543913590e+00,
5.495701808470343e+00, -2.508119407963729e-01, -1.033336805188275e+01,
2.058206174022696e+00, 3.005196404434536e+00, 1.895271519261179e-01,
-1.366712619544035e+01, 9.174273500427571e+00, 1.308035047183137e+00,
7.582483555345630e-01, 1.342894119539738e+01, 8.150962739835197e-01,
9.573997524127799e+00, 3.656041099570748e+00, -1.691837171757428e+01,
-1.000794942676095e+01, 4.037916420863084e+00, -2.165349975052457e+01,
1.224997310475737e+01, 8.931959709415896e+00, -2.719979700993482e+01,
-1.336991297045334e+00, 1.205208585535480e+01, -5.865773380275607e+00,
5.822035968646623e+00, -1.772947685296684e+01, -3.719034857650577e+00,
3.297673554184369e+01, 3.094810605935434e+00, -1.058323609940044e+01,
5.705191175493285e+00, 3.157991982009096e+01, -1.030346979453155e+02,
1.523630505213953e+01, 1.058892954089402e+00, 4.073827850089683e+01,
-2.550169633491879e+01, 1.342135911733172e+01, -8.469270479053920e+00,
2.188579128701993e+01, -2.374096091252054e+01, 6.566889077582605e+00,
-2.921689613161226e+00, -1.219655572227807e+01, 8.030308224861855e-01,
-5.358521300225619e+00, 3.242410310557495e+01, 3.009831953360738e+01,
8.428034477797201e+00, -2.768195659262251e+00, -2.612865371730357e+01,
2.584394800443577e+01, 2.873209676444302e+01, -1.345881355431275e+01,
4.577185870158609e+00, -9.354807342864506e+00, -4.711568128723775e+00,
-4.600199454964407e+00, -1.671902212950557e+00, -8.248372339000667e+00,
3.030248937196248e+00, 1.413996996689212e+00, -1.218920667339318e+00,
-1.417574344242241e+01, 5.107988982811123e+00, 6.152322957685278e+00,
1.109362743690941e+01, -1.568832271345392e+01, 4.841903530356637e+00,
1.763959729828060e+00, 3.525062368639977e+00, -1.036806910628041e+01,
-6.063358129008719e+00, -1.206482441328418e+00, -1.991872987032255e+00,
1.023929017791492e+00, 1.237426992885700e+00, -6.783586316236748e+00,
1.149233988435501e+00, 3.071945657326799e+00, -6.555557456642748e+00,
1.306645660626644e+01, 7.926942919462804e+00, 2.159904757533207e+01,
-1.420615830636763e+01, -1.107680223788326e+01, 7.370544150195949e-01,
1.204367327051688e+01, 2.710929956167281e+01, 1.664777768567712e+00,
1.183321982160630e-02, -6.980562770454092e+00, 7.455683747590094e+00,
-2.658998350186516e+00, -8.302868027284820e+00, -6.337481785887297e+00,
-6.106904203398649e+00, -7.146000479902837e+00, -6.696088743207126e-01,
1.311057392859502e+01, 8.926188943482614e+00, -9.534503850997579e+00,
-2.915289782625513e+01, 1.160743305987034e+01, -1.286893758102742e+01,
7.837284801523699e-02, -2.027487353398696e+01, 2.902974135661225e+01,
9.515853526092559e-01, -1.230957559069626e+01, -6.155207216224525e+01,
1.140707447240318e+00, 5.441969770646123e+00, 1.176994782800485e+01,
-2.293896938585332e+00, -3.868120407086591e+00, 1.280865498300695e-01,
-2.721068981694238e+01, 1.248183858129054e+01, -1.766449063299761e+01,
2.979221468063228e+00, 1.722038014344108e+00, 1.223927235665254e+00,
1.712075278214080e+00, 1.329651833837225e+01, 8.521957708881786e+00,
2.497206788160430e+01, 5.216338489486644e+00, 4.060549627743134e+00,
4.614302056524596e+00, 5.509848607737710e+00, -1.258882357986752e+01,
2.695491336050920e+00, 9.079476247538755e+00, -1.951529671132320e+01,
4.761072630319521e+00, 6.898759846663463e+00, 1.685239395134785e+01,
1.588273316205846e+01, 2.796829163370463e+01, -2.907334065020779e+00,
1.172755170200869e+01, -1.383808223617945e+01, 1.712273324781860e+00,
-4.951129750616929e+00, -2.227731777214407e+00, 1.032330870142750e+01,
-1.360370242762761e+01, 2.080888706868533e+01, 1.499765328430926e+01,
1.529463749438579e+01, -9.326636684341391e-01, 7.338165499000439e+00,

-1.505905566973693e+01, -3.825453987489226e+00, -4.166612862194375e+00,
-2.111681998002073e+00, -1.381296220815801e+01, 8.383222783583031e+00,
-7.809315133250887e+00, -2.092263479411120e+00, 3.215754108360652e-01,
2.165565663235144e+01, 7.520940282807034e+00, -3.294645946980216e+00,
-7.745905415152149e+00, 9.451890145044485e+00, -1.881726242867925e+01,
-1.083042446176133e+01, -8.661206217919117e+00, -9.726786878971014e+00,
1.601212183899147e+01, 1.084242456185426e+01, -3.283044542265044e+01,
1.054035183827265e+00, -1.441322831053160e+01, 1.705327350616383e+01,
6.964426896714154e+00, 2.302650608647493e+00, -8.465830233406072e+00,
3.041386008523054e-01, 1.030902695290438e+01, 1.505807782792629e+00,
1.246492128640997e+00, 3.947371478987982e+00, -5.450643712561647e+00,
-3.724860897211775e+01, 9.295519842191116e+00, -2.459138053497407e+00,
1.810375765182380e+01, 6.337151073108565e+01, 4.376989729327733e+00,
-3.485593125067458e+01, 2.528021748266124e+01, -2.701690596573505e+00,
-3.490162270226559e+00, -2.398603451394787e+00, 2.560231678138009e+01,
-1.047389532539669e+01, 7.123685411953067e+00, 5.519349380716318e+00,
-1.085063325778924e+01, 2.026239495162821e+01, 1.206284540507743e+01,
-5.112731872462724e-01, -4.912667038340359e+00, -1.305101933415679e+00,
1.581744926588647e+00, -1.526136033666078e+01, -8.597660918407602e+00,
1.412293641289442e+00, 4.990672170203357e+00, 5.532031966945547e+01,
-2.110389350571232e+01, -5.245362239558192e+01, 3.285420517027442e+01,
-2.977698762784939e+01, 1.761416486232847e+01, -2.258985020084704e+01,
-2.061762107538081e+01, -8.675139881682226e+00, -3.140791032248625e+01,
2.050877901118445e+01, -5.645557162551408e+00, 5.224905371008097e+00,
6.393568294525752e+00, -1.086417880525992e+01, -8.631389267730381e+00,
8.760069016979477e+00, -6.048993557855160e+00, 2.387528778534681e-01,
2.313058813930195e+01, -9.150325795582901e+00, 9.508819705094162e+00,
-7.442089764285964e+00, -1.646590296576141e+01, -9.071132265498365e+00,
7.253135322751388e+00, 9.405557509644055e-02, 4.717518298263115e+00,
-2.793095446788271e+00, -1.653437320753717e+00, -6.162994919319771e+00,
2.631170013671973e+00, 3.988793699228948e+00, -1.221282813173450e+00,
7.695028189285120e-01, 4.673470546909087e+00, 1.336107274327165e+01,
-5.854189147036084e-01, -1.159089247233955e+00, 6.916044371498022e+00,
-7.445115352421386e-01, -1.850350658976382e+01, 3.458433294063214e+00,
-1.046578284530154e+00, 2.132365038234726e+00, 1.240829749434915e+00,
-9.428946653552575e+00, -2.095473696217359e+01, 3.161782055027125e+00,
3.634520567356085e-01, 1.187160534753115e+01, 1.768556931241311e+00,
6.850811627395189e-01, 3.380107206068383e+00, 1.442728603110454e+01,
-3.620120786402723e+00, -1.144830944033773e+01, -1.882402439708689e+00,
4.904784513307795e-01, -2.659750322516234e+00, 1.843029624587895e+00,
5.232625705749508e-01, 1.775047673530836e+00, -2.297016169327855e+00,
-1.165212895125313e+00, 1.106361523559964e+00, 1.085359883564875e+00,
-2.605326772424981e+00, 2.336074529221282e+00, 2.946017486604013e+00,
-9.441624326318880e-01, -1.154619910752085e+01, -3.312082226208037e+00,
4.643758357480067e+00, -5.236041179736375e+00, -2.975090397013650e+00,
4.763080582516854e-01, -7.883711860454683e-01, -1.610459456170501e+00,
1.088404008958139e+00, -2.988841381257284e-01, -2.351665949176571e+00,
9.679776748304050e-01, -6.651743220504939e-01, -1.033140103489350e+01,
-1.935281146104163e+00, -2.254366404154817e+00, -4.436679607689682e+00,
4.826973907800539e+01, -2.955064674648921e+00, -1.773284048478306e+01,
4.683866955165748e+00, 6.132417243339979e-02, -4.913568946563619e-01,
1.295818406209339e+00, 9.398195444977911e-01, -2.719984435652028e-01,
3.938006031475948e+00, 6.177448241381599e-02, -1.564874529625262e+00,
1.173552451575615e+00, -7.683777102802513e-01, 2.653927891312599e+00,
-5.495984105596969e-01, 1.326609100915087e+00, -3.185720102777677e+00,
-4.747312500445836e+00, -3.290709719356703e+00, -1.702361932655170e+01,
1.085020498795842e+01, 1.161393535008814e+01, 1.148501624794207e+01,
-4.254088575507249e-01, -5.278500169407260e-01, 2.469296526363330e+01,
-4.289704832434016e+01, 4.701373191693041e-01, 1.672430460124790e+00,
4.757054017147449e-01, -6.087431082579862e+00, 2.153054448243811e+01,
-1.169165221870069e+00, 2.580931178169982e-01, -3.567587245848109e+00,
-1.173234585594734e+01, 4.284148740822941e-01, -1.860400117081616e+01,
1.916566141997117e+01, 1.148813947410018e+00, -2.029349194120107e-01,
-2.439281368297836e+00, 1.993695925592683e+00, -1.005981194577029e+00,
7.808703015045083e-02, -1.904952191591125e+00, 6.513491659245895e-01,
-7.979081616185092e-01, -3.257101090852283e+00, -2.129924079331195e+00,
3.674898471391561e+00, 1.157033358357090e+00, 2.668536293757173e+00,
-2.182288565280748e+00, 4.395154638300799e-01, -1.113911188429168e+00,
-2.073547815092376e-02, 2.120800045731887e-01, 3.360753829958786e+00,

-6.245058027574891e-01, -1.457954337899403e+00, -2.110671240046737e-02,
-1.063604964938521e+00, 6.695044057203078e-01, 2.123096456568903e+00,
2.363078993656135e-02, 3.326489634342821e+00, 3.387429018691909e-01,
3.014480377702492e+00, 1.561992220364647e+00, -1.521029458876302e+00,
-3.544942885323918e+00, 1.260435398218770e-01, 2.068655938518684e+00,
1.373861907634400e+00, -1.990300007126860e+01, -2.097021835548150e+00,
2.112214968130340e+01, 3.244766404487805e+00, -3.384515142690109e+00,
-8.887056181424860e+00, 1.279053781619400e+00, 2.225371945284642e+00,
-2.402985558069300e+00, 8.369703667280335e-01, 5.187792360584515e+00,
1.336373822683009e+00, -1.858130058911100e+00, 1.757478555889230e+01,
4.924617233708836e+00, -3.199695896494276e+00, 1.603699255038309e+01,
-2.801994312093445e+01, -7.519623143600607e+00, 3.201755764873801e+00,
2.274182906357627e+00, -2.503378767553147e+00, -2.078597084642410e+00,
3.606265821483572e-01, -3.485467803491686e-01, 9.699334900196542e-01,
-1.248772343957115e+00, 9.144616312741140e-01, 2.385593993948885e+00,
1.528106945757852e+00, -3.204208381443653e+00, -1.616096511834961e+00,
-5.618732094049426e-01, 2.337628336704283e+00, -1.137390909597189e-01,
-4.530101621077210e-01, 1.803446794838548e+01, 4.708461713591677e+00,
-2.044683814212552e+00, -5.439117697697501e+00, -2.992051870905324e+01,
-2.627112808512978e+00, 1.796463372260682e+01, -1.187675991691008e+00,
-6.508057725326100e-01, -2.398455608832072e-01, 2.933195817977431e-01,
2.354404162371520e+00, 3.977207168627025e+00, -2.533264066376334e+00,
-1.671250067605808e+00, 8.082877426107873e-02, -2.792087750679664e+00,
-3.859950647420214e-02, -8.521119066471617e-01, -1.068242969793162e+00,
1.018261324789892e+00, 6.398726434064927e-01, -1.213324218193145e+01,
-1.261083255945134e+00, 1.204100785609345e+01, 7.599070954872811e-01,
5.250201052718509e-01, -3.071995376510091e+00, -4.669978693748058e+00,
3.067886543745797e+00, 1.921044768827043e+00, 4.543496369421751e+01,
1.699176550949505e+00, 2.012848722182744e+00, -2.338614861363877e+00,
-4.879321137436008e+01, 1.295024384041855e+00, 3.169299316500090e-01,
9.928558298114906e-01, 3.960084958719359e+00, -3.171010213118153e+00,
-8.324468443117580e-01, 4.463878778727478e-01, 1.138179392201050e+00,
4.411018025596295e+00, 1.195628824504683e-01, 2.318634971076765e+00,
-4.429017830579014e+00, 2.319264235005327e+00, 5.762205197400716e-01,
8.865290339696824e-01, -3.659591752204936e+00, -1.530695921672674e+01,
-1.743158696625229e+01, 5.702737355392236e-01, -4.166061620430360e+00,
3.799471162303255e+00, 3.323915019068432e+01, -2.502671091482795e-02,
6.205221968936869e+00, -4.099171662664758e-01, -5.564706770953149e+00,
1.212634457071130e+00, -2.236410321185692e+00, -3.262401173444578e+01,
-3.827399615698100e+00, -2.812176438437506e+01, 2.763984224952058e+01,
4.775497892008725e+00, 3.352891843742985e+01, -4.657605530223248e+00,
-2.289188884777545e+00, -1.089895785708014e+00, 4.771496505218211e+00,
-2.092900015250362e+00, -6.013865466921438e-01, 1.171472086967155e+00,
8.891268151003971e-01, 2.055177208224308e+01, -1.092284439088997e+00,
-1.435986613857175e+01, 4.220448132226750e+00, -4.572017340566951e+00,
3.813191031268905e+00, -3.009982538503275e+00, -1.966743541770994e+00,
-4.524068508646514e-01, -7.968235860430388e-01, 7.670342011094493e-01,
-2.119142608235432e+01, 2.343497286443480e+01, -4.525757916429438e+01,
-3.119833462635385e+00, 2.784837010401846e+00, 4.524412778893866e+01,
-2.561452845331314e+00, -3.853782661196873e+00, -5.602622640510593e-02,
1.081240026667991e+01, 1.608696369397246e-01, -1.223720808533250e+01,
1.327477182884169e+00, 4.049407777202643e-01, -1.743510003702366e+00,
-1.027351985755102e+00, -6.892844108878009e-01, 5.768623752326472e-01,
8.354801799610566e-01, -5.952140507889799e-01, -2.756107533508857e+00,
1.298590679959491e+00, -4.519817118838447e-01, -2.479839849548908e-01,
-8.695312377980461e-01, -2.289843227877078e-01, 1.474557743916580e-02,
-1.823179189110627e+00, 7.820043140878747e-01, -7.387892735272467e-01,
-7.278653586199029e-01, 1.078954399500569e+00, -9.763824198810446e-02,
7.182030548488257e-01, -4.882089014611350e-02, -4.044934528456195e-02,
-8.219994979268266e-01, -1.424952968767224e-01, 2.588713498447885e-01,
1.309383853280969e+00, -5.910146441206619e-02, -4.752897575973141e-01,
5.066360574726912e-01, 5.538299638841643e-01, -3.003107560443125e-01,
-7.418096825752941e-01, -4.208058387274209e-01, -7.517737138542527e-01,
-1.056342937333575e-01, 6.114708041025806e-01, -2.511222890699693e-01,
1.920509130082286e-01, 8.148483818301787e-02, -1.818652314817173e-01,
-3.874688068513649e-01, -2.809745663517779e-01, -1.039849275996392e-01,
6.762570448926842e-01, 8.024002743320824e-02, 1.519358236762945e-02,
-1.112780669405858e+00, -5.720629128161213e-01, 1.262236608438863e-01,
-6.093636581835404e-01, 7.764786606818134e-01, 3.459156966135906e-01,

-8.323289263962773e-01, -3.084532988639049e-01, -4.008247430251953e-01,
-1.060986968247208e+00, 7.096624426775953e-01, -2.298611296638830e-02,
-6.791797512828271e-02, 1.129551447602480e+00, 4.096749251914681e-01,
5.147585484565376e-01, -1.489870257761939e-01, -3.614013776505018e-01,
-3.747093488982474e-01, -3.685120577455603e-01, -3.810293535292735e-01,
2.638524171022701e-01, -3.407314485655696e-01, -5.546554556094564e-01,
1.843555545192913e-01, 7.565546022981324e-01, -3.311344007796981e-01,
3.428403688227075e-01, -1.380644663232544e-01, 2.439641699848533e-01,
-5.197487470321644e-01, -2.540188805871366e-01, -4.360994497831511e-02,
-6.095222658712287e-01, 1.752024644582240e-01, -2.351447673623439e-01,
-2.895445961166876e-01, 1.230095614757009e-01, 1.848116692224537e-01,
-5.608698946733690e-01, -1.015445401318883e+00, 8.747715562355302e-02,
-1.874606215881369e-01, 7.505388397560928e-02, -1.387817764806660e-01,
1.531708748760491e-02, -3.001916997051519e-01, -4.242745688672876e-02,
-8.473117775862470e-03, 6.352810372907991e-03, -1.422578521324087e-02}
